# The RNA polymerase I transcription inhibitor CX-5461 cooperates with topoisomerase 1 inhibition by enhancing the DNA damage response in homologous recombination-proficient high-grade serous ovarian cancer

**DOI:** 10.1038/s41416-020-01158-z

**Published:** 2020-11-11

**Authors:** Shunfei Yan, Jiachen Xuan, Natalie Brajanovski, Madeleine R. C. Tancock, Piyush B. Madhamshettiwar, Kaylene J. Simpson, Sarah Ellis, Jian Kang, Carleen Cullinane, Karen E. Sheppard, Katherine M. Hannan, Ross D. Hannan, Elaine Sanij, Richard B. Pearson, Keefe T. Chan

**Affiliations:** 1 Sir Peter MacCallum Department of Oncology, University of Melbourne, Melbourne, VIC Australia; 2 Division of Cancer Research, Peter MacCallum Cancer Centre, Melbourne, VIC Australia; 3 Department of Biochemistry and Molecular Biology, Monash University, Clayton, VIC Australia; 4 Victorian Centre for Functional Genomics, Peter MacCallum Cancer Centre, Melbourne, VIC Australia; 5 Department of Biochemistry and Molecular Biology, University of Melbourne, Melbourne, VIC Australia; 6 John Curtin School of Medical Research, Australian National University, Canberra, ACT Australia; 7 School of Biomedical Sciences, University of Queensland, Brisbane, QLD Australia; 8 Department of Clinical Pathology, University of Melbourne, Melbourne, VIC Australia

**Keywords:** Targeted therapies, Ovarian cancer

## Abstract

**Background:**

Intrinsic and acquired drug resistance represent fundamental barriers to the cure of high-grade serous ovarian carcinoma (HGSC), the most common histological subtype accounting for the majority of ovarian cancer deaths. Defects in homologous recombination (HR) DNA repair are key determinants of sensitivity to chemotherapy and poly-ADP ribose polymerase inhibitors. Restoration of HR is a common mechanism of acquired resistance that results in patient mortality, highlighting the need to identify new therapies targeting HR-proficient disease. We have shown promise for CX-5461, a cancer therapeutic in early phase clinical trials, in treating HR-deficient HGSC.

**Methods:**

Herein, we screen the whole protein-coding genome to identify potential targets whose depletion cooperates with CX-5461 in HR-proficient HGSC.

**Results:**

We demonstrate robust proliferation inhibition in cells depleted of DNA topoisomerase 1 (TOP1). Combining the clinically used TOP1 inhibitor topotecan with CX-5461 potentiates a G2/M cell cycle checkpoint arrest in multiple HR-proficient HGSC cell lines. The combination enhances a nucleolar DNA damage response and global replication stress without increasing DNA strand breakage, significantly reducing clonogenic survival and tumour growth in vivo.

**Conclusions:**

Our findings highlight the possibility of exploiting TOP1 inhibition to be combined with CX-5461 as a non-genotoxic approach in targeting HR-proficient HGSC.

## Background

High-grade serous ovarian carcinoma (HGSC) is the most prevalent histological subtype of epithelial ovarian cancer with the worst prognosis. HGSC is characterised by nearly universal *TP53* mutations (>96%), and 50% of HGSC harbour defects in homologous recombination (HR) DNA repair genes, the most common alterations occurring in breast cancer-related antigen 1 (*BRCA1*) and *BRCA2*, which are necessary for maintaining genomic integrity.^1^ Defects in HR confer exquisite sensitivity of HGSC to standard DNA-damaging chemotherapies (carboplatin/cisplatin and paclitaxel) as well as the recently Food Drug Administration-approved poly-ADP ribose polymerase (PARP) inhibitors (olaparib, rucaparib, niraparib, talazoparib), which are synthetic lethal by generating catastrophic DNA damage and cell death in cells lacking HR.^2^ However, resistance to these therapies frequently develops via multiple mechanisms, including stabilisation of stalled DNA replication forks, increased drug efflux, decreased PARP trapping and restoration of HR, a common form of resistance observed in the clinic that inevitably results in patient mortality.^3–7^ Of the 50% primary HGSC tumours that are HR-proficient, 20% harbour *CCNE1* amplifications, which are associated with intrinsic platinum-based chemotherapy, PARP-inhibitor resistance and poor clinical outcome.^8^ Therefore, new therapies targeting HR-proficient HGSCs arising from both intrinsic and acquired resistance are urgently needed to improve patient outcomes.

Increased activation of key oncogenic signalling pathways (PI3K/AKT, RAS/MAPK and MYC) upstream of ribosome biogenesis constitutes an additional hallmark of HGSC,^9^ and we hypothesise that inhibiting ribosome biogenesis can provide an effective cancer therapeutic option.^10^ Indeed, we have shown encouraging responses with the ribosomal RNA gene (rDNA) transcription inhibitor CX-5461, which inhibits the production of the major ribosomal RNA (rRNA) components of the ribosome, in a Phase I clinical trial in haematological malignancies.^11^ We observed beneficial responses in patients who were wild type (five out of eight) or mutant (one out of five) for *TP53*, highlighting CX-5461’s p53-dependent and -independent therapeutic activity, and a favourable toxicity profile. Furthermore, clinical trials in advanced solid tumours are ongoing,^12^ emphasising the potential promise of CX-5461 in treating a broad range of cancers.

Mechanistically, we have demonstrated in cells with intact p53 that CX-5461 induces an impaired ribosome biogenesis checkpoint leading to apoptosis, cell cycle arrest or senescence.^10,13,14^ However, in cells with inactivated p53 such as HGSC, CX-5461 induces replication stress and the DNA damage response (DDR), leading to a G2/M cell cycle checkpoint arrest in HR-proficient cells and cell death in HR-deficient cells.^15^ Furthermore, combining PARP inhibitors with CX-5461 enhanced cytotoxicity and therapeutic benefit in HR-deficient HGSC models in vitro and in vivo.^15^ CX-5461 has a distinct sensitivity profile compared to PARP inhibitors involving meiotic recombination 11 (MRE11)-dependent degradation of replication forks. These findings highlight the potential of identifying combination therapies to improve the efficacy of CX-5461 in targeting HGSC.

In this report, we performed a whole protein-coding genome RNA interference (RNAi) screen to identify potential targets whose inhibition can enhance the efficacy of CX-5461 in treating HR-proficient HGSC. We demonstrate the CX-5461 exhibits a unique sensitivity pattern distinct to those reported for G-quadruplex stabilisers and topoisomerase 2 (TOP2) poisons previously thought to function in an equivalent mechanism to CX-5461.^16^ Importantly, we find that DNA topoisomerase I (TOP1) inhibition can be combined with CX-5461 to target HR-proficient HGSC cells. TOP1 has been shown to localise to rDNA to release torsional stress during transcription and DNA replication of the highly repetitive and transcribed rDNA repeats.^17,18^ We demonstrate that the combination of the TOP1 inhibitor topotecan and CX-5461 exacerbates replication stress at the rDNA repeats and across the genome. We show that the combination of CX-5461 and topotecan inhibits proliferation of HR-proficient HGSC by enhancing G2/M checkpoint arrest induced by replication stress and activation of the ATR pathway without further generating DNA strand breaks compared to single-agent treatment. Furthermore, the combination of CX-5461 and topotecan leads to significantly improved regression of HR-proficient HGSC tumours in vivo, highlighting the combination as a promising approach for treating HR-proficient HGSC.

### Methods

#### Cell culture

Human HGSC cell lines (OVCAR4, OVCAR3 and CAOV3) were obtained from the National Cancer Institute. All cell lines were short tandem repeat (STR) characterised against American Tissue Type Collection or ExPASy databases to ensure the authenticity of origin. Mycoplasma tests were performed routinely by PCR. All cells were cultured in RPMI-1640 media (Gibco) supplemented with 10% foetal bovine serum (FBS) (Sigma-Aldrich) and 2 mM GlutaMax™ (Gibco) at 37 °C and 5% CO_2_.

#### Generation of inducible BRCA2 knockdown cell lines

The pLKO^TetOn^ construct expressing a doxycycline-inducible short hairpin RNA (shRNA) targeting *BRCA2* (GGGAAACACUCAGAUUAAA) was a kind gift from Madalena Tarsounas.^19^ OVCAR4 cells were transduced with VSVG-pseudotyped lentivirus produced in HEK-293T cells and 4 µg/µL polybrene, and cells were selected with 1 µg/mL puromycin for 3 days prior to use in experiments.

#### Generation of TOP1 knockout cell lines

OVCAR4 and OVCAR3 cells (3 × 10^5^) in 20 µL SF Cell Line Nucleofector™ Solution (Lonza) were nucleofected with ribonucleoprotein complexes containing 3.4 µL phosphate-buffered saline (PBS), 0.6 µL Alt-R® S.p. Cas9 nuclease-purified Cas9 protein (Integrated DNA Technologies) and 1 µL of 300 µM chemically modified EZ scaffold (Synthego) control single guide RNA (sgRNA) (CAUUUCUCAGUGCUAUAGAG) or 0.5 µL of each *TOP1* sgRNA (sgRNA #1: ACUCACUCAUCCUCAUCUCG; sgRNA #2: CAAACAUAAAGACAGAGACA) using a 4D-Nucleofector™ (Lonza).

#### Reagents and antibodies

CX-5461 was provided by SYNkinase and prepared in 50 mM NaH_2_PO_4_. Topotecan (Hycamtin®, Novartis) was obtained from the Peter Mac pharmacy and was dissolved in 0.9% saline. DBL™ doxorubicin hydrochloride injection was purchased from Hospira and diluted in PBS. The pan-caspase inhibitor Q-VD-OPh (Cat. #1901) was purchased from APExBIO and dissolved in dimethyl sulfoxide. A list of antibodies used in this study is provided in Supplementary Table S1.

#### Genome-wide protein-coding RNAi primary screen and analysis

On a screening day, 160 μL DharmaFECT 4 (Horizon Discovery) was mixed with 50 mL Opti-MEM® (Gibco) (sufficient for 16 assay plates), and 44 μL lipid:Opti-MEM mixture was then aliquoted to each well of a 384-well black-walled plate (Corning, Cat. #3712) containing 6 μL of 1 μM SMARTpool small interfering RNA (siRNA) (Horizon Discovery) using a BioTek EL406™ washer dispenser (final SMARTpool concentration 40 nM). The transfection mixture was mixed and complexed for 20 min, and 12.5 μL mixture was then aliquoted into three plates (four replicate plates in total) using a Caliper Sciclone ALH3000 liquid handler. During this period, OVCAR4 cells were trypsinised and resuspended at 5.6 × 10^4^ cells/mL. Twenty-five microlitres of OVCAR4 cells (1400 cells) was then dispensed into 384-well plates with the final concentration of the SMARTpool siRNA at 40 nM, and plates were incubated at 37 °C and 5% CO_2_. At 24 h post transfection, the medium was replaced with cell culture medium containing either 80 nM CX-5461 or 400 nM NaH_2_PO_4_. Cells were incubated for another 48 h and fixed with 2% paraformaldehyde in PBS for 10 min, followed by permeabilisation with 0.3% Triton X-100 in PBS for 10 min. Cells were washed with PBS, stained with 100 ng/mL 4′,6-diamidino-2-phenylindole (DAPI) for 20 min. Cells were washed twice with PBS and 25 fields were imaged using the ArrayScan VTI high-content system (Thermo Fisher Scientific) with a ×20/0.4 NA objective and an ORCA-ER camera and a 5-ms exposure time. The Cellomics Morphology V4 Bioapplication was used to analyse cell number as determined by DAPI staining.

To identify synergistic gene candidates, we used a combination of the difference in relative cell number between vehicle- and CX-5461-treated target siRNA plates normalised to ON-TARGETplus (Horizon Discovery) non-targeting control (siControl) ≥0.25, and a value for the coefficient of drug interaction (CDI) ≤0.9, which was calculated based on a Bliss independence model:^20^

CDI = (relative cell number (siRNA + CX-5461))/(relative cell number (siRNA + vehicle) × relative cell number (siControl + CX-5461)).

#### Secondary deconvolution screen

Based on the above criteria, we selected 372 genes for a secondary deconvolution screen using the four individual siRNA duplexes that comprised the SMARTpool arrayed separately (1 duplex/well). For the secondary screen, the 1 μM SMARTpool siRNAs were replaced by 0.45 μM individual siRNA duplexes for a final concentration of 25 nM/duplex. We modified our selection criteria to define high-confidence hits as those with a difference in relative cell number between vehicle- and CX-5461-treated target siRNA plates normalised to siControl ≥0.15 and Bliss independence ≤0.8. We classified genes with ≥2 high-confidence hits as those displaying synergy.

#### Quantitative real-time PCR

The RNeasy® Mini Kit (Qiagen) was used to extract total RNA as per the manufacturer’s instructions. One hundred nanograms of total RNA was used as a template for cDNA synthesis using SuperScript™ III reverse transcriptase (Invitrogen™ #18080093), hexameric random primers and dNTPs. Quantitative real-time PCR (qPCR) reactions were performed using Fast SYBR® Green reagents in a StepOnePlus^™^ Real-Time PCR system (Applied Biosystems™) with a +0.7 °C melt increment. RNase-free water was used as a negative control. Changes in target gene expression were normalised to *NONO* housekeeping gene and fold change was determined by using 2^(−ΔΔ*C*_t_). Primer sequences are listed in Supplementary Table S2.

#### Immunoblotting

Cells were washed twice with PBS and whole-cell lysates were prepared in Western solubilisation buffer (20 mM HEPES pH 7.9, 2% sodium dodecyl sulfate, 0.5 mM EDTA). Twenty micrograms of protein was transferred to polyvinylidene fluoride membranes, which were blocked in 5% skim milk TBS 0.1% Tween® 20 (TBST) for 45 min at room temperature (RT). Membranes were incubated with primary antibodies overnight at 4 °C, washed three times in TBST for 10 min, incubated with horseradish peroxidase-conjugated secondary antibodies for 1 h at RT and then washed. Membranes were visualised using Western Lightning™ Plus enhanced chemiluminescence (PerkinElmer) by exposure to film (Fujifilm SuperRX) or imaged by a ChemiDoc™ Touch Imaging System (Bio-Rad Technology). Digital scans of film were acquired using an Epson Perfection V700 Photo at ≥300 dpi (dots per inch).

#### Immunofluorescence

Five thousand cells were seeded into 8-well Nunc™ Lab-Tek™ II Chamber Slides™ (Cat. #154534) per well. Cells were cultured for 72 h, followed by drug treatments at the indicated timepoints and doses together with 10 μM 5-ethynyl-2′-deoxyuridine (EdU) (Sigma-Aldrich, Cat. #900584). Cells were fixed in 4% paraformaldehyde and permeabilised with 0.5% Triton X-100 in PBS. EdU was fluorescently labelled with 0.5 μM Click-iT™ EdU Alexa Fluor™ 647 Azide (Invitrogen™ #A10277) in labelling buffer (100 mM Tris, pH 8.5, 100 mM ascorbic acid, 1 mM CuSO_4_) for 30 min at RT and subsequently stained with the indicated antibodies. Cell nuclei were stained with 500 ng/mL DAPI diluted in PBS. Slides were mounted with Vectashield® Antifade Mounting Media (Vector Laboratories, Cat. #H-1000). Images were captured by a Zeiss LSM 780 confocal microscope using a ×20/0.8 NA Plan-apochromat objective and were analysed with FIJI v1.52p^21^ and CellProfiler v3.1.19.^22^

#### Cell cycle analysis

Cells were labelled with 10 μM 5-bromo-2′-deoxyuridine (BrdU) (Thermo Fisher Scientific, Cat. #B23151) for 30 min, collected with supernatant and fixed with ice-cold 80% ethanol. Fixed cells were centrifuged, resuspended in 2 N HCl + 0.5% Triton X-100 (v/v) and acid was neutralised with 0.1 M Na_2_B_4_O_7_·10H_2_O (pH 8.5). Cells (2 × 10^5^) were incubated with 100 μL anti-BrdU antibody (0.5 μg/mL) in dilution buffer (PBS + 2% FBS) + 0.5% Tween-20 for 30 min at room temperature, washed with dilution buffer and resuspended in 100 μL in Alexa Fluor 488 donkey anti-mouse IgG (5 μg/mL) in dilution buffer + 0.5% Tween-20 for 30 min on ice and then then washed. Cells were resuspended in 10 μg/mL propidium iodide (PI) (Sigma-Aldrich, Cat. #P4170) in dilution buffer and analysed by flow cytometry on a BD FACSCanto™ II. Quantitation of cell cycle populations was performed using the FlowJo v9.3.2 software.

#### Clonogenic assay

Cells (1 × 10^4^/well) were seeded into 6-well plates (BD Falcon). Cells were cultured for 24 h, and then drugged as indicated. At 48 h after drug treatment, the drugs were removed by washing twice with PBS. Cells were then cultured in normal medium for another 5 days when the vehicle-treated cells reached confluency. Cells were then fixed with 100% methanol for 10 min, stained with 0.1% crystal violet solution for 20 min, thoroughly washed with PBS and the stained plates were dried, imaged on an IncuCyte® ZOOM (Essen Bioscience) using a ×10/0.3 NA objective and analysed for cell confluence.

#### DNA fibre analysis

DNA fibre analysis was performed using methods as those previously described.^15^ OVCAR4 cells (1 × 10^6^) were plated in 10-cm dishes for 24 h. The medium was replaced with culture medium containing 50 μM 5-chloro-2-deoxyuridine (CIdU) and cells were incubated at 37 °C 5% CO_2_ for 30 min. Cells were washed three times with PBS, and fresh pre-warmed medium containing 250 µM 5-iodo-2′-deoxyuridine (IdU) was added and cells were incubated at 37 °C 5% CO_2_ for 30 min. Cells were washed three times with warm PBS and treated as indicated for 3 h at 37 °C 5% CO_2_. Labelled cells were trypsinised, pelleted, washed twice with PBS and resuspended in a pre-warmed solution (50 °C) composed of PBS with 0.05% phenol red-free trypsin. Cellular suspensions were then mixed carefully with an equal volume of 1.2% low melt agarose (Bio-Rad, 1613111), and the mixture was dispensed into a plug mould (Bio-Rad, 1703713) and allowed to set at 4 °C for 1 h. Solidified plugs were then pushed out of the mould and transferred into 2-mL polypropylene tubes containing 0.5 M EDTA pH 8, 1% (v/v) Sarkosyl (Sigma-Aldrich, 61743), and proteinase K (Roche Applied Science, 3115828001). The agarose plugs were incubated in this buffer at 50 °C overnight. Following incubation, agarose plugs were washed extensively (1 M Tris, 0.1 M EDTA pH 8) and digested with β-agarase (New England Biolabs, M0392S) overnight in 0.5 M MES hydrate (Sigma-Aldrich, M5287) pH 5.5 at 42 °C. Samples were then poured carefully into a FiberComb reservoir (Genomic Vision, RES-001) and the DNA solution combed onto silanised coverslips (Genomic Vision, COV-002) at a constant speed of 250 µm/s, using the Molecular Combing System by Genomic Vision (MCS-001). For easy handling following combing, coverslips were adhered to glass SuperFrost® Plus slides (Menzel Gläser) using cyanoacrylate glue and then baked in an incubator at 65 °C for 2 h to irreversibly crosslink DNA to the surface. Next, samples were immersed in a solution containing 0.5 M NaOH and 1 M NaCl for 8 min at room temperature to denature the combed DNA, before being washed thoroughly with PBS. Coverslips were subsequently dehydrated by incubating them in increasing concentrations of 70%, 90% and 100% of ethanol for 5 min each, followed by air drying. To minimise non-specific binding of antibodies, blocking buffer containing 0.1% Triton X-100 and 5% bovine serum albumin was applied to all slides for 30 min at room temperature prior to staining. DNA fibres attached to coverslips were then probed with rat anti-BrdU antibody (1:100, Abcam ab6323) specific to CldU and mouse anti-BrdU antibody (1:50, Becton Dickinson, 347580) specific to IdU for 1 h at 37 °C in a humidified chamber. Following staining, slides were washed three times with PBS and subsequently incubated with Alexa Fluor 488-conjugated goat anti-rat antibody (Invitrogen, A-11006) and Alexa Fluor 594-labelled donkey anti-mouse antibody (Invitrogen, A-21203) at 1:200 dilutions, for 1 h at 37 °C. Lastly, slides were washed three times in PBS, mounted and visualised using the Nikon C2 confocal microscopy at ×40 magnification. Images were taken of 100 fibres per condition. Only high-quality and well-separated DNA fibres (not entangled DNA regions) were measured using ImageJ software (1.47v, NIH). The ratio of IdU to CldU tracks in each fibre was calculated and graphed using GraphPad Prism 8.

#### DNA comet assay

DNA comet assays were performed using the CometAssay® Reagent Kit (Trevigen, Cat. #4250-050-K) according to the manufacturer’s protocol. Briefly, cells treated with indicated drugs were trypsinised and washed once with ice-cold PBS, and then resuspended in ice-cold PBS at 1 × 10^5^ cells/mL. Cells were mixed with molten low-melt agarose at 37 °C at a ratio of 1:10 and 50 μL of the mixture was immediately pipetted onto a CometSlide™. The slides were incubated in the dark at 4 °C for 30 min to solidify the agarose and immersed in 4 °C lysis solution overnight. Slides were then immersed in freshly prepared alkaline unwinding solution for 1 h at 4 °C in the dark, and electrophoresed in 4 °C alkaline electrophoresis solution at 300 mA for 40 min. The slides were washed with ddH_2_O twice, followed by 70% ethanol for 5 min. Slides were dried at 37 °C for 15 min, and then stained with 2.5 μg/mL PI in PBS for 30 min at room temperature. Slides were rinsed twice in ddH_2_O and completely dried at 37 °C. Images were captured using a VS120 Virtual Slide Microscope (Olympus) using a ×10 objective and analysed with the OpenComet v1.3.1^23^ plugin for ImageJ 1.51.

#### Xenograft transplantation

All animal studies were conducted according to the protocols approved by the Animal Experimentation Ethics Committee at the Peter MacCallum Cancer Centre. Non-obese diabetic severe-combined immunodeficiency gamma (NSG) mice (6–8 weeks old female) were purchased from the Garvan Institute (Australian BioResources) and housed in animal cages under standard laboratory conditions. OVCAR3 cells (6 × 10^6^) in 100 μL ice-cold PBS:Matrigel (1:1) were subcutaneously injected into the right flank of mice anaesthetised with isoflurane. Tumour-bearing mice were weighed and measured twice weekly with callipers. Once tumours reached an average volume of 100 mm^3^, mice were randomised into four groups of ten mice. Mice were dosed with vehicle (25 mM NaH_2_PO_4_) or CX-5461 (30 mg/kg) via oral gavage twice weekly, while topotecan 5 mg/kg or vehicle (0.9% saline) was delivered via intraperitoneal injection twice weekly. Mice were dosed for 4 consecutive weeks for a total of eight doses. Bodyweight and tumour volumes were monitored daily during and after the treatment period. Mice were euthanised by cervical dislocation upon reaching any ethical endpoint of the experiment (signs of distress or tumour volume ≥1200 mm^3^).

#### Statistical analysis software

Prism 8 (GraphPad) was used for all the statistical analyses as indicated, including dose–response curves, two-sided *t* tests, Wilcoxon’s rank-sum non-parametric tests, ordinary one-way analysis of variance (ANOVA), Kruskal–Wallis non-parametric one-way ANOVA and Mantel–Cox tests. Synergy was quantified using Combenefit v2.02.^24^

## Results

### A functional genomics screen identifies a network of genes that when depleted cooperates with CX-5461 to inhibit HR-proficient HGSC cell proliferation

**Fig. 1:**
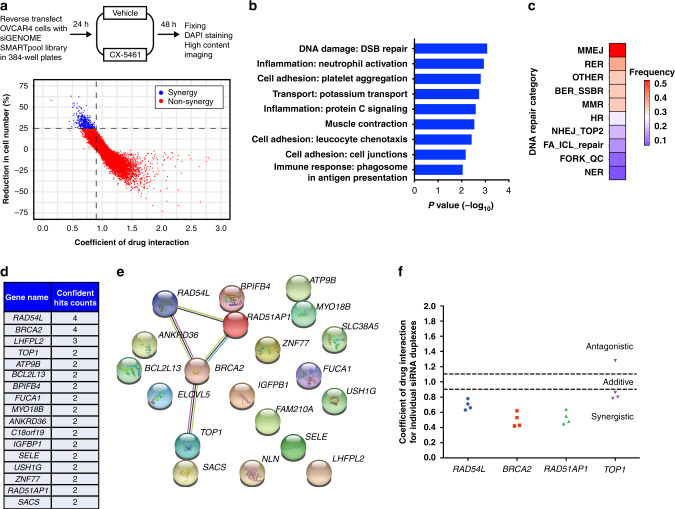
A genome-wide RNAi screen identifies a network of genes that when depleted synergise with CX-5461 in inhibiting HGSC cell proliferation. **a** Schematic representation of the genome-wide primary screen design. OVCAR4 cells were reverse transfected with the siGENOME SMARTpool library. After 24 h, cells were treated with vehicle control (400 nM NaH_2_PO_4_) or 80 nM CX-5461 (GI_25_–GI_50_ dose for proliferation at 2 days (Table S7)). At 48 h post transfection, cells were fixed, DAPI stained and imaged by high content microscopy to determine cell number. **b** Gene ontology analysis of hits identified in the primary screen. Significant enriched functional processes (*P* value <0.01) associated with the primary screen hits. **c** Heatmap of the frequency of primary hits involved in DNA repair pathways. **d** Table of confident hits identified in the secondary deconvolution screen. **e** STRING network of the 20 validated screen hits. **f** Plot of Coefficient of drug interaction for individual siRNA duplexes against *RAD54L*, *BRCA2*, *RAD51AP1* and *TOP1* genes, modified from ref. ^15^ (CC BY-NC 4.0).

To identify genes that, when depleted, could synergise with CX-5461 to inhibit HR-proficient HGSC cell proliferation, we used human OVCAR4 cells^25^ in a protein-coding genome-wide RNAi screen (Fig. 1a). Based on quantifying the reduction in cell number relative to control siRNA (siControl) and the CDI to assess combinatorial effects, we identified 372 genes whose knockdown demonstrated synergy with CX-5461 (Table S3). Gene ontology analysis of these candidates identified enrichment in several functional processes, with DNA damage:double-strand break (DSB) repair being the most significant (Fig. 1b and Table S4). Furthermore, analysis of the gene candidates involved in different DNA repair pathways showed a unique sensitivity pattern distinct to those reported for pyridostatin, a G-quadruplex stabiliser, and TOP2 poisons thought to function via an equivalent mechanism to CX-5461 (Fig. 1c and Table S5).^16,26^ Deconvolution screening using individual siRNA duplexes and more stringent metrics validated 20 genes using a concordance criterion of two out of four (Fig. 1d and Table S6). STRING network analysis demonstrated significant enrichment of genes involved in the HR pathway (Fig. 1e), including *BRCA2* (4/4 siRNA duplexes validated). To further confirm this finding, we generated OVCAR4 cells expressing a doxycycline-inducible *BRCA2* shRNA (Fig. S1A).^19^ Treating OVCAR4 cells depleted of BRCA2 with CX-5461 led to a two-fold reduction in cell proliferation (Fig. S1B), which is consistent with the magnitude of reduction previously reported in the PEO1 ovarian cancer cell line harbouring BRCA2 knockout.^26^ Together, these data indicate that inducing HR deficiency in HR-proficient cells can enhance their sensitivity to CX-5461.

Of the validated candidates, we also identified TOP1 (2/4 siRNA duplexes validated) (Fig. 1f), whose physiological function is to relieve DNA supercoiling that occurs during transcription and replication by generating single-strand breaks.^17^ To further validate this result, we knocked out TOP1 in OVCAR4 cells using clustered interspaced short palindromic repeats/Cas9-mediated gene editing (Fig. S1C). Clonogenic assays showed significantly reduced colony formation in TOP1-knockout cells treated with CX-5461 compared to control (Fig. S1D). Knockout of TOP1 in another HR-proficient HGSC cell line OVCAR3 (Fig. S1E) also resulted in decreased colony formation (Fig. S1F) upon treatment with CX-5461 compared to control. Collectively, these data suggest that TOP1 depletion cooperates with CX-5461 in inhibiting HR-proficient HGSC cell proliferation.

### The combination of CX-5461 and topotecan induces a robust G2/M cell cycle checkpoint arrest

**Fig. 2:**
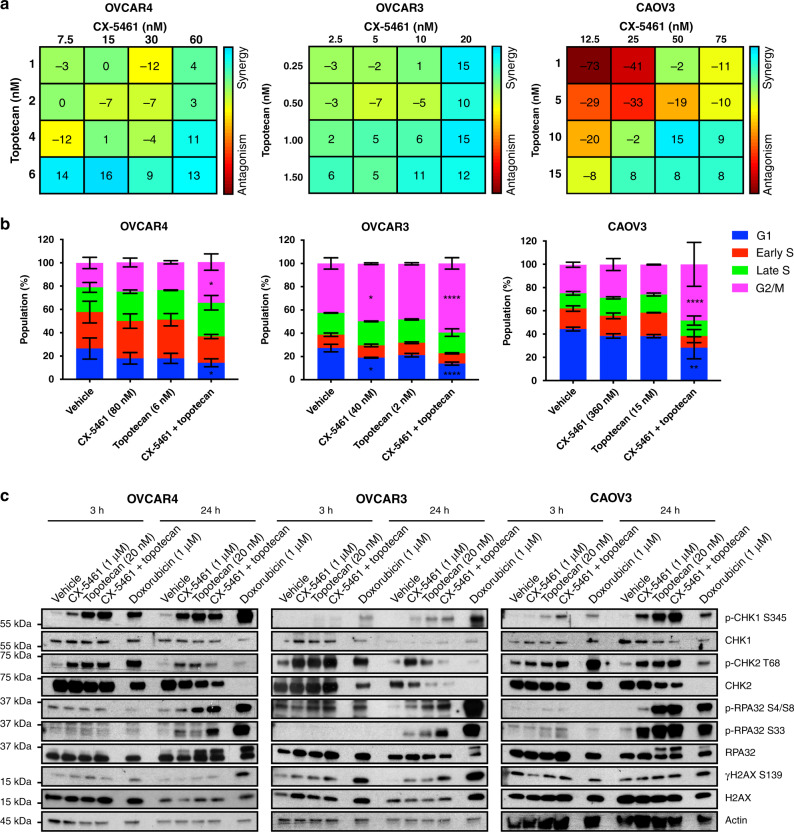
The TOP1 inhibitor topotecan synergises with CX-5461 in multiple HR-proficient HGSC cell lines. **a** 2D mapped surface Combenefit BLISS plots show synergy scores for OVCAR4, OVCAR3 and CAOV3 cells treated in drug checkerboard assays with the indicated concentrations of CX-5461 and topotecan for 9 days. The range of concentrations for each drug was determined based on the GI_25_–GI_50_ dose at 5 days (Table S7). **b** Cell cycle analysis of OVCAR4, OVCAR3 and CAOV3 cells treated with vehicle, CX-5461 (80 nM, OVCAR4; 40 nM, OVCAR3; 360 nM, CAOV3; GI_50_ for proliferation at 48 h), topotecan (6 nM, OVCAR4; 2 nM, OVCAR3; 15 nM, CAOV3; GI_50_ for proliferation at 48 h) or CX-5461 and topotecan. Quantification of the percentage of G1, early S, late S and G2/M phase cells is presented as mean ± SEM and statistical significance was determined by Kruskal–Wallis one-way ANOVA (*P < 0.05; **P < 0.01; ****P < 0.001; ****P < 0.0001). **c** Representative Western blot analysis of DDR signalling in OVCAR4, OVCAR3 and CAOV3 cells treated with vehicle, 1 μM CX-5461, 20 nM topotecan, CX-5461 and topotecan or 1 μM doxorubicin for 3 or 24 h (*n* = 3 experiments). The concentration for each drug was determined based on the TGI dose at 2 days. Actin was probed as a loading control.

The TOP1 inhibitor topotecan is used in salvage therapy for recurrent HGSC,^27^ with a Phase III clinical trial showing 29% and 6.5% objective response rates in platinum-sensitive and -refractory patients, respectively.^28^ Given that we observed combinatorial effects between CX-5461 and TOP1 depletion, we reasoned that CX-5461 could extend the utility of TOP1 inhibitor topotecan to target HR-proficient HGSC. We performed dose–response proliferation assays of three HR-proficient cell lines (OVCAR4, OVCAR3 and CAOV3) treated with CX-5461 or topotecan (Fig. S2A, B) to determine appropriate concentrations for comparison in drug checkerboard assays. Drug checkerboard assays demonstrated synergistic suppression of proliferation in all three cell lines (Fig. 2a).

To determine the cellular response contributing to the ability of the combination of CX-5461 and topotecan to inhibit cell proliferation, we performed cell cycle analyses of OVCAR4, OVCAR3 and CAOV3 cells. While treatment with CX-5461 or topotecan led to a trend toward an increase in G2/M cell cycle arrest compared to control, we observed significant enrichment in cells arresting in G2/M with the combination (Fig. 2b and Fig. S3A). In addition, we performed qPCR of *CDKN2A* (Fig. S3B) and *CDKN1A* (Fig. S3C), encoding for the cyclin-dependent kinase inhibitors p16 and p21, respectively, in OVCAR4 cells treated with vehicle, CX-5461, topotecan or the combination. We found that p21 but not p16 was induced in the combination, supporting p53-independent induction of p21 during the G2/M cell cycle arrest. We also examined the sub-G1 population to determine the impact of the treatments on cell death. OVCAR4 and CAOV3 but not OVCAR3 cells showed a significant increase in the sub-G1 population with combined CX-5461 and topotecan compared to control (Fig. S3D). The increase in cell death upon combined treatment was partially rescued by the caspase inhibitor Q-VD-OPh, implicating a role for apoptosis. Taken together, these data demonstrate that the combination of CX-5461 and topotecan can, at least in part, promote cell death but predominantly induces cell cycle arrest in HR-proficient HGSC cells.

### Combined treatment of CX-5461 and topotecan potentiates the DDR

**Fig. 3:**
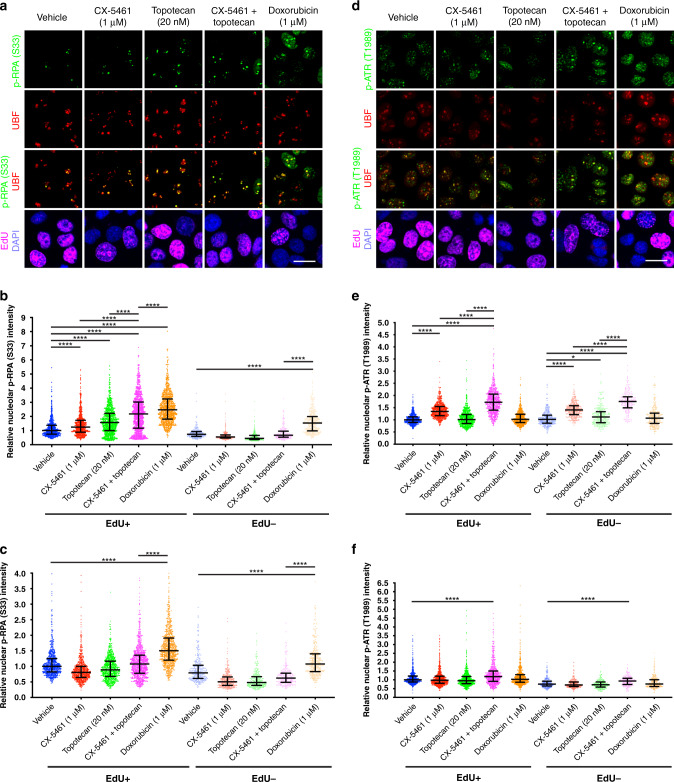
CX-5461 plus topotecan potentiates the recruitment of phosphorylated RPA and ATR to nucleoli. **a**–**f** OVCAR4 cells were treated with vehicle, 1 μM CX-5461, 20 nM topotecan, CX-5461 and topotecan or 1 μM doxorubicin for 3 h. **a**, **d** Representative images and quantification of relative (**b** and **e**) nucleolar or (**c** and **f**) nuclear fluorescence intensity normalised to the Vehicle EdU^+^ median for (**b**, **c**) phospho-RPA (S33) or (**e**, **f**) phospho-ATR (T1989), respectively. Cells were stained for UBF, DAPI or EdU to label nucleoli, nuclei or replicating cells, respectively. Scale bar is 20 μm. Data are presented as median with interquartile range and statistical significance for increased p-RPA or p-ATR was determined by Kruskal–Wallis one-way ANOVA (*P < 0.05; ****P < 0.0001).

We previously showed the p53-independent response to CX-5461 is mediated by ataxia telangiectasia mutated (ATM) and ATM and Rad3-related (ATR), both of which are critical regulators of the DDR pathway^15,29^ and are activated by various forms of DNA damage^30^; hence, we hypothesised that the DDR would be a key mediator of the G2/M cell cycle checkpoint response to CX-5461 plus topotecan. To test this hypothesis, we first assessed DDR activation in OVCAR4, OVCAR3 and CAOV3 cells upon vehicle, CX-5461, topotecan or combination treatment for 3 or 24 h using Western blotting of phosphorylated checkpoint kinase 1 (CHK1) and CHK2 (downstream effectors of ATR and ATM, respectively), phospho-replication protein A (RPA) (Ser4/Ser8) and phospho-RPA (Ser33), which protect single-stranded DNA (ssDNA) (markers of replication stress), and γH2AX (Ser139) (a marker of DNA damage) (Fig. 2c). Given recent reports identifying CX-5461 as a potential TOP2 poison,^16,31,32^ we also compared the response to the treatments with that of the TOP2 inhibitor doxorubicin. We observed increased phospho-CHK1 and phospho-CHK2 within 3 h upon treatment with CX-5461, topotecan or the combination compared to vehicle. At 24 h post treatment, in addition to increased activation of phospho-CHK1 and phospho-CHK2, among all three cell lines we consistently found increased phospho-RPA (Ser4/Ser8) and phospho-RPA (Ser33) with the combination treatment to the same level as that of doxorubicin compared to single-agent treatment, indicating significant induction of replication stress; however, we did not observe dramatic changes in global γH2AX (Ser139) by comparison to doxorubicin treatment (Fig. 3c).

As we have demonstrated, CX-5461 alone induces the formation of ssDNA and recruitment of phosphorylated RPA to the nucleolar periphery (the site of rDNA transcription),^15^ we first examined nucleolar localisation of phosphorylated RPA (Ser33) (Fig. 3a), a marker of stalled replication forks.^33^ We found a robust increase in phospho-RPA (Ser33) recruitment to the nucleoli marked by upstream binding transcription factor staining with the combination compared to single-agent treatment at 3 h, particularly in replicating cells marked by EdU incorporation (Fig. 3b). The increased phospho-RPA (Ser33) recruitment indicative of replication fork stalling was specific to nucleoli as we did not observe any changes in overall nuclear phospho-RPA (Ser33) intensity (Fig. 3c). In contrast, doxorubicin treatment resulted in a significant increase in phospho-RPA (Ser33) in nucleoli and across the genome. We also examined the localisation of phosphorylated ATR, which is recruited to RPA-coated ssDNA (Fig. 3d). Treatment with CX-5461 in combination with topotecan enhanced nucleolar (Fig. 3e) and global (Fig. 3f) phospho-ATR staining as compared to vehicle, although this was independent of DNA replication. Importantly, while nucleolar and global phospho-RPA (Ser33) was robustly induced upon doxorubicin treatment, phospho-ATR was not induced at this time point, indicating that the combination of CX-5461 and topotecan induces the DDR differently to doxorubicin. Together, these data demonstrate that CX-5461 plus topotecan induces the RPA/ATR/DDR axis and a robust G2/M cell cycle arrest and cell death via a mechanism distinct from the TOP2 poison doxorubicin.

### The combination of CX-5461 and topotecan enhances replication stress without inducing DNA strand breaks

**Fig. 4:**
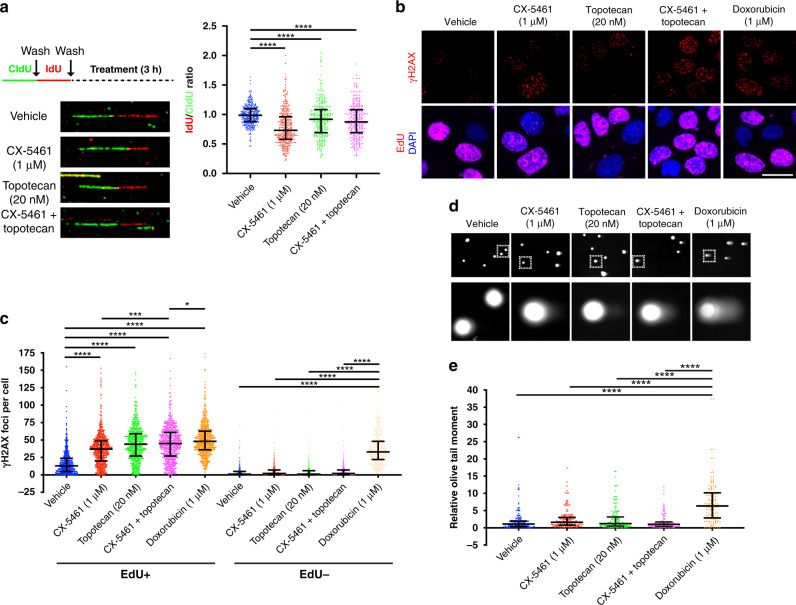
The combination of CX-5461 and topotecan does not result in enhanced replication fork degradation or DNA strand break generation. **a–e** OVCAR4 cells were treated with 1 μM CX-5461, 20 nM topotecan, CX-5461 and topotecan or 1 μM doxorubicin (as indicated) for 3 h. **a** Representative images and quantification of decreased IdU to CldU ratio is represented as median with interquartile range and statistical significance was determined by Kruskal–Wallis one-way ANOVA (****P < 0.0001). **b** Representative images and **c** quantification of foci number for γH2AX. Cells were stained for EdU and DAPI to label replicating cells and nuclei, respectively. Scale bar is 20 μm. Data are presented as median with interquartile range and statistical significance for increased foci per cell was determined by Kruskal–Wallis one-way ANOVA (*P < 0.05; ***P < 0.001, ****P < 0.0001). **d** Representative images and **e** quantification of alkaline comet assay is presented as median with interquartile range and statistical significance was determined by Kruskal–Wallis one-way ANOVA (**P < 0.01; ****P < 0.0001 from *n* = 3 experiments).

We have shown that the CX-5461-mediated DDR is associated with degradation of stalled replication forks and replication-dependent γH2AX foci formation indicative of DNA damage in HGSC cells.^15^ To examine the impact of the combination of CX-5461 and topotecan on replication fork stability, we performed DNA fibre analysis in OVCAR4 cells treated with vehicle, CX-5461, topotecan or the combination (Fig. 4a). In agreement with our previous observation in HR-proficient OVCAR8 cells,^15^ CX-5461 treatment-induced replication fork degradation in OVCAR4 cells, as indicated by a reduced IdU to CIdU ratio. Topotecan also increased replication fork destabilisation compared to control. However, the combination of CX-5461 and topotecan did not result in enhanced fork degradation compared with either drug alone. To examine the persistence of stalled replication forks, we assessed the levels of phosphorylated RPA (Ser4/Ser8) upon treatment with vehicle, CX-5461, topotecan or the combination at 24 h (Fig. S4A). While we did not observe marked changes in nucleolar phospho-RPA (Ser4/Ser8) (Fig. S4B), we observed a significantly greater increase in nuclear Ser4/Ser8 phosphorylation of RPA in cells treated with the CX-5461 plus topotecan combination compared to single-agent therapy (Fig. S4C), indicating that the combination treatment leads to enhanced persistent replication stress. The data, therefore, suggest that the CX-5461 and topotecan combination enhances replication stress via different effects of each drug on fork progression and stability, possibly through fork degradation by CX-5461 and fork stalling by TOP1 trapping.

To examine the effect of combining CX-5461 and topotecan on DNA damage, we examined γH2AX foci formation in OVCAR4 cells (Fig. 4b). Treatment with CX-5461, topotecan or the combination led to increased γH2AX foci formation compared to vehicle exclusively in S-phase EdU-positive cells (Fig. 4c). Interestingly, in EdU-positive cells treatment with the combination of CX-5461 and topotecan led to γH2AX foci formation almost to the same level as that observed upon doxorubicin treatment. However, doxorubicin treatment also induced γH2AX foci formation in EdU-negative cells, highlighting that activation of the DDR to the combination of CX-5461 and topotecan requires active DNA replication.

Replication stress due to stalled replication forks can induce ATR/ATM activation and γH2AX prior to the formation of DNA strand breaks.^34^ Furthermore, although phosphorylated H2AX is considered to be a marker of DNA damage, it does not always equate to DNA strand breakage.^35^ We performed comet assays of OVCAR4 cells after 3 h of treatment with vehicle, CX-5461, topotecan or the combination to test if the treatments that induced γH2AX foci levels were associated with DNA strand breaks (Fig. 4d). Alkaline comet assays, which can detect single-strand breaks, DSBs, DNA cross-linking and alkali labile sites,^36^ revealed that CX-5461 and topotecan alone or in combination did not induce comet tails. This was in striking contrast to doxorubicin, which induced prominent comet tails indicating extensive DNA breakage (Fig. 4e). Thus, the enhanced replication stress by the CX-5461 plus topotecan combination does not generate DNA strand breaks, which may be beneficial in reducing any potential genotoxicity of this therapeutic approach.

### CX-5461 plus topotecan inhibits clonogenic survival and blocks tumour growth

**Fig. 5:**
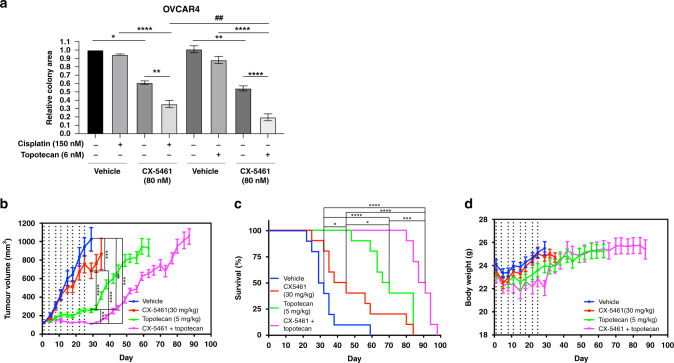
CX-5461 plus topotecan inhibits HR-proficient HGSC clonogenic survival and tumour growth. **a** Clonogenic survival assays for OVCAR4 cells treated with vehicle or approximate GI_50_ doses of CX-5461 in the absence or presence of the indicated concentrations of cisplatin or topotecan for 48 h. Drugs were washed out and cells were cultured for an additional 5 days, fixed and stained with crystal violet. Relative colony area normalised to vehicle without cisplatin is presented as mean ± SEM and statistical significance for the decrease in relative colony area was determined by one-way ANOVA (*P < 0.05; **P < 0.01; ****P < 0.0001; CX-5461 with cisplatin vs. CX-5461 with topotecan, ^##^*P* < 0.01). **b** Mean tumour volume of OVCAR3 flank tumours treated with vehicle, CX-5461 (30 mg/kg), topotecan (5 mg/kg) or CX-5461 (30 mg/kg) and topotecan (5 mg/kg) (*n* = 10 mice/group). Data are presented as mean ± SEM and statistical significance was determined on day 22 by one-way ANOVA (vehicle vs. CX-5461, topotecan or combination, CX-5461 vs. topotecan or combination, ****P < 0.0001; topotecan vs. combination, ***P < 0.001). **c** Kaplan–Meier survival curves of tumour-bearing mice. Statistical significance was determined by log-rank Mantel–Cox tests (vehicle vs. CX-5461, CX-5461 vs. topotecan, *P < 0.05; vehicle vs. topotecan or combination, CX-5461 vs. combination, ****P < 0.0001; topotecan vs. combination, ***P < 0.001). **d** Mouse body weight. Dashed line indicates day of drug dosing.

We have demonstrated recently that CX-5461 acts to overcome fork protection in olaparib-resistant HGSC cells.^15^ Cells resistant to PARP inhibitors are also tolerant to topotecan and cisplatin, indicating that stalled fork protection and stabilisation confer a general resistance to replication stress‐inducing chemotherapeutics.^3,4^ Nevertheless, to compare the cellular response of the CX-5461 plus topotecan combination therapy with standard HGSC platinum-based therapy, we assessed colony formation of OVCAR4 cisplatin-sensitive, olaparib-resistant cells (Fig. S2C, D) treated with vehicle, cisplatin, CX-5461, topotecan, CX-5461 plus cisplatin or CX-5461 plus topotecan, followed by drug washout (Fig. 5a). While we found a decrease in colony formation due to each drug treatment alone compared to control (Fig. 5a), the decrease in colony formation was significantly more robust with CX-5461 plus cisplatin or CX-5461 plus topotecan compared to single-agent treatment. Moreover, the combination of CX-5461 and topotecan led to a significantly greater decrease in colony formation when compared with CX-5461 plus cisplatin, highlighting CX-5461 plus topotecan as a promising alternative to standard chemotherapy.

To determine the impact on tumour growth in vivo, we established tumours from HR-proficient OVCAR3 cells harbouring amplified *CCNE1*, which confers primary treatment resistance and poor outcome of HGSC.^8^ We assessed tumour growth (Fig. 5b) and survival (Fig. 5c) in response to treatment with CX-5461 (30 mg/kg), topotecan (5 mg/kg) or CX-5461 plus topotecan. We chose a standard topotecan dose of 5 mg/kg, which is well below the dose reported to potentially result in haematologic toxicity (12.5 mg/kg).^37^ Mice were dosed twice weekly for 4 weeks and then monitored until the animals reached an ethical endpoint. CX-5461 or topotecan treatment alone delayed tumour growth and prolonged survival as compared to vehicle treatment. Strikingly, the combined treatment of CX-5461 and topotecan blocked tumour progression during the entirety of drug treatment, significantly extending survival. The combination was well tolerated with a mean weight loss <10% (Fig. 5d). Taken together, these data demonstrate that the combination of CX-5461 and topotecan robustly inhibits the growth of HR-proficient HGSC cells and tumours.

## Discussion

We and others have identified a new paradigm for treating oncogene-driven cancers by targeting ribosome biogenesis.^10,11,13–15,26,29,38–40^ We have demonstrated that CX-5461 can inhibit Pol I recruitment to rDNA,^10^ induce a ribosome biogenesis checkpoint in cells with intact p53^13^ and activate a p53-independent DDR.^29^ We have also exploited CX-5461’s ability to activate the p53-independent DDR and its different sensitivity profile to standard therapies to target HGSC.^15^ CX-5461 is synthetic lethal with HR deficiency in vitro and in vivo in HGSC via a mechanism that is distinct from PARP inhibitors.^15^ Here, we found TOP1 inhibition, which has been shown to be effective in HR-deficient disease,^41^ can cooperate with CX-5461 in HR-proficient HGSC. Thus, our findings can potentially expand the application of both CX-5461 and TOP1 inhibitors such as topotecan in clinical trials to the 50% of HGSC patients who have HR-proficient tumours and to patients with HR-deficient tumours that have acquired chemotherapeutic resistance due to restored HR.

Recently, several studies have identified that CX-5461 has a similar sensitivity profile to DNA TOP2 poisons^16,31,32^ and requires TOP2 for cytotoxicity.^31,32^ TOP2α is a component of the RNA polymerase I pre-initiation complex^42^ and it is possible that CX-5461’s ability to trap TOP2 can contribute to its 200-fold selectivity for Pol I transcription inhibition over the other RNA polymerases^10,43^ as well as its therapeutic efficacy across the genome. In contrast, through an independent approach utilising a genome-wide RNAi screen, we show CX-5461 has a different sensitivity profile to those recently reported for TOP2 poisons (Fig. 1c).^16,31,32^ We have identified that TOP1 inhibition synergises with CX-5461 to inhibit HR-proficient HGSC cell proliferation, a critical finding supported by other studies demonstrating decreased survival upon treatment with pyridostatin in HeLa cells^32^ or with CX-5461 in *Eμ-Myc* lymphoma^31^ depleted of TOP1. Both TOP1 and TOP2 can localise to rDNA and are involved in regulating torsional stress during transcription and DNA replication.^18,42^ However, the differences in how they do so could explain why deficiencies in TOP1 and TOP2 confer sensitivity and resistance, respectively, to CX-5461. Indeed, a report in yeast has shown that TOP1 plays a critical role behind elongating RNA Pol I, while TOP2 plays a more critical role in front of elongating Pol I.^44^ In addition, G-quadruplex stabilisers such as pyridostatin can induce TOP2-dependent DNA DSBs that are countered by TOP1, likely through TOP1’s ability to regulate negative supercoiling behind RNA Pol I.^32^ With the combination of CX-5461 and the TOP1 inhibitor topotecan in HR-proficient HGSC cells, we found robust nucleolar recruitment of phosphorylated RPA (Ser33) and ATR. We previously found that CX-5461 enhances rDNA chromatin accessibility to MNase.^29^ Thus, we hypothesise that topotecan’s ability to enhance negative torsional stress combined with increased chromatin accessibility upon treatment with CX-5461 contributes to enhanced nucleolar recruitment of activated RPA and ATR, leading to a robust nucleolar and global DDR in a manner distinct to the TOP2 poison doxorubicin. Future studies utilising chromatin immunoprecipitation and electron microscopy of TOP1 and TOP2 at rDNA will be important to further elucidate the spatial mechanisms by which TOP1 and TOP2 confer sensitivity and resistance to CX-5461.

Intriguingly, we found either depletion of TOP1 or treatment with the TOP1 inhibitor topotecan could enhance the antiproliferative response to CX-5461 in HR-proficient HGSC. Previous studies have shown that TOP1 deficiency can result in the accumulation of stalled replication forks^45,46^ and sublethal doses of TOP1 inhibitors can slow replication fork progression and induce reversed forks.^47,48^ We have also shown that CX-5461 induces MRE11-dependent degradation of replication forks.^15^ Therefore, we propose that fork degradation by CX-5461 and fork stalling by TOP1 depletion/inhibition together contribute to enhanced persistent replication stress. Overall, the combination of CX-5461 and topotecan leads to enhanced replication stress and DDR without eliciting DNA breakage, highlighting a lower potential for genotoxicity that normally leads to adverse side effects from damaging normal cells. Nonetheless, even in the absence of DNA breakage, the combination promotes a robust G2/M arrest that impairs clonogenic survival and maintains potent anti-tumour activity.

As a complement to our previous findings that CX-5461 has efficacy in HGSC harbouring HR deficiency and high MYC activity driving Pol I transcription and/or MYC-driven global transcription and replication stress, here we identify a potent anti-proliferative combination between CX-5461 and TOP1 inhibition in HR-proficient HGSC. We show that CX-5461 and the TOP1 inhibitor topotecan cooperate by enhancing DDR and replication stress without generating DNA strand breaks, leading to a robust G2/M cell cycle arrest, inhibition of clonogenic survival and tumour growth in vivo. Topotecan as a salvage therapy is used at maximum-tolerated doses, which can cause myelosuppression that limits its use clinically. However, we found that using low-dose topotecan cooperates with CX-5461. We suggest that further investigation into modifying dosing strategies with TOP1 inhibitors such as topotecan will facilitate using this class of drugs in combination with CX-5461 as a promising therapeutic option for HGSC patients.

## Supplementary information

Supplementary Information

## Data Availability

The datasets are presented in the additional supporting files.
