# Supplementary material for: The RNA polymerase I transcription inhibitor CX-5461 cooperates with topoisomerase 1 inhibition by enhancing the DNA damage response in homologous recombination-proficient high-grade serous ovarian cancer
Source: Br J Cancer. 2020 Nov 11;124(3):616–27. doi: 10.1038/s41416-020-01158-z (PMC7851142; doi:10.1038/s41416-020-01158-z)
Supplement: Supplementary file 1 — Supplementary Information [file 41416_2020_1158_MOESM1_ESM.pdf]

Supplementary Figure Legends

Figure S1. Depletion of BRCA2 or TOP1 sensitizes HSGC cells to CX-5461.

(A and B) OVCAR4 cells expressing doxycycline-inducible shRNA against BRCA2 in the absence (-shBRCA2) or presence (+shBRCA2) of 0.2 µg/mL doxycycline for 48 h were treated with vehicle or 80 nM CX-5461 for 48 h. **(A)** Quantification of relative BRCA2 mRNA expression is presented as mean ± SD and statistical significance was determined by two-tailed unpaired *t* test (***, P = 0.0001 from n=2 experiments). **(B)** Dose response curves after 48 h treatment and the summary table shows the 50% inhibition of cell growth (GI_{50}) and total growth inhibition (TGI) doses as median with interquartile range (n=3 experiments). **(C)** Western blot of TOP1 expression in OVCAR4 cells 14 d post-nucleofection with control or TOP1 sgRNA. Actin was probed as a loading control. **(D)** Representative images of clonogenic survival assay for OVCAR4 cells treated with vehicle, 80 nM CX-5461, 6 nM topotecan, CX-5461 and topotecan, 150 nM cisplatin or 3 µM olaparib for 48 h. Drugs were washed out and cells were cultured for an additional 5 d, fixed and stained with crystal violet. Quantification of decreased relative colony area to control knockout cells treated with vehicle is presented as mean ± SEM and statistical significance was determined by one-way ANOVA (**, $P < 0.01$; ****, $P < 0.0001$). Statistical significance for decreased relative colony area (control or TOP1 knockout cells treated with CX-5461) was determined by one-way ANOVA (##, $P < 0.01$; ####, $P < 0.0001$). **(E and F)** OVCAR3 cells were analyzed as in **(C and D)**.

Figure S2. Proliferation assays of HR-proficient cell lines in response to drug therapies.

Dose response curves were generated from HR-proficient OVCAR4, OVCAR3 and CAOV3 cells treated with (**A)** CX-5461, **(B)** topotecan, **(C)** cisplatin and **(D)** olaparib for 5 d, as measured by cell number (DAPI staining) and % cell proliferation is presented as mean ± SEM with an ordinary nonlinear regression fit. The 50% inhibition of cell growth (GI_{50}) and total growth inhibition (TGI) doses were determined by curve interpolation and are shown as median with interquartile range (n=3 experiments).

Figure S3. The combination of CX-5461 and topotecan leads to cell cycle arrest and cell death in HR-proficient HGSC.

(A) FACS plots of cell cycle analysis of OVCAR4, OVCAR3 and CAOV3 cells treated with Vehicle, CX-5461 (80 nM, OVCAR4; 40 nM, OVCAR3; 360 nM, CAOV3; GI_{50} for proliferation at 48 h), Topotecan (6 nM, OVCAR4; 2 nM, OVCAR3; 15 nM, CAOV3; GI_{50} for proliferation at 48 h) or CX-5461 and Topotecan. **(B and C)** Cells were treated as indicated and qPCR of **(B)** *CDKN2A* or **(C)** *CDKN1A* was performed. Quantification of relative RNA expression from is presented as mean ± SEM and statistical significance was determined by Kruskal-Wallis one-way ANOVA (**, $P <$ 0.01; ****, $P < 0.001$ from n=3 experiments). **(D)** Quantification of the percentage of Sub G1 cells treated with control (DMSO) or 10 µM Q-VD-OPh is presented as mean ± SEM and statistical significance was determined by one-way ANOVA (*, $P < 0.05$; **, $P < 0.01$; ***, $P < 0.001$; ****, $P < 0.0001$), (##, P < 0.01; ###, P < 0.001 for Q-VD-OPh as compared with DMSO control).

Figure S4. Treatment with the combination of CX-5461 and topotecan induces global replication stress.

(A and B) OVCAR4 cells were treated with vehicle, 1 μM CX-5461, 20 nM topotecan, CX-5461 and topotecan for 24 h. **(A)** Representative images and quantification of **(B)** relative nucleolar or **(C)** nuclear fluorescence intensity for phospho-RPA (Ser4/Ser8). Cells were stained for UBF and DAPI to label nucleoli and nuclei, respectively. Scale bar is 20 μm. Data are presented as median with interquartile range and statistical significance for increased p-RPA was determined by Kruskal-Wallis one-way ANOVA (*, $P < 0.05$; **, $P < 0.01$; ****, $P < 0.0001$).

Yan et al. Figure S1

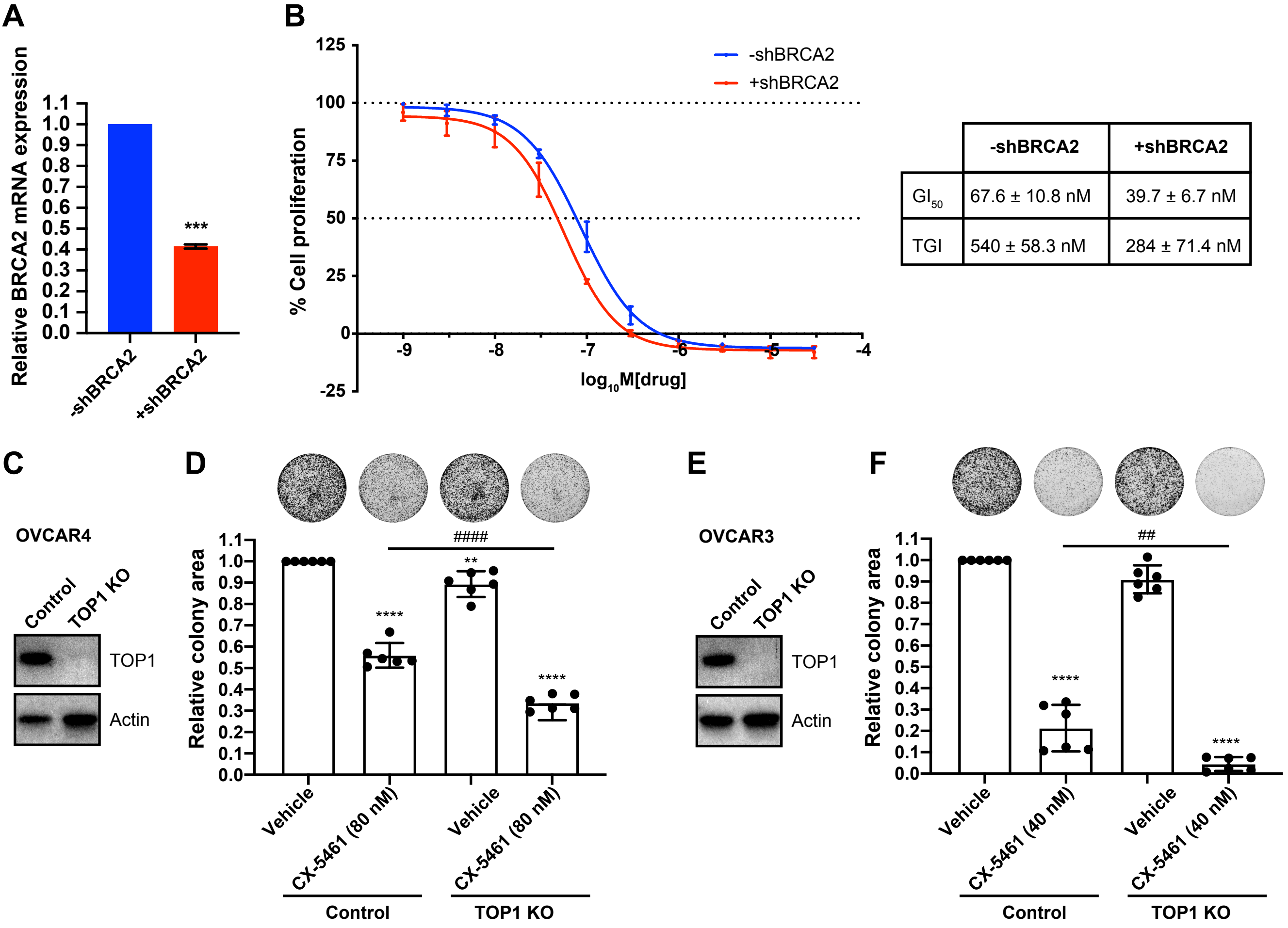

	-shBRCA2	+shBRCA2
GI_{50}	67.6 ± 10.8 nM	39.7 ± 6.7 nM
TGI	540 ± 58.3 nM	284 ± 71.4 nM

Yan et al. Figure S2

A

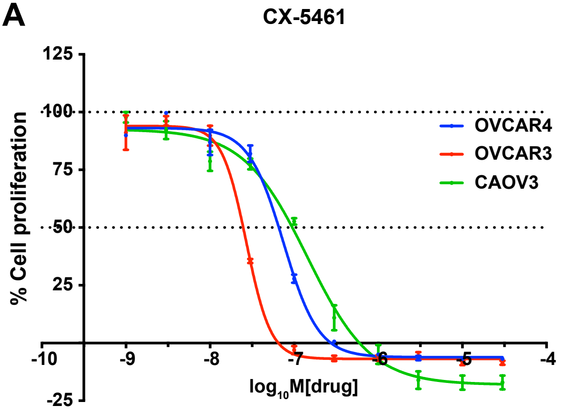

	OVCAR4	OVCAR3	CAOV3
GI_{50} (nM)	65.6 ± 4.1	25.2 ± 1.7	97 ± 8.5
TGI (nM)	279 ± 34	71 ± 18	667 ± 183

B

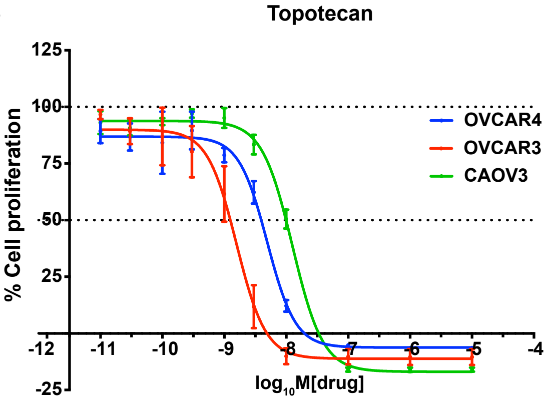

	OVCAR4	OVCAR3	CAOV3
GI_{50} (nM)	4.0 ± 0.4	1.2 ± 0.4	9.9 ± 1.0
TGI (nM)	20 ± 2.5	5.5 ± 0.9	32.6 ± 1.3

C

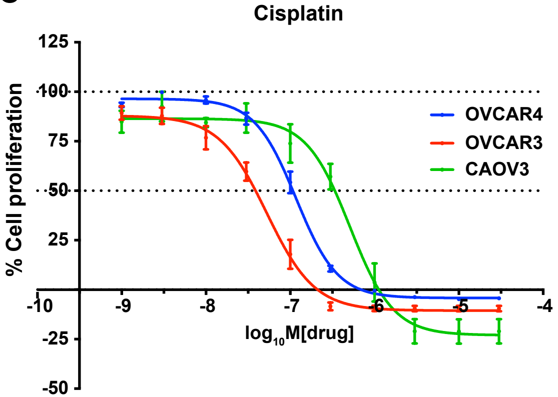

	OVCAR4	OVCAR3	CAOV3
GI_{50} (nM)	108 ± 13	40 ± 8	330 ± 89
TGI (µM)	0.68 ± 0.04	0.22 ± 0.06	1.37 ± 0.34

D

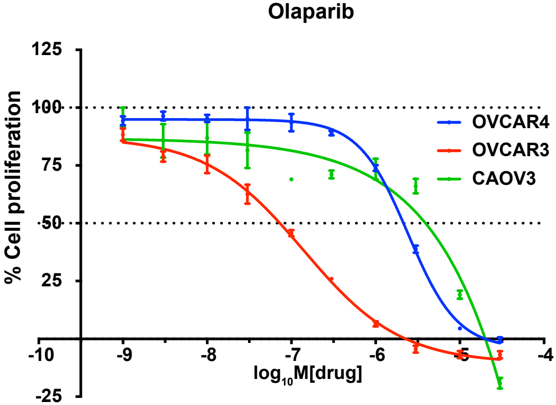

	OVCAR4	OVCAR3	CAOV3
GI_{50} (nM)	2144 ± 110	73 ± 11	4405 ± 833
TGI (µM)	20.94 ± 3.54	2.59 ± 0.78	17.98 ± 2.23

Yan et al. Figure S3

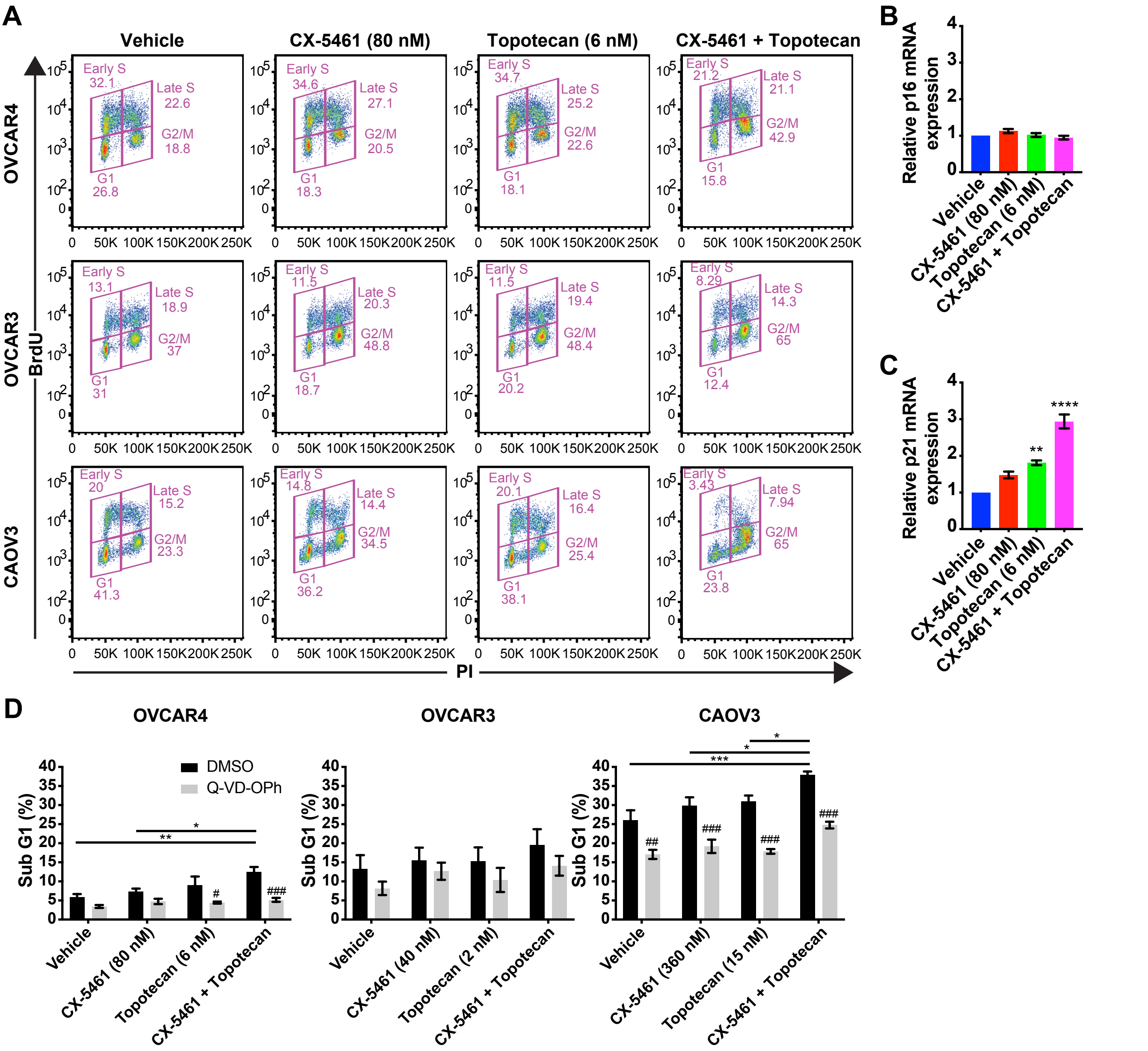

Yan et al. Figure S4

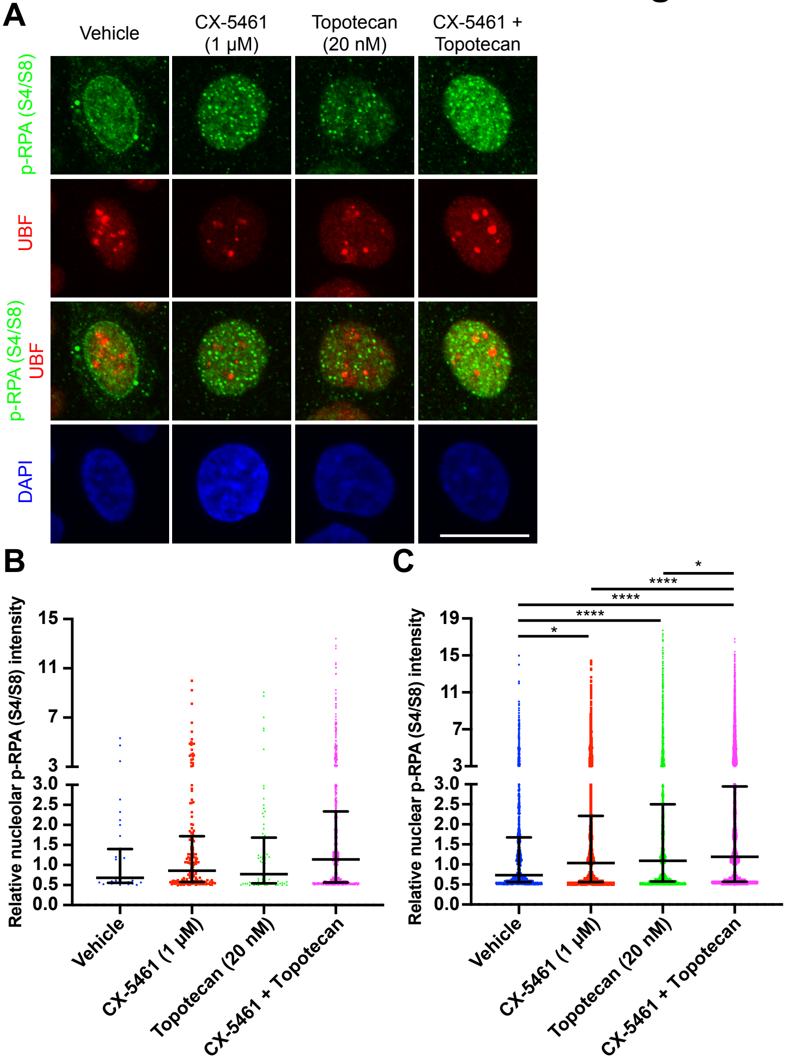

Supplementary Table S1. Antibodies used in this study.

Primary Antibodies

**Target**	**Clone**	**Species**	**Company**	**Cat. No.**	**Dilution**	**Application**
Phospho-CHK1 (Ser345)	133D3	Rabbit	Cell Signaling Technology	2348	1:1000	WB
CHK1		Rabbit	Santa Cruz Biotechnology	sc-7898	1:1000	WB
Phospho-CHK2 (Thr68)	C13C1	Rabbit	Cell Signaling Technology	2197	1:1000	WB
CHK2	D9C6	Rabbit	Cell Signaling Technology	6334	1:1000	WB
Phospho-ATR (Ser1989)		Rabbit	GeneTex	GTX128145	1:100	IF
ɣH2AX		Rabbit	Abcam	ab81299	1:2000, 1:100	WB, IF
H2AX		Rabbit	Abcam	ab20669	1:2000	WB
Phospho-RPA2 (Ser33)		Rabbit	Novus Biologicals	NB100-544	1:2000, 1:200	WB, IF
Phospho-RPA32 (Ser4/Ser8)		Rabbit	Bethyl Laboratories	A300-254A	1:1000, 1:100	WB, IF
RPA32/RPA2	4E4	Rat	Cell Signaling Technology	2208	1:1000	WB
RAD51		Rabbit	Abcam	ab63801	1:100	IF
TOP1		Rabbit	Abcam	ab3825	1:1000	WB
UBF	F-9	Mouse	Santa Cruz Biotechnology	sc-13125	1:100	IF
UBF	WT1F	Rabbit	in-house		1:400	IF
Tubulin	B-5-1-2	Mouse	Sigma-Aldrich	T5168	1:10000	WB
β-Actin HRP conjugate	AC-15	Mouse	Abcam	ab49900	1:20000	WB
BrdU	B44	Mouse	BD Biosciences	347580	1:50	FACS

Secondary Antibodies

anti-mouse IgG (H+L) HRP conjugate	Goat	Bio-Rad Laboratories	172-1011	1:5000	WB
anti-rabbit IgG (H+L) HRP conjugate	Goat	Bio-Rad Laboratories	170-6515	1:5000	WB
anti-rat IgG (H+L) HRP conjugate	Goat	Abcam	ab97057	1:5000	WB
anti-mouse IgG (H+L) Alexa Fluor 488	Goat	Invitrogen	A-11001	1:500	IF
anti-rabbit IgG (H+L) Alexa Fluor 594	Goat	Invitrogen	A-11012	1:500	IF
anti-mouse IgG (H+L) Alexa Fluor 488	Donkey	Invitrogen	A-21202	1:100	FACS

Supplementary Table S2. Primers used for qRT-PCR

Gene	Forward (5'-3')	Reverse (5'-3')
NONO	CATCAAGGAGGCTCGTGAGAAG	TGGTTGTGCAGCTCTTCCATCC
BRCA2	GCGCGGTTTTTGTCAGCTTA	TGGTCCTAAATCTGCTTTGTTGC
CDKN2A	GGGGGCACCAGAGGCAGT	GGTTGTGGCGGGGGCAGTT
CDKN1A	GGCAGACCAGCATGACAGATT	GCGGATTAGGGCTTCCTCTT

Supplementary Table S3. Primary screen results.

control.average.cell.count.normalised.to.OTP: relative cell number of siRNA treated cells compared to OTP-NT transfected cell w/o CX-5461 treatment; **drug.average.cell.count.normalised.to.OTP:** relative cell number of siRNA+CX-5461 treated cells compared to OTP-NT transfected cell w/ CX-5461 treatment; **combined.survival.rate:** relative cell number of siRNA+CX-5461 treated cells compared to OTP-NT transfected cell w/o CX-5461 treatment; **reduction.of.cell.confluence:** (control.average.cell.count.normalised.to.OTP - drug.average.cell.count.normalised.to.OTP)*100

gene.name	gene.name.alias	description	control.average.cell.count.normalised.to.OTP	drug.average.cell.count.normalised.to.OTP	CDI	combined.survival.rate
BLK	BLK;MGC10442;MODY11	B lymphoid tyrosine kinase	1.21	0.58	0.48	0.48
EVC	EVC;DWF-1;EVC1;EVCL;MGC105107	Ellis van Creveld syndrome	0.98	0.48	0.5	0.38
FAM3D	FAM3D;EF7;OIT1	family with sequence similarity 3, member D	1.1	0.56	0.51	0.39
CACNA1G	CACNA1G;Ca(V)T.1;Cav3.1;MGC117234;NBR13	calcium channel, voltage-dependent, T type, alpha 1G subunit	1	0.5	0.51	0.4
DNAJC24	DNAJC24;DPH4;JJJ3;ZCSL3	DnaJ (Hsp40) homolog, subfamily C, member 24	1	0.46	0.51	0.35
FAM167B	FAM167B;C1orf90;MGC10820	family with sequence similarity 167, member B	0.94	0.54	0.52	0.35
CTNNBIP1	CTNNBIP1;ICAT;MGC15093	catenin, beta interacting protein 1	1.14	0.62	0.53	0.44
KBTBD2	KBTBD2;BKLHD1	kelch repeat and BTB (POZ) domain containing 2	1.12	0.57	0.53	0.45
POT1	POT1;DKFZp586D211;HPOT1	protection of telomeres 1 homolog (S. pombe)	0.88	0.46	0.53	0.32
GBGT1	GBGT1;A3GALNT;FS;MGC44848;UNQ2513	globoside alpha-1,3-N-acetylgalactosaminyltransferase 1	1.05	0.56	0.57	0.44
DLGAP2	DLGAP2;DAP2;SAPAP2	discs, large (Drosophila) homolog-associated protein 2	0.96	0.53	0.57	0.38
SLC38A5	SLC38A5;JM24;SN2;SNAT5;pp7194	solute carrier family 38, member 5	0.92	0.51	0.57	0.38
EHD2	EHD2;FLJ96617;PAST2	EH-domain containing 2	1.34	0.8	0.58	0.6
CLIC5	CLIC5;CLIC5B;FLJ90663;MST130;MSTP130;dJ447E21.4	chloride intracellular channel 5	1.25	0.76	0.58	0.59
PRRG3	PRRG3;MGC149510;MGC156177;PRGP3;TMG3	proline rich Gla (G-carboxyglutamic acid) 3 (transmembrane)	0.96	0.58	0.59	0.4
TMEM150A	TMEM150A;FLJ90024;TM6P1;TMEM150	transmembrane protein 150A	0.94	0.58	0.59	0.42
PUM2	PUM2;FLJ36528;KIAA0235;MGC138251;MGC138253;PUMH2;PUML2	pumilio homolog 2 (Drosophila)	0.91	0.52	0.59	0.4
SPIN2B	SPIN2B;SPIN-2;SPIN2_duplicate;dJ323P24.2	spindlin family, member 2B	1.03	0.58	0.6	0.47
DDX39B	DDX39B;BAT1;D6S81E;UAP56	DEAD (Asp-Glu-Ala-Asp) box polypeptide 39B	0.99	0.59	0.6	0.45
C1QTNF3	C1QTNF3;C1ATNF3;CORCS;CORS;CORS-26;CORS26;CTRP3;FLJ37576	C1q and tumor necrosis factor related protein 3	0.95	0.56	0.6	0.45
ACY3	ACY3;ACY-3;HCBP1;MGC9740	aspartoacylase (aminocyclase) 3	0.93	0.56	0.6	0.38
ANKRD36	ANKRD36;FLJ33607;KIAA1641;MGC167812;UNQ2430	ankyrin repeat domain 36	1.38	0.76	0.61	0.6
MYF5	MYF5;bHLHc2	myogenic factor 5	1.14	0.68	0.61	0.51
TMEM179	TMEM179;C14orf90;FLJ42486;TMEM179A	transmembrane protein 179	1.13	0.62	0.61	0.48
EVC2	EVC2;LBN	Ellis van Creveld syndrome 2	1.09	0.69	0.61	0.47
RAD54L	RAD54L;HR54;RAD54A;hHR54;hRAD54	RAD54-like (S. cerevisiae)	1.09	0.7	0.61	0.46
CPNE7	CPNE7;MGC34192	copine VII	0.87	0.52	0.61	0.39
HOXB1	HOXB1;HOX2;HOX2I;Hox-2.9;MGC116843;MGC116844;MGC116845	homeobox B1	1.12	0.77	0.62	0.53
PTX4	PTX4;C16orf38	pentraxin 4, long	0.94	0.61	0.62	0.41
GMPR	GMPR;GMPR1	guanosine monophosphate reductase	1.09	0.66	0.63	0.48
UNC5C	UNC5C;UNC5H3	unc-5 homolog C (C. elegans)	1.05	0.7	0.63	0.54
NUDT9	NUDT9;MGC3037;NUDT10	nudix (nucleoside diphosphate linked moiety X)-type motif 9	1.09	0.7	0.64	0.54
FCRL6	FCRL6;FLJ16056;FcRH6	Fc receptor-like 6	1	0.64	0.64	0.46
TRMT12	TRMT12;FLJ20772;TRM12;TYW2	tRNA methyltransferase 12 homolog (S. cerevisiae)	0.99	0.66	0.64	0.48
DUSP23	DUSP23;DUSP25;FLJ20442;LDP-3;MOSP;RP11-190A12.1;VHZ	dual specificity phosphatase 23	0.99	0.64	0.64	0.5
C12orf59	C12orf59;FLJ31166;MGC111385	chromosome 12 open reading frame 59	0.94	0.64	0.64	0.5
VTI1A	VTI1A;MVti1;VTI1RP2;Vti1-rp2	vesicle transport through interaction with t-SNAREs homolog 1A (yeast)	0.85	0.56	0.64	0.44
ZC3H11A	ZC3H11A;DKFZp686D03108;DKFZp686F14109;DKFZp781G2455;KIAA0663;ZC3HDC11A	zinc finger CCCH-type containing 11A	1.36	0.88	0.65	0.63
CDKAL1	CDKAL1;FLJ20342;FLJ46705;MGC75469	CDK5 regulatory subunit associated protein 1-like 1	1.34	0.85	0.65	0.64
ZNF296	ZNF296;ZFP296;ZNF342	zinc finger protein 296	1.15	0.72	0.65	0.55
AP1AR	AP1AR;2C18;C4orf16;GBAR;gamma-BAR	adaptor-related protein complex 1 associated regulatory protein	1.08	0.77	0.65	0.51
O3FAR1	O3FAR1;DKFZp686F0824;GPR120;GPR129;GT01;MGC119984;PGR4	omega-3 fatty acid receptor 1	1.07	0.67	0.65	0.61
STRADA	STRADA;FLJ90524;LYK5;NY-BR-96;PMSE;STRAD;Stlk	STE20-related kinase adaptor alpha	1.04	0.73	0.65	0.52
GPC1	GPC1;FLJ38078;glypican	glypican 1	1.03	0.7	0.65	0.48
LHFPL2	LHFPL2;DKFZp781E0375;KIAA0206	lipoma HMGIC fusion partner-like 2	1	0.64	0.65	0.48
SIDT1	SIDT1;B830021E24Rik;FLJ20174;SID-1;SID1	SID1 transmembrane family, member 1	0.99	0.62	0.65	0.47
KHDC1	KHDC1;C6orf148;MGC10818;NDG1;bA257K9.4	KH homology domain containing 1	0.98	0.7	0.65	0.51
CARKD	CARKD;FLJ10769;FLJ34548;FLJ52550;LP3298	carbohydrate kinase domain containing	0.98	0.62	0.65	0.47
UNCX	UNCX;UNCX4.1	UNC homeobox	0.97	0.62	0.65	0.46
ARFGAP2	ARFGAP2;FLJ14576;FLJ26000;IRZ;NBLA10535;ZFP289;ZNF289	ADP-ribosylation factor GTPase activating protein 2	0.94	0.64	0.65	0.52
CCL11	CCL11;MGC22554;SCYA11	chemokine (C-C motif) ligand 11	0.92	0.62	0.65	0.46
DAPP1	DAPP1;BAM32;DKFZp667E0716	dual adaptor of phosphotyrosine and 3-phosphoinositides	0.85	0.57	0.65	0.39
TMOD3	TMOD3;UTMOD	tropomodulin 3 (ubiquitous)	1.23	0.83	0.66	0.63
PTPN7	PTPN7;BPTP-4;FLJ18703;HEPTP;LC-PTP;LPTP;PTPNI	protein tyrosine phosphatase, non-receptor type 7	1.1	0.71	0.66	0.56
DNAJC21	DNAJC21;DNAJA5;GS3;JJJ1	DnaJ (Hsp40) homolog, subfamily C, member 21	1.07	0.73	0.66	0.53
HLA-DPB1	HLA-DPB1;DPB1;FLJ57621;FLJ61008;HLA-DP1B;HLA-DPB	major histocompatibility complex, class II, DP beta 1	1.05	0.64	0.66	0.51
ZNF274	ZNF274;DKFZp686K08243;FLJ37843;HFB101;ZF2;ZKSCAN19	zinc finger protein 274	1.05	0.67	0.66	0.51
MT1M	MT1M;MGC118949;MGC40498;MT-1M;MT-IM;MT1;MT1K	metallothionein 1M	0.94	0.66	0.66	0.46
CCDC24	CCDC24;MGC45441;RP5-1198O20.2	coiled-coil domain containing 24	0.86	0.57	0.66	0.43
NAGPA	NAGPA;APAA;UCE	N-acetylglucosamine-1-phosphodiester alpha-N-acetylglucosaminidase	0.86	0.55	0.66	0.41
MLLT10	MLLT10;AF10;DKFZp686E10210;MGC75086	myeloid/lymphoid or mixed-lineage leukemia (trithorax homolog, Drosophila); translocated to, 10	1.19	0.78	0.67	0.7
HLA-DPA1	HLA-DPA1;DP(W3);DP(W4);HLA-DP1A;HLADP;HLASB;PLT1	major histocompatibility complex, class II, DP alpha 1	1.15	0.75	0.67	0.59
NES	NES;FLJ21841	nestin	1.02	0.67	0.67	0.56
PALLD	PALLD;CGI151;FLJ22190;FLJ38193;FLJ39139;FLJ61376;KIAA0992;MYN;PNCA1;SIH002	palladin, cytoskeletal associated protein	1.01	0.72	0.67	0.51
ZNF673	ZNF673;FLJ20344	zinc finger family member 673	0.98	0.67	0.67	0.5
CD83	CD83;BL11;HB15	CD83 molecule	0.88	0.6	0.67	0.46
GRIK5	GRIK5;EAA2;GRIK2;KA2	glutamate receptor, ionotropic, kainate 5	1.22	0.87	0.68	0.68
ESRP1	ESRP1;FLJ20171;RBM35A;RMB35A	epithelial splicing regulatory protein 1	1.15	0.83	0.68	0.58
ANXA13	ANXA13;ANX13;ISA;MGC150460	annexin A13	1.14	0.75	0.68	0.57
FOXN4	FOXN4;FLJ35967	forkhead box N4	1.08	0.77	0.68	0.51
CT47A4	CT47A4;CT47.4;CT47A1;CT47A10;CT47A11;CT47A12;CT47A2;CT47A3;CT47A5;CT47A6;CT47A7;CT47A8;CT47A9	cancer/testis antigen family 47, member A4	1.08	0.7	0.68	0.54
AKR7A2	AKR7A2;AFAR;AFAR1;AFB1-AR1;AKR7	aldo-keto reductase family 7, member A2 (aflatoxin aldehyde reductase)	1.06	0.76	0.68	0.5
UFC1	UFC1;HSPC155	ubiquitin-fold modifier conjugating enzyme 1	1.04	0.77	0.68	0.57
HHIPL1	HHIPL1;KIAA1822;UNQ9245	HHIP-like 1	1.01	0.68	0.68	0.51
SLK	SLK;KIAA0204;LOSK;MGC133067;STK2;bA16H23.1;se20-9	STE20-like kinase	1	0.72	0.68	0.52
CNNM4	CNNM4;ACDP4;FLJ37746;FLJ42791;KIAA1592	cyclin M4	0.99	0.65	0.68	0.52
GALNT14	GALNT14;FLJ12691;FLJ13977;GALNT15;GalNac-T10;GalNac-T14	UDP-N-acetyl-alpha-D-galactosamine:polypeptide N-acetylgalactosaminyltransferase 14 (GalNAc-T14)	0.96	0.66	0.68	0.51
EMR1	EMR1;TM7LN3	egf-like module containing, mucin-like, hormone receptor-like 1	0.88	0.59	0.68	0.53
CHD7	CHD7;FLJ20357;FLJ20361;IS3;KAL5;KIAA1416	chromodomain helicase DNA binding protein 7	0.86	0.56	0.68	0.44
ALPK1	ALPK1;8430410J10Rik;FLJ22670;KIAA1527;LAK	alpha-kinase 1	1.22	0.9	0.69	0.64
GIF	GIF;IF;IFMH;INF;TCN3	gastric intrinsic factor (vitamin B synthesis)	1.14	0.8	0.69	0.63
PRAMEF3	PRAMEF3	PRAME family member 3	1.12	0.84	0.69	0.56
PPP1CB	PPP1CB;MGC3672;PP-1B;PP1beta;PPP1CD	protein phosphatase 1, catalytic subunit, beta isozyme	1.1	0.75	0.69	0.59
ZNF77	ZNF77;pT1	zinc finger protein 77	1.09	0.74	0.69	0.55
CBX8	CBX8;PC3;RC1	chromobox homolog 8	1.09	0.8	0.69	0.6
KCNA6	KCNA6;FLJ25134;HBK2;KV1.6	potassium voltage-gated channel, shaker-related subfamily, member 6	1.04	0.71	0.69	0.55
MAGEB4	MAGEB4;CT3.6;MGC33144	melanoma antigen family B, 4	1.01	0.74	0.69	0.54
SPIN1	SPIN1;SPIN	spindlin 1	0.96	0.68	0.69	0.51
SCGN	SCGN;CALBL;DJ501N12.8;SECRET;SEGN;setagin	secretagogin, EF-hand calcium binding protein	0.88	0.63	0.69	0.42
DENND5B	DENND5B;DKFZp686P1174;FLJ41648;FLJ43333;MGC24039	DENN/MADD domain containing 5B	0.88	0.54	0.69	0.45
CPM	CPM	carboxypeptidase M	0.86	0.54	0.69	0.41
OR5M8	OR5M8;OR11-194	olfactory receptor, family 5, subfamily M, member 8	1.44	0.98	0.7	0.75
KCNJ14	KCNJ14;IRK4;KIAA1942;KIR2.4;MGC46111	potassium inwardly-rectifying channel, subfamily J, member 14	1.36	1	0.7	0.77
ROCK1	ROCK1;MGC131603;MGC43611;P160ROCK;PRO0435;ROCK-I	Rho-associated, coiled-coil containing protein kinase 1	1.26	0.88	0.7	0.73
KRTAP13-3	KRTAP13-3;KAP13.3;MGC119828	keratin associated protein 13-3	1.19	0.84	0.7	0.59
MORC4	MORC4;FLJ11565;ZCW4;ZCWCC2;dJ75H8.2	MORC family CW-type zinc finger 4	1.14	0.86	0.7	0.57
UBE2H	UBE2H;E2-20K;UBC8;UBCH;UBCH2	ubiquitin-conjugating enzyme E2H	1.14	0.74	0.7	0.55
SCYL1	SCYL1;GKLP;MGC78454;NKTL;NTKL;P105;TAPK;TEIF;TRAP	SCY1-like 1 (S. cerevisiae)	1.13	0.84	0.7	0.6
SPRR2B	SPRR2B	small proline-rich protein 2B	1.09	0.78	0.7	0.54
TSPAN16	TSPAN16;TM-8;TM4-B;TM4SF16	tetraspanin 16	1.06	0.74	0.7	0.52
KCNE1	KCNE1;FLJ18426;FLJ38123;FLJ94103;ISK;JLNS;JLNS2;LQT2/5;LQT5;MGC33114;MinK	potassium voltage-gated channel, Isk-related family, member 1	1.05	0.76	0.7	0.59
WWP2	WWP2;AIP2;WWp2-like	WW domain containing E3 ubiquitin protein ligase 2	1.04	0.66	0.7	0.5
SIRT2	SIRT2;FLJ35621;FLJ37491;SIR2;SIR2L;SIR2L2	sirtuin 2	1.04	0.68	0.7	0.6
C22orf36	C22orf36;MGC131773	chromosome 22 open reading frame 36	1.03	0.7	0.7	0.57
NOMO3	NOMO3;Nomo	NODAL modulator 3	1.01	0.63	0.7	0.53
C20orf152	C20orf152;MGC138880;dJ954P9.1	chromosome 20 open reading frame 152	0.98	0.66	0.7	0.51
EID3	EID3;FLJ25832;NSE4B;NSMCE4B	EP300 interacting inhibitor of differentiation 3	0.9	0.62	0.7	0.47
CT47A9	CT47A9;CT47.9;CT47A1;CT47A10;CT47A11;CT47A12;CT47A2;CT47A3;CT47A4;CT47A5;CT47A6;CT47A7;CT47A8	cancer/testis antigen family 47, member A9	0.89	0.6	0.7	0.47
FAM190B	FAM190B;FLJ14262;FLJ25809;Gcap14;KIAA1128;bA486O22.1	family with sequence similarity 190, member B	1.32	0.99	0.71	0.73
AGMO	AGMO;FLJ16237;MGC131748;TMEM195	alkylglycerol monooxygenase	1.31	1.04	0.71	0.72
ZNF227	ZNF227	zinc finger protein 227	1.27	0.88	0.71	0.66
TSKU	TSKU;E2IG4;LRRC54;TSK	tsukushi small leucine rich proteoglycan homolog (Xenopus laevis)	1.23	0.91	0.71	0.65
HK1	HK1;HK1-ta;HK1-tb;HK1-tc;HKI;HXK1	hexokinase 1	1.17	0.83	0.71	0.69
FZD6	FZD6;FZ-6;FZ6;HFZ6;NDNC10	frizzled family receptor 6	1.17	0.81	0.71	0.73
GALNT4	GALNT4;FLJ93314;GALNAC-T4;GALNACT4	UDP-N-acetyl-alpha-D-galactosamine:polypeptide N-acetylgalactosaminyltransferase 4 (GalNAc-T4)	1.12	0.77	0.71	0.67
PARP2	PARP2;ADPRT2;ADPRTL2;ADPRTL3;PARP-2;pADPRT-2	poly (ADP-ribose) polymerase 2	1.12	0.78	0.71	0.57
MC4R	MC4R;MGC126851;MGC138197	melanocortin 4 receptor	1.1	0.76	0.71	0.69
TRADD	TRADD;Hs.89862;MGC11078	TNFRSF1A-associated via death domain	1.1	0.8	0.71	0.63
PLA2G12A	PLA2G12A;GXII;PLA2G12;ROSSY	phospholipase A2, group XIIA	1.07	0.82	0.71	0.61
L3MBTL1	L3MBTL1;DKFZp586P1522;FLJ41181;H-L(3)MBT;KIAA0681;L3MBTL;dJ138B7.3	l(3)mbt-like 1 (Drosophila)	1.07	0.74	0.71	0.56
TYROBP	TYROBP;DAP12;KARAP;PLOSL	TYRO protein tyrosine kinase binding protein	1.06	0.76	0.71	0.51
SERPINH1	SERPINH1;AsTP3;CBP1;CBP2;HSP47;OI10;PPROM;RA-A47;SERPINH2;gp46	serpin peptidase inhibitor, clade H (heat shock protein 47), member 1, (collagen binding protein 1)	1.06	0.73	0.71	0.55
C18orf19	C18orf19;HsT2329;MGC24180	chromosome 18 open reading frame 19	1.04	0.73	0.71	0.55
C14orf43	C14orf43;C14orf117;FLJ00335;LSR68;c14_5541	chromosome 14 open reading frame 43	1.02	0.77	0.71	0.54

PLCB1	PLCB1;EIEE12;FLJ45792;PI-PLC;PLC-154;PLC-I;PLC154;PLCB1A;PLCB1B	phospholipase C, beta 1 (phosphoinositide-specific)	1.01	0.73	0.71	0.49
RNF24	RNF24;G1L	ring finger protein 24	0.99	0.7	0.71	0.62
RAD51AP1	RAD51AP1;PIR51	RAD51 associated protein 1	0.99	0.62	0.71	0.48
DCAF12	DCAF12;CT102;DKFZp434O125;KIAA1892;MGC1058;TCC52;WDR40A	DDB1 and CUL4 associated factor 12	0.94	0.64	0.71	0.49
CORO1A	CORO1A;CLABP;CLIPINA;FLJ41407;HCORO1;MGC117380;TACO;p57	coronin, actin binding protein, 1A	0.93	0.68	0.71	0.52
FHOD1	FHOD1;FHOS	formin homology 2 domain containing 1	0.93	0.68	0.71	0.52
RALY	RALY;HNRPCL2;MGC117312;P542	RNA binding protein, autoantigenic (hnRNP-associated with lethal yellow homolog (mouse))	0.93	0.64	0.71	0.49
CD22	CD22;FLJ22814;MGC130020;SIGLEC-2;SIGLEC2	CD22 molecule	0.87	0.6	0.71	0.46
CSF3	CSF3;C17orf33;CSF3OS;GCSF;MGC45931	colony stimulating factor 3 (granulocyte)	0.86	0.56	0.71	0.45
GJB5	GJB5;CX31.1	gap junction protein, beta 5, 31.1kDa	1.2	0.83	0.72	0.6
OR7D2	OR7D2;FLJ38149;HTPCRH03;OR19-10;OR19-4	olfactory receptor, family 7, subfamily D, member 2	1.15	0.8	0.72	0.61
BCORL1	BCORL1;BCoR-L1;CXorf10;FLJ11362;FLJ11632	BCL6 corepressor-like 1	1.15	0.88	0.72	0.65
CCT8L2	CCT8L2;CESK1;MGC118839;MGC118840	chaperonin containing TCP1, subunit 8 (theta)-like 2	1.14	0.86	0.72	0.67
DLK2	DLK2;EGFL9;MGC111055;MGC2487	delta-like 2 homolog (Drosophila)	1.1	0.83	0.72	0.67
CDX2	CDX2;CDX-3;CDX3	caudal type homeobox 2	1.05	0.72	0.72	0.52
FLAD1	FLAD1;FAD1;FADS;MGC31803;MGC40255;RP11-307C12.7	FAD1 flavin adenine dinucleotide synthetase homolog (S. cerevisiae)	1.04	0.7	0.72	0.55
SLCO3A1	SLCO3A1;FLJ40478;OATP-D;OATP3A1;OATPD;SLC21A11	solute carrier organic anion transporter family, member 3A1	1.02	0.75	0.72	0.59
BEND6	BEND6;C6orf65;FLJ30162;bA203B9.1	BEN domain containing 6	1.02	0.76	0.72	0.57
HOXA11	HOXA11;HOX1;HOX1I	homeobox A11	1.01	0.71	0.72	0.61
MYO18B	MYO18B;DKFZp434C2422;DKFZp779C1668;FLJ41942	myosin XVIIIB	1	0.7	0.72	0.51
C2orf88	C2orf88;MGC13057	chromosome 2 open reading frame 88	1	0.74	0.72	0.5
DKFZp761E19	DKFZp761E198;PP1030	uncharacterized protein DKFZp761E198	0.98	0.72	0.72	0.52
UBR1	UBR1;JBS;MGC142065;MGC142067	ubiquitin protein ligase E3 component n-recognin 1	0.98	0.68	0.72	0.62
THAP11	THAP11;CTG-B43a;CTG-B45d;RONIN	THAP domain containing 11	0.94	0.66	0.72	0.5
PRMT3	PRMT3;HRMT1L3	protein arginine methyltransferase 3	0.93	0.64	0.72	0.48
GTF3C2	GTF3C2;KIAA0011;TFIIIC-BETA;TFIIIC110	general transcription factor IIIC, polypeptide 2, beta 110kDa	0.9	0.64	0.72	0.48
MPP3	MPP3;DLG3	membrane protein, palmitoylated 3 (MAGUK p55 subfamily member 3)	0.88	0.62	0.72	0.56
ADO	ADO;C10orf22;DKFZp564C046;FLJ14547	2-aminoethanethiol (cysteamine) dioxygenase	0.85	0.59	0.72	0.45
HMX2	HMX2;H6L;Nkx5-2	H6 family homeobox 2	1.5	0.98	0.73	0.82
HOXA13	HOXA13;HOX1;HOX1J	homeobox A13	1.21	0.85	0.73	0.61
GDPD1	GDPD1;FLJ27503;FLJ37451;GDE4;MGC35046	glycerophosphodiester phosphodiesterase domain containing 1	1.17	0.84	0.73	0.65
DNAJC2	DNAJC2;MPHOSPH11;MPP11;ZRF1;ZUO1	DnaJ (Hsp40) homolog, subfamily C, member 2	1.08	0.76	0.73	0.58
TWIST2	TWIST2;DERMO1;MGC117334;bHLHa39	twist homolog 2 (Drosophila)	1.08	0.74	0.73	0.65
NLN	NLN;AGTBP;DKFZp564F123;EP24.16;FLJ23002;KIAA1226;MEP;MOP	neurolysin (metallopeptidase M3 family)	1.07	0.72	0.73	0.54
OR5D16	OR5D16;OR11-154	olfactory receptor, family 5, subfamily D, member 16	1.06	0.8	0.73	0.53
ADCY6	ADCY6;AC6;DKFZp779F075;KIAA0422	adenylate cyclase 6	1.05	0.78	0.73	0.61
SACS	SACS;ARSACS;DKFZp686B15167;DNAJC29	spastic ataxia of Charlevoix-Saguenay (sacsin)	1.02	0.72	0.73	0.52
NXNL2	NXNL2;C9orf121;RDCVF2	nucleoredoxin-like 2	0.96	0.69	0.73	0.52
SELE	SELE;CD62E;ELAM;ELAM1;ESEL;LECAM2	selectin E	0.93	0.66	0.73	0.52
MESP1	MESP1;MGC10676;bHLHc5	mesoderm posterior 1 homolog (mouse)	0.86	0.61	0.73	0.46
ARHGAP35	ARHGAP35;GRF-1;GRLF1;KIAA1722;MGC10745;P190-A;P190A;p190ARhoGAP;p190RhoGAP	Rho GTPase activating protein 35	1.35	0.97	0.74	0.76
ZNF691	ZNF691;Zfp691	zinc finger protein 691	1.33	0.98	0.74	0.7
ANO4	ANO4;FLJ34221;FLJ34272;FLJ35277;MGC130026;TMEM16D	anoctamin 4	1.25	0.9	0.74	0.68
ARIH1	ARIH1;ARI;DKFZp686O13120;FLJ20329;FLJ93118;HARI;HHARI;UBCH7BP	ariadne homolog, ubiquitin-conjugating enzyme E2 binding protein, 1 (Drosophila)	1.23	0.9	0.74	0.81
YAF2	YAF2;DKFZp779H1820;MGC41856	YY1 associated factor 2	1.22	0.84	0.74	0.74
KCNMB4	KCNMB4	potassium large conductance calcium-activated channel, subfamily M, beta member 4	1.21	0.94	0.74	0.73
EAF2	EAF2;BM040;TRAITS;U19	ELL associated factor 2	1.2	0.92	0.74	0.71
CCL27	CCL27;ALP;CTACK;CTAK;ESKINE;ILC;PESKY;SCYA27	chemokine (C-C motif) ligand 27	1.09	0.81	0.74	0.55
ATP9B	ATP9B;ATPASEP;ATPIIB;DKFZp686H2093;FLJ46612;MGC150650;MGC150651;MGC61572;NEO1L	ATPase, class II, type 9B	1.09	0.78	0.74	0.57
EPN2	EPN2;EHB21;KIAA1065	epsin 2	1.05	0.8	0.74	0.57
NKPD1	NKPD1;FLJ33600	NTPase, KAP family P-loop domain containing 1	0.96	0.68	0.74	0.54
MRGPRG	MRGPRG;GPR169;MRGG	MAS-related GPR, member G	0.95	0.69	0.74	0.62
METTL7B	METTL7B;MGC17301	methyltransferase like 7B	0.9	0.64	0.74	0.5
BCOR	BCOR;ANOP2;FLJ20285;FLJ38041;KIAA1575;MAA2;MCOPS2;MGC131961;MGC71031	BCL6 corepressor	0.9	0.64	0.74	0.52
EDDM3A	EDDM3A;EP3A;FAM12A;HE3-ALPHA;HE3A;HE3ALPHA;MGC119614;MGC119615	epididymal protein 3A	0.88	0.63	0.74	0.48
YTHDF1	YTHDF1;C20orf21;FLJ20391	YTH domain family, member 1	1.42	1.02	0.75	0.81
TFB1M	TFB1M;CGI75;mtTFB;mtTFB1	transcription factor B1, mitochondrial	1.4	1.08	0.75	0.85
CALCB	CALCB;CALC2;CGRP-II;CGRP2;FLJ30166	calcitonin-related polypeptide beta	1.29	0.99	0.75	0.78
TRIM23	TRIM23;ARD1;ARFD1;RNF46	tripartite motif containing 23	1.27	0.94	0.75	0.84
AGAP6	AGAP6;CTGLF3;bA324H6.1	ArfGAP with GTPase domain, ankyrin repeat and PH domain 6	1.15	0.86	0.75	0.6
CNKSR3	CNKSR3;FLJ31349;MAGI1	CNKSR family member 3	1.11	0.8	0.75	0.61
RHOXF1	RHOXF1;MGC119030;MGC119033;OTEX;PEPP1	Rhox homeobox family, member 1	1.08	0.78	0.75	0.62
SERPINA11	SERPINA11	serpin peptidase inhibitor, clade A (alpha-1 antiproteinase, antitrypsin), member 11	1.08	0.8	0.75	0.6
TNFRSF14	TNFRSF14;ATAR;CD270;HVEA;HVEM;LIGHTR;TR2	tumor necrosis factor receptor superfamily, member 14	1.07	0.8	0.75	0.54
POLRMT	POLRMT;APOLMT;MTRNAP;MTRPOL;h-mtRPOL	polymerase (RNA) mitochondrial (DNA directed)	1.06	0.76	0.75	0.56
GPR143	GPR143;NYS6;OA1	G protein-coupled receptor 143	1.05	0.73	0.75	0.55
NEUROD1	NEUROD1;BETA2;BHF-1;MODY6;NEUROD;bHLHa3	neurogenic differentiation 1	1.04	0.76	0.75	0.55
RENBP	RENBP;RBP;RNBP	renin binding protein	1.04	0.78	0.75	0.58
RAB39B	RAB39B;MRX72	RAB39B, member RAS oncogene family	1.01	0.74	0.75	0.6
RALA	RALA;MGC48949;RAL	v-ral simian leukemia viral oncogene homolog A (ras related)	1.01	0.72	0.75	0.53
B3GNT2	B3GNT2;B3GN-T2;B3GNT;B3GNT-2;B3GNT1;BETA3GNT;BGNT2;BGnT-2	UDP-GlcNAc:betaGal beta-1,3-N-acetylglucosaminyltransferase 2	1.01	0.72	0.75	0.57
FER	FER;PPP1R74;TYK3	fer (fps/fes related) tyrosine kinase	1	0.75	0.75	0.62
PET112	PET112;PET112L	PET112 homolog (yeast)	0.98	0.73	0.75	0.54
UBE2QL1	UBE2QL1;FLJ25076;FLJ39293	ubiquitin-conjugating enzyme E2Q family-like 1	0.97	0.66	0.75	0.5
C10orf10	C10orf10;DEPP;FIG	chromosome 10 open reading frame 10	0.96	0.71	0.75	0.54
AFAP1	AFAP1;AFAP;AFAP-110;FLJ56849	actin filament associated protein 1	0.96	0.7	0.75	0.52
NUB1	NUB1;BS4;NUB1L;NYREN18	negative regulator of ubiquitin-like proteins 1	0.94	0.68	0.75	0.52
P4HA3	P4HA3	prolyl 4-hydroxylase, alpha polypeptide III	0.91	0.66	0.75	0.47
KLK13	KLK13;DKFZp586J1923;KLK-L4;KLKL4	kallikrein-related peptidase 13	1.52	1.15	0.76	0.86
DNASE1L1	DNASE1L1;DNAS1L1;DNASEX;DNL1L;G4.8;XIB	deoxyribonuclease I-like 1	1.38	1.07	0.76	0.85
LRRC41	LRRC41;MGC126571;MGC126573;MUF1;RP4-636H5.2	leucine rich repeat containing 41	1.31	1	0.76	0.72
KCNAB3	KCNAB3;AKR6A9;KCNA3.1B;KCNA3B;KV-BETA-3;MGC116886	potassium voltage-gated channel, shaker-related subfamily, beta member 3	1.29	1.02	0.76	0.79
PTPRA	PTPRA;HEPTP;HLPR;HPTPA;HPTPalpha;LRP;PTPA;PTPRL2;R-PTP-alpha;RPTPA	protein tyrosine phosphatase, receptor type, A	1.21	0.86	0.76	0.64
YIPF6	YIPF6;FinGER6;MGC21416	Yip1 domain family, member 6	1.19	0.81	0.76	0.67
FAM122C	FAM122C	family with sequence similarity 122C	1.13	0.77	0.76	0.6
KCNK17	KCNK17;K2p17.1;TALK-2;TALK2;TASK-4;TASK4	potassium channel, subfamily K, member 17	1.11	0.84	0.76	0.65
USH1G	USH1G;ANKS4A;FLJ33924;SANS	Usher syndrome 1G (autosomal recessive)	1.1	0.8	0.76	0.61
C3orf75	C3orf75;FLJ20211;TMEM103	chromosome 3 open reading frame 75	1.1	0.82	0.76	0.66
FGF11	FGF11;FHF3;FLJ16061;MGC102953;MGC45269	fibroblast growth factor 11	1.06	0.8	0.76	0.62
TUBGCP6	TUBGCP6;GCP-6;GCP6	tubulin, gamma complex associated protein 6	1.04	0.78	0.76	0.58
CYP2S1	CYP2S1	cytochrome P450, family 2, subfamily S, polypeptide 1	1.04	0.77	0.76	0.58
FZD1	FZD1;DKFZp564G072;FLJ95923	frizzled family receptor 1	1.01	0.74	0.76	0.66
C6orf62	C6orf62;DKFZp564G182;FLJ12619;Nbla00237;XTP12;dJ30M3.2	chromosome 6 open reading frame 62	1	0.74	0.76	0.56
SPINK13	SPINK13;HBVDNAPTP1;HESPINTOR;LiESP6;MGC149260;SPINK5L3	serine peptidase inhibitor, Kazal type 13 (putative)	1	0.74	0.76	0.58
ERF	ERF;PE-2;PE2	Ets2 repressor factor	0.99	0.74	0.76	0.58
PER2	PER2;FASPS;KIAA0347	period homolog 2 (Drosophila)	0.97	0.72	0.76	0.54
ENO3	ENO3;GSD13;MSE	enolase 3 (beta, muscle)	0.96	0.68	0.76	0.54
CD2AP	CD2AP;CMS;DKFZp586H0519	CD2-associated protein	0.95	0.7	0.76	0.54
ZMYND17	ZMYND17;FLJ39565	zinc finger, MYND-type containing 17	0.9	0.62	0.76	0.49
C20orf118	C20orf118;dJ132F21.2	chromosome 20 open reading frame 118	1.26	0.95	0.77	0.72
CXCL3	CXCL3;CINC-2b;GRO3;GROg;MIP-2b;MIP2B;SCYB3	chemokine (C-X-C motif) ligand 3	1.21	0.94	0.77	0.64
KCNJ11	KCNJ11;BIR;HHF2;IKATP;KIR6.2;MGC133230;PHHI;TNDM3	potassium inwardly-rectifying channel, subfamily J, member 11	1.19	0.92	0.77	0.72
RPL17	RPL17;FLJ18762;FLJ92089;L17;MGC117162;PD-1;RPL23	ribosomal protein L17	1.17	0.92	0.77	0.7
GAPDH	GAPDH;G3PD;GAPD;MGC88685	glyceraldehyde-3-phosphate dehydrogenase	1.16	0.82	0.77	0.66
CMA1	CMA1;CYH;MCT1;MGC119890;MGC119891;chymase	chymase 1, mast cell	1.15	0.87	0.77	0.65
OR5B17	OR5B17;OR11-237;OR5B20P	olfactory receptor, family 5, subfamily B, member 17	1.15	0.9	0.77	0.69
ESM1	ESM1;endocan	endothelial cell-specific molecule 1	1.13	0.86	0.77	0.6
CLCN3	CLCN3;CLC3;ClC-3;DKFZp564I0463	chloride channel 3	1.12	0.87	0.77	0.68
LDHAL6A	LDHAL6A;LDH6A;MGC23940	lactate dehydrogenase A-like 6A	1.1	0.81	0.77	0.59
STAT4	STAT4;SLEB11	signal transducer and activator of transcription 4	1.1	0.79	0.77	0.63
BRD1	BRD1;BRL;BRPF1;BRPF2;DKFZp686F0325	bromodomain containing 1	1.08	0.82	0.77	0.62
PRSS22	PRSS22;BSSP-4;MGC9599;hBSSP-4	protease, serine, 22	1.06	0.74	0.77	0.56
DDT	DDT;DDCT;FLJ78842	D-dopachrome tautomerase	1.06	0.81	0.77	0.63
TRUB1	TRUB1;PUS4	TruB pseudouridine (psi) synthase homolog 1 (E. coli)	1.05	0.77	0.77	0.56
KRTAP6-2	KRTAP6-2;KAP6.2	keratin associated protein 6-2	1.02	0.76	0.77	0.58
GGT2	GGT2;GGT;GGT 2	gamma-glutamyltransferase 2	1.02	0.75	0.77	0.59
OR6N2	OR6N2;OR1-23	olfactory receptor, family 6, subfamily N, member 2	0.98	0.68	0.77	0.55
KCNG3	KCNG3;KV10.1;KV6.3	potassium voltage-gated channel, subfamily G, member 3	1.34	1.04	0.78	0.81
RASSF10	RASSF10	Ras association (RalGDS/AF-6) domain family (N-terminal) member 10	1.27	0.89	0.78	0.74
RAB20	RAB20;FLJ20429	RAB20, member RAS oncogene family	1.27	1.02	0.78	0.81
GCET2	GCET2;GCAT2;HGAL;MGC40441	germinal center expressed transcript 2	1.25	0.98	0.78	0.66
LYG1	LYG1;SALW1939	lysozyme G-like 1	1.25	0.99	0.78	0.76
USP35	USP35	ubiquitin specific peptidase 35	1.2	0.94	0.78	0.73
CALD1	CALD1;CDM;H-CAD;HCAD;L-CAD;LCAD;MGC21352;NAG22	caldesmon 1	1.2	0.92	0.78	0.76
FLJ45831	FLJ45831	uncharacterized FLJ45831	1.17	0.82	0.78	0.68
TYSND1	TYSND1;FLJ40378;MGC131934;MGC34695;NET41	trypsin domain containing 1	1.16	0.88	0.78	0.67
BCL2L13	BCL2L13;BCL-RAMBO;Bcl2-L-13;MIL1	BCL2-like 13 (apoptosis facilitator)	1.13	0.84	0.78	0.61
PCGF2	PCGF2;MEL-18;MGC10545;RNF110;ZNF144	polycomb group ring finger 2	1.12	0.86	0.78	0.77
LPGAT1	LPGAT1;FAM34A;FAM34A1;NET8	lysophosphatidylglycerol acyltransferase 1	1.07	0.8	0.78	0.57

PCYT2	PCYT2;ET	phosphate cytidylyltransferase 2, ethanolamine	1.04	0.76	0.78	0.6
TOP1	TOP1;TOPI	topoisomerase (DNA) I	1.02	0.76	0.78	0.57
PLSCR5	PLSCR5	phospholipid scramblase family, member 5	1.01	0.71	0.78	0.55
COL24A1	COL24A1;MGC142214	collagen, type XXIV, alpha 1	1	0.71	0.78	0.59
ZDHHC21	ZDHHC21;9130404H11Rik;DHHC-21;DNZ1;HSPC097	zinc finger, DHHC-type containing 21	0.94	0.66	0.78	0.53
ABCC10	ABCC10;EST182763;MRP7;SIMRP7	ATP-binding cassette, sub-family C (CFTR/MRP), member 10	0.91	0.66	0.78	0.59
WDYHV1	WDYHV1;C8orf32;FLJ10204	WDYHV motif containing 1	0.91	0.64	0.78	0.5
EDC4	EDC4;Ge-1;HEDLS;RCD-8	enhancer of mRNA decapping 4	0.88	0.62	0.78	0.48
KCNMA1	KCNMA1;BKTM;DKFZp686K1437;KCa1.1;MGC71881;MaxiK;SAKCA;SLO;SLO-ALPHA;SLO1;bA205K10.1;mSLO1	potassium large conductance calcium-activated channel, subfamily M, alpha member 1	1.52	1.19	0.79	0.93
DDIT4	DDIT4;Dig2;FLJ20500;REDD-1;REDD1;RP11-442H21.1;RTP801	DNA-damage-inducible transcript 4	1.37	0.97	0.79	0.81
TJP3	TJP3;MGC119546;ZO-3;ZO3	tight junction protein 3 (zona occludens 3)	1.23	0.88	0.79	0.73
RGAG4	RGAG4;6430402L03Rik;KIAA2001;MAR5;MART5	retrotransposon gag domain containing 4	1.2	0.94	0.79	0.68
PPP2R4	PPP2R4;MGC2184;PP2A;PR53;PTPA	protein phosphatase 2A activator, regulatory subunit 4	1.18	0.86	0.79	0.65
USP37	USP37;KIAA1594;MGC117261	ubiquitin specific peptidase 37	1.05	0.76	0.79	0.57
PSG5	PSG5;FL-NCA-3;PSG	pregnancy specific beta-1-glycoprotein 5	1.02	0.72	0.79	0.6
FUCA1	FUCA1;FUCA	fucosidase, alpha-L- 1, tissue	1.02	0.76	0.79	0.59
PID1	PID1;FLJ20701;NYGGF4;PCLI1	phosphotyrosine interaction domain containing 1	1.02	0.72	0.79	0.56
KIAA0355	KIAA0355	KIAA0355	1.01	0.74	0.79	0.63
MLH3	MLH3;HNPCC7;MGC138372	mutL homolog 3 (E. coli)	0.97	0.72	0.79	0.57
ISG20L2	ISG20L2;FLJ12671	interferon stimulated exonuclease gene 20kDa-like 2	0.97	0.71	0.79	0.57
HERC4	HERC4;DKFZp564G092;KIAA1593	hect domain and RLD 4	0.95	0.69	0.79	0.52
AFG3L2	AFG3L2;FLJ25993;SCA28	AFG3 ATPase family gene 3-like 2 (S. cerevisiae)	0.92	0.66	0.79	0.5
RIC8B	RIC8B;FLJ10620;MGC39476;RIC8;hSyn	resistance to inhibitors of cholinesterase 8 homolog B (C. elegans)	0.9	0.64	0.79	0.49
DBT	DBT;BCATE2;E2;E2B;MGC9061	dihydrolipoamide branched chain transacylase E2	1.22	0.93	0.8	0.67
GOLM1	GOLM1;C9orf155;FLJ22634;FLJ23608;GOLPH2;GP73;PSEC0257;bA379P1.3	golgi membrane protein 1	1.22	0.88	0.8	0.73
LRRC52	LRRC52;FLJ25811	leucine rich repeat containing 52	1.19	0.92	0.8	0.69
BPIFB4	BPIFB4;C20orf186;LPLUNC4;RY2G5;dJ726C3.5	BPI fold containing family B, member 4	1.12	0.8	0.8	0.67
M6PR	M6PR;CD-MPR;FLJ32994;MPR 46;MPR-46;MPR46;SMPR	mannose-6-phosphate receptor (cation dependent)	1.08	0.82	0.8	0.59
SSR4	SSR4;TRAPD	signal sequence receptor, delta	1.06	0.8	0.8	0.62
MEI1	MEI1;MGC40042	meiosis inhibitor 1	1.02	0.74	0.8	0.58
CSF2RA	CSF2RA;CD116;CDw116;CSF2R;CSF2RAX;CSF2RAY;CSF2RX;CSF2RY;GM-CSF-R-alpha;GMCSFR;GMR;MGC3848;MGC4838	colony stimulating factor 2 receptor, alpha, low-affinity (granulocyte-macrophage)	1	0.74	0.8	0.59
FYN	FYN;MGC45350;SLK;SYN;p59-FYN	FYN oncogene related to SRC, FGR, YES	0.9	0.66	0.67	0.46
RAB4A	RAB4A;HRES-1/RAB4;RAB4	RAB4A, member RAS oncogene family	0.88	0.67	0.68	0.46
PACS2	PACS2;FLJ25488;KIAA0602;PACS-2;PACS1L	phosphofurin acidic cluster sorting protein 2	0.85	0.62	0.68	0.42
MZT1	MZT1;C13orf37;FLJ21869;MGC150539;MOZART1	mitotic spindle organizing protein 1	0.85	0.61	0.68	0.49
ARL8A	ARL8A;ARL10B;FLJ45195;GIE2	ADP-ribosylation factor-like 8A	1.02	0.78	0.7	0.52
GFI1	GFI1;FLJ94509;GFI-1;SCN2;ZNF163	growth factor independent 1 transcription repressor	0.86	0.62	0.71	0.49
SFTA2	SFTA2;GSGL541;SFTPG;UNQ541	surfactant associated 2	1	0.78	0.72	0.52
RAB11FIP2	RAB11FIP2;KIAA0941;Rab11-FIP2;nRip11	RAB11 family interacting protein 2 (class I)	0.94	0.7	0.72	0.52
CT47A7	CT47A7;CT47.7;CT47A1;CT47A10;CT47A11;CT47A12;CT47A2;CT47A3;CT47A4;CT47A5;CT47A6;CT47A8;CT47A9	cancer/testis antigen family 47, member A7	0.89	0.68	0.72	0.48
OGFOD2	OGFOD2;DKFZp686H15154;FLJ13491;FLJ37501;MGC120434;MGC120436	2-oxoglutarate and iron-dependent oxygenase domain containing 2	0.86	0.66	0.72	0.46
SMAD4	SMAD4;DPC4;JIP;MADH4	SMAD family member 4	1.21	0.97	0.73	0.68
NDUFA11	NDUFA11;B14.7;CI-B14.7;FLJ76501	NADH dehydrogenase (ubiquinone) 1 alpha subcomplex, 11, 14.7kDa	1.04	0.8	0.73	0.53
KLHL7	KLHL7;KLHL6;SBBI26	kelch-like 7 (Drosophila)	0.98	0.76	0.73	0.53
ZSCAN18	ZSCAN18;DKFZp586B1122;FLJ12895;FLJ38919;FLJ44152;MGC2427;MGC4074;MGC8682;ZNF447	zinc finger and SCAN domain containing 18	0.97	0.76	0.73	0.53
GPAM	GPAM;GPAT;GPAT1;KIAA1560;MGC26846	glycerol-3-phosphate acyltransferase, mitochondrial	0.97	0.76	0.73	0.57
SPRR2G	SPRR2G	small proline-rich protein 2G	0.94	0.72	0.73	0.5
TFCP2	TFCP2;LBP1C;LSF;LSF1D;SEF;TFCP2C	transcription factor CP2	0.94	0.74	0.73	0.55
PKN2	PKN2;MGC150606;MGC71074;PAK2;PRK2;PRKCL2;PRO2042;Pak-2	protein kinase N2	0.92	0.68	0.73	0.56
FTSJ2	FTSJ2;DKFZp686J14194;FJH1	FtsJ homolog 2 (E. coli)	0.9	0.66	0.73	0.53
C14orf48	C14orf48;DKFZp434O1910;MGC33356;c14_5713	chromosome 14 open reading frame 48	0.87	0.66	0.73	0.44
CDC42EP4	CDC42EP4;BORG4;CEP4;KAIA1777;MGC17125;MGC3740	CDC42 effector protein (Rho GTPase binding) 4	0.86	0.62	0.73	0.48
MRPL3	MRPL3;MRL3;RPML3	mitochondrial ribosomal protein L3	0.86	0.66	0.73	0.53
OSBPL1A	OSBPL1A;FLJ10217;ORP-1;ORP1;OSBPL1B	oxysterol binding protein-like 1A	1.18	0.96	0.74	0.67
OC90	OC90;PLA2L	otoconin 90	0.97	0.77	0.74	0.58
HK3	HK3;HKIII;HXK3	hexokinase 3 (white cell)	1.19	0.96	0.75	0.69
ZFP3	ZFP3;FLJ30726;ZNF752	zinc finger protein 3 homolog (mouse)	1.12	0.89	0.75	0.63
KRT72	KRT72;K6IRS2;K6irs;KRT6;KRT6IRS2;MGC142246	keratin 72	1.11	0.9	0.75	0.6
GPX8	GPX8;UNQ847	glutathione peroxidase 8 (putative)	1.1	0.88	0.75	0.61
KRT16	KRT16;CK16;FNEPPK;K16;K1CP;KRT16A;NEPPK	keratin 16	1.01	0.78	0.75	0.56
NRXN3	NRXN3;C14orf60;KIAA0743;MGC176711	neurexin 3	0.98	0.76	0.75	0.57
NPAT	NPAT;E14;E14/NPAT;p220	nuclear protein, ataxia-telangiectasia locus	0.98	0.76	0.75	0.58
TNR	TNR;MGC149328;TN-R	tenascin R (restrictin, janusin)	0.95	0.72	0.75	0.56
KLHL11	KLHL11;FLJ10572	kelch-like 11 (Drosophila)	0.92	0.72	0.75	0.51
AAAS	AAAS;AAA;AAASb;ADRACALA;ADRACALIN;ALADIN;DKFZp586G1624	achalasia, adrenocortical insufficiency, alacrimia	0.92	0.68	0.75	0.53
FAM32A	FAM32A;OTAG12	family with sequence similarity 32, member A	0.9	0.7	0.75	0.5
DNAJC25	DNAJC25;bA16L21.2.1	DnaJ (Hsp40) homolog, subfamily C , member 25	0.85	0.61	0.75	0.44
TTYH1	TTYH1	tweety homolog 1 (Drosophila)	1.18	0.94	0.76	0.73
CIC	CIC;KIAA0306	capicua homolog (Drosophila)	1.07	0.84	0.76	0.56
ZNF519	ZNF519;FLJ36809;FLJ42861;HsT2362	zinc finger protein 519	1.03	0.82	0.76	0.61
DGKQ	DGKQ;DAGK;DAGK4;DAGK7	diacylglycerol kinase, theta 110kDa	0.98	0.74	0.76	0.61
PPA2	PPA2;FLJ20459;MGC49850;SID6-306	pyrophosphatase (inorganic) 2	0.89	0.66	0.76	0.52
SKIV2L	SKIV2L;170A;DDX13;HLP;SKI2;SKI2W;SKIV2	superkiller viralicidic activity 2-like (S. cerevisiae)	1.21	0.98	0.77	0.76
GLRA4	GLRA4	glycine receptor, alpha 4	1.19	0.98	0.77	0.76
TCTEX1D4	TCTEX1D4	Tctex1 domain containing 4	1.17	0.96	0.77	0.74
CHRND	CHRND;ACHRD;CMS2A;FCCMS;SCCMS	cholinergic receptor, nicotinic, delta	1.15	0.92	0.77	0.72
SORBS1	SORBS1;CAP;DKFZp451C066;DKFZp586P1422;FLAF2;FLJ12406;KIAA1296;R85FL;SH3D5;SH3P12;SORB1	sorbin and SH3 domain containing 1	1.13	0.9	0.77	0.61
MSMP	MSMP;PSMP	microseminoprotein, prostate associated	1.04	0.8	0.77	0.66
CNGB1	CNGB1;CNCG2;CNCG3L;CNCG4;CNG4;CNGB1B;GAR1;GARP;RCNC2;RCNCb;RCNCbeta;RP45	cyclic nucleotide gated channel beta 1	1.02	0.78	0.77	0.61
SLC35E2	SLC35E2;DKFZp686M0869;FLJ34996;FLJ44537;KIAA0447;MGC104754;MGC117254;MGC126715;MGC138494	solute carrier family 35, member E2	0.97	0.76	0.77	0.6
SLC43A1	SLC43A1;LAT3;PB39;POV1;R00504	solute carrier family 43, member 1	0.96	0.72	0.77	0.56
TRIM65	TRIM65;4732463G12Rik	tripartite motif containing 65	0.96	0.74	0.77	0.66
PARP1	PARP1;ADPRT;ADPRT 1;ADPRT1;PARP;PARP-1;PPOL;pADPRT-1	poly (ADP-ribose) polymerase 1	0.94	0.7	0.77	0.54
PPP1R17	PPP1R17;C7orf16;GSBS	protein phosphatase 1, regulatory subunit 17	0.94	0.74	0.77	0.56
KANK2	KANK2;ANKRD25;DKFZp434N161;FLJ20004;KIAA1518;MGC119707;MXRA3;SIP	KN motif and ankyrin repeat domains 2	0.93	0.7	0.77	0.53
PIK3R3	PIK3R3;DKFZp686P05226;FLJ41892;p55;p55-GAMMA	phosphoinositide-3-kinase, regulatory subunit 3 (gamma)	0.91	0.7	0.77	0.58
GCNT3	GCNT3;C2/4GnT;C24GNT;C2GNT2;C2GNTM;GNTM	glucosaminyl (N-acetyl) transferase 3, mucin type	0.9	0.66	0.77	0.48
ATP6V1A	ATP6V1A;ATP6A1;ATP6V1A1;HO68;VA68;VPP2;Vma1	ATPase, H+ transporting, lysosomal 70kDa, V1 subunit A	0.9	0.7	0.77	0.56
CNTNAP3	CNTNAP3;CASPR3;CNTNAP3A;RP11-138L21.1;RP11-290L7.1	contactin associated protein-like 3	0.88	0.68	0.77	0.46
TRIM28	TRIM28;FLJ29029;KAP1;RNF96;TF1B;TIF1B	tripartite motif containing 28	0.86	0.66	0.77	0.59
LPPR3	LPPR3;LPR3;PRG-2;PRG2	lipid phosphate phosphatase-related protein type 3	0.86	0.64	0.77	0.46
PIK3R4	PIK3R4;MGC102700;VPS15;p150	phosphoinositide-3-kinase, regulatory subunit 4	1.47	1.24	0.78	0.88
CLCN5	CLCN5;CLC5;CLCK2;DENTS;NPHL1;NPHL2;XLRH;XRN;hClC-K2;hClC-K2	chloride channel 5	1.23	1	0.78	0.77
TIMM22	TIMM22;TEX4;TIM22	translocase of inner mitochondrial membrane 22 homolog (yeast)	1.19	0.96	0.78	0.73
GATA5	GATA5;bB379O24.1	GATA binding protein 5	1	0.76	0.78	0.57
SNX7	SNX7;DKFZp564F052;MGC8717	sorting nexin 7	0.97	0.76	0.78	0.51
CCDC65	CCDC65;FLJ25663;FLJ35732;NYD-SP28	coiled-coil domain containing 65	0.96	0.74	0.78	0.54
CCL14	CCL14;CC-1;CC-3;CKB1;FLJ16015;HCC-1;HCC-1(1-74);HCC-1/HCC-3;HCC-3;MCIF;NCC-2;NCC2;SCYA14;SCYL2;SY14	chemokine (C-C motif) ligand 14	0.96	0.72	0.78	0.52
C8G	C8G;C8C;MGC142186	complement component 8, gamma polypeptide	0.96	0.72	0.78	0.52
FKBP14	FKBP14;FKBP22;FLJ20731	FK506 binding protein 14, 22 kDa	0.96	0.73	0.78	0.57
SRRM1	SRRM1;160-KD;MGC39488;POP101;SRM160	serine/arginine repetitive matrix 1	0.96	0.72	0.78	0.52
PLG	PLG;DKFZp779M0222	plasminogen	0.95	0.74	0.78	0.55
FKBP1B	FKBP1B;FKBP12.6;FKBP1L;OTK4;PKBP1L;PPIase	FK506 binding protein 1B, 12.6 kDa	0.92	0.72	0.78	0.53
PRADC1	PRADC1;C2orf7;MGC13004;PAP21	protease-associated domain containing 1	0.92	0.7	0.78	0.53
CHDH	CHDH	choline dehydrogenase	0.88	0.64	0.78	0.51
FCGRT	FCGRT;FCRN;alpha-chain	Fc fragment of IgG, receptor, transporter, alpha	0.88	0.67	0.78	0.47
PDIA5	PDIA5;FLJ30401;PDIR	protein disulfide isomerase family A, member 5	0.88	0.66	0.78	0.52
USP18	USP18;ISG43;UBP43	ubiquitin specific peptidase 18	0.86	0.62	0.78	0.47
KCNA3	KCNA3;HGK5;HLK3;HPCN3;HUKIII;KV1.3;MK3;PCN3	potassium voltage-gated channel, shaker-related subfamily, member 3	1.43	1.19	0.79	0.92
DGKK	DGKK	diacylglycerol kinase, kappa	1.09	0.86	0.79	0.71
DPYSL3	DPYSL3;CRMP-4;CRMP4;DRP-3;DRP3;LCRMP;ULIP;ULIP-1	dihydropyrimidinase-like 3	1.08	0.86	0.79	0.67
MUC17	MUC17;MUC3	mucin 17, cell surface associated	1.06	0.84	0.79	0.59
CCBP2	CCBP2;CCR10;CCR9;CMKBR9;D6;MGC126678;MGC138250;hD6	chemokine binding protein 2	1.06	0.82	0.79	0.73
DDX52	DDX52;HUSSY19;ROK1	DEAD (Asp-Glu-Ala-Asp) box polypeptide 52	1.04	0.84	0.79	0.66
ITSN1	ITSN1;ITSN;MGC134948;MGC134949;SH3D1A;SH3P17	intersectin 1 (SH3 domain protein)	1.04	0.8	0.79	0.63
BANF2	BANF2;BAF-L;BAF2;BAFL;C20orf179;MGC62093;MGC72082	barrier to autointegration factor 2	1.01	0.8	0.79	0.58
ISG20	ISG20;CD25;HEM45	interferon stimulated exonuclease gene 20kDa	1	0.76	0.79	0.6
SPCS3	SPCS3;DKFZp564J1864;FLJ22649;PRO3567;SPC22/23;SPC3;YLR066W	signal peptidase complex subunit 3 homolog (S. cerevisiae)	1	0.78	0.79	0.58
C15orf37	C15orf37;FLJ33788	chromosome 15 open reading frame 37	0.96	0.73	0.79	0.56
ZNF711	ZNF711;CMPX1;MRX97;ZNF4;ZNF5;ZNF6;Zfp711;dJ75N13.1	zinc finger protein 711	0.92	0.7	0.79	0.53
HS3ST4	HS3ST4;3-OST-4;30ST4;3OST4;h3-OST-4	heparan sulfate (glucosamine) 3-O-sulfotransferase 4	0.92	0.72	0.79	0.56
CMTM8	CMTM8;CKLFSF8;CKLFSF8-V2	CKLF-like MARVEL transmembrane domain containing 8	0.9	0.69	0.79	0.52
MOBP	MOBP;MGC87379	myelin-associated oligodendrocyte basic protein	0.89	0.68	0.79	0.55
ZNF625	ZNF625	zinc finger protein 625	0.87	0.66	0.79	0.48
CACNB2	CACNB2;CACNLB2;CAVB2;FLJ23743;MYSB	calcium channel, voltage-dependent, beta 2 subunit	1.27	1.06	0.8	0.82
P2RX4	P2RX4;P2X4;P2X4R	purinergic receptor P2X, ligand-gated ion channel, 4	1.27	1.05	0.8	0.82
WNK3	WNK3;FLJ30437;FLJ42662;KIAA1566;PRKWNK3	WNK lysine deficient protein kinase 3	1.21	0.97	0.8	0.8
DDX6	DDX6;FLJ36338;HLR2;P54;RCK	DEAD (Asp-Glu-Ala-Asp) box polypeptide 6	1.19	0.97	0.8	0.77
RAB6A	RAB6A;RAB6	RAB6A, member RAS oncogene family	1.14	0.9	0.8	0.66

F2R	F2R;CF2R;HTR;PAR-1;PAR1;TR	coagulation factor II (thrombin) receptor	1.1	0.86	0.8	0.77
OR2H2	OR2H2;FAT11;MGC126732;MGC138381;OLFR2;OLFR42B;OR2H3;dJ271M21.2;hs6M1-12	olfactory receptor, family 2, subfamily H, member 2	1.06	0.84	0.8	0.61
PIAS3	PIAS3;FLJ14651;ZMIZ5	protein inhibitor of activated STAT, 3	1.04	0.8	0.8	0.63
SVOP	SVOP;DKFZp761H039	SV2 related protein homolog (rat)	1.02	0.8	0.8	0.56
MORF4L2	MORF4L2;KIAA0026;MORFL2;MRGX	mortality factor 4 like 2	1.01	0.78	0.8	0.63
MCF2L	MCF2L;ARHGEF14;DBS;FLJ12122;KIAA0362;OST	MCF.2 cell line derived transforming sequence-like	1	0.77	0.8	0.61
DRAM1	DRAM1;DRAM;FLJ11259	DNA-damage regulated autophagy modulator 1	1	0.76	0.8	0.59
C15orf2	C15orf2	chromosome 15 open reading frame 2	0.98	0.76	0.8	0.58
TLR5	TLR5;FLJ10052;MGC126430;MGC126431;SLEB1;TIL3	toll-like receptor 5	0.97	0.73	0.8	0.57
DHH	DHH;GDXYM;HHG-3;MGC35145;SRXY7	desert hedgehog	0.96	0.76	0.8	0.57
C9orf169	C9orf169;MGC59937	chromosome 9 open reading frame 169	0.96	0.75	0.8	0.6
PIGU	PIGU;CDC91L1;GAB1;MGC40420	phosphatidylinositol glycan anchor biosynthesis, class U	0.96	0.75	0.8	0.65
SCPEP1	SCPEP1;HSCP1;RISC	serine carboxypeptidase 1	0.92	0.68	0.8	0.51
TMEM211	TMEM211;MGC163519;MGC163521;bA9F11.1	transmembrane protein 211	0.9	0.7	0.8	0.55
ALPL	ALPL;AP-TNAP;APTNAP;FLJ40094;FLJ93059;HOPS;MGC161443;MGC167935;TNAP;TNSALP	alkaline phosphatase, liver/bone/kidney	0.9	0.66	0.8	0.5
FAM160B2	FAM160B2;FLJ11125;FLJ21801;MGC138352;RAI16	family with sequence similarity 160, member B2	0.9	0.7	0.8	0.56
PEX16	PEX16	peroxisomal biogenesis factor 16	0.9	0.68	0.8	0.57
HEXB	HEXB;ENC-1AS	hexosaminidase B (beta polypeptide)	0.9	0.7	0.8	0.52
DDX23	DDX23;MGC8416;PRPF28;U5-100K;U5-100KD;prp28	DEAD (Asp-Glu-Ala-Asp) box polypeptide 23	0.88	0.65	0.8	0.58
TRAM2	TRAM2;KIAA0057	translocation associated membrane protein 2	0.68	0.1	0.14	0.07
UROC1	UROC1;FLJ31300;HMFN0320;MGC135007;MGC135008	urocanase domain containing 1	0.82	0.48	0.52	0.33
C9orf123	C9orf123;MGC4730	chromosome 9 open reading frame 123	0.66	0.36	0.53	0.28
ART5	ART5;MGC22848	ADP-ribosyltransferase 5	0.68	0.42	0.55	0.29
ZNF350	ZNF350;ZBRK1;ZFQR	zinc finger protein 350	0.68	0.36	0.55	0.28
BRCA2	BRCA2;BRCC2;BROVCA2;FACD;FAD;FAD1;FANCB;FANCD;FANCD1;GLM3;PNCA2	breast cancer 2, early onset	0.84	0.53	0.57	0.37
ZNF512	ZNF512;FLJ51176;KIAA1805;MGC111046	zinc finger protein 512	0.82	0.5	0.57	0.37
ZNF79	ZNF79;pT7	zinc finger protein 79	0.66	0.4	0.58	0.27
TRIM11	TRIM11;BIA1;RNF92	tripartite motif containing 11	0.75	0.44	0.59	0.39
NPFFR1	NPFFR1;FLJ10751;GPR147;NPFF1;NPFF1R1;OT7T022	neuropeptide FF receptor 1	0.82	0.48	0.6	0.43
IQCA1	IQCA1;4930465P12Rik;FLJ22527;FLJ33588;IQCA	IQ motif containing with AAA domain 1	0.73	0.46	0.6	0.32
DMRT1	DMRT1;DMT1	doublesex and mab-3 related transcription factor 1	0.84	0.54	0.61	0.42
FBXO31	FBXO31;DKFZp434B027;DKFZp434J1815;FBX14;FBXO14;FLJ22477;Fbx31;MGC15419;MGC9527;pp2386	F-box protein 31	0.76	0.44	0.61	0.32
DGCR2	DGCR2;DGS-C;DKFZp686I1730;IDD;KIAA0163;LAN;SEZ-12	DiGeorge syndrome critical region gene 2	0.79	0.5	0.62	0.36
GABRD	GABRD;EIG10;EJM7;GEFSP5;MGC45284	gamma-aminobutyric acid (GABA) A receptor, delta	0.76	0.48	0.62	0.37
C3orf54	C3orf54;INKA1;MGC20416	chromosome 3 open reading frame 54	0.74	0.45	0.62	0.33
PARP10	PARP10;FLJ14464	poly (ADP-ribose) polymerase family, member 10	0.84	0.47	0.63	0.37
SUV39H2	SUV39H2;FLJ23414;KMT1B	suppressor of variegation 3-9 homolog 2 (Drosophila)	0.8	0.46	0.63	0.37
ANKRD39	ANKRD39;MGC41816	ankyrin repeat domain 39	0.78	0.51	0.63	0.37
SLC16A11	SLC16A11;FLJ90193;MCT11	solute carrier family 16, member 11 (monocarboxylic acid transporter 11)	0.84	0.56	0.64	0.43
C16orf85	C16orf85;FLJ45530	chromosome 16 open reading frame 85	0.78	0.52	0.64	0.35
HAND2	HAND2;DHAND2;FLJ16260;Hed;MGC125303;MGC125304;Thing2;bHLHa26;dHand	heart and neural crest derivatives expressed 2	0.71	0.44	0.64	0.31
GFPT1	GFPT1;GFA;GFAT;GFAT 1;GFAT1;GFAT1m;GFPT	glutamine--fructose-6-phosphate transaminase 1	0.8	0.5	0.65	0.39
ACTN4	ACTN4;ACTININ-4;DKFZp686K23158;FSGS;FSGS1	actinin, alpha 4	0.79	0.54	0.65	0.43
HMCN1	HMCN1;ARMD1;FBLN6;FIBL-6;FIBL6	hemicentin 1	0.77	0.52	0.65	0.41
HIPK1	HIPK1;KIAA0630;MGC26642;MGC33446;MGC33548;Myak;Nbak2	homeodomain interacting protein kinase 1	0.83	0.54	0.66	0.45
CDK20	CDK20;CCRK;CDCH;P42;PNQALRE	cyclin-dependent kinase 20	0.82	0.54	0.66	0.45
S1PR5	S1PR5;EDG8;Edg-8;S1P5;SPPR-1;SPPR-2	sphingosine-1-phosphate receptor 5	0.84	0.54	0.67	0.49
ZNF425	ZNF425;MGC150568	zinc finger protein 425	0.84	0.57	0.67	0.42
AAK1	AAK1;DKFZp686F03202;DKFZp686K16132;FLJ23712;FLJ25931;FLJ31060;FLJ42882;FLJ45252;KIAA1048;MGC138170;MGC164568;MGC164570	AP2 associated kinase 1	0.8	0.53	0.67	0.44
KLHDC9	KLHDC9;KARCA1;MGC33338	kelch domain containing 9	0.84	0.56	0.68	0.42
RASL11B	RASL11B;MGC2827;MGC4499	RAS-like, family 11, member B	0.81	0.56	0.68	0.37
FGFR1OP2	FGFR1OP2;DKFZp564O1863;DKFZp586C1423;FLJ37569;HSPC123-like;WIT3.0	FGFR1 oncogene partner 2	0.8	0.54	0.69	0.41
SHROOM2	SHROOM2;APXL;DKFZp781J074;FLJ39277;HSAPXL	shroom family member 2	0.84	0.58	0.7	0.45
IFI6	IFI6;6-16;FAM14C;G1P3;IFI-6-16;IFI616	interferon, alpha-inducible protein 6	0.79	0.54	0.7	0.4
TRH	TRH;MGC125964;MGC125965	thyrotropin-releasing hormone	0.69	0.44	0.7	0.36
HIST3H3	HIST3H3;H3.4;H3/g;H3FT;H3t;MGC126886;MGC126888	histone cluster 3, H3	0.82	0.56	0.71	0.46
PPID	PPID;CYP-40;CYPD;MGC33096	peptidylprolyl isomerase D	0.82	0.53	0.71	0.47
TCEAL1	TCEAL1;SIIR;p21;pp21	transcription elongation factor A (SII)-like 1	0.84	0.58	0.72	0.43
RDH8	RDH8;PRRDH;SDR28C2	retinol dehydrogenase 8 (all-trans)	0.84	0.59	0.74	0.46
NOP10	NOP10;MGC70651;NOLA3;NOP10P	NOP10 ribonucleoprotein homolog (yeast)	0.83	0.58	0.74	0.49
LIG3	LIG3;LIG2	ligase III, DNA, ATP-dependent	0.64	0.4	0.63	0.3
NR0B2	NR0B2;FLJ17090;SHP;SHP1	nuclear receptor subfamily 0, group B, member 2	0.77	0.55	0.66	0.41
MRC2	MRC2;CD280;CLEC13E;ENDO180;FLJ35911;KIAA0709;UPARAP	mannose receptor, C type 2	0.72	0.48	0.66	0.32
FEM1B	FEM1B;DKFZp451E0710;F1A-ALPHA;FIAA	fem-1 homolog b (C. elegans)	0.78	0.56	0.67	0.42
SNRNP27	SNRNP27;27K;RY1	small nuclear ribonucleoprotein 27kDa (U4/U6.U5)	0.75	0.51	0.67	0.4
TMEM42	TMEM42;MGC29956	transmembrane protein 42	0.69	0.46	0.67	0.32
CPNE2	CPNE2;COPN2;CPN2;DKFZp686E06199;MGC16924	copine II	0.84	0.6	0.68	0.4
PODN	PODN;MGC24995;PCAN;SLRR5A	podocan	0.79	0.55	0.68	0.4
ZNF648	ZNF648	zinc finger protein 648	0.78	0.55	0.68	0.39
ING1	ING1;p24ING1c;p33;p33ING1;p33ING1b;p47;p47ING1a	inhibitor of growth family, member 1	0.75	0.54	0.68	0.41
CD81	CD81;CVID6;S5.7;TAPA1;TSPAN28	CD81 molecule	0.83	0.6	0.69	0.42
ALX3	ALX3;FND1;MGC138212;MGC141988	ALX homeobox 3	0.82	0.61	0.69	0.45
MED9	MED9;FLJ10193;MED25;MGC138234	mediator complex subunit 9	0.68	0.46	0.69	0.35
NCALD	NCALD;MGC33870;MGC74858	neurocalcin delta	0.84	0.62	0.7	0.41
SRSF11	SRSF11;DKFZp686M13204;FLJ18226;NET2;SFRS11;dJ677H15.2;p54	serine/arginine-rich splicing factor 11	0.81	0.6	0.7	0.42
UNC13A	UNC13A;KIAA1032;Munc13-1	unc-13 homolog A (C. elegans)	0.77	0.53	0.7	0.44
RFC5	RFC5;MGC1155;RFC36	replication factor C (activator 1) 5, 36.5kDa	0.76	0.56	0.7	0.43
SLC22A11	SLC22A11;MGC34282;OAT4;hOAT4	solute carrier family 22 (organic anion/urate transporter), member 11	0.74	0.52	0.7	0.41
ZNF394	ZNF394;FLJ12298;ZKSCAN14	zinc finger protein 394	0.7	0.48	0.7	0.36
DLG5	DLG5;KIAA0583;LP-DLG;P-DLG5;PDLG	discs, large homolog 5 (Drosophila)	0.66	0.43	0.7	0.34
PCSK9	PCSK9;FH3;HCHOLA3;LDLCQ1;NARC-1;NARC1;PC9	proprotein convertase subtilisin/kexin type 9	0.64	0.44	0.7	0.33
MOCS1	MOCS1;KIAA0381;MIG11;MOCOD	molybdenum cofactor synthesis 1	0.63	0.4	0.7	0.32
HRK	HRK;DP5;HARAKIRI	harakiri, BCL2 interacting protein (contains only BH3 domain)	0.84	0.62	0.71	0.44
ST6GALNAC2	ST6GALNAC2;FLJ45660;SAITL1;SIAT7;SIAT7B;SIATL1;ST6GalNAII;STHM	ST6 (alpha-N-acetyl-neuraminyl-2,3-beta-galactosyl-1,3)-N-acetylgalactosaminide alpha-2,6-sialyltransferase 2	0.84	0.6	0.71	0.4
UBTF	UBTF;NOR-90;UBF;UBF-1;UBF1	upstream binding transcription factor, RNA polymerase I	0.78	0.55	0.71	0.41
TRIM6	TRIM6;RNF89	tripartite motif containing 6	0.76	0.53	0.71	0.48
LY9	LY9;CD229;SLAMF3;hly9;mLY9	lymphocyte antigen 9	0.74	0.52	0.71	0.37
COMMD4	COMMD4;FLJ20452	COMM domain containing 4	0.7	0.48	0.71	0.4
KIAA1751	KIAA1751;FLJ45476	KIAA1751	0.67	0.45	0.71	0.37
GNRH2	GNRH2;GnRH-II;LH-RHII	gonadotropin-releasing hormone 2	0.82	0.62	0.72	0.43
RNF144B	RNF144B;IBRDC2;KIAA0161;MGC71786;PIR2;bA528A10.3;p53RFP	ring finger protein 144B	0.78	0.55	0.72	0.5
RAB33B	RAB33B;DKFZp434G099;MGC138182	RAB33B, member RAS oncogene family	0.72	0.52	0.72	0.39
GABRR1	GABRR1;MGC163216	gamma-aminobutyric acid (GABA) receptor, rho 1	0.8	0.58	0.73	0.45
OGDH	OGDH;AKGDH;E1k;OGDC	oxoglutarate (alpha-ketoglutarate) dehydrogenase (lipoamide)	0.78	0.55	0.73	0.48
TCEAL6	TCEAL6;Tceal3	transcription elongation factor A (SII)-like 6	0.77	0.53	0.73	0.45
SEC61B	SEC61B	Sec61 beta subunit	0.76	0.56	0.73	0.43
CCND1	CCND1;BCL1;D11S287E;PRAD1;U21B31	cyclin D1	0.75	0.53	0.73	0.46
KCNK6	KCNK6;FLJ12282;K2p6.1;KCNK8;TOSS;TWIK-2;TWIK2	potassium channel, subfamily K, member 6	0.74	0.54	0.73	0.42
KLHL21	KLHL21;KIAA0469;MGC99635	kelch-like 21 (Drosophila)	0.8	0.58	0.74	0.44
C1orf127	C1orf127;FLJ37118;FLJ37833	chromosome 1 open reading frame 127	0.74	0.54	0.74	0.39
FRAT1	FRAT1;FLJ97193	frequently rearranged in advanced T-cell lymphomas	0.7	0.48	0.74	0.38
ZNF630	ZNF630;FLJ20573;FLJ97927;FLJ98691;MGC138344;dJ54B20.2	zinc finger protein 630	0.64	0.42	0.74	0.35
CT47A6	CT47A6;CT47.6;CT47A1;CT47A10;CT47A11;CT47A12;CT47A2;CT47A3;CT47A4;CT47A5;CT47A7;CT47A8;CT47A9	cancer/testis antigen family 47, member A6	0.84	0.62	0.75	0.46
DPEP1	DPEP1;MBD1;MDP;RDP	dipeptidase 1 (renal)	0.82	0.6	0.75	0.45
GOLPH3	GOLPH3;FLJ90675;GOPP1;GPP34;MIDAS	golgi phosphoprotein 3 (coat-protein)	0.78	0.56	0.75	0.43
LOC81691	LOC81691;DKFZp434J0315	exonuclease NEF-sp	0.76	0.56	0.75	0.48
H1F0	H1F0;H10;H1FV;MGC5241	H1 histone family, member 0	0.7	0.5	0.75	0.39
MEFV	MEFV;FMF;MEF;MGC126560;MGC126586;TRIM20	Mediterranean fever	0.84	0.62	0.76	0.56
PIN1	PIN1;DOD;FLJ40239;FLJ77628;MGC10717;UBL5	peptidylprolyl cis/trans isomerase, NIMA-interacting 1	0.82	0.6	0.76	0.43
ECD	ECD;GCR2;HSGT1	ecdysoneless homolog (Drosophila)	0.77	0.54	0.76	0.48
MTX2	MTX2;MGC111067	metaxin 2	0.74	0.54	0.76	0.41
MYLK	MYLK;AAT7;DKFZp686I10125;FLJ12216;KRP;MLCK;MLCK1;MLCK108;MLCK210;MSTP083;MYLK1;smMLCK	myosin light chain kinase	0.8	0.6	0.77	0.53
RHOBTB3	RHOBTB3;KIAA0878	Rho-related BTB domain containing 3	0.84	0.62	0.78	0.49
GARS	GARS;CMT2D;DSMAV;GlyRS;HMN5;SMAD1	glycyl-tRNA synthetase	0.84	0.64	0.78	0.5
DPH2	DPH2;DPH2L2	DPH2 homolog (S. cerevisiae)	0.77	0.54	0.78	0.42
SELO	SELO;MGC131879	selenoprotein O	0.83	0.62	0.79	0.5
CCT2	CCT2;99D8.1;CCT-beta;CCTB;MGC142074;MGC142076;PRO1633;TCP-1-beta	chaperonin containing TCP1, subunit 2 (beta)	0.82	0.62	0.79	0.44
PPM1A	PPM1A;FLJ42306;MGC9201;PP2C-ALPHA;PP2CA;PP2Calpha	protein phosphatase, Mg2+/Mn2+ dependent, 1A	0.78	0.57	0.79	0.43
NTAN1	NTAN1;DKFZp666E058	N-terminal asparagine amidase	0.7	0.5	0.79	0.42
ZNF513	ZNF513;FLJ32203;HMFT0656;RP58	zinc finger protein 513	0.83	0.63	0.8	0.53
ERP27	ERP27;C12orf46;FLJ32115;PDIA8	endoplasmic reticulum protein 27	0.82	0.59	0.8	0.46
TDP2	TDP2;AD022;EAP2;MGC111021;MGC9099;TTRAP;dJ30M3.3	tyrosyl-DNA phosphodiesterase 2	0.93	0.74	0.72	0.51
PKIB	PKIB;FLJ23817;PRKACN2	protein kinase (cAMP-dependent, catalytic) inhibitor beta	0.93	0.74	0.73	0.52
CCDC19	CCDC19;NESG1	coiled-coil domain containing 19	1.03	0.84	0.74	0.56
KLHL30	KLHL30;FLJ43374	kelch-like 30 (Drosophila)	0.92	0.74	0.74	0.57
OAS3	OAS3;MGC133260;p100	2'-5'-oligoadenylate synthetase 3, 100kDa	0.9	0.72	0.74	0.54
RET	RET;CDHF12;CDHR16;HSCR1;MEN2A;MEN2B;MTC1;PTC;RET-ELE1;RET51	ret proto-oncogene	0.87	0.68	0.74	0.49
ABCC11	ABCC11;EWWD;MRP8;WW	ATP-binding cassette, sub-family C (CFTR/MRP), member 11	0.85	0.67	0.74	0.5
RBM7	RBM7;FLJ11153	RNA binding motif protein 7	1.02	0.84	0.75	0.62

KCTD3	KCTD3;MGC43935;NY-REN-45	potassium channel tetramerisation domain containing 3	0.92	0.74	0.75	0.57
C3orf62	C3orf62;FLJ43654;MGC23381;MGC61663;MGC62079	chromosome 3 open reading frame 62	0.91	0.74	0.75	0.5
ZNF687	ZNF687;DKFZp781I1719;KIAA1441	zinc finger protein 687	0.86	0.68	0.75	0.48
TGM2	TGM2;G-ALPHA-h;GNAH;TG2;TGC	transglutaminase 2 (C polypeptide, protein-glutamine-gamma-glutamyltransferase)	1.07	0.89	0.76	0.66
EMILIN3	EMILIN3;C20orf130;DKFZp434A2410;EMILIN5;dJ620E11.4	elastin microfibril interfacer 3	1	0.84	0.76	0.62
NGRN	NGRN;DSC92	neugrin, neurite outgrowth associated	1	0.82	0.76	0.55
FAM123C	FAM123C;FLJ38377	family with sequence similarity 123C	0.98	0.82	0.76	0.6
FAM113B	FAM113B;MGC16044	family with sequence similarity 113, member B	0.96	0.78	0.76	0.54
SAC3D1	SAC3D1;HSU79266;SHD1	SAC3 domain containing 1	0.95	0.77	0.76	0.54
TNFRSF6B	TNFRSF6B;DCR3;DJ583P15.1.1;M68;M68E;TR6	tumor necrosis factor receptor superfamily, member 6b, decoy	0.92	0.73	0.76	0.51
CARD16	CARD16;COP;COP1;PSEUDO-ICE	caspase recruitment domain family, member 16	0.92	0.73	0.76	0.52
TGFBR3	TGFBR3;BGCAN;betaglycan	transforming growth factor, beta receptor III	0.87	0.71	0.76	0.51
SLC4A8	SLC4A8;DKFZp761B2318;FLJ46462;NBC3;NDCBE	solute carrier family 4, sodium bicarbonate cotransporter, member 8	0.86	0.7	0.76	0.52
LRP4	LRP4;CLSS;KIAA0816;LRP-4;LRP10;MEGF7;SOST2	low density lipoprotein receptor-related protein 4	0.86	0.67	0.76	0.46
CYFIP1	CYFIP1;FLJ45151;P140SRA-1;SHYC;SRA1	cytoplasmic FMR1 interacting protein 1	1.08	0.91	0.77	0.67
CD2	CD2;FLJ46032;SRBC;T11	CD2 molecule	1.05	0.9	0.77	0.63
APOPT1	APOPT1;APOP1;C14orf153;MGC2562	apoptogenic 1	1.02	0.83	0.77	0.58
CERS6	CERS6;CerS5;LASS6;MGC129949;MGC129950	ceramide synthase 6	0.94	0.79	0.77	0.58
C13orf16	C13orf16;MGC35169;bA474D23.1	chromosome 13 open reading frame 16	0.91	0.76	0.77	0.51
ARPC5L	ARPC5L;ARC16-2;MGC3038	actin related protein 2/3 complex, subunit 5-like	0.9	0.72	0.77	0.49
KIF3A	KIF3A	kinesin family member 3A	0.88	0.73	0.77	0.55
KPNA5	KPNA5;IPOA6;SRP6	karyopherin alpha 5 (importin alpha 6)	0.86	0.7	0.77	0.52
GRIPAP1	GRIPAP1;DKFZp434P0630;GRASP-1;KIAA1167;MGC126593;MGC126595	GRIP1 associated protein 1	0.85	0.68	0.77	0.45
AMZ2	AMZ2	archaelysin family metallopeptidase 2	1.13	0.94	0.78	0.66
C15orf26	C15orf26;FLJ38615	chromosome 15 open reading frame 26	1.12	0.95	0.78	0.63
RAB11FIP4	RAB11FIP4;FLJ00131;KIAA1821;MGC11316;MGC126566;RAB11-FIP4	RAB11 family interacting protein 4 (class II)	1.08	0.92	0.78	0.68
OTOGL	OTOGL;C12orf64;FLJ90579;MGC119410	otogelin-like	1.02	0.85	0.78	0.59
ITPR2	ITPR2;IP3R2	inositol 1,4,5-trisphosphate receptor, type 2	1	0.82	0.78	0.64
KLHL9	KLHL9;FLJ21815	kelch-like 9 (Drosophila)	0.99	0.8	0.78	0.55
RAB3IP	RAB3IP;FLJ14660;FLJ22548;MGC71495;RABIN3	RAB3A interacting protein (rabin3)	0.98	0.81	0.78	0.57
VRK3	VRK3	vaccinia related kinase 3	0.97	0.82	0.78	0.58
GAGE5	GAGE5;CT4.5	G antigen 5	0.95	0.77	0.78	0.52
CHRNA3	CHRNA3;LNCR2;MGC104879;NACHRA3;PAOD2	cholinergic receptor, nicotinic, alpha 3	0.91	0.75	0.78	0.58
SCD5	SCD5;ACOD4;FADS4;FLJ21032;HSCD5;SCD2;SCD4	stearoyl-CoA desaturase 5	0.9	0.79	0.78	0.54
HIST1H2BM	HIST1H2BM;H2B/e;H2BFE;dJ160A22.3	histone cluster 1, H2bm	0.9	0.74	0.78	0.59
PDE12	PDE12;2'-PDE	phosphodiesterase 12	0.89	0.7	0.78	0.5
NKX2-8	NKX2-8;NKX2.8;NKX2H;Nkx2-9	NK2 homeobox 8	0.88	0.73	0.78	0.48
KCNJ6	KCNJ6;BIR1;GIRK-2;GIRK2;KATP-2;KATP2;KCNJ7;KIR3.2;MGC126596;hiGIRK2	potassium inwardly-rectifying channel, subfamily J, member 6	0.86	0.7	0.78	0.54
CSF3R	CSF3R;CD114;GCSFR	colony stimulating factor 3 receptor (granulocyte)	0.86	0.73	0.78	0.54
CHMP4A	CHMP4A;C14orf123;CHMP4;CHMP4B;FLJ61658;HSPC134;MGC142093;MGC142095;SHAX2;SNF7;SNF7-1;VPS32-1;VPS32A	charged multivesicular body protein 4A	1.17	0.99	0.79	0.76
SERINC1	SERINC1;KIAA1253;TDE1L;TDE2;TMS-2	serine incorporator 1	1.17	0.98	0.79	0.75
TOMM22	TOMM22;1C9-2;MST065;MSTP065;TOM22	translocase of outer mitochondrial membrane 22 homolog (yeast)	1.15	0.96	0.79	0.67
IER5	IER5;MGC102760;SBBI48	immediate early response 5	1.08	0.89	0.79	0.67
AGFG1	AGFG1;DKFZp686I15205;HRB;MGC116938;MGC116940;RAB;RIP	ArfGAP with FG repeats 1	1.06	0.92	0.79	0.68
ATP6V0B	ATP6V0B;ATP6F;HATPL;VMA16	ATPase, H+ transporting, lysosomal 21kDa, V0 subunit b	1.05	0.86	0.79	0.65
SMARCA1	SMARCA1;DKFZp686D1623;FLJ41547;ISWI;NURF140;SNF2L;SNF2L1;SNF2LB;SNF2LT;SWI;SWI2	SWI/SNF related, matrix associated, actin dependent regulator of chromatin, subfamily a, member 1	1.04	0.92	0.79	0.63
SRSF10	SRSF10;DKFZp686H0644;FLJ30749;FLJ43846;FUSIP1;FUSIP2;NSSR;SFRS13;SFRS13A;SRp38;SRrp40;TASR;TASR1;TASR2	serine/arginine-rich splicing factor 10	1.03	0.86	0.79	0.6
RERG	RERG;MGC15754	RAS-like, estrogen-regulated, growth inhibitor	1.02	0.84	0.79	0.65
SOSTDC1	SOSTDC1;DKFZp564D206;ECTODIN;USAG1	sclerostin domain containing 1	1.02	0.83	0.79	0.63
CHMP5	CHMP5;C9orf83;HSPC177;PNAS-2;SNF7DC2;Vps60	charged multivesicular body protein 5	1.02	0.86	0.79	0.6
GUCY2E	GUCY2E;GC-E;GCD	guanylate cyclase 2E	0.99	0.84	0.79	0.59
OR13F1	OR13F1;OR9-6	olfactory receptor, family 13, subfamily F, member 1	0.97	0.78	0.79	0.6
MUC1	MUC1;CD227;EMA;H23AG;KL-6;MAM6;MUC-1;MUC-1/SEC;MUC-1/X;MUC1/ZD;PEM;PEMT;PUM	mucin 1, cell surface associated	0.96	0.8	0.79	0.64
AGBL2	AGBL2;FLJ23598	ATP/GTP binding protein-like 2	0.92	0.74	0.79	0.58
SEMA6D	SEMA6D;FLJ11598;KIAA1479	sema domain, transmembrane domain (TM), and cytoplasmic domain, (semaphorin) 6D	0.92	0.74	0.79	0.53
MYO7A	MYO7A;DFNA11;DFNB2;MYOVIIA;MYU7A;NSRD2;USH1B	myosin VIIA	0.92	0.81	0.79	0.56
NUBP2	NUBP2;CFD1;NUBP1	nucleotide binding protein 2	0.92	0.76	0.79	0.59
CNOT7	CNOT7;CAF1;hCAF-1	CCR4-NOT transcription complex, subunit 7	0.92	0.77	0.79	0.6
C1orf144	C1orf144;DKFZp566C0424;MGC70432	chromosome 1 open reading frame 144	0.91	0.74	0.79	0.55
C7orf64	C7orf64;DKFZP564O0523;DKFZp686D1651;HSPC304	chromosome 7 open reading frame 64	0.9	0.74	0.79	0.6
AMIGO2	AMIGO2;ALI1;DEGA	adhesion molecule with Ig-like domain 2	0.9	0.74	0.79	0.56
HMMR	HMMR;CD168;IHABP;MGC119494;MGC119495;RHAMM	hyaluronan-mediated motility receptor (RHAMM)	0.9	0.79	0.79	0.55
TWF2	TWF2;A6RP;A6r;FLJ56277;PTK9L	twinfilin, actin-binding protein, homolog 2 (Drosophila)	0.9	0.77	0.79	0.55
FAM45A	FAM45A	family with sequence similarity 45, member A	0.9	0.77	0.79	0.51
HADHB	HADHB;ECHB;MGC87480;MTPB;TP-BETA	hydroxyacyl-CoA dehydrogenase/3-ketoacyl-CoA thiolase/enoyl-CoA hydratase (trifunctional protein), beta subunit	0.88	0.76	0.79	0.56
GPM6B	GPM6B;M6B;MGC17150;MGC54284	glycoprotein M6B	0.87	0.72	0.79	0.48
PDK4	PDK4;FLJ40832	pyruvate dehydrogenase kinase, isozyme 4	0.87	0.74	0.79	0.53
COL4A3BP	COL4A3BP;CERT;CERTL;FLJ20597;GPBP;STARD11	collagen, type IV, alpha 3 (Goodpasture antigen) binding protein	0.86	0.74	0.79	0.52
VOPP1	VOPP1;DKFZp564K0822;ECOP;FLJ20532;GASP	vesicular, overexpressed in cancer, prosurvival protein 1	0.86	0.7	0.79	0.47
LRP5	LRP5;BMND1;EVR1;EVR4;HBM;LR3;LRP7;OPPG;OPS;OPTA1;VBCH2	low density lipoprotein receptor-related protein 5	0.85	0.7	0.79	0.53
MAOB	MAOB;MGC26382	monoamine oxidase B	1.09	0.9	0.8	0.71
UNC13B	UNC13B;MGC133279;MGC133280;MUNC13;UNC13;Unc13h2	unc-13 homolog B (C. elegans)	1.06	0.88	0.8	0.67
OCIAD1	OCIAD1;ASRIJ;FLJ20455;MGC111072;OCIA;TPA018	OCIA domain containing 1	1.06	0.9	0.8	0.63
SEMA3G	SEMA3G;FLJ00014;MGC119473;sem2	sema domain, immunoglobulin domain (Ig), short basic domain, secreted, (semaphorin) 3G	1.04	0.86	0.8	0.64
DCST2	DCST2;FLJ32934	DC-STAMP domain containing 2	1.03	0.89	0.8	0.68
HELLS	HELLS;FLJ10339;LSH;PASG;SMARCA6	helicase, lymphoid-specific	1.02	0.91	0.8	0.63
RNASEH2A	RNASEH2A;AGS4;JUNB;RNASEHI;RNHIA;RNHL	ribonuclease H2, subunit A	1.01	0.9	0.8	0.62
RPP25	RPP25;FLJ20374	ribonuclease P/MRP 25kDa subunit	0.99	0.82	0.8	0.56
ZNF7	ZNF7;FLJ38706;HF.16;KOX4;zf30	zinc finger protein 7	0.98	0.79	0.8	0.54
H2AFB3	H2AFB3;H2ABBD;H2AFB;H2AFB2	H2A histone family, member B3	0.98	0.84	0.8	0.59
ABCD3	ABCD3;ABC43;PMP70;PXMP1;ZWS2	ATP-binding cassette, sub-family D (ALD), member 3	0.96	0.86	0.8	0.59
NSMCE1	NSMCE1;NSE1	non-SMC element 1 homolog (S. cerevisiae)	0.95	0.78	0.8	0.59
CRELD2	CRELD2;DKFZp667O055;MGC11256	cysteine-rich with EGF-like domains 2	0.94	0.78	0.8	0.58
FAM20B	FAM20B;gxk1	family with sequence similarity 20, member B	0.93	0.77	0.8	0.58
MRPS25	MRPS25;DKFZp313H0817;FLJ00023;MRP-S25;RPMS25	mitochondrial ribosomal protein S25	0.92	0.73	0.8	0.55
MRPS16	MRPS16;COXPD2;FLJ22062;FLJ40972;MRP-S16;RPMS16	mitochondrial ribosomal protein S16	0.92	0.74	0.8	0.52
PBK	PBK;CT84;FLJ14385;Nori-3;SPK;TOPK	PDZ binding kinase	0.92	0.8	0.8	0.57
DYNLRB1	DYNLRB1;BITH;BLP;DNCL2A;DNLC2A;ROBLD1	dynein, light chain, roadblock-type 1	0.9	0.77	0.8	0.58
FXYD5	FXYD5;DYSAD;IWU1;KCT1;OIT2;PRO6241;RIC	FXYD domain containing ion transport regulator 5	0.9	0.73	0.8	0.56
TEX13A	TEX13A;MGC131984	testis expressed 13A	0.88	0.7	0.8	0.49
CLK1	CLK1;CLK;CLK/STY;STY	CDC-like kinase 1	0.88	0.7	0.8	0.58
CT47A1	CT47A1;CT47.1;CT47A10;CT47A11;CT47A12;CT47A2;CT47A3;CT47A4;CT47A5;CT47A6;CT47A7;CT47A8;CT47A9	cancer/testis antigen family 47, member A1	0.88	0.73	0.8	0.52
OR2T1	OR2T1;OR1-25	olfactory receptor, family 2, subfamily T, member 1	0.87	0.68	0.8	0.55
CCDC17	CCDC17;FLJ17921;FLJ33084	coiled-coil domain containing 17	0.87	0.74	0.8	0.58
MATK	MATK;CHK;CTK;DKFZp434N1212;HHYLTK;HYL;HYLTK;Lsk;MGC1708;MGC2101	megakaryocyte-associated tyrosine kinase	0.87	0.69	0.8	0.57
INS-IGF2	INS-IGF2;INSIGF	INS-IGF2 readthrough	0.86	0.72	0.8	0.54
ATG9B	ATG9B;APG9L2;NOS3AS;SONE	ATG9 autophagy related 9 homolog B (S. cerevisiae)	0.86	0.72	0.8	0.49
PPCS	PPCS;FLJ11838;MGC117357;MGC138220	phosphopantothenoylcysteine synthetase	0.86	0.76	0.8	0.52
BIRC8	BIRC8;ILP-2;ILP2;hILP2	baculoviral IAP repeat containing 8	0.85	0.67	0.8	0.6
SSTR4	SSTR4	somatostatin receptor 4	0.85	0.66	0.8	0.6
CCDC144NL	CCDC144NL;MGC87631	coiled-coil domain containing 144 family, N-terminal like	0.7	0.51	0.67	0.34
WNK4	WNK4;PHA2B;PRKWNK4	WNK lysine deficient protein kinase 4	0.65	0.47	0.67	0.34
ZNF322P1	ZNF322P1;FLJ23393;ZNF322;ZNF322B	zinc finger protein 322 pseudogene 1	0.64	0.46	0.69	0.35
WFS1	WFS1;FLJ51211;WFRS;WFS;WFSL	Wolfram syndrome 1 (wolframin)	0.68	0.53	0.7	0.36
C9orf131	C9orf131;FLJ00273;MGC41945	chromosome 9 open reading frame 131	0.64	0.48	0.7	0.33
GMDS	GMDS;GMD;SDR3E1	GDP-mannose 4,6-dehydratase	0.63	0.44	0.71	0.31
FAM134A	FAM134A;C2orf17;DKFZp686C2379;MGC3035	family with sequence similarity 134, member A	0.73	0.58	0.72	0.38
TSPAN5	TSPAN5;NET-4;NET4;TM4SF9;TSPAN-5	tetraspanin 5	0.7	0.52	0.72	0.35
PRMT6	PRMT6;FLJ10559;FLJ51477;HRMT1L6	protein arginine methyltransferase 6	0.68	0.54	0.72	0.37
KCNS2	KCNS2;KV9.2	potassium voltage-gated channel, delayed-rectifier, subfamily S, member 2	0.66	0.49	0.72	0.38
NTM	NTM;HNT;IGLON2;MGC60329;NTRI	neurotrimin	0.66	0.52	0.72	0.38
RUNDC3A	RUNDC3A;RAP2IP;RPIP-8;RPIP8	RUN domain containing 3A	0.64	0.48	0.72	0.39
RNF220	RNF220;C1orf164;FLJ10597	ring finger protein 220	0.63	0.45	0.72	0.4
ACTN3	ACTN3;MGC117002;MGC117005	actinin, alpha 3	0.62	0.44	0.72	0.36
RAD51B	RAD51B;MGC34245;R51H2;RAD51L1;REC2	RAD51 homolog B (S. cerevisiae)	0.81	0.66	0.73	0.45
RRN3	RRN3;A-270G1.2;DKFZp566E104;MGC104238;TIFIA	RRN3 RNA polymerase I transcription factor homolog (S. cerevisiae)	0.7	0.52	0.73	0.35
LGALS14	LGALS14;CLC2;MGC22235;PPL13	lectin, galactoside-binding, soluble, 14	0.68	0.5	0.73	0.34
EYS	EYS;C6orf178;C6orf179;C6orf180;EGFL10;EGFL11;KIAA0663;RP25;SPAM;bA166P24.2;bA307F22.3;bA74E24.1;dJ1018A4.2;dJ22I17.2;dJ303F19.1	eyes shut homolog (Drosophila)	0.68	0.51	0.73	0.39
SEC14L2	SEC14L2;C22orf6;KIAA1186;KIAA1658;MGC65053;SPF;TAP;TAP1	SEC14-like 2 (S. cerevisiae)	0.79	0.63	0.74	0.48
LRRC71	LRRC71;C1orf92;FLJ32884;FLJ77598;MGC45468;RP11-356J7.1	leucine rich repeat containing 71	0.79	0.63	0.74	0.49
PARVG	PARVG	parvin, gamma	0.77	0.6	0.74	0.42
NIF3L1	NIF3L1;ALS2CR1;CALS-7	NIF3 NGG1 interacting factor 3-like 1 (S. pombe)	0.77	0.58	0.74	0.44
SCYL3	SCYL3;PACE-1;PACE1;RP1-97P20.2	SCY1-like 3 (S. cerevisiae)	0.74	0.59	0.74	0.42
TNRC6B	TNRC6B;KIAA1093	trinucleotide repeat containing 6B	0.7	0.55	0.74	0.37
CHRM5	CHRM5;HM5;MGC41838	cholinergic receptor, muscarinic 5	0.68	0.54	0.74	0.41
KRT73	KRT73;IRT6IRS3;K6IRS3;KRT6IRS3;MGC129635;MGC129636	keratin 73	0.66	0.47	0.74	0.36
COBL	COBL;DKFZp686G13227;KIAA0633;MGC131893	cordon-bleu homolog (mouse)	0.66	0.51	0.74	0.34
KRT86	KRT86;FLJ25176;HB6;Hb1;KRTHB1;KRTHB6;MNX;hHb6	keratin 86	0.66	0.5	0.74	0.37
FAM173B	FAM173B;FLJ20667	family with sequence similarity 173, member B	0.84	0.65	0.75	0.49

CLTCL1	CLTCL1;CHC22;CLH22;CLTCL;CLTD;FLJ36032	clathrin, heavy chain-like 1	0.83	0.64	0.75	0.48
R3HDML	R3HDML;MGC129564;dJ881L22.3	R3H domain containing-like	0.78	0.6	0.75	0.41
TMEM53	TMEM53;FLJ22353;NET4	transmembrane protein 53	0.78	0.62	0.75	0.44
MAPKAPK3	MAPKAPK3;3PK;MAPKAP3;MAPKAPK-3	mitogen-activated protein kinase-activated protein kinase 3	0.78	0.64	0.75	0.45
RPS6KA3	RPS6KA3;CLS;HU-3;ISPK-1;MAPKAPK1B;MRX19;RSK;RSK2;S6K-alpha3;p90-RSK2;pp90RSK2	ribosomal protein S6 kinase, 90kDa, polypeptide 3	0.77	0.58	0.75	0.48
PRAMEF9	PRAMEF9;PRAMEF15	PRAME family member 9	0.77	0.6	0.75	0.45
TBRG1	TBRG1;FLJ14621;FLJ25020;FLJ90113;MGC129890;NIAM;TB-5	transforming growth factor beta regulator 1	0.76	0.59	0.75	0.45
CREB1	CREB1;CREB;MGC9284	cAMP responsive element binding protein 1	0.76	0.62	0.75	0.46
PCDHB12	PCDHB12;PCDH-BETA12	protocadherin beta 12	0.72	0.55	0.75	0.37
MTRF1	MTRF1;MGC47721;MRF1;MTTRF1;RF1	mitochondrial translational release factor 1	0.72	0.56	0.75	0.43
RYBP	RYBP;AAP1;DEDAF;YEAF1	RING1 and YY1 binding protein	0.72	0.53	0.75	0.39
SND1	SND1;TDRD11;p100	staphylococcal nuclease and tudor domain containing 1	0.72	0.6	0.75	0.41
NUMB	NUMB;S171	numb homolog (Drosophila)	0.7	0.54	0.75	0.42
GDF15	GDF15;GDF-15;MIC-1;MIC1;NAG-1;PDF;PLAB;PTGFB	growth differentiation factor 15	0.69	0.5	0.75	0.39
DPYS	DPYS;DHP;DHPase	dihydropyrimidinase	0.68	0.51	0.75	0.34
APOBEC3D	APOBEC3D;APOBEC3DE;APOBEC3E;ARP6	apolipoprotein B mRNA editing enzyme, catalytic polypeptide-like 3D	0.68	0.54	0.75	0.41
PLAG1	PLAG1;PSA;SGPA;ZNF912	pleiomorphic adenoma gene 1	0.67	0.52	0.75	0.4
NRG1	NRG1;ARIA;GGF;GGF2;HGL;HRG;HRG1;HRGA;MST131;NDF;SMDF	neuregulin 1	0.66	0.53	0.75	0.4
C3orf37	C3orf37;MGC111075	chromosome 3 open reading frame 37	0.64	0.52	0.75	0.35
DDX41	DDX41;ABS;MGC8828	DEAD (Asp-Glu-Ala-Asp) box polypeptide 41	0.62	0.44	0.75	0.35
40422	SEPT10;FLJ11619	septin 10	0.82	0.64	0.76	0.46
USP27X	USP27X;USP22L;USP27	ubiquitin specific peptidase 27, X-linked	0.73	0.61	0.76	0.42
LILRB1	LILRB1;CD85;CD85J;FLJ37515;ILT2;LIR-1;LIR1;MIR-7;MIR7	leukocyte immunoglobulin-like receptor, subfamily B (with TM and ITIM domains), member 1	0.72	0.59	0.76	0.45
PPIC	PPIC;CYPC;MGC3673	peptidylprolyl isomerase C (cyclophilin C)	0.72	0.6	0.76	0.44
FOXK2	FOXK2;ILF;ILF-1;ILF1	forkhead box K2	0.7	0.56	0.76	0.42
COMP	COMP;EDM1;EPD1;MED;MGC131819;MGC149768;PSACH;THBS5	cartilage oligomeric matrix protein	0.67	0.52	0.76	0.4
CCL15	CCL15;HCC-2;HMRP-2B;LKN-1;LKN1;MIP-1D;MIP-5;MRP-2B;NCC-3;NCC3;SCYA15;SCYL3;SY15	chemokine (C-C motif) ligand 15	0.65	0.46	0.76	0.39
ABHD8	ABHD8;FLJ11743;MGC14280;MGC2512	abhydrolase domain containing 8	0.63	0.5	0.76	0.38
NTN3	NTN3;NTN2L	netrin 3	0.63	0.48	0.76	0.39
TMCC2	TMCC2;FLJ38497;HUCEP11;KIAA0481	transmembrane and coiled-coil domain family 2	0.84	0.67	0.77	0.5
SGK3	SGK3;CISK;DKFZp781N0293;SGK2;SGKL	serum/glucocorticoid regulated kinase family, member 3	0.83	0.7	0.77	0.49
PRKD2	PRKD2;PKD2;nPKC-D2	protein kinase D2	0.81	0.67	0.77	0.48
NRP2	NRP2;MGC126574;NP2;NPN2;PRO2714;VEGF165R2	neuropilin 2	0.8	0.64	0.77	0.5
MAF1	MAF1;MGC20332;MGC31779;MGC39758	MAF1 homolog (S. cerevisiae)	0.8	0.64	0.77	0.49
PIK3R2	PIK3R2;P85B;p85;p85-BETA	phosphoinositide-3-kinase, regulatory subunit 2 (beta)	0.78	0.59	0.77	0.49
IGFBP3	IGFBP3;BP-53;IBP3	insulin-like growth factor binding protein 3	0.77	0.63	0.77	0.44
A2M	A2M;A2MD;CPAMD5;DKFZp779B086;FWP007;S863-7	alpha-2-macroglobulin	0.75	0.63	0.77	0.43
ZFPM2	ZFPM2;DIH3;FOG2;MGC129663;MGC129664;ZNF89B;hFOG-2	zinc finger protein, multitype 2	0.74	0.55	0.77	0.41
PHKG2	PHKG2;GSD9C	phosphorylase kinase, gamma 2 (testis)	0.74	0.57	0.77	0.48
INCA1	INCA1;MGC148150;MGC148151	inhibitor of CDK, cyclin A1 interacting protein 1	0.71	0.56	0.77	0.44
KREMEN2	KREMEN2;KRM2;MGC10791;MGC16709	kringle containing transmembrane protein 2	0.71	0.54	0.77	0.4
FOSL1	FOSL1;FRA;FRA1;fra-1	FOS-like antigen 1	0.66	0.48	0.77	0.38
SRP14	SRP14;ALURBP;MGC14326	signal recognition particle 14kDa (homologous Alu RNA binding protein)	0.66	0.52	0.77	0.41
DDX11	DDX11;CHL1;CHLR1;KRG2;MGC133249;MGC9335	DEAD/H (Asp-Glu-Ala-Asp/His) box polypeptide 11	0.65	0.49	0.77	0.38
TMCO3	TMCO3;C13orf11;FLJ20623	transmembrane and coiled-coil domains 3	0.65	0.48	0.77	0.39
ZNF460	ZNF460;HZF8;ZNF272	zinc finger protein 460	0.65	0.47	0.77	0.38
ARPC1B	ARPC1B;ARC41;p40-ARC;p41-ARC	actin related protein 2/3 complex, subunit 1B, 41kDa	0.62	0.46	0.77	0.35
PKD2L1	PKD2L1;PCL;PKD2L;PKDL;TRPP3	polycystic kidney disease 2-like 1	0.82	0.66	0.78	0.51
CHCHD1	CHCHD1;C10orf34;C2360;FLJ25854	coiled-coil-helix-coiled-coil-helix domain containing 1	0.82	0.66	0.78	0.5
ZNF664	ZNF664;DKFZp761B128;MGC126579;ZFOC1;ZNF176	zinc finger protein 664	0.8	0.68	0.78	0.45
IL17D	IL17D;FLJ30846;IL-17D;IL-22;IL-27;IL27	interleukin 17D	0.78	0.6	0.78	0.45
ERRFI1	ERRFI1;GENE-33;MIG-6;MIG6;RALT	ERBB receptor feedback inhibitor 1	0.78	0.62	0.78	0.46
NKX2-2	NKX2-2;NKX2.2;NKX2B	NK2 homeobox 2	0.77	0.6	0.78	0.4
HIST1H4J	HIST1H4J;H4/e;H4F2iv;H4FE;HIST1H4A;HIST1H4B;HIST1H4C;HIST1H4D;HIST1H4E;HIST1H4F;HIST1H4H;HIST1H4I;HIST1H4K;HIST1H4L;HIST2H4A;HIST2H4B;HIST4H4;MGC166960;MGC29783;dJ160A22.2	histone cluster 1, H4j	0.77	0.6	0.78	0.45
LINC00303	LINC00303;C1orf157;FLJ40343;MGC120329;MGC120330;MGC120332;NCRNA00303	long intergenic non-protein coding RNA 303	0.77	0.66	0.78	0.44
MBD1	MBD1;CXXC3;PCM1;RFT	methyl-CpG binding domain protein 1	0.76	0.59	0.78	0.44
HOXD10	HOXD10;HOX4;HOX4D;HOX4E;Hox-4.4	homeobox D10	0.75	0.57	0.78	0.49
MYOM2	MYOM2;TTNAP	myomesin (M-protein) 2, 165kDa	0.74	0.57	0.78	0.4
SOX6	SOX6;HSSOX6;SOXD	SRY (sex determining region Y)-box 6	0.74	0.64	0.78	0.46
TRIM5	TRIM5;RNF88;TRIM5alpha	tripartite motif containing 5	0.74	0.57	0.78	0.51
CARD17	CARD17;INCA	caspase recruitment domain family, member 17	0.72	0.54	0.78	0.41
CT47A3	CT47A3;CT47.3;CT47A1;CT47A10;CT47A11;CT47A12;CT47A2;CT47A4;CT47A5;CT47A6;CT47A7;CT47A8;CT47A9	cancer/testis antigen family 47, member A3	0.72	0.58	0.78	0.39
TULP3	TULP3;MGC45295;TUBL3	tubby like protein 3	0.71	0.57	0.78	0.43
ENY2	ENY2;FLJ20480;e(y)2	enhancer of yellow 2 homolog (Drosophila)	0.7	0.52	0.78	0.4
TSEN34	TSEN34;LENG5;PCH2C;SEN34;SEN34L	tRNA splicing endonuclease 34 homolog (S. cerevisiae)	0.68	0.49	0.78	0.43
CHD4	CHD4;DKFZp686E06161;Mi-2b;Mi2-BETA	chromodomain helicase DNA binding protein 4	0.64	0.5	0.78	0.44
PIK3CB	PIK3CB;DKFZp779K1237;MGC133043;P110BETA;PI3K;PI3KBETA;PIK3C1	phosphoinositide-3-kinase, catalytic, beta polypeptide	0.84	0.72	0.79	0.51
MIOS	MIOS;FLJ20323	missing oocyte, meiosis regulator, homolog (Drosophila)	0.84	0.66	0.79	0.5
SP2	SP2	Sp2 transcription factor	0.83	0.64	0.79	0.5
STRBP	STRBP;DKFZp434N214;FLJ11307;FLJ14223;FLJ14984;FLJ21960;ILF3L;MGC21529;MGC3405;SPNR;p74	spermatid perinuclear RNA binding protein	0.83	0.7	0.79	0.56
ZNF333	ZNF333;KIAA1806	zinc finger protein 333	0.82	0.68	0.79	0.46
TNNT3	TNNT3;DKFZp779M2348;TNTF	troponin T type 3 (skeletal, fast)	0.82	0.68	0.79	0.55
RAI2	RAI2	retinoic acid induced 2	0.82	0.71	0.79	0.52
COMTD1	COMTD1;FLJ23841	catechol-O-methyltransferase domain containing 1	0.82	0.64	0.79	0.49
P2RX7	P2RX7;MGC20089;P2X7	purinergic receptor P2X, ligand-gated ion channel, 7	0.8	0.66	0.79	0.51
RNASE1	RNASE1;MGC12408;RIB1;RNS1	ribonuclease, RNase A family, 1 (pancreatic)	0.8	0.61	0.79	0.48
HMBOX1	HMBOX1;FLJ21616;HNF1LA;PBHNF	homeobox containing 1	0.79	0.62	0.79	0.45
SCML1	SCML1	sex comb on midleg-like 1 (Drosophila)	0.78	0.69	0.79	0.47
C9orf114	C9orf114;CENP-32;DKFZp566D143;HSPC109;MGC29492;RP11-545E17.7	chromosome 9 open reading frame 114	0.76	0.58	0.79	0.44
OPHN1	OPHN1;ARHGAP41;MRX60;OPN1	oligophrenin 1	0.76	0.6	0.79	0.42
DDX49	DDX49;FLJ10432;R27090_2	DEAD (Asp-Glu-Ala-Asp) box polypeptide 49	0.74	0.57	0.79	0.44
EMX2	EMX2	empty spiracles homeobox 2	0.72	0.56	0.79	0.4
SCN1B	SCN1B;GEFSP1	sodium channel, voltage-gated, type I, beta	0.72	0.56	0.79	0.44
GPR172A	GPR172A;D15Ertd747e;GPCR41;PAR1;RFT3;hRFT3	G protein-coupled receptor 172A	0.72	0.6	0.79	0.48
THOC4	THOC4;ALY;ALY/REF;BEF;REF	THO complex 4	0.72	0.56	0.79	0.4
DBF4	DBF4;ASK;CHIF;DBF4A;ZDBF1	DBF4 homolog (S. cerevisiae)	0.71	0.6	0.79	0.43
CC2D2A	CC2D2A;JBTS9;KIAA1345;MKS6	coiled-coil and C2 domain containing 2A	0.64	0.52	0.79	0.35
PCDHAC2	PCDHAC2;MGC71598;PCDH-ALPHA-C2	protocadherin alpha subfamily C, 2	0.62	0.52	0.79	0.36
HBM	HBM;HBAP2	hemoglobin, mu	0.84	0.65	0.8	0.49
GBF1	GBF1;ARF1GEF;FLJ21263;FLJ21500;KIAA0248;MGC134877;MGC134878	golgi brefeldin A resistant guanine nucleotide exchange factor 1	0.84	0.66	0.8	0.55
TCF19	TCF19;SC1;SC1-1	transcription factor 19	0.84	0.72	0.8	0.54
SF1	SF1;BBP;D11S636;MBBP;ZFM1;ZNF162	splicing factor 1	0.82	0.64	0.8	0.46
CHIC1	CHIC1;BRX;DKFZp313P1931;DKFZp686F2342	cysteine-rich hydrophobic domain 1	0.81	0.68	0.8	0.48
ECEL1	ECEL1;DINE;ECEX;XCE	endothelin converting enzyme-like 1	0.8	0.62	0.8	0.47
FAM189B	FAM189B;C1orf2;COTE1	family with sequence similarity 189, member B	0.8	0.66	0.8	0.5
NAAA	NAAA;ASAHL;PLT	N-acylethanolamine acid amidase	0.79	0.62	0.8	0.48
BIK	BIK;BIP1;BP4;NBK	BCL2-interacting killer (apoptosis-inducing)	0.78	0.6	0.8	0.49
SSX2IP	SSX2IP;ADIP;FLJ10848;KIAA0923;MGC75026	synovial sarcoma, X breakpoint 2 interacting protein	0.76	0.6	0.8	0.41
ZNF789	ZNF789	zinc finger protein 789	0.74	0.66	0.8	0.43
ARHGAP19	ARHGAP19;DKFZp313K217;MGC138804;MGC138805;MGC14258	Rho GTPase activating protein 19	0.74	0.62	0.8	0.5
CMBL	CMBL;FLJ23617	carboxymethylenebutenolidase homolog (Pseudomonas)	0.73	0.64	0.8	0.44
C1orf115	C1orf115;FLJ14146;RP11-322F10.4	chromosome 1 open reading frame 115	0.73	0.55	0.8	0.45
HIST2H2AA4	HIST2H2AA4;H2A/R;HIST2H2AA3	histone cluster 2, H2aa4	0.73	0.58	0.8	0.41
TBC1D13	TBC1D13;FLJ10743	TBC1 domain family, member 13	0.72	0.6	0.8	0.4
RBBP8	RBBP8;CTIP;RIM;SAE2	retinoblastoma binding protein 8	0.7	0.54	0.8	0.41
LINC00176	LINC00176;DKFZp434G015;FLJ17673;FLJ27267;NCRNA00176;PRR17	long intergenic non-protein coding RNA 176	0.7	0.58	0.8	0.43
CYB5RL	CYB5RL;FLJ16295	cytochrome b5 reductase-like	0.69	0.53	0.8	0.42
SLC23A3	SLC23A3;E2BP3;FLJ31168;SVCT3;Yspl1	solute carrier family 23 (nucleobase transporters), member 3	0.68	0.52	0.8	0.45
NAT10	NAT10;ALP;DKFZp434C116;FLJ10774;FLJ12179;FLJ23850;KIAA1709;NET43	N-acetyltransferase 10 (GCN5-related)	0.68	0.54	0.8	0.42
ARNTL	ARNTL;BMAL1;BMAL1c;JAP3;MGC47515;MOP3;PASD3;TIC;bHLHe5	aryl hydrocarbon receptor nuclear translocator-like	0.66	0.59	0.8	0.41
TIMM13	TIMM13;TIM13;TIM13B;TIMM13A;TIMM13B;ppv1	translocase of inner mitochondrial membrane 13 homolog (yeast)	0.66	0.55	0.8	0.42
SMCP	SMCP;HSMCSGEN1;MCS;MCSP;MGC26305;MGC26519	sperm mitochondria-associated cysteine-rich protein	0.62	0.46	0.8	0.38
GMPPA	GMPPA	GDP-mannose pyrophosphorylase A	0.62	0.52	0.8	0.35
RTN2	RTN2;NSP2;NSPL1	reticulon 2	1.3	1	0.81	0.85
C12orf66	C12orf66;FLJ32549	chromosome 12 open reading frame 66	1.3	1	0.81	0.84
RAB3A	RAB3A	RAB3A, member RAS oncogene family	1.29	1.02	0.81	0.8
MPP7	MPP7;FLJ32798	membrane protein, palmitoylated 7 (MAGUK p55 subfamily member 7)	1.23	0.96	0.81	0.69
TMEM143	TMEM143;FLJ10922	transmembrane protein 143	1.15	0.83	0.81	0.65
ADAM20	ADAM20	ADAM metallopeptidase domain 20	1.05	0.78	0.81	0.59
SETDB2	SETDB2;C13orf4;CLLD8;CLLL8;DKFZp586I0123;DKFZp761J1217;KMT1F	SET domain, bifurcated 2	0.98	0.73	0.81	0.65
SLC5A6	SLC5A6;SMVT	solute carrier family 5 (sodium-dependent vitamin transporter), member 6	0.97	0.72	0.81	0.64
PALMD	PALMD;C1orf11;FLJ20271;PALML	palmdelphin	0.94	0.68	0.81	0.57
CASZ1	CASZ1;CST;FLJ12223;FLJ20321;SRG;ZNF693;dJ734G22.1	castor zinc finger 1	1.38	1.12	0.82	0.81
RANBP3	RANBP3;DKFZp586I1520	RAN binding protein 3	1.36	1.09	0.82	0.82
RFFL	RFFL;CARP-2;CARP2;FRING;RIFIFYLIN;RNF189;RNF34L	ring finger and FYVE-like domain containing 1	1.31	1.02	0.82	0.73
RNF5	RNF5;RING5;RMA1	ring finger protein 5	1.25	0.99	0.82	0.71
ADD1	ADD1;ADDA;MGC3339;MGC44427	adducin 1 (alpha)	1.23	0.96	0.82	0.69
PPP4C	PPP4C;PP4;PP4C;PPH3;PPP4;PPX	protein phosphatase 4, catalytic subunit	1.11	0.85	0.82	0.64
CAND1	CAND1;DKFZp434M1414;FLJ10114;FLJ10929;FLJ38691;FLJ90441;KIAA0829;TIP120;TIP120A	cullin-associated and neddylation-dissociated 1	1.05	0.8	0.82	0.59

SLC38A10	SLC38A10;FLJ35718;FLJ46399;MGC15523	solute carrier family 38, member 10	0.97	0.71	0.82	0.6
MIR22HG	MIR22HG;C17orf91;DKFZp686O06159;MGC14376;MGC149751	MIR22 host gene (non-protein coding)	0.96	0.7	0.82	0.56
TMEM101	TMEM101;FLJ23987;MGC4251	transmembrane protein 101	1.46	1.21	0.83	0.87
TEPP	TEPP;MGC129930;MGC129931	testis, prostate and placenta expressed	1.42	1.13	0.83	0.86
FBXL17	FBXL17;DKFZp434C1715;FBXO13;Fbl17;Fbx13;MGC161422;MGC161424	F-box and leucine-rich repeat protein 17	1.4	1.12	0.83	0.81
EBAG9	EBAG9;EB9;PDAF;RCAS1	estrogen receptor binding site associated, antigen, 9	1.2	0.9	0.83	0.74
PRAF2	PRAF2;FLJ57916	PRA1 domain family, member 2	1.18	0.88	0.83	0.7
PTPN20B	PTPN20B;DKFZp566K0524;DKFZp781P23155;bA42B19.1	protein tyrosine phosphatase, non-receptor type 20B	1.17	0.9	0.83	0.67
MARVELD3	MARVELD3;FLJ32280;MARVD3;MRVLDC3	MARVEL domain containing 3	1.12	0.84	0.83	0.67
STAG2	STAG2;DKFZp686P168;DKFZp781H1753;FLJ25871;SA-2;SA2;SCC3B;bA517O1.1	stromal antigen 2	1.12	0.84	0.83	0.7
CDC42EP3	CDC42EP3;BORG2;CEP3;FLJ46903;UB1	CDC42 effector protein (Rho GTPase binding) 3	1.08	0.82	0.83	0.68
FAM200A	FAM200A;C7orf38;DKFZp727G131;FLJ36794	family with sequence similarity 200, member A	1.03	0.76	0.83	0.59
SURF6	SURF6;FLJ30322;RRP14	surfeit 6	1	0.75	0.83	0.62
C9orf170	C9orf170;FLJ45537	chromosome 9 open reading frame 170	1	0.75	0.83	0.6
LRRN4CL	LRRN4CL;MGC61707	LRRN4 C-terminal like	1.38	1.09	0.84	0.92
UBE2D4	UBE2D4;FLJ32004;HBUCE1	ubiquitin-conjugating enzyme E2D 4 (putative)	1.15	0.88	0.84	0.67
ZNF468	ZNF468;DKFZp781B1474;DKFZp781N07108	zinc finger protein 468	1.14	0.86	0.84	0.67
CIB3	CIB3;KIP3;MGC138405;MGC142151;MGC96922	calcium and integrin binding family member 3	1.12	0.86	0.84	0.71
PDZD8	PDZD8;FLJ25412;FLJ34427;PDZK8;bA129M16.2	PDZ domain containing 8	1.06	0.79	0.84	0.62
IGFBP1	IGFBP1;AFBP;IBP1;IGF-BP25;PP12;hIGFBP-1	insulin-like growth factor binding protein 1	1.02	0.77	0.84	0.61
UBE3C	UBE3C;KIAA0010;KIAA10	ubiquitin protein ligase E3C	1.43	1.12	0.85	0.84
C1orf51	C1orf51;FLJ25889	chromosome 1 open reading frame 51	1.34	1.07	0.85	0.91
ZNF675	ZNF675;FLJ36350;TBZF;TIZ	zinc finger protein 675	1.22	0.92	0.85	0.72
UBE2R2	UBE2R2;CDC34B;E2-CDC34B;FLJ20419;MGC10481;UBC3B	ubiquitin-conjugating enzyme E2R 2	1.16	0.9	0.85	0.68
CUL4A	CUL4A	cullin 4A	1.47	1.18	0.87	0.89
APBA2	APBA2;D15S1518E;HsT16821;LIN-10;MGC99508;MGC:14091;MINT2;X11-BETA;X11L	amyloid beta (A4) precursor protein-binding, family A, member 2	1.42	1.1	0.87	0.92
ERAP2	ERAP2;FLJ23633;FLJ23701;FLJ23807;L-RAP;LRAP	endoplasmic reticulum aminopeptidase 2	1.35	1.09	0.87	0.82
OR2A1	OR2A1;OR2A42	olfactory receptor, family 2, subfamily A, member 1	1.27	1	0.87	0.83
TCTEX1D2	TCTEX1D2;MGC33212	Tctex1 domain containing 2	1.25	1	0.89	0.84
CLSTN2	CLSTN2;ALC-GAMMA;CDHR13;CS2;CSTN2;FLJ39113;FLJ39499;MGC119560;alcagamma	calsyntenin 2	1.24	1.02	0.81	0.68
ADAMTS15	ADAMTS15;MGC126403	ADAM metallopeptidase with thrombospondin type 1 motif, 15	1.24	1	0.81	0.75
FAM22G	FAM22G	family with sequence similarity 22, member G	1.23	1.02	0.81	0.78
HCN4	HCN4;SSS2	hyperpolarization activated cyclic nucleotide-gated potassium channel 4	1.19	0.96	0.81	0.75
FADD	FADD;MGC8528;MORT1	Fas (TNFRSF6)-associated via death domain	1.18	0.98	0.81	0.78
MMP17	MMP17;MT4-MMP	matrix metallopeptidase 17 (membrane-inserted)	1.15	0.91	0.81	0.68
FGF23	FGF23;ADHR;HPDR2;HYPF;PHPTC	fibroblast growth factor 23	1.14	0.94	0.81	0.75
RABL2A	RABL2A;FLJ78724;MGC117180	RAB, member of RAS oncogene family-like 2A	1.13	0.9	0.81	0.63
MAP1LC3C	MAP1LC3C;ATG8J;LC3C	microtubule-associated protein 1 light chain 3 gamma	1.04	0.82	0.81	0.66
CCDC76	CCDC76;FLJ10287;FLJ11219	coiled-coil domain containing 76	1.03	0.8	0.81	0.61
TXNDC5	TXNDC5;ENDOPDI;ERP46;FLJ21789;HCC-2;MGC3178;PDIA15;STRF8;UNQ364	thioredoxin domain containing 5 (endoplasmic reticulum)	1.03	0.82	0.81	0.71
XRCC5	XRCC5;FLJ39089;KARP-1;KARP1;KU80;KUB2;Ku86;NFIV	X-ray repair complementing defective repair in Chinese hamster cells 5 (double-strand-break rejoining)	1.02	0.81	0.81	0.57
RSPH4A	RSPH4A;CILD11;FLJ37974;MGC126303;RSHL3;RSPH6B;dJ412I7.1	radial spoke head 4 homolog A (Chlamydomonas)	1	0.79	0.81	0.62
TANK	TANK;I-TRAF;TRAF2	TRAF family member-associated NFKB activator	0.99	0.78	0.81	0.63
PRDM15	PRDM15;C21orf83;PFM15;ZNF298	PR domain containing 15	0.99	0.78	0.81	0.65
ARRDC1	ARRDC1;MGC40555	arrestin domain containing 1	0.98	0.77	0.81	0.62
SORBS2	SORBS2;ARGBP2;FLJ93447;KIAA0777;PRO0618	sorbin and SH3 domain containing 2	0.94	0.72	0.81	0.58
DCUN1D2	DCUN1D2;C13orf17;FLJ10704;FLJ20092	DCN1, defective in cullin neddylation 1, domain containing 2 (S. cerevisiae)	0.89	0.66	0.81	0.49
FASTKD2	FASTKD2;KIAA0971	FAST kinase domains 2	1.16	0.92	0.82	0.7
ZCWPW1	ZCWPW1;DKFZp434N0510;FLJ10057;ZCW1	zinc finger, CW type with PWWP domain 1	1.15	0.92	0.82	0.7
SLC23A1	SLC23A1;MGC22361;SLC23A2;SVCT1;YSPL3	solute carrier family 23 (nucleobase transporters), member 1	1.12	0.9	0.82	0.67
SERAC1	SERAC1;FLJ14917;FLJ30544	serine active site containing 1	1.12	0.9	0.82	0.72
NLRP3	NLRP3;AGTAVPRL;AII;AVP;C1orf7;CIAS1;CLR1.1;FCAS;FCU;FLJ95925;MWS;NALP3;PYPAF1	NLR family, pyrin domain containing 3	1.08	0.86	0.82	0.63
NLRP12	NLRP12;CLR19.3;FCAS2;NALP12;PAN6;PYPAF7;RNO;RNO2	NLR family, pyrin domain containing 12	1.08	0.86	0.82	0.64
PCDHB1	PCDHB1;MGC138301;MGC138303;PCDH-BETA1	protocadherin beta 1	1.05	0.85	0.82	0.64
C9orf3	C9orf3;AOPEP;AP-O;APO;C90RF3;FLJ14675;FLJ40923;FLJ55832;ONPEP	chromosome 9 open reading frame 3	1.03	0.83	0.82	0.63
INSC	INSC	inscuteable homolog (Drosophila)	1.02	0.78	0.82	0.64
LEPRE1	LEPRE1;GROS1;MGC117314;OI8;P3H1	leucine proline-enriched proteoglycan (leprecan) 1	1.02	0.82	0.82	0.58
KL	KL	klotho	0.99	0.78	0.82	0.58
MAPK10	MAPK10;FLJ12099;FLJ33785;JNK3;JNK3A;MGC50974;PRKM10;p493F12;p54bSAPK	mitogen-activated protein kinase 10	0.97	0.77	0.82	0.69
OSBP	OSBP;OSBP1	oxysterol binding protein	0.94	0.73	0.82	0.57
NT5DC3	NT5DC3;FLJ11266;TU12B1-TY;TU12B1TY	5'-nucleotidase domain containing 3	0.86	0.64	0.82	0.49
MED26	MED26;CRSP7;CRSP70	mediator complex subunit 26	1.26	1.02	0.83	0.8
HCK	HCK;JTK9	hemopoietic cell kinase	1.18	0.98	0.83	0.81
CFD	CFD;ADIPSIN;ADN;DF;PFD	complement factor D (adipsin)	1.14	0.93	0.83	0.69
LRRC1	LRRC1;FLJ10775;FLJ11834;LANO;dJ523E19.1	leucine rich repeat containing 1	1.14	0.94	0.83	0.64
TRIM27	TRIM27;RFP;RNF76	tripartite motif containing 27	1.14	0.94	0.83	0.83
LUZP2	LUZP2;FLJ45795;KFSP2566;PRO6246	leucine zipper protein 2	1.13	0.92	0.83	0.69
ZNF284	ZNF284;DKFZp781F1775;ZNF284L	zinc finger protein 284	1.12	0.9	0.83	0.7
QSER1	QSER1;FLJ21924	glutamine and serine rich 1	1.07	0.87	0.83	0.65
TBX3	TBX3;TBX3-ISO;UMS;XHL	T-box 3	1.04	0.83	0.83	0.62
ZNF714	ZNF714;DKFZp547O168;FLJ33668;FLJ43674	zinc finger protein 714	1.04	0.84	0.83	0.64
GPR56	GPR56;BFPP;DKFZp781L1398;TM7LN4;TM7XN1	G protein-coupled receptor 56	1.03	0.8	0.83	0.6
EXT2	EXT2;SOTV	exostosin 2	1.02	0.82	0.83	0.61
COX5B	COX5B;COXVB	cytochrome c oxidase subunit Vb	1.01	0.8	0.83	0.63
ZNF565	ZNF565;FLJ36991	zinc finger protein 565	1	0.8	0.83	0.58
CHST4	CHST4;GST3;GlcNAc6ST2;HECGLCNAC6ST;LSST	carbohydrate (N-acetylglucosamine 6-O) sulfotransferase 4	0.98	0.76	0.83	0.6
FOXO3	FOXO3;AF6q21;DKFZp781A0677;FKHRL1;FKHRL1P2;FOXO2;FOXO3A;MGC12739;MGC31925	forkhead box O3	0.98	0.75	0.83	0.66
B3GNT6	B3GNT6;B3Gn-T6;IMAGE:4907098;MGC119334;MGC119336;MGC119337	UDP-GlcNAc:betaGal beta-1,3-N-acetylglucosaminyltransferase 6 (core 3 synthase)	0.93	0.73	0.83	0.57
KCNA2	KCNA2;HBK5;HK4;HUKIV;KV1.2;MGC50217;MK2;NGK1;RBK2	potassium voltage-gated channel, shaker-related subfamily, member 2	1.28	1.08	0.84	0.84
ZNF491	ZNF491;FLJ34791;MGC126639;MGC126641	zinc finger protein 491	1.25	1.03	0.84	0.78
VPS8	VPS8;FLJ32099;KIAA0804	vacuolar protein sorting 8 homolog (S. cerevisiae)	1.16	0.96	0.84	0.86
NFU1	NFU1;HIRIP;HIRIP5;MGC142252;MGC142254;MMDS1;NIFUC;Nfu;NifU	NFU1 iron-sulfur cluster scaffold homolog (S. cerevisiae)	1.14	0.94	0.84	0.7
GPR173	GPR173;SREB3	G protein-coupled receptor 173	1.09	0.89	0.84	0.81
PTPN1	PTPN1;PTP1B	protein tyrosine phosphatase, non-receptor type 1	1.08	0.84	0.84	0.63
OR4F3	OR4F3;OR4F16;OR4F29	olfactory receptor, family 4, subfamily F, member 3	1.06	0.86	0.84	0.72
EVPL	EVPL;EVPK	envoplakin	1.02	0.82	0.84	0.59
TDP1	TDP1;FLJ11090;MGC104252	tyrosyl-DNA phosphodiesterase 1	1.02	0.82	0.84	0.59
LRRC37B	LRRC37B	leucine rich repeat containing 37B	0.94	0.7	0.84	0.55
NUDCD1	NUDCD1;CML66;FLJ14991;OVA66	NudC domain containing 1	0.92	0.68	0.84	0.53
MXRA8	MXRA8;DKFZp586E2023;MGC3047	matrix-remodelling associated 8	0.9	0.68	0.84	0.54
IKZF3	IKZF3;AIO;AIOLOS;ZNFN1A3	IKAROS family zinc finger 3 (Aiolos)	1.25	1.05	0.85	0.74
MOAP1	MOAP1;MAP-1;PNMA4	modulator of apoptosis 1	1.23	1.02	0.85	0.85
PNMAL1	PNMAL1;FLJ10781	PNMA-like 1	1.19	0.95	0.85	0.8
ATP8B1	ATP8B1;ATPIC;BRIC;FIC1;PFIC;PFIC1	ATPase, aminophospholipid transporter, class I, type 8B, member 1	1.18	0.94	0.85	0.83
FBXO48	FBXO48	F-box protein 48	1.17	0.95	0.85	0.69
RNF168	RNF168;FLJ35794;FLJ39749	ring finger protein 168	1.15	0.93	0.85	0.67
FGF3	FGF3;HBGF-3;INT2	fibroblast growth factor 3	1.1	0.9	0.85	0.7
GCLM	GCLM;GLCLR	glutamate-cysteine ligase, modifier subunit	1.06	0.86	0.85	0.62
GRPEL2	GRPEL2;DKFZp451C205;FLJ23713;FLJ33918;Mt-GrpE#2	GrpE-like 2, mitochondrial (E. coli)	1.06	0.86	0.85	0.66
CLPB	CLPB;FLJ13152;HSP78;SKD3	ClpB caseinolytic peptidase B homolog (E. coli)	1.05	0.83	0.85	0.66
ANKRA2	ANKRA2;ANKRA	ankyrin repeat, family A (RFXANK-like), 2	1.04	0.84	0.85	0.71
GALNTL2	GALNTL2;DKFZp686H1113;GALNT13;GALNT15;GALNT7;PIH5	UDP-N-acetyl-alpha-D-galactosamine:polypeptide N-acetylgalactosaminyltransferase-like 2	1.03	0.82	0.85	0.65
ADCYAP1R1	ADCYAP1R1;PAC1;PAC1R;PACAPR;PACAPRI	adenylate cyclase activating polypeptide 1 (pituitary) receptor type I	1.02	0.8	0.85	0.6
TMPRSS5	TMPRSS5;MGC141886;MGC148044;SPINESIN	transmembrane protease, serine 5	0.99	0.77	0.85	0.58
AZGP1	AZGP1;ZA2G;ZAG	alpha-2-glycoprotein 1, zinc-binding	0.91	0.7	0.85	0.56
FOLH1	FOLH1;FGCP;FOLH;GCP2;GCPII;NAALAD1;NAALAdase;PSM;PSMA;mGCP	folate hydrolase (prostate-specific membrane antigen) 1	0.9	0.7	0.85	0.52
REG4	REG4;GISP;REG-IV;RELP	regenerating islet-derived family, member 4	1.23	1.02	0.86	0.83
TBPL1	TBPL1;MGC:8389;MGC:9620;STUD;TLF;TLP;TRF2	TBP-like 1	1.19	0.99	0.86	0.86
PCGF1	PCGF1;2010002K04Rik;FLJ43754;MGC10882;NSPC1;RNF3A-2;RNF68	polycomb group ring finger 1	1.17	0.96	0.86	0.69
TSG101	TSG101;TSG10;VPS23	tumor susceptibility gene 101	1.17	0.93	0.86	0.69
NAT1	NAT1;AAC1;MNAT;NAT-1;NATI	N-acetyltransferase 1 (arylamine N-acetyltransferase)	1.15	0.95	0.86	0.68
NINL	NINL;FLJ11792;KIAA0980;NLP;dJ691N24.1	ninein-like	1.14	0.93	0.86	0.75
CLEC2L	CLEC2L;FLJ32986	C-type lectin domain family 2, member L	1.1	0.86	0.86	0.71
SLC26A4	SLC26A4;DFNB4;EVA;PDS;TDH2B	solute carrier family 26, member 4	1.08	0.87	0.86	0.77
NXT1	NXT1;MTR2;P15	NTF2-like export factor 1	1.05	0.84	0.86	0.74
CUL2	CUL2;MGC131970	cullin 2	1.05	0.84	0.86	0.63
SCRN1	SCRN1;KIAA0193;SES1	secernin 1	1.04	0.82	0.86	0.62
LRFN4	LRFN4;FIGLER6;MGC3103;SALM3;SALM3.	leucine rich repeat and fibronectin type III domain containing 4	1	0.77	0.86	0.62
IRS4	IRS4;IRS-4;PY160	insulin receptor substrate 4	0.99	0.76	0.86	0.64
ATP5E	ATP5E;ATPE;MC5DN3;MGC104243	ATP synthase, H+ transporting, mitochondrial F1 complex, epsilon subunit	0.99	0.79	0.86	0.7
TNKS	TNKS;PARP-5a;PARP5A;PARPL;TIN1;TINF1;TNKS1;pART5	tankyrase, TRF1-interacting ankyrin-related ADP-ribose polymerase	0.98	0.77	0.86	0.68
IL31	IL31;IL-31	interleukin 31	0.95	0.73	0.86	0.61
MAEA	MAEA;EMLP;EMP;HLC-10;PIG5	macrophage erythroblast attacher	0.86	0.66	0.86	0.53
ZNF17	ZNF17;FLJ40864;FLJ46058;FLJ46615;HPF3;KIAA1947;KOX10	zinc finger protein 17	1.36	1.15	0.87	0.87
SHC2	SHC2;SCK;SHCB;SLI	SHC (Src homology 2 domain containing) transforming protein 2	1.21	1	0.87	0.77
RNF139	RNF139;HRCA1;MGC31961;RCA1;TRC8	ring finger protein 139	1.19	0.98	0.87	0.71
F2RL2	F2RL2;PAR3	coagulation factor II (thrombin) receptor-like 2	1.17	0.96	0.87	0.72
SLC15A4	SLC15A4;PHT1;PTR4	solute carrier family 15, member 4	1.17	0.97	0.87	0.7
ADAMTS6	ADAMTS6;ADAM-TS 6;ADAM-TS6;ADAMTS-6	ADAM metallopeptidase with thrombospondin type 1 motif, 6	1.1	0.88	0.87	0.66

KRTAP22-1	KRTAP22-1;KAP22.1;MGC126731;MGC126735	keratin associated protein 22-1	1.05	0.82	0.87	0.65
PLXNB3	PLXNB3;FLJ76953;PLEXB3;PLEXR;PLXN6	plexin B3	1.05	0.82	0.87	0.69
C20orf112	C20orf112;C20orf113;DKFZp566G1424;dJ1184F4.2;dJ1184F4.4	chromosome 20 open reading frame 112	1.02	0.8	0.87	0.66
TASP1	TASP1;C20orf13;FLJ20212;MGC39159;dJ585I14.2	taspase, threonine aspartase, 1	0.98	0.78	0.87	0.59
PHF15	PHF15;JADE2;KIAA0239	PHD finger protein 15	1.32	1.12	0.88	0.81
ADAM19	ADAM19;MADDAM;MLTNB	ADAM metallopeptidase domain 19	1.29	1.06	0.88	0.79
C6orf203	C6orf203;FLJ34245;PRED31;RP11-59I9.1	chromosome 6 open reading frame 203	1.21	1.01	0.88	0.85
P2RY11	P2RY11;P2Y11	purinergic receptor P2Y, G-protein coupled, 11	1.21	0.99	0.88	0.74
HECA	HECA;HDC;HDCL;HHDC;dJ225E12.1	headcase homolog (Drosophila)	1.15	0.92	0.88	0.73
VAV3	VAV3;FLJ40431	vav 3 guanine nucleotide exchange factor	1.14	0.93	0.88	0.74
PPP6C	PPP6C;FLJ92648;MGC12249;PP6;PP6C	protein phosphatase 6, catalytic subunit	1.13	0.92	0.88	0.7
FBLN7	FBLN7;DKFZp547D0610;FLJ37440;TM14	fibulin 7	1.1	0.87	0.88	0.73
TMPPE	TMPPE;FLJ45032;FLJ46376	transmembrane protein with metallophosphoesterase domain	1.05	0.83	0.88	0.69
FAM110C	FAM110C	family with sequence similarity 110, member C	1.01	0.8	0.88	0.66
C5orf43	C5orf43;DKFZp686E2158;DKFZp686O1385	chromosome 5 open reading frame 43	0.99	0.78	0.88	0.65
NFKBIZ	NFKBIZ;FLJ30225;FLJ34463;IKBZ;INAP;MAIL	nuclear factor of kappa light polypeptide gene enhancer in B-cells inhibitor, zeta	0.98	0.77	0.88	0.65
ROBO4	ROBO4;ECSM4;FLJ20798;MGC133352;MGC133353;MRB	roundabout homolog 4, magic roundabout (Drosophila)	0.95	0.75	0.88	0.63
TLCD1	TLCD1	TLC domain containing 1	0.94	0.72	0.88	0.57
PRIMA1	PRIMA1;PRIMA	proline rich membrane anchor 1	1.21	0.97	0.89	0.77
S1PR2	S1PR2;AGR16;EDG-5;EDG5;Gpcr13;H218;LPB2;S1P2	sphingosine-1-phosphate receptor 2	1.21	1.01	0.89	0.76
UBE2A	UBE2A;HHR6A;MRXS30;MRXSN;RAD6A;UBC2	ubiquitin-conjugating enzyme E2A	1.2	0.99	0.89	0.74
TAB2	TAB2;CHTD2;FLJ21885;KIAA0733;MAP3K7IP2	TGF-beta activated kinase 1/MAP3K7 binding protein 2	1.17	0.94	0.89	0.78
SFT2D3	SFT2D3;MGC5391	SFT2 domain containing 3	1.12	0.89	0.89	0.69
UBA1	UBA1;A1S9;A1S9T;A1ST;AMCX1;GXP1;MGC4781;POC20;SMAX2;UBA1A;UBE1;UBE1X	ubiquitin-like modifier activating enzyme 1	1.1	0.9	0.89	0.68
MB	MB;MGC13548;PVALB	myoglobin	1.02	0.82	0.89	0.65
C1orf222	C1orf222;MGC133181	chromosome 1 open reading frame 222	1.01	0.8	0.89	0.67
ATXN7L2	ATXN7L2;FLJ00381;MGC46534	ataxin 7-like 2	0.96	0.76	0.89	0.6
GRB7	GRB7	growth factor receptor-bound protein 7	1.19	0.96	0.9	0.8
SGPP2	SGPP2;FLJ39004;SPP2	sphingosine-1-phosphate phosphatase 2	1.17	0.97	0.9	0.73
ARF4	ARF4;ARF2	ADP-ribosylation factor 4	1.12	0.92	0.9	0.82
SLAMF8	SLAMF8;BLAME;CD353;FLJ20442;MGC129578;SBBI42	SLAM family member 8	1.15	0.94	0.91	0.75
UBE2G1	UBE2G1;E217K;UBC7;UBE2G	ubiquitin-conjugating enzyme E2G 1	1.29	1.08	0.92	0.81
PP2D1	PP2D1;C3orf48;FLJ25449	protein phosphatase 2C-like domain containing 1	1.29	1.08	0.92	0.82
TMEM102	TMEM102;CBAP;FLJ36878	transmembrane protein 102	1.13	0.93	0.92	0.72
CAPRIN2	CAPRIN2;C1QDC1;EEG-1;EEG1;FLJ11391;FLJ22569;KIAA1873;MGC102894;MGC134847;MGC134848;RNG140	caprin family member 2	0.84	0.61	0.81	0.48
ASTN2	ASTN2;KIAA0634;bA67K19.1	astrotactin 2	0.8	0.58	0.81	0.48
NAA50	NAA50;FLJ13194;MAK3;NAT13;NAT5;SAN;hNAT5;hSAN	N(alpha)-acetyltransferase 50, NatE catalytic subunit	0.8	0.6	0.81	0.54
CD109	CD109;CPAMD7;DKFZp762L1111;FLJ38569;FLJ41966;RP11-525G3.1	CD109 molecule	0.77	0.56	0.82	0.47
ADIG	ADIG;KIAA1219;MGC149650;MGC39724;SMAF1	adipogenin	0.76	0.56	0.83	0.44
FAM111B	FAM111B;CANP	family with sequence similarity 111, member B	0.76	0.56	0.83	0.44
LILRA6	LILRA6;CD85b;ILT5;ILT8;LILRB3;LILRB6	leukocyte immunoglobulin-like receptor, subfamily A (with TM domain), member 6	0.97	1.4	1.62	1.09
HDAC8	HDAC8;HD8;HDACL1;RPD3	histone deacetylase 8	0.88	1.31	1.62	1.16
MLPH	MLPH;MGC2771;MGC59733;SLAC2-A	melanophilin	0.86	1.34	1.62	0.96
BCKDHB	BCKDHB;E1B;FLJ17880;dJ279A18.1	branched chain keto acid dehydrogenase E1, beta polypeptide	0.86	1.34	1.63	1.05
AUP1	AUP1	ancient ubiquitous protein 1	0.9	1.45	1.65	1.09
SAMD5	SAMD5;dJ875H10.1	sterile alpha motif domain containing 5	0.88	1.28	1.65	1
ZNF790	ZNF790;FLJ20350;MGC62100	zinc finger protein 790	0.85	1.24	1.65	0.97
PHKB	PHKB;DKFZp781E15103;FLJ41698	phosphorylase kinase, beta	0.95	1.36	1.44	1.12
DNAJC28	DNAJC28;C21orf55;C21orf78	DnaJ (Hsp40) homolog, subfamily C, member 28	0.88	1.21	1.44	0.87
PLK2	PLK2;SNK	polo-like kinase 2	0.91	1.31	1.45	1.08
GAS2L3	GAS2L3;G2L3	growth arrest-specific 2 like 3	0.9	1.25	1.45	0.96
SYNE2	SYNE2;DKFZp434H2235;DKFZp686E01115;DKFZp686H1931;EDMD5;FLJ11014;FLJ43727;FLJ45710;FLJ46790;KIAA1011;NUA;NUANCE;Nesprin-2;SYNE-2;TROPH	spectrin repeat containing, nuclear envelope 2	0.86	1.12	1.45	0.89
INTU	INTU;FLJ41326;INT;KIAA1284;PDZD6;PDZK6	inturned planar cell polarity effector homolog (Drosophila)	1.01	1.31	1.46	1.03
MRPL39	MRPL39;C21orf92;FLJ20451;L39mt;MGC104174;MGC3400;MRP-L5;MRPL5;PRED22;PRED66;RPML5	mitochondrial ribosomal protein L39	1.02	1.5	1.47	1.24
FRY	FRY;13CDNA73;214K23.2;C13orf14;CG003;bA207N4.2;bA37E23.1	furry homolog (Drosophila)	0.89	1.17	1.47	0.91
SMOC2	SMOC2;MST117;MSTP117;MSTP140;SMAP2;bA270C4A.1;bA37D8.1;dJ421D16.1	SPARC related modular calcium binding 2	0.86	1.15	1.47	0.91
FKSG83	FKSG83;MGC129573	FKSG83	0.94	1.34	1.49	1.21
MRPS2	MRPS2;MRP-S2;S2mt	mitochondrial ribosomal protein S2	0.94	1.38	1.5	1.04
PTPRM	PTPRM;MGC166994;PTPRL1;R-PTP-MU;RPTPM;RPTPU;hR-PTPu	protein tyrosine phosphatase, receptor type, M	0.88	1.34	1.51	1.04
NAPSA	NAPSA;KAP;Kdap;NAP1;NAPA;SNAPA	napsin A aspartic peptidase	0.88	1.33	1.54	0.99
PPFIA3	PPFIA3;KIAA0654;LPNA3;MGC126567;MGC126569	protein tyrosine phosphatase, receptor type, f polypeptide (PTPRF), interacting protein (liprin), alpha 3	1.05	1.5	1.55	1.13
SCARF2	SCARF2;NSR1;SREC-II;SREC2;SRECRP-1;VDEGS	scavenger receptor class F, member 2	0.94	1.42	1.6	1.12
FGF19	FGF19	fibroblast growth factor 19	1.02	1.43	1.26	0.99
MEN1	MEN1;MEAI;MENIN;SCG2	multiple endocrine neoplasia I	0.94	1.2	1.26	0.93
BAGE	BAGE;BAGE1;CT2.1	B melanoma antigen	0.9	1.17	1.26	0.89
TBL1XR1	TBL1XR1;C21;DC42;FLJ12894;IRA1;TBLR1	transducin (beta)-like 1 X-linked receptor 1	0.85	1.11	1.26	0.74
KRTAP5-2	KRTAP5-2;KRTAP5-8;KRTAP5.2	keratin associated protein 5-2	1.15	1.46	1.27	0.99
RNF43	RNF43;DKFZp781H02126;DKFZp781H0392;FLJ20315;FLJ77466;FLJ99338;MGC125630;RNF124;URCC	ring finger protein 43	1.08	1.35	1.27	1.21
SUCLA2	SUCLA2;A-BETA;MTDPS5;SCS-betaA	succinate-CoA ligase, ADP-forming, beta subunit	0.98	1.38	1.27	0.95
FAM72A	FAM72A	ENTREZ STATUS: REMAPPED TO GENE ID: 728833	0.92	1.19	1.27	0.83
FAM188B	FAM188B;C7orf67;FLJ22374;FLJ51453;MGC44277	family with sequence similarity 188, member B	0.9	1.2	1.27	0.84
SLC9A5	SLC9A5;NHE5	solute carrier family 9 (sodium/hydrogen exchanger), member 5	0.89	1.21	1.27	0.8
ZNF260	ZNF260;OZRF1;PEX1;ZFP260	zinc finger protein 260	0.87	1.21	1.27	0.8
TRIM46	TRIM46;FLJ23229;GENEY;TRIFIC	tripartite motif containing 46	0.98	1.25	1.28	1.12
CEACAM19	CEACAM19;CEAL1;MGC105097	carcinoembryonic antigen-related cell adhesion molecule 19	0.92	1.23	1.28	0.88
IFI35	IFI35;FLJ21753;IFP35	interferon-induced protein 35	0.9	1.19	1.28	0.81
ITGB7	ITGB7	integrin, beta 7	0.85	1.15	1.28	0.76
PIGQ	PIGQ;FLJ56783;GPI1;MGC12693;c407A10.1	phosphatidylinositol glycan anchor biosynthesis, class Q	1.03	1.29	1.29	1.08
PLIN1	PLIN1;FPLD4;PERI;PLIN	perilipin 1	1.01	1.27	1.29	0.96
MAPK6	MAPK6;DKFZp686F03189;ERK3;HsT17250;PRKM6;p97MAPK	mitogen-activated protein kinase 6	0.94	1.3	1.29	0.93
MRAS	MRAS;FLJ42964;M-RAs;R-RAS3;RRAS3	muscle RAS oncogene homolog	0.94	1.28	1.29	0.84
NUP155	NUP155;KIAA0791;N155	nucleoporin 155kDa	0.94	1.23	1.29	0.94
OR4D10	OR4D10;OR11-251;OR4D10P;OST711	olfactory receptor, family 4, subfamily D, member 10	0.93	1.25	1.29	0.84
DYRK4	DYRK4	dual-specificity tyrosine-(Y)-phosphorylation regulated kinase 4	0.92	1.19	1.29	0.99
METTL20	METTL20;C12orf72;MGC50559	methyltransferase like 20	0.9	1.19	1.29	0.94
SLC22A9	SLC22A9;FLJ23666;HOAT4;OAT4;OAT7;UST3H;ust3	solute carrier family 22 (organic anion transporter), member 9	0.94	1.25	1.3	0.99
NANP	NANP;C20orf147;HDHD4;MGC26833;dJ694B14.3	N-acetylneuraminic acid phosphatase	0.92	1.23	1.3	0.97
FAM82A1	FAM82A1;FAM82A;FLJ32954;FLJ38143;MGC33318;PRO34163;PYST9371;RMD-2;RMD2;RMD4	family with sequence similarity 82, member A1	0.86	1.19	1.3	0.91
ORC4	ORC4;FLJ46668;ORC4L;ORC4P	origin recognition complex, subunit 4	1.07	1.42	1.31	0.98
ZIK1	ZIK1;MGC119699;MGC119700;MGC119701;ZNF762	zinc finger protein interacting with K protein 1 homolog (mouse)	1.04	1.32	1.31	1.01
AASDH	AASDH;ACSF4;LYS2;NRPS1098;NRPS998	aminoadipate-semialdehyde dehydrogenase	0.98	1.29	1.31	0.87
TMEM208	TMEM208	transmembrane protein 208	0.98	1.33	1.31	0.91
OPN1MW2	OPN1MW2;GOP;OPN1MW	opsin 1 (cone pigments), medium-wave-sensitive 2	0.94	1.3	1.31	0.91
POTEB	POTEB;A26B1;CT104.5;MGC119371;MGC119373;POTE-15;POTE15	POTE ankyrin domain family, member B	0.9	1.21	1.31	0.81
EPC1	EPC1;DKFZp781P2312;Epl1	enhancer of polycomb homolog 1 (Drosophila)	0.88	1.16	1.31	0.9
GPC5	GPC5	glypican 5	0.94	1.25	1.32	0.85
DDX50	DDX50;GU2;GUB;MGC3199;RH-II/GuB	DEAD (Asp-Glu-Ala-Asp) box polypeptide 50	0.93	1.19	1.32	0.88
PIGP	PIGP;DCRC;DCRC-S;DSCR5;DSRC	phosphatidylinositol glycan anchor biosynthesis, class P	0.92	1.21	1.32	0.87
CACNG2	CACNG2;FLJ41437;MGC138502;MGC138504;MRD10	calcium channel, voltage-dependent, gamma subunit 2	0.91	1.27	1.32	0.99
USP10	USP10;KIAA0190;MGC2621;UBPO	ubiquitin specific peptidase 10	0.89	1.16	1.32	0.87
TBR1	TBR1;MGC141978;TBR-1;TES-56	T-box, brain, 1	0.88	1.15	1.32	0.8
NKRF	NKRF;ITBA4;NRF	NFKB repressing factor	0.86	1.18	1.32	0.88
FBLN5	FBLN5;ARMD3;DANCE;EVEC;FIBL-5;FLJ90059;UP50	fibulin 5	0.85	1.16	1.32	0.88
PTP4A1	PTP4A1;DKFZp779M0721;HH72;PRL-1;PRL1;PTP(CAAX1);PTPCAAX1	protein tyrosine phosphatase type IVA, member 1	1.13	1.48	1.33	1.16
PTPN9	PTPN9;MEG2;PTPMEG2	protein tyrosine phosphatase, non-receptor type 9	0.99	1.31	1.33	1.02
TRIM43B	TRIM43B	tripartite motif containing 43B	0.98	1.28	1.33	1.15
F5	F5;FVL;PCCF	coagulation factor V (proaccelerin, labile factor)	0.94	1.21	1.33	0.85
GDAP2	GDAP2;FLJ20142;MACROD3;dJ776P7.1	ganglioside induced differentiation associated protein 2	1	1.31	1.34	0.99
TSSC1	TSSC1	tumor suppressing subtransferable candidate 1	0.9	1.21	1.34	0.82
NPIPL1	NPIPL1;A-575C2.4;A-761H5.5	nuclear pore complex interacting protein-like 1	0.87	1.13	1.34	0.86
CXorf38	CXorf38;MGC39350	chromosome X open reading frame 38	0.85	1.19	1.34	0.85
CCDC22	CCDC22;CXorf37	coiled-coil domain containing 22	1.02	1.32	1.35	1.01
PTPRK	PTPRK;DKFZp686C2268;DKFZp779N1045;R-PTP-kappa	protein tyrosine phosphatase, receptor type, K	0.91	1.23	1.35	0.95
ACTR5	ACTR5;Arp5;FLJ12785;INO80M	ARP5 actin-related protein 5 homolog (yeast)	0.9	1.25	1.35	0.85
GPD2	GPD2;GDH2;GPDM;mGPDH	glycerol-3-phosphate dehydrogenase 2 (mitochondrial)	0.9	1.23	1.35	0.95
GRAP	GRAP;MGC64880	GRB2-related adaptor protein	0.88	1.17	1.35	0.97
DDC	DDC;AADC	dopa decarboxylase (aromatic L-amino acid decarboxylase)	0.86	1.14	1.35	0.8
SCRG1	SCRG1;MGC26468;SCRG-1	stimulator of chondrogenesis 1	1.04	1.36	1.36	1.07
SPATA5L1	SPATA5L1;FLJ12286;MGC5347	spermatogenesis associated 5-like 1	1.04	1.32	1.36	1.17
CCDC70	CCDC70;DKFZp434K1172;FLJ25853	coiled-coil domain containing 70	0.94	1.24	1.36	0.93
BDH1	BDH1;BDH;MGC2723;MGC4347;MGC9788;SDR9C1	3-hydroxybutyrate dehydrogenase, type 1	0.92	1.26	1.36	0.98
HHAT	HHAT;FLJ10724;FLJ34867;MART2;SKI1;Skn	hedgehog acyltransferase	0.88	1.18	1.37	0.83
TARP	TARP;CD3G;TCRG;TCRGC1;TCRGC2	TCR gamma alternate reading frame protein	0.88	1.23	1.37	0.94
NT5E	NT5E;CD73;E5NT;NT;NT5;NTE;eN;eNT	5'-nucleotidase, ecto (CD73)	1.05	1.34	1.38	1.01
MTMR6	MTMR6	myotubularin related protein 6	1.01	1.29	1.38	0.97
CCR7	CCR7;BLR2;CD197;CDw197;CMKBR7;EBI1	chemokine (C-C motif) receptor 7	1	1.27	1.38	0.96
GOLT1A	GOLT1A;CGI-141;FLJ42654;GOT1;HMFN1187;MGC62027;RP11-203F10.3;YMR292W	golgi transport 1A	0.94	1.24	1.38	0.95
CNGA3	CNGA3;ACHM2;CCNC1;CCNCa;CCNCalpha;CNCG3;CNG3	cyclic nucleotide gated channel alpha 3	0.9	1.24	1.38	0.96
SHANK1	SHANK1;SPANK-1;SSTRIP;synamon	SH3 and multiple ankyrin repeat domains 1	0.9	1.29	1.38	0.91

RNF31	RNF31;FLJ10111;FLJ23501;HOIP;MGC19975;ZIBRA	ring finger protein 31	0.88	1.16	1.38	0.83
RBM5	RBM5;FLJ39876;G15;LUCA15;RMB5	RNA binding motif protein 5	0.86	1.23	1.38	0.84
SCRIB	SCRIB;CRIB1;SCRB1;SCRIB1;Vartul	scribbled homolog (Drosophila)	1.07	1.46	1.4	1.1
CLN3	CLN3;BTS;JNCL;MGC102840	ceroid-lipofuscinosis, neuronal 3	0.94	1.31	1.4	0.89
FBXO33	FBXO33;BMND12;Fbx33;c14_5247	F-box protein 33	0.92	1.23	1.4	0.88
DTX3	DTX3;FLJ34766;MGC138863;MGC138864;RNF154	deltex homolog 3 (Drosophila)	0.91	1.27	1.4	1.13
SDC3	SDC3;SDCN;SYND3	syndecan 3	0.85	1.15	1.4	0.9
ZNF326	ZNF326;FLJ20403;MGC61591;ZAN75;Zfp326;dJ871E2.1	zinc finger protein 326	0.98	1.44	1.41	1.03
PDE6D	PDE6D;PDED	phosphodiesterase 6D, cGMP-specific, rod, delta	0.97	1.38	1.41	1.06
HIST2H2AC	HIST2H2AC;H2A;H2A-GL101;H2A/q;H2AFQ;MGC74460	histone cluster 2, H2ac	0.97	1.34	1.41	1.12
PRRC1	PRRC1;FLJ32875	proline-rich coiled-coil 1	0.9	1.23	1.41	0.92
PDE6A	PDE6A;CGPR-A;PDEA;RP43	phosphodiesterase 6A, cGMP-specific, rod, alpha	0.89	1.19	1.41	0.93
CFH	CFH;AHUS1;AMBP1;ARMD4;ARMS1;CFHL3;FH;FHL1;HF;HF1;HF2;HUS;MGC88246	complement factor H	0.86	1.17	1.41	0.84
CDC42BPG	CDC42BPG;DMPK2;HSMDPKIN;KAPPA-200;MRCKG;MRCKgamma	CDC42 binding protein kinase gamma (DMPK-like)	0.85	1.2	1.41	0.99
SRPX	SRPX;DRS;ETX1;SRPX1	sushi-repeat containing protein, X-linked	0.88	1.21	1.42	0.95
DUSP3	DUSP3;VHR	dual specificity phosphatase 3	0.99	1.31	1.43	0.99
EPC2	EPC2;DKFZp566F2124;EPC-LIKE	enhancer of polycomb homolog 2 (Drosophila)	0.94	1.31	1.43	1
PNOC	PNOC;PPNOC	prepronociceptin	0.86	1.21	1.43	0.91
GLS2	GLS2;GA;GLS;LGA;MGC71567;hLGA	glutaminase 2 (liver, mitochondrial)	1.04	1.29	1.26	0.91
GSDMD	GSDMD;DF5L;DFNA5L;FLJ12150;GSDMDC1	gasdermin D	0.96	1.21	1.26	0.83
LRRN4	LRRN4;C20orf75;FLJ23994;MGC25027;NLRR4;dJ1056H1.1	leucine rich repeat neuronal 4	1.19	1.44	1.27	1.05
F13A1	F13A1;F13A	coagulation factor XIII, A1 polypeptide	0.96	1.21	1.28	0.9
STAP2	STAP2;BKS;FLJ20234	signal transducing adaptor family member 2	0.98	1.23	1.3	0.94
HRH1	HRH1;H1-R;hisH1	histamine receptor H1	0.94	1.19	1.3	1.07
PYROXD1	PYROXD1;FLJ22028	pyridine nucleotide-disulphide oxidoreductase domain 1	0.88	1.13	1.3	0.94
VCPIP1	VCPIP1;DKFZp686G038;DUBA3;FLJ23132;FLJ60694;KIAA1850;VCIP135	valosin containing protein (p97)/p47 complex interacting protein 1	0.98	1.23	1.31	0.89
ADCY3	ADCY3;AC3;KIAA0511	adenylate cyclase 3	0.92	1.17	1.31	0.84
ELOVL5	ELOVL5;HELO1;dJ483K16.1	ELOVL fatty acid elongase 5	0.88	1.13	1.31	0.79
BZW2	BZW2;MST017;MSTP017	basic leucine zipper and W2 domains 2	0.9	1.15	1.34	0.9
HTR4	HTR4;5-HT4;5-HT4R	5-hydroxytryptamine (serotonin) receptor 4	0.96	1.21	1.35	0.91
CALCOCO2	CALCOCO2;MGC17318;NDP52	calcium binding and coiled-coil domain 2	0.86	1.11	1.35	0.8
FAM47C	FAM47C	family with sequence similarity 47, member C	0.9	1.15	1.36	0.93
CENPB	CENPB	centromere protein B, 80kDa	0.94	1.19	1.37	1.05
ADAL	ADAL;DKFZp313B2137;FLJ44620	adenosine deaminase-like	1.06	1.36	1.25	1.02
CACNG3	CACNG3	calcium channel, voltage-dependent, gamma subunit 3	1.04	1.34	1.25	1.04
ZFP41	ZFP41;DKFZp761O1618;FLJ00028;FLJ38705;ZNF753	zinc finger protein 41 homolog (mouse)	0.98	1.3	1.25	0.92
PRAMEF4	PRAMEF4;RP5-845O24.6	PRAME family member 4	0.89	1.18	1.25	0.78
MORC3	MORC3;NXP2;ZCW5;ZCWCC3	MORC family CW-type zinc finger 3	0.89	1.21	1.25	0.81
ARID2	ARID2;BAF200;DKFZp686G052;DKFZp779P0222;FLJ30619;KIAA1557;p200	AT rich interactive domain 2 (ARID, RFX-like)	0.88	1.14	1.25	0.86
SLC35G1	SLC35G1;C10orf60;FLJ33990;TMEM20	solute carrier family 35, member G1	0.88	1.15	1.25	0.77
NOC3L	NOC3L;AD24;C10orf117;FAD24;FLJ12820	nucleolar complex associated 3 homolog (S. cerevisiae)	0.86	1.15	1.25	0.88
GBP2	GBP2;DKFZp451C2311	guanylate binding protein 2, interferon-inducible	1.17	1.42	1.25	1.02
GUF1	GUF1;EF-4;FLJ13220	GUF1 GTPase homolog (S. cerevisiae)	0.67	1.13	1.62	0.81
RGPD5	RGPD5;BS-63;BS63;DKFZp686I1842;RGP5;RGPD6	RANBP2-like and GRIP domain containing 5	0.62	0.99	1.62	0.75
FKBP5	FKBP5;AIG6;FKBP51;FKBP54;MGC111006;P54;PPIase;Ptg-10	FK506 binding protein 5	0.68	1.07	1.64	0.76
SSH1	SSH1;FLJ21928;FLJ38102;KIAA1298;SSH1L	slingshot homolog 1 (Drosophila)	0.74	1.12	1.65	0.84
OR6B1	OR6B1;OR7-3;OR7-9	olfactory receptor, family 6, subfamily B, member 1	0.66	1.07	1.65	0.81
NRP1	NRP1;BDCA4;CD304;DKFZp686A03134;DKFZp781F1414;NP1;NRP;VEGF165R	neuropilin 1	0.62	0.96	1.65	0.76
HSDL1	HSDL1;MGC125994;MGC125995;MGC126032;SDR12C3	hydroxysteroid dehydrogenase like 1	0.77	1.29	1.66	1
ATAD5	ATAD5;C17orf41;ELG1;FLJ12735;FRAG1	ATPase family, AAA domain containing 5	0.72	1.07	1.66	0.84
TREM1	TREM1;CD354;TREM-1	triggering receptor expressed on myeloid cells 1	0.74	1.19	1.7	0.94
GSTM2	GSTM2;GST4;GSTM;GSTM2-2;GTHMUS;MGC117303	glutathione S-transferase mu 2 (muscle)	0.7	1.12	1.7	0.88
CLVS1	CLVS1;CRALBPL;FLJ37248;MGC34646;RLBP1L1	clavesin 1	0.66	1.12	1.71	0.84
LAIR1	LAIR1;CD305;LAIR-1	leukocyte-associated immunoglobulin-like receptor 1	0.68	1.26	1.74	0.83
TTPAL	TTPAL;C20orf121;DKFZp686E0870;MGC2470	tocopherol (alpha) transfer protein-like	0.66	1.05	1.76	0.84
TUBB2B	TUBB2B;DKFZp566F223;FLJ98847;MGC8685;bA506K6.1	tubulin, beta 2B class IIb	0.62	1	1.81	0.79
TRAPPC10	TRAPPC10;EHOC-1;EHOC1;FLJ54223;FLJ54817;FLJ55683;GT334;MGC126777;TMEM1;TRS130;TRS30	trafficking protein particle complex 10	0.7	1.36	1.85	1.01
CRYAB	CRYAB;CRYA2;CTPP2;HSPB5	crystallin, alpha B	0.77	1.5	2.01	1.14
HTATIP2	HTATIP2;CC3;FLJ26963;SDR44U1;TIP30	HIV-1 Tat interactive protein 2, 30kDa	0.62	1.23	2.09	0.97
CFTR	CFTR;ABC35;ABCC7;CF;CFTR/MRP;MRP7;TNR-CFTR;dJ760C5.1	cystic fibrosis transmembrane conductance regulator (ATP-binding cassette sub-family C, member 7)	0.84	1.23	1.44	0.95
FDFT1	FDFT1;DGPT;ERG9;SQS;SS	farnesyl-diphosphate farnesyltransferase 1	0.68	0.98	1.44	0.66
CDON	CDON;CDO;HPE11;MGC111524;ORCAM	Cdon homolog (mouse)	0.62	0.92	1.44	0.67
CCDC86	CCDC86;FLJ22321;MGC2574	coiled-coil domain containing 86	0.61	0.9	1.44	0.67
DHPS	DHPS;DHS;DS;MIG13	deoxyhypusine synthase	0.83	1.23	1.45	0.98
MRTO4	MRTO4;C1orf33;MRT4;dJ657E11.4	mRNA turnover 4 homolog (S. cerevisiae)	0.82	1.24	1.45	0.89
LINC00336	LINC00336;C6orf227;FLJ43752;NCRNA00336	long intergenic non-protein coding RNA 336	0.78	1.12	1.45	0.84
CCDC135	CCDC135;C16orf50;DKFZp434I099	coiled-coil domain containing 135	0.69	1.06	1.45	0.82
ATG2A	ATG2A;KIAA0404;MGC117153	ATG2 autophagy related 2 homolog A (S. cerevisiae)	0.64	0.98	1.45	0.69
C12orf53	C12orf53;DKFZp547D2210;PANP	chromosome 12 open reading frame 53	0.62	0.94	1.45	0.67
COPS6	COPS6;CSN6;MOV34-34KD	COP9 constitutive photomorphogenic homolog subunit 6 (Arabidopsis)	0.62	0.88	1.45	0.66
RAPGEFL1	RAPGEFL1;Link-GEFII;MGC134798;MGC134799	Rap guanine nucleotide exchange factor (GEF)-like 1	0.82	1.23	1.46	0.84
FZD10	FZD10;CD350;FZ-10;Fz10;FzE7;hFz10	frizzled family receptor 10	0.8	1.14	1.46	1.02
HRASLS5	HRASLS5;HRLP5;RLP1;iNAT	HRAS-like suppressor family, member 5	0.69	0.98	1.46	0.74
ERI1	ERI1;3'HEXO;HEXO;MGC35395;THEX1	exoribonuclease 1	0.82	1.19	1.47	0.83
CAPZA2	CAPZA2;CAPPA2;CAPZ	capping protein (actin filament) muscle Z-line, alpha 2	0.8	1.21	1.47	0.83
ZNF140	ZNF140;pHZ-39	zinc finger protein 140	0.78	1.13	1.47	0.89
TMEM229B	TMEM229B;C14orf83;FLJ33387	transmembrane protein 229B	0.74	1.02	1.47	0.83
NUDT3	NUDT3;DIPP;DIPP-1;DIPP1	nudix (nucleoside diphosphate linked moiety X)-type motif 3	0.72	0.98	1.47	0.74
LITAF	LITAF;FLJ38636;MGC116698;MGC116700;MGC116701;MGC125274;MGC125275;MGC125276;PIG7;SIMPLE;TP53I7	lipopolysaccharide-induced TNF factor	0.71	1.02	1.47	0.72
MBIP	MBIP	MAP3K12 binding inhibitory protein 1	0.67	1.01	1.47	0.77
GPLD1	GPLD1;GPIPLD;GPIPLDM;MGC22590;PIGPLD;PIGPLD1	glycosylphosphatidylinositol specific phospholipase D1	0.66	0.99	1.47	0.78
ELMOD3	ELMOD3;FLJ21977;FLJ35601;LST3;MGC111036;RBED1;RBM29	ELMO/CED-12 domain containing 3	0.8	1.12	1.48	0.88
CAPN1	CAPN1;CANP;CANP1;CANPL1;muCANP;muCL	calpain 1, (mu/I) large subunit	0.72	0.98	1.48	0.73
RILPL2	RILPL2;FLJ30380;FLJ32372;MGC7036;RLP2	Rab interacting lysosomal protein-like 2	0.7	1.09	1.48	0.73
TMEM85	TMEM85;FLJ90746;MGC24415;PIG17	transmembrane protein 85	0.64	1	1.48	0.7
ANKMY2	ANKMY2;DKFZp564O043;ZMYND20	ankyrin repeat and MYND domain containing 2	0.63	0.97	1.48	0.66
SETX	SETX;ALS4;AOA2;DKFZp781B151;FLJ12840;FLJ43459;KIAA0625;SCAR1;bA479K20.2	senataxin	0.8	1.06	1.49	0.83
FCER1G	FCER1G;FCRG	Fc fragment of IgE, high affinity I, receptor for; gamma polypeptide	0.78	1.12	1.49	0.87
ROCK2	ROCK2;KIAA0619;ROCK-II	Rho-associated, coiled-coil containing protein kinase 2	0.77	1.25	1.49	0.89
C20orf43	C20orf43;CDAO5;HSPC164;SHUJUN-3	chromosome 20 open reading frame 43	0.74	1.06	1.49	0.82
AVPR2	AVPR2;ADHR;DI1;DIR;DIR3;MGC126533;MGC138386;NDI;V2R	arginine vasopressin receptor 2	0.66	0.94	1.49	0.86
BARX2	BARX2;MGC133368;MGC133369	BARX homeobox 2	0.66	0.97	1.49	0.83
SLC5A3	SLC5A3;SMIT;SMIT2	solute carrier family 5 (sodium/myo-inositol cotransporter), member 3	0.66	0.99	1.49	0.77
XKR4	XKR4;KIAA1889;XRG4	XK, Kell blood group complex subunit-related family, member 4	0.62	0.88	1.49	0.68
L1CAM	L1CAM;CAML1;CD171;HSAS;HSAS1;MASA;MIC5;N-CAM-L1;N-CAML1;NCAM-L1;S10;SPG1	L1 cell adhesion molecule	0.62	0.9	1.5	0.68
POU4F2	POU4F2;BRN3.2;BRN3B;Brn-3b	POU class 4 homeobox 2	0.84	1.21	1.51	0.95
ATG16L2	ATG16L2;ATG16B;FLJ00012;WDR80	ATG16 autophagy related 16-like 2 (S. cerevisiae)	0.8	1.08	1.51	0.84
SBNO2	SBNO2;FLJ00173;KIAA0963;SNO;STNO	strawberry notch homolog 2 (Drosophila)	0.78	1.06	1.51	0.83
CREBL2	CREBL2;MGC117311;MGC138362	cAMP responsive element binding protein-like 2	0.62	0.9	1.51	0.71
SLC16A6	SLC16A6;MCT6;MCT7	solute carrier family 16, member 6 (monocarboxylic acid transporter 7)	0.74	1.12	1.52	0.87
THAP9	THAP9;FLJ23320;FLJ34093	THAP domain containing 9	0.68	1	1.52	0.78
CPAMD8	CPAMD8;FLJ42058;FLJ90618;K-CAP;KIAA1283;VIP	C3 and PZP-like, alpha-2-macroglobulin domain containing 8	0.68	1.06	1.52	0.71
SIN3A	SIN3A;DKFZp434K2235;FLJ90319;KIAA0700	SIN3 homolog A, transcription regulator (yeast)	0.63	0.9	1.52	0.79
GYPC	GYPC;CD236;CD236R;GE;GPC;GPD;GYPD;MGC117309;MGC126191;MGC126192;PAS-2;PAS-2'	glycophorin C (Gerbich blood group)	0.82	1.19	1.53	0.93
TRIM29	TRIM29;ATDC;FLJ36085	tripartite motif containing 29	0.81	1.22	1.54	1.1
DMKN	DMKN;FLJ79374;UNQ729;ZD52F10	dermokine	0.8	1.09	1.54	0.85
FREM1	FREM1;BNAR;C9orf143;C9orf145;C9orf154;FLJ25461;MOTA;TILRR	FRAS1 related extracellular matrix 1	0.77	1.16	1.54	0.91
RNASE8	RNASE8	ribonuclease, RNase A family, 8	0.73	1.04	1.54	0.92
UCN2	UCN2;SRP;UCN-II;UCNI;UR;URP	urocortin 2	0.64	1.02	1.54	0.68
TMEM192	TMEM192;FLJ38482	transmembrane protein 192	0.82	1.23	1.55	0.94
MCM8	MCM8;C20orf154;MGC119522;MGC119523;MGC12866;MGC4816;REC;dJ967N21.5	minichromosome maintenance complex component 8	0.79	1.17	1.55	0.91
E4F1	E4F1;E4F;MGC99614	E4F transcription factor 1	0.62	0.91	1.55	0.71
OXCT1	OXCT1;OXCT;SCOT	3-oxoacid CoA transferase 1	0.82	1.26	1.56	0.89
GJB4	GJB4;CX30.3;EKV;MGC21116	gap junction protein, beta 4, 30.3kDa	0.66	0.94	1.56	0.84
FKBP6	FKBP6;FKBP36;MGC87179;PPIase	FK506 binding protein 6, 36kDa	0.62	0.94	1.56	0.69
CPO	CPO;MGC138281;MGC138283	carboxypeptidase O	0.75	1.09	1.57	0.81
PRAC	PRAC;C17orf92;MGC32520	prostate cancer susceptibility candidate	0.69	1.06	1.57	0.8
TLK1	TLK1;KIAA0137;PKU-beta	tousled-like kinase 1	0.66	1.01	1.57	0.91
PTGER1	PTGER1;EP1	prostaglandin E receptor 1 (subtype EP1), 42kDa	0.61	0.9	1.57	0.68
MTHFR	MTHFR	methylenetetrahydrofolate reductase (NAD(P)H)	0.76	1.2	1.58	0.81
PDZD2	PDZD2;AIPC;KIAA0300;PAPIN;PDZK3;PIN1	PDZ domain containing 2	0.76	1.08	1.58	0.84
PRRX1	PRRX1;AGOTC;PHOX1;PMX1;PRX1	paired related homeobox 1	0.74	1.12	1.58	0.88
ZNF341	ZNF341	zinc finger protein 341	0.63	1.01	1.58	0.73
CCRL1	CCRL1;CC-CKR-11;CCBP2;CCR-11;CCR10;CCR11;CCX CKR;CCX-CKR;CKR-11;PPR1;VSHK1	chemokine (C-C motif) receptor-like 1	0.62	0.92	1.58	0.69
CAP2	CAP2	CAP, adenylate cyclase-associated protein, 2 (yeast)	0.8	1.25	1.59	0.94
GFRA1	GFRA1;DKFZp313E1012;DKFZp686J0156;FLJ10561;FLJ31546;GDNFR;GDNFRA;GFR-ALPHA-1;MGC23045;RET1L;RETL1;TRNR1	GDNF family receptor alpha 1	0.79	1.19	1.59	0.94
PLCXD2	PLCXD2;FLJ31579	phosphatidylinositol-specific phospholipase C, X domain containing 2	0.65	0.96	1.6	0.85
SORL1	SORL1;C11orf32;FLJ21930;FLJ39258;LR11;LRP9;SORLA;SorLA-1;gp250	sortilin-related receptor, L(DLR class) A repeats containing	0.64	0.98	1.6	0.76
CD82	CD82;4F9;C33;GR15;IA4;KAI1;R2;SAR2;ST6;TSPAN27	CD82 molecule	0.63	0.9	1.6	0.7

FAM3C	FAM3C;ILEI	family with sequence similarity 3, member C	0.63	1.04	1.6	0.79
NUCKS1	NUCKS1;FLJ21480;FLJ32016;FLJ38536;NUCKS	nuclear casein kinase and cyclin-dependent kinase substrate 1	0.69	1.19	1.61	0.85
MRPL52	MRPL52	mitochondrial ribosomal protein L52	0.62	0.94	1.61	0.72
CD70	CD70;CD27L;CD27LG;TNFSF7	CD70 molecule	0.8	1.06	1.26	0.75
AMPD1	AMPD1;MAD;MADA	adenosine monophosphate deaminase 1	0.78	1.09	1.26	0.75
LRRC49	LRRC49;FLJ20156	leucine rich repeat containing 49	0.76	1.02	1.26	0.71
WDPCP	WDPCP;BBS15;C2orf86;DKFZp686C12204;FRITZ;FRTZ	WD repeat containing planar cell polarity effector	0.68	0.94	1.26	0.68
RBBP4	RBBP4;NURF55;RBAP48	retinoblastoma binding protein 4	0.84	1.12	1.27	0.74
CDK9	CDK9;C-2k;CDC2L4;CTK1;PITALRE;TAK	cyclin-dependent kinase 9	0.8	1.1	1.27	0.78
GCOM1	GCOM1;FLJ30973;GRINL1A;Gcom2;Gup;Gup1;Gup2;MGC126694;MGC138353;MYOZAP	GRINL1A complex locus	0.7	0.96	1.27	0.64
PARN	PARN;DAN	poly(A)-specific ribonuclease	0.7	0.97	1.27	0.67
GTF3A	GTF3A;AP2;TFIIIA	general transcription factor IIIA	0.66	0.92	1.27	0.64
PRF1	PRF1;FLH2;HPLH2;MGC65093;P1;PFN1;PFP	perforin 1 (pore forming protein)	0.84	1.11	1.28	0.84
KLHL35	KLHL35;26597;DKFZp566C0546;FLJ33790	kelch-like 35 (Drosophila)	0.83	1.1	1.28	0.75
UAP1L1	UAP1L1;RP11-229P13.18	UDP-N-acteylglucosamine pyrophosphorylase 1-like 1	0.82	1.08	1.28	0.82
RNPC3	RNPC3;DKFZp667O0118;FLJ20008;FLJ25070;KIAA183;RBM40;RNP	RNA-binding region (RNP1, RRM) containing 3	0.77	1.06	1.28	0.81
ADI1	ADI1;APL1;ARD;FLJ10913;MTCBP1;Ni-ARD;SIPL	acireductone dioxygenase 1	0.72	0.98	1.28	0.64
BRSK2	BRSK2;C11orf7;FLJ41362;PEN11B;SAD1;STK29	BR serine/threonine kinase 2	0.7	0.97	1.28	0.69
TMEM206	TMEM206;C1orf75;FLJ10874;RP11-384C4.5	transmembrane protein 206	0.7	0.96	1.28	0.68
ZNF697	ZNF697;MGC45731	zinc finger protein 697	0.64	0.9	1.28	0.6
GNS	GNS;G6S;MGC21274	glucosamine (N-acetyl)-6-sulfatase	0.84	1.1	1.29	0.85
ACCS	ACCS;ACS;PHACS	1-aminocyclopropane-1-carboxylate synthase homolog (Arabidopsis)(non-functional)	0.82	1.11	1.29	0.9
40057	SEPT9;AF17q25;FLJ75490;KIAA0991;MSF;MSF1;NAPB;PNUTL4;SINT1;SeptD1	septin 9	0.8	1.11	1.29	0.73
DBN1	DBN1;D0S117E;DKFZp434D064	drebrin 1	0.8	1.08	1.29	0.81
MRPL14	MRPL14;L14mt;L32mt;MGC70566;MRP-L14;MRP-L32;MRPL32;RMPL32;RPML32	mitochondrial ribosomal protein L14	0.76	1.04	1.29	0.69
EFEMP2	EFEMP2;FBLN4;MBP1;UPH1	EGF containing fibulin-like extracellular matrix protein 2	0.74	1	1.29	0.74
IMPAD1	IMPAD1;FLJ20421;GPAPP;IMPA3	inositol monophosphatase domain containing 1	0.74	1.01	1.29	0.67
USE1	USE1;P31;SLT1	unconventional SNARE in the ER 1 homolog (S. cerevisiae)	0.73	1.02	1.29	0.75
EML1	EML1;ELP79;EMAP;EMAPL;FLJ45033;HuEMAP	echinoderm microtubule associated protein like 1	0.72	0.99	1.29	0.73
TTC14	TTC14;DKFZp313M1015;DRDL5813;FLJ00166;KIAA1980;PRO19630	tetratricopeptide repeat domain 14	0.84	1.12	1.3	0.85
GPM6A	GPM6A;GPM6;M6A	glycoprotein M6A	0.82	1.12	1.3	0.86
RANGRF	RANGRF;DKFZp686F02139;HSPC236;MGC110973;MOG1;RANGNRF	RAN guanine nucleotide release factor	0.82	1.1	1.3	0.74
PSMG1	PSMG1;C21LRP;DSCR2;LRPC21;PAC1	proteasome (prosome, macropain) assembly chaperone 1	0.82	1.12	1.3	0.83
SH3D21	SH3D21;C1orf113;FLJ22938	SH3 domain containing 21	0.81	1.08	1.3	0.78
SMAD7	SMAD7;CRCS3;FLJ16482;MADH7;MADH8	SMAD family member 7	0.8	1.15	1.3	0.79
VAV1	VAV1;VAV	vav 1 guanine nucleotide exchange factor	0.74	1.02	1.3	0.67
DLAT	DLAT;DLTA;PDC-E2;PDCE2	dihydrolipoamide S-acetyltransferase	0.74	1.02	1.3	0.67
HINFP	HINFP;DKFZp434F162;HiNF-P;MIZF;ZNF743	histone H4 transcription factor	0.7	0.96	1.3	0.71
C8orf37	C8orf37;FLJ30600;smalltalk	chromosome 8 open reading frame 37	0.8	1.16	1.31	0.85
FAM153B	FAM153B;DKFZp434D115	family with sequence similarity 153, member B	0.78	1.06	1.31	0.71
IL1RL2	IL1RL2;IL1R-rp2;IL1RRP2	interleukin 1 receptor-like 2	0.78	1.09	1.31	0.72
C19orf20	C19orf20;GTRGEO22;PGs1	chromosome 19 open reading frame 20	0.78	1.06	1.31	0.8
C22orf32	C22orf32;DDDD;dJ186O1.1	chromosome 22 open reading frame 32	0.78	1.05	1.31	0.78
PPP6R2	PPP6R2;KIAA0685;PP6R2;SAP190;SAPS2	protein phosphatase 6, regulatory subunit 2	0.78	1.1	1.31	0.77
HOOK3	HOOK3;FLJ31058;HK3	hook homolog 3 (Drosophila)	0.74	1	1.31	0.75
ROR1	ROR1;MGC99659;NTRKR1;dJ537F10.1	receptor tyrosine kinase-like orphan receptor 1	0.73	1.03	1.31	0.73
NDST2	NDST2;HSST2;MGC129697;MGC129705;NST2	N-deacetylase/N-sulfotransferase (heparan glucosaminyl) 2	0.72	1.03	1.31	0.71
FASTKD1	FASTKD1;FLJ21901;KIAA1800	FAST kinase domains 1	0.72	1	1.31	0.77
CACNA2D2	CACNA2D2;CACNA2D;KIAA0558;LUAC11.1	calcium channel, voltage-dependent, alpha 2/delta subunit 2	0.68	0.94	1.31	0.73
CDK7	CDK7;CAK1;CDKN7;HCAK;MO15;STK1;p39MO15	cyclin-dependent kinase 7	0.64	0.9	1.31	0.65
PRKACA	PRKACA;MGC102831;MGC48865;PKACA	protein kinase, cAMP-dependent, catalytic, alpha	0.64	0.9	1.31	0.64
FGF21	FGF21	fibroblast growth factor 21	0.82	1.1	1.32	0.87
OCIAD2	OCIAD2;DKFZp686C03164;MGC45416	OCIA domain containing 2	0.82	1.08	1.32	0.78
THADA	THADA;FLJ21877;FLJ44016;FLJ44876;FLJ77530;GITA;KIAA1767	thyroid adenoma associated	0.8	1.1	1.32	0.82
ZNF624	ZNF624;KIAA1349;MGC119602;MGC119603;MGC119605	zinc finger protein 624	0.76	1.02	1.32	0.7
ARHGEF19	ARHGEF19;FLJ33962;WGEF	Rho guanine nucleotide exchange factor (GEF) 19	0.76	1.05	1.32	0.85
XKR9	XKR9;XRG9	XK, Kell blood group complex subunit-related family, member 9	0.72	0.98	1.32	0.66
TEX10	TEX10;FLJ20287;FLJ31157;FLJ34350;bA208F1.2	testis expressed 10	0.72	0.98	1.32	0.68
ZSWIM4	ZSWIM4	zinc finger, SWIM-type containing 4	0.8	1.1	1.33	0.74
SREBF2	SREBF2;SREBP2;bHLHd2	sterol regulatory element binding transcription factor 2	0.8	1.08	1.33	0.72
CLDN10	CLDN10;CPETRL3;OSP-L	claudin 10	0.75	1.02	1.33	0.8
FEM1C	FEM1C;EUROIMAGE686608;EUROIMAGE783647;FEM1A;KIAA1785	fem-1 homolog c (C. elegans)	0.74	1.03	1.33	0.73
TMEM59	TMEM59;C1orf8;FLJ42977;HSPC001	transmembrane protein 59	0.74	1.01	1.33	0.75
ARSJ	ARSJ;FLJ23548	arylsulfatase family, member J	0.72	1.01	1.33	0.67
KRTAP4-2	KRTAP4-2;KAP4.2;KRTAP4.2;MGC119940	keratin associated protein 4-2	0.72	1	1.33	0.68
QDPR	QDPR;DHPR;FLJ42391;PKU2;SDR33C1	quinoid dihydropteridine reductase	0.72	1.04	1.33	0.72
GALNT8	GALNT8;FLJ17364;GALNAC-T8	UDP-N-acetyl-alpha-D-galactosamine:polypeptide N-acetylgalactosaminyltransferase 8 (GalNAc-T8)	0.71	1	1.33	0.66
SDK1	SDK1;FLJ31425	sidekick homolog 1, cell adhesion molecule (chicken)	0.7	0.98	1.33	0.72
LCAT	LCAT	lecithin-cholesterol acyltransferase	0.69	1.02	1.33	0.71
ETV3L	ETV3L;FLJ16478	ets variant 3-like	0.68	0.96	1.33	0.67
C4orf36	C4orf36;MGC26744	chromosome 4 open reading frame 36	0.66	0.96	1.33	0.67
OR2D2	OR2D2;OR11-610;OR2D1;hg27	olfactory receptor, family 2, subfamily D, member 2	0.82	1.08	1.34	0.81
TFAP2B	TFAP2B;AP-2B;AP2-B;MGC21381	transcription factor AP-2 beta (activating enhancer binding protein 2 beta)	0.82	1.1	1.34	0.86
PLD1	PLD1	phospholipase D1, phosphatidylcholine-specific	0.82	1.09	1.34	0.77
ORC2	ORC2;ORC2L	origin recognition complex, subunit 2	0.8	1.07	1.34	0.73
AMMECR1	AMMECR1;AMMERC1	Alport syndrome, mental retardation, midface hypoplasia and elliptocytosis chromosomal region gene 1	0.78	1.1	1.34	0.82
CNOT6	CNOT6;CCR4;KIAA1194	CCR4-NOT transcription complex, subunit 6	0.76	1.06	1.34	0.76
C2orf18	C2orf18;ANT2BP;FLJ20555	chromosome 2 open reading frame 18	0.74	1.02	1.34	0.77
LRRTM3	LRRTM3;MGC131810	leucine rich repeat transmembrane neuronal 3	0.72	1	1.34	0.74
ST3GAL4	ST3GAL4;CGS23;FLJ11867;FLJ46764;NANTA3;SAT3;SIAT4;SIAT4C;ST3GalIV;STZ	ST3 beta-galactoside alpha-2,3-sialyltransferase 4	0.68	1	1.34	0.69
NUPL1	NUPL1;KIAA0410;PRO2463	nucleoporin like 1	0.68	0.94	1.34	0.63
CASK	CASK;CAGH39;CAMGUK;CMG;FGS4;FLJ22219;FLJ31914;LIN2;MICPCH;TNRC8	calcium/calmodulin-dependent serine protein kinase (MAGUK family)	0.68	0.98	1.34	0.69
TTC18	TTC18;FLJ12173;FLJ25765;RP11-152N13.6	tetratricopeptide repeat domain 18	0.64	0.9	1.34	0.63
ESPN	ESPN;DFNB36;DKFZp434A196;DKFZp434G2126	espin	0.64	0.9	1.34	0.73
SLC39A2	SLC39A2;6A1;Eti-1;MGC119190;ZIP-2;ZIP2	solute carrier family 39 (zinc transporter), member 2	0.62	0.88	1.34	0.58
PTOV1	PTOV1;ACID2;DKFZp586I111;MGC71475;PTOV-1	prostate tumor overexpressed 1	0.84	1.16	1.35	0.78
PLEKHA8P1	PLEKHA8P1;FLJ14156;PLEKHA9	pleckstrin homology domain containing, family A member 8 pseudogene 1	0.84	1.19	1.35	0.85
C6orf120	C6orf120;bA160E12.4	chromosome 6 open reading frame 120	0.82	1.08	1.35	0.85
TAT	TAT	tyrosine aminotransferase	0.78	1.05	1.35	0.71
PKM2	PKM2;CTHBP;MGC3932;OIP3;PK3;PKM;TCB;THBP1	pyruvate kinase, muscle	0.78	1.05	1.35	0.87
KCNMB1	KCNMB1;K(VCA)beta;SLO-BETA;hslo-beta	potassium large conductance calcium-activated channel, subfamily M, beta member 1	0.77	1.12	1.35	0.86
MYBPC3	MYBPC3;CMH4;DKFZp779E1762;FHC;MYBP-C	myosin binding protein C, cardiac	0.75	1.02	1.35	0.68
COL25A1	COL25A1;AMY;CLAC;CLAC-P;CLACP	collagen, type XXV, alpha 1	0.73	1.01	1.35	0.8
UBXN2B	UBXN2B;p37	UBX domain protein 2B	0.72	1	1.35	0.75
SLC25A39	SLC25A39;CGI69;FLJ22407	solute carrier family 25, member 39	0.72	1	1.35	0.75
ECI1	ECI1;DCI	enoyl-CoA delta isomerase 1	0.7	1.01	1.35	0.66
ANO3	ANO3;C11orf25;TMEM16C	anoctamin 3	0.67	0.94	1.35	0.63
CRISP2	CRISP2;CRISP-2;CT36;GAPDL5;MGC111136;TPX1;TSP1	cysteine-rich secretory protein 2	0.66	0.93	1.35	0.63
PCDHB7	PCDHB7;MGC111391;MGC163205;PCDH-BETA7	protocadherin beta 7	0.66	0.92	1.35	0.69
C17orf48	C17orf48;NBLA03831	chromosome 17 open reading frame 48	0.64	0.92	1.35	0.65
DFFA	DFFA;DFF-45;DFF1;ICAD	DNA fragmentation factor, 45kDa, alpha polypeptide	0.62	0.88	1.35	0.58
INADL	INADL;Cipp;FLJ26982;InaD-like;PATJ;hINADL	InaD-like (Drosophila)	0.84	1.17	1.36	0.89
ANXA6	ANXA6;ANX6;CBP68	annexin A6	0.81	1.07	1.36	0.81
FAM71A	FAM71A;FLJ32796;RP11-338C15.4	family with sequence similarity 71, member A	0.8	1.06	1.36	0.79
GRAMD1B	GRAMD1B;KIAA1201;MGC125622;MGC125623;MGC125625	GRAM domain containing 1B	0.8	1.06	1.36	0.79
PLA2G6	PLA2G6;Cal-PLA2;GVI;INAD1;IPLA2-VIA;NBIA2A;NBIA2B;PARK14;PLA2;PNPLA9;iPLA2	phospholipase A2, group VI (cytosolic, calcium-independent)	0.78	1.09	1.36	0.86
RPS9	RPS9;S9	ribosomal protein S9	0.77	1.08	1.36	0.82
OR2L3	OR2L3	olfactory receptor, family 2, subfamily L, member 3	0.76	1.03	1.36	0.7
PLEKHM1	PLEKHM1;AP162;B2;KIAA0356;OPTB6	pleckstrin homology domain containing, family M (with RUN domain) member 1	0.73	1.02	1.36	0.68
CCL4L1	CCL4L1;AT744.2;CCL4L;LAG-1;LAG1;SCYA4L	chemokine (C-C motif) ligand 4-like 1	0.72	1.01	1.36	0.68
PIGX	PIGX;FLJ20522	phosphatidylinositol glycan anchor biosynthesis, class X	0.68	1.01	1.36	0.77
MRP63	MRP63;MGC3243;bMRP63	mitochondrial ribosomal protein 63	0.66	0.93	1.36	0.75
ARRDC4	ARRDC4;FLJ36045	arrestin domain containing 4	0.84	1.14	1.37	0.94
PCDHA8	PCDHA8;PCDH-ALPHA8	protocadherin alpha 8	0.83	1.11	1.37	0.84
CGREF1	CGREF1;CGR11	cell growth regulator with EF-hand domain 1	0.8	1.06	1.37	0.81
CELSR3	CELSR3;CDHF11;EGFL1;FMI1;HFMI1;MEGF2;RESDA1	cadherin, EGF LAG seven-pass G-type receptor 3 (flamingo homolog, Drosophila)	0.79	1.05	1.37	0.94
TERF2	TERF2;TRBF2;TRF2	telomeric repeat binding factor 2	0.77	1.08	1.37	0.72
OTUD6A	OTUD6A;DUBA2;FLJ25831;HSHIN6	OTU domain containing 6A	0.76	1.06	1.37	0.79
SDHA	SDHA;CMD1GG;FP;PGL5;SDH1;SDH2;SDHF	succinate dehydrogenase complex, subunit A, flavoprotein (Fp)	0.74	1.07	1.37	0.71
TMPRSS15	TMPRSS15;ENTK;MGC133046;PRSS7	transmembrane protease, serine 15	0.74	1	1.37	0.75
PAQR4	PAQR4;FLJ30002	progestin and adipoQ receptor family member IV	0.72	1	1.37	0.68
MED15	MED15;ARC105;CAG7A;CTG7A;DKFZp686A2214;DKFZp762B1216;FLJ42282;FLJ42935;PCQAP;TIG-1;TIG1;TNRC7	mediator complex subunit 15	0.72	0.99	1.37	0.68
ZNF285	ZNF285;FLJ30747;ZNF285A	zinc finger protein 285	0.72	1.06	1.37	0.74
ZNF514	ZNF514;MGC126229;MGC126230	zinc finger protein 514	0.71	0.98	1.37	0.66
GRIN1	GRIN1;MRD8;NMDA1;NMDAR1;NR1	glutamate receptor, ionotropic, N-methyl D-aspartate 1	0.61	0.87	1.37	0.68
SPCS2	SPCS2;KIAA0102;MGC117366	signal peptidase complex subunit 2 homolog (S. cerevisiae)	0.61	0.88	1.37	0.6
RNF114	RNF114;PSORS12;ZNF313	ring finger protein 114	0.84	1.11	1.38	0.8
GAPVD1	GAPVD1;DKFZp434C212;GAPEX5;KIAA1521;MGC138847;MGC138848;RAP6	GTPase activating protein and VPS9 domains 1	0.83	1.09	1.38	0.88
FOXD3	FOXD3;AIS1;Genesis;HFH2	forkhead box D3	0.78	1.09	1.38	0.85

RILP	RILP;FLJ31193	Rab interacting lysosomal protein	0.74	1	1.38	0.76
CDKL5	CDKL5;EIEE2;ISSX;STK9	cyclin-dependent kinase-like 5	0.72	1	1.38	0.83
ACN9	ACN9	ACN9 homolog (S. cerevisiae)	0.72	0.98	1.38	0.67
TBX21	TBX21;T-PET;T-bet;TBET;TBLYM	T-box 21	0.71	0.98	1.38	0.77
SLC22A25	SLC22A25;HIMTP;MGC120420;UST6	solute carrier family 22, member 25	0.7	0.96	1.38	0.67
PARD3B	PARD3B;ALS2CR19;FLJ92364;MGC16131;PAR3B;PAR3L;PAR3LC;PAR3beta;Par3Lb	par-3 partitioning defective 3 homolog B (C. elegans)	0.69	0.95	1.38	0.69
XPO4	XPO4;FLJ13046;KIAA1721;exp4	exportin 4	0.69	1	1.38	0.74
PLCB2	PLCB2;FLJ38135	phospholipase C, beta 2	0.68	0.95	1.38	0.74
GTF2H3	GTF2H3;BTF2;TFB4;TFIIH	general transcription factor IIH, polypeptide 3, 34kDa	0.62	0.88	1.38	0.68
GTF2F1	GTF2F1;BTF4;RAP74;TF2F1;TFIIF	general transcription factor IIF, polypeptide 1, 74kDa	0.79	1.1	1.39	0.75
ICA1	ICA1;ICA69;ICAp69	islet cell autoantigen 1, 69kDa	0.77	1.1	1.39	0.83
ZNF829	ZNF829;DKFZp686K21248;DKFZp779O175;FLJ27459;MGC129866;MGC129867	zinc finger protein 829	0.76	1.03	1.39	0.78
MAGEC2	MAGEC2;CT10;HCA587;MAGEE1;MGC13377	melanoma antigen family C, 2	0.74	1.04	1.39	0.79
AMBRA1	AMBRA1;DCAF3;FLJ20294;KIAA1736;MGC33725;WDR94	autophagy/beclin-1 regulator 1	0.72	0.98	1.39	0.74
CMAS	CMAS	cytidine monophosphate N-acetylneuraminic acid synthetase	0.72	0.98	1.39	0.84
C4orf33	C4orf33;FLJ33703	chromosome 4 open reading frame 33	0.71	0.97	1.39	0.72
KCNN2	KCNN2;KCa2.2;SK2;SKCA2;hSK2	potassium intermediate/small conductance calcium-activated channel, subfamily N, member 2	0.7	1.02	1.39	0.79
EML5	EML5;DKFZp781D1122;EMAP-2	echinoderm microtubule associated protein like 5	0.69	0.98	1.39	0.71
NEK5	NEK5;MGC75495	NIMA (never in mitosis gene a)-related kinase 5	0.68	0.95	1.39	0.78
PDCD1LG2	PDCD1LG2;B7DC;Btdc;CD273;MGC142238;MGC142240;PD-L2;PDCD1L2;PDL2;bA574F11.2	programmed cell death 1 ligand 2	0.68	0.96	1.39	0.66
EXOC4	EXOC4;MGC27170;REC8;SEC8;SEC8L1;Sec8p	exocyst complex component 4	0.84	1.27	1.4	0.97
PRSS42	PRSS42;TESSP2	protease, serine, 42	0.77	1.06	1.4	0.8
ARHGEF9	ARHGEF9;COLLYBISTIN;EIEE8;HPEM-2;KIAA0424;PEM-2;PEM2	Cdc42 guanine nucleotide exchange factor (GEF) 9	0.74	1.07	1.4	0.73
INTS3	INTS3;C1orf193;C1orf60;DKFZp686E1950;DKFZp686O20115;DKFZp781I1253;FLJ21919;FLJ44849;INT3;SOSS-A	integrator complex subunit 3	0.66	0.94	1.4	0.67
KRT3	KRT3;CK3;FLJ95909;K3	keratin 3	0.62	0.89	1.4	0.61
MTMR11	MTMR11;CRA;RP11-212K13.1	myotubularin related protein 11	0.8	1.17	1.41	0.84
CDK6	CDK6;MGC59692;PLSTIRE	cyclin-dependent kinase 6	0.76	1.16	1.41	0.82
GPR26	GPR26;DKFZp761H2121;FLJ33774;MGC138216	G protein-coupled receptor 26	0.74	1	1.41	0.9
SARDH	SARDH;DMGDHL1;FLJ36475;SAR;SARD;SDH	sarcosine dehydrogenase	0.74	1	1.41	0.78
ADAMTS9	ADAMTS9;FLJ42955;KIAA1312	ADAM metallopeptidase with thrombospondin type 1 motif, 9	0.74	1.02	1.41	0.77
MAMDC4	MAMDC4;AEGP;DKFZp434M1411	MAM domain containing 4	0.74	1.02	1.41	0.76
CAPG	CAPG;AFCP;MCP	capping protein (actin filament), gelsolin-like	0.74	1.02	1.41	0.77
WDR7	WDR7;KIAA0541;TRAG	WD repeat domain 7	0.68	1	1.41	0.75
TAS1R1	TAS1R1;GPR70;T1R1;TR1	taste receptor, type 1, member 1	0.62	0.89	1.41	0.64
CHURC1	CHURC1;C14orf52;FLJ33064;FLJ51978;FLJ78804;chch	churchill domain containing 1	0.84	1.16	1.42	0.88
AAGAB	AAGAB;DKFZp667D2317;DKFZp686G08119;FLJ11506;p34	alpha- and gamma-adaptin binding protein	0.83	1.15	1.42	0.87
HDAC7	HDAC7;DKFZp586J0917;FLJ99588;HD7A;HDAC7A	histone deacetylase 7	0.76	1.17	1.42	0.77
FLNA	FLNA;ABP-280;ABPX;CVD1;DKFZp434P031;FLJ43642;FLN;FLN-A;FLN1;FMD;MNS;NHBP;OPD;OPD1;OPD2;XLVD;XMVD	filamin A, alpha	0.75	1.04	1.42	0.73
MUDENG	MUDENG;C14orf108;FLJ10813;MuD	MU-2/AP1M2 domain containing, death-inducing	0.73	1.07	1.42	0.81
SERINC5	SERINC5;C5orf12;TPO1	serine incorporator 5	0.72	1.12	1.42	0.75
KIAA0586	KIAA0586;Talpid3	KIAA0586	0.66	0.94	1.42	0.65
LRPAP1	LRPAP1;A2MRAP;A2RAP;HBP44;MGC138272;MRAP;RAP	low density lipoprotein receptor-related protein associated protein 1	0.84	1.12	1.43	0.89
RS1	RS1;RS;XLRS1	retinoschisin 1	0.72	1	1.43	0.72
TP53BP1	TP53BP1;53BP1;FLJ41424;MGC138366;p202	tumor protein p53 binding protein 1	0.72	1.1	1.43	0.72
DCLK2	DCLK2;CL2;CLICK-II;CLICK2;CLIK2;DCAMKL2;DCDC3;DCDC3B;DCK2;DKFZp761I032;MGC45428	doublecortin-like kinase 2	0.7	0.97	1.43	0.87
KRTAP4-1	KRTAP4-1;KAP4.1;KAP4.10;KRTAP4-10;KRTAP4.10;MGC118980;MGC118981	keratin associated protein 4-1	0.68	0.95	1.43	0.72
SMYD1	SMYD1;BOP;KMT3D;ZMYND18;ZMYND22	SET and MYND domain containing 1	0.67	0.94	1.43	0.73
ARFGEF2	ARFGEF2;BIG2;FLJ23723;PVNH2;dJ1164I10.1	ADP-ribosylation factor guanine nucleotide-exchange factor 2 (brefeldin A-inhibited)	0.64	0.94	1.43	0.63
RCC1	RCC1;CHC1;RCC1-I;SNHG3-RCC1	regulator of chromosome condensation 1	0.64	0.99	1.43	0.76
SLC25A6	SLC25A6;AAC3;ANT3;ANT3Y;MGC17525	solute carrier family 25 (mitochondrial carrier; adenine nucleotide translocator), member 6	0.64	0.9	1.43	0.78
HBP1	HBP1;FLJ16340	HMG-box transcription factor 1	0.61	0.86	1.45	0.65
CCDC165	CCDC165;KIAA0802	coiled-coil domain containing 165	0.63	0.88	1.47	0.68
RLN2	RLN2;H2;RLXH2;bA12D24.1.1;bA12D24.1.2	relaxin 2	0.78	1.03	1.26	0.77
TCP11L1	TCP11L1;FLJ11336;FLJ11386;dJ85M6.3	t-complex 11 (mouse)-like 1	0.65	0.9	1.26	0.66
IL15	IL15;IL-15;MGC9721	interleukin 15	0.77	1.02	1.27	0.72
WWTR1	WWTR1;DKFZp586I1419;FLJ27004;FLJ45718;TAZ	WW domain containing transcription regulator 1	0.77	1.02	1.27	0.73
ZFAND3	ZFAND3;FLJ13222;FLJ17799;TEX27	zinc finger, AN1-type domain 3	0.64	0.89	1.27	0.65
STMN3	STMN3;SCLIP	stathmin-like 3	0.77	1.02	1.28	0.77
DOLK	DOLK;CDG1M;DK;DK1;KIAA1094;SEC59;TMEM15	dolichol kinase	0.71	0.96	1.28	0.74
QPRT	QPRT;QPRTase	quinolinate phosphoribosyltransferase	0.84	1.09	1.29	0.87
LRRC43	LRRC43;MGC35140	leucine rich repeat containing 43	0.76	1.01	1.29	0.68
CASC3	CASC3;BTZ;MLN51	cancer susceptibility candidate 3	0.76	1.01	1.29	0.67
LDOC1L	LDOC1L;DKFZp761O17121;Mar6;Mart6;dJ1033E15.2	leucine zipper, down-regulated in cancer 1-like	0.73	0.98	1.29	0.7
TOM1L1	TOM1L1;OK/KNS-CL.3;SRCASM	target of myb1 (chicken)-like 1	0.7	0.95	1.29	0.77
SLC4A1	SLC4A1;AE1;BND3;CD233;DI;EMPB3;EPB3;FR;MGC116750;MGC116753;MGC126619;MGC126623;RTA1A;SW;WD;WD1;WR	solute carrier family 4, anion exchanger, member 1 (erythrocyte membrane protein band 3, Diego blood group)	0.66	0.91	1.29	0.6
RUSC1-AS1	RUSC1-AS1;C1orf104;FLJ35976;RP11-21N7.3	RUSC1 antisense RNA 1 (non-protein coding)	0.76	1.01	1.3	0.76
FOPNL	FOPNL;C16orf63;DKFZp686N1651;FLJ31153;FOR20;PHSECRG2	FGFR1OP N-terminal like	0.75	1	1.3	0.75
SLC23A2	SLC23A2;KIAA0238;NBTL1;SLC23A1;SVCT2;YSPL2	solute carrier family 23 (nucleobase transporters), member 2	0.79	1.04	1.31	0.7
PLEKHN1	PLEKHN1;DKFZp434H2010;MGC120613;MGC120616;RP11-54O7.7	pleckstrin homology domain containing, family N member 1	0.65	0.9	1.31	0.63
GAS2L1	GAS2L1;GAR22;MGC17243	growth arrest-specific 2 like 1	0.65	0.9	1.31	0.6
HNF1A	HNF1A;HNF-1A;HNF1;IDDM20;LFB1;MODY3;TCF-1;TCF1	HNF1 homeobox A	0.76	1.01	1.32	0.78
ITIH1	ITIH1;H1P;IATIH;IGHEP1;ITI-HC1;ITIH;MGC126415;SHAP	inter-alpha-trypsin inhibitor heavy chain 1	0.64	0.89	1.33	0.67
GALNT3	GALNT3;DKFZp686C10199;GalNAc-T3;HFTC;HHS;MGC61909	UDP-N-acetyl-alpha-D-galactosamine:polypeptide N-acetylgalactosaminyltransferase 3 (GalNAc-T3)	0.62	0.87	1.33	0.58
ACSL3	ACSL3;ACS3;FACL3;PRO2194	acyl-CoA synthetase long-chain family member 3	0.84	1.09	1.34	0.77
SLC26A6	SLC26A6;DKFZp586E1422	solute carrier family 26, member 6	0.78	1.03	1.34	0.86
GTF2H5	GTF2H5;C6orf175;TFB5;TFIIH;TGF2H5;TTD;TTD-A;TTDA;bA120J8.2	general transcription factor IIH, polypeptide 5	0.73	0.98	1.34	0.67
NIN	NIN;KIAA1565	ninein (GSK3B interacting protein)	0.64	0.89	1.35	0.61
TNFAIP8	TNFAIP8;GG2-1;MDC-3.13;SCC-S2;SCCS2	tumor necrosis factor, alpha-induced protein 8	0.78	1.03	1.36	0.89
MAN2B2	MAN2B2;KIAA0935	mannosidase, alpha, class 2B, member 2	0.67	0.92	1.37	0.62
KLHL34	KLHL34;FLJ34960;MGC125650	kelch-like 34 (Drosophila)	0.84	1.09	1.38	0.88
ABCD1	ABCD1;ABC42;ALD;ALDP;AMN	ATP-binding cassette, sub-family D (ALD), member 1	0.78	1.03	1.38	0.75
LY6E	LY6E;RIG-E;RIGE;SCA-2;SCA2;TSA-1	lymphocyte antigen 6 complex, locus E	0.78	1.03	1.38	0.74
VAMP4	VAMP4;VAMP-4;VAMP24	vesicle-associated membrane protein 4	0.7	0.95	1.38	0.71
CEND1	CEND1;BM88;FLJ90066;MGC34326	cell cycle exit and neuronal differentiation 1	0.61	0.86	1.38	0.65
ACTBL2	ACTBL2;ACT;DKFZp686D0972	actin, beta-like 2	0.76	1.01	1.39	0.77
NACC2	NACC2;BEND9;BTBD14;BTBD14A;MGC23427	NACC family member 2, BEN and BTB (POZ) domain containing	0.75	1	1.39	0.72
ETV1	ETV1;DKFZp781L0674;ER81;MGC104699;MGC120533;MGC120534	ets variant 1	0.84	1.16	1.25	0.8
ALS2CR8	ALS2CR8;CARF;DKFZp667N246;FLJ12675;FLJ21579;NYD-SP24	amyotrophic lateral sclerosis 2 (juvenile) chromosome region, candidate 8	0.84	1.1	1.25	0.73
AKR1B10	AKR1B10;AKR1B11;AKR1B12;ALDRLn;ARL-1;ARL1;HIS;HSI;MGC14103	aldo-keto reductase family 1, member B10 (aldose reductase)	0.76	1.02	1.25	0.67
DKK4	DKK4;DKK-4;MGC129562;MGC129563	dickkopf homolog 4 (Xenopus laevis)	0.7	0.96	1.25	0.7
THEMIS	THEMIS;C6orf190;C6orf207;FLJ40584;GASP;MGC163388;SPOT;TSEPA;bA325O24.3;bA325O24.4	thymocyte selection associated	0.83	1.08	1.25	0.87
HIST2H3A	HIST2H3A;H3/n;H3/o;HIST2H3C;HIST2H3D	histone cluster 2, H3a	0.82	1.07	1.25	0.81
LINGO1	LINGO1;FLJ14594;LERN1;LRRN6A;MGC17422;UNQ201	leucine rich repeat and Ig domain containing 1	0.71	0.96	1.25	0.73
RPRD1B	RPRD1B;C20orf77;CREPT;DKFZp434P0735;FLJ44520;NET60;dJ1057B20.2	regulation of nuclear pre-mRNA domain containing 1B	1.04	1.3	1.13	0.95
ZFYVE1	ZFYVE1;DFCP1;KIAA1589;TAFF1;ZNFN2A1	zinc finger, FYVE domain containing 1	1.02	1.28	1.14	0.94
OSR1	OSR1;ODD	odd-skipped related 1 (Drosophila)	1.14	1.4	1.15	0.98
KITLG	KITLG;DKFZp686F2250;FPH2;KL-1;Kitl;MGF;SCF;SF;SHEP7;kit-ligand	KIT ligand	0.97	1.23	1.15	0.85
RAB27B	RAB27B;C25KG	RAB27B, member RAS oncogene family	1.13	1.4	1.17	0.93
KRTAP9-4	KRTAP9-4;KAP9.4;KRTAP9.4	keratin associated protein 9-4	1.04	1.32	1.17	0.97
ARID3C	ARID3C	AT rich interactive domain 3C (BRIGHT-like)	0.96	1.22	1.17	0.89
CAMKMT	CAMKMT;C2orf34;CLNMT;CaM KMT;Cam;FLJ23451;KMT	calmodulin-lysine N-methyltransferase	0.94	1.21	1.17	0.8
MBD6	MBD6;KIAA1887	methyl-CpG binding domain protein 6	1.09	1.35	1.18	1.01
CDK16	CDK16;FLJ16665;PCTAIRE;PCTAIRE1;PCTGAIRE;PCTK1	cyclin-dependent kinase 16	0.97	1.23	1.18	0.87
ERBB4	ERBB4;HER4;MGC138404;p180erbB4	v-erb-a erythroblastic leukemia viral oncogene homolog 4 (avian)	0.9	1.16	1.18	0.82
SLC14A1	SLC14A1;FLJ33745;FLJ41687;HUT11;HsT1341;JK;RACH1;UT-B1;UT1;UTE	solute carrier family 14 (urea transporter), member 1 (Kidd blood group)	0.92	1.21	1.19	0.84
HNF1B	HNF1B;FJHN;HNF1beta;HNF2;HPC11;LF-B3;LFB3;MODY5;TCF2;VHNF1	HNF1 homeobox B	0.88	1.16	1.19	0.8
SPATS2L	SPATS2L;DKFZp564A2416;DNAPTP6;SGNP	spermatogenesis associated, serine-rich 2-like	1.08	1.41	1.2	1.09
EEF1D	EEF1D;EF-1D;EF1D;FLJ20897;FP1047	eukaryotic translation elongation factor 1 delta (guanine nucleotide exchange protein)	1.03	1.31	1.2	0.86
CEP95	CEP95;CCDC45;DKFZp667E1824	centrosomal protein 95kDa	1.01	1.27	1.2	0.91
CEP76	CEP76;C18orf9;FLJ12542;HsT1705	centrosomal protein 76kDa	0.92	1.2	1.2	0.8
FRA10AC1	FRA10AC1;C10orf4;F26C11.1-like;FRA10A	fragile site, folic acid type, rare, fra(10)(q23.3) or fra(10)(q24.2) candidate 1	1.01	1.28	1.21	0.96
NF1	NF1;DKFZp686J1293;FLJ21220;NFNS;VRNF;WSS	neurofibromin 1	0.96	1.23	1.21	0.81
EPHB2	EPHB2;CAPB;DRT;EK5;EPHT3;ERK;Hek5;MGC87492;PCBC;Tyro5	EPH receptor B2	0.91	1.18	1.21	0.84
KLHL3	KLHL3;FLJ40871;KIAA1129;MGC44594	kelch-like 3 (Drosophila)	0.9	1.19	1.21	0.88
ZAP70	ZAP70;FLJ17670;FLJ17679;SRK;STD;TZK;ZAP-70	zeta-chain (TCR) associated protein kinase 70kDa	0.86	1.13	1.21	0.8
EXPH5	EXPH5;DKFZp586F1223;DKFZp781H0795;KIAA0624;MGC133291;MGC134967;SLAC2-B;SLAC2B	exophilin 5	0.86	1.12	1.21	0.86
ALG13	ALG13;CXorf45;FLJ23018;FLJ31785;GLT28D1;MDS031;MGC12423;YGL047W	asparagine-linked glycosylation 13 homolog (S. cerevisiae)	1.08	1.39	1.22	0.99
WDR52	WDR52;DKFZp781C082;FLJ11142;MGC126631;MGC142113	WD repeat domain 52	0.94	1.23	1.22	0.83
DTYMK	DTYMK;CDC8;FLJ44192;MGC198617;PP3731;TMPK;TYMK	deoxythymidylate kinase (thymidylate kinase)	0.94	1.23	1.22	0.87
PREB	PREB;MGC3467;SEC12	prolactin regulatory element binding	0.91	1.19	1.22	0.78
BHLHA15	BHLHA15;BHLHB8;MIST1	basic helix-loop-helix family, member a15	0.88	1.15	1.22	0.8
VEGFA	VEGFA;MGC70609;MVCD1;VEGF;VPF	vascular endothelial growth factor A	1.27	1.57	1.23	1.06
PDGFC	PDGFC;FALLOTEIN;SCDGF	platelet derived growth factor C	1.06	1.34	1.23	0.93
SLC37A4	SLC37A4;G6PT1;G6PT2;G6PT3;GSD1b;GSD1c;GSD1d;MGC15729;TRG-19;TRG19	solute carrier family 37 (glucose-6-phosphate transporter), member 4	1.02	1.28	1.23	1.02
TMX4	TMX4;DJ971N18.2;KIAA1162;PDIA14;TXNDC13	thioredoxin-related transmembrane protein 4	0.92	1.21	1.23	0.8
NEDD1	NEDD1;FLJ35902;GCP-WD;TUBGCP7	neural precursor cell expressed, developmentally down-regulated 1	0.89	1.19	1.23	0.87
MBOAT1	MBOAT1;LPEAT1;MGC44669;OACT1;dJ434O11.1	membrane bound O-acyltransferase domain containing 1	0.85	1.12	1.23	0.87
PARP12	PARP12;FLJ22693;MST109;MSTP109;ZC3H1;ZC3HDC1	poly (ADP-ribose) polymerase family, member 12	0.92	1.18	1.24	0.85
VPREB1	VPREB1;IGI;IGVPB;VPREB	pre-B lymphocyte 1	0.9	1.16	1.24	0.79

TM2D1	TM2D1;BBP	TM2 domain containing 1	0.86	1.15	1.24	0.76
BRWD3	BRWD3;BRODL;FLJ33254;FLJ38568;MRX93	bromodomain and WD repeat domain containing 3	1.29	1.54	1.16	1.15
KCTD6	KCTD6;KCASH3;MGC27385	potassium channel tetramerisation domain containing 6	1.04	1.29	1.18	1
LCOR	LCOR;FLJ38026;KIAA1795;MLR2;RP11-175O19.1	ligand dependent nuclear receptor corepressor	0.87	1.12	1.18	0.74
FAM171B	FAM171B;FLJ34104;KIAA1946	family with sequence similarity 171, member B	1	1.25	1.2	0.88
PKDREJ	PKDREJ	polycystic kidney disease (polycystin) and REJ homolog (sperm receptor for egg jelly homolog, sea urchin)	0.94	1.19	1.2	0.92
A1CF	A1CF;ACF;ACF64;ACF65;APOBEC1CF;ASP;MGC163391	APOBEC1 complementation factor	1.02	1.27	1.21	0.92
MOSC1	MOSC1;FLJ22390;MARC1	MOCO sulphurase C-terminal domain containing 1	0.96	1.21	1.21	0.93
RNF130	RNF130;G1RZFP;GOLIATH;GP;MGC117241;MGC138647;MGC99542	ring finger protein 130	1.2	1.45	1.22	1.3
UBXN4	UBXN4;FLJ23318;KIAA0242;KIAA2042;UBXD2;UBXDC1;erasin	UBX domain protein 4	0.91	1.16	1.22	0.87
CKS2	CKS2;CKSHS2	CDC28 protein kinase regulatory subunit 2	1.12	1.37	1.24	1.14
IL18	IL18;IGIF;IL-18;IL-1g;IL1F4;MGC12320	interleukin 18 (interferon-gamma-inducing factor)	0.88	1.13	1.24	0.77
IGF2R	IGF2R;CD222;CIMPR;M6P-R;MPR1;MPRI	insulin-like growth factor 2 receptor	0.82	1.08	1.21	0.76
PAPD4	PAPD4;FLJ38499;GLD2	PAP associated domain containing 4	0.77	1.03	1.21	0.75
COBLL1	COBLL1;COBLR1;KIAA0977	COBL-like 1	0.82	1.08	1.22	0.8
OR4F16	OR4F16;OR1-1;OR4F29;OR4F3;OR7-21	olfactory receptor, family 4, subfamily F, member 16	0.81	1.08	1.22	0.79
PDZK1IP1	PDZK1IP1;DD96;MAP17;RP1-18D14.5;SPAP	PDZK1 interacting protein 1	0.8	1.08	1.22	0.79
KCTD16	KCTD16;DKFZp781A1155;KIAA1317;MGC138167	potassium channel tetramerisation domain containing 16	0.76	1.02	1.22	0.75
COMMD9	COMMD9;FLJ31106	COMM domain containing 9	0.76	1.03	1.23	0.76
PTTG1IP	PTTG1IP;C21orf1;C21orf3;PBF	pituitary tumor-transforming 1 interacting protein	0.76	1.02	1.23	0.75
ATOH8	ATOH8;FLJ14708;FLJ38730;HATH6;bHLHa21	atonal homolog 8 (Drosophila)	0.82	1.08	1.24	0.8
SLTM	SLTM;DKFZp762G052;FLJ10005;FLJ13213;Met	SAFB-like, transcription modulator	0.82	1.08	1.24	0.83
INTS10	INTS10;C8orf35;FLJ10569;INT10	integrator complex subunit 10	0.81	1.06	1.21	0.71
TTC21B	TTC21B;ATD4;FLJ11457;JBTS11;NPHP12;THM1	tetratricopeptide repeat domain 21B	0.83	1.08	1.22	0.76
SCNN1G	SCNN1G;BESC3;ENaCg;ENaCgamma;PHA1;SCNEG	sodium channel, nonvoltage-gated 1, gamma	0.77	1.02	1.23	0.79
GLRX3	GLRX3;FLJ11864;GLRX4;GRX3;GRX4;PICOT;TXNL2;TXNL3	glutaredoxin 3	0.79	1.04	1.24	0.68
OPCML	OPCML;IGLON1;OBCAM;OPCM	opioid binding protein/cell adhesion molecule-like	1.12	1.35	1.26	0.97
LSM1	LSM1;CASM;YJL124C	LSM1 homolog, U6 small nuclear RNA associated (S. cerevisiae)	1.06	1.28	1.26	0.98
GPATCH8	GPATCH8;GPATC8;KIAA0553	G patch domain containing 8	1.02	1.14	1.26	0.89
IFT57	IFT57;ESRRBL1;FLJ10147;HIPPI;MHS4R2	intraflagellar transport 57 homolog (Chlamydomonas)	1	1.24	1.26	0.87
COX7B	COX7B	cytochrome c oxidase subunit VIIb	1	1.13	1.26	0.9
ASPHD2	ASPHD2;FLJ39838	aspartate beta-hydroxylase domain containing 2	0.98	1.2	1.26	0.85
CYORF15B	CYORF15B	ENTREZ STATUS: REMAPPED TO GENE ID: 246126	0.97	1.08	1.26	0.87
CLYBL	CLYBL;CLB;bA134O15.1	citrate lyase beta like	0.92	1.07	1.26	0.95
DUSP19	DUSP19;DUSP17;LMWDSP3;MGC138210;SKRP1;TS-DSP1	dual specificity phosphatase 19	0.92	1.08	1.26	0.82
SCRN2	SCRN2;Ses2	secernin 2	0.91	1.14	1.26	0.85
TBC1D23	TBC1D23;DKFZp667G062;FLJ11046;NS4ATP1	TBC1 domain family, member 23	0.91	1.03	1.26	0.81
CAPN6	CAPN6;CANPX;CAPNX;CalpM;DJ914P14.1	calpain 6	0.9	1.04	1.26	0.78
ALDH7A1	ALDH7A1;ATQ1;EPD;FLJ11738;FLJ92814;PDE	aldehyde dehydrogenase 7 family, member A1	0.89	1.06	1.26	0.83
GPR63	GPR63;PSP24(beta);PSP24B	G protein-coupled receptor 63	0.88	1.07	1.26	0.97
FAM181B	FAM181B;MGC33846	family with sequence similarity 181, member B	0.88	1	1.26	0.79
PCDH1	PCDH1;FLJ53887;MGC45991;PC42;PCDH42	protocadherin 1	0.88	1.08	1.26	0.9
DBX1	DBX1	developing brain homeobox 1	0.86	0.97	1.26	0.76
CCBL1	CCBL1;FLJ95217;GTK;KAT1;KATI;MGC29624	cysteine conjugate-beta lyase, cytoplasmic	0.86	1.09	1.26	0.74
NAPEPLD	NAPEPLD;DKFZp781D1098;FMP30;NAPE-PLD	N-acyl phosphatidylethanolamine phospholipase D	0.99	1.15	1.27	1.02
TARBP1	TARBP1;FLJ30482;TRM3;TRP-185;TRP185	TAR (HIV-1) RNA binding protein 1	0.95	1.15	1.27	0.83
CCKBR	CCKBR;CCK-B;CCK2R;GASR	cholecystokinin B receptor	0.94	1.1	1.27	0.83
SPRN	SPRN;FLJ41197;SHADOO;SHO;bA108K14.1	shadow of prion protein homolog (zebrafish)	0.94	1.16	1.27	0.91
PRSS55	PRSS55;T-SP1;UNQ9391	protease, serine, 55	0.92	1.06	1.27	0.85
ZNF718	ZNF718;FLJ90036	zinc finger protein 718	0.91	1.12	1.27	0.86
TEKT5	TEKT5;FLJ32871;MGC163208;MGC163210	tektin 5	0.9	1.02	1.27	0.79
ATP6V1E2	ATP6V1E2;ATP6E1;ATP6EL2;ATP6V1EL2;MGC9341;VMA4	ATPase, H+ transporting, lysosomal 31kDa, V1 subunit E2	0.88	1.12	1.27	0.76
GAL	GAL;GALN;GLNN;GMAP;MGC40167	galanin prepropeptide	0.88	1	1.27	0.8
TMEM183B	TMEM183B;C1orf37-DUP	transmembrane protein 183B	0.88	1.09	1.27	0.82
DDTL	DDTL	D-dopachrome tautomerase-like	0.86	1.1	1.27	0.84
VPS52	VPS52;ARE1;DKFZp547I194;RP5-1033B10;SAC2;SACM2L;dJ1033B10.5	vacuolar protein sorting 52 homolog (S. cerevisiae)	0.86	1.04	1.27	0.8
EPM2A	EPM2A;EPM2;MELF	epilepsy, progressive myoclonus type 2A, Lafora disease (laforin)	1.12	1.33	1.28	1
PPP1R3F	PPP1R3F;HB2E	protein phosphatase 1, regulatory subunit 3F	1.03	1.19	1.28	0.95
PRR3	PRR3;CAT56;MGC161733;MGC161735	proline rich 3	1	1.16	1.28	0.92
PTPRG	PTPRG;HPTPG;PTPG;R-PTP-GAMMA;RPTPG	protein tyrosine phosphatase, receptor type, G	0.97	1.15	1.28	0.87
39508	MARCH8;MARCH-VIII;MIR;RNF178;c-MIR	membrane-associated ring finger (C3HC4) 8	0.88	1.08	1.28	0.78
PLEKHO2	PLEKHO2;DKFZp761K2312;FLJ38884;PLEKHQ1;PP1628;pp9099	pleckstrin homology domain containing, family O member 2	0.86	0.99	1.28	0.77
CEP97	CEP97;2810403B08Rik;FLJ23047;FLJ26462;LRRIQ2	centrosomal protein 97kDa	0.86	1	1.28	0.8
NSF	NSF;SKD2	N-ethylmaleimide-sensitive factor	0.86	1.08	1.28	0.93
DUSP18	DUSP18;DSP18;DUSP20;LMWDSP20;MGC32658;bK963H5.1	dual specificity phosphatase 18	1.07	1.27	1.29	0.96
MID2	MID2;FLJ37715;FLJ41813;FXY2;RNF60;TRIM1	midline 2	0.96	1.18	1.29	0.85
LOC402715	LOC402715	karyopherin alpha 2 (RAG cohort 1, importin alpha 1) pseudogene	0.92	1.14	1.29	0.87
GSTM1	GSTM1;GST1;GSTM1-1;GSTM1a-1a;GSTM1b-1b;GTH4;GTM1;H-B;MGC26563;MU;MU-1	glutathione S-transferase mu 1	0.9	1.14	1.29	0.97
TRIP12	TRIP12;KIAA0045;MGC138849;MGC138850;ULF	thyroid hormone receptor interactor 12	0.9	1.07	1.29	0.81
DEFB105A	DEFB105A;BD-5;DEFB-5;DEFB105;DEFB105B	defensin, beta 105A	0.88	1.09	1.29	0.83
PILRB	PILRB;FDFACT1;FDFACT2	paired immunoglobin-like type 2 receptor beta	0.86	1.07	1.29	0.77
TCEB1	TCEB1;SIII;eloC	transcription elongation factor B (SIII), polypeptide 1 (15kDa, elongin C)	0.85	1.04	1.29	0.81
TMEM144	TMEM144;FLJ11155	transmembrane protein 144	1.04	1.21	1.3	0.94
ZNF606	ZNF606;FLJ14260;KIAA1852;ZNF328	zinc finger protein 606	1.03	1.19	1.3	0.99
ILKAP	ILKAP;DKFZp434J2031;FLJ10181;ILKAP2;ILKAP3;MGC4846;PP2C-DELTA	integrin-linked kinase-associated serine/threonine phosphatase	0.98	1.18	1.3	0.89
AVIL	AVIL;ADVIL;DKFZp779O1812;DOC6;FLJ12386;MGC133244;p92	advillin	0.93	1.09	1.3	0.91
EMR4P	EMR4P;EMR4;FIRE;GPR127;PGR16	egf-like module containing, mucin-like, hormone receptor-like 4 pseudogene	0.92	1.12	1.3	0.84
TUBGCP5	TUBGCP5;GCP5;KIAA1899	tubulin, gamma complex associated protein 5	0.88	1.1	1.3	0.79
C5orf35	C5orf35;MGC33648	chromosome 5 open reading frame 35	0.86	1.07	1.3	0.81
UFD1L	UFD1L;UFD1	ubiquitin fusion degradation 1 like (yeast)	0.86	1.1	1.3	0.83
MMP9	MMP9;CLG4B;GELB;MANDP2;MMP-9	matrix metallopeptidase 9 (gelatinase B, 92kDa gelatinase, 92kDa type IV collagenase)	1.04	1.25	1.31	0.94
TEX101	TEX101;CT131;GTPR867;MGC4766;NYD-SP8;PRO1884;SGRG;TES101RP	testis expressed 101	0.98	1.16	1.31	0.9
RNF185	RNF185;FLJ38628	ring finger protein 185	0.94	1.18	1.31	0.85
MCCC1	MCCC1;DKFZp686B20267;FLJ25545;MCC-B;MCCA	methylcrotonoyl-CoA carboxylase 1 (alpha)	0.92	1.15	1.31	0.83
NCAPG	NCAPG;CAPG;CHCG;FLJ12450;HCAP-G;MGC126525;NY-MEL-3	non-SMC condensin I complex, subunit G	0.9	1.05	1.31	0.82
RTN4	RTN4;ASY;NI220/250;NOGO;NOGO-A;NOGOC;NSP;NSP-CL;Nbla00271;Nbla10545;Nogo-B;Nogo-C;RTN-X;RTN4-A;RTN4-B1;RTN4-B2;RTN4-C	reticulon 4	0.89	1.06	1.31	0.84
COX19	COX19;MGC104475	COX19 cytochrome c oxidase assembly homolog (S. cerevisiae)	0.87	1.08	1.31	0.88
RPIA	RPIA;RPI	ribose 5-phosphate isomerase A	0.9	1.1	1.32	0.97
WSB1	WSB1;SWIP1;WSB-1	WD repeat and SOCS box containing 1	0.86	1.09	1.32	0.79
ANKRD17	ANKRD17;FLJ22206;GTAR;KIAA0697;NY-BR-16	ankyrin repeat domain 17	0.86	1.1	1.32	0.86
SLC50A1	SLC50A1;HsSWEET1;RAG1AP1;SCP;SWEET1;slv	solute carrier family 50 (sugar transporter), member 1	0.86	1.08	1.32	0.87
C12orf65	C12orf65;COXPD7;FLJ38663	chromosome 12 open reading frame 65	1	1.18	1.33	0.92
DUSP14	DUSP14;MKP-L;MKP6	dual specificity phosphatase 14	0.96	1.18	1.33	0.89
PPM1G	PPM1G;MGC1675;MGC2870;PP2CG;PP2CGAMMA;PPP2CG	protein phosphatase, Mg2+/Mn2+ dependent, 1G	0.88	1.08	1.33	0.82
ARL4A	ARL4A;ARL4	ADP-ribosylation factor-like 4A	0.87	1.1	1.33	0.86
DCLRE1B	DCLRE1B;APOLLO;DKFZp686C24187;FLJ12810;FLJ13998;SNM1B;SNMIB	DNA cross-link repair 1B	0.85	1.04	1.33	0.92
TBC1D9B	TBC1D9B;FLJ41880;KIAA0676;MGC157757	TBC1 domain family, member 9B (with GRAM domain)	1.1	1.31	1.34	1.03
TMEM27	TMEM27;NX-17;NX17	transmembrane protein 27	0.9	1.14	1.34	0.96
GLT25D1	GLT25D1;FLJ22329;MGC117270	glycosyltransferase 25 domain containing 1	0.87	1.1	1.34	0.9
FAM175A	FAM175A;ABRA1;CCDC98;FLJ11520;FLJ12642;FLJ13614	family with sequence similarity 175, member A	0.86	1.03	1.34	0.8
MAGEA12	MAGEA12;CT1.12;MAGE12	melanoma antigen family A, 12	0.96	1.16	1.35	0.92
ZNF554	ZNF554;FLJ34817	zinc finger protein 554	0.92	1.12	1.35	0.89
GLS	GLS;AAD20;DKFZp686O15119;FLJ10358;GLS1;KIAA0838	glutaminase	0.89	1.13	1.37	0.9
CCPG1	CCPG1;CPR8;FLJ60574;KIAA1254	cell cycle progression 1	0.86	1.06	1.38	0.83
KRT77	KRT77;K1B;KRT1B;MGC148087	keratin 77	0.96	1.2	1.39	0.93
SEZ6L	SEZ6L	seizure related 6 homolog (mouse)-like	1.22	1.44	1.25	1.22
RCC2	RCC2;DKFZp762N0610;KIAA1470;TD-60	regulator of chromosome condensation 2	0.97	1.18	1.25	0.92
ZXDA	ZXDA;ZNF896	zinc finger, X-linked, duplicated A	0.95	1.15	1.25	0.83
MAT2B	MAT2B;MAT-II;MATIIbeta;MGC12237;Nbla02999;SDR23E1;TGR	methionine adenosyltransferase II, beta	0.95	1.12	1.25	0.88
USP7	USP7;HAUSP;TEF1	ubiquitin specific peptidase 7 (herpes virus-associated)	0.95	1.17	1.25	0.88
HDDC2	HDDC2;C6orf74;MGC87330;NS5ATP2;dJ167O5.2	HD domain containing 2	0.94	1.14	1.25	0.86
FBXL2	FBXL2;DKFZp564P0622;FBL2;FBL3	F-box and leucine-rich repeat protein 2	0.92	1.1	1.25	0.8
TTC27	TTC27;FLJ20272	tetratricopeptide repeat domain 27	0.92	1.11	1.25	0.84
ACSM4	ACSM4	acyl-CoA synthetase medium-chain family member 4	0.92	1.15	1.25	0.8
PDPK1	PDPK1;MGC20087;MGC35290;PDK1;PRO0461	3-phosphoinositide dependent protein kinase-1	0.9	1.08	1.25	0.98
ARL1	ARL1;ARFL1	ADP-ribosylation factor-like 1	0.89	1.09	1.25	0.77
TMED6	TMED6;MGC23911;PRO34237;SPLL9146	transmembrane emp24 protein transport domain containing 6	0.88	0.98	1.25	0.82
CCDC140	CCDC140;FLJ32447;MGC133159	coiled-coil domain containing 140	0.88	1.03	1.25	0.87
SLC25A35	SLC25A35;FLJ40217;MGC120446;MGC120448	solute carrier family 25, member 35	0.88	1.06	1.25	0.77
RPL41	RPL41;L41	ribosomal protein L41	0.88	1	1.25	0.79
RPRM	RPRM;FLJ90327;REPRIMO	reprimo, TP53 dependent G2 arrest mediator candidate	0.86	1.07	1.25	0.79
GABPA	GABPA;E4TF1-60;E4TF1A;NFT2;NRF2;NRF2A;RCH04A07	GA binding protein transcription factor, alpha subunit 60kDa	0.86	1.1	1.25	0.87
CFDP1	CFDP1;BCNT;BUCENTAUR;CENP-29;CP27;SWC5;Yeti;p97	craniofacial development protein 1	0.85	1.09	1.25	0.83
TMEM106A	TMEM106A;FLJ50420;FLJ50717;FLJ77644;MGC182077;MGC182126;MGC20235	transmembrane protein 106A	0.7	0.9	1.44	0.7
SKOR1	SKOR1;CORL1;FUSSEL15;LBXCOR1	SKI family transcriptional corepressor 1	0.7	0.9	1.44	0.7
UBR7	UBR7;C14orf130;FLJ10483;MGC9518	ubiquitin protein ligase E3 component n-recognin 7 (putative)	0.68	0.9	1.44	0.72
C17orf46	C17orf46;FLJ25414	chromosome 17 open reading frame 46	0.64	0.88	1.44	0.68
ACOXL	ACOXL;FLJ11042	acyl-CoA oxidase-like	0.64	0.86	1.44	0.68
MYNN	MYNN;OSZF;ZBTB31;ZNF902	myoneurin	0.72	0.95	1.46	0.79

PPP1R1A	PPP1R1A;I1;IPP1	protein phosphatase 1, regulatory (inhibitor) subunit 1A	0.62	0.82	1.46	0.62
CDRT15	CDRT15	CMT1A duplicated region transcript 15	0.77	1	1.47	0.79
PPP1R12A	PPP1R12A;M130;MBS;MGC133042;MYPT1	protein phosphatase 1, regulatory subunit 12A	0.65	0.88	1.47	0.66
TMEM9B	TMEM9B;C11orf15	TMEM9 domain family, member B	0.74	0.98	1.48	0.77
LYRM2	LYRM2;DJ122O8.2;FLJ18193;FLJ18317;FLJ21130;MGC104253	LYR motif containing 2	0.72	0.94	1.48	0.74
IGFALS	IGFALS;ALS	insulin-like growth factor binding protein, acid labile subunit	0.64	0.86	1.5	0.71
C1orf87	C1orf87;FLJ44073;MGC34837	chromosome 1 open reading frame 87	0.66	0.9	1.53	0.71
YIPF1	YIPF1;DJ167A19.1;FinGER1	Yip1 domain family, member 1	0.84	0.96	1.26	0.77
TMEM38A	TMEM38A;MGC3169;TRIC-A;TRICA	transmembrane protein 38A	0.84	0.95	1.26	0.79
TRIM54	TRIM54;MURF;MURF-3;RNF30;muRF3	tripartite motif containing 54	0.83	1.02	1.26	0.92
SFT2D1	SFT2D1;C6orf83;MGC19825;pRGR1	SFT2 domain containing 1	0.83	1.04	1.26	0.75
NOVA2	NOVA2;ANOVA;NOVA3	neuro-oncological ventral antigen 2	0.82	1.06	1.26	0.73
THOC7	THOC7;FLJ23445;NIF3L1BP1;fSAP24	THO complex 7 homolog (Drosophila)	0.82	0.98	1.26	0.79
CYGB	CYGB;HGB;STAP	cytoglobin	0.81	0.98	1.26	0.75
RFX2	RFX2;FLJ14226	regulatory factor X, 2 (influences HLA class II expression)	0.8	0.99	1.26	0.7
MAGEA8	MAGEA8;CT1.8;MAGE8;MGC2182	melanoma antigen family A, 8	0.8	0.96	1.26	0.73
NDUFB4	NDUFB4;B15;CI-B15;MGC5105	NADH dehydrogenase (ubiquinone) 1 beta subcomplex, 4, 15kDa	0.8	0.94	1.26	0.75
KLHDC3	KLHDC3;PEAS;RP1-20C7.3;dJ20C7.3;hPEAS	kelch domain containing 3	0.8	0.98	1.26	0.82
TAGAP	TAGAP;ARHGAP47;FLJ32631;FLJ39771;IDDM21;MGC133247;MGC27381;TAGAP1	T-cell activation RhoGTPase activating protein	0.78	0.96	1.26	0.81
PTPRT	PTPRT;KIAA0283;RPTPrho	protein tyrosine phosphatase, receptor type, T	0.78	0.9	1.26	0.68
POGZ	POGZ;KIAA0461;MGC71543;SUHW5;ZNF280E;ZNF635;ZNF635m	pogo transposable element with ZNF domain	0.78	0.96	1.26	0.83
ZNF638	ZNF638;DKFZp686P1231;MGC26130;MGC90196;NP220;ZFML;Zfp638	zinc finger protein 638	0.78	0.94	1.26	0.75
SLC25A46	SLC25A46	solute carrier family 25, member 46	0.78	0.95	1.26	0.73
SLC25A13	SLC25A13;ARALAR2;CITRIN;CTLN2	solute carrier family 25, member 13 (citrin)	0.78	0.97	1.26	0.75
MTOR	MTOR;FLJ44809;FRAP;FRAP1;FRAP2;RAFT1;RAPT1	mechanistic target of rapamycin (serine/threonine kinase)	0.78	0.98	1.26	0.81
ITGA11	ITGA11;HsT18964	integrin, alpha 11	0.77	0.96	1.26	0.71
EAPP	EAPP;BM036;C14orf11;FLJ20578;MGC4957	E2F-associated phosphoprotein	0.77	0.86	1.26	0.68
TTC30A	TTC30A;FLJ13946;FLJ77601	tetratricopeptide repeat domain 30A	0.76	0.92	1.26	0.7
NR2F1	NR2F1;COUP-TFI;EAR-3;EAR3;ERBAL3;NR2F2;SVP44;TCFCOUP1;TFCOUP1	nuclear receptor subfamily 2, group F, member 1	0.76	0.91	1.26	0.71
TARS	TARS;MGC9344;ThrRS	threonyl-tRNA synthetase	0.76	0.88	1.26	0.71
SMARCA5	SMARCA5;ISWI;SNF2H;WCRF135;hISWI;hSNF2H	SWI/SNF related, matrix associated, actin dependent regulator of chromatin, subfamily a, member 5	0.75	0.88	1.26	0.77
NEU4	NEU4;FLJ35928;MGC102757;MGC18222	sialidase 4	0.74	0.88	1.26	0.7
TSPAN3	TSPAN3;TM4-A;TM4SF8;TSPAN-3	tetraspanin 3	0.74	0.9	1.26	0.68
NEURL4	NEURL4;FLJ35680;KIAA1787	neuralized homolog 4 (Drosophila)	0.74	0.91	1.26	0.76
MITD1	MITD1	MIT, microtubule interacting and transport, domain containing 1	0.73	0.88	1.26	0.67
LCT	LCT;LAC;LPH;LPH1	lactase	0.72	0.89	1.26	0.65
DYNC1LI1	DYNC1LI1;DNCLI1;FLJ10219;LIC1	dynein, cytoplasmic 1, light intermediate chain 1	0.72	0.84	1.26	0.67
LAMP2	LAMP2;CD107b;LAMP-2;LAMPB;LGP110	lysosomal-associated membrane protein 2	0.72	0.9	1.26	0.66
ATP10A	ATP10A;ATP10C;ATPVA;ATPVC;KIAA0566	ATPase, class V, type 10A	0.71	0.84	1.26	0.67
OR52B2	OR52B2;OR11-70	olfactory receptor, family 52, subfamily B, member 2	0.7	0.91	1.26	0.62
ZNF582	ZNF582;FLJ30927	zinc finger protein 582	0.7	0.8	1.26	0.63
RASAL1	RASAL1;RASAL	RAS protein activator like 1 (GAP1 like)	0.7	0.86	1.26	0.67
DNTTIP1	DNTTIP1;C20orf167;Tdif1;dJ447F3.4	deoxynucleotidyltransferase, terminal, interacting protein 1	0.7	0.82	1.26	0.65
COPE	COPE;FLJ13241;epsilon-COP	coatomer protein complex, subunit epsilon	0.7	0.92	1.26	0.69
EID1	EID1;C15orf3;CRI1;EID-1;IRO45620;MGC138883;MGC138884;PTD014;RBP21	EP300 interacting inhibitor of differentiation 1	0.7	0.84	1.26	0.68
NADKD1	NADKD1;C5orf33;FLJ30596;MGC43298	NAD kinase domain containing 1	0.7	0.84	1.26	0.68
BPIFB1	BPIFB1;C20orf114;LPLUNC1;MGC14597	BPI fold containing family B, member 1	0.7	0.93	1.26	0.65
SNTB2	SNTB2;D16S2531E;EST25263;SNT2B2;SNT3;SNTL	syntrophin, beta 2 (dystrophin-associated protein A1, 59kDa, basic component 2)	0.69	0.78	1.26	0.65
MRPL17	MRPL17;L17mt;LIP2;MRP-L17;MRP-L26;RPL17L;RPML26	mitochondrial ribosomal protein L17	0.69	0.92	1.26	0.65
CLEC4C	CLEC4C;BDCA2;CD303;CLECSF11;CLECSF7;DLEC;HECL;MGC125789;MGC125791;MGC125792;MGC125793;PRO34150	C-type lectin domain family 4, member C	0.68	0.87	1.26	0.59
FAM26F	FAM26F;C6orf187;dJ93H18.5	family with sequence similarity 26, member F	0.68	0.92	1.26	0.7
IPO9	IPO9;DKFZp761M1547;FLJ10402;Imp9	importin 9	0.68	0.83	1.26	0.67
TOX3	TOX3;CAGF9;TNRC9	TOX high mobility group box family member 3	0.66	0.85	1.26	0.63
TMEM86B	TMEM86B;MGC30208	transmembrane protein 86B	0.66	0.8	1.26	0.61
PDS5B	PDS5B;APRIN;AS3;CG008;FLJ23236;KIAA0979	PDS5, regulator of cohesion maintenance, homolog B (S. cerevisiae)	0.66	0.83	1.26	0.56
FAM108A1	FAM108A1;C19orf27;MGC5244	family with sequence similarity 108, member A1	0.66	0.84	1.26	0.56
CNIH4	CNIH4	cornichon homolog 4 (Drosophila)	0.66	0.74	1.26	0.58
CCL4L2	CCL4L2;AT744.2;CCL4L;SCYA4L	chemokine (C-C motif) ligand 4-like 2	0.66	0.84	1.26	0.59
ANKRD43	ANKRD43	ankyrin repeat domain 43	0.65	0.78	1.26	0.63
SSH3	SSH3;FLJ10928;FLJ20515;FLJ42240;SSH3L	slingshot homolog 3 (Drosophila)	0.65	0.76	1.26	0.57
TEX2	TEX2;DKFZp781G0721;HT008;KIAA1738;TMEM96	testis expressed 2	0.65	0.84	1.26	0.63
CSE1L	CSE1L;CAS;CSE1;MGC117283;MGC130036;MGC130037;XPO2	CSE1 chromosome segregation 1-like (yeast)	0.65	0.76	1.26	0.6
OSTBETA	OSTBETA;FLJ26090;MGC118959;MGC118960;MGC118961;OSTB	organic solute transporter beta	0.65	0.8	1.26	0.57
EDA2R	EDA2R;EDA-A2R;EDAA2R;TNFRSF27;XEDAR	ectodysplasin A2 receptor	0.64	0.76	1.26	0.6
VCX	VCX;MGC118975;VCX-10r;VCX-B1;VCX1;VCX10R;VCXB1	variable charge, X-linked	0.64	0.84	1.26	0.63
FCRLB	FCRLB;FCRL2;FCRLM2;FCRLY;FLJ31052;FREB-2;FcRY;RP11-474I16.6	Fc receptor-like B	0.64	0.83	1.26	0.62
CHST8	CHST8;GALNAC4ST1;GalNAc4ST	carbohydrate (N-acetylgalactosamine 4-0) sulfotransferase 8	0.64	0.82	1.26	0.63
FAM117A	FAM117A	family with sequence similarity 117, member A	0.64	0.87	1.26	0.58
FGF4	FGF4;HBGF-4;HST;HST-1;HSTF1;K-FGF;KFGF	fibroblast growth factor 4	0.64	0.79	1.26	0.58
HN1L	HN1L;C16orf34;FLJ13092;KIAA1426;L11	hematological and neurological expressed 1-like	0.64	0.72	1.26	0.56
MAMSTR	MAMSTR;FLJ36070;MASTR;MGC117259	MEF2 activating motif and SAP domain containing transcriptional regulator	0.64	0.72	1.26	0.6
CXorf61	CXorf61;CT83;FLJ20611;FLJ22913;KK-LC-1;KKLC1	chromosome X open reading frame 61	0.62	0.8	1.26	0.6
CST5	CST5;MGC71922	cystatin D	0.62	0.77	1.26	0.64
CDC42EP2	CDC42EP2;BORG1;CEP2	CDC42 effector protein (Rho GTPase binding) 2	0.62	0.76	1.26	0.57
MTA3	MTA3;KIAA1266	metastasis associated 1 family, member 3	0.62	0.8	1.26	0.53
ADAT2	ADAT2;DEADC1;DKFZp686L1118;FLJ44213;TAD2;dJ20N2;dJ20N2.1	adenosine deaminase, tRNA-specific 2	0.62	0.77	1.26	0.54
SMAP1	SMAP1;FLJ13159;FLJ42245;SMAP-1	small ArfGAP 1	0.62	0.76	1.26	0.61
STRC	STRC;DFNB16;MGC156147	stereocilin	0.61	0.78	1.26	0.53
CRYM	CRYM;DFNA40;THBP	crystallin, mu	0.61	0.75	1.26	0.58
CLDN19	CLDN19;HOMG5	claudin 19	0.84	1.06	1.27	0.72
ANXA10	ANXA10;ANX14	annexin A10	0.83	1.03	1.27	0.77
SPAG4	SPAG4;CT127;SUN4	sperm associated antigen 4	0.83	1.03	1.27	0.86
CAMTA1	CAMTA1;KIAA0833	calmodulin binding transcription activator 1	0.83	1.07	1.27	0.85
FBXO36	FBXO36;FLJ37592;FLJ41090;Fbx36	F-box protein 36	0.82	1	1.27	0.72
ABTB1	ABTB1;BPOZ;BTB3;EF1ABP;MGC20585	ankyrin repeat and BTB (POZ) domain containing 1	0.82	1	1.27	0.78
FN1	FN1;CIG;DKFZp686F10164;DKFZp686H0342;DKFZp686I1370;DKFZp686O13149;ED-B;FINC;FN;FNZ;GFND;GFND2;LETS;MSF	fibronectin 1	0.82	1.02	1.27	0.75
PSTPIP2	PSTPIP2;MAYP;MGC34175	proline-serine-threonine phosphatase interacting protein 2	0.82	0.99	1.27	0.84
GRAMD4	GRAMD4;DIP;KIAA0767;MGC149847;MGC90497;dA59H18.1;dJ439F8.1	GRAM domain containing 4	0.82	0.94	1.27	0.73
CETN2	CETN2;CALT;CEN2	centrin, EF-hand protein, 2	0.82	1.03	1.27	0.86
ADARB2	ADARB2;ADAR3;FLJ25034;FLJ36975;FLJ41340;RED2	adenosine deaminase, RNA-specific, B2	0.81	1.05	1.27	0.8
SLC25A34	SLC25A34;DKFZp781A10161;RP11-169K16.2	solute carrier family 25, member 34	0.8	0.97	1.27	0.82
BMF	BMF;FLJ00065	Bcl2 modifying factor	0.8	0.93	1.27	0.74
RXFP3	RXFP3;GPCR135;MGC141998;MGC142000;RLN3R1;RXFPR3;SALPR	relaxin/insulin-like family peptide receptor 3	0.8	0.93	1.27	0.7
WDR60	WDR60;FLJ10300;FLJ23575	WD repeat domain 60	0.8	0.9	1.27	0.7
ZBED3	ZBED3;MGC15435	zinc finger, BED-type containing 3	0.8	1.01	1.27	0.7
BTNL8	BTNL8;FLJ21458;FLJ51528;MGC149619	butyrophilin-like 8	0.79	1	1.27	0.7
C16orf70	C16orf70;C16orf6;FLJ12076;LIN10;lin-10	chromosome 16 open reading frame 70	0.78	1.02	1.27	0.74
UBXN6	UBXN6;DKFZp667D109;FLJ00394;UBXD1;UBXDC2	UBX domain protein 6	0.78	1.01	1.27	0.8
GM2A	GM2A;GM2-AP;SAP-3	GM2 ganglioside activator	0.78	0.94	1.27	0.74
KIAA2022	KIAA2022	KIAA2022	0.77	0.93	1.27	0.79
PPM1H	PPM1H;ARHCL1;FLJ13253;KIAA1157;NERPP-2C;URCC2	protein phosphatase, Mg2+/Mn2+ dependent, 1H	0.77	0.98	1.27	0.76
NDUFV3	NDUFV3;CI-10k;CI-9KD	NADH dehydrogenase (ubiquinone) flavoprotein 3, 10kDa	0.77	1	1.27	0.76
TACR2	TACR2;NK2R;NKNAR;SKR;TAC2R	tachykinin receptor 2	0.76	0.88	1.27	0.66
GALT	GALT	galactose-1-phosphate uridylyltransferase	0.76	0.98	1.27	0.65
LRIG2	LRIG2;DKFZp451C181;KIAA0806;LIG-2;LIG2	leucine-rich repeats and immunoglobulin-like domains 2	0.76	0.86	1.27	0.68
AFMID	AFMID;DKFZp686F03259;KF;MGC167063	arylformamidase	0.76	0.97	1.27	0.67
MSL2	MSL2;FLJ10546;FLJ54913;KIAA1585;MSL-2;MSL2L1;RNF184	male-specific lethal 2 homolog (Drosophila)	0.76	0.96	1.27	0.86
XKR5	XKR5;HARL2754;UNQ2754;XRG5A;XRG5BM	XK, Kell blood group complex subunit-related family, member 5	0.74	0.9	1.27	0.73
C9orf125	C9orf125;MGC12992	chromosome 9 open reading frame 125	0.74	0.9	1.27	0.75
H2AFB2	H2AFB2;H2A.Bbd;H2AFB3	H2A histone family, member B2	0.74	0.85	1.27	0.67
ANKRD34B	ANKRD34B;DP58	ankyrin repeat domain 34B	0.73	0.9	1.27	0.67
CCNG1	CCNG1;CCNG	cyclin G1	0.73	0.86	1.27	0.76
VSTM2A	VSTM2A;MGC33530;VSTM2	V-set and transmembrane domain containing 2A	0.72	0.94	1.27	0.68
MURC	MURC;CAVIN4;cavin-4	muscle-related coiled-coil protein	0.72	0.9	1.27	0.74
USP38	USP38;FLJ35970;HP43.8KD;KIAA1891	ubiquitin specific peptidase 38	0.72	0.84	1.27	0.63
ZNF259	ZNF259;MGC110983;ZPR1	zinc finger protein 259	0.72	0.9	1.27	0.74
ENSA	ENSA;ARPP-19e;MGC4319;MGC78563;MGC8394	endosulfine alpha	0.71	0.92	1.27	0.66
LCN6	LCN6;LCN5;UNQ643;hLcn5	lipocalin 6	0.7	0.8	1.27	0.63
IMPG2	IMPG2;IPM200;RP56;SPACRCAN	interphotoreceptor matrix proteoglycan 2	0.69	0.84	1.27	0.68
TREML4	TREML4;MGC126829;TLT4	triggering receptor expressed on myeloid cells-like 4	0.69	0.92	1.27	0.66
ZDHHC1	ZDHHC1;C16orf1;HSU90653;ZNF377	zinc finger, DHHC-type containing 1	0.68	0.76	1.27	0.61
C1QL4	C1QL4;C1QTNF11;MGC131708	complement component 1, q subcomponent-like 4	0.68	0.83	1.27	0.63
GLE1	GLE1;GLE1L;LCCS;LCCS1;hGLE1	GLE1 RNA export mediator homolog (yeast)	0.68	0.84	1.27	0.63
CTDNEP1	CTDNEP1;DULLARD;HSA011916;NET56	CTD nuclear envelope phosphatase 1	0.68	0.9	1.27	0.6
CWC27	CWC27;NY-CO-10;SDCCAG10	CWC27 spliceosome-associated protein homolog (S. cerevisiae)	0.68	0.82	1.27	0.59
GHRL	GHRL;MTLRP	ghrelin/obestatin prepropeptide	0.67	0.83	1.27	0.69
ENTPD4	ENTPD4;KIAA0392;LALP70;LAP70;LYSAL1;NTPDase-4;UDPase	ectonucleoside triphosphate diphosphohydrolase 4	0.67	0.82	1.27	0.6
YWHAE	YWHAE;14-3-3E;FLJ45465;FLJ53559;KCIP-1;MDCR;MDS	tyrosine 3-monooxygenase/tryptophan 5-monooxygenase activation protein, epsilon polypeptide	0.67	0.84	1.27	0.63
TUBA4A	TUBA4A;FLJ30169;H2-ALPHA;TUBA1	tubulin, alpha 4a	0.66	0.83	1.27	0.58

ZNF771	ZNF771;DSC43	zinc finger protein 771	0.65	0.79	1.27	0.61
CT45A2	CT45A2;CT45-2;CT45.2	cancer/testis antigen family 45, member A2	0.65	0.88	1.27	0.58
FTMT	FTMT;MTF	ferritin mitochondrial	0.64	0.82	1.27	0.59
PKIA	PKIA;PRKACN1	protein kinase (cAMP-dependent, catalytic) inhibitor alpha	0.64	0.88	1.27	0.63
HES7	HES7;SCDO4;bHLHb37	hairy and enhancer of split 7 (Drosophila)	0.64	0.75	1.27	0.6
STRN3	STRN3;SG2NA	striatin, calmodulin binding protein 3	0.64	0.81	1.27	0.55
AKIRIN1	AKIRIN1;C1orf108;FLJ12666;RP11-781D11.2;STRF2	akirin 1	0.64	0.86	1.27	0.62
EXOSC10	EXOSC10;PM-Scl;PM/Scl-100;PMSCL;PMSCL2;RRP6;Rrp6p;p2;p3;p4	exosome component 10	0.64	0.79	1.27	0.71
VPS26A	VPS26A;FLJ12930;HB58;Hbeta58;PEP8A;VPS26	vacuolar protein sorting 26 homolog A (S. pombe)	0.64	0.74	1.27	0.59
PLSCR1	PLSCR1;MMTRA1B	phospholipid scramblase 1	0.63	0.76	1.27	0.55
RHOJ	RHOJ;ARHJ;FLJ14445;MGC34777;RASL7B;TC10B;TCL	ras homolog gene family, member J	0.62	0.86	1.27	0.64
FLJ43860	FLJ43860	FLJ43860 protein	0.62	0.82	1.27	0.62
ZNF713	ZNF713;FLJ39963	zinc finger protein 713	0.62	0.82	1.27	0.59
DOK1	DOK1;MGC117395;MGC138860;P62DOK	docking protein 1, 62kDa (downstream of tyrosine kinase 1)	0.62	0.8	1.27	0.54
C7orf55	C7orf55;FLJ35699;FMC1	chromosome 7 open reading frame 55	0.62	0.76	1.27	0.58
FAM49A	FAM49A;DKFZp566A1524;FLJ11080;FLJ33961	family with sequence similarity 49, member A	0.62	0.75	1.27	0.61
EP400	EP400;CAGH32;DKFZP434I225;FLJ42018;FLJ45115;P400;TNRC12	E1A binding protein p400	0.62	0.8	1.27	0.54
OR10H3	OR10H3;MGC119147;MGC119149	olfactory receptor, family 10, subfamily H, member 3	0.61	0.8	1.27	0.61
METRNL	METRNL;MGC99788	meteorin, glial cell differentiation regulator-like	0.61	0.8	1.27	0.59
ETS1	ETS1;ETS-1;EWSR2;FLJ10768	v-ets erythroblastosis virus E26 oncogene homolog 1 (avian)	0.61	0.74	1.27	0.58
CTAGE6P	CTAGE6P;CTAGE6;MGC41943	CTAGE family, member 6, pseudogene	0.61	0.75	1.27	0.57
PNISR	PNISR;C6orf111;DKFZp564B0769;FLJ14752;FLJ14853;FLJ14992;FLJ90147;HSPC306;MGC104269;RP11-98I9.2;SFRS18;SRrp130;bA98I9.2	PNN-interacting serine/arginine-rich protein	0.61	0.76	1.27	0.61
GUCA1B	GUCA1B;DKFZp686E1183;GCAP2;GUCA2;RP48	guanylate cyclase activator 1B (retina)	0.84	1.05	1.28	0.9
SH3PXD2B	SH3PXD2B;FAD49;FLJ20831;FTHS;HOFI;KIAA1295;TKS4;TSK4	SH3 and PX domains 2B	0.84	0.97	1.28	0.76
CD1E	CD1E;CD1A;FLJ17609;R2	CD1e molecule	0.82	0.99	1.28	0.84
ZNF654	ZNF654;FLJ10997;FLJ21142	zinc finger protein 654	0.82	1.03	1.28	0.74
ASS1	ASS1;ASS;CTLN1	argininosuccinate synthase 1	0.82	0.98	1.28	0.87
SPESP1	SPESP1;ESP;MGC24663;SP-ESP	sperm equatorial segment protein 1	0.81	0.98	1.28	0.83
PLVAP	PLVAP;FELS;PV-1;PV1;gp68	plasmalemma vesicle associated protein	0.81	0.94	1.28	0.75
TAF1C	TAF1C;MGC:39976;SL1;TAFI110;TAFI95	TATA box binding protein (TBP)-associated factor, RNA polymerase I, C, 110kDa	0.8	1.04	1.28	0.7
HIST1H3E	HIST1H3E;H3.1;H3/d;H3FD	histone cluster 1, H3e	0.79	0.96	1.28	0.7
GHSR	GHSR	growth hormone secretagogue receptor	0.79	0.98	1.28	0.89
ADAMTSL3	ADAMTSL3;KIAA1233;MGC150716;MGC150717	ADAMTS-like 3	0.78	0.97	1.28	0.78
SORCS2	SORCS2	sortilin-related VPS10 domain containing receptor 2	0.78	0.97	1.28	0.87
ZNF449	ZNF449;FLJ23614;ZSCAN19	zinc finger protein 449	0.77	0.96	1.28	0.72
CD3G	CD3G;CD3-GAMMA;FLJ17620;FLJ17664;FLJ79544;FLJ94613;MGC138597;T3G	CD3g molecule, gamma (CD3-TCR complex)	0.76	0.94	1.28	0.68
STC1	STC1;STC	stanniocalcin 1	0.76	0.95	1.28	0.72
BTF3L4	BTF3L4;MGC23908;MGC88389	basic transcription factor 3-like 4	0.76	0.92	1.28	0.74
AP2B1	AP2B1;ADTB2;AP105B;AP2-BETA;CLAPB1;DKFZp781K0743	adaptor-related protein complex 2, beta 1 subunit	0.76	0.98	1.28	0.76
ACTL8	ACTL8;CT57	actin-like 8	0.75	0.9	1.28	0.73
ZNF320	ZNF320;DKFZp686G16228;ZFPL	zinc finger protein 320	0.74	0.92	1.28	0.7
NR2E3	NR2E3;ESCS;MGC49976;PNR;RNR;RP37;rd7	nuclear receptor subfamily 2, group E, member 3	0.74	0.88	1.28	0.77
MRVI1	MRVI1;IRAG;JAW1L	murine retrovirus integration site 1 homolog	0.74	0.94	1.28	0.64
ITFG3	ITFG3;C16orf9;DKFZp761D0211;FLJ32603;gs19	integrin alpha FG-GAP repeat containing 3	0.73	0.88	1.28	0.71
KRIT1	KRIT1;CAM;CCM1	KRIT1, ankyrin repeat containing	0.73	0.84	1.28	0.66
DPCD	DPCD;DKFZp566F084	deleted in primary ciliary dyskinesia homolog (mouse)	0.73	0.9	1.28	0.69
KIAA1456	KIAA1456;C8orf79;FLJ36980;MGC43113	KIAA1456	0.73	0.91	1.28	0.72
TMEM74	TMEM74;FLJ30668;NET36	transmembrane protein 74	0.71	0.87	1.28	0.66
WFDC13	WFDC13;C20orf138;WAP13;dJ601O1.3	WAP four-disulfide core domain 13	0.7	0.81	1.28	0.67
LMAN1L	LMAN1L;ERGIC-53L;ERGL	lectin, mannose-binding, 1 like	0.7	0.92	1.28	0.7
FRS3	FRS3;FRS2B;FRS2beta;MGC17167;SNT-2;SNT2	fibroblast growth factor receptor substrate 3	0.7	0.92	1.28	0.7
FUT4	FUT4;CD15;ELFT;FCT3A;FUC-TIV;FUTIV;LeX;SSEA-1	fucosyltransferase 4 (alpha (1,3) fucosyltransferase, myeloid-specific)	0.7	0.88	1.28	0.62
PPP1R21	PPP1R21;CCDC128;FLJ16566;KLRAQ1;MGC111781	protein phosphatase 1, regulatory subunit 21	0.69	0.88	1.28	0.64
TTC33	TTC33;OSRF	tetratricopeptide repeat domain 33	0.68	0.78	1.28	0.61
MFF	MFF;C2orf33;DKFZp666J168;GL004;MGC110913	mitochondrial fission factor	0.68	0.9	1.28	0.67
OGT	OGT;FLJ23071;HRNT1;MGC22921;O-GLCNAC	O-linked N-acetylglucosamine (GlcNAc) transferase (UDP-N-acetylglucosamine:polypeptide-N-acetylglucosaminyl transferase)	0.68	0.84	1.28	0.59
TMEM198	TMEM198;FLJ41180;MGC99813	transmembrane protein 198	0.67	0.86	1.28	0.59
DRG2	DRG2	developmentally regulated GTP binding protein 2	0.67	0.83	1.28	0.58
RASGRP1	RASGRP1;CALDAG-GEFI;CALDAG-GEFII;MGC129998;MGC129999;RASGRP;V;hRasGRP1	RAS guanyl releasing protein 1 (calcium and DAG-regulated)	0.66	0.86	1.28	0.57
TCTE1	TCTE1;D6S46;FLJ50041;MGC33600	t-complex-associated-testis-expressed 1	0.66	0.81	1.28	0.61
PTHLH	PTHLH;BDE2;HHM;MGC14611;PLP;PTHR;PTHRP	parathyroid hormone-like hormone	0.66	0.78	1.28	0.61
ALS2CL	ALS2CL;DKFZp686P238;MGC129698;RN49018	ALS2 C-terminal like	0.66	0.88	1.28	0.65
TMEM167B	TMEM167B;C1orf119;FLJ90710	transmembrane protein 167B	0.66	0.8	1.28	0.62
FAM194A	FAM194A;C3orf44;MGC39662	family with sequence similarity 194, member A	0.66	0.82	1.28	0.62
CORT	CORT;CST-14;CST-17;CST-29	cortistatin	0.66	0.82	1.28	0.62
FCHO1	FCHO1;KIAA0290	FCH domain only 1	0.64	0.78	1.28	0.57
FOXP1	FOXP1;12CC4;FLJ23741;MGC12942;MGC88572;MGC99551;QRF1;hFKH1B	forkhead box P1	0.64	0.76	1.28	0.68
PRRG2	PRRG2;PRGP2	proline rich Gla (G-carboxyglutamic acid) 2	0.63	0.8	1.28	0.56
ULBP3	ULBP3;RAET1N	UL16 binding protein 3	0.63	0.82	1.28	0.55
DHRS12	DHRS12;FLJ13639;SDR40C1	dehydrogenase/reductase (SDR family) member 12	0.62	0.8	1.28	0.57
BMP7	BMP7;OP-1	bone morphogenetic protein 7	0.62	0.73	1.28	0.58
IDH3B	IDH3B;FLJ11043;H-IDHB;MGC903;RP46	isocitrate dehydrogenase 3 (NAD+) beta	0.62	0.86	1.28	0.64
GPR64	GPR64;EDDM6;FLJ00282;HE6;MGC104454;MGC138738;MGC138739;TM7LN2	G protein-coupled receptor 64	0.61	0.72	1.28	0.55
C16orf53	C16orf53;FLJ22459;GAS;MGC4606;PA1	chromosome 16 open reading frame 53	0.84	1.04	1.29	0.8
CRISPLD2	CRISPLD2;CRISP11;DKFZp434B044;LCRISP2;MGC74865	cysteine-rich secretory protein LCCL domain containing 2	0.82	1.06	1.29	0.72
ZNF264	ZNF264	zinc finger protein 264	0.82	1.01	1.29	0.77
CELA1	CELA1;ELA1	chymotrypsin-like elastase family, member 1	0.82	1.04	1.29	0.79
CELA2B	CELA2B;ELA2B;MGC142100;MGC142102;MGC97052	chymotrypsin-like elastase family, member 2B	0.81	0.96	1.29	0.72
DES	DES;CMD1I;CSM1;CSM2;FLJ12025;FLJ39719;FLJ41013;FLJ41793	desmin	0.8	1.03	1.29	0.72
TMPRSS3	TMPRSS3;DFNB10;DFNB8;ECHOS1;TADG12	transmembrane protease, serine 3	0.8	1.02	1.29	0.76
HINT2	HINT2;HIT-17	histidine triad nucleotide binding protein 2	0.8	0.96	1.29	0.82
RRAGD	RRAGD;DKFZp761H171;FLJ44176;RAGD;bA11D8.2.1	Ras-related GTP binding D	0.8	1.02	1.29	0.79
PIK3CG	PIK3CG;PI3CG;PI3K;PI3Kgamma;PIK3	phosphoinositide-3-kinase, catalytic, gamma polypeptide	0.79	1.01	1.29	0.84
CUEDC1	CUEDC1;DKFZp547L163;FLJ20739	CUE domain containing 1	0.79	1	1.29	0.83
WDR75	WDR75;DKFZp781N1244;FLJ12519;NET16;UTP17	WD repeat domain 75	0.79	0.97	1.29	0.78
LRAT	LRAT;LCA14;MGC33103	lecithin retinol acyltransferase (phosphatidylcholine--retinol O-acyltransferase)	0.78	0.94	1.29	0.83
FAM129C	FAM129C;BCNP1;FLJ39802	family with sequence similarity 129, member C	0.78	0.88	1.29	0.7
FOLR3	FOLR3;FR-G;FR-gamma;gamma-hFR	folate receptor 3 (gamma)	0.78	0.91	1.29	0.73
FCHO2	FCHO2	FCH domain only 2	0.78	0.9	1.29	0.7
IFNGR1	IFNGR1;CD119;FLJ45734;IFNGR	interferon gamma receptor 1	0.78	0.94	1.29	0.83
PRMT1	PRMT1;ANM1;HCP1;HRMT1L2;IR1B4	protein arginine methyltransferase 1	0.78	0.98	1.29	0.72
ABI1	ABI1;ABI-1;ABLBP4;E3B1;NAP1BP;SSH3BP;SSH3BP1	abl-interactor 1	0.77	0.89	1.29	0.71
FETUB	FETUB;16G2;Gugu;IRL685	fetuin B	0.76	0.97	1.29	0.8
OR7D4	OR7D4;OR19-7;OR19-B;OR19B;OR7D4P;hg105	olfactory receptor, family 7, subfamily D, member 4	0.76	0.96	1.29	0.75
ATP6V1G1	ATP6V1G1;ATP6G;ATP6G1;ATP6GL;ATP6J;DKFZp547P234;Vma10	ATPase, H+ transporting, lysosomal 13kDa, V1 subunit G1	0.75	0.94	1.29	0.8
MAGEB1	MAGEB1;CT3.1;DAM10;MAGE-Xp;MAGEL1;MGC9322	melanoma antigen family B, 1	0.74	0.97	1.29	0.74
NFYB	NFYB;CBF-A;CBF-B;HAP3;NF-YB	nuclear transcription factor Y, beta	0.74	0.94	1.29	0.66
CWC15	CWC15;C11orf5;Cwf15;HSPC148;ORF5	CWC15 spliceosome-associated protein homolog (S. cerevisiae)	0.74	0.92	1.29	0.72
CLTA	CLTA;LCA	clathrin, light chain A	0.74	0.9	1.29	0.76
DCAF13	DCAF13;DKFZp564O0463;GM83;MGC126859;MGC138247;WDSOF1	DDB1 and CUL4 associated factor 13	0.74	0.92	1.29	0.7
MAVS	MAVS;CARDIF;DKFZp547C224;DKFZp666M015;FLJ27482;FLJ31698;FLJ35386;FLJ38051;FLJ41962;IPS-1;IPS1;KIAA1271;MGC3260;VISA	mitochondrial antiviral signaling protein	0.74	0.92	1.29	0.69
PPIL3	PPIL3;CYPJ	peptidylprolyl isomerase (cyclophilin)-like 3	0.73	0.92	1.29	0.72
MFGE8	MFGE8;BA46;EDIL1;HMFG;HsT19888;MFG-E8;MFGM;OAcGD3S;SED1;SPAG10;hP47	milk fat globule-EGF factor 8 protein	0.73	0.94	1.29	0.69
GRM6	GRM6;CSNB1B;DKFZp686H1993;GPRC1F;MGLUR6;mGlu6	glutamate receptor, metabotropic 6	0.72	0.89	1.29	0.8
CD1D	CD1D;CD1A;MGC34622;R3	CD1d molecule	0.72	0.9	1.29	0.68
WDR88	WDR88;PQWD	WD repeat domain 88	0.7	0.88	1.29	0.66
ZNF740	ZNF740;MGC61706;Zfp740	zinc finger protein 740	0.7	0.82	1.29	0.64
PYCR1	PYCR1;ARCL2B;P5C;P5CR;PIG45;PP222;PRO3;PYCR	pyrroline-5-carboxylate reductase 1	0.7	0.9	1.29	0.61
C1orf27	C1orf27;FLJ20505;ODR4;TTG1;odr-4	chromosome 1 open reading frame 27	0.7	0.88	1.29	0.66
HTATSF1	HTATSF1;TAT-SF1;dJ196E23.2	HIV-1 Tat specific factor 1	0.7	0.94	1.29	0.63
C8orf34	C8orf34;DKFZp547E186;FLJ36872;FLJ37331;VEST-1;VEST1	chromosome 8 open reading frame 34	0.69	0.88	1.29	0.65
TRIO	TRIO;ARHGEF23;FLJ42780;tgat	triple functional domain (PTPRF interacting)	0.69	0.87	1.29	0.72
TPPP2	TPPP2;C14orf8;P18;p25beta	tubulin polymerization-promoting protein family member 2	0.68	0.87	1.29	0.63
KIAA1958	KIAA1958;FLJ39294;MGC142075	KIAA1958	0.68	0.92	1.29	0.61
SPON2	SPON2;DIL-1;DIL1;DKFZp686G21139;FLJ16313;FLJ22401;FLJ34460;M-SPONDIN;MINDIN	spondin 2, extracellular matrix protein	0.67	0.9	1.29	0.67
OR2T27	OR2T27	olfactory receptor, family 2, subfamily T, member 27	0.66	0.76	1.29	0.61
ZBTB47	ZBTB47;DKFZp434N0615;KIAA1190;ZNF651	zinc finger and BTB domain containing 47	0.66	0.86	1.29	0.65
ZBTB3	ZBTB3;FLJ23392	zinc finger and BTB domain containing 3	0.66	0.89	1.29	0.72
PITPNB	PITPNB;PI-TP-beta;PtdInsTP;VIB1B	phosphatidylinositol transfer protein, beta	0.66	0.9	1.29	0.6
BPHL	BPHL;BPH-RP;MCNAA;MGC125930;MGC41865;VACVASE	biphenyl hydrolase-like (serine hydrolase)	0.65	0.78	1.29	0.69
APOBEC3G	APOBEC3G;ARCD;ARP-9;ARP9;CEM-15;CEM15;FLJ12740;bK150C2.7;dJ494G10.1	apolipoprotein B mRNA editing enzyme, catalytic polypeptide-like 3G	0.64	0.76	1.29	0.68
ADAM32	ADAM32;FLJ26299;FLJ29004	ADAM metallopeptidase domain 32	0.64	0.82	1.29	0.61
PROK2	PROK2;BV8;KAL4;MIT1;PK2	prokineticin 2	0.64	0.8	1.29	0.62
SOCS1	SOCS1;CIS1;CISH1;JAB;SOCS-1;SSI-1;SSI1;TIP3	suppressor of cytokine signaling 1	0.64	0.78	1.29	0.56
HIST1H4F	HIST1H4F;H4;H4/c;H4FC	histone cluster 1, H4f	0.64	0.83	1.29	0.56
KLHL28	KLHL28;BTBD5;FLJ20081	kelch-like 28 (Drosophila)	0.64	0.87	1.29	0.62
ZNF584	ZNF584;FLJ39899	zinc finger protein 584	0.64	0.81	1.29	0.65
RBM15	RBM15;FLJ12479;FLJ21943;MGC119584;OTT;OTT1;SPEN	RNA binding motif protein 15	0.64	0.81	1.29	0.62
PLIN2	PLIN2;ADFP;ADRP;MGC10598	perilipin 2	0.64	0.74	1.29	0.59

FAAH2	FAAH2;AMDD;FLJ31204	fatty acid amide hydrolase 2	0.63	0.8	1.29	0.62
ZNF461	ZNF461;GIOT-1;GIOT1;MGC33911	zinc finger protein 461	0.62	0.82	1.29	0.61
ZBTB12	ZBTB12;Bat9;C6orf46;D6S59E;G10;NG35	zinc finger and BTB domain containing 12	0.62	0.81	1.29	0.58
MAP3K7	MAP3K7;MEKK7;TAK1;TGF1a	mitogen-activated protein kinase kinase kinase 7	0.62	0.8	1.29	0.66
CD244	CD244;2B4;NAIL;NKR2B4;Nmrk;SLAMF4	CD244 molecule, natural killer cell receptor 2B4	0.61	0.8	1.29	0.53
BBX	BBX;HBP2;HSPC339;MDS001	bobby sox homolog (Drosophila)	0.61	0.82	1.29	0.56
SERPINB2	SERPINB2;HsT1201;PAI;PAI-2;PAI2;PLANH2	serpin peptidase inhibitor, clade B (ovalbumin), member 2	0.84	1.08	1.3	0.81
GIMAP6	GIMAP6;DKFZp686A01175;FLJ22690;IAN-2;IAN-6;IAN2;IAN6	GTPase, IMAP family member 6	0.83	1.04	1.3	0.75
OR2T35	OR2T35	olfactory receptor, family 2, subfamily T, member 35	0.83	1.04	1.3	0.84
PM20D2	PM20D2;ACY1L2;bA63L7.3	peptidase M20 domain containing 2	0.82	1.05	1.3	0.87
KCNA1	KCNA1;AEMK;EA1;HBK1;HUK1;KV1.1;MBK1;MGC126782;MGC138385;MK1;RBK1	potassium voltage-gated channel, shaker-related subfamily, member 1 (episodic ataxia with myokymia)	0.8	1.04	1.3	0.81
FLRT1	FLRT1;MGC21624	fibronectin leucine rich transmembrane protein 1	0.8	1.02	1.3	0.85
LPAR2	LPAR2;EDG-4;EDG4;FLJ93869;LPA-2;LPA2	lysophosphatidic acid receptor 2	0.8	1.01	1.3	0.91
CTNNAL1	CTNNAL1;CLLP;FLJ08121;alpha-CATU	catenin (cadherin-associated protein), alpha-like 1	0.8	1.01	1.3	0.73
SMN1	SMN1;BCD541;SMA;SMA1;SMA2;SMA3;SMA4;SMA@;SMN;SMN2;SMNT;T-BCD541	survival of motor neuron 1, telomeric	0.8	1.02	1.3	0.77
MRPS34	MRPS34;MGC2616;MRP-S12;MRP-S34;MRPS12	mitochondrial ribosomal protein S34	0.8	1	1.3	0.77
SPCS1	SPCS1;SPC1;SPC12;YJR010C-A	signal peptidase complex subunit 1 homolog (S. cerevisiae)	0.8	0.96	1.3	0.72
KCNB1	KCNB1;DRK1;KV2.1;h-DRK1	potassium voltage-gated channel, Shab-related subfamily, member 1	0.79	1.01	1.3	0.78
HDAC11	HDAC11;FLJ22237;HD11	histone deacetylase 11	0.78	1.02	1.3	0.79
BAD	BAD;BBC2;BCL2L8	BCL2-associated agonist of cell death	0.78	1	1.3	0.77
PREPL	PREPL;FLJ16627;KIAA0436	prolyl endopeptidase-like	0.78	0.94	1.3	0.83
ANPEP	ANPEP;APN;CD13;GP150;LAP1;P150;PEPN	alanyl (membrane) aminopeptidase	0.77	0.98	1.3	0.74
POU2F1	POU2F1;FLJ42836;OCT1;OTF1;oct-1B	POU class 2 homeobox 1	0.76	0.94	1.3	0.7
CNBP	CNBP;CNBP1;DM2;FLJ11631;PROMM;RNF163;ZCCHC22;ZNF9	CCHC-type zinc finger, nucleic acid binding protein	0.76	0.96	1.3	0.83
FADS6	FADS6	fatty acid desaturase domain family, member 6	0.75	0.94	1.3	0.81
DEFA3	DEFA3;DEF3;HNP-3;HNP3;HP-3;HP3	defensin, alpha 3, neutrophil-specific	0.74	0.91	1.3	0.77
C1orf95	C1orf95;DKFZp761P211	chromosome 1 open reading frame 95	0.74	0.92	1.3	0.69
BCAM	BCAM;AU;CD239;LU;MSK19	basal cell adhesion molecule (Lutheran blood group)	0.74	0.9	1.3	0.72
HEMK1	HEMK1;FLJ22320;HEMK;MTQ1	HemK methyltransferase family member 1	0.72	0.86	1.3	0.76
MRPS23	MRPS23;HSPC329;MRP-S23	mitochondrial ribosomal protein S23	0.72	0.9	1.3	0.65
DUS4L	DUS4L;DUS4;MGC133233;PP35	dihydrouridine synthase 4-like (S. cerevisiae)	0.72	0.89	1.3	0.69
ECSCR	ECSCR;ARIA;ECSM2	endothelial cell-specific chemotaxis regulator	0.72	0.85	1.3	0.71
TMEM145	TMEM145;FLJ90805	transmembrane protein 145	0.71	0.92	1.3	0.66
PREX1	PREX1;KIAA1415;P-REX1	phosphatidylinositol-3,4,5-trisphosphate-dependent Rac exchange factor 1	0.7	0.87	1.3	0.67
MDC1	MDC1;DKFZp781A0122;KIAA0170;MGC166888;NFBD1	mediator of DNA-damage checkpoint 1	0.7	0.89	1.3	0.7
PTGS1	PTGS1;COX1;COX3;PCOX1;PGG/HS;PGHS-1;PGHS1;PHS1;PTGHS	prostaglandin-endoperoxide synthase 1 (prostaglandin G/H synthase and cyclooxygenase)	0.7	0.88	1.3	0.63
GPR123	GPR123;FLJ25875;FLJ37356	G protein-coupled receptor 123	0.68	0.84	1.3	0.63
ZNF669	ZNF669;FLJ12606	zinc finger protein 669	0.68	0.86	1.3	0.65
EIF5	EIF5;EIF-5A	eukaryotic translation initiation factor 5	0.68	0.89	1.3	0.69
C8orf85	C8orf85;AARD	chromosome 8 open reading frame 85	0.68	0.92	1.3	0.61
C5orf46	C5orf46;MGC23985	chromosome 5 open reading frame 46	0.66	0.78	1.3	0.62
PRDM16	PRDM16;KIAA1675;MEL1;MGC166915;PFM13	PR domain containing 16	0.66	0.86	1.3	0.69
CFB	CFB;AHUS4;BF;BFD;CFAB;FB;FBI12;FLJ54899;GBG;H2-Bf;PBF2	complement factor B	0.66	0.85	1.3	0.64
TPD52L2	TPD52L2;D54;DKFZp686A1765	tumor protein D52-like 2	0.66	0.89	1.3	0.59
ZFP91	ZFP91;DMS-8;DSM-8;FLJ57065;PZF;ZFP-91;ZNF757	zinc finger protein 91 homolog (mouse)	0.66	0.84	1.3	0.63
CDX1	CDX1;MGC116915	caudal type homeobox 1	0.64	0.84	1.3	0.56
BHLHB9	BHLHB9;KIAA1701;p60TRP	basic helix-loop-helix domain containing, class B, 9	0.64	0.85	1.3	0.64
ICAM3	ICAM3;CD50;CDW50;ICAM-R	intercellular adhesion molecule 3	0.64	0.8	1.3	0.64
ZMYND8	ZMYND8;MGC31836;PRKCBP1;PRO2893;RACK7	zinc finger, MYND-type containing 8	0.64	0.82	1.3	0.6
STX17	STX17;FLJ20651;MGC102796;MGC126613;MGC126615	syntaxin 17	0.64	0.88	1.3	0.65
SAFB	SAFB;DKFZp779C1727;HAP;HET;SAF-B1;SAFB1	scaffold attachment factor B	0.64	0.88	1.3	0.58
ZMAT3	ZMAT3;FLJ12296;FLJ31683;MGC10613;PAG608;WIG-1;WIG1	zinc finger, matrin-type 3	0.63	0.8	1.3	0.61
C18orf10	C18orf10;DKFZp586M1523;HsT3006;L17;PGs2	chromosome 18 open reading frame 10	0.62	0.76	1.3	0.64
ATP1B2	ATP1B2;AMOG	ATPase, Na+/K+ transporting, beta 2 polypeptide	0.61	0.8	1.3	0.62
OPALIN	OPALIN;HTMP10;TMEM10;TMP10	oligodendrocytic myelin paranodal and inner loop protein	0.83	1.06	1.31	0.76
MED24	MED24;ARC100;CRSP100;CRSP4;DRIP100;KIAA0130;MGC8748;THRAP4;TRAP100	mediator complex subunit 24	0.83	1	1.31	0.88
ZNF559	ZNF559;MGC13105;NBLA00121	zinc finger protein 559	0.82	0.97	1.31	0.77
GPR88	GPR88;STRG	G protein-coupled receptor 88	0.82	1.04	1.31	0.93
AUH	AUH	AU RNA binding protein/enoyl-CoA hydratase	0.8	1	1.31	0.78
PRELID2	PRELID2;FLJ38376;MGC21644	PRELI domain containing 2	0.79	0.93	1.31	0.73
FAM163A	FAM163A;C1orf76;MGC16664;NDSP;RP11-12M5.2	family with sequence similarity 163, member A	0.78	0.9	1.31	0.71
RHO	RHO;CSNBAD1;MGC138309;MGC138311;OPN2;RP4	rhodopsin	0.78	1	1.31	0.9
CMC1	CMC1;C3orf68;DKFZp779D0833;MGC61571	COX assembly mitochondrial protein homolog (S. cerevisiae)	0.77	1	1.31	0.83
COL18A1	COL18A1;FLJ27325;FLJ34914;KNO;KNO1;KS;MGC74745	collagen, type XVIII, alpha 1	0.77	0.97	1.31	0.74
SENP3	SENP3;DKFZp586K0919;DKFZp762A152;SMT3IP1;SSP3	SUMO1/sentrin/SMT3 specific peptidase 3	0.77	0.99	1.31	0.74
IL6ST	IL6ST;CD130;CDW130;DKFZp564F053;GP130;IL-6RB	interleukin 6 signal transducer (gp130, oncostatin M receptor)	0.77	0.96	1.31	0.75
SLC6A20	SLC6A20;MGC161475;SIT1;XT3;Xtrp3	solute carrier family 6 (proline IMINO transporter), member 20	0.76	0.98	1.31	0.73
ZNF434	ZNF434;FLJ20417;FLJ31901;MGC4179	zinc finger protein 434	0.75	0.94	1.31	0.72
THSD1	THSD1;MGC74971;TMTSP;UNQ3010	thrombospondin, type I, domain containing 1	0.74	0.97	1.31	0.67
KLK7	KLK7;PRSS6;SCCE;hK7	kallikrein-related peptidase 7	0.74	0.96	1.31	0.72
PRKCA	PRKCA;AAG6;MGC129900;MGC129901;PKC-alpha;PKCA;PRKACA	protein kinase C, alpha	0.74	0.94	1.31	0.85
ADAD2	ADAD2;FLJ00337;TENRL	adenosine deaminase domain containing 2	0.74	0.87	1.31	0.69
ARHGEF3	ARHGEF3;DKFZp434F2429;FLJ98126;GEF3;MGC118905;STA3;XPLN	Rho guanine nucleotide exchange factor (GEF) 3	0.74	0.98	1.31	0.68
SNRK	SNRK;DKFZp779A1866;FLJ20224;HSNFRK;KIAA0096	SNF related kinase	0.74	0.96	1.31	0.79
ATG13	ATG13;FLJ20698;KIAA0652	ATG13 autophagy related 13 homolog (S. cerevisiae)	0.74	0.95	1.31	0.71
RNF44	RNF44;KIAA1100	ring finger protein 44	0.73	0.92	1.31	0.67
PALM2	PALM2;AKAP2	paralemmin 2	0.73	0.94	1.31	0.78
GPR20	GPR20	G protein-coupled receptor 20	0.72	0.88	1.31	0.66
USP46	USP46;FLJ11850;FLJ12552;FLJ14283;FLJ39393	ubiquitin specific peptidase 46	0.72	0.92	1.31	0.69
NRIP1	NRIP1;FLJ77253;RIP140	nuclear receptor interacting protein 1	0.72	0.91	1.31	0.66
PDHB	PDHB;DKFZp564K0164;PDHBD;PHE1B	pyruvate dehydrogenase (lipoamide) beta	0.72	0.92	1.31	0.65
CYB5A	CYB5A;CYB5;MCB5	cytochrome b5 type A (microsomal)	0.72	0.92	1.31	0.65
SEC14L1	SEC14L1;DKFZp686C06176;PRELID4A;SEC14L	SEC14-like 1 (S. cerevisiae)	0.71	0.86	1.31	0.77
FOXRED1	FOXRED1;H17	FAD-dependent oxidoreductase domain containing 1	0.7	0.88	1.31	0.67
SLC16A1	SLC16A1;FLJ36745;HHF7;MCT;MCT1;MGC44475	solute carrier family 16, member 1 (monocarboxylic acid transporter 1)	0.7	0.88	1.31	0.64
TSPO2	TSPO2;BZRPL1;MGC129806;MGC129807	translocator protein 2	0.7	0.92	1.31	0.64
IGDCC4	IGDCC4;DDM36;FLJ42051;KIAA1628;NOPE	immunoglobulin superfamily, DCC subclass, member 4	0.7	0.92	1.31	0.64
TEDDM1	TEDDM1;EDDM9;Epdd1;HE9;RP1-223H12.2;TMEM45C	transmembrane epididymal protein 1	0.69	0.87	1.31	0.66
GPR12	GPR12;FLJ18149;FLJ97704;GPCR12;GPCR21;MGC138349;MGC138351	G protein-coupled receptor 12	0.68	0.87	1.31	0.79
MRPS36	MRPS36;MGC22896;MRP-S36	mitochondrial ribosomal protein S36	0.68	0.92	1.31	0.61
PRRC2A	PRRC2A;BAT2;D6S51;D6S51E;DKFZp686D09175;G2	proline-rich coiled-coil 2A	0.67	0.9	1.31	0.68
FGD1	FGD1;AAS;FGDY;MRXS16;ZFYVE3	FYVE, RhoGEF and PH domain containing 1	0.66	0.88	1.31	0.59
LMBRD1	LMBRD1;C6orf209;FLJ11240;FLJ11854;LMBD1;NESI	LMBR1 domain containing 1	0.66	0.83	1.31	0.64
ZNF839	ZNF839;C14orf131;FLJ11132	zinc finger protein 839	0.65	0.82	1.31	0.66
EIF2S2	EIF2S2;DKFZp686L18198;EIF2;EIF2B;EIF2beta;MGC8508;PPP1R67	eukaryotic translation initiation factor 2, subunit 2 beta, 38kDa	0.65	0.84	1.31	0.63
SPACA5B	SPACA5B;SPACA5	sperm acrosome associated 5B	0.65	0.88	1.31	0.63
DYNC1I1	DYNC1I1;DNCI1;DNCIC1	dynein, cytoplasmic 1, intermediate chain 1	0.64	0.82	1.31	0.71
GNG4	GNG4;DKFZp547K1018;FLJ23803;FLJ34187	guanine nucleotide binding protein (G protein), gamma 4	0.64	0.85	1.31	0.66
WDR55	WDR55;FLJ20195;FLJ21702	WD repeat domain 55	0.64	0.82	1.31	0.62
RBMY1J	RBMY1J	RNA binding motif protein, Y-linked, family 1, member J	0.64	0.82	1.31	0.65
TLX3	TLX3;HOX11L2;MGC29804;RNX	T-cell leukemia homeobox 3	0.63	0.86	1.31	0.57
ARHGAP6	ARHGAP6;RHOGAP6;RHOGAPX-1	Rho GTPase activating protein 6	0.62	0.85	1.31	0.63
DZIP3	DZIP3;FLJ13327;FLJ57977;FLJ58022;FLJ58223;KIAA0675;PPP1R66;UURF2;hRUL138	DAZ interacting protein 3, zinc finger	0.62	0.79	1.31	0.71
GP6	GP6;BDPLT11;GPIV;GPVI;MGC138168	glycoprotein VI (platelet)	0.84	1.07	1.32	0.76
SPTB	SPTB;EL3;HS2;HSPTB1;SPH2	spectrin, beta, erythrocytic	0.83	1	1.32	0.79
GUK1	GUK1;FLJ42686;FLJ43710;GMK	guanylate kinase 1	0.83	1.06	1.32	0.96
MOB2	MOB2;HCCA2	MOB kinase activator 2	0.81	1.02	1.32	0.78
LYSMD4	LYSMD4;FLJ33008;MGC99501	LysM, putative peptidoglycan-binding, domain containing 4	0.8	1	1.32	0.81
FAM134C	FAM134C;FLJ33806	family with sequence similarity 134, member C	0.78	0.92	1.32	0.72
TSPAN31	TSPAN31;SAS	tetraspanin 31	0.78	0.98	1.32	0.79
FCRL4	FCRL4;CD307d;FCRH4;IGFP2;IRTA1;MGC150522;MGC150523	Fc receptor-like 4	0.77	0.96	1.32	0.78
HS6ST3	HS6ST3;DKFZp761K2315	heparan sulfate 6-O-sulfotransferase 3	0.77	0.95	1.32	0.84
CNOT8	CNOT8;CAF1;CALIF;POP2;hCAF1	CCR4-NOT transcription complex, subunit 8	0.77	1	1.32	0.7
ZSCAN1	ZSCAN1;FLJ33779;MGC104472;MZF-1;ZNF915	zinc finger and SCAN domain containing 1	0.76	0.96	1.32	0.73
WDR45	WDR45;WDRX1;WIPI-4;WIPI4	WD repeat domain 45	0.76	0.95	1.32	0.77
CAT	CAT;MGC138422;MGC138424	catalase	0.75	0.96	1.32	0.83
ZNF101	ZNF101;DKFZp570I0164;HZF12;MGC149565;MGC149566	zinc finger protein 101	0.74	0.95	1.32	0.68
WDR74	WDR74;FLJ10439;FLJ21730	WD repeat domain 74	0.74	0.93	1.32	0.71
LIPT1	LIPT1;MGC12290;MGC13378	lipoyltransferase 1	0.7	0.9	1.32	0.67
HIST1H2BJ	HIST1H2BJ;H2B/r;H2BFR	histone cluster 1, H2bj	0.7	0.89	1.32	0.64
TTF2	TTF2;HuF2	transcription termination factor, RNA polymerase II	0.7	0.9	1.32	0.7
PRAME	PRAME;CT130;MAPE;OIP4	preferentially expressed antigen in melanoma	0.7	0.9	1.32	0.68
MRPL32	MRPL32;L32mt;MRP-L32;bMRP-59b	mitochondrial ribosomal protein L32	0.7	0.86	1.32	0.76
NLRP6	NLRP6;CLR11.4;NALP6;PAN3;PYPAF5	NLR family, pyrin domain containing 6	0.68	0.86	1.32	0.66
ZNF75A	ZNF75A;FLJ31529;MGC59959	zinc finger protein 75a	0.68	0.89	1.32	0.71
USP36	USP36;DUB1	ubiquitin specific peptidase 36	0.68	0.9	1.32	0.67
LDHD	LDHD;DLD;MGC57726	lactate dehydrogenase D	0.67	0.86	1.32	0.61
ZSCAN5B	ZSCAN5B;ZNF495B	zinc finger and SCAN domain containing 5B	0.66	0.88	1.32	0.66
TRIP11	TRIP11;ACG1A;CEV14;GMAP-210;TRIP230	thyroid hormone receptor interactor 11	0.66	0.88	1.32	0.68

FBXO24	FBXO24;DKFZp434I1122;FBX24	F-box protein 24	0.64	0.8	1.32	0.58
EIF2B4	EIF2B4;DKFZp586J0119;EIF-2B;EIF2B;EIF2Bdelta	eukaryotic translation initiation factor 2B, subunit 4 delta, 67kDa	0.64	0.8	1.32	0.63
RPL29	RPL29;HIP;HUMRPL29;L29;MGC88589	ribosomal protein L29	0.64	0.82	1.32	0.65
CPOX	CPOX;CPO;CPX;HCP	coproporphyrinogen oxidase	0.63	0.77	1.32	0.68
MRPS12	MRPS12;MPR-S12;MT-RPS12;RPMS12;RPS12;RPSM12	mitochondrial ribosomal protein S12	0.62	0.8	1.32	0.61
TNPO3	TNPO3;IPO12;MTR10A;TRN-SR;TRN-SR2;TRNSR	transportin 3	0.62	0.85	1.32	0.69
ZNF165	ZNF165;CT53;LD65;ZSCAN7	zinc finger protein 165	0.61	0.82	1.32	0.63
TCF7L2	TCF7L2;TCF-4;TCF4	transcription factor 7-like 2 (T-cell specific, HMG-box)	0.84	1.04	1.33	0.83
DMBX1	DMBX1;MBX;OTX3;PAXB	diencephalon/mesencephalon homeobox 1	0.82	1.04	1.33	0.8
KTI12	KTI12;MGC20419;RP11-91A18.3;TOT4	KTI12 homolog, chromatin associated (S. cerevisiae)	0.8	1.04	1.33	0.78
CANT1	CANT1;DBQD;SCAN-1;SCAN1;SHAPY	calcium activated nucleotidase 1	0.8	1.03	1.33	0.76
UNC5A	UNC5A;FLJ16449;KIAA1976;UNC5H1	unc-5 homolog A (C. elegans)	0.78	1	1.33	0.76
SBNO1	SBNO1;FLJ10701;FLJ10833;FLJ14066;FLJ16176;FLJ35199;MOP3;Sno	strawberry notch homolog 1 (Drosophila)	0.77	0.99	1.33	0.76
TMEM2	TMEM2	transmembrane protein 2	0.76	0.99	1.33	0.82
GAGE2C	GAGE2C;CT4.2;GAGE-2;GAGE-2B;GAGE-2C;GAGE2;GAGE2A;GAGE2B;MGC120097;MGC96883;MGC96930;MGC96942	G antigen 2C	0.75	0.97	1.33	0.76
GRAMD2	GRAMD2	GRAM domain containing 2	0.74	0.97	1.33	0.81
CD55	CD55;CR;CROM;DAF;TC	CD55 molecule, decay accelerating factor for complement (Cromer blood group)	0.74	0.87	1.33	0.68
C15orf57	C15orf57;CCDC32;FLJ25915;MGC20481	chromosome 15 open reading frame 57	0.73	0.86	1.33	0.68
UQCC	UQCC;BFZB;C20orf44;CBP3;MGC104353;MGC141902	ubiquinol-cytochrome c reductase complex chaperone	0.73	0.94	1.33	0.72
BAIAP3	BAIAP3;BAP3;KIAA0734;MGC138334	BAI1-associated protein 3	0.72	0.94	1.33	0.78
TRAPPC2	TRAPPC2;MIP2A;SEDL;SEDT;TRAPPC2P1;TRS20;ZNF547L;hYP38334	trafficking protein particle complex 2	0.72	0.93	1.33	0.71
SOX12	SOX12;SOX22	SRY (sex determining region Y)-box 12	0.72	0.93	1.33	0.67
ACSBG2	ACSBG2;BGR;BRGL;DKFZp434K1635;MGC111089;PRTD-NY3;PRTDNY3	acyl-CoA synthetase bubblegum family member 2	0.71	0.94	1.33	0.7
CARM1	CARM1;PRMT4	coactivator-associated arginine methyltransferase 1	0.71	0.91	1.33	0.66
IDUA	IDUA;IDA;MPS1	iduronidase, alpha-L-	0.7	0.9	1.33	0.63
GNPTAB	GNPTAB;DKFZp762B226;GNPTA;ICD;KIAA1208;MGC4170	N-acetylglucosamine-1-phosphate transferase, alpha and beta subunits	0.7	0.83	1.33	0.65
PPP1R14A	PPP1R14A;CPI-17;CPI17;PPP1INL	protein phosphatase 1, regulatory (inhibitor) subunit 14A	0.69	0.92	1.33	0.63
FBXL20	FBXL20;DKFZp564O2364;FLJ21037;Fbl2;Fbl20;MGC15482	F-box and leucine-rich repeat protein 20	0.68	0.86	1.33	0.62
FAM122B	FAM122B;DKFZp686L20116;MGC131814;SPACIA2	family with sequence similarity 122B	0.68	0.86	1.33	0.7
TBC1D5	TBC1D5;KIAA0210	TBC1 domain family, member 5	0.66	0.9	1.33	0.68
RBMX2	RBMX2;CGI-79	RNA binding motif protein, X-linked 2	0.66	0.86	1.33	0.68
AGAP7	AGAP7;CTGLF4;bA109G10.1	ArfGAP with GTPase domain, ankyrin repeat and PH domain 7	0.66	0.86	1.33	0.65
EGR3	EGR3;EGR-3;MGC138484;PILOT	early growth response 3	0.65	0.85	1.33	0.66
FAM71E1	FAM71E1;FLJ27522;FLJ43757	family with sequence similarity 71, member E1	0.65	0.89	1.33	0.67
SLC45A1	SLC45A1;DNB5;FLJ26758;KIAA0458	solute carrier family 45, member 1	0.64	0.82	1.33	0.62
SPRY1	SPRY1;hSPRY1	sprouty homolog 1, antagonist of FGF signaling (Drosophila)	0.64	0.86	1.33	0.6
SHARPIN	SHARPIN;DKFZp434N1923;SIPL1	SHANK-associated RH domain interactor	0.64	0.88	1.33	0.66
SAE1	SAE1;AOS1;FLJ3091;HSPC140;SUA1;UBLE1A	SUMO1 activating enzyme subunit 1	0.64	0.78	1.33	0.69
PHOSPHO2	PHOSPHO2;MGC111048;MGC22679	phosphatase, orphan 2	0.64	0.86	1.33	0.6
TM6SF2	TM6SF2;KIAA1926	transmembrane 6 superfamily member 2	0.63	0.86	1.33	0.65
ARF3	ARF3	ADP-ribosylation factor 3	0.63	0.77	1.33	0.69
SLC31A2	SLC31A2;COPT2;CTR2;hCTR2	solute carrier family 31 (copper transporters), member 2	0.62	0.8	1.33	0.56
ILK	ILK;DKFZp686F1765;ILK-2;P59	integrin-linked kinase	0.62	0.79	1.33	0.71
AGAP2	AGAP2;CENTG1;FLJ16430;GGAP2;KIAA0167;PIKE	ArfGAP with GTPase domain, ankyrin repeat and PH domain 2	0.61	0.74	1.33	0.61
ARL15	ARL15;ARFRP2;FLJ20051	ADP-ribosylation factor-like 15	0.84	1	1.34	0.78
MAP7D2	MAP7D2;FLJ14503;MGC104944	MAP7 domain containing 2	0.83	1.07	1.34	0.82
SPTSSA	SPTSSA;C14orf147;MGC24447;SSSPTA	serine palmitoyltransferase, small subunit A	0.83	1	1.34	0.83
C9orf91	C9orf91;DKFZp547P234;FLJ38045;MGC129868;MGC129869;RP11-402G3.2	chromosome 9 open reading frame 91	0.81	1.05	1.34	0.8
CCDC144B	CCDC144B;FLJ36492;MGC126634	coiled-coil domain containing 144B (pseudogene)	0.8	1.02	1.34	0.78
ACTR6	ACTR6;ARP6;FLJ13433;HSPC281;MSTP136;hARP6;hARPX	ARP6 actin-related protein 6 homolog (yeast)	0.77	1	1.34	0.72
AMIGO1	AMIGO1;AMIGO;AMIGO-1	adhesion molecule with Ig-like domain 1	0.74	0.97	1.34	0.74
SRSF6	SRSF6;B52;FLJ08061;MGC5045;SFRS6;SRP55	serine/arginine-rich splicing factor 6	0.74	0.97	1.34	0.76
BEND7	BEND7;C10orf30;FLJ40283;MGC35247	BEN domain containing 7	0.73	0.88	1.34	0.7
GABARAPL1	GABARAPL1;APG8-LIKE;APG8L;ATG8;ATG8B;ATG8L;GEC1	GABA(A) receptor-associated protein like 1	0.72	0.88	1.34	0.7
ZHX1	ZHX1	zinc fingers and homeoboxes 1	0.7	0.94	1.34	0.75
MGEA5	MGEA5;FLJ11229;FLJ23355;KIAA0679;MEA5;NCOAT;OGA	meningioma expressed antigen 5 (hyaluronidase)	0.7	0.92	1.34	0.71
HPR	HPR;A-259H10.2;HP	haptoglobin-related protein	0.68	0.9	1.34	0.68
SLC22A6	SLC22A6;FLJ55736;HOAT1;MGC45260;OAT1;PAHT;ROAT1	solute carrier family 22 (organic anion transporter), member 6	0.68	0.88	1.34	0.63
ZNF710	ZNF710;DKFZp547K1113;FLJ00306;FLJ37393;MGC163413	zinc finger protein 710	0.68	0.88	1.34	0.68
ZNF192	ZNF192;LD5-1;ZKSCAN8	zinc finger protein 192	0.66	0.87	1.34	0.66
RPS19BP1	RPS19BP1;AROS;FLJ21770;MGC52010;S19BP;dJ1104E15.4	ribosomal protein S19 binding protein 1	0.66	0.85	1.34	0.64
H3F3A	H3F3A;H3.3A;H3F3;H3F3B;MGC87782;MGC87783	H3 histone, family 3A	0.64	0.87	1.34	0.59
GET4	GET4;C7orf20;CEE;TRC35	golgi to ER traffic protein 4 homolog (S. cerevisiae)	0.64	0.88	1.34	0.66
PCDH12	PCDH12;VE-cadherin-2;VECAD2	protocadherin 12	0.62	0.8	1.34	0.58
MYBPC2	MYBPC2;MGC163408;MYBPC;MYBPCF	myosin binding protein C, fast type	0.84	1.08	1.35	0.83
SLC17A5	SLC17A5;AST;FLJ22227;FLJ23268;ISSD;NSD;SD;SIALIN;SIASD;SLD	solute carrier family 17 (anion/sugar transporter), member 5	0.83	1.06	1.35	0.84
BTLA	BTLA;BTLA1;CD272;FLJ16065;MGC129743	B and T lymphocyte associated	0.76	0.92	1.35	0.73
OR5F1	OR5F1;OR11-10	olfactory receptor, family 5, subfamily F, member 1	0.76	0.99	1.35	0.71
HSPA4L	HSPA4L;APG-1;HSPH3;Osp94	heat shock 70kDa protein 4-like	0.76	0.98	1.35	0.77
GATAD1	GATAD1;FLJ22489;FLJ40695;ODAG;RG083M05.2	GATA zinc finger domain containing 1	0.75	0.97	1.35	0.75
UPF3A	UPF3A;FLJ35267;FLJ57970;HUPF3A;RENT3A;UPF3	UPF3 regulator of nonsense transcripts homolog A (yeast)	0.74	0.97	1.35	0.74
ADRB1	ADRB1;ADRB1R;B1AR;BETA1AR;RHR	adrenergic, beta-1-, receptor	0.73	0.92	1.35	0.7
GABARAP	GABARAP;ATG8A;FLJ25768;MGC120154;MGC120155;MM46	GABA(A) receptor-associated protein	0.72	0.93	1.35	0.73
LAMTOR1	LAMTOR1;C11orf59;FLJ20625;PDRO;Ragulator1;p18;p27RF-Rho	late endosomal/lysosomal adaptor, MAPK and MTOR activator 1	0.72	0.88	1.35	0.68
AVPR1B	AVPR1B;AVPR3	arginine vasopressin receptor 1B	0.68	0.88	1.35	0.8
ZNF497	ZNF497;FLJ42722;FLJ44773	zinc finger protein 497	0.68	0.89	1.35	0.68
SUSD4	SUSD4;FLJ10052;PRO222	sushi domain containing 4	0.66	0.88	1.35	0.65
MUC16	MUC16;CA125;FLJ14303	mucin 16, cell surface associated	0.66	0.9	1.35	0.61
GSX1	GSX1;GSH1;Gsh-1	GS homeobox 1	0.65	0.78	1.35	0.65
RUNX3	RUNX3;AML2;CBFA3;FLJ34510;MGC16070;PEBP2aC	runt-related transcription factor 3	0.64	0.82	1.35	0.65
BAZ1A	BAZ1A;ACF1;DKFZp586E0518;FLJ14383;WALp1;WCRF180;hACF1	bromodomain adjacent to zinc finger domain, 1A	0.64	0.87	1.35	0.59
ECE1	ECE1;ECE	endothelin converting enzyme 1	0.64	0.8	1.35	0.61
TMEM123	TMEM123;KCT3;PORIMIN;PORMIN	transmembrane protein 123	0.64	0.84	1.35	0.64
HES6	HES6;HES-6;bHLHb41;bHLHc23	hairy and enhancer of split 6 (Drosophila)	0.61	0.79	1.35	0.57
GNPAT	GNPAT;DAP-AT;DAPAT;DHAPAT	glyceronephosphate O-acyltransferase	0.8	1	1.36	0.89
GPR116	GPR116;DKFZp564O1923;FLJ90640;KIAA0758;KPG_001	G protein-coupled receptor 116	0.78	0.98	1.36	0.74
SLC38A1	SLC38A1;ATA1;NAT2;SAT1;SNAT1	solute carrier family 38, member 1	0.78	0.98	1.36	0.87
HOMEZ	HOMEZ;KIAA1443	homeobox and leucine zipper encoding	0.75	0.91	1.36	0.71
FCGBP	FCGBP;FC(GAMMA)BP	Fc fragment of IgG binding protein	0.74	0.98	1.36	0.74
CCDC7	CCDC7;BioT2-A;BioT2-B;BioT2-C;DKFZp686N0559;FLJ32762;RP11-479G22.1	coiled-coil domain containing 7	0.74	0.92	1.36	0.73
EPB41L4B	EPB41L4B;CG1;DKFZp761N1814;EHM2;FLJ21596	erythrocyte membrane protein band 4.1 like 4B	0.73	0.97	1.36	0.74
CAPN7	CAPN7;CALPAIN7;FLJ36423;PALBH	calpain 7	0.72	0.9	1.36	0.68
DCUN1D4	DCUN1D4;FLJ42355;KIAA0276	DCN1, defective in cullin neddylation 1, domain containing 4 (S. cerevisiae)	0.7	0.87	1.36	0.66
TFG	TFG;FLJ36137;TF6;TRKT3	TRK-fused gene	0.7	0.92	1.36	0.71
SNTN	SNTN;FLJ44379;S100A1L;S100AL;sentan	sentan, cilia apical structure protein	0.69	0.83	1.36	0.65
DDAH2	DDAH2;DDAH;DDAHII;G6a;NG30	dimethylarginine dimethylaminohydrolase 2	0.68	0.9	1.36	0.77
SLC12A2	SLC12A2;BSC;BSC2;MGC104233;NKCC1	solute carrier family 12 (sodium/potassium/chloride transporters), member 2	0.68	0.9	1.36	0.63
INPP5A	INPP5A;5PTASE;DKFZp434A1721;MGC116947;MGC116949	inositol polyphosphate-5-phosphatase, 40kDa	0.66	0.83	1.36	0.62
ATP7B	ATP7B;PWD;WC1;WD;WND	ATPase, Cu++ transporting, beta polypeptide	0.64	0.88	1.36	0.68
ZBED2	ZBED2;MGC10796	zinc finger, BED-type containing 2	0.62	0.86	1.36	0.65
KLHL2	KLHL2;ABP-KELCH;MAV;MAYVEN	kelch-like 2, Mayven (Drosophila)	0.62	0.86	1.36	0.58
PRR25	PRR25;gs64	proline rich 25	0.62	0.82	1.36	0.62
NARS	NARS;ASNRS;NARS1	asparaginyl-tRNA synthetase	0.61	0.81	1.36	0.63
PTPN11	PTPN11;BPTP3;CFC;MGC14433;NS1;PTP-1D;PTP2C;SH-PTP2;SH-PTP3;SHP2	protein tyrosine phosphatase, non-receptor type 11	0.84	1.05	1.37	0.79
PTTG1	PTTG1;EAP1;HPTTG;MGC126883;MGC138276;PTTG;TUTR1	pituitary tumor-transforming 1	0.79	1.02	1.37	0.8
GPC6	GPC6;MGC126288;OMIMD1	glypican 6	0.74	0.91	1.37	0.73
SLC35F5	SLC35F5;FLJ22004	solute carrier family 35, member F5	0.74	0.92	1.37	0.73
ATAD1	ATAD1;AFDC1;FLJ14600	ATPase family, AAA domain containing 1	0.74	0.98	1.37	0.7
FAM98B	FAM98B;FLJ38426	family with sequence similarity 98, member B	0.73	0.94	1.37	0.77
ETFA	ETFA;EMA;GA2;MADD	electron-transfer-flavoprotein, alpha polypeptide	0.73	0.92	1.37	0.81
TRERF1	TRERF1;BCAR2;FLJ21198;HSA277276;RAPA;RP1-139D8.5;TReP-132;dJ139D8.5	transcriptional regulating factor 1	0.71	0.95	1.37	0.67
DEF6	DEF6;IBP;SLAT;SWAP70L	differentially expressed in FDCP 6 homolog (mouse)	0.69	0.92	1.37	0.7
KIF5A	KIF5A;D12S1889;MY050;NKHC;SPG10	kinesin family member 5A	0.68	0.9	1.37	0.78
SPIN3	SPIN3;FLJ41127;FLJ54952;MGC40395;SPIN-3;bA445O16.1	spindlin family, member 3	0.68	0.92	1.37	0.69
STX10	STX10;SYN10;hsyn10	syntaxin 10	0.66	0.87	1.37	0.63
IGFBP7	IGFBP7;AGM;FSTL2;IBP-7;IGFBP-7;IGFBP-7v;IGFBPRP1;MAC25;PSF;RAMSVPS;TAF	insulin-like growth factor binding protein 7	0.66	0.86	1.37	0.62
C22orf13	C22orf13;LLN4;MGC1842	chromosome 22 open reading frame 13	0.66	0.86	1.37	0.66
ECHDC3	ECHDC3;FLJ20909	enoyl CoA hydratase domain containing 3	0.64	0.85	1.37	0.73
AIRE	AIRE;AIRE1;APECED;APS1;APSI;PGA1	autoimmune regulator	0.64	0.85	1.37	0.77
BMP1	BMP1;FLJ44432;PCOLC;PCP;PCP2;TLD	bone morphogenetic protein 1	0.64	0.8	1.37	0.6
CKLF	CKLF;C32;CKLF1;CKLF2;CKLF3;CKLF4;UCK-1	chemokine-like factor	0.64	0.84	1.37	0.64
NAIP	NAIP;BIRC1;FLJ18088;FLJ42520;FLJ58811;NLRB1;psiNAIP	NLR family, apoptosis inhibitory protein	0.62	0.83	1.37	0.62
FAM83H	FAM83H;AI3;FLJ46072	family with sequence similarity 83, member H	0.62	0.83	1.37	0.64
CKS1B	CKS1B;CKS1;PNAS-16;PNAS-18;ckshs1	CDC28 protein kinase regulatory subunit 1B	0.62	0.86	1.37	0.71
SPIRE2	SPIRE2;MGC117166;Spir-2	spire homolog 2 (Drosophila)	0.61	0.82	1.37	0.68
EXOG	EXOG;ENDOGL1;ENDOGL2;ENGL;ENGL-a;ENGL-b;ENGLA;ENGLB;MGC125944;MGC125945	endo/exonuclease (5'-3'), endonuclease G-like	0.82	1.06	1.38	0.85
FBXO16	FBXO16;FBX16;MGC125923;MGC125924;MGC125925	F-box protein 16	0.8	1.04	1.38	0.76
C2orf65	C2orf65;D6Mm5e;FLJ44410	chromosome 2 open reading frame 65	0.78	1.02	1.38	0.86
PPM1L	PPM1L;MGC132545;MGC132547;PP2C-epsilon;PP2CE;PPM1-LIKE	protein phosphatase, Mg2+/Mn2+ dependent, 1L	0.74	0.95	1.38	0.71

C19orf59	C19orf59;MCEMP1;MGC132456	chromosome 19 open reading frame 59	0.74	0.98	1.38	0.74
PPM1N	PPM1N;FLJ40125	protein phosphatase, Mg2+/Mn2+ dependent, 1N (putative)	0.74	0.91	1.38	0.73
APBB1	APBB1;FE65;MGC:9072;RIR	amyloid beta (A4) precursor protein-binding, family B, member 1 (Fe65)	0.7	0.88	1.38	0.73
MLLT4	MLLT4;AF6;FLJ34371;RP3-431P23.3	myeloid/lymphoid or mixed-lineage leukemia (trithorax homolog, Drosophila); translocated to, 4	0.68	0.92	1.38	0.7
DPPA3	DPPA3;STELLA	developmental pluripotency associated 3	0.66	0.82	1.38	0.64
NOD1	NOD1;CARD4;CLR7.1;NLRC1	nucleotide-binding oligomerization domain containing 1	0.66	0.88	1.38	0.62
SP140L	SP140L;MGC132667	SP140 nuclear body protein-like	0.65	0.86	1.38	0.62
PDZD4	PDZD4;FLJ34125;KIAA1444;LU1;PDZK4;PDZRN4L	PDZ domain containing 4	0.65	0.85	1.38	0.69
LHX2	LHX2;LH2;MGC138390;hLhx2	LIM homeobox 2	0.64	0.86	1.38	0.65
GJA8	GJA8;CAE;CAE1;CX50;CZP1;MP70	gap junction protein, alpha 8, 50kDa	0.62	0.78	1.38	0.63
SLC2A14	SLC2A14;DKFZp564K1672;GLUT14;SLC2A3P3	solute carrier family 2 (facilitated glucose transporter), member 14	0.62	0.79	1.38	0.7
LOC641522	LOC641522	ENTREZ STATUS: REMAPPED TO GENE ID: 100506084	0.78	0.96	1.39	0.75
HIST1H2BF	HIST1H2BF;H2B/a;H2B/g;H2B/h;H2B/k;H2B/l;H2BFG	histone cluster 1, H2bf	0.76	0.95	1.39	0.79
CHTF18	CHTF18;C16orf41;C321D2.2;C321D2.3;C321D2.4;CHL12;Ctf18;RUVBL	CTF18, chromosome transmission fidelity factor 18 homolog (S. cerevisiae)	0.68	0.86	1.39	0.68
METTL5	METTL5;FLJ10459;HSPC133	methyltransferase like 5	0.68	0.9	1.39	0.65
C3orf20	C3orf20;DKFZp434N1817	chromosome 3 open reading frame 20	0.64	0.84	1.39	0.68
CD14	CD14	CD14 molecule	0.64	0.82	1.39	0.66
DYNC1LI2	DYNC1LI2;DNCLI2;LIC2	dynein, cytoplasmic 1, light intermediate chain 2	0.63	0.86	1.39	0.65
TMEM14B	TMEM14B;FLJ60468;MGC1223	transmembrane protein 14B	0.63	0.85	1.39	0.64
FAM190A	FAM190A;KIAA1680;MGC48628	family with sequence similarity 190, member A	0.63	0.85	1.39	0.61
RETN	RETN;ADSF;FIZZ3;MGC126603;MGC126609;RETN1;RSTN;XCP1	resistin	0.62	0.84	1.39	0.65
TPRX1	TPRX1;FLJ40321;TPRX	tetra-peptide repeat homeobox 1	0.78	0.98	1.4	0.77
TMEM129	TMEM129;D4S2561E;FLJ25600	transmembrane protein 129	0.74	0.98	1.4	0.79
CSRNP3	CSRNP3;FAM130A2;FLJ11703;FLJ32093;FLJ44141;PPP1R73;TAIP-2;TAIP2	cysteine-serine-rich nuclear protein 3	0.71	0.94	1.4	0.76
NTSR2	NTSR2;NTR2	neurotensin receptor 2	0.68	0.89	1.4	0.67
WFDC10A	WFDC10A;C20orf146;WAP10;dJ688G8.3	WAP four-disulfide core domain 10A	0.66	0.83	1.4	0.66
BTBD11	BTBD11;FLJ33957;FLJ42845	BTB (POZ) domain containing 11	0.66	0.84	1.4	0.67
CCDC112	CCDC112;MBC1;MGC39633	coiled-coil domain containing 112	0.61	0.81	1.4	0.66
EFCAB4A	EFCAB4A;CRACR2B;FLJ46033;MGC45840;MGC88858	EF-hand calcium binding domain 4A	0.69	0.92	1.41	0.75
MT2A	MT2A;MT2	metallothionein 2A	0.68	0.86	1.41	0.68
SECISBP2L	SECISBP2L;KIAA0256	SECIS binding protein 2-like	0.68	0.88	1.41	0.7
RNF182	RNF182;FLJ40772;MGC33993	ring finger protein 182	0.66	0.9	1.41	0.81
VAV2	VAV2;VAV-2	vav 2 guanine nucleotide exchange factor	0.65	0.88	1.41	0.63
TMEM127	TMEM127;FLJ20507;FLJ22257	transmembrane protein 127	0.62	0.84	1.41	0.64
LNPEP	LNPEP;CAP;IRAP;P-LAP;PLAP	leucyl/cystinyl aminopeptidase	0.76	0.99	1.42	0.75
C7orf46	C7orf46;DKFZp686F0810;FLJ45875;MGC72075	chromosome 7 open reading frame 46	0.74	0.95	1.42	0.74
PPARD	PPARD;FAAR;MGC3931;NR1C2;NUC1;NUCI;NUCII;PPARB	peroxisome proliferator-activated receptor delta	0.7	0.94	1.42	0.74
BEX1	BEX1;BEX2;HBEX2;HGR74-h	brain expressed, X-linked 1	0.66	0.86	1.42	0.68
BEND2	BEND2;CXorf20;MGC33653	BEN domain containing 2	0.65	0.84	1.42	0.66
FCRL2	FCRL2;CD307b;FCRH2;IFGP4;IRTA4;SPAP1;SPAP1A;SPAP1B;SPAP1C	Fc receptor-like 2	0.62	0.86	1.42	0.63
DPH1	DPH1;DPH2L;DPH2L1;FLJ33211;OVCA1	DPH1 homolog (S. cerevisiae)	0.66	0.84	1.43	0.65
EXOC1	EXOC1;BM-102;FLJ10893;SEC3;SEC3L1;SEC3P	exocyst complex component 1	0.64	0.86	1.43	0.7
IL9R	IL9R;CD129	interleukin 9 receptor	0.62	0.86	1.43	0.75
CPSF3L	CPSF3L;CPSF73L;FLJ13294;FLJ20542;INT11;INTS11;RC-68;RC68	cleavage and polyadenylation specific factor 3-like	0.61	0.84	1.43	0.64
LILRA5	LILRA5;CD85;CD85F;ILT-11;ILT11;LILRB7;LIR-9;LIR9	leukocyte immunoglobulin-like receptor, subfamily A (with TM domain), member 5	0.84	0.94	1.25	0.73
MEX3A	MEX3A;MEX-3A;RKHD4	mex-3 homolog A (C. elegans)	0.84	1.01	1.25	0.73
KIAA0564	KIAA0564;FLJ21779	KIAA0564	0.82	0.98	1.25	0.79
SRGAP1	SRGAP1;ARHGAP13;FLJ22166;KIAA1304	SLIT-ROBO Rho GTPase activating protein 1	0.82	1.02	1.25	0.84
GRINA	GRINA;HNRGW;LFG1;MGC99687;NMDARA1;TMBIM3	glutamate receptor, ionotropic, N-methyl D-aspartate-associated protein 1 (glutamate binding)	0.82	1.01	1.25	0.79
DSTN	DSTN;ACTDP;ADF;bA462D18.2	destrin (actin depolymerizing factor)	0.82	1.02	1.25	0.71
PRPF4B	PRPF4B;KIAA0536;PR4H;PRP4;PRP4H;PRP4K;dJ1013A10.1	PRP4 pre-mRNA processing factor 4 homolog B (yeast)	0.82	1	1.25	0.9
HNRPLL	HNRPLL;SRRF;hnRNPLL	heterogeneous nuclear ribonucleoprotein L-like	0.81	0.98	1.25	0.74
DACT3	DACT3;MGC15476;RRR1	dapper, antagonist of beta-catenin, homolog 3 (Xenopus laevis)	0.8	0.94	1.25	0.76
BAZ2B	BAZ2B;DKFZp434H071;DKFZp762I0516;FLJ45644;WALp4	bromodomain adjacent to zinc finger domain, 2B	0.8	0.98	1.25	0.88
HENMT1	HENMT1;C1orf59;FLJ30525;HEN1;MGC111091	HEN1 methyltransferase homolog 1 (Arabidopsis)	0.79	0.88	1.25	0.69
GSTA4	GSTA4;DKFZp686D21185;GSTA4-4;GTA4	glutathione S-transferase alpha 4	0.77	0.94	1.25	0.8
GALNT1	GALNT1;GALNAC-T1	UDP-N-acetyl-alpha-D-galactosamine:polypeptide N-acetylgalactosaminyltransferase 1 (GalNAc-T1)	0.77	0.93	1.25	0.69
LASP1	LASP1;Lasp-1;MLN50	LIM and SH3 protein 1	0.77	0.98	1.25	0.66
DEFB135	DEFB135;DEFB136	defensin, beta 135	0.77	0.98	1.25	0.71
DBC1	DBC1;DBCCR1;FAM5A	deleted in bladder cancer 1	0.76	0.95	1.25	0.66
STC2	STC2;STC-2;STCRP	stanniocalcin 2	0.76	0.86	1.25	0.68
RAVER1	RAVER1;KIAA1978	ribonucleoprotein, PTB-binding 1	0.76	0.96	1.25	0.65
OR14C36	OR14C36;OR5BF1	olfactory receptor, family 14, subfamily C, member 36	0.74	0.96	1.25	0.73
ASPM	ASPM;ASP;Calmbp1;DKFZp686N06184;FLJ10517;FLJ10549;FLJ43117;MCPH5	asp (abnormal spindle) homolog, microcephaly associated (Drosophila)	0.74	0.95	1.25	0.72
OR2C1	OR2C1;MGC163200;MGC95444;OLFmf3;OR2C2P	olfactory receptor, family 2, subfamily C, member 1	0.73	0.94	1.25	0.71
PLOD1	PLOD1;FLJ42041;LH;LH1;LLH;PLOD	procollagen-lysine, 2-oxoglutarate 5-dioxygenase 1	0.73	0.89	1.25	0.63
ZNF397	ZNF397;MGC13250;ZNF47;ZSCAN15	zinc finger protein 397	0.72	0.88	1.25	0.67
MED7	MED7;CRSP33;CRSP9;MGC12284	mediator complex subunit 7	0.72	0.84	1.25	0.75
ADAM17	ADAM17;ADAM18;CD156B;CSVP;MGC71942;NISBD;TACE	ADAM metallopeptidase domain 17	0.72	0.88	1.25	0.67
C2orf84	C2orf84;FLJ30851	chromosome 2 open reading frame 84	0.72	0.86	1.25	0.66
TXNRD1	TXNRD1;GRIM-12;MGC9145;TR;TR1;TRXR1;TXNR	thioredoxin reductase 1	0.71	0.9	1.25	0.6
CLDN2	CLDN2;SP82	claudin 2	0.7	0.92	1.25	0.74
BCAS1	BCAS1;AIBC1;NABC1	breast carcinoma amplified sequence 1	0.7	0.91	1.25	0.69
MAGI3	MAGI3;MAGI-3;MGC163281;RP4-730K3.1;dJ730K3.2	membrane associated guanylate kinase, WW and PDZ domain containing 3	0.7	0.94	1.25	0.67
ZNF304	ZNF304;MGC129703;MGC129704	zinc finger protein 304	0.7	0.82	1.25	0.7
ALG12	ALG12;CDG1G;ECM39;MGC111358;MGC3136;PP14673;hALG12	asparagine-linked glycosylation 12, alpha-1,6-mannosyltransferase homolog (S. cerevisiae)	0.7	0.88	1.25	0.59
ZNF295	ZNF295;DKFZp781N1974;ZBTB21	zinc finger protein 295	0.7	0.91	1.25	0.69
CTNNBL1	CTNNBL1;C20orf33;FLJ21108;NAP;NYD-SP19;P14L;PP8304;dJ633O20.1	catenin, beta like 1	0.7	0.91	1.25	0.69
EIF3M	EIF3M;B5;FLJ29030;PCID1;hfl-B5	eukaryotic translation initiation factor 3, subunit M	0.7	0.92	1.25	0.66
PMS2P5	PMS2P5;MGC34222;PMS2L5;PMS7	postmeiotic segregation increased 2 pseudogene 5	0.7	0.9	1.25	0.65
SEMA5B	SEMA5B;FLJ10372;KIAA1445;SEMAG;SemG	sema domain, seven thrombospondin repeats (type 1 and type 1-like), transmembrane domain (TM) and short cytoplasmic domain, (semaphorin) 5B	0.69	0.84	1.25	0.66
DNAJC3	DNAJC3;FLJ21288;HP58;P58;P58IPK;PRKRI	DnaJ (Hsp40) homolog, subfamily C, member 3	0.69	0.82	1.25	0.69
NRM	NRM;NRM29	nurim (nuclear envelope membrane protein)	0.68	0.76	1.25	0.61
MTUS1	MTUS1;ATBP;ATIP;DKFZp586D1519;DKFZp686F20243;FLJ14295;KIAA1288;MP44;MTSG1	microtubule associated tumor suppressor 1	0.68	0.82	1.25	0.62
POLR3E	POLR3E;RPC5;SIN	polymerase (RNA) III (DNA directed) polypeptide E (80kD)	0.68	0.85	1.25	0.58
SMAD3	SMAD3;DKFZp586N0721;DKFZp686J10186;HSPC193;HsT17436;JV15-2;LDS1C;MADH3;MGC60396	SMAD family member 3	0.68	0.8	1.25	0.62
MRPL13	MRPL13;L13;L13A;L13mt;RPL13;RPML13	mitochondrial ribosomal protein L13	0.67	0.81	1.25	0.65
ZNF43	ZNF43;DKFZp686L1854;HTF6;KOX27;ZNF39L1	zinc finger protein 43	0.67	0.82	1.25	0.64
C7orf43	C7orf43;DKFZp761G0712;FLJ10925	chromosome 7 open reading frame 43	0.67	0.83	1.25	0.6
NUP35	NUP35;MP44;NP44;NUP53	nucleoporin 35kDa	0.67	0.84	1.25	0.6
SLC28A2	SLC28A2;CNT2;FLJ21468;HCNT2;HsT17153;MGC138252;SPNT1	solute carrier family 28 (sodium-coupled nucleoside transporter), member 2	0.66	0.8	1.25	0.59
PRELID1	PRELID1;MGC87972;PRELI;PX19	PRELI domain containing 1	0.66	0.89	1.25	0.62
C22orf43	C22orf43;MGC33025;MGC75009	chromosome 22 open reading frame 43	0.66	0.84	1.25	0.58
VSTM4	VSTM4;C10orf72;FLJ31737;MGC44086	V-set and transmembrane domain containing 4	0.66	0.75	1.25	0.6
FCGR1B	FCGR1B;CD64b;IGFRB	Fc fragment of IgG, high affinity Ib, receptor (CD64)	0.66	0.74	1.25	0.62
ISLR	ISLR;HsT17563;MGC102816	immunoglobulin superfamily containing leucine-rich repeat	0.65	0.78	1.25	0.6
GRTP1	GRTP1;FLJ22474;MGC138328;MGC138330;TBC1D6	growth hormone regulated TBC protein 1	0.65	0.8	1.25	0.6
IL17RA	IL17RA;CANDF5;CD217;CDw217;FLJ32274;FLJ36453;IL-17RA;IL17R;MGC10262;hIL-17R	interleukin 17 receptor A	0.65	0.75	1.25	0.67
PDHA1	PDHA1;PDHA;PDHCE1A;PHE1A	pyruvate dehydrogenase (lipoamide) alpha 1	0.65	0.82	1.25	0.63
C10orf25	C10orf25;FLJ18393;FLJ30567	chromosome 10 open reading frame 25	0.64	0.84	1.25	0.57
RPA4	RPA4;HSU24186;MGC120333;MGC120334	replication protein A4, 30kDa	0.64	0.84	1.25	0.6
XDH	XDH;XO;XOR	xanthine dehydrogenase	0.64	0.79	1.25	0.68
CA3	CA3;CAIII;Car3;FLJ36434	carbonic anhydrase III, muscle specific	0.64	0.78	1.25	0.61
TMEM107	TMEM107;GRVS638;MGC10744;PRO1268	transmembrane protein 107	0.64	0.86	1.25	0.66
FRRS1	FRRS1;SDFR2;SDR2	ferric-chelate reductase 1	0.64	0.81	1.25	0.58
CNN3	CNN3	calponin 3, acidic	0.64	0.84	1.25	0.57
CATSPERG	CATSPERG;C19orf15;DKFZp434A1022;FLJ38899;FLJ46353;MGC125664	cation channel, sperm-associated, gamma	0.64	0.84	1.25	0.63
FAM159A	FAM159A;MGC52498;PRO7171;WWLS2783	family with sequence similarity 159, member A	0.63	0.8	1.25	0.58
SH3GLB2	SH3GLB2;KIAA1848;PP6569;PP9455	SH3-domain GRB2-like endophilin B2	0.63	0.79	1.25	0.54
OR4C6	OR4C6;OR11-138	olfactory receptor, family 4, subfamily C, member 6	0.62	0.85	1.25	0.63
MTL5	MTL5;CXCDC2;MTLT;TESMIN	metallothionein-like 5, testis-specific (tesmin)	0.62	0.74	1.25	0.53
MAPK11	MAPK11;P38B;P38BETA2;PRKM11;SAPK2;SAPK2B;p38-2;p38Beta	mitogen-activated protein kinase 11	0.62	0.84	1.25	0.59
SYNGAP1	SYNGAP1;DKFZp761G1421;KIAA1938;MRD5;RASA1;RASA5;SYNGAP	synaptic Ras GTPase activating protein 1	0.62	0.75	1.25	0.65
SETMAR	SETMAR;METNASE	SET domain and mariner transposase fusion gene	0.62	0.78	1.25	0.61
IL4R	IL4R;CD124;IL4RA	interleukin 4 receptor	0.62	0.86	1.25	0.6
CDCA3	CDCA3;GRCC8;MGC2577;TOME-1	cell division cycle associated 3	0.62	0.8	1.25	0.61
SPECC1L	SPECC1L;CYTSA;KIAA0376;OBLFC1	sperm antigen with calponin homology and coiled-coil domains 1-like	0.62	0.83	1.25	0.58
FAM153C	FAM153C;FLJ60013;NY-REN-7-like	family with sequence similarity 153, member C	0.61	0.8	1.25	0.56
IL6R	IL6R;CD126;IL-6R-1;IL-6RA;IL6RA;MGC104991;gp80	interleukin 6 receptor	0.61	0.82	1.25	0.61
C1orf55	C1orf55;FLJ35382;RP4-671D7.1;dJ671D7.1	chromosome 1 open reading frame 55	0.61	0.82	1.25	0.64
ARL6IP4	ARL6IP4;MGC814;SFRS20;SR-25;SRp25;SRrp37	ADP-ribosylation-like factor 6 interacting protein 4	1.2	1.01	0.81	0.68
CLCN2	CLCN2;ClC-2;CLC2;ECA2;ECA3;EGI11;EGI3;EGMA;EJM6;EJM8	chloride channel 2	1.14	0.98	0.81	0.76
SH2D6	SH2D6;FLJ35993	SH2 domain containing 6	1.12	0.98	0.81	0.66
HIST1H4E	HIST1H4E;H4/j;H4FJ	histone cluster 1, H4e	1.1	0.94	0.81	0.76
RABGAP1L	RABGAP1L;DKFZp686E1450;FLJ58037;FLJ79118;FLJ79254;HHL;TBC1D18	RAB GTPase activating protein 1-like	1.08	0.92	0.81	0.66
C19orf66	C19orf66;FLJ11286;FLJ40743	chromosome 19 open reading frame 66	1.07	0.88	0.81	0.7
PGP	PGP;MGC4692	phosphoglycolate phosphatase	1.07	0.88	0.81	0.7
BMPR1A	BMPR1A;10q23del;ACVRLK3;ALK3;CD292;SKR5	bone morphogenetic protein receptor, type IA	1.06	0.92	0.81	0.66
BGLAP	BGLAP;BGP;OC	bone gamma-carboxyglutamate (gla) protein	1.04	0.93	0.81	0.68

SLC36A4	SLC36A4;FLJ38932;PAT4	solute carrier family 36 (proton/amino acid symporter), member 4	1.04	0.94	0.81	0.65
GRIN2B	GRIN2B;MGC142178;MGC142180;MRD6;NMDAR2B;NR2B;hNR3	glutamate receptor, ionotropic, N-methyl D-aspartate 2B	1.03	0.88	0.81	0.68
NCK1	NCK1;MGC12668;NCK;NCKalpha;nck-1	NCK adaptor protein 1	1.02	0.89	0.81	0.67
SHB	SHB;RP11-3J10.8;bA3J10.2	Src homology 2 domain containing adaptor protein B	1	0.86	0.81	0.65
URB2	URB2;KIAA0133;MGC138837;MGC138838;NET10;NPA2	URB2 ribosome biogenesis 2 homolog (S. cerevisiae)	1	0.82	0.81	0.65
VCL	VCL;CMD1W;CMH15;MVCL	vinculin	0.98	0.8	0.81	0.53
ZBTB37	ZBTB37;D430004I08Rik;MGC2629;ZNF908	zinc finger and BTB domain containing 37	0.97	0.8	0.81	0.6
COL1A1	COL1A1;OI4	collagen, type I, alpha 1	0.96	0.85	0.81	0.62
DEGS2	DEGS2;C14orf66;DES2;FADS8	degenerative spermatocyte homolog 2, lipid desaturase (Drosophila)	0.96	0.8	0.81	0.62
REV1	REV1;FLJ21523;MGC163283;MGC26225;REV1L	REV1 homolog (S. cerevisiae)	0.95	0.78	0.81	0.6
BCL7A	BCL7A;BCL7	B-cell CLL/lymphoma 7A	0.94	0.78	0.81	0.59
NEUROG3	NEUROG3;Atoh5;Math4B;NGN-3;bHLHa7;ngn3	neurogenin 3	0.93	0.74	0.81	0.61
TPM4	TPM4	tropomyosin 4	0.92	0.74	0.81	0.55
PRORSD1P	PRORSD1P;NCRNA00117;PRDXDD1P;Prdxdd1;Ybakd1	prolyl-tRNA synthetase associated domain containing 1, pseudogene	0.92	0.78	0.81	0.55
KCNC2	KCNC2;KV3.2;MGC138196	potassium voltage-gated channel, Shaw-related subfamily, member 2	0.9	0.76	0.81	0.59
GNE	GNE;DMRV;GLCNE;IBM2;NM;Uae1	glucosamine (UDP-N-acetyl)-2-epimerase/N-acetylmannosamine kinase	0.89	0.78	0.81	0.55
ADSS	ADSS;ADEH;MGC20404	adenylosuccinate synthase	0.89	0.81	0.81	0.56
PEAK1	PEAK1;FLJ21140;FLJ34483;KIAA2002;SGK269	NKF3 kinase family member	0.89	0.77	0.81	0.55
POMZP3	POMZP3;MGC8359;POM-ZP3;POM121	POM121 and ZP3 fusion	0.88	0.75	0.81	0.53
KIAA0090	KIAA0090;RP1-43E13.1	KIAA0090	0.88	0.72	0.81	0.52
INPP5F	INPP5F;FLJ13081;KIAA0966;MGC131851;MGC59773;MSTPO47;SAC2;hSAC2	inositol polyphosphate-5-phosphatase F	0.87	0.68	0.81	0.48
SLC16A13	SLC16A13;MCT13	solute carrier family 16, member 13 (monocarboxylic acid transporter 13)	0.86	0.68	0.81	0.53
COX18	COX18;COX18HS;FLJ38991;MGC126733	COX18 cytochrome c oxidase assembly homolog (S. cerevisiae)	0.86	0.76	0.81	0.51
FCRLA	FCRLA;FCRL;FCRL1;FCRLM1;FCRLX;FCRLb;FCRLc1;FCRLc2;FCRLd;FCRLe;FCRX;FREB;MGC4595	Fc receptor-like A	1.29	1.13	0.82	0.79
KCNG4	KCNG4;KV6.3;KV6.4;MGC129609;MGC4558	potassium voltage-gated channel, subfamily G, member 4	1.23	1.06	0.82	0.82
EPS8	EPS8	epidermal growth factor receptor pathway substrate 8	1.2	1.03	0.82	0.8
TAF1	TAF1;BA2R;CCG1;CCGS;DYT3;DYT3/TAF1;KAT4;N-TAF1;NSCL2;OF;P250;TAF2A;TAFII250;XDP	TAF1 RNA polymerase II, TATA box binding protein (TBP)-associated factor, 250kDa	1.18	1.05	0.82	0.74
RAPGEF5	RAPGEF5;GFR;KIAA0277;MR-GEF;REPAC	Rap guanine nucleotide exchange factor (GEF) 5	1.17	1.06	0.82	0.73
STK32C	STK32C;MGC23665;PKE;RP11-140A10.1;YANK3	serine/threonine kinase 32C	1.15	1.01	0.82	0.73
SIRT6	SIRT6;SIR2L6	sirtuin 6	1.15	0.96	0.82	0.76
DNAJB9	DNAJB9;DKFZp564F1862;ERdj4;MDG-1;MDG1;MST049;MSTP049	DnaJ (Hsp40) homolog, subfamily B, member 9	1.15	0.97	0.82	0.73
FAM75A3	FAM75A3;DKFZp434B204;MGC181958	family with sequence similarity 75, member A3	1.15	1.03	0.82	0.69
MVK	MVK;FLJ96772;LRBP;MK;MVLK	mevalonate kinase	1.09	0.97	0.82	0.69
COIL	COIL;CLN80;p80-coilin	coilin	1.08	0.92	0.82	0.69
ACRC	ACRC;NAAR1	acidic repeat containing	1.07	0.91	0.82	0.69
CACNA1S	CACNA1S;CACNL1A3;CCHL1A3;Cav1.1;HOKPP;HOKPP1;MHS5;TTPP1;hypoPP	calcium channel, voltage-dependent, L type, alpha 1S subunit	1.07	0.91	0.82	0.71
TOE1	TOE1;FLJ13949	target of EGR1, member 1 (nuclear)	1.05	0.89	0.82	0.68
ZNF385D	ZNF385D;FLJ22419;ZNF659	zinc finger protein 385D	1.03	0.93	0.82	0.62
MAP3K10	MAP3K10;MEKK10;MLK2;MST	mitogen-activated protein kinase kinase kinase 10	1.03	0.92	0.82	0.65
ANKS4B	ANKS4B;FLJ38819;HARP;MGC133380;MGC133381	ankyrin repeat and sterile alpha motif domain containing 4B	1.02	0.9	0.82	0.69
ACBD5	ACBD5;DKFZp434A2417;KIAA1996	acyl-CoA binding domain containing 5	1.02	0.86	0.82	0.57
GRIN2A	GRIN2A;EPND;NMDAR2A;NR2A	glutamate receptor, ionotropic, N-methyl D-aspartate 2A	1.01	0.86	0.82	0.67
GALNTL4	GALNTL4;GALNT15;GALNT18;GalNAc-T15;MGC71806	UDP-N-acetyl-alpha-D-galactosamine:polypeptide N-acetylgalactosaminyltransferase-like 4	1	0.82	0.82	0.66
ZNF407	ZNF407;FLJ13839;FLJ20307;KIAA1703	zinc finger protein 407	0.98	0.82	0.82	0.59
YY1AP1	YY1AP1;FLJ10875;FLJ13914;HCCA1;HCCA2;YAP;YY1AP	YY1 associated protein 1	0.98	0.88	0.82	0.58
HNRNPUL1	HNRNPUL1;E1B-AP5;E1BAP5;FLJ12944;HNRPUL1	heterogeneous nuclear ribonucleoprotein U-like 1	0.98	0.84	0.82	0.6
SGK196	SGK196;FLJ23356;MGC126597	protein kinase-like protein SgK196	0.98	0.8	0.82	0.66
ACCN3	ACCN3;ASIC3;DRASIC;SLNAC1;TNaC1	amiloride-sensitive cation channel 3	0.97	0.85	0.82	0.66
KRTCAP3	KRTCAP3;KCP3;MGC126736;MGC126738;MRV222;PRO9898	keratinocyte associated protein 3	0.96	0.82	0.82	0.55
ANKRD13A	ANKRD13A;ANKRD13;NY-REN-25	ankyrin repeat domain 13A	0.96	0.84	0.82	0.56
VIPR2	VIPR2;FLJ16511;PACAP-R-3;PACAP-R3;SCZD16;VIP-R-2;VPAC2;VPAC2R;VPCAP2R	vasoactive intestinal peptide receptor 2	0.95	0.76	0.82	0.69
TPCN2	TPCN2;FLJ41094;SHEP10;TPC2	two pore segment channel 2	0.94	0.8	0.82	0.62
RAD51C	RAD51C;BROVCA3;FANCO;MGC104277;RAD51L2	RAD51 homolog C (S. cerevisiae)	0.94	0.76	0.82	0.52
PARP4	PARP4;ADPRTL1;PARP-4;PARPL;PH5P;VAULT3;VPARP;VWA5C;p193	poly (ADP-ribose) polymerase family, member 4	0.94	0.77	0.82	0.6
GSDMC	GSDMC;MLZE	gasdermin C	0.93	0.8	0.82	0.6
OTX2	OTX2;CPHD6;MCOPS5;MGC45000	orthodenticle homeobox 2	0.92	0.73	0.82	0.53
HCCS	HCCS;CCHL;DKFZp779I1858;MCOPS7	holocytochrome c synthase	0.92	0.76	0.82	0.59
TOMM20	TOMM20;KIAA0016;MAS20;MGC117367;MOM19;TOM20	translocase of outer mitochondrial membrane 20 homolog (yeast)	0.91	0.78	0.82	0.59
SPG21	SPG21;ACP33;GL010;MASPARDIN;MAST	spastic paraplegia 21 (autosomal recessive, Mast syndrome)	0.91	0.77	0.82	0.58
SLC25A21	SLC25A21;MGC126570;ODC;ODC1	solute carrier family 25 (mitochondrial oxodicarboxylate carrier), member 21	0.9	0.76	0.82	0.51
LYRM1	LYRM1;A211C6.1	LYR motif containing 1	0.9	0.78	0.82	0.6
LIN37	LIN37;F25965;MGC9505;ZK418.4;lin-37	lin-37 homolog (C. elegans)	0.9	0.78	0.82	0.56
GMCL1	GMCL1;BTBD13;GCL;GCL1	germ cell-less homolog 1 (Drosophila)	0.9	0.72	0.82	0.54
BLVRB	BLVRB;BVRB;FLR;MGC117413;SDR43U1	biliverdin reductase B (flavin reductase (NADPH))	0.9	0.78	0.82	0.59
ELOVL2	ELOVL2;FLJ20334;SSC2	ELOVL fatty acid elongase 2	0.89	0.76	0.82	0.62
LIMCH1	LIMCH1;DKFZp434I0312;DKFZp686A01247;DKFZp686B2470;DKFZp686G18243;DKFZp686G2094;DKFZp781C1754;DKFZp781I1455;LIMCH1A;LMO7B;MGC72127	LIM and calponin homology domains 1	0.88	0.7	0.82	0.51
DRG1	DRG1;DKFZp434N1827;NEDD3	developmentally regulated GTP binding protein 1	0.88	0.8	0.82	0.55
KIAA0895L	KIAA0895L	KIAA0895-like	0.88	0.76	0.82	0.54
MRPS18B	MRPS18B;C6orf14;DKFZp564H0223;HSPC183;HumanS18a;MRP-S18-2;MRPS18-2;PTD017;S18amt	mitochondrial ribosomal protein S18B	0.87	0.72	0.82	0.49
TTC15	TTC15;FLJ36862;FLJ44560;TRAPPC12	tetratricopeptide repeat domain 15	0.86	0.74	0.82	0.55
PRDX4	PRDX4;AOE37-2;PRX-4	peroxiredoxin 4	0.86	0.7	0.82	0.47
RPP30	RPP30;FLJ38491;TSG15	ribonuclease P/MRP 30kDa subunit	0.86	0.68	0.82	0.52
KHNYN	KHNYN;KIAA0323	KH and NYN domain containing	0.86	0.74	0.82	0.52
NUP210	NUP210;FLJ22389;GP210;KIAA0906;POM210	nucleoporin 210kDa	1.23	1.04	0.83	0.72
NME1	NME1;AWD;GAAD;NB;NBS;NDKA;NDPK-A;NDPKA;NM23;NM23-H1	non-metastatic cells 1, protein (NM23A) expressed in	1.21	1.08	0.83	0.77
MBTD1	MBTD1;FLJ20055;FLJ42067;MGC126785;SA49P01	mbt domain containing 1	1.19	1.04	0.83	0.85
PGK2	PGK2;PGKB;PGKPS;dJ417L20.2	phosphoglycerate kinase 2	1.15	1.02	0.83	0.72
RAMP2	RAMP2	receptor (G protein-coupled) activity modifying protein 2	1.15	1.02	0.83	0.67
TCEAL3	TCEAL3;MGC15737	transcription elongation factor A (SII)-like 3	1.15	0.98	0.83	0.73
NUDCD2	NUDCD2;DKFZp686E10109	NudC domain containing 2	1.12	1	0.83	0.76
LOXL4	LOXL4;FLJ21889;LOXC	lysyl oxidase-like 4	1.08	0.89	0.83	0.66
KLF7	KLF7;UKLF	Kruppel-like factor 7 (ubiquitous)	1.06	0.94	0.83	0.62
PCDHGA5	PCDHGA5;CDH-GAMMA-A5;ME3;MGC142020;PCDH-GAMMA-A5	protocadherin gamma subfamily A, 5	1.06	0.9	0.83	0.68
KRTAP10-11	KRTAP10-11;KAP10.11;KAP18.11;KRTAP18-11;KRTAP18.11	keratin associated protein 10-11	1.05	0.87	0.83	0.63
GGPS1	GGPS1;GGPPS;GGPPS1	geranylgeranyl diphosphate synthase 1	1.05	0.89	0.83	0.7
GMFB	GMFB;GMF	glia maturation factor, beta	1.05	0.92	0.83	0.64
KCNN3	KCNN3;KCa2.3;SK3;SKCA3;hSK3	potassium intermediate/small conductance calcium-activated channel, subfamily N, member 3	1.04	0.91	0.83	0.7
CDK4	CDK4;CMM3;MGC14458;PSK-J3	cyclin-dependent kinase 4	1.04	0.86	0.83	0.71
FIGNL2	FIGNL2	fidgetin-like 2	1.03	0.94	0.83	0.69
DAGLB	DAGLB;DAGLBETA;FLJ33624;FLJ33909;FLJ45532;KCCR13L	diacylglycerol lipase, beta	1.03	0.92	0.83	0.69
TYRO3	TYRO3;BYK;Dtk;FLJ16467;RSE;Sky;Tif	TYRO3 protein tyrosine kinase	1.02	0.84	0.83	0.7
STK16	STK16;FLJ39635;KRCT;MGC16211;MPSK;PKL12;TSF1	serine/threonine kinase 16	1.02	0.84	0.83	0.7
INPPL1	INPPL1;SHIP2	inositol polyphosphate phosphatase-like 1	1.02	0.84	0.83	0.65
MCOLN1	MCOLN1;MG-2;ML4;MLIV;MST080;TRP-ML1;TRPM-L1;TRPML1	mucolipin 1	1.02	0.88	0.83	0.69
HMGB1P1	HMGB1P1;HMG1L1;HMG1L7;HMGB1L1;dJ579F20.1	high mobility group box 1 pseudogene 1	1.02	0.86	0.83	0.68
SAT2	SAT2;SSAT2	spermidine/spermine N1-acetyltransferase family member 2	1.01	0.89	0.83	0.59
C17orf64	C17orf64	chromosome 17 open reading frame 64	1	0.9	0.83	0.6
CPXM1	CPXM1;CPX1;CPXM	carboxypeptidase X (M14 family), member 1	1	0.82	0.83	0.62
MRE11A	MRE11A;ATLD;HNGS1;MRE11;MRE11B	MRE11 meiotic recombination 11 homolog A (S. cerevisiae)	1	0.88	0.83	0.68
ABI3BP	ABI3BP;FLJ41743;FLJ41754;NESHBP;TARSH	ABI family, member 3 (NESH) binding protein	0.99	0.86	0.83	0.57
HOXA9	HOXA9;ABD-B;HOX1;HOX1.7;HOX1G;MGC1934	homeobox A9	0.99	0.86	0.83	0.67
MYLK4	MYLK4;SgK085	myosin light chain kinase family, member 4	0.98	0.86	0.83	0.62
TRIM58	TRIM58;BIA2;DKFZp434C091;DKFZp686N09198	tripartite motif containing 58	0.98	0.8	0.83	0.72
RPRD2	RPRD2;FLJ32145;KIAA0460	regulation of nuclear pre-mRNA domain containing 2	0.98	0.82	0.83	0.55
WNK1	WNK1;HSAN2;HSN2;KDP;KIAA0344;MGC163339;MGC163341;PRKWNK1;PSK;p65	WNK lysine deficient protein kinase 1	0.98	0.8	0.83	0.71
C5orf62	C5orf62;MGC117221;MGC126887;MGC126889;MST150;NID67	chromosome 5 open reading frame 62	0.98	0.84	0.83	0.66
SCN3B	SCN3B;HSA243396;SCNB3	sodium channel, voltage-gated, type III, beta	0.97	0.85	0.83	0.66
C17orf76-AS1	C17orf76-AS1;C17orf45;FLJ25777;MGC40157;NCRNA00188;TSAP19	C17orf76 antisense RNA 1 (non-protein coding)	0.97	0.8	0.83	0.58
NMBR	NMBR	neuromedin B receptor	0.96	0.78	0.83	0.7
NCR2	NCR2;CD336;LY95;NK-p44;NKP44;dJ149M18.1	natural cytotoxicity triggering receptor 2	0.96	0.86	0.83	0.6
TFPT	TFPT;FB1;INO80F;amida	TCF3 (E2A) fusion partner (in childhood Leukemia)	0.96	0.8	0.83	0.55
CSF2RB	CSF2RB;CD131;CDw131;IL3RB;IL5RB	colony stimulating factor 2 receptor, beta, low-affinity (granulocyte-macrophage)	0.94	0.87	0.83	0.6
MST1R	MST1R;CD136;CDw136;PTK8;RON	macrophage stimulating 1 receptor (c-met-related tyrosine kinase)	0.94	0.76	0.83	0.68
C9orf41	C9orf41;FLJ25795	chromosome 9 open reading frame 41	0.94	0.8	0.83	0.61
ZER1	ZER1;C9orf60;Hzyg;RP11-545E17.4;ZYG;ZYG11BL	zer-1 homolog (C. elegans)	0.94	0.82	0.83	0.58
C11orf24	C11orf24;DM4E3	chromosome 11 open reading frame 24	0.94	0.81	0.83	0.61
IVD	IVD;ACAD2;FLJ12715;FLJ34849	isovaleryl-CoA dehydrogenase	0.94	0.78	0.83	0.61
FUT1	FUT1;H;HH;HSC	fucosyltransferase 1 (galactoside 2-alpha-L-fucosyltransferase, H blood group)	0.93	0.74	0.83	0.58
ARX	ARX;CT121;EIEE1;ISSX;MRX29;MRX32;MRX33;MRX36;MRX38;MRX43;MRX54;MRX76;MRX87;MRXS1;PRTS	aristaless related homeobox	0.93	0.82	0.83	0.54
C6orf226	C6orf226	chromosome 6 open reading frame 226	0.92	0.8	0.83	0.56
TMEM35	TMEM35;FLJ14084	transmembrane protein 35	0.91	0.77	0.83	0.59
CCDC153	CCDC153;MGC125447;MGC125448	coiled-coil domain containing 153	0.91	0.76	0.83	0.51
CHRNA9	CHRNA9;HSA243342;MGC142109;MGC142135;NACHRA9	cholinergic receptor, nicotinic, alpha 9	0.9	0.79	0.83	0.61
SYNPO2	SYNPO2;DKFZp686G051	synaptopodin 2	0.9	0.72	0.83	0.58
PBLD	PBLD;FLJ14767;FLJ35507;MAWBP;MAWDBP	phenazine biosynthesis-like protein domain containing	0.9	0.8	0.83	0.62
SCNN1D	SCNN1D;ENaCd;ENaCdelta;MGC149710;MGC149711;SCNED;dNaCh	sodium channel, nonvoltage-gated 1, delta	0.9	0.74	0.83	0.58
STX7	STX7	syntaxin 7	0.9	0.78	0.83	0.58
ASPSCR1	ASPSCR1;ASPCR1;ASPL;ASPS;FLJ45380;RCC17;TUG;UBXD9;UBXN9	alveolar soft part sarcoma chromosome region, candidate 1	0.9	0.76	0.83	0.55
AP1G1	AP1G1;ADTG;CLAPG1;MGC18255	adaptor-related protein complex 1, gamma 1 subunit	0.9	0.76	0.83	0.51

SYNDIG1	SYNDIG1;C20orf39;FLJ14220;IFITMD5;TMEM90B	synapse differentiation inducing 1	0.9	0.76	0.83	0.61
DNAI1	DNAI1;CILD1;ICS;ICS1;MGC26204;PCD	dynein, axonemal, intermediate chain 1	0.89	0.76	0.83	0.6
EEFSEC	EEFSEC;EFSEC;SELB	eukaryotic elongation factor, selenocysteine-tRNA-specific	0.89	0.82	0.83	0.57
POLR1A	POLR1A;A190;DKFZp586M0122;FLJ21915;MGC87965;RPA1;RPA194;RPO1-4;RPO14	polymerase (RNA) I polypeptide A, 194kDa	0.89	0.82	0.83	0.56
FSIP1	FSIP1;FLJ35989	fibrous sheath interacting protein 1	0.88	0.76	0.83	0.54
ARL11	ARL11;ARLTS1;FLJ33930;MGC17429	ADP-ribosylation factor-like 11	0.88	0.74	0.83	0.53
ATP1B4	ATP1B4	ATPase, Na+/K+ transporting, beta 4 polypeptide	0.88	0.74	0.83	0.49
SLC25A41	SLC25A41;FLJ40442;MGC34725	solute carrier family 25, member 41	0.88	0.71	0.83	0.53
TFAP2A	TFAP2A;AP-2;AP-2alpha;AP2TF;BOFS;FLJ51761;TFAP2	transcription factor AP-2 alpha (activating enhancer binding protein 2 alpha)	0.88	0.8	0.83	0.56
NLRP8	NLRP8;CLR19.2;NALP8;NOD16;PAN4	NLR family, pyrin domain containing 8	0.86	0.72	0.83	0.57
HNMT	HNMT;HMT;HNMT-S1;HNMT-S2	histamine N-methyltransferase	0.86	0.7	0.83	0.52
APH1A	APH1A;6530402N02Rik;APH-1;APH-1A;CGI-78	anterior pharynx defective 1 homolog A (C. elegans)	0.86	0.73	0.83	0.55
38412	MARCH5;FLJ20445;MARCH-V;MITOL;RNF153	membrane-associated ring finger (C3HC4) 5	0.86	0.7	0.83	0.63
FNDC3B	FNDC3B;DKFZp686D14170;DKFZp762K137;FAD104;FLJ23399;MGC10002;PRO4979;YVTM2421	fibronectin type III domain containing 3B	0.86	0.68	0.83	0.57
USP1	USP1;UBP	ubiquitin specific peptidase 1	0.86	0.71	0.83	0.53
CYP26B1	CYP26B1;CYP26A2;DKFZp686G0638;MGC129613;P450RAI-2	cytochrome P450, family 26, subfamily B, polypeptide 1	0.85	0.68	0.83	0.49
FANCE	FANCE;FACE;FAE	Fanconi anemia, complementation group E	0.85	0.73	0.83	0.52
CLIC3	CLIC3	chloride intracellular channel 3	0.85	0.73	0.83	0.57
SBDS	SBDS;FLJ10917;SDS;SWDS	Shwachman-Bodian-Diamond syndrome	0.85	0.74	0.83	0.52
ANKHD1	ANKHD1;MASK;VBARP	ankyrin repeat and KH domain containing 1	0.85	0.73	0.83	0.49
ARL3	ARL3;ARFL3	ADP-ribosylation factor-like 3	1.29	1.12	0.84	0.85
CCDC132	CCDC132;FLJ20097;FLJ23581;KIAA1861;MGC176659	coiled-coil domain containing 132	1.26	1.08	0.84	0.81
BSND	BSND;BART;DFNB73;MGC119283;MGC119284;MGC119285	Bartter syndrome, infantile, with sensorineural deafness (Barttin)	1.22	1.07	0.84	0.83
RALGPS1	RALGPS1;KIAA0351;RALGEF2;RALGPS1A	Ral GEF with PH domain and SH3 binding motif 1	1.21	1.04	0.84	0.79
CLCN4	CLCN4;CLC4;ClC-4;ClC-4A;MGC163150	chloride channel 4	1.19	1.04	0.84	0.81
AP1M2	AP1M2;AP1-mu2;HSMU1B;MU-1B;MU1B;mu2	adaptor-related protein complex 1, mu 2 subunit	1.17	1.01	0.84	0.76
SLC12A4	SLC12A4;FLJ17069;FLJ40489;KCC1	solute carrier family 12 (potassium/chloride transporters), member 4	1.12	0.95	0.84	0.74
AGPAT4	AGPAT4;1-AGPAT4;LPAAT-delta;dJ473J16.2	1-acylglycerol-3-phosphate O-acyltransferase 4 (lysophosphatidic acid acyltransferase, delta)	1.09	0.92	0.84	0.62
IRF1	IRF1;IRF-1;MAR	interferon regulatory factor 1	1.09	0.98	0.84	0.65
FAM21A	FAM21A;bA56A21.1	family with sequence similarity 21, member A	1.09	0.96	0.84	0.71
SLC25A19	SLC25A19;DNC;MCPHA;MUP1;TPC	solute carrier family 25 (mitochondrial thiamine pyrophosphate carrier), member 19	1.08	0.9	0.84	0.66
MANSC1	MANSC1;9130403P13Rik;FLJ10298;LOH12CR3	MANSC domain containing 1	1.08	0.92	0.84	0.67
CSK	CSK;MGC117393	c-src tyrosine kinase	1.08	0.98	0.84	0.7
STK38L	STK38L;KIAA0965;NDR2	serine/threonine kinase 38 like	1.06	0.96	0.84	0.68
SPEG	SPEG;APEG-1;APEG1;BPEG;KIAA1297;MGC12676;SPEGalpha;SPEGbeta	SPEG complex locus	1.05	0.96	0.84	0.68
CACNB1	CACNB1;CAB1;CACNLB1;CCHLB1;MGC41896	calcium channel, voltage-dependent, beta 1 subunit	1.04	0.88	0.84	0.68
TRAPPC3	TRAPPC3;BET3	trafficking protein particle complex 3	1.04	0.93	0.84	0.65
DYNLT1	DYNLT1;CW-1;MGC111571;TCTEL1;tctex-1	dynein, light chain, Tctex-type 1	1.03	0.92	0.84	0.71
MRPL18	MRPL18;L18mt;MRP-L18	mitochondrial ribosomal protein L18	1.03	0.87	0.84	0.59
OTOP1	OTOP1;MGC163302;MGC163304	otopetrin 1	1.02	0.84	0.84	0.66
CDO1	CDO1	cysteine dioxygenase, type I	1.02	0.84	0.84	0.59
DGKG	DGKG;DAGK3;DGK-GAMMA;MGC104993;MGC133330	diacylglycerol kinase, gamma 90kDa	1.02	0.86	0.84	0.71
RAB14	RAB14;FBP;RAB-14	RAB14, member RAS oncogene family	1.02	0.86	0.84	0.58
CDKN2C	CDKN2C;INK4C;p18;p18-INK4C	cyclin-dependent kinase inhibitor 2C (p18, inhibits CDK4)	1.01	0.84	0.84	0.7
C9orf85	C9orf85;MGC61599	chromosome 9 open reading frame 85	1	0.86	0.84	0.66
GYLTL1B	GYLTL1B;FLJ35207;LARGE2	glycosyltransferase-like 1B	1	0.91	0.84	0.68
SRGAP2	SRGAP2;ARHGAP34;FNBP2;KIAA0456;SRGAP3	SLIT-ROBO Rho GTPase activating protein 2	1	0.82	0.84	0.62
SNAPC3	SNAPC3;MGC132011;MGC33124;PTFbeta;SNAP50	small nuclear RNA activating complex, polypeptide 3, 50kDa	1	0.82	0.84	0.69
KIR2DL5B	KIR2DL5B;KIR2DL5;KIR2DL5.2;KIR2DLX	killer cell immunoglobulin-like receptor, two domains, long cytoplasmic tail, 5B	1	0.82	0.84	0.69
C5orf20	C5orf20;DCNP1	chromosome 5 open reading frame 20	0.98	0.86	0.84	0.62
LHX3	LHX3;CPHD3;DKFZp762A2013;LIM3;M2-LHX3	LIM homeobox 3	0.98	0.88	0.84	0.58
CHMP6	CHMP6;FLJ11749;VPS20	charged multivesicular body protein 6	0.98	0.86	0.84	0.61
IL17F	IL17F;CANDF6;IL-17F;ML-1;ML1	interleukin 17F	0.97	0.86	0.84	0.61
C5orf62	C5orf62;MGC117221;MGC126887;MGC126889;MST150;NID67	chromosome 5 open reading frame 62	0.96	0.84	0.84	0.65
TIMP1	TIMP1;CLGI;EPA;EPO;FLJ90373;HCI;TIMP	TIMP metallopeptidase inhibitor 1	0.95	0.82	0.84	0.65
SYT6	SYT6	synaptotagmin VI	0.94	0.79	0.84	0.54
FAM115C	FAM115C;FAM139A;FLJ40722;MGC142090	family with sequence similarity 115, member C	0.94	0.82	0.84	0.62
E2F2	E2F2;E2F-2	E2F transcription factor 2	0.94	0.77	0.84	0.57
REV3L	REV3L;POLZ;REV3	REV3-like, catalytic subunit of DNA polymerase zeta (yeast)	0.94	0.75	0.84	0.59
CRYZ	CRYZ;DKFZp779E0834;FLJ41475	crystallin, zeta (quinone reductase)	0.94	0.76	0.84	0.59
CDK13	CDK13;CDC2L;CDC2L5;CHED;FLJ35215;KIAA1791;hCDK13	cyclin-dependent kinase 13	0.94	0.86	0.84	0.61
LEPROT	LEPROT;FLJ37482;FLJ90360;LEPR;OB-RGRP;OBRGRP;VPS55	leptin receptor overlapping transcript	0.94	0.77	0.84	0.58
SEMA3E	SEMA3E;KIAA0331;M-SEMAH;M-SemaK;SEMAH;coll-5	sema domain, immunoglobulin domain (Ig), short basic domain, secreted, (semaphorin) 3E	0.92	0.76	0.84	0.61
ALDH1A3	ALDH1A3;ALDH1A6;ALDH6;RALDH3	aldehyde dehydrogenase 1 family, member A3	0.92	0.78	0.84	0.52
ABHD11	ABHD11;WBSCR21	abhydrolase domain containing 11	0.92	0.76	0.84	0.56
DTNB	DTNB;MGC17163;MGC57126	dystrobrevin, beta	0.92	0.82	0.84	0.61
DENND5A	DENND5A;FLJ22354;FLJ33829;FLJ43455;KIAA1091;RAB6IP1	DENN/MADD domain containing 5A	0.92	0.8	0.84	0.61
ANKRD36B	ANKRD36B;FLJ21281;FLJ90089;KIAA1641;MGC149864;MGC149865;MGC167812	ankyrin repeat domain 36B	0.92	0.8	0.84	0.6
CLUL1	CLUL1;RA337M	clusterin-like 1 (retinal)	0.91	0.74	0.84	0.6
TMEM178	TMEM178;MGC33926	transmembrane protein 178	0.91	0.82	0.84	0.63
KCNC1	KCNC1;FLJ41162;FLJ42249;FLJ43491;KV3.1;KV4;MGC129855;NGK2	potassium voltage-gated channel, Shaw-related subfamily, member 1	0.9	0.8	0.84	0.62
STAT5A	STAT5A;MGF;STAT5	signal transducer and activator of transcription 5A	0.9	0.77	0.84	0.52
CDK5R2	CDK5R2;NCK5AI;P39;p39nck5ai	cyclin-dependent kinase 5, regulatory subunit 2 (p39)	0.9	0.75	0.84	0.62
REL	REL;C-Rel	v-rel reticuloendotheliosis viral oncogene homolog (avian)	0.9	0.73	0.84	0.57
SLC45A4	SLC45A4;KIAA1126	solute carrier family 45, member 4	0.9	0.8	0.84	0.57
COQ7	COQ7;CAT5;CLK-1;CLK1	coenzyme Q7 homolog, ubiquinone (yeast)	0.9	0.77	0.84	0.55
ACAD8	ACAD8;ACAD-8;ARC42;FLJ22590	acyl-CoA dehydrogenase family, member 8	0.9	0.71	0.84	0.56
SUB1	SUB1;MGC102747;P15;PC4;p14	SUB1 homolog (S. cerevisiae)	0.9	0.71	0.84	0.57
STAC	STAC;FLJ32331;STAC1	SH3 and cysteine rich domain	0.89	0.76	0.84	0.52
EFNA2	EFNA2;ELF-1;EPLG6;HEK7-L;LERK-6;LERK6	ephrin-A2	0.89	0.8	0.84	0.53
SOD1	SOD1;ALS;ALS1;IPOA;SOD;hSod1;homodimer	superoxide dismutase 1, soluble	0.89	0.74	0.84	0.55
C7orf74	C7orf74;FLJ11436;FLJ25556;FLJ36031;MGC16181	chromosome 7 open reading frame 74	0.89	0.74	0.84	0.55
TTI1	TTI1;KIAA0406;smg-10	Tel2 interacting protein 1 homolog (S. pombe)	0.89	0.78	0.84	0.55
OR52K1	OR52K1;OR11-8	olfactory receptor, family 52, subfamily K, member 1	0.88	0.74	0.84	0.5
DNAH1	DNAH1;DNAHC1;HDHC7;HL-11;HL11;XLHSRF-1	dynein, axonemal, heavy chain 1	0.88	0.78	0.84	0.63
CABP7	CABP7;CALN2;MGC57793	calcium binding protein 7	0.88	0.76	0.84	0.58
KRTAP10-9	KRTAP10-9;KAP10.9;KAP18.9;KRTAP18-9	keratin associated protein 10-9	0.88	0.72	0.84	0.54
MLL4	MLL4;HRX2;KIAA0304;MLL2;TRX2;WBP7	myeloid/lymphoid or mixed-lineage leukemia 4	0.88	0.74	0.84	0.66
C15orf39	C15orf39;DKFZp434H132;FLJ46337;MGC117209	chromosome 15 open reading frame 39	0.88	0.72	0.84	0.55
TMEM158	TMEM158;BBP;DKFZp586E1621;RIS1;p40BBP	transmembrane protein 158 (gene/pseudogene)	0.88	0.74	0.84	0.54
ITGA2	ITGA2;BDPLT9;BR;CD49B;GPIa;VLA-2;VLAA2	integrin, alpha 2 (CD49B, alpha 2 subunit of VLA-2 receptor)	0.87	0.77	0.84	0.51
CHAC2	CHAC2	ChaC, cation transport regulator homolog 2 (E. coli)	0.86	0.74	0.84	0.53
OAS1	OAS1;IFI-4;OIAS;OIASI	2'-5'-oligoadenylate synthetase 1, 40/46kDa	0.86	0.74	0.84	0.59
RASA4	RASA4;CAPRI;FLJ59070;GAPL;KIAA0538;MGC131890	RAS p21 protein activator 4	0.86	0.7	0.84	0.6
BIN3	BIN3;MGC14978	bridging integrator 3	0.86	0.74	0.84	0.56
NUP188	NUP188;FLJ21639;KIAA0169	nucleoporin 188kDa	0.86	0.76	0.84	0.53
TMEM173	TMEM173;ERIS;FLJ38577;MITA;MPYS;NET23;STING	transmembrane protein 173	0.86	0.78	0.84	0.52
EEA1	EEA1;MST105;MSTP105;ZFYVE2	early endosome antigen 1	0.85	0.7	0.84	0.63
MTHFD2	MTHFD2;NMDMC	methylenetetrahydrofolate dehydrogenase (NADP+ dependent) 2, methenyltetrahydrofolate cyclohydrolase	0.85	0.78	0.84	0.54
CYP7B1	CYP7B1;CBAS3;CP7B;SPG5A	cytochrome P450, family 7, subfamily B, polypeptide 1	1.38	1.23	0.85	0.95
C8orf86	C8orf86;FLJ43582	chromosome 8 open reading frame 86	1.28	1.12	0.85	0.84
BBS9	BBS9;B1;C18;D1;MGC118917;PTHB1	Bardet-Biedl syndrome 9	1.25	1.18	0.85	0.86
GLRB	GLRB	glycine receptor, beta	1.25	1.12	0.85	0.86
ABCF1	ABCF1;ABC27;ABC50	ATP-binding cassette, sub-family F (GCN20), member 1	1.24	1.06	0.85	0.82
ACCN4	ACCN4;ASIC4;BNAC4;MGC17248;MGC24860	amiloride-sensitive cation channel 4, pituitary	1.23	1.04	0.85	0.81
SETD1A	SETD1A;KIAA0339;KMT2F;Set1;Set1A	SET domain containing 1A	1.22	1.1	0.85	0.86
ELAVL1	ELAVL1;ELAV1;HUR;Hua;MelG	ELAV (embryonic lethal, abnormal vision, Drosophila)-like 1 (Hu antigen R)	1.21	1.04	0.85	0.8
HSF2BP	HSF2BP	heat shock transcription factor 2 binding protein	1.19	1.06	0.85	0.86
TECTA	TECTA;DFNA12;DFNA8;DFNB21	tectorin alpha	1.18	1	0.85	0.67
TMCO1	TMCO1;CFSMR;HP10122;PCIA3;PNAS-136;RP11-466F5.7;TMCC4	transmembrane and coiled-coil domains 1	1.18	1.05	0.85	0.75
KCTD2	KCTD2;KIAA0176	potassium channel tetramerisation domain containing 2	1.17	0.99	0.85	0.77
PAPPA2	PAPPA2;PAPP-A2;PAPPE;PLAC3	pappalysin 2	1.13	0.94	0.85	0.71
GDE1	GDE1;363E6.2;MIR16	glycerophosphodiester phosphodiesterase 1	1.13	1.02	0.85	0.67
UQCRFS1	UQCRFS1;RIP1;RIS1;RISP;UQCR5	ubiquinol-cytochrome c reductase, Rieske iron-sulfur polypeptide 1	1.11	0.92	0.85	0.65
HMGCS1	HMGCS1;HMGCS;MGC90332	3-hydroxy-3-methylglutaryl-CoA synthase 1 (soluble)	1.1	0.92	0.85	0.69
ELK3	ELK3;ERP;NET;SAP2	ELK3, ETS-domain protein (SRF accessory protein 2)	1.09	0.9	0.85	0.64
GNPDA1	GNPDA1;GNP1;GNPDA;GNPI;GPI;HLN;KIAA0060	glucosamine-6-phosphate deaminase 1	1.09	0.92	0.85	0.68
PANK3	PANK3;DKFZp686O22247;FLJ12899;MGC16863	pantothenate kinase 3	1.09	1	0.85	0.71
PRKCZ	PRKCZ;PKC-ZETA;PKC2	protein kinase C, zeta	1.08	0.92	0.85	0.76
TRIP10	TRIP10;CIP4;HSTP;STOT;STP	thyroid hormone receptor interactor 10	1.08	0.89	0.85	0.68
MYL10	MYL10;MYLC2PL;PLRLC	myosin, light chain 10, regulatory	1.08	0.95	0.85	0.71
SSX4	SSX4;CT5.4;MGC119056;MGC12411;SSX4B	synovial sarcoma, X breakpoint 4	1.07	0.88	0.85	0.65
SLC35F4	SLC35F4;C14orf36;FLJ37712;c14_5373	solute carrier family 35, member F4	1.06	0.94	0.85	0.7
KCNE1L	KCNE1L;KCNE5	KCNE1-like	1.06	0.95	0.85	0.74
CTH	CTH;MGC9471	cystathionase (cystathionine gamma-lyase)	1.06	0.87	0.85	0.63
CDK5	CDK5;PSSALRE	cyclin-dependent kinase 5	1.06	0.98	0.85	0.7
THOC3	THOC3;MGC5469;TEX1	THO complex 3	1.06	0.88	0.85	0.7
RNF167	RNF167;5730408C10Rik;DKFZp566H073;RING105	ring finger protein 167	1.05	0.9	0.85	0.67
ADAMTS18	ADAMTS18;ADAMTS21;KNO2	ADAM metallopeptidase with thrombospondin type 1 motif, 18	1.04	0.88	0.85	0.66

TESC	TESC;CHP3;FLJ20607;TSC	tescalcin	1.04	0.86	0.85	0.65
H6PD	H6PD;DKFZp686A01246;G6PDH;GDH;MGC87643	hexose-6-phosphate dehydrogenase (glucose 1-dehydrogenase)	1.04	0.89	0.85	0.6
GADD45GIP1	GADD45GIP1;CKBBP2;CRIF1;MGC4667;MGC4758;PLINP-1;PRG6;Plinp1	growth arrest and DNA-damage-inducible, gamma interacting protein 1	1.04	0.92	0.85	0.7
SAR1B	SAR1B;ANDD;CMRD;GTBPB;SARA2	SAR1 homolog B (S. cerevisiae)	1.04	0.86	0.85	0.64
IFT27	IFT27;RABL4;RAYL	intraflagellar transport 27 homolog (Chlamydomonas)	1.04	0.9	0.85	0.6
DAB1	DAB1	disabled homolog 1 (Drosophila)	1.03	0.96	0.85	0.67
EGR1	EGR1;AT225;G0S30;KROX-24;NGFI-A;TIS8;ZIF-268;ZNF225	early growth response 1	1.02	0.94	0.85	0.7
FXC1	FXC1;TIM10B;TIMM10B;Tim9b	fracture callus 1 homolog (rat)	1.02	0.87	0.85	0.59
IGFBP6	IGFBP6;IBP6	insulin-like growth factor binding protein 6	1.02	0.87	0.85	0.59
YIPF5	YIPF5;DKFZp313L2216;FinGER5;SB140;SMAP-5;SMAP5;YIP1A	Yip1 domain family, member 5	1.02	0.88	0.85	0.6
CPNE8	CPNE8;MGC129645;MGC129646	copine VIII	1	0.9	0.85	0.63
HCRTR2	HCRTR2;OX2R	hypocretin (orexin) receptor 2	1	0.83	0.85	0.75
AGFG2	AGFG2;HRBL;RABR	ArfGAP with FG repeats 2	1	0.87	0.85	0.66
ERVW-1	ERVW-1;ENV;ENVW;ERVWE1;HERV-7q;HERV-W-ENV;HERV7Q;HERVW;HERVWENV	endogenous retrovirus group W, member 1	1	0.88	0.85	0.66
ARHGEF17	ARHGEF17;FLJ90019;KIAA0337;P164RHOGEF;TEM4;p164-RhoGEF	Rho guanine nucleotide exchange factor (GEF) 17	0.98	0.85	0.85	0.67
SCHIP1	SCHIP1;FLJ39160;SCHIP-1	schwannomin interacting protein 1	0.98	0.83	0.85	0.58
RNASE4	RNASE4;MGC9306;RNS4	ribonuclease, RNase A family, 4	0.97	0.88	0.85	0.66
KCNH1	KCNH1;EAG;EAG1;Kv10.1;MGC142269;h-eag	potassium voltage-gated channel, subfamily H (eag-related), member 1	0.96	0.82	0.85	0.63
LHX9	LHX9	LIM homeobox 9	0.96	0.78	0.85	0.56
FEZ1	FEZ1	fasciculation and elongation protein zeta 1 (zygin I)	0.96	0.79	0.85	0.6
MUTYH	MUTYH;CYP2C;MGC4416;MYH	mutY homolog (E. coli)	0.96	0.81	0.85	0.6
TMEM14A	TMEM14A;C6orf73	transmembrane protein 14A	0.96	0.84	0.85	0.58
EBPL	EBPL;EBRP	emopamil binding protein-like	0.96	0.79	0.85	0.58
OR6T1	OR6T1;OR11-277	olfactory receptor, family 6, subfamily T, member 1	0.94	0.82	0.85	0.63
KCNA10	KCNA10;Kcn1;Kv1.8	potassium voltage-gated channel, shaker-related subfamily, member 10	0.94	0.84	0.85	0.65
RBKS	RBKS;DKFZp686G13268;RBSK	ribokinase	0.94	0.86	0.85	0.61
RIMS4	RIMS4;C20orf190;RIM 4;RIM-4;RIM4;RIM4-gamma;RIM4gamma	regulating synaptic membrane exocytosis 4	0.94	0.82	0.85	0.62
NUP50	NUP50;MGC39961;NPAP60;NPAP60L	nucleoporin 50kDa	0.94	0.78	0.85	0.59
ARV1	ARV1	ARV1 homolog (S. cerevisiae)	0.94	0.76	0.85	0.64
IPO8	IPO8;FLJ21954;FLJ26580;RANBP8	importin 8	0.94	0.79	0.85	0.66
ATP11A	ATP11A;ATPIH;ATPIS	ATPase, class VI, type 11A	0.94	0.8	0.85	0.54
CCZ1	CCZ1;C7orf28A;H_DJ1163J12.2	CCZ1 vacuolar protein trafficking and biogenesis associated homolog (S. cerevisiae)	0.94	0.85	0.85	0.69
STRADB	STRADB;ALS2CR2;CALS-21;ILPIP;ILPIPA;MGC102916;PAPK	STE20-related kinase adaptor beta	0.94	0.8	0.85	0.66
ZNF177	ZNF177;PIGX	zinc finger protein 177	0.92	0.79	0.85	0.55
C1orf111	C1orf111;RP11-565P22.3	chromosome 1 open reading frame 111	0.92	0.86	0.85	0.63
NANOS2	NANOS2;NOS2	nanos homolog 2 (Drosophila)	0.92	0.86	0.85	0.63
SPRYD4	SPRYD4;DKFZp686N0877	SPRY domain containing 4	0.92	0.86	0.85	0.57
YBX2	YBX2;CONTRIN;CSDA3;DBPC;MGC45104;MSY2	Y box binding protein 2	0.92	0.76	0.85	0.65
QTRT1	QTRT1;FP3235;TGT	queuine tRNA-ribosyltransferase 1	0.92	0.82	0.85	0.63
UFM1	UFM1;BM-002;C13orf20;bA131P10.1	ubiquitin-fold modifier 1	0.92	0.8	0.85	0.61
ARHGDIB	ARHGDIB;D4;GDIA2;GDID4;LYGDI;Ly-GDI;RAP1GN1;RhoGDI2	Rho GDP dissociation inhibitor (GDI) beta	0.91	0.78	0.85	0.61
OR51G2	OR51G2;OR11-28	olfactory receptor, family 51, subfamily G, member 2	0.9	0.75	0.85	0.57
MYO1A	MYO1A;BBMI;DFNA48;MIHC;MYHL	myosin IA	0.9	0.85	0.85	0.59
ZC3H6	ZC3H6;FLJ16526;FLJ41410;FLJ45877;KIAA2035;ZC3HDC6	zinc finger CCCH-type containing 6	0.9	0.78	0.85	0.59
ARTN	ARTN;ENOVIN;EVN;NBN	artemin	0.9	0.76	0.85	0.63
C15orf29	C15orf29;FLJ22557;MGC117356	chromosome 15 open reading frame 29	0.9	0.73	0.85	0.59
CDCA7	CDCA7;FLJ14722;FLJ14736;JPO1;MGC34109	cell division cycle associated 7	0.9	0.8	0.85	0.6
FASN	FASN;FAS;MGC14367;MGC15706;OA-519;SDR27X1	fatty acid synthase	0.9	0.71	0.85	0.57
CPT1A	CPT1A;CPT1;CPT1-L;L-CPT1	carnitine palmitoyltransferase 1A (liver)	0.9	0.75	0.85	0.53
ZFP28	ZFP28;DKFZp686K03205;KIAA1431;MGC104540;mkr5	zinc finger protein 28 homolog (mouse)	0.89	0.74	0.85	0.62
RPS6KA1	RPS6KA1;HU-1;MAPKAPK1A;RSK;RSK1	ribosomal protein S6 kinase, 90kDa, polypeptide 1	0.89	0.82	0.85	0.58
C19orf23	C19orf23;MGC39338	chromosome 19 open reading frame 23	0.88	0.76	0.85	0.51
ZNF607	ZNF607;FLJ14802;MGC13071	zinc finger protein 607	0.88	0.72	0.85	0.55
PIM3	PIM3;pim-3	pim-3 oncogene	0.88	0.81	0.85	0.57
EHD4	EHD4;PAST4	EH-domain containing 4	0.88	0.72	0.85	0.52
GAST	GAST;GAS	gastrin	0.87	0.77	0.85	0.55
XPO7	XPO7;EXP7;KIAA0745;RANBP16	exportin 7	0.87	0.75	0.85	0.51
KIR3DL1	KIR3DL1;CD158E1;KIR;KIR3DL1/S1;MGC119726;MGC119728;MGC126589;MGC126591;NKAT3;NKB1;NKB1B	killer cell immunoglobulin-like receptor, three domains, long cytoplasmic tail, 1	0.86	0.75	0.85	0.57
EEPD1	EEPD1;HSPC107;KIAA1706	endonuclease/exonuclease/phosphatase family domain containing 1	0.86	0.7	0.85	0.57
PITX2	PITX2;ARP1;Brx1;IDG2;IGDS;IGDS2;IHG2;IRID2;MGC111022;MGC20144;Otlx2;PTX2;RGS;RIEG;RIEG1;RS	paired-like homeodomain 2	0.86	0.78	0.85	0.51
ZNF275	ZNF275	zinc finger protein 275	0.86	0.73	0.85	0.51
SLIT3	SLIT3;FLJ10764;MEGF5;SLIL2;SLIT1;Slit-3;slit2	slit homolog 3 (Drosophila)	0.86	0.74	0.85	0.5
TMEM205	TMEM205;MGC110858;MGC3205;UNQ501	transmembrane protein 205	0.86	0.73	0.85	0.49
SACM1L	SACM1L;DKFZp686A0231;KIAA0851;SAC1	SAC1 suppressor of actin mutations 1-like (yeast)	0.86	0.74	0.85	0.58
OR10S1	OR10S1;OR11-279	olfactory receptor, family 10, subfamily S, member 1	0.85	0.72	0.85	0.49
C6orf125	C6orf125;M19;MGC14833;bA6B20.2	chromosome 6 open reading frame 125	0.85	0.75	0.85	0.57
TRPM2	TRPM2;EREG1;KNP3;LTRPC2;MGC133383;NUDT9H;NUDT9L1;TRPC7	transient receptor potential cation channel, subfamily M, member 2	1.31	1.17	0.86	0.91
LCE4A	LCE4A;LEP8;SPRL4A	late cornified envelope 4A	1.3	1.15	0.86	0.91
MEGF9	MEGF9;EGFL5	multiple EGF-like-domains 9	1.25	1.07	0.86	0.77
TIMM8A	TIMM8A;DDP;DDP1;DFN1;MGC12262;MTS;TIM8	translocase of inner mitochondrial membrane 8 homolog A (yeast)	1.22	1.03	0.86	0.73
GTF2A1	GTF2A1;MGC129969;MGC129970;TF2A1;TFIIA	general transcription factor IIA, 1, 19/37kDa	1.19	1.06	0.86	0.83
DLG3	DLG3;KIAA1232;MRX;MRX90;NEDLG;SAP102;XLMR	discs, large homolog 3 (Drosophila)	1.18	1.1	0.86	0.78
AK7	AK7;FLJ32864	adenylate kinase 7	1.16	0.99	0.86	0.82
PIGF	PIGF;MGC32646;MGC33136	phosphatidylinositol glycan anchor biosynthesis, class F	1.14	1	0.86	0.8
GEMIN5	GEMIN5;DKFZp586M1824;GEMIN-5;MGC142174	gem (nuclear organelle) associated protein 5	1.13	0.94	0.86	0.74
PTPRU	PTPRU;FLJ37530;FMI;PCP-2;PTP;PTP-J;PTP-PI;PTP-RO;PTPPSI;PTPRO;PTPU2;R-PTP-PSI;R-PTP-U;hPTP-J	protein tyrosine phosphatase, receptor type, U	1.12	0.97	0.86	0.75
PPP4R1	PPP4R1;PP4(Rmeg);PP4R1	protein phosphatase 4, regulatory subunit 1	1.11	0.94	0.86	0.73
GPC2	GPC2;DKFZp547M109;FLJ38962	glypican 2	1.1	0.94	0.86	0.78
YY2	YY2;ZNF631	YY2 transcription factor	1.1	0.96	0.86	0.67
HOOK1	HOOK1;HK1;MGC10642	hook homolog 1 (Drosophila)	1.1	0.96	0.86	0.66
CER1	CER1;DAND4;MGC119894;MGC119895;MGC96951	cerberus 1, cysteine knot superfamily, homolog (Xenopus laevis)	1.09	0.98	0.86	0.74
C12orf12	C12orf12;MGC26598	chromosome 12 open reading frame 12	1.09	0.99	0.86	0.71
PAPSS1	PAPSS1;ATPSK1;PAPSS;SK1	3'-phosphoadenosine 5'-phosphosulfate synthase 1	1.09	0.93	0.86	0.77
BCLAF1	BCLAF1;BTF;KIAA0164;bK211L9.1	BCL2-associated transcription factor 1	1.09	1	0.86	0.77
ACAN	ACAN;AGC1;AGCAN;CSPG1;CSPGCP;MSK16;SEDK	aggrecan	1.08	0.89	0.86	0.69
PI4K2A	PI4K2A;DKFZp761G1923;PI4KII;PIK42A;RP11-548K23.6	phosphatidylinositol 4-kinase type 2 alpha	1.08	0.92	0.86	0.77
YIF1A	YIF1A;54TM;FinGER7;YIF1;YIF1P	Yip1 interacting factor homolog A (S. cerevisiae)	1.07	0.92	0.86	0.66
SGCG	SGCG;A4;DAGA4;DMDA;DMDA1;LGMD2C;MAM;MGC130048;SCARMD2;SCG3;TYPE	sarcoglycan, gamma (35kDa dystrophin-associated glycoprotein)	1.06	0.87	0.86	0.67
C9orf71	C9orf71;MGC34760;RP11-274B18.1	chromosome 9 open reading frame 71	1.06	0.9	0.86	0.68
XRN1	XRN1;DKFZp434P0721;DKFZp686B22225;DKFZp686F19113;FLJ41903;SEP1	5'-3' exoribonuclease 1	1.05	0.88	0.86	0.63
MNAT1	MNAT1;CAP35;MAT1;RNF66;TFB3	menage a trois homolog 1, cyclin H assembly factor (Xenopus laevis)	1.04	0.89	0.86	0.8
ARNT	ARNT;HIF-1-beta;HIF-1beta;HIF1-beta;HIF1B;HIF1BETA;TANGO;bHLHe2	aryl hydrocarbon receptor nuclear translocator	1.04	0.86	0.86	0.62
MYL6	MYL6;ESMLC;LC17;LC17-GI;LC17-NM;LC17A;LC17B;MLC-3;MLC1SM;MLC3NM;MLC3SM	myosin, light chain 6, alkali, smooth muscle and non-muscle	1.04	0.89	0.86	0.69
ENDOV	ENDOV;FLJ35220;MGC74455	endonuclease V	1.04	0.87	0.86	0.7
CHRNA4	CHRNA4;BFNC;EBN;EBN1;FLJ95812;NACHR;NACHRA4;NACRA4	cholinergic receptor, nicotinic, alpha 4	1.03	0.93	0.86	0.72
NME3	NME3;DR-nm23;KIAA0516;NDPK-C;NDPKC;NM23-H3;NM23H3;c371H6.2	non-metastatic cells 3, protein expressed in	1.02	0.87	0.86	0.72
NPPB	NPPB;BNP	natriuretic peptide B	1.02	0.85	0.86	0.63
RBM33	RBM33;DKFZp434D1319;MGC20460;PRR8	RNA binding motif protein 33	1.02	0.92	0.86	0.66
UGT8	UGT8;CGT;UGT4	UDP glycosyltransferase 8	1.02	0.88	0.86	0.59
TWF1	TWF1;A6;MGC23788;MGC41876;PTK9	twinfilin, actin-binding protein, homolog 1 (Drosophila)	1.02	0.86	0.86	0.72
NAE1	NAE1;A-116A10.1;APPBP1;ula-1	NEDD8 activating enzyme E1 subunit 1	1.01	0.84	0.86	0.6
APOM	APOM;G3a;HSPC336;MGC22400;NG20	apolipoprotein M	1	0.86	0.86	0.58
LPXN	LPXN;LDPL	leupaxin	1	0.86	0.86	0.77
SCAND1	SCAND1;RAZ1;SDP1	SCAN domain containing 1	1	0.91	0.86	0.64
C17orf61	C17orf61;MGC40107	chromosome 17 open reading frame 61	1	0.9	0.86	0.64
SLC12A9	SLC12A9;CIP1;FLJ46905	solute carrier family 12 (potassium/chloride transporters), member 9	1	0.96	0.86	0.67
MICU1	MICU1;CALC;CBARA1;DKFZp564C246;EFHA3;FLJ12684	mitochondrial calcium uptake 1	1	0.81	0.86	0.68
CD3D	CD3D;CD3-DELTA;T3D	CD3d molecule, delta (CD3-TCR complex)	0.99	0.81	0.86	0.58
CHGB	CHGB;SCG1	chromogranin B (secretogranin 1)	0.99	0.88	0.86	0.67
FBXL5	FBXL5;FBL4;FBL5;FLR1	F-box and leucine-rich repeat protein 5	0.99	0.82	0.86	0.59
GFPT2	GFPT2;FLJ10380;GFAT2	glutamine-fructose-6-phosphate transaminase 2	0.98	0.86	0.86	0.69
MRFAP1L1	MRFAP1L1;MGC9651	Morf4 family associated protein 1-like 1	0.98	0.89	0.86	0.64
DEDD	DEDD;CASP8IP1;DEDD1;DEFT;FLDED1	death effector domain containing	0.98	0.9	0.86	0.63
PCDHA7	PCDHA7;CNR4;CNRN4;CNRS4;CRNR4;PCDH-ALPHA7	protocadherin alpha 7	0.98	0.86	0.86	0.58
SIGLEC10	SIGLEC10;MGC126774;PRO940;SIGLEC-10;SLG2	sialic acid binding Ig-like lectin 10	0.97	0.8	0.86	0.58
CADM3	CADM3;BIgR;FLJ10698;IGSF4B;NECL1;Necl-1;TSLL1;synCAM3	cell adhesion molecule 3	0.97	0.87	0.86	0.62
FLRT3	FLRT3	fibronectin leucine rich transmembrane protein 3	0.97	0.82	0.86	0.62
FAM109A	FAM109A;FLJ32356;IPIP27A;SES1	family with sequence similarity 109, member A	0.96	0.89	0.86	0.59
ITPRIP	ITPRIP;DANGER;KIAA1754;bA127L20;bA127L20.2	inositol 1,4,5-trisphosphate receptor interacting protein	0.96	0.86	0.86	0.61
THUMPD2	THUMPD2;C2orf8;MGC2454	THUMP domain containing 2	0.96	0.78	0.86	0.62
RRM2B	RRM2B;DKFZp686M05248;MGC102856;MGC42116;MTDPS8A;MTDPS8B;P53R2	ribonucleotide reductase M2 B (TP53 inducible)	0.96	0.92	0.86	0.64
CLDN12	CLDN12	claudin 12	0.96	0.84	0.86	0.6
TFAM	TFAM;MtTF1;TCF6;TCF6L1;TCF6L2;TCF6L3;mtTFA	transcription factor A, mitochondrial	0.96	0.79	0.86	0.57
UBA2	UBA2;ARX;FLJ13058;SAE2	ubiquitin-like modifier activating enzyme 2	0.96	0.84	0.86	0.67
C6orf27	C6orf27;FLJ25524;G7c;NG37	chromosome 6 open reading frame 27	0.95	0.8	0.86	0.6
CTSE	CTSE;CATE	cathepsin E	0.94	0.8	0.86	0.6
BUB1	BUB1;BUB1A;BUB1L;hBUB1	budding uninhibited by benzimidazoles 1 homolog (yeast)	0.94	0.8	0.86	0.67
C10orf71	C10orf71;FLJ45913	chromosome 10 open reading frame 71	0.93	0.82	0.86	0.65
SLC16A12	SLC16A12;CJMG;DKFZp686E188;MCT12	solute carrier family 16, member 12 (monocarboxylic acid transporter 12)	0.93	0.82	0.86	0.65

CYP4F11	CYP4F11;CYPIVF11	cytochrome P450, family 4, subfamily F, polypeptide 11	0.93	0.75	0.86	0.59
CALM3	CALM3;CALM1;CALM2;PHKD;PHKD3	calmodulin 3 (phosphorylase kinase, delta)	0.93	0.79	0.86	0.66
SCAF8	SCAF8;KIAA1116;RBM16	SR-related CTD-associated factor 8	0.93	0.83	0.86	0.62
OR51A4	OR51A4	olfactory receptor, family 51, subfamily A, member 4	0.92	0.76	0.86	0.6
NEB	NEB;DKFZp686C1456;FLJ11505;FLJ36536;FLJ39568;FLJ39584;NEB177D;NEM2	nebulin	0.92	0.86	0.86	0.63
GIP	GIP	gastric inhibitory polypeptide	0.92	0.82	0.86	0.54
FEZF2	FEZF2;FEZ;FEZL;FLJ10142;TOF;ZFP312;ZNF312	FEZ family zinc finger 2	0.92	0.77	0.86	0.58
CASKIN1	CASKIN1;ANKS5A	CASK interacting protein 1	0.92	0.83	0.86	0.62
FARP1	FARP1;CDEP;MGC87400;PLEKHC2;PPP1R75	FERM, RhoGEF (ARHGEF) and pleckstrin domain protein 1 (chondrocyte-derived)	0.92	0.77	0.86	0.64
ARFGEF1	ARFGEF1;ARFGEP1;BIG1;DKFZP434L057;P200	ADP-ribosylation factor guanine nucleotide-exchange factor 1 (brefeldin A-inhibited)	0.92	0.84	0.86	0.59
GBA3	GBA3;CBGL1;GLUC;KLrP;MGC104276;MGC126878	glucosidase, beta, acid 3 (cytosolic)	0.9	0.85	0.86	0.59
C10orf137	C10orf137;EDRF1;MGC125705	chromosome 10 open reading frame 137	0.9	0.8	0.86	0.55
HSPA4	HSPA4;APG-2;HS24/P52;HSPH2;MGC131852;RY;hsp70;hsp70RY	heat shock 70kDa protein 4	0.9	0.74	0.86	0.58
CCDC120	CCDC120	coiled-coil domain containing 120	0.89	0.76	0.86	0.55
STAC3	STAC3;MGC2793	SH3 and cysteine rich domain 3	0.88	0.77	0.86	0.52
TMSB4Y	TMSB4Y;MGC26307;TB4Y	thymosin beta 4, Y-linked	0.88	0.8	0.86	0.56
GLP2R	GLP2R	glucagon-like peptide 2 receptor	0.88	0.7	0.86	0.52
ATP9A	ATP9A;ATPIIA;KIAA0611	ATPase, class II, type 9A	0.88	0.7	0.86	0.62
STRN4	STRN4;FLJ35594;ZIN;zinedin	striatin, calmodulin binding protein 4	0.88	0.78	0.86	0.59
LTA4H	LTA4H	leukotriene A4 hydrolase	0.88	0.84	0.86	0.58
PTPN14	PTPN14;MGC126803;PEZ;PTP36	protein tyrosine phosphatase, non-receptor type 14	0.87	0.68	0.86	0.52
FPR2	FPR2;ALXR;FMLP-R-II;FMLPX;FPR2A;FPRH1;FPRH2;FPRL1;HM63;LXA4R	formyl peptide receptor 2	0.86	0.72	0.86	0.65
CACNG5	CACNG5;MGC126656;MGC126682	calcium channel, voltage-dependent, gamma subunit 5	0.86	0.76	0.86	0.59
COLQ	COLQ;EAD;FLJ55041	collagen-like tail subunit (single strand of homotrimer) of asymmetric acetylcholinesterase	0.86	0.77	0.86	0.52
AQP11	AQP11;AQPX1	aquaporin 11	0.86	0.76	0.86	0.62
MAPK13	MAPK13;MGC99536;PRKM13;SAPK4;p38delta	mitogen-activated protein kinase 13	0.86	0.8	0.86	0.57
CHST2	CHST2;C6ST;GST-2;GST2;Gn6ST-1;glcNAc6ST-1	carbohydrate (N-acetylglucosamine-6-O) sulfotransferase 2	0.86	0.74	0.86	0.5
COMMD3	COMMD3;BUP;C10orf8;DKFZp686K0399;FLJ45471	COMM domain containing 3	0.86	0.8	0.86	0.57
NPC2	NPC2;EDDM1;HE1;MGC1333	Niemann-Pick disease, type C2	0.86	0.77	0.86	0.58
XXYLT1	XXYLT1;C3orf21;FLJ35155	xyloside xylosyltransferase 1	0.86	0.78	0.86	0.56
TMEM200A	TMEM200A;KIAA1913;TTMA;TTMC	transmembrane protein 200A	0.85	0.76	0.86	0.52
ZNF780A	ZNF780A;ZNF780	zinc finger protein 780A	0.85	0.72	0.86	0.54
RAB13	RAB13	RAB13, member RAS oncogene family	0.85	0.78	0.86	0.59
CLCC1	CLCC1;KIAA0761;MCLC	chloride channel CLIC-like 1	1.42	1.3	0.87	1.01
CSRP1	CSRP1;CRP;CRP1;CSRP;CYRP;D1S181E;DKFZp686M148	cysteine and glycine-rich protein 1	1.36	1.21	0.87	0.92
GRK1	GRK1;GPRK1;RHOK;RK	G protein-coupled receptor kinase 1	1.3	1.13	0.87	0.93
SEC11C	SEC11C;SEC11L3;SPC21;SPCS4C	SEC11 homolog C (S. cerevisiae)	1.26	1.08	0.87	0.81
DLGAP5	DLGAP5;DLG7;HURP;KIAA0008	discs, large (Drosophila) homolog-associated protein 5	1.24	1.08	0.87	0.84
ZNF385A	ZNF385A;DKFZp586G1122;HZF;RZF;ZFP385;ZNF385	zinc finger protein 385A	1.18	1.06	0.87	0.81
MAPK9	MAPK9;JNK-55;JNK2;JNK2A;JNK2ALPHA;JNK2B;JNK2BETA;PRKM9;SAPK;p54a;p54aSAPK	mitogen-activated protein kinase 9	1.18	1.02	0.87	0.85
MAP3K6	MAP3K6;ASK2;MAPKKK6;MEKK6;MGC125653;MGC20114	mitogen-activated protein kinase kinase kinase 6	1.17	1.1	0.87	0.78
RAB34	RAB34;RAB39;RAH	RAB34, member RAS oncogene family	1.17	1.04	0.87	0.8
MACROD2	MACROD2;C20orf133	MACRO domain containing 2	1.14	1.04	0.87	0.74
EPHB4	EPHB4;HTK;MYK1;TYRO11	EPH receptor B4	1.13	0.96	0.87	0.86
TMBIM6	TMBIM6;BAXI1;BI-1;TEGT	transmembrane BAX inhibitor motif containing 6	1.12	1	0.87	0.68
GCLC	GCLC;GCL;GCS;GLCL;GLCLC	glutamate-cysteine ligase, catalytic subunit	1.11	0.94	0.87	0.81
LEP	LEP;FLJ94114;OB;OBS	leptin	1.1	0.93	0.87	0.68
ABL1	ABL1;ABL;JTK7;bcr/abl;c-ABL;p150;v-abl	c-abl oncogene 1, non-receptor tyrosine kinase	1.1	0.96	0.87	0.79
RIPK4	RIPK4;ANKK2;ANKRD3;DIK;MGC129992;MGC129993;PKK;RIP4	receptor-interacting serine-threonine kinase 4	1.1	0.96	0.87	0.8
CNPPD1	CNPPD1;C2orf24;CGI-57	cyclin Pas1/PHO80 domain containing 1	1.1	0.91	0.87	0.7
ATP8A2	ATP8A2;ATP;ATPIB;DKFZp434B1913;IB;ML-1	ATPase, aminophospholipid transporter, class I, type 8A, member 2	1.09	0.97	0.87	0.76
PPP1CA	PPP1CA;MGC15877;MGC1674;PP-1A;PP1alpha;PPP1A	protein phosphatase 1, catalytic subunit, alpha isozyme	1.09	0.96	0.87	0.75
NUDT6	NUDT6;ASFGF2;FGF-AS;FGF2AS;GFG-1;GFG1	nudix (nucleoside diphosphate linked moiety X)-type motif 6	1.08	1.04	0.87	0.72
C9orf96	C9orf96;MGC43306;RP11-244N20.8;SgK071;Sk521	chromosome 9 open reading frame 96	1.08	0.92	0.87	0.83
ZNF600	ZNF600;DKFZp686F06123;KR-ZNF1	zinc finger protein 600	1.07	0.96	0.87	0.69
DEFB118	DEFB118;C20orf63;DEFB-18;ESC42;ESP13.6	defensin, beta 118	1.06	0.9	0.87	0.67
BRD9	BRD9;DKFZp434D0711;DKFZp686L0539;FLJ43530;LAVS3040;PRO9856	bromodomain containing 9	1.06	0.98	0.87	0.69
MYOG	MYOG;MYF4;MYOGENIN;bHLHc3	myogenin (myogenic factor 4)	1.06	0.94	0.87	0.63
GNAI2	GNAI2;GIP;GNAI2B;H_LUCA15.1;H_LUCA16.1	guanine nucleotide binding protein (G protein), alpha inhibiting activity polypeptide 2	1.06	0.88	0.87	0.66
LSP1	LSP1;WP34;pp52	lymphocyte-specific protein 1	1.05	0.96	0.87	0.67
LSM10	LSM10;MGC15749;MST074;MSTP074	LSM10, U7 small nuclear RNA associated	1.05	0.97	0.87	0.68
FRMPD4	FRMPD4;KIAA0316;MGC142260;MGC142262;PDZD10;PDZK10	FERM and PDZ domain containing 4	1.04	0.9	0.87	0.65
KRT6B	KRT6B;CK6B;K6B;KRTL1;PC2	keratin 6B	1.04	0.94	0.87	0.71
IRF7	IRF7;IRF-7H;IRF7A	interferon regulatory factor 7	1.04	0.98	0.87	0.73
C19orf40	C19orf40;FAAP24;FLJ46828;MGC32020	chromosome 19 open reading frame 40	1.04	0.97	0.87	0.74
ZFAND6	ZFAND6;AWP1;ZA20D3;ZFAND5B	zinc finger, AN1-type domain 6	1.04	0.87	0.87	0.63
FTO	FTO;KIAA1752;MGC5149	fat mass and obesity associated	1.03	0.92	0.87	0.64
PPP2R1B	PPP2R1B;MGC26454;PP2A-Abeta;PR65B	protein phosphatase 2, regulatory subunit A, beta	1.03	0.84	0.87	0.63
C6orf124	C6orf124;HGC6.4;dJ431P23.3	chromosome 6 open reading frame 124	1.02	0.86	0.87	0.66
C21orf91	C21orf91;C21orf14;C21orf38;DKFZp781D1223;EURL;YG81	chromosome 21 open reading frame 91	1.02	0.86	0.87	0.69
VASP	VASP	vasodilator-stimulated phosphoprotein	1.02	0.96	0.87	0.71
BAZ1B	BAZ1B;WBSCR10;WBSCR9;WSTF	bromodomain adjacent to zinc finger domain, 1B	1.02	0.86	0.87	0.78
TRIM50	TRIM50;FLJ32804;MGC138357;MGC138359;TRIM50A	tripartite motif containing 50	1.01	0.86	0.87	0.77
C20orf54	C20orf54;BVVLS;MGC10698;RFT2;bA371L19.1;hRFT2	chromosome 20 open reading frame 54	1.01	0.85	0.87	0.65
ZMYND10	ZMYND10;BLU;FLU	zinc finger, MYND-type containing 10	1	0.92	0.87	0.61
AP4M1	AP4M1;CPSQ3;MU-4;MU-ARP2	adaptor-related protein complex 4, mu 1 subunit	1	0.82	0.87	0.69
CPNE5	CPNE5;COPN5;CPN5;DKFZp666C234;KIAA1599	copine V	0.99	0.88	0.87	0.7
NIPSNAP3A	NIPSNAP3A;DKFZp564D177;FLJ13953;MGC14553;NIPSNAP4;TASSC	nipsnap homolog 3A (C. elegans)	0.99	0.91	0.87	0.61
BBS2	BBS2;BBS;MGC20703	Bardet-Biedl syndrome 2	0.99	0.84	0.87	0.66
LBR	LBR;DHCR14B;FLJ43126;LMN2R;MGC9041;PHA	lamin B receptor	0.99	0.8	0.87	0.64
CDV3	CDV3;H41	CDV3 homolog (mouse)	0.99	0.94	0.87	0.62
FAM153A	FAM153A;FLJ75901;KIAA0752;NY-REN-7	family with sequence similarity 153, member A	0.99	0.83	0.87	0.69
NRSN1	NRSN1;VMP;p24	neurensin 1	0.98	0.88	0.87	0.65
CAPN11	CAPN11;calpain11	calpain 11	0.98	0.84	0.87	0.63
OR1M1	OR1M1;OR19-5;OR19-6	olfactory receptor, family 1, subfamily M, member 1	0.98	0.83	0.87	0.65
MDGA1	MDGA1;DKFZp686K0262;DKFZp686L0262;FLJ45018;GPIM;MAMDC3	MAM domain containing glycosylphosphatidylinositol anchor 1	0.98	0.83	0.87	0.65
WNT3	WNT3;INT4;MGC131950;MGC138321;MGC138323	wingless-type MMTV integration site family, member 3	0.98	0.88	0.87	0.6
LY6K	LY6K;CT97;FLJ35226;HSJ001348;ly-6K	lymphocyte antigen 6 complex, locus K	0.98	0.87	0.87	0.63
DLX3	DLX3;AI4;TDO	distal-less homeobox 3	0.98	0.93	0.87	0.7
KLF4	KLF4;EZF;GKLF	Kruppel-like factor 4 (gut)	0.98	0.86	0.87	0.66
RPS6KL1	RPS6KL1;FLJ35734;MGC11287	ribosomal protein S6 kinase-like 1	0.98	0.92	0.87	0.65
ACTL7A	ACTL7A	actin-like 7A	0.97	0.87	0.87	0.58
DDIT4L	DDIT4L;REDD2;Rtp801L	DNA-damage-inducible transcript 4-like	0.97	0.86	0.87	0.66
ANKRD12	ANKRD12;ANCO-2;ANCO1;FLJ20053;GAC-1;KIAA0874;NbIa00144	ankyrin repeat domain 12	0.97	0.8	0.87	0.59
HABP4	HABP4;IHABP4;Ki-1/57;MGC825;SERBP1L	hyaluronan binding protein 4	0.97	0.82	0.87	0.66
ATRX	ATRX;ATR2;JMS;MGC2094;MRXHF1;RAD54;RAD54L;SFM1;SHS;XH2;XNP;ZNF-HX	alpha thalassemia/mental retardation syndrome X-linked	0.97	0.82	0.87	0.6
CLDN16	CLDN16;HOMG3;PCLN1	claudin 16	0.97	0.93	0.87	0.64
RWDD2B	RWDD2B;C21orf6	RWD domain containing 2B	0.97	0.81	0.87	0.62
MED13	MED13;ARC250;DRIP250;HSPC221;KIAA0593;THRAP1;TRAP240	mediator complex subunit 13	0.96	0.82	0.87	0.58
BANK1	BANK1;BANK;FLJ20706;FLJ34204	B-cell scaffold protein with ankyrin repeats 1	0.95	0.8	0.87	0.61
MRPL33	MRPL33;C2orf1;L33mt;MGC111093;MGC13694;MGC23922;MRP-L33;RPL33L	mitochondrial ribosomal protein L33	0.95	0.86	0.87	0.65
GLDC	GLDC;GCE;GCSP;HYGN1;MGC138198;MGC138200	glycine dehydrogenase (decarboxylating)	0.95	0.79	0.87	0.57
CHTF8	CHTF8;CTF8;DERPC;FLJ20400	CTF8, chromosome transmission fidelity factor 8 homolog (S. cerevisiae)	0.95	0.82	0.87	0.6
CSH2	CSH2;CS-2;CSB;hCS-B	chorionic somatomammotropin hormone 2	0.94	0.8	0.87	0.63
COG3	COG3;SEC34	component of oligomeric golgi complex 3	0.94	0.8	0.87	0.64
SEPHS2	SEPHS2;SPS2	selenophosphate synthetase 2	0.94	0.79	0.87	0.62
APOL4	APOL4;APOL-IV;APOLIV	apolipoprotein L, 4	0.93	0.8	0.87	0.6
GSX2	GSX2;GSH2	GS homeobox 2	0.92	0.86	0.87	0.66
FBXO42	FBXO42;Fbx42;JFK;KIAA1332	F-box protein 42	0.92	0.76	0.87	0.55
NOV	NOV;CCN3;IGFBP9	nephroblastoma overexpressed gene	0.92	0.86	0.87	0.65
ARHGEF2	ARHGEF2;DKFZp547L106;DKFZp547P1516;FLJ45440;GEF;GEF-H1;GEFH1;KIAA0651;LFP40;P40	Rho/Rac guanine nucleotide exchange factor (GEF) 2	0.92	0.86	0.87	0.64
BRI3BP	BRI3BP;BNAS1;HCCR-1;HCCR-2;HCCRBP-1;KG19	BRI3 binding protein	0.92	0.82	0.87	0.55
GSTK1	GSTK1;GST;GST 13-13;GST13;GST13-13;GSTK1-1;hGSTK1	glutathione S-transferase kappa 1	0.92	0.86	0.87	0.57
RAB7B	RAB7B;MGC16212;MGC9726;RAB7	RAB7B, member RAS oncogene family	0.92	0.78	0.87	0.65
ZNF222	ZNF222	zinc finger protein 222	0.91	0.82	0.87	0.56
C10orf82	C10orf82;FLJ40268;MGC33547	chromosome 10 open reading frame 82	0.91	0.81	0.87	0.58
C19orf60	C19orf60;FLJ20850;FLJ30108;FLJ34606;FLJ37391	chromosome 19 open reading frame 60	0.91	0.8	0.87	0.6
CDC37P1	CDC37P1;A-761H5.2	cell division cycle 37 homolog (S. cerevisiae) pseudogene 1	0.91	0.77	0.87	0.62
ICA1L	ICA1L;ALS2CR14;ALS2CR15;DKFZp434E1919;MGC138440	islet cell autoantigen 1,69kDa-like	0.9	0.84	0.87	0.65
METTL11A	METTL11A;AD-003;C9orf32;NRMT;NTM1A	methyltransferase like 11A	0.9	0.74	0.87	0.6
PPP2R5C	PPP2R5C;B56G;MGC23064;PR61G	protein phosphatase 2, regulatory subunit B', gamma	0.9	0.78	0.87	0.58
GPC4	GPC4;K-glypican	glypican 4	0.89	0.78	0.87	0.53
SPTA1	SPTA1;EL2;HPP;HS3;SPH3;SPTA	spectrin, alpha, erythrocytic 1 (elliptocytosis 2)	0.88	0.8	0.87	0.65
IL17RB	IL17RB;CRL4;EVI27;IL17BR;IL17RH1;MGC5245	interleukin 17 receptor B	0.88	0.84	0.87	0.63
ETV5	ETV5;ERM	ets variant 5	0.88	0.75	0.87	0.53
XKR7	XKR7;C20orf159;dJ310O13.4	XK, Kell blood group complex subunit-related family, member 7	0.88	0.79	0.87	0.59
CAMKK1	CAMKK1;CAMKKA;DKFZp761M0423;MGC34095	calcium/calmodulin-dependent protein kinase kinase 1, alpha	0.88	0.76	0.87	0.63
PAPOLG	PAPOLG;FLJ11805;FLJ12972;FLJ13482;FLJ14187;MGC133307;MGC133308	poly(A) polymerase gamma	0.88	0.82	0.87	0.62
ZNF83	ZNF83;FLJ11015;FLJ14876;FLJ30097;FLJ90585;HPF1;MGC33853;ZNF816B	zinc finger protein 83	0.88	0.74	0.87	0.53

KCTD5	KCTD5;FLJ20040	potassium channel tetramerisation domain containing 5	0.88	0.8	0.87	0.61
EXO1	EXO1;HEX1;hExoI	exonuclease 1	0.88	0.72	0.87	0.56
MRGPRX1	MRGPRX1;GPCR;MRGX1;SNSR4	MAS-related GPR, member X1	0.87	0.74	0.87	0.66
GALNT5	GALNT5;GALNAC-T5;MGC165041	UDP-N-acetyl-alpha-D-galactosamine:polypeptide N-acetylgalactosaminyltransferase 5 (GalNAc-T5)	0.86	0.7	0.87	0.55
FOXD4L1	FOXD4L1;FOXD5;bA395L14.1	forkhead box D4-like 1	0.86	0.72	0.87	0.59
FOXJ2	FOXJ2;FHX	forkhead box J2	0.86	0.7	0.87	0.56
ZFYVE21	ZFYVE21;MGC2550;ZF21	zinc finger, FYVE domain containing 21	0.86	0.67	0.87	0.56
ERCC2	ERCC2;COFS2;EM9;MGC102762;MGC126218;MGC126219;TTD;XPD	excision repair cross-complementing rodent repair deficiency, complementation group 2	0.86	0.72	0.87	0.54
STX12	STX12;MGC51957;STX13;STX14	syntaxin 12	0.86	0.82	0.87	0.6
HSPA8	HSPA8;HSC54;HSC70;HSC71;HSP71;HSP73;HSPA10;LAP1;MGC131511;MGC29929;NIP71	heat shock 70kDa protein 8	0.86	0.7	0.87	0.56
AKAP2	AKAP2;AKAP-2;AKAPKL;DKFZp564L0716;FLJ53197;FLJ98336;PRKA2	A kinase (PRKA) anchor protein 2	0.86	0.72	0.87	0.55
FAM75A1	FAM75A1;C9orf36;C9orf36A	family with sequence similarity 75, member A1	0.86	0.74	0.87	0.54
RALGAPB	RALGAPB;DKFZp686N19168;DKFZp781M2411;FLJ43270;FLJ43928;KIAA1219;RalGAPbeta;dJ1100H13.1	Ral GTPase activating protein, beta subunit (non-catalytic)	0.86	0.78	0.87	0.52
TOX4	TOX4;C14orf92;KIAA0737;LCP1;MIG7	TOX high mobility group box family member 4	0.85	0.8	0.87	0.62
LOC646627	LOC646627	phospholipase inhibitor	0.85	0.71	0.87	0.51
ACVR2A	ACVR2A;ACTRII;ACVR2	activin A receptor, type IIA	1.33	1.16	0.88	0.96
ZNF18	ZNF18;HDSG1;KOX11;ZKSCAN6;ZNF535;Zfp535	zinc finger protein 18	1.26	1.08	0.88	0.9
COPS2	COPS2;ALIEN;CSN2;SGN2;TRIP15	COP9 constitutive photomorphogenic homolog subunit 2 (Arabidopsis)	1.25	1.09	0.88	0.81
VARS2	VARS2;MGC138259;MGC142165;VARS2L;VARSL	valyl-tRNA synthetase 2, mitochondrial (putative)	1.21	1.11	0.88	0.79
MAGI2	MAGI2;ACVRIP1;AIP-1;AIP1;ARIP1;MAGI-2;SSCAM	membrane associated guanylate kinase, WW and PDZ domain containing 2	1.19	1.04	0.88	0.86
IRF2BP1	IRF2BP1;DKFZp434M154	interferon regulatory factor 2 binding protein 1	1.19	1	0.88	0.72
GSTM3	GSTM3;GST5;GSTB;GSTM3-3;GTM3;MGC3310;MGC3704	glutathione S-transferase mu 3 (brain)	1.16	1.04	0.88	0.82
DCX	DCX;DBCN;DC;FLJ51296;LISX;SCLH;XLIS	doublecortin	1.15	1.04	0.88	0.78
ATF7IP2	ATF7IP2;FLJ12668;MCAF2	activating transcription factor 7 interacting protein 2	1.15	1	0.88	0.75
NR1I3	NR1I3;CAR;CAR1;MB67;MGC150433;MGC97144;MGC97209	nuclear receptor subfamily 1, group I, member 3	1.14	1.09	0.88	0.76
GABRQ	GABRQ;MGC129629;MGC129630;THETA	gamma-aminobutyric acid (GABA) receptor, theta	1.14	1.04	0.88	0.81
MOXD1	MOXD1;DKFZp564G202;MOX;PRO5780;dJ248E1.1	monooxygenase, DBH-like 1	1.14	0.96	0.88	0.69
BCL11B	BCL11B;CTIP-2;CTIP2;RIT1;ZNF856B;hRIT1-alpha	B-cell CLL/lymphoma 11B (zinc finger protein)	1.14	1.04	0.88	0.71
HCST	HCST;DAP10;DKFZP586C1522;KAP10;PIK3AP	hematopoietic cell signal transducer	1.14	0.97	0.88	0.76
PRKAA1	PRKAA1;AMPK;AMPKa1;MGC33776;MGC57364	protein kinase, AMP-activated, alpha 1 catalytic subunit	1.13	0.98	0.88	0.82
GRM7	GRM7;FLJ40498;GLUR7;GPRC1G;MGLU7;MGLUR7	glutamate receptor, metabotropic 7	1.12	0.98	0.88	0.76
CARD8	CARD8;CARDINAL;DACAR;DAKAR;DKFZp779L0366;FLJ18119;FLJ18121;KIAA0955;MGC57162;NDPP;NDPP1;TUCAN	caspase recruitment domain family, member 8	1.12	1	0.88	0.68
CCDC27	CCDC27;FLJ32825;MGC138313;MGC138317	coiled-coil domain containing 27	1.11	1.07	0.88	0.71
RIN3	RIN3;DKFZp762H1613;FLJ11700;FLJ22439	Ras and Rab interactor 3	1.11	1	0.88	0.76
ESAM	ESAM;W117m	endothelial cell adhesion molecule	1.1	0.96	0.88	0.79
ZNF263	ZNF263;FPM315;ZKSCAN12	zinc finger protein 263	1.1	0.99	0.88	0.71
IGSF22	IGSF22;FLJ37794	immunoglobulin superfamily, member 22	1.09	0.91	0.88	0.74
PDF	PDF	peptide deformylase (mitochondrial)	1.09	1.05	0.88	0.74
RNF32	RNF32;FKSG33;HSD15	ring finger protein 32	1.08	0.91	0.88	0.66
RNF213	RNF213;ALO17;C17orf27;DKFZp762N1115;FLJ13051;KIAA1554;KIAA1618;MGC46622;MGC9929;MYMY2;NET57;mysterin;mystr	ring finger protein 213	1.08	0.94	0.88	0.85
ZMYM4	ZMYM4;CDIR;DKFZp686B09210;DKFZp686L0547;KIAA0425;MYM;ZNF198L3;ZNF262	zinc finger, MYM-type 4	1.08	0.94	0.88	0.68
CXorf67	CXorf67;MGC47837	chromosome X open reading frame 67	1.07	0.97	0.88	0.73
TRIM59	TRIM59;MGC129860;MGC129861;MGC26631;MRF1;RNF104;TRIM57;TSBF1	tripartite motif containing 59	1.06	0.92	0.88	0.82
GADD45B	GADD45B;DKFZp566B133;GADD45BETA;MYD118	growth arrest and DNA-damage-inducible, beta	1.06	0.97	0.88	0.73
KIAA1310	KIAA1310;FLJ10081;FLJ23799;NSL3;Rcd1	KIAA1310	1.06	0.97	0.88	0.73
VAPB	VAPB;ALS8;VAMP-B;VAP-B	VAMP (vesicle-associated membrane protein)-associated protein B and C	1.06	0.91	0.88	0.69
SLC27A6	SLC27A6;ACSVL2;DKFZp779M0564;FACVL2;FATP6;VLCS-H1	solute carrier family 27 (fatty acid transporter), member 6	1.05	0.92	0.88	0.68
CACNA1B	CACNA1B;BIII;CACNL1A5;CACNN;Cav2.2	calcium channel, voltage-dependent, N type, alpha 1B subunit	1.05	0.92	0.88	0.71
NDOR1	NDOR1;MGC138148;NR1;bA350O14.9	NADPH dependent diflavin oxidoreductase 1	1.05	0.9	0.88	0.78
VANGL1	VANGL1;KITENIN;LPP2;MGC5338;STB2;STBM2	vang-like 1 (van gogh, Drosophila)	1.05	0.96	0.88	0.73
GLT6D1	GLT6D1;GLTDC1;GT6M7	glycosyltransferase 6 domain containing 1	1.04	0.92	0.88	0.62
MOS	MOS;MGC119962;MGC119963;MSV	v-mos Moloney murine sarcoma viral oncogene homolog	1.04	0.99	0.88	0.7
PTGER2	PTGER2;EP2	prostaglandin E receptor 2 (subtype EP2), 53kDa	1.04	0.9	0.88	0.8
H3F3C	H3F3C;H3.5	H3 histone, family 3C	1.04	0.86	0.88	0.73
CCL28	CCL28;CCK1;MEC;MGC71902;SCYA28	chemokine (C-C motif) ligand 28	1.03	0.88	0.88	0.69
PAGE1	PAGE1;AL5;CT16.3;GAGE-9;GAGEB1;PAGE-1	P antigen family, member 1 (prostate associated)	1.02	0.93	0.88	0.69
OR1F1	OR1F1;OLFMF;OR16-36;OR16-37;OR16-88;OR16-89;OR16-90;OR1F10;OR1F13P;OR1F4;OR1F5;OR1F6;OR1F7;OR1F8;OR1F9;OR3-145;ORL1023	olfactory receptor, family 1, subfamily F, member 1	1.02	0.87	0.88	0.66
DUSP22	DUSP22;FLJ35864;JKAP;JSP1;LMWDSP2;MKPX;VHX	dual specificity phosphatase 22	1.02	0.83	0.88	0.63
TAF8	TAF8;43;FLJ32821;II;TAF;TAFII-43;TAFII43;TBN	TAF8 RNA polymerase II, TATA box binding protein (TBP)-associated factor, 43kDa	1.02	0.88	0.88	0.71
MTMR3	MTMR3;FLJ32333;FYVE-DSP1;KIAA0371;ZFYVE10	myotubularin related protein 3	1.02	0.89	0.88	0.7
SLC9A6	SLC9A6;KIAA0267;MRSA;NHE6	solute carrier family 9 (sodium/hydrogen exchanger), member 6	1.02	0.88	0.88	0.69
SIGMAR1	SIGMAR1;FLJ25585;MGC3851;OPRS1;SR-BP1;SRBP	sigma non-opioid intracellular receptor 1	1.02	0.86	0.88	0.77
CYP17A1	CYP17A1;CPT7;CYP17;P450C17;S17AH	cytochrome P450, family 17, subfamily A, polypeptide 1	1.01	0.84	0.88	0.61
DSG2	DSG2;ARVC10;ARVD10;CDHF5;CMD1BB;HDGC;MGC117034;MGC117036;MGC117037	desmoglein 2	1.01	0.92	0.88	0.69
VLDLR	VLDLR;CARMQ1;CHRMQ1;FLJ35024;VLDLRCH	very low density lipoprotein receptor	1.01	0.94	0.88	0.63
DOC2A	DOC2A;Doc2	double C2-like domains, alpha	1	0.84	0.88	0.61
SGTA	SGTA;SGT;alphaSGT;hSGT	small glutamine-rich tetratricopeptide repeat (TPR)-containing, alpha	1	0.82	0.88	0.7
WDR26	WDR26;CDW2;FLJ21016;MIP2	WD repeat domain 26	1	0.86	0.88	0.69
SLC16A7	SLC16A7;MCT2	solute carrier family 16, member 7 (monocarboxylic acid transporter 2)	1	0.85	0.88	0.61
CETP	CETP;BPIFF;HDLCQ10	cholesteryl ester transfer protein, plasma	0.99	0.84	0.88	0.61
MAP1LC3B	MAP1LC3B;ATG8F;LC3B;MAP1A/1BLC3	microtubule-associated protein 1 light chain 3 beta	0.99	0.86	0.88	0.59
TGM5	TGM5;MGC141907;TGASE5;TGASEX;TGM6;TGMX;TGX	transglutaminase 5	0.99	0.9	0.88	0.7
SCN4B	SCN4B	sodium channel, voltage-gated, type IV, beta	0.99	0.92	0.88	0.71
ZC3H13	ZC3H13;DKFZp434D1812;FLJ35669;KIAA0853	zinc finger CCCH-type containing 13	0.99	0.85	0.88	0.63
GPRC5D	GPRC5D;MGC129713;MGC129714	G protein-coupled receptor, family C, group 5, member D	0.98	0.8	0.88	0.6
TLX1	TLX1;HOX11;MGC163402;TCL3	T-cell leukemia homeobox 1	0.98	0.85	0.88	0.66
CLDN4	CLDN4;CPE-R;CPER;CPETR;CPETR1;WBSCR8;hCPE-R	claudin 4	0.98	0.92	0.88	0.61
TRAF3IP1	TRAF3IP1;DKFZp434F124;MIP-T3;MIPT3	TNF receptor-associated factor 3 interacting protein 1	0.98	0.9	0.88	0.6
JOSD1	JOSD1;KIAA0063;dJ508I15.2	Josephin domain containing 1	0.98	0.82	0.88	0.63
METTL18	METTL18;AsTP2;C1orf156;HPM1;MGC9084	methyltransferase like 18	0.98	0.83	0.88	0.63
CYP27A1	CYP27A1;CP27;CTX;CYP27	cytochrome P450, family 27, subfamily A, polypeptide 1	0.97	0.8	0.88	0.63
CARS2	CARS2;DKFZp667A2315;DKFZp686G08243;FLJ12118;FLJ42514	cysteinyl-tRNA synthetase 2, mitochondrial (putative)	0.97	0.9	0.88	0.63
SHC3	SHC3;DKFZp686H1544;FLJ45325;N-Shc;NSHC;RAI;SHCC	SHC (Src homology 2 domain containing) transforming protein 3	0.96	0.93	0.88	0.68
LRRC23	LRRC23;LRPB7	leucine rich repeat containing 23	0.96	0.86	0.88	0.64
TOMM70A	TOMM70A;FLJ90470	translocase of outer mitochondrial membrane 70 homolog A (S. cerevisiae)	0.96	0.89	0.88	0.63
BOLA3	BOLA3;MMDS2	bolA homolog 3 (E. coli)	0.96	0.87	0.88	0.62
KRTAP8-1	KRTAP8-1;KAP8.1	keratin associated protein 8-1	0.95	0.86	0.88	0.65
C2orf67	C2orf67;FLJ23861;MSL1v2	chromosome 2 open reading frame 67	0.95	0.87	0.88	0.59
MAGEB3	MAGEB3;CT3.5	melanoma antigen family B, 3	0.94	0.86	0.88	0.65
PPY	PPY;PNP	pancreatic polypeptide	0.94	0.9	0.88	0.66
APOLD1	APOLD1;DKFZp434F0318;FLJ25138;FLJ95166;VERGE	apolipoprotein L domain containing 1	0.94	0.82	0.88	0.61
ENPP7	ENPP7;ALK-SMase;MGC50179	ectonucleotide pyrophosphatase/phosphodiesterase 7	0.94	0.82	0.88	0.64
ENPP4	ENPP4;KIAA0879;NPP4	ectonucleotide pyrophosphatase/phosphodiesterase 4 (putative)	0.94	0.89	0.88	0.58
CHP2	CHP2	calcineurin B homologous protein 2	0.93	0.86	0.88	0.69
DACT1	DACT1;DAPPER;DAPPER1;DPR1;FRODO;HDPR1;THYEX3	dapper, antagonist of beta-catenin, homolog 1 (Xenopus laevis)	0.93	0.84	0.88	0.64
EZH2	EZH2;ENX-1;ENX1;EZH1;KMT6;KMT6A;MGC9169	enhancer of zeste homolog 2 (Drosophila)	0.93	0.87	0.88	0.57
ZNF682	ZNF682;BC39498_3;FLJ90362	zinc finger protein 682	0.92	0.82	0.88	0.59
ZNF169	ZNF169;MGC51961	zinc finger protein 169	0.92	0.73	0.88	0.6
OSGEPL1	OSGEPL1;Qri7	O-sialoglycoprotein endopeptidase-like 1	0.92	0.8	0.88	0.6
ACAD11	ACAD11;ACAD-11;FLJ12592;MGC150619	acyl-CoA dehydrogenase family, member 11	0.92	0.88	0.88	0.61
C11orf35	C11orf35;MGC35138	chromosome 11 open reading frame 35	0.92	0.78	0.88	0.6
HBXIP	HBXIP;MGC71071;XIP	hepatitis B virus x interacting protein	0.92	0.78	0.88	0.61
PRSS23	PRSS23;MGC5107;SIG13;SPUVE;ZSIG13	protease, serine, 23	0.92	0.8	0.88	0.6
PCBP1	PCBP1;HNRPE1;HNRPX;hnRNP-E1;hnRNP-X	poly(rC) binding protein 1	0.92	0.81	0.88	0.63
XPO6	XPO6;EXP6;FLJ22519;KIAA0370;RANBP20	exportin 6	0.91	0.84	0.88	0.68
GPR15	GPR15;MGC126828;MGC126830	G protein-coupled receptor 15	0.9	0.77	0.88	0.69
LHX4	LHX4;CPHD4;FLJ22769	LIM homeobox 4	0.9	0.78	0.88	0.58
TMTC2	TMTC2;DKFZp762A217;IBDBP1	transmembrane and tetratricopeptide repeat containing 2	0.9	0.84	0.88	0.6
PRKACB	PRKACB;DKFZp781I2452;MGC41879;MGC9320;PKACB	protein kinase, cAMP-dependent, catalytic, beta	0.9	0.78	0.88	0.65
RAP1GDS1	RAP1GDS1;GDS1;MGC118859;MGC118861;SmgGDS	RAP1, GTP-GDP dissociation stimulator 1	0.9	0.86	0.88	0.64
IPO7	IPO7;FLJ14581;Imp7;MGC138673;RANBP7	importin 7	0.9	0.88	0.88	0.61
ARHGDIG	ARHGDIG;RHOGDI-3	Rho GDP dissociation inhibitor (GDI) gamma	0.89	0.8	0.88	0.61
TRAF3	TRAF3;CAP-1;CD40bp;CRAF1;LAP1	TNF receptor-associated factor 3	0.89	0.78	0.88	0.69
CDK2AP2	CDK2AP2;DOC-1R;FLJ10636;p14	cyclin-dependent kinase 2 associated protein 2	0.89	0.86	0.88	0.57
DPM3	DPM3;CDG1O;MGC125904;MGC125905;MGC34275	dolichyl-phosphate mannosyltransferase polypeptide 3	0.89	0.79	0.88	0.54
LANCL3	LANCL3;FLJ42925	LanC lantibiotic synthetase component C-like 3 (bacterial)	0.88	0.81	0.88	0.58
TP53TG3	TP53TG3;P53TG3;TP53TG3A;TP53TG3B;TP53TG3C;TP53TG3D	TP53 target 3	0.88	0.75	0.88	0.57
ZNF225	ZNF225;MGC119735	zinc finger protein 225	0.88	0.75	0.88	0.61
CCDC13	CCDC13;FLJ25467	coiled-coil domain containing 13	0.88	0.73	0.88	0.59
LOC91431	LOC91431	ENTREZ STATUS: REMAPPED TO GENE ID: 55345	0.88	0.74	0.88	0.53
KLF14	KLF14;BTEB5	Kruppel-like factor 14	0.88	0.74	0.88	0.54
GALC	GALC	galactosylceramidase	0.88	0.82	0.88	0.55
NCDN	NCDN;KIAA0607	neurochondrin	0.88	0.75	0.88	0.6
TMEM170A	TMEM170A;FLJ37611;TMEM170	transmembrane protein 170A	0.88	0.84	0.88	0.56
SLC25A23	SLC25A23;APC2;MCSC2;MGC2615;SCaMC-3	solute carrier family 25 (mitochondrial carrier; phosphate carrier), member 23	0.88	0.8	0.88	0.63
DET1	DET1;FLJ10103;MGC126156;MGC126157	de-etiolated homolog 1 (Arabidopsis)	0.87	0.78	0.88	0.56
LRRC37A3	LRRC37A3;FLJ34306;KIAA0563;LRRC37A;MGC41826;MGC57168	leucine rich repeat containing 37, member A3	0.86	0.68	0.88	0.53
FLJ22184	FLJ22184	putative uncharacterized protein FLJ22184	0.86	0.78	0.88	0.59
ADIPOR2	ADIPOR2;ACDCR2;FLJ21432;MGC4640;PAQR2	adiponectin receptor 2	0.86	0.79	0.88	0.6

ABL2	ABL2;ABLL;ARG;FLJ22224;FLJ31718;FLJ41441	v-abl Abelson murine leukemia viral oncogene homolog 2	0.86	0.76	0.88	0.63
TSR2	TSR2;DT1P1A10;MGC20451;WGG1	TSR2, 20S rRNA accumulation, homolog (S. cerevisiae)	0.86	0.84	0.88	0.56
TBC1D22A	TBC1D22A;C22orf4;HSC79E021	TBC1 domain family, member 22A	0.86	0.78	0.88	0.56
THRAP3	THRAP3;FLJ22082;MGC133082;MGC133083;TRAP150	thyroid hormone receptor associated protein 3	0.86	0.76	0.88	0.51
SLC39A6	SLC39A6;LIV-1	solute carrier family 39 (zinc transporter), member 6	0.85	0.68	0.88	0.54
YEATS4	YEATS4;4930573H17Rik;B230215M10Rik;GAS41;NUBI-1;YAF9	YEATS domain containing 4	0.85	0.79	0.88	0.52
GRM4	GRM4;GPRC1D;MGC177594;MGLUR4;mGlu4	glutamate receptor, metabotropic 4	1.38	1.19	0.89	1.08
GPRIN2	GPRIN2;GRIN2;KIAA0514;MGC15171	G protein regulated inducer of neurite outgrowth 2	1.31	1.27	0.89	0.85
PCK2	PCK2;PEPCK;PEPCK-M;PEPCK2	phosphoenolpyruvate carboxykinase 2 (mitochondrial)	1.25	1.07	0.89	0.97
C2orf42	C2orf42;FLJ20558	chromosome 2 open reading frame 42	1.25	1.19	0.89	0.83
TCHP	TCHP;MGC10854;TpMs	trichoplein, keratin filament binding	1.25	1.15	0.89	0.83
TRIM3	TRIM3;BERP;FLJ16135;HAC1;RNF22;RNF97	tripartite motif containing 3	1.22	1.04	0.89	0.75
MAP3K2	MAP3K2;MEKK2;MEKK2B	mitogen-activated protein kinase kinase kinase 2	1.21	1.06	0.89	0.88
BTN1A1	BTN1A1;BT;BTN	butyrophilin, subfamily 1, member A1	1.2	1.04	0.89	0.73
THEM4	THEM4;CTMP;FLJ27206;MGC29636	thioesterase superfamily member 4	1.2	1.14	0.89	0.79
IFT88	IFT88;D13S1056E;DAF19;MGC26259;TG737;TTC10;hTg737	intraflagellar transport 88 homolog (Chlamydomonas)	1.19	1.15	0.89	0.88
RASSF9	RASSF9;P-CIP1;PAMCI	Ras association (RalGDS/AF-6) domain family (N-terminal) member 9	1.17	1	0.89	0.72
KPTN	KPTN;2E4	kaptin (actin binding protein)	1.17	1.06	0.89	0.84
CHST7	CHST7;C6ST-2	carbohydrate (N-acetylglucosamine 6-O) sulfotransferase 7	1.17	1.02	0.89	0.79
SPSB2	SPSB2;FLJ17395;GRCC9;MGC2519;SSB2	splA/ryanodine receptor domain and SOCS box containing 2	1.17	1	0.89	0.71
EHHADH	EHHADH;ECHD;L-PBE;LBFP;LBP;MGC120586;PBFE	enoyl-CoA, hydratase/3-hydroxyacyl CoA dehydrogenase	1.17	1.02	0.89	0.76
INVS	INVS;INV;KIAA0573;MGC133080;MGC133081;NPH2;NPHP2	inversin	1.15	1.06	0.89	0.8
SMO	SMO;FZD11;Gx;SMOH	smoothened, frizzled family receptor	1.15	0.96	0.89	0.72
CITED4	CITED4	Cbp/p300-interacting transactivator, with Glu/Asp-rich carboxy-terminal domain, 4	1.14	1.12	0.89	0.78
ZNF614	ZNF614;FLJ21941;MGC120638	zinc finger protein 614	1.11	0.98	0.89	0.81
C3orf39	C3orf39;AGO61;FLJ14566	chromosome 3 open reading frame 39	1.11	0.96	0.89	0.74
DNAH2	DNAH2;DNAHC2;DNHD3;FLJ46675;KIAA1503	dynein, axonemal, heavy chain 2	1.1	0.98	0.89	0.71
CD40	CD40;Bp50;CDW40;MGC9013;TNFRSF5;p50	CD40 molecule, TNF receptor superfamily member 5	1.1	1	0.89	0.79
OTUD5	OTUD5;DKFZp761A052;DUBA;MGC104871	OTU domain containing 5	1.1	1.02	0.89	0.7
CHRM2	CHRM2;FLJ43243;HM2;MGC120006;MGC120007	cholinergic receptor, muscarinic 2	1.09	0.94	0.89	0.85
NBEA	NBEA;BCL8B;LYST2	neurobeachin	1.09	1.01	0.89	0.76
KRT4	KRT4;CK-4;CK4;CYK4;FLJ31692;K4	keratin 4	1.09	1	0.89	0.76
CERCAM	CERCAM;CEECAM1;GLT25D3;MGC149620;MGC149621	cerebral endothelial cell adhesion molecule	1.09	0.97	0.89	0.72
PTP4A3	PTP4A3;PRL-3;PRL-R;PRL3	protein tyrosine phosphatase type IVA, member 3	1.09	0.9	0.89	0.68
WNT16	WNT16	wingless-type MMTV integration site family, member 16	1.08	1	0.89	0.75
RNF216	RNF216;TRIAD3;U7I1;UBCE7IP1;ZIN	ring finger protein 216	1.08	0.95	0.89	0.86
TWISTNB	TWISTNB	TWIST neighbor	1.08	0.92	0.89	0.71
RFK	RFK;FLJ11149;RIFK	riboflavin kinase	1.08	0.95	0.89	0.79
EHMT2	EHMT2;BAT8;C6orf30;DKFZp686H08213;FLJ35547;G9A;GAT8;KMT1C;NG36	euchromatic histone-lysine N-methyltransferase 2	1.08	0.9	0.89	0.72
USP28	USP28;KIAA1515	ubiquitin specific peptidase 28	1.08	0.9	0.89	0.67
PPIAL4A	PPIAL4A;COAS2;MGC163248;MGC163250;PPIAL4;PPIAL4B;PPIAL4C;PPIAL4D;PPIAL4G	peptidylprolyl isomerase A (cyclophilin A)-like 4A	1.08	1.02	0.89	0.71
IL10	IL10;CSIF;IL-10;IL10A;MGC126450;MGC126451;TGIF	interleukin 10	1.06	1	0.89	0.66
RETNLB	RETNLB;FIZZ1;FIZZ2;HXCP2;RELM-beta;RELMb;RELMbeta;XCP2	resistin like beta	1.06	0.98	0.89	0.67
FAM108B1	FAM108B1;C9orf77;RP11-409O11.2	family with sequence similarity 108, member B1	1.06	0.96	0.89	0.76
OTUD7B	OTUD7B;CEZANNE;ZA20D1	OTU domain containing 7B	1.06	0.94	0.89	0.84
TMEM237	TMEM237;ALS2CR4;DKFZp313L091;FLJ33282	transmembrane protein 237	1.06	0.97	0.89	0.66
XAGE1D	XAGE1D;CT12.1;CT12.1D;CTP9;XAGE1A;XAGE1B;XAGE1C;XAGE1E	X antigen family, member 1D	1.06	1.02	0.89	0.68
SST	SST;SMST	somatostatin	1.05	1.02	0.89	0.75
SEMA4F	SEMA4F;M-SEMA;PRO2353;SEMAM;SEMAW;m-Sema-M	sema domain, immunoglobulin domain (Ig), transmembrane domain (TM) and short cytoplasmic domain, (semaphorin) 4F	1.05	1.02	0.89	0.75
UBE2B	UBE2B;E2-17kDa;HHR6B;HR6B;RAD6B;UBC2	ubiquitin-conjugating enzyme E2B	1.05	0.86	0.89	0.65
CEP104	CEP104;GlyBP;KIAA0562	centrosomal protein 104kDa	1.05	0.91	0.89	0.72
KIR3DS1	KIR3DS1;CD158E2;KIR-123FM;KIR-G1;MGC119726;MGC119728;MGC125316;NKAT10	killer cell immunoglobulin-like receptor, three domains, short cytoplasmic tail, 1	1.04	0.96	0.89	0.72
PPAT	PPAT;ATASE;GPAT;PRAT	phosphoribosyl pyrophosphate amidotransferase	1.04	0.86	0.89	0.77
LPCAT3	LPCAT3;C3F;LPCAT;LPLAT 5;LPSAT;MBOAT5;OACT5;nessy	lysophosphatidylcholine acyltransferase 3	1.03	0.9	0.89	0.67
TEKT4	TEKT4;MGC27019	tektin 4	1.02	0.94	0.89	0.7
HOXA3	HOXA3;HOX1;HOX1E;MGC10155	homeobox A3	1.02	0.95	0.89	0.64
PPIH	PPIH;CYP-20;CYPH;MGC5016;SnuCyp-20;USA-CYP	peptidylprolyl isomerase H (cyclophilin H)	1.02	0.88	0.89	0.65
CUTA	CUTA;ACHAP;C6orf82;MGC111154	cutA divalent cation tolerance homolog (E. coli)	1.02	0.96	0.89	0.69
ASPG	ASPG;C14orf76;GPA/WT	asparaginase homolog (S. cerevisiae)	1.02	0.86	0.89	0.62
TTC36	TTC36;HBP21	tetratricopeptide repeat domain 36	1.01	0.94	0.89	0.69
C19orf39	C19orf39;FLJ35119;MGC149562;MGC78620	chromosome 19 open reading frame 39	1.01	0.86	0.89	0.66
CDH18	CDH18;CDH14;CDH14L;CDH24	cadherin 18, type 2	1	0.93	0.89	0.7
DLX4	DLX4;BP1;DLX7;DLX8;DLX9	distal-less homeobox 4	1	0.92	0.89	0.72
TM2D3	TM2D3;BLP2	TM2 domain containing 3	1	0.98	0.89	0.65
C18orf32	C18orf32;FLJ23458	chromosome 18 open reading frame 32	1	0.88	0.89	0.73
MYCBPAP	MYCBPAP;AMAP-1;AMAP1;DKFZp434N1415	MYCBP associated protein	0.99	0.87	0.89	0.68
ZNF337	ZNF337	zinc finger protein 337	0.99	0.9	0.89	0.69
ZNF707	ZNF707	zinc finger protein 707	0.99	0.96	0.89	0.73
OFD1	OFD1;71-7A;CXorf5;JBTS10;MGC117039;MGC117040;SGBS2	oral-facial-digital syndrome 1	0.99	0.89	0.89	0.6
TXLNG	TXLNG;CXorf15;ELRG;FIAT;FLJ11209;FLJ33481;LSR5;MGC126621;MGC126625	taxilin gamma	0.99	0.9	0.89	0.71
SRSF9	SRSF9;SFRS9;SRp30c	serine/arginine-rich splicing factor 9	0.99	0.84	0.89	0.71
DHRS2	DHRS2;HEP27;SDR25C1	dehydrogenase/reductase (SDR family) member 2	0.98	0.96	0.89	0.66
C19orf25	C19orf25;FLJ36666	chromosome 19 open reading frame 25	0.98	0.84	0.89	0.65
UTRN	UTRN;DMDL;DRP;DRP1;FLJ23678	utrophin	0.98	0.82	0.89	0.64
FXYD4	FXYD4;CHIF	FXYD domain containing ion transport regulator 4	0.97	0.9	0.89	0.69
PTK2B	PTK2B;CADTK;CAKB;FADK2;FAK2;PKB;PTK;PYK2;RAFTK	PTK2B protein tyrosine kinase 2 beta	0.97	0.92	0.89	0.66
HIST1H4H	HIST1H4H;H4/h;H4FH	histone cluster 1, H4h	0.97	0.89	0.89	0.67
SMNDC1	SMNDC1;SMNR;SPF30	survival motor neuron domain containing 1	0.97	0.88	0.89	0.67
DCAF5	DCAF5;BCRG2;BCRP2;D14S1461E;DKFZp434A035;KIAA1824;WDR22	DDB1 and CUL4 associated factor 5	0.97	0.84	0.89	0.63
SLC43A3	SLC43A3;DKFZp762A227;EEG1;FOAP-13;PRO1659;SEEEG-1	solute carrier family 43, member 3	0.96	0.88	0.89	0.59
CDC42SE2	CDC42SE2;FLJ21967;SPEC2	CDC42 small effector 2	0.96	0.84	0.89	0.62
FLT3	FLT3;CD135;FLK-2;FLK2;STK1	fms-related tyrosine kinase 3	0.95	0.84	0.89	0.7
PDE2A	PDE2A;CGS-PDE;PDE2A1;PED2A4;cGSPDE	phosphodiesterase 2A, cGMP-stimulated	0.95	0.86	0.89	0.57
ASPHD1	ASPHD1	aspartate beta-hydroxylase domain containing 1	0.95	0.92	0.89	0.7
OR10G8	OR10G8;OR11-274;OR11-282	olfactory receptor, family 10, subfamily G, member 8	0.94	0.88	0.89	0.71
C15orf58	C15orf58;VTC2	chromosome 15 open reading frame 58	0.94	0.79	0.89	0.67
LDLRAD1	LDLRAD1	low density lipoprotein receptor class A domain containing 1	0.94	0.84	0.89	0.6
EPHX2	EPHX2;CEH;SEH	epoxide hydrolase 2, cytoplasmic	0.94	0.78	0.89	0.69
LMBR1L	LMBR1L;FLJ10494;FLJ36251;KIAA1174;LIMR	limb region 1 homolog (mouse)-like	0.94	0.86	0.89	0.66
TCTN1	TCTN1;FLJ21127;JBTS13;TECT1	tectonic family member 1	0.94	0.91	0.89	0.61
GGCX	GGCX;FLJ26629;VKCFD1	gamma-glutamyl carboxylase	0.94	0.8	0.89	0.58
HBS1L	HBS1L;DKFZp686L13262;EF-1a;ERFS;HBS1;HSPC276	HBS1-like (S. cerevisiae)	0.94	0.8	0.89	0.59
FERD3L	FERD3L;MGC119861;N-TWIST;NATO3;NTWIST;bHLHa31	Fer3-like (Drosophila)	0.93	0.9	0.89	0.66
LRRC25	LRRC25;FLJ38116;MAPA	leucine rich repeat containing 25	0.93	0.84	0.89	0.64
FKBPL	FKBPL;DIR1;NG7;WISP39	FK506 binding protein like	0.93	0.86	0.89	0.64
LONP2	LONP2;LONP;LONPL;MGC4840	lon peptidase 2, peroxisomal	0.93	0.82	0.89	0.61
YWHAB	YWHAB;GW128;HS1;KCIP-1;YWHAA	tyrosine 3-monooxygenase/tryptophan 5-monooxygenase activation protein, beta polypeptide	0.93	0.84	0.89	0.58
NFE2	NFE2;NF-E2;p45	nuclear factor (erythroid-derived 2), 45kDa	0.92	0.8	0.89	0.59
CALHM1	CALHM1;FAM26C;MGC39514;MGC39617	calcium homeostasis modulator 1	0.92	0.84	0.89	0.58
CA4	CA4;CAIV;Car4;RP17	carbonic anhydrase IV	0.92	0.78	0.89	0.57
SOHLH1	SOHLH1;C9orf157;NOHLH;TEB2;bA100C15.3;bHLHe80	spermatogenesis and oogenesis specific basic helix-loop-helix 1	0.92	0.82	0.89	0.59
KCNK7	KCNK7;K2p7.1;MGC118782;MGC118784;TWIK3	potassium channel, subfamily K, member 7	0.92	0.86	0.89	0.67
E2F5	E2F5;E2F-5	E2F transcription factor 5, p130-binding	0.92	0.79	0.89	0.68
SLC35F2	SLC35F2;DKFZp667H1615;FLJ13018;HSNOV1	solute carrier family 35, member F2	0.92	0.84	0.89	0.64
ASNA1	ASNA1;ARSA-I;ARSA1;ASNA-I;GET3;MGC3821;TRC40;hASNA-I	arsA arsenite transporter, ATP-binding, homolog 1 (bacterial)	0.92	0.82	0.89	0.61
CCT4	CCT4;CCT-DELTA;Cctd;MGC126164;MGC126165;SRB	chaperonin containing TCP1, subunit 4 (delta)	0.92	0.88	0.89	0.65
MB21D1	MB21D1;C6orf150;MGC131892;MGC142166;MGC142168	Mab-21 domain containing 1	0.92	0.86	0.89	0.65
BIN2	BIN2;BRAP-1	bridging integrator 2	0.91	0.76	0.89	0.64
PLA2G4C	PLA2G4C;CPLA2-gamma;DKFZp586C0423;FLJ42247;FLJ44164	phospholipase A2, group IVC (cytosolic, calcium-independent)	0.91	0.9	0.89	0.62
CCDC125	CCDC125;KENAE	coiled-coil domain containing 125	0.91	0.8	0.89	0.58
MXI1	MXI1;MAD2;MGC43220;MXD2;MXI;bHLHc11	MAX interactor 1	0.91	0.82	0.89	0.63
SPIN2A	SPIN2A;DXF34;MGC88670;RP3-323P24.2;SPIN2;dJ323P24.1	spindlin family, member 2A	0.9	0.87	0.89	0.58
CRIPAK	CRIPAK;FLJ34443;MGC150528	cysteine-rich PAK1 inhibitor	0.9	0.77	0.89	0.59
SNCA	SNCA;MGC110988;NACP;PARK1;PARK4;PD1	synuclein, alpha (non A4 component of amyloid precursor)	0.9	0.78	0.89	0.59
IRGQ	IRGQ;FLJ12521;FLJ38849;FLJ42796;FLJ46713;IRGQ1	immunity-related GTPase family, Q	0.9	0.78	0.89	0.57
SLC38A9	SLC38A9;FLJ46104;FLJ90709;MGC120544	solute carrier family 38, member 9	0.9	0.88	0.89	0.64
UBE2O	UBE2O;E2-230K;FLJ12878;KIAA1734	ubiquitin-conjugating enzyme E2O	0.9	0.74	0.89	0.55
NEDD8	NEDD8;FLJ43224;MGC104393;MGC125896;MGC125897;NEDD-8	neural precursor cell expressed, developmentally down-regulated 8	0.9	0.8	0.89	0.63
MAL2	MAL2	mal, T-cell differentiation protein 2 (gene/pseudogene)	0.9	0.84	0.89	0.67
PLAC1	PLAC1;CT92	placenta-specific 1	0.89	0.77	0.89	0.61
LCE3B	LCE3B;LEP14	late cornified envelope 3B	0.89	0.78	0.89	0.62
DSTYK	DSTYK;DustyPK;KIAA0472;RIP5;RIPK5	dual serine/threonine and tyrosine protein kinase	0.89	0.79	0.89	0.65
FAM71C	FAM71C;MGC39520	family with sequence similarity 71, member C	0.88	0.75	0.89	0.57
PLA2G5	PLA2G5;DKFZp686C2294;GV-PLA2;MGC46205;PLA2-10;hVPLA(2)	phospholipase A2, group V	0.88	0.82	0.89	0.63
HAP1	HAP1;HAP2;HIP5;HLP;hHLP1	huntingtin-associated protein 1	0.88	0.83	0.89	0.58
ALOXE3	ALOXE3;E-LOX;MGC119694;MGC119695;MGC119696;eLOX3	arachidonate lipoxygenase 3	0.88	0.79	0.89	0.61
CLCNKB	CLCNKB;CLCKB;ClC-K2;ClC-Kb;MGC24087;hClC-Kb	chloride channel Kb	0.88	0.78	0.89	0.61
TLR3	TLR3;CD283	toll-like receptor 3	0.88	0.74	0.89	0.54
RFNG	RFNG	RFNG O-fucosylpeptide 3-beta-N-acetylglucosaminyltransferase	0.88	0.77	0.89	0.6

EMID2	EMID2;COL26A1;EMI6;EMU2;MGC129848;SH2B	EMI domain containing 2	0.88	0.8	0.89	0.54
IFRD2	IFRD2;FLJ40446;IFNRP;SKMc15;SM15	interferon-related developmental regulator 2	0.88	0.82	0.89	0.61
HDAC1	HDAC1;DKFZp686H12203;GON-10;HD1;RPD3;RPD3L1	histone deacetylase 1	0.88	0.82	0.89	0.63
ECH1	ECH1;HPXEL	enoyl CoA hydratase 1, peroxisomal	0.88	0.79	0.89	0.62
PCDHA6	PCDHA6;CNR2;CNRN2;CNRS2;CRNR2;PCDH-ALPHA6	protocadherin alpha 6	0.88	0.82	0.89	0.58
ZG16B	ZG16B;HRPE773;JCLN2;PAUF;PRO1567	zymogen granule protein 16 homolog B (rat)	0.88	0.76	0.89	0.57
TRMT2B	TRMT2B;CXorf34;FLJ12687;dJ341D10.3	TRM2 tRNA methyltransferase 2 homolog B (S. cerevisiae)	0.88	0.79	0.89	0.61
C2orf3	C2orf3;DNABF;GCF;TCF9	chromosome 2 open reading frame 3	0.87	0.8	0.89	0.57
ADORA3	ADORA3;A3AR;AD026;bA552M11.5	adenosine A3 receptor	0.86	0.74	0.89	0.67
FAM131A	FAM131A;C3orf40;FLAT715;MGC21688;PRO1378	family with sequence similarity 131, member A	0.86	0.82	0.89	0.63
DBF4B	DBF4B;ASKL1;CHIFB;DRF1;FLJ13087;MGC15009;ZDBF1B	DBF4 homolog B (S. cerevisiae)	0.86	0.68	0.89	0.54
PRSS33	PRSS33;EOS	protease, serine, 33	0.86	0.75	0.89	0.56
PCDHGA7	PCDHGA7;PCDH-GAMMA-A7	protocadherin gamma subfamily A, 7	0.86	0.77	0.89	0.52
TBC1D3G	TBC1D3G	TBC1 domain family, member 3G	0.85	0.79	0.89	0.59
PDSS1	PDSS1;COQ1;DPS;MGC70953;RP13-16H11.3;SPS;TPRT;TPT;TPT 1;hDPS1	prenyl (decaprenyl) diphosphate synthase, subunit 1	0.85	0.73	0.89	0.63
DUSP9	DUSP9;MKP-4;MKP4	dual specificity phosphatase 9	1.69	1.52	0.9	1.18
ANKRD44	ANKRD44;FLJ11570;MGC21968;MGC70444;PP6-ARS-B	ankyrin repeat domain 44	1.55	1.52	0.9	1.12
ARHGAP20	ARHGAP20;KIAA1391;RARHOGAP	Rho GTPase activating protein 20	1.33	1.23	0.9	0.93
KCTD7	KCTD7;EPM3;FLJ32069;FLJ45891	potassium channel tetramerisation domain containing 7	1.31	1.17	0.9	0.92
KCNH4	KCNH4;BEC2;ELK1;Kv12.3	potassium voltage-gated channel, subfamily H (eag-related), member 4	1.27	1.13	0.9	0.88
CCNB1IP1	CCNB1IP1;C14orf18;HEI10	cyclin B1 interacting protein 1, E3 ubiquitin protein ligase	1.25	1.15	0.9	0.91
WDR34	WDR34;MGC20486;bA216B9.3	WD repeat domain 34	1.23	1.13	0.9	0.9
MAST4	MAST4;DKFZp686E18148;DKFZp686N1467;FLJ16540;FLJ33039;KIAA0303	microtubule associated serine/threonine kinase family member 4	1.21	1.08	0.9	0.89
MAP9	MAP9;ASAP;FLJ21159	microtubule-associated protein 9	1.18	1.1	0.9	0.79
KLK2	KLK2;KLK2A2;MGC12201;hK2	kallikrein-related peptidase 2	1.17	1.04	0.9	0.78
CDC14A	CDC14A;cdc14;hCDC14	CDC14 cell division cycle 14 homolog A (S. cerevisiae)	1.17	1.04	0.9	0.82
PSMG3	PSMG3;C7orf48;MGC10911;PAC3	proteasome (prosome, macropain) assembly chaperone 3	1.16	1	0.9	0.77
HIF1A	HIF1A;HIF-1alpha;HIF1;HIF1-ALPHA;MOP1;PASD8;bHLHe78	hypoxia inducible factor 1, alpha subunit (basic helix-loop-helix transcription factor)	1.16	1.04	0.9	0.77
UQCR10	UQCR10;HSPC051;HSPC151;QCR9;UCCR7.2;UCRC	ubiquinol-cytochrome c reductase, complex III subunit X	1.16	1.02	0.9	0.76
AARS2	AARS2;AARSL;COXPD8;KIAA1270;MT-ALARS;MTALARS;bA444E17.1	alanyl-tRNA synthetase 2, mitochondrial (putative)	1.15	1.09	0.9	0.78
BAHD1	BAHD1;KIAA0945	bromo adjacent homology domain containing 1	1.15	1	0.9	0.72
GNA13	GNA13;G13;MGC46138	guanine nucleotide binding protein (G protein), alpha 13	1.15	1.14	0.9	0.79
DCBLD2	DCBLD2;CLCP1;ESDN	discoidin, CUB and LCCL domain containing 2	1.14	0.98	0.9	0.76
ZNF599	ZNF599;FLJ50920	zinc finger protein 599	1.13	1	0.9	0.75
ESRRB	ESRRB;DFNB35;ERR2;ERRb;ESRL2;NR3B2	estrogen-related receptor beta	1.12	0.94	0.9	0.83
SLC40A1	SLC40A1;FPN1;HFE4;IREG1;MST079;MTP1;SLC11A3	solute carrier family 40 (iron-regulated transporter), member 1	1.12	1.07	0.9	0.71
LOC652859	LOC652859	ENTREZ STATUS: DISCONTINUED	1.12	0.98	0.9	0.88
KIR3DL2	KIR3DL2;CD158K;MGC125321;NKAT-4;NKAT4;NKAT4B;p140	killer cell immunoglobulin-like receptor, three domains, long cytoplasmic tail, 2	1.11	0.96	0.9	0.73
PPP2R2C	PPP2R2C;B55-GAMMA;IMYPNO;IMYPNO1;MGC33570;PR52;PR55G	protein phosphatase 2, regulatory subunit B, gamma	1.11	1	0.9	0.78
OR9G1	OR9G1;OR9G5	olfactory receptor, family 9, subfamily G, member 1	1.1	1.03	0.9	0.78
FARSA	FARSA;CML33;FARSL;FARSLA;FRSA;PheHA	phenylalanyl-tRNA synthetase, alpha subunit	1.1	0.99	0.9	0.73
TAGLN2	TAGLN2;HA1756;KIAA0120	transgelin 2	1.09	0.96	0.9	0.72
UBE2D2	UBE2D2;E2(17)KB2;PUBC1;UBC4;UBC4/5;UBCH5B	ubiquitin-conjugating enzyme E2D 2	1.09	0.91	0.9	0.68
KRT33B	KRT33B;HA3II;Ha-3II;KRTHA3A;KRTHA3B;hHa3-II	keratin 33B	1.08	0.95	0.9	0.71
PJA2	PJA2;KIAA0438;Neurodap1;RNF131	praja ring finger 2	1.08	0.96	0.9	0.86
RFESD	RFESD	Rieske (Fe-S) domain containing	1.07	1.04	0.9	0.79
WHSC1	WHSC1;FLJ23286;KIAA1090;MGC176638;MMSET;NSD2;REIIBP;TRX5;WHS	Wolf-Hirschhorn syndrome candidate 1	1.07	0.93	0.9	0.67
FAS	FAS;ALPS1A;APO-1;APT1;CD95;FAS1;FASTM;TNFRSF6	Fas (TNF receptor superfamily, member 6)	1.07	1.04	0.9	0.78
MCRS1	MCRS1;ICP22BP;INO80Q;MCRS2;MSP58;P78	microspherule protein 1	1.07	1.06	0.9	0.77
PIWIL4	PIWIL4;DKFZp686P01248;FLJ36156;HIWI2;MIWI2	piwi-like 4 (Drosophila)	1.06	0.99	0.9	0.67
TRIM13	TRIM13;CAR;DLEU5;LEU5;RFP2;RNF77	tripartite motif containing 13	1.06	0.94	0.9	0.84
SYTL1	SYTL1;FLJ14996;JFC1;SLP1	synaptotagmin-like 1	1.06	0.94	0.9	0.71
PPP2R2D	PPP2R2D;MDS026	protein phosphatase 2, regulatory subunit B, delta	1.06	0.95	0.9	0.75
GLIS2	GLIS2;FLJ38247;NKL;NPHP7	GLIS family zinc finger 2	1.05	0.97	0.9	0.76
DOCK6	DOCK6;AOS2;ZIR1	dedicator of cytokinesis 6	1.05	1.04	0.9	0.76
BCO2	BCO2;B-DIOX-II;BCDO2;FLJ34464	beta-carotene oxygenase 2	1.04	0.94	0.9	0.69
C9orf103	C9orf103;bA522I20.2	chromosome 9 open reading frame 103	1.04	0.88	0.9	0.75
C14orf142	C14orf142;MGC104244;MGC133207;PNAS-127	chromosome 14 open reading frame 142	1.04	0.91	0.9	0.72
PTPMT1	PTPMT1;DUSP23;FLJ46081;MOSP;PLIP	protein tyrosine phosphatase, mitochondrial 1	1.04	0.94	0.9	0.73
CYP27B1	CYP27B1;CP2B;CYP1;CYP1alpha;CYP27B;P450c1;PDDR;VDD1;VDDR;VDDRI;VDR	cytochrome P450, family 27, subfamily B, polypeptide 1	1.03	0.9	0.9	0.71
C1QL3	C1QL3;C1QTNF13;C1ql;CTRP13;K100	complement component 1, q subcomponent-like 3	1.03	1.01	0.9	0.67
SLC12A6	SLC12A6;ACCPN;DKFZp434D2135;KCC3;KCC3A;KCC3B	solute carrier family 12 (potassium/chloride transporters), member 6	1.02	0.9	0.9	0.7
TPP1	TPP1;CLN2;LPIC;MGC21297;TPP-1	tripeptidyl peptidase I	1.02	0.92	0.9	0.69
DNAJC8	DNAJC8;HSPC331;SPF31	DnaJ (Hsp40) homolog, subfamily C, member 8	1.02	0.93	0.9	0.65
RABL2B	RABL2B;FLJ93981;FLJ98216	RAB, member of RAS oncogene family-like 2B	1.02	0.91	0.9	0.67
CACNG1	CACNG1;CACNLG	calcium channel, voltage-dependent, gamma subunit 1	1.01	0.94	0.9	0.73
BCDIN3D	BCDIN3D	BCDIN3 domain containing	1.01	0.86	0.9	0.7
KCMF1	KCMF1;DEBT91;DKFZp434L1021;FIGC;PCMF;ZZZ1	potassium channel modulatory factor 1	1.01	0.93	0.9	0.71
ZNF221	ZNF221;MGC138186;MGC141986	zinc finger protein 221	1	0.94	0.9	0.67
LILRB4	LILRB4;CD85K;HM18;ILT3;LILRB5;LIR-5;LIR5	leukocyte immunoglobulin-like receptor, subfamily B (with TM and ITIM domains), member 4	1	0.86	0.9	0.67
SLC41A1	SLC41A1;MgtE	solute carrier family 41, member 1	1	0.84	0.9	0.74
SHKBP1	SHKBP1;Sb1	SH3KBP1 binding protein 1	1	0.91	0.9	0.62
CDH7	CDH7;CDH7L1	cadherin 7, type 2	0.99	0.86	0.9	0.66
ITPKB	ITPKB;IP3-3KB;IP3K;IP3K-B;IP3KB;PIG37	inositol-trisphosphate 3-kinase B	0.99	0.86	0.9	0.78
OSBPL11	OSBPL11;FLJ13012;FLJ13164;ORP-11;ORP11;OSBP12;TCCCIA00292	oxysterol binding protein-like 11	0.99	0.92	0.9	0.69
MMP16	MMP16;C8orf57;DKFZp761D112;MMP-X2;MT-MMP2;MT-MMP3;MT3-MMP	matrix metallopeptidase 16 (membrane-inserted)	0.98	0.88	0.9	0.66
HOXA2	HOXA2;HOX1K	homeobox A2	0.98	0.88	0.9	0.68
ASTE1	ASTE1;MGC129980	asteroid homolog 1 (Drosophila)	0.98	0.96	0.9	0.74
ATP2C2	ATP2C2;DKFZp686H22230;KIAA0703;SPCA2	ATPase, Ca++ transporting, type 2C, member 2	0.98	0.88	0.9	0.6
MED22	MED22;MED24;MGC48682;SURF5;surf-5	mediator complex subunit 22	0.98	0.86	0.9	0.66
SLC30A1	SLC30A1;ZNT1;ZRC1	solute carrier family 30 (zinc transporter), member 1	0.98	0.87	0.9	0.64
OR5AR1	OR5AR1;OR11-209	olfactory receptor, family 5, subfamily AR, member 1	0.97	0.88	0.9	0.67
CACNA1C	CACNA1C;CACH2;CACN2;CACNL1A1;CCHL1A1;CaV1.2;MGC120730;TS	calcium channel, voltage-dependent, L type, alpha 1C subunit	0.97	0.86	0.9	0.68
ATG12	ATG12;APG12;APG12L;FBR93;HAPG12	ATG12 autophagy related 12 homolog (S. cerevisiae)	0.97	0.88	0.9	0.68
MAP1S	MAP1S;BPY2IP1;C19orf5;FLJ10669;MAP8;MGC133087;VCY2IP-1;VCY2IP1	microtubule-associated protein 1S	0.97	0.82	0.9	0.65
RAX	RAX;MCOP3;RX	retina and anterior neural fold homeobox	0.96	0.82	0.9	0.64
C11orf83	C11orf83;CCDS41658.1;MGC87549;UNQ655	chromosome 11 open reading frame 83	0.96	0.84	0.9	0.64
TRIB1	TRIB1;C8FW;GIG2;SKIP1;TRB1	tribbles homolog 1 (Drosophila)	0.96	0.85	0.9	0.7
RAB31	RAB31;Rab22B	RAB31, member RAS oncogene family	0.96	0.88	0.9	0.7
C10orf12	C10orf12;DKFZp564P1916;FLJ13022	chromosome 10 open reading frame 12	0.96	0.85	0.9	0.64
MYH14	MYH14;DFNA4;DKFZp667A1311;FLJ13881;FLJ43092;KIAA2034;MHC16;MYH17;NMHC II-C;NMHC-II-C;myosin	myosin, heavy chain 14, non-muscle	0.96	0.84	0.9	0.63
GPBP1	GPBP1;DKFZp761C169;GPBP;MGC126339;SSH6;VASCULIN	GC-rich promoter binding protein 1	0.96	0.9	0.9	0.64
ZNHIT6	ZNHIT6;BCD1;C1orf181;FLJ12602;FLJ20729;FLJ20760;MGC131963;NY-BR-75	zinc finger, HIT-type containing 6	0.96	0.85	0.9	0.64
XPOT	XPOT;XPO3	exportin, tRNA (nuclear export receptor for tRNAs)	0.96	0.88	0.9	0.59
CSNK2A1	CSNK2A1;CK2A1;CKII	casein kinase 2, alpha 1 polypeptide	0.96	0.94	0.9	0.66
PMEL	PMEL;D12S53E;ME20;ME20-M;ME20M;P1;P100;PMEL17;SI;SIL;SILV;gp100	premelanosome protein	0.96	0.92	0.9	0.61
HRASLS	HRASLS;A-C1;H-REV107;HRASLS1;HSD28	HRAS-like suppressor	0.95	0.84	0.9	0.65
PRR4	PRR4;DKFZp779L1763;FLJ18757;LPRP;PROL4	proline rich 4 (lacrimal)	0.94	0.86	0.9	0.58
PRKCQ	PRKCQ;MGC126514;MGC141919;PRKCT;nPKC-theta	protein kinase C, theta	0.94	0.91	0.9	0.65
EPB41	EPB41;4.1R;EL1;HE	erythrocyte membrane protein band 4.1 (elliptocytosis 1, RH-linked)	0.94	0.84	0.9	0.59
VAT1	VAT1;FLJ20230;VATI	vesicle amine transport protein 1 homolog (T. californica)	0.94	0.79	0.9	0.63
BOLA2B	BOLA2B;BOLA2	bolA homolog 2B (E. coli)	0.94	0.76	0.9	0.64
CKMT1B	CKMT1B;CKMT;CKMT1;CKMT1A;UMTCK	creatine kinase, mitochondrial 1B	0.94	0.86	0.9	0.71
C7orf28B	C7orf28B;DKFZp586I1023;FLJ60592;H_NH0577018.2;MGC19819	chromosome 7 open reading frame 28B	0.94	0.83	0.9	0.63
SIGLEC6	SIGLEC6;CD327;CD33L;CD33L1;CD33L2;CDW327;OBBP1	sialic acid binding Ig-like lectin 6	0.93	0.78	0.9	0.67
DCTPP1	DCTPP1;MGC5627;RS21C6;XTP3TPA	dCTP pyrophosphatase 1	0.93	0.83	0.9	0.69
SLC45A2	SLC45A2;1A1;AIM1;MATP;SHEP5	solute carrier family 45, member 2	0.92	0.9	0.9	0.6
KIR2DL4	KIR2DL4;CD158D;G9P;KIR103;KIR103AS	killer cell immunoglobulin-like receptor, two domains, long cytoplasmic tail, 4	0.92	0.79	0.9	0.61
RAB42	RAB42;MGC45806;RP4-669K10.6	RAB42, member RAS oncogene family	0.92	0.9	0.9	0.66
C20orf165	C20orf165;TSG23;dJ337O18.8	chromosome 20 open reading frame 165	0.92	0.85	0.9	0.57
PLA2G2E	PLA2G2E	phospholipase A2, group IIE	0.92	0.8	0.9	0.57
C16orf48	C16orf48;DAKV6410;DKFZp434A1319	chromosome 16 open reading frame 48	0.92	0.85	0.9	0.63
CYP2W1	CYP2W1;MGC34287	cytochrome P450, family 2, subfamily W, polypeptide 1	0.92	0.84	0.9	0.66
OPN3	OPN3;ECPN	opsin 3	0.92	0.77	0.9	0.58
GMNN	GMNN;Gem	geminin, DNA replication inhibitor	0.92	0.86	0.9	0.59
PAF1	PAF1;F23149_1;FLJ11123;PD2	Paf1, RNA polymerase II associated factor, homolog (S. cerevisiae)	0.92	0.9	0.9	0.61
WNT7A	WNT7A	wingless-type MMTV integration site family, member 7A	0.92	0.91	0.9	0.66
CLMP	CLMP;ACAM;ASAM;FLJ22415	CXADR-like membrane protein	0.92	0.74	0.9	0.62
TMX1	TMX1;DKFZp564E1962;PDIA11;TMX;TXNDC;TXNDC1	thioredoxin-related transmembrane protein 1	0.92	0.79	0.9	0.58
C20orf106	C20orf106;MGC104272;dJ1153D9.3	chromosome 20 open reading frame 106	0.9	0.84	0.9	0.63
C16orf82	C16orf82;FLJ18616;TNT	chromosome 16 open reading frame 82	0.9	0.84	0.9	0.64
ZNF224	ZNF224;BMZF-2;BMZF2;KOX22;ZNF255;ZNF27	zinc finger protein 224	0.9	0.78	0.9	0.63
IPMK	IPMK	inositol polyphosphate multikinase	0.9	0.78	0.9	0.7
L3MBTL2	L3MBTL2;H-l(3)mbt-l;L3MBT	l(3)mbt-like 2 (Drosophila)	0.9	0.8	0.9	0.6
UBE2D1	UBE2D1;E2(17)KB1;SFT;UBC4/5;UBCH5;UBCH5A	ubiquitin-conjugating enzyme E2D 1	0.9	0.74	0.9	0.56
TRAPPC4	TRAPPC4;HSPC172;PTD009;SBDN;SYNBINDIN;TRS23	trafficking protein particle complex 4	0.9	0.82	0.9	0.56
BRD2	BRD2;D6S113E;DKFZp686N0336;FLJ31942;FSH;FSRG1;KIAA9001;NAT;RING3;RNF3	bromodomain containing 2	0.9	0.8	0.9	0.67
CCDC91	CCDC91;DKFZp779L1558;FLJ11088;p56	coiled-coil domain containing 91	0.89	0.76	0.9	0.59
WDR4	WDR4;TRM82	WD repeat domain 4	0.89	0.78	0.9	0.58

FAM102B	FAM102B;DKFZp686N01110;DKFZp779B126	family with sequence similarity 102, member B	0.88	0.86	0.9	0.6
PRAMEF12	PRAMEF12	PRAME family member 12	0.88	0.86	0.9	0.6
MCART6	MCART6;DKFZp686O1267	mitochondrial carrier triple repeat 6	0.88	0.82	0.9	0.62
KATNAL2	KATNAL2;DKFZp667C165;MGC33211	katanin p60 subunit A-like 2	0.88	0.82	0.9	0.62
PAX6	PAX6;AN;AN2;D11S812E;MGC17209;MGDA;WAGR	paired box 6	0.88	0.84	0.9	0.65
GRIN2C	GRIN2C;NMDAR2C;NR2C	glutamate receptor, ionotropic, N-methyl D-aspartate 2C	0.88	0.82	0.9	0.64
LYPD3	LYPD3;C4.4A	LY6/PLAUR domain containing 3	0.88	0.85	0.9	0.57
ENPP1	ENPP1;ARHR2;M6S1;NPP1;NPPS;PC-1;PCA1;PDNP1	ectonucleotide pyrophosphatase/phosphodiesterase 1	0.88	0.86	0.9	0.6
INPP5B	INPP5B;5PTase;MGC65156;MGC71303	inositol polyphosphate-5-phosphatase, 75kDa	0.88	0.79	0.9	0.62
KLF13	KLF13;BTEB3;FKLF2;NSLP1;RFLAT-1;RFLAT1	Kruppel-like factor 13	0.88	0.74	0.9	0.58
C19orf12	C19orf12;DKFZp762D096;MGC10922	chromosome 19 open reading frame 12	0.88	0.79	0.9	0.53
SH3BP4	SH3BP4;BOG25;TTP	SH3-domain binding protein 4	0.88	0.8	0.9	0.54
ZNF3	ZNF3;A8-51;FLJ20216;HF.12;KOX25;PP838;Zfp113	zinc finger protein 3	0.88	0.82	0.9	0.55
KIAA0240	KIAA0240;DKFZp686P0250	KIAA0240	0.87	0.83	0.9	0.58
MBD4	MBD4;MED1	methyl-CpG binding domain protein 4	0.87	0.78	0.9	0.57
SAT1	SAT1;DC21;KFSD;KFSDX;SAT;SSAT;SSAT-1	spermidine/spermine N1-acetyltransferase 1	0.87	0.77	0.9	0.58
GRXCR1	GRXCR1;DFNB25	glutaredoxin, cysteine rich 1	0.87	0.76	0.9	0.58
MYO3B	MYO3B	myosin IIIB	0.86	0.77	0.9	0.64
C4orf42	C4orf42;MGC149426;MGC149427;MGC21675	chromosome 4 open reading frame 42	0.86	0.73	0.9	0.62
ARHGEF10L	ARHGEF10L;FLJ10521;GrinchGEF;KIAA1626	Rho guanine nucleotide exchange factor (GEF) 10-like	0.86	0.84	0.9	0.56
CHCHD7	CHCHD7;FLJ40966;MGC2217	coiled-coil-helix-coiled-coil-helix domain containing 7	0.86	0.78	0.9	0.55
PHACTR1	PHACTR1;KIAA1733;MGC126575;MGC126577;RPEL;RPEL1;dJ257A7.2	phosphatase and actin regulator 1	0.85	0.84	0.9	0.61
PFKFB3	PFKFB3;FLJ37326;IPFK2;PFK2	6-phosphofructo-2-kinase/fructose-2,6-biphosphatase 3	0.85	0.76	0.9	0.63
SEPHS1	SEPHS1;MGC4980;SELD;SPS;SPS1	selenophosphate synthetase 1	0.85	0.71	0.9	0.56
TLR2	TLR2;CD282;TIL4	toll-like receptor 2	1.33	1.23	0.91	0.97
NMT1	NMT1;NMT	N-myristoyltransferase 1	1.29	1.25	0.91	0.83
OR2H1	OR2H1;6M1-16;HS6M1-16;OLFR42A-9004-14;OR2H6;OR2H8;OR6-2;dJ994E9.4	olfactory receptor, family 2, subfamily H, member 1	1.26	1.1	0.91	0.84
SIDT2	SIDT2;DKFZp686L17253;FLJ90656	SID1 transmembrane family, member 2	1.23	1.14	0.91	0.79
SMG6	SMG6;C17orf31;EST1A;KIAA0732;SMG-6	smg-6 homolog, nonsense mediated mRNA decay factor (C. elegans)	1.21	1.07	0.91	0.8
ARMCX2	ARMCX2;ALEX2;KIAA0512;MGC13343;MGC8742	armadillo repeat containing, X-linked 2	1.21	1.06	0.91	0.81
LRSAM1	LRSAM1;FLJ31641;RIFLE;TAL	leucine rich repeat and sterile alpha motif containing 1	1.2	1.05	0.91	0.76
CENPQ	CENPQ;C6orf139;CENP-Q;FLJ10545	centromere protein Q	1.2	1.1	0.91	0.79
GNAO1	GNAO1;DKFZp686O0962;G-ALPHA-o;GNAO	guanine nucleotide binding protein (G protein), alpha activating activity polypeptide O	1.19	1.12	0.91	0.75
SLC20A1	SLC20A1;DKFZp686J2397;FLJ41426;GLVR1;Glvr-1;PIT1;PiT-1	solute carrier family 20 (phosphate transporter), member 1	1.18	1.14	0.91	0.75
ASB16	ASB16;FLJ30165;MGC141876;MGC141877	ankyrin repeat and SOCS box containing 16	1.17	1.01	0.91	0.73
PCDH9	PCDH9	protocadherin 9	1.16	1.08	0.91	0.85
USP44	USP44;DKFZp434D0127;FLJ14528	ubiquitin specific peptidase 44	1.16	1.03	0.91	0.78
BIRC7	BIRC7;KIAP;LIVIN;ML-IAP;MLIAP;RNF50	baculoviral IAP repeat containing 7	1.16	1	0.91	0.72
SLC25A33	SLC25A33;BMSC-MCP;MGC4399	solute carrier family 25, member 33	1.16	1.12	0.91	0.86
ESX1	ESX1;ESX1L;ESXR1	ESX homeobox 1	1.14	0.99	0.91	0.8
AFF2	AFF2;FMR2;FMR2P;FRAXE;MRX2;OX19	AF4/FMR2 family, member 2	1.14	1.1	0.91	0.78
ZNF410	ZNF410;APA-1;APA1	zinc finger protein 410	1.14	1.12	0.91	0.83
DSC2	DSC2;ARVD11;CDHF2;DG2;DGII/III;DKFZp686I11137;DSC3	desmocollin 2	1.12	1.03	0.91	0.82
GEMIN6	GEMIN6;FLJ23459	gem (nuclear organelle) associated protein 6	1.12	1.04	0.91	0.79
FAT4	FAT4;CDHF14;CDHR11;FAT-J;FATJ;FLJ23056;FLJ77878;NBLA00548	FAT tumor suppressor homolog 4 (Drosophila)	1.11	0.96	0.91	0.81
IQCH	IQCH;DKFZp434F2114;FLJ12476;NYDSP5	IQ motif containing H	1.1	1.03	0.91	0.77
UNC93A	UNC93A;MGC119395;MGC119397;Unc-93A;dJ366N23.1;dJ366N23.2	unc-93 homolog A (C. elegans)	1.1	1.04	0.91	0.74
NT5C3	NT5C3;MGC27337;MGC87109;MGC87828;P5'N-1;PN-I;PSN1;UMPH;UMPH1;cN-III	5'-nucleotidase, cytosolic III	1.1	0.94	0.91	0.7
CLIP2	CLIP2;CLIP;CLIP-115;CYLN2;KIAA0291;MGC11333;WBSCR3;WBSCR4;WSCR3;WSCR4	CAP-GLY domain containing linker protein 2	1.1	0.93	0.91	0.74
ACP6	ACP6;ACPL1;LPAP;PACPL1	acid phosphatase 6, lysophosphatidic	1.1	0.93	0.91	0.7
SENP5	SENP5;DKFZp564O1016;FLJ42398;MGC27076	SUMO1/sentrin specific peptidase 5	1.1	1	0.91	0.75
GJA1	GJA1;AVSD3;CX43;DFNB38;GJAL;HSS;ODDD	gap junction protein, alpha 1, 43kDa	1.1	0.96	0.91	0.71
ALOX12	ALOX12;12-LOX;12S-LOX;LOG12	arachidonate 12-lipoxygenase	1.09	0.99	0.91	0.73
TMEM63A	TMEM63A;KIAA0489;KIAA0792	transmembrane protein 63A	1.09	1.08	0.91	0.72
POLB	POLB;MGC125976	polymerase (DNA directed), beta	1.09	1.03	0.91	0.8
GRAMD1C	GRAMD1C;DKFZp434C0328;DKFZp686K06117;FLJ35862;FLJ40464	GRAM domain containing 1C	1.08	0.96	0.91	0.72
LRRC4	LRRC4;MGC133342;MGC133343;NGL-2	leucine rich repeat containing 4	1.08	0.97	0.91	0.81
NXPH1	NXPH1;NPH1	neurexophilin 1	1.08	1.01	0.91	0.79
RAB28	RAB28;MGC41862	RAB28, member RAS oncogene family	1.08	1.05	0.91	0.69
USP45	USP45;MGC14793	ubiquitin specific peptidase 45	1.08	0.98	0.91	0.73
ETFDH	ETFDH;ETFQO;MADD	electron-transferring-flavoprotein dehydrogenase	1.08	1	0.91	0.79
SGMS2	SGMS2;MGC26963;SMS2	sphingomyelin synthase 2	1.08	0.96	0.91	0.74
SURF2	SURF2;SURF-2	surfeit 2	1.08	1.01	0.91	0.77
USP34	USP34;FLJ43910;KIAA0570;KIAA0729;MGC104459	ubiquitin specific peptidase 34	1.08	0.98	0.91	0.73
API5	API5;AAC-11;AAC11;FLJ11587	apoptosis inhibitor 5	1.08	1.02	0.91	0.77
SKOR2	SKOR2;CORL2;FUSSEL18	SKI family transcriptional corepressor 2	1.08	1.02	0.91	0.77
ERAS	ERAS;HRAS2;HRASP;MGC126691;MGC126693	ES cell expressed Ras	1.06	0.94	0.91	0.75
FBXL7	FBXL7;FBL6;FBL7	F-box and leucine-rich repeat protein 7	1.06	0.92	0.91	0.66
DUSP2	DUSP2;PAC-1;PAC1	dual specificity phosphatase 2	1.06	0.97	0.91	0.76
SNX17	SNX17;KIAA0064	sorting nexin 17	1.06	0.99	0.91	0.76
CRYBA1	CRYBA1;CRYB1	crystallin, beta A1	1.05	1	0.91	0.75
RASGEF1B	RASGEF1B;FLJ31695;GPIG4;MGC46251	RasGEF domain family, member 1B	1.05	0.98	0.91	0.75
IGF2BP1	IGF2BP1;CRD-BP;CRDBP;IMP-1;IMP1;VICKZ1;ZBP1	insulin-like growth factor 2 mRNA binding protein 1	1.05	0.92	0.91	0.7
CORIN	CORIN;ATC2;CRN;Lrp4;MGC119742;TMPRSS10	corin, serine peptidase	1.04	0.94	0.91	0.7
RIPK3	RIPK3;RIP3	receptor-interacting serine-threonine kinase 3	1.04	1.02	0.91	0.73
SH3BP1	SH3BP1;ARHGAP43	SH3-domain binding protein 1	1.04	0.98	0.91	0.77
ESCO1	ESCO1;A930014I12Rik;CTF;ECO1;EFO1;ESO1;KIAA1911;MGC105022	establishment of cohesion 1 homolog 1 (S. cerevisiae)	1.04	0.93	0.91	0.69
JMJD1C	JMJD1C;DKFZp761F0118;FLJ14374;KIAA1380;RP11-10C13.2;TRIP8	jumonji domain containing 1C	1.04	0.92	0.91	0.72
ATP5G1	ATP5G1;ATP5A;ATP5G	ATP synthase, H+ transporting, mitochondrial Fo complex, subunit C1 (subunit 9)	1.04	0.9	0.91	0.76
LCMT1	LCMT1;LCMT;PPMT1	leucine carboxyl methyltransferase 1	1.04	0.92	0.91	0.65
PCDHGA3	PCDHGA3;PCDH-GAMMA-A3	protocadherin gamma subfamily A, 3	1.04	0.95	0.91	0.66
TRPV1	TRPV1;DKFZp434K0220;VR1	transient receptor potential cation channel, subfamily V, member 1	1.03	0.98	0.91	0.76
SLC9A1	SLC9A1;APNH;FLJ42224;NHE-1;NHE1	solute carrier family 9 (sodium/hydrogen exchanger), member 1	1.03	0.92	0.91	0.73
PCID2	PCID2;DKFZp686C20226;F10;FLJ11305;FLJ99362;MGC16774	PCI domain containing 2	1.03	0.93	0.91	0.67
DCDC2	DCDC2;DCDC2A;RU2;RU2S	doublecortin domain containing 2	1.03	0.96	0.91	0.73
SNCB	SNCB	synuclein, beta	1.02	0.9	0.91	0.64
TMEM204	TMEM204;C16orf30;CLP24;FLJ20898;MGC111564	transmembrane protein 204	1.02	0.9	0.91	0.68
C16orf87	C16orf87;MGC62018	chromosome 16 open reading frame 87	1.02	0.84	0.91	0.7
EMP1	EMP1;CL-20;EMP-1;TMP	epithelial membrane protein 1	1.02	0.96	0.91	0.65
GLIPR1	GLIPR1;CRISP7;GLIPR;RTVP1	GLI pathogenesis-related 1	1.02	0.97	0.91	0.68
SPINK8	SPINK8	serine peptidase inhibitor, Kazal type 8 (putative)	1.02	0.96	0.91	0.69
CCNO	CCNO;CCNU;FLJ22422;UDG2;UNG2	cyclin O	1.01	0.86	0.91	0.68
STRA13	STRA13;CENP-X;FAAP10;MGC14480;MHF2	stimulated by retinoic acid 13 homolog (mouse)	1.01	0.95	0.91	0.72
FAM96B	FAM96B	family with sequence similarity 96, member B	1.01	0.82	0.91	0.64
SNX2	SNX2;MGC5204;TRG-9	sorting nexin 2	1.01	1.02	0.91	0.74
COL6A5	COL6A5;COL29A1;FLJ35880;VWA4	collagen, type VI, alpha 5	1.01	0.92	0.91	0.64
OR6K6	OR6K6;OR1-21	olfactory receptor, family 6, subfamily K, member 6	1	0.86	0.91	0.66
PCDH20	PCDH20;FLJ22218;PCDH13	protocadherin 20	1	0.88	0.91	0.71
ODZ2	ODZ2;DKFZp686A1568;TEN-M2;TNM2	odz, odd Oz/ten-m homolog 2 (Drosophila)	1	0.94	0.91	0.67
FHOD3	FHOD3;FHOS2;Formactin2	formin homology 2 domain containing 3	1	0.94	0.91	0.71
MAMDC2	MAMDC2;MGC21981	MAM domain containing 2	1	0.97	0.91	0.68
C17orf76	C17orf76;FLJ35696	chromosome 17 open reading frame 76	1	0.82	0.91	0.63
TMEM104	TMEM104;FLJ00021;FLJ20255	transmembrane protein 104	1	0.82	0.91	0.63
NRN1L	NRN1L;MGC118990;MGC118993;UNQ2446	neuritin 1-like	1	0.91	0.91	0.66
PAX3	PAX3;CDHS;HUP2;MGC120381;MGC120382;MGC120383;MGC120384;MGC134778;WS1;WS3	paired box 3	0.99	0.9	0.91	0.7
TSSK1B	TSSK1B;SPOGA4;STK22D;TSK1;TSSK1	testis-specific serine kinase 1B	0.99	0.89	0.91	0.74
ATCAY	ATCAY;BNIP-H;CLAC;KIAA1872	ataxia, cerebellar, Cayman type	0.99	0.92	0.91	0.7
FKBP9	FKBP9;DKFZp586B1723;FKBP60;FKBP63;MGC126772;MGC138258;PPIase	FK506 binding protein 9, 63 kDa	0.99	0.84	0.91	0.74
OSGEP	OSGEP;FLJ20411;GCPL1;KAE1;OSGEP1;PRSMG1	O-sialoglycoprotein endopeptidase	0.99	0.82	0.91	0.62
CDC20B	CDC20B;FLJ37927	cell division cycle 20 homolog B (S. cerevisiae)	0.98	0.99	0.91	0.72
TRPM6	TRPM6;CHAK2;HMGX;HOMG;HOMG1;HSH	transient receptor potential cation channel, subfamily M, member 6	0.98	0.88	0.91	0.73
SPRED1	SPRED1;FLJ33903;NFLS	sprouty-related, EVH1 domain containing 1	0.98	0.8	0.91	0.64
POLD4	POLD4;POLDS;p12	polymerase (DNA-directed), delta 4	0.98	0.93	0.91	0.72
PLEKHA3	PLEKHA3;FAPP1;FLJ20067	pleckstrin homology domain containing, family A (phosphoinositide binding specific) member 3	0.98	0.91	0.91	0.65
ZNF644	ZNF644;BM-005;KIAA1221;MGC60165;MGC70410;MYP21;NatF;ZEP-2	zinc finger protein 644	0.98	0.86	0.91	0.65
NUDC	NUDC;HNUDC;MNUDC;NPD011	nuclear distribution gene C homolog (A. nidulans)	0.98	0.9	0.91	0.69
CTTN	CTTN;EMS1;FLJ34459	cortactin	0.98	0.92	0.91	0.7
FAM75A2	FAM75A2;FLJ56363	family with sequence similarity 75, member A2	0.98	0.84	0.91	0.68
TIMD4	TIMD4;FLJ27515;SMUCKLER;TIM4	T-cell immunoglobulin and mucin domain containing 4	0.97	0.78	0.91	0.61
FCGR2C	FCGR2C;CD32;CD32C;CDW32;FCG2;FCRIIC;IGFR2	Fc fragment of IgG, low affinity IIc, receptor for (CD32) (gene/pseudogene)	0.97	0.9	0.91	0.69
UBL4B	UBL4B;FLJ25690	ubiquitin-like 4B	0.96	0.92	0.91	0.65
NUDT4	NUDT4;DIPP2;DIPP2alpha;DIPP2beta;DKFZp686I1281;KIAA0487	nudix (nucleoside diphosphate linked moiety X)-type motif 4	0.96	0.8	0.91	0.61
BHMT	BHMT;BHMT1	betaine--homocysteine S-methyltransferase	0.96	0.89	0.91	0.7
PSD	PSD;KIAA2011;PSD1;TYL	pleckstrin and Sec7 domain containing	0.96	0.83	0.91	0.7
ALPI	ALPI;IAP	alkaline phosphatase, intestinal	0.96	0.82	0.91	0.62
NXNL1	NXNL1;RDCVF;TXNL6	nucleoredoxin-like 1	0.96	0.88	0.91	0.59
GAS8	GAS8;GAS11;MGC138326	growth arrest-specific 8	0.96	0.86	0.91	0.68
NFKB2	NFKB2;H2TF1;LYT-10;LYT10;p52	nuclear factor of kappa light polypeptide gene enhancer in B-cells 2 (p49/p100)	0.96	0.86	0.91	0.66
USP53	USP53;DKFZp781E1417	ubiquitin specific peptidase 53	0.96	0.84	0.91	0.62

RNF121	RNF121;FLJ11099	ring finger protein 121	0.96	0.86	0.91	0.78
DDAH1	DDAH1;DDAH;FLJ21264;FLJ25539	dimethylarginine dimethylaminohydrolase 1	0.96	0.87	0.91	0.68
BOLA2	BOLA2;BOLA2A;My016	bolA homolog 2 (E. coli)	0.96	0.82	0.91	0.69
C4B	C4B;C4B1;C4B12;C4B2;C4B3;C4B5;C4F;CH;CO4;CPAMD3;FLJ60561;MGC164979	complement component 4B (Chido blood group)	0.96	0.85	0.91	0.64
GBAS	GBAS;NIPSNAP2	glioblastoma amplified sequence	0.95	0.9	0.91	0.68
PPME1	PPME1;FLJ22226;PME-1	protein phosphatase methylesterase 1	0.95	0.93	0.91	0.69
HIST1H3I	HIST1H3I;H3.f;H3/f;H3FF;HIST1H3A;HIST1H3B;HIST1H3C;HIST1H3D;HIST1H3E;HIST1H3F;HIST1H3G;HIST1H3H;HIST1H3J	histone cluster 1, H3i	0.95	0.9	0.91	0.68
C5	C5;CPAMD4;FLJ17816;FLJ17822;MGC142298	complement component 5	0.94	0.9	0.91	0.64
MTMR8	MTMR8;FLJ20126;FLJ60798	myotubularin related protein 8	0.94	0.86	0.91	0.67
GREM2	GREM2;CKTSF1B2;DAND3;PRDC	gremlin 2	0.94	0.81	0.91	0.69
ZNF253	ZNF253;BMZF-1;BMZF1;FLJ90391;ZNF411	zinc finger protein 253	0.94	0.9	0.91	0.73
CTSS	CTSS;FLJ50259;MGC3886	cathepsin S	0.94	0.79	0.91	0.59
COQ4	COQ4;CGI-92	coenzyme Q4 homolog (S. cerevisiae)	0.94	0.82	0.91	0.59
CYP20A1	CYP20A1;CYP-M;MGC22229	cytochrome P450, family 20, subfamily A, polypeptide 1	0.94	0.9	0.91	0.59
SYCN	SYCN;FLJ27441;INSSA1;SYL	syncollin	0.93	0.76	0.91	0.63
ARMC8	ARMC8;HSPC056;MGC10058;MGC4880	armadillo repeat containing 8	0.93	0.9	0.91	0.63
PRSS21	PRSS21;ESP-1;ESP1;TEST1;TESTISIN	protease, serine, 21 (testisin)	0.92	0.82	0.91	0.62
39326	SEPT7;CDC10;CDC3;DKFZp686M10191;NBLA02942;SEPT7A	septin 7	0.92	0.78	0.91	0.69
TECR	TECR;GPSN2;MRT14;SC2;TER	trans-2,3-enoyl-CoA reductase	0.92	0.86	0.91	0.68
NCKAP5	NCKAP5;ERIH1;ERIH2;FLJ34870;NAP5	NCK-associated protein 5	0.92	0.8	0.91	0.61
PLEKHG4B	PLEKHG4B;FLJ43173;KIAA1909	pleckstrin homology domain containing, family G (with RhoGef domain) member 4B	0.91	0.82	0.91	0.68
UBR5	UBR5;DD5;EDD;EDD1;FLJ11310;HYD;KIAA0896;MGC57263	ubiquitin protein ligase E3 component n-recognin 5	0.91	0.76	0.91	0.57
CECR1	CECR1;ADA2;ADGF;IDGFL	cat eye syndrome chromosome region, candidate 1	0.9	0.8	0.91	0.59
KDR	KDR;CD309;FLK1;VEGFR;VEGFR2	kinase insert domain receptor (a type III receptor tyrosine kinase)	0.9	0.88	0.91	0.63
HFE2	HFE2;HFE2A;HJV;JH;MGC23953;RGMC	hemochromatosis type 2 (juvenile)	0.9	0.86	0.91	0.69
C18orf1	C18orf1	chromosome 18 open reading frame 1	0.9	0.8	0.91	0.6
BFSP2	BFSP2;CP47;CP49;LIFL-L;MGC142078;MGC142080;PHAKOSIN	beaded filament structural protein 2, phakinin	0.9	0.9	0.91	0.66
MCART1	MCART1;CG7943;FLJ37273;MGC14836	mitochondrial carrier triple repeat 1	0.9	0.8	0.91	0.6
PSTK	PSTK;C10orf89;MGC35392	phosphoseryl-tRNA kinase	0.9	0.78	0.91	0.71
P2RY1	P2RY1;P2Y1	purinergic receptor P2Y, G-protein coupled, 1	0.9	0.8	0.91	0.71
ELAC1	ELAC1;D29;FLJ59261	elaC homolog 1 (E. coli)	0.9	0.86	0.91	0.57
LCK	LCK;LSK;YT16;p56lck;pp58lck	lymphocyte-specific protein tyrosine kinase	0.9	0.82	0.91	0.68
SEC22C	SEC22C;DKFZp761F2321;MGC13261;MGC5373;SEC22L3	SEC22 vesicle trafficking protein homolog C (S. cerevisiae)	0.9	0.82	0.91	0.59
BRD3	BRD3;FLJ23227;FLJ41328;KIAA0043;ORFX;RING3L	bromodomain containing 3	0.9	0.82	0.91	0.68
EXT1	EXT1;EXT;LGCR;LGS;TRPS2;TTV	exostosin 1	0.9	0.78	0.91	0.58
ZW10	ZW10;HZW10;KNTC1AP;MGC149821	ZW10, kinetochore associated, homolog (Drosophila)	0.9	0.78	0.91	0.65
MAP4K5	MAP4K5;GCKR;KHS;KHS1;MAPKKKK5	mitogen-activated protein kinase kinase kinase kinase 5	0.9	0.82	0.91	0.68
MZT2B	MZT2B;FAM128B;FLJ14346;MGC87017;MOZART2B	mitotic spindle organizing protein 2B	0.9	0.73	0.91	0.57
PPPDE1	PPPDE1;C1orf121;FAM152A;FLJ21998;PNAS-4	PPPDE peptidase domain containing 1	0.9	0.89	0.91	0.59
ANXA8	ANXA8;ANX8;FLJ53095	annexin A8	0.89	0.84	0.91	0.63
SLC10A3	SLC10A3;DXS253E;P3	solute carrier family 10 (sodium/bile acid cotransporter family), member 3	0.89	0.8	0.91	0.66
H3F3B	H3F3B;H3.3B;H3F3A	H3 histone, family 3B (H3.3B)	0.89	0.86	0.91	0.6
FAM204A	FAM204A;C10orf84;FLJ13188;bA319I23.1	family with sequence similarity 204, member A	0.89	0.72	0.91	0.61
CCDC96	CCDC96;FLJ90575;MGC138479	coiled-coil domain containing 96	0.88	0.83	0.91	0.61
MAP3K3	MAP3K3;MAPKKK3;MEKK3	mitogen-activated protein kinase kinase kinase 3	0.88	0.86	0.91	0.61
IFT20	IFT20	intraflagellar transport 20 homolog (Chlamydomonas)	0.88	0.86	0.91	0.66
PDE8A	PDE8A;FLJ16150;HsT19550	phosphodiesterase 8A	0.88	0.77	0.91	0.56
METTL10	METTL10;C10orf138;Em:AC068896.3;FLJ13019	methyltransferase like 10	0.88	0.78	0.91	0.61
LRIG3	LRIG3;FLJ26573;FLJ90440;KIAA3016;LIG3	leucine-rich repeats and immunoglobulin-like domains 3	0.88	0.84	0.91	0.62
NAPG	NAPG;GAMMASNAP	N-ethylmaleimide-sensitive factor attachment protein, gamma	0.88	0.82	0.91	0.62
AGAP8	AGAP8;CTGLF5	ArfGAP with GTPase domain, ankyrin repeat and PH domain 8	0.88	0.77	0.91	0.62
ODF3B	ODF3B;ODF3L3	outer dense fiber of sperm tails 3B	0.88	0.72	0.91	0.6
PTCHD1	PTCHD1;FLJ30296;MGC149798	patched domain containing 1	0.87	0.72	0.91	0.6
CCL21	CCL21;6Ckine;CKb9;ECL;MGC34555;SCYA21;SLC;TCA4	chemokine (C-C motif) ligand 21	0.87	0.82	0.91	0.62
ZFR2	ZFR2;KIAA1086	zinc finger RNA binding protein 2	0.87	0.8	0.91	0.54
C3orf35	C3orf35;APRG1	chromosome 3 open reading frame 35	0.86	0.82	0.91	0.58
STARD5	STARD5;MGC10327	StAR-related lipid transfer (START) domain containing 5	0.86	0.7	0.91	0.59
RTBDN	RTBDN;FLJ36353	retbindin	0.86	0.8	0.91	0.55
RNMTL1	RNMTL1;FLJ10581	RNA methyltransferase like 1	0.86	0.74	0.91	0.54
CYP4X1	CYP4X1;CYPIVX1;MGC40051	cytochrome P450, family 4, subfamily X, polypeptide 1	0.86	0.85	0.91	0.63
DDR1	DDR1;CAK;CD167;DDR;EDDR1;HGK2;MCK10;NEP;NTRK4;PTK3;PTK3A;RTK6;TRKE	discoidin domain receptor tyrosine kinase 1	0.86	0.84	0.91	0.6
PAFAH1B3	PAFAH1B3;FLJ44990;PAFAHG	platelet-activating factor acetylhydrolase 1b, catalytic subunit 3 (29kDa)	0.86	0.83	0.91	0.55
PPP1R7	PPP1R7;SDS22	protein phosphatase 1, regulatory subunit 7	0.86	0.73	0.91	0.55
MASTL	MASTL;FLJ14813;GREATWALL;GW;GWL;MAST-L;RP11-85G18.2;THC2;hGWL	microtubule associated serine/threonine kinase-like	0.86	0.78	0.91	0.64
ZNF84	ZNF84;HPF2	zinc finger protein 84	0.85	0.76	0.91	0.63
PRKRA	PRKRA;DYT16;PACT;RAX	protein kinase, interferon-inducible double stranded RNA dependent activator	0.85	0.78	0.91	0.53
PRKCI	PRKCI;DXS1179E;MGC26534;PKCI;nPKC-iota	protein kinase C, iota	0.85	0.84	0.91	0.6
TRIM7	TRIM7;GNIP;RNF90	tripartite motif containing 7	1.35	1.19	0.92	0.86
CLCA3P	CLCA3P;CLCA3	chloride channel accessory 3, pseudogene	1.3	1.2	0.92	0.93
STARD13	STARD13;ARHGAP37;DLC2;FLJ37385;GT650	StAR-related lipid transfer (START) domain containing 13	1.29	1.16	0.92	0.87
OR9G9	OR9G9	olfactory receptor, family 9, subfamily G, member 9	1.29	1.15	0.92	0.88
TNFAIP3	TNFAIP3;A20;MGC104522;MGC138687;MGC138688;OTUD7C;TNFA1P2	tumor necrosis factor, alpha-induced protein 3	1.23	1.07	0.92	0.78
PPIF	PPIF;CYP3;Cyp-D;FLJ90798;MGC117207	peptidylprolyl isomerase F	1.23	1.09	0.92	0.79
FLJ90834	FLJ90834	ENTREZ STATUS: REMAPPED TO GENE ID: 100505621	1.21	1.09	0.92	0.82
MTHFD2L	MTHFD2L;FLJ13105;MGC45532;MGC72244	methylenetetrahydrofolate dehydrogenase (NADP+ dependent) 2-like	1.2	1.14	0.92	0.86
GSTM5	GSTM5;GSTM5-5;GTM5	glutathione S-transferase mu 5	1.19	1.06	0.92	0.83
HERC2	HERC2;D15F37S1;DKFZp547P028;KIAA0393;SHEP1;jdf2;p528	hect domain and RLD 2	1.19	1.01	0.92	0.76
PRDX3	PRDX3;AOP-1;AOP1;HBC189;MER5;MGC104387;MGC24293;PRO1748;SP-22;prx-III	peroxiredoxin 3	1.19	1.1	0.92	0.74
TRIM36	TRIM36;HAPRIN;RBCC728;RNF98	tripartite motif containing 36	1.18	1.06	0.92	0.96
GAP43	GAP43;B-50;PP46	growth associated protein 43	1.17	1.11	0.92	0.84
GSDMA	GSDMA;FLJ39120;GSDM;GSDM1;MGC129596	gasdermin A	1.15	1.08	0.92	0.81
STEAP3	STEAP3;STMP3;TSAP6;dudlin-2	STEAP family member 3, metalloreductase	1.15	1.06	0.92	0.71
OIT3	OIT3;FLJ39116;LZP	oncoprotein induced transcript 3	1.14	1	0.92	0.72
ZACN	ZACN;L2;LGICZ;LGICZ1;MGC129841;ZAC;ZAC1	zinc activated ligand-gated ion channel	1.14	1.04	0.92	0.81
MRPL11	MRPL11;L11MT;MGC111024;MRP-L11	mitochondrial ribosomal protein L11	1.14	1	0.92	0.77
TPRA1	TPRA1;FLJ32197;GPR175;TMEM227;TPRA40	transmembrane protein, adipocyte asscociated 1	1.14	1	0.92	0.9
CCT8L1P	CCT8L1P;CCT8L1	chaperonin containing TCP1, subunit 8 (theta)-like 1, pseudogene	1.14	1.02	0.92	0.78
SLC8A2	SLC8A2;NCX2	solute carrier family 8 (sodium/calcium exchanger), member 2	1.12	0.99	0.92	0.73
CCDC43	CCDC43;FLJ31795	coiled-coil domain containing 43	1.12	0.97	0.92	0.79
VEZF1	VEZF1;DB1;ZNF161	vascular endothelial zinc finger 1	1.12	0.97	0.92	0.77
C7orf70	C7orf70;MGC12966;SIPAR	chromosome 7 open reading frame 70	1.12	1.04	0.92	0.76
BTRC	BTRC;BETA-TRCP;FBW1A;FBXW1;FBXW1A;FWD1;MGC4643;bTrCP;bTrCP1;betaTrCP	beta-transducin repeat containing	1.11	0.98	0.92	0.7
ZNF254	ZNF254;BMZF-5;FLJ58216;HD-ZNF1;ZNF539;ZNF91L	zinc finger protein 254	1.11	0.99	0.92	0.8
DOCK10	DOCK10;DKFZp781A1532;DRIP2;Nbla10300;ZIZ3	dedicator of cytokinesis 10	1.1	0.99	0.92	0.75
SNX15	SNX15;HSAF001435	sorting nexin 15	1.1	1.06	0.92	0.71
C7orf30	C7orf30	chromosome 7 open reading frame 30	1.1	0.98	0.92	0.74
NOA1	NOA1;C4orf14;MGC3232;hAtNOS1;hNOA1;mAtNOS1	nitric oxide associated 1	1.1	1.05	0.92	0.8
CD38	CD38;T10	CD38 molecule	1.09	1.06	0.92	0.7
PAK6	PAK6;PAK5	p21 protein (Cdc42/Rac)-activated kinase 6	1.09	1	0.92	0.82
WNT6	WNT6	wingless-type MMTV integration site family, member 6	1.09	1.06	0.92	0.74
ANO9	ANO9;PIG5;TMEM16J;TP53I5	anoctamin 9	1.09	1.07	0.92	0.75
TBC1D7	TBC1D7;DKFZp686N2317;FLJ32666;PIG51;TBC7;dJ257A7.3	TBC1 domain family, member 7	1.09	0.99	0.92	0.82
MACC1	MACC1;7A5;SH3BP4L	metastasis associated in colon cancer 1	1.09	0.98	0.92	0.77
FOXP3	FOXP3;AIID;DIETER;IPEX;MGC141961;MGC141963;PIDX;XPID	forkhead box P3	1.08	0.94	0.92	0.74
C4orf43	C4orf43;FLJ11184;FLJ52950	chromosome 4 open reading frame 43	1.08	1.04	0.92	0.7
CACNG8	CACNG8;FLJ16240;FLJ30598	calcium channel, voltage-dependent, gamma subunit 8	1.08	1.04	0.92	0.81
RIMKLA	RIMKLA;FAM80A;MGC47816;NAAGS;NAAGS-II	ribosomal modification protein rimK-like family member A	1.08	1.02	0.92	0.76
ADAMTSL1	ADAMTSL1;ADAMTSL-1;ADAMTSR1;C9orf94;DKFZp686L03130;FLJ35283;FLJ41032;FLJ46891;MGC118803;MGC118805;MGC40193;PUNCTIN	ADAMTS-like 1	1.06	0.97	0.92	0.72
MYL3	MYL3;CMH8;MLC1SB;MLC1V;VLC1	myosin, light chain 3, alkali; ventricular, skeletal, slow	1.06	1.05	0.92	0.79
DNAJB4	DNAJB4;DNAJW;DjB4;HLJ1	DnaJ (Hsp40) homolog, subfamily B, member 4	1.06	0.93	0.92	0.71
NT5C2	NT5C2;GMP;NT5B;PNT5;cN-II	5'-nucleotidase, cytosolic II	1.06	0.98	0.92	0.77
EYA3	EYA3;DKFZp686C132	eyes absent homolog 3 (Drosophila)	1.05	0.97	0.92	0.75
GABPB1	GABPB1;BABPB2;E4TF1;E4TF1-47;E4TF1-53;E4TF1B;GABPB;GABPB2;NRF2B1;NRF2B2	GA binding protein transcription factor, beta subunit 1	1.05	0.98	0.92	0.65
SLC8A3	SLC8A3;NCX3	solute carrier family 8 (sodium/calcium exchanger), member 3	1.04	0.92	0.92	0.72
OR2V2	OR2V2;OR2V3;OST713	olfactory receptor, family 2, subfamily V, member 2	1.04	0.98	0.92	0.7
C2orf78	C2orf78;COG5373;FLJ43987;hCG1989538	chromosome 2 open reading frame 78	1.04	1	0.92	0.72
SLC38A4	SLC38A4;ATA3;FLJ10191;MGC126876;NAT3;PAAT	solute carrier family 38, member 4	1.04	0.98	0.92	0.78
WDR5B	WDR5B;FLJ11287;MGC49879	WD repeat domain 5B	1.04	0.93	0.92	0.7
CEP120	CEP120;CCDC100;DKFZp686I06246;FLJ36090;FLJ38327	centrosomal protein 120kDa	1.04	0.9	0.92	0.73
GAS7	GAS7;KIAA0394;MGC1348;MLL/GAS7	growth arrest-specific 7	1.03	0.9	0.92	0.67
TIMP3	TIMP3;HSMRK222;K222;K222TA2;SFD	TIMP metallopeptidase inhibitor 3	1.03	0.92	0.92	0.68
LCN12	LCN12;MGC34753;MGC48935	lipocalin 12	1.02	0.91	0.92	0.67
MAPK12	MAPK12;ERK3;ERK6;P38GAMMA;PRKM12;SAPK-3;SAPK3	mitogen-activated protein kinase 12	1.02	0.91	0.92	0.82
SIPA1	SIPA1;MGC102688;MGC17037;SPA1	signal-induced proliferation-associated 1	1.02	0.9	0.92	0.75
NBN	NBN;AT-V1;AT-V2;ATV;FLJ10155;MGC87362;NBS;NBS1;P95	nibrin	1.02	1.04	0.92	0.72
EIF2AK1	EIF2AK1;HCR;HRI;KIAA1369	eukaryotic translation initiation factor 2-alpha kinase 1	1.02	1	0.92	0.71
EPHA8	EPHA8;EEK;EK3;HEK3;KIAA1459	EPH receptor A8	1.01	0.94	0.92	0.77
ABCD2	ABCD2;ABC39;ALDL1;ALDR;ALDRP;hALDR	ATP-binding cassette, sub-family D (ALD), member 2	1	0.84	0.92	0.67
ARHGAP25	ARHGAP25;KAIA0053	Rho GTPase activating protein 25	1	0.88	0.92	0.7

IL1RN	IL1RN;DIRA;ICIL-1RA;IL-1RN;IL-1ra;IL-1ra3;IL1F3;IL1RA;IRAP;MGC10430;MVCD4	interleukin 1 receptor antagonist	1	0.9	0.92	0.64
RGS9	RGS9;MGC111763;MGC26458;PERRS;RGS9L	regulator of G-protein signaling 9	1	0.98	0.92	0.75
SAMD14	SAMD14;FLJ36890	sterile alpha motif domain containing 14	1	0.88	0.92	0.67
ACR	ACR	acrosin	1	0.92	0.92	0.69
EHBP1	EHBP1;HPC12;KIAA0903;NACSIN	EH domain binding protein 1	1	0.84	0.92	0.65
DOCK1	DOCK1;DOCK180;ced5	dedicator of cytokinesis 1	1	0.91	0.92	0.75
SMEK2	SMEK2;FLFL2;FLJ31474;KIAA1387;PP4R3B;PSY2;smk1	SMEK homolog 2, suppressor of mek1 (Dictyostelium)	1	0.87	0.92	0.7
GBE1	GBE1;GBE	glucan (1,4-alpha-), branching enzyme 1	1	0.84	0.92	0.75
FMO5	FMO5	flavin containing monooxygenase 5	0.99	0.89	0.92	0.66
C17orf66	C17orf66;FLJ32830	chromosome 17 open reading frame 66	0.99	0.9	0.92	0.65
GPR119	GPR119;GPCR2;MGC119957	G protein-coupled receptor 119	0.99	0.84	0.92	0.63
MCCD1	MCCD1	mitochondrial coiled-coil domain 1	0.99	0.97	0.92	0.7
HIST1H2BN	HIST1H2BN;H2B/d;H2BFD;HIST1H3I;MGC125414;MGC125415;MGC125416;MGC9388	histone cluster 1, H2bn	0.99	0.87	0.92	0.67
SNX14	SNX14;MGC13217;RGS-PX2	sorting nexin 14	0.99	0.93	0.92	0.7
NXN	NXN;FLJ12614;NRX;TRG-4	nucleoredoxin	0.99	0.88	0.92	0.65
ACE2	ACE2;ACEH;DKFZp434A014	angiotensin I converting enzyme (peptidyl-dipeptidase A) 2	0.98	0.9	0.92	0.67
GPR97	GPR97;PB99;PGR26	G protein-coupled receptor 97	0.98	0.87	0.92	0.78
IL11	IL11;AGIF;IL-11	interleukin 11	0.98	0.92	0.92	0.72
CRELD1	CRELD1;AVSD2;CIRRIN;DKFZp566D213	cysteine-rich with EGF-like domains 1	0.98	0.86	0.92	0.72
SCMH1	SCMH1;Scml3	sex comb on midleg homolog 1 (Drosophila)	0.98	0.96	0.92	0.72
PANK2	PANK2;C20orf48;FLJ17232;HARP;HSS;MGC15053;NBIA1;PKAN	pantothenate kinase 2	0.98	0.99	0.92	0.7
VPS53	VPS53;FLJ10979;FLJ41112;FLJ61757;HCCS1;MGC39512;hVps53L;pp13624	vacuolar protein sorting 53 homolog (S. cerevisiae)	0.98	0.94	0.92	0.68
ISOC1	ISOC1	isochorismatase domain containing 1	0.98	0.97	0.92	0.73
CDH1	CDH1;Arc-1;CD324;CDHE;ECAD;LCAM;UVO	cadherin 1, type 1, E-cadherin (epithelial)	0.98	0.94	0.92	0.66
OPRL1	OPRL1;KOR-3;MGC34578;NOCIR;OOR;ORL1	opiate receptor-like 1	0.97	0.88	0.92	0.78
KLHL26	KLHL26;FLJ11078	kelch-like 26 (Drosophila)	0.97	0.86	0.92	0.65
FANCL	FANCL;FAAP43;FLJ10335;PHF9;POG	Fanconi anemia, complementation group L	0.97	0.91	0.92	0.66
C8orf55	C8orf55;DSCD75	chromosome 8 open reading frame 55	0.97	0.94	0.92	0.67
THYN1	THYN1;MDS012;MGC12187;MY105;THY28;THY28KD	thymocyte nuclear protein 1	0.97	0.86	0.92	0.65
DNAJC14	DNAJC14;DNAJ;DRIP78;HDJ3;LIP6	DnaJ (Hsp40) homolog, subfamily C, member 14	0.97	0.88	0.92	0.68
ABCF2	ABCF2;ABC28;DKFZp586K1823;EST133090;HUSSY18;M-ABC1	ATP-binding cassette, sub-family F (GCN20), member 2	0.97	0.87	0.92	0.68
SLC13A3	SLC13A3;NADC3;SDCT2	solute carrier family 13 (sodium-dependent dicarboxylate transporter), member 3	0.96	0.82	0.92	0.72
KLHDC7B	KLHDC7B;MGC16635	kelch domain containing 7B	0.96	0.94	0.92	0.72
HES3	HES3;bHLHb43	hairy and enhancer of split 3 (Drosophila)	0.96	0.9	0.92	0.62
RGNEF	RGNEF;DKFZp686P12164;FLJ21817;KIAA1998;RIP2;p190RHOGEF	190 kDa guanine nucleotide exchange factor	0.96	0.88	0.92	0.6
DZIP1	DZIP1;DZIP;DZIPt1;KIAA0996;RP11-23E3.3	DAZ interacting protein 1	0.96	0.92	0.92	0.63
CYHR1	CYHR1;CHRP;KIAA0496;MGC13010	cysteine/histidine-rich 1	0.96	0.95	0.92	0.63
AIDA	AIDA;C1orf80;FLJ12806;FLJ32421;RP11-378J18.7	axin interactor, dorsalization associated	0.96	0.96	0.92	0.64
FADS1	FADS1;D5D;FADS6;FADSD5;FLJ38956;FLJ90273;LLCDL1;TU12	fatty acid desaturase 1	0.96	0.82	0.92	0.65
C15orf23	C15orf23;FLJ14502;MGC141728;MGC141729;SKAP;TRAF4AF1	chromosome 15 open reading frame 23	0.96	0.88	0.92	0.63
PLEKHB2	PLEKHB2;EVT2;FLJ20783;FLJ23679;FLJ30436	pleckstrin homology domain containing, family B (evectins) member 2	0.96	0.92	0.92	0.74
UGT1A10	UGT1A10;UDPGT;UGT-1J;UGT1-10;UGT1.10;UGT1J	UDP glucuronosyltransferase 1 family, polypeptide A10	0.96	0.88	0.92	0.65
MAGEA11	MAGEA11;CT1.11;MAGE-11;MAGE11;MAGEA-11;MGC10511	melanoma antigen family A, 11	0.95	0.85	0.92	0.67
SCGB3A2	SCGB3A2;LU103;PNSP1;UGRP1;pnSP-1	secretoglobin, family 3A, member 2	0.95	0.92	0.92	0.65
CTBP2	CTBP2	C-terminal binding protein 2	0.95	0.84	0.92	0.62
OR8S1	OR8S1	olfactory receptor, family 8, subfamily S, member 1	0.94	0.83	0.92	0.65
CARD6	CARD6;CINCIN1	caspase recruitment domain family, member 6	0.94	0.89	0.92	0.64
SGK2	SGK2;H-SGK2;dJ138B7.2	serum/glucocorticoid regulated kinase 2	0.94	0.86	0.92	0.72
TTC35	TTC35;KIAA0103	tetratricopeptide repeat domain 35	0.94	0.78	0.92	0.61
GAK	GAK;DNAJ26;DNAJC26;FLJ16629;FLJ40395;MGC99654	cyclin G associated kinase	0.94	0.92	0.92	0.65
STK24	STK24;MST3;MST3B;STE20;STK3	serine/threonine kinase 24	0.94	0.86	0.92	0.71
RBL1	RBL1;CP107;MGC40006;PRB1;p107	retinoblastoma-like 1 (p107)	0.94	0.85	0.92	0.66
GCN1L1	GCN1L1;GCN1;GCN1L;KIAA0219;PRIC295	GCN1 general control of amino-acid synthesis 1-like 1 (yeast)	0.94	0.86	0.92	0.63
OR52W1	OR52W1;OR11-71;OR52W1P	olfactory receptor, family 52, subfamily W, member 1	0.93	0.86	0.92	0.58
ACADS	ACADS;ACAD3;SCAD	acyl-CoA dehydrogenase, C-2 to C-3 short chain	0.93	0.82	0.92	0.59
IL7	IL7;IL-7	interleukin 7	0.92	0.82	0.92	0.7
DFFB	DFFB;CAD;CPAN;DFF-40;DFF2;DFF40	DNA fragmentation factor, 40kDa, beta polypeptide (caspase-activated DNase)	0.92	0.86	0.92	0.67
ASMT	ASMT;ASMTY;HIOMT;HIOMTY	acetylserotonin O-methyltransferase	0.92	0.81	0.92	0.59
PRICKLE2	PRICKLE2;DKFZp686D143;DKFZp686H1748;DKFZp686M031;EPM5	prickle homolog 2 (Drosophila)	0.92	0.84	0.92	0.75
FUNDC2	FUNDC2;DC44;FLJ33773;HCBP6;HCC3;MGC131676;MGC2495;PD03104	FUN14 domain containing 2	0.92	0.92	0.92	0.7
TMUB2	TMUB2;MGC3123	transmembrane and ubiquitin-like domain containing 2	0.92	0.92	0.92	0.62
PCYOX1L	PCYOX1L;MGC3265	prenylcysteine oxidase 1 like	0.92	0.88	0.92	0.63
C14orf119	C14orf119;FLJ20671;FLJ21141;MGC74723	chromosome 14 open reading frame 119	0.92	0.82	0.92	0.62
PPIP5K1	PPIP5K1;DKFZp313L0221;HISPPD2A;IP6K;IPS1;KIAA0377;MGC51871;VIP1;hsVIP1	diphosphoinositol pentakisphosphate kinase 1	0.92	0.78	0.92	0.59
LAMB4	LAMB4	laminin, beta 4	0.91	0.88	0.92	0.62
TM4SF5	TM4SF5	transmembrane 4 L six family member 5	0.91	0.82	0.92	0.61
WBSCR27	WBSCR27;MGC40131	Williams Beuren syndrome chromosome region 27	0.91	0.9	0.92	0.59
ZNF485	ZNF485	zinc finger protein 485	0.91	0.8	0.92	0.61
GSK3A	GSK3A;DKFZp686D0638	glycogen synthase kinase 3 alpha	0.91	0.84	0.92	0.69
JTB	JTB;HJTB;HSPC222;PAR;hJT	jumping translocation breakpoint	0.91	0.88	0.92	0.6
PCDH7	PCDH7;BH-Pcdh;BHPCDH;FLJ97161	protocadherin 7	0.9	0.87	0.92	0.59
NLRX1	NLRX1;CLR11.3;DLNB26;FLJ21478;MGC131937;MGC21025;NOD26;NOD5;NOD9	NLR family member X1	0.9	0.88	0.92	0.62
HSF2	HSF2;HSF 2;HSTF 2;MGC117376;MGC156196;MGC75048	heat shock transcription factor 2	0.9	0.86	0.92	0.67
DNAJC18	DNAJC18;MGC29463	DnaJ (Hsp40) homolog, subfamily C, member 18	0.9	0.9	0.92	0.67
IKBKAP	IKBKAP;DKFZp781H1425;DYS;ELP1;FD;FLJ12497;IKAP;IKI3;TOT1	inhibitor of kappa light polypeptide gene enhancer in B-cells, kinase complex-associated protein	0.9	0.9	0.92	0.64
POLR1C	POLR1C;RPA39;RPA40;RPA5;RPAC1;TCS3	polymerase (RNA) I polypeptide C, 30kDa	0.9	0.8	0.92	0.6
ACO1	ACO1;ACONS;IREB1;IREBP;IREBP1;IRP1	aconitase 1, soluble	0.9	0.82	0.92	0.61
TJP2	TJP2;MGC26306;X104;ZO2	tight junction protein 2 (zona occludens 2)	0.9	0.9	0.92	0.64
NCOA3	NCOA3;ACTR;AIB-1;AIB1;CAGH16;CTG26;KAT13B;MGC141848;RAC3;SRC-3;SRC3;TNRC14;TNRC16;TRAM-1;bHLHe42;pCIP	nuclear receptor coactivator 3	0.9	0.88	0.92	0.58
CA13	CA13;CAXIII;FLJ36434;FLJ37995;MGC59868	carbonic anhydrase XIII	0.89	0.8	0.92	0.62
NRBP2	NRBP2;DKFZp434P086;MGC138699;TRG16;pp9320	nuclear receptor binding protein 2	0.89	0.88	0.92	0.63
C9orf47	C9orf47;C9orf108;FLJ37523;bA791O21.3	chromosome 9 open reading frame 47	0.89	0.86	0.92	0.7
SPINK14	SPINK14;SPINK5L2	serine peptidase inhibitor, Kazal type 14 (putative)	0.89	0.84	0.92	0.64
MRAP	MRAP;B27;C21orf61;FALP;FGD2;GCCD2	melanocortin 2 receptor accessory protein	0.88	0.85	0.92	0.63
NID1	NID1;NID	nidogen 1	0.88	0.88	0.92	0.68
MAP1A	MAP1A;FLJ77111;MAP1L;MTAP1A	microtubule-associated protein 1A	0.88	0.84	0.92	0.63
TCEAL2	TCEAL2;MY0876G05;my048	transcription elongation factor A (SII)-like 2	0.88	0.88	0.92	0.59
FUT11	FUT11;MGC119338;MGC119339;MGC33202	fucosyltransferase 11 (alpha (1,3) fucosyltransferase)	0.88	0.84	0.92	0.57
ZNF689	ZNF689;DKFZp762C173;FLJ90415;TIPUH1	zinc finger protein 689	0.88	0.8	0.92	0.6
C1orf31	C1orf31	chromosome 1 open reading frame 31	0.88	0.86	0.92	0.61
PHTF2	PHTF2;DKFZp564F013;FLJ33324;MGC86999	putative homeodomain transcription factor 2	0.88	0.89	0.92	0.65
LACTB2	LACTB2	lactamase, beta 2	0.88	0.82	0.92	0.65
RBBP5	RBBP5;RBQ3;SWD1	retinoblastoma binding protein 5	0.88	0.83	0.92	0.57
ERVFRD-1	ERVFRD-1;ERVFRDE1;FLJ41944;FLJ90611;GLLL6191;HERV-FRD;HERV-W/FRD;MGC87585;UNQ6191;envFRD	endogenous retrovirus group FRD, member 1	0.88	0.81	0.92	0.56
KIR3DX1	KIR3DX1;FLJ00060;KIR3DL0;LENG12	killer cell immunoglobulin-like receptor, three domains, X1	0.87	0.8	0.92	0.58
ATP8A1	ATP8A1;ATPASEII;ATPIA;ATPP2;MGC130042;MGC130043;MGC26327	ATPase, aminophospholipid transporter (APLT), class I, type 8A, member 1	0.87	0.8	0.92	0.63
TAS2R41	TAS2R41;MGC119928;MGC119929;T2R41;T2R59	taste receptor, type 2, member 41	0.87	0.88	0.92	0.65
C18orf45	C18orf45;FLJ44259;MGC11386;MGC138577	chromosome 18 open reading frame 45	0.87	0.79	0.92	0.57
TSPY1	TSPY1;CT78;DYS14;TSPY;pJA923	testis specific protein, Y-linked 1	0.86	0.72	0.92	0.57
SYN1	SYN1;SYN1a;SYN1b;SYNI	synapsin I	0.86	0.82	0.92	0.64
GLTPD2	GLTPD2	glycolipid transfer protein domain containing 2	0.86	0.84	0.92	0.68
ARL6IP6	ARL6IP6;AIP-6;MGC33864;PFAAP1	ADP-ribosylation-like factor 6 interacting protein 6	0.86	0.8	0.92	0.57
CTF1	CTF1;CT-1;CT1	cardiotrophin 1	0.86	0.82	0.92	0.64
FAM129A	FAM129A;C1orf24;FLJ38228;NIBAN	family with sequence similarity 129, member A	0.86	0.84	0.92	0.59
INS	INS;IDDM2;ILPR;IRDN;MODY10	insulin	0.86	0.82	0.92	0.56
FGF2	FGF2;BFGF;FGFB;HBGF-2	fibroblast growth factor 2 (basic)	0.85	0.78	0.92	0.57
TATDN2	TATDN2;KIAA0218;MGC126819;MGC126825	TatD DNase domain containing 2	1.4	1.34	0.93	1.06
RNF130	RNF130;G1RZFP;GOLIATH;GP;MGC117241;MGC138647;MGC99542	ring finger protein 130	1.38	1.19	0.93	0.89
SRPK3	SRPK3;MGC102944;MSSK-1;MSSK1;STK23	SRSF protein kinase 3	1.32	1.32	0.93	0.95
PGRMC2	PGRMC2;DG6;PMBP	progesterone receptor membrane component 2	1.3	1.19	0.93	0.83
ASB10	ASB10	ankyrin repeat and SOCS box containing 10	1.27	1.12	0.93	0.81
RAPSN	RAPSN;MGC3597;RAPSYN;RNF205	receptor-associated protein of the synapse	1.26	1.12	0.93	0.81
TAF11	TAF11;MGC:15243;TAF2I;TAFII28	TAF11 RNA polymerase II, TATA box binding protein (TBP)-associated factor, 28kDa	1.25	1.12	0.93	0.81
ZWILCH	ZWILCH;FLJ10036;FLJ16343;KNTC1AP;MGC111034;hZwilch	Zwilch, kinetochore associated, homolog (Drosophila)	1.23	1.15	0.93	0.78
MMD	MMD;MMA;PAQR11	monocyte to macrophage differentiation-associated	1.22	1.09	0.93	0.88
ZNF45	ZNF45;KOX5;ZNF13	zinc finger protein 45	1.21	1.15	0.93	0.9
ATXN3	ATXN3;AT3;ATX3;JOS;MJD;MJD1;SCA3	ataxin 3	1.19	1.12	0.93	0.75
EIF2A	EIF2A;EIF-2A;MST089;MSTP004;MSTP089	eukaryotic translation initiation factor 2A, 65kDa	1.19	1.16	0.93	0.77
LHX5	LHX5;MGC129689	LIM homeobox 5	1.18	1.05	0.93	0.76
BARD1	BARD1	BRCA1 associated RING domain 1	1.17	1.04	0.93	0.75
MAP3K8	MAP3K8;COT;EST;ESTF;FLJ10486;MEKK8;TPL2;Tpl-2;c-COT	mitogen-activated protein kinase kinase kinase 8	1.16	1.06	0.93	0.88
CEP192	CEP192;PPP1R62	centrosomal protein 192kDa	1.16	1.1	0.93	0.84
ASB8	ASB8;FLJ21255;MGC5540	ankyrin repeat and SOCS box containing 8	1.16	1.02	0.93	0.74
MYL2	MYL2;CMH10;DKFZp779C0562;MLC2	myosin, light chain 2, regulatory, cardiac, slow	1.15	1.1	0.93	0.74
RNF150	RNF150;MGC125502	ring finger protein 150	1.15	0.98	0.93	0.74
PRICKLE3	PRICKLE3;LMO6	prickle homolog 3 (Drosophila)	1.14	1.01	0.93	0.73
PICALM	PICALM;CALM;CLTH;LAP	phosphatidylinositol binding clathrin assembly protein	1.14	0.96	0.93	0.77
TRIM17	TRIM17;RBCC;RNF16;terf	tripartite motif containing 17	1.13	1	0.93	0.72
CKB	CKB;B-CK;CKBB	creatine kinase, brain	1.12	1.04	0.93	0.86

PRKCSH	PRKCSH;AGE-R2;G19P1;PCLD;PKCSH;PLD1	protein kinase C substrate 80K-H	1.12	1.1	0.93	0.8
SHOX	SHOX;GCFX;PHOG;SHOXY;SS	short stature homeobox	1.1	1.01	0.93	0.74
KIF5B	KIF5B;KINH;KNS;KNS1;UKHC	kinesin family member 5B	1.1	1.08	0.93	0.84
RASAL3	RASAL3;DKFZp667E013;FLJ00087;FLJ21438	RAS protein activator like 3	1.1	1.06	0.93	0.71
INPP5J	INPP5J;INPP5;MGC129984;PIB5PA;PIPP	inositol polyphosphate-5-phosphatase J	1.1	0.95	0.93	0.71
FOXA3	FOXA3;FKHH3;HNF3G;MGC10179;TCF3G	forkhead box A3	1.09	0.99	0.93	0.73
IRX3	IRX3;FLJ99187;IRX-1;IRXB1	iroquois homeobox 3	1.08	0.91	0.93	0.76
HIST2H4A	HIST2H4A;FO108;H4;H4/n;H4F2;H4FN;HIST1H4A;HIST1H4B;HIST1H4C;HIST1H4D;HIST1H4E;HIST1H4F;HIST1H4H;HIST1H4I;HIST1H4J;HIST1H4K;HIST1H4L;HIST2H4;HIST2H4B;HIST4H4	histone cluster 2, H4a	1.08	1	0.93	0.74
LIPA	LIPA;CESD;LAL	lipase A, lysosomal acid, cholesterol esterase	1.08	1	0.93	0.74
SLC38A2	SLC38A2;ATA2;KIAA1382;PRO1068;SAT2;SNAT2	solute carrier family 38, member 2	1.08	0.97	0.93	0.7
CELA3B	CELA3B;CBPP;E1;EL-1;ELA3B	chymotrypsin-like elastase family, member 3B	1.08	0.98	0.93	0.77
EPCAM	EPCAM;DIAR5;EGP-2;EGP314;EGP40;ESA;GA733-2;HNPCC8;KS1/4;KSA;M4S1;MIC18;MK-1;TACSTD1;TROP1	epithelial cell adhesion molecule	1.08	1.01	0.93	0.7
USP29	USP29;HOM-TES-84/86;MGC163266;MGC163270	ubiquitin specific peptidase 29	1.07	0.99	0.93	0.74
KBTBD5	KBTBD5;FLJ32015;MGC125350;SRYP	kelch repeat and BTB (POZ) domain containing 5	1.07	0.97	0.93	0.73
ANKRD24	ANKRD24;KIAA1981	ankyrin repeat domain 24	1.06	1.02	0.93	0.68
THOC5	THOC5;C22orf19;Fmip;PK1.3;fSAP79	THO complex 5	1.06	1.05	0.93	0.74
DEPTOR	DEPTOR;DEP.6;DEPDC6;DKFZp564B1778;FLJ12341;FLJ12428;FLJ13854	DEP domain containing MTOR-interacting protein	1.06	0.99	0.93	0.69
ADAMTS16	ADAMTS16;ADAMTS16s;FLJ16731	ADAM metallopeptidase with thrombospondin type 1 motif, 16	1.05	0.96	0.93	0.72
SPP1	SPP1;BNSP;BSPI;ETA-1;MGC110940;OPN	secreted phosphoprotein 1	1.05	0.92	0.93	0.78
NPS	NPS	neuropeptide S	1.05	0.88	0.93	0.73
CXorf57	CXorf57;FLJ10178;FLJ14191	chromosome X open reading frame 57	1.04	0.87	0.93	0.68
DNASE1L2	DNASE1L2;DNAS1L2	deoxyribonuclease I-like 2	1.04	0.95	0.93	0.67
GOLGA6L9	GOLGA6L9	golgin A6 family-like 9	1.04	0.95	0.93	0.79
ZBTB39	ZBTB39;KIAA0352;ZNF922	zinc finger and BTB domain containing 39	1.03	0.99	0.93	0.66
BPGM	BPGM;DPGM	2,3-bisphosphoglycerate mutase	1.03	0.94	0.93	0.74
PKN1	PKN1;DBK;MGC46204;PAK-1;PAK1;PKN;PKN-ALPHA;PRK1;PRKCL1	protein kinase N1	1.03	0.96	0.93	0.79
SLC26A2	SLC26A2;D5S1708;DTD;DTDST;EDM4;MST153;MSTP157	solute carrier family 26 (sulfate transporter), member 2	1.03	1.02	0.93	0.67
FAM154B	FAM154B;DKFZp666G057	family with sequence similarity 154, member B	1.02	1	0.93	0.72
SELS	SELS;ADO15;MGC104346;MGC2553;SBBI8;SEPS1;VIMP	selenoprotein S	1.02	0.9	0.93	0.76
ULK3	ULK3;DKFZp434C131;FLJ90566	unc-51-like kinase 3 (C. elegans)	1.02	1.02	0.93	0.72
MSI2	MSI2;FLJ36569;MGC3245;MSI2H	musashi homolog 2 (Drosophila)	1.02	0.96	0.93	0.65
GNAI1	GNAI1;Gi	guanine nucleotide binding protein (G protein), alpha inhibiting activity polypeptide 1	1.02	0.92	0.93	0.66
RAB6C	RAB6C;WTH3	RAB6C, member RAS oncogene family	1.02	0.92	0.93	0.68
PTEN	PTEN;10q23del;BZS;DEC;GLM2;MGC11227;MHAM;MMAC1;PTEN1;TEP1	phosphatase and tensin homolog	1.02	0.94	0.93	0.74
CRYBB2	CRYBB2;CCA2;CRYB2;CRYB2A;D22S665	crystallin, beta B2	1.01	0.94	0.93	0.64
PTPN20A	PTPN20A;CT126;MGC142033;PTPN20B;bA142I17.1;hPTPN20	protein tyrosine phosphatase, non-receptor type 20A	1.01	0.94	0.93	0.73
PAN3	PAN3	PAN3 poly(A) specific ribonuclease subunit homolog (S. cerevisiae)	1.01	0.9	0.93	0.69
ATRN	ATRN;DPPT-L;KIAA0548;MGC126754;MGCA	attractin	1.01	0.9	0.93	0.69
TFDP3	TFDP3;CT30;DP4;HCA661;MGC161639	transcription factor Dp family, member 3	1	0.96	0.93	0.72
UQCRB	UQCRB;FLJ92016;FLJ97033;QCR7;QP-C;QPC;UQBC;UQBP;UQCR6;UQPC	ubiquinol-cytochrome c reductase binding protein	1	0.9	0.93	0.7
DAPK2	DAPK2;DRP-1;DRP1;MGC119312	death-associated protein kinase 2	1	1	0.93	0.71
ZNF706	ZNF706;PNAS-106;PNAS-113	zinc finger protein 706	1	0.92	0.93	0.67
PRKRIP1	PRKRIP1;C114;FLJ13902;FLJ40957;KRBOX3	PRKR interacting protein 1 (IL11 inducible)	1	0.96	0.93	0.76
TMEM97	TMEM97;MAC30	transmembrane protein 97	1	0.97	0.93	0.72
DPM1	DPM1;CDGIE;MPDS	dolichyl-phosphate mannosyltransferase polypeptide 1, catalytic subunit	1	0.9	0.93	0.65
GRXCR2	GRXCR2	glutaredoxin, cysteine rich 2	1	0.97	0.93	0.78
GEMIN2	GEMIN2;SIP1;SIP1-delta	gem (nuclear organelle) associated protein 2	1	0.98	0.93	0.69
FCER2	FCER2;CD23;CD23A;CLEC4J;FCE2;IGEBF	Fc fragment of IgE, low affinity II, receptor for (CD23)	0.99	0.86	0.93	0.68
ZNF446	ZNF446;FLJ20626;ZKSCAN20;ZSCAN30	zinc finger protein 446	0.99	0.9	0.93	0.68
WDFY1	WDFY1;FENS-1;WDF1;ZFYVE17	WD repeat and FYVE domain containing 1	0.99	0.89	0.93	0.7
PRAMEF8	PRAMEF8	PRAME family member 8	0.99	0.94	0.93	0.68
KCNK2	KCNK2;K2p2.1;MGC126742;MGC126744;TPKC1;TREK;TREK-1;TREK1;hTREK-1c;hTREK-1e	potassium channel, subfamily K, member 2	0.98	0.94	0.93	0.73
CHRNB1	CHRNB1;ACHRB;CHRNB;CMS1D;CMS2A;FLJ57107;SCCMS	cholinergic receptor, nicotinic, beta 1 (muscle)	0.98	0.94	0.93	0.73
ADORA2A	ADORA2A;ADORA2;RDC8;hA2aR	adenosine A2a receptor	0.98	0.88	0.93	0.79
C8orf82	C8orf82;MGC70857	chromosome 8 open reading frame 82	0.98	0.92	0.93	0.64
SNX11	SNX11;MGC111019	sorting nexin 11	0.98	0.94	0.93	0.71
ABHD5	ABHD5;CDS;CGI58;IECN2;MGC8731;NCIE2	abhydrolase domain containing 5	0.98	0.9	0.93	0.68
ATXN2L	ATXN2L;A2D;A2LG;A2LP;A2RP	ataxin 2-like	0.98	0.86	0.93	0.73
CDC16	CDC16;ANAPC6;APC6;CUT9	cell division cycle 16 homolog (S. cerevisiae)	0.98	0.9	0.93	0.67
CDC42BPB	CDC42BPB;DKFZp686H1431;FLJ37601;FLJ44730;KIAA1124;MRCKB	CDC42 binding protein kinase beta (DMPK-like)	0.98	0.9	0.93	0.75
ZNF813	ZNF813;FLJ16542;FLJ34141	zinc finger protein 813	0.98	0.96	0.93	0.68
KCNJ5	KCNJ5;CIR;GIRK4;KATP1;KIR3.4;LQT13	potassium inwardly-rectifying channel, subfamily J, member 5	0.97	0.9	0.93	0.7
SSFA2	SSFA2;CS-1;CS1;DKFZp313O1039;DKFZp779G0129;FLJ45996;KIAA1927;KRAP;SPAG13	sperm specific antigen 2	0.97	0.87	0.93	0.71
FOXJ3	FOXJ3;MGC165036;MGC176686	forkhead box J3	0.97	0.88	0.93	0.62
FAM155A	FAM155A	family with sequence similarity 155, member A	0.97	0.92	0.93	0.7
PCDHGB4	PCDHGB4;CDH20;FIB2;MGC138293;MGC142167;PCDH-GAMMA-B4	protocadherin gamma subfamily B, 4	0.97	0.86	0.93	0.66
GNA14	GNA14	guanine nucleotide binding protein (G protein), alpha 14	0.96	0.99	0.93	0.68
C7orf52	C7orf52;DKFZp547K159;FLJ39237	chromosome 7 open reading frame 52	0.96	0.94	0.93	0.66
MAN2A2	MAN2A2;MANA2X	mannosidase, alpha, class 2A, member 2	0.96	0.88	0.93	0.61
ANKRD11	ANKRD11;ANCO-1;ANCO1;KBGS;LZ16;T13	ankyrin repeat domain 11	0.96	0.92	0.93	0.63
LTBP1	LTBP1;MGC163161	latent transforming growth factor beta binding protein 1	0.96	0.92	0.93	0.7
VPS33A	VPS33A;FLJ22395;FLJ23187	vacuolar protein sorting 33 homolog A (S. cerevisiae)	0.96	0.8	0.93	0.67
SCCPDH	SCCPDH;FLJ43187;NET11;RP11-439E19.2	saccharopine dehydrogenase (putative)	0.96	0.97	0.93	0.64
BMPER	BMPER;CRIM3;CV-2;CV2	BMP binding endothelial regulator	0.95	0.8	0.93	0.66
CCDC23	CCDC23	coiled-coil domain containing 23	0.95	0.79	0.93	0.62
KCTD4	KCTD4;bA321C24.3	potassium channel tetramerisation domain containing 4	0.94	0.93	0.93	0.72
GUCY1B3	GUCY1B3;GC-S-beta-1;GC-SB3;GUC1B3;GUCB3;GUCSB3;GUCY1B1	guanylate cyclase 1, soluble, beta 3	0.94	0.8	0.93	0.64
MGMT	MGMT	O-6-methylguanine-DNA methyltransferase	0.94	0.88	0.93	0.59
LATS2	LATS2;FLJ13161;KPM	LATS, large tumor suppressor, homolog 2 (Drosophila)	0.94	0.88	0.93	0.73
RNASEH1	RNASEH1;H1RNA;RNH1	ribonuclease H1	0.94	0.83	0.93	0.65
PHF19	PHF19;MGC131698;MGC149712;MGC149713;MGC23929;MTF2L1;PCL3	PHD finger protein 19	0.94	0.86	0.93	0.77
NUAK1	NUAK1;ARK5;KIAA0537	NUAK family, SNF1-like kinase, 1	0.94	0.95	0.93	0.67
TOM1L2	TOM1L2;FLJ21501;FLJ32746	target of myb1-like 2 (chicken)	0.94	0.88	0.93	0.6
CENPV	CENPV;3110013H01Rik;CENP-V;PRR6;p30	centromere protein V	0.94	0.77	0.93	0.61
KAZN	KAZN;DKFZp686D08115;FLJ10199;FLJ32593;FLJ43398;FLJ43806;KAZ;KIAA1026;MGC126686;MGC142181	kazrin, periplakin interacting protein	0.94	0.84	0.93	0.64
QRFPR	QRFPR;AQ27;GPR103;MGC149217;SP9155	pyroglutamylated RFamide peptide receptor	0.94	0.85	0.93	0.76
STX5	STX5;SED5;STX5A	syntaxin 5	0.93	0.92	0.93	0.65
PRR11	PRR11;FLJ11029	proline rich 11	0.93	0.92	0.93	0.65
ABCA4	ABCA4;ABC10;ABCR;ARMD2;CORD3;DKFZp781N1972;FFM;FLJ17534;RMP;RP19;STGD;STGD1	ATP-binding cassette, sub-family A (ABC1), member 4	0.92	0.84	0.93	0.62
MBD5	MBD5;FLJ11113;FLJ30517;KIAA1461;MRD1	methyl-CpG binding domain protein 5	0.92	0.92	0.93	0.68
FFAR1	FFAR1;FFA1R;GPCR40;GPR40	free fatty acid receptor 1	0.92	0.78	0.93	0.59
FAM65A	FAM65A;FLJ13725;KIAA1930	family with sequence similarity 65, member A	0.92	0.82	0.93	0.63
CCNDBP1	CCNDBP1;DIP1;GCIP;HHM	cyclin D-type binding-protein 1	0.92	0.9	0.93	0.67
MYO6	MYO6;DFNA22;DFNB37;KIAA0389	myosin VI	0.92	0.84	0.93	0.63
OSBPL9	OSBPL9;FLJ12492;FLJ14629;FLJ14801;FLJ32055;FLJ34384;MGC15035;ORP-9;ORP9	oxysterol binding protein-like 9	0.92	0.9	0.93	0.63
42248	42248	15 kDa selenoprotein	0.92	0.88	0.93	0.67
HNRNPA1	HNRNPA1;HNRPA1;HNRPA1L3;MGC102835;hnRNP A1;hnRNP-A1	heterogeneous nuclear ribonucleoprotein A1	0.92	0.84	0.93	0.7
TXNRD3NB	TXNRD3NB;MGC163418;MGC163420;TR2IT1;TXNRD3IT1;TXNRD3NT1	thioredoxin reductase 3 neighbor	0.92	0.82	0.93	0.59
SPACA4	SPACA4;SAMP14	sperm acrosome associated 4	0.91	0.88	0.93	0.62
NGEF	NGEF;ARHGEF27;EPHEXIN	neuronal guanine nucleotide exchange factor	0.91	0.82	0.93	0.65
OR2F2	OR2F2;OR7-1	olfactory receptor, family 2, subfamily F, member 2	0.9	0.82	0.93	0.62
CDH11	CDH11;CAD11;CDHOB;OB;OSF-4	cadherin 11, type 2, OB-cadherin (osteoblast)	0.9	0.86	0.93	0.7
TMPRSS6	TMPRSS6;IRIDA	transmembrane protease, serine 6	0.9	0.82	0.93	0.62
BMP2	BMP2;BDA2;BMP2A	bone morphogenetic protein 2	0.9	0.89	0.93	0.69
RRAGA	RRAGA;FIP-1;FIP1;RAGA	Ras-related GTP binding A	0.9	0.83	0.93	0.62
KLRG2	KLRG2;CLEC15B;FLJ44186;MGC131846	killer cell lectin-like receptor subfamily G, member 2	0.9	0.74	0.93	0.58
FAM8A1	FAM8A1;AHCP;FLJ23721	family with sequence similarity 8, member A1	0.9	0.76	0.93	0.61
DNAJC5	DNAJC5;CLN4B;CSP;DKFZp434N1429;DKFZp761N1221;DNAJC5A;FLJ00118;FLJ13070	DnaJ (Hsp40) homolog, subfamily C, member 5	0.9	0.87	0.93	0.66
TRABD	TRABD;LP6054;MGC110928;PP2447	TraB domain containing	0.9	0.84	0.93	0.61
P2RX1	P2RX1;P2X1	purinergic receptor P2X, ligand-gated ion channel, 1	0.89	0.82	0.93	0.64
TBCEL	TBCEL;EI;FLJ14177;LRRC35;MGC10233	tubulin folding cofactor E-like	0.89	0.74	0.93	0.58
AKT1	AKT1;AKT;MGC99656;PKB;PKB-ALPHA;PRKBA;RAC;RAC-ALPHA	v-akt murine thymoma viral oncogene homolog 1	0.89	0.82	0.93	0.68
C2orf85	C2orf85;FLJ33590;MGC142077;MGC142079	chromosome 2 open reading frame 85	0.89	0.86	0.93	0.58
COL11A1	COL11A1;CO11A1;COLL6;STL2	collagen, type XI, alpha 1	0.88	0.8	0.93	0.64
C15orf40	C15orf40;FLJ33606;MGC29937	chromosome 15 open reading frame 40	0.88	0.84	0.93	0.6
THBD	THBD;AHUS6;BDCA3;CD141;THRM;TM	thrombomodulin	0.88	0.78	0.93	0.58
SHISA2	SHISA2;C13orf13;PRO28631;TMEM46;WGAR9166;bA398O19.2;hShisa	shisa homolog 2 (Xenopus laevis)	0.88	0.8	0.93	0.61
FOXO4	FOXO4;AFX;AFX1;MGC120490;MLLT7	forkhead box O4	0.88	0.82	0.93	0.55
IKBKE	IKBKE;IKK-E;IKK-i;IKKE;IKKI;KIAA0151;MGC125294;MGC125295;MGC125297	inhibitor of kappa light polypeptide gene enhancer in B-cells, kinase epsilon	0.88	0.88	0.93	0.62
GPR180	GPR180;DKFZp586C1322;DKFZp667K0825;ITR	G protein-coupled receptor 180	0.88	0.8	0.93	0.58
TRIM44	TRIM44;DIPB;HSA249128;MC7;MGC3490	tripartite motif containing 44	0.88	0.9	0.93	0.66
ASNS	ASNS;TS11	asparagine synthetase (glutamine-hydrolyzing)	0.88	0.8	0.93	0.62
RYR3	RYR3	ryanodine receptor 3	0.87	0.8	0.93	0.62
HLA-DRA	HLA-DRA;FLJ51114;HLA-DRA1;MLRW	major histocompatibility complex, class II, DR alpha	0.87	0.82	0.93	0.63
SPARC	SPARC;ON	secreted protein, acidic, cysteine-rich (osteonectin)	0.87	0.89	0.93	0.65
VCX3B	VCX3B;MGC119815;VCX-C;VCXC	variable charge, X-linked 3B	0.87	0.8	0.93	0.6
NPR3	NPR3;ANP-C;ANPR-C;ANPRC;C5orf23;FLJ14054;GUCY2B;MGC22189;NPR-C;NPRC	natriuretic peptide receptor C/guanylate cyclase C (atrionatriuretic peptide receptor C)	0.87	0.79	0.93	0.71
SERPINB7	SERPINB7;DKFZp686D06190;MEGSIN;MGC120014;MGC120015	serpin peptidase inhibitor, clade B (ovalbumin), member 7	0.86	0.78	0.93	0.63
CD1C	CD1C;BDCA1;CD1;CD1A;R7	CD1c molecule	0.86	0.81	0.93	0.56

ODZ1	ODZ1;ODZ3;TEN-M1;TNM;TNM1	odz, odd Oz/ten-m homolog 1 (Drosophila)	0.86	0.82	0.93	0.55
HOOK2	HOOK2;FLJ26218;HK2	hook homolog 2 (Drosophila)	0.86	0.76	0.93	0.64
TCTA	TCTA	T-cell leukemia translocation altered gene	0.86	0.79	0.93	0.66
NDUFA4	NDUFA4;CI-9k;CI-MLRQ;FLJ27440;MGC104422;MGC126843;MGC126845;MLRQ	NADH dehydrogenase (ubiquinone) 1 alpha subcomplex, 4, 9kDa	0.86	0.76	0.93	0.55
TBCE	TBCE;HRD;KCS;KCS1;pac2	tubulin folding cofactor E	0.86	0.82	0.93	0.62
SLC25A32	SLC25A32;FLJ23872;MFT;MFTC	solute carrier family 25, member 32	0.86	0.76	0.93	0.55
SLC34A2	SLC34A2;FLJ90534;NAPI-3B;NAPI-IIb;NPTIIb	solute carrier family 34 (sodium phosphate), member 2	0.86	0.84	0.93	0.56
PTH1R	PTH1R;MGC138426;MGC138452;PFE;PTHR;PTHR1	parathyroid hormone 1 receptor	0.86	0.78	0.93	0.7
PROK1	PROK1;EGVEGF;PK1;PRK1	prokineticin 1	0.85	0.76	0.93	0.57
C11orf71	C11orf71;FLJ20010	chromosome 11 open reading frame 71	0.85	0.79	0.93	0.57
TP53RK	TP53RK;BUD32;C20orf64;Nori-2;Nori-2p;PRPK;dJ101A2	TP53 regulating kinase	0.85	0.84	0.93	0.61
ATAD3B	ATAD3B;AAA-TOB3;KIAA1273;TOB3	ATPase family, AAA domain containing 3B	0.85	0.81	0.93	0.54
PRSS1	PRSS1;DKFZp779A0837;FLJ57296;MGC120175;MGC149362;TRP1;TRY1;TRY4;TRYP1	protease, serine, 1 (trypsin 1)	1.41	1.32	0.94	0.99
RAB2B	RAB2B;FLJ14824	RAB2B, member RAS oncogene family	1.33	1.27	0.94	1.01
EDNRA	EDNRA;ETA;ETAR;ETRA	endothelin receptor type A	1.32	1.16	0.94	0.87
A4GALT	A4GALT;A14GALT;A4GALT1;Gb3S;P(k);P1;PK	alpha 1,4-galactosyltransferase	1.29	1.23	0.94	0.98
PADI3	PADI3;MGC126307;MGC126308;PAD3;PDI3	peptidyl arginine deiminase, type III	1.28	1.16	0.94	1
ADNP	ADNP;ADNP1;KIAA0784	activity-dependent neuroprotector homeobox	1.22	1.19	0.94	0.81
PRSS58	PRSS58;FLJ16649;MGC35022;PRSS1;TRY1;TRYX3;UNQ2540	protease, serine, 58	1.22	1.14	0.94	0.85
KISS1R	KISS1R;AXOR12;GPR54;HOT7T175	KISS1 receptor	1.21	1.04	0.94	0.79
XYLB	XYLB;FLJ10343;FLJ12539;FLJ22075	xylulokinase homolog (H. influenzae)	1.21	1.11	0.94	1
ITGA7	ITGA7;FLJ25220	integrin, alpha 7	1.19	1.08	0.94	0.84
ZNF557	ZNF557;FLJ96454;MGC4054	zinc finger protein 557	1.19	1.17	0.94	0.83
PLAC8L1	PLAC8L1	PLAC8-like 1	1.18	1.17	0.94	0.9
PEX7	PEX7;PTS2R;RCDP1;RD	peroxisomal biogenesis factor 7	1.17	1.12	0.94	0.75
IQCE	IQCE;1700028P05Rik;KIAA1023;MGC41907	IQ motif containing E	1.17	1.08	0.94	0.81
HMOX1	HMOX1;HO-1;HSP32;bK286B10	heme oxygenase (decycling) 1	1.17	1.09	0.94	0.81
CAMK2G	CAMK2G;CAMK;CAMK-II;CAMKG;FLJ16043;MGC26678	calcium/calmodulin-dependent protein kinase II gamma	1.17	1.09	0.94	0.9
RFWD2	RFWD2;COP1;FLJ10416;RNF200	ring finger and WD repeat domain 2	1.17	1.06	0.94	0.77
LCA5L	LCA5L;C21orf13;MGC33295	Leber congenital amaurosis 5-like	1.16	1.06	0.94	0.84
VWA5B1	VWA5B1;FLJ32784;FLJ43845	von Willebrand factor A domain containing 5B1	1.16	1.04	0.94	0.8
NRG4	NRG4;DKFZp779N0541;DKFZp779N1944;HRG4	neuregulin 4	1.16	0.98	0.94	0.82
PRSS35	PRSS35;C6orf158;MGC46520;dJ223E3.1	protease, serine, 35	1.14	1.06	0.94	0.8
PHF21B	PHF21B;BHC80L;FLJ34161;PHF4	PHD finger protein 21B	1.14	1.02	0.94	0.74
SSBP4	SSBP4;MGC3181	single stranded DNA binding protein 4	1.14	1.04	0.94	0.82
RGAG1	RGAG1;KIAA1318;MAR9;MART9;MGC142230	retrotransposon gag domain containing 1	1.12	0.94	0.94	0.73
RDH12	RDH12;FLJ30273;LCA13;LCA3;SDR7C2	retinol dehydrogenase 12 (all-trans/9-cis/11-cis)	1.12	0.98	0.94	0.78
C1R	C1R	complement component 1, r subcomponent	1.12	0.97	0.94	0.73
ZNF75D	ZNF75D;D8C6;MGC126327;ZNF75;ZNF82	zinc finger protein 75D	1.11	0.94	0.94	0.79
SH2D2A	SH2D2A;F2771;SCAP;TSAD;VRAP	SH2 domain containing 2A	1.1	1	0.94	0.76
MAGEA6	MAGEA6;CT1.6;MAGE-3b;MAGE3B;MAGE6;MGC52297	melanoma antigen family A, 6	1.1	1.08	0.94	0.76
LOC653111	LOC653111	ENTREZ STATUS: REMAPPED TO GENE ID: 642612	1.1	1	0.94	0.72
NIM1	NIM1;MGC42105	serine/threonine-protein kinase NIM1	1.1	1.02	0.94	0.85
BIVM	BIVM;FLJ44301;FLJ44556;MGC133326	basic, immunoglobulin-like variable motif containing	1.09	1.06	0.94	0.73
KIAA0415	KIAA0415;SPG48	KIAA0415	1.09	1.06	0.94	0.81
HPS1	HPS1;HPS;MGC5277	Hermansky-Pudlak syndrome 1	1.09	1.06	0.94	0.8
COG2	COG2;LDLC	component of oligomeric golgi complex 2	1.09	0.92	0.94	0.73
KAT6A	KAT6A;MGC167033;MOZ;MYST3;RUNXBP2;ZNF220	K(lysine) acetyltransferase 6A	1.09	1	0.94	0.75
TTLL2	TTLL2;C6orf104;NYD-TSPG;dJ366N23.3	tubulin tyrosine ligase-like family, member 2	1.08	0.96	0.94	0.81
tAKR	tAKR	aldo-keto reductase, truncated	1.08	1.08	0.94	0.81
OR2AG2	OR2AG2;OR11-76;OR2AG2P	olfactory receptor, family 2, subfamily AG, member 2	1.08	1.07	0.94	0.86
MEIS2	MEIS2;HsT18361;MGC2820;MRG1	Meis homeobox 2	1.08	1	0.94	0.78
OR2M5	OR2M5;OR2M5P	olfactory receptor, family 2, subfamily M, member 5	1.08	0.98	0.94	0.75
SCRN3	SCRN3;FLJ23142;MGC149597;SES3	secernin 3	1.08	0.98	0.94	0.71
STX2	STX2;EPIM;EPM;MGC51014;STX2A;STX2B;STX2C	syntaxin 2	1.08	0.99	0.94	0.73
STAU1	STAU1;FLJ25010;STAU	staufen, RNA binding protein, homolog 1 (Drosophila)	1.08	1.02	0.94	0.73
KCNAB1	KCNAB1;AKR6A3;KCNA1B;KV-BETA-1;Kvb1.3;hKvBeta3;hKvb3	potassium voltage-gated channel, shaker-related subfamily, beta member 1	1.07	1.04	0.94	0.81
FSD1	FSD1;GLFND;MGC3213;MIR1	fibronectin type III and SPRY domain containing 1	1.07	0.97	0.94	0.7
NXPH4	NXPH4;NPH4	neurexophilin 4	1.07	0.98	0.94	0.77
PARP14	PARP14;BAL2;KIAA1268;PARP-14;pART8	poly (ADP-ribose) polymerase family, member 14	1.07	1	0.94	0.74
DIP2C	DIP2C;FLJ34444;FLJ44075;KIAA0934	DIP2 disco-interacting protein 2 homolog C (Drosophila)	1.06	1.08	0.94	0.72
CC2D1B	CC2D1B;KIAA1836	coiled-coil and C2 domain containing 1B	1.06	1.03	0.94	0.77
PDIK1L	PDIK1L;CLIK1L;STK35L2	PDLIM1 interacting kinase 1 like	1.06	0.99	0.94	0.82
DR1	DR1;NC2;NC2-BETA	down-regulator of transcription 1, TBP-binding (negative cofactor 2)	1.06	0.97	0.94	0.68
ATP2B4	ATP2B4;ATP2B2;DKFZp686G08106;DKFZp686M088;MXRA1;PMCA4;PMCA4b;PMCA4x	ATPase, Ca++ transporting, plasma membrane 4	1.06	1	0.94	0.74
FOXR2	FOXR2;FOXN6;MGC21658	forkhead box R2	1.05	1.02	0.94	0.81
PPTC7	PPTC7;DKFZp686M07120;MGC133072;TA-PP2C;TAPP2C	PTC7 protein phosphatase homolog (S. cerevisiae)	1.05	0.98	0.94	0.76
PITPNM1	PITPNM1;DRES9;FLJ44997;NIR2;PITPNM;RDGB;RDGB1;RDGBA;RDGBA1;Rd9	phosphatidylinositol transfer protein, membrane-associated 1	1.05	1.08	0.94	0.75
ATP1A1	ATP1A1;MGC3285;MGC51750	ATPase, Na+/K+ transporting, alpha 1 polypeptide	1.05	1.06	0.94	0.79
ARAP3	ARAP3;CENTD3;DRAG1;FLJ21065	ArfGAP with RhoGAP domain, ankyrin repeat and PH domain 3	1.05	1.03	0.94	0.69
WNT2B	WNT2B;WNT13;XWNT2	wingless-type MMTV integration site family, member 2B	1.04	0.96	0.94	0.8
TMEM201	TMEM201;NET5	transmembrane protein 201	1.04	0.94	0.94	0.72
ZNF627	ZNF627;FLJ90365	zinc finger protein 627	1.04	1.02	0.94	0.73
SIX1	SIX1;BOS3;DFNA23;TIP39	SIX homeobox 1	1.03	0.93	0.94	0.67
PTBP2	PTBP2;FLJ34897;PTB;PTBLP;brPTB;nPTB;nPTB5;nPTB6;nPTB7;nPTB8	polypyrimidine tract binding protein 2	1.03	0.98	0.94	0.78
CXCR5	CXCR5;BLR1;CD185;MDR15;MGC117347	chemokine (C-X-C motif) receptor 5	1.02	0.9	0.94	0.68
BEGAIN	BEGAIN;KIAA1446	brain-enriched guanylate kinase-associated homolog (rat)	1.02	0.94	0.94	0.76
PAQR7	PAQR7;MPRA;PGLP;mSR	progestin and adipoQ receptor family member VII	1.02	0.97	0.94	0.66
DPH3	DPH3;DELGIP;DELGIP1;DESR1;DPH3A;KTI11;MGC20197;ZCSL2	DPH3, KTI11 homolog (S. cerevisiae)	1.02	0.9	0.94	0.73
LEMD1	LEMD1;CT50;LEMP-1	LEM domain containing 1	1.01	0.99	0.94	0.74
NUP62CL	NUP62CL;FLJ20130	nucleoporin 62kDa C-terminal like	1.01	0.99	0.94	0.71
TRMT5	TRMT5;KIAA1393;MGC111453;TRM5	TRM5 tRNA methyltransferase 5 homolog (S. cerevisiae)	1.01	0.94	0.94	0.68
RNF19B	RNF19B;FLJ90005;IBRDC3;NKLAM	ring finger protein 19B	1.01	0.94	0.94	0.85
RNF149	RNF149;DNAPTP2;FLJ90504	ring finger protein 149	1.01	0.94	0.94	0.7
GSTA3	GSTA3;GSTA3-3;GTA3;MGC22232	glutathione S-transferase alpha 3	1	0.94	0.94	0.69
GNG2	GNG2	guanine nucleotide binding protein (G protein), gamma 2	1	0.95	0.94	0.73
SGSM1	SGSM1;KIAA1941;MGC133298;RUTBC2	small G protein signaling modulator 1	1	0.94	0.94	0.64
GIN1	GIN1;FLJ20125;GIN-1;TGIN1;ZH2C2	gypsy retrotransposon integrase 1	1	1.01	0.94	0.71
SELM	SELM;MGC40146;SEPM	selenoprotein M	1	0.92	0.94	0.77
ADK	ADK;AK	adenosine kinase	1	0.94	0.94	0.78
BHLHE22	BHLHE22;BHLHB5;Beta3;CAGL85;TNRC20	basic helix-loop-helix family, member e22	1	0.92	0.94	0.7
TNNC2	TNNC2	troponin C type 2 (fast)	0.99	0.91	0.94	0.68
PLEKHA7	PLEKHA7;DKFZp686M22243	pleckstrin homology domain containing, family A member 7	0.99	0.83	0.94	0.65
ALKBH3	ALKBH3;ABH3;DEPC-1;DEPC1;FLJ43614;MGC118790;MGC118792;MGC118793;PCA1	alkB, alkylation repair homolog 3 (E. coli)	0.99	0.94	0.94	0.63
PGC	PGC;FLJ99563;PEPC;PGII	progastricsin (pepsinogen C)	0.98	0.86	0.94	0.65
MICALL1	MICALL1;DKFZp686M2226;FLJ45921;KIAA1668;MICAL-L1;MIRAB13	MICAL-like 1	0.98	1.01	0.94	0.67
IGBP1	IGBP1;ALPHA-4;IBP1	immunoglobulin (CD79A) binding protein 1	0.98	0.86	0.94	0.64
CUBN	CUBN;FLJ90055;FLJ90747;IFCR;MGA1;gp280	cubilin (intrinsic factor-cobalamin receptor)	0.97	0.84	0.94	0.75
GPR141	GPR141;PGR13	G protein-coupled receptor 141	0.97	0.88	0.94	0.79
ZNF569	ZNF569;FLJ32053;ZAP1;ZNF	zinc finger protein 569	0.97	0.96	0.94	0.67
PDIA2	PDIA2;PDA2;PDI;PDIP;PDIR	protein disulfide isomerase family A, member 2	0.97	0.92	0.94	0.71
HNRNPA3	HNRNPA3;2610510D13Rik;D10S102;FBRNP;HNRPA3;MGC138232;MGC142030	heterogeneous nuclear ribonucleoprotein A3	0.97	0.88	0.94	0.67
TMEM225	TMEM225;PMP22CD	transmembrane protein 225	0.97	0.94	0.94	0.7
SRCIN1	SRCIN1;KIAA1684;P140;SNIP	SRC kinase signaling inhibitor 1	0.97	0.89	0.94	0.67
JKAMP	JKAMP;C14orf100;HSPC213;HSPC327;JAMP	JNK1/MAPK8-associated membrane protein	0.96	0.87	0.94	0.68
ZNF768	ZNF768;FLJ23436	zinc finger protein 768	0.95	0.98	0.94	0.65
LY6G6F	LY6G6F;C6orf21;G6f;LY6G6D;NG32	lymphocyte antigen 6 complex, locus G6F	0.94	0.92	0.94	0.69
KLK4	KLK4;AI2A1;ARM1;EMSP;EMSP1;KLK-L1;MGC116827;MGC116828;PRSS17;PSTS;kallikrein	kallikrein-related peptidase 4	0.94	0.83	0.94	0.62
LCN10	LCN10	lipocalin 10	0.94	0.94	0.94	0.75
PIGS	PIGS;DKFZp686K20216;FLJ45226	phosphatidylinositol glycan anchor biosynthesis, class S	0.94	0.87	0.94	0.66
SMC1A	SMC1A;CDLS2;DKFZp686L19178;DXS423E;KIAA0178;MGC138332;SB1.8;SMC1;SMC1L1;SMC1alpha;SMCB	structural maintenance of chromosomes 1A	0.94	0.86	0.94	0.67
PIAS1	PIAS1;DDXBP1;GBP;GU/RH-II;MGC141878;MGC141879;ZMIZ3	protein inhibitor of activated STAT, 1	0.94	0.84	0.94	0.66
CPA4	CPA4;CPA3	carboxypeptidase A4	0.94	0.82	0.94	0.61
CD96	CD96;DKFZp667E2122;MGC22596;TACTILE	CD96 molecule	0.93	0.9	0.94	0.71
CNTNAP1	CNTNAP1;CASPR;CNTNAP;NRXN4;P190	contactin associated protein 1	0.93	0.94	0.94	0.7
DOHH	DOHH;HLRC1;MGC4293;hDOHH	deoxyhypusine hydroxylase/monooxygenase	0.93	0.88	0.94	0.68
ZNF660	ZNF660;FLJ36870	zinc finger protein 660	0.92	0.78	0.94	0.61
ACMSD	ACMSD	aminocarboxymuconate semialdehyde decarboxylase	0.92	0.86	0.94	0.67
SLA	SLA;SLA1;SLAP	Src-like-adaptor	0.92	0.84	0.94	0.63
CTHRC1	CTHRC1	collagen triple helix repeat containing 1	0.92	0.89	0.94	0.68
SLC16A2	SLC16A2;AHDS;DXS128;DXS128E;MCT 7;MCT 8;MCT7;MCT8;MRX22;XPCT	solute carrier family 16, member 2 (monocarboxylic acid transporter 8)	0.92	0.86	0.94	0.63
BMP8B	BMP8B;BMP8;MGC131757;OP2	bone morphogenetic protein 8b	0.92	0.84	0.94	0.65
C2CD2L	C2CD2L;KIAA0285;TMEM24	C2CD2-like	0.92	0.89	0.94	0.61
TIMM10	TIMM10;TIM10;TIM10A	translocase of inner mitochondrial membrane 10 homolog (yeast)	0.92	0.9	0.94	0.73
PAFAH2	PAFAH2;FLJ26025;HSD-PLA2	platelet-activating factor acetylhydrolase 2, 40kDa	0.92	0.79	0.94	0.7
MED29	MED29;DKFZp434H247;IXL	mediator complex subunit 29	0.92	0.89	0.94	0.66
AMOTL1	AMOTL1;JEAP	angiomotin like 1	0.92	0.89	0.94	0.68
GCC2	GCC2;GCC185;KIAA0336;RANBP2L4;REN53	GRIP and coiled-coil domain containing 2	0.92	0.9	0.94	0.68
NCAPD2	NCAPD2;CAP-D2;CNAP1;KIAA0159;hCAP-D2	non-SMC condensin I complex, subunit D2	0.92	0.94	0.94	0.72

PPP1CC	PPP1CC;PP-1G;PPP1G	protein phosphatase 1, catalytic subunit, gamma isozyme	0.92	0.82	0.94	0.61
CEP19	CEP19;C3orf34;MGC14126	centrosomal protein 19kDa	0.92	0.9	0.94	0.64
PSG1	PSG1;B1G1;CD66f;DHFRP2;FLJ90598;FLJ90654;PBG1;PSBG1;PSGGA;PSGIIA;SP1	pregnancy specific beta-1-glycoprotein 1	0.91	0.76	0.94	0.61
PXMP4	PXMP4;PMP24	peroxisomal membrane protein 4, 24kDa	0.91	0.82	0.94	0.59
RTKN2	RTKN2;DKFZp686J10120;PLEKHK1;bA531F24.1	rhotekin 2	0.91	0.88	0.94	0.63
DBNL	DBNL;ABP1;HIP-55;SH3P7	drebrin-like	0.91	0.9	0.94	0.64
VPS35	VPS35;DKFZp434E1211;DKFZp434P1672;FLJ10752;FLJ13588;FLJ20388;MEM3;PARK17	vacuolar protein sorting 35 homolog (S. cerevisiae)	0.91	0.84	0.94	0.63
CST9L	CST9L;FLJ92394;bA218C14.1	cystatin 9-like	0.9	0.84	0.94	0.57
ESRRG	ESRRG;DKFZp781L1617;ERR3;ERRgamma;FLJ16023;KIAA0832;NR3B3	estrogen-related receptor gamma	0.9	0.85	0.94	0.57
KCNK10	KCNK10;FLJ43399;K2p10.1;TREK-2;TREK2	potassium channel, subfamily K, member 10	0.9	0.84	0.94	0.66
ZNF433	ZNF433;FLJ40981	zinc finger protein 433	0.9	0.82	0.94	0.64
FOXD4L3	FOXD4L3;FOXD4L2;FOXD6;MGC119257	forkhead box D4-like 3	0.9	0.89	0.94	0.72
NTRK1	NTRK1;DKFZp781I14186;MTC;TRK;TRK1;TRKA;Trk-A;p140-TrkA	neurotrophic tyrosine kinase, receptor, type 1	0.9	0.82	0.94	0.74
CA11	CA11;CARPX1	carbonic anhydrase XI	0.9	0.8	0.94	0.58
CHIC2	CHIC2;BTL;MGC21173	cysteine-rich hydrophobic domain 2	0.9	0.88	0.94	0.66
C19orf51	C19orf51;FLJ36139;FLJ40069	chromosome 19 open reading frame 51	0.9	0.9	0.94	0.69
EIF4EBP1	EIF4EBP1;4E-BP1;4EBP1;BP-1;MGC4316;PHAS-I	eukaryotic translation initiation factor 4E binding protein 1	0.9	0.84	0.94	0.66
SLC9A3R1	SLC9A3R1;EBP50;NHERF;NHERF1;NPHLOP2	solute carrier family 9 (sodium/hydrogen exchanger), member 3 regulator 1	0.9	0.81	0.94	0.62
EPAS1	EPAS1;ECYT4;HIF2A;HLF;MOP2;PASD2;bHLHe73	endothelial PAS domain protein 1	0.9	0.84	0.94	0.62
RHCG	RHCG;C15orf6;PDRC2;RHGK;SLC42A3	Rh family, C glycoprotein	0.89	0.86	0.94	0.62
ARNT2	ARNT2;KIAA0307;bHLHe1	aryl-hydrocarbon receptor nuclear translocator 2	0.89	0.92	0.94	0.64
ZNF395	ZNF395;DKFZp434K1210;HDBP-2;HDBP2;HDRF-2;PBF;PRF-1;PRF1;Si-1-8-14	zinc finger protein 395	0.89	0.87	0.94	0.58
MOCS2	MOCS2;MCBPE;MOCO1;MOCS2A;MOCS2B;MPTS	molybdenum cofactor synthesis 2	0.89	0.78	0.94	0.62
LAMC1	LAMC1;LAMB2;MGC87297	laminin, gamma 1 (formerly LAMB2)	0.89	0.82	0.94	0.61
NOX3	NOX3;GP91-3;MOX-2	NADPH oxidase 3	0.88	0.82	0.94	0.61
ANKRD20A2	ANKRD20A2;MGC176486	ankyrin repeat domain 20 family, member A2	0.88	0.85	0.94	0.67
LMTK3	LMTK3;KIAA1883;LMR3;TYKLM3	lemur tyrosine kinase 3	0.88	0.82	0.94	0.74
HOXB3	HOXB3;HOX2;HOX2G;Hox-2.7	homeobox B3	0.88	0.8	0.94	0.59
SGCB	SGCB;A3b;LGMD2E;SGC	sarcoglycan, beta (43kDa dystrophin-associated glycoprotein)	0.88	0.85	0.94	0.65
NPHP3	NPHP3;DKFZp667K242;DKFZp781K1312;FLJ30691;FLJ36696;KIAA2000;MGC78666;MKS7;NPH3;RHPD	nephronophthisis 3 (adolescent)	0.88	0.82	0.94	0.56
ZC3H3	ZC3H3;KIAA0150;ZC3HDC3	zinc finger CCCH-type containing 3	0.88	0.79	0.94	0.61
DDOST	DDOST;AGER1;KIAA0115;MGC2191;OKSWcl45;OST;OST48;WBP1	dolichyl-diphosphooligosaccharide--protein glycosyltransferase	0.88	0.78	0.94	0.62
OR7G2	OR7G2;OR19-6;OST260	olfactory receptor, family 7, subfamily G, member 2	0.87	0.78	0.94	0.57
PGA5	PGA5;PGA3;PGA4	pepsinogen 5, group I (pepsinogen A)	0.87	0.81	0.94	0.61
FGFBP3	FGFBP3;C10orf13;FGF-BP3;MGC39320	fibroblast growth factor binding protein 3	0.87	0.88	0.94	0.61
IL1R2	IL1R2;CD121b;IL1RB;MGC47725	interleukin 1 receptor, type II	0.87	0.82	0.94	0.64
C17orf77	C17orf77;FLJ31882	chromosome 17 open reading frame 77	0.86	0.79	0.94	0.6
TMC5	TMC5;FLJ13593	transmembrane channel-like 5	0.86	0.72	0.94	0.6
C10orf53	C10orf53;FLJ60598	chromosome 10 open reading frame 53	0.86	0.84	0.94	0.63
TSSK6	TSSK6;CT72;FLJ24002;SSTK;TSSK4	testis-specific serine kinase 6	0.86	0.86	0.94	0.62
TTLL10	TTLL10;FLJ36119;FLJ42131;TTLL5	tubulin tyrosine ligase-like family, member 10	0.86	0.83	0.94	0.62
MED12	MED12;ARC240;CAGH45;FGS1;HOPA;KIAA0192;OKS;OPA1;TNRC11;TRAP230	mediator complex subunit 12	0.86	0.79	0.94	0.61
TAF6	TAF6;DKFZp781E21155;MGC:8964;TAF(II)70;TAF(II)80;TAF2E;TAFII-70;TAFII-80;TAFII70;TAFII80;TAFII85	TAF6 RNA polymerase II, TATA box binding protein (TBP)-associated factor, 80kDa	0.86	0.85	0.94	0.56
TBC1D15	TBC1D15;DKFZp686M1379;DKFZp761D0223;FLJ12085;FLJ33191;RAB7-GAP	TBC1 domain family, member 15	0.86	0.82	0.94	0.59
PEG10	PEG10;EDR;HB-1;KIAA1051;MEF3L;Mar2;Mart2;RGAG3	paternally expressed 10	0.86	0.84	0.94	0.64
PGAM4	PGAM4;PGAM-B;PGAM1;PGAM3;dJ1000K24.1	phosphoglycerate mutase family member 4	0.86	0.8	0.94	0.62
CLEC18A	CLEC18A;FLJ77955;MGC164804;MRCL;MRCL1	C-type lectin domain family 18, member A	0.86	0.78	0.94	0.61
IQCC	IQCC;FLJ10547;MGC120341;RP4-622L5.6	IQ motif containing C	0.85	0.8	0.94	0.57
TARSL2	TARSL2;FLJ25005	threonyl-tRNA synthetase-like 2	0.85	0.81	0.94	0.64
ECE2	ECE2;KIAA0604;MGC17664;MGC2408;MGC78487	endothelin converting enzyme 2	0.85	0.78	0.94	0.57
C9orf21	C9orf21	chromosome 9 open reading frame 21	0.85	0.82	0.94	0.67
APLF	APLF;APFL;C2orf13;FLJ16593;MGC47799;PALF;Xip1	aprataxin and PNKP like factor	0.85	0.83	0.94	0.63
ZNF500	ZNF500;ZKSCAN18	zinc finger protein 500	1.46	1.51	0.95	1.11
MOB3C	MOB3C;MGC26743;MOB1E;MOBKL2C	MOB kinase activator 3C	1.4	1.33	0.95	0.9
DOPEY2	DOPEY2;21orf5;C21orf5	dopey family member 2	1.29	1.27	0.95	0.91
HCAR1	HCAR1;GPR104;GPR81;HCA1;LACR1;TA-GPCR	hydroxycarboxylic acid receptor 1	1.26	1.16	0.95	1.05
DARS2	DARS2;ASPRS;FLJ10514;LBSL;MT-ASPRS;RP3-383J4.2	aspartyl-tRNA synthetase 2, mitochondrial	1.25	1.25	0.95	0.89
KCNJ10	KCNJ10;BIRK-10;KCNJ13-PEN;KIR1.2;KIR4.1;SESAME	potassium inwardly-rectifying channel, subfamily J, member 10	1.23	1.19	0.95	0.93
MPPE1	MPPE1;PGAP5	metallophosphoesterase 1	1.23	1.19	0.95	0.94
JHDM1D	JHDM1D;KDM7A;KIAA1718	jumonji C domain containing histone demethylase 1 homolog D (S. cerevisiae)	1.23	1.12	0.95	0.81
MYCBP2	MYCBP2;DKFZp686M08244;FLJ10106;FLJ13826;FLJ21597;FLJ21646;KIAA0916;PAM	MYC binding protein 2	1.23	1.11	0.95	0.8
TMEM184C	TMEM184C;FLJ10846;TMEM34	transmembrane protein 184C	1.21	1.11	0.95	0.84
SERPINB9	SERPINB9;CAP-3;CAP3;PI9	serpin peptidase inhibitor, clade B (ovalbumin), member 9	1.19	1.09	0.95	0.77
THOC1	THOC1;HPR1;P84;P84N5	THO complex 1	1.18	1	0.95	0.84
HIST1H2AJ	HIST1H2AJ;H2A/E;H2AFE;dJ160A22.4	histone cluster 1, H2aj	1.17	1.09	0.95	0.91
WDSUB1	WDSUB1;FLJ36175;UBOX6;WDSAM1	WD repeat, sterile alpha motif and U-box domain containing 1	1.17	1.06	0.95	0.77
MEX3D	MEX3D;KIAA2031;MEX-3D;MEX3;OK/SW-cl.4;RKHD1;RNF193;TINO	mex-3 homolog D (C. elegans)	1.17	1.06	0.95	0.77
IGFBP2	IGFBP2;IBP2;IGF-BP53	insulin-like growth factor binding protein 2, 36kDa	1.16	1.14	0.95	0.87
KRT85	KRT85;HB5;Hb-5;KRTHB5;hHb5	keratin 85	1.15	1.13	0.95	0.84
OCRL	OCRL;INPP5F;LOCR;NPHL2;OCRL-1;OCRL1	oculocerebrorenal syndrome of Lowe	1.15	1.09	0.95	0.85
VCX3A	VCX3A;MGC118976;MGC125730;MGC125796;VCX-8r;VCX-A;VCX3;VCX8R;VCXA	variable charge, X-linked 3A	1.14	1.05	0.95	0.79
CYP11B2	CYP11B2;ALDOS;CPN2;CYP11B;CYP11BL;P-450C18;P450C18;P450aldo	cytochrome P450, family 11, subfamily B, polypeptide 2	1.14	1.04	0.95	0.75
GPR113	GPR113;FLJ16767;PGR23;hGPCR37	G protein-coupled receptor 113	1.14	1	0.95	0.76
MTIF2	MTIF2	mitochondrial translational initiation factor 2	1.14	1.02	0.95	0.81
FAF2	FAF2;ETEA;KIAA0887;UBXD8;UBXN3B	Fas associated factor family member 2	1.13	1.12	0.95	0.81
C17orf67	C17orf67	chromosome 17 open reading frame 67	1.13	0.97	0.95	0.75
FBN1	FBN1;ACMICD;FBN;GPHYSD2;MASS;MFS1;OCTD;SGS;SSKS;WMS;WMS2	fibrillin 1	1.12	1.1	0.95	0.83
TOR1A	TOR1A;DQ2;DYT1	torsin family 1, member A (torsin A)	1.12	0.98	0.95	0.73
KBTBD6	KBTBD6;DKFZp547E1912;DKFZp547M073;FLJ58260	kelch repeat and BTB (POZ) domain containing 6	1.11	1.15	0.95	0.84
TMEM50B	TMEM50B;C21orf4;DKFZp686C2482;FLJ26146;HCVP7TP3	transmembrane protein 50B	1.11	1.11	0.95	0.9
FGF17	FGF17;FGF-13	fibroblast growth factor 17	1.1	1.15	0.95	0.8
CHRFAM7A	CHRFAM7A;CHRNA7;CHRNA7-DR1;D-10;MGC120482;MGC120483	CHRNA7 (cholinergic receptor, nicotinic, alpha 7, exons 5-10) and FAM7A (family with sequence similarity 7A, exons A-E) fusion	1.1	0.99	0.95	0.84
PDP1	PDP1;FLJ32517;FLJ56179;MGC119646;PDH;PDP;PDPC;PPM2C	pyruvate dehyrogenase phosphatase catalytic subunit 1	1.1	0.96	0.95	0.72
IFT81	IFT81;CDV-1;CDV-1R;CDV1;CDV1R;MGC102777;MGC4027	intraflagellar transport 81 homolog (Chlamydomonas)	1.09	1.07	0.95	0.8
PRDM4	PRDM4;MGC45046;PFM1	PR domain containing 4	1.09	1	0.95	0.72
TRIM8	TRIM8;GERP;RNF27	tripartite motif containing 8	1.09	1	0.95	0.72
COL19A1	COL19A1;COL9A1L;D6S228E	collagen, type XIX, alpha 1	1.08	0.97	0.95	0.82
GABRP	GABRP;MGC126386;MGC126387	gamma-aminobutyric acid (GABA) A receptor, pi	1.08	1.05	0.95	0.81
SH3BP2	SH3BP2;3BP-2;3BP2;CRBM;CRPM;FLJ42079;FLJ54978	SH3-domain binding protein 2	1.08	1.08	0.95	0.76
CARD9	CARD9;CANDF2;hCARD9	caspase recruitment domain family, member 9	1.08	1.06	0.95	0.81
C19orf54	C19orf54;FLJ17063;FLJ41131;MGC103014	chromosome 19 open reading frame 54	1.08	1.01	0.95	0.75
ZGPAT	ZGPAT;GPATC6;GPATCH6;KIAA1847;MGC44880;ZC3H9;ZC3HDC9;ZIP	zinc finger, CCCH-type with G patch domain	1.08	0.92	0.95	0.71
CBL	CBL;C-CBL;CBL2;NSLL;RNF55	Cas-Br-M (murine) ecotropic retroviral transforming sequence	1.08	0.98	0.95	0.7
SLC7A1	SLC7A1;ATRC1;CAT-1;ERR;HCAT1;REC1L	solute carrier family 7 (cationic amino acid transporter, y+ system), member 1	1.08	1.07	0.95	0.83
PCDHA13	PCDHA13;CNR5;CNRN5;CNRS5;CRNR5;PCDH-ALPHA13	protocadherin alpha 13	1.08	0.98	0.95	0.75
TAS2R45	TAS2R45;GPR59;MGC119923;MGC119924;MGC119925;T2R45;ZG24P	taste receptor, type 2, member 45	1.08	0.98	0.95	0.83
NRGN	NRGN;RC3;hng	neurogranin (protein kinase C substrate, RC3)	1.07	1	0.95	0.73
GIT2	GIT2;CAT-2;CAT2;DKFZp686G01261;KIAA0148;MGC760	G protein-coupled receptor kinase interacting ArfGAP 2	1.06	1.01	0.95	0.7
LARS2	LARS2;KIAA0028;LEURS;MGC26121	leucyl-tRNA synthetase 2, mitochondrial	1.06	0.96	0.95	0.69
TRAPPC6A	TRAPPC6A;MGC2650;TRS33	trafficking protein particle complex 6A	1.06	1.04	0.95	0.77
RMI2	RMI2;BLAP18;C16orf75;MGC24665	RMI2, RecQ mediated genome instability 2, homolog (S. cerevisiae)	1.06	1	0.95	0.72
CXorf65	CXorf65	chromosome X open reading frame 65	1.06	0.96	0.95	0.81
TOX2	TOX2;C20orf100;GCX-1;MGC15880;dJ1108D11.2;dJ495O3.1	TOX high mobility group box family member 2	1.05	0.99	0.95	0.71
WARS2	WARS2;TrpRS	tryptophanyl tRNA synthetase 2, mitochondrial	1.05	0.93	0.95	0.82
CSNK1G3	CSNK1G3	casein kinase 1, gamma 3	1.05	1	0.95	0.83
GPR149	GPR149;IEDA;PGR10	G protein-coupled receptor 149	1.04	0.97	0.95	0.87
ZFP161	ZFP161;MGC126126;ZBTB14;ZF5;ZFP-161;ZFP-5;ZNF478	zinc finger protein 161 homolog (mouse)	1.04	1	0.95	0.69
QKI	QKI;DKFZp586I0923;Hqk;QK;QK1;QK3;hqkI	QKI, KH domain containing, RNA binding	1.04	1.08	0.95	0.8
USP47	USP47;DKFZp686C13257;FLJ20727;TRFP	ubiquitin specific peptidase 47	1.04	0.98	0.95	0.73
FBXO40	FBXO40;FBX40;KIAA1195;MGC129902;MGC129903	F-box protein 40	1.03	0.94	0.95	0.68
FANCF	FANCF;FAF;MGC126856	Fanconi anemia, complementation group F	1.03	0.96	0.95	0.72
KIAA1199	KIAA1199;CCSP1;TMEM2L	KIAA1199	1.02	1	0.95	0.69
MYO5A	MYO5A;GS1;MYH12;MYO5;MYR12	myosin VA (heavy chain 12, myoxin)	1.02	0.99	0.95	0.78
SNX5	SNX5;FLJ10931	sorting nexin 5	1.02	0.93	0.95	0.67
ZNRF2	ZNRF2;RNF202	zinc and ring finger 2	1.02	0.92	0.95	0.67
STAT1	STAT1;CANDF7;DKFZp686B04100;ISGF-3;STAT91	signal transducer and activator of transcription 1, 91kDa	1.02	0.96	0.95	0.82
AXDND1	AXDND1;C1orf125;DKFZp686H1423;FLJ25438;FLJ32940;RP11-215I23.2	axonemal dynein light chain domain containing 1	1.02	0.99	0.95	0.74
PCDHA4	PCDHA4;CNR1;CNRN1;CRNR1;MGC138307;MGC142169;PCDH-ALPHA4	protocadherin alpha 4	1.02	1	0.95	0.76
ZNF683	ZNF683;MGC33414	zinc finger protein 683	1.01	0.86	0.95	0.67
MRPL28	MRPL28;MAAT1;MGC8499;p15	mitochondrial ribosomal protein L28	1.01	0.98	0.95	0.66
CPS1	CPS1;CPSASE1	carbamoyl-phosphate synthase 1, mitochondrial	1	0.88	0.95	0.7
ZNF721	ZNF721;FLJ11452;KIAA1982	zinc finger protein 721	1	1.02	0.95	0.71
CHAMP1	CHAMP1;C13orf8;CAMP;CHAMP;FLJ90413;KIAA1802;ZNF828	chromosome alignment maintaining phosphoprotein 1	1	0.9	0.95	0.75
HOXC11	HOXC11;HOX3H;MGC4906	homeobox C11	0.99	1	0.95	0.66
RHPN1	RHPN1;KIAA1929;ODF5;RHOPHILIN;RHPN	rhophilin, Rho GTPase binding protein 1	0.99	0.96	0.95	0.66
TSGA10IP	TSGA10IP;FAM161C;FLJ32880;MGC120465;MGC120466;MGC120468	testis specific, 10 interacting protein	0.99	0.88	0.95	0.72
LRRC8E	LRRC8E;FLJ23420	leucine rich repeat containing 8 family, member E	0.99	0.96	0.95	0.72
ZNF776	ZNF776;DKFZp686K10134;DKFZp781G1213;FLJ30886;FLJ38288	zinc finger protein 776	0.99	1.04	0.95	0.69
PGBD3	PGBD3;FLJ90201	piggyBac transposable element derived 3	0.99	0.88	0.95	0.75

RGS4	RGS4;DKFZp761F1924;MGC2124;MGC60244;RGP4;SCZD9	regulator of G-protein signaling 4	0.98	0.94	0.95	0.65
C14orf37	C14orf37;c14_5376	chromosome 14 open reading frame 37	0.98	0.88	0.95	0.74
MT1E	MT1E;MT1;MTD	metallothionein 1E	0.98	0.9	0.95	0.71
UNC45A	UNC45A;FLJ10043;GC-UNC45;GCUNC-45;GCUNC45;IRO039700;SMAP-1;SMAP1	unc-45 homolog A (C. elegans)	0.98	1	0.95	0.7
PHF7	PHF7;DKFZp434L1850;HSPC045;HSPC226;MGC26088;NYD-SP6	PHD finger protein 7	0.97	0.88	0.95	0.63
C16orf55	C16orf55;FLJ31606	chromosome 16 open reading frame 55	0.97	0.9	0.95	0.68
RAB12	RAB12;FLJ45927;MGC104724	RAB12, member RAS oncogene family	0.97	0.94	0.95	0.72
TMEM9	TMEM9;TMEM9A	transmembrane protein 9	0.97	0.89	0.95	0.7
BBS4	BBS4	Bardet-Biedl syndrome 4	0.96	0.95	0.95	0.71
PRSS27	PRSS27;CAPH2;MPN	protease, serine 27	0.96	0.9	0.95	0.68
CELSR2	CELSR2;CDHF10;EGFL2;FLJ34118;FLJ42737;FLJ45143;FLJ45845;Flamingo1;KIAA0279;MEGF3	cadherin, EGF LAG seven-pass G-type receptor 2 (flamingo homolog, Drosophila)	0.96	0.89	0.95	0.81
MSRB2	MSRB2;CBS-1;CBS1;MGC26104;MSRB;PILB	methionine sulfoxide reductase B2	0.96	0.89	0.95	0.7
SEC24A	SEC24A	SEC24 family, member A (S. cerevisiae)	0.96	0.98	0.95	0.65
HOXC4	HOXC4;HOX3;HOX3E;cp19	homeobox C4	0.96	0.88	0.95	0.62
GTF3C4	GTF3C4;FLJ21002;KAT12;MGC138450;TFIII90;TFIIIC290;TFIIIC90;TFIIICDELTA	general transcription factor IIIC, polypeptide 4, 90kDa	0.95	0.9	0.95	0.67
SENP6	SENP6;FLJ11355;FLJ11887;KIAA0389;KIAA0797;SSP1;SUSP1	SUMO1/sentrin specific peptidase 6	0.95	0.89	0.95	0.67
CPSF7	CPSF7;CFIm59;FLJ12529;FLJ39024;MGC9315	cleavage and polyadenylation specific factor 7, 59kDa	0.95	0.88	0.95	0.71
TSPYL5	TSPYL5;KIAA1750	TSPY-like 5	0.94	0.94	0.95	0.71
SYT2	SYT2;FLJ42519;SytII	synaptotagmin II	0.94	0.86	0.95	0.66
GTDC1	GTDC1;FLJ44822;Hmat-Xa;mat-Xa	glycosyltransferase-like domain containing 1	0.94	0.86	0.95	0.68
LRG1	LRG1;FLJ45787;HMFT1766;LRG	leucine-rich alpha-2-glycoprotein 1	0.94	0.89	0.95	0.62
RBM41	RBM41;FLJ11016;FLJ13670	RNA binding motif protein 41	0.94	0.94	0.95	0.67
DPP3	DPP3;DPPIII;FLJ11387;FLJ22331	dipeptidyl-peptidase 3	0.94	0.82	0.95	0.62
SLMO2	SLMO2;C20orf45;PRELID3B;dJ543J19.5	slowmo homolog 2 (Drosophila)	0.94	0.87	0.95	0.65
LPAR6	LPAR6;ARWH1;LAH3;MGC120358;P2RY5;P2Y5	lysophosphatidic acid receptor 6	0.94	0.84	0.95	0.63
DEFB106B	DEFB106B	defensin, beta 106B	0.94	0.8	0.95	0.67
AFF4	AFF4;AF5Q31;MCEF;MGC75036	AF4/FMR2 family, member 4	0.93	0.88	0.95	0.62
CD5L	CD5L;AIM;API6;PRO229;SP-ALPHA;Spalpha	CD5 molecule-like	0.92	0.86	0.95	0.6
SEC61A2	SEC61A2;FLJ10578	Sec61 alpha 2 subunit (S. cerevisiae)	0.92	0.84	0.95	0.65
SMYD5	SMYD5;NN8-4AG;RAI15;RRG1;ZMYND23	SMYD family member 5	0.92	0.84	0.95	0.63
TMEM222	TMEM222;C1orf160;DKFZp564D0478;MGC111002	transmembrane protein 222	0.92	0.94	0.95	0.66
BLOC1S3	BLOC1S3;BLOS3;FLJ26641;FLJ26676;HPS8;RP	biogenesis of lysosomal organelles complex-1, subunit 3	0.92	0.84	0.95	0.64
ACTR1B	ACTR1B;ARP1B;CTRN2;PC3	ARP1 actin-related protein 1 homolog B, centractin beta (yeast)	0.92	0.9	0.95	0.62
SOX4	SOX4;EVI16	SRY (sex determining region Y)-box 4	0.92	0.93	0.95	0.61
HERPUD2	HERPUD2;FLJ22313;FLJ31032	HERPUD family member 2	0.92	0.82	0.95	0.67
OSTCP1	OSTCP1;DC2L;OSTCL	oligosaccharyltransferase complex subunit pseudogene 1	0.92	0.84	0.95	0.64
PCDP1	PCDP1;MGC33657	primary ciliary dyskinesia protein 1	0.92	0.95	0.95	0.63
ZNRF1	ZNRF1;DKFZp434E229;FLJ14846;MGC15430;NIN283	zinc and ring finger 1	0.91	0.84	0.95	0.76
TRAF6	TRAF6;MGC:3310;RNF85	TNF receptor-associated factor 6	0.91	0.86	0.95	0.77
WDR13	WDR13;DKFZp779C2057;FLJ20563;MG21	WD repeat domain 13	0.91	0.9	0.95	0.68
ATP11B	ATP11B;ATPIF;ATPIR;DKFZp434J238;DKFZp434N1615;KIAA0956;MGC46576	ATPase, class VI, type 11B	0.91	0.84	0.95	0.66
LDHAL6B	LDHAL6B;LDH6B;LDHAL6;LDHL	lactate dehydrogenase A-like 6B	0.9	0.94	0.95	0.65
TMEM163	TMEM163;DC29;DKFZp566N034;DKFZp666J217;FLJ30176;SV31	transmembrane protein 163	0.9	0.9	0.95	0.64
PAQR8	PAQR8;C6orf33;FLJ32521;FLJ46206;LMPB1;MPRB	progestin and adipoQ receptor family member VIII	0.9	0.82	0.95	0.63
FAM63A	FAM63A;FLJ11280;FLJ43504;RP11-316M1.5	family with sequence similarity 63, member A	0.9	0.82	0.95	0.63
EPB49	EPB49;DMT;FLJ78462;FLJ98848	erythrocyte membrane protein band 4.9 (dematin)	0.9	0.88	0.95	0.59
STIM1	STIM1;D11S4896E;GOK;STIM1L	stromal interaction molecule 1	0.9	0.91	0.95	0.64
ZNF526	ZNF526;DKFZp762O059;KIAA1951;MGC4267	zinc finger protein 526	0.9	0.83	0.95	0.67
MGAT1	MGAT1;GLCNAC-TI;GLCT1;GLYT1;GNT-1;GNT-I;MGAT	mannosyl (alpha-1,3-)-glycoprotein beta-1,2-N-acetylglucosaminyltransferase	0.9	0.82	0.95	0.59
AP1S2	AP1S2;MGC:1902;MRX59;SIGMA1B	adaptor-related protein complex 1, sigma 2 subunit	0.9	0.9	0.95	0.6
SEC62	SEC62;Dtrp1;FLJ32803;HTP1;TLOC1;TP-1	SEC62 homolog (S. cerevisiae)	0.9	0.94	0.95	0.69
IFIT1B	IFIT1B;DKFZp781M1841;IFIT1L;bA149I23.6	interferon-induced protein with tetratricopeptide repeats 1B	0.9	0.9	0.95	0.63
AKR1E2	AKR1E2;AKR1CL2;AKRDC1;LoopADR;MGC10612	aldo-keto reductase family 1, member E2	0.9	0.82	0.95	0.64
ERN2	ERN2;IRE1-BETA;IRE1b	endoplasmic reticulum to nucleus signaling 2	0.89	0.92	0.95	0.65
HIGD1B	HIGD1B;CLST11240;CLST11240-15	HIG1 hypoxia inducible domain family, member 1B	0.89	0.92	0.95	0.68
MIS12	MIS12;2510025F08Rik;KNTC2AP;MGC2488;MTW1;hMis12	MIS12, MIND kinetochore complex component, homolog (S. pombe)	0.89	0.86	0.95	0.58
RBM22	RBM22;Cwc2;FLJ10290;ZC3H16;fSAP47	RNA binding motif protein 22	0.89	0.82	0.95	0.62
CUL1	CUL1;MGC149834;MGC149835	cullin 1	0.89	0.78	0.95	0.59
OR5AK2	OR5AK2	olfactory receptor, family 5, subfamily AK, member 2	0.88	0.86	0.95	0.59
TBC1D8B	TBC1D8B;FLJ20298	TBC1 domain family, member 8B (with GRAM domain)	0.88	0.87	0.95	0.63
NLRP10	NLRP10;CLR11.1;NALP10;NOD8;PAN5;PYNOD	NLR family, pyrin domain containing 10	0.88	0.92	0.95	0.64
SHPRH	SHPRH;FLJ27258;FLJ37625;FLJ45012;FLJ90837;KIAA2023;MGC134886;bA545I5.2	SNF2 histone linker PHD RING helicase	0.88	0.82	0.95	0.74
CCDC48	CCDC48;C3orf73;DKFZp686B0148;FLJ12053;FLJ12057;MGC138400	coiled-coil domain containing 48	0.88	0.88	0.95	0.62
FANCA	FANCA;FA;FA-H;FA1;FAA;FACA;FAH;FANCH;MGC75158	Fanconi anemia, complementation group A	0.88	0.82	0.95	0.7
RWDD2A	RWDD2A;MGC13523;MGC138208;RWDD2;dJ747H23.2	RWD domain containing 2A	0.88	0.88	0.95	0.66
TBC1D22B	TBC1D22B;C6orf197;DKFZp762J0110;FLJ20337;MGC125626;MGC125627;RP4-744I24.2;dJ744I24.2	TBC1 domain family, member 22B	0.88	0.82	0.95	0.6
BICD1	BICD1;BICD	bicaudal D homolog 1 (Drosophila)	0.88	0.88	0.95	0.71
RTF1	RTF1;GTL7;KIAA0252	Rtf1, Paf1/RNA polymerase II complex component, homolog (S. cerevisiae)	0.88	0.83	0.95	0.6
TJP1	TJP1;DKFZp686M05161;MGC133289;ZO-1	tight junction protein 1 (zona occludens 1)	0.88	0.92	0.95	0.63
CRIP1	CRIP1;CRHP;CRIP;CRP1;FLJ40971	cysteine-rich protein 1 (intestinal)	0.87	0.81	0.95	0.68
KLF2	KLF2;LKLF	Kruppel-like factor 2 (lung)	0.87	0.79	0.95	0.57
SPATA6	SPATA6;FLJ10007;HASH;SRF-1;SRF1	spermatogenesis associated 6	0.86	0.83	0.95	0.56
P2RY4	P2RY4;NRU;P2P;P2Y4;UNR	pyrimidinergic receptor P2Y, G-protein coupled, 4	0.86	0.8	0.95	0.71
TBX18	TBX18	T-box 18	0.86	0.8	0.95	0.57
MYL5	MYL5	myosin, light chain 5, regulatory	0.86	0.74	0.95	0.61
PPP1R16A	PPP1R16A;MGC14333;MYPT3	protein phosphatase 1, regulatory subunit 16A	0.86	0.82	0.95	0.55
SNX16	SNX16;DKFZp666H147	sorting nexin 16	0.86	0.84	0.95	0.56
MED27	MED27;CRAP34;CRSP34;CRSP8;MGC11274;TRAP37	mediator complex subunit 27	0.86	0.88	0.95	0.66
SLC19A2	SLC19A2;TC1;THT1;THTR1;TRMA	solute carrier family 19 (thiamine transporter), member 2	0.86	0.75	0.95	0.67
RRAS2	RRAS2;TC21	related RAS viral (r-ras) oncogene homolog 2	0.86	0.84	0.95	0.65
HP1BP3	HP1BP3;HP1-BP74;MGC43701	heterochromatin protein 1, binding protein 3	0.86	0.86	0.95	0.61
MUTED	MUTED;DKFZp686E2287;FLJ18427;MU	muted homolog (mouse)	0.86	0.77	0.95	0.65
AMY1B	AMY1B;AMY1;AMY1A;AMY1C;MGC177995	amylase, alpha 1B (salivary)	0.86	0.8	0.95	0.6
TMEM92	TMEM92;FLJ33318	transmembrane protein 92	0.85	0.88	0.95	0.59
PLEKHG2	PLEKHG2;ARHGEF42;CLG;DKFZp667J2325;FLJ00018;FLJ22458;FLJ38638	pleckstrin homology domain containing, family G (with RhoGef domain) member 2	0.85	0.76	0.95	0.65
PGGT1B	PGGT1B;BGGI;GGTI	protein geranylgeranyltransferase type I, beta subunit	0.85	0.78	0.95	0.55
ZNF765	ZNF765;DKFZp686J2252	zinc finger protein 765	0.85	0.86	0.95	0.6
DDX58	DDX58;DKFZp434J1111;DKFZp686N19181;FLJ13599;RIG-I	DEAD (Asp-Glu-Ala-Asp) box polypeptide 58	0.85	0.86	0.95	0.65
ANKRD28	ANKRD28;KIAA0379;PITK	ankyrin repeat domain 28	0.85	0.82	0.95	0.59
IFT43	IFT43;C14orf179;CED3;FLJ32173;MGC16028	intraflagellar transport 43 homolog (Chlamydomonas)	0.85	0.88	0.95	0.59
CCL19	CCL19;CKb11;ELC;MGC34433;MIP-3b;MIP3B;SCYA19	chemokine (C-C motif) ligand 19	1.27	1.25	0.96	0.86
TIMM17B	TIMM17B;DXS9822;TIM17B	translocase of inner mitochondrial membrane 17 homolog B (yeast)	1.25	1.19	0.96	0.99
PHF6	PHF6;BFLS;BORJ;CENP-31;MGC14797	PHD finger protein 6	1.23	1.13	0.96	0.82
C9orf5	C9orf5;CG-2;CG2;FLJ23668;FLJ33224	chromosome 9 open reading frame 5	1.23	1.14	0.96	0.89
BMI1	BMI1;FLVI2/BMI1;MGC12685;PCGF4;RNF51	BMI1 polycomb ring finger oncogene	1.23	1.13	0.96	0.81
POLDIP3	POLDIP3;KIAA1649;PDIP46;SKAR	polymerase (DNA-directed), delta interacting protein 3	1.21	1.12	0.96	0.81
PPFIBP2	PPFIBP2;Cclp1;DKFZp781K06126;MGC42541	PTPRF interacting protein, binding protein 2 (liprin beta 2)	1.2	1.07	0.96	0.81
KCND1	KCND1;KV4.1	potassium voltage-gated channel, Shal-related subfamily, member 1	1.18	1.12	0.96	0.87
GPR55	GPR55	G protein-coupled receptor 55	1.17	1.04	0.96	0.79
RNF144A	RNF144A;KIAA0161;RNF144;UBCE7IP4	ring finger protein 144A	1.17	1.08	0.96	0.78
CAMKK2	CAMKK2;CAMKK;CAMKKB;KIAA0787;MGC15254	calcium/calmodulin-dependent protein kinase kinase 2, beta	1.17	1.12	0.96	0.93
ZRSR2	ZRSR2;MGC142014;MGC142040;U2AF1-RS2;U2AF1L2;U2AF1RS2;URP	zinc finger (CCCH type), RNA-binding motif and serine/arginine rich 2	1.17	1	0.96	0.78
IMP5	IMP5;SPPL2c	intramembrane protease 5	1.16	1.1	0.96	0.83
CHCHD6	CHCHD6;MGC13016;PPP1R23	coiled-coil-helix-coiled-coil-helix domain containing 6	1.16	1.15	0.96	0.79
PPM1D	PPM1D;PP2C-DELTA;WIP1	protein phosphatase, Mg2+/Mn2+ dependent, 1D	1.15	1.08	0.96	0.85
HSD11B1	HSD11B1;11-DH;11-beta-HSD1;HDL;HSD11;HSD11B;HSD11L;MGC13539;SDR26C1	hydroxysteroid (11-beta) dehydrogenase 1	1.14	1.05	0.96	0.78
BRCA1	BRCA1;BRCAI;BRCC1;BROVCA1;IRIS;PNCA4;PPP1R53;PSCP;RNF53	breast cancer 1, early onset	1.13	1.04	0.96	0.75
PHF20	PHF20;C20orf104;FLJ33479;GLEA2;HCA58;NZF;TZP	PHD finger protein 20	1.13	1.04	0.96	0.75
TRIM42	TRIM42;FLJ40097;MGC163346;PPP1R40	tripartite motif containing 42	1.12	1.04	0.96	0.75
CXCL2	CXCL2;CINC-2a;GRO2;GROb;MGSA-b;MIP-2a;MIP2;MIP2A;SCYB2	chemokine (C-X-C motif) ligand 2	1.12	1.03	0.96	0.75
PLCG1	PLCG1;NCKAP3;PLC-II;PLC1;PLC148;PLCgamma1	phospholipase C, gamma 1	1.12	1.14	0.96	0.75
ATG14	ATG14;ATG14L;BARKOR;KIAA0831;MGC126291;MGC126292	ATG14 autophagy related 14 homolog (S. cerevisiae)	1.12	0.97	0.96	0.8
DFNB31	DFNB31;CIP98;DKFZp434N014;KIAA1526;USH2D;WHRN;WI	deafness, autosomal recessive 31	1.11	0.96	0.96	0.8
HEXIM1	HEXIM1;CLP1;EDG1;FLJ13562;HIS1;MAQ1	hexamethylene bis-acetamide inducible 1	1.11	1.01	0.96	0.82
GOLGA8E	GOLGA8E	golgin A8 family, member E	1.1	1.05	0.96	0.76
FBXO25	FBXO25;FBX25;MGC20256;MGC51975	F-box protein 25	1.1	1.01	0.96	0.73
C11orf88	C11orf88;FLJ46266	chromosome 11 open reading frame 88	1.08	1.08	0.96	0.82
B3GAT2	B3GAT2;GLCATS;KIAA1963;MGC138535	beta-1,3-glucuronyltransferase 2 (glucuronosyltransferase S)	1.08	1.06	0.96	0.84
RNF150	RNF150;MGC125502	ring finger protein 150	1.08	1	0.96	0.72
SSH2	SSH2;KIAA1725;MGC78588;SSH-2	slingshot homolog 2 (Drosophila)	1.08	0.97	0.96	0.73
HIST1H1T	HIST1H1T;H1FT;H1t;MGC163222;dJ221C16.2	histone cluster 1, H1t	1.07	0.98	0.96	0.83
DUSP13	DUSP13;BEDP;DUSP13A;DUSP13B;FLJ32450;MDSP;SKRP4;TMDP	dual specificity phosphatase 13	1.07	0.94	0.96	0.71
SYTL3	SYTL3;MGC105130;MGC118883;MGC118884;MGC118885;SLP3	synaptotagmin-like 3	1.07	1.02	0.96	0.91
ZNF8	ZNF8;HF.18;Zfp128	zinc finger protein 8	1.07	1.05	0.96	0.8
NFYA	NFYA;CBF-A;CBF-B;FLJ11236;HAP2;NF-YA	nuclear transcription factor Y, alpha	1.07	1.05	0.96	0.83
PTER	PTER;RPR-1	phosphotriesterase related	1.07	1.01	0.96	0.71
B3GALNT2	B3GALNT2;B3GalNAc-T2;MGC39558	beta-1,3-N-acetylgalactosaminyltransferase 2	1.07	1.02	0.96	0.76
PROSER1	PROSER1;C13orf23;FLJ12661;FLJ23780;bA50D16.2	proline and serine rich 1	1.07	1.06	0.96	0.81
GRIK2	GRIK2;EAA4;GLR6;GLUK6;GLUR6;MGC74427;MRT6	glutamate receptor, ionotropic, kainate 2	1.06	1.01	0.96	0.79

EIF5A2	EIF5A2;EIF-5A2;eIF5AII	eukaryotic translation initiation factor 5A2	1.06	1	0.96	0.7
CCNI2	CCNI2;FLJ16793	cyclin I family, member 2	1.06	1.04	0.96	0.79
USP11	USP11;UHX1	ubiquitin specific peptidase 11	1.06	0.94	0.96	0.7
SLFN5	SLFN5;MGC150611;MGC150612;MGC19764	schlafen family member 5	1.05	0.93	0.96	0.82
UBE2W	UBE2W;FLJ11011;UBC16;hUBC-16	ubiquitin-conjugating enzyme E2W (putative)	1.05	0.94	0.96	0.7
ARL5A	ARL5A;ARFLP5;ARL5	ADP-ribosylation factor-like 5A	1.05	0.98	0.96	0.72
ROBO2	ROBO2;KIAA1568;SAX3	roundabout, axon guidance receptor, homolog 2 (Drosophila)	1.04	0.9	0.96	0.72
MYL6B	MYL6B;MLC1SA	myosin, light chain 6B, alkali, smooth muscle and non-muscle	1.04	1.08	0.96	0.73
MRPS22	MRPS22;C3orf5;COXPD5;GIBT;MRP-S22;RPMS22	mitochondrial ribosomal protein S22	1.04	1.06	0.96	0.74
BMPR2	BMPR2;BMPR-II;BMPR3;BMR2;BRK-3;FLJ41585;FLJ76945;PPH1;T-ALK	bone morphogenetic protein receptor, type II (serine/threonine kinase)	1.04	1.08	0.96	0.77
RRBP1	RRBP1;DKFZp586A1420;ES/130;ES130;FLJ36146;MGC157720;MGC157721;RRp;hES	ribosome binding protein 1 homolog 180kDa (dog)	1.03	0.94	0.96	0.68
PDCD6IP	PDCD6IP;AIP1;Alix;DRIP4;HP95;MGC17003	programmed cell death 6 interacting protein	1.03	0.97	0.96	0.73
PPM1F	PPM1F;CAMKP;CaMKPase;FEM-2;KIAA0015;POPX2;hFEM-2	protein phosphatase, Mg2+/Mn2+ dependent, 1F	1.02	0.91	0.96	0.68
ATP5G2	ATP5G2;ATP5A	ATP synthase, H+ transporting, mitochondrial Fo complex, subunit C2 (subunit 9)	1.02	0.91	0.96	0.81
ITGA3	ITGA3;CD49C;FLJ34631;FLJ34704;GAP-B3;GAPB3;MSK18;VCA-2;VL3A;VLA3a	integrin, alpha 3 (antigen CD49C, alpha 3 subunit of VLA-3 receptor)	1.02	1	0.96	0.79
RNF14	RNF14;ARA54;FLJ26004;HFB30;TRIAD2	ring finger protein 14	1.02	0.96	0.96	0.86
SDC4	SDC4;MGC22217;SYND4	syndecan 4	1.02	1	0.96	0.69
KPNA2	KPNA2;IPOA1;QIP2;RCH1;SRP1alpha	karyopherin alpha 2 (RAG cohort 1, importin alpha 1)	1.02	0.92	0.96	0.73
SIM2	SIM2;HMC13F06;HMC29C01;MGC119447;SIM;bHLHe15	single-minded homolog 2 (Drosophila)	1.01	0.96	0.96	0.7
MAGOHB	MAGOHB;FLJ10292;MGN2;mago;magoh	mago-nashi homolog B (Drosophila)	1.01	1	0.96	0.68
OR4F5	OR4F5	olfactory receptor, family 4, subfamily F, member 5	1.01	1.01	0.96	0.67
PPP1R1B	PPP1R1B;DARPP-32;DARPP32;FLJ20940	protein phosphatase 1, regulatory (inhibitor) subunit 1B	1	0.89	0.96	0.67
CRAMP1L	CRAMP1L;HN1L;MGC176736;TCE4	Crm, cramped-like (Drosophila)	1	1	0.96	0.67
FABP5	FABP5;E-FABP;EFABP;PA-FABP;PAFABP	fatty acid binding protein 5 (psoriasis-associated)	1	0.94	0.96	0.73
FNBP1	FNBP1;FBP17;KIAA0554;MGC126804	formin binding protein 1	1	0.92	0.96	0.67
SHROOM3	SHROOM3;APXL3;KIAA1481;SHRM;ShrmL	shroom family member 3	1	1.03	0.96	0.72
ARRDC3	ARRDC3;KIAA1376;TLIMP	arrestin domain containing 3	1	0.99	0.96	0.75
TMEM147	TMEM147;MGC1936;NIFIE14	transmembrane protein 147	1	1.04	0.96	0.79
PRDM1	PRDM1;BLIMP1;MGC118922;MGC118923;MGC118924;MGC118925;PRDI-BF1	PR domain containing 1, with ZNF domain	0.99	1.02	0.96	0.77
C21orf7	C21orf7;HC21ORF7;TAK1L;TAKL;TAKL-1;TAKL-2;TAKL-4	chromosome 21 open reading frame 7	0.99	0.9	0.96	0.76
HOPX	HOPX;CAMEO;HOD;HOP;LAGY;MGC20820;NECC1;OB1;SMAP31;TOTO	HOP homeobox	0.99	0.92	0.96	0.75
HCFC2	HCFC2;FLJ94012;HCF-2;HCF2	host cell factor C2	0.99	0.94	0.96	0.73
BICD2	BICD2;KIAA0699;bA526D8.1	bicaudal D homolog 2 (Drosophila)	0.99	0.97	0.96	0.67
EIF2C3	EIF2C3;AGO3;FLJ12765;MGC86946	eukaryotic translation initiation factor 2C, 3	0.99	0.92	0.96	0.68
UBR3	UBR3;DKFZp434P117;DKFZp686N10185;FLJ37422;KIAA2024;MGC126489;ZNF650	ubiquitin protein ligase E3 component n-recognin 3 (putative)	0.99	0.94	0.96	0.84
ARPC4	ARPC4;ARC20;MGC13544;P20-ARC	actin related protein 2/3 complex, subunit 4, 20kDa	0.99	0.92	0.96	0.66
SMG9	SMG9;C19orf61;DKFZp564H1322;F17127_1;FLJ12886	smg-9 homolog, nonsense mediated mRNA decay factor (C. elegans)	0.99	0.95	0.96	0.69
SDR42E1	SDR42E1	short chain dehydrogenase/reductase family 42E, member 1	0.99	0.92	0.96	0.68
ASTN1	ASTN1;ASTN;KIAA1747	astrotactin 1	0.98	0.94	0.96	0.68
BEST2	BEST2;FLJ20132;VMD2L1	bestrophin 2	0.98	0.88	0.96	0.72
NUDT10	NUDT10;APS2;DIPP3a;hDIPP3alpha	nudix (nucleoside diphosphate linked moiety X)-type motif 10	0.98	0.91	0.96	0.71
ZSWIM1	ZSWIM1;C20orf162	zinc finger, SWIM-type containing 1	0.98	0.88	0.96	0.75
RNASEH2C	RNASEH2C;AGS3;AYP1;FLJ20974;MGC22934	ribonuclease H2, subunit C	0.98	0.92	0.96	0.69
COL4A2	COL4A2;DKFZp686I14213;FLJ22259	collagen, type IV, alpha 2	0.98	0.92	0.96	0.7
WASH5P	WASH5P;FLJ98676	WAS protein family homolog 5 pseudogene	0.98	0.97	0.96	0.67
C4orf40	C4orf40	chromosome 4 open reading frame 40	0.97	0.96	0.96	0.73
RAB41	RAB41	RAB41, member RAS oncogene family	0.97	0.92	0.96	0.64
ZNF605	ZNF605	zinc finger protein 605	0.97	0.98	0.96	0.79
QPCTL	QPCTL;FLJ20084;gQC	glutaminyl-peptide cyclotransferase-like	0.97	0.88	0.96	0.72
UCHL1	UCHL1;PARK5;PGP 9.5;PGP9.5;PGP95;Uch-L1	ubiquitin carboxyl-terminal esterase L1 (ubiquitin thiolesterase)	0.97	0.91	0.96	0.69
GATSL3	GATSL3	GATS protein-like 3	0.97	0.96	0.96	0.73
TAS2R42	TAS2R42;T2R24;T2R55;TAS2R55;hT2R55	taste receptor, type 2, member 42	0.96	1	0.96	0.66
PENK	PENK	proenkephalin	0.96	0.94	0.96	0.65
ARSD	ARSD;ASD	arylsulfatase D	0.96	0.86	0.96	0.75
CRY1	CRY1;PHLL1	cryptochrome 1 (photolyase-like)	0.96	0.88	0.96	0.8
HPCAL1	HPCAL1;BDR1;HLP2;VILIP-3	hippocalcin-like 1	0.96	0.96	0.96	0.72
STT3B	STT3B;FLJ90106;SIMP;STT3-B	STT3, subunit of the oligosaccharyltransferase complex, homolog B (S. cerevisiae)	0.96	0.97	0.96	0.75
C5orf39	C5orf39;AX2R;AXIIR	chromosome 5 open reading frame 39	0.95	0.86	0.96	0.7
HHIPL2	HHIPL2;FLJ13840;KIAA1822L	HHIP-like 2	0.95	0.94	0.96	0.7
TRPC5	TRPC5;TRP5	transient receptor potential cation channel, subfamily C, member 5	0.94	0.88	0.96	0.69
CACNA1A	CACNA1A;APCA;BI;CACNL1A4;CAV2.1;EA2;FHM;HPCA;MHP;MHP1;SCA6	calcium channel, voltage-dependent, P/Q type, alpha 1A subunit	0.94	0.96	0.96	0.75
AQP5	AQP5	aquaporin 5	0.94	0.88	0.96	0.62
MSLN	MSLN;MPF;SMRP	mesothelin	0.94	0.92	0.96	0.7
TMEM126B	TMEM126B;MGC111203	transmembrane protein 126B	0.94	0.86	0.96	0.69
C9orf40	C9orf40;FLJ10110;FLJ25795	chromosome 9 open reading frame 40	0.94	0.9	0.96	0.62
ID2	ID2;GIG8;ID2A;ID2H;MGC26389;bHLHb26	inhibitor of DNA binding 2, dominant negative helix-loop-helix protein	0.94	0.88	0.96	0.62
EPB41L1	EPB41L1;4.1N;DKFZp686H17242;KIAA0338;MGC11072;MRD11	erythrocyte membrane protein band 4.1-like 1	0.94	0.96	0.96	0.63
CEBPG	CEBPG;GPE1BP;IG/EBP-1	CCAAT/enhancer binding protein (C/EBP), gamma	0.94	0.88	0.96	0.62
CDNF	CDNF;ARMETL1	cerebral dopamine neurotrophic factor	0.93	0.88	0.96	0.66
BTNL2	BTNL2;BTL-II;HSBLMHC1;SS2	butyrophilin-like 2 (MHC class II associated)	0.92	0.86	0.96	0.67
ERG	ERG;erg-3;p55	v-ets erythroblastosis virus E26 oncogene homolog (avian)	0.92	0.86	0.96	0.64
SLC2A9	SLC2A9;GLUT9;GLUTX;UAQTL2;URATv1	solute carrier family 2 (facilitated glucose transporter), member 9	0.92	0.93	0.96	0.72
LIN52	LIN52;C14orf46;c14_5549	lin-52 homolog (C. elegans)	0.92	0.92	0.96	0.66
BECN1	BECN1;ATG6;VPS30;beclin1	beclin 1, autophagy related	0.92	0.96	0.96	0.72
UBE2N	UBE2N;MGC131857;MGC8489;UBC13;UbcH-ben	ubiquitin-conjugating enzyme E2N	0.92	0.8	0.96	0.6
USP19	USP19;ZMYND9	ubiquitin specific peptidase 19	0.92	0.9	0.96	0.6
CDK15	CDK15;ALS2CR7;PFTAIRE2;PFTK2	cyclin-dependent kinase 15	0.92	0.88	0.96	0.73
CNKSR2	CNKSR2;CNK2;KIAA0902;KSR2;MAGUIN	connector enhancer of kinase suppressor of Ras 2	0.91	0.92	0.96	0.68
KBTBD7	KBTBD7;DKFZp434E2318	kelch repeat and BTB (POZ) domain containing 7	0.91	0.86	0.96	0.72
CP	CP;CP-2	ceruloplasmin (ferroxidase)	0.91	0.95	0.96	0.71
RGS19	RGS19;GAIP;RGSGAIP	regulator of G-protein signaling 19	0.91	0.84	0.96	0.63
RBMS2	RBMS2;FLJ39093;FLJ40023;FLJ43262;SCR3	RNA binding motif, single stranded interacting protein 2	0.91	0.92	0.96	0.75
MET	MET;AUTS9;HGFR;RCCP2;c-Met	met proto-oncogene (hepatocyte growth factor receptor)	0.91	0.94	0.96	0.66
IL24	IL24;C49A;FISP;IL10B;MDA7;MOB5;ST16	interleukin 24	0.9	0.82	0.96	0.63
ENTPD3	ENTPD3;CD39L3;FLJ93839;HB6;NTPDase-3	ectonucleoside triphosphate diphosphohydrolase 3	0.9	0.81	0.96	0.64
KRTAP9-3	KRTAP9-3;KAP9.3;KRTAP9.3	keratin associated protein 9-3	0.9	0.89	0.96	0.67
LY6G6C	LY6G6C;C6orf24;G6c;NG24	lymphocyte antigen 6 complex, locus G6C	0.9	0.94	0.96	0.69
C15orf27	C15orf27;FLJ30304;FLJ38190	chromosome 15 open reading frame 27	0.9	0.92	0.96	0.64
IFT140	IFT140;DKFZp564L232;FLJ10306;FLJ30571;KIAA0590;WDTC2;c305C8.4;c380F5.1;gs114	intraflagellar transport 140 homolog (Chlamydomonas)	0.9	0.9	0.96	0.68
FABP3	FABP3;FABP11;H-FABP;M-FABP;MDGI;O-FABP	fatty acid binding protein 3, muscle and heart (mammary-derived growth inhibitor)	0.9	0.84	0.96	0.62
REM1	REM1;GD:REM;GES;MGC48669;REM	RAS (RAD and GEM)-like GTP-binding 1	0.9	0.84	0.96	0.72
PAXIP1	PAXIP1;CAGF29;FLJ41049;PACIP1;PAXIP1L;PTIP;TNRC2	PAX interacting (with transcription-activation domain) protein 1	0.9	0.82	0.96	0.63
ZBTB9	ZBTB9;MGC23166;ZNF919	zinc finger and BTB domain containing 9	0.9	0.82	0.96	0.6
AKTIP	AKTIP;FT1;FTS	AKT interacting protein	0.9	0.79	0.96	0.59
C19orf22	C19orf22;MGC16353	chromosome 19 open reading frame 22	0.9	0.84	0.96	0.63
PHLDA3	PHLDA3;TIH1	pleckstrin homology-like domain, family A, member 3	0.9	0.84	0.96	0.68
TGS1	TGS1;DKFZp762A163;FLJ22995;NCOA6IP;PIMT;PIPMT	trimethylguanosine synthase 1	0.9	0.86	0.96	0.64
IER3	IER3;DIF-2;DIF2;GLY96;IEX-1;IEX-1L;IEX1;PRG1	immediate early response 3	0.9	0.9	0.96	0.64
HECTD1	HECTD1;FLJ38315;KIAA1131	HECT domain containing 1	0.9	0.79	0.96	0.59
CLEC18B	CLEC18B;MRCL2	C-type lectin domain family 18, member B	0.9	0.84	0.96	0.64
VN1R2	VN1R2;V1RL2	vomeronasal 1 receptor 2	0.89	0.84	0.96	0.75
SLC25A25	SLC25A25;KIAA1896;MCSC;MGC105138;MGC119514;MGC119515;MGC119516;MGC119517;PCSCL;RP11-395P17.4;SCAMC-2	solute carrier family 25 (mitochondrial carrier; phosphate carrier), member 25	0.89	0.84	0.96	0.61
POLR3D	POLR3D;BN51T;RPC4;RPC53;TSBN51	polymerase (RNA) III (DNA directed) polypeptide D, 44kDa	0.89	0.78	0.96	0.69
ABHD12	ABHD12;ABHD12A;BEM46L2;C20orf22;DKFZp434P106;PHARC;dJ965G21.2	abhydrolase domain containing 12	0.89	0.9	0.96	0.64
PRNP	PRNP;ASCR;CD230;CJD;GSS;MGC26679;PRIP;PrP;PrP27-30;PrP33-35C;PrPc;prion	prion protein	0.89	0.88	0.96	0.59
AMPH	AMPH;AMPH1	amphiphysin	0.88	0.88	0.96	0.63
DHFRL1	DHFRL1;DHFRP4;FLJ16119	dihydrofolate reductase-like 1	0.88	0.85	0.96	0.58
C9orf37	C9orf37	chromosome 9 open reading frame 37	0.88	0.83	0.96	0.62
C6orf70	C6orf70;FLJ11152;dJ266L20.3	chromosome 6 open reading frame 70	0.88	0.89	0.96	0.61
MGLL	MGLL;HU-K5;HUK5;MAGL;MGL	monoglyceride lipase	0.88	0.86	0.96	0.68
SRM	SRM;PAPT;SPDSY;SPS1;SRML1	spermidine synthase	0.88	0.84	0.96	0.62
IGF2-AS	IGF2-AS;IGF2AS;MGC168198;PEG8	insulin-like growth factor 2 antisense (non-protein coding)	0.88	0.88	0.96	0.62
C3orf36	C3orf36;FLJ22173;MGC125760;MGC125761	chromosome 3 open reading frame 36	0.87	0.83	0.96	0.6
AQP6	AQP6;AQP2L;KID	aquaporin 6, kidney specific	0.87	0.84	0.96	0.56
GCNT4	GCNT4;C2GNT3	glucosaminyl (N-acetyl) transferase 4, core 2	0.87	0.88	0.96	0.59
TRIM9	TRIM9;KIAA0282;RNF91;SPRING	tripartite motif containing 9	0.86	0.79	0.96	0.6
ABCC8	ABCC8;ABC36;HHF1;HI;HRINS;MRP8;PHHI;SUR;SUR1;SUR1delta2;TNDM2	ATP-binding cassette, sub-family C (CFTR/MRP), member 8	0.86	0.82	0.96	0.65
COL27A1	COL27A1;FLJ11895;KIAA1870;MGC11337	collagen, type XXVII, alpha 1	0.86	0.79	0.96	0.61
C14orf101	C14orf101;FLJ20392	chromosome 14 open reading frame 101	0.86	0.79	0.96	0.61
KCNK15	KCNK15;K2p15.1;KCNK11;KCNK14;KIAA0237;KT3.3;TASK-5;TASK5;dJ781B1.1	potassium channel, subfamily K, member 15	0.86	0.88	0.96	0.69
MTCH2	MTCH2;MIMP	mitochondrial carrier 2	0.86	0.77	0.96	0.66
TMED7	TMED7;FLJ57776;FLJ90481	transmembrane emp24 protein transport domain containing 7	0.86	0.88	0.96	0.61
SLC17A2	SLC17A2;MGC138238;NPT3	solute carrier family 17 (sodium phosphate), member 2	0.85	0.84	0.96	0.58
ZDHHC23	ZDHHC23;MGC42530;NIDD	zinc finger, DHHC-type containing 23	0.85	0.82	0.96	0.56
TYRP1	TYRP1;CAS2;CATB;GP75;TRP;TYRP;b-PROTEIN	tyrosinase-related protein 1	1.32	1.31	0.97	1.04
ARFIP2	ARFIP2;FLJ18046;FLJ18697;FLJ99239;POR1	ADP-ribosylation factor interacting protein 2	1.31	1.27	0.97	0.89
NAALADL2	NAALADL2	N-acetylated alpha-linked acidic dipeptidase-like 2	1.26	1.21	0.97	0.91
GRIA1	GRIA1;GLUH1;GLUR1;GLURA;GluA1;HBGR1;MGC133252	glutamate receptor, ionotropic, AMPA 1	1.23	1.24	0.97	0.96
FAM150A	FAM150A;MGC163513;MGC163515;UNQ9433	family with sequence similarity 150, member A	1.22	1.07	0.97	0.89

SIRPA	SIRPA;BIT;CD172A;MFR;MYD-1;P84;PTPNS1;SHPS1;SIRP	signal-regulatory protein alpha	1.22	1.17	0.97	0.91
NTRK3	NTRK3;TRKC;gp145(trkC)	neurotrophic tyrosine kinase, receptor, type 3	1.2	1.24	0.97	0.89
CLMN	CLMN;FLJ12383;FLJ43048;KIAA0500;KIAA1188	calmin (calponin-like, transmembrane)	1.2	1.19	0.97	0.94
C14orf102	C14orf102;FLJ10008;FLJ14051	chromosome 14 open reading frame 102	1.19	1.23	0.97	0.86
PHF11	PHF11;APY;BCAP;IGEL;IGER;IGHER;NY-REN-34;NYREN34	PHD finger protein 11	1.19	1.1	0.97	0.8
PRPF19	PRPF19;NMP200;PRP19;PSO4;SNEV;UBOX4;hPSO4	PRP19/PSO4 pre-mRNA processing factor 19 homolog (S. cerevisiae)	1.19	1.1	0.97	0.79
C10orf46	C10orf46;FLJ40409;MGC33215	chromosome 10 open reading frame 46	1.17	1.04	0.97	0.78
KRT39	KRT39;CK-39;K39;KA35	keratin 39	1.15	1.09	0.97	0.82
TSR1	TSR1;FLJ10534;KIAA1401;MGC131829	TSR1, 20S rRNA accumulation, homolog (S. cerevisiae)	1.15	1.21	0.97	0.89
GOT1	GOT1;GIG18	glutamic-oxaloacetic transaminase 1, soluble (aspartate aminotransferase 1)	1.15	1.18	0.97	0.91
CXADR	CXADR;CAR;CAR4/6;HCAR	coxsackie virus and adenovirus receptor	1.15	1.08	0.97	0.98
MLL	MLL;ALL-1;CXXC7;FLJ11783;HRX;HTRX1;KMT2A;MLL/GAS7;MLL1A;TET1-MLL;TRX1	myeloid/lymphoid or mixed-lineage leukemia (trithorax homolog, Drosophila)	1.14	1.04	0.97	0.82
CARD18	CARD18;ICEBERG;UNQ5804;pseudo-ICE	caspase recruitment domain family, member 18	1.14	1.09	0.97	0.82
HEATR5B	HEATR5B;KIAA1414	HEAT repeat containing 5B	1.13	1.18	0.97	0.91
APOA5	APOA5;APOAV;FLJ97995;MGC126836;MGC126838;RAP3	apolipoprotein A-V	1.12	1.07	0.97	0.86
INTS12	INTS12;INT12;PHF22	integrator complex subunit 12	1.12	1.04	0.97	0.75
STRN	STRN;MGC125642;SG2NA	striatin, calmodulin binding protein	1.12	1.06	0.97	0.89
RRP1B	RRP1B;KIAA0179;NNP1L;Nnp1;RRP1	ribosomal RNA processing 1 homolog B (S. cerevisiae)	1.12	1.19	0.97	0.87
LRFN5	LRFN5;C14orf146;DKFZp686G0210;FIGLER8;FLJ30803;SALM5	leucine rich repeat and fibronectin type III domain containing 5	1.09	1.06	0.97	0.72
UBL4A	UBL4A;DX254E;DXS254E;G6PD;GDX;GET5;MDY2;TMA24;UBL4	ubiquitin-like 4A	1.09	1.08	0.97	0.86
NMD3	NMD3;FLJ21053	NMD3 homolog (S. cerevisiae)	1.09	1.05	0.97	0.76
PTPRD	PTPRD;HPTP;HPTPD;HPTPDELTA;MGC119750;MGC119751;MGC119752;MGC119753;PTPD;RPTPDELTA	protein tyrosine phosphatase, receptor type, D	1.08	0.98	0.97	0.73
NLRP1	NLRP1;CARD7;CLR17.1;DEFCAP;DEFCAP-L/S;DKFZp586O1822;KIAA0926;NAC;NALP1;PP1044;SLEV1;VAMAS1	NLR family, pyrin domain containing 1	1.08	1.06	0.97	0.76
ARHGAP4	ARHGAP4;C1;KIAA0131;RGC1;RhoGAP4;SrGAP4;p115	Rho GTPase activating protein 4	1.08	1.1	0.97	0.78
PSMB8	PSMB8;D6S216;D6S216E;JMP;LMP7;MGC1491;NKJO;PSMB5i;RING10	proteasome (prosome, macropain) subunit, beta type, 8 (large multifunctional peptidase 7)	1.08	0.97	0.97	0.73
SGSM3	SGSM3;DKFZp761D051;MAP;RUSC3;RUTBC3	small G protein signaling modulator 3	1.08	0.94	0.97	0.79
CX3CL1	CX3CL1;ABCD-3;C3Xkine;CXC3;CXC3C;NTN;NTT;SCYD1;fractalkine;neurotactin	chemokine (C-X3-C motif) ligand 1	1.08	1.01	0.97	0.77
COX8A	COX8A;COX;COX8;COX8-2;COX8L;VIII;VIII-L	cytochrome c oxidase subunit VIIIA (ubiquitous)	1.08	1.02	0.97	0.8
OOEP	OOEP;C6orf156;FLOPED;HOEP19;KHDC2	oocyte expressed protein homolog (dog)	1.08	1.15	0.97	0.84
DPP7	DPP7;DPP2;DPPII;QPP	dipeptidyl-peptidase 7	1.07	0.97	0.97	0.72
FAM40A	FAM40A;FLJ14743;KIAA1761;MGC148091;RP4-773N10.1	family with sequence similarity 40, member A	1.06	1.05	0.97	0.73
ABT1	ABT1;hABT1	activator of basal transcription 1	1.06	1	0.97	0.72
DYRK1A	DYRK1A;DYRK;DYRK1;HP86;MNB;MNBH;MRD7	dual-specificity tyrosine-(Y)-phosphorylation regulated kinase 1A	1.06	1.12	0.97	0.79
ZNF22	ZNF22;HKR-T1;KOX15;ZNF422;Zfp422	zinc finger protein 22 (KOX 15)	1.05	1	0.97	0.78
DTD1	DTD1;C20orf88;DUEB;HARS2;MGC119131;MGC41905;bA379J5.3;bA555E18.1;pqn-68	D-tyrosyl-tRNA deacylase 1 homolog (S. cerevisiae)	1.05	1.01	0.97	0.73
RAB22A	RAB22A;MGC16770	RAB22A, member RAS oncogene family	1.05	1.01	0.97	0.75
CNTNAP2	CNTNAP2;AUTS15;CASPR2;CDFE;DKFZp781D1846;NRXN4;PTHSL1	contactin associated protein-like 2	1.04	1.02	0.97	0.68
ZCCHC6	ZCCHC6;DKFZp666B142;DKFZp686C11112;DKFZp686F119;DKFZp686I1269;PAPD6	zinc finger, CCHC domain containing 6	1.04	0.96	0.97	0.74
AKR1B15	AKR1B15;AKR1B10L	aldo-keto reductase family 1, member B15	1.04	1.05	0.97	0.75
ARL9	ARL9	ADP-ribosylation factor-like 9	1.03	0.97	0.97	0.69
METTL6	METTL6;MGC24132	methyltransferase like 6	1.03	0.93	0.97	0.74
OTP	OTP;MGC3161	orthopedia homeobox	1.02	1.04	0.97	0.77
KCNJ2	KCNJ2;ATFB9;HHBIRK1;HHIRK1;IRK1;KIR2.1;LQT7;SQT3	potassium inwardly-rectifying channel, subfamily J, member 2	1.02	0.99	0.97	0.77
CHRNA2	CHRNA2	cholinergic receptor, nicotinic, alpha 2 (neuronal)	1.02	0.99	0.97	0.77
ZNF175	ZNF175;OTK18	zinc finger protein 175	1.02	0.88	0.97	0.73
IRF2	IRF2;DKFZp686F0244;IRF-2	interferon regulatory factor 2	1.02	0.92	0.97	0.74
RAB3D	RAB3D;D2-2;GOV;RAB16;RAD3D	RAB3D, member RAS oncogene family	1.02	1.02	0.97	0.81
PDCL3	PDCL3;HTPHLP;MGC3062;PHLP2A;PHLP3;VIAF;VIAF1	phosducin-like 3	1.02	0.89	0.97	0.71
SS18	SS18;MGC116875;SSXT;SYT	synovial sarcoma translocation, chromosome 18	1.02	0.98	0.97	0.73
DCAF12L1	DCAF12L1;KIAA1892L;WDR40B	DDB1 and CUL4 associated factor 12-like 1	1.02	0.97	0.97	0.73
KRTAP17-1	KRTAP17-1;KAP17.1;KRTAP16.1;KRTAP17.1	keratin associated protein 17-1	1.01	1.02	0.97	0.76
SH2B1	SH2B1;DKFZp547G1110;FLJ30542;KIAA1299;PSM;SH2B	SH2B adaptor protein 1	1.01	1.03	0.97	0.73
RAB5A	RAB5A;RAB5	RAB5A, member RAS oncogene family	1.01	1.04	0.97	0.69
OR4S1	OR4S1;OR11-100	olfactory receptor, family 4, subfamily S, member 1	1	1.02	0.97	0.72
CPNE9	CPNE9;KIAA4217	copine family member IX	1	1.06	0.97	0.71
DNAI2	DNAI2;CILD9	dynein, axonemal, intermediate chain 2	1	0.87	0.97	0.73
PRAMEF7	PRAMEF7;MGC178110	PRAME family member 7	1	0.94	0.97	0.74
C12orf51	C12orf51;DKFZp586O1022;FLJ10510;FLJ30092;FLJ30208;FLJ34154;KIAA0614;MGC126531	chromosome 12 open reading frame 51	1	0.88	0.97	0.67
COL9A2	COL9A2;DJ39G22.4;EDM2;MED;STL5	collagen, type IX, alpha 2	1	1	0.97	0.76
ARMCX6	ARMCX6;FLJ20811	armadillo repeat containing, X-linked 6	1	0.94	0.97	0.7
FAM83D	FAM83D;C20orf129;CHICA;FLJ38341;dJ616B8.3	family with sequence similarity 83, member D	1	1.01	0.97	0.73
SEC24B	SEC24B;MGC48822;SEC24	SEC24 family, member B (S. cerevisiae)	1	0.96	0.97	0.79
TUBB8	TUBB8;FLJ40100;bA631M21.2	tubulin, beta 8 class VIII	1	1.04	0.97	0.81
UQCR11	UQCR11;0710008D09Rik;QCR10;UQCR	ubiquinol-cytochrome c reductase, complex III subunit XI	1	0.95	0.97	0.74
LPIN1	LPIN1;DKFZp781P1796;KIAA0188;PAP1	lipin 1	0.99	0.97	0.97	0.66
TLN1	TLN1;ILWEQ;KIAA1027;TLN	talin 1	0.99	0.91	0.97	0.77
GPRC5A	GPRC5A;GPCR5A;RAI3;RAIG1	G protein-coupled receptor, family C, group 5, member A	0.99	0.94	0.97	0.84
IGLL3P	IGLL3P;16.1;IGLL3	immunoglobulin lambda-like polypeptide 3, pseudogene	0.99	1.03	0.97	0.79
NDRG2	NDRG2;DKFZp781G1938;FLJ25522;KIAA1248;SYLD	NDRG family member 2	0.98	0.97	0.97	0.77
MLLT1	MLLT1;ENL;LTG19;YEATS1	myeloid/lymphoid or mixed-lineage leukemia (trithorax homolog, Drosophila); translocated to, 1	0.98	1.03	0.97	0.77
TP53BP2	TP53BP2;53BP2;ASPP2;BBP;P53BP2;PPP1R13A	tumor protein p53 binding protein, 2	0.98	0.9	0.97	0.76
MRPL12	MRPL12;5c5-2;FLJ60124;L12mt;MGC8610;MRP-L31/34;MRPL7;MRPL7/L12;RPML12	mitochondrial ribosomal protein L12	0.98	0.98	0.97	0.67
TRHDE	TRHDE;FLJ22381;PAP-II;PGPEP2;TRH-DE	thyrotropin-releasing hormone degrading enzyme	0.98	0.88	0.97	0.66
CENPBD1	CENPBD1;FLJ23771;FLJ31569;MGC13198;MGC16385	CENPB DNA-binding domains containing 1	0.98	0.98	0.97	0.66
OVCH1	OVCH1;OVCH	ovochymase 1	0.97	0.93	0.97	0.7
KIF2B	KIF2B;FLJ53902	kinesin family member 2B	0.97	0.92	0.97	0.79
SPRR4	SPRR4;MGC138375;MGC138377	small proline-rich protein 4	0.97	0.94	0.97	0.65
CLCN6	CLCN6;CLC-6;KIAA0046	chloride channel 6	0.97	0.94	0.97	0.73
NAB2	NAB2;MADER;MGC75085	NGFI-A binding protein 2 (EGR1 binding protein 2)	0.97	0.9	0.97	0.65
RNMT	RNMT;DKFZp686H1252;KIAA0398;MET;RG7MT1;hCMT1c	RNA (guanine-7-) methyltransferase	0.97	0.92	0.97	0.72
ALG9	ALG9;CDG1L;DIBD1;DKFZp586M2420;FLJ21845;LOH11CR1J	asparagine-linked glycosylation 9, alpha-1,2-mannosyltransferase homolog (S. cerevisiae)	0.97	0.96	0.97	0.72
URGCP	URGCP;DKFZp666G166;DKFZp686O0457;URG4	upregulator of cell proliferation	0.97	0.94	0.97	0.64
NDUFS7	NDUFS7;CI-20;CI-20KD;FLJ45860;FLJ46880;MGC120002;MY017;PSST	NADH dehydrogenase (ubiquinone) Fe-S protein 7, 20kDa (NADH-coenzyme Q reductase)	0.96	0.87	0.97	0.69
CHST6	CHST6;MCDC1	carbohydrate (N-acetylglucosamine 6-O) sulfotransferase 6	0.96	0.96	0.97	0.76
PLEKHG5	PLEKHG5;DSMA4;GEF720;KIAA0720	pleckstrin homology domain containing, family G (with RhoGef domain) member 5	0.96	0.96	0.97	0.72
PDP2	PDP2;KIAA1348;PPM2C2	pyruvate dehyrogenase phosphatase catalytic subunit 2	0.96	0.92	0.97	0.72
TMED1	TMED1;IL1RL1LG;Il1rl1l;MGC1270;ST2L	transmembrane emp24 protein transport domain containing 1	0.96	0.9	0.97	0.65
POLQ	POLQ;DKFZp781A0112;POLH;PRO0327	polymerase (DNA directed), theta	0.96	0.92	0.97	0.68
DMXL1	DMXL1;DKFZp779O1239;FLJ44772	Dmx-like 1	0.96	0.97	0.97	0.66
PRKX	PRKX;PKX1	protein kinase, X-linked	0.96	1.01	0.97	0.71
ZP3	ZP3;ZP3A;ZP3B;ZPC;Zp-3	zona pellucida glycoprotein 3 (sperm receptor)	0.95	0.97	0.97	0.68
SH3D19	SH3D19;EBP;EVE1;Kryn;MGC105136;MGC118910;MGC118911;MGC118912;MGC118913;SH3P19	SH3 domain containing 19	0.95	0.9	0.97	0.75
SERPINA12	SERPINA12;OL-64	serpin peptidase inhibitor, clade A (alpha-1 antiproteinase, antitrypsin), member 12	0.94	0.89	0.97	0.72
GPR83	GPR83;GIR;GPR72	G protein-coupled receptor 83	0.94	0.88	0.97	0.79
ZNF667	ZNF667;DKFZp686O111;FLJ14011;FLJ45518;MIPU1	zinc finger protein 667	0.94	0.82	0.97	0.64
KRT74	KRT74;HTSS2;K6IRS4;KRT5C;KRT6IRS4	keratin 74	0.94	0.93	0.97	0.69
TMEM135	TMEM135;DKFZp686I1974;FLJ22104;PMP52	transmembrane protein 135	0.94	0.97	0.97	0.68
BRF2	BRF2;BRFU;FLJ11052;TFIIIB50	BRF2, subunit of RNA polymerase III transcription initiation factor, BRF1-like	0.94	0.92	0.97	0.62
FZD3	FZD3;Fz-3	frizzled family receptor 3	0.94	0.84	0.97	0.63
INO80D	INO80D;FLJ20309	INO80 complex subunit D	0.94	0.94	0.97	0.67
COQ6	COQ6;FLJ51173;FLJ53037;FLJ58904;FLJ79255	coenzyme Q6 homolog, monooxygenase (S. cerevisiae)	0.94	0.86	0.97	0.68
ZIM2	ZIM2;ZNF656	zinc finger, imprinted 2	0.94	0.94	0.97	0.63
CD300LB	CD300LB;CD300b;CLM-7;CLM7;CMRF35-A2;IREM-3;IREM3;TREM-5;TREM5	CD300 molecule-like family member b	0.93	0.94	0.97	0.68
MLLT3	MLLT3;AF9;FLJ2035;YEATS3	myeloid/lymphoid or mixed-lineage leukemia (trithorax homolog, Drosophila); translocated to, 3	0.93	0.92	0.97	0.64
IFT122	IFT122;CED;SPG;WDR10;WDR10p;WDR140	intraflagellar transport 122 homolog (Chlamydomonas)	0.93	0.85	0.97	0.72
TTYH3	TTYH3;KIAA1691	tweety homolog 3 (Drosophila)	0.93	0.9	0.97	0.7
OR1D2	OR1D2;MGC119942;MGC119943;OLFR1;OR17-4	olfactory receptor, family 1, subfamily D, member 2	0.92	0.87	0.97	0.66
OR52A4	OR52A4	olfactory receptor, family 52, subfamily A, member 4	0.92	0.94	0.97	0.69
TCL1B	TCL1B;SYN-1;TML1	T-cell leukemia/lymphoma 1B	0.92	0.8	0.97	0.64
SALL2	SALL2;FLJ10414;FLJ55746;HSAL2;KIAA0360;Sal-2;ZNF795;p150(Sal2)	sal-like 2 (Drosophila)	0.92	0.86	0.97	0.68
CHRD	CHRD;MGC133038	chordin	0.92	0.86	0.97	0.69
FXYD7	FXYD7;FLJ25096	FXYD domain containing ion transport regulator 7	0.92	0.94	0.97	0.73
NPM3	NPM3;PORMIN;TMEM123	nucleophosmin/nucleoplasmin 3	0.92	0.92	0.97	0.7
MDFIC	MDFIC;HIC	MyoD family inhibitor domain containing	0.92	0.97	0.97	0.74
SIRT5	SIRT5;FLJ36950;SIR2L5	sirtuin 5	0.92	0.88	0.97	0.61
ARMC10	ARMC10;FLJ37850;MGC3195;PNAS-112;PNAS112;SVH	armadillo repeat containing 10	0.92	0.87	0.97	0.65
GTF3C3	GTF3C3;TFIIIC102;TFIIICgamma;TFiiiC2-102	general transcription factor IIIC, polypeptide 3, 102kDa	0.92	0.96	0.97	0.72
RAF1	RAF1;CRAF;NS5;Raf-1;c-Raf	v-raf-1 murine leukemia viral oncogene homolog 1	0.92	0.95	0.97	0.68
LTN1	LTN1;C21orf10;C21orf98;FLJ11053;KIAA0714;RNF160;ZNF294	listerin E3 ubiquitin protein ligase 1	0.92	0.88	0.97	0.79
ZNF137P	ZNF137P;MGC119990;MGC119991;ZNF137;pHZ-30	zinc finger protein 137, pseudogene	0.92	0.9	0.97	0.63
TIGIT	TIGIT;DKFZp667A205;FLJ39873;VSIG9;VSTM3;WUCAM	T cell immunoreceptor with Ig and ITIM domains	0.92	0.92	0.97	0.69
COL16A1	COL16A1;447AA	collagen, type XVI, alpha 1	0.91	0.89	0.97	0.62
TYMP	TYMP;ECGF;ECGF1;MEDPS1;MNGIE;MTDPS1;PDECGF;TP;hPD-ECGF	thymidine phosphorylase	0.91	0.88	0.97	0.69
EBP	EBP;CDPX2;CHO2;CPX;CPXD	emopamil binding protein (sterol isomerase)	0.91	0.84	0.97	0.61
TSSK2	TSSK2;DGS-G;FLJ38613;SPOGA2;STK22B;TSK2	testis-specific serine kinase 2	0.9	0.94	0.97	0.67
ATP5G3	ATP5G3;MGC125738;P3	ATP synthase, H+ transporting, mitochondrial Fo complex, subunit C3 (subunit 9)	0.9	0.94	0.97	0.7
RHOF	RHOF;ARHF;FLJ20247;RIF	ras homolog gene family, member F (in filopodia)	0.9	0.87	0.97	0.68
TMEM87B	TMEM87B;FLJ14681	transmembrane protein 87B	0.9	0.84	0.97	0.65
SOAT1	SOAT1;ACACT;ACAT;ACAT1;RP11-215I23.2;SOAT;STAT	sterol O-acyltransferase 1	0.9	0.81	0.97	0.65

IL20	IL20;IL-20;IL10D;MGC96907;ZCYTO10	interleukin 20	0.89	0.82	0.97	0.6
LNX1	LNX1;LNX;MPDZ;PDZRN2	ligand of numb-protein X 1	0.89	0.85	0.97	0.76
AKNA	AKNA;FLJ31001;FLJ33184;KIAA1968;RP11-82I1.4	AT-hook transcription factor	0.89	0.84	0.97	0.63
ZNF354A	ZNF354A;EZNF;HKL1;KID-1;KID1;TCF17	zinc finger protein 354A	0.89	0.86	0.97	0.64
CCNJ	CCNJ;bA690P14.1	cyclin J	0.89	0.84	0.97	0.66
LPIN2	LPIN2;KIAA0249	lipin 2	0.89	0.82	0.97	0.69
FAM102A	FAM102A;C9orf132;EEIG1;MGC50853;bA203J24.7	family with sequence similarity 102, member A	0.89	0.9	0.97	0.65
NOMO2	NOMO2;Nomo;PM5	NODAL modulator 2	0.89	0.82	0.97	0.6
RAPGEF4	RAPGEF4;CAMP-GEFII;CGEF2;EPAC2;Nbla00496	Rap guanine nucleotide exchange factor (GEF) 4	0.88	0.84	0.97	0.67
CCIN	CCIN	calicin	0.88	0.81	0.97	0.69
OR6V1	OR6V1;GPR138	olfactory receptor, family 6, subfamily V, member 1	0.88	0.88	0.97	0.67
CD300A	CD300A;CLM-8;CMRF-35-H9;CMRF-35H;CMRF35-H;CMRF35-H9;CMRF35H;CMRF35H9;IGSF12;IRC1;IRC1/IRC2;IRC2;IRp60	CD300a molecule	0.88	0.82	0.97	0.66
BSN	BSN;ZNF231	bassoon (presynaptic cytomatrix protein)	0.88	0.88	0.97	0.63
PFKFB4	PFKFB4	6-phosphofructo-2-kinase/fructose-2,6-biphosphatase 4	0.88	0.8	0.97	0.6
TAF5	TAF5;TAF2D;TAFII100	TAF5 RNA polymerase II, TATA box binding protein (TBP)-associated factor, 100kDa	0.88	0.84	0.97	0.59
KHDRBS3	KHDRBS3;Etle;SALP;SLM-2;SLM2;T-STAR;TSTAR;etoile	KH domain containing, RNA binding, signal transduction associated 3	0.88	0.89	0.97	0.67
TGFBR1	TGFBR1;AAT5;ACVRLK4;ALK-5;ALK5;LDS1A;LDS2A;MSSE;SKR4;TGFR-1	transforming growth factor, beta receptor 1	0.88	0.85	0.97	0.71
UBP1	UBP1;DKFZp686L1745;LBP-1B;LBP-1a;LBP1A;LBP1B	upstream binding protein 1 (LBP-1a)	0.88	0.86	0.97	0.58
CAMSAP2	CAMSAP2;CAMSAP1L1;KIAA1078;MGC150680;MGC150681	calmodulin regulated spectrin-associated protein family, member 2	0.88	0.88	0.97	0.66
TRIM34	TRIM34;IFP1;RNF21	tripartite motif containing 34	0.88	0.83	0.97	0.67
TAS2R1	TAS2R1;MGC126778;MGC126780;T2R1;TRB7	taste receptor, type 2, member 1	0.87	0.88	0.97	0.6
FGF10	FGF10	fibroblast growth factor 10	0.87	0.86	0.97	0.66
DCC	DCC;CRC18;CRCR1;IGDCC1	deleted in colorectal carcinoma	0.87	0.81	0.97	0.58
NPBWR1	NPBWR1;GPR7;MGC129755	neuropeptides B/W receptor 1	0.87	0.82	0.97	0.74
CHCHD8	CHCHD8;DKFZp762H1711;E2IG2;MGC117206	coiled-coil-helix-coiled-coil-helix domain containing 8	0.87	0.83	0.97	0.62
C1orf220	C1orf220;DKFZp564J047;FLJ35530;FLJ40199	chromosome 1 open reading frame 220	0.86	0.79	0.97	0.67
PAPPA	PAPPA;ASBABP2;DIPLA1;IGFBP-4ase;PAPA;PAPP-A;PAPPA1	pregnancy-associated plasma protein A, pappalysin 1	0.86	0.76	0.97	0.58
SLC30A3	SLC30A3;ZNT3	solute carrier family 30 (zinc transporter), member 3	0.86	0.78	0.97	0.69
LONRF2	LONRF2;FLJ45273;MGC126711;MGC126713;RNF192	LON peptidase N-terminal domain and ring finger 2	0.86	0.82	0.97	0.73
PACSIN1	PACSIN1;KIAA1379;SDPI	protein kinase C and casein kinase substrate in neurons 1	0.86	0.9	0.97	0.64
MAL	MAL	mal, T-cell differentiation protein	0.86	0.82	0.97	0.66
TBC1D3B	TBC1D3B;FLJ78399;PRC17;TBC1D3I	TBC1 domain family, member 3B	0.86	0.84	0.97	0.6
PHIP	PHIP;DCAF14;FLJ20705;FLJ45918;MGC90216;WDR11;ndrp	pleckstrin homology domain interacting protein	0.86	0.88	0.97	0.65
LDLR	LDLR;FH;FHC;LDLCQ2	low density lipoprotein receptor	0.86	0.78	0.97	0.63
PPFIBP1	PPFIBP1;L2;SGT2;hSGT2;hSgt2p	PTPRF interacting protein, binding protein 1 (liprin beta 1)	0.86	0.88	0.97	0.71
SLPI	SLPI;ALK1;ALP;BLPI;HUSI;HUSI-I;MPI;WAP4;WFDC4	secretory leukocyte peptidase inhibitor	0.86	0.81	0.97	0.57
NDUFAF3	NDUFAF3;2P1;C3orf60;DKFZp564J0123;E3-3;MGC10527	NADH dehydrogenase (ubiquinone) 1 alpha subcomplex, assembly factor 3	0.86	0.9	0.97	0.69
SCARB1	SCARB1;CD36L1;CLA-1;CLA1;HDLQTL6;MGC138242;SR-BI;SRB1	scavenger receptor class B, member 1	0.85	0.78	0.97	0.62
TM9SF3	TM9SF3;EP70-P-iso;SMBP	transmembrane 9 superfamily member 3	0.85	0.84	0.97	0.63
KBTBD12	KBTBD12;DKFZp779E2271;FLJ46299;KLHDC6	kelch repeat and BTB (POZ) domain containing 12	0.85	0.79	0.97	0.6
FAM86C1	FAM86C1;FAM86C;FLJ10661;FLJ27199;MGC45068	family with sequence similarity 86, member C1	0.85	0.82	0.97	0.59
ZNF622	ZNF622;MGC17552;MGC2485;ZPR9	zinc finger protein 622	1.49	1.6	0.98	1.17
MTMR9	MTMR9;C8orf9;DKFZp434K171;LIP-STYX;MGC126672;MTMR8	myotubularin related protein 9	1.31	1.28	0.98	1
COL15A1	COL15A1;FLJ38566	collagen, type XV, alpha 1	1.28	1.14	0.98	0.9
GEN1	GEN1;DKFZp781F0986;FLJ36312;FLJ40869;Gen	Gen homolog 1, endonuclease (Drosophila)	1.21	1.16	0.98	0.82
FBXO22	FBXO22;FBX22;FISTC1;FLJ13986;MGC31799	F-box protein 22	1.2	1.12	0.98	0.81
NME4	NME4;NDPK-D;NM23H4;nm23-H4	non-metastatic cells 4, protein expressed in	1.19	1.14	0.98	1.02
EPHA2	EPHA2;ARCC2;ECK	EPH receptor A2	1.18	1.25	0.98	0.89
TRIM22	TRIM22;GPSTAF50;RNF94;STAF50	tripartite motif containing 22	1.17	1.09	0.98	0.79
IL34	IL34;C16orf77;IL-34;MGC34647	interleukin 34	1.17	1.02	0.98	0.79
CISD1	CISD1;C10orf70;MGC14684;ZCD1;mitoNEET	CDGSH iron sulfur domain 1	1.17	1.24	0.98	0.95
ZNF592	ZNF592;CAMOS;KIAA0211;MGC138437;MGC138439;SCAR5	zinc finger protein 592	1.15	1.08	0.98	0.78
FRAS1	FRAS1;DKFZp686I05113;DKFZp686P08111;FLJ14927;FLJ22031;KIAA1500	Fraser syndrome 1	1.13	1	0.98	0.83
COL5A3	COL5A3	collagen, type V, alpha 3	1.12	1.06	0.98	0.81
CDCA2	CDCA2;FLJ25804;MGC129906;MGC129907;Repo-Man	cell division cycle associated 2	1.12	1.06	0.98	0.84
SLC15A2	SLC15A2;FLJ33407;PEPT2	solute carrier family 15 (H+/peptide transporter), member 2	1.1	1.09	0.98	0.73
JMY	JMY;FLJ37870;MGC163496;WHDC1L3	junction mediating and regulatory protein, p53 cofactor	1.1	1.02	0.98	0.86
RSL1D1	RSL1D1;CSIG;DKFZP564M182;MGC138433;MGC142259;PBK1;UTP30	ribosomal L1 domain containing 1	1.1	1.16	0.98	0.81
SEC61A1	SEC61A1;HSEC61;SEC61;SEC61A	Sec61 alpha 1 subunit (S. cerevisiae)	1.1	1.04	0.98	0.82
38047	MARCH4;MARCH-IV;MGC104908;RNF174	membrane-associated ring finger (C3HC4) 4	1.09	1.02	0.98	0.74
HIST1H4C	HIST1H4C;H4/g;H4FG;dJ221C16.1	histone cluster 1, H4c	1.09	1.1	0.98	0.84
HOXC8	HOXC8;HOX3;HOX3A	homeobox C8	1.08	1.04	0.98	0.79
TNFSF12-TNF	TNFSF12-TNFSF13;TWE-PRIL	TNFSF12-TNFSF13 readthrough	1.08	1.09	0.98	0.83
ANO7	ANO7;D-TMPP;DTMPP;IPCA-5;IPCA5;NGEP;PCANAP5;PCANAP5L;TMEM16G	anoctamin 7	1.07	1.08	0.98	0.82
PCGF3	PCGF3;DKFZp686D20235;DONG1;FLJ36550;FLJ43813;MGC129615;MGC40413;RNF3;RNF3A	polycomb group ring finger 3	1.07	1	0.98	0.73
MRPL16	MRPL16;FLJ20484;L16mt;MRP-L16	mitochondrial ribosomal protein L16	1.07	1.08	0.98	0.82
RASSF3	RASSF3;MGC119194;MGC119195;MGC119197;RASSF5	Ras association (RalGDS/AF-6) domain family member 3	1.06	1.02	0.98	0.8
EEF1B2	EEF1B2;EEF1B;EEF1B1;EF1B	eukaryotic translation elongation factor 1 beta 2	1.06	1.1	0.98	0.86
USP8	USP8;FLJ34456;HumORF8;KIAA0055;MGC129718;UBPY	ubiquitin specific peptidase 8	1.06	1.02	0.98	0.77
SPRYD5P	SPRYD5P	SPRY domain containing 5 pseudogene	1.06	1	0.98	0.72
KCNS1	KCNS1;KV9.1	potassium voltage-gated channel, delayed-rectifier, subfamily S, member 1	1.05	1.02	0.98	0.8
CRYBA4	CRYBA4;MCOPCT4	crystallin, beta A4	1.05	1	0.98	0.76
ZMYND11	ZMYND11;BRAM1;BS69;MGC111056	zinc finger, MYND-type containing 11	1.05	0.98	0.98	0.71
PCNX	PCNX;FLJ23409;FLJ45663;KIAA0805;KIAA0995;PCNXL1	pecanex homolog (Drosophila)	1.04	1.07	0.98	0.8
HIST4H4	HIST4H4;H4/p;HIST1H4A;HIST1H4B;HIST1H4C;HIST1H4D;HIST1H4E;HIST1H4F;HIST1H4H;HIST1H4I;HIST1H4J;HIST1H4K;HIST1H4L;HIST2H4A;HIST2H4B;MGC24116	histone cluster 4, H4	1.03	1.02	0.98	0.69
SESN3	SESN3;MGC29667;SEST3	sestrin 3	1.03	1.1	0.98	0.81
MBOAT2	MBOAT2;FLJ14415;FLJ90298;LPCAT4;OACT2	membrane bound O-acyltransferase domain containing 2	1.03	0.9	0.98	0.7
KEL	KEL;CD238;ECE3	Kell blood group, metallo-endopeptidase	1.02	0.98	0.98	0.74
BVES	BVES;HBVES;MGC42413;POP1;POPDC1	blood vessel epicardial substance	1.02	1.02	0.98	0.77
NKG7	NKG7;GIG1;GMP-17;p15-TIA-1	natural killer cell group 7 sequence	1.02	0.99	0.98	0.69
DLEU7	DLEU7;FLJ44882;MGC138214	deleted in lymphocytic leukemia, 7	1.02	0.91	0.98	0.72
ENDOU	ENDOU;MGC133268;P11;PP11;PRSS26	endonuclease, polyU-specific	1.02	0.92	0.98	0.69
PDCD10	PDCD10;CCM3;MGC1212;MGC24477;TFAR15	programmed cell death 10	1.01	1.04	0.98	0.84
ZNF816	ZNF816;MGC125619;ZNF816A	zinc finger protein 816	1.01	1.04	0.98	0.74
C6orf97	C6orf97;FLJ23305;bA282P11.1	chromosome 6 open reading frame 97	1	1	0.98	0.73
DUSP26	DUSP26;DUSP24;LDP-4;MGC1136;MGC2627;MKP8;NATA1;SKRP3	dual specificity phosphatase 26 (putative)	1	1.08	0.98	0.79
NLRC3	NLRC3;CLR16.2;FLJ00348;NOD3	NLR family, CARD domain containing 3	1	0.98	0.98	0.71
GRK1	GRK1;GPRK1;RHOK;RK	G protein-coupled receptor kinase 1	1	0.94	0.98	0.85
SLC35F3	SLC35F3;FLJ37712	solute carrier family 35, member F3	1	0.94	0.98	0.69
TMEM59L	TMEM59L;BSMAP;C19orf4	transmembrane protein 59-like	1	0.96	0.98	0.72
ATXN7L1	ATXN7L1;ATXN7L4;FLJ40255;FLJ58242;KIAA1218;MGC10760;MGC33190	ataxin 7-like 1	1	1.01	0.98	0.75
RNASEH2B	RNASEH2B;AGS2;DLEU8;FLJ11712	ribonuclease H2, subunit B	1	1.04	0.98	0.73
OXR1	OXR1;FLJ10125;FLJ38829;FLJ40849;FLJ41673;FLJ42450;FLJ45656	oxidation resistance 1	1	0.97	0.98	0.8
NOMO1	NOMO1;NOMO3;Nomo;PM5	NODAL modulator 1	1	0.93	0.98	0.75
AK5	AK5;AK6;MGC33326	adenylate kinase 5	0.99	1.04	0.98	0.74
C16orf86	C16orf86	chromosome 16 open reading frame 86	0.99	1	0.98	0.76
RNF126	RNF126;FLJ20552;MGC1022;MGC14317	ring finger protein 126	0.99	0.96	0.98	0.86
MEOX1	MEOX1;MOX1	mesenchyme homeobox 1	0.99	0.99	0.98	0.76
TROVE2	TROVE2;RO60;RORNP;SSA2	TROVE domain family, member 2	0.99	1.02	0.98	0.73
PHLDB2	PHLDB2;DKFZp313O2433;DKFZp434G227;DKFZp686J05113;FLJ21791;LL5b;LL5beta	pleckstrin homology-like domain, family B, member 2	0.99	0.94	0.98	0.71
TTC30B	TTC30B;FLJ30990;IFT70;fleer	tetratricopeptide repeat domain 30B	0.98	1.04	0.98	0.79
DUOX1	DUOX1;LNOX1;MGC138840;MGC138841;NOXEF1;THOX1	dual oxidase 1	0.98	1.02	0.98	0.77
CHGA	CHGA;CGA	chromogranin A (parathyroid secretory protein 1)	0.98	0.93	0.98	0.73
RYR1	RYR1;CCO;MHS;MHS1;RYDR;RYR;RYR-1;SKRR	ryanodine receptor 1 (skeletal)	0.98	1.02	0.98	0.79
BACH2	BACH2	BTB and CNC homology 1, basic leucine zipper transcription factor 2	0.98	0.94	0.98	0.69
RBMS1	RBMS1;C2orf12;DKFZp564H0764;HCC-4;MGC15146;MGC3331;MGC70597;MGC97258;MGC97270;MGC97282;MGC99543;MSSP;MSSP-1;MSSP-2;MSSP-3;SCR2;YC1	RNA binding motif, single stranded interacting protein 1	0.98	0.87	0.98	0.69
LRP8	LRP8;APOER2;HSZ75190;LRP-8;MCI1	low density lipoprotein receptor-related protein 8, apolipoprotein e receptor	0.98	1.05	0.98	0.78
CELSR1	CELSR1;CDHF9;DKFZp434P0729;FMI2;HFMI2;ME2	cadherin, EGF LAG seven-pass G-type receptor 1 (flamingo homolog, Drosophila)	0.98	0.94	0.98	0.84
ACAT1	ACAT1;ACAT;MAT;T2;THIL	acetyl-CoA acetyltransferase 1	0.98	1.02	0.98	0.77
NAP1L4	NAP1L4;MGC4565;NAP1L4b;NAP2;NAP2L;hNAP2	nucleosome assembly protein 1-like 4	0.98	0.91	0.98	0.77
CTDSP2	CTDSP2;OS4;PSR2;SCP2	CTD (carboxy-terminal domain, RNA polymerase II, polypeptide A) small phosphatase 2	0.98	0.89	0.98	0.67
TMEM215	TMEM215;DKFZp686I2292;FLJ43330	transmembrane protein 215	0.97	0.92	0.98	0.72
FAAH	FAAH;FAAH-1;MGC102823;MGC138146	fatty acid amide hydrolase	0.97	0.88	0.98	0.7
ADRBK2	ADRBK2;BARK2;GRK3	adrenergic, beta, receptor kinase 2	0.97	1.02	0.98	0.73
KCNH2	KCNH2;ERG1;HERG;HERG1;Kv11.1;LQT2;SQT1	potassium voltage-gated channel, subfamily H (eag-related), member 2	0.97	1.02	0.98	0.73
ELL	ELL;C19orf17;DKFZp434I1916;ELL1;MEN;PPP1R68	elongation factor RNA polymerase II	0.97	1	0.98	0.74
CCDC50	CCDC50;C3orf6;DFNA44;YMER	coiled-coil domain containing 50	0.97	0.94	0.98	0.7
TMEM179B	TMEM179B	transmembrane protein 179B	0.97	0.98	0.98	0.74
NSUN5	NSUN5;FLJ10267;MGC986;NOL1;NOL1R;NSUN5A;WBSCR20;WBSCR20A;p120	NOP2/Sun domain family, member 5	0.97	0.88	0.98	0.7
HSPA1A	HSPA1A;FLJ54303;FLJ54370;FLJ54392;FLJ54408;FLJ75127;HSP70-1;HSP70-1A;HSP70I;HSP72;HSPA1;HSPA1B	heat shock 70kDa protein 1A	0.97	0.9	0.98	0.65
S100A10	S100A10;42C;ANX2L;ANX2LG;CAL1L;CLP11;Ca[1];GP11;MGC111133;P11;p10	S100 calcium binding protein A10	0.97	0.93	0.98	0.77
KRT15	KRT15;CK15;K15;K1CO	keratin 15	0.96	0.92	0.98	0.77
ANKRD13B	ANKRD13B;FLJ20418;FLJ25555	ankyrin repeat domain 13B	0.96	0.92	0.98	0.69
TBC1D3F	TBC1D3F;FLJ56187;FLJ58835;TBC1D3	TBC1 domain family, member 3F	0.96	0.97	0.98	0.73
KIAA0922	KIAA0922;DKFZp586H1322;FLJ10592;TMEM131L	KIAA0922	0.96	0.97	0.98	0.66
PATZ1	PATZ1;MAZR;PATZ;RIAZ;ZBTB19;ZNF278;ZSG;dJ400N23	POZ (BTB) and AT hook containing zinc finger 1	0.96	0.94	0.98	0.84
FHL3	FHL3;MGC19547;MGC23614;MGC8696;SLIM2	four and a half LIM domains 3	0.96	0.94	0.98	0.64
NDUFA10	NDUFA10;CI-42KD;CI-42k;FLJ21923;MGC5103	NADH dehydrogenase (ubiquinone) 1 alpha subcomplex, 10, 42kDa	0.96	0.91	0.98	0.78
GGNBP2	GGNBP2;DIF-3;DIF3;FLJ21230;FLJ22561;FLJ42090;LCRG1;LZK1;ZFP403;ZNF403	gametogenetin binding protein 2	0.96	0.88	0.98	0.74

TPR	TPR	translocated promoter region (to activated MET oncogene)	0.96	0.99	0.98	0.69
ALPK3	ALPK3;FLJ12881;FLJ21176;KIAA1330;MAK;MIDORI	alpha-kinase 3	0.95	0.92	0.98	0.77
PLEKHH2	PLEKHH2;KIAA2028;PLEKHH1L	pleckstrin homology domain containing, family H (with MyTH4 domain) member 2	0.95	1.01	0.98	0.67
DENND4A	DENND4A;FLJ33949;IRLB;KIAA0476;MYCPBP	DENN/MADD domain containing 4A	0.95	1.01	0.98	0.78
LZIC	LZIC;MGC15436	leucine zipper and CTNNBIP1 domain containing	0.95	0.84	0.98	0.7
OR7G3	OR7G3;OST085	olfactory receptor, family 7, subfamily G, member 3	0.94	0.93	0.98	0.65
CSPG4	CSPG4;HMW-MAA;MCSP;MCSPG;MEL-CSPG;MSK16;NG2	chondroitin sulfate proteoglycan 4	0.94	0.9	0.98	0.73
DDR2	DDR2;MIG20a;NTRKR3;TKT;TYRO10	discoidin domain receptor tyrosine kinase 2	0.94	0.98	0.98	0.7
TMEM130	TMEM130;DKFZp761L1417;FLJ42643	transmembrane protein 130	0.94	0.86	0.98	0.7
FAM120C	FAM120C;CXorf17;FLJ20506;FLJ43676;ORF34	family with sequence similarity 120C	0.94	0.88	0.98	0.68
FANCG	FANCG;FAG;XRCC9	Fanconi anemia, complementation group G	0.94	0.92	0.98	0.67
HBEGF	HBEGF;DTR;DTS;DTSF;HEGFL	heparin-binding EGF-like growth factor	0.94	0.94	0.98	0.75
KIAA1598	KIAA1598;DKFZp686A0439;MGC40476	KIAA1598	0.94	0.9	0.98	0.68
CCDC116	CCDC116;FLJ36046	coiled-coil domain containing 116	0.93	0.98	0.98	0.68
ZNF417	ZNF417;MGC34079	zinc finger protein 417	0.93	0.9	0.98	0.65
PCGF5	PCGF5;MGC16202;RNF159	polycomb group ring finger 5	0.93	0.9	0.98	0.81
CLIP1	CLIP1;CLIP;CLIP-170;CLIP170;CYLN1;MGC131604;RSN	CAP-GLY domain containing linker protein 1	0.93	0.88	0.98	0.67
IP6K1	IP6K1;IHPK1;MGC9925;PiUS	inositol hexakisphosphate kinase 1	0.93	0.98	0.98	0.7
NPSR1	NPSR1;ASRT2;GPR154;GPRA;NPSR;PGR14;VRR1	neuropeptide S receptor 1	0.92	0.88	0.98	0.79
ENPP2	ENPP2;ATX;ATX-X;AUTOTAXIN;FLJ26803;LysoPLD;NPP2;PD-IALPHA;PDNP2	ectonucleotide pyrophosphatase/phosphodiesterase 2	0.92	0.93	0.98	0.74
VMO1	VMO1;ERGA6350;MGC125880;MGC125881;PRO21055	vitelline membrane outer layer 1 homolog (chicken)	0.92	0.98	0.98	0.72
FAM40B	FAM40B	family with sequence similarity 40, member B	0.92	0.9	0.98	0.61
IFIT3	IFIT3;CIG-49;GARG-49;IFI60;IFIT4;IRG2;ISG60;RIG-G	interferon-induced protein with tetratricopeptide repeats 3	0.92	0.88	0.98	0.65
ARHGEF15	ARHGEF15;ARGEF15;E5;Ephexin5;FLJ13791;KIAA0915;MGC44868;Vsm-RhoGEF	Rho guanine nucleotide exchange factor (GEF) 15	0.92	0.92	0.98	0.66
STXBP5	STXBP5;FLJ30922;LGL3;LLGL3;MGC141942;MGC141968;Nbla04300	syntaxin binding protein 5 (tomosyn)	0.92	0.88	0.98	0.7
KIFAP3	KIFAP3;FLJ22818;KAP-3;KAP3;SMAP;Smg-GDS;dJ190I16.1	kinesin-associated protein 3	0.92	0.87	0.98	0.64
RAVER2	RAVER2;DKFZp762D1011;FLJ10770;KIAA1579	ribonucleoprotein, PTB-binding 2	0.92	0.95	0.98	0.68
GPS2	GPS2;AMF-1;MGC104294;MGC119287;MGC119288;MGC119289	G protein pathway suppressor 2	0.92	0.86	0.98	0.62
PDXK	PDXK;C21orf124;C21orf97;DKFZp566A071;FLJ31940;FLJ37311;MGC15873;MGC31754;MGC52346;PKH;PNK	pyridoxal (pyridoxine, vitamin B6) kinase	0.92	0.9	0.98	0.75
JAK1	JAK1;JAK1A;JAK1B;JTK3	Janus kinase 1	0.92	0.96	0.98	0.69
AIMP2	AIMP2;JTV-1;JTV1;P38	aminoacyl tRNA synthetase complex-interacting multifunctional protein 2	0.92	0.86	0.98	0.66
C16orf93	C16orf93;MGC104706	chromosome 16 open reading frame 93	0.92	0.88	0.98	0.66
RAB11FIP1	RAB11FIP1;DKFZp686E2214;FLJ22524;FLJ22622;MGC78448;NOEL1A;RCP;rab11-FIP1	RAB11 family interacting protein 1 (class I)	0.91	0.86	0.98	0.66
DEK	DEK;D6S231E	DEK oncogene	0.91	0.98	0.98	0.68
MPEG1	MPEG1;MGC132657;MGC138435;MPG1;MPS1;Mpg-1	macrophage expressed 1	0.9	0.9	0.98	0.68
ANGPTL4	ANGPTL4;ANGPTL2;ARP4;FIAF;HFARP;NL2;PGAR;pp1158	angiopoietin-like 4	0.9	0.92	0.98	0.61
SLC10A7	SLC10A7;C4orf13;DKFZp313H0531;DKFZp566M114;DKFZp779O2438;MGC25043;P7	solute carrier family 10 (sodium/bile acid cotransporter family), member 7	0.9	0.94	0.98	0.7
ZFAT	ZFAT;AITD3;KIAA1485;MGC126815;MGC126817;ZFAT1;ZNF406	zinc finger and AT hook domain containing	0.9	0.94	0.98	0.72
SYT17	SYT17	synaptotagmin XVII	0.9	0.88	0.98	0.63
FBXO11	FBXO11;FBX11;FLJ12673;MGC44383;PRMT9;UBR6;VIT1	F-box protein 11	0.9	0.84	0.98	0.61
LRP6	LRP6;ADCAD2;FLJ90062;FLJ90421	low density lipoprotein receptor-related protein 6	0.9	0.82	0.98	0.72
FIBP	FIBP;FGFIBP;FIBP-1	fibroblast growth factor (acidic) intracellular binding protein	0.9	0.85	0.98	0.67
NPLOC4	NPLOC4;FLJ20657;FLJ23742;KIAA1499;NPL4	nuclear protein localization 4 homolog (S. cerevisiae)	0.9	0.86	0.98	0.65
EIF1	EIF1;A121;EIF-1;EIF1A;ISO1;SUI1	eukaryotic translation initiation factor 1	0.9	0.98	0.98	0.67
KRT222	KRT222;KA21;KRT222P;MGC45562	keratin 222	0.9	0.85	0.98	0.65
DSCR9	DSCR9;NCRNA00038	Down syndrome critical region gene 9 (non-protein coding)	0.89	0.9	0.98	0.6
TMEM140	TMEM140;FLJ11000	transmembrane protein 140	0.89	0.9	0.98	0.67
MSX2	MSX2;CRS2;FPP;HOX8;MSH;PFM;PFM1	msh homeobox 2	0.89	0.86	0.98	0.63
BIN1	BIN1;AMPH2;AMPHL;DKFZp547F068;MGC10367;SH3P9	bridging integrator 1	0.89	0.9	0.98	0.68
FLJ37396	FLJ37396	transmembrane protein FLJ37396	0.88	0.9	0.98	0.6
CHRNA7	CHRNA7;CHRNA7-2;NACHRA7	cholinergic receptor, nicotinic, alpha 7	0.88	0.84	0.98	0.66
DMRTC2	DMRTC2	DMRT-like family C2	0.88	0.86	0.98	0.58
PCDHB16	PCDHB16;KIAA1621;ME1;PCDH-BETA16;PCDH3X;PCDHB8a	protocadherin beta 16	0.88	0.89	0.98	0.67
FAM84A	FAM84A;FLJ35392;NSE1;PP11517	family with sequence similarity 84, member A	0.88	0.78	0.98	0.61
ARMC5	ARMC5;FLJ00019;FLJ13063	armadillo repeat containing 5	0.88	0.83	0.98	0.63
MYO5B	MYO5B;KIAA1119	myosin VB	0.88	0.94	0.98	0.7
ITGB4	ITGB4;CD104	integrin, beta 4	0.88	0.91	0.98	0.6
GMIP	GMIP;ARHGAP46	GEM interacting protein	0.88	0.84	0.98	0.66
BANP	BANP;BEND1;DKFZp761H172;FLJ10177;FLJ20538;SMAR1;SMARBP1	BTG3 associated nuclear protein	0.88	0.88	0.98	0.67
PPL	PPL;KIAA0568;MGC134872	periplakin	0.88	0.88	0.98	0.69
CPD	CPD;GP180	carboxypeptidase D	0.88	0.86	0.98	0.64
MAZ	MAZ;PUR1;Pur-1;SAF-1;SAF-2;SAF-3;ZF87;ZNF801;Zif87	MYC-associated zinc finger protein (purine-binding transcription factor)	0.88	0.84	0.98	0.65
TMEM154	TMEM154;FLJ32028	transmembrane protein 154	0.87	0.9	0.98	0.63
SBSN	SBSN;MGC75533;UNQ698	suprabasin	0.87	0.88	0.98	0.65
FBXO6	FBXO6;FBG2;FBS2;FBX6;Fbx6b	F-box protein 6	0.87	0.82	0.98	0.59
RPL36A	RPL36A;L36A;L44L;MGC72020;MIG6;RPL44	ribosomal protein L36a	0.87	0.86	0.98	0.58
DDX21	DDX21;DKFZp686F21172;GUA;GURDB;RH-II/GU;RH-II/GuA	DEAD (Asp-Glu-Ala-Asp) box polypeptide 21	0.87	0.9	0.98	0.59
ADAM22	ADAM22;ADAM 22;MDC2;MGC149832	ADAM metallopeptidase domain 22	0.86	0.83	0.98	0.62
SCG2	SCG2;CHGC;SN;SgII	secretogranin II	0.86	0.85	0.98	0.58
CEP152	CEP152;FLJ21594;KIAA0912;MCPH4;SCKL5	centrosomal protein 152kDa	0.86	0.84	0.98	0.6
SPA17	SPA17;CT22;SP17;SP17-1	sperm autoantigenic protein 17	0.86	0.88	0.98	0.66
DNAJC30	DNAJC30;MGC12943;WBSCR18	DnaJ (Hsp40) homolog, subfamily C, member 30	0.86	0.76	0.98	0.63
APAF1	APAF1;APAF-1;CED4;DKFZp781B1145	apoptotic peptidase activating factor 1	0.86	0.84	0.98	0.63
NMT2	NMT2	N-myristoyltransferase 2	0.86	0.82	0.98	0.61
TIAL1	TIAL1;MGC33401;TCBP;TIAR	TIA1 cytotoxic granule-associated RNA binding protein-like 1	0.86	0.8	0.98	0.62
RPRD1A	RPRD1A;FLJ10656;HsT3101;MGC19513;P15RS	regulation of nuclear pre-mRNA domain containing 1A	0.86	0.87	0.98	0.66
POGK	POGK;BASS2;KIAA1513;KIAA15131;KRBOX2	pogo transposable element with KRAB domain	0.86	0.88	0.98	0.71
AATF	AATF;CHE-1;CHE1;DED	apoptosis antagonizing transcription factor	0.86	0.93	0.98	0.65
RABL5	RABL5;DKFZp761N0823;FLJ13225;FLJ14117	RAB, member RAS oncogene family-like 5	0.86	0.82	0.98	0.62
CCDC30	CCDC30;FLJ20972;PFD6L;PFDN6L	coiled-coil domain containing 30	0.86	0.88	0.98	0.65
OTOL1	OTOL1;C1QTNF15	otolin 1	0.85	0.86	0.98	0.65
C1QTNF1	C1QTNF1;CTRP1;FLJ90694;GIP;ZSIG37	C1q and tumor necrosis factor related protein 1	0.85	0.87	0.98	0.7
USP20	USP20;KIAA1003;LSFR3A;VDU2	ubiquitin specific peptidase 20	0.85	0.83	0.98	0.62
NPIP	NPIP;FLJ42525;FLJ44848;morpheus	nuclear pore complex interacting protein	1.25	1.27	0.99	0.96
BRPF3	BRPF3	bromodomain and PHD finger containing, 3	1.23	1.16	0.99	0.83
CSNK1A1L	CSNK1A1L;CK1;MGC33182	casein kinase 1, alpha 1-like	1.2	1.29	0.99	0.92
TRAIP	TRAIP;RNF206;TRIP	TRAF interacting protein	1.19	1.14	0.99	0.82
SAAL1	SAAL1;FLJ41463;SPACIA1	serum amyloid A-like 1	1.19	1.26	0.99	0.97
ENAM	ENAM;ADAI;AI1C;AIH2	enamelin	1.17	1.17	0.99	0.79
PDZRN3	PDZRN3;LNX3;SEMACAP3	PDZ domain containing ring finger 3	1.16	1.09	0.99	0.79
P4HB	P4HB;DSI;ERBA2L;GIT;P4Hbeta;PDI;PDIA1;PHDB;PO4DB;PO4HB;PROHB	prolyl 4-hydroxylase, beta polypeptide	1.16	1.19	0.99	0.93
FAM69A	FAM69A;FLJ23493	family with sequence similarity 69, member A	1.14	1.11	0.99	0.83
UGT1A3	UGT1A3;UDPGT;UDPGT 1-3;UGT-1C;UGT1-03;UGT1.3;UGT1C	UDP glucuronosyltransferase 1 family, polypeptide A3	1.14	1.11	0.99	0.78
GCNT1	GCNT1;C2GNT;C2GNT-L;C2GNT1;G6NT;MGC126335;MGC126336;NACGT2;NAGCT2	glucosaminyl (N-acetyl) transferase 1, core 2	1.13	1.14	0.99	0.9
C17orf28	C17orf28;DMC1;FLJ43526	chromosome 17 open reading frame 28	1.12	1.01	0.99	0.8
CCL20	CCL20;CKb4;LARC;MIP-3a;MIP3A;SCYA20;ST38	chemokine (C-C motif) ligand 20	1.11	1.05	0.99	0.76
SCEL	SCEL;FLJ21667;MGC22531	sciellin	1.11	1.09	0.99	0.98
RNF20	RNF20;BRE1;BRE1A;FLJ11189;FLJ20382;KIAA2779;MGC129667;MGC129668;hBRE1	ring finger protein 20	1.11	1.07	0.99	0.97
C3orf33	C3orf33;AC3-33;FLJ31139	chromosome 3 open reading frame 33	1.1	1.12	0.99	0.83
HR	HR;ALUNC;AU;FLJ98880;HSA277165;MUHH;MUHH1	hairless homolog (mouse)	1.1	1.04	0.99	0.75
CTNS	CTNS;CTNS-LSB;PQLC4	cystinosin, lysosomal cystine transporter	1.1	1.2	0.99	0.84
NEDD4L	NEDD4L;FLJ33870;KIAA0439;NEDD4-2;NEDD4.2;RSP5;hNEDD4-2	neural precursor cell expressed, developmentally down-regulated 4-like	1.1	1.01	0.99	0.76
ZNF518B	ZNF518B;KIAA1729	zinc finger protein 518B	1.09	1.02	0.99	0.86
POMGNT1	POMGNT1;DKFZp761B182;FLJ20277;GNTI.2;GnT I.2;MDDGA3;MDDGB3;MDDGC3;MEB;MGAT1.2;gnT-I.2	protein O-linked mannose beta1,2-N-acetylglucosaminyltransferase	1.09	1.16	0.99	0.89
UBLCP1	UBLCP1;CPUB1;FLJ25267;MGC10067	ubiquitin-like domain containing CTD phosphatase 1	1.09	1.06	0.99	0.83
EIF2AK2	EIF2AK2;EIF2AK1;MGC126524;PKR;PRKR	eukaryotic translation initiation factor 2-alpha kinase 2	1.09	1.16	0.99	0.83
PTP4A2	PTP4A2;HH13;HH7-2;HU-PP-1;OV-1;PRL-2;PRL2;PTP4A;PTPCAAX2;ptp-IV1a;ptp-IV1b	protein tyrosine phosphatase type IVA, member 2	1.09	1.08	0.99	0.85
KCNK16	KCNK16;K2p16.1;MGC133123;TALK-1;TALK1	potassium channel, subfamily K, member 16	1.08	1.13	0.99	0.88
TMEM68	TMEM68;FLJ32370;MGC87778	transmembrane protein 68	1.08	1.02	0.99	0.83
TTL	TTL;MGC46235	tubulin tyrosine ligase	1.08	1	0.99	0.8
SQLE	SQLE;FLJ30795	squalene epoxidase	1.08	1.05	0.99	0.74
TRIM39	TRIM39;MGC32984;RNF23;TFP;TRIM39B	tripartite motif containing 39	1.07	1.02	0.99	0.73
IL10RB	IL10RB;CDW210B;CRF2-4;CRFB4;D21S58;D21S66;IL-10R2	interleukin 10 receptor, beta	1.07	1.01	0.99	0.73
H2AFV	H2AFV;FLJ26479;H2AV;MGC10170;MGC10831;MGC1947	H2A histone family, member V	1.07	1.04	0.99	0.78
DDX20	DDX20;DKFZp434H052;DP103;GEMIN3	DEAD (Asp-Glu-Ala-Asp) box polypeptide 20	1.06	1.06	0.99	0.82
TINAGL1	TINAGL1;ARG1;LCN7;LIECG3;TINAGRP	tubulointerstitial nephritis antigen-like 1	1.06	0.97	0.99	0.73
EPHA5	EPHA5;CEK7;EHK1;HEK7;TYRO4	EPH receptor A5	1.05	1.05	0.99	0.87
C15orf17	C15orf17;FLJ00005	chromosome 15 open reading frame 17	1.05	1.02	0.99	0.85
TBC1D16	TBC1D16;FLJ20748;MGC25062	TBC1 domain family, member 16	1.05	1.1	0.99	0.77
HERC5	HERC5;CEB1;CEBP1	hect domain and RLD 5	1.04	0.94	0.99	0.71
ALG10	ALG10;DIE2;FLJ14751;KCR1	asparagine-linked glycosylation 10, alpha-1,2-glucosyltransferase homolog (S. pombe)	1.04	1.06	0.99	0.84
SLC7A2	SLC7A2;ATRC2;CAT2;HCAT2	solute carrier family 7 (cationic amino acid transporter, y+ system), member 2	1.04	1.06	0.99	0.8
ADCY9	ADCY9;AC9;FLJ12046	adenylate cyclase 9	1.04	1.04	0.99	0.7
FH	FH;HLRCC;LRCC;MCL;MCUL1	fumarate hydratase	1.04	1.04	0.99	0.7
PMS1	PMS1;DKFZp781M0253;FLJ98259;HNPCC3;PMSL1;hPMS1	PMS1 postmeiotic segregation increased 1 (S. cerevisiae)	1.03	1.11	0.99	0.83
DCBLD1	DCBLD1;DKFZp686L21135;MGC46341;dJ94G16.1	discoidin, CUB and LCCL domain containing 1	1.03	1.04	0.99	0.79
DEFB119	DEFB119;DEFB-19;DEFB-20;DEFB120;ESC42-RELA;ESC42-RELB;MGC71893	defensin, beta 119	1.02	0.95	0.99	0.8
HIST1H2AA	HIST1H2AA;H2AA;H2AFR;bA317E16.2	histone cluster 1, H2aa	1.02	0.9	0.99	0.75
COCH	COCH;COCH-5B2;COCH5B2;DFNA9	coagulation factor C homolog, cochlin (Limulus polyphemus)	1.02	0.92	0.99	0.73

KRTAP5-9	KRTAP5-9;KRN1;KRTAP5-1;KRTAP5.9;MGC126793	keratin associated protein 5-9	1.02	1	0.99	0.89
SLC25A20	SLC25A20;CAC;CACT	solute carrier family 25 (carnitine/acylcarnitine translocase), member 20	1.02	0.94	0.99	0.75
CCDC97	CCDC97;FLJ40267;MGC20255	coiled-coil domain containing 97	1.02	1.08	0.99	0.83
OLA1	OLA1;DKFZp313H1942;DOC45;GBP45;GTBP9;GTPBP9	Obg-like ATPase 1	1.01	1.04	0.99	0.77
VPS13C	VPS13C;DKFZp686E0570;FLJ21361	vacuolar protein sorting 13 homolog C (S. cerevisiae)	1	1.02	0.99	0.78
LRRC37A	LRRC37A;DKFZp686N1231;KIAA0563	leucine rich repeat containing 37A	1	0.97	0.99	0.73
BTN2A2	BTN2A2;BT2.2;BTF2;FLJ41908	butyrophilin, subfamily 2, member A2	1	1.02	0.99	0.77
MRS2	MRS2;HPT;MGC78523;MRS2L	MRS2 magnesium homeostasis factor homolog (S. cerevisiae)	1	0.97	0.99	0.81
NOX5	NOX5;MGC149776;MGC149777	NADPH oxidase, EF-hand calcium binding domain 5	1	0.98	0.99	0.77
TTI2	TTI2;C8orf41;FLJ23263	Tel2 interacting protein 2 homolog (S. pombe)	1	1.04	0.99	0.75
OR12D3	OR12D3;MGC119267;MGC125888;hs6M1-27	olfactory receptor, family 12, subfamily D, member 3	0.99	1.02	0.99	0.83
MYL9	MYL9;LC20;MGC3505;MLC-2C;MLC2;MRLC1;MYRL2	myosin, light chain 9, regulatory	0.99	0.88	0.99	0.7
SLC2A1	SLC2A1;DYT17;DYT18;GLUT;GLUT1;GLUT1DS;MGC141895;MGC141896;PED	solute carrier family 2 (facilitated glucose transporter), member 1	0.99	1.04	0.99	0.79
TAL2	TAL2	T-cell acute lymphocytic leukemia 2	0.98	0.98	0.99	0.66
CYP26A1	CYP26A1;CP26;CYP26;P450RAI;P450RAI1	cytochrome P450, family 26, subfamily A, polypeptide 1	0.98	1.02	0.99	0.68
PDSS2	PDSS2;C6orf210;DLP1;bA59I9.3;hDLP1	prenyl (decaprenyl) diphosphate synthase, subunit 2	0.98	0.97	0.99	0.65
UBA7	UBA7;D8;MGC12713;UBA1B;UBE1L;UBE2	ubiquitin-like modifier activating enzyme 7	0.98	0.9	0.99	0.67
TRIM73	TRIM73;FLJ99347;MGC45477;TRIM50B	tripartite motif containing 73	0.98	0.96	0.99	0.85
CABP4	CABP4;CSNB2B	calcium binding protein 4	0.98	1.02	0.99	0.82
LYRM5	LYRM5	LYR motif containing 5	0.98	1.03	0.99	0.79
ATXN7	ATXN7;ADCAII;FLJ17787;OPCA3;SCA7	ataxin 7	0.98	0.94	0.99	0.71
NFYC	NFYC;CBF-C;CBFC;DKFZp667G242;FLJ45775;H1TF2A;HAP5;HSM;NF-YC	nuclear transcription factor Y, gamma	0.98	0.9	0.99	0.8
CPVL	CPVL;HVLP;MGC10029	carboxypeptidase, vitellogenic-like	0.98	0.97	0.99	0.72
TMEM194A	TMEM194A;DKFZp686N1768;KIAA0286;TMEM194	transmembrane protein 194A	0.98	0.94	0.99	0.71
GPR126	GPR126;APG1;DREG;PS1TP2;VIGR	G protein-coupled receptor 126	0.97	0.93	0.99	0.84
KIAA1217	KIAA1217;DKFZp761L0424;MGC31990;SKT	KIAA1217	0.97	0.87	0.99	0.69
NXF5	NXF5	nuclear RNA export factor 5	0.96	0.9	0.99	0.76
TTLL9	TTLL9;C20orf125;MGC120486;MGC120487;dJ310O13.1	tubulin tyrosine ligase-like family, member 9	0.96	0.89	0.99	0.72
ZNF570	ZNF570;FLJ30791	zinc finger protein 570	0.96	0.94	0.99	0.78
LCK	LCK;LSK;YT16;p56lck;pp58lck	lymphocyte-specific protein tyrosine kinase	0.96	0.88	0.99	0.66
LY75	LY75;CD205;CLEC13B;DEC-205;GP200-MR6;LY-75	lymphocyte antigen 75	0.96	0.95	0.99	0.66
RGMA	RGMA;RGM	RGM domain family, member A	0.96	0.96	0.99	0.67
C17orf90	C17orf90;MGC104712	chromosome 17 open reading frame 90	0.96	0.98	0.99	0.74
GRAMD1A	GRAMD1A;FLJ22411;FLJ90346;KIAA1533	GRAM domain containing 1A	0.96	1.04	0.99	0.69
PITPNA	PITPNA;MGC99649;PI-TPalpha;PITPN;VIB1A	phosphatidylinositol transfer protein, alpha	0.96	0.88	0.99	0.77
STMN1	STMN1;C1orf215;FLJ32206;LAP18;Lag;MGC138869;MGC138870;OP18;PP17;PP19;PR22;SMN	stathmin 1	0.96	1	0.99	0.74
IFI27L1	IFI27L1;FAM14B;ISG12C	interferon, alpha-inducible protein 27-like 1	0.96	0.94	0.99	0.71
TFF2	TFF2;SML1;SP	trefoil factor 2	0.95	0.92	0.99	0.74
ATAT1	ATAT1;C6orf134;DKFZp547J097;FLJ13158;MEC17;Nbla00487;TAT	alpha tubulin acetyltransferase 1	0.95	0.9	0.99	0.69
DHRS4L1	DHRS4L1;SDR25C4	dehydrogenase/reductase (SDR family) member 4 like 1	0.95	0.94	0.99	0.66
SFTPC	SFTPC;PSP-C;SFTP2;SMDP2;SP-C	surfactant protein C	0.94	0.84	0.99	0.7
NMUR1	NMUR1;(FM-3);FM-3;FM3;GPC-R;GPR66;NMU1R	neuromedin U receptor 1	0.94	0.88	0.99	0.8
CRB2	CRB2;FLJ16786;FLJ38464	crumbs homolog 2 (Drosophila)	0.94	0.98	0.99	0.79
TSC1	TSC1;KIAA0243;LAM;MGC86987;TSC	tuberous sclerosis 1	0.94	0.91	0.99	0.73
AGPAT9	AGPAT9;AGPAT8;GPAT3;LPAAT-theta;MAG1;MGC11324	1-acylglycerol-3-phosphate O-acyltransferase 9	0.94	0.92	0.99	0.65
METTL2A	METTL2A;FLJ12760;METTL2	methyltransferase like 2A	0.94	0.91	0.99	0.68
DCAF6	DCAF6;1200006M05Rik;ARCAP;FLJ23798;IQWD1;MSTP055;NRIP;PC326	DDB1 and CUL4 associated factor 6	0.94	1.01	0.99	0.78
PHAX	PHAX;FLJ13193;RNUXA	phosphorylated adaptor for RNA export	0.94	0.9	0.99	0.69
ZNF536	ZNF536;KIAA0390	zinc finger protein 536	0.93	0.86	0.99	0.73
CSMD2	CSMD2;KIAA1884;dJ1007G16.1;dJ1007G16.2;dJ947L8.1	CUB and Sushi multiple domains 2	0.93	0.92	0.99	0.63
GALNTL1	GALNTL1;GALNACT16;GALNT16;KIAA1130;MGC141855	UDP-N-acetyl-alpha-D-galactosamine:polypeptide N-acetylgalactosaminyltransferase-like 1	0.93	0.92	0.99	0.68
FAM135A	FAM135A;DKFZp781H2319;FLJ13577;KIAA1411	family with sequence similarity 135, member A	0.93	0.92	0.99	0.66
ERCC4	ERCC4;ERCC11;RAD1;XPF	excision repair cross-complementing rodent repair deficiency, complementation group 4	0.93	0.98	0.99	0.65
TRIM32	TRIM32;BBS11;HT2A;LGMD2H;TATIP	tripartite motif containing 32	0.93	0.92	0.99	0.82
KCTD15	KCTD15;MGC25497;MGC2628	potassium channel tetramerisation domain containing 15	0.93	0.96	0.99	0.74
USH2A	USH2A;RP39;US2;USH2;dJ1111A8.1	Usher syndrome 2A (autosomal recessive, mild)	0.92	0.89	0.99	0.7
DDN	DDN;KIAA0749	dendrin	0.92	0.94	0.99	0.71
PPM1M	PPM1M;FLJ32332;PP2C-eta;PP2CE;PP2Ceta	protein phosphatase, Mg2+/Mn2+ dependent, 1M	0.92	0.84	0.99	0.64
LAMA5	LAMA5;KIAA1907	laminin, alpha 5	0.92	0.88	0.99	0.64
KLHL12	KLHL12;C3IP1;DKIR;FLJ27152	kelch-like 12 (Drosophila)	0.92	0.88	0.99	0.63
UBE2G2	UBE2G2;UBC7	ubiquitin-conjugating enzyme E2G 2	0.92	0.84	0.99	0.63
BRP44L	BRP44L;dJ68L15.3	brain protein 44-like	0.92	0.88	0.99	0.67
WTAP	WTAP;DKFZp686F20131;KIAA0105;MGC3925	Wilms tumor 1 associated protein	0.92	0.9	0.99	0.67
SLC27A5	SLC27A5;ACSB;ACSVL6;FACVL3;FATP5;FLJ22987;VLACSR;VLCS-H2;VLCSH2	solute carrier family 27 (fatty acid transporter), member 5	0.91	0.96	0.99	0.63
SHOC2	SHOC2;FLJ60412;KIAA0862;SIAA0862;SOC2;SUR8	soc-2 suppressor of clear homolog (C. elegans)	0.91	0.98	0.99	0.72
OR1L3	OR1L3;OR9-28;OR9-D	olfactory receptor, family 1, subfamily L, member 3	0.9	0.92	0.99	0.61
RSPO2	RSPO2;CRISTIN2;MGC35555;MGC43342	R-spondin 2	0.9	0.94	0.99	0.66
CDH26	CDH26;VR20	cadherin 26	0.9	0.89	0.99	0.62
TCEA3	TCEA3;TFIIS;TFIIS.H	transcription elongation factor A (SII), 3	0.9	0.86	0.99	0.63
RSPH3	RSPH3;RSHL2;RSP3;dJ111C20.1	radial spoke 3 homolog (Chlamydomonas)	0.9	0.93	0.99	0.7
DDB2	DDB2;DDBB;FLJ34321;UV-DDB2	damage-specific DNA binding protein 2, 48kDa	0.9	0.86	0.99	0.75
TPST2	TPST2	tyrosylprotein sulfotransferase 2	0.9	0.88	0.99	0.65
RECQL4	RECQL4;RECQ4	RecQ protein-like 4	0.9	0.86	0.99	0.61
LONP1	LONP1;LON;LONP;LonHS;MGC1498;PIM1;PRSS15;hLON	lon peptidase 1, mitochondrial	0.9	0.88	0.99	0.66
C17orf79	C17orf79;COPR5;FLJ21119;HSA272196;TTP1	chromosome 17 open reading frame 79	0.9	0.96	0.99	0.67
BTNL9	BTNL9;BTN3;VDLS1900	butyrophilin-like 9	0.89	0.92	0.99	0.61
ETAA1	ETAA1;ETAA16;FLJ22647	Ewing tumor-associated antigen 1	0.89	0.9	0.99	0.65
POFUT2	POFUT2;C21orf80;FUT13	protein O-fucosyltransferase 2	0.89	0.88	0.99	0.6
RELB	RELB;I-REL;IREL;REL-B	v-rel reticuloendotheliosis viral oncogene homolog B	0.89	0.84	0.99	0.61
IER2	IER2;ETR101;MGC111472;MGC15265	immediate early response 2	0.89	0.92	0.99	0.61
SORCS3	SORCS3;SORCS	sortilin-related VPS10 domain containing receptor 3	0.88	0.84	0.99	0.76
ADCY4	ADCY4;AC4	adenylate cyclase 4	0.88	0.84	0.99	0.61
POLR3G	POLR3G;RPC32;RPC7	polymerase (RNA) III (DNA directed) polypeptide G (32kD)	0.88	0.83	0.99	0.65
TEC	TEC;MGC126760;MGC126762;PSCTK4	tec protein tyrosine kinase	0.88	0.94	0.99	0.67
KRTAP5-10	KRTAP5-10;KRTAP5.10	keratin associated protein 5-10	0.88	0.9	0.99	0.62
NHEJ1	NHEJ1;FLJ12610;XLF	nonhomologous end-joining factor 1	0.88	0.84	0.99	0.66
ATP2A2	ATP2A2;ATP2B;DAR;DD;DKFZp686P0211;FLJ20293;FLJ38063;MGC45367;SERCA2	ATPase, Ca++ transporting, cardiac muscle, slow twitch 2	0.88	0.88	0.99	0.68
PCDHGB6	PCDHGB6;PCDH-GAMMA-B6	protocadherin gamma subfamily B, 6	0.88	0.89	0.99	0.6
ZNF287	ZNF287;MGC126536;MGC141923;ZKSCAN13	zinc finger protein 287	0.87	0.88	0.99	0.67
COL1A2	COL1A2;OI4	collagen, type I, alpha 2	0.87	0.88	0.99	0.69
TCF20	TCF20;AR1;KIAA0292;SPBP	transcription factor 20 (AR1)	0.87	0.84	0.99	0.66
RAB5B	RAB5B	RAB5B, member RAS oncogene family	0.87	0.86	0.99	0.58
GNB2	GNB2	guanine nucleotide binding protein (G protein), beta polypeptide 2	0.87	0.85	0.99	0.7
UGGT2	UGGT2;FLJ10873;FLJ11485;HUGT2;MGC117360;MGC150689;MGC87276;UGCGL2;UGT2	UDP-glucose glycoprotein glucosyltransferase 2	0.87	0.94	0.99	0.66
PGM5	PGM5;PGMRP	phosphoglucomutase 5	0.86	0.9	0.99	0.6
MYOCD	MYOCD;MYCD	myocardin	0.86	0.83	0.99	0.65
PRDM14	PRDM14;MGC59730;PFM11	PR domain containing 14	0.86	0.84	0.99	0.62
TXK	TXK;BTKL;MGC22473;PSCTK5;PTK4;RLK;TKL	TXK tyrosine kinase	0.86	0.86	0.99	0.67
HCN3	HCN3;KIAA1535;MGC131493	hyperpolarization activated cyclic nucleotide-gated potassium channel 3	0.86	0.89	0.99	0.69
NRIP3	NRIP3;C11orf14;NY-SAR-105	nuclear receptor interacting protein 3	0.86	0.9	0.99	0.73
KLHL17	KLHL17;RP11-54O7.6	kelch-like 17 (Drosophila)	0.86	0.91	0.99	0.64
AMN	AMN;PRO1028	amnionless homolog (mouse)	0.86	0.88	0.99	0.66
REXO1	REXO1;ELOABP1;EloA-BP1;KIAA1138;REX1;TCEB3BP1	REX1, RNA exonuclease 1 homolog (S. cerevisiae)	0.86	0.82	0.99	0.6
MTHFS	MTHFS;FLJ30410;HsT19268	5,10-methenyltetrahydrofolate synthetase (5-formyltetrahydrofolate cyclo-ligase)	0.86	0.92	0.99	0.69
E2F8	E2F8;E2F-8;FLJ23311	E2F transcription factor 8	0.86	0.81	0.99	0.66
SEC11A	SEC11A;1810012E07Rik;SEC11L1;SPC18;SPCS4A;sid2895	SEC11 homolog A (S. cerevisiae)	0.86	0.84	0.99	0.63
MUS81	MUS81;FLJ21012;FLJ44872;SLX3	MUS81 endonuclease homolog (S. cerevisiae)	0.86	0.85	0.99	0.57
XRCC2	XRCC2;DKFZp781P0919	X-ray repair complementing defective repair in Chinese hamster cells 2	0.86	0.87	0.99	0.69
MAP7	MAP7;E-MAP-115;EMAP115	microtubule-associated protein 7	0.86	0.84	0.99	0.7
SSSCA1	SSSCA1;p27	Sjogren syndrome/scleroderma autoantigen 1	0.86	0.86	0.99	0.58
HIAT1	HIAT1;DKFZp564L0864	hippocampus abundant transcript 1	0.86	0.8	0.99	0.63
LIMS3	LIMS3;FLJ38851;FLJ59126;FLJ79109;MGC138438;PINCH-3	LIM and senescent cell antigen-like domains 3	0.86	0.88	0.99	0.71
MECOM	MECOM;AML1-EVI-1;EVI1;MDS1;MDS1-EVI1;MGC163392;MGC97004;PRDM3	MDS1 and EVI1 complex locus	0.86	0.88	0.99	0.59
BPIFB3	BPIFB3;C20orf185;LPLUNC3;RYA3;dJ726C3.4	BPI fold containing family B, member 3	0.86	0.82	0.99	0.63
MTNR1B	MTNR1B;FGQTL2;MEL-1B-R;MT2	melatonin receptor 1B	0.85	0.82	0.99	0.74
MAP2	MAP2;DKFZp686I2148;MAP2A;MAP2B;MAP2C	microtubule-associated protein 2	0.85	0.85	0.99	0.57
TTC19	TTC19;2010204O13Rik;FLJ20343;MGC138312;MGC19520	tetratricopeptide repeat domain 19	0.85	0.82	0.99	0.61
NID2	NID2;NID-2	nidogen 2 (osteonidogen)	0.85	0.82	0.99	0.62
TMEM30A	TMEM30A;C6orf67;CDC50A;FLJ10856	transmembrane protein 30A	1.36	1.21	1	0.97
XAF1	XAF1;BIRC4BP;HSXIAPAF1;XIAPAF1	XIAP associated factor 1	1.31	1.31	1	0.94
ADAMTS19	ADAMTS19;FLJ16042	ADAM metallopeptidase with thrombospondin type 1 motif, 19	1.3	1.19	1	0.9
PRSS12	PRSS12;BSSP-3;BSSP3;MGC12722;MRT1	protease, serine, 12 (neurotrypsin, motopsin)	1.29	1.27	1	0.95
DUSP4	DUSP4;HVH2;MKP-2;MKP2;TYP	dual specificity phosphatase 4	1.24	1.15	1	0.87
ZNF93	ZNF93;HPF34;HTF34;TF34;ZNF505	zinc finger protein 93	1.23	1.31	1	0.92
PPP2R5B	PPP2R5B;B56B;FLJ35411;PR61B	protein phosphatase 2, regulatory subunit B', beta	1.23	1.21	1	0.95
PRICKLE4	PRICKLE4;C6orf49;MGC120398;OBTP;OEBT;TOMM6	prickle homolog 4 (Drosophila)	1.22	1.17	1	0.84
NOTCH1	NOTCH1;TAN1;hN1	notch 1	1.21	1.21	1	0.89
TSPAN13	TSPAN13;FLJ22934;NET-6;NET6;TM4SF13	tetraspanin 13	1.21	1.23	1	0.85
CAMK1G	CAMK1G;CLICKIII;VWS1;dJ272L16.1	calcium/calmodulin-dependent protein kinase IG	1.17	1.17	1	0.97

KIF21B	KIF21B;FLJ16314	kinesin family member 21B	1.17	1.19	1	0.95
LOC644006	LOC644006	ring finger protein 4 pseudogene	1.17	1.12	1	0.81
C17orf57	C17orf57;FLJ36110;FLJ40342;FLJ58126	chromosome 17 open reading frame 57	1.16	1.13	1	0.84
WDR86	WDR86;FLJ51355;MGC129839	WD repeat domain 86	1.16	1.13	1	0.84
CCDC88B	CCDC88B;BRLZ;CCDC88;DKFZp434G0920;FLJ00354;FLJ37970;HKRP3;gipie	coiled-coil domain containing 88B	1.15	1.12	1	0.84
WRNIP1	WRNIP1;FLJ22526;RP11-420G6.2;WHIP;bA420G6.2	Werner helicase interacting protein 1	1.15	1.17	1	0.78
FBXW9	FBXW9;Fbw9;MGC10870	F-box and WD repeat domain containing 9	1.14	1.09	1	0.79
FAM150B	FAM150B;PRO1097;RGPG542	family with sequence similarity 150, member B	1.14	1.21	1	0.85
HADH	HADH;HAD;HADH1;HADHSC;HCDH;HHF4;MGC8392;MSCHAD;SCHAD	hydroxyacyl-CoA dehydrogenase	1.14	1.1	1	0.78
PNRC2	PNRC2;FLJ20312;MGC99541	proline-rich nuclear receptor coactivator 2	1.14	1.17	1	0.89
GRWD1	GRWD1;CDW4;GRWD;KIAA1942;RRB1;WDR28	glutamate-rich WD repeat containing 1	1.13	1.11	1	0.92
ZBTB41	ZBTB41;DKFZp686C06120;FLJ36199;FRBZ1;RP11-469L3.1;ZNF924	zinc finger and BTB domain containing 41	1.13	1	1	0.79
SLC5A12	SLC5A12;DKFZp564G223;MGC52019;SMCT2	solute carrier family 5 (sodium/glucose cotransporter), member 12	1.12	1.19	1	0.79
PIP5K1C	PIP5K1C;KIAA0589;LCCS3;PIP5K-GAMMA;PIP5Kgamma;PIPKIg_v4	phosphatidylinositol-4-phosphate 5-kinase, type I, gamma	1.12	1.21	1	0.86
TRIM33	TRIM33;ECTO;FLJ32925;PTC7;RFG7;TF1G;TIF1G;TIF1GAMMA;TIFGAMMA	tripartite motif containing 33	1.12	1.07	1	0.78
WHSC1L1	WHSC1L1;DKFZp667H044;FLJ20353;MGC126766;MGC142029;NSD3;pp14328	Wolf-Hirschhorn syndrome candidate 1-like 1	1.12	1.08	1	0.78
PHF3	PHF3;KIAA0244;MGC142210;MGC142212	PHD finger protein 3	1.12	1.11	1	0.99
PM20D1	PM20D1;Cps1;FLJ32569	peptidase M20 domain containing 1	1.11	1.1	1	0.82
GGT1	GGT1;CD224;D22S672;D22S732;GGT;GGT 1;GTG;MGC96892;MGC96904;MGC96963	gamma-glutamyltransferase 1	1.11	1.02	1	0.76
PTX3	PTX3;TNFAIP5;TSG-14	pentraxin 3, long	1.11	1.16	1	0.94
LOC642678	LOC642678	ENTREZ STATUS: DISCONTINUED	1.1	1.05	1	0.76
PTGFR	PTGFR;FP;MGC120498;MGC46203	prostaglandin F receptor (FP)	1.09	1.01	1	0.76
ADIPOQ	ADIPOQ;ACDC;ACRP30;ADIPQTL1;ADPN;APM-1;APM1;GBP28	adiponectin, C1Q and collagen domain containing	1.09	1.19	1	0.8
C15orf44	C15orf44;DKFZp564O1664	chromosome 15 open reading frame 44	1.08	1.05	1	0.78
CT47A11	CT47A11;CT47.11;CT47A1;CT47A10;CT47A12;CT47A2;CT47A3;CT47A4;CT47A5;CT47A6;CT47A7;CT47A8;CT47A9	cancer/testis antigen family 47, member A11	1.08	1.08	1	0.78
AP3S2	AP3S2;AP3S3;FLJ35955;sigma3b	adaptor-related protein complex 3, sigma 2 subunit	1.07	1.06	1	0.75
RSPRY1	RSPRY1;KIAA1972	ring finger and SPRY domain containing 1	1.07	1.02	1	0.74
SCGB1D4	SCGB1D4	secretoglobin, family 1D, member 4	1.06	1.17	1	0.86
KIF5C	KIF5C;FLJ44735;KIAA0531;KINN;MGC111478;NKHC;NKHC-2;NKHC2	kinesin family member 5C	1.06	1.08	1	0.86
GALNT9	GALNT9;GALNAC-T9	UDP-N-acetyl-alpha-D-galactosamine:polypeptide N-acetylgalactosaminyltransferase 9 (GalNAc-T9)	1.06	0.98	1	0.87
RNF157	RNF157	ring finger protein 157	1.06	1.05	1	0.95
ST6GALNAC6	ST6GALNAC6;RP11-203J24.3;SIAT7F;ST6GALNACVI	ST6 (alpha-N-acetyl-neuraminyl-2,3-beta-galactosyl-1,3)-N-acetylgalactosaminide alpha-2,6-sialyltransferase 6	1.06	1.12	1	0.74
TNKS2	TNKS2;PARP-5b;PARP-5c;PARP5B;PARP5C;TANK2;TNKL;pART6	tankyrase, TRF1-interacting ankyrin-related ADP-ribose polymerase 2	1.06	0.98	1	0.86
NOTUM	NOTUM	notum pectinacetylesterase homolog (Drosophila)	1.05	1.09	1	0.81
FAIM	FAIM;FAIM1	Fas apoptotic inhibitory molecule	1.05	1.02	1	0.83
MKL2	MKL2;DKFZp686J1745;FLJ31823;FLJ45623;MRTF-B;NPD001	MKL/myocardin-like 2	1.05	1.05	1	0.82
POM121L12	POM121L12	POM121 membrane glycoprotein-like 12	1.05	0.94	1	0.78
G6PC2	G6PC2;IGRP;MGC141936	glucose-6-phosphatase, catalytic, 2	1.04	0.97	1	0.73
USP30	USP30;FLJ40511;MGC10702	ubiquitin specific peptidase 30	1.04	0.95	1	0.71
ZC3H8	ZC3H8;Fliz1;ZC3HDC8	zinc finger CCCH-type containing 8	1.04	1.12	1	0.86
DUSP16	DUSP16;FLJ36298;FLJ40991;KIAA1700;MGC129701;MGC129702;MKP-7;MKP7	dual specificity phosphatase 16	1.04	1.02	1	0.8
SHQ1	SHQ1;DKFZp686H07226;FLJ10539;Shq1p	SHQ1 homolog (S. cerevisiae)	1.04	1.1	1	0.77
PHKA1	PHKA1;MGC132604;PHKA	phosphorylase kinase, alpha 1 (muscle)	1.03	1.12	1	0.79
TSC22D4	TSC22D4;THG-1;THG1;TILZ2	TSC22 domain family, member 4	1.03	1.06	1	0.79
VDAC1	VDAC1;MGC111064;PORIN;VDAC-1	voltage-dependent anion channel 1	1.03	1.08	1	0.84
NMU	NMU	neuromedin U	1.03	1.01	1	0.84
TTPA	TTPA;ATTP;AVED;TTP1;alphaTTP	tocopherol (alpha) transfer protein	1.02	0.98	1	0.71
TSNAXIP1	TSNAXIP1;MGC111443;TXI1	translin-associated factor X interacting protein 1	1.02	0.96	1	0.82
C3orf67	C3orf67;FLJ42117;FLJ42930;MGC48416	chromosome 3 open reading frame 67	1.02	1.02	1	0.69
FLJ35776	FLJ35776	uncharacterized LOC649446	1.02	1.03	1	0.71
UBE2I	UBE2I;C358B7.1;P18;UBC9	ubiquitin-conjugating enzyme E2I	1.02	0.94	1	0.71
MTMR4	MTMR4;FYVE-DSP2;KIAA0647;ZFYVE11	myotubularin related protein 4	1.02	0.94	1	0.71
BABAM1	BABAM1;C19orf62;FLJ20571;MERIT40;NBA1	BRISC and BRCA1 A complex member 1	1.02	1.09	1	0.77
CES5A	CES5A;CAUXIN;CES4C1;CES5;CES7;FLJ31547	carboxylesterase 5A	1.02	0.98	1	0.71
CCDC85A	CCDC85A;KIAA1912	coiled-coil domain containing 85A	1	1.07	1	0.75
INHA	INHA	inhibin, alpha	1	0.94	1	0.74
TRIM56	TRIM56;DKFZp667O116;FLJ35608;RNF109	tripartite motif containing 56	1	0.99	1	0.89
BIRC6	BIRC6;APOLLON;BRUCE;FLJ13726;FLJ13786;KIAA1289	baculoviral IAP repeat containing 6	1	0.92	1	0.69
CAMLG	CAMLG;CAML;MGC163197	calcium modulating ligand	1	0.9	1	0.75
FAM188A	FAM188A;C10orf97;CARP;DERP5;FLJ13397;MST126;RP11-394I23.1;my042	family with sequence similarity 188, member A	1	0.96	1	0.73
TMX3	TMX3;FLJ20793;KIAA1830;PDIA13;TXNDC10	thioredoxin-related transmembrane protein 3	1	0.93	1	0.74
WDR90	WDR90;C16orf15;C16orf16;C16orf17;C16orf18;C16orf19;FLJ36483;FLJ44660;KIAA1924	WD repeat domain 90	0.99	0.94	1	0.76
GLYR1	GLYR1;BM045;HIBDL;N-PAC;NP60	glyoxylate reductase 1 homolog (Arabidopsis)	0.99	0.95	1	0.73
CEP44	CEP44;KIAA1712;PS1TP3	centrosomal protein 44kDa	0.99	0.95	1	0.77
EDAR	EDAR;DL;ED1R;ED3;ED5;EDA-A1R;EDA1R;EDA3;FLJ94390;HRM1	ectodysplasin A receptor	0.98	0.98	1	0.66
CTSG	CTSG;CATG;CG;MGC23078	cathepsin G	0.98	0.97	1	0.72
LRRC15	LRRC15;LIB	leucine rich repeat containing 15	0.98	1.09	1	0.8
C3orf27	C3orf27;GR6	chromosome 3 open reading frame 27	0.98	1.04	1	0.81
SRGAP3	SRGAP3;ARHGAP14;KIAA0411;MEGAP;SRGAP2;WRP	SLIT-ROBO Rho GTPase activating protein 3	0.98	0.88	1	0.68
TMEM47	TMEM47;BCMP1;DKFZp564E153;DKFZp761J17121;FLJ18096;MGC32949;TM4SF10	transmembrane protein 47	0.98	0.96	1	0.8
LCLAT1	LCLAT1;1AGPAT8;AGPAT8;ALCAT1;FLJ37965;HSRG1849;LYCAT;UNQ1849	lysocardiolipin acyltransferase 1	0.98	0.96	1	0.75
GRPR	GRPR	gastrin-releasing peptide receptor	0.97	0.94	1	0.84
MAF	MAF;MGC71685;c-MAF	v-maf musculoaponeurotic fibrosarcoma oncogene homolog (avian)	0.97	1.04	1	0.78
LAPTM5	LAPTM5;CLAST6;FLJ61683;FLJ97251;MGC125860;MGC125861	lysosomal protein transmembrane 5	0.97	0.92	1	0.78
RAB40C	RAB40C;RARL;RASL8C	RAB40C, member RAS oncogene family	0.97	0.92	1	0.67
PICK1	PICK1;MGC15204;PICK;PRKCABP	protein interacting with PRKCA 1	0.97	0.94	1	0.69
NPAS2	NPAS2;FLJ23138;MGC71151;MOP4;PASD4;bHLHe9	neuronal PAS domain protein 2	0.97	0.9	1	0.8
GZMB	GZMB;CCPI;CGL-1;CGL1;CSP-B;CSPB;CTLA1;CTSGL1;HLP;SECT	granzyme B (granzyme 2, cytotoxic T-lymphocyte-associated serine esterase 1)	0.96	0.96	1	0.72
ATP2B3	ATP2B3;PMCA3;PMCA3a	ATPase, Ca++ transporting, plasma membrane 3	0.96	0.92	1	0.68
DAZL	DAZL;DAZH;DAZL1;DAZLA;MGC26406;SPGYLA	deleted in azoospermia-like	0.95	0.98	1	0.76
GAB3	GAB3	GRB2-associated binding protein 3	0.95	0.98	1	0.67
GBP1	GBP1	guanylate binding protein 1, interferon-inducible	0.95	0.96	1	0.74
THAP2	THAP2;DKFZp564I0422	THAP domain containing, apoptosis associated protein 2	0.94	0.94	1	0.68
ZNF658	ZNF658;DKFZp572C163;FLJ32813;MGC35232	zinc finger protein 658	0.94	0.92	1	0.69
RCOR2	RCOR2	REST corepressor 2	0.94	0.9	1	0.76
KIF27	KIF27;DKFZp434D0917;RP11-575L7.3	kinesin family member 27	0.94	0.94	1	0.63
MAFB	MAFB;KRML;MGC43127	v-maf musculoaponeurotic fibrosarcoma oncogene homolog B (avian)	0.94	0.98	1	0.65
TULP1	TULP1;LCA15;RP14;TUBL1	tubby like protein 1	0.94	0.86	1	0.77
ZNF688	ZNF688	zinc finger protein 688	0.94	0.94	1	0.68
RCBTB2	RCBTB2;CHC1L	regulator of chromosome condensation (RCC1) and BTB (POZ) domain containing protein 2	0.94	0.96	1	0.72
C9orf46	C9orf46;FLJ14688;FLJ39176;MDS030	chromosome 9 open reading frame 46	0.94	0.9	1	0.69
FAM98C	FAM98C;FLJ44669	family with sequence similarity 98, member C	0.94	1.01	1	0.67
HIBCH	HIBCH;HIBYLCOAH	3-hydroxyisobutyryl-CoA hydrolase	0.94	0.92	1	0.64
ZDHHC20	ZDHHC20;FLJ25952;MGC126005	zinc finger, DHHC-type containing 20	0.94	0.94	1	0.68
SRP72	SRP72	signal recognition particle 72kDa	0.94	1.01	1	0.72
TCERG1	TCERG1;CA150;MGC133200;TAF2S;Urn1	transcription elongation regulator 1	0.94	1	1	0.75
GPR125	GPR125;PGR21;TEM5L	G protein-coupled receptor 125	0.94	0.92	1	0.82
TRIML2	TRIML2;FLJ25801;FLJ56315;MGC138164;MGC138166;SPRYD6	tripartite motif family-like 2	0.93	0.9	1	0.65
MPL	MPL;C-MPL;CD110;MPLV;TPOR	myeloproliferative leukemia virus oncogene	0.93	0.93	1	0.69
LRRC27	LRRC27	leucine rich repeat containing 27	0.93	0.9	1	0.65
POU6F1	POU6F1;BRN5;MPOU;TCFB1	POU class 6 homeobox 1	0.93	1	1	0.75
YIPF4	YIPF4;FinGER4;MGC11061	Yip1 domain family, member 4	0.93	0.88	1	0.72
MRPL19	MRPL19;KIAA0104;L19mt;MGC20675;MRP-L15;MRP-L19;MRPL15;RLX1;RPML15	mitochondrial ribosomal protein L19	0.93	0.83	1	0.69
C6orf204	C6orf204;MGC131785;NY-BR-15;bA57K17.2	chromosome 6 open reading frame 204	0.92	0.96	1	0.78
IL12B	IL12B;CLMF;CLMF2;IL-12B;NKSF;NKSF2	interleukin 12B (natural killer cell stimulatory factor 2, cytotoxic lymphocyte maturation factor 2, p40)	0.92	0.9	1	0.7
GPR101	GPR101;GPCR6	G protein-coupled receptor 101	0.92	0.9	1	0.81
C2orf57	C2orf57;MGC35154	chromosome 2 open reading frame 57	0.92	0.93	1	0.63
TMEM132E	TMEM132E	transmembrane protein 132E	0.92	0.88	1	0.71
GDAP1L1	GDAP1L1;DKFZp761K228;dJ881L22.1;dJ995J12.1.1	ganglioside-induced differentiation-associated protein 1-like 1	0.92	0.94	1	0.64
ZNHIT2	ZNHIT2;C11orf5;FON;MGC120285;MGC120286	zinc finger, HIT-type containing 2	0.92	0.92	1	0.66
SP5	SP5	Sp5 transcription factor	0.92	0.9	1	0.64
MED25	MED25;ACID1;ARC92;CMT2B2;DKFZp434K0512;MGC70671;P78;PTOV2	mediator complex subunit 25	0.92	0.82	1	0.64
TRMT6	TRMT6;GCD10;Gcd10p;MGC5029	tRNA methyltransferase 6 homolog (S. cerevisiae)	0.92	0.94	1	0.72
SLC44A3	SLC44A3;CTL3;MGC45474	solute carrier family 44, member 3	0.92	0.87	1	0.7
MFAP3	MFAP3;DKFZp586F0219	microfibrillar-associated protein 3	0.92	0.94	1	0.72
YES1	YES1;HsT441;P61-YES;Yes;c-yes	v-yes-1 Yamaguchi sarcoma viral oncogene homolog 1	0.92	0.92	1	0.76
DNAJC11	DNAJC11;FLJ10737;dJ126A5.1	DnaJ (Hsp40) homolog, subfamily C, member 11	0.92	0.9	1	0.71
DAZAP2	DAZAP2;KIAA0058;MGC14319;MGC766;PRTB	DAZ associated protein 2	0.92	0.9	1	0.73
TMEM171	TMEM171;PRP2	transmembrane protein 171	0.91	0.87	1	0.67
SLC5A2	SLC5A2;SGLT2	solute carrier family 5 (sodium/glucose cotransporter), member 2	0.91	0.88	1	0.76
TIGD2	TIGD2;DKFZp667D1322	tigger transposable element derived 2	0.91	0.88	1	0.71
CDH10	CDH10	cadherin 10, type 2 (T2-cadherin)	0.9	0.96	1	0.64
PRSS2	PRSS2;MGC111183;MGC120174;MGC120175;TRY2;TRY8;TRYP2	protease, serine, 2 (trypsin 2)	0.9	0.92	1	0.7
PROZ	PROZ;PZ	protein Z, vitamin K-dependent plasma glycoprotein	0.9	0.89	1	0.67
DIO3	DIO3;5DIII;D3;DIOIII;TXDI3	deiodinase, iodothyronine, type III	0.9	0.88	1	0.62
STH	STH;MAPTIT;MGC163191;MGC163193	saitohin	0.9	0.95	1	0.67
C6orf81	C6orf81;FLJ25390	chromosome 6 open reading frame 81	0.9	0.9	1	0.61
FOXD2	FOXD2;FKHL17;FREAC-9;FREAC9	forkhead box D2	0.9	0.84	1	0.74
C7orf31	C7orf31	chromosome 7 open reading frame 31	0.9	0.92	1	0.7

CDYL2	CDYL2;FLJ38866;PCCP1	chromodomain protein, Y-like 2	0.9	0.88	1	0.62
PSEN2	PSEN2;AD3L;AD4;CMD1V;PS2;STM2	presenilin 2 (Alzheimer disease 4)	0.9	0.88	1	0.63
KLHL22	KLHL22;KELCHL	kelch-like 22 (Drosophila)	0.9	0.94	1	0.67
CD99L2	CD99L2;CD99B;DKFZp761H2024;MIC2L1	CD99 molecule-like 2	0.9	0.86	1	0.68
REXO2	REXO2;DKFZp566E144;MGC111570;REX2;RFN;SFN	REX2, RNA exonuclease 2 homolog (S. cerevisiae)	0.9	1.01	1	0.69
FOLH1B	FOLH1B;FOLH2;FOLHP;GCP3;GCPIII;PSM;PSMA-LIKE;PSMAL	folate hydrolase 1B	0.9	0.89	1	0.67
PDE7B	PDE7B;MGC88256;bA472E5.1	phosphodiesterase 7B	0.89	0.9	1	0.7
PFDN4	PFDN4;C1;PFD4	prefoldin subunit 4	0.89	0.94	1	0.66
THOC6	THOC6;MGC2655;WDR58;fSAP35	THO complex 6 homolog (Drosophila)	0.89	0.89	1	0.64
IFRD1	IFRD1;PC4;TIS7	interferon-related developmental regulator 1	0.89	0.86	1	0.7
GAGE4	GAGE4;CT4.4	G antigen 4	0.88	0.9	1	0.69
SPINLW1	SPINLW1;CT71;CT72;EPPIN;WAP7;WFDC7;dJ461P17.2	serine peptidase inhibitor-like, with Kunitz and WAP domains 1 (eppin)	0.88	0.92	1	0.62
ENPP3	ENPP3;B10;CD203c;NPP3;PD-IBETA;PDNP3	ectonucleotide pyrophosphatase/phosphodiesterase 3	0.88	0.86	1	0.64
GAD2	GAD2;GAD65;MGC161605;MGC161607	glutamate decarboxylase 2 (pancreatic islets and brain, 65kDa)	0.88	0.88	1	0.68
INPP5D	INPP5D;MGC104855;MGC142140;MGC142142;SHIP;SHIP1;SIP-145;hp51CN	inositol polyphosphate-5-phosphatase, 145kDa	0.88	0.88	1	0.69
CCDC66	CCDC66;DKFZp686C0433	coiled-coil domain containing 66	0.88	0.85	1	0.65
MZF1	MZF1;MZF-1;MZF1B;ZNF42;ZSCAN6;Zfp98	myeloid zinc finger 1	0.88	0.84	1	0.61
ZFYVE20	ZFYVE20;FLJ34993;MGC126210;Rabenosyn-5	zinc finger, FYVE domain containing 20	0.88	0.9	1	0.65
UST	UST;2OST	uronyl-2-sulfotransferase	0.88	0.84	1	0.61
CRTC2	CRTC2;TORC-2;TORC2	CREB regulated transcription coactivator 2	0.88	0.87	1	0.7
RAD9A	RAD9A;RAD9	RAD9 homolog A (S. pombe)	0.88	0.9	1	0.71
SRD5A3	SRD5A3;CDG1P;CDG1Q;FLJ13352;SRD5A2L;SRD5A2L1	steroid 5 alpha-reductase 3	0.88	0.87	1	0.64
NKIRAS1	NKIRAS1;KBRAS1;kappaB-Ras1	NFKB inhibitor interacting Ras-like 1	0.88	0.85	1	0.6
TMPRSS4	TMPRSS4;CAPH2;MT-SP2;TMPRSS3	transmembrane protease, serine 4	0.88	0.86	1	0.65
RAB18	RAB18;RAB18LI1;WARBM3	RAB18, member RAS oncogene family	0.88	0.83	1	0.66
TAS2R20	TAS2R20;MGC119118;MGC119119;MGC119120;MGC119121;MGC134637;T2R20;T2R49;T2R56;TAS2R49	taste receptor, type 2, member 20	0.88	0.82	1	0.7
TAB1	TAB1;3'-Tab1;MAP3K7IP1;MGC57664	TGF-beta activated kinase 1/MAP3K7 binding protein 1	0.88	0.94	1	0.67
NLRP4	NLRP4;CLR19.5;CT58;FLJ32126;NALP4;PAN2;PYPAF4;RNH2	NLR family, pyrin domain containing 4	0.87	0.78	1	0.61
CFC1	CFC1;CFC1B;CRYPTIC;DTGA2;FLJ77897;HTX2;MGC133213	cripto, FRL-1, cryptic family 1	0.87	0.78	1	0.65
CEP68	CEP68;FLJ25920;FLJ36750;KIAA0582	centrosomal protein 68kDa	0.87	0.92	1	0.64
FBLIM1	FBLIM1;CAL;DKFZp434G171;FBLP-1;FBLP1	filamin binding LIM protein 1	0.87	0.94	1	0.72
HACL1	HACL1;2-HPCL;FLJ55041;HPCL;HPCL2;PHYH2	2-hydroxyacyl-CoA lyase 1	0.87	0.81	1	0.64
FAM82B	FAM82B;FLJ20665;RMD-1;RMD1	family with sequence similarity 82, member B	0.87	0.85	1	0.64
ANKRD40	ANKRD40;DKFZp451K241;MGC15396	ankyrin repeat domain 40	0.87	0.93	1	0.72
FFAR2	FFAR2;FFA2R;GPR43	free fatty acid receptor 2	0.86	0.83	1	0.75
ODF3	ODF3;CT135;SHIPPO1;TISP50;hSHIPPO	outer dense fiber of sperm tails 3	0.86	0.86	1	0.6
CHST1	CHST1;C6ST;GST-1;KS6ST;KSGal6ST;KSST	carbohydrate (keratan sulfate Gal-6) sulfotransferase 1	0.86	0.92	1	0.69
ALKBH8	ALKBH8;ABH8;FLJ38204;MGC10235	alkB, alkylation repair homolog 8 (E. coli)	0.86	0.91	1	0.64
RELL2	RELL2;C5orf16;FLJ90583	RELT-like 2	0.86	0.88	1	0.6
PGLYRP1	PGLYRP1;MGC126894;MGC126896;PGLYRP;PGRP;PGRP-S;PGRPS;TAG7;TNFSF3L	peptidoglycan recognition protein 1	0.86	0.84	1	0.65
PDE6B	PDE6B;CSNB3;CSNBAD2;PDEB;RP40;rd1	phosphodiesterase 6B, cGMP-specific, rod, beta	0.86	0.86	1	0.58
ASB7	ASB7;FLJ22551;FLJ25192	ankyrin repeat and SOCS box containing 7	0.86	0.82	1	0.59
ZFAND2B	ZFAND2B;AIRAPL	zinc finger, AN1-type domain 2B	0.86	0.9	1	0.7
ATP5D	ATP5D	ATP synthase, H+ transporting, mitochondrial F1 complex, delta subunit	0.86	0.84	1	0.68
FDPS	FDPS;FPPS;FPS	farnesyl diphosphate synthase	0.86	0.8	1	0.7
OR7C1	OR7C1;CIT-HSP-146E8;HSTPCR86P;OR19-5;OR7C4;TPCR86	olfactory receptor, family 7, subfamily C, member 1	0.85	0.89	1	0.67
AIFM2	AIFM2;AMID;DKFZp686L1298;PRG3;RP11-367H5.2	apoptosis-inducing factor, mitochondrion-associated, 2	0.85	0.88	1	0.69
TCN2	TCN2;D22S676;D22S750;II;TC;TC II;TC-2;TC2;TCII	transcobalamin II	0.85	0.82	1	0.71
PHF1	PHF1;MTF2L2;PCL1;PHF2	PHD finger protein 1	0.85	0.84	1	0.75
ZNF766	ZNF766	zinc finger protein 766	0.85	0.83	1	0.62
AGRN	AGRN;FLJ45064	agrin	0.85	0.76	1	0.64
ANKH	ANKH;ANK;CCAL2;CMDJ;CPPDD;FLJ27166;HANK;MANK	ankylosis, progressive homolog (mouse)	0.85	0.8	1	0.63
TSNAX	TSNAX;TRAX	translin-associated factor X	0.85	0.82	1	0.59
IL32	IL32;IL-32alpha;IL-32beta;IL-32delta;IL-32gamma;NK4;TAIF;TAIFa;TAIFb;TAIFc;TAIFd	interleukin 32	1.25	1.19	1.01	0.97
CTSD	CTSD;CLN10;CPSD;MGC2311	cathepsin D	1.25	1.17	1.01	0.87
TSPAN1	TSPAN1;NET1;TM4C;TM4SF	tetraspanin 1	1.23	1.36	1.01	0.91
MMEL1	MMEL1;MGC119454;MGC119455;MGC119456;MMEL2;NEP2;NEPII;NL1;NL2;SEP	membrane metallo-endopeptidase-like 1	1.22	1.13	1.01	0.85
MT4	MT4;MT-4;MT-IV;MTIV	metallothionein 4	1.21	1.2	1.01	0.96
ADAM11	ADAM11;MDC	ADAM metallopeptidase domain 11	1.21	1.13	1.01	0.84
C8orf38	C8orf38;MGC40214	chromosome 8 open reading frame 38	1.21	1.28	1.01	0.92
SCAPER	SCAPER;FLJ31533;FLJ31953;FLJ46212;KIAA1454;ZNF291;Zfp291	S-phase cyclin A-associated protein in the ER	1.21	1.29	1.01	0.91
MMP20	MMP20;AI2A2;MMP-20	matrix metallopeptidase 20	1.2	1.12	1.01	0.84
STUB1	STUB1;CHIP;HSPABP2;NY-CO-7;SDCCAG7;UBOX1	STIP1 homology and U-box containing protein 1, E3 ubiquitin protein ligase	1.19	1.15	1.01	0.83
KIAA1377	KIAA1377	KIAA1377	1.16	1.23	1.01	0.87
CATSPER1	CATSPER1;CATSPER;MGC33335;MGC33368;SPGF7	cation channel, sperm associated 1	1.16	1.22	1.01	0.94
NAV1	NAV1;DKFZp781D0314;FLJ12560;FLJ14203;KIAA1151;MGC14961;POMFIL3;STEERIN1;UNC53H1	neuron navigator 1	1.14	1.16	1.01	0.92
UBR2	UBR2;C6orf133;DKFZp686C08114;FLJ36357;KIAA0349;MGC71112;RP3-392M17.3;bA49A4.1;dJ242G1.1;dJ392M17.3	ubiquitin protein ligase E3 component n-recognin 2	1.13	1.09	1.01	0.79
ZNF585A	ZNF585A;FLJ23765;FLJ25829;FLJ31827	zinc finger protein 585A	1.12	1.02	1.01	0.85
ADORA2B	ADORA2B;ADORA2	adenosine A2b receptor	1.12	1.11	1.01	1
ACTA2	ACTA2;AAT6;ACTSA;MYMY5	actin, alpha 2, smooth muscle, aorta	1.12	1.09	1.01	0.78
PCDHA3	PCDHA3;MGC141669;PCDH-ALPHA3	protocadherin alpha 3	1.12	1.18	1.01	0.88
SPERT	SPERT;CBY2;FLJ35810;NURIT	spermatid associated	1.11	1.19	1.01	0.84
MALT1	MALT1;DKFZp434L132;MLT;MLT1	mucosa associated lymphoid tissue lymphoma translocation gene 1	1.11	1.03	1.01	0.77
DNM2	DNM2;CMT2M;CMTDI1;CMTDIB;DI-CMTB;DYN2;DYNII	dynamin 2	1.1	1.18	1.01	0.82
CHD2	CHD2;DKFZp547I1315;DKFZp686E01200;DKFZp781D1727;FLJ38614	chromodomain helicase DNA binding protein 2	1.09	1.08	1.01	0.77
STOML2	STOML2;SLP-2	stomatin (EPB72)-like 2	1.09	0.98	1.01	0.82
CD248	CD248;CD164L1;MGC119478;MGC119479;TEM1	CD248 molecule, endosialin	1.08	1.13	1.01	0.84
TRPM5	TRPM5;LTRPC5;MTR1	transient receptor potential cation channel, subfamily M, member 5	1.08	1.08	1.01	0.85
CCDC155	CCDC155;DKFZp434A2223;FLJ32658	coiled-coil domain containing 155	1.08	1.04	1.01	0.87
AFAP1L2	AFAP1L2;FLJ14564;KIAA1914;XB130	actin filament associated protein 1-like 2	1.08	1.06	1.01	0.79
SMYD4	SMYD4;KIAA1936;ZMYND21	SET and MYND domain containing 4	1.07	1.05	1.01	0.83
S100A13	S100A13	S100 calcium binding protein A13	1.07	1.05	1.01	0.8
HKDC1	HKDC1;FLJ22761;FLJ37767;MGC125688	hexokinase domain containing 1	1.07	1	1.01	0.88
CD33	CD33;FLJ00391;SIGLEC-3;SIGLEC3;p67	CD33 molecule	1.06	0.96	1.01	0.8
TRIM14	TRIM14;KIAA0129	tripartite motif containing 14	1.06	1.02	1.01	0.74
APIP	APIP;APIP2;CGI29;MMRP19;dJ179L10.2	APAF1 interacting protein	1.06	0.96	1.01	0.75
ATG4A	ATG4A;APG4A;AUTL2	ATG4 autophagy related 4 homolog A (S. cerevisiae)	1.06	1.06	1.01	0.79
MXRA5	MXRA5;DKFZp564I1922	matrix-remodelling associated 5	1.06	1.13	1.01	0.8
HLA-DMA	HLA-DMA;D6S222E;DMA;HLADM;RING6	major histocompatibility complex, class II, DM alpha	1.04	1.02	1.01	0.72
IL28A	IL28A;IFNL2;IL-28A	interleukin 28A (interferon, lambda 2)	1.04	0.95	1.01	0.79
TMEM98	TMEM98;DKFZp564K1964	transmembrane protein 98	1.04	1.09	1.01	0.81
SPSB1	SPSB1;SSB-1;SSB1	splA/ryanodine receptor domain and SOCS box containing 1	1.04	1.01	1.01	0.73
C12orf49	C12orf49;FLJ21415	chromosome 12 open reading frame 49	1.04	1	1.01	0.81
CTSL1	CTSL1;CATL;CTSL;FLJ31037;MEP	cathepsin L1	1.04	0.96	1.01	0.72
DCP2	DCP2;FLJ33245;NUDT20	DCP2 decapping enzyme homolog (S. cerevisiae)	1.04	0.95	1.01	0.79
PPT1	PPT1;CLN1;INCL;PPT	palmitoyl-protein thioesterase 1	1.04	1.03	1.01	0.72
PLA2G15	PLA2G15;ACS;DKFZp564A0122;GXVPLA2;LLPL;LPLA2;LYPLA3	phospholipase A2, group XV	1.04	0.98	1.01	0.78
FPGT	FPGT;GFPP	fucose-1-phosphate guanylyltransferase	1.03	1.15	1.01	0.8
ZNF800	ZNF800;FLJ43301;MGC130032;MGC130033	zinc finger protein 800	1.03	1.01	1.01	0.77
TRPC4AP	TRPC4AP;C20orf188;TRRP4AP;TRUSS	transient receptor potential cation channel, subfamily C, member 4 associated protein	1.03	1.01	1.01	0.71
CCR5	CCR5;CC-CKR-5;CCCKR5;CD195;CKR-5;CKR5;CMKBR5;FLJ78003;IDDM22	chemokine (C-C motif) receptor 5	1.02	1	1.01	0.89
PAG1	PAG1;CBP;FLJ37858;MGC138364;PAG	phosphoprotein associated with glycosphingolipid microdomains 1	1.02	1.07	1.01	0.77
CNTLN	CNTLN;C9orf101;C9orf39;FLJ20276;FLJ25636;RP11-340N12.1;bA340N12.1	centlein, centrosomal protein	1.02	0.94	1.01	0.74
TRIM61	TRIM61;FLJ60274;MGC102996;RNF35	tripartite motif containing 61	1.02	1.02	1.01	0.91
LAMA1	LAMA1;LAMA;S-LAM-alpha	laminin, alpha 1	1.02	1.08	1.01	0.72
FGGY	FGGY;FLJ10986	FGGY carbohydrate kinase domain containing	1.02	1.02	1.01	0.69
PDPN	PDPN;AGGRUS;GP36;GP40;Gp38;HT1A-1;OTS8;PA2.26;T1A;T1A-2	podoplanin	1.02	0.99	1.01	0.75
WDR92	WDR92;FLJ31741	WD repeat domain 92	1.02	1	1.01	0.76
LRP12	LRP12;DKFZp781F1053;FLJ12929;ST7	low density lipoprotein receptor-related protein 12	1.02	0.94	1.01	0.71
AGGF1	AGGF1;FLJ10283;GPATC7;GPATCH7;HSU84971;HUS84971;VG5Q	angiogenic factor with G patch and FHA domains 1	1.02	1.11	1.01	0.77
CLNS1A	CLNS1A;CLCI;CLNS1B;ICln	chloride channel, nucleotide-sensitive, 1A	1.02	1.02	1.01	0.8
PIP4K2C	PIP4K2C;FLJ22055;PIP5K2C	phosphatidylinositol-5-phosphate 4-kinase, type II, gamma	1.02	1.02	1.01	0.85
C10orf67	C10orf67;MGC46732;RP11-792P23.2	chromosome 10 open reading frame 67	1.01	1.07	1.01	0.75
NACA2	NACA2;ANAC;MGC71999;NACAL	nascent polypeptide-associated complex alpha subunit 2	1.01	1.09	1.01	0.76
KCNC3	KCNC3;KSHIIID;KV3.3;SCA13	potassium voltage-gated channel, Shaw-related subfamily, member 3	1.01	1.08	1.01	0.84
MREG	MREG;DSU;FLJ10116;MGC90296;WDT2	melanoregulin	1.01	1.06	1.01	0.76
HAMP	HAMP;HEPC;HFE2B;LEAP1;PLTR	hepcidin antimicrobial peptide	1	1.05	1.01	0.8
KLK1	KLK1;KLKR;Klk6;hK1	kallikrein 1	1	0.93	1.01	0.7
SLC22A17	SLC22A17;24p3R;BOCT;BOIT;NGALR;NGALR2;NGALR3;hBOIT	solute carrier family 22, member 17	1	0.99	1.01	0.69
LPHN1	LPHN1;CIRL1;CL1;LEC2	latrophilin 1	1	1	1.01	0.89
TPM3	TPM3;FLJ41118;MGC14582;MGC3261;MGC72094;NEM1;OK/SW-cl.5;TM-5;TM3;TM30;TM30nm;TM5;TPMsk3;TRK;hscp30	tropomyosin 3	1	0.98	1.01	0.78
CAP1	CAP1;CAP;CAP1-PEN	CAP, adenylate cyclase-associated protein 1 (yeast)	1	1.01	1.01	0.7
PCDHGB7	PCDHGB7;ME6;PCDH-GAMMA-B7	protocadherin gamma subfamily B, 7	1	1.04	1.01	0.75
HSD17B1	HSD17B1;EDH17B2;EDHB17;HSD17;MGC138140;SDR28C1	hydroxysteroid (17-beta) dehydrogenase 1	0.99	0.96	1.01	0.69
BSDC1	BSDC1;DKFZp686B09139;FLJ10276	BSD domain containing 1	0.99	0.88	1.01	0.69
OR5AU1	OR5AU1;OR14-38	olfactory receptor, family 5, subfamily AU, member 1	0.98	0.96	1.01	0.77
C4orf32	C4orf32;FLJ39370	chromosome 4 open reading frame 32	0.98	0.98	1.01	0.71
SLC25A43	SLC25A43	solute carrier family 25, member 43	0.98	0.96	1.01	0.83
KIAA0753	KIAA0753;MGC130040;MGC130041	KIAA0753	0.98	1	1.01	0.7

VPS26B	VPS26B;MGC10485;Pep8b	vacuolar protein sorting 26 homolog B (S. pombe)	0.98	0.94	1.01	0.72
TRIL	TRIL;KIAA0644	TLR4 interactor with leucine-rich repeats	0.98	0.98	1.01	0.88
DPYD	DPYD;DHP;DHPDHASE;DPD;MGC132008;MGC70799	dihydropyrimidine dehydrogenase	0.97	0.98	1.01	0.66
NFKBIE	NFKBIE;IKBE	nuclear factor of kappa light polypeptide gene enhancer in B-cells inhibitor, epsilon	0.97	0.96	1.01	0.71
ANKRD10	ANKRD10;DKFZp686B07190;FLJ20093	ankyrin repeat domain 10	0.97	1.02	1.01	0.77
RP2	RP2;DELXp11.3;KIAA0215;NME10;TBCCD2;XRP2	retinitis pigmentosa 2 (X-linked recessive)	0.97	0.94	1.01	0.68
GSN	GSN;ADF;AGEL;DKFZp313L0718	gelsolin	0.97	0.93	1.01	0.79
MELK	MELK;HPK38;KIAA0175	maternal embryonic leucine zipper kinase	0.97	1.05	1.01	0.75
CELF4	CELF4;BRUNOL-4;BRUNOL4	CUGBP, Elav-like family member 4	0.97	0.94	1.01	0.68
CT45A6	CT45A6;CT45-6;CT45.6;CT45A4	cancer/testis antigen family 45, member A6	0.97	0.92	1.01	0.78
TRPV6	TRPV6;ABP/ZF;CAT1;CATL;ECAC2;HSA277909;LP6728;ZFAB	transient receptor potential cation channel, subfamily V, member 6	0.96	0.96	1.01	0.75
TNFAIP1	TNFAIP1;B12;B61;EDP1;MGC2317;hBACURD2	tumor necrosis factor, alpha-induced protein 1 (endothelial)	0.96	1.02	1.01	0.8
SFN	SFN;YWHAS	stratifin	0.96	1	1.01	0.75
CACYBP	CACYBP;GIG5;MGC87971;RP1-102G20.6;S100A6BP;SIP	calcyclin binding protein	0.96	1.04	1.01	0.8
PRDM11	PRDM11;PFM8	PR domain containing 11	0.95	0.98	1.01	0.78
PDK3	PDK3	pyruvate dehydrogenase kinase, isozyme 3	0.95	0.95	1.01	0.79
SAP30	SAP30	Sin3A-associated protein, 30kDa	0.95	0.94	1.01	0.73
SFMBT1	SFMBT1;DKFZp434L243;RU1;SFMBT	Scm-like with four mbt domains 1	0.95	0.97	1.01	0.67
NOS2	NOS2;HEP-NOS;INOS;NOS;NOS2A	nitric oxide synthase 2, inducible	0.95	1.02	1.01	0.67
ATP10B	ATP10B;ATPVB;FLJ21477;KIAA0715	ATPase, class V, type 10B	0.94	0.95	1.01	0.74
FAM70A	FAM70A;FLJ20716	family with sequence similarity 70, member A	0.94	0.94	1.01	0.68
OR51V1	OR51V1;OR11-36;OR51A12	olfactory receptor, family 51, subfamily V, member 1	0.94	0.94	1.01	0.64
DOCK3	DOCK3;KIAA0299;MOCA;PBP	dedicator of cytokinesis 3	0.94	0.92	1.01	0.74
SRPX2	SRPX2;BPP;CBPS;PMGX;RESDX;SRPUL	sushi-repeat containing protein, X-linked 2	0.94	0.9	1.01	0.73
SLC9A3	SLC9A3;MGC126718;MGC126720;NHE3	solute carrier family 9 (sodium/hydrogen exchanger), member 3	0.94	1	1.01	0.77
HOXD4	HOXD4;HHO.C13;HOX-5.1;HOX4;HOX4B;Hox-4.2	homeobox D4	0.94	0.92	1.01	0.65
EXTL3	EXTL3;BOTV;DKFZp686C2342;EXTR1;KIAA0519;REG;REGR;RPR	exostoses (multiple)-like 3	0.94	0.92	1.01	0.66
CMIP	CMIP;KIAA1694;TCMIP	c-Maf inducing protein	0.94	0.94	1.01	0.64
SEMA3A	SEMA3A;Hsema-I;Hsema-III;MGC133243;SEMA1;SEMAD;SEMAIII;SEMAL;SemD;coll-1	sema domain, immunoglobulin domain (Ig), short basic domain, secreted, (semaphorin) 3A	0.94	1	1.01	0.8
DOCK7	DOCK7;KIAA1771;ZIR2	dedicator of cytokinesis 7	0.94	0.9	1.01	0.69
ARL2BP	ARL2BP;BART;BART1	ADP-ribosylation factor-like 2 binding protein	0.94	0.99	1.01	0.66
C1QBP	C1QBP;GC1QBP;HABP1;SF2p32;gC1Q-R;gC1qR;p32	complement component 1, q subcomponent binding protein	0.94	1	1.01	0.67
FAM13C	FAM13C;FAM13C1;MGC33233	family with sequence similarity 13, member C	0.94	0.96	1.01	0.65
GNRH1	GNRH1;GNRH;GRH;LHRH;LNRH	gonadotropin-releasing hormone 1 (luteinizing-releasing hormone)	0.93	0.96	1.01	0.72
MIP	MIP;AQP0;LIM1;MIP26;MP26	major intrinsic protein of lens fiber	0.93	0.97	1.01	0.76
C1orf135	C1orf135;AIBP;FLJ14264;MGC2603	chromosome 1 open reading frame 135	0.93	0.92	1.01	0.69
EMILIN2	EMILIN2;EMILIN-2;FLJ33200;FOAP-10	elastin microfibril interfacer 2	0.93	0.88	1.01	0.75
PRB4	PRB4;Po	proline-rich protein BstNI subfamily 4	0.92	0.9	1.01	0.68
CYP3A5	CYP3A5;CP35;CYPIIIA5;DKFZp686L16231;FLJ31317;P450PCN3;PCN3	cytochrome P450, family 3, subfamily A, polypeptide 5	0.92	0.98	1.01	0.79
LHFPL1	LHFPL1;MGC118798;MGC118800;MGC118801	lipoma HMGIC fusion partner-like 1	0.92	0.88	1.01	0.75
ACHE	ACHE;ACEE;ARACHE;N-ACHE;YT	acetylcholinesterase	0.92	0.9	1.01	0.78
TSPAN9	TSPAN9;NET-5;NET5;PP1057	tetraspanin 9	0.92	0.9	1.01	0.68
NRSN2	NRSN2;C20orf98;FLJ23329;dJ1103G7.6	neurensin 2	0.92	1.01	1.01	0.74
XPA	XPA;XP1;XPAC	xeroderma pigmentosum, complementation group A	0.92	0.91	1.01	0.64
SRRD	SRRD;HC/HCC;SRR1L	SRR1 domain containing	0.92	0.84	1.01	0.65
MYBBP1A	MYBBP1A;FLJ37886;P160;PAP2	MYB binding protein (P160) 1a	0.92	0.96	1.01	0.76
ARF6	ARF6;DKFZp564M0264	ADP-ribosylation factor 6	0.92	0.92	1.01	0.68
NECAP2	NECAP2;FLJ10420	NECAP endocytosis associated 2	0.92	0.9	1.01	0.71
MFSD6	MFSD6;FLJ20160;MMR2;hMMR2	major facilitator superfamily domain containing 6	0.92	0.96	1.01	0.72
RALGAPA2	RALGAPA2;AS250;C20orf74;FLJ12819;KIAA1272;bA287B20.1;dJ1049G11;dJ1049G11.4	Ral GTPase activating protein, alpha subunit 2 (catalytic)	0.92	0.98	1.01	0.69
LRP2	LRP2;DBS;GP330	low density lipoprotein receptor-related protein 2	0.91	0.92	1.01	0.62
PPARG	PPARG;CIMT1;GLM1;NR1C3;PPARG1;PPARG2;PPARgamma	peroxisome proliferator-activated receptor gamma	0.91	0.92	1.01	0.71
KIAA1984	KIAA1984;MGC15438;PARF;RP11-216L13.9;bA216L13.7	KIAA1984	0.91	0.89	1.01	0.67
FAM133B	FAM133B;MGC40405	family with sequence similarity 133, member B	0.91	0.96	1.01	0.69
SPTLC1	SPTLC1;HSAN;HSAN1;HSN1;LBC1;LCB1;MGC14645;SPT1;SPTI;hLCB1	serine palmitoyltransferase, long chain base subunit 1	0.91	1	1.01	0.75
RSPH9	RSPH9;C6orf206;CILD12;FLJ30845;MRPS18AL1	radial spoke head 9 homolog (Chlamydomonas)	0.91	0.88	1.01	0.67
OR2C3	OR2C3;OR2C4;OR2C5P;OST742	olfactory receptor, family 2, subfamily C, member 3	0.9	0.86	1.01	0.72
GPR77	GPR77;C5L2;GPF77	G protein-coupled receptor 77	0.9	0.85	1.01	0.64
TUBA3D	TUBA3D;H2-ALPHA;TUBA3C	tubulin, alpha 3d	0.9	0.94	1.01	0.7
GBX2	GBX2	gastrulation brain homeobox 2	0.9	0.91	1.01	0.71
TOLLIP	TOLLIP;FLJ33531;IL-1RAcPIP	toll interacting protein	0.9	0.88	1.01	0.71
C11orf57	C11orf57;FLJ10726	chromosome 11 open reading frame 57	0.9	0.98	1.01	0.76
TCEAL4	TCEAL4;DKFZp686M0279;FLJ21174;NPD017	transcription elongation factor A (SII)-like 4	0.9	0.97	1.01	0.75
SKI	SKI;SKV	v-ski sarcoma viral oncogene homolog (avian)	0.9	0.96	1.01	0.67
CNOT4	CNOT4;CLONE243;NOT4;NOT4H	CCR4-NOT transcription complex, subunit 4	0.9	0.9	1.01	0.81
SUV420H1	SUV420H1;CGI85;KMT5B;MGC118906;MGC118909;MGC21161;MGC703	suppressor of variegation 4-20 homolog 1 (Drosophila)	0.9	0.92	1.01	0.62
SCAMP2	SCAMP2	secretory carrier membrane protein 2	0.9	0.99	1.01	0.74
HNRNPA0	HNRNPA0;HNRPA0	heterogeneous nuclear ribonucleoprotein A0	0.9	0.9	1.01	0.68
ACOT13	ACOT13;MGC4961;PNAS-27;THEM2	acyl-CoA thioesterase 13	0.9	0.9	1.01	0.67
B3GNT4	B3GNT4;B3GN-T4;beta3Gn-T4	UDP-GlcNAc:betaGal beta-1,3-N-acetylglucosaminyltransferase 4	0.89	0.87	1.01	0.64
GJD2	GJD2;CX36;GJA9;MGC138315;MGC138319	gap junction protein, delta 2, 36kDa	0.89	0.94	1.01	0.67
SH3BP5	SH3BP5;SAB	SH3-domain binding protein 5 (BTK-associated)	0.89	0.86	1.01	0.66
FAM101B	FAM101B;MGC45871	family with sequence similarity 101, member B	0.89	0.93	1.01	0.67
FGF9	FGF9;GAF;HBFG-9;MGC119914;MGC119915;SYNS3	fibroblast growth factor 9 (glia-activating factor)	0.89	0.88	1.01	0.65
MARVELD2	MARVELD2;DFNB49;FLJ30532;MARVD2;MRVLDC2;Tric	MARVEL domain containing 2	0.89	0.85	1.01	0.72
GLUD2	GLUD2;GDH2;GLUDP1	glutamate dehydrogenase 2	0.88	0.94	1.01	0.61
OR6Y1	OR6Y1;OR1-11;OR6Y2	olfactory receptor, family 6, subfamily Y, member 1	0.88	0.92	1.01	0.61
C9orf43	C9orf43;MGC17358	chromosome 9 open reading frame 43	0.88	0.86	1.01	0.68
POLN	POLN;POL4P	polymerase (DNA directed) nu	0.88	0.9	1.01	0.6
ATP2A1	ATP2A1;ATP2A;SERCA1	ATPase, Ca++ transporting, cardiac muscle, fast twitch 1	0.88	0.87	1.01	0.61
GOLGA6B	GOLGA6B;GOLGA;GOLGA6D	golgin A6 family, member B	0.88	0.92	1.01	0.67
TUBE1	TUBE1;FLJ22589;FLJ44203;TUBE;dJ142L7.2	tubulin, epsilon 1	0.88	0.84	1.01	0.66
STX11	STX11;FHL4;HLH4;HPLH4	syntaxin 11	0.88	0.91	1.01	0.61
SLC7A10	SLC7A10;FLJ20839;HASC-1;asc-1	solute carrier family 7 (neutral amino acid transporter light chain, asc system), member 10	0.88	0.88	1.01	0.68
HIST1H4L	HIST1H4L;H4.k;H4/k;H4FK;HIST1H4A;HIST1H4B;HIST1H4C;HIST1H4D;HIST1H4E;HIST1H4F;HIST1H4H;HIST1H4I;HIST1H4J;HIST1H4K;HIST2H4A;HIST2H4B;HIST4H4	histone cluster 1, H4l	0.88	0.91	1.01	0.61
MMS19	MMS19;FLJ34167;FLJ95146;MET18;MGC99604;MMS19L;hMMS19	MMS19 nucleotide excision repair homolog (S. cerevisiae)	0.88	0.9	1.01	0.61
NDUFS1	NDUFS1;CI-75Kd;CI-75k;MGC26839;PRO1304	NADH dehydrogenase (ubiquinone) Fe-S protein 1, 75kDa (NADH-coenzyme Q reductase)	0.88	0.86	1.01	0.64
SFR1	SFR1;C10orf78;FLJ41960;MEI5;MEIR5;bA373N18.1	SWI5-dependent recombination repair 1	0.88	0.87	1.01	0.72
SRSF12	SRSF12;FLJ14459;FLJ33484;FLJ41221;RP11-63L7.3;SFRS13B;SFRS19;SRrp35	serine/arginine-rich splicing factor 12	0.88	0.88	1.01	0.73
INSL6	INSL6;RIF1	insulin-like 6	0.87	0.82	1.01	0.7
CASP1	CASP1;ICE;IL1BC;P45	caspase 1, apoptosis-related cysteine peptidase (interleukin 1, beta, convertase)	0.87	0.87	1.01	0.65
OR51I2	OR51I2;OR11-38	olfactory receptor, family 51, subfamily I, member 2	0.87	0.86	1.01	0.67
PRKAR2A	PRKAR2A;MGC3606;PKR2;PRKAR2	protein kinase, cAMP-dependent, regulatory, type II, alpha	0.87	0.87	1.01	0.72
PLD4	PLD4;C14orf175	phospholipase D family, member 4	0.86	0.84	1.01	0.62
COQ3	COQ3;DHHBMT;DHHBMTASE;UG0215E05;bA9819.1	coenzyme Q3 homolog, methyltransferase (S. cerevisiae)	0.86	0.88	1.01	0.7
SLC38A7	SLC38A7;FLJ10815;FLJ12724;SNAT7	solute carrier family 38, member 7	0.86	0.84	1.01	0.61
FGFR4	FGFR4;CD334;JTK2;MGC20292;TKF	fibroblast growth factor receptor 4	0.86	0.94	1.01	0.67
NAT14	NAT14;KLP1	N-acetyltransferase 14 (GCN5-related, putative)	0.86	0.86	1.01	0.63
KIAA1804	KIAA1804;MLK4;RP5-862P8.2;dJ862P8.3	mixed lineage kinase 4	0.86	0.87	1.01	0.72
C21orf59	C21orf59;C21orf48;FLJ20467;FLJ37137;FLJ40247	chromosome 21 open reading frame 59	0.86	0.96	1.01	0.7
CBFB	CBFB;PEBP2B	core-binding factor, beta subunit	0.86	0.85	1.01	0.66
PSENEN	PSENEN;MSTP064;PEN-2;PEN2	presenilin enhancer 2 homolog (C. elegans)	0.86	0.91	1.01	0.69
C7orf68	C7orf68;FLJ21076;HIG-2;HIG2;MGC138388	chromosome 7 open reading frame 68	0.86	0.86	1.01	0.72
PRR21	PRR21	proline rich 21	0.86	0.82	1.01	0.69
AP3M1	AP3M1;MGC22164	adaptor-related protein complex 3, mu 1 subunit	0.85	0.82	1.01	0.59
ZNF431	ZNF431;DKFZp313E0830;KIAA1969	zinc finger protein 431	1.37	1.25	1.02	1.05
MYOD1	MYOD1;MYF3;MYOD;PUM;bHLHc1	myogenic differentiation 1	1.35	1.31	1.02	0.95
SLC25A5	SLC25A5;2F1;AAC2;ANT2;T2;T3	solute carrier family 25 (mitochondrial carrier; adenine nucleotide translocator), member 5	1.28	1.44	1.02	0.99
MMP10	MMP10;SL-2;STMY2	matrix metallopeptidase 10 (stromelysin 2)	1.25	1.27	1.02	0.95
SSPO	SSPO;FLJ36112;FLJ45737;KIAA2036;SCO-spondin	SCO-spondin homolog (Bos taurus)	1.22	1.29	1.02	0.93
NCEH1	NCEH1;AADACL1;NCEH	neutral cholesterol ester hydrolase 1	1.21	1.19	1.02	0.86
PLEKHF2	PLEKHF2;FLJ13187;PHAFIN2;ZFYVE18	pleckstrin homology domain containing, family F (with FYVE domain) member 2	1.19	1.25	1.02	0.94
PCDHGA2	PCDHGA2;PCDH-GAMMA-A2	protocadherin gamma subfamily A, 2	1.17	1.24	1.02	0.83
PJA1	PJA1;PRAJA1;RNF70	praja ring finger 1	1.16	1.13	1.02	0.82
CCNF	CCNF;FBX1;FBXO1	cyclin F	1.16	1.13	1.02	0.82
SPOCK3	SPOCK3;HSAJ1454;TES-3;TICN3	sparc/osteonectin, cwcv and kazal-like domains proteoglycan (testican) 3	1.14	1.04	1.02	0.87
TRPC4	TRPC4;HTRP-4;HTRP4;MGC119570;MGC119571;MGC119572;MGC119573;TRP4	transient receptor potential cation channel, subfamily C, member 4	1.14	1.15	1.02	0.9
CACNG4	CACNG4;MGC11138;MGC24983	calcium channel, voltage-dependent, gamma subunit 4	1.14	1.15	1.02	0.89
DIRC2	DIRC2;FLJ14784;RCC4	disrupted in renal carcinoma 2	1.14	1.12	1.02	0.88
HLA-DRB4	HLA-DRB4;DRB4;HLA-DR4B	major histocompatibility complex, class II, DR beta 4	1.13	1.04	1.02	0.83
TOB2	TOB2;TOB4;TOBL;TROB2	transducer of ERBB2, 2	1.12	1.14	1.02	0.77
LGMN	LGMN;AEP;LGMN1;PRSC1	legumain	1.12	1.04	1.02	0.78
ESD	ESD;FGH;FLJ11763	esterase D	1.1	1.14	1.02	0.76
APBB1IP	APBB1IP;INAG1;PREL1;RARP1;RIAM	amyloid beta (A4) precursor protein-binding, family B, member 1 interacting protein	1.09	1.14	1.02	0.87
FUT2	FUT2;B12QTL1;SE;SEC2;Se2;sej	fucosyltransferase 2 (secretor status included)	1.09	1.08	1.02	0.77
ZNRF4	ZNRF4;RNF204;SPERIZIN;Ssrzf1;spzn	zinc and ring finger 4	1.09	1.07	1.02	0.77
XPR1	XPR1;FLJ90308;SYG1;X3	xenotropic and polytropic retrovirus receptor 1	1.09	1.05	1.02	0.79
VCY	VCY;BPY1;MGC119817;VCY1;VCY1A	variable charge, Y-linked	1.08	1.07	1.02	0.77
FBXO45	FBXO45;Fbx45	F-box protein 45	1.08	1.05	1.02	0.76

GK	GK;GK1;GKD	glycerol kinase	1.08	1.1	1.02	0.91
ICK	ICK;ECO;KIAA0936;LCK2;MGC46090;MRK	intestinal cell (MAK-like) kinase	1.07	1.17	1.02	0.84
MAGEA9B	MAGEA9B;MAGEA9	melanoma antigen family A, 9B	1.07	1.15	1.02	0.93
MYLK3	MYLK3;MGC126319;MGC126320;MLCK;MLCK2;caMLCK	myosin light chain kinase 3	1.06	1.08	1.02	0.9
ENPP5	ENPP5;KIAA0879	ectonucleotide pyrophosphatase/phosphodiesterase 5 (putative)	1.06	1.06	1.02	0.82
HIST1H4A	HIST1H4A;H4/a;H4FA	histone cluster 1, H4a	1.06	1.12	1.02	0.77
37316	MARCH2;MARCH-II;RNF172	membrane-associated ring finger (C3HC4) 2	1.06	1.04	1.02	0.75
TMEFF1	TMEFF1;C9orf2;CT120.1;H7365;TR-1	transmembrane protein with EGF-like and two follistatin-like domains 1	1.06	1.12	1.02	0.76
MTNR1A	MTNR1A;MEL-1A-R;MT1	melatonin receptor 1A	1.05	1	1.02	0.75
RAC3	RAC3	ras-related C3 botulinum toxin substrate 3 (rho family, small GTP binding protein Rac3)	1.05	1.08	1.02	0.73
ZNF346	ZNF346;DKFZp547M223;JAZ;Zfp346	zinc finger protein 346	1.05	1.05	1.02	0.88
YLPM1	YLPM1;C14orf170;ZAP113;ZAP3	YLP motif containing 1	1.05	1.1	1.02	0.74
ZNF626	ZNF626;MGC104224	zinc finger protein 626	1.04	0.96	1.02	0.8
FUZ	FUZ;FLJ22688;FY	fuzzy homolog (Drosophila)	1.04	1.15	1.02	0.77
EID2	EID2;CRI2;EID-2;MGC20452	EP300 interacting inhibitor of differentiation 2	1.04	0.98	1.02	0.73
OR5R1	OR5R1;OR11-185;OR5R1P	olfactory receptor, family 5, subfamily R, member 1	1.03	1.05	1.02	0.71
ADAM23	ADAM23;MDC3	ADAM metallopeptidase domain 23	1.03	1.04	1.02	0.78
CHD5	CHD5;DKFZp434N231;KIAA0444	chromodomain helicase DNA binding protein 5	1.03	1	1.02	0.72
GARNL3	GARNL3;DKFZp434O131;DKFZp761J1523;FLJ38360;bA356B19.1	GTPase activating Rap/RanGAP domain-like 3	1.03	0.99	1.02	0.84
HCRTR1	HCRTR1;OX1R	hypocretin (orexin) receptor 1	1.03	1.02	1.02	0.92
C3AR1	C3AR1;AZ3B;C3AR;HNFAG09	complement component 3a receptor 1	1.03	0.98	1.02	0.74
ALOX5	ALOX5;5-LO;5-LOX;5LPG;LOG5;MGC163204	arachidonate 5-lipoxygenase	1.02	1.08	1.02	0.84
MYB	MYB;Cmyb;c-myb;c-myb_CDS;efg	v-myb myeloblastosis viral oncogene homolog (avian)	1.02	0.99	1.02	0.78
RASSF5	RASSF5;MGC10823;MGC17344;Maxp1;NORE1;NORE1A;NORE1B;RAPL;RASSF3	Ras association (RalGDS/AF-6) domain family member 5	1.02	1.14	1.02	0.83
BPNT1	BPNT1;PIP	3'(2'), 5'-bisphosphate nucleotidase 1	1.02	0.97	1.02	0.73
NIPSNAP3B	NIPSNAP3B;FLJ11275;NIPSNAP3	nipsnap homolog 3B (C. elegans)	1.01	1.06	1.02	0.73
ABI3	ABI3;NESH;SSH3BP3	ABI family, member 3	1.01	0.92	1.02	0.77
ODZ3	ODZ3;FLJ10474;FLJ10886;KIAA1455;TNM3;Ten-m3	odz, odd Oz/ten-m homolog 3 (Drosophila)	1.01	1.05	1.02	0.76
UBQLN3	UBQLN3;TUP-1	ubiquilin 3	1	1.06	1.02	0.79
AQP9	AQP9;AQP-9;HsT17287;SSC1	aquaporin 9	1	1	1.02	0.73
PLXNA2	PLXNA2;FLJ11751;FLJ30634;KIAA0463;OCT;PLXN2	plexin A2	1	0.98	1.02	0.75
CRMP1	CRMP1;CRMP-1;DPYSL1;DRP-1;DRP1;ULIP-3	collapsin response mediator protein 1	1	0.96	1.02	0.75
MAPK15	MAPK15;ERK7;ERK8	mitogen-activated protein kinase 15	1	1.01	1.02	0.84
KRTAP10-12	KRTAP10-12;KAP10.12;KRTAP18-12;KRTAP18.12	keratin associated protein 10-12	1	1.09	1.02	0.84
TUSC2	TUSC2;C3orf11;FUS1;PAP;PDAP2	tumor suppressor candidate 2	1	0.92	1.02	0.77
UBQLN1	UBQLN1;DA41;DSK2;FLJ90054;PLIC-1;UBQN;XDRP1	ubiquilin 1	1	1.11	1.02	0.81
CCDC167	CCDC167;C6orf129;HSPC265;MGC131656	coiled-coil domain containing 167	1	1.04	1.02	0.79
MT1P2	MT1P2	metallothionein 1 pseudogene 2	0.99	1.08	1.02	0.76
C5orf28	C5orf28;FLJ21657;MGC149524;MGC90226	chromosome 5 open reading frame 28	0.98	1.07	1.02	0.75
MTHFD1L	MTHFD1L;DKFZp586G1517;FLJ21145;FTHFSDC1;MTC1THFS;dJ292B18.2	methylenetetrahydrofolate dehydrogenase (NADP+ dependent) 1-like	0.98	0.96	1.02	0.75
UBE2V2	UBE2V2;DDVIT1;DDVit-1;EDAF-1;EDPF-1;EDPF1;MMS2;UEV-2;UEV2	ubiquitin-conjugating enzyme E2 variant 2	0.98	0.92	1.02	0.69
KIAA0317	KIAA0317	KIAA0317	0.98	0.92	1.02	0.68
GGH	GGH;GH	gamma-glutamyl hydrolase (conjugase, folylpolygammaglutamyl hydrolase)	0.98	0.92	1.02	0.69
SAR1A	SAR1A;SAR1;SARA1;Sara;masra2	SAR1 homolog A (S. cerevisiae)	0.98	1.09	1.02	0.72
BAGE4	BAGE4;CT2.4;MGC119851;MGC119852;MGC119853;MLL3P	B melanoma antigen family, member 4	0.98	1.04	1.02	0.85
CHRNG	CHRNG;ACHRG;MGC133376	cholinergic receptor, nicotinic, gamma	0.97	1.04	1.02	0.81
RAB26	RAB26;V46133	RAB26, member RAS oncogene family	0.97	0.9	1.02	0.8
BLOC1S2	BLOC1S2;BLOS2;FLJ30135;MGC10120;RP11-316M21.4	biogenesis of lysosomal organelles complex-1, subunit 2	0.97	0.97	1.02	0.72
KIAA1704	KIAA1704;LSR7;RP11-245H20.2;bA245H20.2	KIAA1704	0.97	0.97	1.02	0.73
CEACAM21	CEACAM21;CEACAM3;FLJ13540;MGC119874;R29124_1	carcinoembryonic antigen-related cell adhesion molecule 21	0.96	0.95	1.02	0.72
OR3A2	OR3A2;OLFRA04;OR17-14;OR17-228;OR228	olfactory receptor, family 3, subfamily A, member 2	0.96	0.96	1.02	0.73
APCDD1	APCDD1;B7323;DRAPC1;HHS;HTS	adenomatosis polyposis coli down-regulated 1	0.96	1.02	1.02	0.72
BCAN	BCAN;BEHAB;CSPG7;MGC13038	brevican	0.96	0.92	1.02	0.67
KCNC4	KCNC4;C1orf30;HKSHIIIC;KSHIIIC;KV3.4;MGC126818	potassium voltage-gated channel, Shaw-related subfamily, member 4	0.96	0.97	1.02	0.75
FAM92B	FAM92B;FLJ44299;MGC138149	family with sequence similarity 92, member B	0.96	1.02	1.02	0.76
KRTAP10-8	KRTAP10-8;KAP10.8;KRTAP18-8;KRTAP18.8	keratin associated protein 10-8	0.96	1	1.02	0.75
AQP7	AQP7;AQP7L;AQP9;AQPap;MGC149555;MGC149556	aquaporin 7	0.96	1.02	1.02	0.79
SEMA7A	SEMA7A;CD108;CDw108;H-SEMA-K1;H-Sema-L;JMH;MGC126692;MGC126696;SEMAK1;SEMAL	semaphorin 7A, GPI membrane anchor (John Milton Hagen blood group)	0.96	1.01	1.02	0.73
TMCO4	TMCO4;DKFZp686C23231;RP5-1056L3.6	transmembrane and coiled-coil domains 4	0.96	0.96	1.02	0.8
GOLGA3	GOLGA3;GCP170;MEA-2	golgin A3	0.96	0.99	1.02	0.67
YME1L1	YME1L1;FTSH;MEG4;PAMP;YME1L	YME1-like 1 (S. cerevisiae)	0.96	0.96	1.02	0.72
G3BP1	G3BP1;G3BP;HDH-VIII;MGC111040	GTPase activating protein (SH3 domain) binding protein 1	0.96	0.97	1.02	0.83
SREK1	SREK1;DKFZp564B176;MGC133045;SFRS12;SRrp508;SRrp86	splicing regulatory glutamine/lysine-rich protein 1	0.96	1.02	1.02	0.72
DNMBP	DNMBP;ARHGEF36;KIAA1010;TUBA	dynamin binding protein	0.95	0.99	1.02	0.67
MDM2	MDM2;HDMX;MGC5370;MGC71221;hdm2	Mdm2 p53 binding protein homolog (mouse)	0.95	0.92	1.02	0.67
PLXNC1	PLXNC1;CD232;PLXN-C1;VESPR	plexin C1	0.94	1	1.02	0.67
ATP1A2	ATP1A2;FHM2;MGC59864;MHP2	ATPase, Na+/K+ transporting, alpha 2 polypeptide	0.94	0.94	1.02	0.73
ULK2	ULK2;ATG1B;KIAA0623;Unc51.2	unc-51-like kinase 2 (C. elegans)	0.94	0.96	1.02	0.8
FOXE1	FOXE1;FKHL15;FOXE2;HFKH4;HFKL5;TITF2;TTF-2;TTF2	forkhead box E1 (thyroid transcription factor 2)	0.94	0.92	1.02	0.68
PTPN18	PTPN18;BDP1;PTP-HSCF	protein tyrosine phosphatase, non-receptor type 18 (brain-derived)	0.94	0.9	1.02	0.68
C11orf84	C11orf84;DKFZp762N0114	chromosome 11 open reading frame 84	0.94	0.91	1.02	0.77
DGKD	DGKD;DGKdelta;FLJ26930;KIAA0145;dgkd-2	diacylglycerol kinase, delta 130kDa	0.94	0.94	1.02	0.84
LAYN	LAYN;FLJ30977;FLJ31092	layilin	0.94	0.98	1.02	0.74
RABIF	RABIF;MSS4;RASGFR3;RASGRF3	RAB interacting factor	0.94	0.91	1.02	0.66
TBCB	TBCB;CG22;CKAP1;CKAPI;MGC14625	tubulin folding cofactor B	0.94	0.94	1.02	0.7
PGAM1	PGAM1;PGAM-B;PGAMA	phosphoglycerate mutase 1 (brain)	0.94	0.95	1.02	0.74
AMY1C	AMY1C;AMY1;AMY1A;AMY1B	amylase, alpha 1C (salivary)	0.94	1	1.02	0.67
GPSM3	GPSM3;AGS4;C6orf9;G18;G18.1a;G18.1b;G18.2;NG1	G-protein signaling modulator 3	0.93	0.99	1.02	0.66
DTWD2	DTWD2;FLJ33977;MGC138579;MGC138580	DTW domain containing 2	0.93	0.84	1.02	0.66
RIPK2	RIPK2;CARD3;CARDIAK;CCK;GIG30;RICK;RIP2	receptor-interacting serine-threonine kinase 2	0.93	0.94	1.02	0.78
IDI1	IDI1;IPP1;IPPI1	isopentenyl-diphosphate delta isomerase 1	0.93	0.96	1.02	0.64
ACSM1	ACSM1;BUCS1;MACS1;MGC150532	acyl-CoA synthetase medium-chain family member 1	0.92	0.9	1.02	0.65
CDH8	CDH8;Nbla04261	cadherin 8, type 2	0.92	0.98	1.02	0.66
SIRPG	SIRPG;CD172g;FLJ42230;SIRP-B2;SIRPB2;SIRPgamma;bA77C3.1	signal-regulatory protein gamma	0.92	1	1.02	0.77
AQP1	AQP1;AQP-CHIP;CHIP28;CO;MGC26324	aquaporin 1 (Colton blood group)	0.92	0.93	1.02	0.69
HSD17B14	HSD17B14;DHRS10;SDR47C1;retSDR3	hydroxysteroid (17-beta) dehydrogenase 14	0.92	1.02	1.02	0.76
ZMAT5	ZMAT5	zinc finger, matrin-type 5	0.92	0.92	1.02	0.7
CPT2	CPT2;CPT1;CPTASE;IIAE4	carnitine palmitoyltransferase 2	0.92	0.9	1.02	0.64
EPB41L4A	EPB41L4A;DKFZp566L203;EPB41L4;FLJ38738;NBL4	erythrocyte membrane protein band 4.1 like 4A	0.92	1.02	1.02	0.75
PMS2	PMS2;HNPCC4;PMS2CL;PMSL2	PMS2 postmeiotic segregation increased 2 (S. cerevisiae)	0.92	0.92	1.02	0.79
PTK7	PTK7;CCK4	PTK7 protein tyrosine kinase 7	0.92	1.02	1.02	0.72
C16orf88	C16orf88;101F10.1;FAM191A	chromosome 16 open reading frame 88	0.92	0.97	1.02	0.74
TNFSF12	TNFSF12;APO3L;DR3LG;MGC129581;MGC20669;TWEAK	tumor necrosis factor (ligand) superfamily, member 12	0.92	0.96	1.02	0.73
FBLN2	FBLN2	fibulin 2	0.91	0.96	1.02	0.64
MCOLN3	MCOLN3;FLJ11006;FLJ36629;MGC71509;TRP-ML3;TRPML3	mucolipin 3	0.91	0.92	1.02	0.72
TMTC1	TMTC1;FLJ31400;FLJ41625;OLF;TMTC1A	transmembrane and tetratricopeptide repeat containing 1	0.91	0.99	1.02	0.7
KIAA1009	KIAA1009;C6orf84;FLJ13551;QN1	KIAA1009	0.9	0.86	1.02	0.73
GPR152	GPR152;MGC148162;PGR5	G protein-coupled receptor 152	0.9	0.88	1.02	0.79
PRKCG	PRKCG;MGC57564;PKC-gamma;PKCC;PKCG;SCA14	protein kinase C, gamma	0.9	0.99	1.02	0.7
FBXL12	FBXL12;FLJ20188;Fbl12	F-box and leucine-rich repeat protein 12	0.9	0.88	1.02	0.64
TTLL12	TTLL12;FLJ41795;KIAA0153;dJ526I14.2	tubulin tyrosine ligase-like family, member 12	0.9	0.9	1.02	0.67
NOTCH2	NOTCH2;AGS2;HJCYS;hN2	notch 2	0.9	0.86	1.02	0.68
FAM193A	FAM193A;C4orf8	family with sequence similarity 193, member A	0.9	0.96	1.02	0.72
TP53TG5	TP53TG5;C20orf10;CLG01	TP53 target 5	0.9	0.92	1.02	0.62
FMO2	FMO2;FLJ40826;FMO1B1	flavin containing monooxygenase 2 (non-functional)	0.89	0.96	1.02	0.64
OBP2B	OBP2B;MGC119022;hOBPIIb	odorant binding protein 2B	0.89	1.02	1.02	0.7
HOXB5	HOXB5;HHO.C10;HOX2;HOX2A;HU-1;Hox2.1	homeobox B5	0.89	0.88	1.02	0.62
AKAP10	AKAP10;D-AKAP2;MGC9414;PRKA10	A kinase (PRKA) anchor protein 10	0.89	0.87	1.02	0.65
GSTO1	GSTO1;DKFZp686H13163;GSTTLp28;P28	glutathione S-transferase omega 1	0.89	0.98	1.02	0.73
DPP8	DPP8;DP8;DPRP1;FLJ14920;FLJ20283;MGC26191;MSTP141	dipeptidyl-peptidase 8	0.89	0.9	1.02	0.67
CABIN1	CABIN1;CAIN;KIAA0330;PPP3IN	calcineurin binding protein 1	0.89	0.94	1.02	0.71
LSG1	LSG1;FLJ11301;FLJ27294	large subunit GTPase 1 homolog (S. cerevisiae)	0.89	0.96	1.02	0.68
EBF2	EBF2;COE2;EBF-2;FLJ11500;O/E-3;OE-3	early B-cell factor 2	0.88	0.9	1.02	0.63
IL20RA	IL20RA;FLJ40993;IL-20R1;ZCYTOR7	interleukin 20 receptor, alpha	0.88	0.86	1.02	0.62
CABP1	CABP1;CALBRAIN;HCALB_BR	calcium binding protein 1	0.88	0.92	1.02	0.66
ADAMTSL4	ADAMTSL4;TSRC1	ADAMTS-like 4	0.88	0.8	1.02	0.62
FCN1	FCN1;FCNM	ficolin (collagen/fibrinogen domain containing) 1	0.88	0.88	1.02	0.66
GPATCH3	GPATCH3;DKFZp686E0221;FLJ12455;GPATC3	G patch domain containing 3	0.88	0.8	1.02	0.62
PROP1	PROP1;CPHD2;PROP-1	PROP paired-like homeobox 1	0.88	0.87	1.02	0.75
C6orf168	C6orf168;FLJ30539;MGC2817;dJ273F20	chromosome 6 open reading frame 168	0.88	0.94	1.02	0.67
FGF18	FGF18;FGF-18;ZFGF5	fibroblast growth factor 18	0.88	0.95	1.02	0.62
HERC2P3	HERC2P3;D15F37S4	hect domain and RLD 2 pseudogene 3	0.88	0.92	1.02	0.7
NBPF11	NBPF11	neuroblastoma breakpoint family, member 11	0.88	0.87	1.02	0.67
MIS18BP1	MIS18BP1;C14orf106;FLJ11186;HSA242977;KIAA1903;KNL2;M18BP1	MIS18 binding protein 1	0.88	0.88	1.02	0.67
LPPR4	LPPR4;KIAA0455;LPR4;PHP1;PRG-1;PRG1;RP4-788L13.1	lipid phosphate phosphatase-related protein type 4	0.87	0.82	1.02	0.62
THAP10	THAP10	THAP domain containing 10	0.87	0.92	1.02	0.61
MLF1	MLF1	myeloid leukemia factor 1	0.87	0.86	1.02	0.68
GTF2A2	GTF2A2;HsT18745;TF2A2;TFIIA	general transcription factor IIA, 2, 12kDa	0.87	0.84	1.02	0.66
ZFP106	ZFP106;DKFZp451A239;FLJ34610;FLJ45841;SH3BP3;ZNF474	zinc finger protein 106 homolog (mouse)	0.87	0.79	1.02	0.66

HIST1H2BK	HIST1H2BK;H2B/S;H2BFAiii;H2BFT;MGC131989	histone cluster 1, H2bk	0.87	0.9	1.02	0.65
GYPE	GYPE;GPE;MNS;MiIX	glycophorin E (MNS blood group)	0.86	0.84	1.02	0.61
RGR	RGR;RP44	retinal G protein coupled receptor	0.86	0.86	1.02	0.77
FOXD4L3	FOXD4L3	ENTREZ STATUS: REMAPPED TO GENE ID: 286380	0.86	0.9	1.02	0.68
CHST11	CHST11;C4ST;C4ST-1;C4ST1;DKFZp667A035;FLJ41682;HSA269537	carbohydrate (chondroitin 4) sulfotransferase 11	0.86	0.81	1.02	0.72
MNX1	MNX1;HB9;HLXB9;HOXHB9;SCRA1	motor neuron and pancreas homeobox 1	0.86	0.88	1.02	0.68
LPP	LPP;DKFZp779O0231;FLJ30652;FLJ41512	LIM domain containing preferred translocation partner in lipoma	0.86	0.9	1.02	0.68
PLP1	PLP1;HLD1;MMPL;PLP;PLP/DM20;PMD;SPG2	proteolipid protein 1	0.85	0.96	1.02	0.7
OR4D5	OR4D5;OR11-276	olfactory receptor, family 4, subfamily D, member 5	0.85	0.84	1.02	0.64
OPRM1	OPRM1;KIAA0403;LMOR;MOR;MOR1;OPRM	opioid receptor, mu 1	1.48	1.4	1.03	1.06
CTCFL	CTCFL;BORIS;CT27;CTCF-T;HMGB1L1;MGC163358;MGC169105;MGC169106;dJ579F20.2	CCCTC-binding factor (zinc finger protein)-like	1.29	1.46	1.03	1.07
LMTK2	LMTK2;AATYK2;BREK;FLJ46659;KIAA1079;KPI-2;KPI2;LMR2;cprk	lemur tyrosine kinase 2	1.25	1.4	1.03	0.99
PRKAG2	PRKAG2;AAKG;AAKG2;CMH6;H91620p;WPWS	protein kinase, AMP-activated, gamma 2 non-catalytic subunit	1.25	1.38	1.03	0.98
ADSSL1	ADSSL1;FLJ38602	adenylosuccinate synthase like 1	1.23	1.3	1.03	1.03
C21orf33	C21orf33;ES1;GT335;HES1;KNPH;KNPI	chromosome 21 open reading frame 33	1.22	1.2	1.03	0.92
MRGPRD	MRGPRD;MRGD;TGR7	MAS-related GPR, member D	1.21	1.16	1.03	0.87
TRIM67	TRIM67;FLJ44831;TNL	tripartite motif containing 67	1.19	1.17	1.03	0.85
CBLB	CBLB;Cbl-b;DKFZp686J10223;DKFZp779A0729;DKFZp779F1443;FLJ36865;FLJ41152;RNF56	Cas-Br-M (murine) ecotropic retroviral transforming sequence b	1.19	1.16	1.03	0.84
TMED8	TMED8;FAM15B;MGC126559	transmembrane emp24 protein transport domain containing 8	1.18	1.18	1.03	0.93
GPR160	GPR160;GPCR1;GPCR150	G protein-coupled receptor 160	1.18	1.17	1.03	1.06
CDKN3	CDKN3;CDI1;CIP2;FLJ25787;KAP;KAP1;MGC70625	cyclin-dependent kinase inhibitor 3	1.18	1.14	1.03	0.86
DCAF4L1	DCAF4L1;MGC126021;MGC126022;WDR21B	DDB1 and CUL4 associated factor 4-like 1	1.17	1.07	1.03	0.84
NCOR2	NCOR2;CTG26;FLJ25011;N-CoR2;SMAP270;SMRT;SMRTE;SMRTE-tau;TNRC14;TRAC;TRAC-1;TRAC1	nuclear receptor corepressor 2	1.15	1.27	1.03	0.83
WDR93	WDR93	WD repeat domain 93	1.13	1.21	1.03	0.9
DPCR1	DPCR1;MGC126710;MGC126712;PBLT	diffuse panbronchiolitis critical region 1	1.12	1.05	1.03	0.87
MKRN3	MKRN3;D15S9;MGC88288;RNF63;ZFP127;ZNF127	makorin ring finger protein 3	1.12	1.1	1.03	0.79
TRIM45	TRIM45;FLJ13181;RNF99	tripartite motif containing 45	1.12	1.15	1.03	1.03
AP1S3	AP1S3	adaptor-related protein complex 1, sigma 3 subunit	1.12	1.2	1.03	0.91
CASP7	CASP7;CMH-1;ICE-LAP3;MCH3	caspase 7, apoptosis-related cysteine peptidase	1.12	1.15	1.03	0.86
ZNF33A	ZNF33A;FLJ23404;KIAA0065;KOX2;KOX31;KOX5;NF11A;ZNF11;ZNF11A;ZNF33;ZZAPK	zinc finger protein 33A	1.1	1.11	1.03	0.86
LRRC37A2	LRRC37A2;FLJ45049	leucine rich repeat containing 37, member A2	1.1	1.08	1.03	0.87
MID1	MID1;BBBG1;FLJ57031;FLJ58683;FLJ76288;FXY;GBBB1;MIDIN;OGS1;OS;OSX;RNF59;TRIM18;XPRF;ZNFXY	midline 1 (Opitz/BBB syndrome)	1.1	1.07	1.03	0.78
LOC646498	LOC646498	uncharacterized protein LOC646498	1.1	1.08	1.03	0.83
MMP2	MMP2;CLG4;CLG4A;MMP-II;MONA;TBE-1	matrix metallopeptidase 2 (gelatinase A, 72kDa gelatinase, 72kDa type IV collagenase)	1.09	1.12	1.03	0.84
ABCA7	ABCA7;ABCA-SSN;ABCX;FLJ40025	ATP-binding cassette, sub-family A (ABC1), member 7	1.09	1.16	1.03	0.91
SV2A	SV2A;KIAA0736;SV2	synaptic vesicle glycoprotein 2A	1.09	1.19	1.03	0.79
GOLIM4	GOLIM4;GIMPC;GOLPH4;GPP130;P138	golgi integral membrane protein 4	1.09	1.16	1.03	0.88
C11orf58	C11orf58;DKFZp686N0232;IMAGE145052;MGC117265;SMAP	chromosome 11 open reading frame 58	1.09	1.17	1.03	0.84
CDH23	CDH23;CDHR23;DKFZp434P2350;FLJ00233;FLJ36499;KIAA1774;KIAA1812;MGC102761;USH1D	cadherin-related 23	1.08	1.15	1.03	0.91
DPEP2	DPEP2;MBD2	dipeptidase 2	1.08	1.09	1.03	0.82
RBX1	RBX1;BA554C12.1;FLJ60363;MGC13357;MGC1481;RNF75;ROC1	ring-box 1, E3 ubiquitin protein ligase	1.08	1.06	1.03	0.77
NECAB1	NECAB1;EFCBP1;STIP-1	N-terminal EF-hand calcium binding protein 1	1.07	1.14	1.03	0.76
AMICA1	AMICA1;AMICA;CREA7-1;CREA7-4;FLJ37080;Gm638;JAML;MGC118814;MGC118815	adhesion molecule, interacts with CXADR antigen 1	1.07	1	1.03	0.78
KIF25	KIF25;KNSL3;MGC163361	kinesin family member 25	1.07	1.12	1.03	0.86
USP16	USP16;UBP-M	ubiquitin specific peptidase 16	1.07	1.02	1.03	0.77
PVR	PVR;CD155;FLJ25946;HVED;NECL5;Necl-5;PVS;TAGE4	poliovirus receptor	1.07	1.02	1.03	0.77
IGF2	IGF2;C11orf43;FLJ22066;FLJ44734;IGF-II;PP9974	insulin-like growth factor 2 (somatomedin A)	1.06	1.04	1.03	0.75
CSNK1G2	CSNK1G2;CK1g2	casein kinase 1, gamma 2	1.06	1.09	1.03	0.9
CCDC82	CCDC82;FLJ23518;HSPC048	coiled-coil domain containing 82	1.06	1.17	1.03	0.81
FKBP4	FKBP4;FKBP51;FKBP52;FKBP59;HBI;Hsp56;PPIase;p52	FK506 binding protein 4, 59kDa	1.06	1.04	1.03	0.76
PSPH	PSPH;PSP;PSPHD	phosphoserine phosphatase	1.05	1.02	1.03	0.76
PAK1IP1	PAK1IP1;FLJ20624;MAK11;PIP1;RP11-421M1.5;WDR84;bA421M1.5;hPIP1	PAK1 interacting protein 1	1.05	1.12	1.03	0.85
BMPR1B	BMPR1B;ALK-6;ALK6;CDw293	bone morphogenetic protein receptor, type IB	1.04	1.06	1.03	0.87
MLC1	MLC1;KIAA0027;LVM;MLC;VL	megalencephalic leukoencephalopathy with subcortical cysts 1	1.04	1.07	1.03	0.84
ZNF613	ZNF613;FLJ13590	zinc finger protein 613	1.04	1.09	1.03	0.87
ZNF318	ZNF318;FLJ21852;TZF;ZFP318	zinc finger protein 318	1.04	0.97	1.03	0.81
PLEKHF1	PLEKHF1;APPD;MGC4090;PHAFIN1;ZFYVE15	pleckstrin homology domain containing, family F (with FYVE domain) member 1	1.04	0.96	1.03	0.77
THG1L	THG1L;FLJ11601;FLJ20546;ICF45	tRNA-histidine guanylyltransferase 1-like (S. cerevisiae)	1.04	1.12	1.03	0.8
CTSL2	CTSL2;CATL2;CTSU;CTSV;MGC125957	cathepsin L2	1.04	0.98	1.03	0.73
ARNTL2	ARNTL2;BMAL2;CLIF;MGC149671;MGC149672;MOP9;PASD9;bHLHe6	aryl hydrocarbon receptor nuclear translocator-like 2	1.04	1.15	1.03	0.76
LIMS1	LIMS1;PINCH;PINCH-1;PINCH1	LIM and senescent cell antigen-like domains 1	1.04	1.03	1.03	0.79
PLIN5	PLIN5;LSDA5;LSDP5;MLDP;OXPAT	perilipin 5	1.04	1.17	1.03	0.78
SLITRK2	SLITRK2;CXorf2;DKFZp451E1911;KIAA1854;MGC129912;MGC129913;SLITL1	SLIT and NTRK-like family, member 2	1.03	1.1	1.03	0.75
CHRM1	CHRM1;HM1;M1;M1R;MGC30125	cholinergic receptor, muscarinic 1	1.03	1.04	1.03	0.93
ZNF432	ZNF432	zinc finger protein 432	1.03	1.05	1.03	0.87
ZNF362	ZNF362;FLJ25476;MGC138318;RN;lin-29	zinc finger protein 362	1.03	1.12	1.03	0.79
DPY30	DPY30;HDPY-30;Saf19	dpy-30 homolog (C. elegans)	1.03	1.1	1.03	0.83
EIF4G2	EIF4G2;AAG1;DAP5;FLJ41344;NAT1;P97	eukaryotic translation initiation factor 4 gamma, 2	1.03	1.02	1.03	0.74
ZPLD1	ZPLD1	zona pellucida-like domain containing 1	1.02	0.93	1.03	0.73
HTR1B	HTR1B;5-HT1B;5-HT1DB;HTR1D2;HTR1DB;S12	5-hydroxytryptamine (serotonin) receptor 1B	1.02	1.02	1.03	0.91
TSSC4	TSSC4	tumor suppressing subtransferable candidate 4	1.02	1.08	1.03	0.82
LGALS8	LGALS8;Gal-8;PCTA-1;PCTA1;Po66-CBP	lectin, galactoside-binding, soluble, 8	1.02	1.08	1.03	0.85
PCDHGC4	PCDHGC4;MGC119489;MGC24978;PCDH-GAMMA-C4	protocadherin gamma subfamily C, 4	1.02	1.04	1.03	0.78
CYP2E1	CYP2E1;CPE1;CYP2E;P450-J;P450C2E	cytochrome P450, family 2, subfamily E, polypeptide 1	1.01	0.97	1.03	0.85
CUL5	CUL5;VACM-1;VACM1	cullin 5	1.01	1.04	1.03	0.81
LINS	LINS;FLJ10583;LINS1;WINS1	lines homolog (Drosophila)	1.01	1.11	1.03	0.78
HIST1H2BL	HIST1H2BL;H2B/c;H2BFC;dJ97D16.4	histone cluster 1, H2bl	1	0.92	1.03	0.76
MTAP	MTAP;MSAP;c86fus	methylthioadenosine phosphorylase	1	1	1.03	0.72
BCAT1	BCAT1;BCATC;BCT1;DKFZp686E12175;ECA39;MECA39;PNAS121;PP18	branched chain amino-acid transaminase 1, cytosolic	1	1.06	1.03	0.82
MED17	MED17;CRSP6;CRSP77;DRIP80;FLJ10812;TRAP80	mediator complex subunit 17	1	0.98	1.03	0.71
CRYGS	CRYGS;CRYG8	crystallin, gamma S	0.99	1.05	1.03	0.8
CYP4F22	CYP4F22;FLJ39501;LI3	cytochrome P450, family 4, subfamily F, polypeptide 22	0.99	0.94	1.03	0.84
PRPF40B	PRPF40B;HYPC	PRP40 pre-mRNA processing factor 40 homolog B (S. cerevisiae)	0.99	0.98	1.03	0.74
GDI2	GDI2;FLJ16452;FLJ37352;RABGDIB	GDP dissociation inhibitor 2	0.99	1.08	1.03	0.71
GANC	GANC;FLJ40082;MGC138256	glucosidase, alpha; neutral C	0.99	0.98	1.03	0.71
CNTF	CNTF;HCNTF	ciliary neurotrophic factor	0.99	0.99	1.03	0.7
TAS2R3	TAS2R3;T2R3	taste receptor, type 2, member 3	0.98	0.9	1.03	0.75
COG6	COG6;COD2;DKFZp313D191;KIAA1134	component of oligomeric golgi complex 6	0.98	0.98	1.03	0.78
HAUS3	HAUS3;C4orf15;DKFZp686I1868;IT1;MGC4701;dgt3	HAUS augmin-like complex, subunit 3	0.98	0.9	1.03	0.75
CRCP	CRCP;CGRP-RCP;CGRPRCP;MGC111194;RCP;RCP9	CGRP receptor component	0.98	1.04	1.03	0.7
C16orf89	C16orf89;MGC45438	chromosome 16 open reading frame 89	0.97	1.02	1.03	0.68
37135	SEPT1;DIFF6;LARP;MGC20394;PNUTL3;SEP1	septin 1	0.97	0.96	1.03	0.69
C1orf38	C1orf38;ICB-1;THEMIS2	chromosome 1 open reading frame 38	0.97	1.05	1.03	0.85
MOGS	MOGS;CDG2B;GCS1	mannosyl-oligosaccharide glucosidase	0.97	0.95	1.03	0.75
VASH2	VASH2;FLJ12505;RP11-275G3.1	vasohibin 2	0.96	1.01	1.03	0.76
ANGPTL6	ANGPTL6;AGF;ARP5	angiopoietin-like 6	0.96	1.02	1.03	0.68
HSPBP1	HSPBP1;FES1	HSPA (heat shock 70kDa) binding protein, cytoplasmic cochaperone 1	0.96	1	1.03	0.69
OR5A1	OR5A1;OR11-249;OR5A1P;OST181	olfactory receptor, family 5, subfamily A, member 1	0.95	0.88	1.03	0.74
ZNF10	ZNF10;KOX1	zinc finger protein 10	0.95	1.02	1.03	0.77
FIS1	FIS1;TTC11	fission 1 (mitochondrial outer membrane) homolog (S. cerevisiae)	0.95	0.97	1.03	0.72
FAM21C	FAM21C;FAM21A;KIAA0592;VPEF	family with sequence similarity 21, member C	0.95	0.96	1.03	0.77
SPAG8	SPAG8;BS-84;HSD-1;MGC26201;SMP1;SPAG3;hSMP-1	sperm associated antigen 8	0.94	1.02	1.03	0.82
BDNF	BDNF;MGC34632	brain-derived neurotrophic factor	0.94	0.94	1.03	0.69
CLDN18	CLDN18;DKFZp564B2062;SFTA5;SFTPJ	claudin 18	0.94	0.94	1.03	0.78
ABCG1	ABCG1;ABC8;MGC34313;WHITE1	ATP-binding cassette, sub-family G (WHITE), member 1	0.94	0.89	1.03	0.78
INSRR	INSRR;IRR	insulin receptor-related receptor	0.94	1.04	1.03	0.74
HABP2	HABP2;FSAP;HABP;HGFAL;PHBP	hyaluronan binding protein 2	0.94	0.96	1.03	0.72
ANKLE1	ANKLE1;ANKRD41;FLJ39369;LEM3;LEMD6	ankyrin repeat and LEM domain containing 1	0.94	0.94	1.03	0.72
KCNQ1	KCNQ1;ATFB1;ATFB3;FLJ26167;JLNS1;KCNA8;KCNA9;KVLQT1;Kv1.9;Kv7.1;LQT;LQT1;RWS;SQT2;WRS	potassium voltage-gated channel, KQT-like subfamily, member 1	0.94	0.96	1.03	0.75
XK	XK;KX;MCLDS;X1k;XKR1	X-linked Kx blood group (McLeod syndrome)	0.94	0.94	1.03	0.73
C9orf95	C9orf95;FLJ20559;NRK1;bA235O14.2	chromosome 9 open reading frame 95	0.94	0.94	1.03	0.68
ZNF248	ZNF248;KIAA0137;MGC131882;bA162G10.3	zinc finger protein 248	0.94	1	1.03	0.76
CBFA2T2	CBFA2T2;DKFZp313F2116;EHT;MTGR1;ZMYND3;p85	core-binding factor, runt domain, alpha subunit 2; translocated to, 2	0.94	0.96	1.03	0.71
GSG2	GSG2;HASPIN	germ cell associated 2 (haspin)	0.94	0.96	1.03	0.79
CD7	CD7;GP40;LEU-9;TP41;Tp40	CD7 molecule	0.94	1	1.03	0.76
MPP5	MPP5;FLJ12615;PALS1	membrane protein, palmitoylated 5 (MAGUK p55 subfamily member 5)	0.94	0.97	1.03	0.65
ZKSCAN1	ZKSCAN1;9130423L19Rik;KOX18;MGC138429;PHZ-37;ZNF139;ZNF36	zinc finger with KRAB and SCAN domains 1	0.94	0.93	1.03	0.71
HAT1	HAT1;KAT1	histone acetyltransferase 1	0.94	0.96	1.03	0.71
RYK	RYK;D3S3195;JTK5;JTK5A;RYK1	RYK receptor-like tyrosine kinase	0.94	0.97	1.03	0.8
OR5D18	OR5D18;OR11-143	olfactory receptor, family 5, subfamily D, member 18	0.93	0.92	1.03	0.66
BNC1	BNC1;BNC;BSN1;HsT19447	basonuclin 1	0.93	0.97	1.03	0.65
TMEM37	TMEM37;PR;PR1	transmembrane protein 37	0.93	0.95	1.03	0.74
HSF1	HSF1;HSTF1	heat shock transcription factor 1	0.93	0.96	1.03	0.75
LRRC14B	LRRC14B	leucine rich repeat containing 14B	0.93	0.98	1.03	0.74
NCAM2	NCAM2;MGC51008;NCAM21	neural cell adhesion molecule 2	0.92	0.92	1.03	0.73
40238	MARCH10;FLJ35757;MARCH-X;RNF190	membrane-associated ring finger (C3HC4) 10	0.92	0.93	1.03	0.83
GZMH	GZMH;CCP-X;CGL-2;CSP-C;CTLA1;CTSGL2	granzyme H (cathepsin G-like 2, protein h-CCPX)	0.92	0.88	1.03	0.65
ARHGAP22	ARHGAP22;RhoGAP2	Rho GTPase activating protein 22	0.92	0.92	1.03	0.72
RBM45	RBM45;DRB1;FLJ44612;MGC42237	RNA binding motif protein 45	0.92	0.9	1.03	0.65

MRPS31	MRPS31;IMOGN38;MRP-S31;S31mt	mitochondrial ribosomal protein S31	0.92	0.96	1.03	0.66
UPF3B	UPF3B;HUPF3B;MRXS14;RENT3B;UPF3X	UPF3 regulator of nonsense transcripts homolog B (yeast)	0.92	1.03	1.03	0.76
ASB6	ASB6;FLJ20548;MGC1024	ankyrin repeat and SOCS box containing 6	0.92	0.9	1.03	0.65
C10orf28	C10orf28;FLJ25458;FLJ37160;GIDRP86;GIDRP88;PSORT	chromosome 10 open reading frame 28	0.92	0.9	1.03	0.69
GOLGA2	GOLGA2;GM130;MGC20672	golgin A2	0.92	0.92	1.03	0.74
QRSL1	QRSL1;DKFZp564C1278;FLJ10989;FLJ12189;FLJ13447;GatA	glutaminyl-tRNA synthase (glutamine-hydrolyzing)-like 1	0.92	0.96	1.03	0.76
DNPEP	DNPEP;ASPEP;DAP	aspartyl aminopeptidase	0.92	0.88	1.03	0.66
GPSM2	GPSM2;DFNB82;LGN;PINS	G-protein signaling modulator 2	0.92	0.92	1.03	0.7
WDR6	WDR6;FLJ10218;FLJ52552;FLJ56107;MGC126756;MGC142027	WD repeat domain 6	0.92	0.99	1.03	0.66
LUC7L2	LUC7L2;CGI-74;FLJ10657;LUC7B2	LUC7-like 2 (S. cerevisiae)	0.92	0.92	1.03	0.73
DCTN2	DCTN2;DCTN50;DYNAMITIN;RBP50	dynactin 2 (p50)	0.92	0.9	1.03	0.69
YBX1	YBX1;BP-8;CSDA2;CSDB;DBPB;MDR-NF1;MGC104858;MGC110976;MGC117250;NSEP-1;NSEP1;YB-1;YB1	Y box binding protein 1	0.92	0.9	1.03	0.66
ERI3	ERI3;FLJ22943;MGC2683;PINT1;PRNPIP	ERI1 exoribonuclease family member 3	0.92	0.98	1.03	0.65
SLC9B1	SLC9B1;MGC131641;NHEDC1	solute carrier family 9, subfamily B (cation proton antiporter 2), member 1	0.92	1.02	1.03	0.78
FAM207A	FAM207A;C21orf70	family with sequence similarity 207, member A	0.92	0.98	1.03	0.74
SIK1	SIK1;MSK;SIK;SNF1LK	salt-inducible kinase 1	0.92	0.95	1.03	0.79
TAS2R19	TAS2R19;MGC126853;MGC138203;MSTP058;T2R19;T2R23;T2R48;TAS2R23;TAS2R48	taste receptor, type 2, member 19	0.92	0.92	1.03	0.72
PRKD3	PRKD3;EPK2;PKC-NU;PKD3;PRKCN;nPKC-NU	protein kinase D3	0.91	0.93	1.03	0.77
BASP1	BASP1;CAP-23;CAP23;MGC8555;NAP-22;NAP22	brain abundant, membrane attached signal protein 1	0.91	0.88	1.03	0.75
RAB21	RAB21;KIAA0118	RAB21, member RAS oncogene family	0.91	1	1.03	0.66
C13orf35	C13orf35;FLJ26443	chromosome 13 open reading frame 35	0.9	0.9	1.03	0.72
CD164L2	CD164L2;UNQ6122	CD164 sialomucin-like 2	0.9	1.01	1.03	0.67
GPRC5C	GPRC5C;MGC131820;RAIG-3;RAIG3	G protein-coupled receptor, family C, group 5, member C	0.9	0.9	1.03	0.81
IL23A	IL23A;IL-23;IL-23A;IL23P19;MGC79388;P19;SGRF	interleukin 23, alpha subunit p19	0.9	0.94	1.03	0.65
RARRES2	RARRES2;HP10433;TIG2	retinoic acid receptor responder (tazarotene induced) 2	0.9	0.9	1.03	0.67
GPR137	GPR137;C11orf4;GPR137A;TM7SF1L1	G protein-coupled receptor 137	0.9	0.88	1.03	0.71
IGFL1	IGFL1;APRG644;UNQ644	IGF-like family member 1	0.9	0.94	1.03	0.67
MAP1B	MAP1B;DKFZp686E1099;DKFZp686F1345;FLJ38954;FUTSCH;MAP5	microtubule-associated protein 1B	0.9	1.01	1.03	0.74
CAV2	CAV2;CAV;MGC12294	caveolin 2	0.9	0.96	1.03	0.65
NDUFA13	NDUFA13;B16.6;CDA016;FLJ58045;FLJ59191;GRIM-19;GRIM19	NADH dehydrogenase (ubiquinone) 1 alpha subcomplex, 13	0.9	0.9	1.03	0.67
HIGD1A	HIGD1A;DKFZp564K247;HIG1	HIG1 hypoxia inducible domain family, member 1A	0.9	0.96	1.03	0.69
FAM196A	FAM196A;C10orf141;FLJ45557	family with sequence similarity 196, member A	0.9	0.98	1.03	0.69
ENTHD1	ENTHD1;CACNA1I;FLJ25421;dJ370M22.3	ENTH domain containing 1	0.89	0.92	1.03	0.66
C7orf54	C7orf54;MGC138346;NAG8;NSG-X	chromosome 7 open reading frame 54	0.89	0.88	1.03	0.67
TKTL2	TKTL2;DKFZp434L1717;FLJ32975	transketolase-like 2	0.89	0.88	1.03	0.65
AASS	AASS;LKR/SDH;LKRSDH;LORSDH	aminoadipate-semialdehyde synthase	0.89	0.9	1.03	0.63
ANKRD2	ANKRD2;ARPP;MGC104314	ankyrin repeat domain 2 (stretch responsive muscle)	0.89	1	1.03	0.74
OR51F2	OR51F2;OR11-23	olfactory receptor, family 51, subfamily F, member 2	0.88	0.88	1.03	0.66
LOC729974	LOC729974	ret finger protein-like 4A-like	0.88	0.88	1.03	0.8
ABO	ABO;A3GALNT;A3GALT1;GTB;NAGAT	ABO blood group (transferase A, alpha 1-3-N-acetylgalactosaminyltransferase; transferase B, alpha 1-3-galactosyltransferase)	0.88	0.84	1.03	0.74
SLC16A4	SLC16A4;MCT4;MCT5	solute carrier family 16, member 4 (monocarboxylic acid transporter 5)	0.88	0.87	1.03	0.63
LRRC3	LRRC3;C21orf102	leucine rich repeat containing 3	0.88	0.85	1.03	0.72
ZNF548	ZNF548;FLJ32932	zinc finger protein 548	0.88	0.9	1.03	0.63
NEDD4	NEDD4;KIAA0093;MGC176705;NEDD4-1;RPF1	neural precursor cell expressed, developmentally down-regulated 4	0.88	0.84	1.03	0.63
TBC1D19	TBC1D19;FLJ11082	TBC1 domain family, member 19	0.88	0.94	1.03	0.71
MLX	MLX;MAD7;MXD7;TCFL4;bHLHd13	MAX-like protein X	0.88	0.88	1.03	0.65
PMM2	PMM2;CDG1;CDG1a;CDGS;PMM 2	phosphomannomutase 2	0.88	0.87	1.03	0.63
GAPT	GAPT;C5orf29;FLJ33641;MGC70478	GRB2-binding adaptor protein, transmembrane	0.88	0.8	1.03	0.63
TMEM231	TMEM231;ALYE870;FLJ22167;PRO1886	transmembrane protein 231	0.88	0.91	1.03	0.62
GAGE2E	GAGE2E;GAGE-2E;GAGE8	G antigen 2E	0.88	0.88	1.03	0.63
PNMA2	PNMA2;KIAA0883;MA2;MM2;RGAG2	paraneoplastic antigen MA2	0.87	0.88	1.03	0.73
NLK	NLK;DKFZp761G1211;FLJ21033	nemo-like kinase	0.87	0.89	1.03	0.74
SLC6A15	SLC6A15;DKFZp761I0921;FLJ10316;MGC87066;NTT73;SBAT1;V7-3;hv7-3	solute carrier family 6 (neutral amino acid transporter), member 15	0.86	0.96	1.03	0.72
INHBC	INHBC;IHBC	inhibin, beta C	0.86	0.89	1.03	0.6
BCL2L15	BCL2L15;Bfk;C1orf178;FLJ22588	BCL2-like 15	0.86	0.86	1.03	0.68
ITPR1	ITPR1;DKFZp313E1334;DKFZp313N1434;INSP3R1;IP3R;IP3R1;SCA15;SCA16	inositol 1,4,5-trisphosphate receptor, type 1	0.86	0.88	1.03	0.69
AMHR2	AMHR2;AMHR;MISR2;MISRII;MRII	anti-Mullerian hormone receptor, type II	0.86	0.86	1.03	0.78
KLHL25	KLHL25;ENC-2;ENC2;FLJ12587;FLJ45015	kelch-like 25 (Drosophila)	0.86	0.91	1.03	0.68
MEIS3	MEIS3;DKFZp547H236;MRG2	Meis homeobox 3	0.86	0.85	1.03	0.61
EHF	EHF;ESE3;ESE3B;ESEJ	ets homologous factor	0.86	0.84	1.03	0.65
GDF7	GDF7;BMP12	growth differentiation factor 7	0.86	0.94	1.03	0.75
C19orf26	C19orf26;DOS;MGC40084	chromosome 19 open reading frame 26	0.86	0.92	1.03	0.69
ADPRHL2	ADPRHL2;ARH3;FLJ20446	ADP-ribosylhydrolase like 2	0.86	0.91	1.03	0.72
SYT14	SYT14;FLJ34198;MGC163195;SCAR11;sytXIV	synaptotagmin XIV	0.86	0.89	1.03	0.62
ACOX1	ACOX1;ACOX;MGC1198;PALMCOX;SCOX	acyl-CoA oxidase 1, palmitoyl	0.86	0.99	1.03	0.68
KIAA0141	KIAA0141;DELE	KIAA0141	0.86	0.92	1.03	0.65
ZNF623	ZNF623;MGC103965;MGC104128	zinc finger protein 623	0.86	0.88	1.03	0.73
WRB	WRB;CHD5;FLJ51808	tryptophan rich basic protein	0.86	0.89	1.03	0.6
CRIPT	CRIPT	cysteine-rich PDZ-binding protein	0.86	0.98	1.03	0.72
CHORDC1	CHORDC1;CHP1;FLJ37289	cysteine and histidine-rich domain (CHORD) containing 1	0.86	0.94	1.03	0.66
SDR16C5	SDR16C5;FLJ33105;RDH#2;RDH-E2;RDHE2	short chain dehydrogenase/reductase family 16C, member 5	0.86	0.89	1.03	0.69
TADA1	TADA1;ADA1;HFI1;RP1-9E21.4;STAF42;TADA1L;hADA1	transcriptional adaptor 1	0.86	0.83	1.03	0.67
GOLGA2P5	GOLGA2P5;DKFZp434A0411;DKFZp434K1926;DKFZp434M0331;GOLGA2B;GOLGA2L1	golgin A2 pseudogene 5	0.86	0.89	1.03	0.64
NEURL2	NEURL2;C20orf163;MGC125934;MGC125935;OZZ;Ozz-E3	neuralized homolog 2 (Drosophila)	0.85	0.84	1.03	0.61
RANBP10	RANBP10;FLJ31165;KIAA1464	RAN binding protein 10	0.85	0.92	1.03	0.74
NSMAF	NSMAF;FAN	neutral sphingomyelinase (N-SMase) activation associated factor	0.85	0.93	1.03	0.75
LRFN2	LRFN2;FIGLER2;KIAA1246;RP11-535K1.2;SALM1	leucine rich repeat and fibronectin type III domain containing 2	1.34	1.54	1.04	1.12
PPM1J	PPM1J;DKFZp434P1514;FLJ35951;MGC19531;MGC90149;PP2CZ;PP2Czeta;PPP2CZ	protein phosphatase, Mg2+/Mn2+ dependent, 1J	1.28	1.31	1.04	1.03
MIB2	MIB2;FLJ20648;FLJ39787;FLJ41406;ZZANK1;ZZZ5	mindbomb homolog 2 (Drosophila)	1.22	1.21	1.04	0.87
OTUD4	OTUD4;DKFZp434I0721;DUBA6;HIN1;HSHIN1;KIAA1046	OTU domain containing 4	1.2	1.29	1.04	0.97
AHI1	AHI1;AHI-1;DKFZp686J1653;FLJ14023;FLJ20069;JBTS3;ORF1;dJ71N10.1	Abelson helper integration site 1	1.19	1.27	1.04	0.97
NLRP5	NLRP5;CLR19.8;MATER;NALP5;PAN11;PYPAF8	NLR family, pyrin domain containing 5	1.17	1.19	1.04	0.89
SLC25A38	SLC25A38;FLJ20551;FLJ22703	solute carrier family 25, member 38	1.17	1.18	1.04	0.89
CHKB	CHKB;CHETK;CHKL;CK;CKB;CKEKB;EK;EKB;MDCMC	choline kinase beta	1.15	1.15	1.04	1.04
TNFSF9	TNFSF9;4-1BB-L;CD137L	tumor necrosis factor (ligand) superfamily, member 9	1.13	1.21	1.04	0.81
ZDHHC16	ZDHHC16;APH2;MGC2993	zinc finger, DHHC-type containing 16	1.13	1.2	1.04	0.91
CXorf36	CXorf36;4930578C19Rik;DIA1R;DKFZp313K0825;EPQL1862;FLJ14103;FLJ55198;PRO3743;bA435K1.1	chromosome X open reading frame 36	1.12	1.21	1.04	0.9
ADAMTS12	ADAMTS12;PRO4389	ADAM metallopeptidase with thrombospondin type 1 motif, 12	1.12	1.16	1.04	0.87
C5orf41	C5orf41;DKFZp313F2319;DKFZp686G2059;LRF	chromosome 5 open reading frame 41	1.12	1.12	1.04	0.85
MEX3C	MEX3C;FLJ38871;MEX-3C;RKHD2;RNF194	mex-3 homolog C (C. elegans)	1.12	1.12	1.04	0.81
ANKRD20A5F	ANKRD20A5P;ANKRD20A5;MGC26718	ankyrin repeat domain 20 family, member A5, pseudogene	1.12	1.21	1.04	0.82
C10orf2	C10orf2;ATXN8;FLJ21832;IOSCA;MTDPS7;PEO;PEO1;PEOA3;SANDO;SCA8;TWINL	chromosome 10 open reading frame 2	1.11	1.1	1.04	0.87
SPPL2A	SPPL2A;IMP3;PSL2	signal peptide peptidase-like 2A	1.1	1.06	1.04	0.8
SGPP1	SGPP1;SPPase1	sphingosine-1-phosphate phosphatase 1	1.08	1.04	1.04	0.79
RNF8	RNF8;FLJ12013;KIAA0646	ring finger protein 8	1.08	1.08	1.04	0.78
RNF115	RNF115;BCA2;ZNF364	ring finger protein 115	1.07	1.1	1.04	0.99
LOX	LOX;MGC105112	lysyl oxidase	1.06	1.11	1.04	0.88
IPO13	IPO13;IMP13;KAP13;KIAA0724;LGL2;RANBP13	importin 13	1.06	1.07	1.04	0.86
OR8G1	OR8G1;HSTPCR25;OR8G1P;TPCR25	olfactory receptor, family 8, subfamily G, member 1	1.06	1.08	1.04	0.81
PVRL1	PVRL1;CD111;CLPED1;ED4;HIgR;HVEC;MGC142031;MGC16207;OFC7;PRR;PRR1;PVRR;PVRR1;SK-12;nectin-1	poliovirus receptor-related 1 (herpesvirus entry mediator C)	1.05	1.13	1.04	0.76
ASL	ASL;ASAL	argininosuccinate lyase	1.05	1.07	1.04	0.79
ARAP2	ARAP2;CENTD1;FLJ13675;FLJ44916;KIAA0580;PARX	ArfGAP with RhoGAP domain, ankyrin repeat and PH domain 2	1.05	1.04	1.04	0.88
KCNH7	KCNH7;ERG3;HERG3;Kv11.3;MGC45986	potassium voltage-gated channel, subfamily H (eag-related), member 7	1.04	1.08	1.04	0.84
C1S	C1S;FLJ44757	complement component 1, s subcomponent	1.04	1.07	1.04	0.81
NKX6-1	NKX6-1;NKX6.1;NKX6A	NK6 homeobox 1	1.04	1.05	1.04	0.78
AXIN1	AXIN1;AXIN;MGC52315;PPP1R49	axin 1	1.04	1.07	1.04	0.89
TMEM65	TMEM65	transmembrane protein 65	1.04	1.05	1.04	0.78
PHF23	PHF23;FLJ16355;FLJ22884;MGC2941;hJUNE-1b	PHD finger protein 23	1.04	1.04	1.04	0.75
PPP1R39	PPP1R39;FLJ23654;HEPP1;MGC149788;MGC149789;MGC90410;RNF158;SH3RF2	protein phosphatase 1, regulatory subunit 39	1.04	1.04	1.04	0.75
CD177	CD177;HNA-2a;HNA2A;NB1;NB1 GP;PRV-1;PRV1	CD177 molecule	1.03	1.07	1.04	0.77
COPS4	COPS4;CSN4;MGC10899;MGC15160	COP9 constitutive photomorphogenic homolog subunit 4 (Arabidopsis)	1.03	1.02	1.04	0.86
TAS2R31	TAS2R31;T2R31;T2R44;T2R53;TAS2R44	taste receptor, type 2, member 31	1.03	1.1	1.04	0.75
EPX	EPX;EPO;EPP;EPX-PEN	eosinophil peroxidase	1.02	1.03	1.04	0.73
ABP1	ABP1;ABP;AOC1;DAO;DAO1;KAO	amiloride binding protein 1 (amine oxidase (copper-containing))	1.02	1.12	1.04	0.87
LY6H	LY6H;NMLY6	lymphocyte antigen 6 complex, locus H	1.02	1.04	1.04	0.87
PTPN4	PTPN4;PTPMEG;PTPMEG1	protein tyrosine phosphatase, non-receptor type 4 (megakaryocyte)	1.02	0.98	1.04	0.74
KCNJ12	KCNJ12;FLJ14167;IRK-2;IRK2;KCNJN1;Kir2.2;Kir2.2v;hIRK;hIRK1;hkir2.2x;kcnj12x	potassium inwardly-rectifying channel, subfamily J, member 12	1.02	1.06	1.04	0.83
RABL3	RABL3;MGC23920	RAB, member of RAS oncogene family-like 3	1.02	1.02	1.04	0.74
CEACAM1	CEACAM1;BGP;BGP1;BGPI	carcinoembryonic antigen-related cell adhesion molecule 1 (biliary glycoprotein)	1.01	1.04	1.04	0.86
SLC2A3	SLC2A3;FLJ90380;GLUT3	solute carrier family 2 (facilitated glucose transporter), member 3	1.01	1.05	1.04	0.82
NUP85	NUP85;FLJ12549;Nup75	nucleoporin 85kDa	1.01	1.02	1.04	0.73
SLC35B1	SLC35B1;UGTREL1	solute carrier family 35, member B1	1.01	1.09	1.04	0.75
VSIG10	VSIG10;FLJ20674;MGC1013	V-set and immunoglobulin domain containing 10	1.01	1.04	1.04	0.86
C20orf26	C20orf26;DKFZp434K156;dJ1002M8.3;dJ1178H5.4	chromosome 20 open reading frame 26	1	1.06	1.04	0.81
ACSL4	ACSL4;ACS4;FACL4;LACS4;MRX63;MRX68	acyl-CoA synthetase long-chain family member 4	1	1.1	1.04	0.83
CCAR1	CCAR1;MGC44628;RP11-437A18.1	cell division cycle and apoptosis regulator 1	1	1.08	1.04	0.82
MTMR7	MTMR7;DKFZp781E194;MGC163449;MGC163451	myotubularin related protein 7	0.99	0.95	1.04	0.72
PARP9	PARP9;BAL;BAL1;DKFZp666B0810;DKFZp686M15238;FLJ26637;FLJ35310;FLJ41418;FLJ43593;MGC:7868	poly (ADP-ribose) polymerase family, member 9	0.99	1.07	1.04	0.77
C11orf2	C11orf2;ANG2;ANG3;C11orf3;FFR;FLJ13879;FLJ36472	chromosome 11 open reading frame 2	0.99	1.03	1.04	0.7

CLASP1	CLASP1;DKFZp686D1968;DKFZp686H2039;FLJ33821;FLJ41222;KIAA0622;MAST1;MGC131895	cytoplasmic linker associated protein 1	0.99	0.94	1.04	0.74
NPHS1	NPHS1;CNF;NPHN;nephrin	nephrosis 1, congenital, Finnish type (nephrin)	0.98	1.07	1.04	0.71
MTSS1	MTSS1;DKFZp781P2223;FLJ44694;KIAA0429;MIM;MIMA;MIMB	metastasis suppressor 1	0.98	0.98	1.04	0.7
PDE1C	PDE1C;Hcam3	phosphodiesterase 1C, calmodulin-dependent 70kDa	0.98	1.09	1.04	0.72
CHST10	CHST10;HNK-1ST;HNK1ST;MGC17148	carbohydrate sulfotransferase 10	0.98	0.94	1.04	0.83
VTI1B	VTI1B;VTI1;VTI1-LIKE;VTI1L;VTI2;v-SNARE;vti1-rp1	vesicle transport through interaction with t-SNAREs homolog 1B (yeast)	0.98	1.05	1.04	0.71
ATP13A1	ATP13A1;ATP13A;DKFZp761L1623;FLJ31858;FLJ41786;FLJ43873;FLJ90317;KIAA1825	ATPase type 13A1	0.98	0.95	1.04	0.76
SPECC1	SPECC1;CYTSB;FLJ36955;HCMOGT-1;HCMOGT1;NSP	sperm antigen with calponin homology and coiled-coil domains 1	0.98	1.08	1.04	0.83
GGA2	GGA2;FLJ20966;KIAA1080;VEAR	golgi-associated, gamma adaptin ear containing, ARF binding protein 2	0.98	1.01	1.04	0.69
CCDC15	CCDC15;FLJ13215	coiled-coil domain containing 15	0.97	1.04	1.04	0.78
PKP3	PKP3	plakophilin 3	0.97	0.96	1.04	0.81
ZBTB7B	ZBTB7B;DKFZp686G01254;THPOK;ZBTB15;ZFP67;ZNF857B;c-Krox;hcKrox	zinc finger and BTB domain containing 7B	0.97	0.99	1.04	0.7
PI4KAP2	PI4KAP2;FLJ44912;MGC31920	phosphatidylinositol 4-kinase, catalytic, alpha pseudogene 2	0.97	1.02	1.04	0.74
TERF1	TERF1;FLJ41416;PIN2;TRBF1;TRF;TRF1;hTRF1-AS;t-TRF1	telomeric repeat binding factor (NIMA-interacting) 1	0.97	0.96	1.04	0.7
ERMP1	ERMP1;FXNA;KIAA1815;bA207C16.3	endoplasmic reticulum metallopeptidase 1	0.97	1.05	1.04	0.79
CD1A	CD1A;CD1;FCB6;HTA1;R4;T6	CD1a molecule	0.96	0.96	1.04	0.73
TMEM176A	TMEM176A;GS188;HCA112	transmembrane protein 176A	0.96	1.07	1.04	0.82
DDO	DDO;DASOX;DDO-1;DDO-2;FLJ45203	D-aspartate oxidase	0.96	1.06	1.04	0.7
ARSE	ARSE;ASE;CDPX;CDPX1;CDPXR;MGC163310	arylsulfatase E (chondrodysplasia punctata 1)	0.96	0.97	1.04	0.71
LIFR	LIFR;CD118;FLJ98106;FLJ99923;LIF-R;SJS2;STWS;SWS	leukemia inhibitory factor receptor alpha	0.96	1	1.04	0.74
FAM107A	FAM107A;DRR1;FLJ30158;FLJ45473;TU3A	family with sequence similarity 107, member A	0.96	0.98	1.04	0.73
LDLRAD3	LDLRAD3	low density lipoprotein receptor class A domain containing 3	0.96	0.98	1.04	0.74
C12orf5	C12orf5;TIGAR	chromosome 12 open reading frame 5	0.96	0.96	1.04	0.69
IDE	IDE;FLJ35968;INSULYSIN	insulin-degrading enzyme	0.96	0.98	1.04	0.74
HEBP2	HEBP2;C6ORF34B;C6orf34;KIAA1244;PP23;SOUL	heme binding protein 2	0.96	0.9	1.04	0.71
KDM4D	KDM4D;FLJ10251;JMJD2D;MGC141909	lysine (K)-specific demethylase 4D	0.96	1.01	1.04	0.69
SPINK5	SPINK5;DKFZp686K19184;FLJ21544;FLJ97536;FLJ97596;FLJ99794;LEKTI;LETKI;NETS;NS;VAKTI	serine peptidase inhibitor, Kazal type 5	0.95	0.91	1.04	0.8
TMEM209	TMEM209;FLJ14803;NET31	transmembrane protein 209	0.95	1.02	1.04	0.77
LLPH	LLPH;C12orf31;MGC104302;MGC14817;hLLP	LLP homolog, long-term synaptic facilitation (Aplysia)	0.95	0.99	1.04	0.71
AHSG	AHSG;A2HS;AHS;FETUA;HSGA	alpha-2-HS-glycoprotein	0.94	1.02	1.04	0.76
PQLC1	PQLC1;FLJ22378	PQ loop repeat containing 1	0.94	1.01	1.04	0.73
MSX1	MSX1;HOX7;HYD1;STHAG1	msh homeobox 1	0.94	0.98	1.04	0.76
SLC25A30	SLC25A30;KMCP1	solute carrier family 25, member 30	0.94	1.02	1.04	0.76
C6orf130	C6orf130;FLJ17636;MGC19570;dJ34B21.3	chromosome 6 open reading frame 130	0.94	1.02	1.04	0.68
TRPM7	TRPM7;CHAK;CHAK1;FLJ20117;FLJ25718;LTRPC7;TRP-PLIK	transient receptor potential cation channel, subfamily M, member 7	0.94	0.97	1.04	0.76
SNX24	SNX24;PRO1284	sorting nexin 24	0.94	0.98	1.04	0.67
C3orf58	C3orf58;DIA1;MGC33365	chromosome 3 open reading frame 58	0.94	1.05	1.04	0.8
C1orf213	C1orf213;FLJ90508	chromosome 1 open reading frame 213	0.93	1.01	1.04	0.72
MOGAT3	MOGAT3;DC7;DGAT2L7;MGAT3;MGC119203;MGC119204	monoacylglycerol O-acyltransferase 3	0.93	0.94	1.04	0.66
TACR1	TACR1;NK1R;NKIR;SPR;TAC1R	tachykinin receptor 1	0.93	0.93	1.04	0.85
ANKRD6	ANKRD6;FLJ12637;FLJ34916	ankyrin repeat domain 6	0.93	0.94	1.04	0.71
TOMM40	TOMM40;C19orf1;D19S1177E;PER-EC1;PEREC1;TOM40	translocase of outer mitochondrial membrane 40 homolog (yeast)	0.93	1.04	1.04	0.8
PCDHGA11	PCDHGA11;PCDH-GAMMA-A11	protocadherin gamma subfamily A, 11	0.93	0.94	1.04	0.74
SLC4A10	SLC4A10;NBCn2;NCBE	solute carrier family 4, sodium bicarbonate transporter, member 10	0.92	0.91	1.04	0.71
SLC35E4	SLC35E4;MGC129826	solute carrier family 35, member E4	0.92	0.87	1.04	0.69
STXBP4	STXBP4;FLJ16496;MGC149829;MGC50337;Synip	syntaxin binding protein 4	0.92	1	1.04	0.67
NECAB3	NECAB3;APBA2BP;EFCBP3;NIP1;STIP3;SYTIP2;XB51;dJ63M2.4;dJ63M2.5	N-terminal EF-hand calcium binding protein 3	0.92	0.93	1.04	0.7
MCTS1	MCTS1;FLJ39637;MCT-1;MCT1	malignant T cell amplified sequence 1	0.92	0.94	1.04	0.7
TP53RK	TP53RK;BUD32;C20orf64;Nori-2;Nori-2p;PRPK;dJ101A2	TP53 regulating kinase	0.92	0.95	1.04	0.71
PIGA	PIGA;GPI3;PIG-A	phosphatidylinositol glycan anchor biosynthesis, class A	0.92	0.94	1.04	0.7
CHCHD2	CHCHD2;C7orf17	coiled-coil-helix-coiled-coil-helix domain containing 2	0.92	0.93	1.04	0.73
RTP1	RTP1;MGC35450	receptor (chemosensory) transporter protein 1	0.91	0.92	1.04	0.66
TMEM91	TMEM91;FLJ27310;FLJ45695;IFITMD6	transmembrane protein 91	0.91	0.99	1.04	0.67
SLC25A1	SLC25A1;CTP;SEA;SLC20A3	solute carrier family 25 (mitochondrial carrier; citrate transporter), member 1	0.91	0.88	1.04	0.78
CCDC62	CCDC62;CT109;ERAP75;FLJ25432;FLJ40344;TSP-NY	coiled-coil domain containing 62	0.9	0.96	1.04	0.72
CASR	CASR;CAR;EIG8;FHH;FIH;GPRC2A;HHC;HHC1;MGC138441;NSHPT;PCAR1	calcium-sensing receptor	0.9	0.91	1.04	0.82
LCE2D	LCE2D;LEP12;SPRL1A	late cornified envelope 2D	0.9	0.96	1.04	0.71
CDH4	CDH4;CAD4;FLJ22202;FLJ40547;MGC126700;MGC138355;RCAD	cadherin 4, type 1, R-cadherin (retinal)	0.9	0.84	1.04	0.71
LYPD6	LYPD6;MGC52057	LY6/PLAUR domain containing 6	0.9	0.98	1.04	0.72
ASRGL1	ASRGL1;ALP;ALP1;CRASH;FLJ22316	asparaginase like 1	0.9	0.94	1.04	0.63
ALAS1	ALAS1;ALAS;ALAS3;ALASH;MIG4	aminolevulinate, delta-, synthase 1	0.9	0.92	1.04	0.65
RAB10	RAB10	RAB10, member RAS oncogene family	0.9	0.88	1.04	0.7
CIB4	CIB4;KIP4	calcium and integrin binding family member 4	0.89	1	1.04	0.67
ZNF230	ZNF230;FDZF2	zinc finger protein 230	0.89	0.82	1.04	0.69
EFNB3	EFNB3;EFL6;EPLG8;LERK8	ephrin-B3	0.89	0.89	1.04	0.81
CEP135	CEP135;CEP4;KIAA0635	centrosomal protein 135kDa	0.89	1	1.04	0.77
CDK3	CDK3	cyclin-dependent kinase 3	0.89	0.9	1.04	0.8
HIPK2	HIPK2;DKFZp686K02111;FLJ23711;PRO0593	homeodomain interacting protein kinase 2	0.89	0.99	1.04	0.71
GOT1L1	GOT1L1;MGC33309	glutamic-oxaloacetic transaminase 1-like 1	0.88	0.88	1.04	0.63
PDE4A	PDE4A;DPDE2;PDE4;PDE46	phosphodiesterase 4A, cAMP-specific	0.88	0.84	1.04	0.75
GNB5	GNB5;FLJ37457;FLJ43714;GB5	guanine nucleotide binding protein (G protein), beta 5	0.88	0.88	1.04	0.72
PFKFB2	PFKFB2;DKFZp781D2217;MGC138308;MGC138310;PFK-2/FBPase-2	6-phosphofructo-2-kinase/fructose-2,6-biphosphatase 2	0.88	0.84	1.04	0.64
GOPC	GOPC;CAL;FIG;GOPC1;PIST;dJ94G16.2	golgi-associated PDZ and coiled-coil motif containing	0.88	0.91	1.04	0.67
RNF181	RNF181	ring finger protein 181	0.88	0.9	1.04	0.81
CIZ1	CIZ1;LSFR1;NP94;ZNF356	CDKN1A interacting zinc finger protein 1	0.88	0.99	1.04	0.74
SUMO2	SUMO2;HSMT3;MGC117191;SMT3B;SMT3H2;SUMO3;Smt3A	SMT3 suppressor of mif two 3 homolog 2 (S. cerevisiae)	0.88	0.9	1.04	0.75
HYOU1	HYOU1;DKFZp686N08236;FLJ94899;FLJ97572;Grp170;HSP12A;ORP150	hypoxia up-regulated 1	0.88	1	1.04	0.75
CERS5	CERS5;FLJ25304;LASS5;MGC45411;Trh4	ceramide synthase 5	0.88	1	1.04	0.74
GTF2H2D	GTF2H2D;DKFZp686M0199;GTF2H2C;MGC75049;MGC75050	general transcription factor IIH, polypeptide 2D	0.88	0.95	1.04	0.72
RP1L1	RP1L1;DCDC4B	retinitis pigmentosa 1-like 1	0.87	0.87	1.04	0.67
GRIK3	GRIK3;EAA5;GLR7;GLUR7;GluR7a	glutamate receptor, ionotropic, kainate 3	0.87	0.95	1.04	0.74
ALDH1B1	ALDH1B1;ALDH5;ALDHX;MGC2230	aldehyde dehydrogenase 1 family, member B1	0.87	0.88	1.04	0.65
CEACAM18	CEACAM18	carcinoembryonic antigen-related cell adhesion molecule 18	0.87	0.89	1.04	0.74
KIAA1407	KIAA1407;FLJ43314	KIAA1407	0.86	0.92	1.04	0.7
ESR1	ESR1;DKFZp686N23123;ER;ESR;ESRA;Era;NR3A1	estrogen receptor 1	0.86	0.86	1.04	0.62
NDUFS4	NDUFS4;AQDQ;CI-18	NADH dehydrogenase (ubiquinone) Fe-S protein 4, 18kDa (NADH-coenzyme Q reductase)	0.86	0.97	1.04	0.72
NFASC	NFASC;DKFZp686P2250;FLJ46866;KIAA0756;NF;NRCAML	neurofascin	0.86	0.88	1.04	0.66
KCNH3	KCNH3;BEC1;ELK2;KIAA1282;Kv12.2	potassium voltage-gated channel, subfamily H (eag-related), member 3	0.86	0.92	1.04	0.71
NRARP	NRARP;MGC61598	NOTCH-regulated ankyrin repeat protein	0.86	0.8	1.04	0.62
FGFR1	FGFR1;BFGFR;CD331;CEK;FGFBR;FGFR-1;FLG;FLJ99988;FLT-2;FLT2;HBGFR;KAL2;N-SAM;OGD;bFGF-R-1	fibroblast growth factor receptor 1	0.86	0.88	1.04	0.73
HOMER1	HOMER1;HOMER;HOMER1A;HOMER1B;HOMER1C;SYN47;Ves-1	homer homolog 1 (Drosophila)	0.86	0.92	1.04	0.63
RBPMS	RBPMS;FLJ32971;HERMES	RNA binding protein with multiple splicing	0.86	0.94	1.04	0.66
POLR1E	POLR1E;FLJ13390;FLJ13970;FLJ43482;PAF53;PRAF1;RP11-405L18.3	polymerase (RNA) I polypeptide E, 53kDa	0.86	0.86	1.04	0.66
ATF1	ATF1;EWS-ATF1;FUS/ATF-1;TREB36	activating transcription factor 1	0.86	0.88	1.04	0.65
BAG2	BAG2;BAG-2;KIAA0576;MGC149462;dJ417I1.2	BCL2-associated athanogene 2	0.86	0.96	1.04	0.72
RHOQ	RHOQ;ARHQ;RASL7A;TC10;TC10A	ras homolog gene family, member Q	0.86	0.84	1.04	0.66
FBXL3	FBXL3;FBL3;FBL3A;FBXL3A	F-box and leucine-rich repeat protein 3	0.86	0.86	1.04	0.62
OR4N5	OR4N5	olfactory receptor, family 4, subfamily N, member 5	0.85	0.9	1.04	0.69
C13orf15	C13orf15;KIAA0564;MGC87338;RGC-32;RGC32;bA157L14.2	chromosome 13 open reading frame 15	0.85	0.88	1.04	0.64
WDR73	WDR73;FLJ14888	WD repeat domain 73	0.85	0.84	1.04	0.68
SMPD4	SMPD4;FLJ20297;FLJ20756;KIAA1418;NET13;NSMASE-3;NSMASE3	sphingomyelin phosphodiesterase 4, neutral membrane (neutral sphingomyelinase-3)	0.85	0.91	1.04	0.66
PMS2P3	PMS2P3;MGC126647;PMS2L3;PMS2L9;PMS5;PMSR3	postmeiotic segregation increased 2 pseudogene 3	0.85	0.88	1.04	0.6
FBXL8	FBXL8;FBL8;FLJ11278;MGC19959	F-box and leucine-rich repeat protein 8	1.26	1.27	1.05	0.92
TIMM50	TIMM50;MGC102733;TIM50;TIM50L	translocase of inner mitochondrial membrane 50 homolog (S. cerevisiae)	1.25	1.31	1.05	1.01
TM9SF2	TM9SF2;FLJ26287;MGC117391;P76	transmembrane 9 superfamily member 2	1.22	1.23	1.05	0.95
MRGPRX3	MRGPRX3;GPCR;MRGX3;SNSR1;SNSR2	MAS-related GPR, member X3	1.21	1.25	1.05	1.12
CALML6	CALML6;CAGLP	calmodulin-like 6	1.2	1.13	1.05	0.88
NDUFAF4	NDUFAF4;C6orf66;HRPAP20;My013;bA22L21.1	NADH dehydrogenase (ubiquinone) 1 alpha subcomplex, assembly factor 4	1.2	1.29	1.05	0.97
CXorf40A	CXorf40A;CXorf40;EOLA1;FLJ52212	chromosome X open reading frame 40A	1.19	1.23	1.05	0.92
HCG27	HCG27;FLJ40123;MGC129794;MGC129795;bCX101P6.9;bPG299F13.9;bQB115I13.2	HLA complex group 27 (non-protein coding)	1.17	1.11	1.05	0.92
IWS1	IWS1;DKFZp761G0123;FLJ10006;FLJ14655;FLJ32319;MGC126375;MGC126376	IWS1 homolog (S. cerevisiae)	1.17	1.09	1.05	0.86
LMO7	LMO7;FBX20;FBXO20;KIAA0858;LOMP	LIM domain 7	1.16	1.2	1.05	1.08
PINX1	PINX1;FLJ20565;LPTL;LPTS;MGC8850	PIN2/TERF1 interacting, telomerase inhibitor 1	1.13	1.15	1.05	0.91
APEH	APEH;ACPH;APH;D3F15S2;D3S48E;DNF15S2;MGC2178;OPH	N-acylaminoacyl-peptide hydrolase	1.13	1.09	1.05	0.82
MYBPC1	MYBPC1;MYBPCC;MYBPCS;slow-type	myosin binding protein C, slow type	1.12	1.17	1.05	0.97
NUDT18	NUDT18;FLJ22494	nudix (nucleoside diphosphate linked moiety X)-type motif 18	1.12	1.11	1.05	0.9
SERINC3	SERINC3;AIGP1;DIFF33;SBBI99;TDE;TDE1;TMS-1	serine incorporator 3	1.12	1.19	1.05	0.92
ITGA4	ITGA4;CD49D;IA4;MGC90518	integrin, alpha 4 (antigen CD49D, alpha 4 subunit of VLA-4 receptor)	1.1	1.12	1.05	0.83
FBXL18	FBXL18;FLJ10776;FLJ11467;FLJ26934;FLJ38075;FLJ41541;Fbl18	F-box and leucine-rich repeat protein 18	1.1	1.1	1.05	0.8
SLC35A1	SLC35A1;CDG2F;CMPST;CST;FLJ76955;hCST	solute carrier family 35 (CMP-sialic acid transporter), member A1	1.1	1.08	1.05	0.86
PFKM	PFKM;GSD7;MGC8699;PFK-1;PFK1;PFKA;PFKX	phosphofructokinase, muscle	1.1	1.13	1.05	1.02
PAQR3	PAQR3;RKTG	progestin and adipoQ receptor family member III	1.09	1.25	1.05	0.92
DCUN1D5	DCUN1D5;FLJ32431;FLJ37425;MGC2714	DCN1, defective in cullin neddylation 1, domain containing 5 (S. cerevisiae)	1.09	1.05	1.05	0.79
RNF25	RNF25;AO7;FLJ13906	ring finger protein 25	1.08	1.08	1.05	0.78
RUFY1	RUFY1;FLJ22251;RABIP4;ZFYVE12	RUN and FYVE domain containing 1	1.08	1.08	1.05	0.78
MTSS1L	MTSS1L;ABBA-1	metastasis suppressor 1-like	1.07	1.22	1.05	0.81
SMPDL3A	SMPDL3A;ASM3A;ASML3a;FLJ20177;yR36GH4.1	sphingomyelin phosphodiesterase, acid-like 3A	1.06	1.23	1.05	0.85
USP4	USP4;MGC149848;MGC149849;UNP;Unph	ubiquitin specific peptidase 4 (proto-oncogene)	1.06	1.09	1.05	0.82
TPMT	TPMT	thiopurine S-methyltransferase	1.06	1.08	1.05	0.79

LACC1	LACC1;C13orf31;DKFZp686D11119;FLJ38725	laccase (multicopper oxidoreductase) domain containing 1	1.06	0.99	1.05	0.78
COA5	COA5;6330578E17Rik;C2orf64;MGC52110;Pet191	cytochrome C oxidase assembly factor 5	1.06	1.08	1.05	0.85
CD53	CD53;MOX44;TSPAN25	CD53 molecule	1.05	1.14	1.05	0.86
RC3H2	RC3H2;FLJ20301;FLJ20713;MGC52176;MNAB;RNF164	ring finger and CCCH-type domains 2	1.05	1.05	1.05	0.76
EIF2C2	EIF2C2;AGO2;MGC3183;Q10	eukaryotic translation initiation factor 2C, 2	1.05	1.06	1.05	0.79
PARK2	PARK2;AR-JP;LPRS2;PDJ;PRKN	parkinson protein 2, E3 ubiquitin protein ligase (parkin)	1.04	1.09	1.05	0.97
PYDC1	PYDC1;ASC2;POP1;PYC1	PYD (pyrin domain) containing 1	1.04	1.2	1.05	0.88
OR10V1	OR10V1;OR11-256	olfactory receptor, family 10, subfamily V, member 1	1.04	1.16	1.05	0.81
SIRT7	SIRT7;MGC126840;MGC126842;SIR2L7	sirtuin 7	1.04	1.08	1.05	0.8
RP9	RP9;PAP-1	retinitis pigmentosa 9 (autosomal dominant)	1.04	1.12	1.05	0.86
KIF6	KIF6;C6orf102;DKFZp451I2418;MGC33317;dJ1043E3.1;dJ137F1.4;dJ188D3.1	kinesin family member 6	1.03	1.12	1.05	0.83
PCNP	PCNP;DKFZp781I24156	PEST proteolytic signal containing nuclear protein	1.03	1.04	1.05	0.82
KRT1	KRT1;CK1;EHK;EHK1;EPPK;K1;KRT1A;NEPPK	keratin 1	1.02	0.97	1.05	0.8
PGLYRP3	PGLYRP3;MGC149197;PGRP-Ialpha;PGRPIA	peptidoglycan recognition protein 3	1.02	1.05	1.05	0.74
TIGD7	TIGD7;FLJ98002;FLJ99447;Sancho	tigger transposable element derived 7	1.02	1.12	1.05	0.84
ZNF238	ZNF238;C2H2-171;RP58;TAZ-1;ZBTB18	zinc finger protein 238	1.02	1.14	1.05	0.75
NPEPL1	NPEPL1;FLJ11583;FLJ42065;bA261P9.2	aminopeptidase-like 1	1.02	0.99	1.05	0.74
RNF166	RNF166;MGC14381;MGC2647	ring finger protein 166	1.02	1.03	1.05	0.74
ZNF180	ZNF180;HHZ168	zinc finger protein 180	1.02	1.03	1.05	0.79
PPAP2B	PPAP2B;Dri42;LPP3;MGC15306;PAP2B;VCIP	phosphatidic acid phosphatase type 2B	1.02	1	1.05	0.75
EFNA1	EFNA1;B61;ECKLG;EFL1;EPLG1;LERK-1;LERK1;TNFAIP4	ephrin-A1	1.01	0.94	1.05	0.79
MTMR2	MTMR2;CMT4B;CMT4B1;KIAA1073	myotubularin related protein 2	1.01	1.05	1.05	0.82
SOX30	SOX30	SRY (sex determining region Y)-box 30	1	0.95	1.05	0.76
AGMAT	AGMAT;FLJ23384	agmatine ureohydrolase (agmatinase)	1	1.12	1.05	0.74
BCAS3	BCAS3;DKFZp686O1527;FLJ20128;GAOB1;MAAB;MGC4973	breast carcinoma amplified sequence 3	1	1.09	1.05	0.75
ANKRD49	ANKRD49;FGIF;FLJ20189;FLJ20441;GBIF	ankyrin repeat domain 49	1	1.14	1.05	0.88
CAPS	CAPS;CAPS1;MGC126562	calcyphosine	1	1.07	1.05	0.82
ATE1	ATE1;MGC26724	arginyltransferase 1	1	1	1.05	0.78
NDUFB8	NDUFB8;ASHI;CI-ASHI	NADH dehydrogenase (ubiquinone) 1 beta subcomplex, 8, 19kDa	1	1.04	1.05	0.78
SNX6	SNX6;MGC3157;MSTP010;TFAF2	sorting nexin 6	1	1.04	1.05	0.87
RFXANK	RFXANK;ANKRA1;BLS;F14150_1;MGC138628;RFX-B	regulatory factor X-associated ankyrin-containing protein	1	1.08	1.05	0.86
PSPHP1	PSPHP1;CO9;PSPHL	phosphoserine phosphatase pseudogene 1	1	1.05	1.05	0.82
FAM179B	FAM179B;DKFZp686D12126;KIAA0423	family with sequence similarity 179, member B	0.99	1.01	1.05	0.76
WDFY2	WDFY2;PROF;RP11-147H23.1;WDF2;ZFYVE22	WD repeat and FYVE domain containing 2	0.99	1.08	1.05	0.81
SPATA13	SPATA13;ARHGEF29;ASEF2;FLJ31208;FLJ35435;MGC129988;MGC129989	spermatogenesis associated 13	0.99	1.02	1.05	0.76
GYG2	GYG2;GN-2;GN2	glycogenin 2	0.99	1	1.05	0.79
MESDC1	MESDC1;MGC99595	mesoderm development candidate 1	0.99	1.07	1.05	0.81
LDHA	LDHA;GSD11;LDH1;LDHM	lactate dehydrogenase A	0.99	1	1.05	0.72
SPTSSB	SPTSSB;ADMP;C3orf57;MGC104229;SSSPTB	serine palmitoyltransferase, small subunit B	0.99	1	1.05	0.81
ODF2L	ODF2L;KIAA1229;MGC111060;RP5-977L11.1;dJ977L11.1	outer dense fiber of sperm tails 2-like	0.98	0.92	1.05	0.72
C2orf43	C2orf43;FLJ21820	chromosome 2 open reading frame 43	0.98	1.07	1.05	0.77
SUGP2	SUGP2;DKFZp686M2322;DKFZp779L2418;FLJ21855;SFRS14	SURP and G patch domain containing 2	0.98	1.07	1.05	0.71
NALCN	NALCN;CanIon;FLJ23913;FLJ44659;FLJ44764;MGC74524;VGCNL1;bA430M15.1	sodium leak channel, non-selective	0.97	1.06	1.05	0.82
SHE	SHE;DKFZp451D1511;DKFZp686E14106;RP11-350G8.8	Src homology 2 domain containing E	0.97	0.96	1.05	0.77
C20orf196	C20orf196;FLJ25067	chromosome 20 open reading frame 196	0.97	0.98	1.05	0.75
UBE2E1	UBE2E1;UBCH6	ubiquitin-conjugating enzyme E2E 1	0.97	0.93	1.05	0.7
BEND5	BEND5;C1orf165;FLJ11588	BEN domain containing 5	0.97	1	1.05	0.83
KCND2	KCND2;KIAA1044;KV4.2;MGC119702;MGC119703;RK5	potassium voltage-gated channel, Shal-related subfamily, member 2	0.96	1.05	1.05	0.81
PLA1A	PLA1A;PS-PLA1;PSPLA1	phospholipase A1 member A	0.96	1.02	1.05	0.81
ZNF782	ZNF782;FLJ16636	zinc finger protein 782	0.96	0.96	1.05	0.77
IAH1	IAH1;MGC102860	isoamyl acetate-hydrolyzing esterase 1 homolog (S. cerevisiae)	0.96	1.07	1.05	0.82
N4BP1	N4BP1;FLJ31821;KIAA0615;MGC176730	NEDD4 binding protein 1	0.96	0.98	1.05	0.7
C14orf159	C14orf159;DKFZp686I02128;FLJ20950;FLJ39943;FLJ39975	chromosome 14 open reading frame 159	0.96	1.05	1.05	0.78
HIRA	HIRA;DGCR1;TUP1;TUPLE1	HIR histone cell cycle regulation defective homolog A (S. cerevisiae)	0.96	1.04	1.05	0.8
CBWD1	CBWD1;COBP	COBW domain containing 1	0.96	0.98	1.05	0.73
PITRM1	PITRM1;KIAA1104;MGC138192;MGC141929;MP1;PreP	pitrilysin metallopeptidase 1	0.96	1	1.05	0.75
ALDH18A1	ALDH18A1;GSAS;MGC117316;P5CS;PYCS	aldehyde dehydrogenase 18 family, member A1	0.96	1.09	1.05	0.78
KCNK13	KCNK13;K2p13.1;THIK-1;THIK1	potassium channel, subfamily K, member 13	0.94	0.98	1.05	0.76
SLC2A12	SLC2A12;GLUT12;GLUT8	solute carrier family 2 (facilitated glucose transporter), member 12	0.94	0.96	1.05	0.69
FRAT2	FRAT2;MGC10562	frequently rearranged in advanced T-cell lymphomas 2	0.94	0.96	1.05	0.69
RIN2	RIN2;MACS;RASSF4	Ras and Rab interactor 2	0.94	1.01	1.05	0.68
POLE3	POLE3;CHARAC17;CHRAC17;YBL1;p17	polymerase (DNA directed), epsilon 3 (p17 subunit)	0.94	0.99	1.05	0.77
AP1M1	AP1M1;AP47;CLAPM2;CLTNM;MU-1A	adaptor-related protein complex 1, mu 1 subunit	0.94	1.02	1.05	0.78
UBAP2	UBAP2;FLJ22435;FLJ43947;FLJ46567;KIAA1491;UBAP-2;bA176F3.5	ubiquitin associated protein 2	0.94	0.94	1.05	0.74
TMEM106C	TMEM106C;MGC111210;MGC5576	transmembrane protein 106C	0.94	1.06	1.05	0.81
C2orf40	C2orf40;ECRG4	chromosome 2 open reading frame 40	0.93	1	1.05	0.75
HVCN1	HVCN1;HV1;MGC15619;VSOP	hydrogen voltage-gated channel 1	0.93	1.03	1.05	0.79
SLC44A5	SLC44A5;CTL5;FLJ34081;MGC34032	solute carrier family 44, member 5	0.93	0.94	1.05	0.72
DRP2	DRP2;MGC133255	dystrophin related protein 2	0.92	1	1.05	0.71
CHSY3	CHSY3;CHSY2;CSS3	chondroitin sulfate synthase 3	0.92	0.97	1.05	0.71
ZNF610	ZNF610;DKFZp547A1010;FLJ36040;MGC102679	zinc finger protein 610	0.92	0.86	1.05	0.69
SLC7A3	SLC7A3;ATRC3;CAT-3;CAT3;FLJ14541;MGC20687	solute carrier family 7 (cationic amino acid transporter, y+ system), member 3	0.92	0.95	1.05	0.71
TG	TG;AITD3;TGN	thyroglobulin	0.92	1.02	1.05	0.67
GRHL1	GRHL1;LBP32;MGR;NH32;TFCP2L2	grainyhead-like 1 (Drosophila)	0.92	1.04	1.05	0.8
SLC43A2	SLC43A2;FLJ23848;LAT4;MGC34680	solute carrier family 43, member 2	0.92	0.95	1.05	0.71
ZNF473	ZNF473;HZFP100;ZN473	zinc finger protein 473	0.92	0.98	1.05	0.66
NR2C2AP	NR2C2AP;TRA16	nuclear receptor 2C2-associated protein	0.92	0.88	1.05	0.69
LRTOMT	LRTOMT;COMT2;DFNB63;FLJ52451;LRRC51;LRTOMT1;LRTOMT2	leucine rich transmembrane and 0-methyltransferase domain containing	0.92	0.97	1.05	0.66
RGL4	RGL4;MGC119678;MGC119680;Rgr	ral guanine nucleotide dissociation stimulator-like 4	0.9	0.86	1.05	0.71
ATOH1	ATOH1;ATH1;HATH1;MATH-1;bHLHa14	atonal homolog 1 (Drosophila)	0.9	0.98	1.05	0.73
EPO	EPO;EP;MGC138142;MVCD2	erythropoietin	0.9	0.98	1.05	0.74
SLC46A1	SLC46A1;FLJ39875;HCP1;MGC9564;PCFT	solute carrier family 46 (folate transporter), member 1	0.9	0.92	1.05	0.67
ZNF367	ZNF367;CDC14B;FLJ33970;ZFF29	zinc finger protein 367	0.9	0.92	1.05	0.77
SCFD2	SCFD2;FLJ21060;FLJ39514;STXBP1L1	sec1 family domain containing 2	0.9	0.98	1.05	0.66
FADS2	FADS2;D6D;DES6;FADSD6;LLCDL2;SLL0262;TU13	fatty acid desaturase 2	0.9	0.88	1.05	0.7
CDKN2AIP	CDKN2AIP;CARF;FLJ20036	CDKN2A interacting protein	0.9	0.96	1.05	0.72
MAGOH	MAGOH;MAGOH1;MAGOHA	mago-nashi homolog, proliferation-associated (Drosophila)	0.9	0.96	1.05	0.65
SOD2	SOD2;IPOB;MNSOD;MVCD6	superoxide dismutase 2, mitochondrial	0.9	1.01	1.05	0.67
IMPDH2	IMPDH2;IMPD2;IMPDH-II	IMP (inosine 5'-monophosphate) dehydrogenase 2	0.9	0.91	1.05	0.67
BRD7	BRD7;BP75;CELTIX1;NAG4	bromodomain containing 7	0.9	0.89	1.05	0.75
ANP32A	ANP32A;C15orf1;I1PP2A;LANP;MAPM;MGC119787;MGC150373;PHAP1;PHAPI;PP32	acidic (leucine-rich) nuclear phosphoprotein 32 family, member A	0.9	0.96	1.05	0.66
CRIM1	CRIM1;CRIM-1;MGC138194;S52	cysteine rich transmembrane BMP regulator 1 (chordin-like)	0.9	0.94	1.05	0.78
C3orf23	C3orf23;DKFZp313N0621;FLJ41686;MGC119530;MGC119531;MGC119532;MGC119533;TOAG1	chromosome 3 open reading frame 23	0.89	1	1.05	0.7
TXNL4B	TXNL4B;DLP;Dim2;FLJ20511	thioredoxin-like 4B	0.89	0.92	1.05	0.71
FAM69B	FAM69B;C9orf136;MGC20262;pp6977	family with sequence similarity 69, member B	0.89	1	1.05	0.77
RCAN3	RCAN3;DSCR1L2;MCIP3;RCN3;hRCN3	RCAN family member 3	0.89	0.91	1.05	0.69
SERPINA9	SERPINA9;GCET1;MGC131906;SERPINA11;SERPINA11b	serpin peptidase inhibitor, clade A (alpha-1 antiproteinase, antitrypsin), member 9	0.88	0.96	1.05	0.66
RASGEF1A	RASGEF1A;CG4853;FLJ37817	RasGEF domain family, member 1A	0.88	0.94	1.05	0.67
WAPAL	WAPAL;FOE;KIAA0261;WAPL	wings apart-like homolog (Drosophila)	0.88	0.89	1.05	0.68
ERO1L	ERO1L;ERO1-alpha;ERO1A	ERO1-like (S. cerevisiae)	0.88	1	1.05	0.75
DLL4	DLL4;MGC126344;hdelta2	delta-like 4 (Drosophila)	0.87	0.86	1.05	0.73
CDSN	CDSN;D6S586E;HTSS;HTSS1;PSS;S	corneodesmosin	0.87	0.86	1.05	0.73
TIMMDC1	TIMMDC1;C3orf1;FLJ22597	translocase of inner mitochondrial membrane domain containing 1	0.87	0.95	1.05	0.64
APCS	APCS;MGC88159;PTX2;SAP	amyloid P component, serum	0.86	0.82	1.05	0.66
RHOH	RHOH;ARHH;TTF	ras homolog gene family, member H	0.86	0.84	1.05	0.74
GDPD2	GDPD2;FLJ20207;GDE3;OBDPF	glycerophosphodiester phosphodiesterase domain containing 2	0.86	0.86	1.05	0.64
MEOX2	MEOX2;GAX;MOX2	mesenchyme homeobox 2	0.86	0.84	1.05	0.67
HPCA	HPCA;BDR2	hippocalcin	0.86	0.87	1.05	0.67
SPHK2	SPHK2;SK 2;SK-2;SPK 2;SPK-2	sphingosine kinase 2	0.86	0.98	1.05	0.7
TNNT1	TNNT1;ANM;FLJ98147;MGC104241;STNT;TNT;TNTS	troponin T type 1 (skeletal, slow)	0.86	0.87	1.05	0.7
INSR	INSR;CD220;HHF5	insulin receptor	0.86	0.96	1.05	0.69
TSPAN2	TSPAN2;FLJ12082;NET3;TSN2;TSPAN-2	tetraspanin 2	0.86	0.88	1.05	0.66
LIG1	LIG1;MGC117397;MGC130025	ligase I, DNA, ATP-dependent	0.86	0.98	1.05	0.73
NIPA1	NIPA1;FSP3;MGC102724;MGC35570;SPG6	non imprinted in Prader-Willi/Angelman syndrome 1	0.86	0.89	1.05	0.67
RELA	RELA;MGC131774;NFKB3;p65	v-rel reticuloendotheliosis viral oncogene homolog A (avian)	0.86	0.9	1.05	0.75
LRRFIP1	LRRFIP1;FLAP-1;FLAP1;FLIIAP1;GCF-2;GCF2;HUFI-1;MGC10947;MGC119738;MGC119739;TRIP	leucine rich repeat (in FLII) interacting protein 1	0.86	0.88	1.05	0.66
NPR2	NPR2;AMDM;ANPRB;ANPb;GUC2B;GUCY2B;NPRB;NPRBi	natriuretic peptide receptor B/guanylate cyclase B (atrionatriuretic peptide receptor B)	0.85	0.89	1.05	0.74
FAM83F	FAM83F	family with sequence similarity 83, member F	0.85	0.85	1.05	0.69
FOXN2	FOXN2;HTLF	forkhead box N2	0.85	0.96	1.05	0.63
EGFR	EGFR;ERBB;ERBB1;HER1;PIG61;mENA	epidermal growth factor receptor	0.85	0.96	1.05	0.68
TMEM189	TMEM189;KUA	transmembrane protein 189	0.85	0.8	1.05	0.67
ATRNL1	ATRNL1;ALP;FLJ45344;KIAA0534;bA338L11.1;bA454H24.1	attractin-like 1	1.38	1.42	1.06	1.08
TRIM63	TRIM63;FLJ32380;IRF;MURF1;MURF2;RNF28;SMRZ	tripartite motif containing 63	1.24	1.25	1.06	0.9
PCDHB14	PCDHB14;MGC120065;PCDH-BETA14	protocadherin beta 14	1.21	1.32	1.06	0.88
TP73	TP73;P73	tumor protein p73	1.21	1.19	1.06	0.95
GRB10	GRB10;GRB-IR;Grb-10;IRBP;KIAA0207;MEG1;RSS	growth factor receptor-bound protein 10	1.2	1.24	1.06	0.94
RNF112	RNF112;BFP;FLJ99413;ZNF179	ring finger protein 112	1.18	1.21	1.06	0.87
CHI3L2	CHI3L2;YKL-39;YKL39	chitinase 3-like 2	1.17	1.21	1.06	0.86
RNF122	RNF122;FLJ12526;MGC126622	ring finger protein 122	1.17	1.19	1.06	0.85

C9orf150	C9orf150;FLJ38505;FLJ90271;MGC46502;bA3L8.2	chromosome 9 open reading frame 150	1.14	1.17	1.06	0.92
TMEM64	TMEM64;DKFZp762C1112	transmembrane protein 64	1.14	1.26	1.06	0.9
DTX3L	DTX3L;BBAP	deltex 3-like (Drosophila)	1.14	1.15	1.06	0.83
RNF217	RNF217;C6orf172;IBRDC1;MGC26996;dJ84N20.1	ring finger protein 217	1.13	1.19	1.06	1.07
RANBP1	RANBP1;HTF9A;MGC88701	RAN binding protein 1	1.13	1.16	1.06	0.91
RFPL2	RFPL2;RNF79	ret finger protein-like 2	1.12	1.14	1.06	0.83
GPR146	GPR146;PGR8	G protein-coupled receptor 146	1.12	1.1	1.06	0.83
IRX5	IRX5;IRX-2a;IRXB2	iroquois homeobox 5	1.12	1.19	1.06	0.8
ZDHHC12	ZDHHC12;FLJ14524;MGC13153;MGC54050;ZNF400	zinc finger, DHHC-type containing 12	1.12	1.13	1.06	0.86
TMTC3	TMTC3;DKFZp686C0968;DKFZp686M1969;DKFZp686O22167;DKFZp686O2342;FLJ90492;SMILE	transmembrane and tetratricopeptide repeat containing 3	1.12	1.27	1.06	0.97
BATF	BATF;B-ATF;BATF1;SFA-2;SFA2	basic leucine zipper transcription factor, ATF-like	1.11	1.19	1.06	0.95
BAP1	BAP1;DKFZp686N04275;FLJ35406;FLJ37180;HUCEP-13;KIAA0272;UCHL2	BRCA1 associated protein-1 (ubiquitin carboxy-terminal hydrolase)	1.1	1.15	1.06	0.86
DPYSL4	DPYSL4;CRMP3;DRP-4;ULIP4	dihydropyrimidinase-like 4	1.08	1.21	1.06	0.8
DTX4	DTX4;KIAA0937;MGC141899;RNF155	deltex homolog 4 (Drosophila)	1.08	1.09	1.06	0.79
LRIG1	LRIG1;DKFZp586O1624;LIG-1;LIG1	leucine-rich repeats and immunoglobulin-like domains 1	1.08	1.18	1.06	0.81
MMP7	MMP7;MMP-7;MPSL1;PUMP-1	matrix metallopeptidase 7 (matrilysin, uterine)	1.08	1.13	1.06	0.85
AEBP2	AEBP2;MGC17922	AE binding protein 2	1.08	1.19	1.06	0.84
ELOF1	ELOF1;ELF1	elongation factor 1 homolog (S. cerevisiae)	1.08	1.25	1.06	0.84
PCYOX1	PCYOX1;KIAA0908;PCL1	prenylcysteine oxidase 1	1.07	1.08	1.06	0.78
STON1	STON1;DKFZp781K2462;FLJ95267;MGC149803;MGC149804;SBLF;STN1;STNB1	stonin 1	1.07	1.02	1.06	0.8
CPA5	CPA5;FLJ35969	carboxypeptidase A5	1.06	1.12	1.06	0.84
ZNF419	ZNF419;FLJ23233;ZNF419A	zinc finger protein 419	1.06	1.15	1.06	0.86
RNF7	RNF7;CKBBP1;ROC2;SAG	ring finger protein 7	1.06	1.08	1.06	0.78
SEC24C	SEC24C;KIAA0079	SEC24 family, member C (S. cerevisiae)	1.06	1.08	1.06	0.82
CHD1	CHD1;DKFZp686E2337	chromodomain helicase DNA binding protein 1	1.05	1.06	1.06	0.77
GCM1	GCM1;GCMA;hGCMa	glial cells missing homolog 1 (Drosophila)	1.04	1.06	1.06	0.76
RAB30	RAB30	RAB30, member RAS oncogene family	1.04	1.06	1.06	0.76
BCL6	BCL6;BCL5;BCL6A;LAZ3;ZBTB27;ZNF51	B-cell CLL/lymphoma 6	1.04	1.11	1.06	0.75
MGRN1	MGRN1;KIAA0544;RNF156	mahogunin, ring finger 1	1.04	1.05	1.06	0.76
MED23	MED23;ARC130;CRSP130;CRSP133;CRSP3;DKFZp434H0117;DRIP130;MRT18;SUR-2;SUR2	mediator complex subunit 23	1.04	1.06	1.06	0.83
COL12A1	COL12A1;BA209D8.1;COL12A1L;DJ234P15.1	collagen, type XII, alpha 1	1.04	1.08	1.06	0.81
F3	F3;CD142;FLJ17960;TF;TFA	coagulation factor III (thromboplastin, tissue factor)	1.04	1.12	1.06	0.75
TSPAN14	TSPAN14;DC-TM4F2;MGC11352;TM4SF14	tetraspanin 14	1.04	1.08	1.06	0.81
XPNPEP1	XPNPEP1;APP1;SAMP;XPNPEP;XPNPEPL;XPNPEPL1	X-prolyl aminopeptidase (aminopeptidase P) 1, soluble	1.04	1.02	1.06	0.77
SGK494	SGK494;FLJ25006;FLJ78978;MGC39533	uncharacterized serine/threonine-protein kinase SgK494	1.04	1.19	1.06	0.85
DPY19L2	DPY19L2;FLJ32949;FLJ36166;SPGF9	dpy-19-like 2 (C. elegans)	1.03	1.13	1.06	0.81
CD8B	CD8B;CD8B1;LEU2;LY3;LYT3;MGC119115;P37	CD8b molecule	1.03	1.08	1.06	0.78
KIF13B	KIF13B;GAKIN;KIAA0639	kinesin family member 13B	1.03	1.02	1.06	0.9
VCAN	VCAN;CSPG2;DKFZp686K06110;ERVR;GHAP;PG-M;WGN;WGN1	versican	1.03	1.05	1.06	0.8
HSPH1	HSPH1;DKFZp686M05240;HSP105;HSP105A;HSP105B;KIAA0201;NY-CO-25	heat shock 105kDa/110kDa protein 1	1.03	1.01	1.06	0.89
RNF128	RNF128;FLJ23516;GRAIL	ring finger protein 128	1.02	1.06	1.06	0.8
XKR6	XKR6;C8orf21;C8orf5;C8orf7;FLJ31557;XRG6	XK, Kell blood group complex subunit-related family, member 6	1.02	1.05	1.06	0.79
KRTAP6-3	KRTAP6-3;KAP6.3	keratin associated protein 6-3	1.02	1.05	1.06	0.79
MAN1A2	MAN1A2;MAN1B	mannosidase, alpha, class 1A, member 2	1.02	1.1	1.06	0.88
MRFAP1	MRFAP1;PAM14;PGR1	Morf4 family associated protein 1	1.02	1.03	1.06	0.83
ADAM9	ADAM9;CORD9;KIAA0021;MCMP;MDC9;Mltng	ADAM metallopeptidase domain 9	1.02	1.06	1.06	0.8
SHC4	SHC4;MGC34023;RaLP;SHCD	SHC (Src homology 2 domain containing) family, member 4	1.01	1.1	1.06	0.82
TMEM196	TMEM196;DKFZp686H19205;DKFZp686K0753;MGC42090	transmembrane protein 196	1.01	0.97	1.06	0.8
NDUFB2	NDUFB2;AGGG;CI-AGGG;MGC70788	NADH dehydrogenase (ubiquinone) 1 beta subcomplex, 2, 8kDa	1.01	1.12	1.06	0.75
MKNK1	MKNK1;MNK1	MAP kinase interacting serine/threonine kinase 1	1.01	1.07	1.06	0.89
ADCY2	ADCY2;AC2;FLJ16822;FLJ45092;HBAC2;KIAA1060;MGC133314	adenylate cyclase 2 (brain)	1	1.02	1.06	0.74
WFIKKN2	WFIKKN2;GASP-1;WFIKKNRP;hGASP-1	WAP, follistatin/kazal, immunoglobulin, kunitz and netrin domain containing 2	1	1.14	1.06	0.88
SH2B2	SH2B2;APS	SH2B adaptor protein 2	1	1.12	1.06	0.79
TCEAL8	TCEAL8;MGC45400	transcription elongation factor A (SII)-like 8	1	1.02	1.06	0.78
KRT82	KRT82;HB2;Hb-2;KRTHB2	keratin 82	0.99	0.94	1.06	0.73
NLGN3	NLGN3;HNL3;KIAA1480	neuroligin 3	0.99	1.12	1.06	0.84
LHFPL4	LHFPL4;MGC133162	lipoma HMGIC fusion partner-like 4	0.99	1.08	1.06	0.78
39142	MARCH7;AXO;AXOT;DKFZp586F1122;MARCH-VII;RNF177	membrane-associated ring finger (C3HC4) 7	0.99	1.02	1.06	0.92
DNAJC7	DNAJC7;DJ11;DJC7;TPR2;TTC2	DnaJ (Hsp40) homolog, subfamily C, member 7	0.99	0.98	1.06	0.83
PRR5-ARHGA	PRR5-ARHGAP8	PRR5-ARHGAP8 readthrough	0.99	1.16	1.06	0.85
SIRT4	SIRT4;MGC130046;MGC130047;MGC57437;SIR2L4	sirtuin 4	0.98	1.09	1.06	0.77
ZSCAN29	ZSCAN29;FLJ35867;MGC129894;MGC129895;ZNF690;Zfp690	zinc finger and SCAN domain containing 29	0.98	1.01	1.06	0.76
NELL2	NELL2;NRP2	NEL-like 2 (chicken)	0.98	0.93	1.06	0.78
SUPT16H	SUPT16H;CDC68;FACTP140;FLJ10857;FLJ14010;FLJ34357;SPT16/CDC68	suppressor of Ty 16 homolog (S. cerevisiae)	0.98	1.03	1.06	0.77
NTRK2	NTRK2;GP145-TrkB;TRKB;trk-B	neurotrophic tyrosine kinase, receptor, type 2	0.97	1.12	1.06	0.79
KRT27	KRT27;KRT25C	keratin 27	0.97	1.12	1.06	0.82
ZKSCAN4	ZKSCAN4;FLJ32136;P1P373C6;ZNF307;ZNF427;p373c6.1	zinc finger with KRAB and SCAN domains 4	0.97	1	1.06	0.75
ARHGAP10	ARHGAP10;FLJ20896;FLJ41791;GRAF2;PS-GAP;PSGAP	Rho GTPase activating protein 10	0.97	1.01	1.06	0.84
RPUSD1	RPUSD1;C16orf40;MGC19600;RLUCL	RNA pseudouridylate synthase domain containing 1	0.97	1.08	1.06	0.72
RNF170	RNF170;DKFZp564A022;FLJ38306	ring finger protein 170	0.97	1.01	1.06	0.91
TRNAU1AP	TRNAU1AP;FLJ20503;PRO1902;RP4-669K10.4;SECP43;TRSPAP1	tRNA selenocysteine 1 associated protein 1	0.97	1.1	1.06	0.85
FCRL5	FCRL5;BXMAS1;CD307;CD307e;DKFZp667E2019;DKFZp667F216;FCRH5;FLJ00333;FLJ00397;IRTA2;MGC119590;MGC119592;MGC119593;PRO820	Fc receptor-like 5	0.96	1.01	1.06	0.72
MON1A	MON1A;FLJ97088;MGC13272;SAND1	MON1 homolog A (yeast)	0.96	1.06	1.06	0.76
DEPDC5	DEPDC5;DEP.5;DKFZp779P2027	DEP domain containing 5	0.96	1	1.06	0.83
RHD	RHD;CD240D;DIIIc;MGC165007;RH;RH30;RHCED;RHDVA(TT);RHDel;RHPII;RHXIII;Rh4;RhDCw;RhII;RhK562-II;RhPI	Rh blood group, D antigen	0.95	1.07	1.06	0.7
CACNA2D4	CACNA2D4;RCD4	calcium channel, voltage-dependent, alpha 2/delta subunit 4	0.95	0.97	1.06	0.76
TTC7B	TTC7B;TTC7L1;c14_5685	tetratricopeptide repeat domain 7B	0.95	1.01	1.06	0.71
AP3S1	AP3S1;CLAPS3;Sigma3A	adaptor-related protein complex 3, sigma 1 subunit	0.95	1.04	1.06	0.79
CPEB3	CPEB3	cytoplasmic polyadenylation element binding protein 3	0.94	0.94	1.06	0.79
PAAF1	PAAF1;FLJ11848;PAAF;Rpn14;WDR71	proteasomal ATPase-associated factor 1	0.94	0.96	1.06	0.69
MTMR1	MTMR1	myotubularin related protein 1	0.94	1	1.06	0.78
OSBPL2	OSBPL2;FLJ20223;KIAA0772;MGC4307;MGC8342;ORP-2;ORP2	oxysterol binding protein-like 2	0.94	1.04	1.06	0.69
ITIH6	ITIH6;ITIH5L;UNQ6369;dJ14O9.1	inter-alpha-trypsin inhibitor heavy chain family, member 6	0.94	1.02	1.06	0.7
C9orf93	C9orf93;DKFZp686P12113;FLJ39267;FLJ46740;MGC50805;bA536D16.1;bA778P13.1	chromosome 9 open reading frame 93	0.93	0.96	1.06	0.76
TMEM182	TMEM182;DKFZp779G1758;DKFZp779K1556;DKFZp779M1870;FLJ30294	transmembrane protein 182	0.93	1.05	1.06	0.73
PIGG	PIGG;DKFZp434A1810;DKFZp434C1712;DKFZp434M1131;FLJ20265;FLJ39925;GPI7;LAS21;MGC131903;PRO4405;RLGS1930	phosphatidylinositol glycan anchor biosynthesis, class G	0.93	0.94	1.06	0.73
DNAJC13	DNAJC13;FLJ25863;KIAA0678;RME8	DnaJ (Hsp40) homolog, subfamily C, member 13	0.93	1.02	1.06	0.7
CHAT	CHAT;CHOACTASE;CMS1A;CMS1A2	choline O-acetyltransferase	0.92	0.94	1.06	0.68
PAPLN	PAPLN;DKFZp434F053;MGC50452	papilin, proteoglycan-like sulfated glycoprotein	0.92	0.94	1.06	0.68
PDLIM4	PDLIM4;RIL	PDZ and LIM domain 4	0.92	1.02	1.06	0.77
PSD4	PSD4;EFA6B;FLJ36237;FLJ37279;TIC	pleckstrin and Sec7 domain containing 4	0.92	1	1.06	0.75
TMEM67	TMEM67;JBTS6;MECKELIN;MGC26979;MKS3;NPHP11;TNEM67	transmembrane protein 67	0.92	1.06	1.06	0.78
ALDH4A1	ALDH4A1;ALDH4;DKFZp779M035;P5CD;P5CDh	aldehyde dehydrogenase 4 family, member A1	0.92	0.92	1.06	0.73
CNDP2	CNDP2;CN2;CPGL;FLJ10830;FLJ35544;HsT2298;PEPA	CNDP dipeptidase 2 (metallopeptidase M20 family)	0.92	0.97	1.06	0.73
EDIL3	EDIL3;DEL1;MGC26287	EGF-like repeats and discoidin I-like domains 3	0.91	0.99	1.06	0.66
VCX2	VCX2;MGC118977;MGC125729;MGC125794;MGC125795;VCX-2r;VCX2R;VCXB	variable charge, X-linked 2	0.91	0.99	1.06	0.68
HIVEP3	HIVEP3;FLJ16752;KBP-1;KBP1;KIAA1555;KRC;SHN3;Schnurri-3;ZAS3;ZNF40C	human immunodeficiency virus type I enhancer binding protein 3	0.9	1.05	1.06	0.73
PTPRB	PTPRB;DKFZp686E2262;DKFZp686H15164;FLJ44133;HPTP-BETA;HPTPB;MGC142023;MGC59935;PTPB;R-PTP-BETA;VEPTP	protein tyrosine phosphatase, receptor type, B	0.9	0.94	1.06	0.73
ZNF668	ZNF668;FLJ13479;FLJ14492	zinc finger protein 668	0.9	0.97	1.06	0.72
RAET1G	RAET1G;ULBP5	retinoic acid early transcript 1G	0.9	0.92	1.06	0.7
MRPL35	MRPL35;L35mt;MRP-L35	mitochondrial ribosomal protein L35	0.9	1	1.06	0.8
TMEM150C	TMEM150C;FLJ12993	transmembrane protein 150C	0.9	1.04	1.06	0.69
MTPN	MTPN;FLJ31098;FLJ99857;GCDP;V-1	myotrophin	0.9	0.92	1.06	0.72
KCNA5	KCNA5;ATFB7;HCK1;HK2;HPCN1;KV1.5;MGC117058;MGC117059;PCN1	potassium voltage-gated channel, shaker-related subfamily, member 5	0.89	1	1.06	0.77
LCE2B	LCE2B;LEP10;SPRL1B;XP5	late cornified envelope 2B	0.89	0.99	1.06	0.74
TAP2	TAP2;ABC18;ABCB3;APT2;D6S217E;PSF2;RING11	transporter 2, ATP-binding cassette, sub-family B (MDR/TAP)	0.89	0.9	1.06	0.71
ALG3	ALG3;CDG1D;CDGS4;D16Ertd36e;NOT56L;Not56	asparagine-linked glycosylation 3, alpha-1,3- mannosyltransferase homolog (S. cerevisiae)	0.89	0.95	1.06	0.64
TMEM207	TMEM207;UNQ846	transmembrane protein 207	0.88	0.88	1.06	0.74
FGL2	FGL2;T49;pT49	fibrinogen-like 2	0.88	1	1.06	0.75
IL2RG	IL2RG;CD132;CIDX;IL-2RG;IMD4;P64;SCIDX;SCIDX1	interleukin 2 receptor, gamma	0.88	0.92	1.06	0.67
CD207	CD207;CLEC4K	CD207 molecule, langerin	0.88	0.96	1.06	0.73
SP8	SP8;BTD	Sp8 transcription factor	0.88	0.9	1.06	0.69
TTC26	TTC26;DYF13;FLJ12571;MGC163211;dyf-13	tetratricopeptide repeat domain 26	0.88	0.91	1.06	0.69
ZMYM6	ZMYM6;Buster2;MYM;ZNF198L4;ZNF258	zinc finger, MYM-type 6	0.88	0.91	1.06	0.68
RAC2	RAC2;EN-7;Gx;HSPC022	ras-related C3 botulinum toxin substrate 2 (rho family, small GTP binding protein Rac2)	0.88	0.92	1.06	0.65
AGPAT3	AGPAT3;LPAAT-GAMMA1;MGC4604	1-acylglycerol-3-phosphate O-acyltransferase 3	0.88	0.87	1.06	0.69
FAM162A	FAM162A;C3orf28;E2IG5;HGTD-P	family with sequence similarity 162, member A	0.88	1.01	1.06	0.7
LMNB2	LMNB2;LAMB2;LMN2;MGC2721	lamin B2	0.88	0.98	1.06	0.72
HDLBP	HDLBP;FLJ16432;HBP;PRO2900;VGL	high density lipoprotein binding protein	0.88	1.01	1.06	0.76
EIF2AK3	EIF2AK3;DKFZp781H1925;PEK;PERK;WRS	eukaryotic translation initiation factor 2-alpha kinase 3	0.87	0.99	1.06	0.7
POU2AF1	POU2AF1;BOB1;OBF-1;OBF1;OCAB	POU class 2 associating factor 1	0.86	0.87	1.06	0.68
POLR3GL	POLR3GL;FLJ34890;MGC3200;RPC32HOM;flj32422	polymerase (RNA) III (DNA directed) polypeptide G (32kD)-like	0.86	0.99	1.06	0.73
SOX7	SOX7;MGC10895	SRY (sex determining region Y)-box 7	0.86	0.82	1.06	0.69
ALG5	ALG5;RP11-421P11.2;bA421P11.2	asparagine-linked glycosylation 5, dolichyl-phosphate beta-glucosyltransferase homolog (S. cerevisiae)	0.86	0.89	1.06	0.7
ZNF785	ZNF785;FLJ32130	zinc finger protein 785	0.86	0.98	1.06	0.76
TAOK2	TAOK2;KIAA0881;MAP3K17;PSK;PSK1;PSK1-BETA;TAO1;TAO2	TAO kinase 2	0.86	0.92	1.06	0.76
SENP1	SENP1;SuPr-2	SUMO1/sentrin specific peptidase 1	0.86	0.9	1.06	0.68
VPS29	VPS29;DC15;DKFZp564F0223;FLJ20492;PEP11	vacuolar protein sorting 29 homolog (S. cerevisiae)	0.86	0.89	1.06	0.66
MEAF6	MEAF6;C1orf149;CDABP0189;CENP-28;Eaf6;FLJ11730;NY-SAR-91	MYST/Esa1-associated factor 6	0.86	0.97	1.06	0.74

IFNA17	IFNA17;IFN-alphaI;IFNA;INFA;LEIF2C1	interferon, alpha 17	0.85	0.86	1.06	0.63
TCP10L	TCP10L;C21orf77;FLJ10932;MGC34297;TCP10A-2	t-complex 10 (mouse)-like	0.85	0.86	1.06	0.63
AGXT2L2	AGXT2L2;MGC117348;MGC15875;MGC45484	alanine-glyoxylate aminotransferase 2-like 2	0.85	0.86	1.06	0.67
ETNK1	ETNK1;EKI;EKI 1;EKI1;Nbla10396	ethanolamine kinase 1	0.85	0.9	1.06	0.74
TNFRSF10B	TNFRSF10B;CD262;DR5;KILLER;KILLER/DR5;TRAIL-R2;TRAILR2;TRICK2;TRICK2A;TRICK2B;TRICKB;ZTNFR9	tumor necrosis factor receptor superfamily, member 10b	0.85	0.9	1.06	0.61
EXOC2	EXOC2;FLJ11026;SEC5;SEC5L1;Sec5p	exocyst complex component 2	0.85	0.92	1.06	0.62
SFT2D2	SFT2D2;FLJ34085;UNQ512;dJ747L4.C1.2	SFT2 domain containing 2	0.85	0.98	1.06	0.65
CAMSAP1	CAMSAP1;DKFZp434F195;DKFZp434G2311;FLJ31228;MGC163452;PRO2405;bA100C15.1	calmodulin regulated spectrin-associated protein 1	1.41	1.51	1.07	1.02
MAP2K5	MAP2K5;HsT17454;MAPKK5;MEK5;PRKMK5	mitogen-activated protein kinase kinase 5	1.29	1.48	1.07	1.06
HOXA4	HOXA4;HOX1;HOX1D	homeobox A4	1.23	1.29	1.07	0.91
KLK9	KLK9;KLK-L3;KLKL3;MGC50513	kallikrein-related peptidase 9	1.23	1.21	1.07	0.91
STON1-GTF2A	STON1-GTF2A1L;MGC126821;MGC126823;SALF	STON1-GTF2A1L readthrough	1.21	1.35	1.07	1.02
SYTL4	SYTL4;DKFZp451P0116;FLJ40960;SLP4	synaptotagmin-like 4	1.18	1.25	1.07	1.12
SNX13	SNX13;KIAA0713;RGS-PX1	sorting nexin 13	1.18	1.23	1.07	0.99
KIAA1244	KIAA1244;A7322;ARFGEF3;BIG3;C6orf92;FLJ32064;PPP1R33;dJ171N11.1	KIAA1244	1.16	1.17	1.07	0.99
PTH2R	PTH2R;PTHR2	parathyroid hormone 2 receptor	1.15	1.14	1.07	0.86
ZNF438	ZNF438;FLJ32761;MGC126671;bA330O11.1	zinc finger protein 438	1.14	1.08	1.07	0.84
INPP4B	INPP4B;MGC132014	inositol polyphosphate-4-phosphatase, type II, 105kDa	1.14	1.19	1.07	0.94
ALDH1A2	ALDH1A2;MGC26444;RALDH(II);RALDH2;RALDH2-T	aldehyde dehydrogenase 1 family, member A2	1.12	1.21	1.07	0.94
CATSPER2	CATSPER2;MGC33346	cation channel, sperm associated 2	1.12	1.19	1.07	0.92
TPSG1	TPSG1;PRSS31;TMT;trpA	tryptase gamma 1	1.12	1.18	1.07	0.89
MIPEP	MIPEP;HMIP;MIP	mitochondrial intermediate peptidase	1.11	1.09	1.07	0.82
ANP32E	ANP32E;LANP-L;LANPL;MGC5350	acidic (leucine-rich) nuclear phosphoprotein 32 family, member E	1.1	1.15	1.07	0.9
PRICKLE1	PRICKLE1;EPM1B;FLJ31627;FLJ31937;MGC138902;MGC138903;RILP	prickle homolog 1 (Drosophila)	1.1	1.12	1.07	0.81
OR51I1	OR51I1	olfactory receptor, family 51, subfamily I, member 1	1.09	1.14	1.07	0.86
CUL5	CUL5;VACM-1;VACM1	cullin 5	1.09	1.08	1.07	0.81
RNF103	RNF103;HKF-1;KF-1;KF1;MGC102815;MGC41857;ZFP-103;ZFP103	ring finger protein 103	1.08	1.1	1.07	0.8
FBXO34	FBXO34;DKFZp547C162;FLJ20725;Fbx34;MGC126434;MGC126435	F-box protein 34	1.07	1.09	1.07	0.79
NT5DC2	NT5DC2;FLJ12442	5'-nucleotidase domain containing 2	1.07	1.2	1.07	0.86
DFNB59	DFNB59;PJVK	deafness, autosomal recessive 59	1.07	1.15	1.07	0.78
KIAA1274	KIAA1274;PALD	KIAA1274	1.06	1.12	1.07	0.86
KCTD1	KCTD1;C18orf5	potassium channel tetramerisation domain containing 1	1.06	1.19	1.07	0.83
SAA4	SAA4;C-SAA;CSAA	serum amyloid A4, constitutive	1.04	1.01	1.07	0.8
FAM26E	FAM26E;C6orf188;MGC45451;dJ493F7.3	family with sequence similarity 26, member E	1.04	1.16	1.07	0.83
TMPRSS9	TMPRSS9;FLJ16193	transmembrane protease, serine 9	1.04	1.03	1.07	0.77
C9orf7	C9orf7;D9S2135;FLOWER	chromosome 9 open reading frame 7	1.04	1.21	1.07	0.89
CCNH	CCNH;CAK;p34;p37	cyclin H	1.04	1.03	1.07	0.91
THNSL1	THNSL1;FLJ22002;TSH1	threonine synthase-like 1 (S. cerevisiae)	1.04	1.11	1.07	0.92
RNPEP	RNPEP;DKFZp547H084	arginyl aminopeptidase (aminopeptidase B)	1.04	1.02	1.07	0.77
OR3A1	OR3A1;OLFRA03;OR17-40;OR17-82;OR40	olfactory receptor, family 3, subfamily A, member 1	1.03	1	1.07	0.79
CXorf23	CXorf23	chromosome X open reading frame 23	1.03	1.14	1.07	0.76
FBXO43	FBXO43;EMI2;ERP1;FBX43	F-box protein 43	1.02	1.04	1.07	0.75
RAB37	RAB37;FLJ30284;FLJ32507	RAB37, member RAS oncogene family	1.02	1.06	1.07	0.83
LOH12CR1	LOH12CR1;LOH1CR12	loss of heterozygosity, 12, chromosomal region 1	1.02	1.03	1.07	0.87
CCNG2	CCNG2	cyclin G2	1.02	1.12	1.07	0.89
CUL9	CUL9;DKFZp686G1042;DKFZp686P2024;H7AP1;PARC;RP3-330M21.2	cullin 9	1.02	1.05	1.07	0.76
TRIM6-TRIM3	TRIM6-TRIM34;IFP1;RNF21;TRIM34	TRIM6-TRIM34 readthrough	1.01	1.03	1.07	0.75
NEIL2	NEIL2;FLJ31644;MGC2832;MGC4505;NEH2;NEI2	nei endonuclease VIII-like 2 (E. coli)	1.01	1.05	1.07	0.77
PPP3R1	PPP3R1;CALNB1;CNB;CNB1	protein phosphatase 3, regulatory subunit B, alpha	1.01	1	1.07	0.76
LPAR1	LPAR1;EDG2;GPR26;Gpcr26;LPA1;Mrec1.3;VZG1;edg-2;rec.1.3;vzg-1	lysophosphatidic acid receptor 1	1	1	1.07	0.75
YPEL2	YPEL2;DKFZp761C2021	yippee-like 2 (Drosophila)	1	1.03	1.07	0.79
ANKRD34A	ANKRD34A;ANKRD34;DKFZp761F202	ankyrin repeat domain 34A	1	1.12	1.07	0.83
GLI2	GLI2;HPE9;THP1;THP2	GLI family zinc finger 2	1	0.99	1.07	0.88
PBX3	PBX3	pre-B-cell leukemia homeobox 3	1	0.98	1.07	0.87
KIAA1539	KIAA1539;FLJ11560;P1.11659_5;RP11-182N22.6;bA182N22.6	KIAA1539	1	1.04	1.07	0.83
CLOCK	CLOCK;KAT13D;KIAA0334;bHLHe8	clock homolog (mouse)	1	1.04	1.07	0.77
E2F4	E2F4;E2F-4	E2F transcription factor 4, p107/p130-binding	1	1.06	1.07	0.79
FAM129B	FAM129B;C9orf88;DKFZP434H0820;FLJ13518;FLJ22151;FLJ22298;MEG-3;MINERVA;OC58;bA356B19.6	family with sequence similarity 129, member B	1	1.11	1.07	0.8
EXD2	EXD2;C14orf114;DKFZp781A0133;DKFZp781L15100;EXDL2;FLJ10738	exonuclease 3'-5' domain containing 2	1	0.94	1.07	0.74
PSTPIP1	PSTPIP1;CD2BP1;CD2BP1L;CD2BP1S;H-PIP;PAPAS;PSTPIP	proline-serine-threonine phosphatase interacting protein 1	0.99	1.06	1.07	0.72
ZNF821	ZNF821;FLJ16350	zinc finger protein 821	0.99	1.16	1.07	0.85
SBF1	SBF1;DKFZp761D0422;MGC99700;MTMR5	SET binding factor 1	0.99	0.98	1.07	0.74
CCNY	CCNY;C10orf9;CBCP1;CCNX;CFP1;FLJ95513	cyclin Y	0.99	0.94	1.07	0.74
AFF3	AFF3;LAF4;MLLT2-like	AF4/FMR2 family, member 3	0.98	1.16	1.07	0.8
HPS6	HPS6;FLJ22501;MGC20522;RP11-302K17.1	Hermansky-Pudlak syndrome 6	0.98	1.02	1.07	0.77
PCDHB2	PCDHB2;MGC111392;PCDH-BETA2	protocadherin beta 2	0.98	1.08	1.07	0.74
ATP13A3	ATP13A3;AFURS1;DKFZp686K16189;FLJ90613	ATPase type 13A3	0.98	1.02	1.07	0.75
ANXA3	ANXA3;ANX3	annexin A3	0.98	0.99	1.07	0.87
PNP	PNP;FLJ94043;FLJ97288;FLJ97312;MGC117396;MGC125915;MGC125916;NP;PRO1837;PUNP	purine nucleoside phosphorylase	0.98	1.02	1.07	0.72
ZNF703	ZNF703;FLJ14299;ZEPPO1;ZNF503L;ZPO1	zinc finger protein 703	0.97	1.01	1.07	0.76
LMO4	LMO4	LIM domain only 4	0.97	0.99	1.07	0.78
ZBTB33	ZBTB33;ZNF-kaiso;ZNF348	zinc finger and BTB domain containing 33	0.97	1	1.07	0.77
LRTM1	LRTM1	leucine-rich repeats and transmembrane domains 1	0.96	1.12	1.07	0.82
QPCT	QPCT;GCT;QC;sQC	glutaminyl-peptide cyclotransferase	0.96	1.08	1.07	0.72
DSE	DSE;DSEP;DSEPI;SART-2;SART2	dermatan sulfate epimerase	0.96	1.03	1.07	0.74
NPAS1	NPAS1;MOP5;PASD5;bHLHe11	neuronal PAS domain protein 1	0.96	1.01	1.07	0.78
IL17RC	IL17RC;FLJ95963;FLJ96005;IL17-RL;IL17RL;MGC10763	interleukin 17 receptor C	0.96	0.97	1.07	0.82
ZNF566	ZNF566;FLJ14779;MGC12515	zinc finger protein 566	0.96	1.03	1.07	0.72
ATXN1	ATXN1;ATX1;D6S504E;SCA1	ataxin 1	0.96	0.98	1.07	0.76
AMMECR1L	AMMECR1L;FLJ38552;MGC4268	AMME chromosomal region gene 1-like	0.96	1	1.07	0.75
GLT8D1	GLT8D1;DKFZp781O20198;FLJ14611;MSTP139	glycosyltransferase 8 domain containing 1	0.96	1.01	1.07	0.74
MAPKAPK2	MAPKAPK2;MK2	mitogen-activated protein kinase-activated protein kinase 2	0.96	1.11	1.07	0.79
AKNAD1	AKNAD1;C1orf62;MGC26989	AKNA domain containing 1	0.96	0.92	1.07	0.71
MCMBP	MCMBP;C10orf119;FLJ13081;FLJ36756;MCM-BP	minichromosome maintenance complex binding protein	0.96	1	1.07	0.84
SMN2	SMN2;BCD541;C-BCD541;FLJ76644;MGC20996;MGC5208;SMN1;SMNC	survival of motor neuron 2, centromeric	0.95	1.03	1.07	0.79
OR56A4	OR56A4;OR11-49	olfactory receptor, family 56, subfamily A, member 4	0.94	1.02	1.07	0.71
OLFML2B	OLFML2B;MGC51337;RP11-227F8.1	olfactomedin-like 2B	0.94	1.04	1.07	0.75
TMEM79	TMEM79;FLJ16057;FLJ32254;MGC13102	transmembrane protein 79	0.94	1.07	1.07	0.75
ELMO3	ELMO3;CED-12;CED12;ELMO-3;FLJ13824	engulfment and cell motility 3	0.94	1.05	1.07	0.71
TENC1	TENC1;C1-TEN;C1TEN;DKFZp686D13244;FLJ16320;KIAA1075;TNS2	tensin like C1 domain containing phosphatase (tensin 2)	0.94	1	1.07	0.83
SYT13	SYT13;KIAA1427	synaptotagmin XIII	0.94	1.04	1.07	0.7
EFNA4	EFNA4;EFL4;EPLG4;FLJ57652;LERK4;MGC125826	ephrin-A4	0.94	1	1.07	0.83
NEU3	NEU3;FLJ12388;SIAL3	sialidase 3 (membrane sialidase)	0.94	0.92	1.07	0.82
PUS3	PUS3;2610020J05Rik;FLJ23638	pseudouridylate synthase 3	0.94	0.98	1.07	0.69
LANCL1	LANCL1;GPR69A;p40	LanC lantibiotic synthetase component C-like 1 (bacterial)	0.94	0.98	1.07	0.88
CBX3	CBX3;HECH;HP1-GAMMA;HP1Hs-gamma	chromobox homolog 3	0.94	0.97	1.07	0.74
SEC16B	SEC16B;DKFZp686C2486;FLJ23871;FLJ25761;FLJ33652;FLJ36620;LZTR2;PGPR-p117;RGPR;SEC16S	SEC16 homolog B (S. cerevisiae)	0.93	0.99	1.07	0.71
CDS1	CDS1;CDS	CDP-diacylglycerol synthase (phosphatidate cytidylyltransferase) 1	0.93	0.93	1.07	0.74
CEACAM8	CEACAM8;CD66b;CD67;CGM6;NCA-95	carcinoembryonic antigen-related cell adhesion molecule 8	0.92	0.92	1.07	0.78
PRRT2	PRRT2;DKFZp547J199;FLJ25513;IFITMD1	proline-rich transmembrane protein 2	0.92	0.94	1.07	0.72
NAP1L5	NAP1L5;DRLM	nucleosome assembly protein 1-like 5	0.92	0.88	1.07	0.73
RDH5	RDH5;FLJ39337;FLJ97089;HSD17B9;RDH1;SDR9C5	retinol dehydrogenase 5 (11-cis/9-cis)	0.92	0.9	1.07	0.8
YPEL5	YPEL5	yippee-like 5 (Drosophila)	0.92	0.98	1.07	0.71
CDC40	CDC40;EHB3;FLJ10564;MGC102802;PRP17;PRPF17	cell division cycle 40 homolog (S. cerevisiae)	0.92	1.01	1.07	0.77
TEAD3	TEAD3;DTEF-1;ETFR-1;TEAD-3;TEAD5;TEF-5;TEF5	TEA domain family member 3	0.92	1	1.07	0.79
AGK	AGK;FLJ10842;MULK	acylglycerol kinase	0.92	0.98	1.07	0.81
ARGLU1	ARGLU1;DKFZp686O08106;FLJ10154	arginine and glutamate rich 1	0.92	0.99	1.07	0.67
CERS4	CERS4;FLJ12089;LASS4;Trh1	ceramide synthase 4	0.92	0.93	1.07	0.78
SYCP2L	SYCP2L;C6orf177;NO145;dJ62D2.1	synaptonemal complex protein 2-like	0.91	1.05	1.07	0.81
CTRL	CTRL;CTRL1;MGC70821	chymotrypsin-like	0.91	0.9	1.07	0.67
TWSG1	TWSG1;TSG	twisted gastrulation homolog 1 (Drosophila)	0.91	0.87	1.07	0.73
GBP6	GBP6;DKFZp686G0786;FLJ39135	guanylate binding protein family, member 6	0.9	0.99	1.07	0.74
ARHGAP30	ARHGAP30;FLJ00267;FLJ44128	Rho GTPase activating protein 30	0.9	0.94	1.07	0.72
ALPP	ALPP;ALP;FLJ61142;PALP;PLAP	alkaline phosphatase, placental	0.9	0.9	1.07	0.67
MCC	MCC;DKFZp762O1615;FLJ38893;FLJ46755;MCC1	mutated in colorectal cancers	0.9	0.92	1.07	0.7
OPN4	OPN4;MGC142118;MOP	opsin 4	0.9	0.9	1.07	0.67
GINS3	GINS3;FLJ13912;PSF3	GINS complex subunit 3 (Psf3 homolog)	0.9	1.04	1.07	0.7
VAMP5	VAMP5	vesicle-associated membrane protein 5 (myobrevin)	0.9	0.94	1.07	0.73
NFX1	NFX1;DKFZp779G2416;MGC20369;NFX2	nuclear transcription factor, X-box binding 1	0.9	0.92	1.07	0.8
TFDP1	TFDP1;DP1;DRTF1;Dp-1	transcription factor Dp-1	0.9	0.97	1.07	0.75
GLUD1	GLUD1;GDH;GDH1;GLUD;MGC132003	glutamate dehydrogenase 1	0.9	0.93	1.07	0.69
C1orf114	C1orf114;FLJ25846;RP1-206D15.2	chromosome 1 open reading frame 114	0.89	1.04	1.07	0.69
USF2	USF2;FIP;bHLHb12	upstream transcription factor 2, c-fos interacting	0.89	0.92	1.07	0.68
CCDC37	CCDC37;FLJ40083;MGC120558	coiled-coil domain containing 37	0.88	1	1.07	0.77
SLC27A2	SLC27A2;ACSVL1;FACVL1;FATP2;HsT17226;VLACS;VLCS;hFACVL1	solute carrier family 27 (fatty acid transporter), member 2	0.88	0.91	1.07	0.65
SCN8A	SCN8A;CERIII;CIAT;MED;NaCh6;Nav1.6;PN4	sodium channel, voltage gated, type VIII, alpha subunit	0.88	1	1.07	0.77
NFATC1	NFATC1;MGC138448;NF-ATC;NFAT2;NFATc	nuclear factor of activated T-cells, cytoplasmic, calcineurin-dependent 1	0.88	0.94	1.07	0.63
FGF8	FGF8;AIGF;FGF-8;HBGF-8;KAL6;MGC149376	fibroblast growth factor 8 (androgen-induced)	0.88	0.9	1.07	0.7
ZNF577	ZNF577;FLJ32291;MGC4400	zinc finger protein 577	0.88	0.98	1.07	0.66

HBZ	HBZ	hemoglobin, zeta	0.88	0.87	1.07	0.77
RWDD3	RWDD3;DKFZp566K023;FLJ51225;RSUME	RWD domain containing 3	0.88	0.99	1.07	0.8
MRPL40	MRPL40;FLJ41774;MGC9400;MRP-L22;NLVCF;URIM	mitochondrial ribosomal protein L40	0.88	0.91	1.07	0.7
B3GALTL	B3GALTL;B3GLCT;B3GTL;B3Glc-T;Gal-T;beta3Glc-T	beta 1,3-galactosyltransferase-like	0.88	0.93	1.07	0.67
ASB3	ASB3;ASB-3;FLJ10123;FLJ10421;MGC12531;MGC132002;MGC996	ankyrin repeat and SOCS box containing 3	0.88	0.9	1.07	0.65
C20orf72	C20orf72;FLJ14597;bA504H3.4	chromosome 20 open reading frame 72	0.88	0.92	1.07	0.69
FKBP8	FKBP8;FKBP38;FKBPr38	FK506 binding protein 8, 38kDa	0.88	0.86	1.07	0.68
GDA	GDA;CYPIN;GUANASE;KIAA1258;MGC9982;NEDASIN	guanine deaminase	0.88	0.92	1.07	0.65
CD46	CD46;AHUS2;MCP;MGC26544;MIC10;TLX;TRA2.10	CD46 molecule, complement regulatory protein	0.88	1	1.07	0.77
UCK2	UCK2;DKFZp686M04245;TSA903;UK;UMPK	uridine-cytidine kinase 2	0.88	0.94	1.07	0.78
RBMY1D	RBMY1D	RNA binding motif protein, Y-linked, family 1, member D	0.88	0.98	1.07	0.74
ADAMTS4	ADAMTS4;ADAMTS-2;ADAMTS-4;ADMP-1;KIAA0688	ADAM metallopeptidase with thrombospondin type 1 motif, 4	0.87	0.92	1.07	0.69
PQLC2	PQLC2;FLJ20320;FLJ23906;FLJ26661;FLJ26665	PQ loop repeat containing 2	0.87	0.84	1.07	0.7
PCMTD1	PCMTD1;FLJ10883	protein-L-isoaspartate (D-aspartate) O-methyltransferase domain containing 1	0.87	0.94	1.07	0.63
TRIM64B	TRIM64B	tripartite motif containing 64B	0.87	0.92	1.07	0.83
SLC35F1	SLC35F1;C6orf169;FLJ13018;dJ230I3.1	solute carrier family 35, member F1	0.86	0.94	1.07	0.63
PPP1R14D	PPP1R14D;CPI17-like;FLJ20251;GBPI-1;GBPI1;MGC119014;MGC119016	protein phosphatase 1, regulatory (inhibitor) subunit 14D	0.86	0.94	1.07	0.64
TIMP4	TIMP4	TIMP metallopeptidase inhibitor 4	0.86	0.95	1.07	0.63
UMODL1	UMODL1	uromodulin-like 1	0.86	0.86	1.07	0.7
SOX5	SOX5;L-SOX5;MGC35153	SRY (sex determining region Y)-box 5	0.86	0.96	1.07	0.72
RASA2	RASA2;GAP1M	RAS p21 protein activator 2	0.86	0.86	1.07	0.68
RINT1	RINT1;DKFZp667H2324;RINT-1	RAD50 interactor 1	0.86	1.02	1.07	0.67
BCCIP	BCCIP;TOK-1;TOK1	BRCA2 and CDKN1A interacting protein	0.86	0.94	1.07	0.74
SNRPN	SNRPN;DKFZp686C0927;DKFZp686M12165;DKFZp761I1912;DKFZp762N022;FLJ33569;FLJ36996;FLJ39265;HCERN3;MGC29886;PWCR;RT-LI;SM-D;SMN;SNRNP-N;SNURF-SNRPN;sm-N	small nuclear ribonucleoprotein polypeptide N	0.86	0.89	1.07	0.64
HIST1H2BH	HIST1H2BH;H2B/j;H2BFJ	histone cluster 1, H2bh	0.86	0.97	1.07	0.78
ATP1B1	ATP1B1;ATP1B;MGC1798	ATPase, Na+/K+ transporting, beta 1 polypeptide	0.86	0.89	1.07	0.66
ATP5C1	ATP5C1;ATP5C;ATP5CL1	ATP synthase, H+ transporting, mitochondrial F1 complex, gamma polypeptide 1	0.86	0.9	1.07	0.63
LOC340843	LOC340843	ENTREZ STATUS: REMAPPED TO GENE ID: 653567	0.86	0.86	1.07	0.7
PCDHGB5	PCDHGB5;PCDH-GAMMA-B5	protocadherin gamma subfamily B, 5	0.86	0.96	1.07	0.64
PRTG	PRTG;FLJ12795;FLJ13221;FLJ25756;IGDCC5	protogenin	0.85	0.96	1.07	0.64
ACOT2	ACOT2;CTE1A;MTE1;PTE2;PTE2A;ZAP128	acyl-CoA thioesterase 2	0.85	0.88	1.07	0.62
C12orf45	C12orf45;MGC40397	chromosome 12 open reading frame 45	0.85	1	1.07	0.73
MRPL46	MRPL46;C15orf4;LIECG2;MGC22762;P2ECSL	mitochondrial ribosomal protein L46	0.85	0.92	1.07	0.63
SMG7	SMG7;C1orf16;EST1C;FLJ23717;KIAA0250;SGA56M	smg-7 homolog, nonsense mediated mRNA decay factor (C. elegans)	0.85	0.94	1.07	0.68
ZNF587	ZNF587;FLJ14710;FLJ20813;FLJ52732;ZF6	zinc finger protein 587	0.85	0.86	1.07	0.73
NDFIP2	NDFIP2;FLJ25842;KIAA1165;N4WBP5A	Nedd4 family interacting protein 2	1.26	1.33	1.08	1.01
GJA10	GJA10;CX62;RP11-63K6.6	gap junction protein, alpha 10, 62kDa	1.25	1.47	1.08	1.07
SLC1A4	SLC1A4;ASCT1;SATT	solute carrier family 1 (glutamate/neutral amino acid transporter), member 4	1.21	1.26	1.08	0.91
TPI1	TPI1;MGC88108;TIM;TPI	triosephosphate isomerase 1	1.17	1.35	1.08	0.89
C12orf52	C12orf52;FLJ14827;RITA	chromosome 12 open reading frame 52	1.15	1.31	1.08	0.92
NEBL	NEBL;FLJ53769;LASP2;LNEBL;MGC119746;MGC119747	nebulette	1.14	1.2	1.08	0.9
NAP1L1	NAP1L1;FLJ16112;MGC23410;MGC8688;NAP1;NAP1L;NRP	nucleosome assembly protein 1-like 1	1.14	1.26	1.08	0.96
ADAM15	ADAM15;MDC15	ADAM metallopeptidase domain 15	1.12	1.12	1.08	0.84
ZCCHC13	ZCCHC13;CNBP2;ZNF9L	zinc finger, CCHC domain containing 13	1.11	1.07	1.08	0.83
RXFP4	RXFP4;GPCR142;GPR100;MGC126556;MGC126558;RLN3R2;RXFPR4	relaxin/insulin-like family peptide receptor 4	1.11	1.11	1.08	0.84
EDEM1	EDEM1;EDEM;FLJ51559;FLJ51560;KIAA0212	ER degradation enhancer, mannosidase alpha-like 1	1.1	1.13	1.08	0.81
VIL1	VIL1;D2S1471;VIL	villin 1	1.09	1.14	1.08	0.9
GPR37	GPR37;EDNRBL;PAELR;hET(B)R-LP	G protein-coupled receptor 37 (endothelin receptor type B-like)	1.07	1.07	1.08	0.81
NAT9	NAT9;DKFZp564C103;EBSP, hNATL	N-acetyltransferase 9 (GCN5-related, putative)	1.06	1.08	1.08	0.91
NCOA5	NCOA5;CIA;bA465L10.6	nuclear receptor coactivator 5	1.05	1.25	1.08	0.91
KCNJ3	KCNJ3;GIRK1;KGA;KIR3.1	potassium inwardly-rectifying channel, subfamily J, member 3	1.04	1.12	1.08	0.87
TMEM136	TMEM136;MGC17839	transmembrane protein 136	1.04	1.08	1.08	0.83
C20orf108	C20orf108;5A3;DKFZp434A1114;dJ1167H4.1	chromosome 20 open reading frame 108	1.04	1.15	1.08	0.88
SMURF2	SMURF2;DKFZp686F0270;MGC138150	SMAD specific E3 ubiquitin protein ligase 2	1.04	1.04	1.08	0.78
KDELR1	KDELR1;ERD2;ERD2.1;HDEL;PM23	KDEL (Lys-Asp-Glu-Leu) endoplasmic reticulum protein retention receptor 1	1.04	1.14	1.08	0.9
PADI1	PADI1;HPAD10;PAD1;PDI;PDI1	peptidyl arginine deiminase, type I	1.03	1.06	1.08	0.77
DUSP10	DUSP10;MKP-5;MKP5	dual specificity phosphatase 10	1.03	1.04	1.08	0.78
SYVN1	SYVN1;HRD1;KIAA1810;MGC40372	synovial apoptosis inhibitor 1, synoviolin	1.03	1.1	1.08	0.99
CREB5	CREB5;CRE-BPA	cAMP responsive element binding protein 5	1.02	1.12	1.08	0.75
MFHAS1	MFHAS1;FLJ23354;LRRC65;MASL1	malignant fibrous histiocytoma amplified sequence 1	1.02	1.07	1.08	0.75
IGF2BP2	IGF2BP2;IMP-2;IMP2;VICKZ2;p62	insulin-like growth factor 2 mRNA binding protein 2	1.02	1.19	1.08	0.92
UBASH3A	UBASH3A;CLIP4;STS-2;TULA	ubiquitin associated and SH3 domain containing A	1.01	1.01	1.08	0.89
VPS39	VPS39;FLJ21681;FLJ46546;KIAA0770;TLP;VAM6;hVam6p	vacuolar protein sorting 39 homolog (S. cerevisiae)	1.01	1.12	1.08	0.85
FAM155B	FAM155B;CXorf63;TED;TMEM28;bB57D9.1	family with sequence similarity 155, member B	1	1.12	1.08	0.77
C18orf25	C18orf25;ARKL1;MGC12909;MGC87799	chromosome 18 open reading frame 25	1	1.15	1.08	0.81
VPS25	VPS25;DERP9;EAP20;FAP20;MGC10540	vacuolar protein sorting 25 homolog (S. cerevisiae)	1	1.04	1.08	0.8
FYTTD1	FYTTD1;DKFZp761B1514;UIF	forty-two-three domain containing 1	1	1.17	1.08	0.78
USP9X	USP9X;DFFRX;FAF;FAM	ubiquitin specific peptidase 9, X-linked	1	1.07	1.08	0.8
HSP90AA1	HSP90AA1;FLJ31884;HSP86;HSP89A;HSP90A;HSP90N;HSPC1;HSPCA;HSPCAL1;HSPCAL4;HSPN;Hsp89;Hsp90;LAP2	heat shock protein 90kDa alpha (cytosolic), class A member 1	1	1.07	1.08	0.79
SMAD9	SMAD9;MADH6;MADH9;SMAD8;SMAD8A;SMAD8B	SMAD family member 9	0.99	1	1.08	0.79
STK33	STK33	serine/threonine kinase 33	0.99	1.06	1.08	0.88
CYP4F3	CYP4F3;CPF3;CYP4F;LTB4H	cytochrome P450, family 4, subfamily F, polypeptide 3	0.98	1.12	1.08	0.74
JAKMIP1	JAKMIP1;FLJ31564;Gababrbp;JAMIP1;MARLIN1	janus kinase and microtubule interacting protein 1	0.98	1.15	1.08	0.77
CHRDL2	CHRDL2;BNF1;CHL2;DKFZp586N2124;FKSG37	chordin-like 2	0.98	1.04	1.08	0.78
ANKRD53	ANKRD53;FLJ12056;FLJ36160	ankyrin repeat domain 53	0.98	1.15	1.08	0.77
AHSA2	AHSA2;DKFZp564C236;FLJ34679;FLJ41715;Hch1	AHA1, activator of heat shock 90kDa protein ATPase homolog 2 (yeast)	0.98	1.09	1.08	0.82
OR2A4	OR2A4;OR2A10	olfactory receptor, family 2, subfamily A, member 4	0.98	1.09	1.08	0.82
OR4F4	OR4F4;OLA-7501;OR4F18	olfactory receptor, family 4, subfamily F, member 4	0.98	1.01	1.08	0.78
FAM180A	FAM180A;UNQ1940	family with sequence similarity 180, member A	0.97	1.01	1.08	0.8
USP15	USP15;FLJ55073;KIAA0529;MGC131982;MGC149838;MGC74854;UNPH-2;UNPH4	ubiquitin specific peptidase 15	0.97	0.97	1.08	0.72
UBE2Q1	UBE2Q1;GTAP;NICE-5;UBE2Q	ubiquitin-conjugating enzyme E2Q family member 1	0.97	0.96	1.08	0.72
NT5C	NT5C;DNT;DNT1;P5N2;PN-I;PN-II;UMPH2;cdN;dNT-1	5', 3'-nucleotidase, cytosolic	0.96	0.96	1.08	0.72
CHD6	CHD6;CHD5;KIAA1335;RIGB	chromodomain helicase DNA binding protein 6	0.96	1.01	1.08	0.71
MPHOSPH9	MPHOSPH9;DKFZp434J034;FLJ12954;MPP-9;MPP9	M-phase phosphoprotein 9	0.96	0.93	1.08	0.74
HMG20B	HMG20B;BRAF25;BRAF35;FLJ26127;HMGX2;HMGXB2;PP7706;SMARCE1r;SOXL;pp8857	high mobility group 20B	0.95	1.01	1.08	0.78
WBP2	WBP2;MGC18269;WBP-2	WW domain binding protein 2	0.95	0.92	1.08	0.77
SERPINB6	SERPINB6;CAP;DFNB91;DKFZp686I04222;MGC111370;MSTP057;PI6;PTI;SPI3	serpin peptidase inhibitor, clade B (ovalbumin), member 6	0.95	1.08	1.08	0.72
MSMO1	MSMO1;DESP4;ERG25;MGC104344;SC4MOL	methylsterol monooxygenase 1	0.95	0.98	1.08	0.77
PCDHGA12	PCDHGA12;CDH21;FIB3;KIAA0588;PCDH-GAMMA-A12	protocadherin gamma subfamily A, 12	0.95	0.98	1.08	0.71
FABP7	FABP7;B-FABP;BLBP;DKFZp547J2313;FABPB;MRG	fatty acid binding protein 7, brain	0.94	1.02	1.08	0.79
SERINC4	SERINC4;FLJ40363	serine incorporator 4	0.94	0.96	1.08	0.74
HPX	HPX;FLJ56652;HX	hemopexin	0.94	0.98	1.08	0.74
MT1B	MT1B;MT1;MT1Q;MTP	metallothionein 1B	0.94	0.92	1.08	0.73
EXTL1	EXTL1;EXTL;MGC70794	exostoses (multiple)-like 1	0.94	0.98	1.08	0.84
KCNF1	KCNF1;IK8;KCNF;KV5.1;MGC33316;kH1	potassium voltage-gated channel, subfamily F, member 1	0.94	1.06	1.08	0.82
MFSD7	MFSD7;FLJ22269	major facilitator superfamily domain containing 7	0.94	1.09	1.08	0.76
HCFC1R1	HCFC1R1;FLJ20568;HPIP;MGC70711;MGC99622	host cell factor C1 regulator 1 (XPO1 dependent)	0.94	1	1.08	0.78
CLEC16A	CLEC16A;Gop-1;KIAA0350;MGC111457	C-type lectin domain family 16, member A	0.94	0.92	1.08	0.76
GBA2	GBA2;DKFZp762K054;KIAA1605;MGC16895	glucosidase, beta (bile acid) 2	0.94	1.08	1.08	0.71
SPAST	SPAST;ADPSP;FSP2;KIAA1083;SPG4	spastin	0.94	1	1.08	0.75
PDAP1	PDAP1;HASPP28;PAP;PAP1	PDGFA associated protein 1	0.94	0.98	1.08	0.8
C22orf28	C22orf28;DJ149A16.6;RP1-149A16.6	chromosome 22 open reading frame 28	0.94	1.06	1.08	0.76
MINOS1	MINOS1;C1orf151;FLJ36999;FLJ75158;MIO10	mitochondrial inner membrane organizing system 1	0.94	0.96	1.08	0.81
EIF1AD	EIF1AD;MGC11102;haponin	eukaryotic translation initiation factor 1A domain containing	0.93	1.03	1.08	0.78
C10orf90	C10orf90;FATS;FLJ32938;bA422P15.2	chromosome 10 open reading frame 90	0.92	1.04	1.08	0.78
PDZD3	PDZD3;FLJ22756;IKEPP;NHERF4;PDZK2	PDZ domain containing 3	0.92	0.94	1.08	0.77
ACTL6B	ACTL6B;ACTL6;BAF53B	actin-like 6B	0.92	0.94	1.08	0.76
SFI1	SFI1;MGC131712;MGC150663;MGC156283;MGC57874;PISD;hSfi1p	Sfi1 homolog, spindle assembly associated (yeast)	0.92	0.89	1.08	0.74
CCNI	CCNI;CYC1;CYI	cyclin I	0.92	0.92	1.08	0.81
PGD	PGD;6PGD	phosphogluconate dehydrogenase	0.92	0.92	1.08	0.74
PRR20A	PRR20A;FLJ40296;MGC149404;MGC149405;PRR20	proline rich 20A	0.92	1.04	1.08	0.69
CRYBG3	CRYBG3;DKFZp667G2110;DKFZp686L13211;FLJ60082	beta-gamma crystallin domain containing 3	0.92	0.97	1.08	0.8
SZT2	SZT2;C1orf84;FLJ00408;FLJ10387;FLJ23425;FLJ34502;KIAA0467;RP11-506B15.3;SZT2A;SZT2B	seizure threshold 2 homolog (mouse)	0.92	1.02	1.08	0.71
LTB4R2	LTB4R2;BLT2;BLTR2;JULF2;KPG_004;LTB4-R 2;LTB4-R2;NOP9	leukotriene B4 receptor 2	0.91	0.97	1.08	0.86
DUSP27	DUSP27	dual specificity phosphatase 27 (putative)	0.9	0.96	1.08	0.69
TRAF2	TRAF2;MGC:45012;TRAP;TRAP3	TNF receptor-associated factor 2	0.9	0.99	1.08	0.75
GALNT7	GALNT7;GALNAC-T7;GalNAcT7	UDP-N-acetyl-alpha-D-galactosamine:polypeptide N-acetylgalactosaminyltransferase 7 (GalNAc-T7)	0.9	0.9	1.08	0.79
NDUFB11	NDUFB11;ESSS;FLJ20494;MGC111182;NP17.3;Np15;P17.3	NADH dehydrogenase (ubiquinone) 1 beta subcomplex, 11, 17.3kDa	0.9	1	1.08	0.72
NETO2	NETO2;BTCL2;FLJ10430;FLJ14724;FLJ90456;NEOT2	neuropilin (NRP) and tolloid (TLL)-like 2	0.9	0.94	1.08	0.67
SDCBP	SDCBP;MDA-9;ST1;SYCL;TACIP18	syndecan binding protein (syntenin)	0.9	0.98	1.08	0.66
GNG12	GNG12;FLJ31352;FLJ34695	guanine nucleotide binding protein (G protein), gamma 12	0.9	1.02	1.08	0.76
FAM13A	FAM13A;ARHGAP48;FAM13A1;FLJ34562;MGC105131	family with sequence similarity 13, member A	0.9	1.05	1.08	0.78
DAXX	DAXX;BING2;DAP6;EAP1;MGC126245;MGC126246	death-domain associated protein	0.89	0.95	1.08	0.7
TMEM87A	TMEM87A;DKFZp564G2022	transmembrane protein 87A	0.89	0.92	1.08	0.7
LOC392395	LOC392395	ENTREZ STATUS: REMAPPED TO GENE ID: 256158	0.89	0.93	1.08	0.75
LPL	LPL;HDLCQ11;LIPD	lipoprotein lipase	0.88	0.92	1.08	0.64
APOC3	APOC3;APOCIII;HALP2;MGC150353	apolipoprotein C-III	0.88	0.92	1.08	0.79
MCHR1	MCHR1;GPR24;MCH1R;MGC32129;SLC1	melanin-concentrating hormone receptor 1	0.88	0.88	1.08	0.66
GRID1	GRID1;KIAA1220	glutamate receptor, ionotropic, delta 1	0.88	0.98	1.08	0.76

FBXO8	FBXO8;FBS;FBX8	F-box protein 8	0.88	0.91	1.08	0.65
IKZF5	IKZF5;DKFZp781B0249;FLJ22973;PEGASUS;ZNFN1A5	IKAROS family zinc finger 5 (Pegasus)	0.88	0.85	1.08	0.66
ANP32C	ANP32C;PP32R1	acidic (leucine-rich) nuclear phosphoprotein 32 family, member C	0.88	0.93	1.08	0.7
MIER1	MIER1;DKFZp781G0451;ER1;KIAA1610;MGC131940;MGC150640;MGC150641;MI-ER1;hMI-ER1	mesoderm induction early response 1 homolog (Xenopus laevis)	0.88	1.02	1.08	0.78
C7orf42	C7orf42;FLJ10099;FLJ13090	chromosome 7 open reading frame 42	0.88	1.04	1.08	0.7
PAK2	PAK2;PAK65;PAKgamma	p21 protein (Cdc42/Rac)-activated kinase 2	0.88	1.02	1.08	0.72
SUCLG2	SUCLG2;GBETA	succinate-CoA ligase, GDP-forming, beta subunit	0.88	0.97	1.08	0.76
DNAJB11	DNAJB11;ABBP-2;ABBP2;DJ9;Dj-9;EDJ;ERdj3;ERj3;ERj3p;PRO1080;UNQ537;hDj-9	DnaJ (Hsp40) homolog, subfamily B, member 11	0.88	0.94	1.08	0.78
TRIM64C	TRIM64C	tripartite motif containing 64C	0.88	0.93	1.08	0.84
SELP	SELP;CD62;CD62P;FLJ45155;GMP140;GRMP;LECAM3;PADGEM;PSEL	selectin P (granule membrane protein 140kDa, antigen CD62)	0.87	0.96	1.08	0.73
LRP11	LRP11;FLJ14735;MANSC3;MGC39092;bA350J20.3	low density lipoprotein receptor-related protein 11	0.87	0.9	1.08	0.71
MPZ	MPZ;CHM;CMT1;CMT1B;CMT2I;CMT2J;CMT4E;CMTDI3;DSS;HMSNIB;MPP;P0	myelin protein zero	0.86	0.97	1.08	0.72
CLDN5	CLDN5;AWAL;BEC1;CPETRL1;TMVCF	claudin 5	0.86	0.9	1.08	0.71
SQRDL	SQRDL	sulfide quinone reductase-like (yeast)	0.86	0.93	1.08	0.72
SCOC	SCOC;SCOCO	short coiled-coil protein	0.86	0.96	1.08	0.72
CHEK2	CHEK2;CDS1;CHK2;HuCds1;LFS2;PP1425;RAD53	CHK2 checkpoint homolog (S. pombe)	0.86	1	1.08	0.71
RIPK1	RIPK1;FLJ39204;RIP;RIP1	receptor (TNFRSF)-interacting serine-threonine kinase 1	0.86	0.92	1.08	0.76
RBM23	RBM23;CAPERbeta;FLJ10482;MGC4458;RNPC4	RNA binding motif protein 23	0.86	0.94	1.08	0.72
PARL	PARL;PSARL;PSARL1;PSENIP2;RHBDS1	presenilin associated, rhomboid-like	0.86	1.01	1.08	0.67
RSC1A1	RSC1A1;RS1	regulatory solute carrier protein, family 1, member 1	0.86	0.94	1.08	0.75
TRIM77P	TRIM77P;TRIM77	tripartite motif containing 77, pseudogene	0.86	0.91	1.08	0.82
ROPN1L	ROPN1L;ASP;FLJ23003;FLJ25776;RSPH11	rhophilin associated tail protein 1-like	0.85	0.88	1.08	0.69
CCDC150	CCDC150;DKFZp434P055;FLJ10202;FLJ38811;FLJ39660;MGC72171	coiled-coil domain containing 150	0.85	0.82	1.08	0.69
GJD4	GJD4;CX40.1;FLJ90023;RP11-425A6.2	gap junction protein, delta 4, 40.1kDa	0.85	0.9	1.08	0.68
ENTPD8	ENTPD8;GLSR2492;NTPDase-8;UNQ2492	ectonucleoside triphosphate diphosphohydrolase 8	0.85	1.02	1.08	0.7
FAM125A	FAM125A;CFBP;FLJ32495	family with sequence similarity 125, member A	0.85	0.98	1.08	0.76
ATP6V1E1	ATP6V1E1;ATP6E;ATP6E2;ATP6V1E;P31;Vma4	ATPase, H+ transporting, lysosomal 31kDa, V1 subunit E1	0.85	0.88	1.08	0.63
SDHB	SDHB;FLJ92337;IP;PGL4;SDH;SDH1;SDH2;SDHIP	succinate dehydrogenase complex, subunit B, iron sulfur (Ip)	0.85	0.9	1.08	0.7
UGT1A8	UGT1A8;UDPGT;UDPGT 1-8;UGT-1H;UGT1-08;UGT1.8;UGT1A8S;UGT1H	UDP glucuronosyltransferase 1 family, polypeptide A8	0.85	0.89	1.08	0.66
SH2B3	SH2B3;IDDM20;LNK	SH2B adaptor protein 3	1.33	1.51	1.09	1.08
MAGED2	MAGED2;11B6;BCG1;HCA10;JCL-1;MAGE-D2;MAGED;MGC8386	melanoma antigen family D, 2	1.28	1.38	1.09	1.15
ZNF146	ZNF146;MGC125660;MGC125661;OZF	zinc finger protein 146	1.27	1.32	1.09	1.01
IGSF5	IGSF5;GSF5;JAM4	immunoglobulin superfamily, member 5	1.22	1.21	1.09	0.96
MC2R	MC2R;ACTHR;MGC125798	melanocortin 2 receptor (adrenocorticotropic hormone)	1.21	1.29	1.09	1.16
KIN	KIN;BTCD;KIN17	KIN, antigenic determinant of recA protein homolog (mouse)	1.21	1.19	1.09	0.95
SMARCD2	SMARCD2;BAF60B;CRACD2;Rsc6p	SWI/SNF related, matrix associated, actin dependent regulator of chromatin, subfamily d, member 2	1.19	1.32	1.09	1.05
FZD4	FZD4;CD344;EVR1;FEVR;FZD4S;Fz-4;FzE4;GPCR;MGC34390	frizzled family receptor 4	1.16	1.24	1.09	1.12
ZNF749	ZNF749;FLJ16263;FLJ16360	zinc finger protein 749	1.16	1.22	1.09	0.93
ZNF423	ZNF423;Ebfaz;KIAA0760;MGC138520;MGC138522;OAZ;Roaz;ZFP423;Zfp104	zinc finger protein 423	1.15	1.17	1.09	1.03
NPHP4	NPHP4;FLJ46306;KIAA0673;POC10;SLSN4	nephronophthisis 4	1.14	1.23	1.09	1.02
SLC6A19	SLC6A19;B0AT1;FLJ20680;FLJ34635;HND	solute carrier family 6 (neutral amino acid transporter), member 19	1.12	1.31	1.09	0.86
MUL1	MUL1;C1orf166;FLJ12875;GIDE;MAPL;MULAN;RNF218;RP11-401M16.2	mitochondrial E3 ubiquitin protein ligase 1	1.12	1.21	1.09	1.09
TSSK1B	TSSK1B;SPOGA4;STK22D;TSK1;TSSK1	testis-specific serine kinase 1B	1.11	1.3	1.09	0.93
TPSD1	TPSD1;MCP7-LIKE;MCP7L1;MGC95428;MMCP-7L	tryptase delta 1	1.1	1.19	1.09	0.89
WTIP	WTIP	Wilms tumor 1 interacting protein	1.1	1.29	1.09	0.99
FBXO18	FBXO18;FBH1;FLJ14590;Fbx18;MGC131916;MGC141935;MGC141937	F-box protein, helicase, 18	1.1	1.15	1.09	0.83
EEF1A2	EEF1A2;EEF1AL;EF-1-alpha-2;EF1A;FLJ41696;HS1;STN;STNL	eukaryotic translation elongation factor 1 alpha 2	1.09	1.21	1.09	0.94
TRIM52	TRIM52;MGC16175;RNF102	tripartite motif containing 52	1.08	1.12	1.09	0.81
PHF17	PHF17;FLJ22479;JADE1;KIAA1807	PHD finger protein 17	1.08	1.12	1.09	0.81
PHF13	PHF13;MGC43399;PHF5;SPOC1	PHD finger protein 13	1.07	1.12	1.09	0.81
KIAA1432	KIAA1432;CIP150;FLJ12580;bA207C16.1	KIAA1432	1.07	1.18	1.09	0.8
F2RL1	F2RL1;GPR11;PAR2	coagulation factor II (thrombin) receptor-like 1	1.07	1.13	1.09	1.02
OCLM	OCLM;TISR	oculomedin	1.06	1.19	1.09	0.91
PTPRN2	PTPRN2;IA-2beta;IAR;ICAAR;PTPRP;R-PTP-N2	protein tyrosine phosphatase, receptor type, N polypeptide 2	1.06	1.15	1.09	0.9
PRSS16	PRSS16;FLJ36271;FLJ40714;FLJ44172;TSSP	protease, serine, 16 (thymus)	1.06	1.06	1.09	0.79
TBCC	TBCC;CFC	tubulin folding cofactor C	1.06	1.12	1.09	0.88
COPS7B	COPS7B;CSN7B;FLJ12612;MGC111077	COP9 constitutive photomorphogenic homolog subunit 7B (Arabidopsis)	1.06	1.21	1.09	0.91
SNX25	SNX25;FLJ23161;SBBI31	sorting nexin 25	1.06	1.1	1.09	0.93
SUCLG1	SUCLG1;FLJ21114;FLJ43513;GALPHA;MTDPS9;SUCLA1	succinate-CoA ligase, alpha subunit	1.06	1.12	1.09	0.8
CBLC	CBLC;CBL-3;CBL-SL;RNF57	Cas-Br-M (murine) ecotropic retroviral transforming sequence c	1.05	1.1	1.09	0.79
FCAMR	FCAMR;CD351;FCA/MR	Fc receptor, IgA, IgM, high affinity	1.04	1.18	1.09	0.85
ADCYAP1	ADCYAP1;MGC126852;PACAP	adenylate cyclase activating polypeptide 1 (pituitary)	1.04	1.02	1.09	0.82
TAF12	TAF12;TAF2J;TAFII20	TAF12 RNA polymerase II, TATA box binding protein (TBP)-associated factor, 20kDa	1.04	1.2	1.09	0.8
THUMPD3	THUMPD3;DKFZp434F091	THUMP domain containing 3	1.04	1.01	1.09	0.84
METTL17	METTL17;FLJ20859;METT11D1	methyltransferase like 17	1.04	1.01	1.09	0.79
NKX3-2	NKX3-2;BAPX1;MGC138171;NKX3.2;NKX3B;SMMD	NK3 homeobox 2	1.03	1.1	1.09	0.78
CDKN2AIPNL	CDKN2AIPNL;MGC13017	CDKN2A interacting protein N-terminal like	1.03	1.17	1.09	0.88
LOC649055	LOC649055	ENTREZ STATUS: REMAPPED TO GENE ID: 342931	1.03	1.07	1.09	0.77
CELA3A	CELA3A;ELA3;ELA3A	chymotrypsin-like elastase family, member 3A	1.02	1.1	1.09	0.83
TRIM75	TRIM75	tripartite motif containing 75	1.01	1.05	1.09	0.76
GPR75	GPR75;GPRchr2;MGC132002;WI31133	G protein-coupled receptor 75	1.01	1.02	1.09	0.77
CDCP1	CDCP1;CD318;SIMA135;TRASK	CUB domain containing protein 1	1.01	1.2	1.09	0.88
TRIM55	TRIM55;MURF-2;RNF29;muRF2	tripartite motif containing 55	1	1.04	1.09	0.75
KRTAP4-5	KRTAP4-5;KAP4.5;KRTAP4.5	keratin associated protein 4-5	1	1.05	1.09	0.76
SH3GL2	SH3GL2;CNSA2;EEN-B1;FLJ20276;FLJ25015;SH3D2A;SH3P4	SH3-domain GRB2-like 2	1	1.08	1.09	0.8
PRRG1	PRRG1;PRGP1	proline rich Gla (G-carboxyglutamic acid) 1	1	1.12	1.09	0.84
PCMT1	PCMT1;PIMT	protein-L-isoaspartate (D-aspartate) O-methyltransferase	1	1.02	1.09	0.81
KDM5D	KDM5D;HY;HYA;JARID1D;KIAA0234;SMCY	lysine (K)-specific demethylase 5D	1	1.06	1.09	0.96
DNMT3L	DNMT3L;MGC1090	DNA (cytosine-5-)-methyltransferase 3-like	0.99	1.05	1.09	0.74
ILDR1	ILDR1;DFNB42;ILDR1alpha;ILDR1alpha';ILDR1beta;MGC50831	immunoglobulin-like domain containing receptor 1	0.99	1.1	1.09	0.84
C7orf62	C7orf62;MGC26647	chromosome 7 open reading frame 62	0.98	1.12	1.09	0.85
ZCCHC8	ZCCHC8;DKFZp434E2220	zinc finger, CCHC domain containing 8	0.98	0.96	1.09	0.77
C2orf56	C2orf56;MidA	chromosome 2 open reading frame 56	0.98	1.07	1.09	0.73
EIF2AK4	EIF2AK4;GCN2;KIAA1338	eukaryotic translation initiation factor 2 alpha kinase 4	0.98	1.15	1.09	0.82
CTNND1	CTNND1;CAS;CTNND;KIAA0384;P120CAS;P120CTN;p120;p120(CAS);p120(CTN)	catenin (cadherin-associated protein), delta 1	0.98	1.08	1.09	0.73
SS18L1	SS18L1;CREST;KIAA0693;LP2261;MGC26711;MGC78386	synovial sarcoma translocation gene on chromosome 18-like 1	0.97	1.01	1.09	0.77
OR10H2	OR10H2;MGC138383	olfactory receptor, family 10, subfamily H, member 2	0.96	1.15	1.09	0.84
IGFL4	IGFL4	IGF-like family member 4	0.96	0.94	1.09	0.74
DRD3	DRD3;D3DR;ETM1;FET1;MGC149204;MGC149205	dopamine receptor D3	0.96	1	1.09	0.9
UGT3A2	UGT3A2;MGC119426;MGC119429	UDP glycosyltransferase 3 family, polypeptide A2	0.96	1.06	1.09	0.84
JPH4	JPH4;FLJ34253;JP4;JPHL1;KIAA1831	junctophilin 4	0.96	1.07	1.09	0.82
STK19	STK19;D6S60;D6S60E;G11;HLA-RP1;MGC117388;RP1	serine/threonine kinase 19	0.96	1.02	1.09	0.92
SEZ6L2	SEZ6L2;FLJ90517;PSK-1	seizure related 6 homolog (mouse)-like 2	0.96	1.15	1.09	0.79
GTPBP8	GTPBP8;HSPC135	GTP-binding protein 8 (putative)	0.96	1.02	1.09	0.77
MAP2K2	MAP2K2;FLJ26075;MAPKK2;MEK2;MKK2;PRKMK2	mitogen-activated protein kinase kinase 2	0.96	1.01	1.09	0.91
USP31	USP31;KIAA1203	ubiquitin specific peptidase 31	0.96	0.97	1.09	0.72
SUMF2	SUMF2;DKFZp566I1024;DKFZp686I1024;DKFZp686L17160;DKFZp781L1035;MGC99485;pFGE	sulfatase modifying factor 2	0.96	1.09	1.09	0.73
SLC6A17	SLC6A17;NTT4	solute carrier family 6, member 17	0.95	0.93	1.09	0.74
GOLGA6A	GOLGA6A;FLJ75859;GLP;GOLGA6	golgin A6 family, member A	0.95	1.1	1.09	0.85
WBSCR17	WBSCR17;DKFZp434I2216;DKFZp761D2324;GALNT16;GALNT20;GALNTL3	Williams-Beuren syndrome chromosome region 17	0.94	0.95	1.09	0.84
SLC15A3	SLC15A3;FLJ26631;OCTP;PHT2;PTR3	solute carrier family 15, member 3	0.94	1.03	1.09	0.69
H2AFJ	H2AFJ;FLJ10903;FLJ52230;H2AJ;MGC921	H2A histone family, member J	0.94	0.97	1.09	0.82
MMP24	MMP24;MMP25;MT-MMP5;MT5-MMP	matrix metallopeptidase 24 (membrane-inserted)	0.94	0.94	1.09	0.7
KDSR	KDSR;DHSR;FLJ36555;FLJ92680;FVT1;SDR35C1	3-ketodihydrosphingosine reductase	0.94	1.01	1.09	0.71
HN1	HN1;ARM2;HN1A	hematological and neurological expressed 1	0.94	0.98	1.09	0.75
CUL3	CUL3	cullin 3	0.94	0.95	1.09	0.71
SLC9B2	SLC9B2;FLJ23984;NHA2;NHE10;NHEDC2	solute carrier family 9, subfamily B (cation proton antiporter 2), member 2	0.94	1	1.09	0.75
PCDHA2	PCDHA2;PCDH-ALPHA2	protocadherin alpha 2	0.94	1.01	1.09	0.84
GLB1L	GLB1L;MGC10771	galactosidase, beta 1-like	0.93	0.97	1.09	0.74
PRB3	PRB3;G1;MGC116862;MGC116863;MGC116864;PRG	proline-rich protein BstNI subfamily 3	0.93	1.05	1.09	0.72
PLD5	PLD5;FLJ40773;MGC120565;MGC120566;MGC120567;PLDC	phospholipase D family, member 5	0.93	1.08	1.09	0.71
RBL2	RBL2;FLJ26459;P130;Rb2	retinoblastoma-like 2 (p130)	0.93	1.09	1.09	0.82
P2RX3	P2RX3;MGC129956;P2X3	purinergic receptor P2X, ligand-gated ion channel, 3	0.92	1.03	1.09	0.8
KCNK3	KCNK3;K2p3.1;OAT1;TASK;TASK-1;TBAK1	potassium channel, subfamily K, member 3	0.92	1.04	1.09	0.81
HELT	HELT;HCM1228;HESL;Mgn;bHLHb44	helt bHLH transcription factor	0.92	1.04	1.09	0.74
C14orf80	C14orf80;MGC16771	chromosome 14 open reading frame 80	0.92	0.89	1.09	0.71
RBM14	RBM14;COAA;DKFZp779J0927;MGC15912;MGC31756;PSP2;SIP;SYTIP1;TMEM137	RNA binding motif protein 14	0.92	0.96	1.09	0.73
MTHFD1	MTHFD1;MTHFC;MTHFD	methylenetetrahydrofolate dehydrogenase (NADP+ dependent) 1, methenyltetrahydrofolate cyclohydrolase, formyltetrahydrofolate synthetase	0.92	0.93	1.09	0.74
GAGE12I	GAGE12I;AL4;CT4.7;GAGE-7B;GAGE7B	G antigen 12I	0.92	0.98	1.09	0.7
XKRX	XKRX;FLJ60646;XKR2;XPLAC;XRG2	XK, Kell blood group complex subunit-related, X-linked	0.9	0.94	1.09	0.79
KCNA4	KCNA4;HBK4;HK1;HPCN2;HUKII;KCNA4L;KCNA8;KV1.4;PCN2	potassium voltage-gated channel, shaker-related subfamily, member 4	0.9	0.97	1.09	0.76
MC1R	MC1R;CMM5;MGC14337;MSH-R;SHEP2	melanocortin 1 receptor (alpha melanocyte stimulating hormone receptor)	0.9	0.95	1.09	0.85
POU2F3	POU2F3;Epoc-1;FLJ40063;MGC126698;OCT-11;OCT11;OTF-11;PLA-1;PLA1;Skn-1a	POU class 2 homeobox 3	0.9	0.94	1.09	0.74
SLC2A10	SLC2A10;ATS;GLUT10;MGC126706	solute carrier family 2 (facilitated glucose transporter), member 10	0.9	0.88	1.09	0.7
FEM1A	FEM1A;DKFZp762M136;EPRAP	fem-1 homolog a (C. elegans)	0.9	1	1.09	0.76
DTNBP1	DTNBP1;DBND;DKFZp564K192;FLJ30031;HPS7;MGC20210;My031;SDY	dystrobrevin binding protein 1	0.9	1.01	1.09	0.77
HMGN4	HMGN4;HMG17L3;MGC5145;NHC	high mobility group nucleosomal binding domain 4	0.9	1.06	1.09	0.78
USF1	USF1;FCHL;FCHL1;HYPLIP1;MLTF;MLTFI;UEF;bHLHb11	upstream transcription factor 1	0.9	0.98	1.09	0.72
FCHSD2	FCHSD2;KIAA0769;NWK;SH3MD3	FCH and double SH3 domains 2	0.89	0.87	1.09	0.69

WDR76	WDR76;CDW14;FLJ12973	WD repeat domain 76	0.89	0.93	1.09	0.71
GLUL	GLUL;GLNS;GS;PIG43;PIG59	glutamate-ammonia ligase	0.89	0.93	1.09	0.67
ADAM21	ADAM21;ADAM 21;ADAM31;MGC125389	ADAM metallopeptidase domain 21	0.88	0.95	1.09	0.71
PRR16	PRR16;DSC54;MGC104614	proline rich 16	0.88	1.04	1.09	0.79
CCT6B	CCT6B;CCT-zeta-2;CCTZ-2;Cctz2;TCP-1-zeta-2;TSA303	chaperonin containing TCP1, subunit 6B (zeta 2)	0.88	0.96	1.09	0.71
ADAMTS3	ADAMTS3;ADAMTS-4;KIAA0366	ADAM metallopeptidase with thrombospondin type 1 motif, 3	0.88	0.96	1.09	0.72
ZG16	ZG16	ENTREZ STATUS: REMAPPED TO GENE ID: 653808	0.88	0.99	1.09	0.75
DDX60	DDX60;FLJ10787;FLJ20035	DEAD (Asp-Glu-Ala-Asp) box polypeptide 60	0.88	0.97	1.09	0.65
ISLR2	ISLR2;KIAA1465;LINX	immunoglobulin superfamily containing leucine-rich repeat 2	0.88	0.98	1.09	0.73
FBXW8	FBXW8;FBW6;FBW8;FBX29;FBXO29;FBXW6;MGC33534	F-box and WD repeat domain containing 8	0.88	0.92	1.09	0.67
UBXN1	UBXN1;2B28;SAKS1;UBXD10	UBX domain protein 1	0.88	1.01	1.09	0.75
KLF10	KLF10;EGRA;TIEG;TIEG1	Kruppel-like factor 10	0.88	0.97	1.09	0.77
FXN	FXN;CyaY;FA;FARR;FRDA;MGC57199;X25	frataxin	0.88	1.04	1.09	0.73
HIST1H1D	HIST1H1D;H1.3;H1D;H1F3;MGC138176	histone cluster 1, H1d	0.88	0.87	1.09	0.69
LOC399940	LOC399940	SPRY domain containing 5 pseudogene	0.88	0.94	1.09	0.85
PCDHGA10	PCDHGA10;PCDH-GAMMA-A10	protocadherin gamma subfamily A, 10	0.88	0.99	1.09	0.75
FAM69C	FAM69C;C18orf51	family with sequence similarity 69, member C	0.88	0.93	1.09	0.71
TRPV3	TRPV3;VRL3	transient receptor potential cation channel, subfamily V, member 3	0.87	0.95	1.09	0.74
SHBG	SHBG;ABP;MGC126834;MGC138391;SBP;TEBG	sex hormone-binding globulin	0.87	0.98	1.09	0.74
RECK	RECK;ST15	reversion-inducing-cysteine-rich protein with kazal motifs	0.87	0.94	1.09	0.78
RUFY3	RUFY3;KIAA0871;RIPX;SINGAR1	RUN and FYVE domain containing 3	0.87	0.98	1.09	0.73
ZNF12	ZNF12;GIOT-3;HZF11;KOX3;ZNF325	zinc finger protein 12	0.87	0.96	1.09	0.7
PSAT1	PSAT1;EPIP;MGC1460;PSA;PSAT	phosphoserine aminotransferase 1	0.87	0.88	1.09	0.78
MDP1	MDP1;FN6PASE;MDP-1;MGC5987	magnesium-dependent phosphatase 1	0.87	1	1.09	0.77
HTR3E	HTR3E;5-HT3c1;MGC120035;MGC120036;MGC120037	5-hydroxytryptamine (serotonin) receptor 3, family member E	0.86	0.99	1.09	0.76
JAK3	JAK3;JAK-3;JAK3_HUMAN;JAKL;L-JAK;LJAK	Janus kinase 3	0.86	0.94	1.09	0.78
ANKRD16	ANKRD16	ankyrin repeat domain 16	0.86	0.92	1.09	0.72
ZMYM5	ZMYM5;HSPC050;MYM;ZNF198L1;ZNF237	zinc finger, MYM-type 5	0.86	0.99	1.09	0.71
ITGA9	ITGA9;ALPHA-RLC;ITGA4L;RLC	integrin, alpha 9	0.86	0.91	1.09	0.67
CYP2A7	CYP2A7;CPA7;CPAD;CYP2A;CYPIIA7;P450-IIA4	cytochrome P450, family 2, subfamily A, polypeptide 7	0.86	0.91	1.09	0.64
DSCR6	DSCR6;RIPPLY3	Down syndrome critical region gene 6	0.86	1.02	1.09	0.75
TCIRG1	TCIRG1;ATP6N1C;ATP6V0A3;Atp6i;OC-116kDa;OC116;OPTB1;Stv1;TIRC7;Vph1;a3	T-cell, immune regulator 1, ATPase, H+ transporting, lysosomal V0 subunit A3	0.86	0.9	1.09	0.7
GAA	GAA;LYAG	glucosidase, alpha; acid	0.86	0.9	1.09	0.7
LRCH2	LRCH2;KIAA1495;MGC150671;dA204F4.4	leucine-rich repeats and calponin homology (CH) domain containing 2	0.86	0.96	1.09	0.73
AFF1	AFF1;AF4;MGC134969;MLLT2;PBM1	AF4/FMR2 family, member 1	0.86	0.94	1.09	0.68
IMMT	IMMT;DKFZp779P1653;HMP;MGC111146;MINOS2;P87;P87/89;P89;PIG52	inner membrane protein, mitochondrial	0.86	0.94	1.09	0.65
CXCL17	CXCL17;DMC;Dcip1;MGC138300;UNQ473;VCC-1;VCC1	chemokine (C-X-C motif) ligand 17	0.85	0.9	1.09	0.71
ZNF556	ZNF556;FLJ11637	zinc finger protein 556	0.85	0.89	1.09	0.68
TET2	TET2;FLJ20032;KIAA1546;MDS;MGC125715	tet methylcytosine dioxygenase 2	1.29	1.4	1.1	1.01
WNT3A	WNT3A;MGC119418;MGC119419;MGC119420	wingless-type MMTV integration site family, member 3A	1.19	1.34	1.1	1.02
UHRF1BP1	UHRF1BP1;C6orf107;FLJ20302;ICBP90;dJ349A12.1	UHRF1 binding protein 1	1.19	1.18	1.1	0.98
TPP2	TPP2;FLJ40359;TPP-2	tripeptidyl peptidase II	1.17	1.19	1.1	0.9
GLOD5	GLOD5	glyoxalase domain containing 5	1.15	1.26	1.1	0.91
ZRANB3	ZRANB3;4933425L19Rik;MGC105033;MGC75012	zinc finger, RAN-binding domain containing 3	1.15	1.27	1.1	0.99
MRPL36	MRPL36;BRIP1;L36mt;MGC104245;MRP-L36;PRPL36;RPMJ	mitochondrial ribosomal protein L36	1.15	1.19	1.1	1.01
ANAPC1	ANAPC1;APC1;MCPR;TSG24	anaphase promoting complex subunit 1	1.14	1.12	1.1	0.94
UNKL	UNKL;C16orf28;FLJ12623;FLJ23360;KIAA0734;MGC5179;ZC3H5L;ZC3HDC5L	unkempt homolog (Drosophila)-like	1.13	1.19	1.1	0.85
GOLGA8G	GOLGA8G;DKFZp434K052	golgin A8 family, member G	1.12	1.08	1.1	0.85
RNF135	RNF135;MGC13061;MMFD;REUL;Riplet	ring finger protein 135	1.1	1.16	1.1	0.84
STK10	STK10;LOK;PRO2729	serine/threonine kinase 10	1.1	1.22	1.1	1.01
ANKIB1	ANKIB1;DKFZp434A0225;FLJ33123;KIAA1386	ankyrin repeat and IBR domain containing 1	1.1	1.16	1.1	0.84
VPS41	VPS41;HVPS41;HVSP41;hVps41p	vacuolar protein sorting 41 homolog (S. cerevisiae)	1.1	1.16	1.1	0.84
KLHL6	KLHL6;FLJ00029	kelch-like 6 (Drosophila)	1.09	1.16	1.1	0.89
ZNF593	ZNF593;ZT86	zinc finger protein 593	1.09	1.25	1.1	0.85
RNF141	RNF141;MGC8715;ZFP26;ZNF230	ring finger protein 141	1.08	1.14	1.1	0.82
RBCK1	RBCK1;C20orf18;HOIL-1;HOIL1;RBCK2;RNF54;UBCE7IP3;XAP3;XAP4;ZRANB4	RanBP-type and C3HC4-type zinc finger containing 1	1.08	1.15	1.1	0.83
PTPRE	PTPRE;DKFZp313F1310;FLJ57799;FLJ58245;HPTPE;PTPE;R-PTP-EPSILON	protein tyrosine phosphatase, receptor type, E	1.07	1.17	1.1	0.91
TRIM15	TRIM15;RNF93;ZNF178;ZNFB7	tripartite motif containing 15	1.06	1.12	1.1	0.81
PTPN3	PTPN3;DKFZp686N0569;PTP-H1;PTPH1	protein tyrosine phosphatase, non-receptor type 3	1.06	1.17	1.1	0.91
RB1CC1	RB1CC1;CC1;FIP200	RB1-inducible coiled-coil 1	1.06	1.17	1.1	0.81
CDAN1	CDAN1;CDA1;CDAI;DLT;PRO1295	congenital dyserythropoietic anemia, type I	1.05	1.1	1.1	0.93
STAG3L3	STAG3L3;STAG3L1;STAG3L2	stromal antigen 3-like 3	1.05	1.1	1.1	0.85
SCN9A	SCN9A;ETHA;FEB3B;GEFSP7;NE-NA;NENA;Nav1.7;PN1	sodium channel, voltage-gated, type IX, alpha subunit	1.04	1.14	1.1	0.88
RNF180	RNF180;MGC120326;MGC120328	ring finger protein 180	1.04	1.09	1.1	0.79
SERHL	SERHL;BK126B4.1;BK126B4.2;HS126B42;dJ222E13.1	serine hydrolase-like	1.03	1.09	1.1	0.79
ELL2	ELL2	elongation factor, RNA polymerase II, 2	1.03	1.09	1.1	0.83
KDM5B	KDM5B;CT31;FLJ10538;FLJ12459;FLJ12491;FLJ16281;FLJ23670;JARID1B;PLU-1;PLU1;PUT1;RBBP2H1A	lysine (K)-specific demethylase 5B	1.03	1.08	1.1	0.78
CAPN13	CAPN13;FLJ23523	calpain 13	1.02	1.03	1.1	0.77
DDA1	DDA1;C19orf58;MGC2594;PCIA1	DET1 and DDB1 associated 1	1.02	1.06	1.1	0.86
S100A9	S100A9;60B8AG;CAGB;CFAG;CGLB;L1AG;LIAG;MAC387;MIF;MRP14;NIF;P14	S100 calcium binding protein A9	1	1	1.1	0.79
MAP4K2	MAP4K2;BL44;GCK;RAB8IP	mitogen-activated protein kinase kinase kinase kinase 2	1	1.19	1.1	0.84
PMP22	PMP22;CMT1A;CMT1E;DSS;GAS-3;HMSNIA;HNPP;MGC20769;Sp110	peripheral myelin protein 22	1	1.13	1.1	0.78
SCNN1A	SCNN1A;BESC2;ENaCa;ENaCalpha;FLJ21883;SCNEA;SCNN1	sodium channel, nonvoltage-gated 1 alpha	1	1.09	1.1	0.85
TXNL1	TXNL1;TRP32;TXL-1;TXNL;Txl	thioredoxin-like 1	1	1.06	1.1	0.79
DCUN1D1	DCUN1D1;DCNL1;DCUN1L1;RP42;SCCRO;SCRO;Tes3	DCN1, defective in cullin neddylation 1, domain containing 1 (S. cerevisiae)	1	1.01	1.1	0.76
C2orf50	C2orf50;FLJ25143;MGC149401	chromosome 2 open reading frame 50	0.99	0.97	1.1	0.76
DOK3	DOK3;DOKL;FLJ22570;FLJ39939	docking protein 3	0.99	1.03	1.1	0.83
C9orf78	C9orf78;HSPC220;bA409K20.3	chromosome 9 open reading frame 78	0.99	1.15	1.1	0.92
ACTR10	ACTR10;ACTR11;Arp11;HARP11	actin-related protein 10 homolog (S. cerevisiae)	0.99	1.07	1.1	0.8
DEPDC1B	DEPDC1B;BRCC3;FLJ11252;XTP1	DEP domain containing 1B	0.99	1.14	1.1	0.76
FEZF1	FEZF1;FEZ;ZNF312B	FEZ family zinc finger 1	0.98	1.04	1.1	0.8
IL28B	IL28B;IFNL3;IL-28B;IL28C	interleukin 28B (interferon, lambda 3)	0.98	1.12	1.1	0.85
TTLL5	TTLL5;KIAA0998;MGC117189;STAMP	tubulin tyrosine ligase-like family, member 5	0.98	0.97	1.1	0.76
PDZK1	PDZK1;CAP70;CLAMP;NHERF-3;NHERF3;PDZD1	PDZ domain containing 1	0.97	1.02	1.1	0.74
PDE4B	PDE4B;DKFZp686F2182;DPDE4;MGC126529;PDE4B5;PDEIVB	phosphodiesterase 4B, cAMP-specific	0.96	1.02	1.1	0.73
SESTD1	SESTD1;DKFZp434O0515;SOLO	SEC14 and spectrin domains 1	0.96	1.09	1.1	0.82
PARM1	PARM1;Cipar1;DKFZP564O0823;PARM-1;WSC4	prostate androgen-regulated mucin-like protein 1	0.96	1.04	1.1	0.86
TDRD10	TDRD10;DKFZp434M202	tudor domain containing 10	0.95	0.99	1.1	0.8
ITGB1BP2	ITGB1BP2;CHORDC3;ITGB1BP;MELUSIN;MGC119214	integrin beta 1 binding protein (melusin) 2	0.95	1.08	1.1	0.82
PIN4	PIN4;EPVH;MGC138486;PAR14;PAR17	protein (peptidylprolyl cis/trans isomerase) NIMA-interacting, 4 (parvulin)	0.95	1.06	1.1	0.71
DENND4C	DENND4C;C9orf55;C9orf55B;DKFZp686I09113;FLJ20686;bA513M16.3	DENN/MADD domain containing 4C	0.95	0.99	1.1	0.8
TRPM7	TRPM7;CHAK;CHAK1;FLJ20117;FLJ25718;LTRPC7;TRP-PLIK	transient receptor potential cation channel, subfamily M, member 7	0.95	1.02	1.1	0.92
IFT52	IFT52;C20orf9;CGI-53;NGD2;NGD5;dJ1028D15.1	intraflagellar transport 52 homolog (Chlamydomonas)	0.95	1.04	1.1	0.75
EFCAB5	EFCAB5;DKFZp434G2420;DKFZp686I0638;FLJ46247	EF-hand calcium binding domain 5	0.94	1.1	1.1	0.85
GUCA2A	GUCA2A;GCAP-I;GUCA2;STARA	guanylate cyclase activator 2A (guanylin)	0.94	0.92	1.1	0.77
SCGB2A1	SCGB2A1;LPHC;MGB2;MGC71973;UGB3	secretoglobin, family 2A, member 1	0.94	0.98	1.1	0.82
FNDC3A	FNDC3A;FLJ31509;FNDC3;HUGO;KIAA0970;bA203I16.1;bA203I16.5	fibronectin type III domain containing 3A	0.94	0.92	1.1	0.72
ATG4D	ATG4D;APG4-D;APG4D;AUTL4	ATG4 autophagy related 4 homolog D (S. cerevisiae)	0.94	1.02	1.1	0.77
GOLGA5	GOLGA5;GOLIM5;RFG5;ret-II	golgin A5	0.94	1.02	1.1	0.85
QARS	QARS;GLNRS;PRO2195	glutaminyl-tRNA synthetase	0.94	1.02	1.1	0.77
TRAP1	TRAP1;HSP75;HSP90L	TNF receptor-associated protein 1	0.94	0.97	1.1	0.77
C7orf51	C7orf51;FLJ37538	chromosome 7 open reading frame 51	0.93	1.03	1.1	0.72
MRPS33	MRPS33;FLJ21123;MRP-S33;PTD003	mitochondrial ribosomal protein S33	0.93	1.07	1.1	0.8
RNF183	RNF183;FLJ31197;MGC4734	ring finger protein 183	0.92	1	1.1	0.89
ZNF35	ZNF35;HF.10;HF10;Zfp105	zinc finger protein 35	0.92	1	1.1	0.74
FAM160A2	FAM160A2;C11orf56;DKFZp566M1046;FLJ22665;KIAA1759	family with sequence similarity 160, member A2	0.92	0.98	1.1	0.74
C18orf56	C18orf56	chromosome 18 open reading frame 56	0.92	0.98	1.1	0.75
ACOT8	ACOT8;HNAACTE;PTE-1;PTE-2;PTE1;PTE2;hACTE-III;hTE	acyl-CoA thioesterase 8	0.92	1.08	1.1	0.71
CAMK2D	CAMK2D;CAMKD;DKFZp686G23119;DKFZp686I2288;MGC44911	calcium/calmodulin-dependent protein kinase II delta	0.92	1	1.1	0.83
RECQL	RECQL;RECQL1;RecQ1	RecQ protein-like (DNA helicase Q1-like)	0.92	1.1	1.1	0.82
FAM60A	FAM60A;C12orf14;TERA	family with sequence similarity 60, member A	0.92	1.08	1.1	0.76
GLRX5	GLRX5;C14orf87;FLB4739;GRX5;MGC14129;PR01238;PRO1238	glutaredoxin 5	0.92	1.06	1.1	0.76
SLC3A2	SLC3A2;4F2;4F2HC;4T2HC;CD98;CD98HC;MDU1;NACAE	solute carrier family 3 (activators of dibasic and neutral amino acid transport), member 2	0.92	1	1.1	0.74
EXD1	EXD1;EXDL1;MGC33637	exonuclease 3'-5' domain containing 1	0.92	1.08	1.1	0.83
TMEM223	TMEM223;MGC3196	transmembrane protein 223	0.92	0.92	1.1	0.73
CXorf66	CXorf66	chromosome X open reading frame 66	0.92	1	1.1	0.73
SGK1	SGK1;SGK	serum/glucocorticoid regulated kinase 1	0.91	1.08	1.1	0.77
PROS1	PROS1;PROS;PS21;PS22;PS23;PS24;PS25;PSA	protein S (alpha)	0.91	0.98	1.1	0.69
DOM3Z	DOM3Z;DOM3L;NG6;RAI1	dom-3 homolog Z (C. elegans)	0.91	0.98	1.1	0.74
ADRA1A	ADRA1A;ADRA1C;ADRA1L1;ALPHA1AAR	adrenergic, alpha-1A-, receptor	0.9	0.95	1.1	0.86
GEMIN8	GEMIN8;FAM51A1;FLJ20514;FLJ43072	gem (nuclear organelle) associated protein 8	0.9	1.07	1.1	0.71
PYCARD	PYCARD;ASC;CARD5;MGC10332;TMS;TMS-1;TMS1	PYD and CARD domain containing	0.9	0.94	1.1	0.74
LSM11	LSM11;FLJ38273	LSM11, U7 small nuclear RNA associated	0.9	0.97	1.1	0.73
DIP2A	DIP2A;C21orf106;DIP2	DIP2 disco-interacting protein 2 homolog A (Drosophila)	0.9	0.99	1.1	0.77
PFKFB3	PFKFB3;FLJ37326;IPFK2;PFK2	6-phosphofructo-2-kinase/fructose-2,6-biphosphatase 3	0.9	0.92	1.1	0.69
C12orf57	C12orf57;C10;FLJ17308;GRCC10	chromosome 12 open reading frame 57	0.9	0.94	1.1	0.72
B4GALT3	B4GALT3;beta4Gal-T3	UDP-Gal:betaGlcNAc beta 1,4- galactosyltransferase, polypeptide 3	0.9	1.04	1.1	0.69
ZNF428	ZNF428;C19orf37;MGC51082;Zfp428	zinc finger protein 428	0.89	1.06	1.1	0.71
ARHGAP17	ARHGAP17;DKFZp564A1363;FLJ37567;FLJ43368;MGC87805;MST066;MST110;MSTP038;MSTP066;MSTP110;NADRIN;PP367;PP4534;RICH1;RICH1B;WBP15	Rho GTPase activating protein 17	0.89	1.02	1.1	0.69

DOC2B	DOC2B;DOC2BL	double C2-like domains, beta	0.88	1.02	1.1	0.82
TRIM72	TRIM72;MG53	tripartite motif containing 72	0.88	0.96	1.1	0.86
TEF	TEF;KIAA1655	thyrotrophic embryonic factor	0.88	0.9	1.1	0.72
MYO18A	MYO18A;DKFZp686L0243;KIAA0216;MYSPDZ;SPR210	myosin XVIIIA	0.88	0.94	1.1	0.71
GLMN	GLMN;FAP;FAP48;FAP68;FKBPAP;GLML;GVM;VMGLOM	glomulin, FKBP associated protein	0.88	0.88	1.1	0.73
GAN	GAN;FLJ38059;GAN1;KLHL16	gigaxonin	0.88	0.94	1.1	0.68
MPHOSPH6	MPHOSPH6;MPP;MPP-6;MPP6	M-phase phosphoprotein 6	0.88	0.97	1.1	0.67
USP48	USP48;DKFZp762M1713;MGC132556;MGC14879;RAP1GA1;USP31	ubiquitin specific peptidase 48	0.88	0.97	1.1	0.72
CLPX	CLPX	ClpX caseinolytic peptidase X homolog (E. coli)	0.88	0.94	1.1	0.71
AAMP	AAMP	angio-associated, migratory cell protein	0.88	0.92	1.1	0.78
WDR82	WDR82;MST107;MSTP107;PRO2730;PRO34047;SWD2;TMEM113;WDR82A	WD repeat domain 82	0.88	0.92	1.1	0.7
CSNK1A1	CSNK1A1;CK1;HLCDGP1;PRO2975	casein kinase 1, alpha 1	0.88	1.05	1.1	0.74
PIDD	PIDD;DKFZp434D229;LRDD;MGC16925	p53-induced death domain protein	0.88	1	1.1	0.67
PKD1L2	PKD1L2;DKFZp686J19100;FLJ45333;PC1L2	polycystic kidney disease 1-like 2	0.87	1.01	1.1	0.78
PLP2	PLP2;A4;A4LSB;MGC126187	proteolipid protein 2 (colonic epithelium-enriched)	0.87	0.93	1.1	0.71
BPY2B	BPY2B;BPY2;VCY2;VCY2B	basic charge, Y-linked, 2B	0.87	0.98	1.1	0.7
ASB12	ASB12;FLJ39577	ankyrin repeat and SOCS box containing 12	0.86	0.91	1.1	0.66
RGS6	RGS6;DKFZp313G1241;FLJ43552;GAP;MGC142132	regulator of G-protein signaling 6	0.86	1	1.1	0.66
VAX1	VAX1;MGC126743;MGC126745	ventral anterior homeobox 1	0.86	0.94	1.1	0.64
CCDC57	CCDC57;FLJ00130;FLJ23754;FLJ43953;MGC102869	coiled-coil domain containing 57	0.86	0.94	1.1	0.68
TBC1D10C	TBC1D10C;CARABIN;FLJ00332;MGC46488	TBC1 domain family, member 10C	0.86	0.9	1.1	0.76
TMEM120B	TMEM120B	transmembrane protein 120B	0.86	0.92	1.1	0.77
CDC73	CDC73;C1orf28;FLJ23316;HPTJT;HRPT2;HYX	cell division cycle 73, Paf1/RNA polymerase II complex component, homolog (S. cerevisiae)	0.86	0.98	1.1	0.71
BLZF1	BLZF1;GOLGIN-45;JEM-1;JEM-1s;JEM1;MGC22497	basic leucine zipper nuclear factor 1	0.86	0.92	1.1	0.67
PACRGL	PACRGL;C4orf28;MGC29898	PARK2 co-regulated-like	0.86	0.92	1.1	0.69
CDKL3	CDKL3;NKIAMRE	cyclin-dependent kinase-like 3	0.85	0.94	1.1	0.78
NUDT13	NUDT13	nudix (nucleoside diphosphate linked moiety X)-type motif 13	0.85	0.91	1.1	0.64
DDX55	DDX55;FLJ16577;KIAA1595;MGC33209	DEAD (Asp-Glu-Ala-Asp) box polypeptide 55	0.85	0.9	1.1	0.66
CEP350	CEP350;CAP350;FLJ38282;FLJ44058;GM133;KIAA0480	centrosomal protein 350kDa	0.85	0.9	1.1	0.69
SPPL2B	SPPL2B;IMP-4;IMP4;KIAA1532;MGC111084;PSL1	signal peptide peptidase-like 2B	1.24	1.28	1.11	0.96
RBM6	RBM6;3G2;DEF-3;DEF3;DKFZp686B0877;FLJ36517;FLJ39446;FLJ42036;HLC-11;NY-LU-12;g16	RNA binding motif protein 6	1.15	1.38	1.11	1.02
C12orf60	C12orf60;FLJ31651;MGC47869	chromosome 12 open reading frame 60	1.11	1.33	1.11	1.02
GRASP	GRASP;TAMALIN	GRP1 (general receptor for phosphoinositides 1)-associated scaffold protein	1.11	1.29	1.11	1.05
TRIM26	TRIM26;AFP;FLJ16483;RNF95;ZNF173	tripartite motif containing 26	1.1	1.17	1.11	0.84
MLL3	MLL3;DKFZp686C08112;FLJ12625;FLJ38309;HALR;KIAA1506;KMT2C;MGC119851;MGC119852;MGC119853	myeloid/lymphoid or mixed-lineage leukemia 3	1.1	1.17	1.11	0.85
HRC	HRC;MGC133236	histidine rich calcium binding protein	1.09	1.16	1.11	0.84
PRCP	PRCP;HUMPCP;MGC2202;PCP	prolylcarboxypeptidase (angiotensinase C)	1.09	1.12	1.11	0.84
PABPC5	PABPC5;FLJ51677;PABP5	poly(A) binding protein, cytoplasmic 5	1.08	1.23	1.11	0.85
TRIM2	TRIM2;KIAA0517;RNF86	tripartite motif containing 2	1.08	1.15	1.11	0.83
TMEM132D	TMEM132D;KIAA1944;MGC138770;MGC138771;MOLT	transmembrane protein 132D	1.07	1.14	1.11	0.87
GBP7	GBP7;FLJ38822;GBP4L	guanylate binding protein 7	1.06	1.17	1.11	0.84
UQCRH	UQCRH;MGC111572;QCR6;UQCR8	ubiquinol-cytochrome c reductase hinge protein	1.06	1.09	1.11	0.87
MEX3B	MEX3B;DKFZp434J0617;MEX-3B;MGC117199;RKHD3;RNF195	mex-3 homolog B (C. elegans)	1.06	1.15	1.11	1.03
BMP2K	BMP2K;BIKE;DKFZp434K0614;DKFZp434P0116	BMP2 inducible kinase	1.06	1.17	1.11	0.97
KIAA0020	KIAA0020;HLA-HA8;MGC8749;PEN;PUF6;XTP5	KIAA0020	1.06	1.15	1.11	0.87
KCNK18	KCNK18;K2p18.1;MGR13;TRESK;TRESK-2;TRESK2;TRIK	potassium channel, subfamily K, member 18	1.04	1.14	1.11	0.89
MYO7B	MYO7B;DKFZp686A08248	myosin VIIB	1.04	1.1	1.11	0.85
SLC18A3	SLC18A3;MGC12716;VACHT	solute carrier family 18 (vesicular acetylcholine), member 3	1.04	1.11	1.11	0.82
KIAA1841	KIAA1841;MGC39557	KIAA1841	1.04	1.09	1.11	0.92
RNF222	RNF222	ring finger protein 222	1.04	1.1	1.11	0.8
REEP6	REEP6;C19orf32;DP1L1;FLJ25383;TB2L1	receptor accessory protein 6	1.04	1.21	1.11	0.87
TSPAN33	TSPAN33;MGC50844;PEN	tetraspanin 33	1.04	1.03	1.11	0.81
ESYT1	ESYT1;FAM62A;KIAA0747;MBC2	extended synaptotagmin-like protein 1	1.04	1.03	1.11	0.81
RGS7	RGS7	regulator of G-protein signaling 7	1.02	1.11	1.11	0.95
UFSP2	UFSP2;C4orf20;FLJ11200	UFM1-specific peptidase 2	1.02	1.07	1.11	0.86
PCSK7	PCSK7;FLJ23503;FLJ45329;LPC;PC7;PC8;SPC7	proprotein convertase subtilisin/kexin type 7	1.02	1.12	1.11	0.84
AGTRAP	AGTRAP;ATRAP;MGC29646	angiotensin II receptor-associated protein	1.02	1.1	1.11	0.79
TARS2	TARS2;FLJ12528;TARSL1	threonyl-tRNA synthetase 2, mitochondrial (putative)	1.02	1.17	1.11	0.84
EXOSC3	EXOSC3;MGC15120;MGC723;RP11-3J10.8;RRP40;Rrp40p;bA3J10.7;hRrp-40;hRrp40p;p10	exosome component 3	1.02	1.03	1.11	0.82
COPS7A	COPS7A;CSN7A;MGC110877	COP9 constitutive photomorphogenic homolog subunit 7A (Arabidopsis)	1.02	1.18	1.11	0.81
AGAP3	AGAP3;CENTG3;CRAG;FLJ16146;FLJ34452;MRIP-1	ArfGAP with GTPase domain, ankyrin repeat and PH domain 3	1.02	1.08	1.11	0.91
CDC37L1	CDC37L1;CDC37B;FLJ20639;HARC	cell division cycle 37 homolog (S. cerevisiae)-like 1	1	1.08	1.11	0.85
IFNGR2	IFNGR2;AF-1;IFGR2;IFNGT1	interferon gamma receptor 2 (interferon gamma transducer 1)	1	1.08	1.11	0.8
ERBB2	ERBB2;CD340;HER-2;HER-2/neu;HER2;MLN 19;NEU;NGL;TKR1	v-erb-b2 erythroblastic leukemia viral oncogene homolog 2, neuro/glioblastoma derived oncogene homolog (avian)	1	1.1	1.11	0.92
PPIB	PPIB;CYP-S1;CYPB;MGC14109;MGC2224;OI9;SCYLP	peptidylprolyl isomerase B (cyclophilin B)	1	1.03	1.11	0.91
TSHZ2	TSHZ2;C20orf17;DKFZp686K2480;FLJ33887;OVC10-2;TSH2;ZABC2;ZNF218	teashirt zinc finger homeobox 2	0.99	1.19	1.11	0.91
NT5M	NT5M;dNT-2;dNT2;mdN	5',3'-nucleotidase, mitochondrial	0.99	1.02	1.11	0.77
LIPH	LIPH;AH;ARWH2;LAH2;LPDLR;PLA1B;mPA-PLA1	lipase, member H	0.98	1.1	1.11	0.87
CUEDC2	CUEDC2;C10orf66;MGC2491;bA18I14.5	CUE domain containing 2	0.98	1.14	1.11	0.92
ARFGAP1	ARFGAP1;ARF1GAP;HRIHFB2281;MGC39924	ADP-ribosylation factor GTPase activating protein 1	0.98	1.16	1.11	0.77
EGFL8	EGFL8;C6orf8;FLJ44493;FLJ52513;FLJ75166;FLJ78536;FLJ97375;MGC44938;MGC59719;NG3	EGF-like-domain, multiple 8	0.98	1.04	1.11	0.8
TXLNB	TXLNB;C6orf198;DKFZp451A175;LST001;MDP77;dJ522B19.2	taxilin beta	0.97	1.07	1.11	0.77
USP42	USP42;FLJ12697	ubiquitin specific peptidase 42	0.97	1.04	1.11	0.77
HINT1	HINT1;FLJ30414;FLJ32340;HINT;PKCI-1;PRKCNH1	histidine triad nucleotide binding protein 1	0.97	1.06	1.11	0.74
C14orf93	C14orf93;FLJ12154	chromosome 14 open reading frame 93	0.96	0.96	1.11	0.8
NBPF3	NBPF3;AE2;DKFZp434D177	neuroblastoma breakpoint family, member 3	0.96	1.01	1.11	0.82
TMF1	TMF1;ARA160;TMF	TATA element modulatory factor 1	0.96	1.04	1.11	0.73
PEBP1	PEBP1;HCNP;HCNPpp;PBP;PEBP;PEBP-1;RKIP	phosphatidylethanolamine binding protein 1	0.96	1.15	1.11	0.88
CYP2A13	CYP2A13;CPAD;CYP2A	cytochrome P450, family 2, subfamily A, polypeptide 13	0.94	0.98	1.11	0.77
THBS3	THBS3;MGC119564;MGC119565;TSP3	thrombospondin 3	0.94	0.99	1.11	0.83
ITIH5	ITIH5;DKFZp686F0145;ITI-HC5;MGC10848	inter-alpha-trypsin inhibitor heavy chain family, member 5	0.94	0.94	1.11	0.74
TSNARE1	TSNARE1;FLJ31164;MGC142116	t-SNARE domain containing 1	0.94	0.94	1.11	0.73
DEDD2	DEDD2;FLAME-3	death effector domain containing 2	0.94	1.01	1.11	0.73
EFHA1	EFHA1;1110008L20Rik;FLJ25016;FLJ34588	EF-hand domain family, member A1	0.94	1.03	1.11	0.74
BIRC3	BIRC3;AIP1;API2;CIAP2;HAIP1;HIAP1;MALT2;MIHC;RNF49;c-IAP2	baculoviral IAP repeat containing 3	0.94	0.99	1.11	0.72
SNX12	SNX12;MGC118982;MGC118983	sorting nexin 12	0.94	1.06	1.11	0.81
RAB27A	RAB27A;GS2;HsT18676;MGC117246;RAB27;RAM	RAB27A, member RAS oncogene family	0.94	1.01	1.11	0.74
GNAI3	GNAI3;87U6;FLJ26559	guanine nucleotide binding protein (G protein), alpha inhibiting activity polypeptide 3	0.94	0.96	1.11	0.85
OPN1MW	OPN1MW;CBBM;CBD;COD5;GCP;GOP;MGC176615;MGC177321;MGC198468;MGC198469;OPN1MW1;OPN1MW2	opsin 1 (cone pigments), medium-wave-sensitive	0.94	0.97	1.11	0.73
PHKG1	PHKG1;PHKG	phosphorylase kinase, gamma 1 (muscle)	0.93	1.12	1.11	0.79
ALDH3A1	ALDH3A1;ALDH3;ALDHIII;MGC10406	aldehyde dehydrogenase 3 family, member A1	0.93	1.04	1.11	0.83
KIAA1522	KIAA1522	KIAA1522	0.93	0.99	1.11	0.76
ZDHHC9	ZDHHC9;CGI89;CXorf11;DHHC9;MMSA1;MRXSZ;ZDHHC10;ZNF379;ZNF380	zinc finger, DHHC-type containing 9	0.93	1.06	1.11	0.81
TSHZ1	TSHZ1;NY-CO-33;SDCCAG33;TSH1	teashirt zinc finger homeobox 1	0.92	1.09	1.11	0.77
S100A2	S100A2;CAN19;MGC111539;S100L	S100 calcium binding protein A2	0.92	0.99	1.11	0.75
RGL1	RGL1;KIAA0959;RGL	ral guanine nucleotide dissociation stimulator-like 1	0.92	1.04	1.11	0.8
RNF214	RNF214;DKFZp547C195	ring finger protein 214	0.92	1.01	1.11	0.91
AMFR	AMFR;GP78;RNF45	autocrine motility factor receptor	0.92	1.02	1.11	0.91
STXBP3	STXBP3;MUNC18-3;MUNC18C;PSP;UNC-18C	syntaxin binding protein 3	0.92	1.07	1.11	0.72
APP	APP;AAA;ABETA;ABPP;AD1;APPI;CTFgamma;CVAP;PN-II;PN2	amyloid beta (A4) precursor protein	0.92	0.95	1.11	0.71
DCAF15	DCAF15;C19orf72;MGC99481	DDB1 and CUL4 associated factor 15	0.92	0.94	1.11	0.74
UAP1	UAP1;AGX;AGX1;AGX2;SPAG2	UDP-N-acteylglucosamine pyrophosphorylase 1	0.91	0.94	1.11	0.75
APOBR	APOBR;APOB100R;APOB48R	apolipoprotein B receptor	0.91	0.98	1.11	0.77
ACPP	ACPP;5'-NT;ACP-3;ACP3;PAP	acid phosphatase, prostate	0.9	0.93	1.11	0.7
RNF186	RNF186;FLJ20225;RP11-91K11.1	ring finger protein 186	0.9	1	1.11	0.89
C1orf212	C1orf212;FLJ90372;MGC138436	chromosome 1 open reading frame 212	0.9	0.96	1.11	0.74
DUX4	DUX4;DUX10	double homeobox 4	0.9	1.02	1.11	0.78
TM9SF1	TM9SF1;HMP70;MP70	transmembrane 9 superfamily member 1	0.9	0.97	1.11	0.78
ZNF480	ZNF480;MGC32104	zinc finger protein 480	0.9	1.06	1.11	0.74
NOC2L	NOC2L;DKFZp564C186;FLJ35172;NET15;NET7;NIR	nucleolar complex associated 2 homolog (S. cerevisiae)	0.9	1	1.11	0.72
ZFR	ZFR;FLJ41312;ZFR1	zinc finger RNA binding protein	0.9	0.96	1.11	0.69
TOR1AIP1	TOR1AIP1;DKFZp586G011;FLJ13142;LAP1B;MGC3413	torsin A interacting protein 1	0.9	0.96	1.11	0.73
GDI1	GDI1;1A;FLJ41411;GDIL;MRX41;MRX48;OPHN2;RABGD1A;RABGDIA;XAP-4	GDP dissociation inhibitor 1	0.9	1.06	1.11	0.82
ORC5	ORC5;ORC5L;ORC5P;ORC5T	origin recognition complex, subunit 5	0.9	1.02	1.11	0.69
RSPH6A	RSPH6A;RSHL1;RSP4;RSP6;RSPH4B	radial spoke head 6 homolog A (Chlamydomonas)	0.9	0.98	1.11	0.82
EMB	EMB;MGC71745	embigin	0.89	1.02	1.11	0.73
HECTD2	HECTD2;FLJ16050	HECT domain containing 2	0.89	0.91	1.11	0.68
CCDC127	CCDC127;FLJ25701	coiled-coil domain containing 127	0.89	1.01	1.11	0.76
C1orf201	C1orf201;FLJ33340	chromosome 1 open reading frame 201	0.89	0.96	1.11	0.72
TMEM161B	TMEM161B;FLB3342;MGC33214;PRO1313	transmembrane protein 161B	0.89	0.94	1.11	0.76
ARL6IP1	ARL6IP1;AIP1;ARL6IP;ARMER;KIAA0069	ADP-ribosylation factor-like 6 interacting protein 1	0.89	0.96	1.11	0.73
AKAP3	AKAP3;AKAP110;CT82;FSP95;PRKA3;SOB1	A kinase (PRKA) anchor protein 3	0.88	0.96	1.11	0.7
APLNR	APLNR;AGTRL1;APJ;APJR;FLJ90771;FLJ96609;HG11;MGC45246	apelin receptor	0.88	0.96	1.11	0.86
MPRIP	MPRIP;KIAA0864;M-RIP;MRIP;RHOIP3;RIP3;p116Rip	myosin phosphatase Rho interacting protein	0.88	0.99	1.11	0.69
ZNF791	ZNF791;FLJ90396;MGC126179;MGC126180	zinc finger protein 791	0.88	0.92	1.11	0.75
EXOC6	EXOC6;DKFZp761I2124;EXOC6A;FLJ1125;FLJ11251;MGC33397;SEC15;SEC15L;SEC15L1;SEC15L3;Sec15p	exocyst complex component 6	0.88	0.94	1.11	0.71
C4orf46	C4orf46	chromosome 4 open reading frame 46	0.88	0.94	1.11	0.68
RAB3GAP2	RAB3GAP2;DKFZp434D245;FLJ14579;KIAA0839;RAB3-GAP150;RAB3GAP150;WARBM2;p150	RAB3 GTPase activating protein subunit 2 (non-catalytic)	0.88	0.93	1.11	0.67
SLC35A3	SLC35A3;DKFZp781P1297	solute carrier family 35 (UDP-N-acetylglucosamine (UDP-GlcNAc) transporter), member A3	0.88	0.98	1.11	0.72

KCTD10	KCTD10;FLJ41739;ULRO61	potassium channel tetramerisation domain containing 10	0.88	1.02	1.11	0.79
ERBB2IP	ERBB2IP;ERBIN;LAP2	erbb2 interacting protein	0.88	0.94	1.11	0.74
RHBDD2	RHBDD2;NPD007;RHBDL7	rhomboid domain containing 2	0.88	1.02	1.11	0.73
PRDX5	PRDX5;ACR1;AOEB166;B166;MGC117264;MGC142283;MGC142285;PLP;PMP20;PRDX6;PRXV	peroxiredoxin 5	0.88	0.99	1.11	0.77
NSUN7	NSUN7;FLJ14001	NOP2/Sun domain family, member 7	0.87	0.94	1.11	0.71
ARFRP1	ARFRP1;ARL18;ARP;Arp1	ADP-ribosylation factor related protein 1	0.87	0.92	1.11	0.72
SELENBP1	SELENBP1;FLJ13813;LPSB;SBP56;SP56;hSBP;hSP56	selenium binding protein 1	0.87	0.99	1.11	0.75
MKL1	MKL1;BSAC;MAL;MRTF-A	megakaryoblastic leukemia (translocation) 1	0.87	0.97	1.11	0.65
SLFN13	SLFN13;DKFZp666J196;DKFZp686I026;FLJ31952;SLFN10	schlafen family member 13	0.87	1.02	1.11	0.79
NCKAP1L	NCKAP1L;HEM1	NCK-associated protein 1-like	0.86	0.94	1.11	0.7
CCL5	CCL5;D17S136E;MGC17164;RANTES;SCYA5;SISd;TCP228	chemokine (C-C motif) ligand 5	0.86	0.98	1.11	0.74
BEX5	BEX5;MGC104434;MGC126446;NGFRAP1L1	brain expressed, X-linked 5	0.86	0.94	1.11	0.68
CAMK1D	CAMK1D;CKLiK;CaM-K1;CaMKID	calcium/calmodulin-dependent protein kinase ID	0.86	1.02	1.11	0.73
ELF2	ELF2;EU32;NERF;NERF-1A;NERF-1B;NERF-1a,b;NERF-2	E74-like factor 2 (ets domain transcription factor)	0.86	0.9	1.11	0.71
KLHDC4	KLHDC4;DKFZp434G0522;FLJ00104	kelch domain containing 4	0.86	0.93	1.11	0.71
CCRN4L	CCRN4L;CCR4L;MGC142054;MGC142060;MGC4120817;MGC78549;NOC	CCR4 carbon catabolite repression 4-like (S. cerevisiae)	0.86	0.92	1.11	0.68
TIPRL	TIPRL;MGC3794;TIP;TIP41;dJ69E11.3	TIP41, TOR signaling pathway regulator-like (S. cerevisiae)	0.86	0.93	1.11	0.7
37500	SEPT2;DIFF6;KIAA0158;NEDD5;Pnutl3;hNedd5	septin 2	0.86	1.01	1.11	0.72
TCEA1	TCEA1;GTF2S;SII;TCEA;TF2S;TFIIS	transcription elongation factor A (SII), 1	0.86	0.95	1.11	0.71
LOC652759	LOC652759	ENTREZ STATUS: DISCONTINUED	0.86	0.9	1.11	0.66
CT47A10	CT47A10;CT47;CT47.10	cancer/testis antigen family 47, member A10	0.86	0.98	1.11	0.75
OR2Y1	OR2Y1;OR5-2	olfactory receptor, family 2, subfamily Y, member 1	0.85	1.04	1.11	0.76
YY1	YY1;DELTA;INO80S;NF-E1;UCRBP;YIN-YANG-1	YY1 transcription factor	0.85	0.91	1.11	0.67
RPF1	RPF1;BXDC5;DKFZp761G0415;DKFZp761M0215;FLJ12475;RP11-118B23.1	ribosome production factor 1 homolog (S. cerevisiae)	0.85	1.01	1.11	0.71
HOXA1	HOXA1;BSAS;HOX1;HOX1F;MGC45232	homeobox A1	1.45	1.58	1.12	1.23
RAMP3	RAMP3	receptor (G protein-coupled) activity modifying protein 3	1.29	1.45	1.12	0.98
HOXD13	HOXD13;BDE;BDSD;HOX4I;SPD	homeobox D13	1.25	1.34	1.12	0.96
LGR5	LGR5;FEX;GPR49;GPR67;GRP49;HG38;MGC117008	leucine-rich repeat containing G protein-coupled receptor 5	1.23	1.28	1.12	0.96
GTPBP3	GTPBP3;FLJ14700;FLJ17836;GTPBG3;MSS1;MTGP1;THDF1	GTP binding protein 3 (mitochondrial)	1.23	1.4	1.12	1.07
MEF2A	MEF2A;ADCAD1;RSRFC4;RSRFC9;mef2	myocyte enhancer factor 2A	1.19	1.27	1.12	0.92
FUBP3	FUBP3;FBP3;FLJ25229	far upstream element (FUSE) binding protein 3	1.19	1.2	1.12	0.95
TEX264	TEX264;DKFZp451H0417;FLJ13935;SIG11;ZSIG11	testis expressed 264	1.17	1.32	1.12	0.95
KLF17	KLF17;FLJ40160;ZNF393;Zfp393	Kruppel-like factor 17	1.16	1.17	1.12	0.91
TXNRD3	TXNRD3;TGR;TR2;TRXR3	thioredoxin reductase 3	1.16	1.21	1.12	1.07
UBE2J2	UBE2J2;NCUBE-2;NCUBE2;PRO2121	ubiquitin-conjugating enzyme E2, J2	1.16	1.19	1.12	0.9
ALDH6A1	ALDH6A1;MGC40271;MMSADHA;MMSDH	aldehyde dehydrogenase 6 family, member A1	1.16	1.38	1.12	0.91
BDKRB2	BDKRB2;B2R;BK-2;BK2;BKR2;BRB2;DKFZp686O088	bradykinin receptor B2	1.15	1.19	1.12	0.9
FAM81A	FAM81A;MGC26690	family with sequence similarity 81, member A	1.15	1.23	1.12	0.94
HEATR6	HEATR6;ABC1;DKFZp686D22141;FLJ22087;MGC148096	HEAT repeat containing 6	1.15	1.35	1.12	0.96
LIX1L	LIX1L;DKFZp762F237;MGC46719	Lix1 homolog (mouse)-like	1.14	1.28	1.12	0.92
RAD18	RAD18;RNF73	RAD18 homolog (S. cerevisiae)	1.13	1.21	1.12	0.88
TBXAS1	TBXAS1;BDPLT14;CYP5;CYP5A1;FLJ52771;GHOSAL;THAS;TS;TXAS;TXS	thromboxane A synthase 1 (platelet)	1.12	1.27	1.12	1.01
HCAR3	HCAR3;GPR109B;HCA3;HM74;PUMAG;Puma-g	hydroxycarboxylic acid receptor 3	1.12	1.17	1.12	0.88
GNG11	GNG11;GNGT11	guanine nucleotide binding protein (G protein), gamma 11	1.11	1.27	1.12	0.97
TRIM60	TRIM60;FLJ35882;MGC119325;RNF129;RNF33	tripartite motif containing 60	1.1	1.18	1.12	0.85
RNF125	RNF125;FLJ20456;MGC21737;TRAC1	ring finger protein 125	1.09	1.17	1.12	0.84
PHF21A	PHF21A;BHC80;KIAA1696	PHD finger protein 21A	1.08	1.16	1.12	0.84
GRIN3B	GRIN3B;NR3B	glutamate receptor, ionotropic, N-methyl-D-aspartate 3B	1.08	1.2	1.12	0.94
IFNAR2	IFNAR2;IFN-R;IFN-alpha-REC;IFNABR;IFNARB	interferon (alpha, beta and omega) receptor 2	1.08	1.13	1.12	0.9
TCL6	TCL6;FLJ13652;TNG1;TNG2	T-cell leukemia/lymphoma 6 (non-protein coding)	1.06	1.14	1.12	0.87
RNF149	RNF149;DNAPTP2;FLJ90504	ring finger protein 149	1.06	1.13	1.12	0.82
FCHSD1	FCHSD1;FLJ00007;NWK2	FCH and double SH3 domains 1	1.05	1.06	1.12	0.85
GIPR	GIPR;MGC126722;PGQTL2	gastric inhibitory polypeptide receptor	1.04	1.07	1.12	0.81
CDKN2B	CDKN2B;CDK4I;INK4B;MTS2;P15;TP15;p15INK4b	cyclin-dependent kinase inhibitor 2B (p15, inhibits CDK4)	1.04	1.15	1.12	1.03
NUP54	NUP54;MGC13407	nucleoporin 54kDa	1.04	1.21	1.12	0.91
CASP2	CASP2;CASP-2;ICH1;NEDD-2;NEDD2;PPP1R57	caspase 2, apoptosis-related cysteine peptidase	1.04	1.15	1.12	0.86
ADCK3	ADCK3;ARCA2;CABC1;COQ8;MGC4849;SCAR9	aarF domain containing kinase 3	1.04	1.24	1.12	0.81
TAMM41	TAMM41;C3orf31;DKFZp434E0519;MGC16471;TAM41	TAM41, mitochondrial translocator assembly and maintenance protein, homolog (S. cerevisiae)	1.04	1.06	1.12	0.84
C1QTNF9B-AS	C1QTNF9B-AS1;PCOTH	C1QTNF9B antisense RNA 1 (non-protein coding)	1.03	1.16	1.12	0.79
P2RX6	P2RX6;MGC129625;P2RXL1;P2X6;P2XM	purinergic receptor P2X, ligand-gated ion channel, 6	1.02	1.21	1.12	0.94
HSD17B12	HSD17B12;KAR;SDR12C1	hydroxysteroid (17-beta) dehydrogenase 12	1.02	1.04	1.12	0.92
CT45A3	CT45A3;CT45-3;CT45.3;RP13-36C9.1	cancer/testis antigen family 45, member A3	1.02	1.16	1.12	0.92
PTGDR	PTGDR;AS1;ASRT1;DP;DP1;MGC49004;PTGDR1	prostaglandin D2 receptor (DP)	1	1.04	1.12	0.79
OLR1	OLR1;CLEC8A;LOX1;LOXIN;SCARE1;SLOX1	oxidized low density lipoprotein (lectin-like) receptor 1	1	1.08	1.12	0.78
KCNE4	KCNE4;MGC20353;MIRP3	potassium voltage-gated channel, Isk-related family, member 4	1	1.11	1.12	0.87
ROR2	ROR2;BDB;BDB1;MGC163394;NTRKR2	receptor tyrosine kinase-like orphan receptor 2	1	1.1	1.12	0.92
CENPL	CENPL;C1orf155;CENP-L;FLJ31044;FLJ31786;RP3-383J4.1;dJ383J4.3	centromere protein L	1	1.18	1.12	0.91
ZNF148	ZNF148;BERF-1;BFCOL1;DKFZp686L18248;HT-BETA;ZBP-89;ZFP148;pHZ-52	zinc finger protein 148	1	1.08	1.12	0.93
PPP1R14B	PPP1R14B;PHI-1;PLCB3N;PNG;SOM172	protein phosphatase 1, regulatory (inhibitor) subunit 14B	1	1.04	1.12	0.78
PTPN13	PTPN13;DKFZp686J1497;FAP-1;PNP1;PTP-BAS;PTP-BL;PTP1E;PTPL1;PTPLE;hPTP1E	protein tyrosine phosphatase, non-receptor type 13 (APO-1/CD95 (Fas)-associated phosphatase)	0.99	1.02	1.12	0.77
RBM18	RBM18;MGC2734	RNA binding motif protein 18	0.99	1.14	1.12	0.86
SMAD5	SMAD5;DKFZp781C1895;DKFZp781O1323;DWFC;JV5-1;MADH5	SMAD family member 5	0.99	1.07	1.12	0.76
PGK1	PGK1;MGC117307;MGC142128;MGC8947;MIG10;PGKA	phosphoglycerate kinase 1	0.99	1.08	1.12	0.97
ELL3	ELL3;FLJ22637	elongation factor RNA polymerase II-like 3	0.98	1.06	1.12	0.83
RARRES3	RARRES3;HRASLS4;MGC8906;PLA1/2-3;RIG1;TIG3	retinoic acid receptor responder (tazarotene induced) 3	0.98	1.09	1.12	0.81
UNC119B	UNC119B;MGC5139;POC7B	unc-119 homolog B (C. elegans)	0.98	0.98	1.12	0.78
ME2	ME2;ODS1	malic enzyme 2, NAD(+)-dependent, mitochondrial	0.98	1.04	1.12	0.81
USO1	USO1;P115;TAP;VDP	USO1 vesicle docking protein homolog (yeast)	0.98	1.1	1.12	0.77
PAM16	PAM16;MAGMAS;TIM16;TIMM16	presequence translocase-associated motor 16 homolog (S. cerevisiae)	0.98	1.02	1.12	0.87
ZFYVE9	ZFYVE9;MADHIP;NSP;SARA;SMADIP	zinc finger, FYVE domain containing 9	0.97	1	1.12	0.75
FAM20C	FAM20C;DKFZp547C074;DMP4;RNS	family with sequence similarity 20, member C	0.97	0.99	1.12	0.79
TUBG2	TUBG2;MGC131994	tubulin, gamma 2	0.97	1.06	1.12	0.88
LRBA	LRBA;BGL;CDC4L;DKFZp686A09128;DKFZp686K03100;DKFZp686P2258;FLJ16600;FLJ25686;LAB300;LBA;MGC72098	LPS-responsive vesicle trafficking, beach and anchor containing	0.97	1.06	1.12	0.88
GLRA1	GLRA1;MGC138878;MGC138879;STHE	glycine receptor, alpha 1	0.96	1.07	1.12	0.84
ZFAND2A	ZFAND2A;AIRAP	zinc finger, AN1-type domain 2A	0.96	1.17	1.12	0.78
C1orf116	C1orf116;DKFZp666H2010;FLJ36507;MGC2742;MGC4309;SARG	chromosome 1 open reading frame 116	0.94	0.94	1.12	0.73
MMD2	MMD2;FLJ37205;PAQR10	monocyte to macrophage differentiation-associated 2	0.94	1.02	1.12	0.78
RPS6KA5	RPS6KA5;MGC1911;MSK1;MSPK1;RLPK	ribosomal protein S6 kinase, 90kDa, polypeptide 5	0.94	1.14	1.12	0.81
LARP4	LARP4;DKFZp686E039;MGC74631	La ribonucleoprotein domain family, member 4	0.94	1.02	1.12	0.78
SSB	SSB;LARP3;La	Sjogren syndrome antigen B (autoantigen La)	0.94	1.01	1.12	0.78
FRYL	FRYL;DKFZp686E205;FLJ16177;KIAA0826	FRY-like	0.93	0.99	1.12	0.8
CENPO	CENPO;CENP-O;ICEN-36;MCM21R;MGC11266	centromere protein O	0.93	1.07	1.12	0.8
RB1	RB1;OSRC;RB;p105-Rb;pRb;pp110	retinoblastoma 1	0.93	1	1.12	0.72
TMPO	TMPO;CMD1T;LAP2;LEMD4;MGC61508;PRO0868;TP	thymopoietin	0.93	1.05	1.12	0.72
ACE	ACE;ACE1;CD143;DCP;DCP1;MGC26566;MVCD3	angiotensin I converting enzyme (peptidyl-dipeptidase A) 1	0.92	1.03	1.12	0.77
WT1	WT1;AWT1;EWS-WT1;GUD;NPHS4;WAGR;WIT-2;WT33	Wilms tumor 1	0.92	1.04	1.12	0.83
DIRAS1	DIRAS1;Di-Ras1;FLJ42681;GBTS1;RIG	DIRAS family, GTP-binding RAS-like 1	0.92	1	1.12	0.86
DDX3Y	DDX3Y;DBY	DEAD (Asp-Glu-Ala-Asp) box polypeptide 3, Y-linked	0.91	0.98	1.12	0.7
LRCH1	LRCH1;CHDC1;FLJ41660;KIAA1016;NP81	leucine-rich repeats and calponin homology (CH) domain containing 1	0.91	1.04	1.12	0.79
CTSF	CTSF;CATSF	cathepsin F	0.91	0.94	1.12	0.71
APOOL	APOOL;CXorf33;FAM121A;MGC129748;UNQ8193	apolipoprotein O-like	0.91	1.06	1.12	0.76
E2F7	E2F7;FLJ12981	E2F transcription factor 7	0.91	1.06	1.12	0.79
GPR107	GPR107;DKFZp667C222;FLJ16312;FLJ20998;FLJ22591;GCDRP;KIAA1624;LUSTR1;MGC126118;MGC15440;RP11-88G17;bA138E2.2	G protein-coupled receptor 107	0.91	0.94	1.12	0.71
LTF	LTF;GIG12;HLF2;LF	lactotransferrin	0.9	1	1.12	0.75
C7orf41	C7orf41;Ells1;FLJ25903	chromosome 7 open reading frame 41	0.9	1.01	1.12	0.73
ZNF674	ZNF674;DKFZp686H0940;MRX92;ZNF673B	zinc finger protein 674	0.9	0.91	1.12	0.72
XPC	XPC;RAD4;XP3;XPCC	xeroderma pigmentosum, complementation group C	0.9	0.94	1.12	0.83
ADPGK	ADPGK;2610017G09Rik;ADP-GK;DKFZp434B195	ADP-dependent glucokinase	0.9	1.01	1.12	0.83
FIGNL1	FIGNL1	fidgetin-like 1	0.9	1.04	1.12	0.7
SCAI	SCAI;C9orf126;FLJ11758;FLJ36664;MGC120525;MGC120526;MGC120528;NET40	suppressor of cancer cell invasion	0.9	0.99	1.12	0.83
TNF	TNF;DIF;TNF-alpha;TNFA;TNFSF2	tumor necrosis factor	0.89	1.06	1.12	0.8
RHBDL1	RHBDL1;RHBDL;RRP	rhomboid, veinlet-like 1 (Drosophila)	0.89	0.98	1.12	0.73
B3GNT3	B3GNT3;B3GAL-T8;B3GN-T3;B3GNT-3;HP10328;TMEM3;beta3Gn-T3	UDP-GlcNAc:betaGal beta-1,3-N-acetylglucosaminyltransferase 3	0.89	0.92	1.12	0.81
HTR1A	HTR1A;5-HT1A;5HT1a;ADRB2RL1;ADRBRL1	5-hydroxytryptamine (serotonin) receptor 1A	0.88	0.91	1.12	0.69
APOD	APOD	apolipoprotein D	0.88	0.91	1.12	0.81
SEBOX	SEBOX;OG-9;OG9;OG9X	SEBOX homeobox	0.88	0.9	1.12	0.72
LY6G5C	LY6G5C;C6orf20;G5C;LY6G5CA;LY6G5CB;NG33	lymphocyte antigen 6 complex, locus G5C	0.88	1.01	1.12	0.69
ABHD6	ABHD6	abhydrolase domain containing 6	0.88	1	1.12	0.79
SPTY2D1	SPTY2D1;DKFZp686F1942;DKFZp686I068;FLJ39441	SPT2, Suppressor of Ty, domain containing 1 (S. cerevisiae)	0.88	0.99	1.12	0.71
PLCB3	PLCB3;FLJ37084	phospholipase C, beta 3 (phosphatidylinositol-specific)	0.88	1	1.12	0.67
SRRT	SRRT;ARS2;ASR2;MGC126427;serrate	serrate RNA effector molecule homolog (Arabidopsis)	0.88	1.04	1.12	0.73
ING5	ING5;FLJ23842;p28ING5	inhibitor of growth family, member 5	0.87	0.87	1.12	0.73
UBD	UBD;FAT10;GABBR1;UBD-3	ubiquitin D	0.86	0.9	1.12	0.71
CBLN3	CBLN3;PRO1486	cerebellin 3 precursor	0.86	1.02	1.12	0.79
PCDH18	PCDH18;DKFZp434B0923;KIAA1562;PCDH68L	protocadherin 18	0.86	0.96	1.12	0.67
PDGFRL	PDGFRL;PDGRL;PRLTS	platelet-derived growth factor receptor-like	0.86	0.96	1.12	0.8
KIAA1826	KIAA1826	KIAA1826	0.86	1	1.12	0.72
STK3	STK3;FLJ90748;KRS1;MST2	serine/threonine kinase 3	0.86	0.96	1.12	0.8
SEC23IP	SEC23IP;P125;P125A	SEC23 interacting protein	0.86	0.96	1.12	0.67

POC1A	POC1A;DKFZp434C245;MGC131902;PIX2;WDR51A	POC1 centriolar protein homolog A (Chlamydomonas)	0.86	0.92	1.12	0.7
TRAF5	TRAF5;MGC:39780;RNF84	TNF receptor-associated factor 5	0.85	0.94	1.12	0.85
CENPC1	CENPC1;CENP-C;CENPC;MIF2;hcp-4	centromere protein C 1	0.85	1	1.12	0.67
ST13	ST13;AAG2;FAM10A1;FAM10A4;FLJ27260;HIP;HOP;HSPABP;HSPABP1;MGC129952;P48;PRO0786;SNC6	suppression of tumorigenicity 13 (colon carcinoma) (Hsp70 interacting protein)	0.85	0.9	1.12	0.71
SLC9A3R2	SLC9A3R2;E3KARP;MGC104639;NHE3RF2;NHERF-2;NHERF2;OCTS2;SIP-1;SIP1;TKA-1	solute carrier family 9 (sodium/hydrogen exchanger), member 3 regulator 2	1.21	1.31	1.13	0.94
ZNF239	ZNF239;HOK-2;MOK2	zinc finger protein 239	1.18	1.31	1.13	1.09
LILRB2	LILRB2;CD85D;ILT4;LILRA6;LIR-2;LIR2;MIR-10;MIR10	leukocyte immunoglobulin-like receptor, subfamily B (with TM and ITIM domains), member 2	1.16	1.19	1.13	0.95
CPLX2	CPLX2;921-L;CPX-2;CPX2;Hfb1;MGC138492	complexin 2	1.14	1.33	1.13	0.91
GRM7	GRM7;FLJ40498;GLUR7;GPRC1G;MGLU7;MGLUR7	glutamate receptor, metabotropic 7	1.12	1.17	1.13	0.88
TRIM40	TRIM40;RNF35	tripartite motif containing 40	1.09	1.17	1.13	0.85
CPE	CPE	carboxypeptidase E	1.08	1.21	1.13	0.91
GTF2F2	GTF2F2;BTF4;RAP30;TF2F2;TFIIF	general transcription factor IIF, polypeptide 2, 30kDa	1.07	1.12	1.13	1
ZNF236	ZNF236;ZNF236A;ZNF236B	zinc finger protein 236	1.06	1.07	1.13	0.9
INPP5K	INPP5K;PPS;SKIP	inositol polyphosphate-5-phosphatase K	1.06	1.19	1.13	0.93
KRTAP3-2	KRTAP3-2;KAP3.2;KRTAP3.2	keratin associated protein 3-2	1.05	1.23	1.13	0.93
CNGA2	CNGA2;CNCA;CNCA1;CNG2;FLJ46312;OCNC1;OCNCALPHA;OCNCa	cyclic nucleotide gated channel alpha 2	1.05	1.18	1.13	0.92
PCDHB10	PCDHB10;PCDH-BETA10;PCHB10	protocadherin beta 10	1.04	1.06	1.13	0.88
DST	DST;BP240;BPA;BPAG1;CATX-15;CATX15;D6S1101;DKFZp564B2416;DMH;DT;FLJ46791;KIAA0465;KIAA1470;MACF2	dystonin	1.04	1.06	1.13	0.85
BOD1L	BOD1L;FAM44A;FLJ33215;KIAA1327	biorientation of chromosomes in cell division 1-like	1.04	1.1	1.13	0.9
CCDC146	CCDC146;KIAA1505	coiled-coil domain containing 146	1.03	1.14	1.13	0.9
USP39	USP39;HSPC332;MGC75069;SAD1;SNRNP65	ubiquitin specific peptidase 39	1.03	1.07	1.13	0.81
CBLL1	CBLL1;FLJ23109;HAKAI;MGC163401;MGC163403;RNF188	Cas-Br-M (murine) ecotropic retroviral transforming sequence-like 1	1.02	1.15	1.13	1.03
ACAP3	ACAP3;CENTB5;KIAA1716	ArfGAP with coiled-coil, ankyrin repeat and PH domains 3	1.02	1.09	1.13	0.92
EPPK1	EPPK1;EPIPL;EPIPL1	epiplakin 1	1.01	1.1	1.13	0.87
NUDT14	NUDT14;UGPP;UGPPase	nudix (nucleoside diphosphate linked moiety X)-type motif 14	1	1.05	1.13	0.79
COQ10B	COQ10B;FLJ13448	coenzyme Q10 homolog B (S. cerevisiae)	1	1.18	1.13	0.84
TOP2B	TOP2B;TOPIIB;top2beta	topoisomerase (DNA) II beta 180kDa	1	1.07	1.13	0.84
SEC31A	SEC31A;ABP125;ABP130;DKFZp686N07171;HSPC334;KIAA0905;MGC90305;SEC31L1	SEC31 homolog A (S. cerevisiae)	1	1.1	1.13	0.83
RNF13	RNF13;FLJ93817;MGC13689;RZF	ring finger protein 13	0.99	1.1	1.13	0.99
WDR66	WDR66;FLJ39783;MGC33630	WD repeat domain 66	0.98	1.18	1.13	0.82
PSMC3IP	PSMC3IP;GT198;HOP2;HUMGT198A;ODG3;TBPIP	PSMC3 interacting protein	0.98	1.14	1.13	0.85
SLC39A14	SLC39A14;KIAA0062;LZT-Hs4;NET34;ZIP14;cig19	solute carrier family 39 (zinc transporter), member 14	0.98	1.14	1.13	0.79
MEF2C	MEF2C;C5DELq14.3; DEL5q14.3	myocyte enhancer factor 2C	0.97	1.11	1.13	0.88
FAM126A	FAM126A;DRCTNNB1A;HCC;HLD5;HYCC1	family with sequence similarity 126, member A	0.97	1.06	1.13	0.81
40787	40787	septin 11	0.97	1.12	1.13	0.88
OR9G4	OR9G4;OR11-216	olfactory receptor, family 9, subfamily G, member 4	0.96	1.14	1.13	0.85
C20orf177	C20orf177;dJ551D2.5	chromosome 20 open reading frame 177	0.96	1.05	1.13	0.8
BNIP3L	BNIP3L;BNIP3a;NIX	BCL2/adenovirus E1B 19kDa interacting protein 3-like	0.96	1.05	1.13	0.82
IPO5	IPO5;DKFZp686O1576;FLJ43041;IMB3;KPNB3;MGC2068;RANBP5;imp5	importin 5	0.96	1	1.13	0.88
LYPD6B	LYPD6B;CT116	LY6/PLAUR domain containing 6B	0.95	1.02	1.13	0.86
TGFBRAP1	TGFBRAP1;TRAP-1;TRAP1	transforming growth factor, beta receptor associated protein 1	0.95	1.04	1.13	0.74
CAPZA1	CAPZA1;CAPPA1;CAPZ;CAZ1	capping protein (actin filament) muscle Z-line, alpha 1	0.95	1.02	1.13	0.79
BAZ2A	BAZ2A;DKFZp781B109;FLJ13768;FLJ13780;FLJ45876;KIAA0314;TIP5;WALp3	bromodomain adjacent to zinc finger domain, 2A	0.95	1.12	1.13	0.84
UCHL5	UCHL5;CGI-70;INO80R;UCH-L5;UCH37	ubiquitin carboxyl-terminal hydrolase L5	0.95	1.06	1.13	0.8
S100A8	S100A8;60B8AG;CAGA;CFAG;CGLA;CP-10;L1Ag;MA387;MIF;MRP8;NIF;P8	S100 calcium binding protein A8	0.94	1.15	1.13	0.85
CRP	CRP;MGC149895;MGC88244;PTX1	C-reactive protein, pentraxin-related	0.94	1.04	1.13	0.81
MMP3	MMP3;CHDS6;MGC126102;MGC126103;MGC126104;MMP-3;SL-1;STMY;STMY1;STR1	matrix metallopeptidase 3 (stromelysin 1, progelatinase)	0.94	1.06	1.13	0.79
CHRM4	CHRM4;HM4;M4R	cholinergic receptor, muscarinic 4	0.94	0.99	1.13	0.74
MRPS18A	MRPS18A;FLJ10548;HumanS18b;MRP-S18-3;MRPS18-3;S18bmt	mitochondrial ribosomal protein S18A	0.94	1	1.13	0.84
XG	XG;MGC118758;MGC118759;MGC118760;MGC118761;PBDX	Xg blood group	0.93	1.02	1.13	0.78
BCL2L2	BCL2L2;BCL-W;BCL2-L-2;BCLW;KIAA0271;PPP1R51	BCL2-like 2	0.93	1.12	1.13	0.74
FRMPD2	FRMPD2;MGC178184;MGC178187;MGC35285;MGC87776;MGC87777;MGC90186;PDZD5C;PDZK4;PDZK5C	FERM and PDZ domain containing 2	0.92	1.07	1.13	0.81
HEATR4	HEATR4;MGC48595	HEAT repeat containing 4	0.92	0.99	1.13	0.8
CDH13	CDH13;CDHH;P105	cadherin 13, H-cadherin (heart)	0.92	1.08	1.13	0.81
C1GALT1C1	C1GALT1C1;C1GALT2;C38H2-L1;COSMC;MGC19947;MST143	C1GALT1-specific chaperone 1	0.92	1.12	1.13	0.75
MCF2L2	MCF2L2;ARHGEF22;DKFZp686H2395;DKFZp686K0690;FLJ42509;KIAA0861	MCF.2 cell line derived transforming sequence-like 2	0.91	1.02	1.13	0.75
CD247	CD247;CD3-ZETA;CD3H;CD3Q;CD3Z;T3Z;TCRZ	CD247 molecule	0.91	1.09	1.13	0.72
PHLPP2	PHLPP2;KIAA0931;PHLPPL	PH domain and leucine rich repeat protein phosphatase 2	0.91	0.96	1.13	0.84
WDR65	WDR65;FLJ32000;VWS2	WD repeat domain 65	0.9	1.08	1.13	0.83
CA8	CA8;CA-VIII;CALS;CAMRQ3;CARP;MGC120502;MGC99509	carbonic anhydrase VIII	0.9	0.98	1.13	0.71
BDKRB1	BDKRB1;B1BKR;B1R;BKB1R;BKR1;BRADYB1	bradykinin receptor B1	0.9	0.96	1.13	0.72
ZNF100	ZNF100;FLJ44587	zinc finger protein 100	0.9	1.05	1.13	0.79
AEBP1	AEBP1;ACLP;FLJ33612	AE binding protein 1	0.9	0.94	1.13	0.7
CDH5	CDH5;7B4;CD144;FLJ17376	cadherin 5, type 2 (vascular endothelium)	0.9	0.98	1.13	0.74
STARD3NL	STARD3NL;MENTHO;MGC3251	STARD3 N-terminal like	0.9	0.97	1.13	0.79
PARG	PARG;FLJ54459;FLJ60257;FLJ60456;PARG99	poly (ADP-ribose) glycohydrolase	0.9	0.94	1.13	0.75
PLOD2	PLOD2;LH2;TLH	procollagen-lysine, 2-oxoglutarate 5-dioxygenase 2	0.9	1.09	1.13	0.82
DENR	DENR;DRP;DRP1;SMAP-3	density-regulated protein	0.9	1.07	1.13	0.75
SKP2	SKP2;FBL1;FBXL1;FLB1;MGC1366	S-phase kinase-associated protein 2 (p45)	0.9	0.96	1.13	0.7
ELOVL1	ELOVL1;Ssc1	ELOVL fatty acid elongase 1	0.9	0.98	1.13	0.77
PCDHA5	PCDHA5;CNR6;CNRN6;CNRS6;CRNR6;PCDH-ALPHA5	protocadherin alpha 5	0.9	0.92	1.13	0.77
DAZAP1	DAZAP1;MGC19907	DAZ associated protein 1	0.89	0.99	1.13	0.77
EIF4EBP2	EIF4EBP2;4EBP2;PHASII	eukaryotic translation initiation factor 4E binding protein 2	0.89	1.06	1.13	0.86
GAD1	GAD1;CPSQ1;FLJ45882;GAD;SCP	glutamate decarboxylase 1 (brain, 67kDa)	0.88	0.96	1.13	0.68
EFEMP1	EFEMP1;DHRD;DRAD;FBLN3;FBNL;FLJ35535;MGC111353;MLVT;MTLV;S1-5	EGF containing fibulin-like extracellular matrix protein 1	0.88	0.96	1.13	0.69
SLC39A10	SLC39A10;DKFZp781L10106;LZT-Hs2;MGC126565;MGC138428	solute carrier family 39 (zinc transporter), member 10	0.88	1.03	1.13	0.84
ANXA7	ANXA7;ANX7;SNX;SYNEXIN	annexin A7	0.88	0.99	1.13	0.77
BACE2	BACE2;AEPLC;ALP56;ASP1;ASP21;BAE2;CEAP1;DRAP	beta-site APP-cleaving enzyme 2	0.88	0.98	1.13	0.74
TACO1	TACO1;CCDC44	translational activator of mitochondrially encoded cytochrome c oxidase I	0.88	1	1.13	0.72
SPATA18	SPATA18;FLJ32906;Mieap;SPETEX1	spermatogenesis associated 18 homolog (rat)	0.87	0.97	1.13	0.72
WDR81	WDR81;FLJ23776;FLJ33817	WD repeat domain 81	0.87	1	1.13	0.75
DSN1	DSN1;C20orf172;FLJ13346;KNL3;MGC32987;MIS13;dJ469A13.2;hKNL-3	DSN1, MIND kinetochore complex component, homolog (S. cerevisiae)	0.87	0.96	1.13	0.72
DMRT2	DMRT2	doublesex and mab-3 related transcription factor 2	0.86	0.96	1.13	0.71
MAK	MAK;RP62;dJ417M14.2	male germ cell-associated kinase	0.86	1.04	1.13	0.74
FAM27L	FAM27L;MGC35151	family with sequence similarity 27-like	0.86	0.97	1.13	0.7
PELI3	PELI3;MGC35521	pellino homolog 3 (Drosophila)	0.86	0.92	1.13	0.75
KDELC1	KDELC1;EP58;KDEL1;MGC5302	KDEL (Lys-Asp-Glu-Leu) containing 1	0.86	0.99	1.13	0.78
HOXB4	HOXB4;HOX-2.6;HOX2;HOX2F	homeobox B4	0.86	1	1.13	0.76
TMEM70	TMEM70;FLJ20533;MC5DN2	transmembrane protein 70	0.86	1.01	1.13	0.72
FAM105B	FAM105B;FLJ34884	family with sequence similarity 105, member B	0.86	0.94	1.13	0.72
SETDB1	SETDB1;ESET;H3-K9-HMTase4;KG1T;KIAA0067;KMT1E	SET domain, bifurcated 1	0.86	0.94	1.13	0.68
PRSS8	PRSS8;CAP1;PROSTASIN	protease, serine, 8	0.86	0.96	1.13	0.72
KHDRBS1	KHDRBS1;FLJ34027;Sam68;p62;p68	KH domain containing, RNA binding, signal transduction associated 1	0.86	0.91	1.13	0.8
TAF13	TAF13;MGC22425;TAF(II)18;TAF2K;TAFII-18;TAFII18	TAF13 RNA polymerase II, TATA box binding protein (TBP)-associated factor, 18kDa	0.86	0.99	1.13	0.66
JAG1	JAG1;AGS;AHD;AWS;CD339;HJ1;JAGL1;MGC104644	jagged 1	0.86	1.03	1.13	0.68
COX17	COX17;MGC104397;MGC117386	COX17 cytochrome c oxidase assembly homolog (S. cerevisiae)	0.86	1.02	1.13	0.67
ENO1	ENO1;ENO1L1;MPB1;NNE;PPH	enolase 1, (alpha)	0.86	0.95	1.13	0.67
XKRY2	XKRY2;XKRYP7	XK, Kell blood group complex subunit-related, Y-linked 2	0.86	0.94	1.13	0.74
MIEN1	MIEN1;C17orf37;C35;MGC14832;ORB3;RDX12;XTP4	migration and invasion enhancer 1	0.86	0.92	1.13	0.78
GTF2IRD2	GTF2IRD2;FLJ21423;FLJ37938;FP630;GTF2IRD2A;MGC75203	GTF2I repeat domain containing 2	0.85	0.99	1.13	0.67
CHMP3	CHMP3;FLJ38114;NEDF;VPS24	charged multivesicular body protein 3	1.23	1.35	1.14	1.03
CASP3	CASP3;CPP32;CPP32B;SCA-1	caspase 3, apoptosis-related cysteine peptidase	1.21	1.27	1.14	0.96
HMGXB4	HMGXB4;HMG2L1;HMGBCG;THC211630	HMG box domain containing 4	1.14	1.16	1.14	0.97
ABCB9	ABCB9;EST122234;KIAA1520;TAPL	ATP-binding cassette, sub-family B (MDR/TAP), member 9	1.12	1.24	1.14	0.97
38777	MARCH6;DOA10;KIAA0597;MARCH-VI;RNF176;TEB4	membrane-associated ring finger (C3HC4) 6	1.12	1.22	1.14	0.88
TMEM57	TMEM57;FLJ10747;FLJ23007;MACOILIN	transmembrane protein 57	1.08	1.17	1.14	0.9
MAPK7	MAPK7;BMK1;ERK4;ERK5;PRKM7	mitogen-activated protein kinase 7	1.06	1.2	1.14	0.99
FSTL1	FSTL1;FLJ50214;FLJ52277;FRP;FSL1	follistatin-like 1	1.06	1.16	1.14	0.94
SH3RF1	SH3RF1;FLJ21602;KIAA1494;POSH;RNF142;SH3MD2	SH3 domain containing ring finger 1	1.04	1.17	1.14	1.04
NDUFS2	NDUFS2;CI-49	NADH dehydrogenase (ubiquinone) Fe-S protein 2, 49kDa (NADH-coenzyme Q reductase)	1.04	1.27	1.14	0.83
LRRC17	LRRC17;P37NB	leucine rich repeat containing 17	1.03	1.05	1.14	0.87
UPP2	UPP2;UDRPASE2;UP2;UPASE2	uridine phosphorylase 2	1.03	1.24	1.14	0.82
SH2D4B	SH2D4B;FLJ41984	SH2 domain containing 4B	1.03	1.21	1.14	0.92
C1orf9	C1orf9;CH1;OPT;SLP1	chromosome 1 open reading frame 9	1.03	1.11	1.14	0.94
ISCA1	ISCA1;HBLD2;ISA1;MGC4276;RP11-507D14.2;hIscA	iron-sulfur cluster assembly 1 homolog (S. cerevisiae)	1.03	1.15	1.14	0.86
ZNF774	ZNF774;MGC75360	zinc finger protein 774	1.02	1.19	1.14	0.89
NUS1	NUS1;C6orf68;MGC117249;MGC7199;MGC:7199;NgBR	nuclear undecaprenyl pyrophosphate synthase 1 homolog (S. cerevisiae)	1.02	1.12	1.14	0.86
RCHY1	RCHY1;ARNIP;CHIMP;DKFZp586C1620;PIRH2;PRO1996;RNF199;ZNF363;hARNIP	ring finger and CHY zinc finger domain containing 1	1	1.09	1.14	0.79
FOXK1	FOXK1;FLJ14977;FOXK1L	forkhead box K1	0.99	1.01	1.14	0.8
NOVA1	NOVA1;Nova-1	neuro-oncological ventral antigen 1	0.96	1.12	1.14	0.75
PTPN21	PTPN21;PTPD1;PTPRL10	protein tyrosine phosphatase, non-receptor type 21	0.96	1.02	1.14	0.77
HNRPDL	HNRPDL;HNRNP;JKTBP;JKTBP2;laAUF1	heterogeneous nuclear ribonucleoprotein D-like	0.96	1.04	1.14	0.76
ZNF330	ZNF330;HSA6591;NOA36	zinc finger protein 330	0.96	1.04	1.14	0.75
MAP4K3	MAP4K3;GLK;MAPKKKK3;MEKKK 3;RAB8IPL1	mitogen-activated protein kinase kinase kinase kinase 3	0.96	1.06	1.14	0.96
PRPS2	PRPS2;PRSII	phosphoribosyl pyrophosphate synthetase 2	0.96	1.19	1.14	0.84
SCYL2	SCYL2;CVAK104;FLJ10074;KIAA1360	SCY1-like 2 (S. cerevisiae)	0.96	1.07	1.14	0.8
SSRP1	SSRP1;FACT;FACT80;T160	structure specific recognition protein 1	0.95	1.11	1.14	0.8
APOA1BP	APOA1BP;AIBP;MGC119143;MGC119144;MGC119145;YJEFN1	apolipoprotein A-I binding protein	0.95	1.15	1.14	0.93
PRSS45	PRSS45;TESSP5	protease, serine, 45	0.95	1.09	1.14	0.85

PUS10	PUS10;CCDC139;DOBI;FLJ32312;MGC126729;MGC126755	pseudouridylate synthase 10	0.94	1.1	1.14	0.84
GAMT	GAMT;PIG2;TP53I2	guanidinoacetate N-methyltransferase	0.94	1.02	1.14	0.74
PDCD6	PDCD6;ALG-2;FLJ46208;MGC111017;MGC119050;MGC9123;PEF1B	programmed cell death 6	0.94	1	1.14	0.88
ARMCX3	ARMCX3;ALEX3;DKFZp781N1954;KIAA0443;MGC12199;dJ545K15.2	armadillo repeat containing, X-linked 3	0.94	1.04	1.14	0.87
GDF11	GDF11;BMP-11;BMP11	growth differentiation factor 11	0.93	1.08	1.14	0.86
CCDC63	CCDC63;FLJ35843	coiled-coil domain containing 63	0.92	1.13	1.14	0.79
OR6Q1	OR6Q1;OR11-226	olfactory receptor, family 6, subfamily Q, member 1	0.92	0.98	1.14	0.83
TTLL13	TTLL13;FLJ46079;MGC33417	tubulin tyrosine ligase-like family, member 13	0.92	1	1.14	0.81
CAMK2A	CAMK2A;CAMKA;KIAA0968	calcium/calmodulin-dependent protein kinase II alpha	0.92	1.14	1.14	0.81
MFSD5	MFSD5;MGC11308	major facilitator superfamily domain containing 5	0.92	1	1.14	0.77
LMBR1	LMBR1;ACHP;C7orf2;DIF14;FLJ11665;PPD2;TPT	limb region 1 homolog (mouse)	0.92	1.03	1.14	0.86
ATP2C1	ATP2C1;ATP2C1A;BCPM;HHD;KIAA1347;PMR1;SPCA1;hSPCA1	ATPase, Ca++ transporting, type 2C, member 1	0.92	1	1.14	0.73
ZNF652	ZNF652;DKFZp781E2122;KIAA0924	zinc finger protein 652	0.91	1.04	1.14	0.75
SEMA4C	SEMA4C;FLJ20369;KIAA1739;M-SEMA-F;MGC126382;MGC126383;SEMACL1;SEMAF;SEMAI	sema domain, immunoglobulin domain (Ig), transmembrane domain (TM) and short cytoplasmic domain, (semaphorin) 4C	0.91	0.98	1.14	0.83
MRPS14	MRPS14;DJ262D12.2;HSMRPS14;MRP-S14;S14mt	mitochondrial ribosomal protein S14	0.91	1.08	1.14	0.88
S100A16	S100A16;DT1P1A7;MGC17528;S100F	S100 calcium binding protein A16	0.91	1.01	1.14	0.8
CD48	CD48;BCM1;BLAST;BLAST1;MEM-102;SLAMF2;hCD48;mCD48	CD48 molecule	0.9	1.02	1.14	0.85
KLB	KLB;BKL;MGC142213	klotho beta	0.9	0.95	1.14	0.76
LRRC29	LRRC29;FBL9;FBXL9	leucine rich repeat containing 29	0.9	0.98	1.14	0.71
GDAP1	GDAP1;CMT4;CMT4A;CMTRIA	ganglioside-induced differentiation-associated protein 1	0.9	0.99	1.14	0.71
NUDT15	NUDT15;FLJ10956;MGC104352;MTH2;RP11-90M2.1	nudix (nucleoside diphosphate linked moiety X)-type motif 15	0.9	0.96	1.14	0.72
CNPY2	CNPY2;HP10390;MSAP;TMEM4;ZSIG9	canopy 2 homolog (zebrafish)	0.9	0.98	1.14	0.75
SMC6	SMC6;FLJ22116;FLJ35534;SMC-6;SMC6L1;hSMC6	structural maintenance of chromosomes 6	0.9	0.9	1.14	0.71
SPON1	SPON1;KIAA0762;MGC10724;f-spondin	spondin 1, extracellular matrix protein	0.9	0.93	1.14	0.77
BAGE5	BAGE5;CT2.5	B melanoma antigen family, member 5	0.89	1.06	1.14	0.72
CRYGA	CRYGA;CRY-g-A;CRYG1;CRYG5	crystallin, gamma A	0.88	0.91	1.14	0.76
AOX1	AOX1;AO;AOH1	aldehyde oxidase 1	0.88	0.93	1.14	0.83
ZNF44	ZNF44;DKFZp434F1811;DKFZp686L21136;GIOT-2;KOX7;ZNF;ZNF504;ZNF55;ZNF58	zinc finger protein 44	0.88	0.97	1.14	0.76
C10orf92	C10orf92;C10orf123;C10orf124;DKFZp434A1721;FLJ46273;bA288G11.4;bA288G11.5;bB137A17.2	chromosome 10 open reading frame 92	0.88	1.06	1.14	0.79
MST1	MST1;D3F15S2;DNF15S2;HGFL;MSP;NF15S2	macrophage stimulating 1 (hepatocyte growth factor-like)	0.88	0.98	1.14	0.74
C15orf53	C15orf53;FLJ35695;MGC148119	chromosome 15 open reading frame 53	0.88	1.03	1.14	0.7
ST3GAL5	ST3GAL5;SATI;SIAT9;SIATGM3S;ST3GalV	ST3 beta-galactoside alpha-2,3-sialyltransferase 5	0.88	0.98	1.14	0.84
PIGH	PIGH;GPI-H	phosphatidylinositol glycan anchor biosynthesis, class H	0.88	0.94	1.14	0.82
SCLY	SCLY;SCL;hSCL	selenocysteine lyase	0.88	0.99	1.14	0.7
IRAK4	IRAK4;IPD1;IRAK-4;NY-REN-64;REN64	interleukin-1 receptor-associated kinase 4	0.88	1	1.14	0.83
XPO5	XPO5;FLJ14239;FLJ32057;FLJ45606;KIAA1291;exp5	exportin 5	0.88	0.9	1.14	0.75
DYM	DYM;DMC;FLJ20071;FLJ90130;SMC	dymeclin	0.88	0.97	1.14	0.73
SLC36A2	SLC36A2;FLJ16051;MGC119658;MGC119660;PAT2;TRAMD1	solute carrier family 36 (proton/amino acid symporter), member 2	0.87	0.95	1.14	0.69
ZNF335	ZNF335;NIF-1;NIF1;NIF2	zinc finger protein 335	0.87	0.9	1.14	0.72
COX4I1	COX4I1;COX4;COX4-1;COXIV;FLJ23483;MGC72016	cytochrome c oxidase subunit IV isoform 1	0.87	0.99	1.14	0.77
NCAPH	NCAPH;BRRN1;CAP-H;HCAP-H	non-SMC condensin I complex, subunit H	0.87	1.02	1.14	0.76
OR2A14	OR2A14;OR2A14P;OR2A6;OST182	olfactory receptor, family 2, subfamily A, member 14	0.86	0.88	1.14	0.74
CDH20	CDH20;CDH7L3;Cdh7;FLJ37047	cadherin 20, type 2	0.86	0.88	1.14	0.71
TTBK1	TTBK1;BDTK;KIAA1855;RP3-330M21.4	tau tubulin kinase 1	0.86	0.98	1.14	0.81
GPIHBP1	GPIHBP1;GPI-HBP1	glycosylphosphatidylinositol anchored high density lipoprotein binding protein 1	0.86	0.93	1.14	0.71
COL22A1	COL22A1	collagen, type XXII, alpha 1	0.86	1	1.14	0.69
BTBD10	BTBD10;GMRP-1;GMRP1;MGC13007	BTB (POZ) domain containing 10	0.86	0.98	1.14	0.76
GYG1	GYG1;GSD15;GYG	glycogenin 1	0.86	0.91	1.14	0.72
HELQ	HELQ;HEL308;MGC20604	helicase, POLQ-like	0.86	0.97	1.14	0.72
TSPAN11	TSPAN11;VSSW1971	tetraspanin 11	0.85	1	1.14	0.75
SYNGR2	SYNGR2;MGC102914	synaptogyrin 2	0.85	0.94	1.14	0.72
ARPC5	ARPC5;ARC16;MGC88523;dJ127C7.3;p16-Arc	actin related protein 2/3 complex, subunit 5, 16kDa	0.85	1.02	1.14	0.72
C5orf34	C5orf34;FLJ32363;MGC46448	chromosome 5 open reading frame 34	1.23	1.25	1.15	0.98
CD3E	CD3E;FLJ18683;T3E;TCRE	CD3e molecule, epsilon (CD3-TCR complex)	1.12	1.15	1.15	0.92
GABBR1	GABBR1;FLJ92613;GABABR1;GABBR1-3;GB1;GPRC3A;dJ271M21.1.1;dJ271M21.1.2	gamma-aminobutyric acid (GABA) B receptor, 1	1.12	1.18	1.15	0.89
MKNK2	MKNK2;GPRK7;MNK2	MAP kinase interacting serine/threonine kinase 2	1.12	1.29	1.15	1.07
GPT2	GPT2;ALT2	glutamic pyruvate transaminase (alanine aminotransferase) 2	1.12	1.31	1.15	1.04
ACTR3B	ACTR3B;ARP11;ARP3BETA;DKFZp686O24114	ARP3 actin-related protein 3 homolog B (yeast)	1.1	1.33	1.15	0.95
PIBF1	PIBF1;C13orf24;KIAA1008;PIBF;RP11-505F3.1	progesterone immunomodulatory binding factor 1	1.08	1.25	1.15	0.85
AHRR	AHRR;AHH;AHHR;KIAA1234;MGC167813;MGC176630;bHLHe77	aryl-hydrocarbon receptor repressor	1.08	1.19	1.15	0.91
TBX20	TBX20;ASD4	T-box 20	1.05	1.25	1.15	0.86
PAPD7	PAPD7;LAK-1;LAK1;POLK;POLS;TRF4;TRF4-1;TRF41;TUTASE5	PAP associated domain containing 7	1.05	1.24	1.15	0.98
TTC8	TTC8;BBS8;RP51	tetratricopeptide repeat domain 8	1.02	1.1	1.15	0.93
PHOSPHO1	PHOSPHO1	phosphatase, orphan 1	1.01	1.11	1.15	0.85
IER5L	IER5L;MGC70833;bA247A12.2	immediate early response 5-like	1.01	1.22	1.15	0.87
LY6G5B	LY6G5B;C6orf19;G5b	lymphocyte antigen 6 complex, locus G5B	1.01	1.04	1.15	0.87
MAPK8	MAPK8;JNK;JNK1;JNK1A2;JNK21B1/2;PRKM8;SAPK1	mitogen-activated protein kinase 8	1.01	1.17	1.15	0.96
ANKRD35	ANKRD35;FLJ25124;MGC126667;MGC126669	ankyrin repeat domain 35	1	1.02	1.15	0.8
PRPF39	PRPF39;FLJ11128;FLJ20666;FLJ45460;MGC149842;MGC149843	PRP39 pre-mRNA processing factor 39 homolog (S. cerevisiae)	1	1.16	1.15	0.8
GABRA1	GABRA1;ECA4;EJM;EJM5	gamma-aminobutyric acid (GABA) A receptor, alpha 1	0.99	1.13	1.15	0.88
RLN1	RLN1;H1;RLXH1;bA12D24.3.1;bA12D24.3.2	relaxin 1	0.99	1.12	1.15	0.93
UQCRC1	UQCRC1;D3S3191;QCR1;UQCR1	ubiquinol-cytochrome c reductase core protein I	0.99	1.05	1.15	0.79
ZNF568	ZNF568;DKFZp667O0524;DKFZp686B0797;FLJ34820;FLJ35804	zinc finger protein 568	0.98	1	1.15	0.84
MAP3K14	MAP3K14;FTDCR1B;HS;HSNIK;NIK	mitogen-activated protein kinase kinase kinase 14	0.98	1.12	1.15	0.93
PPP2R2B	PPP2R2B;B55BETA;FLJ95686;MGC24888;PP2AB55BETA;PP2ABBETA;PP2APR55B;PP2APR55BETA;PR2AB55BETA;PR2ABBETA;PR2APR55BETA;PR52B;PR55-BETA;PR55BETA;SCA12	protein phosphatase 2, regulatory subunit B, beta	0.97	1.04	1.15	0.78
VWF	VWF;F8VWF;VWD	von Willebrand factor	0.97	1.19	1.15	0.78
DDX27	DDX27;DKFZp667N057;DRS1;FLJ12917;FLJ20596;FLJ22238;MGC1018;MGC163147;PP3241;RHLP;Rrp3p;dJ686N3.1	DEAD (Asp-Glu-Ala-Asp) box polypeptide 27	0.97	1.04	1.15	0.83
GAS2L2	GAS2L2;GAR17	growth arrest-specific 2 like 2	0.96	1.13	1.15	0.84
ATP6V0E2	ATP6V0E2;ATP6V0E2L;C7orf32	ATPase, H+ transporting V0 subunit e2	0.96	1.06	1.15	0.86
SRSF8	SRSF8;SFRS2B;SRP46	serine/arginine-rich splicing factor 8	0.96	1.08	1.15	0.81
FTCD	FTCD;LCHC1	formiminotransferase cyclodeaminase	0.95	1.05	1.15	0.76
GALM	GALM;IBD1	galactose mutarotase (aldose 1-epimerase)	0.95	1.05	1.15	0.76
NEFM	NEFM;NEF3;NF-M;NFM	neurofilament, medium polypeptide	0.94	1.17	1.15	0.81
HECW1	HECW1;KIAA0322;NEDL1	HECT, C2 and WW domain containing E3 ubiquitin protein ligase 1	0.94	1	1.15	0.75
DOCK8	DOCK8;FLJ00026;FLJ00152;FLJ00346;MRD2;ZIR8	dedicator of cytokinesis 8	0.94	1.03	1.15	0.79
OXER1	OXER1;GPCR;GPR170;TG1019	oxoeicosanoid (OXE) receptor 1	0.94	1.05	1.15	0.95
CEP170	CEP170;FAM68A;KAB;KIAA0470	centrosomal protein 170kDa	0.94	1.05	1.15	0.79
ALDH1L2	ALDH1L2;DKFZp686A16126;DKFZp686M064;DKFZp686P14145;FLJ36769;FLJ38508;MGC119536;MGC119537;mtFDH	aldehyde dehydrogenase 1 family, member L2	0.94	1.16	1.15	0.87
PNRC1	PNRC1;B4-2;PNAS-145;PROL2;PRR2	proline-rich nuclear receptor coactivator 1	0.94	1.04	1.15	0.75
GAB1	GAB1	GRB2-associated binding protein 1	0.93	1.02	1.15	0.74
STX3	STX3;FLJ30906;STX3A	syntaxin 3	0.93	1.14	1.15	0.75
SPPL3	SPPL3;DKFZp586C1324;IMP2;MDHV1887;MGC126674;MGC126676;MGC90402;PRO4332;PSL4	signal peptide peptidase-like 3	0.93	1.06	1.15	0.8
IYD	IYD;C6orf71;DEHAL1;TDH4;dJ422F24.1	iodotyrosine deiodinase	0.92	1.03	1.15	0.73
MT1H	MT1H;MGC70702;MT-0;MT-1H;MT-IH;MT1	metallothionein 1H	0.92	1.08	1.15	0.75
ABHD14A	ABHD14A;DKFZp564O243;DORZ1;FLJ60317;FLJ60467	abhydrolase domain containing 14A	0.92	0.98	1.15	0.78
VPS72	VPS72;CFL1;Swc2;TCFL1;YL-1;YL1	vacuolar protein sorting 72 homolog (S. cerevisiae)	0.92	1.04	1.15	0.73
NPTX2	NPTX2;NARP;NP-II;NP2	neuronal pentraxin II	0.92	1.02	1.15	0.78
TSPAN7	TSPAN7;A15;CCG-B7;CD231;DXS1692E;MRX58;MXS1;TALLA-1;TM4SF2;TM4SF2b	tetraspanin 7	0.91	1.07	1.15	0.8
TMEM232	TMEM232;DKFZp686G0585;FLJ35903;FLJ43080	transmembrane protein 232	0.91	1.05	1.15	0.71
LACRT	LACRT;MGC71934	lacritin	0.9	1.06	1.15	0.81
NCOA2	NCOA2;GRIP1;KAT13C;MGC138808;NCoA-2;SRC2;TIF2;bHLHe75	nuclear receptor coactivator 2	0.9	0.96	1.15	0.76
PDCD2L	PDCD2L;MGC13096	programmed cell death 2-like	0.9	0.92	1.15	0.72
SFXN1	SFXN1;FLJ12876	sideroflexin 1	0.9	1.05	1.15	0.83
ZMPSTE24	ZMPSTE24;FACE-1;FACE1;FLJ14968;HGPS;PRO1;STE24;Ste24p	zinc metallopeptidase (STE24 homolog, S. cerevisiae)	0.9	1.03	1.15	0.77
ZNF804A	ZNF804A;C2orf10	zinc finger protein 804A	0.89	1.01	1.15	0.75
KCNQ5	KCNQ5;Kv7.5	potassium voltage-gated channel, KQT-like subfamily, member 5	0.89	1.02	1.15	0.79
TRIM49	TRIM49;RNF18;TRIM49L2	tripartite motif containing 49	0.88	1	1.15	0.9
PLCH1	PLCH1;DKFZp434C1372;MGC117152;PLCL3	phospholipase C, eta 1	0.88	0.98	1.15	0.72
C19orf46	C19orf46;FLJ36445	chromosome 19 open reading frame 46	0.88	0.98	1.15	0.75
SIGIRR	SIGIRR;MGC110992;TIR8	single immunoglobulin and toll-interleukin 1 receptor (TIR) domain	0.88	0.99	1.15	0.8
SUSD1	SUSD1	sushi domain containing 1	0.88	0.95	1.15	0.8
GCC1	GCC1;FLJ22035;GCC1P;GCC88;MGC20706	GRIP and coiled-coil domain containing 1	0.88	0.92	1.15	0.76
HS6ST1	HS6ST1;DKFZp547H098;FLJ25392;HS6ST;MGC116899;MGC116901	heparan sulfate 6-O-sulfotransferase 1	0.88	1	1.15	0.7
RNF26	RNF26;MGC2642	ring finger protein 26	0.88	0.99	1.15	0.89
PYGB	PYGB;MGC9213	phosphorylase, glycogen; brain	0.88	0.99	1.15	0.7
KIAA1683	KIAA1683;MGC131731	KIAA1683	0.87	0.99	1.15	0.82
POPDC3	POPDC3;MGC22671;POP3;RP11-99L11.2;bA355M14.1	popeye domain containing 3	0.87	1.02	1.15	0.78
THEM5	THEM5;FLJ37964;MGC126659;MGC138444	thioesterase superfamily member 5	0.86	0.88	1.15	0.69
SCARA5	SCARA5;FLJ23907;MGC45780;NET33;Tesr	scavenger receptor class A, member 5 (putative)	0.86	0.94	1.15	0.72
FSCN2	FSCN2;RFSN;RP30	fascin homolog 2, actin-bundling protein, retinal (Strongylocentrotus purpuratus)	0.86	0.9	1.15	0.71
FAM105A	FAM105A;FLJ11127;FLJ43660;NET20	family with sequence similarity 105, member A	0.86	1.08	1.15	0.72
ZKSCAN3	ZKSCAN3;FLJ33906;KIAA0426;ZF47;ZFP306;ZNF306;ZNF309;ZSCAN13;Zfp47;dJ874C20.1;dJ874C20.1.;zfp-47	zinc finger with KRAB and SCAN domains 3	0.86	0.98	1.15	0.73
MARS2	MARS2;MetRS;mtMetRS	methionyl-tRNA synthetase 2, mitochondrial	0.86	0.99	1.15	0.77
SP3	SP3;DKFZp686O1631;SPR2	Sp3 transcription factor	0.86	1	1.15	0.77
TNFAIP8L2	TNFAIP8L2;FLJ23467;TIPE2	tumor necrosis factor, alpha-induced protein 8-like 2	0.85	0.95	1.15	0.72
TAS2R5	TAS2R5;MGC126635;MGC126637;T2R5	taste receptor, type 2, member 5	0.85	0.94	1.15	0.72
PIGR	PIGR;FLJ22667;MGC125361;MGC125362	polymeric immunoglobulin receptor	0.85	1.04	1.15	0.69
ACAP1	ACAP1;CENTB1;KIAA0050	ArfGAP with coiled-coil, ankyrin repeat and PH domains 1	0.85	1.02	1.15	0.68
FPR1	FPR1;FMLP;FPR	formyl peptide receptor 1	1.27	1.37	1.16	1.03

HCRT	HCRT;NRCLP1;OX;PPOX	hypocretin (orexin) neuropeptide precursor	1.2	1.39	1.16	0.94
PHF5A	PHF5A;INI;MGC1346;Rds3;SAP14b;SF3b14b;bK223H9.2	PHD finger protein 5A	1.12	1.25	1.16	0.9
ABCG2	ABCG2;ABC15;ABCP;BCRP;BCRP1;BMDP;CD338;CDw338;EST157481;MGC102821;MRX;MXR;MXR1	ATP-binding cassette, sub-family G (WHITE), member 2	1.1	1.23	1.16	0.89
OR52D1	OR52D1;OR11-43	olfactory receptor, family 52, subfamily D, member 1	1.08	1.31	1.16	0.87
LONRF1	LONRF1;FLJ23749;RNF191	LON peptidase N-terminal domain and ring finger 1	1.08	1.21	1.16	0.87
NEK7	NEK7	NIMA (never in mitosis gene a)-related kinase 7	1.08	1.22	1.16	1.1
CCDC81	CCDC81;FLJ16339;FLJ23514	coiled-coil domain containing 81	1.07	1.11	1.16	0.87
CILP	CILP;CILP-1;HsT18872	cartilage intermediate layer protein, nucleotide pyrophosphohydrolase	1.07	1.16	1.16	0.87
CHFR	CHFR;FLJ10796;FLJ33629;RNF116;RNF196	checkpoint with forkhead and ring finger domains	1.07	1.22	1.16	1.1
ACPT	ACPT	acid phosphatase, testicular	1.06	1.22	1.16	0.95
TRIM62	TRIM62;DEAR1;FLJ10759;FLJ16558	tripartite motif containing 62	1.05	1.17	1.16	0.84
NADSYN1	NADSYN1;FLJ10631;FLJ36703;FLJ40627	NAD synthetase 1	1.04	1.19	1.16	0.84
EPN3	EPN3;FLJ20778;MGC129899	epsin 3	1.01	1.19	1.16	0.91
TRIM41	TRIM41;MGC1127;MGC31991;RINCK	tripartite motif containing 41	1.01	1.12	1.16	0.81
COL5A2	COL5A2;MGC105115	collagen, type V, alpha 2	1	1.17	1.16	0.81
TMPRSS2	TMPRSS2;FLJ41954;PP9284;PRSS10	transmembrane protease, serine 2	1	1.08	1.16	0.81
ZNF323	ZNF323;FLJ23407;FLJ41286;ZNF20-Lp;ZNF310P;dJ874C20.2	zinc finger protein 323	1	1.17	1.16	0.79
UCKL1	UCKL1;UCK1-LIKE;UCK1L;URKL1	uridine-cytidine kinase 1-like 1	1	1.16	1.16	0.96
CYC1	CYC1;UQCR4	cytochrome c-1	0.99	1.14	1.16	0.85
FAM65C	FAM65C;C20orf175;FLJ00360;FLJ32230;dJ530I15.2	family with sequence similarity 65, member C	0.98	1.11	1.16	0.87
INTS9	INTS9;CPSF2L;FLJ10871;INT9;RC74	integrator complex subunit 9	0.98	1.17	1.16	0.87
TOR1AIP2	TOR1AIP2;FLJ77012;IFRG15;LULL1;MGC120074;MGC120075;MGC120076;MGC120077;MGC126581;MGC138430;NET9;RP11-12M5.5	torsin A interacting protein 2	0.98	1.16	1.16	0.87
USMG5	USMG5;DAPIT;DKFZp566D211;HCVFTP2;MGC14697;bA792D24.4	up-regulated during skeletal muscle growth 5 homolog (mouse)	0.97	1.13	1.16	0.77
PLXNA4	PLXNA4;DKFZp434G0625;DKFZp566O0546;FAYV2820;FLJ35026;FLJ38287;KIAA1550;PLEXA4;PLXNA4A;PLXNA4B;PRO34003	plexin A4	0.96	1.17	1.16	0.84
RAB40B	RAB40B;FLJ42385;RAR;SEC4L	RAB40B, member RAS oncogene family	0.96	1.07	1.16	0.77
CXorf48	CXorf48;BJHCC20A;CT55;FLJ20527	chromosome X open reading frame 48	0.95	1.06	1.16	0.81
IL1A	IL1A;IL-1A;IL1;IL1-ALPHA;IL1F1	interleukin 1, alpha	0.94	1.04	1.16	0.77
FAM64A	FAM64A;FLJ10156;FLJ10491;RCS1	family with sequence similarity 64, member A	0.94	1.06	1.16	0.89
TPCN1	TPCN1;FLJ20612;KIAA1169;TPC1	two pore segment channel 1	0.94	1.08	1.16	0.84
TECPR1	TECPR1;DKFZP434B0335;FLJ23419;FLJ90593	tectonin beta-propeller repeat containing 1	0.94	1.11	1.16	0.81
C11orf1	C11orf1;FLJ23499	chromosome 11 open reading frame 1	0.93	0.98	1.16	0.78
BRIP1	BRIP1;BACH1;FANCJ;FLJ90232;MGC126521;MGC126523;OF	BRCA1 interacting protein C-terminal helicase 1	0.93	1.15	1.16	0.76
DCLRE1C	DCLRE1C;A-SCID;DCLREC1C;FLJ11360;FLJ36438;RS-SCID;SCIDA;SNM1C;hSNM1C	DNA cross-link repair 1C	0.92	1.14	1.16	0.75
GLB1	GLB1;EBP;ELNR1;MPS4B	galactosidase, beta 1	0.92	1.04	1.16	0.73
VMA21	VMA21;MEAX;MGC125514;MGC125516;MGC131652;XMEA	VMA21 vacuolar H+-ATPase homolog (S. cerevisiae)	0.92	1.09	1.16	0.86
SLC4A9	SLC4A9;AE4	solute carrier family 4, sodium bicarbonate cotransporter, member 9	0.91	1.04	1.16	0.77
RCCD1	RCCD1;MGC14386	RCC1 domain containing 1	0.91	1.03	1.16	0.77
ABCC5	ABCC5;ABC33;DKFZp686C1782;EST277145;MOAT-C;MOATC;MRP5;SMRP;pABC11	ATP-binding cassette, sub-family C (CFTR/MRP), member 5	0.91	1.1	1.16	0.74
NFS1	NFS1;IscS;NIFS	NFS1 nitrogen fixation 1 homolog (S. cerevisiae)	0.91	1.01	1.16	0.73
WWP1	WWP1;AIP5;DKFZp434D2111;Tiul1;hSDRP1	WW domain containing E3 ubiquitin protein ligase 1	0.91	0.97	1.16	0.73
DPP6	DPP6;DPPX;FLJ55680;MGC46605;VF2	dipeptidyl-peptidase 6	0.9	1.03	1.16	0.77
KIAA1324L	KIAA1324L;EIG121L;FLJ31340;hEIG121L	KIAA1324-like	0.9	1.01	1.16	0.77
HTRA4	HTRA4;FLJ90724	HtrA serine peptidase 4	0.9	1.09	1.16	0.73
TMEM63C	TMEM63C;C14orf171	transmembrane protein 63C	0.9	1.02	1.16	0.77
ADAMTS8	ADAMTS8;ADAM-TS8;FLJ41712;METH2	ADAM metallopeptidase with thrombospondin type 1 motif, 8	0.9	1.04	1.16	0.78
FOXA2	FOXA2;HNF3B;MGC19807;TCF3B	forkhead box A2	0.9	0.97	1.16	0.77
SLC9A8	SLC9A8;DKFZp686C03237;FLJ42500;KIAA0939;MGC138418;NHE8	solute carrier family 9 (sodium/hydrogen exchanger), member 8	0.9	1.07	1.16	0.74
LMF2	LMF2;TMEM112B;TMEM153	lipase maturation factor 2	0.9	1.11	1.16	0.79
KIDINS220	KIDINS220;ARMS;MGC163482	kinase D-interacting substrate, 220kDa	0.9	1	1.16	0.74
MFSD1	MFSD1;FLJ14153;SMAP4	major facilitator superfamily domain containing 1	0.9	1.09	1.16	0.73
ECT2	ECT2;ARHGEF31;FLJ10461;MGC138291	epithelial cell transforming sequence 2 oncogene	0.9	1.02	1.16	0.8
KLK6	KLK6;Bssp;Klk7;MGC9355;PRSS18;PRSS9;SP59;hK6	kallikrein-related peptidase 6	0.89	0.96	1.16	0.72
STAM2	STAM2;DKFZp564C047;Hbp;STAM2A;STAM2B	signal transducing adaptor molecule (SH3 domain and ITAM motif) 2	0.89	1	1.16	0.8
PURG	PURG;MGC119274;PURG-A;PURG-B	purine-rich element binding protein G	0.88	1.02	1.16	0.7
SLC24A4	SLC24A4;FLJ38852;NCKX4;SHEP6;SLC24A2	solute carrier family 24 (sodium/potassium/calcium exchanger), member 4	0.88	0.94	1.16	0.75
ITGA5	ITGA5;CD49e;FNRA;VLA5A	integrin, alpha 5 (fibronectin receptor, alpha polypeptide)	0.88	0.99	1.16	0.73
PABPC3	PABPC3;PABP3;PABPL3;tPABP	poly(A) binding protein, cytoplasmic 3	0.88	0.92	1.16	0.77
GSK3B	GSK3B	glycogen synthase kinase 3 beta	0.88	0.98	1.16	0.89
PASK	PASK;DKFZp434O051;DKFZp686P2031;KIAA0135;PASKIN;STK37	PAS domain containing serine/threonine kinase	0.87	0.98	1.16	0.89
DIS3L2	DIS3L2;FAM6A;FLJ36974;MGC42174	DIS3 mitotic control homolog (S. cerevisiae)-like 2	0.87	1.01	1.16	0.79
C17orf85	C17orf85;ELG;HSA277841	chromosome 17 open reading frame 85	0.87	0.9	1.16	0.7
POLDIP2	POLDIP2;DKFZp586F1524;PDIP38;POLD4	polymerase (DNA-directed), delta interacting protein 2	0.87	1.04	1.16	0.79
OR10A4	OR10A4;JCG5;OR10A4P	olfactory receptor, family 10, subfamily A, member 4	0.86	0.98	1.16	0.74
C19orf68	C19orf68;MGC50339	chromosome 19 open reading frame 68	0.86	1.04	1.16	0.71
GPN2	GPN2;ATPBD1B;FLJ10349	GPN-loop GTPase 2	0.86	0.88	1.16	0.69
SIAH2	SIAH2;hSiah2	seven in absentia homolog 2 (Drosophila)	0.86	0.98	1.16	0.88
ITGB6	ITGB6	integrin, beta 6	0.86	0.94	1.16	0.74
CDIPT	CDIPT;MGC1328;PIS;PIS1	CDP-diacylglycerol--inositol 3-phosphatidyltransferase	0.86	0.97	1.16	0.71
TGFBR2	TGFBR2;AAT3;FAA3;LDS1B;LDS2B;MFS2;RIIC;TAAD2;TGFR-2;TGFbeta-RII	transforming growth factor, beta receptor II (70/80kDa)	0.86	1.08	1.16	0.76
CPA6	CPA6;CPAH	carboxypeptidase A6	0.85	0.9	1.16	0.68
SNTB1	SNTB1;59-DAP;A1B;BSYN2;DAPA1B;FLJ22442;MGC111389;SNT2;SNT2B1;TIP-43	syntrophin, beta 1 (dystrophin-associated protein A1, 59kDa, basic component 1)	0.85	0.93	1.16	0.79
LPCAT4	LPCAT4;AGPAT7;AYTL3;FLJ10257;LPAAT-eta;LPEAT2	lysophosphatidylcholine acyltransferase 4	0.85	0.93	1.16	0.75
FAM36A	FAM36A;FLJ43269	family with sequence similarity 36, member A	0.85	0.97	1.16	0.8
RCE1	RCE1;FACE2;RCE1A;RCE1B	RCE1 homolog, prenyl protein peptidase (S. cerevisiae)	1.19	1.28	1.17	0.96
CDC14B	CDC14B;CDC14B3;Cdc14B1;Cdc14B2;hCDC14B	CDC14 cell division cycle 14 homolog B (S. cerevisiae)	1.18	1.27	1.17	0.96
FSTL5	FSTL5;DKFZp566D234;KIAA1263	follistatin-like 5	1.15	1.29	1.17	0.93
SCN11A	SCN11A;NAV1.9;NaN;SCN12A;SNS-2	sodium channel, voltage-gated, type XI, alpha subunit	1.12	1.3	1.17	1.02
GRAP2	GRAP2;GADS;GRAP-2;GRB2L;GRBLG;GRID;GRPL;GrbX;Grf40;Mona;P38	GRB2-related adaptor protein 2	1.1	1.16	1.17	0.92
NRCAM	NRCAM;KIAA0343;MGC138845;MGC138846	neuronal cell adhesion molecule	1.1	1.29	1.17	0.88
APOC1	APOC1	apolipoprotein C-I	1.09	1.24	1.17	1.07
ZNF92	ZNF92;HPF12;HTF12;TF12	zinc finger protein 92	1.08	1.31	1.17	0.89
GABRE	GABRE	gamma-aminobutyric acid (GABA) A receptor, epsilon	1.06	1.23	1.17	0.96
WDR24	WDR24;C16orf21;DKFZp434F054;JFP7	WD repeat domain 24	1.06	1.18	1.17	0.86
FZD7	FZD7;FzE3	frizzled family receptor 7	1.05	1.19	1.17	1.08
PHF20L1	PHF20L1;MGC64923	PHD finger protein 20-like 1	1.05	1.17	1.17	0.85
CFI	CFI;AHUS3;C3BINA;C3b-INA;FI;IF;KAF	complement factor I	1.04	1.12	1.17	0.84
TAC4	TAC4;EK;HK-1;MGC133009;MGC133010;PPT-C	tachykinin 4 (hemokinin)	1.04	1.2	1.17	0.9
ARID1A	ARID1A;B120;BAF250;BAF250a;BM029;C1orf4;P270;SMARCF1	AT rich interactive domain 1A (SWI-like)	1.04	1.14	1.17	1
RIOK3	RIOK3;DKFZp779L1370;SUDD	RIO kinase 3 (yeast)	1.03	1.17	1.17	1.06
ZDHHC8	ZDHHC8;DHHC8;ZDHHCL1;ZNF378	zinc finger, DHHC-type containing 8	1.02	1.19	1.17	0.99
HLCS	HLCS;HCS	holocarboxylase synthetase (biotin-(proprionyl-CoA-carboxylase (ATP-hydrolysing)) ligase)	1.02	1.14	1.17	0.82
TFR2	TFR2;HFE3;MGC126368;TFRC2	transferrin receptor 2	1.01	1.17	1.17	0.87
UCP1	UCP1;SLC25A7;UCP	uncoupling protein 1 (mitochondrial, proton carrier)	1	1.08	1.17	0.95
METAP1	METAP1;DKFZp781C0419;KIAA0094;MAP1A;MetAP1A	methionyl aminopeptidase 1	1	1.17	1.17	0.87
EDEM3	EDEM3;C1orf22	ER degradation enhancer, mannosidase alpha-like 3	0.99	1.13	1.17	0.8
OLFM1	OLFM1;AMY;NOE1;NOELIN1;OlfA	olfactomedin 1	0.98	1.12	1.17	0.94
MRGPRE	MRGPRE;GPR167;MGC138408;MRGE	MAS-related GPR, member E	0.98	1.07	1.17	0.81
ONECUT1	ONECUT1;HNF-6;HNF6;HNF6A	one cut homeobox 1	0.98	1.21	1.17	0.81
RNF123	RNF123;DKFZp686C2222;FLJ12565;KPC1;MGC163504	ring finger protein 123	0.98	1.08	1.17	0.78
RCBTB1	RCBTB1;CLLD7;CLLL7;GLP;MGC33184;RP11-185C18.1	regulator of chromosome condensation (RCC1) and BTB (POZ) domain containing protein 1	0.98	1.14	1.17	0.94
MANEA	MANEA;DKFZp686D20120;ENDO;FLJ12838;hEndo	mannosidase, endo-alpha	0.98	1.11	1.17	0.8
RUNDC1	RUNDC1;DKFZp761H0421	RUN domain containing 1	0.98	1.17	1.17	0.93
KRTAP26-1	KRTAP26-1;MGC71787	keratin associated protein 26-1	0.97	1.09	1.17	0.84
C14orf79	C14orf79	chromosome 14 open reading frame 79	0.97	1.1	1.17	0.83
PSG9	PSG9;PS34;PSBG-11;PSBG-9;PSG11;PSGII	pregnancy specific beta-1-glycoprotein 9	0.97	1.13	1.17	0.79
SYT7	SYT7;IPCA-7;IPCA7;MGC150517;PCANAP7;SYT-VII;SYTVII	synaptotagmin VII	0.97	1.08	1.17	0.79
DSCC1	DSCC1;DCC1;MGC5528	defective in sister chromatid cohesion 1 homolog (S. cerevisiae)	0.97	1.11	1.17	0.83
ANKRD42	ANKRD42;FLJ37874;SARP	ankyrin repeat domain 42	0.97	1.02	1.17	0.8
SLC32A1	SLC32A1;VGAT;VIAAT	solute carrier family 32 (GABA vesicular transporter), member 1	0.96	1.08	1.17	0.78
ZSCAN16	ZSCAN16;FLJ22191;ZNF392;ZNF435;dJ265C24.3	zinc finger and SCAN domain containing 16	0.96	1.16	1.17	0.83
ZNF823	ZNF823;HSZFP36	zinc finger protein 823	0.96	1.1	1.17	0.92
GOT2	GOT2;FLJ40994;KAT4;KATIV;mitAAT	glutamic-oxaloacetic transaminase 2, mitochondrial (aspartate aminotransferase 2)	0.96	1.19	1.17	0.78
FGF20	FGF20;FGF-20	fibroblast growth factor 20	0.95	1.02	1.17	0.91
SLC19A3	SLC19A3;THTR2	solute carrier family 19, member 3	0.95	1.03	1.17	0.91
CASQ2	CASQ2;FLJ26321;FLJ93514;PDIB2	calsequestrin 2 (cardiac muscle)	0.94	1.09	1.17	0.74
FAM47A	FAM47A;MGC27003	family with sequence similarity 47, member A	0.94	1.18	1.17	0.9
FBXL19	FBXL19;DKFZp434K0410;Fbl19;JHDM1C;MGC50505	F-box and leucine-rich repeat protein 19	0.94	1.06	1.17	0.76
ASXL2	ASXL2;ASXH2;DKFZp686C1968;FLJ10898;KIAA1685	additional sex combs like 2 (Drosophila)	0.94	1.14	1.17	0.78
SLC16A14	SLC16A14;FLJ30794;MCT14	solute carrier family 16, member 14 (monocarboxylic acid transporter 14)	0.92	1.05	1.17	0.75
KLF15	KLF15;DKFZp779M1320;KKLF	Kruppel-like factor 15	0.92	1.04	1.17	0.75
NGB	NGB	neuroglobin	0.92	1.04	1.17	0.82
ZNF575	ZNF575;FLJ32567	zinc finger protein 575	0.92	1.04	1.17	0.75
FKRP	FKRP;FLJ12576;LGMD2I;MDC1C;MDDGA5;MDDGB5;MDDGC5;MGC2991	fukutin related protein	0.92	1.08	1.17	0.73
SLC35B2	SLC35B2;PAPST1;SLL;UGTrel4	solute carrier family 35, member B2	0.92	1.07	1.17	0.74
C16orf80	C16orf80;EVORF;GTL3;fSAP23	chromosome 16 open reading frame 80	0.92	1.02	1.17	0.83
LRRC16A	LRRC16A;CARMIL;CARMIL1;CARMIL1a;FLJ20048;FLJ43708;LRRC16;dJ501N12.1;dJ501N12.5	leucine rich repeat containing 16A	0.92	1.03	1.17	0.79
RFTN2	RFTN2;C2orf11;FLJ30574;MGC117313;Raftlin-2	raftlin family member 2	0.9	1.01	1.17	0.77
ACSM2B	ACSM2B;ACSM2;HXMA	acyl-CoA synthetase medium-chain family member 2B	0.9	1.03	1.17	0.73
CYP11A1	CYP11A1;CYP11A;P450SCC	cytochrome P450, family 11, subfamily A, polypeptide 1	0.9	0.99	1.17	0.79

SLITRK5	SLITRK5;FLJ58374;KIAA0918;LRRC11;bA364G4.2	SLIT and NTRK-like family, member 5	0.9	1.08	1.17	0.82
ARHGEF10	ARHGEF10;DKFZp686H0726;GEF10;MGC131664	Rho guanine nucleotide exchange factor (GEF) 10	0.9	1.08	1.17	0.85
ATG4B	ATG4B;APG4B;AUTL1;MGC1353	ATG4 autophagy related 4 homolog B (S. cerevisiae)	0.9	0.98	1.17	0.73
RFT1	RFT1;CDG1N;DKFZp667J092;FLJ25945	RFT1 homolog (S. cerevisiae)	0.9	1.02	1.17	0.77
RCN2	RCN2;E6BP;ERC-55;ERC55;TCBP49	reticulocalbin 2, EF-hand calcium binding domain	0.9	1.08	1.17	0.78
ATP5H	ATP5H;ATPQ	ATP synthase, H+ transporting, mitochondrial Fo complex, subunit d	0.9	1.08	1.17	0.85
FLVCR1	FLVCR1;AXPC1;FLJ33420;FLVCR;MFSD7B;PCA;PCARP	feline leukemia virus subgroup C cellular receptor 1	0.89	1.04	1.17	0.81
KLHL1	KLHL1;FLJ30047;KIAA1490;MRP2	kelch-like 1 (Drosophila)	0.88	0.97	1.17	0.82
SEMA6A	SEMA6A;HT018;KIAA1368;SEMA;SEMA6A1;SEMAQ;VIA	sema domain, transmembrane domain (TM), and cytoplasmic domain, (semaphorin) 6A	0.88	0.99	1.17	0.71
LRRC10	LRRC10;HRLRRP;LRRC10A;MGC125812	leucine rich repeat containing 10	0.88	1	1.17	0.79
ADH1A	ADH1A;ADH1	alcohol dehydrogenase 1A (class I), alpha polypeptide	0.88	0.95	1.17	0.84
DPYSL5	DPYSL5;CRAM;CRMP-5;CRMP5;FLJ45383;Ulip6	dihydropyrimidinase-like 5	0.88	0.97	1.17	0.85
PRM2	PRM2;CT94.2;FLJ27447	protamine 2	0.88	0.92	1.17	0.73
PACSIN3	PACSIN3;FLJ17861;SDPIII	protein kinase C and casein kinase substrate in neurons 3	0.88	1.1	1.17	0.73
RBM12B	RBM12B;MGC:33837	RNA binding motif protein 12B	0.88	1.07	1.17	0.76
GCNT2	GCNT2;CCAT;GCNT2C;GCNT5;IGNT;II;MGC163396;NACGT1;NAGCT1;ULG3;bA360O19.2;bA421M1.1	glucosaminyl (N-acetyl) transferase 2, I-branching enzyme (I blood group)	0.88	1.02	1.17	0.71
AGPAT1	AGPAT1;1-AGPAT1;G15;LPAAT-alpha;LPAATA;MGC4007;MGC5423	1-acylglycerol-3-phosphate O-acyltransferase 1 (lysophosphatidic acid acyltransferase, alpha)	0.88	1.1	1.17	0.83
GLOD4	GLOD4;C17orf25;HC71	glyoxalase domain containing 4	0.88	0.99	1.17	0.71
DLG1	DLG1;DKFZp761P0818;DKFZp781B0426;DLGH1;SAP-97;SAP97;dJ1061C18.1.1;hdlg	discs, large homolog 1 (Drosophila)	0.88	1	1.17	0.9
CTSZ	CTSZ;CTSX;FLJ17088	cathepsin Z	0.88	0.96	1.17	0.71
GSTT2B	GSTT2B;GSTT2P;MGC87963	glutathione S-transferase theta 2B (gene/pseudogene)	0.88	0.99	1.17	0.78
MB21D2	MB21D2;C3orf59	Mab-21 domain containing 2	0.88	1.08	1.17	0.77
CDR2L	CDR2L;HUMPPA	cerebellar degeneration-related protein 2-like	0.87	0.96	1.17	0.78
RRP12	RRP12;DKFZp762P1116;FLJ20231;KIAA0690	ribosomal RNA processing 12 homolog (S. cerevisiae)	0.87	0.91	1.17	0.71
CCDC147	CCDC147;C10orf80;FLJ35908;MGC126475;bA127L20.4;bA127L20.5;bA554P13.1	coiled-coil domain containing 147	0.86	0.96	1.17	0.7
UBQLNL	UBQLNL;MGC20470;MGC26958	ubiquilin-like	0.86	1.08	1.17	0.72
ST6GALNAC5	ST6GALNAC5;MGC3184;SIAT7E;ST6GalNAcV	ST6 (alpha-N-acetyl-neuraminyl-2,3-beta-galactosyl-1,3)-N-acetylgalactosaminide alpha-2,6-sialyltransferase 5	0.86	1	1.17	0.74
CXCL5	CXCL5;ENA-78;SCYB5	chemokine (C-X-C motif) ligand 5	0.86	1.05	1.17	0.79
GALNS	GALNS;FLJ17434;FLJ42844;FLJ98217;GALNAC6S;GAS;MPS4A	galactosamine (N-acetyl)-6-sulfate sulfatase	0.86	0.94	1.17	0.75
C1orf174	C1orf174;RP13-531C17.2	chromosome 1 open reading frame 174	0.86	1.08	1.17	0.83
NDUFA9	NDUFA9;CC6;CI-39k;CI39k;MGC111043;NDUFS2L;SDR22E1	NADH dehydrogenase (ubiquinone) 1 alpha subcomplex, 9, 39kDa	0.86	0.97	1.17	0.7
WTH3DI	WTH3DI	RAB6C-like	0.86	1.04	1.17	0.79
C7orf10	C7orf10;DERP13;FLJ11808;FLJ53872;ORF19	chromosome 7 open reading frame 10	0.85	1.02	1.17	0.74
TBC1D1	TBC1D1;KIAA1108;TBC;TBC1	TBC1 (tre-2/USP6, BUB2, cdc16) domain family, member 1	0.85	0.97	1.17	0.74
RQCD1	RQCD1;CNOT9;CT129;RCD1;RCD1+	RCD1 required for cell differentiation1 homolog (S. pombe)	0.85	0.95	1.17	0.73
ARID5B	ARID5B;DESRT;FLJ21150;FLJ41888;MRF-2;MRF2	AT rich interactive domain 5B (MRF1-like)	1.29	1.46	1.18	1.05
GPR110	GPR110;FLJ22684;FLJ30646;KPG_012;MGC125952;PGR19;hGPCR36	G protein-coupled receptor 110	1.27	1.4	1.18	1.05
CCR4	CCR4;CC-CKR-4;CD194;CKR4;CMKBR4;ChemR13;HGCN:14099;K5-5;MGC88293	chemokine (C-C motif) receptor 4	1.17	1.27	1.18	0.96
GLTP	GLTP	glycolipid transfer protein	1.17	1.3	1.18	1.1
RNF6	RNF6;DKFZp686P0776	ring finger protein (C3H2C3 type) 6	1.08	1.21	1.18	0.88
NUCB1	NUCB1;CALNUC;DKFZp686A15286;FLJ40471;NUC	nucleobindin 1	1.04	1.18	1.18	0.91
MAGED1	MAGED1;DLXIN-1;NRAGE	melanoma antigen family D, 1	1.04	1.19	1.18	0.93
RNF39	RNF39;HZF;HZFW;LIRF	ring finger protein 39	1.02	1.15	1.18	0.83
PCSK4	PCSK4;DKFZp434B217;MGC34749;PC4;SPC5	proprotein convertase subtilisin/kexin type 4	1.02	1.19	1.18	0.89
MESDC2	MESDC2;BOCA;KIAA0081;MESD	mesoderm development candidate 2	1.02	1.19	1.18	0.98
C5orf4	C5orf4;FLJ13758	chromosome 5 open reading frame 4	1.01	1.22	1.18	0.93
NUP37	NUP37;FLJ22618;MGC5585;p37	nucleoporin 37kDa	1	1.07	1.18	0.85
SPANXN2	SPANXN2;CT11.7;SPANX-N2	SPANX family, member N2	0.99	1.23	1.18	0.88
TBX6	TBX6;DFNB67	T-box 6	0.99	1.14	1.18	0.8
DEFB107B	DEFB107B;DEFB107A;HsT21816	defensin, beta 107B	0.99	1.21	1.18	0.92
TNFSF14	TNFSF14;CD258;HVEML;LIGHT;LTg;TR2	tumor necrosis factor (ligand) superfamily, member 14	0.98	1.1	1.18	0.85
TFE3	TFE3;RCCP2;RCCX1;TFEA;bHLHe33	transcription factor binding to IGHM enhancer 3	0.98	1.07	1.18	0.94
JAM2	JAM2;C21orf43;CD322;JAM-B;JAMB;PRO245;VE-JAM;VEJAM	junctional adhesion molecule 2	0.97	1.11	1.18	0.84
KIAA0319	KIAA0319;DLX2;DYLX2;DYX2;MGC176717	KIAA0319	0.97	1.19	1.18	0.88
EXOSC5	EXOSC5;MGC111224;MGC12901;RRP41B;RRP46;Rrp46p;hRrp46p;p12B	exosome component 5	0.97	1.06	1.18	0.85
MTPAP	MTPAP;FLJ10486;PAPD1;SPAX4	mitochondrial poly(A) polymerase	0.97	1.17	1.18	0.8
OR11A1	OR11A1;OR11A2;dJ994E9.6;hs6M1-18	olfactory receptor, family 11, subfamily A, member 1	0.96	1.1	1.18	0.89
BCMO1	BCMO1;BCDO;BCDO1;BCMO;BCO;BCO1;FLJ10730	beta-carotene 15,15'-monooxygenase 1	0.96	1.19	1.18	0.79
ASXL1	ASXL1;BOPS;KIAA0978;MDS;MGC117280;MGC71111	additional sex combs like 1 (Drosophila)	0.96	1.1	1.18	0.83
CNN2	CNN2	calponin 2	0.95	1.11	1.18	0.92
C12orf26	C12orf26;FLJ22789	chromosome 12 open reading frame 26	0.94	1.15	1.18	0.83
PCDH10	PCDH10;DKFZp761O2023;KIAA1400;MGC133344;OL-PCDH;PCDH19	protocadherin 10	0.94	1.17	1.18	0.87
C6orf108	C6orf108;RCL;RP3-330M21.3;dJ330M21.3	chromosome 6 open reading frame 108	0.94	1.04	1.18	0.82
PPP3CB	PPP3CB;CALNA2;CALNB;CNA2;PP2Bbeta	protein phosphatase 3, catalytic subunit, beta isozyme	0.94	1.11	1.18	0.86
C1QTNF9B	C1QTNF9B;CTRP9B;MGC117159	C1q and tumor necrosis factor related protein 9B	0.94	1.11	1.18	0.75
HYI	HYI;HT036;MGC20767	hydroxypyruvate isomerase (putative)	0.93	1.04	1.18	0.82
MLST8	MLST8;GBL;GbetaL;LST8;MGC111011;POP3;WAT1	MTOR associated protein, LST8 homolog (S. cerevisiae)	0.93	1.07	1.18	0.84
CTSL3	CTSL3;HCTSL-s	cathepsin L family member 3	0.92	1.15	1.18	0.8
RNF13	RNF13;FLJ93817;MGC13689;RZF	ring finger protein 13	0.92	1.01	1.18	0.76
PPP2CA	PPP2CA;PP2Ac;PP2CA;PP2Calpha;RP-C	protein phosphatase 2, catalytic subunit, alpha isozyme	0.92	1.07	1.18	0.84
LOC642219	LOC642219	ENTREZ STATUS: DISCONTINUED	0.92	1.08	1.18	0.96
BBS12	BBS12;C4orf24;FLJ35630;FLJ41559	Bardet-Biedl syndrome 12	0.91	1.15	1.18	0.81
CEP290	CEP290;3H11Ag;BBS14;CT87;FLJ13615;FLJ21979;JBTS5;JBTS6;KIAA0373;LCA10;MKS4;NPHP6;POC3;SLSN6;rd16	centrosomal protein 290kDa	0.91	1.07	1.18	0.77
UBA3	UBA3;DKFZp566J164;MGC22384;UBE1C;hUBA3	ubiquitin-like modifier activating enzyme 3	0.91	1	1.18	0.74
SMAGP	SMAGP;MGC149453;MGC149454;hSMAGP	small cell adhesion glycoprotein	0.91	1.04	1.18	0.75
HPD	HPD;4-HPPD;4HPPD;GLOD3;HPPDASE;PPD	4-hydroxyphenylpyruvate dioxygenase	0.9	1.12	1.18	0.74
ARPM1	ARPM1;MGC15664	actin related protein M1	0.9	0.96	1.18	0.76
C19orf70	C19orf70;P117;QIL1	chromosome 19 open reading frame 70	0.9	1.07	1.18	0.81
TRIM37	TRIM37;KIAA0898;MUL;POB1;TEF3	tripartite motif containing 37	0.9	1.02	1.18	0.73
NIT2	NIT2;MGC111199	nitrilase family, member 2	0.9	0.98	1.18	0.78
PPP1R32	PPP1R32;C11orf66;FLJ32771;IIIG9	protein phosphatase 1, regulatory subunit 32	0.9	1.12	1.18	0.8
CYTIP	CYTIP;B3-1;CASP;CYBR;CYTHIP;HE;PSCDBP	cytohesin 1 interacting protein	0.89	1.01	1.18	0.73
TSPYL2	TSPYL2;CDA1;CINAP;CTCL;DENTT;HRIHFB2216;NP79;SE204;TSPX	TSPY-like 2	0.88	1.03	1.18	0.86
MFSD8	MFSD8;CLN7;MGC33302	major facilitator superfamily domain containing 8	0.88	0.98	1.18	0.79
ZNF826P	ZNF826P;FLJ44894;ZNF826	zinc finger protein 826, pseudogene	0.88	1.08	1.18	0.82
MASP2	MASP2;MAP19;MASP-2;MASP1P1;sMAP	mannan-binding lectin serine peptidase 2	0.87	1.02	1.18	0.76
DDX17	DDX17;DKFZp761H2016;P72;RH70	DEAD (Asp-Glu-Ala-Asp) box polypeptide 17	0.87	1	1.18	0.86
PRR7	PRR7;MGC10772	proline rich 7 (synaptic)	0.86	1.09	1.18	0.84
C17orf70	C17orf70;FAAP100;FLJ22175;FLJ30151	chromosome 17 open reading frame 70	0.86	1.06	1.18	0.76
C12orf35	C12orf35;FLJ10652;FLJ20696	chromosome 12 open reading frame 35	0.86	0.98	1.18	0.75
RNGTT	RNGTT;CAP1A;DKFZp686J2031;HCE;HCE1;hCAP	RNA guanylyltransferase and 5'-phosphatase	0.86	1	1.18	0.79
NDUFS8	NDUFS8;CI-23k;CI23KD;TYKY	NADH dehydrogenase (ubiquinone) Fe-S protein 8, 23kDa (NADH-coenzyme Q reductase)	0.86	0.93	1.18	0.82
GRID2	GRID2;MGC117022;MGC117023;MGC117024	glutamate receptor, ionotropic, delta 2	0.85	1.04	1.18	0.81
FBXO46	FBXO46;20D7-FC4;FBXO34L;Fbx46	F-box protein 46	0.85	0.96	1.18	0.7
VCAM1	VCAM1;CD106;DKFZp779G2333;INCAM-100;MGC99561	vascular cell adhesion molecule 1	1.31	1.48	1.19	1.25
GRM8	GRM8;FLJ41058;GLUR8;GPRC1H;MGC126724;MGLUR8;mGlu8	glutamate receptor, metabotropic 8	1.23	1.36	1.19	1.02
PTPLA	PTPLA;CAP;HACD1	protein tyrosine phosphatase-like (proline instead of catalytic arginine), member A	1.18	1.4	1.19	1.09
LHCGR	LHCGR;FLJ41504;HHG;LCGR;LGR2;LH/CG-R;LH/CGR;LHR;LHRHR;LSH-R;ULG5	luteinizing hormone/choriogonadotropin receptor	1.17	1.28	1.19	0.97
CALR3	CALR3;CMH19;CRT2;CT93;FLJ25355;MGC26577	calreticulin 3	1.11	1.32	1.19	0.9
LEMD2	LEMD2;NET25;dJ482C21.1	LEM domain containing 2	1.09	1.3	1.19	0.88
FBXO28	FBXO28;CENP-30;FLJ10766;Fbx28;KIAA0483	F-box protein 28	1.06	1.21	1.19	0.87
VIT	VIT;DKFZp313L1517;MGC149746;MGC70561	vitrin	1.04	1.12	1.19	0.93
MLLT6	MLLT6;AF17;FLJ23480	myeloid/lymphoid or mixed-lineage leukemia (trithorax homolog, Drosophila); translocated to, 6	1.04	1.19	1.19	0.86
TNS1	TNS1;DKFZp586K0617;MGC88584;MST091;MST122;MST127;MSTP122;MSTP127;MXRA6;TNS	tensin 1	1.03	1.18	1.19	0.9
COL4A5	COL4A5;ASLN;ATS;CA54;MGC167109;MGC42377	collagen, type IV, alpha 5	1.01	1.18	1.19	0.98
TMEM119	TMEM119	transmembrane protein 119	1.01	1.25	1.19	0.9
ECHS1	ECHS1;SCEH	enoyl CoA hydratase, short chain, 1, mitochondrial	1.01	1.19	1.19	0.89
FBXO39	FBXO39;FLJ18190;FLJ98753;Fbx39;MGC35179	F-box protein 39	1	1.14	1.19	0.82
CLCNKA	CLCNKA;CLCK1;ClC-K1;MGC61490;hClC-Ka	chloride channel Ka	1	1.18	1.19	0.92
KLK14	KLK14;KLK-L6	kallikrein-related peptidase 14	1	1.17	1.19	0.88
ZNF181	ZNF181;HHZ181;MGC44316	zinc finger protein 181	1	1.15	1.19	0.83
TULP4	TULP4;KIAA1397;TUSP	tubby like protein 4	0.99	1.12	1.19	0.81
C16orf78	C16orf78;MGC33367	chromosome 16 open reading frame 78	0.98	1.1	1.19	0.89
TMC3	TMC3	transmembrane channel-like 3	0.98	1.14	1.19	0.92
PHF10	PHF10;BAF45A;FLJ10975;MGC111009;XAP135	PHD finger protein 10	0.98	1.15	1.19	1.02
UBE4B	UBE4B;E4;HDNB1;KIAA0684;UBOX3;UFD2;UFD2A	ubiquitination factor E4B	0.97	1.1	1.19	0.8
GPR176	GPR176;GPR;Gm1012	G protein-coupled receptor 176	0.96	1.06	1.19	0.79
TSC22D2	TSC22D2;KIAA0669;TILZ4a;TILZ4b;TILZ4c	TSC22 domain family, member 2	0.96	1.02	1.19	0.8
WDR69	WDR69;FLJ25955;ODA16	WD repeat domain 69	0.95	1.07	1.19	0.86
TMSL3	TMSL3	thymosin-like 3	0.95	1.19	1.19	0.83
SUN3	SUN3;MGC33329;SUNC1	Sad1 and UNC84 domain containing 3	0.95	1.12	1.19	0.81
ADAM12	ADAM12;MCMP;MCMPMltna;MLTN;MLTNA	ADAM metallopeptidase domain 12	0.94	1.03	1.19	0.77
COL4A6	COL4A6;CXDELq22.3;DELXq22.3;MGC88184	collagen, type IV, alpha 6	0.94	1	1.19	0.84
C10orf95	C10orf95;FLJ14280	chromosome 10 open reading frame 95	0.93	1.04	1.19	0.88
PPP1R26	PPP1R26;KIAA0649	protein phosphatase 1, regulatory subunit 26	0.93	1.14	1.19	0.86
COL2A1	COL2A1;ANFH;AOM;COL11A3;MGC131516;SEDC	collagen, type II, alpha 1	0.92	1.07	1.19	0.89
DDHD1	DDHD1;FLJ34209;FLJ42555;PA-PLA1	DDHD domain containing 1	0.92	1.06	1.19	0.76
CEP85	CEP85;CCDC21;DKFZp434L0117;DKFZp434P232;FLJ13976;FLJ22000	centrosomal protein 85kDa	0.92	1.04	1.19	0.84
HSD17B4	HSD17B4;DBP;MFE-2;MPF-2;SDR8C1	hydroxysteroid (17-beta) dehydrogenase 4	0.91	1.09	1.19	0.73

CHCHD3	CHCHD3;FLJ20420;MINOS3;PPP1R22	coiled-coil-helix-coiled-coil-helix domain containing 3	0.91	1.05	1.19	0.85
MEP1A	MEP1A;PPHA	meprin A, alpha (PABA peptide hydrolase)	0.9	1.06	1.19	0.8
GABRR2	GABRR2	gamma-aminobutyric acid (GABA) receptor, rho 2	0.9	1.11	1.19	0.86
ZNF329	ZNF329;FLJ12586	zinc finger protein 329	0.9	1.02	1.19	0.83
EVI5L	EVI5L	ecotropic viral integration site 5-like	0.9	1.06	1.19	0.77
ICOSLG	ICOSLG;B7-H2;B7H2;B7RP-1;B7RP1;CD275;GL50;ICOS-L;ICOSL;KIAA0653;LICOS	inducible T-cell co-stimulator ligand	0.9	1.13	1.19	0.81
CABP2	CABP2	calcium binding protein 2	0.9	1.08	1.19	0.75
BAG5	BAG5;BAG-5	BCL2-associated athanogene 5	0.9	1.07	1.19	0.83
CTPS2	CTPS2;DKFZp686C17207;FLJ43358;MGC32997	CTP synthase II	0.9	1.03	1.19	0.74
FBXW11	FBXW11;BTRC2;BTRCP2;FBW1B;FBXW1B;Fbw11;Hos;KIAA0696	F-box and WD repeat domain containing 11	0.9	1.03	1.19	0.74
SNRNP40	SNRNP40;40K;FLJ41108;HPRP8BP;MGC1910;PRP8BP;PRPF8BP;RP11-490K7.3;SPF38;WDR57	small nuclear ribonucleoprotein 40kDa (U5)	0.9	1.1	1.19	0.79
TNNI3	TNNI3;CMD1FF;CMD2A;CMH7;MGC116817;RCM1;TNNC1;cTnI	troponin I type 3 (cardiac)	0.89	1.07	1.19	0.72
GPX6	GPX6;GPX5p;GPXP3;GPx-6;GSHPx-6;dJ1186N24;dJ1186N24.1	glutathione peroxidase 6 (olfactory)	0.88	1.02	1.19	0.72
CD209	CD209;CDSIGN;CLEC4L;DC-SIGN;DC-SIGN1;MGC129965	CD209 molecule	0.88	1.06	1.19	0.74
GOLGA4	GOLGA4;GCP2;GOLG;MU-RMS-40.18;p230	golgin A4	0.88	1.06	1.19	0.77
LEO1	LEO1;RDL	Leo1, Paf1/RNA polymerase II complex component, homolog (S. cerevisiae)	0.88	1.02	1.19	0.76
GIPC1	GIPC1;C19orf3;GIPC;GLUT1CBP;Hs.6454;IIP-1;MGC15889;MGC3774;NIP;RGS19IP1;SEMCAP;SYNECTIIN;SYNECTIN;TIP-2	GIPC PDZ domain containing family, member 1	0.88	1.07	1.19	0.81
ADAM8	ADAM8;CD156;MGC134985;MS2	ADAM metallopeptidase domain 8	0.87	0.96	1.19	0.72
XRCC6BP1	XRCC6BP1;KUB3;MGC134817;MGC134818	XRCC6 binding protein 1	0.87	1	1.19	0.91
BGN	BGN;DSPG1;PG-S1;PGI;SLRR1A	biglycan	0.86	1.05	1.19	0.8
RNF11	RNF11;MGC51169;SID1669	ring finger protein 11	0.86	1.01	1.19	0.91
SEMA3F	SEMA3F;SEMA-IV;SEMA4;SEMAK	sema domain, immunoglobulin domain (Ig), short basic domain, secreted, (semaphorin) 3F	0.85	0.99	1.19	0.74
GPR61	GPR61;BALGR;GPCR3	G protein-coupled receptor 61	1.38	1.53	1.2	1.15
ASB9	ASB9;DKFZp564L0862;FLJ20636;MGC4954	ankyrin repeat and SOCS box containing 9	1.22	1.4	1.2	1.01
PCGF6	PCGF6;MBLR;MGC15678;MGC17541;RNF134	polycomb group ring finger 6	1.1	1.31	1.2	1.17
CD300LF	CD300LF;CD300f;CLM-1;CLM1;IREM-1;IREM1;IgSF13;NKIR	CD300 molecule-like family member f	1.08	1.16	1.2	0.91
MRPL1	MRPL1;BM022;FLJ96680;L1MT;MRP-L1	mitochondrial ribosomal protein L1	1.07	1.27	1.2	0.96
TAF1L	TAF1L;MGC134910;TAF2A2	TAF1 RNA polymerase II, TATA box binding protein (TBP)-associated factor, 210kDa-like	1.05	1.25	1.2	1.04
DDX53	DDX53;CAGE;CT26	DEAD (Asp-Glu-Ala-Asp) box polypeptide 53	1.04	1.23	1.2	0.92
ADRA2A	ADRA2A;ADRA2;ADRA2R;ADRAR;ALPHA2AAR;ZNF32	adrenergic, alpha-2A-, receptor	1.04	1.16	1.2	0.87
CDADC1	CDADC1;MGC150615;MGC41774;MGC57136;NYD-SP15;bA103J18.1	cytidine and dCMP deaminase domain containing 1	1.03	1.24	1.2	1.02
SLC16A9	SLC16A9;C10orf36;FLJ43803;MCT9	solute carrier family 16, member 9 (monocarboxylic acid transporter 9)	1.02	1.21	1.2	1
PRKCB	PRKCB;MGC41878;PKC-beta;PKCB;PRKCB1;PRKCB2	protein kinase C, beta	1.01	1.17	1.2	1.06
TNFRSF19	TNFRSF19;TAJ;TAJ-alpha;TRADE;TROY	tumor necrosis factor receptor superfamily, member 19	1	1.16	1.2	0.85
PGBD4	PGBD4;FLJ32638;FLJ37497	piggyBac transposable element derived 4	1	1.16	1.2	0.94
RAB5C	RAB5C;L1880;MGC117217;MGC138857;RAB5CL;RAB5L;RABL	RAB5C, member RAS oncogene family	1	1.18	1.2	0.87
STIP1	STIP1;HOP;IEF-SSP-3521;P60;STI1;STI1L	stress-induced-phosphoprotein 1	1	1.08	1.2	0.9
RNF152	RNF152;FLJ39176;MGC138161	ring finger protein 152	0.98	1.12	1.2	0.81
TXNDC17	TXNDC17;MGC14353;TRP14;TXNL5	thioredoxin domain containing 17	0.97	1.14	1.2	0.8
RNF38	RNF38;FLJ21343	ring finger protein 38	0.97	1.1	1.2	0.8
UBE2V1	UBE2V1;CIR1;CROC-1;CROC1;UBE2V;UEV-1;UEV1;UEV1A	ubiquitin-conjugating enzyme E2 variant 1	0.97	1.06	1.2	0.8
THOC2	THOC2;CXorf3;THO2;dJ506G2.1;hTREX120	THO complex 2	0.96	1.16	1.2	0.8
HSD3B1	HSD3B1;3BETAHSD;HSD3B;HSDB3;HSDB3A;I;SDR11E1	hydroxy-delta-5-steroid dehydrogenase, 3 beta- and steroid delta-isomerase 1	0.94	1.09	1.2	0.77
CTGF	CTGF;CCN2;HCS24;IGFBP8;MGC102839;NOV2	connective tissue growth factor	0.94	1.1	1.2	0.82
HOXC10	HOXC10;HOX3I;MGC5259	homeobox C10	0.92	1.12	1.2	0.75
LIMD1	LIMD1	LIM domains containing 1	0.92	1.06	1.2	0.77
MMADHC	MMADHC;C2orf25;CL25022;cblD	methylmalonic aciduria (cobalamin deficiency) cblD type, with homocystinuria	0.92	1.1	1.2	0.77
PLIN4	PLIN4;KIAA1881;S3-12	perilipin 4	0.92	1.06	1.2	0.76
OR2AT4	OR2AT4;OR11-265	olfactory receptor, family 2, subfamily AT, member 4	0.9	1.09	1.2	0.74
ZNHIT1	ZNHIT1;CG1I;ZNFN4A1	zinc finger, HIT-type containing 1	0.9	1.1	1.2	0.76
RAB2A	RAB2A;LHX;RAB2	RAB2A, member RAS oncogene family	0.9	1	1.2	0.88
NEURL3	NEURL3;LINCR	neuralized homolog 3 (Drosophila) pseudogene	0.9	1.02	1.2	0.74
C10orf140	C10orf140;DKFZp761J229;DLN-1;FLJ45187	chromosome 10 open reading frame 140	0.89	1.04	1.2	0.79
ENC1	ENC1;CCL28;ENC-1;FLJ39259;KLHL35;KLHL37;NRPB;PIG10;TP53I10	ectodermal-neural cortex 1 (with BTB-like domain)	0.89	1	1.2	0.79
HELB	HELB	helicase (DNA) B	0.88	1.02	1.2	0.75
OSR2	OSR2;FLJ90037	odd-skipped related 2 (Drosophila)	0.88	1.02	1.2	0.74
MLXIP	MLXIP;KIAA0867;MIR;MONDOA;bHLHe36	MLX interacting protein	0.88	1	1.2	0.81
ASF1A	ASF1A;CGI-98;CIA;DKFZp547E2110;HSPC146	ASF1 anti-silencing function 1 homolog A (S. cerevisiae)	0.88	1.12	1.2	0.74
HERPUD1	HERPUD1;HERP;KIAA0025;Mif1;SUP	homocysteine-inducible, endoplasmic reticulum stress-inducible, ubiquitin-like domain member 1	0.88	0.96	1.2	0.76
KDM3A	KDM3A;DKFZp686A24246;DKFZp686P07111;JHDM2A;JHMD2A;JMJD1;JMJD1A;KIAA0742;TSGA	lysine (K)-specific demethylase 3A	0.88	1.02	1.2	0.74
PKNOX1	PKNOX1;PREP1;pkonx1c	PBX/knotted 1 homeobox 1	0.87	1.02	1.2	0.72
ACIN1	ACIN1;ACINUS;ACN;DKFZp667N107;KIAA0670;fSAP152	apoptotic chromatin condensation inducer 1	0.87	1.02	1.2	0.79
CSF2	CSF2;GMCSF;MGC131935;MGC138897	colony stimulating factor 2 (granulocyte-macrophage)	0.86	0.96	1.2	0.84
REN	REN;FLJ10761;HNFJ2	renin	0.86	1.02	1.2	0.77
NFIL3	NFIL3;E4BP4;IL3BP1;NF-IL3A;NFIL3A	nuclear factor, interleukin 3 regulated	0.86	1.09	1.2	0.72
IMPA1	IMPA1;IMP;IMPA	inositol(myo)-1(or 4)-monophosphatase 1	0.86	1.04	1.2	0.81
PRKRIR	PRKRIR;DAP4;MGC102750;P52rIPK;THAP0	protein-kinase, interferon-inducible double stranded RNA dependent inhibitor, repressor of (P58 repressor)	0.86	1.05	1.2	0.83
MAP4K4	MAP4K4;FLH21957;FLJ10410;FLJ20373;FLJ90111;HGK;KIAA0687;MEKKK4;NIK	mitogen-activated protein kinase kinase kinase kinase 4	0.86	1.02	1.2	0.85
C4orf49	C4orf49;CESP-1;HUMMR;MGARP;MGC125827;MGC125828;OSAP	chromosome 4 open reading frame 49	0.86	1.06	1.2	0.8
ARHGEF25	ARHGEF25;GEFT;p63RhoGEF	Rho guanine nucleotide exchange factor (GEF) 25	0.86	0.92	1.2	0.77
S1PR1	S1PR1;CD363;CHEDG1;D1S3362;ECGF1;EDG-1;EDG1;FLJ58121;S1P1	sphingosine-1-phosphate receptor 1	0.85	0.95	1.2	0.72
OR10H5	OR10H5;OR19-25;OR19-26	olfactory receptor, family 10, subfamily H, member 5	0.85	1.06	1.2	0.79
C2CD2	C2CD2;C21orf25;C21orf258;DKFZp586F0422;DKFZp686O198;MGC71445;TMEM24L	C2 calcium-dependent domain containing 2	0.85	1.06	1.2	0.79
NUDT5	NUDT5;YSA1;YSA1H;hYSAH1	nudix (nucleoside diphosphate linked moiety X)-type motif 5	1.16	1.3	1.21	0.98
NOL9	NOL9;FLJ23323;MGC131821;MGC138483;NET6	nucleolar protein 9	1.14	1.23	1.21	0.97
RNF4	RNF4;SLX5;SNURF	ring finger protein 4	1.09	1.3	1.21	1.16
GPR62	GPR62;GPCR8;KPG_005;MGC26943	G protein-coupled receptor 62	1.08	1.22	1.21	0.92
ACAP2	ACAP2;CENTB2;CNT-B2;KIAA0041	ArfGAP with coiled-coil, ankyrin repeat and PH domains 2	1.07	1.25	1.21	0.96
TMEM126A	TMEM126A;DKFZp586C1924;OPA7	transmembrane protein 126A	1.04	1.22	1.21	0.92
RAB40AL	RAB40AL;MGC119958;MGC119959;MGC119960;MGC119961;RAR2;RLGP	RAB40A, member RAS oncogene family-like	1.03	1.19	1.21	0.86
KRT12	KRT12;K12	keratin 12	1.02	1.26	1.21	0.91
HARS	HARS;FLJ20491;HRS	histidyl-tRNA synthetase	1.02	1.24	1.21	0.83
WRAP53	WRAP53;DKCB3;FLJ10385;TCAB1;WDR79	WD repeat containing, antisense to TP53	1.01	1.25	1.21	0.94
ECM1	ECM1	extracellular matrix protein 1	1	1.18	1.21	0.83
SYNM	SYNM;DMN;KIAA0353;SYN	synemin, intermediate filament protein	1	1.17	1.21	0.89
TBX19	TBX19;FLJ26302;FLJ34085;FLJ34543;TBS19;TPIT;dJ747L4.1	T-box 19	0.98	1.18	1.21	0.88
TUBD1	TUBD1;FLJ12709;TUBD	tubulin, delta 1	0.98	1.22	1.21	0.96
SLC30A7	SLC30A7;DKFZp686M0368;ZNT7;ZnT-7;ZnTL2	solute carrier family 30 (zinc transporter), member 7	0.98	1.1	1.21	0.98
SORCS1	SORCS1;FLJ41758;FLJ43475;FLJ44957;hSorCS	sortilin-related VPS10 domain containing receptor 1	0.97	1.15	1.21	1.03
C6orf201	C6orf201;MGC87625;dJ1013A10.5	chromosome 6 open reading frame 201	0.97	1.15	1.21	0.86
DNAJC22	DNAJC22;FLJ13236;wus	DnaJ (Hsp40) homolog, subfamily C, member 22	0.97	1.06	1.21	0.84
SLC5A9	SLC5A9;MGC132517;MGC132523;SGLT4	solute carrier family 5 (sodium/glucose cotransporter), member 9	0.96	1.17	1.21	0.8
PHF16	PHF16;JADE3;KIAA0215;MGC138748;MGC138749	PHD finger protein 16	0.96	1.1	1.21	0.8
RAPGEF1	RAPGEF1;C3G;DKFZp781P1719;GRF2	Rap guanine nucleotide exchange factor (GEF) 1	0.95	1.18	1.21	0.81
SEC61G	SEC61G;SSS1	Sec61 gamma subunit	0.95	1.04	1.21	0.82
PTPLB	PTPLB;HACD2	protein tyrosine phosphatase-like (proline instead of catalytic arginine), member b	0.95	1.06	1.21	0.81
CAV3	CAV3;LGMD1C;LQT9;MGC126100;MGC126101;MGC126129;VIP-21;VIP21	caveolin 3	0.94	1.1	1.21	0.95
MAPRE2	MAPRE2;EB1;EB2;RP1	microtubule-associated protein, RP/EB family, member 2	0.94	1.09	1.21	0.79
GLO1	GLO1;GLOD1;GLYI	glyoxalase I	0.94	1.1	1.21	0.82
CTC1	CTC1;AAF-132;AAF132;C17orf68;FLJ22170;MGC133331	CTS telomere maintenance complex component 1	0.94	1.02	1.21	0.8
LARP4B	LARP4B;DKFZp686M23113;KIAA0217;LARP5	La ribonucleoprotein domain family, member 4B	0.94	1.09	1.21	0.79
ACACB	ACACB;ACC2;ACCB;HACC275	acetyl-CoA carboxylase beta	0.92	1.07	1.21	0.77
ZFP2	ZFP2;FLJ21628;ZNF751	zinc finger protein 2 homolog (mouse)	0.91	1.04	1.21	0.84
SUPT7L	SUPT7L;KIAA0764;MGC90306;SPT7L;STAF65;STAF65(gamma);STAF65G;SUPT7H	suppressor of Ty 7 (S. cerevisiae)-like	0.91	1.13	1.21	0.84
TSPAN10	TSPAN10;FLJ39607;OCSP	tetraspanin 10	0.9	0.96	1.21	0.75
BAI1	BAI1;FLJ41988;GDAIF	brain-specific angiogenesis inhibitor 1	0.9	1.02	1.21	0.77
PLA2G2D	PLA2G2D;SPLASH;sPLA2S	phospholipase A2, group IID	0.9	1.1	1.21	0.88
FAN1	FAN1;DKFZp451H236;DKFZp686K16147;KIAA1018;MTMR15	FANCD2/FANCI-associated nuclease 1	0.9	1.03	1.21	0.79
FAM124B	FAM124B;FLJ22746	family with sequence similarity 124B	0.89	0.96	1.21	0.75
SLC29A3	SLC29A3;ENT3;FLJ11160;PHID	solute carrier family 29 (nucleoside transporters), member 3	0.88	1.09	1.21	0.86
ARRB2	ARRB2;ARB2;ARR2;BARR2;DKFZp686L0365	arrestin, beta 2	0.88	1.02	1.21	0.83
CPEB4	CPEB4;KIAA1673	cytoplasmic polyadenylation element binding protein 4	0.88	0.98	1.21	0.77
C19orf53	C19orf53;LYDG10	chromosome 19 open reading frame 53	0.88	1.02	1.21	0.82
BHLHE41	BHLHE41;BHLHB3;DEC2;SHARP1;hDEC2	basic helix-loop-helix family, member e41	0.88	1.05	1.21	0.79
LECT2	LECT2;MGC126628;chm-II;chm2	leukocyte cell-derived chemotaxin 2	0.86	1.08	1.21	0.81
S1PR3	S1PR3;EDG-3;EDG3;FLJ37523;FLJ93220;LPB3;MGC71696;S1P3	sphingosine-1-phosphate receptor 3	0.86	0.96	1.21	0.72
MRRF	MRRF;MRFF;MTRRF;RRF	mitochondrial ribosome recycling factor	0.86	1.03	1.21	0.86
HPDL	HPDL;4-HPPD-L;GLOXD1;MGC15668;RP4-534D1.1	4-hydroxyphenylpyruvate dioxygenase-like	0.85	1.03	1.21	0.74
C9orf50	C9orf50;FLJ35803	chromosome 9 open reading frame 50	0.85	0.92	1.21	0.72
TMEM18	TMEM18;DKFZp434C1714	transmembrane protein 18	1.12	1.24	1.22	0.99
PGAM2	PGAM2;GSD10;MGC88743;PGAM-M;PGAMM	phosphoglycerate mutase 2 (muscle)	1.06	1.19	1.22	0.9
THAP7	THAP7;MGC10963	THAP domain containing 7	1.02	1.25	1.22	0.85
ZNF805	ZNF805;DKFZp686P08106	zinc finger protein 805	1.01	1.09	1.22	0.85
PGS1	PGS1;DKFZp762M186;MGC131960	phosphatidylglycerophosphate synthase 1	1	1.19	1.22	0.84
FXR1	FXR1;FXR1P	fragile X mental retardation, autosomal homolog 1	1	1.19	1.22	0.99
FAM18B1	FAM18B1;FAM18B;FLJ46240;NPD008;YDR084C	family with sequence similarity 18, member B1	1	1.17	1.22	0.92
CCR10	CCR10;GPR2	chemokine (C-C motif) receptor 10	0.99	1.12	1.22	0.85
WNT5B	WNT5B;MGC2648	wingless-type MMTV integration site family, member 5B	0.99	1.15	1.22	0.84
PHF12	PHF12;FLJ34122;KIAA1523;MGC131914;PF1	PHD finger protein 12	0.99	1.15	1.22	0.83

CAPN9	CAPN9;GC36;nCL-4	calpain 9	0.97	1.16	1.22	0.87
CCDC90B	CCDC90B;MDS011;MDS025;MGC104239	coiled-coil domain containing 90B	0.97	1.16	1.22	0.86
C1orf158	C1orf158;DKFZp686C0236;MGC35194	chromosome 1 open reading frame 158	0.96	1.16	1.22	0.84
NGLY1	NGLY1;FLJ11005;FLJ12409;PNG1;PNGase	N-glycanase 1	0.96	1.1	1.22	0.87
WDR47	WDR47;FLJ90135;KIAA0893	WD repeat domain 47	0.94	1.03	1.22	0.8
TH1L	TH1L;NELF-C;NELF-D;TH1	TH1-like (Drosophila)	0.94	1.09	1.22	0.92
UGP2	UGP2;UDPG;UDPGP2;UGPP2;pHC379	UDP-glucose pyrophosphorylase 2	0.94	1.12	1.22	0.82
C20orf96	C20orf96;FLJ40075;dJ1103G7.2	chromosome 20 open reading frame 96	0.9	1.12	1.22	0.85
SRPRB	SRPRB;APMCF1	signal recognition particle receptor, B subunit	0.9	1.04	1.22	0.76
PRSS48	PRSS48;ESSPL	protease, serine, 48	0.9	1.08	1.22	0.81
ALOX5AP	ALOX5AP;FLAP	arachidonate 5-lipoxygenase-activating protein	0.89	1.04	1.22	0.75
HIST3H2A	HIST3H2A;MGC3165	histone cluster 3, H2a	0.89	1.12	1.22	0.75
GJC1	GJC1;CX45;DKFZp686P0738;GJA7	gap junction protein, gamma 1, 45kDa	0.89	1.08	1.22	0.8
BTN2A3P	BTN2A3P;BTN2A3	butyrophilin, subfamily 2, member A3, pseudogene	0.89	1.1	1.22	0.88
MIA	MIA;CD-RAP	melanoma inhibitory activity	0.88	1.1	1.22	0.84
DHRS7B	DHRS7B;DKFZp566O084;FLJ42061;FLJ99908;MGC8916;SDR32C1	dehydrogenase/reductase (SDR family) member 7B	0.88	1.04	1.22	0.78
RDH10	RDH10;SDR16C4	retinol dehydrogenase 10 (all-trans)	0.88	1.02	1.22	0.76
GNL3L	GNL3L;FLJ10613	guanine nucleotide binding protein-like 3 (nucleolar)-like	0.87	1.06	1.22	0.77
EMR3	EMR3	egf-like module containing, mucin-like, hormone receptor-like 3	0.86	0.96	1.22	0.73
TCF4	TCF4;E2-2;ITF-2;ITF2;MGC149723;MGC149724;PTHS;SEF-2;SEF2;SEF2-1;SEF2-1A;SEF2-1B;TCF-4;bHLHb19	transcription factor 4	0.86	0.98	1.22	0.79
SAMD9L	SAMD9L;C7orf6;DRIF2;FLJ39885;KIAA2005;UEF1	sterile alpha motif domain containing 9-like	0.86	0.95	1.22	0.74
DEPDC7	DEPDC7;TR2;dJ85M6.4	DEP domain containing 7	0.86	1	1.22	0.81
GNB3	GNB3	guanine nucleotide binding protein (G protein), beta polypeptide 3	0.86	0.94	1.22	0.78
C14orf118	C14orf118;FLJ10033;FLJ20689;MGC61896	chromosome 14 open reading frame 118	0.86	0.94	1.22	0.75
CELA2A	CELA2A;ELA2A;PE-1	chymotrypsin-like elastase family, member 2A	0.86	1.03	1.22	0.77
IL36B	IL36B;FIL1;FIL1-(ETA);FIL1H;FILI-(ETA);IL-1F8;IL-1H2;IL1-ETA;IL1F8;IL1H2;MGC126880;MGC126882	interleukin 36, beta	0.86	0.97	1.22	0.86
NKTR	NKTR;DKFZp686F1754;DKFZp686G0426;DKFZp686J06106;DKFZp686N24126;MGC90527;p104	natural killer-tumor recognition sequence	0.85	1.04	1.22	0.7
PARK2	PARK2;AR-JP;LPRS2;PDJ;PRKN	parkinson protein 2, E3 ubiquitin protein ligase (parkin)	1.19	1.35	1.23	1.01
CYP2C9	CYP2C9;CPC9;CYP2C;CYP2C10;CYPIIC9;MGC149605;MGC88320;P450IIC9	cytochrome P450, family 2, subfamily C, polypeptide 9	1.17	1.36	1.23	1.07
DIAPH3	DIAPH3;AN;AUNA1;DKFZp434C0931;DKFZp686A13178;DRF3;FLJ34705;NSDAN;diap3;mDia2	diaphanous homolog 3 (Drosophila)	1.1	1.34	1.23	0.99
CPN1	CPN1;CPN;FLJ40792;SCPN	carboxypeptidase N, polypeptide 1	1.04	1.27	1.23	0.95
TRIM43	TRIM43	tripartite motif containing 43	1.04	1.23	1.23	0.89
ADRA1B	ADRA1B;ADRA1;ALPHA1BAR	adrenergic, alpha-1B-, receptor	1.03	1.17	1.23	0.88
RHBDD1	RHBDD1;DKFZp547E052;MGC117258	rhomboid domain containing 1	1	1.1	1.23	0.86
C12orf4	C12orf4;FLJ21158;FLJ23899	chromosome 12 open reading frame 4	0.99	1.1	1.23	0.87
LAMB2	LAMB2;LAMS;NPHS5	laminin, beta 2 (laminin S)	0.99	1.22	1.23	0.95
CYS1	CYS1;FLJ33980	cystin 1	0.99	1.22	1.23	0.83
ZNF334	ZNF334	zinc finger protein 334	0.98	1.08	1.23	0.86
ABHD1	ABHD1;FLJ36128;LABH1	abhydrolase domain containing 1	0.98	1.19	1.23	0.83
AKAP4	AKAP4;AKAP 82;AKAP-4;AKAP82;CT99;FSC1;HI;PRKA4;hAKAP82;p82	A kinase (PRKA) anchor protein 4	0.97	1.21	1.23	0.81
LRRD1	LRRD1;FLJ34004;MGC178225;MGC178229	leucine-rich repeats and death domain containing 1	0.96	1.1	1.23	0.94
SHANK3	SHANK3;DEL22q13.3;KIAA1650;PROSAP2;PSAP2;SCZD15;SPANK-2	SH3 and multiple ankyrin repeat domains 3	0.94	1.12	1.23	0.82
PML	PML;MYL;PP8675;RNF71;TRIM19	promyelocytic leukemia	0.94	1.14	1.23	1.02
ASB13	ASB13;FLJ13134;MGC19879	ankyrin repeat and SOCS box containing 13	0.94	1.1	1.23	0.79
CASP8	CASP8;ALPS2B;CAP4;Casp-8;FLICE;FLJ17672;MACH;MCH5;MGC78473	caspase 8, apoptosis-related cysteine peptidase	0.93	1.12	1.23	0.84
KRTAP20-1	KRTAP20-1;KAP20.1	keratin associated protein 20-1	0.92	1.09	1.23	0.83
SERPINA1	SERPINA1;A1A;A1AT;AAT;MGC23330;MGC9222;PI;PI1;PRO2275;alpha1AT	serpin peptidase inhibitor, clade A (alpha-1 antiproteinase, antitrypsin), member 1	0.92	1.04	1.23	0.78
N6AMT1	N6AMT1;C21orf127;HEMK2;MGC19995;MTQ2;N6AMT	N-6 adenine-specific DNA methyltransferase 1 (putative)	0.9	1.04	1.23	0.82
IBTK	IBTK;BTKI;KIAA1417;MGC142256;MGC142258;RP1-93K22.1	inhibitor of Bruton agammaglobulinemia tyrosine kinase	0.9	1.14	1.23	0.78
GATC	GATC;15E1.2;FLJ37000;MGC129938	glutamyl-tRNA(Gln) amidotransferase, subunit C homolog (bacterial)	0.9	1.08	1.23	0.81
LMAN1	LMAN1;ERGIC-53;ERGIC53;F5F8D;FMFD1;MCFD1;MR60;gp58	lectin, mannose-binding, 1	0.9	1.04	1.23	0.88
DLX2	DLX2;TES-1;TES1	distal-less homeobox 2	0.88	1.06	1.23	0.79
TRAPPC5	TRAPPC5;MGC52424;TRS31	trafficking protein particle complex 5	0.88	1.1	1.23	0.84
OXTR	OXTR;OT-R	oxytocin receptor	0.87	0.99	1.23	0.75
COX6C	COX6C	cytochrome c oxidase subunit VIc	0.87	1.04	1.23	0.73
UQCRC2	UQCRC2;QCR2;UQCR2	ubiquinol-cytochrome c reductase core protein II	0.87	1.06	1.23	0.8
HIST1H3J	HIST1H3J;H3/j;H3FJ;HIST1H3A;HIST1H3B;HIST1H3C;HIST1H3D;HIST1H3E;HIST1H3F;HIST1H3G;HIST1H3H;HIST1H3I	histone cluster 1, H3j	0.86	1.04	1.23	0.78
NSL1	NSL1;C1orf48;DKFZp566O1646;MIS14	NSL1, MIND kinetochore complex component, homolog (S. cerevisiae)	0.86	1	1.23	0.81
CYP2F1	CYP2F1;C2F1;CYP2F;MGC126121	cytochrome P450, family 2, subfamily F, polypeptide 1	0.85	1	1.23	0.72
NAA16	NAA16;DKFZp686O08147;MGC40612;NARG1L	N(alpha)-acetyltransferase 16, NatA auxiliary subunit	1.08	1.31	1.24	1.02
ERI2	ERI2;EXOD1;KIAA1504;MGC16943	ERI1 exoribonuclease family member 2	1.04	1.15	1.24	0.9
C1orf54	C1orf54;FLJ23221	chromosome 1 open reading frame 54	1	1.23	1.24	1.02
TBC1D21	TBC1D21;MGC34741	TBC1 domain family, member 21	1	1.24	1.24	0.84
ACBD6	ACBD6;MGC2404	acyl-CoA binding domain containing 6	0.98	1.08	1.24	0.91
KCNS3	KCNS3;KV9.3;MGC9481	potassium voltage-gated channel, delayed-rectifier, subfamily S, member 3	0.96	1.17	1.24	0.91
SUDS3	SUDS3;FLJ00052;MGC104711;SAP45;SDS3	suppressor of defective silencing 3 homolog (S. cerevisiae)	0.96	1.07	1.24	0.84
SLC39A8	SLC39A8;BIGM103;LZT-Hs6;ZIP8	solute carrier family 39 (zinc transporter), member 8	0.96	1.15	1.24	0.87
NR3C2	NR3C2;FLJ41052;MCR;MGC133092;MLR;MR;NR3C2VIT	nuclear receptor subfamily 3, group C, member 2	0.94	1.14	1.24	0.84
TOPORS	TOPORS;LUN;P53BP3;RP31;TP53BPL	topoisomerase I binding, arginine/serine-rich, E3 ubiquitin protein ligase	0.94	1.1	1.24	0.8
ZNRF3	ZNRF3;BK747E2.3;RNF203	zinc and ring finger 3	0.93	1.1	1.24	0.8
MLL2	MLL2;AAD10;ALR;CAGL114;KMS;KMT2B;KMT2D;MLL4;TNRC21	myeloid/lymphoid or mixed-lineage leukemia 2	0.92	1.09	1.24	0.79
UACA	UACA;FLJ10128;KIAA1561;MGC141967;MGC141969;NUCLING	uveal autoantigen with coiled-coil domains and ankyrin repeats	0.92	1.16	1.24	0.88
ADC	ADC;AZI2;KIAA1945;ODC-p;ODC1L	arginine decarboxylase	0.91	1.14	1.24	0.88
A2ML1	A2ML1;CPAMD9;DKFZp686C1729;DKFZp686D2011;DKFZp686G1812;DKFZp686L1821;DKFZp686O1010;FLJ16045;FLJ25179;FLJ39129;FLJ41597;FLJ41598;FLJ41607	alpha-2-macroglobulin-like 1	0.9	1.1	1.24	0.82
PCDHGB1	PCDHGB1;MGC119466;MGC119467;MGC119469;PCDH-GAMMA-B1	protocadherin gamma subfamily B, 1	0.89	1.09	1.24	0.91
CPXM2	CPXM2;UNQ676	carboxypeptidase X (M14 family), member 2	0.88	1.07	1.24	0.8
PRMT5	PRMT5;HRMT1L5;IBP72;JBP1;SKB1;SKB1Hs	protein arginine methyltransferase 5	0.88	1.07	1.24	0.75
RLIM	RLIM;DKFZp686N06224;FLJ25923;FLJ41093;FLJ42887;FLJ45986;MGC15161;NY-REN-43;RNF12	ring finger protein, LIM domain interacting	0.88	1.04	1.24	0.75
SP140	SP140;LYSP100;LYSP100-A;LYSP100-B;MGC126440	SP140 nuclear body protein	0.86	1.08	1.24	0.72
MICAL1	MICAL1;DKFZp434B1517;FLJ11937;FLJ21739;MICAL;MICAL-1;NICAL	microtubule associated monoxygenase, calponin and LIM domain containing 1	0.86	1.02	1.24	0.79
KRTAP5-11	KRTAP5-11;KRTAP5-5;KRTAP5-6;KRTAP5.11	keratin associated protein 5-11	0.86	0.96	1.24	0.8
RASEF	RASEF;FLJ31614;RAB45	RAS and EF-hand domain containing	0.86	1.03	1.24	0.81
LHPP	LHPP;FLJ44846;FLJ46044;HDHD2B;MGC117251;MGC142189;MGC142191	phospholysine phosphohistidine inorganic pyrophosphate phosphatase	0.86	1.06	1.24	0.83
ME3	ME3;FLJ34862;NADP-ME	malic enzyme 3, NADP(+)-dependent, mitochondrial	0.85	0.98	1.24	0.78
AP3D1	AP3D1;ADTD;hBLVR	adaptor-related protein complex 3, delta 1 subunit	0.85	1.01	1.24	0.73
TCF21	TCF21;POD1;bHLHa23	transcription factor 21	0.84	0.7	0.81	0.55
PDYN	PDYN;ADCA;MGC26418;PENKB;SCA23	prodynorphin	0.83	0.68	0.81	0.49
RHOT2	RHOT2;ARHT2;C16orf39;MIRO-2;MIRO2;RASL	ras homolog gene family, member T2	0.83	0.68	0.81	0.46
SIRT1	SIRT1;SIR2L1	sirtuin 1	0.83	0.72	0.81	0.54
ITM2A	ITM2A;BRICD2A;E25A	integral membrane protein 2A	0.82	0.65	0.81	0.52
KIF17	KIF17;KIAA1405;KIF17B;KIF3X	kinesin family member 17	0.82	0.65	0.81	0.56
ATP5L	ATP5L;ATP5JG	ATP synthase, H+ transporting, mitochondrial Fo complex, subunit G	0.82	0.68	0.81	0.52
LOC652436	LOC652436	ENTREZ STATUS: DISCONTINUED	0.82	0.66	0.81	0.59
INHBA	INHBA;EDF;FRP	inhibin, beta A	0.81	0.66	0.81	0.53
C14orf135	C14orf135;FBP2;FLJ12799;FLJ38170	chromosome 14 open reading frame 135	0.81	0.64	0.81	0.48
UCMA	UCMA;C10orf49;GRP	upper zone of growth plate and cartilage matrix associated	0.81	0.72	0.81	0.52
SKA2	SKA2;FAM33A;FLJ12758;MGC110975	spindle and kinetochore associated complex subunit 2	0.8	0.66	0.81	0.51
NLRP9	NLRP9;CLR19.1;NALP9;NOD6;PAN12	NLR family, pyrin domain containing 9	0.78	0.68	0.81	0.51
C12orf68	C12orf68	chromosome 12 open reading frame 68	0.78	0.62	0.81	0.5
GPSM1	GPSM1;AGS3;DKFZp727I051	G-protein signaling modulator 1	0.78	0.66	0.81	0.49
PECR	PECR;DCRRP;HPDHASE;HSA250303;PVIARL;SDR29C1;TERP	peroxisomal trans-2-enoyl-CoA reductase	0.78	0.68	0.81	0.51
KCNG1	KCNG1;K13;KCNG;KV6.1;MGC12878;kH2	potassium voltage-gated channel, subfamily G, member 1	0.78	0.65	0.81	0.51
GPHN	GPHN;GEPH;GPH;GPHRYN;KIAA1385	gephyrin	0.78	0.64	0.81	0.43
TARDBP	TARDBP;ALS10;TDP-43	TAR DNA binding protein	0.78	0.64	0.81	0.5
C9ORF102	C9ORF102	ENTREZ STATUS: REMAPPED TO GENE ID: 375748	0.78	0.67	0.81	0.47
LYPLA1	LYPLA1;APT-1;LPL1;LYSOPLA;hAPT1	lysophospholipase I	0.77	0.68	0.81	0.48
CCNC	CCNC;CycC	cyclin C	0.76	0.6	0.81	0.52
SCNN1B	SCNN1B;BESC1;ENaCb;ENaCbeta;SCNEB	sodium channel, nonvoltage-gated 1, beta	0.74	0.62	0.81	0.48
FAM127B	FAM127B;CXX1b;DKFZp564B147;MAR8A;MGC8471	family with sequence similarity 127, member B	0.74	0.62	0.81	0.45
UPB1	UPB1;BUP1	ureidopropionase, beta	0.73	0.56	0.81	0.44
IL29	IL29;IFNL1;IL-29	interleukin 29 (interferon, lambda 1)	0.73	0.59	0.81	0.41
RGS8	RGS8;MGC119067;MGC119068;MGC119069	regulator of G-protein signaling 8	0.72	0.6	0.81	0.43
CIR1	CIR1;CIR	corepressor interacting with RBPJ, 1	0.72	0.64	0.81	0.46
BAIAP2L1	BAIAP2L1;FLJ42275;IRTKS	BAI1-associated protein 2-like 1	0.7	0.62	0.81	0.42
DCP1A	DCP1A;FLJ21691;HSA275986;Nbla00360;SMAD4IP1;SMIF	DCP1 decapping enzyme homolog A (S. cerevisiae)	0.68	0.61	0.81	0.41
LARP6	LARP6;ACHN;FLJ11196	La ribonucleoprotein domain family, member 6	0.66	0.52	0.81	0.4
RRNAD1	RRNAD1;C1orf66;CGI-41	ribosomal RNA adenine dimethylase domain containing 1	0.66	0.57	0.81	0.38
FSHR	FSHR;FSHRO;LGR1;MGC141667;MGC141668;ODG1	follicle stimulating hormone receptor	0.65	0.49	0.81	0.37
MTG1	MTG1;GTP;GTPBP7;RP11-108K14.2	mitochondrial GTPase 1 homolog (S. cerevisiae)	0.65	0.57	0.81	0.38
CLN8	CLN8;C8orf61;EPMR;FLJ39417	ceroid-lipofuscinosis, neuronal 8 (epilepsy, progressive with mental retardation)	0.64	0.52	0.81	0.4
COQ9	COQ9;C16orf49;DKFZp434K046	coenzyme Q9 homolog (S. cerevisiae)	0.64	0.57	0.81	0.38
UGGT1	UGGT1;FLJ21763;FLJ23671;FLJ23796;HUGT1;UGCGL1;UGT1	UDP-glucose glycoprotein glucosyltransferase 1	0.64	0.58	0.81	0.4
PRKD1	PRKD1;PKC-MU;PKCM;PKD;PRKCM	protein kinase D1	0.82	0.68	0.82	0.56
SOX10	SOX10;DOM;MGC15649;PCWH;WS2E;WS4;WS4C	SRY (sex determining region Y)-box 10	0.82	0.66	0.82	0.52
LTBP3	LTBP3;DKFZp586M2123;FLJ33431;FLJ39893;FLJ42533;FLJ44138;FLJ45576;LTBP-3;LTBP2;STHAG6;pp6425	latent transforming growth factor beta binding protein 3	0.82	0.68	0.82	0.46
NDUFA2	NDUFA2;B8;CD14;CIB8	NADH dehydrogenase (ubiquinone) 1 alpha subcomplex, 2, 8kDa	0.82	0.72	0.82	0.54
SLC25A4	SLC25A4;AAC1;ANT;ANT1;PEO2;PEO3;T1	solute carrier family 25 (mitochondrial carrier; adenine nucleotide translocator), member 4	0.82	0.66	0.82	0.49
ADA	ADA	adenosine deaminase	0.82	0.72	0.82	0.54
PTAFR	PTAFR;PAFR	platelet-activating factor receptor	0.81	0.62	0.82	0.47

DNAH3	DNAH3;DKFZp434N074;DLP3;DNAHC3B;FLJ31947;FLJ43919;FLJ43964;Hsadhc3	dynein, axonemal, heavy chain 3	0.8	0.64	0.82	0.47
ITPRIPL2	ITPRIPL2;FLJ22994;MGC126798;MGC126800	inositol 1,4,5-trisphosphate receptor interacting protein-like 2	0.8	0.68	0.82	0.55
TPST1	TPST1	tyrosylprotein sulfotransferase 1	0.79	0.71	0.82	0.53
TUBA3E	TUBA3E	tubulin, alpha 3e	0.78	0.68	0.82	0.48
CCDC111	CCDC111;FLJ33167	coiled-coil domain containing 111	0.78	0.7	0.82	0.47
XRCC6	XRCC6;CTC75;CTCBF;G22P1;KU70;ML8;TLAA	X-ray repair complementing defective repair in Chinese hamster cells 6	0.78	0.6	0.82	0.47
FMR1NB	FMR1NB;CT37;FLJ25736;NY-SAR-35;NYSAR35	fragile X mental retardation 1 neighbor	0.77	0.66	0.82	0.44
ZNF182	ZNF182;HHZ150;KOX14;MGC125383;MGC131713;ZNF21;Zfp182	zinc finger protein 182	0.77	0.66	0.82	0.47
MFNG	MFNG	MFNG O-fucosylpeptide 3-beta-N-acetylglucosaminyltransferase	0.76	0.62	0.82	0.48
BRWD1	BRWD1;C21orf107;FLJ43918;N143;WDR9	bromodomain and WD repeat domain containing 1	0.76	0.6	0.82	0.43
TNIP1	TNIP1;ABIN-1;KIAA0113;NAF1;VAN	TNFAIP3 interacting protein 1	0.76	0.64	0.82	0.47
USHBP1	USHBP1;AIEBP;FLJ38709;FLJ90681;MCC2	Usher syndrome 1C binding protein 1	0.75	0.6	0.82	0.46
MEST	MEST;DKFZp686L18234;MGC111102;MGC8703;PEG1	mesoderm specific transcript homolog (mouse)	0.74	0.68	0.82	0.47
TCP10	TCP10;MGC34049;TCP10A	t-complex 10 homolog (mouse)	0.72	0.62	0.82	0.42
ZNF543	ZNF543;DKFZp434H055;MGC119382;MGC119384	zinc finger protein 543	0.72	0.62	0.82	0.5
OIP5	OIP5;5730547N13Rik;CT86;LINT-25;MIS18B;MIS18beta;hMIS18beta	Opa interacting protein 5	0.72	0.57	0.82	0.41
POR	POR;CPR;CYPOR;DKFZp686G04235;FLJ26468;P450R	P450 (cytochrome) oxidoreductase	0.72	0.57	0.82	0.49
ULBP1	ULBP1;RAET1I	UL16 binding protein 1	0.71	0.59	0.82	0.4
GSTO2	GSTO2;bA127L20.1	glutathione S-transferase omega 2	0.7	0.56	0.82	0.48
ALG14	ALG14;MGC19780	asparagine-linked glycosylation 14 homolog (S. cerevisiae)	0.7	0.62	0.82	0.42
CATSPER4	CATSPER4;MGC149223	cation channel, sperm associated 4	0.68	0.55	0.82	0.42
CYP4B1	CYP4B1;CYPIVB1;P-450HP	cytochrome P450, family 4, subfamily B, polypeptide 1	0.67	0.55	0.82	0.43
IBSP	IBSP;BNSP;BSP;BSP-II;SP-II	integrin-binding sialoprotein	0.66	0.58	0.82	0.44
TCF12	TCF12;HEB;HTF4;HsT17266;bHLHb20	transcription factor 12	0.66	0.57	0.82	0.38
VDAC2	VDAC2;FLJ23841;POR	voltage-dependent anion channel 2	0.66	0.52	0.82	0.41
PKIG	PKIG;MGC126458;MGC126459	protein kinase (cAMP-dependent, catalytic) inhibitor gamma	0.64	0.5	0.82	0.36
HAGH	HAGH;GLO2;GLX2;GLXII;HAGH1	hydroxyacylglutathione hydrolase	0.62	0.55	0.82	0.41
ZNF608	ZNF608;DKFZp781C0723;MGC166851;NY-REN-36	zinc finger protein 608	0.62	0.52	0.82	0.4
EFCAB7	EFCAB7;DKFZp666D225;KIAA1799;RP4-534K7.1	EF-hand calcium binding domain 7	0.84	0.68	0.83	0.55
NUDT9	NUDT9;MGC3037;NUDT10	nudix (nucleoside diphosphate linked moiety X)-type motif 9	0.83	0.68	0.83	0.53
DBH	DBH;DBM	dopamine beta-hydroxylase (dopamine beta-monooxygenase)	0.82	0.68	0.83	0.51
PLA2G3	PLA2G3;GIII-SPLA2;SPLA2III	phospholipase A2, group III	0.82	0.65	0.83	0.51
ACBD7	ACBD7;FLJ38219;FLJ52263;MGC33893;bA455B2.2	acyl-CoA binding domain containing 7	0.82	0.71	0.83	0.47
APOL2	APOL2;APOL-II;APOL3	apolipoprotein L, 2	0.82	0.66	0.83	0.53
TGIF2	TGIF2	TGFB-induced factor homeobox 2	0.82	0.66	0.83	0.47
UNK	UNK;KIAA1753;ZC3H5;ZC3HDC5	unkempt homolog (Drosophila)	0.81	0.66	0.83	0.59
KIAA0101	KIAA0101;FLJ58702;NS5ATP9;OEATC-1;OEATC1;PAF;p15(PAF);p15PAF	KIAA0101	0.81	0.66	0.83	0.52
OR10Z1	OR10Z1;OR1-15	olfactory receptor, family 10, subfamily Z, member 1	0.8	0.68	0.83	0.52
DIRAS3	DIRAS3;ARHI;NOEY2	DIRAS family, GTP-binding RAS-like 3	0.8	0.66	0.83	0.51
ZBTB46	ZBTB46;BTBD4;FLJ13502;RINZF;ZNF340;dJ583P15.7;dJ583P15.8	zinc finger and BTB domain containing 46	0.8	0.69	0.83	0.49
RBMXL2	RBMXL2;HNRNPG-T;HNRNPGT;HNRPGT	RNA binding motif protein, X-linked-like 2	0.8	0.7	0.83	0.47
CNOT6L	CNOT6L;CCR4b	CCR4-NOT transcription complex, subunit 6-like	0.8	0.68	0.83	0.54
LRP10	LRP10;DKFZp564C1940;LRP9;MGC142274;MGC142276;MGC8675;MST087	low density lipoprotein receptor-related protein 10	0.8	0.66	0.83	0.45
NSUN4	NSUN4;MGC22960;RP4-603I14.2	NOP2/Sun domain family, member 4	0.8	0.69	0.83	0.51
POLD1	POLD1;CDC2;POLD	polymerase (DNA directed), delta 1, catalytic subunit 125kDa	0.8	0.66	0.83	0.49
C9orf79	C9orf79;FLJ35866	chromosome 9 open reading frame 79	0.78	0.62	0.83	0.45
PP8961	PP8961	ENTREZ STATUS: DISCONTINUED	0.78	0.64	0.83	0.51
IGSF8	IGSF8;CD316;CD81P3;EWI-2;EWI2;KCT-4;LIR-D1;PGRL	immunoglobulin superfamily, member 8	0.78	0.68	0.83	0.51
SULF2	SULF2;DKFZp313E091;FLJ44488;FLJ90554;HSULF-2;KIAA1247;MGC126411	sulfatase 2	0.78	0.72	0.83	0.49
BNIP3	BNIP3;NIP3	BCL2/adenovirus E1B 19kDa interacting protein 3	0.78	0.66	0.83	0.53
EMR2	EMR2;CD312;DKFZp781B135	egf-like module containing, mucin-like, hormone receptor-like 2	0.77	0.62	0.83	0.56
REPS1	REPS1;RALBP1	RALBP1 associated Eps domain containing 1	0.77	0.62	0.83	0.47
FGB	FGB;MGC104327;MGC120405	fibrinogen beta chain	0.76	0.68	0.83	0.51
DNHD1	DNHD1;C11orf47;CCDC35;DHCD1;DKFZp434G0812;DKFZp686J0796;DKFZp686N1238;DNHD1L;FLJ00251;FLJ32752;FLJ35709;FLJ39625;FLJ43897;FLJ46184;MGC133191	dynein heavy chain domain 1	0.76	0.62	0.83	0.45
SPIN4	SPIN4;FLJ44984;MGC133224	spindlin family, member 4	0.76	0.66	0.83	0.47
TSC22D1	TSC22D1;DKFZp686O19206;MGC17597;Ptg-2;TGFB1I4;TSC22	TSC22 domain family, member 1	0.76	0.62	0.83	0.43
COX7A2	COX7A2;COX7AL;COX7AL1;COXVIIAL;COXVIIa-L;MGC118950;MGC118951;MGC118952;MGC126875;MGC126877;VIIAL	cytochrome c oxidase subunit VIIa polypeptide 2 (liver)	0.76	0.62	0.83	0.48
DMD	DMD;BMD;CMD3B;DXS142;DXS164;DXS206;DXS230;DXS239;DXS268;DXS269;DXS270;DXS272	dystrophin	0.74	0.59	0.83	0.45
TP53AIP1	TP53AIP1;P53AIP1	tumor protein p53 regulated apoptosis inducing protein 1	0.74	0.6	0.83	0.46
PLA2R1	PLA2R1;CLEC13C;PLA2-R;PLA2G1R;PLA2IR;PLA2R	phospholipase A2 receptor 1, 180kDa	0.73	0.56	0.83	0.45
IPPK	IPPK;C9orf12;FLJ13163;INSP5K2;IP5K;IPK1;KIAA0699;bA476B13.1	inositol 1,3,4,5,6-pentakisphosphate 2-kinase	0.73	0.6	0.83	0.5
MAP3K1	MAP3K1;MAPKKK1;MEKK;MEKK1;SRXY6	mitogen-activated protein kinase kinase kinase 1	0.73	0.65	0.83	0.46
PPPDE2	PPPDE2;D15Wsu75e;DJ347H13.4;FAM152B;MGC138384	PPPDE peptidase domain containing 2	0.73	0.63	0.83	0.44
KCNIP2	KCNIP2;DKFZp566L1246;KCHIP2;MGC17241	Kv channel interacting protein 2	0.72	0.62	0.83	0.49
HIGD2A	HIGD2A;MGC2198	HIG1 hypoxia inducible domain family, member 2A	0.72	0.53	0.83	0.41
LDHB	LDHB;LDH-B;LDH-H;LDHBD;TRG-5	lactate dehydrogenase B	0.72	0.64	0.83	0.42
DEFB123	DEFB123;DEFB-23;ESC42-RELD	defensin, beta 123	0.7	0.58	0.83	0.39
AS3MT	AS3MT;CYT19	arsenic (+3 oxidation state) methyltransferase	0.7	0.64	0.83	0.47
PIGN	PIGN;MCAHS;MCD4;MDC4;MGC26427;PIG-N	phosphatidylinositol glycan anchor biosynthesis, class N	0.7	0.57	0.83	0.44
ING2	ING2;ING1L;p33ING2	inhibitor of growth family, member 2	0.7	0.55	0.83	0.43
C20orf20	C20orf20;Eaf7;FLJ10914;MRG15BP;MRGBP;URCC4	chromosome 20 open reading frame 20	0.7	0.62	0.83	0.43
SLC22A2	SLC22A2;MGC32628;OCT2	solute carrier family 22 (organic cation transporter), member 2	0.69	0.55	0.83	0.48
EXOSC8	EXOSC8;CIP3;EAP2;OIP2;RP11-421P11.3;RRP43;Rrp43p;bA421P11.3;p9	exosome component 8	0.69	0.57	0.83	0.46
CCDC103	CCDC103;FLJ13094;FLJ34211	coiled-coil domain containing 103	0.68	0.61	0.83	0.41
SIGLEC1	SIGLEC1;CD169;DKFZp667F058;FLJ00051;FLJ00055;FLJ00073;FLJ00411;FLJ32150;SIGLEC-1;SN;dJ1009E24.1	sialic acid binding Ig-like lectin 1, sialoadhesin	0.68	0.56	0.83	0.4
MMS22L	MMS22L;C6orf167;DKFZp686C20164;DKFZp781C2113;FLJ46180;KIAA1900;dJ39B17.2	MMS22-like, DNA repair protein	0.68	0.57	0.83	0.39
RFX5	RFX5	regulatory factor X, 5 (influences HLA class II expression)	0.67	0.56	0.83	0.45
ADCK5	ADCK5;FLJ35454;MGC126708	aarF domain containing kinase 5	0.66	0.59	0.83	0.42
ZMAT2	ZMAT2;FLJ31121;Ptg-12;Snu23;hSNU23	zinc finger, matrin-type 2	0.66	0.56	0.83	0.42
PATE1	PATE1;MGC129554;PATE	prostate and testis expressed 1	0.66	0.56	0.83	0.38
ZNF132	ZNF132;MGC126390;MGC126391;pHZ-12	zinc finger protein 132	0.65	0.55	0.83	0.42
RSPO1	RSPO1;CRISTIN3;FLJ40906;RSPO	R-spondin 1	0.65	0.48	0.83	0.38
TNFSF8	TNFSF8;CD153;CD30L;CD30LG;MGC138144	tumor necrosis factor (ligand) superfamily, member 8	0.64	0.55	0.83	0.41
PPP2CB	PPP2CB;PP2Abeta;PP2CB	protein phosphatase 2, catalytic subunit, beta isozyme	0.61	0.5	0.83	0.39
ABCF3	ABCF3;EST201864;FLJ11198	ATP-binding cassette, sub-family F (GCN20), member 3	0.84	0.76	0.84	0.57
SCT	SCT	secretin	0.83	0.68	0.84	0.52
KCNJ16	KCNJ16;BIR9;KIR5.1;MGC33717	potassium inwardly-rectifying channel, subfamily J, member 16	0.82	0.68	0.84	0.53
SLC22A1	SLC22A1;HOCT1;OCT1;oct1_cds	solute carrier family 22 (organic cation transporter), member 1	0.82	0.66	0.84	0.49
APC2	APC2;APCL	adenomatosis polyposis coli 2	0.82	0.67	0.84	0.57
TXNDC11	TXNDC11;EFP1	thioredoxin domain containing 11	0.82	0.68	0.84	0.5
STK4	STK4;DKFZp686A2068;KRS2;MST1;YSK3	serine/threonine kinase 4	0.82	0.73	0.84	0.52
U2AF1	U2AF1;DKFZp313J1712;FP793;RN;RNU2AF1;U2AF35;U2AFBP	U2 small nuclear RNA auxiliary factor 1	0.82	0.71	0.84	0.54
KCTD18	KCTD18;6530404F10Rik;FLJ31322;FLJ37818	potassium channel tetramerisation domain containing 18	0.81	0.7	0.84	0.53
SPR	SPR;SDR38C1	sepiapterin reductase (7,8-dihydrobiopterin:NADP+ oxidoreductase)	0.81	0.66	0.84	0.52
CACNG7	CACNG7	calcium channel, voltage-dependent, gamma subunit 7	0.8	0.71	0.84	0.55
MUC21	MUC21;C6orf205;KMQK697;MGC125609;MUC-21;bCX31G15.2	mucin 21, cell surface associated	0.8	0.7	0.84	0.47
GATAD2A	GATAD2A;FLJ20085;FLJ21017;p66alpha	GATA zinc finger domain containing 2A	0.8	0.69	0.84	0.5
DPF2	DPF2;MGC10180;REQ;UBID4;ubi-d4	D4, zinc and double PHD fingers family 2	0.8	0.66	0.84	0.6
SPTAN1	SPTAN1;EIEE5;FLJ17738;FLJ44613;NEAS;SPTA2	spectrin, alpha, non-erythrocytic 1 (alpha-fodrin)	0.8	0.7	0.84	0.53
DDX5	DDX5;DKFZp434E109;DKFZp686J01190;G17P1;HLR1;HUMP68;p68	DEAD (Asp-Glu-Ala-Asp) box polypeptide 5	0.8	0.67	0.84	0.5
LAG3	LAG3;CD223	lymphocyte-activation gene 3	0.79	0.7	0.84	0.46
ZFHX3	ZFHX3;ATBF1;ATBT;ZNF927	zinc finger homeobox 3	0.79	0.74	0.84	0.5
FASTK	FASTK;FAST	Fas-activated serine/threonine kinase	0.79	0.66	0.84	0.55
EFCAB6	EFCAB6;DJBP;FLJ23588;HSCBCIP1;KIAA1672;dJ185D5.1	EF-hand calcium binding domain 6	0.78	0.68	0.84	0.49
C2orf53	C2orf53;MGC44505	chromosome 2 open reading frame 53	0.78	0.7	0.84	0.49
TMSB15A	TMSB15A;TMSB15;TMSB15B;TMSL8;TMSNB;Tb15;TbNB	thymosin beta 15a	0.78	0.68	0.84	0.49
CNTNAP5	CNTNAP5;FLJ31966;caspr5	contactin associated protein-like 5	0.77	0.66	0.84	0.46
STMN2	STMN2;FLJ34868;FLJ50995;SCG10;SCGN10	stathmin-like 2	0.77	0.65	0.84	0.44
ISL1	ISL1;ISLET1;Isl-1	ISL LIM homeobox 1	0.77	0.64	0.84	0.57
APLN	APLN;APEL;XNPEP2	apelin	0.77	0.66	0.84	0.51
AK3L2	AK3L2	ENTREZ STATUS: REMAPPED TO GENE ID: 205	0.77	0.68	0.84	0.45
FAM46C	FAM46C;FLJ20202	family with sequence similarity 46, member C	0.76	0.67	0.84	0.52
CIB1	CIB1;CIB;KIP;KIP1;SIP2-28	calcium and integrin binding 1 (calmyrin)	0.76	0.68	0.84	0.45
EXOSC2	EXOSC2;RRP4;Rrp4p;hRrp4p;p7	exosome component 2	0.76	0.61	0.84	0.48
PDE6G	PDE6G;DKFZp686C0587;MGC125749;PDEG;RP57	phosphodiesterase 6G, cGMP-specific, rod, gamma	0.75	0.61	0.84	0.53
KCNK12	KCNK12;K2p12.1;THIK-2;THIK2	potassium channel, subfamily K, member 12	0.75	0.62	0.84	0.48
APEX1	APEX1;APE;APE1;APEN;APEX;APX;HAP1;REF1	APEX nuclease (multifunctional DNA repair enzyme) 1	0.75	0.68	0.84	0.51
ZNF767	ZNF767;FLJ12700;FLJ42870;MGC51818	zinc finger family member 767	0.74	0.68	0.84	0.45
ELK1	ELK1	ELK1, member of ETS oncogene family	0.74	0.64	0.84	0.5
STK32B	STK32B;HSA250839;STK32;STKG6;YANK2	serine/threonine kinase 32B	0.73	0.66	0.84	0.47
BCL10	BCL10;CARMEN;CIPER;CLAP;c-E10;mE10	B-cell CLL/lymphoma 10	0.73	0.66	0.84	0.5
WDR70	WDR70;FLJ10233	WD repeat domain 70	0.73	0.62	0.84	0.42
DSCR4	DSCR4;DCRB;DSCRB	Down syndrome critical region gene 4	0.72	0.64	0.84	0.45
RINL	RINL;FLJ44131;FLJ45909	Ras and Rab interactor-like	0.72	0.6	0.84	0.42
NGF	NGF;Beta-NGF;HSAN5;MGC161426;MGC161428;NGFB	nerve growth factor (beta polypeptide)	0.72	0.66	0.84	0.46
ATP8B2	ATP8B2;ATPID;DKFZp434M0219;KIAA1137	ATPase, class I, type 8B, member 2	0.72	0.56	0.84	0.45
MAPK1IP1L	MAPK1IP1L;C14orf32;MGC23138;MISS;c14_5346	mitogen-activated protein kinase 1 interacting protein 1-like	0.72	0.59	0.84	0.48
DTNA	DTNA;D18S892E;DRP3;DTN;DTN-A;FLJ96209;LVNC1	dystrobrevin, alpha	0.71	0.62	0.84	0.51
IKBKB	IKBKB;FLJ33771;FLJ36218;FLJ38368;FLJ40509;IKK-beta;IKK2;IKKB;MGC131801;NFKBIKB	inhibitor of kappa light polypeptide gene enhancer in B-cells, kinase beta	0.71	0.64	0.84	0.45

ZDHHC6	ZDHHC6;FLJ21952;ZNF376	zinc finger, DHHC-type containing 6	0.7	0.61	0.84	0.41
CERS2	CERS2;FLJ10243;L3;LASS2;MGC987;SP260;TMSG1	ceramide synthase 2	0.7	0.64	0.84	0.47
ASGR1	ASGR1;ASGPR;ASGPR1;CLEC4H1;HL-1	asialoglycoprotein receptor 1	0.68	0.6	0.84	0.4
C16orf7	C16orf7;ATP-BL	chromosome 16 open reading frame 7	0.68	0.63	0.84	0.44
LINC00482	LINC00482;C17orf55;FLJ39421;MGC120553;MGC120556	long intergenic non-protein coding RNA 482	0.68	0.62	0.84	0.41
CHAD	CHAD;SLRR4A	chondroadherin	0.67	0.59	0.84	0.44
ELP3	ELP3;FLJ10422;KAT9	elongation protein 3 homolog (S. cerevisiae)	0.67	0.56	0.84	0.41
RPF2	RPF2;BXDC1;FLJ21087;bA397G5.4	ribosome production factor 2 homolog (S. cerevisiae)	0.67	0.57	0.84	0.39
C5orf45	C5orf45;DKFZp686L2452;MGC65027;MGC78537	chromosome 5 open reading frame 45	0.66	0.6	0.84	0.44
TCF15	TCF15;EC2;PARAXIS;bHLHa40	transcription factor 15 (basic helix-loop-helix)	0.66	0.62	0.84	0.43
MRPL48	MRPL48;FLJ17047;FLJ99260;HSPC290;L48MT;MGC13323;MRP-L48	mitochondrial ribosomal protein L48	0.66	0.54	0.84	0.41
RSG1	RSG1;C1orf89;MGC10731;RP4-733M16.4	REM2 and RAB-like small GTPase 1	0.66	0.57	0.84	0.43
ASGR2	ASGR2;ASGP-R2;ASGPR2;CLEC4H2;FLJ60040;HBXBP;HL-2	asialoglycoprotein receptor 2	0.64	0.6	0.84	0.42
SOX8	SOX8;MGC24837	SRY (sex determining region Y)-box 8	0.64	0.6	0.84	0.41
DCAF17	DCAF17;C2orf37;DKFZp781C2086;FLJ13096;FLJ44210	DDB1 and CUL4 associated factor 17	0.64	0.58	0.84	0.39
ZNF134	ZNF134;MGC138499;MGC141970;pHZ-15	zinc finger protein 134	0.63	0.52	0.84	0.39
ALOX15B	ALOX15B;15-LOX-2	arachidonate 15-lipoxygenase, type B	0.62	0.53	0.84	0.42
EIF3K	EIF3K;EIF3-p28;EIF3S12;HSPC029;M9;MSTP001;PLAC-24;PLAC24;PRO1474;PTD001	eukaryotic translation initiation factor 3, subunit K	0.62	0.56	0.84	0.42
TST	TST;MGC19578;RDS	thiosulfate sulfurtransferase (rhodanese)	0.61	0.56	0.84	0.42
PFDN1	PFDN1;PDF;PFD1	prefoldin subunit 1	0.61	0.54	0.84	0.41
KRTAP12-2	KRTAP12-2;KAP12.2;KRTAP12.2	keratin associated protein 12-2	0.84	0.78	0.85	0.58
SH2D3A	SH2D3A;NSP1	SH2 domain containing 3A	0.84	0.74	0.85	0.5
TMEM214	TMEM214;FLJ20254;FLJ39682	transmembrane protein 214	0.84	0.7	0.85	0.56
UHMK1	UHMK1;DKFZp434C1613;FLJ23015;KIS;KIST	U2AF homology motif (UHM) kinase 1	0.84	0.72	0.85	0.59
FBL	FBL;FIB;FLRN;RNU3IP1	fibrillarin	0.84	0.69	0.85	0.53
NPL	NPL;C112;C1orf13;MGC149582;MGC61869;NAL;NPL1	N-acetylneuraminate pyruvate lyase (dihydrodipicolinate synthase)	0.82	0.68	0.85	0.53
FAM24B	FAM24B;DKFZp667I0323;MGC45962	family with sequence similarity 24, member B	0.82	0.7	0.85	0.48
PTRH1	PTRH1;C9orf115;MGC51999;PTH1	peptidyl-tRNA hydrolase 1 homolog (S. cerevisiae)	0.82	0.68	0.85	0.49
PBX2	PBX2;G17;HOX12;PBX2MHC	pre-B-cell leukemia homeobox 2	0.82	0.77	0.85	0.54
TMEM138	TMEM138	transmembrane protein 138	0.82	0.66	0.85	0.51
KIAA0368	KIAA0368;ECM29;FLJ22036;KIAA1962	KIAA0368	0.82	0.73	0.85	0.59
RIC8A	RIC8A;MGC104517;MGC131931;MGC148073;MGC148074;RIC8	resistance to inhibitors of cholinesterase 8 homolog A (C. elegans)	0.82	0.73	0.85	0.54
TMEM5	TMEM5;HP10481	transmembrane protein 5	0.81	0.75	0.85	0.55
ACP5	ACP5;MGC117378;SPENCDI;TRAP	acid phosphatase 5, tartrate resistant	0.8	0.62	0.85	0.47
POLI	POLI;RAD30B;RAD3OB	polymerase (DNA directed) iota	0.8	0.66	0.85	0.49
PIP5K1A	PIP5K1A	phosphatidylinositol-4-phosphate 5-kinase, type I, alpha	0.8	0.72	0.85	0.51
EPS15	EPS15;AF-1P;AF1P;MLLT5	epidermal growth factor receptor pathway substrate 15	0.8	0.66	0.85	0.5
C9orf102	C9orf102;FLJ37706;MGC30192;MGC43364;RAD26L;SR278	chromosome 9 open reading frame 102	0.78	0.7	0.85	0.47
SYN3	SYN3	synapsin III	0.77	0.68	0.85	0.46
PCDHB3	PCDHB3;PCDH-BETA3	protocadherin beta 3	0.76	0.66	0.85	0.44
RD3	RD3;C1orf36;LCA12	retinal degeneration 3	0.76	0.63	0.85	0.47
C6orf221	C6orf221;ECAT1;HYDM2	chromosome 6 open reading frame 221	0.76	0.7	0.85	0.46
SPIB	SPIB;SPI-B	Spi-B transcription factor (Spi-1/PU.1 related)	0.76	0.63	0.85	0.49
GINS4	GINS4;MGC14799;SLD5	GINS complex subunit 4 (Sld5 homolog)	0.76	0.68	0.85	0.49
NDUFA5	NDUFA5;B13;CI-13KD-B;CI-13kB;DKFZp781K1356;FLJ12147;NUFM;UQOR13	NADH dehydrogenase (ubiquinone) 1 alpha subcomplex, 5, 13kDa	0.76	0.68	0.85	0.45
NCS1	NCS1;DKFZp761L1223;FLUP;FREQ	neuronal calcium sensor 1	0.76	0.66	0.85	0.5
B4GALT2	B4GALT2;B4Gal-T2;B4Gal-T3;beta4Gal-T2	UDP-Gal:betaGlcNAc beta 1,4- galactosyltransferase, polypeptide 2	0.75	0.68	0.85	0.52
MBD3L2	MBD3L2	methyl-CpG binding domain protein 3-like 2	0.74	0.66	0.85	0.53
THEG	THEG;CT56;MGC26138	Theg homolog (mouse)	0.74	0.69	0.85	0.51
MX1	MX1;IFI-78K;IFI78;MX;MxA	myxovirus (influenza virus) resistance 1, interferon-inducible protein p78 (mouse)	0.73	0.58	0.85	0.46
C7orf61	C7orf61	chromosome 7 open reading frame 61	0.72	0.64	0.85	0.47
SLC12A7	SLC12A7;DKFZp434F076;KCC4	solute carrier family 12 (potassium/chloride transporters), member 7	0.72	0.58	0.85	0.42
FAM71B	FAM71B;MGC26988	family with sequence similarity 71, member B	0.71	0.65	0.85	0.5
C11orf67	C11orf67;CK067;FLJ21035;MGC3367	chromosome 11 open reading frame 67	0.71	0.66	0.85	0.51
NDNF	NDNF;C4orf31;FLJ23191	neuron-derived neurotrophic factor	0.71	0.53	0.85	0.42
OPTC	OPTC;OPT	opticin	0.7	0.55	0.85	0.47
ST8SIA5	ST8SIA5;MGC119670;MGC119671;SIAT8E;ST8SiaV	ST8 alpha-N-acetyl-neuraminide alpha-2,8-sialyltransferase 5	0.7	0.58	0.85	0.45
RARRES1	RARRES1;TIG1	retinoic acid receptor responder (tazarotene induced) 1	0.7	0.62	0.85	0.44
NDUFA4L2	NDUFA4L2;FLJ26118;NUOMS	NADH dehydrogenase (ubiquinone) 1 alpha subcomplex, 4-like 2	0.7	0.59	0.85	0.46
SNTG2	SNTG2;G2SYN;MGC133174;SYN5	syntrophin, gamma 2	0.69	0.65	0.85	0.47
GFOD1	GFOD1;ADG-90;C6orf114;FLJ20330;FLJ30569;MGC70653	glucose-fructose oxidoreductase domain containing 1	0.69	0.65	0.85	0.45
KRTAP4-4	KRTAP4-4;KAP4.13;KAP4.4;KRTAP4-13;KRTAP4.13;KRTAP4.4	keratin associated protein 4-4	0.68	0.6	0.85	0.4
TGIF1	TGIF1;HPE4;MGC39747;MGC5066;TGIF	TGFB-induced factor homeobox 1	0.68	0.59	0.85	0.4
C7orf25	C7orf25;FLJ21167;MGC2821	chromosome 7 open reading frame 25	0.68	0.6	0.85	0.46
CAMKV	CAMKV;1G5;MGC8407;VACAMKL	CaM kinase-like vesicle-associated	0.67	0.56	0.85	0.47
TLE4	TLE4;BCE-1;BCE1;E(spl);ESG;ESG4;GRG4	transducin-like enhancer of split 4 (E(sp1) homolog, Drosophila)	0.66	0.6	0.85	0.45
PARS2	PARS2;DKFZp727A071;MGC14416;MGC19467;MT-PRORS	prolyl-tRNA synthetase 2, mitochondrial (putative)	0.66	0.58	0.85	0.43
SLC29A2	SLC29A2;DER12;ENT2;HNP36	solute carrier family 29 (nucleoside transporters), member 2	0.66	0.56	0.85	0.41
OAS2	OAS2;MGC78578	2'-5'-oligoadenylate synthetase 2, 69/71kDa	0.65	0.6	0.85	0.44
SCAMP3	SCAMP3;C1orf3	secretory carrier membrane protein 3	0.65	0.52	0.85	0.45
EMX1	EMX1	empty spiracles homeobox 1	0.64	0.51	0.85	0.41
LRRFIP2	LRRFIP2;DKFZp434H2035;FLJ20248;FLJ22683;FLJ58304;HUFI-2	leucine rich repeat (in FLII) interacting protein 2	0.64	0.57	0.85	0.41
C10orf62	C10orf62;bA548K23.1	chromosome 10 open reading frame 62	0.63	0.52	0.85	0.39
IFT172	IFT172;DKFZp434A163;KIAA1179;SLB;osm-1;wim	intraflagellar transport 172 homolog (Chlamydomonas)	0.63	0.52	0.85	0.4
RBM38	RBM38;HSRNASEB;RNPC1;SEB4B;SEB4D;dJ800J21.2	RNA binding motif protein 38	0.62	0.5	0.85	0.39
APLP2	APLP2;APLP-2;APPH;APPL2;CDEBP	amyloid beta (A4) precursor-like protein 2	0.61	0.52	0.85	0.35
CRTAC1	CRTAC1;ASPIC;ASPIC1;CEP-68;FLJ10320	cartilage acidic protein 1	0.84	0.76	0.86	0.54
NKX2-3	NKX2-3;CSX3;NK2.3;NKX2.3;NKX2C;NKX4-3	NK2 homeobox 3	0.84	0.7	0.86	0.52
OSBPL6	OSBPL6;DKFZp686M06110;FLJ36583;FLJ41254;MGC59642;ORP6	oxysterol binding protein-like 6	0.84	0.74	0.86	0.51
POLK	POLK;DINB1;DINP;POLQ	polymerase (DNA directed) kappa	0.84	0.66	0.86	0.53
TMEM38B	TMEM38B;C9orf87;D4Ertd89e;FLJ10493;TRIC-B;TRICB;bA219P18.1	transmembrane protein 38B	0.84	0.74	0.86	0.56
SAMD8	SAMD8;FLJ25082;SMSr	sterile alpha motif domain containing 8	0.84	0.74	0.86	0.56
EML4	EML4;C2orf2;DKFZp686P18118;ELP120;EMAP-4;EMAPL4;FLJ10942;FLJ32318;ROPP120	echinoderm microtubule associated protein like 4	0.84	0.7	0.86	0.5
CBWD6	CBWD6;DKFZp686L07117;FLJ60409	COBW domain containing 6	0.84	0.7	0.86	0.53
WASF2	WASF2;SCAR2;WASF4;WAVE2;dJ393P12.2	WAS protein family, member 2	0.83	0.68	0.86	0.57
KIAA1279	KIAA1279;DKFZp586B0923;KBP;TTC20	KIAA1279	0.83	0.73	0.86	0.55
MYL12B	MYL12B;MLC-B;MRLC2	myosin, light chain 12B, regulatory	0.83	0.74	0.86	0.49
GRM1	GRM1;GPRC1A;GRM1A;MGLUR1;MGLUR1A;mGlu1	glutamate receptor, metabotropic 1	0.82	0.68	0.86	0.62
IFT74	IFT74;CCDC2;CMG-1;CMG1;FLJ22621;MGC111562	intraflagellar transport 74 homolog (Chlamydomonas)	0.82	0.78	0.86	0.52
UBAC2	UBAC2;FLJ26351;FLJ30001;FLJ30548;FLJ42413;MGC90487;PHGDHL1	UBA domain containing 2	0.82	0.76	0.86	0.53
EFHD1	EFHD1;DKFZp781H0842;FLJ13612;MST133;MSTP133;SWS2	EF-hand domain family, member D1	0.81	0.68	0.86	0.53
LEKR1	LEKR1;FLJ16641;FLJ37161	leucine, glutamate and lysine rich 1	0.8	0.62	0.86	0.48
SERPIND1	SERPIND1;D22S673;HC2;HCF2;HCII;HLS2;LS2	serpin peptidase inhibitor, clade D (heparin cofactor), member 1	0.8	0.68	0.86	0.51
AMDHD2	AMDHD2	amidohydrolase domain containing 2	0.8	0.66	0.86	0.49
ARRB1	ARRB1;ARB1;ARR1	arrestin, beta 1	0.8	0.62	0.86	0.52
SUZ12	SUZ12;CHET9;JJAZ1;KIAA0160	suppressor of zeste 12 homolog (Drosophila)	0.8	0.74	0.86	0.55
AIG1	AIG1;AIG-1;DKFZp686F03136;FLJ10485;dJ95L4.1	androgen-induced 1	0.79	0.66	0.86	0.5
OR52N2	OR52N2;OR11-57	olfactory receptor, family 52, subfamily N, member 2	0.78	0.64	0.86	0.54
CA9	CA9;CAIX;MN	carbonic anhydrase IX	0.78	0.68	0.86	0.46
CDC123	CDC123;C10orf7;D123;FLJ13863	cell division cycle 123 homolog (S. cerevisiae)	0.78	0.71	0.86	0.51
ZNF439	ZNF439;DKFZp571K0837	zinc finger protein 439	0.77	0.63	0.86	0.46
IL11RA	IL11RA;CRSDA;MGC2146	interleukin 11 receptor, alpha	0.77	0.67	0.86	0.52
FN3K	FN3K	fructosamine 3 kinase	0.77	0.66	0.86	0.54
PC	PC;PCB	pyruvate carboxylase	0.77	0.68	0.86	0.47
RAP1GAP2	RAP1GAP2;DKFZp686O238;GARNL4;KIAA1039;RAP1GA3	RAP1 GTPase activating protein 2	0.77	0.69	0.86	0.56
SLN	SLN;MGC12301;MGC125854;MGC125855	sarcolipin	0.76	0.67	0.86	0.45
SMARCD3	SMARCD3;BAF60C;CRACD3;MGC111010;Rsc6p	SWI/SNF related, matrix associated, actin dependent regulator of chromatin, subfamily d, member 3	0.76	0.7	0.86	0.52
PLEKHA8	PLEKHA8;DKFZp313M1034;DKFZp686J09164;FAPP2;MGC3358	pleckstrin homology domain containing, family A (phosphoinositide binding specific) member 8	0.76	0.68	0.86	0.5
RPS6KB1	RPS6KB1;PS6K;S6K;S6K1;STK14A;p70(S6K)-alpha;p70-S6K;p70-alpha	ribosomal protein S6 kinase, 70kDa, polypeptide 1	0.76	0.64	0.86	0.54
TC2N	TC2N;C14orf47;C2CD1;FLJ36557;MTAC2D1;Tac2-N	tandem C2 domains, nuclear	0.76	0.64	0.86	0.52
DPF3	DPF3;CERD4;FLJ14079	D4, zinc and double PHD fingers, family 3	0.75	0.64	0.86	0.58
PLA2G2F	PLA2G2F;FLJ25429;FLJ36326	phospholipase A2, group IIF	0.75	0.65	0.86	0.51
KCTD21	KCTD21;KCASH2	potassium channel tetramerisation domain containing 21	0.75	0.57	0.86	0.45
ABLIM1	ABLIM1;ABLIM;DKFZp781D0148;FLJ14564;KIAA0059;LIMAB1;LIMATIN;MGC1224;abLIM-1	actin binding LIM protein 1	0.75	0.68	0.86	0.5
MANF	MANF;ARMET;ARP;MGC142148;MGC142150	mesencephalic astrocyte-derived neurotrophic factor	0.75	0.67	0.86	0.51
ZNF561	ZNF561;MGC45408	zinc finger protein 561	0.74	0.66	0.86	0.5
LAMB3	LAMB3;BM600-125KDA;FLJ99565;LAM5;LAMNB1	laminin, beta 3	0.74	0.64	0.86	0.5
C19orf28	C19orf28;MGC20700;PP3501	chromosome 19 open reading frame 28	0.74	0.66	0.86	0.44
RPH3AL	RPH3AL;NOC2	rabphilin 3A-like (without C2 domains)	0.73	0.66	0.86	0.43
MND1	MND1;GAJ	meiotic nuclear divisions 1 homolog (S. cerevisiae)	0.72	0.62	0.86	0.45
OGG1	OGG1;HMMH;HOGG1;MUTM;OGH1	8-oxoguanine DNA glycosylase	0.72	0.66	0.86	0.43
SART3	SART3;DSAP1;KIAA0156;MGC138188;P100;RP11-13G14;TIP110;p110;p110(nrb)	squamous cell carcinoma antigen recognized by T cells 3	0.72	0.64	0.86	0.43
TNFRSF12A	TNFRSF12A;CD266;FN14;TWEAKR	tumor necrosis factor receptor superfamily, member 12A	0.72	0.64	0.86	0.48
MSH2	MSH2;COCA1;FCC1;HNPCC;HNPCC1;LCFS2	mutS homolog 2, colon cancer, nonpolyposis type 1 (E. coli)	0.72	0.62	0.86	0.46
KDELR2	KDELR2;ELP-1;ERD2.2;FLJ45532	KDEL (Lys-Asp-Glu-Leu) endoplasmic reticulum protein retention receptor 2	0.71	0.6	0.86	0.51
PPP4R2	PPP4R2;MGC131930;PP4R2	protein phosphatase 4, regulatory subunit 2	0.7	0.63	0.86	0.48
GJB6	GJB6;CX30;DFNA3;DFNA3B;DFNB1B;ED2;EDH;HED	gap junction protein, beta 6, 30kDa	0.69	0.58	0.86	0.43
PRAMEF6	PRAMEF6	PRAME family member 6	0.68	0.62	0.86	0.51
NOBOX	NOBOX;OG-2;OG2;OG2X;POF5;TCAG_12042	NOBOX oogenesis homeobox	0.68	0.62	0.86	0.44

VWA1	VWA1;DKFZp761O051;FLJ22215;WARP	von Willebrand factor A domain containing 1	0.68	0.64	0.86	0.49
XRCC3	XRCC3;CMM6	X-ray repair complementing defective repair in Chinese hamster cells 3	0.68	0.58	0.86	0.39
PRODH	PRODH;FLJ33744;HSPOX2;MGC148078;MGC148079;PIG6;POX;PRODH1;PRODH2;SCZD4;TP53I6	proline dehydrogenase (oxidase) 1	0.67	0.62	0.86	0.47
CXCR2	CXCR2;CD182;CDw128b;CMKAR2;IL8R2;IL8RA;IL8RB	chemokine (C-X-C motif) receptor 2	0.67	0.56	0.86	0.5
MARCO	MARCO;SCARA2	macrophage receptor with collagenous structure	0.66	0.57	0.86	0.38
FRS2	FRS2;FRS2A;FRS2alpha;SNT;SNT-1;SNT1	fibroblast growth factor receptor substrate 2	0.66	0.59	0.86	0.4
SRXN1	SRXN1;C20orf139;FLJ43353;Npn3;SRX1;YKL086W;dJ850E9.2	sulfiredoxin 1	0.66	0.56	0.86	0.41
CDC42SE1	CDC42SE1;SCIP1;SPEC1	CDC42 small effector 1	0.66	0.6	0.86	0.43
DECR1	DECR1;DECR;NADPH;SDR18C1	2,4-dienoyl CoA reductase 1, mitochondrial	0.66	0.55	0.86	0.43
APOL3	APOL3;APOLIII;CG121	apolipoprotein L, 3	0.65	0.55	0.86	0.41
MID1IP1	MID1IP1;FLJ10386;G12-like;MIG12;S14R;STRAIT11499;THRSPL	MID1 interacting protein 1 (gastrulation specific G12 homolog (zebrafish))	0.65	0.61	0.86	0.41
FGD4	FGD4;CMT4H;DKFZp313E1818;DKFZp434K1572;FLJ34370;FLJ42663;FRABP;MGC57222;ZFYVE6	FYVE, RhoGEF and PH domain containing 4	0.64	0.58	0.86	0.39
URM1	URM1;C9orf74;MGC2668	ubiquitin related modifier 1	0.64	0.54	0.86	0.4
FAM131B	FAM131B;KIAA0773	family with sequence similarity 131, member B	0.63	0.59	0.86	0.39
OR8B8	OR8B8;TPCR85	olfactory receptor, family 8, subfamily B, member 8	0.62	0.54	0.86	0.37
KCNQ4	KCNQ4;DFNA2;DFNA2A;KV7.4	potassium voltage-gated channel, KQT-like subfamily, member 4	0.62	0.53	0.86	0.41
EXOSC6	EXOSC6;EAP4;MTR3;Mtr3p;hMtr3p;p11	exosome component 6	0.62	0.5	0.86	0.43
LSM14B	LSM14B;C20orf40;FAM61B;FLJ25473;LSM13;MGC61931;RAP55B;bA11M20.3	LSM14B, SCD6 homolog B (S. cerevisiae)	0.61	0.57	0.86	0.38
KIAA0284	KIAA0284;FAM68C;MGC131679	KIAA0284	0.84	0.72	0.87	0.6
SCAMP4	SCAMP4;FLJ33847;FLJ90105;SCAMP-4	secretory carrier membrane protein 4	0.83	0.74	0.87	0.53
CYP21A2	CYP21A2;CA21H;CAH1;CPS1;CYP21;CYP21B;MGC150536;MGC150537;P450c21B	cytochrome P450, family 21, subfamily A, polypeptide 2	0.82	0.71	0.87	0.48
CES3	CES3;ES31;FLJ21736	carboxylesterase 3	0.82	0.67	0.87	0.53
WBP4	WBP4;FBP21;MGC117310	WW domain binding protein 4 (formin binding protein 21)	0.82	0.69	0.87	0.49
C19orf21	C19orf21;DKFZp686H18209	chromosome 19 open reading frame 21	0.82	0.67	0.87	0.57
KATNB1	KATNB1;KAT	katanin p80 (WD repeat containing) subunit B 1	0.82	0.74	0.87	0.56
IDS	IDS;MPS2;SIDS	iduronate 2-sulfatase	0.82	0.77	0.87	0.58
UBQLN2	UBQLN2;ALS15;CHAP1;DSK2;FLJ10167;FLJ56541;N4BP4;PLIC2	ubiquilin 2	0.82	0.74	0.87	0.56
C14orf183	C14orf183	chromosome 14 open reading frame 183	0.82	0.75	0.87	0.6
POTED	POTED;A26B3;ANKRD21;CT104.1;POTE;POTE-21;POTE21	POTE ankyrin domain family, member D	0.82	0.7	0.87	0.52
WDR31	WDR31;FLJ35921	WD repeat domain 31	0.81	0.66	0.87	0.52
PNMA5	PNMA5;KIAA1934	paraneoplastic antigen like 5	0.8	0.75	0.87	0.5
OTOP2	OTOP2	otopetrin 2	0.8	0.7	0.87	0.48
SPDEF	SPDEF;PDEF;RP11-375E1__A.3;bA375E1.3	SAM pointed domain containing ets transcription factor	0.8	0.68	0.87	0.53
GMEB2	GMEB2;KIAA1269;P79PIF;PIF79	glucocorticoid modulatory element binding protein 2	0.8	0.67	0.87	0.52
TGFB1I1	TGFB1I1;ARA55;HIC-5;HIC5;TSC-5	transforming growth factor beta 1 induced transcript 1	0.79	0.67	0.87	0.47
CDX4	CDX4	caudal type homeobox 4	0.78	0.68	0.87	0.53
C1orf123	C1orf123;FLJ20580	chromosome 1 open reading frame 123	0.78	0.72	0.87	0.51
TMLHE	TMLHE;BBOX2;FLJ10727;TMLD;TMLH;XAP130	trimethyllysine hydroxylase, epsilon	0.77	0.71	0.87	0.47
C11orf94	C11orf94	chromosome 11 open reading frame 94	0.77	0.7	0.87	0.46
TFF3	TFF3;ITF;P1B;TFI	trefoil factor 3 (intestinal)	0.76	0.68	0.87	0.52
PRPS1	PRPS1;ARTS;CMTX5;DFN2;DFNX1;KIAA0967;PPRibP;PRS-I;PRSI	phosphoribosyl pyrophosphate synthetase 1	0.76	0.72	0.87	0.51
NTF4	NTF4;GLC10;GLC1O;NT-4;NT-4/5;NT-5;NT4;NT5;NTF5	neurotrophin 4	0.75	0.64	0.87	0.5
FAM53A	FAM53A;DNTNP	family with sequence similarity 53, member A	0.75	0.7	0.87	0.47
GPR21	GPR21	G protein-coupled receptor 21	0.74	0.6	0.87	0.45
RARB	RARB;HAP;NR1B2;RRB2	retinoic acid receptor, beta	0.74	0.62	0.87	0.54
SPC25	SPC25;MGC22228;SPBC25;hSpc25	SPC25, NDC80 kinetochore complex component, homolog (S. cerevisiae)	0.74	0.63	0.87	0.48
POTEG	POTEG;A26C2;ACTBL1;CT104.4;POTE-14;POTE14;POTE14alpha;POTE22	POTE ankyrin domain family, member G	0.74	0.62	0.87	0.48
FLJ44290	FLJ44290	ENTREZ STATUS: REMAPPED TO GENE ID: 201625	0.74	0.66	0.87	0.45
NLGN4Y	NLGN4Y;KIAA0951	neuroligin 4, Y-linked	0.73	0.7	0.87	0.49
IL16	IL16;FLJ16806;FLJ42735;FLJ44234;LCF;NIL16;PRIL16;prIL-16	interleukin 16	0.72	0.66	0.87	0.5
BTBD6	BTBD6;BDPL	BTB (POZ) domain containing 6	0.72	0.67	0.87	0.47
METTL9	METTL9;DREV;DREV1;FLJ21912;PAP1	methyltransferase like 9	0.72	0.68	0.87	0.45
HPCAL4	HPCAL4;DKFZp761G122;HLP4	hippocalcin like 4	0.71	0.57	0.87	0.49
C7orf11	C7orf11;ABHS;ORF20	chromosome 7 open reading frame 11	0.7	0.6	0.87	0.41
GUSB	GUSB;BG;FLJ39445;MPS7	glucuronidase, beta	0.7	0.58	0.87	0.45
CAMK1	CAMK1;CAMKI;MGC120317;MGC120318	calcium/calmodulin-dependent protein kinase I	0.69	0.6	0.87	0.49
NFKBIL1	NFKBIL1;IKBL;LST1;NFKBIL	nuclear factor of kappa light polypeptide gene enhancer in B-cells inhibitor-like 1	0.69	0.58	0.87	0.43
NCL	NCL;C23;FLJ45706;FLJ59041	nucleolin	0.69	0.63	0.87	0.51
TMSB10	TMSB10;MIG12;TB10	thymosin beta 10	0.69	0.58	0.87	0.44
PHF8	PHF8;DKFZp686E0868;JHDM1F;KIAA1111;MRXSSD;ZNF422	PHD finger protein 8	0.68	0.58	0.87	0.52
RLN3	RLN3;H3;RXN3;ZINS4;insl7	relaxin 3	0.65	0.6	0.87	0.42
DDX3X	DDX3X;DBX;DDX14;DDX3;HLP2	DEAD (Asp-Glu-Ala-Asp) box polypeptide 3, X-linked	0.64	0.62	0.87	0.43
TRIM71	TRIM71;LIN-41;LIN41	tripartite motif containing 71	0.62	0.53	0.87	0.48
BAGE3	BAGE3;CT2.3	B melanoma antigen family, member 3	0.62	0.52	0.87	0.4
C1orf177	C1orf177;FLJ40201	chromosome 1 open reading frame 177	0.61	0.55	0.87	0.41
AFAP1L1	AFAP1L1;FLJ36748;MGC149773	actin filament associated protein 1-like 1	0.61	0.5	0.87	0.41
C1QTNF2	C1QTNF2;CTRP2;zacrp2	C1q and tumor necrosis factor related protein 2	0.84	0.76	0.88	0.6
VGLL4	VGLL4;KIAA0121;VGL-4	vestigial like 4 (Drosophila)	0.84	0.77	0.88	0.62
KIAA1797	KIAA1797;FLJ20375	KIAA1797	0.84	0.78	0.88	0.58
DMTF1	DMTF1;DMP1;DMTF;FLJ25188;FLJ41265;FLJ76054;hDMP1	cyclin D binding myb-like transcription factor 1	0.84	0.69	0.88	0.55
LOXL1	LOXL1;LOL;LOXL	lysyl oxidase-like 1	0.84	0.73	0.88	0.54
STAG3L1	STAG3L1;DKFZp434A0131;FLJ78546;MGC131759;MGC40269;STAG3L2;STAG3L3	stromal antigen 3-like 1	0.84	0.72	0.88	0.54
PHACTR3	PHACTR3;C20orf101;H17739;MGC117178;SCAPIN1;SCAPININ	phosphatase and actin regulator 3	0.82	0.64	0.88	0.54
KRTAP9-2	KRTAP9-2;KAP9.2;KRTAP9.2;MGC130013	keratin associated protein 9-2	0.82	0.8	0.88	0.58
EHD3	EHD3;PAST3	EH-domain containing 3	0.82	0.76	0.88	0.62
CRYZL1	CRYZL1;4P11;DKFZp434M035;QOH-1	crystallin, zeta (quinone reductase)-like 1	0.82	0.72	0.88	0.53
RAB4B	RAB4B;FLJ78649;MGC52123	RAB4B, member RAS oncogene family	0.82	0.68	0.88	0.53
CMTM1	CMTM1;CKLFH;CKLFH1;CKLFSF1;MGC71870	CKLF-like MARVEL transmembrane domain containing 1	0.82	0.75	0.88	0.53
CCDC25	CCDC25;FLJ10853	coiled-coil domain containing 25	0.82	0.76	0.88	0.58
CD2BP2	CD2BP2;FWP010;LIN1;PPP1R59;Snu40;U5-52K	CD2 (cytoplasmic tail) binding protein 2	0.82	0.72	0.88	0.5
SYAP1	SYAP1;DKFZp686K221;FLJ14495;FLJ44185	synapse associated protein 1	0.82	0.76	0.88	0.61
NRBP1	NRBP1;BCON3;FLJ27109;FLJ35541;MADM;MUDPNP;NRBP	nuclear receptor binding protein 1	0.82	0.7	0.88	0.63
NEFL	NEFL;CMT1F;CMT2E;FLJ53642;NF-L;NF68;NFL	neurofilament, light polypeptide	0.8	0.68	0.88	0.55
MEGF11	MEGF11;DKFZp434L121;KIAA1781	multiple EGF-like-domains 11	0.8	0.63	0.88	0.5
KCNQ3	KCNQ3;BFNC2;DKFZp686C0248;EBN2;FLJ37386;FLJ38392;KV7.3	potassium voltage-gated channel, KQT-like subfamily, member 3	0.8	0.73	0.88	0.56
AHDC1	AHDC1;CL23945;DJ159A19.3;RP1-159A19.1	AT hook, DNA binding motif, containing 1	0.8	0.63	0.88	0.49
ETNK2	ETNK2;EKI2;FLJ10761;HMFT1716	ethanolamine kinase 2	0.8	0.7	0.88	0.59
CRBN	CRBN;DKFZp781K0715;MGC27358;MRT2;MRT2A	cereblon	0.8	0.65	0.88	0.49
ZMYM2	ZMYM2;FIM;MYM;RAMP;SCLL;ZNF198	zinc finger, MYM-type 2	0.8	0.62	0.88	0.49
AOC3	AOC3;HPAO;SSAO;VAP-1;VAP1	amine oxidase, copper containing 3 (vascular adhesion protein 1)	0.79	0.65	0.88	0.51
ROBO3	ROBO3;FLJ21044;HGPPS;HGPS;RBIG1;RIG1	roundabout, axon guidance receptor, homolog 3 (Drosophila)	0.79	0.62	0.88	0.52
MYO5C	MYO5C;MGC74969	myosin VC	0.79	0.77	0.88	0.53
RBM10	RBM10;DXS8237E;FLJ40796;GPATC9;GPATCH9;KIAA0122;MGC1132;MGC997;S1-1;TARPS;ZRANB5	RNA binding motif protein 10	0.79	0.7	0.88	0.47
PDE4D	PDE4D;DKFZp686M11213;DPDE3;FLJ97311;HSPDE4D;PDE43;PDE4DN2;STRK1	phosphodiesterase 4D, cAMP-specific	0.78	0.66	0.88	0.58
C12orf43	C12orf43;FLJ12448	chromosome 12 open reading frame 43	0.78	0.65	0.88	0.53
IL1RAP	IL1RAP;C3orf13;FLJ37788;IL-1RAcP;IL1R3	interleukin 1 receptor accessory protein	0.78	0.67	0.88	0.52
TP53INP2	TP53INP2;C20orf110;DKFZp434B2411;DKFZp434O0827;DOR;FLJ21759;FLJ23500;PINH;dJ1181N3.1	tumor protein p53 inducible nuclear protein 2	0.78	0.68	0.88	0.51
RTKN	RTKN	rhotekin	0.78	0.65	0.88	0.55
C20orf4	C20orf4;DKFZp564N1363;bA234K24.2	chromosome 20 open reading frame 4	0.78	0.72	0.88	0.58
MTMR10	MTMR10;FLJ20313	myotubularin related protein 10	0.78	0.66	0.88	0.5
ATP6AP1L	ATP6AP1L;MGC138396	ATPase, H+ transporting, lysosomal accessory protein 1-like	0.78	0.72	0.88	0.58
C6orf195	C6orf195;FLJ31934;MGC120321;MGC120322;MGC120323;RP11-145H9.2;bA145H9.2	chromosome 6 open reading frame 195	0.77	0.66	0.88	0.53
ZNF699	ZNF699;FLJ38144;MGC129880;MGC129881;hang	zinc finger protein 699	0.77	0.6	0.88	0.47
PKD1L1	PKD1L1;PRO19563	polycystic kidney disease 1 like 1	0.76	0.6	0.88	0.48
KCNV2	KCNV2;KV11.1;Kv8.2;MGC120515;RCD3B	potassium channel, subfamily V, member 2	0.76	0.66	0.88	0.51
PPFIA4	PPFIA4	protein tyrosine phosphatase, receptor type, f polypeptide (PTPRF), interacting protein (liprin), alpha 4	0.76	0.62	0.88	0.47
MAN1B1	MAN1B1;ERMan1;MANA-ER;MRT15	mannosidase, alpha, class 1B, member 1	0.76	0.73	0.88	0.54
SWAP70	SWAP70;FLJ39540;KIAA0640;SWAP-70	SWAP switching B-cell complex 70kDa subunit	0.76	0.68	0.88	0.46
PCDHB8	PCDHB8;PCDH-BETA8;PCDH3I	protocadherin beta 8	0.75	0.64	0.88	0.48
PROC	PROC;APC;PC;PROC1	protein C (inactivator of coagulation factors Va and VIIIa)	0.75	0.62	0.88	0.46
SMPDL3B	SMPDL3B;ASML3B	sphingomyelin phosphodiesterase, acid-like 3B	0.75	0.64	0.88	0.49
OSM	OSM;MGC20461	oncostatin M	0.74	0.67	0.88	0.51
EPHB1	EPHB1;ELK;EPHT2;FLJ37986;Hek6;NET	EPH receptor B1	0.74	0.7	0.88	0.5
C11orf87	C11orf87;FLJ33767;LOH11CR1A	chromosome 11 open reading frame 87	0.74	0.68	0.88	0.55
UPRT	UPRT;DKFZp781E1243;FUR1;MGC23937;UPP	uracil phosphoribosyltransferase (FUR1) homolog (S. cerevisiae)	0.74	0.7	0.88	0.47
HSPG2	HSPG2;HSPG;PLC;PRCAN;SJA;SJS;SJS1	heparan sulfate proteoglycan 2	0.74	0.63	0.88	0.47
VTCN1	VTCN1;B7-H4;B7H4;B7S1;B7X;B7h.5;FLJ22418;PRO1291;VCTN1	V-set domain containing T cell activation inhibitor 1	0.74	0.7	0.88	0.51
GPR139	GPR139;GPRg1;PGR3	G protein-coupled receptor 139	0.73	0.62	0.88	0.56
KCNAB2	KCNAB2;AKR6A5;HKvbeta2;HKvbeta2.1;HKvbeta2.2;KCNA2B;KV-BETA-2;MGC117289	potassium voltage-gated channel, shaker-related subfamily, beta member 2	0.73	0.66	0.88	0.52
OGFRL1	OGFRL1;FLJ21079;MGC102783;dJ331H24.1	opioid growth factor receptor-like 1	0.73	0.66	0.88	0.48
MRGPRF	MRGPRF	ENTREZ STATUS: REMAPPED TO GENE ID: 116535	0.72	0.6	0.88	0.55
WNT10B	WNT10B;SHFM6;WNT-12	wingless-type MMTV integration site family, member 10B	0.72	0.63	0.88	0.47
SGMS1	SGMS1;MGC17342;MOB;MOB1;SMS1;TMEM23;hmob33	sphingomyelin synthase 1	0.72	0.62	0.88	0.48
HSBP1	HSBP1;DKFZp686D1664;DKFZp686O24200;NPC-A-13	heat shock factor binding protein 1	0.72	0.6	0.88	0.53
PAM	PAM;PAL;PHM	peptidylglycine alpha-amidating monooxygenase	0.72	0.64	0.88	0.43
CDC34	CDC34;E2-CDC34;UBC3;UBCH3;UBE2R1	cell division cycle 34 homolog (S. cerevisiae)	0.71	0.58	0.88	0.44
RBM28	RBM28;FLJ10377	RNA binding motif protein 28	0.71	0.6	0.88	0.48
B3GALT5	B3GALT5;B3GalT-V;B3GalTx;B3T5;GLCT5;beta3Gal-T5	UDP-Gal:betaGlcNAc beta 1,3-galactosyltransferase, polypeptide 5	0.7	0.66	0.88	0.5
ZZEF1	ZZEF1;FLJ10821;FLJ23789;FLJ45574;KIAA0399;MGC166929;ZZZ4	zinc finger, ZZ-type with EF-hand domain 1	0.7	0.62	0.88	0.45

C4orf34	C4orf34;FLJ13289	chromosome 4 open reading frame 34	0.7	0.6	0.88	0.45
ZBTB17	ZBTB17;MIZ-1;ZNF151;ZNF60;pHZ-67	zinc finger and BTB domain containing 17	0.7	0.68	0.88	0.5
PRR14	PRR14;DKFZp781A13198;MGC3121	proline rich 14	0.7	0.6	0.88	0.45
SLC30A6	SLC30A6;FLJ31101;MGC45055;MST103;MSTP103;ZNT6	solute carrier family 30 (zinc transporter), member 6	0.7	0.64	0.88	0.45
SLC39A1	SLC39A1;ZIP1;ZIRTL	solute carrier family 39 (zinc transporter), member 1	0.7	0.62	0.88	0.48
LCP1	LCP1;CP64;DKFZp781A23186;FLJ25423;FLJ26114;FLJ39956;L-PLASTIN;LC64P;LPL;PLS2	lymphocyte cytosolic protein 1 (L-plastin)	0.7	0.64	0.88	0.46
WDR63	WDR63;FLJ30067;NYD-SP29;RP11-507C22.2	WD repeat domain 63	0.69	0.6	0.88	0.45
ZC3H12B	ZC3H12B;CXorf32;MCPIP2	zinc finger CCCH-type containing 12B	0.68	0.62	0.88	0.46
ZNF441	ZNF441;FLJ38637	zinc finger protein 441	0.68	0.62	0.88	0.42
HSPA1L	HSPA1L;HSP70-1L;HSP70-HOM;HSP70T;hum70t	heat shock 70kDa protein 1-like	0.68	0.61	0.88	0.47
CXCR1	CXCR1;C-C;C-C-CKR-1;CD128;CD181;CDw128a;CKR-1;CMKAR1;IL8R1;IL8RA;IL8RBA	chemokine (C-X-C motif) receptor 1	0.68	0.58	0.88	0.53
DMGDH	DMGDH;DMGDHD;ME2GLYDH	dimethylglycine dehydrogenase	0.66	0.62	0.88	0.44
CAPRIN1	CAPRIN1;GPIAP1;GPIP137;M11S1;RNG105;p137GPI	cell cycle associated protein 1	0.66	0.62	0.88	0.44
BUD31	BUD31;EDG-2;EDG2;G10;MGC111202;YCR063W;fSAP17	BUD31 homolog (S. cerevisiae)	0.65	0.57	0.88	0.41
LAMC3	LAMC3;DKFZp434E202;OCCM	laminin, gamma 3	0.64	0.58	0.88	0.4
NUP214	NUP214;CAIN;CAN;D9S46E;MGC104525;N214	nucleoporin 214kDa	0.64	0.62	0.88	0.43
C9orf172	C9orf172	chromosome 9 open reading frame 172	0.64	0.6	0.88	0.48
SLC25A10	SLC25A10;DIC	solute carrier family 25 (mitochondrial carrier; dicarboxylate transporter), member 10	0.63	0.61	0.88	0.42
NHLRC1	NHLRC1;EPM2A;EPM2B;MALIN;MGC119262;MGC119264;MGC119265;bA204B7.2	NHL repeat containing 1	0.62	0.54	0.88	0.49
EPDR1	EPDR1;EPDR;MERP-1;MERP1;UCC1	ependymin related protein 1 (zebrafish)	0.62	0.53	0.88	0.4
RUVBL1	RUVBL1;ECP54;INO80H;NMP238;PONTIN;Pontin52;RVB1;TIH1;TIP49;TIP49A	RuvB-like 1 (E. coli)	0.62	0.54	0.88	0.41
ATP6V0D1	ATP6V0D1;ATP6D;ATP6DV;FLJ43534;P39;VATX;VMA6;VPATPD	ATPase, H+ transporting, lysosomal 38kDa, V0 subunit d1	0.62	0.6	0.88	0.44
KIR2DL1	KIR2DL1;CD158A;KIR-K64;KIR221;NKAT;NKAT1;p58.1	killer cell immunoglobulin-like receptor, two domains, long cytoplasmic tail, 1	0.61	0.52	0.88	0.4
C2CD4A	C2CD4A;DKFZp686O2263;FAM148A;NLF1	C2 calcium-dependent domain containing 4A	0.61	0.52	0.88	0.4
MYCL1	MYCL1;LMYC;MYCL;bHLHe38	v-myc myelocytomatosis viral oncogene homolog 1, lung carcinoma derived (avian)	0.84	0.74	0.89	0.55
SRC	SRC;ASV;SRC1;c-SRC;p60-Src	v-src sarcoma (Schmidt-Ruppin A-2) viral oncogene homolog (avian)	0.84	0.74	0.89	0.61
RING1	RING1;RING1A;RNF1	ring finger protein 1	0.84	0.74	0.89	0.67
TTC9C	TTC9C;MGC29649	tetratricopeptide repeat domain 9C	0.84	0.8	0.89	0.54
GTPBP5	GTPBP5;FLJ10741;MGC29512;ObgH1;dJ1005F21.2	GTP binding protein 5 (putative)	0.84	0.82	0.89	0.6
INSIG1	INSIG1;CL-6;CL6;MGC1405	insulin induced gene 1	0.84	0.76	0.89	0.51
MON2	MON2;KIAA1040;MGC35493	MON2 homolog (S. cerevisiae)	0.84	0.71	0.89	0.57
ACP1	ACP1;HAAP;MGC111030;MGC3499	acid phosphatase 1, soluble	0.84	0.74	0.89	0.58
MCM2	MCM2;BM28;CCNL1;CDCL1;D3S3194;KIAA0030;MGC10606;MITOTIN;cdc19	minichromosome maintenance complex component 2	0.84	0.74	0.89	0.55
DKC1	DKC1;CBF5;DKC;FLJ97620;NAP57;NOLA4;XAP101	dyskeratosis congenita 1, dyskerin	0.84	0.8	0.89	0.6
RRP7A	RRP7A;BK126B4.3;CGI-96;MGC150422;MGC150423	ribosomal RNA processing 7 homolog A (S. cerevisiae)	0.84	0.77	0.89	0.58
HDGFRP2	HDGFRP2;HDGF2;MGC2641	hepatoma-derived growth factor-related protein 2	0.84	0.72	0.89	0.55
YWHAQ	YWHAQ;14-3-3;1C5;HS1	tyrosine 3-monooxygenase/tryptophan 5-monooxygenase activation protein, theta polypeptide	0.83	0.74	0.89	0.58
HEATR7B2	HEATR7B2;DKFZp781F0822;FLJ40243	HEAT repeat family member 7B2	0.83	0.74	0.89	0.5
FAM117B	FAM117B;ALS2CR13;DKFZp686H01244;FLJ38771	family with sequence similarity 117, member B	0.82	0.7	0.89	0.56
TBRG4	TBRG4;CPR2;FASTKD4;KIAA0948	transforming growth factor beta regulator 4	0.82	0.66	0.89	0.55
B4GALT4	B4GALT4;B4Gal-T4;beta4Gal-T4	UDP-Gal:betaGlcNAc beta 1,4- galactosyltransferase, polypeptide 4	0.82	0.71	0.89	0.62
MRPL22	MRPL22;DKFZp781F1071;L22mt;MRP-L22;MRP-L25;RPML25	mitochondrial ribosomal protein L22	0.82	0.65	0.89	0.54
DHX16	DHX16;DBP2;DDX16;PRO2014;PRP8;PRPF2;Prp2	DEAH (Asp-Glu-Ala-His) box polypeptide 16	0.82	0.72	0.89	0.53
NPC1	NPC1;FLJ98532;NPC	Niemann-Pick disease, type C1	0.82	0.74	0.89	0.57
CRTAP	CRTAP;CASP;LEPREL3;OI7	cartilage associated protein	0.82	0.76	0.89	0.51
ARPC1A	ARPC1A;Arc40;SOP2Hs;SOP2L	actin related protein 2/3 complex, subunit 1A, 41kDa	0.82	0.75	0.89	0.5
PRKACG	PRKACG;KAPG;PKACg	protein kinase, cAMP-dependent, catalytic, gamma	0.81	0.7	0.89	0.63
TRPM6	TRPM6;CHAK2;HMGX;HOMG;HOMG1;HSH	transient receptor potential cation channel, subfamily M, member 6	0.8	0.74	0.89	0.57
GIMAP8	GIMAP8;DKFZp667I133;IAN-9;IAN6;IANT;MGC129545;hIAN6	GTPase, IMAP family member 8	0.8	0.68	0.89	0.53
OR2T3	OR2T3	olfactory receptor, family 2, subfamily T, member 3	0.8	0.74	0.89	0.56
RGS12	RGS12;DKFZp761K1617;DKFZp761K1817	regulator of G-protein signaling 12	0.8	0.72	0.89	0.49
OXSM	OXSM;FASN2D;FLJ20604;KASI;KS	3-oxoacyl-ACP synthase, mitochondrial	0.8	0.75	0.89	0.53
ZNF581	ZNF581;FLJ22550	zinc finger protein 581	0.8	0.74	0.89	0.5
GGA1	GGA1	golgi-associated, gamma adaptin ear containing, ARF binding protein 1	0.8	0.74	0.89	0.56
TRAFD1	TRAFD1;FLN29	TRAF-type zinc finger domain containing 1	0.8	0.77	0.89	0.51
CPNE1	CPNE1;COPN1;CPN1;MGC1142	copine I	0.8	0.67	0.89	0.57
ZNF507	ZNF507	zinc finger protein 507	0.8	0.72	0.89	0.5
WDR36	WDR36;DKFZp686I1650;GLC1G;TA-WDRP;TAWDRP;UTP21	WD repeat domain 36	0.8	0.74	0.89	0.5
CBS	CBS;HIP4	cystathionine-beta-synthase	0.8	0.67	0.89	0.53
DCXR	DCXR;DCR;HCR2;HCRII;KIDCR;P34H;SDR20C1;XR	dicarbonyl/L-xylulose reductase	0.8	0.66	0.89	0.58
OSBPL5	OSBPL5;FLJ42929;OBPH1;ORP5	oxysterol binding protein-like 5	0.79	0.63	0.89	0.5
CYP4F12	CYP4F12;F22329_1	cytochrome P450, family 4, subfamily F, polypeptide 12	0.78	0.69	0.89	0.51
TM7SF2	TM7SF2;ANG1;DHCR14A;NET47	transmembrane 7 superfamily member 2	0.78	0.68	0.89	0.58
CRABP2	CRABP2;CRABP-II;RBP6	cellular retinoic acid binding protein 2	0.78	0.66	0.89	0.52
B3GALNT1	B3GALNT1;B3GALT3;GLCT3;GLOB;Gb4Cer;P;P1;beta3Gal-T3;galT3	beta-1,3-N-acetylgalactosaminyltransferase 1 (globoside blood group)	0.77	0.74	0.89	0.55
TEAD2	TEAD2;ETF;TEF-4;TEF4	TEA domain family member 2	0.77	0.71	0.89	0.54
KIAA0889	KIAA0889;C20orf117;FLJ44670;SOGA;dJ132F21.1	KIAA0889	0.77	0.72	0.89	0.58
BHMT2	BHMT2;FLJ20001	betaine--homocysteine S-methyltransferase 2	0.76	0.63	0.89	0.55
FUT3	FUT3;CD174;FT3B;FucT-III;LE;Les;MGC131739	fucosyltransferase 3 (galactoside 3(4)-L-fucosyltransferase, Lewis blood group)	0.76	0.68	0.89	0.53
KLHDC7A	KLHDC7A;FLJ38753;RP11-422P22.2	kelch domain containing 7A	0.76	0.72	0.89	0.51
DGUOK	DGUOK;MTDPS3;dGK	deoxyguanosine kinase	0.76	0.72	0.89	0.52
ARFGAP3	ARFGAP3;ARFGAP1;FLJ45618	ADP-ribosylation factor GTPase activating protein 3	0.76	0.66	0.89	0.48
NHP2L1	NHP2L1;15.5K;FA-1;FA1;NHPX;OTK27;SNRNP15-5;SNU13;SPAG12;SSFA1	NHP2 non-histone chromosome protein 2-like 1 (S. cerevisiae)	0.74	0.68	0.89	0.51
CT47A8	CT47A8;CT47.8;CT47A1;CT47A10;CT47A11;CT47A12;CT47A2;CT47A3;CT47A4;CT47A5;CT47A6;CT47A7;CT47A9	cancer/testis antigen family 47, member A8	0.74	0.66	0.89	0.48
UBL3	UBL3;DKFZp434K151;FLJ32018;HCG-1;PNSC1	ubiquitin-like 3	0.73	0.68	0.89	0.55
FSD2	FSD2;RP11-127F21;SPRYD1	fibronectin type III and SPRY domain containing 2	0.72	0.62	0.89	0.47
ZNF286A	ZNF286A;KIAA1874;MGC149627;MGC156181;ZNF286	zinc finger protein 286A	0.72	0.68	0.89	0.47
NEK2	NEK2;HsPK21;NEK2A;NLK1	NIMA (never in mitosis gene a)-related kinase 2	0.72	0.7	0.89	0.49
NFRKB	NFRKB;DKFZp547B2013;INO80G	nuclear factor related to kappaB binding protein	0.72	0.72	0.89	0.49
PSMG2	PSMG2;CLAST3;HCCA3;HsT1707;MDS003;MGC15092;PAC2;TNFSF5IP1	proteasome (prosome, macropain) assembly chaperone 2	0.72	0.62	0.89	0.47
GRN	GRN;GEP;GP88;PCDGF;PEPI;PGRN	granulin	0.72	0.66	0.89	0.5
FAM134B	FAM134B;FLJ20152;FLJ22155;FLJ22179;JK-1;JK1	family with sequence similarity 134, member B	0.71	0.67	0.89	0.47
HIBADH	HIBADH;MGC40361;NS5ATP1	3-hydroxyisobutyrate dehydrogenase	0.7	0.68	0.89	0.51
NRAS	NRAS;ALPS4;N-ras;NRAS1;NS6	neuroblastoma RAS viral (v-ras) oncogene homolog	0.7	0.7	0.89	0.48
SLC44A2	SLC44A2;CTL2;DKFZp666A071;FLJ44586;PP1292	solute carrier family 44, member 2	0.7	0.68	0.89	0.5
NKX2-1	NKX2-1;BCH;BHC;NK-2;NKX2.1;NKX2A;TEBP;TITF1;TTF-1;TTF1	NK2 homeobox 1	0.69	0.6	0.89	0.52
KRT10	KRT10;BCIE;BIE;CK10;EHK;K10;KPP	keratin 10	0.68	0.62	0.89	0.47
STYK1	STYK1;DKFZp761P1010;NOK;SuRTK106	serine/threonine/tyrosine kinase 1	0.68	0.66	0.89	0.47
SHROOM4	SHROOM4;KIAA1202;SHAP	shroom family member 4	0.68	0.66	0.89	0.44
CYTH2	CYTH2;ARNO;CTS18;CTS18.1;PSCD2;PSCD2L;SEC7L;Sec7p-L;Sec7p-like	cytohesin 2	0.68	0.62	0.89	0.45
LMCD1	LMCD1	LIM and cysteine-rich domains 1	0.67	0.62	0.89	0.41
CLDN14	CLDN14;DFNB29	claudin 14	0.66	0.58	0.89	0.4
ABLIM2	ABLIM2;DKFZp761F129;FLJ26534;FLJ39684;KIAA1808;MGC141918	actin binding LIM protein family, member 2	0.66	0.6	0.89	0.46
MTIF3	MTIF3;FLJ33676;IF3mt	mitochondrial translational initiation factor 3	0.66	0.55	0.89	0.44
POU3F3	POU3F3;BRN1;OTF8	POU class 3 homeobox 3	0.66	0.6	0.89	0.4
SIRT3	SIRT3;SIR2L3	sirtuin 3	0.66	0.57	0.89	0.45
CHST12	CHST12;C4S-2;C4ST-2;C4ST2	carbohydrate (chondroitin 4) sulfotransferase 12	0.66	0.57	0.89	0.41
ESRRA	ESRRA;ERR1;ERRa;ERRalpha;ESRL1;NR3B1	estrogen-related receptor alpha	0.66	0.6	0.89	0.46
HIST2H2AA3	HIST2H2AA3;H2A;H2A.2;H2A/O;H2A/q;H2AFO;H2a-615;HIST2H2AA	histone cluster 2, H2aa3	0.66	0.61	0.89	0.45
ANXA2	ANXA2;ANX2;ANX2L4;CAL1H;LIP2;LPC2;LPC2D;P36;PAP-IV	annexin A2	0.66	0.62	0.89	0.5
KAT8	KAT8;FLJ14040;MOF;MYST1;hMOF	K(lysine) acetyltransferase 8	0.65	0.64	0.89	0.45
ERICH1	ERICH1	glutamate-rich 1	0.64	0.62	0.89	0.43
C17orf50	C17orf50	chromosome 17 open reading frame 50	0.64	0.54	0.89	0.41
SRD5A1	SRD5A1;S5AR 1	steroid-5-alpha-reductase, alpha polypeptide 1 (3-oxo-5 alpha-steroid delta 4-dehydrogenase alpha 1)	0.64	0.57	0.89	0.46
C7orf60	C7orf60;FLJ31818	chromosome 7 open reading frame 60	0.64	0.62	0.89	0.46
CHERP	CHERP;DAN16;SCAF6;SRA1	calcium homeostasis endoplasmic reticulum protein	0.64	0.6	0.89	0.42
U2AF2	U2AF2;U2AF65	U2 small nuclear RNA auxiliary factor 2	0.64	0.56	0.89	0.45
RHAG	RHAG;CD241;RH2;RH50A;Rh50;Rh50GP;SLC42A1	Rh-associated glycoprotein	0.62	0.58	0.89	0.45
SLC30A10	SLC30A10;DKFZp547M236;ZNT10;ZNT8;ZRC1;ZnT-10	solute carrier family 30, member 10	0.61	0.54	0.89	0.41
SH3BGR	SH3BGR;21-GARP	SH3 domain binding glutamic acid-rich protein	0.84	0.76	0.9	0.59
LGALS4	LGALS4;GAL4;L36LBP	lectin, galactoside-binding, soluble, 4	0.84	0.75	0.9	0.58
CEP57	CEP57;KIAA0092;MVA2;PIG8;TSP57	centrosomal protein 57kDa	0.84	0.78	0.9	0.58
SIRPB1	SIRPB1;CD172b;DKFZp686A05192;FLJ26614;SIRP-BETA-1	signal-regulatory protein beta 1	0.83	0.71	0.9	0.6
HNRNPH1	HNRNPH1;DKFZp686A15170;HNRPH;HNRPH1;hnRNPH	heterogeneous nuclear ribonucleoprotein H1 (H)	0.83	0.74	0.9	0.61
OR4F6	OR4F6;OR15-15;OR4F12	olfactory receptor, family 4, subfamily F, member 6	0.82	0.7	0.9	0.59
BEST1	BEST1;ARB;BEST;BMD;RP50;TU15B;VMD2	bestrophin 1	0.82	0.72	0.9	0.55
GNG7	GNG7;FLJ00058	guanine nucleotide binding protein (G protein), gamma 7	0.82	0.76	0.9	0.54
C16orf5	C16orf5;CDIP;I1	chromosome 16 open reading frame 5	0.82	0.81	0.9	0.59
ZNF609	ZNF609;KIAA0295;MGC164858	zinc finger protein 609	0.82	0.8	0.9	0.59
GALNT6	GALNT6;GALNAC-T6;GalNAcT6	UDP-N-acetyl-alpha-D-galactosamine:polypeptide N-acetylgalactosaminyltransferase 6 (GalNAc-T6)	0.82	0.82	0.9	0.57
ZNF780B	ZNF780B;ZNF779	zinc finger protein 780B	0.82	0.76	0.9	0.57
ID3	ID3;HEIR-1;bHLHb25	inhibitor of DNA binding 3, dominant negative helix-loop-helix protein	0.82	0.8	0.9	0.59
KDM1B	KDM1B;AOF1;C6orf193;DKFZp686I0412;FLJ33898;FLJ34109;FLJ43328;LSD2;bA204B7.3;dJ298J15.2	lysine (K)-specific demethylase 1B	0.82	0.73	0.9	0.65
NSUN5P1	NSUN5P1;FLJ99347;MGC129801;NSUN5B;WBSCR20B	NOP2/Sun domain family, member 5 pseudogene 1	0.82	0.72	0.9	0.54
BPY2C	BPY2C;BPY2;VCY2;VCY2C	basic charge, Y-linked, 2C	0.82	0.77	0.9	0.51
PADI6	PADI6	peptidyl arginine deiminase, type VI	0.81	0.78	0.9	0.58
SLC22A4	SLC22A4;MGC34546;MGC40524;OCTN1	solute carrier family 22 (organic cation/ergothioneine transporter), member 4	0.81	0.7	0.9	0.61
SKAP1	SKAP1;SCAP1;SKAP55	src kinase associated phosphoprotein 1	0.8	0.72	0.9	0.6
ABCC2	ABCC2;ABC30;CMOAT;DJS;KIAA1010;MRP2;cMRP	ATP-binding cassette, sub-family C (CFTR/MRP), member 2	0.8	0.68	0.9	0.54

FAM167A	FAM167A;C8orf13;D8S265;DKFZp761G151;MGC120649;MGC120650;MGC120651	family with sequence similarity 167, member A	0.8	0.76	0.9	0.5
CLDN9	CLDN9	claudin 9	0.8	0.68	0.9	0.57
ZNF266	ZNF266;HZF1	zinc finger protein 266	0.8	0.75	0.9	0.51
RNF146	RNF146;DKFZp434O1427;RP3-351K20.1;dJ351K20.1	ring finger protein 146	0.8	0.71	0.9	0.64
MPP1	MPP1;AAG12;DXS552E;EMP55;MRG1;PEMP	membrane protein, palmitoylated 1, 55kDa	0.8	0.71	0.9	0.59
RBM3	RBM3;IS1-RNPL;RNPL	RNA binding motif (RNP1, RRM) protein 3	0.8	0.66	0.9	0.52
TCOF1	TCOF1;MFD1;TCS1;treacle	Treacher Collins-Franceschetti syndrome 1	0.8	0.72	0.9	0.53
KLHL5	KLHL5;DKFZp586M1418;FLJ11313	kelch-like 5 (Drosophila)	0.8	0.76	0.9	0.51
SPNS1	SPNS1;FLJ38358;HSpin1;LAT;PP2030;SPIN1;SPINL;nrs	spinster homolog 1 (Drosophila)	0.8	0.7	0.9	0.55
SLC35A4	SLC35A4;MGC2541	solute carrier family 35, member A4	0.8	0.73	0.9	0.49
HS6ST2	HS6ST2;MGC130022;MGC130023	heparan sulfate 6-O-sulfotransferase 2	0.79	0.7	0.9	0.54
GNB2L1	GNB2L1;Gnb2-rs1;H12.3;HLC-7;PIG21;RACK1	guanine nucleotide binding protein (G protein), beta polypeptide 2-like 1	0.79	0.66	0.9	0.53
CT45A1	CT45A1;CT45;CT45-1;CT45.1	cancer/testis antigen family 45, member A1	0.79	0.64	0.9	0.53
OR56A3	OR56A3;OR56A2P;OR56A3P;OR56A6	olfactory receptor, family 56, subfamily A, member 3	0.78	0.69	0.9	0.52
CLGN	CLGN	calmegin	0.78	0.69	0.9	0.54
UPP1	UPP1;UDRPASE;UP;UPASE;UPP	uridine phosphorylase 1	0.78	0.7	0.9	0.55
DLX5	DLX5	distal-less homeobox 5	0.78	0.68	0.9	0.53
C15orf32	C15orf32;FLJ32831;MGC129836;MGC129837	chromosome 15 open reading frame 32	0.78	0.76	0.9	0.53
UBA6	UBA6;E1-L2;FLJ10808;FLJ23367;MOP-4;UBE1L2	ubiquitin-like modifier activating enzyme 6	0.78	0.65	0.9	0.49
ACTR3	ACTR3;ARP3	ARP3 actin-related protein 3 homolog (yeast)	0.78	0.75	0.9	0.6
C5orf54	C5orf54;Buster3	chromosome 5 open reading frame 54	0.77	0.74	0.9	0.52
ZNF563	ZNF563;FLJ34797	zinc finger protein 563	0.76	0.62	0.9	0.51
KCNJ15	KCNJ15;IRKK;KIR1.3;KIR4.2;MGC13584	potassium inwardly-rectifying channel, subfamily J, member 15	0.76	0.66	0.9	0.52
ZAR1	ZAR1	zygote arrest 1	0.76	0.7	0.9	0.5
KLF6	KLF6;BCD1;CBA1;COPEB;CPBP;DKFZp686N0199;GBF;PAC1;ST12;ZF9	Kruppel-like factor 6	0.76	0.65	0.9	0.51
IL17REL	IL17REL;FLJ41993	interleukin 17 receptor E-like	0.75	0.66	0.9	0.5
DBNDD1	DBNDD1;FLJ12582;MGC3101	dysbindin (dystrobrevin binding protein 1) domain containing 1	0.75	0.74	0.9	0.49
BTBD2	BTBD2	BTB (POZ) domain containing 2	0.75	0.69	0.9	0.53
STT3A	STT3A;FLJ27038;ITM1;MGC9042;STT3-A;TMC	STT3, subunit of the oligosaccharyltransferase complex, homolog A (S. cerevisiae)	0.75	0.69	0.9	0.53
SNAP47	SNAP47;C1orf142;DKFZp686M10160;FLJ12517;HEL170;SNAP-47;SVAP1	synaptosomal-associated protein, 47kDa	0.75	0.68	0.9	0.49
IL25	IL25;IL17E	interleukin 25	0.74	0.7	0.9	0.5
FBXW10	FBXW10;Fbw10;HREP;SM25H2;SM2SH2	F-box and WD repeat domain containing 10	0.74	0.64	0.9	0.46
BAHCC1	BAHCC1;BAHD2;FLJ23058;KIAA1447	BAH domain and coiled-coil containing 1	0.74	0.62	0.9	0.5
PWWP2B	PWWP2B;FLJ39621;FLJ46823;PWWP2;RP11-273H7.1;bA432J24.1;pp8607	PWWP domain containing 2B	0.74	0.63	0.9	0.54
HOXB7	HOXB7;HHO.C1;HOX2;HOX2C;Hox-2.3	homeobox B7	0.74	0.71	0.9	0.47
TAPBPL	TAPBPL;FLJ10143;TAPBP-R;TAPBPR	TAP binding protein-like	0.74	0.7	0.9	0.57
NOC4L	NOC4L;MGC3162;NET49;NOC4;UTP19	nucleolar complex associated 4 homolog (S. cerevisiae)	0.74	0.71	0.9	0.5
WFDC2	WFDC2;EDDM4;HE4;MGC57529;WAP5;dJ461P17.6	WAP four-disulfide core domain 2	0.74	0.64	0.9	0.49
MUT	MUT;MCM	methylmalonyl CoA mutase	0.74	0.64	0.9	0.55
GGCT	GGCT;C7orf24;CRF21;FLJ11717;GCTG;GGC;MGC3077	gamma-glutamylcyclotransferase	0.74	0.69	0.9	0.56
ARHGDIA	ARHGDIA;GDIA1;MGC117248;RHOGDI;RHOGDI-1	Rho GDP dissociation inhibitor (GDI) alpha	0.74	0.64	0.9	0.56
ITGAV	ITGAV;CD51;DKFZp686A08142;MSK8;VNRA	integrin, alpha V (vitronectin receptor, alpha polypeptide, antigen CD51)	0.74	0.72	0.9	0.47
PABPC4	PABPC4;APP-1;APP1;FLJ43938;PABP4;iPABP	poly(A) binding protein, cytoplasmic 4 (inducible form)	0.74	0.73	0.9	0.53
MYH9	MYH9;BDPLT6;DFNA17;EPSTS;FTNS;MGC104539;MHA;NMHC-II-A;NMMHCA	myosin, heavy chain 9, non-muscle	0.74	0.65	0.9	0.56
DFNA5	DFNA5;ICERE-1	deafness, autosomal dominant 5	0.73	0.67	0.9	0.48
NTHL1	NTHL1;NTH1;OCTS3	nth endonuclease III-like 1 (E. coli)	0.73	0.62	0.9	0.49
ICMT	ICMT;HSTE14;MGC39955;MST098;MSTP098;PCCMT;PCMT;PPMT	isoprenylcysteine carboxyl methyltransferase	0.73	0.71	0.9	0.54
SSU72	SSU72;FLJ13048;HSPC182;PNAS-120	SSU72 RNA polymerase II CTD phosphatase homolog (S. cerevisiae)	0.73	0.68	0.9	0.51
CT47A5	CT47A5;CT47.5;CT47A1;CT47A10;CT47A11;CT47A12;CT47A2;CT47A3;CT47A4;CT47A6;CT47A7;CT47A8;CT47A9	cancer/testis antigen family 47, member A5	0.73	0.66	0.9	0.45
NPAS4	NPAS4;Le-PAS;NXF;PASD10;bHLHe79	neuronal PAS domain protein 4	0.72	0.67	0.9	0.53
NHS	NHS;CXN;DKFZp781F2016;DKFZp781L0254;FLJ22511;SCML1	Nance-Horan syndrome (congenital cataracts and dental anomalies)	0.72	0.58	0.9	0.47
TSPAN32	TSPAN32;ART1;FLJ17158;FLJ97586;MGC22455;PHEMX;PHMX;TSSC6	tetraspanin 32	0.72	0.68	0.9	0.48
ZNF430	ZNF430;DKFZp762K012;FLJ13659;MGC133232	zinc finger protein 430	0.72	0.64	0.9	0.48
CAV1	CAV1;BSCL3;CGL3;MSTP085;VIP21	caveolin 1, caveolae protein, 22kDa	0.72	0.69	0.9	0.52
CDH3	CDH3;CDHP;HJMD;PCAD	cadherin 3, type 1, P-cadherin (placental)	0.72	0.66	0.9	0.47
SETD5	SETD5;DKFZp686J18276;FLJ10707;KIAA1757	SET domain containing 5	0.72	0.67	0.9	0.51
C1orf94	C1orf94;MGC15882	chromosome 1 open reading frame 94	0.71	0.7	0.9	0.46
MORF4	MORF4;CSR;CSRB;SEN;SEN1	mortality factor 4	0.7	0.64	0.9	0.51
DHX32	DHX32;DDX32;DHLP1;FLJ10694;FLJ10889	DEAH (Asp-Glu-Ala-His) box polypeptide 32	0.7	0.66	0.9	0.52
RABEP1	RABEP1;RAB5EP;RABPT5	rabaptin, RAB GTPase binding effector protein 1	0.7	0.64	0.9	0.43
IDH2	IDH2;D2HGA2;ICD-M;IDH;IDHM;IDP;IDPM;mNADP-IDH	isocitrate dehydrogenase 2 (NADP+), mitochondrial	0.7	0.58	0.9	0.52
FCGR3B	FCGR3B;CD16;CD16b;FCG3;FCGR3;FCR-10;FCRIII;FCRIIIb	Fc fragment of IgG, low affinity IIIb, receptor (CD16b)	0.7	0.58	0.9	0.52
CRAT	CRAT;CAT1	carnitine O-acetyltransferase	0.69	0.68	0.9	0.5
SCGB1A1	SCGB1A1;CC10;CC16;CCPBP;CCSP;UGB;UP-1;UP1	secretoglobin, family 1A, member 1 (uteroglobin)	0.69	0.58	0.9	0.5
SNUPN	SNUPN;KPNBL;RNUT1;Snurportin1	snurportin 1	0.69	0.61	0.9	0.43
SLC25A40	SLC25A40;MCFP	solute carrier family 25, member 40	0.69	0.65	0.9	0.46
EBLN2	EBLN2;EBLN-2;FLJ10213	endogenous Bornavirus-like nucleoprotein 2	0.69	0.62	0.9	0.43
DNAL4	DNAL4;PIG27	dynein, axonemal, light chain 4	0.68	0.65	0.9	0.49
ZFYVE26	ZFYVE26;DKFZp686F19106;DKFZp781H1112;FYVE-CENT;KIAA0321;SPG15	zinc finger, FYVE domain containing 26	0.68	0.64	0.9	0.48
DIABLO	DIABLO;DFNA64;DIABLO-S;FLJ10537;FLJ25049;SMAC;SMAC3	diablo, IAP-binding mitochondrial protein	0.68	0.59	0.9	0.44
C1orf85	C1orf85;MGC31963;NCU-G1	chromosome 1 open reading frame 85	0.68	0.66	0.9	0.44
GPANK1	GPANK1;ANKRD59;BAT4;D6S54E;G5;GPATCH10	G patch domain and ankyrin repeats 1	0.67	0.64	0.9	0.45
TRIM69	TRIM69;HSD34;RNF36;Trif	tripartite motif containing 69	0.66	0.59	0.9	0.53
S100A6	S100A6;2A9;5B10;CABP;CACY;PRA	S100 calcium binding protein A6	0.66	0.63	0.9	0.47
FNDC1	FNDC1;AGS8;FNDC2;KIAA1866;MEL4B3;bA243O10.1;dJ322A24.1	fibronectin type III domain containing 1	0.65	0.6	0.9	0.43
WDR91	WDR91	WD repeat domain 91	0.65	0.62	0.9	0.41
PAK7	PAK7;KIAA1264;MGC26232;PAK5	p21 protein (Cdc42/Rac)-activated kinase 7	0.64	0.63	0.9	0.45
PCDHGC5	PCDHGC5;MGC138286;MGC138288;PCDH-GAMMA-C5	protocadherin gamma subfamily C, 5	0.64	0.6	0.9	0.41
LRRN2	LRRN2;FIGLER7;GAC1;LRANK1;LRRN5	leucine rich repeat neuronal 2	0.64	0.62	0.9	0.41
MARS	MARS;FLJ35667;METRS;MRS;MTRNS	methionyl-tRNA synthetase	0.64	0.57	0.9	0.42
SSR1	SSR1;DKFZp781N23103;FLJ14232;FLJ22100;FLJ23034;FLJ78242;FLJ93042;TRAPA	signal sequence receptor, alpha	0.64	0.6	0.9	0.41
RAB19	RAB19;RAB19B	RAB19, member RAS oncogene family	0.63	0.58	0.9	0.42
LUC7L3	LUC7L3;CRA;CREAP-1;CROP;LUC7A;OA48-18;hLuc7A	LUC7-like 3 (S. cerevisiae)	0.63	0.5	0.9	0.4
FCGR3A	FCGR3A;CD16;CD16A;FCG3;FCGR3;FCGRIII;FCR-10;FCRIII;FCRIIIA;IGFR3	Fc fragment of IgG, low affinity IIIa, receptor (CD16a)	0.62	0.52	0.9	0.42
ITGA2B	ITGA2B;BDPLT2;CD41;CD41B;GP2B;GPIIb;GT;GTA;HPA3	integrin, alpha 2b (platelet glycoprotein IIb of IIb/IIIa complex, antigen CD41)	0.62	0.55	0.9	0.41
KIAA1161	KIAA1161;NET37	KIAA1161	0.62	0.58	0.9	0.41
PPP3CC	PPP3CC;CALNA3;CNA3;PP2Bgamma	protein phosphatase 3, catalytic subunit, gamma isozyme	0.62	0.54	0.9	0.43
TIMELESS	TIMELESS;FLJ12640;FLJ20714;TIM;TIM1;hTIM	timeless homolog (Drosophila)	0.62	0.55	0.9	0.44
APPL1	APPL1;APPL;DIP13alpha	adaptor protein, phosphotyrosine interaction, PH domain and leucine zipper containing 1	0.62	0.6	0.9	0.46
RXRA	RXRA;FLJ00280;FLJ00318;FLJ16020;FLJ16733;MGC102720;NR2B1	retinoid X receptor, alpha	0.61	0.58	0.9	0.39
ADH1B	ADH1B;ADH2;DKFZp686C06125	alcohol dehydrogenase 1B (class I), beta polypeptide	0.84	0.74	0.91	0.64
KCNH8	KCNH8;ELK;ELK1;Kv12.1;elk3	potassium voltage-gated channel, subfamily H (eag-related), member 8	0.84	0.76	0.91	0.63
CHRNA10	CHRNA10	cholinergic receptor, nicotinic, alpha 10	0.84	0.7	0.91	0.63
SLCO2A1	SLCO2A1;MATR1;OATP2A1;PGT;SLC21A2	solute carrier organic anion transporter family, member 2A1	0.84	0.85	0.91	0.59
GNPDA2	GNPDA2;GNP2;SB52	glucosamine-6-phosphate deaminase 2	0.84	0.8	0.91	0.53
UNC93B1	UNC93B1;MGC126617;UNC93;UNC93B;Unc-93B1	unc-93 homolog B1 (C. elegans)	0.84	0.72	0.91	0.61
TMUB1	TMUB1;C7orf21;MGC5442	transmembrane and ubiquitin-like domain containing 1	0.84	0.84	0.91	0.56
MSH6	MSH6;GTBP;HNPCC5;HSAP	mutS homolog 6 (E. coli)	0.84	0.7	0.91	0.62
ARHGAP11A	ARHGAP11A;KIAA0013;MGC70740	Rho GTPase activating protein 11A	0.84	0.74	0.91	0.56
LOC728919	LOC728919	ENTREZ STATUS: DISCONTINUED	0.84	0.75	0.91	0.67
ZNF14	ZNF14;GIOT-4;KOX6	zinc finger protein 14	0.83	0.79	0.91	0.53
ANGEL2	ANGEL2;FLJ12793;KIAA0759L	angel homolog 2 (Drosophila)	0.83	0.77	0.91	0.57
LRIT1	LRIT1;DKFZp434K091;FIGLER9;LRRC21;PAL	leucine-rich repeat, immunoglobulin-like and transmembrane domains 1	0.82	0.79	0.91	0.64
SYCE1	SYCE1;C10orf94;CT76;FLJ99976	synaptonemal complex central element protein 1	0.82	0.7	0.91	0.57
STAT2	STAT2;ISGF-3;MGC59816;P113;STAT113	signal transducer and activator of transcription 2, 113kDa	0.82	0.68	0.91	0.61
GATA6	GATA6	GATA binding protein 6	0.82	0.74	0.91	0.55
TADA2A	TADA2A;ADA2;ADA2A;FLJ12705;TADA2L;hADA2	transcriptional adaptor 2A	0.82	0.72	0.91	0.53
CCDC28B	CCDC28B;MGC1203;MGC16441;RP4-622L5.5	coiled-coil domain containing 28B	0.81	0.66	0.91	0.51
FYCO1	FYCO1;CATC2;DKFZp779K1152;FLJ13335;MGC126517;MGC126519;RUFY3;ZFYVE7	FYVE and coiled-coil domain containing 1	0.81	0.72	0.91	0.57
SLC39A3	SLC39A3;ZIP3	solute carrier family 39 (zinc transporter), member 3	0.81	0.68	0.91	0.54
GTF2E2	GTF2E2;FE;TF2E2;TFIIE-B	general transcription factor IIE, polypeptide 2, beta 34kDa	0.81	0.74	0.91	0.57
EIF3L	EIF3L;EIF3EIP;EIF3S11;EIF3S6IP;HSPC021;HSPC025;MSTP005	eukaryotic translation initiation factor 3, subunit L	0.81	0.76	0.91	0.55
SH3TC1	SH3TC1;FLJ20356;FLJ32999;FLJ36243;FLJ46394	SH3 domain and tetratricopeptide repeats 1	0.8	0.71	0.91	0.53
PI16	PI16;CRISP9;DKFZp586B1817;MGC45378;MSMBBP;PSPBP	peptidase inhibitor 16	0.8	0.76	0.91	0.5
KLF9	KLF9;BTEB;BTEB1	Kruppel-like factor 9	0.8	0.7	0.91	0.55
MTERF	MTERF;MGC131634	mitochondrial transcription termination factor	0.8	0.76	0.91	0.5
EPHA1	EPHA1;EPH;EPHT;EPHT1;MGC163163	EPH receptor A1	0.8	0.8	0.91	0.56
ARPC3	ARPC3;ARC21;p21-Arc	actin related protein 2/3 complex, subunit 3, 21kDa	0.8	0.72	0.91	0.53
DNAAF2	DNAAF2;C14orf104;CILD10;FLJ10563;KTU	dynein, axonemal, assembly factor 2	0.8	0.76	0.91	0.57
RBP7	RBP7;CRBP4;CRBPIV;MGC70641	retinol binding protein 7, cellular	0.78	0.69	0.91	0.54
SLC25A16	SLC25A16;D10S105E;GDA;GDC;HGT.1;MGC39851;ML7;hML7	solute carrier family 25 (mitochondrial carrier; Graves disease autoantigen), member 16	0.78	0.79	0.91	0.54
PRKCD	PRKCD;MAY1;MGC49908;PKCD;nPKC-delta	protein kinase C, delta	0.78	0.76	0.91	0.54
RNF10	RNF10;KIAA0262;MGC126758;MGC126764;RIE2	ring finger protein 10	0.78	0.7	0.91	0.63
TAF15	TAF15;Npl3;RBP56;TAF2N;TAFII68;hTAFII68	TAF15 RNA polymerase II, TATA box binding protein (TBP)-associated factor, 68kDa	0.78	0.7	0.91	0.54
LMOD1	LMOD1;1D;64kD;D1;FLJ55689;SM-LMOD	leiomodin 1 (smooth muscle)	0.77	0.73	0.91	0.55
SERTAD4	SERTAD4;DJ667H12.2	SERTA domain containing 4	0.77	0.72	0.91	0.55
PITX1	PITX1;BFT;CCF;POTX;PTX1	paired-like homeodomain 1	0.77	0.74	0.91	0.57
NKX6-3	NKX6-3;FLJ25169;NKX6.3	NK6 homeobox 3	0.77	0.75	0.91	0.58
TOP1MT	TOP1MT	topoisomerase (DNA) I, mitochondrial	0.77	0.74	0.91	0.49

SERPINC1	SERPINC1;AT3;ATIII;MGC22579	serpin peptidase inhibitor, clade C (antithrombin), member 1	0.76	0.68	0.91	0.53
SYNPO2L	SYNPO2L;FLJ12921	synaptopodin 2-like	0.76	0.68	0.91	0.51
KCTD8	KCTD8	potassium channel tetramerisation domain containing 8	0.76	0.72	0.91	0.58
CMTM3	CMTM3;BNAS2;CKLFSF3;FLJ31762;MGC51956	CKLF-like MARVEL transmembrane domain containing 3	0.76	0.75	0.91	0.55
AKAP9	AKAP9;AKAP-9;AKAP350;AKAP450;CG-NAP;HYPERION;KIAA0803;MU-RMS-40.16A;PPP1R45;PRKA9;YOTIAO	A kinase (PRKA) anchor protein (yotiao) 9	0.76	0.74	0.91	0.55
MED13L	MED13L;DKFZp781D0112;FLJ21627;KIAA1025;PROSIT240;THRAP2;TRAP240L	mediator complex subunit 13-like	0.76	0.7	0.91	0.48
OR51F1	OR51F1;OR11-21;OR51F1P	olfactory receptor, family 51, subfamily F, member 1	0.74	0.69	0.91	0.46
AEN	AEN;FLJ12484;FLJ12562;ISG20L1;pp12744	apoptosis enhancing nuclease	0.74	0.6	0.91	0.47
BLVRA	BLVRA;BLVR;BVR;BVRA	biliverdin reductase A	0.74	0.67	0.91	0.5
TRIM25	TRIM25;EFP;RNF147;Z147;ZNF147	tripartite motif containing 25	0.74	0.66	0.91	0.59
PAFAH1B1	PAFAH1B1;LIS1;LIS2;MDCR;MDS;PAFAH	platelet-activating factor acetylhydrolase 1b, regulatory subunit 1 (45kDa)	0.74	0.74	0.91	0.51
OR3A4P	OR3A4P;OLFRA05;OLFRA06;OR17-13;OR17-16;OR17-24;OR17-25;OR24;OR25;OR3A4;OR3A5P	olfactory receptor, family 3, subfamily A, member 4 pseudogene	0.74	0.71	0.91	0.57
ARHGAP33	ARHGAP33;FLJ39019;NOMA-GAP;SNX26;TCGAP	Rho GTPase activating protein 33	0.74	0.69	0.91	0.48
TMEM106B	TMEM106B;FLJ11273;MGC33727	transmembrane protein 106B	0.73	0.72	0.91	0.55
C16orf58	C16orf58;FLJ13868	chromosome 16 open reading frame 58	0.73	0.65	0.91	0.49
DDX54	DDX54;DP97;MGC2835	DEAD (Asp-Glu-Ala-Asp) box polypeptide 54	0.73	0.67	0.91	0.52
OAT	OAT;DKFZp781A11155;GACR;HOGA;OATASE;OKT	ornithine aminotransferase	0.73	0.65	0.91	0.56
SLC22A13	SLC22A13;OAT10;OCTL1;OCTL3;ORCTL-3;ORCTL3	solute carrier family 22 (organic anion transporter), member 13	0.72	0.64	0.91	0.49
FBXO44	FBXO44;DKFZp781J0852;FBG3;FBX30;FBX6A;Fbx44;Fbxo6a;MGC14140	F-box protein 44	0.72	0.62	0.91	0.45
ASAH2B	ASAH2B;ASAH2C;ASAH2L;bA449O16.3	N-acylsphingosine amidohydrolase (non-lysosomal ceramidase) 2B	0.72	0.68	0.91	0.5
ACP2	ACP2	acid phosphatase 2, lysosomal	0.72	0.6	0.91	0.46
THOP1	THOP1;EP24.15;MEPD_HUMAN;MP78;TOP	thimet oligopeptidase 1	0.72	0.64	0.91	0.48
MIER3	MIER3;DKFZp686L09111;DKFZp781G1119;DKFZp781I1119;FLJ35954	mesoderm induction early response 1, family member 3	0.72	0.64	0.91	0.49
KIAA1618	KIAA1618	ENTREZ STATUS: REMAPPED TO GENE ID: 57674	0.72	0.68	0.91	0.49
RHOG	RHOG;ARHG;MGC125835;MGC125836	ras homolog gene family, member G (rho G)	0.72	0.65	0.91	0.49
PPP1R11	PPP1R11;HCG-V;HCGV;IPP3;MGC125741;MGC125742;MGC125743;TCTE5;TCTEX5	protein phosphatase 1, regulatory (inhibitor) subunit 11	0.72	0.66	0.91	0.48
PYGL	PYGL;GSD6	phosphorylase, glycogen, liver	0.72	0.68	0.91	0.53
KRT8	KRT8;CARD2;CK-8;CK8;CYK8;K2C8;K8;KO	keratin 8	0.72	0.66	0.91	0.44
CLNK	CLNK;MGC35197;MIST	cytokine-dependent hematopoietic cell linker	0.72	0.68	0.91	0.45
DCAF11	DCAF11;DKFZp779A1629;PRO2389;WDR23	DDB1 and CUL4 associated factor 11	0.72	0.67	0.91	0.48
FAM151A	FAM151A;C1orf179;MGC27169	family with sequence similarity 151, member A	0.71	0.7	0.91	0.47
INPP1	INPP1;MGC110984	inositol polyphosphate-1-phosphatase	0.7	0.64	0.91	0.49
TNFAIP3	TNFAIP3;A20;MGC104522;MGC138687;MGC138688;OTUD7C;TNFA1P2	tumor necrosis factor, alpha-induced protein 3	0.7	0.64	0.91	0.48
COX16	COX16;C14orf112	COX16 cytochrome c oxidase assembly homolog (S. cerevisiae)	0.7	0.57	0.91	0.48
HNRNPU	HNRNPU;HNRPU;SAF-A;U21.1;hnRNP U	heterogeneous nuclear ribonucleoprotein U (scaffold attachment factor A)	0.7	0.67	0.91	0.47
LRP5L	LRP5L;DKFZp434O0213	low density lipoprotein receptor-related protein 5-like	0.69	0.67	0.91	0.47
IFI16	IFI16;IFNGIP1;MGC9466;PYHIN2	interferon, gamma-inducible protein 16	0.69	0.63	0.91	0.49
SLFN12	SLFN12;FLJ10260;SLFN3	schlafen family member 12	0.69	0.62	0.91	0.46
RANBP6	RANBP6;FLJ54311	RAN binding protein 6	0.69	0.66	0.91	0.54
FCF1	FCF1;Bka;C14orf111;MGC99629;UTP24	FCF1 small subunit (SSU) processome component homolog (S. cerevisiae)	0.68	0.6	0.91	0.45
RALBP1	RALBP1;RIP1;RLIP1;RLIP76	ralA binding protein 1	0.68	0.66	0.91	0.51
OXSR1	OXSR1;KIAA1101;OSR1	oxidative-stress responsive 1	0.68	0.67	0.91	0.48
CLIC1	CLIC1;G6;NCC27	chloride intracellular channel 1	0.68	0.64	0.91	0.5
C14orf180	C14orf180;C14orf77;FLJ38594	chromosome 14 open reading frame 180	0.67	0.62	0.91	0.48
TMEM183A	TMEM183A;C1orf37	transmembrane protein 183A	0.67	0.65	0.91	0.45
BRPF1	BRPF1;BR140	bromodomain and PHD finger containing, 1	0.66	0.58	0.91	0.53
AKAP11	AKAP11;AKAP-11;AKAP220;DKFZp781I12161;FLJ11304;KIAA0629;PPP1R44;PRKA11	A kinase (PRKA) anchor protein 11	0.66	0.61	0.91	0.47
NTN4	NTN4;FLJ23180;PRO3091	netrin 4	0.66	0.62	0.91	0.43
COX15	COX15	COX15 homolog, cytochrome c oxidase assembly protein (yeast)	0.66	0.59	0.91	0.5
PTPRF	PTPRF;FLJ43335;FLJ45062;FLJ45567;LAR	protein tyrosine phosphatase, receptor type, F	0.66	0.6	0.91	0.46
FAM3A	FAM3A;2.19;DLD;DXS560S;XAP-7	family with sequence similarity 3, member A	0.66	0.62	0.91	0.5
NEXN	NEXN;CMH20;MGC104234;MGC138865;MGC138866;NELIN	nexilin (F actin binding protein)	0.66	0.6	0.91	0.5
ATP6V0E1	ATP6V0E1;ATP6H;ATP6V0E;M9.2;Vma21;Vma21p	ATPase, H+ transporting, lysosomal 9kDa, V0 subunit e1	0.66	0.6	0.91	0.44
36951	MARCH1;DKFZp564M1682;FLJ20668;MARCH-I;RNF171	membrane-associated ring finger (C3HC4) 1	0.64	0.58	0.91	0.52
AP1B1	AP1B1;ADTB1;AP105A;BAM22;CLAPB2	adaptor-related protein complex 1, beta 1 subunit	0.64	0.61	0.91	0.49
AQPEP	AQPEP;APQ;FLJ90650;LVRN;MGC125378;MGC125379	laeverin	0.64	0.6	0.91	0.49
MARK1	MARK1;KIAA1477;MARK;MGC126512;MGC126513;Par-1c;Par1c	MAP/microtubule affinity-regulating kinase 1	0.62	0.56	0.91	0.47
RAX2	RAX2;ARMD6;CORD11;MGC15631;QRX;RAXL1	retina and anterior neural fold homeobox 2	0.62	0.55	0.91	0.42
CCDC34	CCDC34;NY-REN-41;RAMA3	coiled-coil domain containing 34	0.62	0.54	0.91	0.41
JRK	JRK;DKFZp686C24207;FLJ45729;JH8	jerky homolog (mouse)	0.62	0.55	0.91	0.41
MAPKAPK5	MAPKAPK5;PRAK	mitogen-activated protein kinase-activated protein kinase 5	0.62	0.6	0.91	0.43
SF3B2	SF3B2;Cus1;SAP145;SF3B145;SF3b1;SF3b150	splicing factor 3b, subunit 2, 145kDa	0.62	0.57	0.91	0.44
STAB2	STAB2;DKFZp434E0321;FEEL2;FELE-2;FELL2;FEX2;HARE	stabilin 2	0.84	0.75	0.92	0.6
NPHS2	NPHS2;PDCN;SRN1	nephrosis 2, idiopathic, steroid-resistant (podocin)	0.84	0.75	0.92	0.65
MCHR2	MCHR2;GPR145;GPRv17;MCH-2R;MCH-R2;MCH2;MCH2R;MCHR-2;SLT	melanin-concentrating hormone receptor 2	0.84	0.75	0.92	0.67
OR1K1	OR1K1;hg99	olfactory receptor, family 1, subfamily K, member 1	0.84	0.76	0.92	0.63
PNLIPRP1	PNLIPRP1;PLRP1	pancreatic lipase-related protein 1	0.84	0.72	0.92	0.64
C7orf34	C7orf34;ctm-1	chromosome 7 open reading frame 34	0.84	0.8	0.92	0.6
SDK2	SDK2;FLJ10832;KIAA1514	sidekick homolog 2 (chicken)	0.84	0.76	0.92	0.63
ZNF211	ZNF211;C2H2-25;CH2H2-25;MGC131841;ZNF-25;ZNFC25	zinc finger protein 211	0.84	0.74	0.92	0.56
CCDC3	CCDC3;DKFZp761F241;FLJ20925	coiled-coil domain containing 3	0.84	0.81	0.92	0.66
FZD5	FZD5;C2orf31;DKFZp434E2135;HFZ5;MGC129692	frizzled family receptor 5	0.84	0.76	0.92	0.68
TEX261	TEX261;MGC32043;TEG-261	testis expressed 261	0.84	0.79	0.92	0.53
ERLIN1	ERLIN1;C10orf69;Erlin-1;KE04;KEO4;SPFH1	ER lipid raft associated 1	0.84	0.76	0.92	0.55
CTU1	CTU1;ATPBD3;MGC17332;NCS6	cytosolic thiouridylase subunit 1 homolog (S. pombe)	0.84	0.82	0.92	0.58
IL7R	IL7R;CD127;CDW127;IL-7R-alpha;IL7RA;ILRA	interleukin 7 receptor	0.83	0.71	0.92	0.63
IL3RA	IL3RA;CD123;IL3R;IL3RAY;IL3RX;IL3RY;MGC34174;hIL-3Ra	interleukin 3 receptor, alpha (low affinity)	0.83	0.85	0.92	0.59
F2	F2;PT	coagulation factor II (thrombin)	0.83	0.76	0.92	0.57
FBXL14	FBXL14;Fbl14;MGC40195	F-box and leucine-rich repeat protein 14	0.83	0.73	0.92	0.53
FANCB	FANCB;FA2;FAAP90;FAAP95;FAB;FACB	Fanconi anemia, complementation group B	0.83	0.76	0.92	0.52
ENTPD7	ENTPD7;DKFZp667O124;FLJ30978;FLJ31830;FLJ41522;FLJ95364;LALP1;MGC141913;RP11-483F11.1	ectonucleoside triphosphate diphosphohydrolase 7	0.83	0.82	0.92	0.62
C18orf8	C18orf8;FLJ23453;HsT2591;MIC1;Mic-1	chromosome 18 open reading frame 8	0.83	0.78	0.92	0.59
ZNF488	ZNF488;FLJ32104	zinc finger protein 488	0.82	0.75	0.92	0.62
PPAPDC3	PPAPDC3;C9orf67;FLJ14662;KIAA0515;MGC12921;NET39	phosphatidic acid phosphatase type 2 domain containing 3	0.82	0.7	0.92	0.53
MYEOV2	MYEOV2	myeloma overexpressed 2	0.82	0.72	0.92	0.58
CBR4	CBR4;FLJ14431;SDR45C1	carbonyl reductase 4	0.82	0.81	0.92	0.53
PCBD2	PCBD2;DCOH2;DCOHM;PHS2	pterin-4 alpha-carbinolamine dehydratase/dimerization cofactor of hepatocyte nuclear factor 1 alpha (TCF1) 2	0.82	0.76	0.92	0.61
DHRS7	DHRS7;SDR34C1;retDSR4;retSDR4	dehydrogenase/reductase (SDR family) member 7	0.82	0.77	0.92	0.61
PARVA	PARVA;CH-ILKBP;FLJ10793;FLJ12254;FLJ25106;FLJ46049;MXRA2	parvin, alpha	0.82	0.78	0.92	0.63
NET1	NET1;ARHGEF8;NET1A	neuroepithelial cell transforming 1	0.82	0.78	0.92	0.59
C10orf118	C10orf118;FLJ10188;FLJ35301;MGC118918;MGC129699	chromosome 10 open reading frame 118	0.82	0.74	0.92	0.59
SUPV3L1	SUPV3L1;SUV3	suppressor of var1, 3-like 1 (S. cerevisiae)	0.82	0.76	0.92	0.59
ASPDH	ASPDH	aspartate dehydrogenase domain containing	0.82	0.78	0.92	0.59
CARD14	CARD14;BIMP2;CARMA2	caspase recruitment domain family, member 14	0.8	0.8	0.92	0.59
ENG	ENG;CD105;END;FLJ41744;HHT1;ORW;ORW1	endoglin	0.8	0.76	0.92	0.51
PPAPDC2	PPAPDC2;FLJ46512;FLJ90191;MGC15483;PDP1;PSDP;bA6J24.6	phosphatidic acid phosphatase type 2 domain containing 2	0.8	0.72	0.92	0.57
SLC25A42	SLC25A42;MGC26694	solute carrier family 25, member 42	0.8	0.7	0.92	0.53
FAM174A	FAM174A;MGC17345;TMEM157;UNQ1912	family with sequence similarity 174, member A	0.8	0.79	0.92	0.61
PTPN2	PTPN2;PTN2;PTPT;TC-PTP;TCELLPTP;TCPTP	protein tyrosine phosphatase, non-receptor type 2	0.8	0.68	0.92	0.51
FNIP1	FNIP1;DKFZp686E18167;DKFZp781P0215;KIAA1961;MGC667	folliculin interacting protein 1	0.8	0.79	0.92	0.55
SERPINE1	SERPINE1;PAI;PAI-1;PAI1;PLANH1	serpin peptidase inhibitor, clade E (nexin, plasminogen activator inhibitor type 1), member 1	0.8	0.68	0.92	0.51
PTGR1	PTGR1;FLJ99229;LTB4DH;MGC34943;PGR1;ZADH3	prostaglandin reductase 1	0.8	0.71	0.92	0.61
TRMT61B	TRMT61B;DKFZp564I2178;FLJ20628	tRNA methyltransferase 61 homolog B (S. cerevisiae)	0.8	0.76	0.92	0.54
PAN2	PAN2;FLJ39360;KIAA0710;USP52	PAN2 poly(A) specific ribonuclease subunit homolog (S. cerevisiae)	0.79	0.72	0.92	0.54
MEGF10	MEGF10;DKFZp781K1852;FLJ41574;KIAA1780	multiple EGF-like-domains 10	0.78	0.74	0.92	0.5
TMEM175	TMEM175;MGC4618	transmembrane protein 175	0.78	0.75	0.92	0.54
AQPEP	AQPEP;APQ;FLJ90650;LVRN;MGC125378;MGC125379	laeverin	0.78	0.72	0.92	0.53
PHYHIPL	PHYHIPL;KIAA1796	phytanoyl-CoA 2-hydroxylase interacting protein-like	0.76	0.71	0.92	0.49
MAGEB6	MAGEB6;CT3.4;FLJ40242;MAGE-B6;MAGEB6A	melanoma antigen family B, 6	0.76	0.68	0.92	0.52
WNK2	WNK2;KIAA1760;NY-CO-43;P/OKcl.13;PRKWNK2;SDCCAG43	WNK lysine deficient protein kinase 2	0.76	0.7	0.92	0.58
GJA4	GJA4;CX37	gap junction protein, alpha 4, 37kDa	0.76	0.76	0.92	0.53
ULK1	ULK1;ATG1;ATG1A;FLJ38455;FLJ46475;UNC51;Unc51.1;hATG1	unc-51-like kinase 1 (C. elegans)	0.76	0.68	0.92	0.61
IGFBPL1	IGFBPL1;IGFBP-RP4;bA113O24.1	insulin-like growth factor binding protein-like 1	0.76	0.7	0.92	0.47
KIF1B	KIF1B;CMT2;CMT2A;CMT2A1;FLJ23699;HMSNII;KIAA0591;KIAA1448;KLP;MGC134844;NBLST1	kinesin family member 1B	0.76	0.72	0.92	0.57
ZMIZ2	ZMIZ2;DKFZp761I2123;KIAA1886;NET27;TRAFIP20;ZIMP7;hZIMP7	zinc finger, MIZ-type containing 2	0.76	0.66	0.92	0.53
HCFC1	HCFC1;CFF;HCF-1;HCF1;HFC1;MGC70925;VCAF	host cell factor C1 (VP16-accessory protein)	0.76	0.68	0.92	0.51
EIF4G3	EIF4G3;eIF-4G 3;eIF4G 3;eIF4GII	eukaryotic translation initiation factor 4 gamma, 3	0.76	0.72	0.92	0.48
ANP32B	ANP32B;APRIL;PHAPI2;SSP29	acidic (leucine-rich) nuclear phosphoprotein 32 family, member B	0.76	0.68	0.92	0.57
CXCL16	CXCL16;CXCLG16;SR-PSOX;SRPSOX	chemokine (C-X-C motif) ligand 16	0.76	0.72	0.92	0.57
ANAPC5	ANAPC5;APC5	anaphase promoting complex subunit 5	0.75	0.68	0.92	0.51
NR2E1	NR2E1;TLL;TLX;XTLL	nuclear receptor subfamily 2, group E, member 1	0.74	0.68	0.92	0.5
KCNE3	KCNE3;DKFZp781H21101;HOKPP;HYPP;MGC102685;MGC129924;MiRP2	potassium voltage-gated channel, Isk-related family, member 3	0.74	0.74	0.92	0.57
ANXA8L2	ANXA8L2;ANXA8;FLJ32754;FLJ39396;FLJ54151;KIAA0187;bA145E20.2	annexin A8-like 2	0.74	0.66	0.92	0.5
CDK10	CDK10;PISSLRE	cyclin-dependent kinase 10	0.74	0.74	0.92	0.52
LIME1	LIME1;FLJ20406;LIME;LP8067;dJ583P15.4	Lck interacting transmembrane adaptor 1	0.74	0.7	0.92	0.5
LRRC26	LRRC26;CAPC;bA350O14.10	leucine rich repeat containing 26	0.74	0.72	0.92	0.51
TIMM8B	TIMM8B;DDP2;FLJ21744;MGC102866;MGC117373;TIM8B	translocase of inner mitochondrial membrane 8 homolog B (yeast)	0.74	0.7	0.92	0.53
SPTLC2	SPTLC2;HSN1C;KIAA0526;LCB2;LCB2A;NSAN1C;SPT2;hLCB2a	serine palmitoyltransferase, long chain base subunit 2	0.74	0.65	0.92	0.57
COX6B2	COX6B2;COXVIB2;CT59;FLJ46422;MGC119094	cytochrome c oxidase subunit VIb polypeptide 2 (testis)	0.73	0.66	0.92	0.51

AR	AR;AIS;DHTR;HUMARA;HYSP1;KD;NR3C4;SBMA;SMAX1;TFM	androgen receptor	0.72	0.61	0.92	0.49
CLEC14A	CLEC14A;C14orf27;CEG1	C-type lectin domain family 14, member A	0.72	0.6	0.92	0.5
TCF7	TCF7;FLJ36364;MGC47735;TCF-1	transcription factor 7 (T-cell specific, HMG-box)	0.72	0.62	0.92	0.49
CLEC11A	CLEC11A;CLECSF3;LSLCL;P47;SCGF	C-type lectin domain family 11, member A	0.72	0.66	0.92	0.49
DTX2	DTX2;KIAA1528;MGC71098;RNF58	deltex homolog 2 (Drosophila)	0.72	0.65	0.92	0.58
DEAF1	DEAF1;NUDR;SPN;ZMYND5	deformed epidermal autoregulatory factor 1 (Drosophila)	0.72	0.7	0.92	0.46
RNF169	RNF169;KIAA1991	ring finger protein 169	0.72	0.65	0.92	0.58
ADCK1	ADCK1;FLJ39600	aarF domain containing kinase 1	0.72	0.66	0.92	0.54
FTL	FTL;MGC71996;NBIA3	ferritin, light polypeptide	0.72	0.68	0.92	0.49
PRB2	PRB2;IB-9;PRPPRB1;Ps;cP7	proline-rich protein BstNI subfamily 2	0.71	0.68	0.92	0.47
COL23A1	COL23A1;DKFZp434K0621	collagen, type XXIII, alpha 1	0.71	0.64	0.92	0.48
C12orf44	C12orf44;ATG101;FLJ11773	chromosome 12 open reading frame 44	0.71	0.7	0.92	0.49
USH1C	USH1C;AIE-75;DFNB18;NY-CO-37;NY-CO-38;PDZ-45;PDZ-73;PDZ-73/NY-CO-38;PDZ73;ush1cpst	Usher syndrome 1C (autosomal recessive, severe)	0.7	0.67	0.92	0.51
VEGFC	VEGFC;Flt4-L;VRP	vascular endothelial growth factor C	0.7	0.66	0.92	0.52
FOS	FOS;AP-1;C-FOS;p55	FBJ murine osteosarcoma viral oncogene homolog	0.7	0.72	0.92	0.49
ZFP36	ZFP36;G0S24;GOS24;NUP475;RNF162A;TIS11;TTP	zinc finger protein 36, C3H type, homolog (mouse)	0.7	0.62	0.92	0.45
ACAA2	ACAA2;DSAEC;FLJ35992;FLJ95265	acetyl-CoA acyltransferase 2	0.7	0.6	0.92	0.47
ARF5	ARF5	ADP-ribosylation factor 5	0.7	0.68	0.92	0.45
NIP7	NIP7;FLJ10296;HSPC031;KD93	nuclear import 7 homolog (S. cerevisiae)	0.7	0.68	0.92	0.48
WHAMM	WHAMM;KIAA1971;WHDC1	WAS protein homolog associated with actin, golgi membranes and microtubules	0.7	0.61	0.92	0.49
SLC39A11	SLC39A11;C17orf26;ZIP11	solute carrier family 39 (metal ion transporter), member 11	0.69	0.62	0.92	0.47
TPM2	TPM2;AMCD1;DA1;DA2B;NEM4;TMSB	tropomyosin 2 (beta)	0.69	0.7	0.92	0.51
FAT1	FAT1;CDHF7;CDHR8;FAT;ME5;hFat1	FAT tumor suppressor homolog 1 (Drosophila)	0.69	0.62	0.92	0.47
SLIT2	SLIT2;FLJ14420;SLIL3;Slit-2	slit homolog 2 (Drosophila)	0.68	0.64	0.92	0.46
NPM1	NPM1;B23;MGC104254;NPM	nucleophosmin (nucleolar phosphoprotein B23, numatrin)	0.68	0.62	0.92	0.48
XYLT1	XYLT1;PXYLT1;XT-I;XT1;XTI;XYLTI	xylosyltransferase I	0.66	0.6	0.92	0.41
PLCXD1	PLCXD1;FLJ11323	phosphatidylinositol-specific phospholipase C, X domain containing 1	0.66	0.66	0.92	0.49
PREP	PREP;MGC16060;PE;PEP	prolyl endopeptidase	0.66	0.61	0.92	0.45
KLC1	KLC1;KLC;KNS2;KNS2A;MGC15245	kinesin light chain 1	0.65	0.66	0.92	0.46
ZNF662	ZNF662;FLJ33347;FLJ45880;MGC149141	zinc finger protein 662	0.64	0.62	0.92	0.44
GOLGB1	GOLGB1;GCP;GCP372;GOLIM1	golgin B1	0.64	0.61	0.92	0.41
JARID2	JARID2;JMJ	jumonji, AT rich interactive domain 2	0.64	0.59	0.92	0.46
ATF6	ATF6;ATF6A;DKFZp686P2194;FLJ21663	activating transcription factor 6	0.64	0.58	0.92	0.43
BCAP31	BCAP31;6C6-AG;BAP31;CDM;DXS1357E	B-cell receptor-associated protein 31	0.64	0.57	0.92	0.43
CHRDL1	CHRDL1;CHL;NRLN1;VOPT;dA141H5.1	chordin-like 1	0.62	0.55	0.92	0.45
RAP1GAP	RAP1GAP;KIAA0474;RAP1GA1;RAP1GAP1;RAP1GAPII;RAPGAP	RAP1 GTPase activating protein	0.62	0.55	0.92	0.41
SMC3	SMC3;BAM;BMH;CDLS3;CSPG6;HCAP;SMC3L1	structural maintenance of chromosomes 3	0.62	0.6	0.92	0.44
PPP1R1C	PPP1R1C;IPP5	protein phosphatase 1, regulatory (inhibitor) subunit 1C	0.84	0.72	0.93	0.55
GPX2	GPX2;GI-GPx;GPRP;GSHPX-GI;GSHPx-2	glutathione peroxidase 2 (gastrointestinal)	0.84	0.76	0.93	0.56
PITPNC1	PITPNC1;RDGB-BETA;RDGBB;RDGBB1	phosphatidylinositol transfer protein, cytoplasmic 1	0.84	0.8	0.93	0.61
NOXO1	NOXO1;MGC20258;P41NOX;P41NOXA;P41NOXB;P41NOXC;SH3PXD5;SNX28	NADPH oxidase organizer 1	0.84	0.82	0.93	0.56
DOCK4	DOCK4;FLJ34238;KIAA0716;MGC134911;MGC134912	dedicator of cytokinesis 4	0.84	0.82	0.93	0.66
CHD3	CHD3;Mi-2a;Mi2-ALPHA;ZFH	chromodomain helicase DNA binding protein 3	0.84	0.8	0.93	0.63
HMBS	HMBS;PBG-D;PBGD;PORC;UPS	hydroxymethylbilane synthase	0.84	0.84	0.93	0.55
ZDHHC7	ZDHHC7;FLJ10792;FLJ20279;SERZ-B;SERZ1;ZNF370	zinc finger, DHHC-type containing 7	0.84	0.81	0.93	0.55
MTMR12	MTMR12;3-PAP;PIP3AP	myotubularin related protein 12	0.84	0.77	0.93	0.58
TMEM66	TMEM66;FLJ22274;FOAP-7;MGC8721;XTP3	transmembrane protein 66	0.84	0.82	0.93	0.59
YAP1	YAP1;YAP;YAP2;YAP65;YKI	Yes-associated protein 1	0.84	0.84	0.93	0.63
METTL13	METTL13;5630401D24Rik;FLJ10310;KIAA0859;feat	methyltransferase like 13	0.84	0.84	0.93	0.63
KANK1	KANK1;ANKRD15;CPSQ2;DKFZp451G231;KANK;KIAA0172;MGC43128	KN motif and ankyrin repeat domains 1	0.83	0.75	0.93	0.57
DNAJB6	DNAJB6;DJ4;DKFZp566D0824;DnaJ;FLJ42837;HHDJ1;HSJ-2;HSJ2;MGC1152;MGC117297;MRJ;MSJ-1	DnaJ (Hsp40) homolog, subfamily B, member 6	0.83	0.74	0.93	0.58
NR1I2	NR1I2;BXR;ONR1;PAR;PAR1;PAR2;PARq;PRR;PXR;SAR;SXR	nuclear receptor subfamily 1, group I, member 2	0.82	0.82	0.93	0.61
S100P	S100P;MIG9	S100 calcium binding protein P	0.82	0.72	0.93	0.61
IRF4	IRF4;LSIRF;MUM1	interferon regulatory factor 4	0.82	0.72	0.93	0.53
EBF3	EBF3;COE3;EBF-3;O/E-2;OE-2	early B-cell factor 3	0.82	0.73	0.93	0.56
RASL12	RASL12;RIS	RAS-like, family 12	0.82	0.82	0.93	0.62
LYSMD2	LYSMD2;DKFZp686I2243;MGC35274	LysM, putative peptidoglycan-binding, domain containing 2	0.82	0.72	0.93	0.59
ANUBL1	ANUBL1;FLJ40185;ZFAND4	AN1, ubiquitin-like, homolog (Xenopus laevis)	0.82	0.78	0.93	0.53
PANK4	PANK4;DKFZp547M242;FLJ10782	pantothenate kinase 4	0.82	0.74	0.93	0.66
CTAG2	CTAG2;CAMEL;CT2;CT6.2;CT6.2a;CT6.2b;ESO2;LAGE-1;LAGE2B;MGC138724;MGC3803	cancer/testis antigen 2	0.82	0.78	0.93	0.56
PPP1R3D	PPP1R3D;DKFZp781L2441;PPP1R6	protein phosphatase 1, regulatory subunit 3D	0.82	0.76	0.93	0.59
SEC16A	SEC16A;FLJ26737;KIAA0310;RP11-413M3.10;SEC16L;p250	SEC16 homolog A (S. cerevisiae)	0.82	0.7	0.93	0.53
OAZ2	OAZ2;AZ2	ornithine decarboxylase antizyme 2	0.82	0.81	0.93	0.53
MALL	MALL;BENE;MGC4419	mal, T-cell differentiation protein-like	0.82	0.82	0.93	0.63
RELL1	RELL1;FLJ21778;MGC50583	RELT-like 1	0.82	0.78	0.93	0.6
PPP5C	PPP5C;FLJ36922;FLJ55954;PP5;PPP5;PPT	protein phosphatase 5, catalytic subunit	0.82	0.72	0.93	0.55
PTPRJ	PTPRJ;CD148;DEP1;HPTPeta;R-PTP-ETA;SCC1	protein tyrosine phosphatase, receptor type, J	0.82	0.7	0.93	0.53
C16orf62	C16orf62;DKFZp313M0539;DKFZp434B0212;FLJ21040;MGC16824	chromosome 16 open reading frame 62	0.82	0.74	0.93	0.56
CS	CS	citrate synthase	0.82	0.82	0.93	0.62
TIPIN	TIPIN;FLJ20516	TIMELESS interacting protein	0.81	0.78	0.93	0.58
TIA1	TIA1;TIA-1	TIA1 cytotoxic granule-associated RNA binding protein	0.81	0.77	0.93	0.56
TSGA10	TSGA10;CEP4L;CT79	testis specific, 10	0.8	0.73	0.93	0.61
CD34	CD34	CD34 molecule	0.8	0.74	0.93	0.52
CYTL1	CYTL1;C17;C4orf4	cytokine-like 1	0.8	0.77	0.93	0.57
RNASET2	RNASET2;FLJ42372;RNASE6PL;bA514O12.3	ribonuclease T2	0.8	0.72	0.93	0.62
SETD2	SETD2;FLJ16420;FLJ22472;FLJ23184;FLJ45883;FLJ46217;HBP231;HIF-1;HIP-1;HYPB;KIAA1732;KMT3A;SET2;p231HBP	SET domain containing 2	0.8	0.73	0.93	0.51
LRPPRC	LRPPRC;CLONE-23970;GP130;LRP130;LSFC	leucine-rich PPR-motif containing	0.8	0.82	0.93	0.6
FLJ46154	FLJ46154	ENTREZ STATUS: REMAPPED TO GENE ID: 100506627	0.8	0.8	0.93	0.56
TELO2	TELO2;CLK2;DKFZp434A073;FLJ10924;KIAA0683;TEL2	TEL2, telomere maintenance 2, homolog (S. cerevisiae)	0.79	0.66	0.93	0.52
KIF2C	KIF2C;KNSL6;MCAK	kinesin family member 2C	0.79	0.72	0.93	0.54
BEND3	BEND3;KIAA1553;RP11-59I9.2	BEN domain containing 3	0.79	0.68	0.93	0.58
LIPC	LIPC;HDLCQ12;HL;HTGL;LIPH	lipase, hepatic	0.78	0.7	0.93	0.61
UBXN10	UBXN10;FLJ25429;UBXD3	UBX domain protein 10	0.78	0.76	0.93	0.54
NPY	NPY;PYY4	neuropeptide Y	0.78	0.68	0.93	0.51
TUSC5	TUSC5;IFITMD3;LOST1	tumor suppressor candidate 5	0.78	0.76	0.93	0.51
RARG	RARG;NR1B3;RARC	retinoic acid receptor, gamma	0.78	0.7	0.93	0.5
OR51B2	OR51B2;HOR5'Beta3;OR51B1P	olfactory receptor, family 51, subfamily B, member 2	0.77	0.78	0.93	0.57
SPN	SPN;CD43;GPL115;LSN	sialophorin	0.77	0.7	0.93	0.49
TEX13B	TEX13B;TGC3B;TSGA5	testis expressed 13B	0.77	0.76	0.93	0.53
ABCA1	ABCA1;ABC-1;ABC1;CERP;FLJ14958;HDLDT1;MGC164864;MGC165011;TGD	ATP-binding cassette, sub-family A (ABC1), member 1	0.77	0.8	0.93	0.55
CSNK1G1	CSNK1G1	casein kinase 1, gamma 1	0.77	0.71	0.93	0.59
ITFG2	ITFG2;MDS028	integrin alpha FG-GAP repeat containing 2	0.77	0.7	0.93	0.53
ICT1	ICT1;DS-1;DS1	immature colon carcinoma transcript 1	0.77	0.72	0.93	0.53
COL14A1	COL14A1;UND	collagen, type XIV, alpha 1	0.76	0.66	0.93	0.56
CLEC2D	CLEC2D;CLAX;LLT1;OCIL	C-type lectin domain family 2, member D	0.76	0.66	0.93	0.52
FAM122A	FAM122A;C9orf42;MGC17347	family with sequence similarity 122A	0.76	0.72	0.93	0.54
PLCD3	PLCD3;MGC71172	phospholipase C, delta 3	0.76	0.68	0.93	0.59
FTSJ3	FTSJ3;EPCS3;FLJ20062	FtsJ homolog 3 (E. coli)	0.76	0.72	0.93	0.48
C19orf50	C19orf50;DKFZp686B1038;FLJ25480;FLJ43375;MGC2749;MST096;MSTP096	chromosome 19 open reading frame 50	0.76	0.64	0.93	0.49
LRTM2	LRTM2;FLJ42697	leucine-rich repeats and transmembrane domains 2	0.75	0.74	0.93	0.52
KIAA1467	KIAA1467;DKFZp781O012	KIAA1467	0.75	0.76	0.93	0.56
FUNDC1	FUNDC1;MGC51029	FUN14 domain containing 1	0.75	0.74	0.93	0.59
GABRB3	GABRB3;ECA5;MGC9051	gamma-aminobutyric acid (GABA) A receptor, beta 3	0.74	0.72	0.93	0.56
KIRREL3	KIRREL3;KIAA1867;KIRRE;MGC126824;MGC126850;MRD4;NEPH2;PRO4502	kin of IRRE like 3 (Drosophila)	0.74	0.72	0.93	0.47
C11orf9	C11orf9;KIAA0954;MGC10781;MRF;Ndt80	chromosome 11 open reading frame 9	0.74	0.73	0.93	0.54
CASKIN2	CASKIN2;ANKS5B;FLJ21609;KIAA1139	CASK interacting protein 2	0.74	0.62	0.93	0.52
TOB1	TOB1;APRO6;MGC104792;MGC34446;PIG49;TOB;TROB;TROB1	transducer of ERBB2, 1	0.74	0.68	0.93	0.53
PABPN1	PABPN1;OPMD;PAB2;PABII;PABP-2;PABP2	poly(A) binding protein, nuclear 1	0.74	0.66	0.93	0.51
DYNLL1	DYNLL1;DLC1;DLC8;DNCL1;DNCLC1;LC8;LC8a;MGC126137;MGC126138;PIN;hdlc1	dynein, light chain, LC8-type 1	0.74	0.66	0.93	0.58
CACNA2D3	CACNA2D3;HSA272268	calcium channel, voltage-dependent, alpha 2/delta subunit 3	0.73	0.71	0.93	0.55
AKAP5	AKAP5;AKAP75;AKAP79;H21	A kinase (PRKA) anchor protein 5	0.73	0.67	0.93	0.5
CBY1	CBY1;C22orf2;CBY;HS508I15A;PGEA1;PIGEA-14;PIGEA14;arb1	chibby homolog 1 (Drosophila)	0.73	0.72	0.93	0.5
UNC5B	UNC5B;UNC5H2;p53RDL1	unc-5 homolog B (C. elegans)	0.73	0.72	0.93	0.55
BRP44	BRP44;DKFZp564B167;MGC125752;MGC125753	brain protein 44	0.73	0.68	0.93	0.49
ALX1	ALX1;CART1;FND3	ALX homeobox 1	0.72	0.72	0.93	0.55
FERMT3	FERMT3;KIND3;MGC10966;MIG-2;MIG2B;UNC112C;URP2;URP2SF	fermitin family member 3	0.72	0.64	0.93	0.49
PPP2R3C	PPP2R3C;C14orf10;FLJ20644;G4-1;G5pr	protein phosphatase 2, regulatory subunit B", gamma	0.72	0.72	0.93	0.48
PNCK	PNCK;BSTK3;CaMK1b;FLJ50403;FLJ50549;FLJ56451;FLJ59811;MGC45419	pregnancy up-regulated non-ubiquitously expressed CaM kinase	0.72	0.72	0.93	0.51
SORBS3	SORBS3;SCAM-1;SCAM1;SH3D4	sorbin and SH3 domain containing 3	0.72	0.65	0.93	0.49
BCL2L11	BCL2L11;BAM;BIM;BIM-alpha6;BIM-beta6;BIM-beta7;BOD;BimEL;BimL	BCL2-like 11 (apoptosis facilitator)	0.72	0.66	0.93	0.52
FAM76A	FAM76A;FLJ41946;MGC34648	family with sequence similarity 76, member A	0.72	0.67	0.93	0.48
COG1	COG1;CDG2G;DKFZp762L1710;KIAA1381;LDLB	component of oligomeric golgi complex 1	0.72	0.7	0.93	0.57
ATIC	ATIC;AICAR;AICARFT;FLJ93545;IMPCHASE;PURH	5-aminoimidazole-4-carboxamide ribonucleotide formyltransferase/IMP cyclohydrolase	0.72	0.74	0.93	0.51
RPGRIP1L	RPGRIP1L;CORS3;DKFZp686C0668;FTM;JBTS7;KIAA1005;MKS5;NPHP8	RPGRIP1-like	0.71	0.72	0.93	0.48
KRT38	KRT38;HA8;KRTHA8;hHa8	keratin 38	0.7	0.64	0.93	0.48
SPI1	SPI1;OF;PU.1;SFPI1;SPI-1;SPI-A	spleen focus forming virus (SFFV) proviral integration oncogene spi1	0.7	0.62	0.93	0.49
LEAP2	LEAP2;LEAP-2	liver expressed antimicrobial peptide 2	0.7	0.7	0.93	0.53
PRKCDBP	PRKCDBP;CAVIN3;HSRBC;MGC20400;SRBC;cavin-3	protein kinase C, delta binding protein	0.7	0.67	0.93	0.45
C22orf39	C22orf39;DKFZp686I08210;MGC74441	chromosome 22 open reading frame 39	0.7	0.62	0.93	0.45

MPPED1	MPPED1;239AB;C22orf1;FAM1A;FLJ50009;FLJ54633;FLJ59965;FLJ78907;MGC88045	metallophosphoesterase domain containing 1	0.7	0.63	0.93	0.48
FNTB	FNTB;FPTB;MGC31935	farnesyltransferase, CAAX box, beta	0.7	0.62	0.93	0.48
AKT1S1	AKT1S1;Lobe;MGC2865;PRAS40	AKT1 substrate 1 (proline-rich)	0.7	0.68	0.93	0.45
DUS1L	DUS1L;DUS1;PP3111	dihydrouridine synthase 1-like (S. cerevisiae)	0.7	0.7	0.93	0.54
SRP54	SRP54	signal recognition particle 54kDa	0.7	0.68	0.93	0.48
PPP1R3C	PPP1R3C;PPP1R5	protein phosphatase 1, regulatory subunit 3C	0.69	0.6	0.93	0.45
GLT8D2	GLT8D2;FLJ31494	glycosyltransferase 8 domain containing 2	0.69	0.65	0.93	0.44
PDXDC1	PDXDC1;KIAA0251;LP8165	pyridoxal-dependent decarboxylase domain containing 1	0.69	0.62	0.93	0.47
ZNF154	ZNF154;MGC176628;MGC176661;pHZ-92	zinc finger protein 154	0.68	0.67	0.93	0.54
C3orf22	C3orf22;MGC34728	chromosome 3 open reading frame 22	0.68	0.63	0.93	0.47
TGM7	TGM7;TGMZ	transglutaminase 7	0.68	0.6	0.93	0.47
ZNF347	ZNF347;ZNF1111	zinc finger protein 347	0.68	0.62	0.93	0.47
TMEM93	TMEM93;MGC2963	transmembrane protein 93	0.68	0.64	0.93	0.46
UHRF2	UHRF2;DKFZp434B0920;DKFZp686G0837;MGC33463;NIRF;RNF107;URF2	ubiquitin-like with PHD and ring finger domains 2	0.68	0.62	0.93	0.56
PLK1S1	PLK1S1;C20orf19;DKFZp586H021;Kiz;Kizuna;MGC102941;MGC141930;NCRNA00153	polo-like kinase 1 substrate 1	0.68	0.64	0.93	0.44
HNRNPH3	HNRNPH3;2H9;FLJ34092;HNRPH3	heterogeneous nuclear ribonucleoprotein H3 (2H9)	0.67	0.6	0.93	0.48
PCK1	PCK1;MGC22652;PEPCK-C;PEPCK1;PEPCKC	phosphoenolpyruvate carboxykinase 1 (soluble)	0.66	0.66	0.93	0.47
EPB42	EPB42;MGC116735;MGC116737;PA;SPH5	erythrocyte membrane protein band 4.2	0.66	0.59	0.93	0.51
CNIH	CNIH;CNIH1;CNIL;MGC117156;TGAM77	cornichon homolog (Drosophila)	0.66	0.64	0.93	0.52
DNAJC19	DNAJC19;Pam18;TIM14;TIMM14	DnaJ (Hsp40) homolog, subfamily C, member 19	0.66	0.68	0.93	0.45
GOLGA7	GOLGA7;GCP16;GOLGA3AP1;GOLGA7A;HSPC041;MGC21096;MGC4876	golgin A7	0.66	0.62	0.93	0.48
PPP2R5E	PPP2R5E	protein phosphatase 2, regulatory subunit B', epsilon isoform	0.66	0.61	0.93	0.48
KDM4A	KDM4A;JHDM3A;JMJD2;JMJD2A;KIAA0677	lysine (K)-specific demethylase 4A	0.66	0.62	0.93	0.49
PYGO1	PYGO1;DKFZp547G0910	pygopus homolog 1 (Drosophila)	0.65	0.6	0.93	0.5
ERV3-1	ERV3-1;ERV-R;ERV3;ERVR;FLJ23884;HERV-R;HERVR;envR	endogenous retrovirus group 3, member 1	0.65	0.62	0.93	0.42
FOXJ1	FOXJ1;FKHL13;HFH-4;HFH4;MGC35202	forkhead box J1	0.64	0.58	0.93	0.45
ZFPM1	ZFPM1;FOG;FOG1;ZNF408;ZNF89A	zinc finger protein, multitype 1	0.64	0.66	0.93	0.48
CPSF3	CPSF3;CPSF;CPSF-73;CPSF73;YSH1	cleavage and polyadenylation specific factor 3, 73kDa	0.64	0.62	0.93	0.42
ZNF80	ZNF80;pT17	zinc finger protein 80	0.63	0.64	0.93	0.47
PAQR9	PAQR9;FLJ41938	progestin and adipoQ receptor family member IX	0.63	0.57	0.93	0.46
C20orf197	C20orf197;FLJ33860;MGC120496;RP5-1017F8.1	chromosome 20 open reading frame 197	0.62	0.6	0.93	0.43
TMEM44	TMEM44;DKFZp686O18124;MGC131692;MGC163169	transmembrane protein 44	0.62	0.54	0.93	0.44
DMPK	DMPK;DM;DM1;DM1PK;DMK;MDPK;MT-PK	dystrophia myotonica-protein kinase	0.62	0.57	0.93	0.47
C20orf144	C20orf144;dJ63M2.6	chromosome 20 open reading frame 144	0.62	0.54	0.93	0.46
DRGX	DRGX;DRG11;PRRXL1	dorsal root ganglia homeobox	0.61	0.58	0.93	0.4
GJA3	GJA3;CX46;CZP3	gap junction protein, alpha 3, 46kDa	0.61	0.55	0.93	0.43
PECAM1	PECAM1;CD31;FLJ34100;FLJ58394;PECAM-1	platelet/endothelial cell adhesion molecule	0.84	0.84	0.94	0.68
ZNF232	ZNF232;ZSCAN11	zinc finger protein 232	0.84	0.76	0.94	0.55
CLEC4F	CLEC4F;CLECSF13;FLJ39110;KCLR	C-type lectin domain family 4, member F	0.84	0.79	0.94	0.54
RFX3	RFX3;MGC87155;bA32F11.1	regulatory factor X, 3 (influences HLA class II expression)	0.84	0.74	0.94	0.59
PROCA1	PROCA1;MGC39650	protein interacting with cyclin A1	0.84	0.76	0.94	0.6
STX1A	STX1A;HPC-1;P35-1;STX1;SYN1A	syntaxin 1A (brain)	0.84	0.76	0.94	0.55
PXDN	PXDN;D2S448;D2S448E;KIAA0230;MG50;PRG2;PXN;VPO	peroxidasin homolog (Drosophila)	0.84	0.78	0.94	0.55
KIF2A	KIF2A;HK2;KIF2	kinesin heavy chain member 2A	0.84	0.84	0.94	0.64
RBP1	RBP1;CRABP-I;CRBP;CRBP1;CRBPI;RBPC	retinol binding protein 1, cellular	0.84	0.88	0.94	0.6
G3BP2	G3BP2	GTPase activating protein (SH3 domain) binding protein 2	0.84	0.78	0.94	0.6
PPP6R3	PPP6R3;C11orf23;DKFZp781E17107;DKFZp781E2374;DKFZp781O2362;FLJ11058;FLJ43065;KIAA1558;MGC125711;MGC125712;PP6R3;SAP190;SAPL;SAPLa;SAPS3	protein phosphatase 6, regulatory subunit 3	0.84	0.75	0.94	0.58
FRG2B	FRG2B	FSHD region gene 2 family, member B	0.83	0.81	0.94	0.54
PPAPDC1A	PPAPDC1A;DPPL2;MGC120299;MGC120300;PPAPDC1	phosphatidic acid phosphatase type 2 domain containing 1A	0.83	0.78	0.94	0.6
OSBPL7	OSBPL7;MGC71150;ORP7	oxysterol binding protein-like 7	0.83	0.82	0.94	0.58
UBFD1	UBFD1;FLJ38870;FLJ42145;UBPH	ubiquitin family domain containing 1	0.83	0.8	0.94	0.6
TNFRSF11A	TNFRSF11A;CD265;FEO;LOH18CR1;ODFR;OFE;OPTB7;OSTS;PDB2;RANK;TRANCER	tumor necrosis factor receptor superfamily, member 11a, NFKB activator	0.82	0.76	0.94	0.56
C9orf116	C9orf116;FLJ13945;MGC29761	chromosome 9 open reading frame 116	0.82	0.8	0.94	0.61
SYNGR3	SYNGR3;MGC20003	synaptogyrin 3	0.82	0.7	0.94	0.56
ARHGEF11	ARHGEF11;DKFZp667F1223;GTRAP48;KIAA0380;PDZ-RHOGEF	Rho guanine nucleotide exchange factor (GEF) 11	0.82	0.74	0.94	0.56
C6orf72	C6orf72;dJ12G14.2	chromosome 6 open reading frame 72	0.82	0.79	0.94	0.54
HIVEP1	HIVEP1;CIRIP;CRYBP1;MBP-1;PRDII-BF1;Schnurri-1;ZAS1;ZNF40;ZNF40A	human immunodeficiency virus type I enhancer binding protein 1	0.82	0.86	0.94	0.59
PIGY	PIGY;MGC14156;PIG-Y;PREY	phosphatidylinositol glycan anchor biosynthesis, class Y	0.82	0.78	0.94	0.58
TRIP13	TRIP13;16E1BP	thyroid hormone receptor interactor 13	0.82	0.76	0.94	0.57
SAMHD1	SAMHD1;DCIP;HDDC1;MOP-5;SBBI88	SAM domain and HD domain 1	0.82	0.86	0.94	0.6
GSPT1	GSPT1;551G9.2;ETF3A;FLJ38048;FLJ39067;GST1;eRF3a	G1 to S phase transition 1	0.82	0.86	0.94	0.59
C2orf83	C2orf83	chromosome 2 open reading frame 83	0.82	0.73	0.94	0.62
SCG5	SCG5;7B2;P7B2;SGNE1;SgV	secretogranin V (7B2 protein)	0.81	0.8	0.94	0.57
WFDC10B	WFDC10B;WAP12	WAP four-disulfide core domain 10B	0.81	0.8	0.94	0.56
NUCB2	NUCB2;NEFA	nucleobindin 2	0.81	0.74	0.94	0.56
LRP3	LRP3	low density lipoprotein receptor-related protein 3	0.81	0.73	0.94	0.57
OTOR	OTOR;FDP;MGC126737;MGC126739;MIAL;MIAL1	otoraplin	0.8	0.68	0.94	0.54
WFDC3	WFDC3;WAP14;dJ447F3.3	WAP four-disulfide core domain 3	0.8	0.75	0.94	0.52
KCNK9	KCNK9;K2p9.1;KT3.2;MGC138268;MGC138270;TASK-3;TASK3	potassium channel, subfamily K, member 9	0.8	0.78	0.94	0.61
SERPINB8	SERPINB8;CAP2;PI8	serpin peptidase inhibitor, clade B (ovalbumin), member 8	0.8	0.73	0.94	0.61
MAP3K12	MAP3K12;DLK;MEKK12;MUK;ZPK;ZPKP1	mitogen-activated protein kinase kinase kinase 12	0.8	0.76	0.94	0.63
SLC38A6	SLC38A6;MGC102697;NAT-1	solute carrier family 38, member 6	0.8	0.76	0.94	0.59
NFATC2IP	NFATC2IP;ESC2;FLJ14639;MGC126790;MGC138387;NIP45;RAD60	nuclear factor of activated T-cells, cytoplasmic, calcineurin-dependent 2 interacting protein	0.8	0.75	0.94	0.54
ASCC2	ASCC2;ASC1p100;p100	activating signal cointegrator 1 complex subunit 2	0.8	0.8	0.94	0.56
ENAH	ENAH;ENA;MENA;NDPP1	enabled homolog (Drosophila)	0.8	0.74	0.94	0.61
RNASEK	RNASEK;MGC48891;MGC71993	ribonuclease, RNase K	0.8	0.74	0.94	0.56
COL18A1-AS1	COL18A1-AS1;C21orf123;MGC129884;MGC129885;NCRNA00175;PRED80	COL18A1 antisense RNA 1 (non-protein coding)	0.8	0.74	0.94	0.56
XAGE2	XAGE2;CT12.2;GAGED3;XAGE-2;XAGE2B	X antigen family, member 2	0.79	0.76	0.94	0.57
STX16	STX16;MGC90328;SYN16	syntaxin 16	0.79	0.68	0.94	0.53
NLGN4X	NLGN4X;ASPGX2;AUTSX2;HLNX;HNL4X;HNLX;KIAA1260;MGC22376;NLGN;NLGN4	neuroligin 4, X-linked	0.78	0.71	0.94	0.61
AGTPBP1	AGTPBP1;CCP1;DKFZp686M20191;KIAA1035;NNA1	ATP/GTP binding protein 1	0.78	0.68	0.94	0.51
PCSK1N	PCSK1N;PROSAAS;SAAS	proprotein convertase subtilisin/kexin type 1 inhibitor	0.78	0.7	0.94	0.59
PANX1	PANX1;MGC21309;MRS1;PX1;UNQ2529	pannexin 1	0.78	0.74	0.94	0.5
NOXRED1	NOXRED1;C14orf148;FLJ32809	NADP-dependent oxidoreductase domain containing 1	0.78	0.74	0.94	0.54
FLJ14054	FLJ14054	ENTREZ STATUS: REMAPPED TO GENE ID: 4883	0.78	0.78	0.94	0.55
DCAF8L1	DCAF8L1;FLJ46244;WDR42B	DDB1 and CUL4 associated factor 8-like 1	0.78	0.81	0.94	0.59
SRSF5	SRSF5;HRS;SFRS5;SRP40	serine/arginine-rich splicing factor 5	0.78	0.74	0.94	0.51
SLC10A6	SLC10A6;MGC129575;MGC129576;SOAT	solute carrier family 10 (sodium/bile acid cotransporter family), member 6	0.77	0.7	0.94	0.51
UCP3	UCP3;SLC25A9	uncoupling protein 3 (mitochondrial, proton carrier)	0.77	0.72	0.94	0.53
C8orf47	C8orf47;FLJ39553	chromosome 8 open reading frame 47	0.77	0.66	0.94	0.51
CYB5R2	CYB5R2;B5R.2	cytochrome b5 reductase 2	0.77	0.72	0.94	0.53
SCAMP1	SCAMP1;SCAMP;SCAMP37	secretory carrier membrane protein 1	0.77	0.68	0.94	0.54
TSPYL6	TSPYL6;DKFZp434B125	TSPY-like 6	0.76	0.72	0.94	0.5
IL17RE	IL17RE;FLJ23658;MGC71884	interleukin 17 receptor E	0.76	0.72	0.94	0.5
C1orf159	C1orf159;FLJ20584;FLJ21143;FLJ36119	chromosome 1 open reading frame 159	0.76	0.65	0.94	0.51
CLDN15	CLDN15;FLJ42715;MGC19536	claudin 15	0.76	0.73	0.94	0.5
CNTROB	CNTROB;LIP8	centrobin, centrosomal BRCA2 interacting protein	0.76	0.7	0.94	0.52
CARD10	CARD10;BIMP1;CARMA3;MGC142219	caspase recruitment domain family, member 10	0.76	0.78	0.94	0.58
PEX2	PEX2;PAF1;PMP3;PMP35;PXMP3;RNF72;ZWS3	peroxisomal biogenesis factor 2	0.76	0.7	0.94	0.63
ARHGAP32	ARHGAP32;GC-GAP;GRIT;KIAA0712;MGC1892;PX-RICS;RICS;p200RhoGAP;p250GAP	Rho GTPase activating protein 32	0.76	0.76	0.94	0.53
CDC42EP1	CDC42EP1;BORG5;CEP1;MGC15316;MSE55	CDC42 effector protein (Rho GTPase binding) 1	0.75	0.68	0.94	0.53
TEKT3	TEKT3;FLJ32828	tektin 3	0.74	0.68	0.94	0.53
HOXD3	HOXD3;HOX1D;HOX4;HOX4A;Hox-4.1;MGC10470	homeobox D3	0.74	0.69	0.94	0.47
PDE8B	PDE8B;ADSD;FLJ11212;PPNAD3	phosphodiesterase 8B	0.74	0.7	0.94	0.54
GLT25D2	GLT25D2;C1orf17;FLJ37771;FLJ37873;KIAA0584	glycosyltransferase 25 domain containing 2	0.74	0.74	0.94	0.52
BAIAP2L2	BAIAP2L2;FLJ22582	BAI1-associated protein 2-like 2	0.74	0.76	0.94	0.5
THSD4	THSD4;ADAMTSL6;FLJ13710;FVSY9334;PRO34005	thrombospondin, type I, domain containing 4	0.74	0.68	0.94	0.51
IRS2	IRS2;IRS-2	insulin receptor substrate 2	0.74	0.67	0.94	0.5
CSGALNACT2	CSGALNACT2;CHGN2;DKFZp686H13226;FLJ43310;GALNACT-2;GALNACT2;MGC40204	chondroitin sulfate N-acetylgalactosaminyltransferase 2	0.74	0.78	0.94	0.54
DNAJC10	DNAJC10;DKFZp434J1813;ERdj5;JPDI;MGC104194	DnaJ (Hsp40) homolog, subfamily C, member 10	0.74	0.66	0.94	0.51
ESYT2	ESYT2;CHR2SYT;E-Syt2;FAM62B;KIAA1228	extended synaptotagmin-like protein 2	0.74	0.76	0.94	0.58
PCYT1B	PCYT1B;CCTB;CTB	phosphate cytidylyltransferase 1, choline, beta	0.73	0.68	0.94	0.51
DNM1	DNM1;DNM	dynamin 1	0.73	0.68	0.94	0.53
VAX2	VAX2;DRES93	ventral anterior homeobox 2	0.73	0.68	0.94	0.53
FOXO1	FOXO1;FKH1;FKHR;FOXO1A	forkhead box O1	0.73	0.74	0.94	0.52
NUSAP1	NUSAP1;ANKT;BM037;FLJ13421;LNP;NUSAP;PRO0310p1;Q0310;SAPL	nucleolar and spindle associated protein 1	0.73	0.7	0.94	0.53
IGSF1	IGSF1;IGCD1;IGDC1;INHBP;KIAA0364;MGC75490;PGSF2;p120	immunoglobulin superfamily, member 1	0.72	0.69	0.94	0.53
TLE2	TLE2;ESG;ESG2;FLJ41188;GRG2	transducin-like enhancer of split 2 (E(sp1) homolog, Drosophila)	0.72	0.64	0.94	0.5
PRX	PRX;CMT4F;KIAA1620	periaxin	0.72	0.74	0.94	0.54
NRG2	NRG2;DON1;HRG2;NTAK	neuregulin 2	0.72	0.68	0.94	0.52
ICAM5	ICAM5;TLCN;TLN	intercellular adhesion molecule 5, telencephalin	0.72	0.69	0.94	0.5
SPOPL	SPOPL;FLJ53775	speckle-type POZ protein-like	0.72	0.7	0.94	0.53
ZDHHC3	ZDHHC3;DHHC-3;DKFZp313D2314;FLJ20209;FLJ45940;GODZ;ZNF373	zinc finger, DHHC-type containing 3	0.72	0.68	0.94	0.53
TRMU	TRMU;MGC99627;MTO2;MTU1;TRMT;TRMT1;TRNT1	tRNA 5-methylaminomethyl-2-thiouridylate methyltransferase	0.71	0.66	0.94	0.49
PLBD1	PLBD1;FLJ22662	phospholipase B domain containing 1	0.71	0.7	0.94	0.49
TMEM26	TMEM26;DKFZp686D09128;MGC35010	transmembrane protein 26	0.7	0.66	0.94	0.46
FAM170A	FAM170A;MGC149385;ZNFD	family with sequence similarity 170, member A	0.7	0.7	0.94	0.56
DEFA5	DEFA5;DEF5;HD-5;MGC129728	defensin, alpha 5, Paneth cell-specific	0.7	0.69	0.94	0.49
DBX2	DBX2;FLJ16139	developing brain homeobox 2	0.7	0.7	0.94	0.5

ZNF641	ZNF641;DKFZp667D1012;FLJ31295	zinc finger protein 641	0.7	0.72	0.94	0.47
MIER2	MIER2;KIAA1193	mesoderm induction early response 1, family member 2	0.7	0.7	0.94	0.49
ZNF503	ZNF503;FLJ45745;MGC2555;NOLZ-1;NOLZ1	zinc finger protein 503	0.7	0.68	0.94	0.49
KIAA0494	KIAA0494;RP11-8J9.3	KIAA0494	0.7	0.68	0.94	0.52
FAM195A	FAM195A;C16orf14;MGC104301;MGC15416;c349E10.1	family with sequence similarity 195, member A	0.7	0.58	0.94	0.46
AKR7L	AKR7L;AFAR3;AFB1-AR3;AKR7A4	aldo-keto reductase family 7-like	0.7	0.66	0.94	0.44
OR2B11	OR2B11	olfactory receptor, family 2, subfamily B, member 11	0.69	0.68	0.94	0.55
ZNF157	ZNF157;HZF22	zinc finger protein 157	0.69	0.66	0.94	0.44
MLKL	MLKL;FLJ34389	mixed lineage kinase domain-like	0.69	0.65	0.94	0.54
PLEKHH1	PLEKHH1	pleckstrin homology domain containing, family H (with MyTH4 domain) member 1	0.69	0.62	0.94	0.49
FAM53B	FAM53B;FLJ13019;KIAA0140;RP11-12J10.2;bA12J10.2	family with sequence similarity 53, member B	0.69	0.71	0.94	0.47
ZNF784	ZNF784;MGC75238	zinc finger protein 784	0.69	0.62	0.94	0.47
AP2A1	AP2A1;ADTAA;AP2-ALPHA;CLAPA1	adaptor-related protein complex 2, alpha 1 subunit	0.69	0.64	0.94	0.48
ZNF391	ZNF391;dJ153G14.3	zinc finger protein 391	0.68	0.6	0.94	0.49
RNF113A	RNF113A;Cwc24;RNF113;ZNF183	ring finger protein 113A	0.68	0.64	0.94	0.57
BBS7	BBS7;BBS2L1;FLJ10715	Bardet-Biedl syndrome 7	0.68	0.62	0.94	0.47
FAM57B	FAM57B;DKFZp434I2117	family with sequence similarity 57, member B	0.68	0.66	0.94	0.45
DCTN6	DCTN6;WS-3;WS3;p27	dynactin 6	0.68	0.62	0.94	0.52
RNF167	RNF167;5730408C10Rik;DKFZp566H073;RING105	ring finger protein 167	0.68	0.63	0.94	0.57
TALDO1	TALDO1;TAL;TAL-H;TALDOR;TALH	transaldolase 1	0.68	0.66	0.94	0.44
C11orf53	C11orf53;MGC50104	chromosome 11 open reading frame 53	0.67	0.69	0.94	0.46
PDLIM1	PDLIM1;CLIM1;CLP-36;CLP36;hCLIM1	PDZ and LIM domain 1	0.67	0.7	0.94	0.48
DHRS11	DHRS11;ARPG836;FLJ39232;MGC4172;SDR24C1	dehydrogenase/reductase (SDR family) member 11	0.67	0.64	0.94	0.5
ESR2	ESR2;ER-BETA;ESR-BETA;ESRB;ESTRB;Erb;NR3A2	estrogen receptor 2 (ER beta)	0.66	0.6	0.94	0.47
ANGPT2	ANGPT2;AGPT2;ANG2	angiopoietin 2	0.66	0.61	0.94	0.45
P2RX2	P2RX2;MGC129601;P2X2	purinergic receptor P2X, ligand-gated ion channel, 2	0.66	0.63	0.94	0.49
ADAT3	ADAT3;FWP005;MST121;MSTP121;S863-5;TAD3	adenosine deaminase, tRNA-specific 3	0.66	0.6	0.94	0.47
RNF41	RNF41;FLRF;MGC45228;NRDP1	ring finger protein 41	0.66	0.62	0.94	0.55
TACC1	TACC1;DKFZp686K18126;FLJ42304;Ga55;KIAA1103	transforming, acidic coiled-coil containing protein 1	0.66	0.64	0.94	0.48
TCEANC2	TCEANC2;C1orf83;FLJ32112;FLJ39169;RP4-758J24.3	transcription elongation factor A (SII) N-terminal and central domain containing 2	0.66	0.66	0.94	0.46
EPS8L3	EPS8L3;EPS8R3;FLJ21522;MGC16817	EPS8-like 3	0.65	0.65	0.94	0.46
GNG5	GNG5;FLJ92393	guanine nucleotide binding protein (G protein), gamma 5	0.65	0.63	0.94	0.48
DNAJC25-GN	DNAJC25-GNG10	DNAJC25-GNG10 readthrough	0.65	0.58	0.94	0.45
CD28	CD28;MGC138290;Tp44	CD28 molecule	0.64	0.61	0.94	0.41
CCL1	CCL1;I-309;P500;SCYA1;SISe;TCA3	chemokine (C-C motif) ligand 1	0.64	0.62	0.94	0.42
CCK	CCK;MGC117187	cholecystokinin	0.64	0.62	0.94	0.47
FKBP7	FKBP7;FKBP23;MGC9420;PPIase	FK506 binding protein 7	0.64	0.64	0.94	0.48
HES2	HES2;bHLHb40	hairy and enhancer of split 2 (Drosophila)	0.64	0.62	0.94	0.47
TFAP4	TFAP4;AP-4;bHLHc41	transcription factor AP-4 (activating enhancer binding protein 4)	0.64	0.57	0.94	0.42
SNN	SNN	stannin	0.64	0.62	0.94	0.47
TCFL5	TCFL5;CHA;E2BP-1;Figlb;MGC46135;bHLHe82	transcription factor-like 5 (basic helix-loop-helix)	0.64	0.62	0.94	0.49
CTCF	CTCF	CCCTC-binding factor (zinc finger protein)	0.64	0.56	0.94	0.44
PDCD5	PDCD5;FLJ42784;MGC9294;TFAR19	programmed cell death 5	0.63	0.62	0.94	0.43
ZNF322	ZNF322;FLJ23393;HCG12;ZNF322A;ZNF388;ZNF489	zinc finger protein 322	0.63	0.58	0.94	0.48
GRK5	GRK5;FLJ39780;GPRK5	G protein-coupled receptor kinase 5	0.62	0.58	0.94	0.48
GDF2	GDF2;BMP-9;BMP9	growth differentiation factor 2	0.62	0.58	0.94	0.41
FBXL16	FBXL16;C16orf22;FLJ33735;Fbl16;MGC33974;c380A1.1	F-box and leucine-rich repeat protein 16	0.62	0.55	0.94	0.4
C16orf42	C16orf42;MGC24381	chromosome 16 open reading frame 42	0.62	0.64	0.94	0.43
MS4A14	MS4A14;MGC104289;MGC49828;MS4A16;NYD-SP21	membrane-spanning 4-domains, subfamily A, member 14	0.62	0.62	0.94	0.46
PABPN1L	PABPN1L;PABPNL1;ePABP2	poly(A) binding protein, nuclear 1-like (cytoplasmic)	0.62	0.52	0.94	0.43
APOA2	APOA2;Apo-AII;ApoA-II;apoAII	apolipoprotein A-II	0.61	0.56	0.94	0.4
MAN1C1	MAN1C1;HMIC;MAN1A3;MAN1C;pp6318	mannosidase, alpha, class 1C, member 1	0.61	0.56	0.94	0.44
NAGK	NAGK;GNK;HSA242910	N-acetylglucosamine kinase	0.61	0.57	0.94	0.48
AVPR1A	AVPR1A;AVPR1	arginine vasopressin receptor 1A	0.84	0.75	0.95	0.56
MYH13	MYH13;MyHC-eo	myosin, heavy chain 13, skeletal muscle	0.84	0.8	0.95	0.54
HSF5	HSF5;FLJ40311;HSF 5;HSTF 5;MGC134827	heat shock transcription factor family member 5	0.84	0.76	0.95	0.61
NPAS3	NPAS3;FLJ10003;FLJ11138;FLJ11605;MOP6;PASD6;bHLHe12	neuronal PAS domain protein 3	0.84	0.83	0.95	0.63
SSX6	SSX6;SSXP2;dJ54B20.1;psiSSX2	synovial sarcoma, X breakpoint 6 (pseudogene)	0.84	0.82	0.95	0.54
SLC35D3	SLC35D3;FRCL1;MGC102873;bA55K22.3	solute carrier family 35, member D3	0.84	0.82	0.95	0.56
ABAT	ABAT;FLJ17813;FLJ30272;GABA-AT;GABAT;NPD009	4-aminobutyrate aminotransferase	0.84	0.74	0.95	0.65
RASL10A	RASL10A;RRP22	RAS-like, family 10, member A	0.84	0.84	0.95	0.56
RHOD	RHOD;ARHD;RHOHP1;RHOM;Rho	ras homolog gene family, member D	0.84	0.86	0.95	0.64
RRAGC	RRAGC;FLJ13311;GTR2;RAGC	Ras-related GTP binding C	0.84	0.84	0.95	0.62
RAB3GAP1	RAB3GAP1;DKFZp434A012;KIAA0066;P130;RAB3GAP;RAB3GAP130;WARBM1	RAB3 GTPase activating protein subunit 1 (catalytic)	0.84	0.78	0.95	0.63
PGM2	PGM2;FLJ10983	phosphoglucomutase 2	0.84	0.85	0.95	0.56
DLD	DLD;DLDH;E3;GCSL;LAD;PHE3	dihydrolipoamide dehydrogenase	0.84	0.78	0.95	0.57
TMEM8A	TMEM8A;M83;TMEM6;TMEM8	transmembrane protein 8A	0.84	0.84	0.95	0.57
KIR2DS3	KIR2DS3;NKAT7	killer cell immunoglobulin-like receptor, two domains, short cytoplasmic tail, 3	0.84	0.76	0.95	0.55
ISCA1P1	ISCA1P1;ISCA1L	iron-sulfur cluster assembly 1 homolog (S. cerevisiae) pseudogene 1	0.84	0.78	0.95	0.59
DOCK5	DOCK5;DKFZp451J181;DKFZp779M164;DKFZp781J211	dedicator of cytokinesis 5	0.83	0.81	0.95	0.61
STAG1	STAG1;DKFZp781D1416;SA1;SCC3A	stromal antigen 1	0.83	0.78	0.95	0.59
CDC25B	CDC25B	cell division cycle 25 homolog B (S. pombe)	0.83	0.73	0.95	0.55
ASF1B	ASF1B;CIA-II;FLJ10604	ASF1 anti-silencing function 1 homolog B (S. cerevisiae)	0.83	0.7	0.95	0.59
CPSF2	CPSF2;CPSF100;KIAA1367	cleavage and polyadenylation specific factor 2, 100kDa	0.83	0.72	0.95	0.57
CD63	CD63;LAMP-3;ME491;MLA1;OMA81H;TSPAN30	CD63 molecule	0.83	0.75	0.95	0.55
MICALCL	MICALCL;Ebitein1;FLJ14966	MICAL C-terminal like	0.82	0.76	0.95	0.57
KHK	KHK	ketohexokinase (fructokinase)	0.82	0.76	0.95	0.69
GPBAR1	GPBAR1;BG37;GPCR19;GPR131;M-BAR;MGC40597;TGR5	G protein-coupled bile acid receptor 1	0.82	0.72	0.95	0.55
HIST1H2BD	HIST1H2BD;H2B.1B;H2B/b;H2BFB;HIRIP2;MGC90432;dJ221C16.6	histone cluster 1, H2bd	0.82	0.8	0.95	0.54
C9orf82	C9orf82;FLJ13657;RP11-337A23.1	chromosome 9 open reading frame 82	0.82	0.82	0.95	0.66
PTRF	PTRF;CAVIN;CAVIN1;CGL4;FLJ90031;cavin-1	polymerase I and transcript release factor	0.82	0.78	0.95	0.55
ADAT1	ADAT1;HADAT1	adenosine deaminase, tRNA-specific 1	0.82	0.72	0.95	0.64
FARS2	FARS2;FARS1;HSPC320;PheRS;dJ520B18.2	phenylalanyl-tRNA synthetase 2, mitochondrial	0.82	0.72	0.95	0.64
RPS6KB2	RPS6KB2;KLS;P70-beta;P70-beta-1;P70-beta-2;S6K-beta2;S6K2;SRK;STK14B;p70(S6K)-beta;p70S6Kb	ribosomal protein S6 kinase, 70kDa, polypeptide 2	0.82	0.78	0.95	0.65
UBE2S	UBE2S;E2-EPF;E2EPF;EPF5	ubiquitin-conjugating enzyme E2S	0.82	0.72	0.95	0.54
HNRNPD	HNRNPD;AUF1;AUF1A;HNRPD;P37;hnRNPD0	heterogeneous nuclear ribonucleoprotein D (AU-rich element RNA binding protein 1, 37kDa)	0.82	0.76	0.95	0.65
NAV3	NAV3;KIAA0938;POMFIL1;STEERIN3;unc53H3	neuron navigator 3	0.81	0.78	0.95	0.57
FAM83B	FAM83B;C6orf143;FLJ30642;MGC126677;MGC138480	family with sequence similarity 83, member B	0.81	0.82	0.95	0.63
KIAA1147	KIAA1147;LCHN;PRO2561	KIAA1147	0.81	0.8	0.95	0.59
COPS5	COPS5;CSN5;JAB1;MGC3149;MOV-34;SGN5	COP9 constitutive photomorphogenic homolog subunit 5 (Arabidopsis)	0.81	0.72	0.95	0.63
FAM206A	FAM206A;C9orf6;CG-8;FLJ20457	family with sequence similarity 206, member A	0.81	0.74	0.95	0.57
C1orf88	C1orf88;FLJ23853;MGC126550;PIFO	chromosome 1 open reading frame 88	0.8	0.84	0.95	0.56
ACSS3	ACSS3;FLJ21963	acyl-CoA synthetase short-chain family member 3	0.8	0.7	0.95	0.61
ITIH3	ITIH3;H3P	inter-alpha-trypsin inhibitor heavy chain 3	0.8	0.73	0.95	0.52
B3GNT5	B3GNT5;B3GN-T5;beta3Gn-T5	UDP-GlcNAc:betaGal beta-1,3-N-acetylglucosaminyltransferase 5	0.8	0.71	0.95	0.57
BNIP1	BNIP1;NIP1;SEC20;TRG-8	BCL2/adenovirus E1B 19kDa interacting protein 1	0.8	0.7	0.95	0.56
MAML1	MAML1;FLJ53540;KIAA0200;Mam-1;Mam1	mastermind-like 1 (Drosophila)	0.8	0.74	0.95	0.64
B3GAT3	B3GAT3;GLCATI	beta-1,3-glucuronyltransferase 3 (glucuronosyltransferase I)	0.8	0.74	0.95	0.54
GPR161	GPR161;FLJ33952;RE2	G protein-coupled receptor 161	0.8	0.74	0.95	0.66
C14orf133	C14orf133;FLJ12707;SPE-39;SPE39;VIPAR;VPS16B;hSPE-39	chromosome 14 open reading frame 133	0.8	0.78	0.95	0.59
ZBTB1	ZBTB1;KIAA0997;ZNF909	zinc finger and BTB domain containing 1	0.8	0.74	0.95	0.62
PUS7	PUS7;FLJ20485;KIAA1897;MGC17720	pseudouridylate synthase 7 homolog (S. cerevisiae)	0.8	0.72	0.95	0.58
COX6B1	COX6B1;COX6B;COXG;COXVIb1	cytochrome c oxidase subunit VIb polypeptide 1 (ubiquitous)	0.8	0.76	0.95	0.59
MDH1	MDH1;MDH-s;MDHA;MGC:1375;MOR2	malate dehydrogenase 1, NAD (soluble)	0.8	0.8	0.95	0.61
B2M	B2M	beta-2-microglobulin	0.8	0.82	0.95	0.62
TNNI1	TNNI1;DKFZp451O223;SSTNI;TNN1	troponin I type 1 (skeletal, slow)	0.79	0.74	0.95	0.56
FAM176B	FAM176B;C1orf78;FLJ10647;RP11-268J15.2	family with sequence similarity 176, member B	0.79	0.75	0.95	0.54
LPA	LPA;AK38;APOA;LP	lipoprotein, Lp(a)	0.78	0.68	0.95	0.51
ARMC4	ARMC4;DKFZp434P1735;FLJ10376;FLJ10817;FLJ32798	armadillo repeat containing 4	0.78	0.72	0.95	0.57
THPO	THPO;MGC163194;MGDF;MKCSF;ML;MPLLG;TPO	thrombopoietin	0.78	0.72	0.95	0.52
SUPT3H	SUPT3H;SPT3;SPT3L	suppressor of Ty 3 homolog (S. cerevisiae)	0.78	0.73	0.95	0.57
TIAM1	TIAM1;FLJ36302	T-cell lymphoma invasion and metastasis 1	0.78	0.72	0.95	0.51
ACSL5	ACSL5;ACS2;ACS5;FACL5	acyl-CoA synthetase long-chain family member 5	0.78	0.72	0.95	0.53
CCL2	CCL2;GDCF-2;HC11;HSMCR30;MCAF;MCP-1;MCP1;MGC9434;SCYA2;SMC-CF	chemokine (C-C motif) ligand 2	0.78	0.73	0.95	0.55
GCSH	GCSH;GCE;NKH	glycine cleavage system protein H (aminomethyl carrier)	0.78	0.68	0.95	0.54
C1orf227	C1orf227	chromosome 1 open reading frame 227	0.78	0.74	0.95	0.5
PPAN	PPAN;BXDC3;MGC14226;MGC45852;SSF;SSF-1;SSF1;SSF2	peter pan homolog (Drosophila)	0.78	0.71	0.95	0.62
HAUS8	HAUS8;DGT4;HICE1;MGC20533;NY-SAR-48	HAUS augmin-like complex, subunit 8	0.78	0.78	0.95	0.56
TRPV4	TRPV4;CMT2C;HMSN2C;OTRPC4;SPSMA;SSQTL1;TRP12;VRL2;VROAC	transient receptor potential cation channel, subfamily V, member 4	0.77	0.72	0.95	0.56
LRRC7	LRRC7;DKFZp686I1147;KIAA1365;MGC144918	leucine rich repeat containing 7	0.77	0.8	0.95	0.53
ARHGEF18	ARHGEF18;KIAA0521;MGC15913;P114-RhoGEF	Rho/Rac guanine nucleotide exchange factor (GEF) 18	0.77	0.72	0.95	0.56
TRAF4	TRAF4;CART1;MLN62;RNF83	TNF receptor-associated factor 4	0.77	0.73	0.95	0.65
HBE1	HBE1;HBE	hemoglobin, epsilon 1	0.76	0.67	0.95	0.59
DMBT1	DMBT1;FLJ61058;GP340;MGC164738;muclin	deleted in malignant brain tumors 1	0.76	0.8	0.95	0.55
TP53I13	TP53I13;DSCP1	tumor protein p53 inducible protein 13	0.76	0.77	0.95	0.59
CCDC126	CCDC126;FLJ23031;MGC104248	coiled-coil domain containing 126	0.76	0.76	0.95	0.54
SNAPC2	SNAPC2;PTFDELTA;SNAP45	small nuclear RNA activating complex, polypeptide 2, 45kDa	0.76	0.77	0.95	0.51
RBPJ	RBPJ;CBF1;IGKJRB;IGKJRB1;KBF2;MGC61669;RBP-J;RBPJK;RBPSUH;SUH;csl	recombination signal binding protein for immunoglobulin kappa J region	0.76	0.74	0.95	0.59
SURF4	SURF4;ERV29;FLJ22993;MGC102753	surfeit 4	0.76	0.7	0.95	0.53

GHRH	GHRH;GHRF;GRF;INN;MGC119781	growth hormone releasing hormone	0.75	0.67	0.95	0.57
COL7A1	COL7A1;EBD1;EBDCT;EBR1	collagen, type VII, alpha 1	0.75	0.64	0.95	0.53
IFT46	IFT46;C11orf2;C11orf60;FLJ21827	intraflagellar transport 46 homolog (Chlamydomonas)	0.75	0.68	0.95	0.52
IL31RA	IL31RA;CRL;CRL3;GLM-R;GLMR;GPL;IL-31RA;MGC125346;PLCA2;PRO21384;hGLM-R	interleukin 31 receptor A	0.74	0.68	0.95	0.53
C16orf46	C16orf46;FLJ32702	chromosome 16 open reading frame 46	0.74	0.67	0.95	0.51
KRT76	KRT76;HUMCYT2A;KRT2B;KRT2P	keratin 76	0.74	0.72	0.95	0.51
ZNF529	ZNF529;KIAA1615	zinc finger protein 529	0.74	0.76	0.95	0.56
ZDHHC18	ZDHHC18;DHHC-18;DHHC18;DKFZp667O2416	zinc finger, DHHC-type containing 18	0.74	0.74	0.95	0.52
MEF2D	MEF2D;DKFZp686I1536	myocyte enhancer factor 2D	0.74	0.7	0.95	0.55
VPRBP	VPRBP;DCAF1;KIAA0800;MGC102804	Vpr (HIV-1) binding protein	0.74	0.64	0.95	0.51
FKBP2	FKBP2;FKBP-13;PPIase	FK506 binding protein 2, 13kDa	0.74	0.72	0.95	0.55
BOD1	BOD1;FAM44B	biorientation of chromosomes in cell division 1	0.74	0.69	0.95	0.52
TMEM146	TMEM146;MGC39581	transmembrane protein 146	0.73	0.74	0.95	0.52
TMC7	TMC7;DKFZp781O2274;FLJ21240	transmembrane channel-like 7	0.73	0.68	0.95	0.57
KLHL18	KLHL18;FLJ13703;FLJ61265;KIAA0795	kelch-like 18 (Drosophila)	0.73	0.7	0.95	0.53
INTS8	INTS8;C8orf52;FLJ20530;INT8;MGC131633	integrator complex subunit 8	0.73	0.69	0.95	0.5
PWP1	PWP1;IEF-SSP-9502	PWP1 homolog (S. cerevisiae)	0.73	0.7	0.95	0.47
PRR23B	PRR23B	proline rich 23B	0.73	0.72	0.95	0.54
SLC17A8	SLC17A8;DFNA25;VGLUT3	solute carrier family 17 (sodium-dependent inorganic phosphate cotransporter), member 8	0.72	0.64	0.95	0.51
GLI1	GLI1;GLI	GLI family zinc finger 1	0.72	0.7	0.95	0.55
FA2H	FA2H;FAAH;FAH1;FAXDC1;FLJ25287;SCS7;SPG35	fatty acid 2-hydroxylase	0.72	0.74	0.95	0.55
NICN1	NICN1;MGC12936	nicolin 1	0.72	0.7	0.95	0.48
C20orf151	C20orf151	chromosome 20 open reading frame 151	0.72	0.72	0.95	0.58
C6orf192	C6orf192;dJ55C23.6	chromosome 6 open reading frame 192	0.72	0.7	0.95	0.48
SMPD1	SMPD1;ASM;ASMASE;NPD	sphingomyelin phosphodiesterase 1, acid lysosomal	0.72	0.66	0.95	0.48
KRT23	KRT23;CK23;DKFZp434G032;HAIK1;K23;MGC26158	keratin 23 (histone deacetylase inducible)	0.72	0.65	0.95	0.5
PRR15L	PRR15L;ATAD4;MGC11242	proline rich 15-like	0.72	0.7	0.95	0.52
FAM71F1	FAM71F1;FAM137A;NYD-SP18	family with sequence similarity 71, member F1	0.71	0.7	0.95	0.57
VPS13B	VPS13B;CHS1;COH1;DKFZp313I0811;KIAA0532	vacuolar protein sorting 13 homolog B (yeast)	0.71	0.7	0.95	0.51
PGLS	PGLS;6PGL	6-phosphogluconolactonase	0.71	0.68	0.95	0.53
C8orf33	C8orf33;FLJ20989	chromosome 8 open reading frame 33	0.71	0.66	0.95	0.5
LIMA1	LIMA1;EPLIN;FLJ38853;MGC131726;SREBP3	LIM domain and actin binding 1	0.71	0.72	0.95	0.5
DROSHA	DROSHA;ETOHI2;HSA242976;RANSE3L;RN3;RNASE3L;RNASEN	drosha, ribonuclease type III	0.71	0.74	0.95	0.52
TAF7L	TAF7L;CT40;FLJ23157;TAF2Q	TAF7-like RNA polymerase II, TATA box binding protein (TBP)-associated factor, 50kDa	0.7	0.64	0.95	0.5
DPRX	DPRX	divergent-paired related homeobox	0.7	0.69	0.95	0.47
TSPAN18	TSPAN18;FLJ14609;TSPAN	tetraspanin 18	0.7	0.66	0.95	0.55
CA2	CA2;CA-II;CAII;Car2	carbonic anhydrase II	0.7	0.63	0.95	0.49
SEPX1	SEPX1;MGC3344;MSRB1;SELR;SELX	selenoprotein X, 1	0.7	0.75	0.95	0.52
ERLEC1	ERLEC1;C2orf30;CIM;CL24936;CL25084;XTP3-B;XTP3TPB	endoplasmic reticulum lectin 1	0.7	0.73	0.95	0.53
VWCE	VWCE;FLJ32009;URG11;VWC1	von Willebrand factor C and EGF domains	0.68	0.66	0.95	0.49
NME6	NME6;IPIA-ALPHA;NDK 6;NM23-H6	non-metastatic cells 6, protein expressed in (nucleoside-diphosphate kinase)	0.68	0.71	0.95	0.5
RRAS	RRAS	related RAS viral (r-ras) oncogene homolog	0.68	0.61	0.95	0.49
PPP1R2	PPP1R2;IPP2;MGC87148	protein phosphatase 1, regulatory (inhibitor) subunit 2	0.68	0.61	0.95	0.46
ATP5I	ATP5I;ATP5K;MGC12532	ATP synthase, H+ transporting, mitochondrial Fo complex, subunit E	0.68	0.6	0.95	0.51
GPR17	GPR17;DKFZp686M18273	G protein-coupled receptor 17	0.67	0.62	0.95	0.56
KRT26	KRT26;CK26;K25IRS2;K26;KRT25B	keratin 26	0.66	0.62	0.95	0.46
ENPP6	ENPP6;MGC33971;NPP6	ectonucleotide pyrophosphatase/phosphodiesterase 6	0.66	0.58	0.95	0.51
OR2L8	OR2L8	olfactory receptor, family 2, subfamily L, member 8	0.66	0.65	0.95	0.5
MAGEL2	MAGEL2;NDNL1;nM15	MAGE-like 2	0.66	0.65	0.95	0.43
NOS1AP	NOS1AP;6330408P19Rik;CAPON;MGC138500	nitric oxide synthase 1 (neuronal) adaptor protein	0.66	0.68	0.95	0.46
BET1L	BET1L;BET1L1;GOLIM3;GS15;HSPC197	blocked early in transport 1 homolog (S. cerevisiae)-like	0.66	0.7	0.95	0.51
KRTAP2-4	KRTAP2-4;KAP2.1B;KAP2.4;KRTAP2.4;MGC119882;MGC119884;MGC119885;MGC74790	keratin associated protein 2-4	0.66	0.6	0.95	0.43
PPHLN1	PPHLN1;HSPC232;MGC48786	periphilin 1	0.66	0.66	0.95	0.44
VRTN	VRTN;C14orf115;FLJ10811;vertnin	vertebrae development homolog (pig)	0.66	0.63	0.95	0.44
EMP3	EMP3;YMP	epithelial membrane protein 3	0.65	0.64	0.95	0.52
FAM116B	FAM116B;MGC33692	family with sequence similarity 116, member B	0.65	0.66	0.95	0.51
MYO1G	MYO1G;HA2;MGC142104;MHAG	myosin IG	0.64	0.6	0.95	0.47
ZNF223	ZNF223	zinc finger protein 223	0.64	0.66	0.95	0.49
ARID4A	ARID4A;RBBP-1;RBBP1;RBP-1;RBP1	AT rich interactive domain 4A (RBP1-like)	0.64	0.59	0.95	0.51
CNN1	CNN1;SMCC;Sm-Calp	calponin 1, basic, smooth muscle	0.64	0.64	0.95	0.45
PMEPA1	PMEPA1;STAG1;TMEPAI	prostate transmembrane protein, androgen induced 1	0.64	0.66	0.95	0.49
TNFAIP2	TNFAIP2;B94;EXOC3L3	tumor necrosis factor, alpha-induced protein 2	0.64	0.59	0.95	0.47
STAG3L2	STAG3L2;MGC131759;STAG3L1;STAG3L3	stromal antigen 3-like 2	0.64	0.58	0.95	0.42
PSMD4	PSMD4;AF;AF-1;ASF;MCB1;Rpn10;S5A;pUB-R5	proteasome (prosome, macropain) 26S subunit, non-ATPase, 4	0.64	0.63	0.95	0.47
CDK11B	CDK11B;CDC2L1;CDK11;CDK11-p110;CDK11-p46;CDK11-p58;CLK-1;FLJ59152;PK58;p58;p58CDC2L1;p58CLK-1	cyclin-dependent kinase 11B	0.64	0.6	0.95	0.5
CILP2	CILP2;CLIP-2;MGC45771	cartilage intermediate layer protein 2	0.61	0.61	0.95	0.49
NR1H3	NR1H3;LXR-a;LXRA;RLD-1	nuclear receptor subfamily 1, group H, member 3	0.61	0.55	0.95	0.43
OLFML2A	OLFML2A;FLJ00237;PRO34319	olfactomedin-like 2A	0.61	0.6	0.95	0.47
CHTOP	CHTOP;C1orf77;DKFZp547E1010;FL-SRAG;FLJ40551;FOP;MGC131924;MGC86949;RP1-178F15.2;SRAG;SRAG-3;SRAG-5;pp7704	chromatin target of PRMT1	0.61	0.6	0.95	0.45
LRRC4C	LRRC4C;KIAA1580;NGL-1;NGL1	leucine rich repeat containing 4C	0.84	0.78	0.96	0.59
PLCD4	PLCD4;MGC12837	phospholipase C, delta 4	0.84	0.82	0.96	0.65
NRG3	NRG3;HRG3;pro-NRG3	neuregulin 3	0.84	0.83	0.96	0.63
GNMT	GNMT	glycine N-methyltransferase	0.84	0.78	0.96	0.61
RASD2	RASD2;MGC:4834;Rhes;TEM2	RASD family, member 2	0.84	0.83	0.96	0.63
ANKRD5	ANKRD5;FLJ21669;dJ839B4.6	ankyrin repeat domain 5	0.84	0.8	0.96	0.59
EHD1	EHD1;FLJ42622;FLJ44618;H-PAST;HPAST1;PAST;PAST1	EH-domain containing 1	0.84	0.77	0.96	0.59
MEIS1	MEIS1;MGC43380	Meis homeobox 1	0.84	0.87	0.96	0.65
SLCO1B7	SLCO1B7;LST-3TM12;LST3	solute carrier organic anion transporter family, member 1B7 (non-functional)	0.84	0.78	0.96	0.59
SLC9A2	SLC9A2;NHE2	solute carrier family 9 (sodium/hydrogen exchanger), member 2	0.83	0.82	0.96	0.63
DGAT1	DGAT1;ARGP1;DGAT	diacylglycerol O-acyltransferase 1	0.83	0.76	0.96	0.57
ACOT6	ACOT6;C14orf42;MGC161656;MGC161658;c14_5530	acyl-CoA thioesterase 6	0.82	0.82	0.96	0.64
LOC285033	LOC285033;MGC126779;MGC126781	uncharacterized LOC285033	0.82	0.87	0.96	0.64
ZNF133	ZNF133;ZNF150;pHZ-13;pHZ-66	zinc finger protein 133	0.82	0.82	0.96	0.55
LINGO4	LINGO4;DAAT9248;LRRN6D;PRO34002	leucine rich repeat and Ig domain containing 4	0.82	0.75	0.96	0.58
PDE4C	PDE4C;DPDE1;MGC126222	phosphodiesterase 4C, cAMP-specific	0.82	0.84	0.96	0.55
ACSM3	ACSM3;SA;SAH	acyl-CoA synthetase medium-chain family member 3	0.82	0.76	0.96	0.56
KIAA0513	KIAA0513	KIAA0513	0.82	0.79	0.96	0.55
VEZT	VEZT;DKFZp761C241;VEZATIN	vezatin, adherens junctions transmembrane protein	0.82	0.77	0.96	0.59
ERCC5	ERCC5;COFS3;ERCM2;UVDR;XPG;XPGC	excision repair cross-complementing rodent repair deficiency, complementation group 5	0.82	0.78	0.96	0.58
BRAP	BRAP;BRAP2;IMP;RNF52	BRCA1 associated protein	0.82	0.78	0.96	0.7
PRR14L	PRR14L;C22orf30;DKFZp781P2328;FLJ23059;FLJ31368;FLJ33247;MGC50372	proline rich 14-like	0.82	0.82	0.96	0.59
GRM2	GRM2;GLUR2;GPRC1B;MGLUR2;mGlu2	glutamate receptor, metabotropic 2	0.81	0.76	0.96	0.68
UBE2L3	UBE2L3;E2-F1;L-UBC;UBCH7;UbcM4	ubiquitin-conjugating enzyme E2L 3	0.81	0.71	0.96	0.54
ARR3	ARR3;ARRX	arrestin 3, retinal (X-arrestin)	0.8	0.81	0.96	0.61
PION	PION;DKFZp667B242;GSAP;MGC126548	pigeon homolog (Drosophila)	0.8	0.78	0.96	0.53
SLC5A11	SLC5A11;KST1;RKST1;SGLT6;SMIT2	solute carrier family 5 (sodium/glucose cotransporter), member 11	0.8	0.84	0.96	0.58
RHOXF2	RHOXF2;CT107;PEPP-2;PEPP2;THG1	Rhox homeobox family, member 2	0.8	0.78	0.96	0.59
BMP8A	BMP8A;FLJ14351;FLJ45264	bone morphogenetic protein 8a	0.8	0.74	0.96	0.56
PER1	PER1;MGC88021;PER;RIGUI;hPER	period homolog 1 (Drosophila)	0.8	0.8	0.96	0.61
FOSB	FOSB;AP-1;DKFZp686C0818;G0S3;GOS3;GOSB;MGC42291	FBJ murine osteosarcoma viral oncogene homolog B	0.8	0.77	0.96	0.6
TNK2	TNK2;ACK;ACK-1;ACK1;FLJ44758;FLJ45547;p21cdc42Hs	tyrosine kinase, non-receptor, 2	0.8	0.76	0.96	0.63
FXYD6	FXYD6	FXYD domain containing ion transport regulator 6	0.8	0.81	0.96	0.62
WDR54	WDR54;FLJ12953	WD repeat domain 54	0.8	0.8	0.96	0.59
MCOLN2	MCOLN2;FLJ36691;TRP-ML2;TRPML2	mucolipin 2	0.79	0.76	0.96	0.59
ATF5	ATF5;ATFX;FLJ34666;HMFN0395	activating transcription factor 5	0.79	0.74	0.96	0.58
CASD1	CASD1;C7orf12;FLJ21213;FLJ21879;FLJ41901;NBLA04196	CAS1 domain containing 1	0.79	0.76	0.96	0.56
FAM118B	FAM118B;FLJ21103	family with sequence similarity 118, member B	0.79	0.83	0.96	0.55
RAE1	RAE1;FLJ30608;MGC117333;MGC126076;MGC126077;MIG14;MRNP41;Mnrp41;dJ481F12.3;dJ800J21.1	RAE1 RNA export 1 homolog (S. pombe)	0.79	0.84	0.96	0.58
GLB1L3	GLB1L3;FLJ90231	galactosidase, beta 1-like 3	0.78	0.66	0.96	0.52
CD8A	CD8A;CD8;Leu2;MAL;p32	CD8a molecule	0.78	0.72	0.96	0.57
KRT71	KRT71;K6IRS1;KRT6IRS;KRT6IRS1;MGC119390;MGC119391	keratin 71	0.78	0.72	0.96	0.54
RBP2	RBP2;CRABP-II;CRBP2;CRBPII;RBPC2	retinol binding protein 2, cellular	0.78	0.78	0.96	0.55
MORN3	MORN3;MGC71497	MORN repeat containing 3	0.78	0.76	0.96	0.57
IRX1	IRX1;IRX-5;IRXA1	iroquois homeobox 1	0.78	0.78	0.96	0.52
ATP5S	ATP5S;ATPW;HSU79253	ATP synthase, H+ transporting, mitochondrial Fo complex, subunit s (factor B)	0.78	0.77	0.96	0.59
HCP5	HCP5;6S2650E;D6S2650E;P5-1	HLA complex P5 (non-protein coding)	0.78	0.67	0.96	0.56
TBKBP1	TBKBP1;KIAA0775;ProSAPiP2;SINTBAD	TBK1 binding protein 1	0.78	0.66	0.96	0.52
STK36	STK36;DKFZp434N0223;FU;KIAA1278	serine/threonine kinase 36	0.78	0.76	0.96	0.63
GPR153	GPR153;DKFZp762B2210;PGR1	G protein-coupled receptor 153	0.78	0.73	0.96	0.65
APRT	APRT;AMP;DKFZp686D13177;MGC125856;MGC125857;MGC129961	adenine phosphoribosyltransferase	0.78	0.7	0.96	0.56
NFKB1	NFKB1;DKFZp686C01211;EBP-1;KBF1;MGC54151;NF-kappa-B;NF-kappaB;NFKB-p105;NFKB-p50;NFkappaB;p105;p50	nuclear factor of kappa light polypeptide gene enhancer in B-cells 1	0.78	0.69	0.96	0.55
TMBIM1	TMBIM1;LFG3;MST100;MSTP100;RECS1	transmembrane BAX inhibitor motif containing 1	0.78	0.82	0.96	0.55
POGLUT1	POGLUT1;C3orf9;CLP46;KDELCL1;KTELC1;MDSRP;MGC32995;Rumi;hCLP46	protein O-glucosyltransferase 1	0.78	0.75	0.96	0.54
CT45A4	CT45A4;CT45-4;CT45.4;CT45A6	cancer/testis antigen family 45, member A4	0.78	0.76	0.96	0.55
C11orf85	C11orf85;MGC126364	chromosome 11 open reading frame 85	0.77	0.77	0.96	0.57
PNLDC1	PNLDC1;FLJ40240;MGC138451;dJ195P10.2	poly(A)-specific ribonuclease (PARN)-like domain containing 1	0.77	0.74	0.96	0.5
C9orf106	C9orf106;bA65J3.5	chromosome 9 open reading frame 106	0.77	0.78	0.96	0.55
KIAA0146	KIAA0146;FLJ35017	KIAA0146	0.77	0.7	0.96	0.6
SNX32	SNX32;DKFZp761P1320;FLJ30934;MGC42112;MGC57276;SNX6B	sorting nexin 32	0.76	0.7	0.96	0.54

ZNF615	ZNF615;DKFZp686O1554;FLJ33710;FLJ39372	zinc finger protein 615	0.76	0.74	0.96	0.5
CDA	CDA;CDD	cytidine deaminase	0.76	0.73	0.96	0.54
GGT7	GGT7;D20S101;GGT4;GGTL3;GGTL5;dJ18C9.2	gamma-glutamyltransferase 7	0.76	0.67	0.96	0.5
ZBTB11	ZBTB11;FLJ13426;MGC133303;ZNF-U69274;ZNF913	zinc finger and BTB domain containing 11	0.76	0.75	0.96	0.57
HEXIM2	HEXIM2;FLJ32384	hexamethylene bis-acetamide inducible 2	0.76	0.7	0.96	0.54
TMEM110	TMEM110;DKFZp667E1121;DKFZp779M0254;FLJ37613;FLJ42395;MGC52022	transmembrane protein 110	0.76	0.8	0.96	0.53
C12orf23	C12orf23;FLJ11721;FLJ13959;MGC17943	chromosome 12 open reading frame 23	0.76	0.74	0.96	0.55
AKR7A3	AKR7A3;AFAR2	aldo-keto reductase family 7, member A3 (aflatoxin aldehyde reductase)	0.75	0.7	0.96	0.51
RNFT2	RNFT2;FLJ14627;TMEM118	ring finger protein, transmembrane 2	0.75	0.71	0.96	0.64
ITPA	ITPA;C20orf37;HLC14-06-P;ITPase;dJ794I6.3	inosine triphosphatase (nucleoside triphosphate pyrophosphatase)	0.75	0.67	0.96	0.59
MYOZ1	MYOZ1;CS-2;FATZ;MYOZ	myozenin 1	0.74	0.69	0.96	0.53
NEU2	NEU2;MGC129579;SIAL2	sialidase 2 (cytosolic sialidase)	0.74	0.67	0.96	0.56
DDX31	DDX31;FLJ13633;FLJ14578;FLJ23349;PPP1R25	DEAD (Asp-Glu-Ala-Asp) box polypeptide 31	0.74	0.67	0.96	0.53
FJX1	FJX1;FLJ22416;FLJ25593	four jointed box 1 (Drosophila)	0.74	0.72	0.96	0.52
TSPAN17	TSPAN17;FBX23;FBXO23;MGC14859;MGC71255;TM4SF17	tetraspanin 17	0.74	0.67	0.96	0.53
ACSS2	ACSS2;ACAS2;ACECS;ACS;ACSA;DKFZp762G026;dJ1161H23.1	acyl-CoA synthetase short-chain family member 2	0.74	0.68	0.96	0.49
EIF3J	EIF3J;EIF3S1;eIF3-alpha;eIF3-p35	eukaryotic translation initiation factor 3, subunit J	0.74	0.73	0.96	0.57
EIF2S3	EIF2S3;EIF2;EIF2G;EIF2gamma;eIF-2gA	eukaryotic translation initiation factor 2, subunit 3 gamma, 52kDa	0.74	0.66	0.96	0.58
SYNRG	SYNRG;AP1GBP1;FLJ34482;MGC104959;SYNG	synergin, gamma	0.74	0.69	0.96	0.58
KRT34	KRT34;HA4;Ha-4;KRTHA4;hHa4	keratin 34	0.73	0.68	0.96	0.51
AGRP	AGRP;AGRT;ART;ASIP2;MGC118963	agouti related protein homolog (mouse)	0.73	0.74	0.96	0.52
FBXO30	FBXO30;FLJ41030;Fbx30;MGC21674	F-box protein 30	0.73	0.66	0.96	0.48
GNB4	GNB4	guanine nucleotide binding protein (G protein), beta polypeptide 4	0.73	0.74	0.96	0.49
KLHDC10	KLHDC10;KIAA0265;slim	kelch domain containing 10	0.73	0.68	0.96	0.49
PHGDH	PHGDH;3-PGDH;3PGDH;MGC3017;PDG;PGAD;PGD;PGDH;SERA	phosphoglycerate dehydrogenase	0.73	0.66	0.96	0.48
LRRC16B	LRRC16B;C14orf121;CARMIL3;crml-1	leucine rich repeat containing 16B	0.72	0.71	0.96	0.53
SOX21	SOX21;SOX25	SRY (sex determining region Y)-box 21	0.72	0.63	0.96	0.5
INSL3	INSL3;MGC119818;MGC119819;RLF;RLNL	insulin-like 3 (Leydig cell)	0.72	0.68	0.96	0.52
CORO7	CORO7;0610011B16Rik;CRN7;FLJ22021;FLJ44188;POD1	coronin 7	0.72	0.69	0.96	0.52
TMEM41B	TMEM41B;KIAA0033;MGC33897;MGC57262	transmembrane protein 41B	0.72	0.68	0.96	0.54
SERTAD1	SERTAD1;SEI1;TRIP-Br1	SERTA domain containing 1	0.72	0.68	0.96	0.53
BRD8	BRD8;SMAP;SMAP2;p120	bromodomain containing 8	0.72	0.68	0.96	0.48
BTAF1	BTAF1;KIAA0940;MGC138406;MOT1;TAF(II)170;TAF172;TAFII170	BTAF1 RNA polymerase II, B-TFIID transcription factor-associated, 170kDa (Mot1 homolog, S. cerevisiae)	0.72	0.64	0.96	0.56
FAM108A2	FAM108A2	ENTREZ STATUS: REMAPPED TO GENE ID: 100289252	0.72	0.7	0.96	0.47
INSIG2	INSIG2;MGC26273	insulin induced gene 2	0.71	0.75	0.96	0.55
PROM2	PROM2;MGC138714;PROML2	prominin 2	0.7	0.66	0.96	0.51
VENTX	VENTX;HPX42B;MGC119910;MGC119911;NA88A;VENTX2	VENT homeobox	0.7	0.74	0.96	0.51
LPCAT1	LPCAT1;AYTL2;FLJ12443;FLJ41609;PFAAP3;lpcat	lysophosphatidylcholine acyltransferase 1	0.7	0.64	0.96	0.48
CYLD	CYLD;BRSS;CDMT;CYLD1;CYLDI;EAC;FLJ20180;FLJ31664;FLJ78684;KIAA0849;MFT;MFT1;SBS;TEM;USPL2	cylindromatosis (turban tumor syndrome)	0.7	0.66	0.96	0.49
RBM25	RBM25;MGC105088;MGC117168;NET52;RED120;RNPC7;S164;Snu71;fSAP94	RNA binding motif protein 25	0.7	0.66	0.96	0.49
FAM136A	FAM136A;FLJ14668	family with sequence similarity 136, member A	0.7	0.64	0.96	0.54
PCNA	PCNA;MGC8367	proliferating cell nuclear antigen	0.7	0.68	0.96	0.54
SC5DL	SC5DL;ERG3;S5DES;SC5D	sterol-C5-desaturase (ERG3 delta-5-desaturase homolog, S. cerevisiae)-like	0.69	0.67	0.96	0.45
SNRNP70	SNRNP70;RNPU1Z;RPU1;SNRP70;Snp1;U1-70K;U170K;U1AP;U1RNP	small nuclear ribonucleoprotein 70kDa (U1)	0.69	0.6	0.96	0.47
DCST1	DCST1;FLJ32785	DC-STAMP domain containing 1	0.68	0.67	0.96	0.51
MED30	MED30;MED30S;MGC9890;THRAP6;TRAP25	mediator complex subunit 30	0.68	0.68	0.96	0.47
IRF9	IRF9;IRF-9;ISGF3;ISGF3G;p48	interferon regulatory factor 9	0.68	0.64	0.96	0.48
TMC6	TMC6;EV1;EVER1;EVIN1;FLJ17776;LAK-4P	transmembrane channel-like 6	0.68	0.65	0.96	0.51
TRAPPC9	TRAPPC9;IBP;IKBKBBP;KIAA1882;MGC4737;MGC4769;MRT13;NIBP;TRS120	trafficking protein particle complex 9	0.68	0.68	0.96	0.55
NEK4	NEK4;MGC33171;NRK2;STK2;pp12301	NIMA (never in mitosis gene a)-related kinase 4	0.68	0.66	0.96	0.55
CSMD1	CSMD1;KIAA1890;PPP1R24	CUB and Sushi multiple domains 1	0.67	0.62	0.96	0.49
GJB3	GJB3;CX31;DFNA2;DFNA2B;EKV;FLJ22486;MGC102938	gap junction protein, beta 3, 31kDa	0.67	0.62	0.96	0.48
SNX4	SNX4;ATG24B	sorting nexin 4	0.67	0.64	0.96	0.45
L3MBTL4	L3MBTL4;HsT1031	l(3)mbt-like 4 (Drosophila)	0.66	0.67	0.96	0.54
NRN1	NRN1;MGC44811;NRN;dJ380B8.2	neuritin 1	0.66	0.64	0.96	0.47
GPRASP2	GPRASP2;FLJ35662;FLJ37327;GASP2	G protein-coupled receptor associated sorting protein 2	0.66	0.61	0.96	0.47
SLC30A4	SLC30A4;ZNT4	solute carrier family 30 (zinc transporter), member 4	0.66	0.62	0.96	0.48
PTGES	PTGES;MGC10317;MGST-IV;MGST1-L1;MGST1L1;MPGES;PGES;PIG12;PP102;PP1294;TP53I12;mPGES-1	prostaglandin E synthase	0.66	0.64	0.96	0.43
ATF4	ATF4;CREB-2;CREB2;TAXREB67;TXREB	activating transcription factor 4 (tax-responsive enhancer element B67)	0.66	0.6	0.96	0.47
MCAT	MCAT;FASN2C;MCT;MGC47838;MT;NET62;fabD	malonyl CoA:ACP acyltransferase (mitochondrial)	0.66	0.64	0.96	0.43
TAB3	TAB3;MAP3K7IP3;MGC45404;NAP1	TGF-beta activated kinase 1/MAP3K7 binding protein 3	0.66	0.67	0.96	0.52
SDR9C7	SDR9C7;FLJ16333;MGC126600;MGC126602;RDHS;SDR-O;SDRO	short chain dehydrogenase/reductase family 9C, member 7	0.66	0.58	0.96	0.47
DHODH	DHODH;DHOdehase;POADS;URA1	dihydroorotate dehydrogenase (quinone)	0.65	0.68	0.96	0.51
ADRBK1	ADRBK1;BARK1;BETA-ARK1;FLJ16718;GRK2	adrenergic, beta, receptor kinase 1	0.65	0.68	0.96	0.48
ZNF160	ZNF160;DKFZp686B16128;F11;FLJ00032;HKr18;HZF5;KIAA1611;KR18	zinc finger protein 160	0.65	0.6	0.96	0.45
C6orf154	C6orf154;FLJ44836;MGC131686;dJ337H4.2	chromosome 6 open reading frame 154	0.64	0.57	0.96	0.48
ADRA1D	ADRA1D;ADRA1;ADRA1A;ADRA1R;ALPHA1;DAR;dJ779E11.2	adrenergic, alpha-1D-, receptor	0.64	0.56	0.96	0.43
ALKBH4	ALKBH4;ABH4;FLJ20013	alkB, alkylation repair homolog 4 (E. coli)	0.64	0.66	0.96	0.51
MRPL27	MRPL27;L27mt;MGC23716	mitochondrial ribosomal protein L27	0.64	0.6	0.96	0.45
TIMM23	TIMM23	ENTREZ STATUS: REMAPPED TO GENE ID: 653252	0.64	0.6	0.96	0.45
SPANXA1	SPANXA1;CT11.1;NAP-X;SPAN-Xa;SPAN-Xb;SPANX;SPANX-A2;SPANX-C;SPANX-D;SPANXA2;SPANXC;SPANXD	sperm protein associated with the nucleus, X-linked, family member A1	0.64	0.62	0.96	0.42
AHCYL2	AHCYL2;ADOHCYASE3;FLJ21719;KIAA0828	adenosylhomocysteinase-like 2	0.63	0.62	0.96	0.48
PSG4	PSG4;PSG9	pregnancy specific beta-1-glycoprotein 4	0.63	0.58	0.96	0.42
WIF1	WIF1;WIF-1	WNT inhibitory factor 1	0.62	0.58	0.96	0.44
GAL3ST2	GAL3ST2;GAL3ST-2;GP3ST	galactose-3-O-sulfotransferase 2	0.62	0.64	0.96	0.48
FBXW4	FBXW4;DAC;FBW4;FBWD4;SHFM3;SHSF3	F-box and WD repeat domain containing 4	0.62	0.57	0.96	0.41
DHX38	DHX38;DDX38;KIAA0224;PRP16;PRPF16	DEAH (Asp-Glu-Ala-His) box polypeptide 38	0.62	0.6	0.96	0.44
AKR1A1	AKR1A1;ALDR1;ALR;ARM;DD3;MGC12529;MGC1380	aldo-keto reductase family 1, member A1 (aldehyde reductase)	0.62	0.6	0.96	0.46
DNAJA3	DNAJA3;FLJ45758;HCA57;TID1;hTID-1	DnaJ (Hsp40) homolog, subfamily A, member 3	0.62	0.62	0.96	0.47
NR5A1	NR5A1;AD4BP;ELP;FTZ1;FTZF1;POF7;SF-1;SF1;SPGF8;SRXY3	nuclear receptor subfamily 5, group A, member 1	0.61	0.63	0.96	0.47
KAT2B	KAT2B;CAF;P;P/CAF;PCAF	K(lysine) acetyltransferase 2B	0.61	0.6	0.96	0.47
TNS3	TNS3;DKFZp686K12123;DKFZp686M1045;FLJ13732;FLJ35545;MGC88434;TEM6;TENS1	tensin 3	0.61	0.54	0.96	0.41
ICAM1	ICAM1;BB2;CD54;P3.58	intercellular adhesion molecule 1	0.61	0.6	0.96	0.47
PTGES3	PTGES3;P23;TEBP;cPGES	prostaglandin E synthase 3 (cytosolic)	0.61	0.54	0.96	0.43
KIF9	KIF9;MGC104186	kinesin family member 9	0.84	0.9	0.97	0.62
CCDC74A	CCDC74A;CCDC74AV2;CCDC74AV3;FLJ40345	coiled-coil domain containing 74A	0.84	0.87	0.97	0.61
CPEB2	CPEB2;CPE-BP2;CPEB-2;MGC119575;MGC119576;MGC119577;hCPEB-2	cytoplasmic polyadenylation element binding protein 2	0.84	0.86	0.97	0.57
PRSS3	PRSS3;MTG;PRSS4;T9;TRY3;TRY4	protease, serine, 3	0.84	0.86	0.97	0.6
B4GALNT4	B4GALNT4;FLJ25045	beta-1,4-N-acetyl-galactosaminyl transferase 4	0.84	0.8	0.97	0.69
MAPK8IP3	MAPK8IP3;DKFZp762N1113;FLJ00027;JIP3;JSAP1;KIAA1066;SYD2;syd	mitogen-activated protein kinase 8 interacting protein 3	0.84	0.78	0.97	0.6
ZNF384	ZNF384;CAGH1;CAGH1A;CIZ;ERDA2;FLJ59043;NMP4;NP;TNRC1	zinc finger protein 384	0.84	0.79	0.97	0.68
TRMT1	TRMT1;FLJ20244;TRM1	TRM1 tRNA methyltransferase 1 homolog (S. cerevisiae)	0.84	0.89	0.97	0.62
ROD1	ROD1;DKFZp781I1117;PTBP3	ROD1 regulator of differentiation 1 (S. pombe)	0.84	0.79	0.97	0.64
HIST1H2AD	HIST1H2AD;H2A.3;H2A/g;H2AFG	histone cluster 1, H2ad	0.84	0.84	0.97	0.63
FLJ37357	FLJ37357	ENTREZ STATUS: REMAPPED TO GENE ID: 1768	0.84	0.85	0.97	0.64
RDH16	RDH16;RODH-4;SDR9C8	retinol dehydrogenase 16 (all-trans)	0.83	0.72	0.97	0.56
RHOB	RHOB;ARH6;ARHB;MST081;MSTP081;RHOH6	ras homolog gene family, member B	0.83	0.76	0.97	0.6
ZBTB25	ZBTB25;C14orf51;KUP;ZNF46	zinc finger and BTB domain containing 25	0.82	0.81	0.97	0.61
COPG2	COPG2;2-COP;DKFZp761N09121;FLJ11781	coatomer protein complex, subunit gamma 2	0.82	0.77	0.97	0.61
COL5A1	COL5A1	collagen, type V, alpha 1	0.82	0.78	0.97	0.65
FLT3LG	FLT3LG;FL;FLT3L	fms-related tyrosine kinase 3 ligand	0.82	0.77	0.97	0.62
DGKZ	DGKZ;DAGK5;DAGK6;DGK-ZETA;hDGKzeta	diacylglycerol kinase, zeta	0.82	0.82	0.97	0.55
METTL4	METTL4;FLJ23017;HsT661;MGC117235	methyltransferase like 4	0.82	0.82	0.97	0.62
IQCB1	IQCB1;NPHP5;PIQ;SLSN5	IQ motif containing B1	0.82	0.82	0.97	0.56
BAK1	BAK1;BAK;BAK-LIKE;BCL2L7;CDN1;MGC117255;MGC3887	BCL2-antagonist/killer 1	0.82	0.77	0.97	0.55
ESCO2	ESCO2;2410004I17Rik;EFO2;RBS	establishment of cohesion 1 homolog 2 (S. cerevisiae)	0.82	0.8	0.97	0.57
CYTH3	CYTH3;ARNO3;GRP1;PSCD3	cytohesin 3	0.82	0.77	0.97	0.58
RAB8A	RAB8A;MEL;RAB8	RAB8A, member RAS oncogene family	0.82	0.8	0.97	0.62
PNN	PNN;DRS;DRSP;SDK3;memA	pinin, desmosome associated protein	0.82	0.72	0.97	0.59
CWC25	CWC25;CCDC49;DKFZp779M0968;FLJ20291	CWC25 spliceosome-associated protein homolog (S. cerevisiae)	0.82	0.83	0.97	0.59
PTPRR	PTPRR;DKFZp781C1038;EC-PTP;FLJ34328;MGC131968;MGC148170;PCPTP1;PTP-SL;PTPBR7;PTPRQ	protein tyrosine phosphatase, receptor type, R	0.81	0.73	0.97	0.55
HIST1H3B	HIST1H3B;H3/l;H3FL	histone cluster 1, H3b	0.81	0.74	0.97	0.57
OR1A1	OR1A1;OR17-7	olfactory receptor, family 1, subfamily A, member 1	0.8	0.73	0.97	0.55
C20orf107	C20orf107;MGC104273;dJ1153D9.4	chromosome 20 open reading frame 107	0.8	0.75	0.97	0.56
PRUNE2	PRUNE2;A214N16.3;BMCC1;BNIPXL;C9orf65;DKFZp762K117;FLJ50060;FLJ54876;FLJ59118;KIAA0367;RP11-58J3.2;bA214N16.3	prune homolog 2 (Drosophila)	0.8	0.8	0.97	0.6
RAD54L2	RAD54L2;ARIP4;FLJ21396;FLJ22400;HSPC325;KIAA0809;SRISNF2L	RAD54-like 2 (S. cerevisiae)	0.8	0.75	0.97	0.65
UNC80	UNC80;C2orf21;FLJ14677;FLJ33496;FLJ53903;KIAA1843;UNC-80	unc-80 homolog (C. elegans)	0.8	0.8	0.97	0.54
CELF3	CELF3;BRUNOL1;CAGH4;ERDA4;MGC57297;TNRC4	CUGBP, Elav-like family member 3	0.8	0.74	0.97	0.63
GTF2A1L	GTF2A1L;ALF;MGC26254	general transcription factor IIA, 1-like	0.8	0.78	0.97	0.52
REG1A	REG1A;ICRF;MGC12447;P19;PSP;PSPS;PSPS1;PTP;REG	regenerating islet-derived 1 alpha	0.79	0.74	0.97	0.56
ZNF385C	ZNF385C	zinc finger protein 385C	0.79	0.77	0.97	0.56
ZNF142	ZNF142;pHZ-49	zinc finger protein 142	0.79	0.8	0.97	0.6
FXR2	FXR2;FMR1L2;FXR2P	fragile X mental retardation, autosomal homolog 2	0.79	0.78	0.97	0.54
C19orf48	C19orf48;MGC13170	chromosome 19 open reading frame 48	0.79	0.82	0.97	0.63
IARS	IARS;FLJ20736;IARS1;ILERS;ILRS;IRS;PRO0785	isoleucyl-tRNA synthetase	0.79	0.77	0.97	0.62
DIMT1	DIMT1;DIM1;DIMT1L;HSA9761;HUSSY5	DIM1 dimethyladenosine transferase 1 homolog (S. cerevisiae)	0.79	0.76	0.97	0.57
LINC00478	LINC00478;C21orf34;C21orf35;FLJ38295	long intergenic non-protein coding RNA 478	0.79	0.84	0.97	0.55
AADACL4	AADACL4	arylacetamide deacetylase-like 4	0.78	0.74	0.97	0.56
CCDC68	CCDC68;FLJ25368;SE57-1	coiled-coil domain containing 68	0.78	0.72	0.97	0.58

ANGPTL2	ANGPTL2;ARP2;HARP;MGC8889	angiopoietin-like 2	0.78	0.76	0.97	0.51
FAM181A	FAM181A;C14orf152	family with sequence similarity 181, member A	0.78	0.68	0.97	0.57
EXOC3	EXOC3;SEC6;SEC6L1;Sec6p	exocyst complex component 3	0.78	0.78	0.97	0.56
C11orf46	C11orf46;FLJ38968;dJ299F11.1	chromosome 11 open reading frame 46	0.78	0.68	0.97	0.53
RNF40	RNF40;BRE1B;DKFZp686K191;KIAA0661;MGC13051;RBP95;STARING	ring finger protein 40	0.78	0.75	0.97	0.67
SELK	SELK;HSPC297;MGC17057	selenoprotein K	0.78	0.68	0.97	0.57
SNURF	SNURF	SNRPN upstream reading frame	0.78	0.73	0.97	0.56
EIF2D	EIF2D;HCA56;LGTN	eukaryotic translation initiation factor 2D	0.78	0.8	0.97	0.64
NXF3	NXF3	nuclear RNA export factor 3	0.77	0.76	0.97	0.61
PLB1	PLB1;PLB;PLB/LIP;hPLB	phospholipase B1	0.77	0.74	0.97	0.55
PKP1	PKP1;B6P;MGC138829	plakophilin 1 (ectodermal dysplasia/skin fragility syndrome)	0.77	0.77	0.97	0.53
ZNF764	ZNF764;MGC13138	zinc finger protein 764	0.77	0.76	0.97	0.57
SLC25A11	SLC25A11;OGC;SLC20A4	solute carrier family 25 (mitochondrial carrier; oxoglutarate carrier), member 11	0.77	0.69	0.97	0.61
LRRC6	LRRC6;LRTP;TSLRP	leucine rich repeat containing 6	0.76	0.76	0.97	0.55
F8	F8;AHF;DXS1253E;F8B;F8C;FVIII;HEMA	coagulation factor VIII, procoagulant component	0.76	0.8	0.97	0.6
NEFH	NEFH;NFH	neurofilament, heavy polypeptide	0.76	0.78	0.97	0.62
IQSEC2	IQSEC2;BRAG1;KIAA0522;MRX1	IQ motif and Sec7 domain 2	0.76	0.8	0.97	0.53
TMEM121	TMEM121;MGC4659;hole	transmembrane protein 121	0.76	0.7	0.97	0.56
TARBP2	TARBP2;LOQS;TRBP;TRBP1;TRBP2	TAR (HIV-1) RNA binding protein 2	0.76	0.78	0.97	0.55
TMEM30B	TMEM30B;CDC50B;MGC126775	transmembrane protein 30B	0.76	0.73	0.97	0.51
STYXL1	STYXL1;DUSP24;MK-STYX	serine/threonine/tyrosine interacting-like 1	0.76	0.68	0.97	0.52
RNF111	RNF111;ARK;DKFZp313E0731;DKFZp686H1966;DKFZp761D081;FLJ38008	ring finger protein 111	0.76	0.73	0.97	0.65
KLHL20	KLHL20;KHLHX;KLEIP;KLHLX;RP3-383J4.3	kelch-like 20 (Drosophila)	0.76	0.8	0.97	0.61
RAB9A	RAB9A;RAB9	RAB9A, member RAS oncogene family	0.76	0.72	0.97	0.62
HARS2	HARS2;HARSL;HARSR;HO3	histidyl-tRNA synthetase 2, mitochondrial (putative)	0.76	0.73	0.97	0.63
SH3BGRL2	SH3BGRL2;FLJ90025	SH3 domain binding glutamic acid-rich protein like 2	0.76	0.76	0.97	0.58
PCBP2	PCBP2;HNRPE2;MGC110998;hnRNP-E2	poly(rC) binding protein 2	0.76	0.72	0.97	0.54
MRI1	MRI1;MGC3207;MTNA;Ypr118w	methylthioribose-1-phosphate isomerase homolog (S. cerevisiae)	0.76	0.8	0.97	0.6
SMARCC1	SMARCC1;BAF155;CRACC1;Rsc8;SRG3;SWI3	SWI/SNF related, matrix associated, actin dependent regulator of chromatin, subfamily c, member 1	0.75	0.7	0.97	0.61
KRT31	KRT31;HA1;Ha-1;KRTHA1;MGC138630;hHa1	keratin 31	0.74	0.73	0.97	0.52
AP4S1	AP4S1;AP47B;CLA20;CLAPS4;CPSQ6;FLJ32366	adaptor-related protein complex 4, sigma 1 subunit	0.74	0.74	0.97	0.56
ADAMTS14	ADAMTS14;FLJ32820	ADAM metallopeptidase with thrombospondin type 1 motif, 14	0.74	0.7	0.97	0.53
CCDC61	CCDC61	coiled-coil domain containing 61	0.74	0.68	0.97	0.5
PRKAB2	PRKAB2;MGC61468	protein kinase, AMP-activated, beta 2 non-catalytic subunit	0.74	0.78	0.97	0.56
CLSTN1	CLSTN1;ALC-ALPHA;CDHR12;CSTN1;FLJ32258;KIAA0911;PIK3CD;XB31alpha;alcalpha1;alcalpha2	calsyntenin 1	0.74	0.74	0.97	0.58
S1PR4	S1PR4;EDG6;LPC1;S1P4;SLP4	sphingosine-1-phosphate receptor 4	0.73	0.66	0.97	0.5
ERC2	ERC2;CAST;CAST1;ELKSL;KIAA0378;MGC133063;MGC133064;SPBC110;Spc110	ELKS/RAB6-interacting/CAST family member 2	0.73	0.74	0.97	0.54
MAN1A1	MAN1A1;HUMM3;HUMM9;MAN9	mannosidase, alpha, class 1A, member 1	0.73	0.78	0.97	0.54
FAM178B	FAM178B	family with sequence similarity 178, member B	0.73	0.73	0.97	0.55
NKX2-5	NKX2-5;CHNG5;CSX;CSX1;FLJ52202;FLJ97166;FLJ97195;FLJ97197;FLJ99536;NKX2.5;NKX2E;NKX4-1	NK2 homeobox 5	0.73	0.7	0.97	0.52
YIPF3	YIPF3;C6orf109;DKFZp566C243;FinGER3;KLIP1;dJ337H4.3	Yip1 domain family, member 3	0.73	0.76	0.97	0.51
ANKRD55	ANKRD55;FLJ11795;MGC126013;MGC126014	ankyrin repeat domain 55	0.72	0.74	0.97	0.53
OR51B5	OR51B5;HOR5'Beta5;OR11-37	olfactory receptor, family 51, subfamily B, member 5	0.72	0.74	0.97	0.55
C6orf25	C6orf25;G6b;MGC142279;MGC142281;NG31	chromosome 6 open reading frame 25	0.72	0.72	0.97	0.55
PCOLCE	PCOLCE;PCPE;PCPE-1;PCPE1	procollagen C-endopeptidase enhancer	0.72	0.72	0.97	0.59
RHOU	RHOU;ARHU;CDC42L1;DJ646B12.2;FLJ10616;WRCH1;fJ646B12.2;hG28K	ras homolog gene family, member U	0.72	0.67	0.97	0.49
HDAC10	HDAC10;DKFZp761B039;HD10;MGC149722	histone deacetylase 10	0.72	0.7	0.97	0.55
ZNF187	ZNF187;MGC2815;SRE-ZBP;ZSCAN26	zinc finger protein 187	0.72	0.74	0.97	0.52
BLM	BLM;BS;MGC126616;MGC131618;MGC131620;RECQ2;RECQL2;RECQL3	Bloom syndrome, RecQ helicase-like	0.72	0.66	0.97	0.48
CHN2	CHN2;ARHGAP3;BCH;CHN2-3;MGC138360;RHOGAP3	chimerin (chimaerin) 2	0.72	0.74	0.97	0.52
SRCRB4D	SRCRB4D;S4D-SRCRB;SRCRB-S4D;SSC4D	scavenger receptor cysteine rich domain containing, group B (4 domains)	0.72	0.68	0.97	0.53
PIAS4	PIAS4;FLJ12419;MGC35296;PIASY;Piasg;ZMIZ6	protein inhibitor of activated STAT, 4	0.72	0.68	0.97	0.49
KIAA0430	KIAA0430;LKAP;PPP1R34	KIAA0430	0.72	0.76	0.97	0.51
AVEN	AVEN;PDCD12	apoptosis, caspase activation inhibitor	0.72	0.74	0.97	0.57
F8A1	F8A1;DXS522E;F8A;HAP40	coagulation factor VIII-associated 1	0.72	0.77	0.97	0.51
LARP1B	LARP1B;DKFZp686L13217;LARP2;MGC117277;MGC75174	La ribonucleoprotein domain family, member 1B	0.71	0.69	0.97	0.5
ANKRD56	ANKRD56	ankyrin repeat domain 56	0.7	0.74	0.97	0.51
S100A1	S100A1;S100;S100-alpha;S100A	S100 calcium binding protein A1	0.7	0.7	0.97	0.53
T	T;MGC104817;TFT	T, brachyury homolog (mouse)	0.7	0.68	0.97	0.46
FAM166A	FAM166A;FLJ40100	family with sequence similarity 166, member A	0.7	0.66	0.97	0.5
PRELP	PRELP;MGC45323;MST161;MSTP161;SLRR2A	proline/arginine-rich end leucine-rich repeat protein	0.7	0.66	0.97	0.52
EIF5A	EIF5A;EIF-5A;EIF5A1;MGC104255;MGC99547;eIF5AI	eukaryotic translation initiation factor 5A	0.7	0.65	0.97	0.51
TAF6L	TAF6L;FLJ11136;MGC4288;PAF65A	TAF6-like RNA polymerase II, p300/CBP-associated factor (PCAF)-associated factor, 65kDa	0.7	0.67	0.97	0.5
HECTD3	HECTD3;FLJ21156;FLJ31983;MGC161630;RP11-69J16.1	HECT domain containing 3	0.7	0.62	0.97	0.47
C8orf84	C8orf84;FLJ40021;RPESP	chromosome 8 open reading frame 84	0.7	0.65	0.97	0.5
LY6G6D	LY6G6D;C6orf23;G6D;LY6-D;MEGT1;MGC150334;NG25	lymphocyte antigen 6 complex, locus G6D	0.69	0.68	0.97	0.49
KPNA1	KPNA1;IPOA5;NPI-1;RCH2;SRP1	karyopherin alpha 1 (importin alpha 5)	0.69	0.65	0.97	0.46
RSL24D1	RSL24D1;C15orf15;HRP-L30-iso;L30;RLP24;RPL24;RPL24L;TVAS3	ribosomal L24 domain containing 1	0.69	0.6	0.97	0.5
FRMD7	FRMD7;FLJ43346;NYS;NYS1	FERM domain containing 7	0.68	0.72	0.97	0.48
NPHP1	NPHP1;FLJ97602;JBTS4;NPH1;SLSN1	nephronophthisis 1 (juvenile)	0.68	0.66	0.97	0.46
MDM4	MDM4;DKFZp781B1423;HDMX;MDMX;MGC132766;MRP1	Mdm4 p53 binding protein homolog (mouse)	0.68	0.65	0.97	0.59
TBC1D10B	TBC1D10B;DKFZp434P1750	TBC1 domain family, member 10B	0.68	0.6	0.97	0.5
EXOC8	EXOC8;EXO84;Exo84p;SEC84	exocyst complex component 8	0.68	0.68	0.97	0.52
RRS1	RRS1;KIAA0112	RRS1 ribosome biogenesis regulator homolog (S. cerevisiae)	0.68	0.66	0.97	0.53
TAP1	TAP1;ABC17;ABCB2;APT1;D6S114E;FLJ26666;FLJ41500;PSF-1;PSF1;RING4;TAP1*0102N;TAP1N	transporter 1, ATP-binding cassette, sub-family B (MDR/TAP)	0.68	0.7	0.97	0.46
ZC3H15	ZC3H15;HT010;LEREPO4;MSTP012	zinc finger CCCH-type containing 15	0.68	0.68	0.97	0.5
KRTAP1-5	KRTAP1-5;KAP1.5;KRTAP1.5;MGC126604	keratin associated protein 1-5	0.67	0.68	0.97	0.55
CCL7	CCL7;FIC;MARC;MCP-3;MCP3;MGC138463;MGC138465;NC28;SCYA6;SCYA7	chemokine (C-C motif) ligand 7	0.66	0.65	0.97	0.52
LCA5	LCA5;C6orf152	Leber congenital amaurosis 5	0.66	0.66	0.97	0.5
LLGL2	LLGL2;HGL;LGL2	lethal giant larvae homolog 2 (Drosophila)	0.66	0.63	0.97	0.53
C16orf74	C16orf74;MGC17624	chromosome 16 open reading frame 74	0.66	0.66	0.97	0.47
DHX33	DHX33;DDX33;DKFZp762F2011;FLJ21972	DEAH (Asp-Glu-Ala-His) box polypeptide 33	0.66	0.63	0.97	0.49
WDR89	WDR89;C14orf150;MGC9907	WD repeat domain 89	0.66	0.64	0.97	0.46
NR1D2	NR1D2;BD73;EAR-1R;RVR	nuclear receptor subfamily 1, group D, member 2	0.66	0.7	0.97	0.52
PPP2R3A	PPP2R3A;PPP2R3;PR130;PR72	protein phosphatase 2, regulatory subunit B'', alpha	0.66	0.6	0.97	0.45
STYX	STYX;FLJ42934	serine/threonine/tyrosine interacting protein	0.66	0.64	0.97	0.5
RDH11	RDH11;ARSDR1;CGI82;FLJ32633;HCBP12;MDT1;PSDR1;RALR1;SCALD;SDR7C1	retinol dehydrogenase 11 (all-trans/9-cis/11-cis)	0.66	0.63	0.97	0.46
HSD17B10	HSD17B10;17b-HSD10;ABAD;CAMR;DUPXp11.22;ERAB;HADH2;HCD2;MHBD;MRPP2;MRX17;MRX31;MRXS10;SCHAD;SDR5C1	hydroxysteroid (17-beta) dehydrogenase 10	0.66	0.62	0.97	0.46
CYB5B	CYB5B;CYB5-M;CYPB5M;DKFZp686M0619;OMB5	cytochrome b5 type B (outer mitochondrial membrane)	0.66	0.57	0.97	0.45
ATP5B	ATP5B;ATPMB;ATPSB;MGC5231	ATP synthase, H+ transporting, mitochondrial F1 complex, beta polypeptide	0.66	0.64	0.97	0.5
CWH43	CWH43;CWH43-C;FLJ21511	cell wall biogenesis 43 C-terminal homolog (S. cerevisiae)	0.66	0.66	0.97	0.44
OPN1SW	OPN1SW;BCP;BOP;CBT	opsin 1 (cone pigments), short-wave-sensitive	0.65	0.58	0.97	0.44
ZNF37A	ZNF37A;FLJ3472;KOX21;ZNF37	zinc finger protein 37A	0.65	0.66	0.97	0.44
C9orf64	C9orf64;MGC10999	chromosome 9 open reading frame 64	0.65	0.65	0.97	0.49
ANGPT4	ANGPT4;AGP4;ANG-3;ANG4;MGC138181;MGC138183	angiopoietin 4	0.64	0.65	0.97	0.49
KNDC1	KNDC1;C10orf23;FLJ16067;RASGEF2;bB439H18.3	kinase non-catalytic C-lobe domain (KIND) containing 1	0.64	0.68	0.97	0.5
SLURP1	SLURP1;ANUP;ARS;ArsB;LY6LS;MDM	secreted LY6/PLAUR domain containing 1	0.64	0.61	0.97	0.46
SYT5	SYT5	synaptotagmin V	0.64	0.66	0.97	0.53
UPK2	UPK2;MGC138598;UP2;UPII	uroplakin 2	0.64	0.64	0.97	0.43
KRT18	KRT18;CYK18;K18	keratin 18	0.64	0.68	0.97	0.5
LHFP	LHFP;MGC22429	lipoma HMGIC fusion partner	0.63	0.63	0.97	0.43
ATP4A	ATP4A;ATP6A	ATPase, H+/K+ exchanging, alpha polypeptide	0.62	0.59	0.97	0.46
KCNT1	KCNT1;FLJ41282;KCa4.1;SLACK;bA100C15.2	potassium channel, subfamily T, member 1	0.62	0.62	0.97	0.48
OLIG1	OLIG1;BHLHB6;BHLHE21	oligodendrocyte transcription factor 1	0.62	0.6	0.97	0.5
KLC3	KLC3;KLC2;KLC2L;KLCt;KNS2B	kinesin light chain 3	0.62	0.55	0.97	0.49
ATP13A2	ATP13A2;FLJ26510;HSA9947;KRPPD;PARK9	ATPase type 13A2	0.62	0.58	0.97	0.51
ASPH	ASPH;AAH;BAH;CASQ2BP1;HAAH;JCTN;junctin	aspartate beta-hydroxylase	0.62	0.58	0.97	0.41
PTCD3	PTCD3;DKFZp666K071;FLJ20758	Pentatricopeptide repeat domain 3	0.62	0.6	0.97	0.43
EIF2B1	EIF2B1;EIF2B;EIF2BA;MGC117409;MGC125868;MGC125869	eukaryotic translation initiation factor 2B, subunit 1 alpha, 26kDa	0.62	0.57	0.97	0.45
PCDHA1	PCDHA1;PCDH-ALPHA1	protocadherin alpha 1	0.62	0.6	0.97	0.42
HAVCR2	HAVCR2;FLJ14428;HAVcr-2;KIM-3;TIM3;TIMD-3;TIMD3;Tim-3	hepatitis A virus cellular receptor 2	0.61	0.64	0.97	0.41
MAB21L2	MAB21L2;FLJ31103	mab-21-like 2 (C. elegans)	0.61	0.6	0.97	0.41
TMEM31	TMEM31;FLJ27357;MGC39655	transmembrane protein 31	0.84	0.86	0.98	0.69
MCEE	MCEE;GLOD2	methylmalonyl CoA epimerase	0.84	0.77	0.98	0.61
ACVRL1	ACVRL1;ACVRLK1;ALK-1;ALK1;HHT;HHT2;ORW2;SKR3;TSR-I	activin A receptor type II-like 1	0.84	0.82	0.98	0.68
RNF165	RNF165;ARKL2	ring finger protein 165	0.84	0.81	0.98	0.72
COMMD5	COMMD5;FLJ13008;HCARG;MGC72046	COMM domain containing 5	0.84	0.82	0.98	0.68
HIVEP2	HIVEP2;HIV-EP2;MBP-2;MIBP1;SHN2;ZAS2;ZNF40B	human immunodeficiency virus type I enhancer binding protein 2	0.84	0.8	0.98	0.59
MON1B	MON1B;HSRG1;SAND2;SRG1	MON1 homolog B (yeast)	0.84	0.82	0.98	0.59
SDCCAG8	SDCCAG8;BBS16;CCCAP;NPHP10;NY-CO-8;SLSN7;hCCCAP	serologically defined colon cancer antigen 8	0.84	0.86	0.98	0.61
DGKH	DGKH;DGKeta;DKFZp761I1510	diacylglycerol kinase, eta	0.84	0.9	0.98	0.63
SGIP1	SGIP1;DKFZp686A16142;DKFZp761D221;FLJ33378;FLJ43054	SH3-domain GRB2-like (endophilin) interacting protein 1	0.84	0.82	0.98	0.59
UBAC1	UBAC1;GBDR1;RP11-432J22.3;UBADC1	UBA domain containing 1	0.84	0.82	0.98	0.6
WARS	WARS;GAMMA-2;IFI53;IFP53	tryptophanyl-tRNA synthetase	0.84	0.82	0.98	0.61
PCDHGB3	PCDHGB3;PCDH-GAMMA-B3	protocadherin gamma subfamily B, 3	0.84	0.79	0.98	0.62
ORC6	ORC6;ORC6L	origin recognition complex, subunit 6	0.84	0.8	0.98	0.57
PAPL	PAPL;FLJ16165	iron/zinc purple acid phosphatase-like protein	0.84	0.88	0.98	0.58
GH2	GH2;GH-V;GHL;GHV;hGH-V	growth hormone 2	0.83	0.84	0.98	0.64
HPN	HPN;TMPRSS1	hepsin	0.83	0.75	0.98	0.56

SOST	SOST;VBCH	sclerostin	0.83	0.86	0.98	0.57
CTDSPL	CTDSPL;C3orf8;HYA22;PSR1;RBSP3;SCP3	CTD (carboxy-terminal domain, RNA polymerase II, polypeptide A) small phosphatase-like	0.83	0.83	0.98	0.6
CISD2	CISD2;ERIS;Miner1;NAF-1;WFS2;ZCD2	CDGSH iron sulfur domain 2	0.83	0.8	0.98	0.6
PAPOLB	PAPOLB;PAPT;TPAP	poly(A) polymerase beta (testis specific)	0.82	0.82	0.98	0.65
SAG	SAG;DKFZp686D1084;DKFZp686I1383;RP47;S-AG	S-antigen; retina and pineal gland (arrestin)	0.82	0.82	0.98	0.57
CNGA4	CNGA4;CNCA2;CNG-4;CNG4;CNG5;CNGB2;MGC126168;MGC126169;OCNC2;OCNCBETA;OCNCb	cyclic nucleotide gated channel alpha 4	0.82	0.85	0.98	0.66
ADPRHL1	ADPRHL1;ARH2	ADP-ribosylhydrolase like 1	0.82	0.79	0.98	0.61
C4A	C4A;C4;C4A2;C4A3;C4A4;C4A6;C4S;CO4;CPAMD2;MGC164979;RG	complement component 4A (Rodgers blood group)	0.82	0.83	0.98	0.56
DNASE1	DNASE1;DKFZp686H0155;DNL1;DRNI;FLJ38093;FLJ44902	deoxyribonuclease I	0.82	0.78	0.98	0.58
SIGLEC5	SIGLEC5;CD170;CD33L2;OB-BP2;OBBP2;SIGLEC-5	sialic acid binding Ig-like lectin 5	0.82	0.76	0.98	0.64
ZNF354C	ZNF354C;KID3	zinc finger protein 354C	0.82	0.84	0.98	0.62
IGSF3	IGSF3;EWI-3;MGC117164;V8	immunoglobulin superfamily, member 3	0.82	0.8	0.98	0.55
PHTF1	PHTF1;PHTF	putative homeodomain transcription factor 1	0.82	0.76	0.98	0.6
NSUN6	NSUN6;4933414E04Rik;FLJ23743;NOPD1	NOP2/Sun domain family, member 6	0.82	0.86	0.98	0.57
DGKA	DGKA;DAGK;DAGK1;DGK-alpha;MGC12821;MGC42356	diacylglycerol kinase, alpha 80kDa	0.82	0.8	0.98	0.66
CLPTM1L	CLPTM1L;CRR9;DKFZp666M1010;DKFZp761M2324;FLJ14400;FLJ32533	CLPTM1-like	0.82	0.82	0.98	0.61
PTMS	PTMS;ParaT	parathymosin	0.82	0.85	0.98	0.6
PPP1R12C	PPP1R12C;DKFZp434D0412;LENG3;MBS85;p84;p85	protein phosphatase 1, regulatory subunit 12C	0.82	0.8	0.98	0.63
MRAP2	MRAP2;C6orf117;RP11-51G5.2;bA51G5.2	melanocortin 2 receptor accessory protein 2	0.81	0.77	0.98	0.56
LTK	LTK;TYK1	leukocyte receptor tyrosine kinase	0.81	0.87	0.98	0.61
ZNF517	ZNF517	zinc finger protein 517	0.81	0.71	0.98	0.57
SREBF1	SREBF1;SREBP-1c;SREBP1;bHLHd1	sterol regulatory element binding transcription factor 1	0.81	0.8	0.98	0.54
MAGEA2B	MAGEA2B;MAGE2;MAGEA2;MGC16973	melanoma antigen family A, 2B	0.81	0.82	0.98	0.59
CELF6	CELF6;BRUNOL6	CUGBP, Elav-like family member 6	0.81	0.72	0.98	0.58
CRX	CRX;CORD2;CRD;LCA7;OTX3	cone-rod homeobox	0.8	0.77	0.98	0.6
C14orf45	C14orf45;FLJ23189	chromosome 14 open reading frame 45	0.8	0.8	0.98	0.58
MSC	MSC;ABF-1;ABF1;MYOR;bHLHa22	musculin	0.8	0.83	0.98	0.55
RELN	RELN;LIS2;PRO1598;RL	reelin	0.8	0.77	0.98	0.58
ARL6	ARL6;BBS3;MGC32934;RP55	ADP-ribosylation factor-like 6	0.8	0.83	0.98	0.58
NAGLU	NAGLU;MPS-IIIB;MPS3B;NAG;UFHSD	N-acetylglucosaminidase, alpha	0.8	0.83	0.98	0.55
KIAA2026	KIAA2026	KIAA2026	0.8	0.78	0.98	0.55
REEP4	REEP4;C8orf20;FLJ22246;FLJ22277	receptor accessory protein 4	0.8	0.82	0.98	0.59
MICB	MICB;PERB11.2	MHC class I polypeptide-related sequence B	0.8	0.8	0.98	0.63
UCP2	UCP2;BMIQ4;SLC25A8;UCPH	uncoupling protein 2 (mitochondrial, proton carrier)	0.8	0.86	0.98	0.6
DRAM2	DRAM2;MGC54289;PRO180;RP5-1180E21.1;TMEM77;WWFQ154	DNA-damage regulated autophagy modulator 2	0.8	0.7	0.98	0.55
FLJ35709	FLJ35709	ENTREZ STATUS: REMAPPED TO GENE ID: 144132	0.8	0.77	0.98	0.58
CELF2	CELF2;BRUNOL3;CUGBP2;ETR-3;ETR3;NAPOR	CUGBP, Elav-like family member 2	0.8	0.8	0.98	0.61
TUSC1	TUSC1;MGC131751;TSG-9;TSG9	tumor suppressor candidate 1	0.79	0.83	0.98	0.58
TFB2M	TFB2M;FLJ22661;FLJ23182;Hkp1;mtTFB2	transcription factor B2, mitochondrial	0.79	0.86	0.98	0.59
MZT2A	MZT2A;FAM128A;MOZART2A	mitotic spindle organizing protein 2A	0.79	0.69	0.98	0.58
MAGEE2	MAGEE2;HCA3;MGC138154	melanoma antigen family E, 2	0.78	0.76	0.98	0.52
FLJ33360	FLJ33360	FLJ33360 protein	0.78	0.78	0.98	0.59
KBTBD3	KBTBD3;BKLHD3;FLJ30685	kelch repeat and BTB (POZ) domain containing 3	0.78	0.8	0.98	0.6
OR13J1	OR13J1;OR9-2	olfactory receptor, family 13, subfamily J, member 1	0.78	0.74	0.98	0.56
CRYGC	CRYGC;CCL;CRYG3	crystallin, gamma C	0.78	0.78	0.98	0.59
NXPH2	NXPH2;MGC125477;MGC125478;MGC125479;MGC125480;NPH2	neurexophilin 2	0.78	0.74	0.98	0.56
LHX1	LHX1;LIM-1;LIM1;MGC126723;MGC138141	LIM homeobox 1	0.78	0.74	0.98	0.64
NDUFB3	NDUFB3;B12	NADH dehydrogenase (ubiquinone) 1 beta subcomplex, 3, 12kDa	0.78	0.72	0.98	0.61
UTF1	UTF1	undifferentiated embryonic cell transcription factor 1	0.78	0.74	0.98	0.53
METRN	METRN;C16orf23;MGC2601;c380A1.2	meteorin, glial cell differentiation regulator	0.78	0.78	0.98	0.53
CD68	CD68;DKFZp686M18236;GP110;SCARD1	CD68 molecule	0.78	0.8	0.98	0.65
C2orf49	C2orf49;FLJ45759;MGC5509;asw	chromosome 2 open reading frame 49	0.78	0.8	0.98	0.58
SPC24	SPC24;FLJ90806;SPBC24	SPC24, NDC80 kinetochore complex component, homolog (S. cerevisiae)	0.78	0.8	0.98	0.57
CDK8	CDK8;K35;MGC126074;MGC126075	cyclin-dependent kinase 8	0.78	0.76	0.98	0.63
SMURF1	SMURF1;KIAA1625	SMAD specific E3 ubiquitin protein ligase 1	0.78	0.71	0.98	0.53
DRAP1	DRAP1;NC2-alpha	DR1-associated protein 1 (negative cofactor 2 alpha)	0.78	0.8	0.98	0.53
SOX11	SOX11	SRY (sex determining region Y)-box 11	0.77	0.68	0.98	0.56
RPL22	RPL22;EAP;HBP15;HBP15/L22;L22	ribosomal protein L22	0.77	0.74	0.98	0.62
ULK4	ULK4;DKFZp434E1822;FAM7C1;FLJ20574;REC01035	unc-51-like kinase 4 (C. elegans)	0.77	0.82	0.98	0.58
ZNF443	ZNF443;ZK1	zinc finger protein 443	0.76	0.78	0.98	0.59
WNT8A	WNT8A;WNT8D	wingless-type MMTV integration site family, member 8A	0.76	0.75	0.98	0.52
CCDC92	CCDC92;FLJ22471	coiled-coil domain containing 92	0.76	0.7	0.98	0.57
IGF1R	IGF1R;CD221;IGFIR;IGFR;JTK13;MGC142170;MGC142172;MGC18216	insulin-like growth factor 1 receptor	0.76	0.74	0.98	0.62
STARD3	STARD3;CAB1;FLJ41370;MLN64;es64	StAR-related lipid transfer (START) domain containing 3	0.76	0.72	0.98	0.52
FLJ39779	FLJ39779	ENTREZ STATUS: DISCONTINUED	0.76	0.77	0.98	0.55
ARHGAP8	ARHGAP8;BPGAP1;FLJ20185;PP610	Rho GTPase activating protein 8	0.76	0.69	0.98	0.55
IPCEF1	IPCEF1;KIAA0403;PIP3-E;RP3-402L9.2	interaction protein for cytohesin exchange factors 1	0.76	0.73	0.98	0.55
NAA38	NAA38;FLJ33242;LSM8	N(alpha)-acetyltransferase 38, NatC auxiliary subunit	0.76	0.78	0.98	0.56
NEK11	NEK11;FLJ23495	NIMA (never in mitosis gene a)- related kinase 11	0.75	0.73	0.98	0.6
HAPLN2	HAPLN2;BRAL1	hyaluronan and proteoglycan link protein 2	0.75	0.72	0.98	0.54
MN1	MN1;MGCR;MGCR1;MGCR1-PEN;dJ353E16.2	meningioma (disrupted in balanced translocation) 1	0.75	0.71	0.98	0.54
LYPD2	LYPD2;LYPDC2;MGC148106;UNQ430	LY6/PLAUR domain containing 2	0.75	0.76	0.98	0.58
C14orf1	C14orf1;ERG28;NET51	chromosome 14 open reading frame 1	0.75	0.72	0.98	0.54
OR2T2	OR2T2;OR1-43;OR2T2P	olfactory receptor, family 2, subfamily T, member 2	0.74	0.8	0.98	0.58
ZNF215	ZNF215;BAZ-2;BAZ2;ZKSCAN11	zinc finger protein 215	0.74	0.74	0.98	0.56
PABPC1L2A	PABPC1L2A;MGC168104;PABPC1L2B;RBM32A	poly(A) binding protein, cytoplasmic 1-like 2A	0.74	0.78	0.98	0.55
ZNF628	ZNF628;MGC105139;ZEC;Zfp628	zinc finger protein 628	0.74	0.76	0.98	0.56
MBNL3	MBNL3;CHCR;FLJ11316;FLJ97142;MBLX;MBLX39;MBXL	muscleblind-like 3 (Drosophila)	0.74	0.75	0.98	0.61
GJD3	GJD3;CX31.9;Cx30.2;GJA11;GJC1	gap junction protein, delta 3, 31.9kDa	0.74	0.65	0.98	0.52
ANAPC11	ANAPC11;APC11;Apc11p;MGC882	anaphase promoting complex subunit 11	0.74	0.7	0.98	0.63
MYD88	MYD88;MYD88D	myeloid differentiation primary response gene (88)	0.74	0.76	0.98	0.5
ZRANB1	ZRANB1;DKFZp762P2216;TRABID	zinc finger, RAN-binding domain containing 1	0.74	0.7	0.98	0.5
YWHAZ	YWHAZ;14-3-3-zeta;KCIP-1;MGC111427;MGC126532;MGC138156;YWHAD	tyrosine 3-monooxygenase/tryptophan 5-monooxygenase activation protein, zeta polypeptide	0.74	0.73	0.98	0.54
TRPM3	TRPM3;GON-2;LTRPC3;MLSN2	transient receptor potential cation channel, subfamily M, member 3	0.73	0.72	0.98	0.56
ODF4	ODF4;CT134;CT136;MGC138215;MGC138219;OPPO1	outer dense fiber of sperm tails 4	0.72	0.69	0.98	0.54
KLHL31	KLHL31;BKLHD6;KBTBD1;KLHL;bA345L23.2	kelch-like 31 (Drosophila)	0.72	0.7	0.98	0.51
OR2S2	OR2S2;OR37A;OST715	olfactory receptor, family 2, subfamily S, member 2	0.72	0.74	0.98	0.5
DPF1	DPF1;BAF45b;MGC150428;MGC150429;NEUD4;neuro-d4	D4, zinc and double PHD fingers family 1	0.72	0.7	0.98	0.63
CEACAM16	CEACAM16;CEAL2	carcinoembryonic antigen-related cell adhesion molecule 16	0.72	0.72	0.98	0.54
LRRC45	LRRC45;MGC20806	leucine rich repeat containing 45	0.72	0.64	0.98	0.5
HOXA7	HOXA7;ANTP;HOX1;HOX1.1;HOX1A	homeobox A7	0.72	0.67	0.98	0.52
HBQ1	HBQ1	hemoglobin, theta 1	0.72	0.7	0.98	0.58
ANGEL1	ANGEL1;FLJ60172;KIAA0759	angel homolog 1 (Drosophila)	0.72	0.7	0.98	0.5
CERK	CERK;DKFZp434E0211;FLJ21430;FLJ23239;KIAA1646;LK4;MGC131878;dA59H18.2;dA59H18.3;hCERK	ceramide kinase	0.72	0.76	0.98	0.54
COX7C	COX7C	cytochrome c oxidase subunit VIIc	0.72	0.73	0.98	0.57
TTC39C	TTC39C;C18orf17;FLJ33761;FLJ90394;HsT2697	tetratricopeptide repeat domain 39C	0.72	0.68	0.98	0.51
NME1-NME2	NME1-NME2;FLJ93990;NM23-LV;NMELV	NME1-NME2 readthrough	0.72	0.74	0.98	0.52
SEL1L3	SEL1L3;DKFZp781J1697;FLJ21629;FLJ41299;KIAA0746;Sel-1L3	sel-1 suppressor of lin-12-like 3 (C. elegans)	0.72	0.68	0.98	0.51
ZNF750	ZNF750;FLJ13841;FLJ51362;MGC125667;MGC125668;ZFP750	zinc finger protein 750	0.7	0.66	0.98	0.5
DIRAS2	DIRAS2;DKFZp761C07121;Di-Ras2	DIRAS family, GTP-binding RAS-like 2	0.7	0.65	0.98	0.47
DHX58	DHX58;D11LGP2;D11lgp2e;LGP2	DEXH (Asp-Glu-X-His) box polypeptide 58	0.7	0.68	0.98	0.47
CD79B	CD79B;AGM6;B29;IGB	CD79b molecule, immunoglobulin-associated beta	0.7	0.66	0.98	0.57
WIPF2	WIPF2;WICH;WIRE	WAS/WASL interacting protein family, member 2	0.7	0.72	0.98	0.52
WRN	WRN;DKFZp686C2056;RECQ3;RECQL2;RECQL3	Werner syndrome, RecQ helicase-like	0.7	0.76	0.98	0.53
TNC	TNC;150-225;GMEM;GP;HXB;JI;MGC167029;TN;TN-C	tenascin C	0.7	0.7	0.98	0.55
KIAA0556	KIAA0556	KIAA0556	0.7	0.76	0.98	0.56
DNAJB2	DNAJB2;HSJ-1;HSJ1;HSPF3	DnaJ (Hsp40) homolog, subfamily B, member 2	0.7	0.67	0.98	0.51
ARFIP1	ARFIP1;HSU52521;MGC117369	ADP-ribosylation factor interacting protein 1	0.7	0.7	0.98	0.56
KIFC1	KIFC1;HSET;KNSL2;MGC1202;MGC149736;MGC149737	kinesin family member C1	0.7	0.75	0.98	0.56
SYBU	SYBU;FLJ20366;GOLSYN;KIAA1472;OCSYN;SNPHL	syntabulin (syntaxin-interacting)	0.7	0.67	0.98	0.56
RAG1	RAG1;MGC43321;RNF74	recombination activating gene 1	0.69	0.67	0.98	0.6
STXBP6	STXBP6;FLJ39638;amisyn	syntaxin binding protein 6 (amisyn)	0.69	0.7	0.98	0.5
MAB21L3	MAB21L3;C1orf161;FLJ38716;RP5-1185H19.1	mab-21-like 3 (C. elegans)	0.69	0.72	0.98	0.51
ABCA12	ABCA12;DKFZp434G232;FLJ41584;ICR2B;LI2	ATP-binding cassette, sub-family A (ABC1), member 12	0.68	0.65	0.98	0.56
SLC28A3	SLC28A3;CNT3	solute carrier family 28 (sodium-coupled nucleoside transporter), member 3	0.68	0.64	0.98	0.56
GPR68	GPR68;GPR12A;MGC111379;MGC156983;OGR1	G protein-coupled receptor 68	0.68	0.64	0.98	0.58
NOS3	NOS3;ECNOS;eNOS	nitric oxide synthase 3 (endothelial cell)	0.68	0.74	0.98	0.51
ZBTB45	ZBTB45;DKFZp547H249;FLJ14486;ZNF499	zinc finger and BTB domain containing 45	0.68	0.66	0.98	0.49
PPP1R13L	PPP1R13L;IASPP;NKIP1;RAI;RAI4	protein phosphatase 1, regulatory subunit 13 like	0.68	0.7	0.98	0.5
PFN2	PFN2;D3S1319E;PFL	profilin 2	0.68	0.64	0.98	0.49
ZNF747	ZNF747;MGC2474	zinc finger protein 747	0.68	0.73	0.98	0.54
ACOT7	ACOT7;ACH1;ACT;BACH;CTE-II;LACH;LACH1;MGC1126;RP1-120G22.10;hBACH	acyl-CoA thioesterase 7	0.68	0.66	0.98	0.51
MRPL15	MRPL15;L15mt;MRP-L15;MRP-L7;RPML7	mitochondrial ribosomal protein L15	0.68	0.7	0.98	0.46
C1orf198	C1orf198;DKFZp667D152;FLJ14525;FLJ16283;FLJ38847;MGC10710	chromosome 1 open reading frame 198	0.68	0.7	0.98	0.5
HSPB8	HSPB8;CMT2L;DHMN2;E2IG1;H11;HMN2;HMN2A;HSP22	heat shock 22kDa protein 8	0.68	0.67	0.98	0.56
CTPS	CTPS	CTP synthase	0.68	0.64	0.98	0.47
TXN	TXN;DKFZp686B1993;MGC61975;TRDX;TRX;TRX1	thioredoxin	0.68	0.72	0.98	0.54
CASP10	CASP10;ALPS2;FLICE2;MCH4	caspase 10, apoptosis-related cysteine peptidase	0.67	0.66	0.98	0.49
NUP62	NUP62;DKFZp547L134;FLJ20822;FLJ43869;IBSN;MGC841;SNDI;p62	nucleoporin 62kDa	0.67	0.66	0.98	0.54

IL36A	IL36A;FIL1;FIL1(EPSILON);FIL1E;IL-1F6;IL1(EPSILON);IL1F6;MGC129552;MGC129553	interleukin 36, alpha	0.67	0.66	0.98	0.45
COLEC10	COLEC10;CLL1;MGC118794;MGC118795	collectin sub-family member 10 (C-type lectin)	0.66	0.63	0.98	0.48
KSR2	KSR2;FLJ25965	kinase suppressor of ras 2	0.66	0.65	0.98	0.54
TREML2	TREML2;C6orf76;FLJ13693;MGC149715;MGC149716;TLT-2;TLT2;dJ238O23.1	triggering receptor expressed on myeloid cells-like 2	0.66	0.63	0.98	0.51
RAB33A	RAB33A;MGC1488;RabS10	RAB33A, member RAS oncogene family	0.66	0.6	0.98	0.53
TPSAB1	TPSAB1;MGC104064;TPS1;TPS2;TPSB1	tryptase alpha/beta 1	0.66	0.6	0.98	0.49
FGF6	FGF6;HBGF-6;HST2	fibroblast growth factor 6	0.66	0.71	0.98	0.53
HOXC12	HOXC12;HOC3F;HOX3;HOX3F	homeobox C12	0.66	0.67	0.98	0.46
ELFN2	ELFN2;KIAA1904;LRRC62;PPP1R29;dJ63G5.3	extracellular leucine-rich repeat and fibronectin type III domain containing 2	0.66	0.62	0.98	0.5
HMG20A	HMG20A;FLJ10739;HMGX1;HMGXB1	high mobility group 20A	0.66	0.7	0.98	0.53
BIRC2	BIRC2;API1;HIAP2;Hiap-2;MIHB;RNF48;c-IAP1;cIAP1	baculoviral IAP repeat containing 2	0.66	0.64	0.98	0.57
SARNP	SARNP;CIP29;HCC1;HSPC316;MGC14726;THO1	SAP domain containing ribonucleoprotein	0.65	0.67	0.98	0.54
ENTPD5	ENTPD5;CD39L4;MGC163357;MGC163359;NTPDase-5;PCPH	ectonucleoside triphosphate diphosphohydrolase 5	0.64	0.62	0.98	0.46
TMEM125	TMEM125;MGC17299	transmembrane protein 125	0.64	0.62	0.98	0.45
MAN2A1	MAN2A1;GOLIM7;MANA2;MANII	mannosidase, alpha, class 2A, member 1	0.64	0.62	0.98	0.48
SLC25A24	SLC25A24;APC1;DKFZp586G0123;SCAMC-1	solute carrier family 25 (mitochondrial carrier; phosphate carrier), member 24	0.64	0.61	0.98	0.49
UXS1	UXS1;FLJ23591;SDR6E1;UGD	UDP-glucuronate decarboxylase 1	0.64	0.64	0.98	0.43
TMEM176B	TMEM176B;FLJ26014;LR8;MGC110857	transmembrane protein 176B	0.63	0.64	0.98	0.47
CHRNB4	CHRNB4	cholinergic receptor, nicotinic, beta 4	0.63	0.66	0.98	0.51
SLC22A3	SLC22A3;EMT;EMTH;OCT3	solute carrier family 22 (extraneuronal monoamine transporter), member 3	0.62	0.66	0.98	0.5
SMEK1	SMEK1;FLFL1;KIAA2010;PP4R3A;smk-1;smk1	SMEK homolog 1, suppressor of mek1 (Dictyostelium)	0.62	0.6	0.98	0.43
MINPP1	MINPP1;DKFZp564L2016;HIPER1;MINPP2;MIPP	multiple inositol-polyphosphate phosphatase 1	0.62	0.62	0.98	0.47
COG4	COG4;CDG2J;COD1;DKFZp586E1519	component of oligomeric golgi complex 4	0.62	0.6	0.98	0.42
SDC2	SDC2;CD362;HSPG;HSPG1;SYND2	syndecan 2	0.62	0.63	0.98	0.48
CRHBP	CRHBP;CRF-BP;CRFBP	corticotropin releasing hormone binding protein	0.61	0.58	0.98	0.44
MED12L	MED12L;FLJ46288;KIAA1635;NOPAR;TNRC11L;TRALP;TRALPUSH	mediator complex subunit 12-like	0.84	0.84	0.99	0.63
THBS2	THBS2;TSP2	thrombospondin 2	0.84	0.87	0.99	0.58
POU4F3	POU4F3;BRN3C;DFNA15;MGC138412	POU class 4 homeobox 3	0.84	0.83	0.99	0.56
NRL	NRL;D14S46E;NRL-MAF;RP27	neural retina leucine zipper	0.84	0.88	0.99	0.58
RLTPR	RLTPR;CARMIL2;CARMIL2b;LRRC16C	RGD motif, leucine rich repeats, tropomodulin domain and proline-rich containing	0.84	0.8	0.99	0.62
EPHA10	EPHA10;FLJ16103;FLJ33655;MGC43817	EPH receptor A10	0.84	0.84	0.99	0.69
PLDN	PLDN;HPS9;PA;PALLID	pallidin homolog (mouse)	0.84	0.81	0.99	0.68
TIPARP	TIPARP;DKFZp434J214;DKFZp686N0351;DKFZp686P1838;FLJ40466;PARP7;pART14	TCDD-inducible poly(ADP-ribose) polymerase	0.84	0.82	0.99	0.61
MCCC2	MCCC2;MCCB	methylcrotonoyl-CoA carboxylase 2 (beta)	0.84	0.92	0.99	0.64
CSRNP2	CSRNP2;C12orf2;C12orf22;FAM130A1;FLJ25576;PPP1R72;TAIP-12	cysteine-serine-rich nuclear protein 2	0.84	0.87	0.99	0.62
CYP4F2	CYP4F2;CPF2	cytochrome P450, family 4, subfamily F, polypeptide 2	0.83	0.88	0.99	0.66
IZUMO1	IZUMO1;FLJ61440;IZUMO;MGC34799	izumo sperm-egg fusion 1	0.83	0.86	0.99	0.61
EIF4ENIF1	EIF4ENIF1;4E-T;Clast4;FLJ21601;FLJ26551	eukaryotic translation initiation factor 4E nuclear import factor 1	0.83	0.76	0.99	0.67
OLFM4	OLFM4;GC1;GW112;KIAA4294;OLM4;OlfD;UNQ362;bA209J19.1;hGC-1;hOLfD	olfactomedin 4	0.82	0.88	0.99	0.65
ADHFE1	ADHFE1;ADH8;FLJ32430;HOT;MGC48605	alcohol dehydrogenase, iron containing, 1	0.82	0.75	0.99	0.66
CD37	CD37;GP52-40;MGC120234;TSPAN26	CD37 molecule	0.82	0.81	0.99	0.57
DLGAP1	DLGAP1;DAP-1;DAP-1-ALPHA;DAP-1-BETA;FLJ38442;GKAP;MGC88156;SAPAP1;hGKAP	discs, large (Drosophila) homolog-associated protein 1	0.82	0.82	0.99	0.55
ZNF510	ZNF510;KIAA0972;MGC33740	zinc finger protein 510	0.82	0.8	0.99	0.67
LOC401052	LOC401052	uncharacterized LOC401052	0.82	0.84	0.99	0.56
VIPR1	VIPR1;FLJ41949;HVR1;II;PACAP-R-2;PACAP-R2;RDC1;V1RG;VAPC1;VIP-R-1;VIPR;VIRG;VPAC1;VPAC1R;VPCAP1R	vasoactive intestinal peptide receptor 1	0.82	0.79	0.99	0.7
PEX10	PEX10;MGC1998;NALD;RNF69	peroxisomal biogenesis factor 10	0.82	0.79	0.99	0.71
UBE3B	UBE3B;DKFZp586K2123;DKFZp686A1051;FLJ45294;MGC131858;MGC78388	ubiquitin protein ligase E3B	0.82	0.74	0.99	0.56
TFDP2	TFDP2;DKFZp313O196;DP2;FLJ39131	transcription factor Dp-2 (E2F dimerization partner 2)	0.82	0.82	0.99	0.64
DNAJC17	DNAJC17;FLJ10634	DnaJ (Hsp40) homolog, subfamily C, member 17	0.81	0.86	0.99	0.6
TMEM22	TMEM22;DKFZp564K2464;MGC3295	transmembrane protein 22	0.81	0.88	0.99	0.64
LZTS1	LZTS1;F37;FEZ1	leucine zipper, putative tumor suppressor 1	0.81	0.82	0.99	0.65
CLK2	CLK2;MGC61500;hCLK2	CDC-like kinase 2	0.81	0.86	0.99	0.61
KIAA1429	KIAA1429;DKFZp434I116;DKFZp781B2117;MGC138493;MGC141940;fSAP121	KIAA1429	0.81	0.8	0.99	0.57
PHB2	PHB2;BAP;BCAP37;Bap37;MGC117268;PNAS-141;REA;p22	prohibitin 2	0.81	0.82	0.99	0.65
ITIH2	ITIH2;H2P;SHAP	inter-alpha-trypsin inhibitor heavy chain 2	0.8	0.76	0.99	0.6
CLEC4M	CLEC4M;CD209L;CD299;DC-SIGN2;DC-SIGNR;DCSIGNR;HP10347;L-SIGN;LSIGN;MGC129964;MGC47866	C-type lectin domain family 4, member M	0.8	0.85	0.99	0.6
SLC18A1	SLC18A1;CGAT;VAT1;VMAT1	solute carrier family 18 (vesicular monoamine), member 1	0.8	0.74	0.99	0.59
DTX1	DTX1;hDx-1	deltex homolog 1 (Drosophila)	0.8	0.78	0.99	0.7
DHX34	DHX34;DDX34;HRH1;KIAA0134	DEAH (Asp-Glu-Ala-His) box polypeptide 34	0.8	0.77	0.99	0.54
ELOVL6	ELOVL6;FACE;FAE;FLJ23378;LCE;MGC5487	ELOVL fatty acid elongase 6	0.8	0.76	0.99	0.59
C2orf55	C2orf55;MGC42367	chromosome 2 open reading frame 55	0.8	0.74	0.99	0.61
CCDC88C	CCDC88C;DAPLE;FLJ00354;HkRP2;KIAA1509	coiled-coil domain containing 88C	0.8	0.82	0.99	0.61
LSMD1	LSMD1;MGC14151;MGC74837;PFAAP2	LSM domain containing 1	0.8	0.78	0.99	0.58
OSBPL3	OSBPL3;DKFZp667P1518;KIAA0704;MGC21526;ORP-3;ORP3;OSBP3	oxysterol binding protein-like 3	0.8	0.79	0.99	0.55
DAPK1	DAPK1;DAPK;DKFZp781I035	death-associated protein kinase 1	0.8	0.79	0.99	0.65
APEX2	APEX2;APE2;APEXL2;XTH2	APEX nuclease (apurinic/apyrimidinic endonuclease) 2	0.8	0.73	0.99	0.58
TMEM167A	TMEM167A;DKFZp686I1152;FLJ30508;MGC23909;TMEM167	transmembrane protein 167A	0.8	0.87	0.99	0.58
ACAT2	ACAT2	acetyl-CoA acetyltransferase 2	0.8	0.78	0.99	0.58
IL36G	IL36G;IL-1F9;IL-1H1;IL-1RP2;IL1E;IL1F9;IL1H1;IL1RP2	interleukin 36, gamma	0.8	0.82	0.99	0.62
LOC149469	LOC149469	ENTREZ STATUS: REMAPPED TO GENE ID: 23334	0.8	0.76	0.99	0.61
CDHR2	CDHR2;FLJ20124;FLJ20383;MGC163154;PCDH24;PCLKC	cadherin-related family member 2	0.8	0.79	0.99	0.55
DHRSX	DHRSX;CXorf11;DHRS5X;DHRS5Y;DHRSXY;DHRSY;SDR46C1	dehydrogenase/reductase (SDR family) X-linked	0.8	0.84	0.99	0.55
TNIK	TNIK	TRAF2 and NCK interacting kinase	0.79	0.77	0.99	0.64
AMDHD1	AMDHD1;MGC35366	amidohydrolase domain containing 1	0.79	0.78	0.99	0.56
HLA-DQA1	HLA-DQA1;CD;CELIAC1;DQ-A1;FLJ27088;FLJ27328;GSE;HLA-DQA;MGC149527	major histocompatibility complex, class II, DQ alpha 1	0.79	0.72	0.99	0.64
HSPA12A	HSPA12A;FLJ13874;KIAA0417	heat shock 70kDa protein 12A	0.79	0.82	0.99	0.66
ZBTB7A	ZBTB7A;DKFZp547O146;FBI-1;FBI1;LRF;MGC99631;ZBTB7;ZNF857A;pokemon	zinc finger and BTB domain containing 7A	0.79	0.78	0.99	0.58
ACVR1B	ACVR1B;ACTRIB;ACVRLK4;ALK4;SKR2	activin A receptor, type IB	0.79	0.78	0.99	0.65
C4orf6	C4orf6;aC1	chromosome 4 open reading frame 6	0.78	0.8	0.99	0.59
B4GALT6	B4GALT6;B4Gal-T6;beta4Gal-T6	UDP-Gal:betaGlcNAc beta 1,4- galactosyltransferase, polypeptide 6	0.78	0.76	0.99	0.57
HKR1	HKR1;ZNF875	HKR1, GLI-Kruppel zinc finger family member	0.78	0.82	0.99	0.61
LILRB5	LILRB5;CD85C;LIR-8;LIR8	leukocyte immunoglobulin-like receptor, subfamily B (with TM and ITIM domains), member 5	0.78	0.86	0.99	0.59
PPP1R16B	PPP1R16B;ANKRD4;KIAA0823;TIMAP	protein phosphatase 1, regulatory subunit 16B	0.78	0.78	0.99	0.61
SUOX	SUOX	sulfite oxidase	0.78	0.72	0.99	0.57
PTPN12	PTPN12;PTP-PEST;PTPG1	protein tyrosine phosphatase, non-receptor type 12	0.78	0.76	0.99	0.59
PIP4K2A	PIP4K2A;FLJ13267;PI5P4KA;PIP5K2A;PIP5KII-alpha;PIP5KIIA;PIPK	phosphatidylinositol-5-phosphate 4-kinase, type II, alpha	0.78	0.78	0.99	0.65
BTF3	BTF3;BETA-NAC;BTF3a;BTF3b;NACB	basic transcription factor 3	0.78	0.83	0.99	0.62
BICC1	BICC1;BICC;DKFZp686I21119	bicaudal C homolog 1 (Drosophila)	0.78	0.74	0.99	0.59
ENKUR	ENKUR;C10orf63;DKFZp781F21103;MGC26778;enkurin	enkurin, TRPC channel interacting protein	0.78	0.8	0.99	0.6
ZNF19	ZNF19;KOX12;MGC51021	zinc finger protein 19	0.77	0.76	0.99	0.52
PLEKHA6	PLEKHA6;KIAA0969;MGC176733;PEPP3	pleckstrin homology domain containing, family A member 6	0.77	0.76	0.99	0.55
LUZP1	LUZP1;FLJ35697;KIAA0601;LUZP	leucine zipper protein 1	0.77	0.81	0.99	0.54
ISOC2	ISOC2;FLJ18582	isochorismatase domain containing 2	0.77	0.76	0.99	0.56
ATP5A1	ATP5A1;ATP5A;ATP5AL2;ATPM;MOM2;OMR;ORM;hATP1	ATP synthase, H+ transporting, mitochondrial F1 complex, alpha subunit 1, cardiac muscle	0.77	0.75	0.99	0.64
LGSN	LGSN;GLULD1;LGS;MGC163238;MGC163240	lengsin, lens protein with glutamine synthetase domain	0.77	0.77	0.99	0.51
SPINK4	SPINK4;MGC133107;PEC-60;PEC60	serine peptidase inhibitor, Kazal type 4	0.76	0.73	0.99	0.59
OR4X1	OR4X1;OR11-104	olfactory receptor, family 4, subfamily X, member 1	0.76	0.72	0.99	0.55
OR2M4	OR2M4;HSHTPCRX18;HTPCRX18;OR1-55;OST710;TPCR100	olfactory receptor, family 2, subfamily M, member 4	0.76	0.79	0.99	0.53
RORB	RORB;NR1F2;ROR-BETA;RZR-BETA;RZRB;bA133M9.1	RAR-related orphan receptor B	0.76	0.7	0.99	0.56
MASP1	MASP1;CRARF;CRARF1;DKFZp686I01199;FLJ26383;MAP1;MASP;MASP3;MAp44;MGC126283;MGC126284;PRSS5;RaRF	mannan-binding lectin serine peptidase 1 (C4/C2 activating component of Ra-reactive factor)	0.76	0.74	0.99	0.55
ZNF708	ZNF708;DKFZp686L10267;FLJ16755;FLJ46310;KOX8;ZNF15;ZNF15L1	zinc finger protein 708	0.76	0.78	0.99	0.59
GPD1	GPD1;FLJ26652	glycerol-3-phosphate dehydrogenase 1 (soluble)	0.76	0.74	0.99	0.57
SLC4A3	SLC4A3;AE3;SLC2C	solute carrier family 4, anion exchanger, member 3	0.76	0.74	0.99	0.64
REPS2	REPS2;POB1	RALBP1 associated Eps domain containing 2	0.76	0.72	0.99	0.57
MED20	MED20;DKFZp586D2223;MGC29869;PRO0213;TRFP	mediator complex subunit 20	0.76	0.73	0.99	0.59
HUWE1	HUWE1;ARF-BP1;HECTH9;HSPC272;Ib772;KIAA0312;LASU1;MULE;UREB1	HECT, UBA and WWE domain containing 1	0.76	0.68	0.99	0.51
PANX3	PANX3;PX3	pannexin 3	0.75	0.72	0.99	0.58
PAGE5	PAGE5;CT16;CT16.1;CT16.2;GAGEE1;PAGE-5	P antigen family, member 5 (prostate associated)	0.75	0.72	0.99	0.54
ACADVL	ACADVL;ACAD6;LCACD;VLCAD	acyl-CoA dehydrogenase, very long chain	0.75	0.71	0.99	0.51
CRCT1	CRCT1;C1orf42;NICE-1	cysteine-rich C-terminal 1	0.74	0.71	0.99	0.53
ST8SIA1	ST8SIA1;GD3S;SIAT8;SIAT8A;ST8SiaI	ST8 alpha-N-acetyl-neuraminide alpha-2,8-sialyltransferase 1	0.74	0.71	0.99	0.51
GSPT2	GSPT2;ERF3B;FLJ10441;GST2	G1 to S phase transition 2	0.74	0.72	0.99	0.53
SLC37A2	SLC37A2;FLJ00171;MGC71430;pp11662	solute carrier family 37 (glycerol-3-phosphate transporter), member 2	0.74	0.78	0.99	0.59
FES	FES;FPS	feline sarcoma oncogene	0.74	0.72	0.99	0.65
KIAA1755	KIAA1755	KIAA1755	0.74	0.69	0.99	0.56
PVRIG	PVRIG;C7orf15;MGC104322;MGC138295;MGC138297;MGC2463	poliovirus receptor related immunoglobulin domain containing	0.74	0.78	0.99	0.55
CWF19L2	CWF19L2;FLJ32343	CWF19-like 2, cell cycle control (S. pombe)	0.74	0.67	0.99	0.53
HOXB6	HOXB6;HOX2;HOX2B;HU-2;Hox-2.2	homeobox B6	0.74	0.7	0.99	0.61
GPR137B	GPR137B;TM7SF1	G protein-coupled receptor 137B	0.74	0.78	0.99	0.59
TNRC18	TNRC18;CAGL79;KIAA1856;TNRC18A	trinucleotide repeat containing 18	0.74	0.76	0.99	0.57
CDKN1B	CDKN1B;CDKN4;KIP1;MEN1B;MEN4;P27KIP1	cyclin-dependent kinase inhibitor 1B (p27, Kip1)	0.74	0.79	0.99	0.57
COL4A1	COL4A1;arresten	collagen, type IV, alpha 1	0.74	0.76	0.99	0.61
APOBEC3B	APOBEC3B;APOBEC1L;ARCD3;ARP4;DJ742C19.2;FLJ21201;PHRBNL;bK150C2.2	apolipoprotein B mRNA editing enzyme, catalytic polypeptide-like 3B	0.74	0.7	0.99	0.54
PDIA3	PDIA3;ER60;ERp57;ERp60;ERp61;GRP57;GRP58;HsT17083;P58;PI-PLC	protein disulfide isomerase family A, member 3	0.74	0.68	0.99	0.51
METTL12	METTL12	methyltransferase like 12	0.74	0.76	0.99	0.51
CX3CR1	CX3CR1;CCRL1;CMKBRL1;CMKDR1;GPR13;GPRV28;V28	chemokine (C-X3-C motif) receptor 1	0.73	0.7	0.99	0.63
SHH	SHH;HHG1;HLP3;HPE3;MCOPCB5;SMMCI;TPT;TPTPS	sonic hedgehog	0.73	0.72	0.99	0.54
GPR44	GPR44;CD294;CRTH2;DL1R;DP2	G protein-coupled receptor 44	0.73	0.69	0.99	0.63
PCDHGB2	PCDHGB2;MGC126854;PCDH-GAMMA-B2	protocadherin gamma subfamily B, 2	0.73	0.75	0.99	0.56

CNST	CNST;C1orf71;FLJ32001;MGC18089;PPP1R64	consortin, connexin sorting protein	0.73	0.64	0.99	0.5
ACOX2	ACOX2;BCOX;BRCACOX;BRCOX;THCCox	acyl-CoA oxidase 2, branched chain	0.72	0.68	0.99	0.53
MYEOV	MYEOV;OCIM	myeloma overexpressed (in a subset of t(11;14) positive multiple myelomas)	0.72	0.65	0.99	0.52
HEYL	HEYL;HEY3;HRT3;MGC12623;bHLHb33	hairy/enhancer-of-split related with YRPW motif-like	0.72	0.78	0.99	0.54
MDFI	MDFI;I-MF;I-mfa	MyoD family inhibitor	0.72	0.75	0.99	0.56
PDGFD	PDGFD;IEGF;MGC26867;SCDGF-B;SCDGFB	platelet derived growth factor D	0.72	0.68	0.99	0.54
HS3ST6	HS3ST6;HS3ST5	heparan sulfate (glucosamine) 3-O-sulfotransferase 6	0.72	0.76	0.99	0.5
CUL4B	CUL4B;DKFZp686F1470;KIAA0695;MRXHF2;MRXS15;MRXSC;SFM2	cullin 4B	0.72	0.66	0.99	0.49
RGPD7	RGPD7	ENTREZ STATUS: DISCONTINUED	0.72	0.73	0.99	0.49
CST8	CST8;CRES	cystatin 8 (cystatin-related epididymal specific)	0.71	0.68	0.99	0.55
PLEKHM3	PLEKHM3;DAPR;DKFZp686O04200;FLJ41210;PLEKHM1L	pleckstrin homology domain containing, family M, member 3	0.71	0.66	0.99	0.51
FMO3	FMO3;FMOII;MGC34400;TMAU;dJ127D3.1	flavin containing monooxygenase 3	0.7	0.77	0.99	0.53
DOK2	DOK2;p56DOK;p56dok-2	docking protein 2, 56kDa	0.7	0.74	0.99	0.59
KRTAP23-1	KRTAP23-1;KAP23.1	keratin associated protein 23-1	0.7	0.68	0.99	0.57
IKBKG	IKBKG;AMCBX1;FIP-3;FIP3;Fip3p;IKK-gamma;IP;IP1;IP2;IPD2;NEMO	inhibitor of kappa light polypeptide gene enhancer in B-cells, kinase gamma	0.7	0.74	0.99	0.53
CPLX1	CPLX1;CPX-I;CPX1	complexin 1	0.7	0.74	0.99	0.59
LGALS9B	LGALS9B;FLJ58146;FLJ75081	lectin, galactoside-binding, soluble, 9B	0.7	0.72	0.99	0.55
NEK9	NEK9;DKFZp434D0935;MGC138306;MGC16714;NERCC;NERCC1;Nek8	NIMA (never in mitosis gene a)- related kinase 9	0.7	0.68	0.99	0.57
NDUFB5	NDUFB5;CISGDH;DKFZp686N02262;FLJ30597;MGC111204;MGC12314;SGDH	NADH dehydrogenase (ubiquinone) 1 beta subcomplex, 5, 16kDa	0.7	0.74	0.99	0.56
TSTA3	TSTA3;FX;P35B;SDR4E1	tissue specific transplantation antigen P35B	0.7	0.64	0.99	0.51
RRP8	RRP8;KIAA0409	ribosomal RNA processing 8, methyltransferase, homolog (yeast)	0.7	0.62	0.99	0.52
SPRY4	SPRY4	sprouty homolog 4 (Drosophila)	0.69	0.7	0.99	0.53
PRRG4	PRRG4;PRGP4;TMG4	proline rich Gla (G-carboxyglutamic acid) 4 (transmembrane)	0.69	0.74	0.99	0.55
HSPA2	HSPA2;HSP70-2;HSP70-3	heat shock 70kDa protein 2	0.69	0.64	0.99	0.56
SMS	SMS;MRSR;SPMSY;SRS;SpS	spermine synthase	0.69	0.67	0.99	0.57
CD200R1L	CD200R1L;CD200R2;CD200RLa	CD200 receptor 1-like	0.69	0.74	0.99	0.55
NBPF7	NBPF7	neuroblastoma breakpoint family, member 7	0.68	0.66	0.99	0.49
HEY1	HEY1;BHLHb31;CHF2;HERP2;HESR1;HRT-1;MGC1274;OAF1	hairy/enhancer-of-split related with YRPW motif 1	0.68	0.74	0.99	0.52
CCDC33	CCDC33;CT61;FLJ23168;FLJ32855;MGC34145	coiled-coil domain containing 33	0.68	0.73	0.99	0.49
MBD3	MBD3	methyl-CpG binding domain protein 3	0.68	0.72	0.99	0.5
PALM	PALM;KIAA0270	paralemmin	0.68	0.66	0.99	0.51
C7orf36	C7orf36	chromosome 7 open reading frame 36	0.68	0.67	0.99	0.5
PDHX	PDHX;DLDBP;E3BP;OPDX;PDX1;proX	pyruvate dehydrogenase complex, component X	0.68	0.64	0.99	0.47
CD99	CD99;HBA71;MIC2;MIC2X;MIC2Y;MSK5X	CD99 molecule	0.68	0.66	0.99	0.52
OR2AG1	OR2AG1;OR11-79;OR2AG3	olfactory receptor, family 2, subfamily AG, member 1	0.67	0.66	0.99	0.46
CYP24A1	CYP24A1;CP24;CYP24;HCAI;MGC126273;MGC126274;P450-CC24	cytochrome P450, family 24, subfamily A, polypeptide 1	0.67	0.66	0.99	0.52
SLC1A7	SLC1A7;AAAT;EAAT5;FLJ36602	solute carrier family 1 (glutamate transporter), member 7	0.67	0.73	0.99	0.5
ABHD14B	ABHD14B;CIB;MGC15429	abhydrolase domain containing 14B	0.67	0.66	0.99	0.49
C17orf81	C17orf81;DERP6;MST071;MSTP071	chromosome 17 open reading frame 81	0.67	0.71	0.99	0.5
MCL1	MCL1;BCL2L3;EAT;MCL1-ES;MCL1L;MCL1S;MGC104264;MGC1839;Mcl-1;TM;bcl2-L-3;mcl1/EAT	myeloid cell leukemia sequence 1 (BCL2-related)	0.67	0.61	0.99	0.54
ADCY5	ADCY5;AC5	adenylate cyclase 5	0.66	0.64	0.99	0.45
ALDH3B2	ALDH3B2;ALDH8	aldehyde dehydrogenase 3 family, member B2	0.66	0.64	0.99	0.46
C1orf131	C1orf131;DKFZp547B1713	chromosome 1 open reading frame 131	0.66	0.62	0.99	0.48
MDM1	MDM1;FLJ95264	Mdm1 nuclear protein homolog (mouse)	0.66	0.68	0.99	0.46
FLVCR2	FLVCR2;C14orf58;CCT;EPV;FLJ20371;FLVCRL14q;MFSD7C;PVHH	feline leukemia virus subgroup C cellular receptor family, member 2	0.66	0.62	0.99	0.5
ERN1	ERN1;FLJ30999;IRE1;IRE1P;IRE1a;MGC163277;MGC163279;hIRE1p	endoplasmic reticulum to nucleus signaling 1	0.66	0.72	0.99	0.51
FLNC	FLNC;ABP-280;ABP280A;ABPA;ABPL;FLJ10186;FLN2;MFM5;MPD4	filamin C, gamma	0.66	0.71	0.99	0.53
ZNF564	ZNF564;FLJ38281;MGC26914	zinc finger protein 564	0.66	0.66	0.99	0.5
SPG11	SPG11;DKFZp762B1512;FLJ21439;KIAA1840	spastic paraplegia 11 (autosomal recessive)	0.66	0.68	0.99	0.49
DIDO1	DIDO1;BYE1;C20orf158;DATF1;DIDO2;DIDO3;DIO-1;DIO1;DKFZp434P1115;FLJ11265;KIAA0333;MGC16140;dJ885L7.8	death inducer-obliterator 1	0.66	0.7	0.99	0.49
ANO10	ANO10;FLJ10375;MGC47890;SCAR10;TMEM16K	anoctamin 10	0.66	0.7	0.99	0.54
MCM3	MCM3;HCC5;MGC1157;P1-MCM3;P1.h;RLFB	minichromosome maintenance complex component 3	0.66	0.63	0.99	0.49
DEFB104B	DEFB104B;BD-4;DEFB-4;DEFB104A;hBD-4	defensin, beta 104B	0.66	0.58	0.99	0.47
PLA2G10	PLA2G10;GXPLA2;GXSPLA2;MGC119918;MGC119919;MGC133367;SPLA2	phospholipase A2, group X	0.64	0.63	0.99	0.49
COX4I2	COX4I2;COX4;COX4-2;COX4B;COX4L2;COXIV-2;dJ857M17.2	cytochrome c oxidase subunit IV isoform 2 (lung)	0.64	0.68	0.99	0.51
ZNF85	ZNF85;HPF4;HTF1;MGC78566	zinc finger protein 85	0.64	0.62	0.99	0.53
HAAO	HAAO;3-HAO;HAO	3-hydroxyanthranilate 3,4-dioxygenase	0.64	0.6	0.99	0.47
A1BG	A1BG;A1B;ABG;DKFZp686F0970;GAB;HYST2477	alpha-1-B glycoprotein	0.64	0.66	0.99	0.54
SLC19A1	SLC19A1;CHMD;FOLT;IFC1;REFC;RFC1	solute carrier family 19 (folate transporter), member 1	0.64	0.65	0.99	0.5
UBIAD1	UBIAD1;RP4-796F18.1;SCCD;TERE1	UbiA prenyltransferase domain containing 1	0.64	0.6	0.99	0.51
C7orf50	C7orf50;MGC11257;YCR016W	chromosome 7 open reading frame 50	0.64	0.6	0.99	0.48
HMGB2	HMGB2;HMG2	high mobility group box 2	0.64	0.64	0.99	0.43
MYO1C	MYO1C;FLJ23903;MMI-beta;MMIb;NMI;myr2	myosin IC	0.64	0.65	0.99	0.5
SULT1A4	SULT1A4;FLJ54864;HAST3;M-PST;ST1A3/ST1A4;TL-PST	sulfotransferase family, cytosolic, 1A, phenol-preferring, member 4	0.64	0.62	0.99	0.46
MRPL23	MRPL23;FLJ45387;L23MRP;RPL23;RPL23L	mitochondrial ribosomal protein L23	0.63	0.68	0.99	0.5
CDYL	CDYL;CDYL1;DKFZp586C1622;MGC131936	chromodomain protein, Y-like	0.63	0.6	0.99	0.43
CMTM7	CMTM7;CKLFSF7;FLJ30992	CKLF-like MARVEL transmembrane domain containing 7	0.63	0.66	0.99	0.47
OR52M1	OR52M1;OR11-11;OR52M1P;OR52M3P	olfactory receptor, family 52, subfamily M, member 1	0.62	0.55	0.99	0.46
BTG1	BTG1	B-cell translocation gene 1, anti-proliferative	0.62	0.59	0.99	0.51
PRKCH	PRKCH;MGC26269;MGC5363;PKC-L;PKCL;PRKCL;nPKC-eta	protein kinase C, eta	0.62	0.66	0.99	0.47
DBI	DBI;ACBD1;ACBP;CCK-RP;EP;MGC70414	diazepam binding inhibitor (GABA receptor modulator, acyl-CoA binding protein)	0.62	0.64	0.99	0.42
GNL2	GNL2;FLJ40906;HUMAUANTIG;NGP1;Ngp-1	guanine nucleotide binding protein-like 2 (nucleolar)	0.62	0.61	0.99	0.47
ECT2L	ECT2L;ARHGEF32;C6orf91;FBXO49;LFDH;dJ509I19.2;dJ509I19.3;dJ509I19.5	epithelial cell transforming sequence 2 oncogene-like	0.62	0.66	0.99	0.46
SLC41A2	SLC41A2;DKFZp434K0427;MGC125330;MGC125331;SLC41A1-L1	solute carrier family 41, member 2	0.61	0.58	0.99	0.51
RPL18P4	RPL18P4;RPL18_1_838	ribosomal protein L18 pseudogene 4	0.61	0.64	0.99	0.45
IRGC	IRGC;CINEMA;IRGC1;Iigp5;R30953_1	immunity-related GTPase family, cinema	0.84	0.9	1	0.69
GPR124	GPR124;DKFZp434C211;DKFZp434J0911;FLJ14390;KIAA1531;TEM5	G protein-coupled receptor 124	0.84	0.81	1	0.73
PTK6	PTK6;BRK;FLJ42088	PTK6 protein tyrosine kinase 6	0.84	0.83	1	0.68
TH	TH;DYT14;DYT5b;TYH	tyrosine hydroxylase	0.84	0.8	1	0.63
NFATC3	NFATC3;NFAT4;NFATX	nuclear factor of activated T-cells, cytoplasmic, calcineurin-dependent 3	0.84	0.82	1	0.64
TSC2	TSC2;FLJ43106;LAM;TSC4	tuberous sclerosis 2	0.84	0.91	1	0.68
PAK1	PAK1;MGC130000;MGC130001;PAKalpha	p21 protein (Cdc42/Rac)-activated kinase 1	0.84	0.82	1	0.68
C6orf145	C6orf145	chromosome 6 open reading frame 145	0.84	0.84	1	0.61
MRPL45	MRPL45;L45mt;MGC11321;MRP-L45	mitochondrial ribosomal protein L45	0.84	0.83	1	0.69
KRT19	KRT19;CK19;K19;K1CS;MGC15366	keratin 19	0.84	0.8	1	0.62
NACA	NACA;MGC117224;NACA1	nascent polypeptide-associated complex alpha subunit	0.84	0.86	1	0.66
PCDHGA6	PCDHGA6;PCDH-GAMMA-A6	protocadherin gamma subfamily A, 6	0.84	0.8	1	0.67
METTL23	METTL23;C17orf95	methyltransferase like 23	0.84	0.88	1	0.62
ZNF280C	ZNF280C;FLJ20095;SUHW3;ZNF633	zinc finger protein 280C	0.83	0.82	1	0.65
CSTF1	CSTF1;CstF-50;CstFp50	cleavage stimulation factor, 3' pre-RNA, subunit 1, 50kDa	0.83	0.84	1	0.64
C3orf26	C3orf26;MGC4308	chromosome 3 open reading frame 26	0.83	0.9	1	0.6
EBF1	EBF1;COE1;EBF;FLJ39389;FLJ41763;O/E-1;OLF1	early B-cell factor 1	0.82	0.88	1	0.61
CPT1C	CPT1C;CATL1;CPT1-B;CPT1P;CPTI-B;CPTIC;FLJ23809	carnitine palmitoyltransferase 1C	0.82	0.81	1	0.6
IQCG	IQCG;DKFZp434B227;FLJ11667;FLJ23571;FLJ37775	IQ motif containing G	0.82	0.82	1	0.59
C3orf45	C3orf45;FLJ38608	chromosome 3 open reading frame 45	0.82	0.79	1	0.6
LMF1	LMF1;C16orf26;FLJ12681;FLJ22302;HMFN1876;JFP11;TMEM112;TMEM112A	lipase maturation factor 1	0.82	0.89	1	0.65
ACVR1	ACVR1;ACTRI;ACVR1A;ACVRLK2;ALK2;FOP;SKR1;TSRI	activin A receptor, type I	0.82	0.82	1	0.68
DUT	DUT;FLJ20622;dUTPase	deoxyuridine triphosphatase	0.82	0.8	1	0.69
C11orf73	C11orf73;FLJ43020;HSPC179;L7RN6	chromosome 11 open reading frame 73	0.82	0.77	1	0.62
ARL13B	ARL13B;ARL2L1;DKFZp686E2075;DKFZp686L2472;DKFZp686M2074;DKFZp761H079;JBTS8;MGC120611;MGC120612	ADP-ribosylation factor-like 13B	0.82	0.73	1	0.57
TMEM160	TMEM160;FLJ20512	transmembrane protein 160	0.82	0.88	1	0.59
IDH3G	IDH3G;H-IDHG	isocitrate dehydrogenase 3 (NAD+) gamma	0.82	0.8	1	0.56
ARL8B	ARL8B;ARL10C;FLJ10702;Gie1	ADP-ribosylation factor-like 8B	0.82	0.88	1	0.68
FAM127A	FAM127A;CXX1;MAR8C;MART8C;MGC117411;Mar8;Mart8	family with sequence similarity 127, member A	0.82	0.84	1	0.62
ZNF616	ZNF616;MGC149778;MGC45556	zinc finger protein 616	0.81	0.8	1	0.67
TXNDC15	TXNDC15;C5orf14;FLJ22625;UNQ335	thioredoxin domain containing 15	0.81	0.74	1	0.66
KRT7	KRT7;CK7;K2C7;K7;MGC129731;MGC3625;SCL	keratin 7	0.81	0.82	1	0.6
OR4L1	OR4L1;OR14-28;OR4L2P	olfactory receptor, family 4, subfamily L, member 1	0.8	0.83	1	0.63
BDH2	BDH2;DHRS6;EFA6R;FLJ13261;PRO20933;SDR15C1;UCPA-OR;UNQ6308	3-hydroxybutyrate dehydrogenase, type 2	0.8	0.86	1	0.64
TYW1	TYW1;FLJ10900;MGC23001;MGC60291;RSAFD1;TYW1A;YPL207W	tRNA-yW synthesizing protein 1 homolog (S. cerevisiae)	0.8	0.86	1	0.67
IRX2	IRX2;IRXA2	iroquois homeobox 2	0.8	0.78	1	0.63
ACTA1	ACTA1;ACTA;ASMA;CFTD;CFTD1;CFTDM;MPFD;NEM1;NEM2;NEM3	actin, alpha 1, skeletal muscle	0.8	0.78	1	0.62
CDC25A	CDC25A;CDC25A2	cell division cycle 25 homolog A (S. pombe)	0.8	0.76	1	0.57
RNF219	RNF219;C13orf7;DKFZp686A01276;DKFZp686N15250;DKFZp686O03173;FLJ13449;FLJ25774	ring finger protein 219	0.8	0.79	1	0.71
MRPL49	MRPL49;C11orf4;L49mt;MGC10656;MRP-L49;NOF;NOF1	mitochondrial ribosomal protein L49	0.8	0.77	1	0.61
MTFR1	MTFR1;CHPPR;FAM54A2;KIAA0009	mitochondrial fission regulator 1	0.8	0.74	1	0.6
HIST1H4B	HIST1H4B;H4/I;H4FI	histone cluster 1, H4b	0.8	0.8	1	0.54
RAB11A	RAB11A;MGC1490;YL8	RAB11A, member RAS oncogene family	0.8	0.8	1	0.54
ANAPC13	ANAPC13;APC13;DKFZp566D193;SWM1	anaphase promoting complex subunit 13	0.8	0.82	1	0.62
FUBP1	FUBP1;FBP;FUBP	far upstream element (FUSE) binding protein 1	0.8	0.77	1	0.57
HSPD1	HSPD1;CPN60;GROEL;HLD4;HSP-60;HSP60;HSP65;HuCHA60;SPG13	heat shock 60kDa protein 1 (chaperonin)	0.8	0.77	1	0.57
CD101	CD101;IGSF2;V7	CD101 molecule	0.8	0.82	1	0.59
ORC3	ORC3;LAT;LATHEO;ORC3L	origin recognition complex, subunit 3	0.8	0.84	1	0.68
B3GNT7	B3GNT7;beta3GnT7	UDP-GlcNAc:betaGal beta-1,3-N-acetylglucosaminyltransferase 7	0.79	0.78	1	0.54
DGCR14	DGCR14;DGCR13;DGS-H;DGS-I;DGSH;DGSI;ES2;Es2el	DiGeorge syndrome critical region gene 14	0.79	0.74	1	0.63
SSX2	SSX2;CT5.2a;HD21;HOM-MEL-40;MGC119055;MGC15364;MGC3884;SSX;SSX2B	synovial sarcoma, X breakpoint 2	0.78	0.74	1	0.63
C11orf16	C11orf16	chromosome 11 open reading frame 16	0.78	0.76	1	0.63
PCSK5	PCSK5;FLJ11149;FLJ16215;PC5;PC6;PC6A;SPC6	proprotein convertase subtilisin/kexin type 5	0.78	0.76	1	0.58
FAR2	FAR2;FLJ10462;MLSTD1;SDR10E2	fatty acyl CoA reductase 2	0.78	0.82	1	0.56

DUS2L	DUS2L;DUS2;FLJ20399;SMM1;URLC8	dihydrouridine synthase 2-like, SMM1 homolog (S. cerevisiae)	0.78	0.84	1	0.65
SLCO4A1	SLCO4A1;OATP-E;OATP1;OATP4A1;OATPE;OATPRP1;POAT;SLC21A12	solute carrier organic anion transporter family, member 4A1	0.78	0.76	1	0.6
RNFT1	RNFT1;MGC111090	ring finger protein, transmembrane 1	0.78	0.77	1	0.69
ELOVL7	ELOVL7;FLJ23563	ELOVL fatty acid elongase 7	0.78	0.8	1	0.57
GLYATL1	GLYATL1;FLJ26507;FLJ34646;GATF-C;GNAT;MGC15397;MGC15937	glycine-N-acyltransferase-like 1	0.77	0.82	1	0.62
FKTN	FKTN;CMD1X;FCMD;LGMD2M;MDDGA4;MDDGB4;MDDGC4;MGC126857;MGC134944;MGC134945;MGC138243	fukutin	0.77	0.82	1	0.66
AXL	AXL;JTK11;UFO	AXL receptor tyrosine kinase	0.77	0.77	1	0.64
POLA2	POLA2;FLJ21662;FLJ37250	polymerase (DNA directed), alpha 2 (70kD subunit)	0.77	0.78	1	0.62
ZNF547	ZNF547;FLJ31100	zinc finger protein 547	0.76	0.75	1	0.67
FCN3	FCN3;FCNH;HAKA1;MGC22543	ficolin (collagen/fibrinogen domain containing) 3 (Hakata antigen)	0.76	0.76	1	0.53
C6orf225	C6orf225;DKFZp586F0922	chromosome 6 open reading frame 225	0.76	0.74	1	0.58
KRTAP5-4	KRTAP5-4;KRTAP5.4	keratin associated protein 5-4	0.76	0.74	1	0.56
PAX2	PAX2	paired box 2	0.76	0.81	1	0.61
DNALI1	DNALI1;P28;dJ423B22.5;hp28	dynein, axonemal, light intermediate chain 1	0.76	0.72	1	0.61
C6orf138	C6orf138;FLJ41841;dJ402H5.2	chromosome 6 open reading frame 138	0.76	0.74	1	0.56
SBK1	SBK1;Sbk	SH3-binding domain kinase 1	0.76	0.83	1	0.59
C1orf86	C1orf86;FLJ30256;FLJ31031;FLJ36608;FLJ43456;FLJ46660;FP7162;MGC120153	chromosome 1 open reading frame 86	0.76	0.72	1	0.58
PRKAR1B	PRKAR1B;PRKAR1	protein kinase, cAMP-dependent, regulatory, type I, beta	0.76	0.83	1	0.59
PRDM2	PRDM2;HUMHOXY1;KMT8;MTB-ZF;RIZ;RIZ1;RIZ2	PR domain containing 2, with ZNF domain	0.76	0.71	1	0.62
TOP3B	TOP3B;FLJ39376	topoisomerase (DNA) III beta	0.76	0.78	1	0.52
FANCD2	FANCD2;DKFZp762A223;FA-D2;FA4;FACD;FAD;FAD2;FANCD;FLJ23826	Fanconi anemia, complementation group D2	0.76	0.78	1	0.59
ANKRD1	ANKRD1;ALRP;C-193;CARP;CVARP;MCARP;bA320F15.2	ankyrin repeat domain 1 (cardiac muscle)	0.76	0.7	1	0.62
TSSK4	TSSK4;C14orf20;MGC133264;STK22E;TSK-4;TSK4;TSSK5	testis-specific serine kinase 4	0.75	0.8	1	0.58
LEFTY2	LEFTY2;EBAF;LEFTA;LEFTYA;MGC46222;TGFB4	left-right determination factor 2	0.75	0.74	1	0.55
SMYD2	SMYD2;HSKM-B;KMT3C;MGC119305;ZMYND14	SET and MYND domain containing 2	0.75	0.7	1	0.55
TBCK	TBCK;MGC16169;TBCKL	TBC1 domain containing kinase	0.75	0.81	1	0.57
MIIP	MIIP;FLJ12438;FLJ38609;IIP45	migration and invasion inhibitory protein	0.75	0.82	1	0.54
HLA-DQB1	HLA-DQB1;CELIAC1;HLA-DQB;IDDM1	major histocompatibility complex, class II, DQ beta 1	0.74	0.73	1	0.54
DEFB113	DEFB113;DEFB-13	defensin, beta 113	0.74	0.7	1	0.51
THTPA	THTPA;MGC2652;THTP;THTPASE	thiamine triphosphatase	0.74	0.68	1	0.52
MBP	MBP;MGC99675	myelin basic protein	0.74	0.68	1	0.54
HMGB1	HMGB1;DKFZp686A04236;HMG1;HMG3;SBP-1	high mobility group box 1	0.74	0.72	1	0.56
ZFP36L2	ZFP36L2;BRF2;ERF-2;ERF2;RNF162C;TIS11D	zinc finger protein 36, C3H type-like 2	0.74	0.74	1	0.61
SPATS2	SPATS2;FLJ13117;P59SCR;SCR59;SPATA10	spermatogenesis associated, serine-rich 2	0.74	0.72	1	0.55
PI4KB	PI4KB;DKFZp686O1820;FLJ30129;NPIK;PI4K-BETA;PI4K92;PI4KBETA;PI4KIIIBETA;PIK4CB	phosphatidylinositol 4-kinase, catalytic, beta	0.74	0.74	1	0.62
ATP6V1D	ATP6V1D;ATP6M;VATD;VMA8	ATPase, H+ transporting, lysosomal 34kDa, V1 subunit D	0.74	0.8	1	0.59
DUX1	DUX1	double homeobox 1	0.74	0.74	1	0.5
OSTC	OSTC	oligosaccharyltransferase complex subunit	0.74	0.71	1	0.57
CAPN3	CAPN3;CANP3;CANPL3;LGMD2;LGMD2A;MGC10767;MGC11121;MGC14344;MGC4403;nCL-1;p94	calpain 3, (p94)	0.73	0.72	1	0.54
MOB3B	MOB3B;FLJ13204;FLJ23916;MGC32960;MOB1D;MOBKL2B	MOB kinase activator 3B	0.73	0.76	1	0.61
MORN5	MORN5;C9orf113;C9orf18;FLJ46909;FLJ50034	MORN repeat containing 5	0.72	0.74	1	0.54
FAM135B	FAM135B;C8ORFK32;MGC126009;MGC126010;MGC33221	family with sequence similarity 135, member B	0.72	0.73	1	0.55
CERKL	CERKL;RP26	ceramide kinase-like	0.72	0.77	1	0.54
GRIP1	GRIP1;GRIP	glutamate receptor interacting protein 1	0.72	0.7	1	0.55
CPZ	CPZ;MGC99682	carboxypeptidase Z	0.72	0.71	1	0.53
ZNF71	ZNF71;EZFIT	zinc finger protein 71	0.72	0.74	1	0.5
FASTKD3	FASTKD3;FLJ23274;MGC142123;MGC5297	FAST kinase domains 3	0.72	0.74	1	0.53
RSAD1	RSAD1;FLJ11164;FLJ20975	radical S-adenosyl methionine domain containing 1	0.72	0.64	1	0.5
TJAP1	TJAP1;DKFZp686F06131;PILT;TJP4	tight junction associated protein 1 (peripheral)	0.72	0.7	1	0.53
ZDHHC17	ZDHHC17;HIP14;HIP3;HYPH;KIAA0946	zinc finger, DHHC-type containing 17	0.72	0.77	1	0.59
CUL7	CUL7;KIAA0076;dJ20C7.5	cullin 7	0.72	0.66	1	0.5
NOL6	NOL6;FLJ21959;MGC14896;MGC14921;MGC20838;NRAP;UTP22;bA311H10.1	nucleolar protein family 6 (RNA-associated)	0.72	0.74	1	0.49
C7orf59	C7orf59;MGC163425;MGC163431	chromosome 7 open reading frame 59	0.72	0.7	1	0.5
RBM34	RBM34;KIAA0117	RNA binding motif protein 34	0.72	0.78	1	0.6
CD59	CD59;16.3A5;1F5;EJ16;EJ30;EL32;FLJ38134;FLJ92039;G344;HRF-20;HRF20;MAC-IP;MACIF;MEM43;MGC2354;MIC11;MIN1;MIN2;MIN3;MIRL;MSK21;p18-20	CD59 molecule, complement regulatory protein	0.72	0.7	1	0.58
RMND5A	RMND5A;CTLH;FLJ12753;FLJ13910;FLJ21795;MGC78451;RMD5;p44CTLH	required for meiotic nuclear division 5 homolog A (S. cerevisiae)	0.72	0.75	1	0.54
FNBP1L	FNBP1L;C1orf39;TOCA1	formin binding protein 1-like	0.72	0.72	1	0.52
ALDOC	ALDOC;ALDC	aldolase C, fructose-bisphosphate	0.71	0.7	1	0.54
GCM2	GCM2;GCMB;hGCMb	glial cells missing homolog 2 (Drosophila)	0.7	0.7	1	0.52
CTRC	CTRC;CLCR;ELA4	chymotrypsin C (caldecrin)	0.7	0.7	1	0.52
RHBDL3	RHBDL3;FLJ45582;MGC119300;MGC119301;RHBDL4;VRHO	rhomboid, veinlet-like 3 (Drosophila)	0.7	0.7	1	0.52
TMEM60	TMEM60;C7orf35;MGC74482	transmembrane protein 60	0.7	0.74	1	0.57
SAMD12	SAMD12;FLJ39458;MGC148139;MGC148140	sterile alpha motif domain containing 12	0.7	0.76	1	0.51
MRPL43	MRPL43;MGC17989;MGC48892;bMRP36a	mitochondrial ribosomal protein L43	0.7	0.66	1	0.52
DERL3	DERL3;C22orf14;IZP6;LLN2;MGC71803;derlin-3	Der1-like domain family, member 3	0.7	0.73	1	0.52
N4BP3	N4BP3;KIAA0341	NEDD4 binding protein 3	0.7	0.77	1	0.56
FMNL2	FMNL2;FHOD2;FLJ37546	formin-like 2	0.7	0.76	1	0.56
PGAP3	PGAP3;AGLA546;CAB2;MGC9753;PER1;PERLD1;PP1498	post-GPI attachment to proteins 3	0.7	0.7	1	0.48
C19orf80	C19orf80;PRO1185;PVPA599;TD26	chromosome 19 open reading frame 80	0.7	0.68	1	0.53
CATSPERB	CATSPERB;C14orf161;CatSper(beta);FLJ14298	cation channel, sperm-associated, beta	0.69	0.72	1	0.51
EIF2C4	EIF2C4;AGO4	eukaryotic translation initiation factor 2C, 4	0.69	0.68	1	0.47
GOLGA7B	GOLGA7B;C10orf132;C10orf133;FLJ43465;MGC131701;bA451M19.3;bA459F3.4	golgin A7 family, member B	0.69	0.69	1	0.5
JAM3	JAM3;FLJ14529;JAM-C;JAMC	junctional adhesion molecule 3	0.68	0.62	1	0.51
ABCC3	ABCC3;ABC31;DKFZp686E22157;EST90757;MLP2;MOAT-D;MRP3;cMOAT2	ATP-binding cassette, sub-family C (CFTR/MRP), member 3	0.68	0.69	1	0.54
ABR	ABR;FLJ45954;MDB	active BCR-related gene	0.68	0.72	1	0.51
ZBTB4	ZBTB4;KAISO-L1;KIAA1538;ZNF903	zinc finger and BTB domain containing 4	0.68	0.72	1	0.51
CBWD3	CBWD3;FLJ60409;bA561O23.1	COBW domain containing 3	0.68	0.71	1	0.47
ADD3	ADD3;ADDL	adducin 3 (gamma)	0.68	0.7	1	0.53
DNMT1	DNMT1;AIM;CXXC9;DNMT;FLJ16293;HSN1E;MCMT;MGC104992	DNA (cytosine-5-)-methyltransferase 1	0.68	0.72	1	0.47
PTDSS1	PTDSS1;KIAA0024;PSS1;PSSA	phosphatidylserine synthase 1	0.68	0.73	1	0.54
KIFC2	KIFC2	kinesin family member C2	0.67	0.69	1	0.55
TMEM115	TMEM115;PL6	transmembrane protein 115	0.67	0.64	1	0.52
SLC24A2	SLC24A2;FLJ45149;NCKX2	solute carrier family 24 (sodium/potassium/calcium exchanger), member 2	0.66	0.7	1	0.53
FAM131C	FAM131C;C1orf117;FLJ36766;RP11-5P18.9	family with sequence similarity 131, member C	0.66	0.72	1	0.55
ITPKC	ITPKC;IP3-3KC;IP3KC	inositol-trisphosphate 3-kinase C	0.66	0.66	1	0.54
YOD1	YOD1;DKFZp451J1719;DUBA8;OTUD2;PRO0907	YOD1 OTU deubiquinating enzyme 1 homolog (S. cerevisiae)	0.66	0.68	1	0.48
ZMIZ1	ZMIZ1;FLJ13541;KIAA1224;MIZ;RAI17;TRAFIP10;ZIMP10;hZIMP10	zinc finger, MIZ-type containing 1	0.66	0.62	1	0.5
ZNF786	ZNF786;DKFZp762I137;MGC156120	zinc finger protein 786	0.66	0.7	1	0.5
SMAD1	SMAD1;BSP-1;BSP1;JV4-1;JV41;MADH1;MADR1	SMAD family member 1	0.66	0.73	1	0.51
GALNT12	GALNT12;CRCS1;FLJ21212;GalNAc-T12	UDP-N-acetyl-alpha-D-galactosamine:polypeptide N-acetylgalactosaminyltransferase 12 (GalNAc-T12)	0.66	0.64	1	0.47
SNAPC1	SNAPC1;PTFgamma;SNAP43	small nuclear RNA activating complex, polypeptide 1, 43kDa	0.66	0.68	1	0.49
SPANXB2	SPANXB2	ENTREZ STATUS: REMAPPED TO GENE ID: 100133171	0.66	0.6	1	0.5
SLIRP	SLIRP;C14orf156;DC50;PD04872	SRA stem-loop interacting RNA binding protein	0.66	0.66	1	0.45
HIST1H2BC	HIST1H2BC;H2B.1;H2B/a;H2B/g;H2B/h;H2B/k;H2B/l;H2BFL;MGC104246;dJ221C16.3	histone cluster 1, H2bc	0.65	0.67	1	0.45
BPIFA3	BPIFA3;C20orf71;MGC44525;SPLUNC3	BPI fold containing family A, member 3	0.65	0.63	1	0.51
ACSM2A	ACSM2A;A-923A4.1;ACSM2;FLJ34659;MGC150530	acyl-CoA synthetase medium-chain family member 2A	0.64	0.62	1	0.49
MAB21L1	MAB21L1;CAGR1;FLJ10197	mab-21-like 1 (C. elegans)	0.64	0.57	1	0.47
ELOVL4	ELOVL4;ADMD;CT118;FLJ17667;FLJ92876;STGD2;STGD3	ELOVL fatty acid elongase 4	0.64	0.7	1	0.52
ZFP112	ZFP112;ZNF228	zinc finger protein 112 homolog (mouse)	0.64	0.66	1	0.5
IL22RA1	IL22RA1;CRF2-9;IL22R;IL22R1	interleukin 22 receptor, alpha 1	0.64	0.69	1	0.52
IRX6	IRX6;IRX-3;IRX7;IRXB3	iroquois homeobox 6	0.64	0.67	1	0.54
ACBD4	ACBD4;FLJ13322;FLJ90623	acyl-CoA binding domain containing 4	0.64	0.62	1	0.52
APOL6	APOL6;APOL-VI;APOLVI;DKFZp667M075;FLJ38562;FLJ90164;MGC57495	apolipoprotein L, 6	0.64	0.7	1	0.52
EMG1	EMG1;C2F;FLJ60792;Grcc2f;NEP1	EMG1 nucleolar protein homolog (S. cerevisiae)	0.64	0.64	1	0.46
MINK1	MINK1;B55;MAP4K6;MGC21111;MINK;YSK2;ZC3;hMINK;hMINKbeta	misshapen-like kinase 1	0.64	0.64	1	0.53
TMC4	TMC4;MGC39329	transmembrane channel-like 4	0.64	0.65	1	0.5
G6PD	G6PD;G6PD1	glucose-6-phosphate dehydrogenase	0.64	0.68	1	0.52
ULBP2	ULBP2;N2DL2;RAET1H	UL16 binding protein 2	0.63	0.66	1	0.49
TMCO2	TMCO2;MGC131705;dJ39G22.2	transmembrane and coiled-coil domains 2	0.62	0.66	1	0.47
PLA2G1B	PLA2G1B;MGC119834;MGC119835;PLA2;PLA2A;PPLA2	phospholipase A2, group IB (pancreas)	0.62	0.59	1	0.43
TBX5	TBX5;HOS	T-box 5	0.62	0.69	1	0.48
HDAC4	HDAC4;AHO3;BDMR;HA6116;HD4;HDAC-4;HDAC-A;HDACA;KIAA0288	histone deacetylase 4	0.62	0.58	1	0.46
TBX10	TBX10;TBX13;TBX7	T-box 10	0.62	0.66	1	0.53
VWC2	VWC2;MGC131845;PSST739;UNQ739	von Willebrand factor C domain containing 2	0.62	0.6	1	0.45
JUB	JUB;Ajuba;MGC15563	jub, ajuba homolog (Xenopus laevis)	0.62	0.62	1	0.45
HSPB3	HSPB3;DHMN2C;HMN2C;HSPL27	heat shock 27kDa protein 3	0.61	0.61	1	0.41
IQCJ	IQCJ	IQ motif containing J	0.84	0.82	1.01	0.62
ZNF474	ZNF474;FLJ32921	zinc finger protein 474	0.84	0.82	1.01	0.63
SYT14L	SYT14L;CHR415SYT;SYTDEP	synaptotagmin XIV-like	0.84	0.82	1.01	0.62
ITGAX	ITGAX;CD11C;SLEB6	integrin, alpha X (complement component 3 receptor 4 subunit)	0.84	0.86	1.01	0.67
RIMBP2	RIMBP2;KIAA0318;MGC15831;RBP2;RIM-BP2	RIMS binding protein 2	0.84	0.8	1.01	0.65
AQP8	AQP8;AQP-8	aquaporin 8	0.84	0.9	1.01	0.7
PBX4	PBX4	pre-B-cell leukemia homeobox 4	0.84	0.82	1.01	0.61
FMNL3	FMNL3;DKFZp762B245;FHOD3;FLJ45265;MGC45819;WBP-3;WBP3	formin-like 3	0.84	0.76	1.01	0.61
ACSF2	ACSF2;ACSMW;AVYV493;FLJ20920	acyl-CoA synthetase family member 2	0.84	0.8	1.01	0.63
UBE2C	UBE2C;UBCH10;dJ447F3.2	ubiquitin-conjugating enzyme E2C	0.84	0.78	1.01	0.59
PRCD	PRCD;RP36	progressive rod-cone degeneration	0.84	0.88	1.01	0.66
MYBPHL	MYBPHL	myosin binding protein H-like	0.83	0.86	1.01	0.64
HSPB7	HSPB7;DKFZp779D0968;FLJ32733;cvHSP	heat shock 27kDa protein family, member 7 (cardiovascular)	0.83	0.82	1.01	0.69

TESK2	TESK2	testis-specific kinase 2	0.83	0.84	1.01	0.69
POLR3C	POLR3C;RPC3;RPC62	polymerase (RNA) III (DNA directed) polypeptide C (62kD)	0.83	0.84	1.01	0.59
PYROXD2	PYROXD2;C10orf33;FLJ12197;FLJ23849;FP3420;MGC13047	pyridine nucleotide-disulphide oxidoreductase domain 2	0.83	0.86	1.01	0.59
CNTN1	CNTN1;F3;GP135	contactin 1	0.82	0.82	1.01	0.61
CRISPLD1	CRISPLD1;CRISP10;DKFZp762F133;LCRISP1	cysteine-rich secretory protein LCCL domain containing 1	0.82	0.84	1.01	0.64
ELMO1	ELMO1;CED-12;CED12;ELMO-1;KIAA0281;MGC126406	engulfment and cell motility 1	0.82	0.88	1.01	0.7
CCDC87	CCDC87;FLJ10786	coiled-coil domain containing 87	0.82	0.86	1.01	0.61
LIM2	LIM2;MP17;MP19	lens intrinsic membrane protein 2, 19kDa	0.82	0.81	1.01	0.68
ETV2	ETV2;ER71;ETSRP71;MGC129834;MGC129835	ets variant 2	0.82	0.86	1.01	0.58
ADRB2	ADRB2;ADRB2R;ADRBR;B2AR;BAR;BETA2AR	adrenergic, beta-2-, receptor, surface	0.82	0.78	1.01	0.58
WDR38	WDR38	WD repeat domain 38	0.82	0.82	1.01	0.59
HERC6	HERC6;FLJ20637	hect domain and RLD 6	0.82	0.76	1.01	0.57
ZNF81	ZNF81;FLJ44367;HFZ20;MRX45;dJ54B20.6	zinc finger protein 81	0.82	0.75	1.01	0.6
FBXL4	FBXL4;FBL4;FBL5	F-box and leucine-rich repeat protein 4	0.82	0.79	1.01	0.57
AMN1	AMN1	antagonist of mitotic exit network 1 homolog (S. cerevisiae)	0.82	0.82	1.01	0.59
CAB39	CAB39;FLJ22682;MO25	calcium binding protein 39	0.82	0.87	1.01	0.62
MKLN1	MKLN1;FLJ11162;TWA2	muskelin 1, intracellular mediator containing kelch motifs	0.82	0.8	1.01	0.63
GTPBP4	GTPBP4;CRFG;FLJ10686;FLJ10690;FLJ39774;NGB;NOG1	GTP binding protein 4	0.82	0.84	1.01	0.58
H2AFY	H2AFY;H2A.y;H2A/y;H2AF12M;H2AFJ;MACROH2A1.1;mH2A1;macroH2A1.2	H2A histone family, member Y	0.82	0.8	1.01	0.67
FGD5	FGD5;ZFYVE23	FYVE, RhoGEF and PH domain containing 5	0.81	0.8	1.01	0.57
MGAT5B	MGAT5B;FLJ25132;GnT-IX;GnT-VB;KIAA2008	mannosyl (alpha-1,6-)-glycoprotein beta-1,6-N-acetyl-glucosaminyltransferase, isozyme B	0.81	0.74	1.01	0.59
PNPLA6	PNPLA6;NTE;NTEMND;SPG39;sws	patatin-like phospholipase domain containing 6	0.81	0.9	1.01	0.62
TSFM	TSFM;EFTS;EFTSMT	Ts translation elongation factor, mitochondrial	0.81	0.82	1.01	0.55
C11orf51	C11orf51;DKFZp564M082	chromosome 11 open reading frame 51	0.81	0.87	1.01	0.67
FGF16	FGF16;FGF-16	fibroblast growth factor 16	0.8	0.78	1.01	0.58
TMEM108	TMEM108;CT124;MGC3040	transmembrane protein 108	0.8	0.88	1.01	0.59
FAM83G	FAM83G;FLJ41564	family with sequence similarity 83, member G	0.8	0.77	1.01	0.65
CCL3L1	CCL3L1;464.2;D17S1718;G0S19-2;LD78;LD78BETA;MGC104178;MGC12815;MGC182017;MIP1AP;SCYA3L;SCYA3L1	chemokine (C-C motif) ligand 3-like 1	0.8	0.79	1.01	0.63
TMEM120A	TMEM120A;NET29;TMPIT	transmembrane protein 120A	0.8	0.85	1.01	0.6
TXN2	TXN2;MT-TRX;MTRX;TRX2	thioredoxin 2	0.8	0.87	1.01	0.65
SCLT1	SCLT1;CAP-1A;CAP1A;FLJ30655;FLJ36042;MGC70542;hCAP-1A	sodium channel and clathrin linker 1	0.8	0.84	1.01	0.62
HIST1H1C	HIST1H1C;H1.2;H1C;H1F2;MGC3992	histone cluster 1, H1c	0.8	0.82	1.01	0.63
LSM7	LSM7;YNL147W	LSM7 homolog, U6 small nuclear RNA associated (S. cerevisiae)	0.8	0.83	1.01	0.56
MYO1E	MYO1E;FSGS6;HuncM-IC;MGC104638;MYO1C	myosin IE	0.8	0.81	1.01	0.63
MTRR	MTRR;MGC129643;MSR;cblE	5-methyltetrahydrofolate-homocysteine methyltransferase reductase	0.8	0.86	1.01	0.65
ORMDL2	ORMDL2;MST095;MSTP095;adoplin-2	ORM1-like 2 (S. cerevisiae)	0.8	0.9	1.01	0.66
PPP2R5D	PPP2R5D;B56D;MGC2134;MGC8949	protein phosphatase 2, regulatory subunit B', delta	0.8	0.74	1.01	0.56
RANGAP1	RANGAP1;Fug1;KIAA1835;MGC20266;SD	Ran GTPase activating protein 1	0.8	0.78	1.01	0.63
GNL3	GNL3;C77032;E2IG3;MGC800;NNP47;NS	guanine nucleotide binding protein-like 3 (nucleolar)	0.8	0.86	1.01	0.6
TRMT61A	TRMT61A;C14orf172;FLJ40452;GCD14;Gcd14p;TRM61;hTRM61	tRNA methyltransferase 61 homolog A (S. cerevisiae)	0.8	0.79	1.01	0.55
ARHGEF37	ARHGEF37;FLJ41603	Rho guanine nucleotide exchange factor (GEF) 37	0.8	0.81	1.01	0.55
ECI2	ECI2;ACBD2;DRS-1;DRS1;HCA88;KIAA0536;PECI;dJ1013A10.3	enoyl-CoA delta isomerase 2	0.8	0.76	1.01	0.6
CCDC47	CCDC47;MSTP041	coiled-coil domain containing 47	0.79	0.79	1.01	0.57
SIAH3	SIAH3;FLJ39203	seven in absentia homolog 3 (Drosophila)	0.79	0.84	1.01	0.68
ZFC3H1	ZFC3H1;CCDC131;DKFZp686A0722;KIAA0546;MGC23401;MGC90200;PSRC2	zinc finger, C3H1-type containing	0.79	0.8	1.01	0.57
N4BP2L1	N4BP2L1;CG018	NEDD4 binding protein 2-like 1	0.78	0.82	1.01	0.54
TP63	TP63;AIS;B(p51A);B(p51B);EEC3;KET;LMS;NBP;OFC8;RHS;SHFM4;TP53CP;TP53L;TP73L;p40;p51;p53CP;p63;p73H;p73L	tumor protein p63	0.78	0.85	1.01	0.63
PCDH8	PCDH8;ARCADLIN;PAPC	protocadherin 8	0.78	0.78	1.01	0.61
KRTAP12-1	KRTAP12-1;KAP12.1;KRTAP12.1	keratin associated protein 12-1	0.78	0.76	1.01	0.55
HLA-DMB	HLA-DMB;D6S221E;RING7	major histocompatibility complex, class II, DM beta	0.78	0.77	1.01	0.57
EFHC1	EFHC1;FLJ10466;FLJ37290;dJ304B14.2	EF-hand domain (C-terminal) containing 1	0.78	0.77	1.01	0.64
WDR53	WDR53;MGC12928;MGC64882	WD repeat domain 53	0.78	0.8	1.01	0.6
MED11	MED11;MGC88387	mediator complex subunit 11	0.78	0.75	1.01	0.61
LACTB	LACTB;FLJ14902;G24;MRPL56	lactamase, beta	0.78	0.82	1.01	0.59
CDCA7L	CDCA7L;DKFZp762L0311;JPO2;R1;RAM2	cell division cycle associated 7-like	0.78	0.82	1.01	0.58
PPP1R36	PPP1R36;C14orf50	protein phosphatase 1, regulatory subunit 36	0.78	0.82	1.01	0.55
POC5	POC5;C5orf37;FLJ35779;MGC120442;MGC120443;MGC120444	POC5 centriolar protein homolog (Chlamydomonas)	0.78	0.77	1.01	0.58
CEP128	CEP128;C14orf145;C14orf61	centrosomal protein 128kDa	0.78	0.77	1.01	0.62
MMP28	MMP28;EPILYSIN;MM28;MMP-28;MMP25	matrix metallopeptidase 28	0.77	0.76	1.01	0.58
EFNA3	EFNA3;EFL2;EPLG3;Ehk1-L;LERK3	ephrin-A3	0.77	0.79	1.01	0.65
CDC42	CDC42;CDC42Hs;G25K	cell division cycle 42 (GTP binding protein, 25kDa)	0.77	0.82	1.01	0.64
FAHD2B	FAHD2B;DKFZp434N062	fumarylacetoacetate hydrolase domain containing 2B	0.77	0.76	1.01	0.57
ZNFX1	ZNFX1;FLJ39275;MGC131926	zinc finger, NFX1-type containing 1	0.77	0.76	1.01	0.56
SETD7	SETD7;FLJ21193;KIAA1717;KMT7;SET7;SET7/9;SET9	SET domain containing (lysine methyltransferase) 7	0.77	0.74	1.01	0.58
PRIM1	PRIM1;MGC12308;p49	primase, DNA, polypeptide 1 (49kDa)	0.77	0.74	1.01	0.58
MYO16	MYO16;KIAA0865;MYR8;Myo16b	myosin XVI	0.76	0.72	1.01	0.58
SLC47A1	SLC47A1;FLJ10847;MATE1;MGC64822	solute carrier family 47, member 1	0.76	0.82	1.01	0.63
CCBE1	CCBE1;FLJ30681;MGC50861	collagen and calcium binding EGF domains 1	0.76	0.7	1.01	0.54
REXO1L1	REXO1L1;GOR	REX1, RNA exonuclease 1 homolog (S. cerevisiae)-like 1	0.76	0.81	1.01	0.54
METTL8	METTL8;FLJ13334;FLJ13984;FLJ31054;FLJ42098;FLJ77788;TIP	methyltransferase like 8	0.76	0.72	1.01	0.57
OAF	OAF;MGC52117;NS5ATP13TP2	OAF homolog (Drosophila)	0.76	0.74	1.01	0.57
TMEM132A	TMEM132A;DKFZp547E212;FLJ20539;GBP;HSPA5BP1;MGC138669	transmembrane protein 132A	0.76	0.77	1.01	0.56
DOT1L	DOT1L;DKFZp586P1823;DOT1;KIAA1814;KMT4	DOT1-like, histone H3 methyltransferase (S. cerevisiae)	0.76	0.72	1.01	0.54
TMEM168	TMEM168;DKFZp564C012;FLJ13576	transmembrane protein 168	0.76	0.81	1.01	0.58
CDKN2A	CDKN2A;ARF;CDK4I;CDKN2;CMM2;INK4;INK4A;MLM;MTS-1;MTS1;P14;P14ARF;P16;P16-INK4A;P16INK4;P16INK4A;P19;P19ARF;TP16	cyclin-dependent kinase inhibitor 2A (melanoma, p16, inhibits CDK4)	0.76	0.72	1.01	0.57
EIF3H	EIF3H;EIF3S3;MGC102958;eIF3-gamma;eIF3-p40	eukaryotic translation initiation factor 3, subunit H	0.76	0.84	1.01	0.58
CABS1	CABS1;C4orf35;CLPH;FLJ32897;NYD-SP26	calcium-binding protein, spermatid-specific 1	0.76	0.74	1.01	0.57
C22orf33	C22orf33;EAN57;MGC35206;cE81G9.2	chromosome 22 open reading frame 33	0.75	0.82	1.01	0.55
OR2L13	OR2L13;MGC40047;OR2L14	olfactory receptor, family 2, subfamily L, member 13	0.75	0.72	1.01	0.58
POTEA	POTEA;A26A1;CT104.3;POTE-8;POTE8	POTE ankyrin domain family, member A	0.75	0.8	1.01	0.54
ITGA10	ITGA10;PRO827	integrin, alpha 10	0.74	0.75	1.01	0.52
PRMT8	PRMT8;HRMT1L3;HRMT1L4	protein arginine methyltransferase 8	0.74	0.73	1.01	0.57
FAIM3	FAIM3;FCMR;TOSO	Fas apoptotic inhibitory molecule 3	0.74	0.78	1.01	0.56
NEUROG2	NEUROG2;Atoh4;MGC46562;Math4A;NGN2;bHLHa8;ngn-2	neurogenin 2	0.74	0.74	1.01	0.51
OR51E1	OR51E1;D-GPCR;FLJ13581;GPR136;GPR164;MGC24137;OR51E1P;OR52A3P;POGR;PSGR2	olfactory receptor, family 51, subfamily E, member 1	0.74	0.72	1.01	0.54
GPA33	GPA33;A33;MGC129986;MGC129987	glycoprotein A33 (transmembrane)	0.74	0.74	1.01	0.62
VEPH1	VEPH1;FLJ12604;KIAA1692;MELT;MGC111426;MGC126709;MGC142115;VEPH	ventricular zone expressed PH domain homolog 1 (zebrafish)	0.74	0.78	1.01	0.56
NCF1	NCF1;FLJ79451;NCF1A;NOXO2;SH3PXD1A;p47phox	neutrophil cytosolic factor 1	0.74	0.76	1.01	0.53
VPS13A	VPS13A;CHAC;CHOREIN;FLJ42030;KIAA0986	vacuolar protein sorting 13 homolog A (S. cerevisiae)	0.74	0.76	1.01	0.51
ACSS1	ACSS1;ACAS2L;AceCS2L;FLJ45659;MGC33843	acyl-CoA synthetase short-chain family member 1	0.74	0.76	1.01	0.6
NUDT2	NUDT2;APAH1;MGC10404	nudix (nucleoside diphosphate linked moiety X)-type motif 2	0.74	0.73	1.01	0.57
SDF4	SDF4;Cab45;SDF-4	stromal cell derived factor 4	0.74	0.72	1.01	0.55
SMC2	SMC2;CAP-E;CAPE;FLJ10093;SMC2L1;hCAP-E	structural maintenance of chromosomes 2	0.74	0.8	1.01	0.52
RBM47	RBM47;DKFZp686F02235;FLJ20273;FLJ21344;FLJ21643;NET18	RNA binding motif protein 47	0.74	0.77	1.01	0.53
MBOAT7	MBOAT7;BB1;FLJ41296;LENG4;LPIAT;LRC4;MBOA7;OACT7;hMBOA-7	membrane bound O-acyltransferase domain containing 7	0.74	0.74	1.01	0.52
SPDYE3	SPDYE3;MGC119294;MGC57359	speedy homolog E3 (Xenopus laevis)	0.74	0.72	1.01	0.54
THRSP	THRSP;LPGP1;Lpgp;MGC21659;S14;SPOT14	thyroid hormone responsive	0.73	0.7	1.01	0.59
CITED1	CITED1;MSG1	Cbp/p300-interacting transactivator, with Glu/Asp-rich carboxy-terminal domain, 1	0.73	0.74	1.01	0.58
KRT35	KRT35;HA5;Ha-5;KRTHA5;hHa5	keratin 35	0.73	0.73	1.01	0.54
IFNE	IFNE;IFN-E;IFNE1;IFNT1;MGC119018;MGC119020;PRO655	interferon, epsilon	0.73	0.75	1.01	0.57
CHRNE	CHRNE;ACHRE;CMS1D;CMS1E;CMS2A;FCCMS;SCCMS	cholinergic receptor, nicotinic, epsilon	0.72	0.72	1.01	0.56
TMEM88	TMEM88;FLJ20025;MGC71744	transmembrane protein 88	0.72	0.75	1.01	0.56
FLJ45422	FLJ45422	ENTREZ STATUS: REMAPPED TO GENE ID: 3139	0.72	0.78	1.01	0.54
COQ5	COQ5;MGC104303;MGC4767	coenzyme Q5 homolog, methyltransferase (S. cerevisiae)	0.72	0.77	1.01	0.51
FGFR1OP	FGFR1OP;FOP	FGFR1 oncogene partner	0.72	0.75	1.01	0.57
UTP14A	UTP14A;FLJ37089;KIAA0266;NYCO16;SDCCAG16;dJ537K23.3	UTP14, U3 small nucleolar ribonucleoprotein, homolog A (yeast)	0.72	0.76	1.01	0.51
SFTPA1	SFTPA1	ENTREZ STATUS: REMAPPED TO GENE ID: 653509	0.72	0.71	1.01	0.53
PPFIA2	PPFIA2;FLJ41378;MGC132572	protein tyrosine phosphatase, receptor type, f polypeptide (PTPRF), interacting protein (liprin), alpha 2	0.71	0.7	1.01	0.55
TSKS	TSKS;STK22S1;TSKS1;TSSKS	testis-specific serine kinase substrate	0.71	0.7	1.01	0.63
SARM1	SARM1;FLJ36296;KIAA0524;SAMD2;SARM	sterile alpha and TIR motif containing 1	0.71	0.68	1.01	0.57
CKAP2L	CKAP2L;FLJ40629;MGC39683	cytoskeleton associated protein 2-like	0.71	0.76	1.01	0.53
MAOA	MAOA	monoamine oxidase A	0.7	0.7	1.01	0.52
MFRP	MFRP;DKFZp586B0621;FLJ30570;MCOP5;MGC32938;NNO2;RD6	membrane frizzled-related protein	0.7	0.68	1.01	0.54
FGF12	FGF12;FGF12B;FHF1	fibroblast growth factor 12	0.7	0.74	1.01	0.49
LPIN3	LPIN3;LIPN3L;SMP2;dJ450M14.2;dJ450M14.3;dJ620E11.2	lipin 3	0.7	0.73	1.01	0.52
LRRC20	LRRC20;FLJ10751;FLJ10844	leucine rich repeat containing 20	0.7	0.73	1.01	0.5
KCNG2	KCNG2;KCNF2;KV6.2	potassium voltage-gated channel, subfamily G, member 2	0.7	0.68	1.01	0.54
SUV420H2	SUV420H2;FLJ51466;FLJ98627;KMT5C;MGC2705	suppressor of variegation 4-20 homolog 2 (Drosophila)	0.7	0.77	1.01	0.58
UTP20	UTP20;DRIM	UTP20, small subunit (SSU) processome component, homolog (yeast)	0.7	0.73	1.01	0.54
SOCS5	SOCS5;CIS6;CISH6;Cish5;KIAA0671;SOCS-5	suppressor of cytokine signaling 5	0.7	0.68	1.01	0.5
C1orf124	C1orf124;DDDL1880;DKFZp547N043;PRO4323;dJ876B10.3	chromosome 1 open reading frame 124	0.7	0.68	1.01	0.52
PNMA1	PNMA1;MA1	paraneoplastic antigen MA1	0.7	0.78	1.01	0.57
SHC1	SHC1;FLJ26504;SHC;SHCA	SHC (Src homology 2 domain containing) transforming protein 1	0.7	0.71	1.01	0.55
GPR89B	GPR89B;FLJ55546;GPHR;GPR89;SH120	G protein-coupled receptor 89B	0.7	0.78	1.01	0.54
ENGASE	ENGASE;DKFZp434P174;FLJ21865	endo-beta-N-acetylglucosaminidase	0.7	0.76	1.01	0.57
SFTPA2	SFTPA2	ENTREZ STATUS: DISCONTINUED	0.7	0.7	1.01	0.49
IMMP1L	IMMP1L;FLJ25059;IMP1;IMP1-LIKE	IMP1 inner mitochondrial membrane peptidase-like (S. cerevisiae)	0.69	0.7	1.01	0.51
SERF1A	SERF1A;4F5;FAM2A;H4F5;SERF1;SERF1B;SMAM1	small EDRK-rich factor 1A (telomeric)	0.69	0.7	1.01	0.48
RAB23	RAB23;DKFZp781H0695;HSPC137;MGC8900	RAB23, member RAS oncogene family	0.69	0.66	1.01	0.52

ZC4H2	ZC4H2;HCA127;KIAA1166	zinc finger, C4H2 domain containing	0.69	0.72	1.01	0.52
ALS2CR12	ALS2CR12	amyotrophic lateral sclerosis 2 (juvenile) chromosome region, candidate 12	0.68	0.7	1.01	0.54
MAP2K3	MAP2K3;MAPKK3;MEK3;MKK3;PRKMK3	mitogen-activated protein kinase kinase 3	0.68	0.68	1.01	0.57
FAM18A	FAM18A;YDR084C	family with sequence similarity 18, member A	0.67	0.69	1.01	0.53
MANBAL	MANBAL	mannosidase, beta A, lysosomal-like	0.67	0.74	1.01	0.54
NUFIP1	NUFIP1;NUFIP;bA540M5.1	nuclear fragile X mental retardation protein interacting protein 1	0.67	0.74	1.01	0.55
MUM1L1	MUM1L1;FLJ33516;MGC129994;MGC129995	melanoma associated antigen (mutated) 1-like 1	0.66	0.66	1.01	0.48
S100A5	S100A5;S100D	S100 calcium binding protein A5	0.66	0.69	1.01	0.46
ZNF234	ZNF234;HZF4;ZNF269	zinc finger protein 234	0.66	0.68	1.01	0.51
C2orf54	C2orf54;FLJ22671;MGC150431;MGC150432	chromosome 2 open reading frame 54	0.66	0.72	1.01	0.56
HOXB8	HOXB8;HOX2;HOX2D;Hox-2.4	homeobox B8	0.66	0.64	1.01	0.51
LRRC28	LRRC28;FLJ34269;FLJ45242;MGC24976	leucine rich repeat containing 28	0.66	0.7	1.01	0.47
LTBP4	LTBP4;FLJ46318;FLJ90018;LTBP-4;LTBP4L;LTBP4S	latent transforming growth factor beta binding protein 4	0.66	0.68	1.01	0.49
ANAPC4	ANAPC4;APC4	anaphase promoting complex subunit 4	0.66	0.64	1.01	0.5
WBP11	WBP11;DKFZp779M1063;NPWBP;SIPP1;WBP-11	WW domain binding protein 11	0.66	0.62	1.01	0.46
NFKBIA	NFKBIA;IKBA;MAD-3;NFKBI	nuclear factor of kappa light polypeptide gene enhancer in B-cells inhibitor, alpha	0.66	0.6	1.01	0.5
DCD	DCD;AIDD;DCD-1;DSEP;HCAP;MGC71930;PIF	dermcidin	0.64	0.66	1.01	0.46
DMRTC1	DMRTC1;DMRTC1B	DMRT-like family C1	0.64	0.68	1.01	0.51
FGD6	FGD6;ZFYVE24	FYVE, RhoGEF and PH domain containing 6	0.64	0.68	1.01	0.45
MFAP2	MFAP2;FLJ50901;MAGP;MAGP-1;MAGP1	microfibrillar-associated protein 2	0.64	0.64	1.01	0.48
GGA3	GGA3;KIAA0154	golgi-associated, gamma adaptin ear containing, ARF binding protein 3	0.64	0.66	1.01	0.51
DDHD2	DDHD2;KIAA0725;SAMWD1	DDHD domain containing 2	0.64	0.57	1.01	0.46
HDHD2	HDHD2;3110052N05Rik;DKFZp564D1378	haloacid dehalogenase-like hydrolase domain containing 2	0.64	0.64	1.01	0.5
C6orf89	C6orf89;FLJ25357;MGC126060;MGC126061;MGC126062	chromosome 6 open reading frame 89	0.64	0.68	1.01	0.46
IP6K3	IP6K3;IHPK3;INSP6K3;MGC102928	inositol hexakisphosphate kinase 3	0.64	0.7	1.01	0.5
MAP6D1	MAP6D1;FLJ12748;MAPO6D1;SL21	MAP6 domain containing 1	0.62	0.6	1.01	0.46
B3GNTL1	B3GNTL1;B3GNT8;MGC126253;MGC126256	UDP-GlcNAc:betaGal beta-1,3-N-acetylglucosaminyltransferase-like 1	0.62	0.58	1.01	0.47
PLEKHA5	PLEKHA5;FLJ10667;FLJ26734;FLJ31492;KIAA1686;PEPP-2;PEPP2	pleckstrin homology domain containing, family A member 5	0.62	0.62	1.01	0.46
IMPACT	IMPACT;MGC33718	Impact homolog (mouse)	0.62	0.61	1.01	0.48
C5orf30	C5orf30;FLJ25291	chromosome 5 open reading frame 30	0.62	0.68	1.01	0.45
SH3BGRL	SH3BGRL;MGC117402;SH3BGR	SH3 domain binding glutamic acid-rich protein like	0.62	0.6	1.01	0.43
CENPN	CENPN;BM039;C16orf60;CENP-N;FLJ13607;FLJ22660	centromere protein N	0.62	0.67	1.01	0.52
RPL7L1	RPL7L1;MGC62004;dJ475N16.4	ribosomal protein L7-like 1	0.62	0.6	1.01	0.46
SNRNP35	SNRNP35;HM-1;MGC138160;U1SNRNPBP	small nuclear ribonucleoprotein 35kDa (U11/U12)	0.62	0.6	1.01	0.47
DPT	DPT;TRAMP	dermatopontin	0.61	0.64	1.01	0.43
ARHGAP1	ARHGAP1;CDC42GAP;RHOGAP;RHOGAP1;p50rhoGAP	Rho GTPase activating protein 1	0.61	0.62	1.01	0.42
C7orf44	C7orf44;FLJ10803	chromosome 7 open reading frame 44	0.61	0.59	1.01	0.45
TXNDC12	TXNDC12;AG1;AGR1;ERP18;ERP19;PDIA16;TLP19;hAG-1	thioredoxin domain containing 12 (endoplasmic reticulum)	0.61	0.64	1.01	0.5
ITGB3	ITGB3;BDPLT2;CD61;GP3A;GPIIIa;GT	integrin, beta 3 (platelet glycoprotein IIIa, antigen CD61)	0.61	0.59	1.01	0.46
ITGAL	ITGAL;CD11A;LFA-1;LFA1A	integrin, alpha L (antigen CD11A (p180), lymphocyte function-associated antigen 1; alpha polypeptide)	0.84	0.84	1.02	0.61
DMC1	DMC1;DMC1H;HsLim15;LIM15;MGC150472;MGC150473;dJ199H16.1	DMC1 dosage suppressor of mck1 homolog, meiosis-specific homologous recombination (yeast)	0.84	0.91	1.02	0.69
ST6GALNAC3	ST6GALNAC3;FLJ13669;FLJ27239;PRO7177;SIAT7C;ST6GALNACIII;STY	ST6 (alpha-N-acetyl-neuraminyl-2,3-beta-galactosyl-1,3)-N-acetylgalactosaminide alpha-2,6-sialyltransferase 3	0.84	0.91	1.02	0.69
S100A3	S100A3;S100E	S100 calcium binding protein A3	0.84	0.93	1.02	0.7
RILPL1	RILPL1;FLJ39378;GOSPEL;MGC105128;MGC99793;RLP1	Rab interacting lysosomal protein-like 1	0.84	0.84	1.02	0.63
SMUG1	SMUG1;FDG;HMUDG;MGC104370;UNG3	single-strand-selective monofunctional uracil-DNA glycosylase 1	0.84	0.84	1.02	0.63
B3GALT6	B3GALT6;beta3GalT6	UDP-Gal:betaGal beta 1,3-galactosyltransferase polypeptide 6	0.84	0.86	1.02	0.64
TBC1D9	TBC1D9;KIAA0882;MDR1	TBC1 domain family, member 9 (with GRAM domain)	0.84	0.82	1.02	0.66
TMEM134	TMEM134;FLJ21749;MGC149891	transmembrane protein 134	0.84	0.84	1.02	0.63
MRPS11	MRPS11;FLJ22512;FLJ23406;HCC-2	mitochondrial ribosomal protein S11	0.84	0.94	1.02	0.69
MAP7D1	MAP7D1;FLJ10350;FLJ39022;MGC117315;PARCC1;RPRC1	MAP7 domain containing 1	0.84	0.94	1.02	0.62
AGAP1	AGAP1;AGAP-1;CENTG2;GGAP1;KIAA1099;MGC71657;cnt-g2	ArfGAP with GTPase domain, ankyrin repeat and PH domain 1	0.84	0.9	1.02	0.6
SSTR2	SSTR2	somatostatin receptor 2	0.83	0.82	1.02	0.74
RAMP1	RAMP1	receptor (G protein-coupled) activity modifying protein 1	0.83	0.82	1.02	0.66
CPSF4	CPSF4;CPSF30;NAR;NEB1	cleavage and polyadenylation specific factor 4, 30kDa	0.83	0.84	1.02	0.69
SSX1	SSX1;CT5.1;MGC150425;MGC5162;SSRC	synovial sarcoma, X breakpoint 1	0.82	0.88	1.02	0.59
PPIL6	PPIL6;MGC27054;MGC27056;MGC41939;RSPH12;bA425D10.6;dJ919F19.1	peptidylprolyl isomerase (cyclophilin)-like 6	0.82	0.79	1.02	0.62
CIITA	CIITA;C2TA;CIITAIV;MHC2TA;NLRA	class II, major histocompatibility complex, transactivator	0.82	0.77	1.02	0.68
KIAA1586	KIAA1586;FLJ30162	KIAA1586	0.82	0.86	1.02	0.58
WDR37	WDR37;FLJ40354;KIAA0982;RP11-529L18.2	WD repeat domain 37	0.82	0.8	1.02	0.62
SEMA3D	SEMA3D;MGC39708;Sema-Z2;coll-2	sema domain, immunoglobulin domain (Ig), short basic domain, secreted, (semaphorin) 3D	0.82	0.84	1.02	0.56
FAH	FAH;FLJ51912	fumarylacetoacetate hydrolase (fumarylacetoacetase)	0.82	0.83	1.02	0.56
LPPR2	LPPR2;DKFZp761E1121;FLJ13055;PRG4	lipid phosphate phosphatase-related protein type 2	0.82	0.82	1.02	0.6
PIK3R1	PIK3R1;GRB1;p85;p85-ALPHA	phosphoinositide-3-kinase, regulatory subunit 1 (alpha)	0.82	0.9	1.02	0.64
CST3	CST3;ARMD11;MGC117328	cystatin C	0.82	0.87	1.02	0.66
CLASP2	CLASP2	cytoplasmic linker associated protein 2	0.82	0.83	1.02	0.58
LAPTM4A	LAPTM4A;HUMORF13;KIAA0108;LAPTM4;MBNT;Mtrp	lysosomal protein transmembrane 4 alpha	0.82	0.78	1.02	0.66
CTU2	CTU2;C16orf84;MGC131681;MGC148063;MGC148064;NCS2;UPF0432	cytosolic thiouridylase subunit 2 homolog (S. pombe)	0.82	0.88	1.02	0.66
DSCR10	DSCR10	Down syndrome critical region gene 10 (non-protein coding)	0.81	0.8	1.02	0.57
FHL1	FHL1;FHL-1;FHL1A;FHL1B;FLH1A;KYOT;MGC111107;SLIM;SLIM-1;SLIM1;SLIMMER;XMPMA	four and a half LIM domains 1	0.81	0.91	1.02	0.66
FICD	FICD;HIP13;HYPE;MGC5623;UNQ3041	FIC domain containing	0.81	0.9	1.02	0.66
WDR25	WDR25;MGC4645	WD repeat domain 25	0.81	0.74	1.02	0.57
ARID3A	ARID3A;BRIGHT;DRIL1;DRIL3;E2FBP1	AT rich interactive domain 3A (BRIGHT-like)	0.81	0.79	1.02	0.57
TAOK3	TAOK3;DKFZp666H245;DPK;FLJ31808;JIK;MAP3K18	TAO kinase 3	0.81	0.88	1.02	0.63
FAM75A7	FAM75A7;AEP1;C9orf36A	family with sequence similarity 75, member A7	0.8	0.77	1.02	0.62
PAQR5	PAQR5;FLJ20190;MPRG	progestin and adipoQ receptor family member V	0.8	0.78	1.02	0.59
RBPJL	RBPJL;RBP-L;RBPSUHL;SUH;SUHL	recombination signal binding protein for immunoglobulin kappa J region-like	0.8	0.87	1.02	0.57
ACTL7B	ACTL7B;Tact1	actin-like 7B	0.8	0.78	1.02	0.57
KNTC1	KNTC1;FLJ36151;KIAA0166;ROD	kinetochore associated 1	0.8	0.78	1.02	0.6
BUD13	BUD13;FLJ27090;MGC13125;fSAP71	BUD13 homolog (S. cerevisiae)	0.8	0.86	1.02	0.61
RAB25	RAB25;CATX-8;RAB11C	RAB25, member RAS oncogene family	0.8	0.76	1.02	0.67
OCM2	OCM2;OCM	oncomodulin 2	0.8	0.86	1.02	0.64
SDR39U1	SDR39U1;C14orf124;HCDI	short chain dehydrogenase/reductase family 39U, member 1	0.8	0.78	1.02	0.6
SGK196	SGK196;FLJ23356;MGC126597	protein kinase-like protein SgK196	0.8	0.82	1.02	0.68
CLEC3A	CLEC3A;CLECSF1;MGC129558;MGC129559	C-type lectin domain family 3, member A	0.79	0.77	1.02	0.59
TBC1D4	TBC1D4;AS160;DKFZp779C0666	TBC1 domain family, member 4	0.79	0.78	1.02	0.59
USP12	USP12;UBH1;USP12L1	ubiquitin specific peptidase 12	0.79	0.8	1.02	0.6
CNRIP1	CNRIP1;C2orf32;CRIP1;DKFZp566K1924	cannabinoid receptor interacting protein 1	0.78	0.84	1.02	0.68
ZNF416	ZNF416;FLJ20557	zinc finger protein 416	0.78	0.85	1.02	0.6
PRR5	PRR5;FLJ20185;FLJ20185k;PP610;PROTOR-1;PROTOR1	proline rich 5 (renal)	0.78	0.8	1.02	0.55
TBK1	TBK1;FLJ11330;NAK;T2K	TANK-binding kinase 1	0.78	0.87	1.02	0.62
IGFBP5	IGFBP5;IBP5	insulin-like growth factor binding protein 5	0.78	0.82	1.02	0.55
WDR16	WDR16;FLJ37528;WDRPUH	WD repeat domain 16	0.77	0.83	1.02	0.58
LIPM	LIPM;LIPL3;bA304I5.1	lipase, family member M	0.77	0.77	1.02	0.6
TNP1	TNP1;TP1	transition protein 1 (during histone to protamine replacement)	0.77	0.76	1.02	0.6
MAX	MAX;MGC10775;MGC11225;MGC18164;MGC34679;MGC36767;bHLHd4;bHLHd5;bHLHd6;bHLHd7;bHLHd8;orf1	MYC associated factor X	0.77	0.79	1.02	0.53
KIF20B	KIF20B;CT90;DKFZp434B0435;DKFZp434P0810;KRMP1;MPHOSPH1;MPP-1;MPP1	kinesin family member 20B	0.77	0.78	1.02	0.58
FRK	FRK;GTK;PTK5;RAK	fyn-related kinase	0.77	0.85	1.02	0.6
SAMD4A	SAMD4A;DKFZp434H0350;DKFZp686A1532;KIAA1053;SAMD4;SMAUG;SMAUG1;SMG;SMGA	sterile alpha motif domain containing 4A	0.77	0.77	1.02	0.6
OTUD6B	OTUD6B;DUBA5	OTU domain containing 6B	0.77	0.8	1.02	0.54
TPRG1	TPRG1;FAM79B;FLJ41238;FLJ43694;MGC126599;MGC126601	tumor protein p63 regulated 1	0.76	0.8	1.02	0.58
FAM149A	FAM149A;DKFZp564J102;MSTP119	family with sequence similarity 149, member A	0.76	0.8	1.02	0.6
GPRASP1	GPRASP1;GASP;GASP-1;GASP1;KIAA0443	G protein-coupled receptor associated sorting protein 1	0.76	0.74	1.02	0.55
CKM	CKM;CKMM;M-CK	creatine kinase, muscle	0.76	0.76	1.02	0.68
BTC	BTC	betacellulin	0.76	0.86	1.02	0.63
ZNF155	ZNF155;MGC161655;pHZ-96	zinc finger protein 155	0.76	0.8	1.02	0.53
GATA4	GATA4;ASD2;MGC126629	GATA binding protein 4	0.76	0.75	1.02	0.65
SECTM1	SECTM1;K12	secreted and transmembrane 1	0.76	0.74	1.02	0.54
KCNH2	KCNH2;ERG1;HERG;HERG1;Kv11.1;LQT2;SQT1	potassium voltage-gated channel, subfamily H (eag-related), member 2	0.76	0.78	1.02	0.61
FPGS	FPGS	folylpolyglutamate synthase	0.76	0.8	1.02	0.63
SLC27A3	SLC27A3;ACSVL3;FATP3;MGC4365;VLCS-3	solute carrier family 27 (fatty acid transporter), member 3	0.76	0.72	1.02	0.57
KLC2	KLC2;FLJ12387	kinesin light chain 2	0.76	0.76	1.02	0.57
MLH1	MLH1;COCA2;FCC2;HNPCC;HNPCC2;MGC5172;hMLH1	mutL homolog 1, colon cancer, nonpolyposis type 2 (E. coli)	0.76	0.74	1.02	0.58
PNO1	PNO1;KHRBP1;RRP20	partner of NOB1 homolog (S. cerevisiae)	0.76	0.81	1.02	0.58
TSN	TSN;BCLF-1;RCHF1;REHF-1;TBRBP;TRSLN	translin	0.76	0.82	1.02	0.58
CAPNS1	CAPNS1;30K;CALPAIN4;CANP;CANPS;CAPN4;CDPS;CSS1	calpain, small subunit 1	0.76	0.77	1.02	0.58
MDH2	MDH2;M-MDH;MDH;MGC:3559;MOR1	malate dehydrogenase 2, NAD (mitochondrial)	0.76	0.78	1.02	0.61
IZUMO2	IZUMO2;C19orf41;MGC33947;PLAL6978;PRO21961;SCRL	IZUMO family member 2	0.76	0.8	1.02	0.6
OR52H1	OR52H1;OR11-41;OR11-45	olfactory receptor, family 52, subfamily H, member 1	0.75	0.78	1.02	0.6
C1orf189	C1orf189	chromosome 1 open reading frame 189	0.75	0.76	1.02	0.55
GSG1L	GSG1L;MGC18079;PRO19651	GSG1-like	0.75	0.74	1.02	0.56
NFKBID	NFKBID;IkappaBNS;MGC11314;MGC149503;TA-NFKBH	nuclear factor of kappa light polypeptide gene enhancer in B-cells inhibitor, delta	0.75	0.8	1.02	0.6
PTCH1	PTCH1;BCNS;FLJ26746;FLJ42602;HPE7;NBCCS;PTC;PTC1;PTCH;PTCH11	patched 1	0.75	0.84	1.02	0.58
TMEM139	TMEM139;FLJ90586	transmembrane protein 139	0.75	0.8	1.02	0.57
CD151	CD151;GP27;MER2;PETA-3;RAPH;SFA1;TSPAN24	CD151 molecule (Raph blood group)	0.75	0.75	1.02	0.6
KDM5A	KDM5A;JARID1A;RBBP-2;RBBP2;RBP2	lysine (K)-specific demethylase 5A	0.75	0.76	1.02	0.53
SSX7	SSX7	synovial sarcoma, X breakpoint 7	0.74	0.78	1.02	0.59
C18orf62	C18orf62;MGC126049	chromosome 18 open reading frame 62	0.74	0.82	1.02	0.6
HSPB9	HSPB9;CT51;FLJ27437	heat shock protein, alpha-crystallin-related, B9	0.74	0.78	1.02	0.6
MAP6	MAP6;FLJ41346;KIAA1878;MTAP6;N-STOP;STOP	microtubule-associated protein 6	0.74	0.78	1.02	0.62

BMP3	BMP3;BMP-3A	bone morphogenetic protein 3	0.74	0.7	1.02	0.56
FAM90A1	FAM90A1;FLJ10408	family with sequence similarity 90, member A1	0.74	0.72	1.02	0.58
KRTAP12-3	KRTAP12-3;KRTAP12.3	keratin associated protein 12-3	0.74	0.8	1.02	0.64
ETV6	ETV6;TEL;TEL/ABL	ets variant 6	0.74	0.7	1.02	0.56
BTBD7	BTBD7;DKFZp686N0544;FUP1;MGC48310	BTB (POZ) domain containing 7	0.74	0.68	1.02	0.57
GON4L	GON4L;DKFZp761I241;FLJ12923;FLJ20203;FLJ23040;GON-4;GON4;KIAA1606;MGC138817;MGC138818;RP11-243J18.4	gon-4-like (C. elegans)	0.74	0.72	1.02	0.58
OSGIN2	OSGIN2;C8orf1;hT41	oxidative stress induced growth inhibitor family member 2	0.74	0.77	1.02	0.57
TRIM24	TRIM24;PTC6;RNF82;TF1A;TIF1;TIF1A;TIF1ALPHA;hTIF1	tripartite motif containing 24	0.74	0.74	1.02	0.67
C16orf90	C16orf90	chromosome 16 open reading frame 90	0.74	0.76	1.02	0.58
DHX35	DHX35;C20orf15;DDX35;FLJ22759;KAIA0875	DEAH (Asp-Glu-Ala-His) box polypeptide 35	0.73	0.75	1.02	0.5
SPATA7	SPATA7;DKFZp686D07199;HSD-3.1;HSD3;LCA3;MGC102934	spermatogenesis associated 7	0.72	0.77	1.02	0.51
KRT20	KRT20;CD20;CK-20;CK20;K20;KRT21;MGC35423	keratin 20	0.72	0.8	1.02	0.58
MATN3	MATN3;DIPOA;EDM5;HOA;OADIP;OS2	matrilin 3	0.72	0.77	1.02	0.54
GPT	GPT;AAT1;ALT1;GPT1	glutamic-pyruvate transaminase (alanine aminotransferase)	0.72	0.78	1.02	0.51
CD19	CD19;B4;CVID3;MGC12802	CD19 molecule	0.72	0.69	1.02	0.58
CCDC80	CCDC80;DRO1;MGC131805;MGC134851;SSG1;URB;okuribin	coiled-coil domain containing 80	0.72	0.76	1.02	0.56
ZYG11B	ZYG11B;FLJ13456;ZYG11	zyg-11 homolog B (C. elegans)	0.72	0.8	1.02	0.53
BRF1	BRF1;BRF;BRF-1;FLJ42674;FLJ43034;GTF3B;MGC105048;TAF3B2;TAF3C;TAFIII90;TF3B90;TFIIIB90;hBRF	BRF1 homolog, subunit of RNA polymerase III transcription initiation factor IIIB (S. cerevisiae)	0.71	0.69	1.02	0.5
WBSCR16	WBSCR16;DKFZp434D0421;MGC189739;MGC44931	Williams-Beuren syndrome chromosome region 16	0.71	0.73	1.02	0.5
CIAO1	CIAO1;CIA1;WDR39	cytosolic iron-sulfur protein assembly 1	0.71	0.77	1.02	0.51
TEX9	TEX9;MGC40181	testis expressed 9	0.7	0.78	1.02	0.58
KIAA1328	KIAA1328	KIAA1328	0.7	0.75	1.02	0.53
BARHL2	BARHL2	BarH-like homeobox 2	0.7	0.69	1.02	0.52
EMILIN1	EMILIN1;DKFZp586M121;EMI;EMILIN;gp115	elastin microfibril interfacer 1	0.7	0.7	1.02	0.54
FAM73B	FAM73B;C9orf54;FLJ00199;FLJ14596	family with sequence similarity 73, member B	0.7	0.68	1.02	0.52
FIGN	FIGN	fidgetin	0.7	0.7	1.02	0.57
ADIPOR1	ADIPOR1;ACDCR1;CGI45;FLJ25385;FLJ42464;PAQR1;TESBP1A	adiponectin receptor 1	0.7	0.74	1.02	0.55
SYT15	SYT15;CHR10SYT;sytXV	synaptotagmin XV	0.69	0.64	1.02	0.53
SNX21	SNX21;C20orf161;MGC29895;PP3993;SNX-L;dJ337O18.4	sorting nexin family member 21	0.69	0.76	1.02	0.51
KDM1A	KDM1A;AOF2;BHC110;KDM1;KIAA0601;LSD1	lysine (K)-specific demethylase 1A	0.69	0.66	1.02	0.52
CXorf22	CXorf22;MGC34831	chromosome X open reading frame 22	0.68	0.68	1.02	0.55
GP5	GP5;CD42d	glycoprotein V (platelet)	0.68	0.71	1.02	0.51
MORN1	MORN1;FLJ13941	MORN repeat containing 1	0.68	0.76	1.02	0.51
H1FOO	H1FOO;MGC50807;osH1	H1 histone family, member O, oocyte-specific	0.68	0.73	1.02	0.51
ALDH16A1	ALDH16A1;MGC10204	aldehyde dehydrogenase 16 family, member A1	0.68	0.69	1.02	0.49
IFT80	IFT80;ATD2;KIAA1374;MGC126543;WDR56	intraflagellar transport 80 homolog (Chlamydomonas)	0.68	0.74	1.02	0.5
GSTP1	GSTP1;DFN7;FAEES3;GST3;GSTP;PI	glutathione S-transferase pi 1	0.68	0.7	1.02	0.47
BPIFA2	BPIFA2;C20orf70;PSP;SPLUNC2;bA49G10.1	BPI fold containing family A, member 2	0.68	0.7	1.02	0.47
CPEB1	CPEB1;CEBP;CPE-BP1;CPEB;FLJ13203;MGC34136;MGC60106	cytoplasmic polyadenylation element binding protein 1	0.67	0.67	1.02	0.51
SLC22A14	SLC22A14;MGC163415;OCTL2;OCTL4;ORCTL4	solute carrier family 22, member 14	0.67	0.68	1.02	0.46
VSTM1	VSTM1;MGC119160;MGC119161;SIRL1;UNQ3033	V-set and transmembrane domain containing 1	0.67	0.66	1.02	0.5
CCT6A	CCT6A;CCT-zeta;CCT-zeta-1;CCT6;Cctz;HTR3;MGC126214;MGC126215;MoDP-2;TCP-1-zeta;TCP20;TCPZ;TTCP20	chaperonin containing TCP1, subunit 6A (zeta 1)	0.67	0.63	1.02	0.56
NRAP	NRAP	nebulin-related anchoring protein	0.66	0.7	1.02	0.53
FAM47B	FAM47B;FLJ35782	family with sequence similarity 47, member B	0.66	0.74	1.02	0.54
OASL	OASL;TRIP14;p59OASL	2'-5'-oligoadenylate synthetase-like	0.66	0.62	1.02	0.5
ZNF324	ZNF324;ZF5128;ZNF324A	zinc finger protein 324	0.66	0.7	1.02	0.53
FAM174B	FAM174B;MGC102891	family with sequence similarity 174, member B	0.66	0.66	1.02	0.53
TTC17	TTC17;DKFZp686D20222;FLJ10890;FLJ13099;FLJ23673	tetratricopeptide repeat domain 17	0.66	0.7	1.02	0.53
SLC37A3	SLC37A3;FLJ16367;MGC32939	solute carrier family 37 (glycerol-3-phosphate transporter), member 3	0.66	0.64	1.02	0.51
TMEM213	TMEM213	transmembrane protein 213	0.66	0.74	1.02	0.54
YBEY	YBEY;C21orf57;FLJ46907	ybeY metallopeptidase (putative)	0.66	0.69	1.02	0.52
ZFYVE27	ZFYVE27;PROTRUDIN;RP11-459F3.2;SPG33	zinc finger, FYVE domain containing 27	0.65	0.68	1.02	0.49
CHRNA1	CHRNA1;ACHRA;ACHRD;CHRNA;CMS2A;FCCMS;SCCMS	cholinergic receptor, nicotinic, alpha 1 (muscle)	0.64	0.69	1.02	0.54
ERCC8	ERCC8;CKN1;CSA	excision repair cross-complementing rodent repair deficiency, complementation group 8	0.64	0.64	1.02	0.55
KCNQ2	KCNQ2;BFNC;BFNS1;EBN;EBN1;EIEE7;ENB1;HNSPC;KCNA11;KV7.2;KVEBN1	potassium voltage-gated channel, KQT-like subfamily, member 2	0.64	0.64	1.02	0.5
ZBTB7C	ZBTB7C;APM-1;APM1;FLJ37907;ZBTB36;ZNF857C	zinc finger and BTB domain containing 7C	0.64	0.66	1.02	0.5
PVRL4	PVRL4;DKFZp686K05193;EDSS1;LNIR;PRR4;nectin-4	poliovirus receptor-related 4	0.64	0.62	1.02	0.54
SPOCD1	SPOCD1;FLJ25348;FLJ39908	SPOC domain containing 1	0.64	0.68	1.02	0.5
ECSIT	ECSIT;SITPEC	ECSIT homolog (Drosophila)	0.64	0.67	1.02	0.49
WBP1	WBP1;MGC15305;WBP-1	WW domain binding protein 1	0.64	0.68	1.02	0.51
ATL3	ATL3;DKFZp564J0863	atlastin GTPase 3	0.64	0.62	1.02	0.52
CEACAM4	CEACAM4;CGM7;CGM7_HUMAN;NCA	carcinoembryonic antigen-related cell adhesion molecule 4	0.63	0.64	1.02	0.44
OTX1	OTX1;FLJ38361;MGC15736	orthodenticle homeobox 1	0.63	0.63	1.02	0.55
NDUFV1	NDUFV1;CI-51K;CI51KD;FLJ59059;UQOR1	NADH dehydrogenase (ubiquinone) flavoprotein 1, 51kDa	0.63	0.63	1.02	0.49
FGD2	FGD2;FLJ00048;FLJ40929;MGC71330;ZFYVE4	FYVE, RhoGEF and PH domain containing 2	0.62	0.61	1.02	0.49
HOXC13	HOXC13;HOX3;HOX3G	homeobox C13	0.62	0.64	1.02	0.43
PCOLCE2	PCOLCE2;PCPE2	procollagen C-endopeptidase enhancer 2	0.62	0.62	1.02	0.47
WNT10A	WNT10A;FLJ14301;OODD;SSPS;STHAG4	wingless-type MMTV integration site family, member 10A	0.62	0.6	1.02	0.44
SSNA1	SSNA1;N14;NA-14;NA14	Sjogren syndrome nuclear autoantigen 1	0.62	0.64	1.02	0.43
OSMR	OSMR;MGC150626;MGC150627;MGC75127;OSMRB;PLCA1	oncostatin M receptor	0.62	0.6	1.02	0.47
XPO1	XPO1;CRM1;DKFZp686B1823;emb;exp1	exportin 1 (CRM1 homolog, yeast)	0.62	0.63	1.02	0.47
FGF14	FGF14;FGF-14;FHF-4;FHF4;MGC119129;SCA27	fibroblast growth factor 14	0.61	0.64	1.02	0.5
TGFB1	TGFB1;CED;DPD1;LAP;TGFB;TGFbeta	transforming growth factor, beta 1	0.61	0.6	1.02	0.47
HLTF	HLTF;HIP116;HIP116A;HLTF1;RNF80;SMARCA3;SNF2L3;ZBU1	helicase-like transcription factor	0.61	0.62	1.02	0.55
RND1	RND1;ARHS;FLJ42294;RHO6;RHOS	Rho family GTPase 1	0.84	0.94	1.03	0.66
TTLL11	TTLL11;C9orf20;bA244O19.1	tubulin tyrosine ligase-like family, member 11	0.84	0.84	1.03	0.63
TPPP	TPPP;TPPP/p25;TPPP1;p24;p25;p25alpha	tubulin polymerization promoting protein	0.84	0.91	1.03	0.64
CHST13	CHST13;C4ST3;MGC119278;MGC119279;MGC119281	carbohydrate (chondroitin 4) sulfotransferase 13	0.84	0.91	1.03	0.6
NADK	NADK;DKFZp686L22239;FLJ13052;FLJ37724;FLJ54695;FLJ77769;FLJ78247;FLJ78307;MGC1900;dJ283E3.1	NAD kinase	0.84	0.93	1.03	0.66
FAM89B	FAM89B;MTVR1	family with sequence similarity 89, member B	0.84	0.84	1.03	0.64
CD164	CD164;MGC-24;MUC-24;endolyn	CD164 molecule, sialomucin	0.84	0.86	1.03	0.71
SIK2	SIK2;DKFZp434K1115;KIAA0781;LOH11CR1I;QIK;SNF1LK2	salt-inducible kinase 2	0.84	0.94	1.03	0.66
COL4A4	COL4A4;CA44	collagen, type IV, alpha 4	0.83	0.87	1.03	0.63
FXYD1	FXYD1;MGC44983;PLM	FXYD domain containing ion transport regulator 1	0.83	0.86	1.03	0.67
CYB5D2	CYB5D2;MGC32124	cytochrome b5 domain containing 2	0.83	0.93	1.03	0.68
AHR	AHR;bHLHe76	aryl hydrocarbon receptor	0.83	0.87	1.03	0.69
ICAM2	ICAM2;CD102	intercellular adhesion molecule 2	0.82	0.9	1.03	0.63
PRTFDC1	PRTFDC1;FLJ11888;HHGP	phosphoribosyl transferase domain containing 1	0.82	0.8	1.03	0.63
DRD2	DRD2;D2DR;D2R	dopamine receptor D2	0.82	0.82	1.03	0.74
SLC7A8	SLC7A8;LAT2;LPI-PC1	solute carrier family 7 (amino acid transporter light chain, L system), member 8	0.82	0.79	1.03	0.7
PUS1	PUS1;MGC11268;MLASA1	pseudouridylate synthase 1	0.82	0.81	1.03	0.58
DBNDD2	DBNDD2;C20orf35;CK1BP;HSMNP1	dysbindin (dystrobrevin binding protein 1) domain containing 2	0.82	0.88	1.03	0.63
FZR1	FZR1;CDC20C;CDH1;FZR;FZR2;HCDH;HCDH1;KIAA1242	fizzy/cell division cycle 20 related 1 (Drosophila)	0.82	0.88	1.03	0.66
LTB	LTB;TNFC;TNFSF3;p33	lymphotoxin beta (TNF superfamily, member 3)	0.82	0.89	1.03	0.62
ST6GALNAC1	ST6GALNAC1;HSY11339;SIAT7A;ST6GalNAcI;STYI	ST6 (alpha-N-acetyl-neuraminyl-2,3-beta-galactosyl-1,3)-N-acetylgalactosaminide alpha-2,6-sialyltransferase 1	0.82	0.86	1.03	0.69
PDXP	PDXP;CIN;FLJ32703;PLP;dJ37E16.5	pyridoxal (pyridoxine, vitamin B6) phosphatase	0.82	0.84	1.03	0.65
BCKDK	BCKDK	branched chain ketoacid dehydrogenase kinase	0.82	0.84	1.03	0.7
SLC27A4	SLC27A4;ACSVL4;FATP4;IPS	solute carrier family 27 (fatty acid transporter), member 4	0.82	0.83	1.03	0.72
TROAP	TROAP;TASTIN	trophinin associated protein (tastin)	0.82	0.9	1.03	0.59
NDUFB9	NDUFB9;B22;DKFZp566O173;FLJ22885;LYRM3;UQOR22	NADH dehydrogenase (ubiquinone) 1 beta subcomplex, 9, 22kDa	0.82	0.84	1.03	0.62
NHP2	NHP2;DKCB2;NHP2P;NOLA2	NHP2 ribonucleoprotein homolog (yeast)	0.82	0.8	1.03	0.68
NOX5	NOX5;MGC149776;MGC149777	NADPH oxidase, EF-hand calcium binding domain 5	0.82	0.82	1.03	0.59
PCDHA10	PCDHA10;CNR8;CNRN8;CNRS8;CRNR8;PCDH-ALPHA10	protocadherin alpha 10	0.82	0.8	1.03	0.62
LOC349236	LOC349236	ENTREZ STATUS: REMAPPED TO GENE ID: 10507	0.82	0.9	1.03	0.63
C15orf55	C15orf55;DKFZp434O192;MGC138683;MGC138684;NUT	chromosome 15 open reading frame 55	0.81	0.86	1.03	0.65
NPY2R	NPY2R	neuropeptide Y receptor Y2	0.81	0.78	1.03	0.58
C17orf82	C17orf82	chromosome 17 open reading frame 82	0.81	0.82	1.03	0.6
FOXD4	FOXD4;FKHL9;FOXD4A;FREAC5;MGC105106	forkhead box D4	0.81	0.8	1.03	0.58
SMCR7	SMCR7;MGC23130;MID49	Smith-Magenis syndrome chromosome region, candidate 7	0.81	0.86	1.03	0.65
DUSP5	DUSP5;DUSP;HVH3	dual specificity phosphatase 5	0.81	0.78	1.03	0.59
GEM	GEM;KIR;MGC26294	GTP binding protein overexpressed in skeletal muscle	0.8	0.81	1.03	0.57
ICAM4	ICAM4;CD242;LW	intercellular adhesion molecule 4 (Landsteiner-Wiener blood group)	0.8	0.84	1.03	0.61
SLC25A2	SLC25A2;MGC119151;MGC119153;ORC2;ORNT2	solute carrier family 25 (mitochondrial carrier; ornithine transporter) member 2	0.8	0.8	1.03	0.58
HTR7	HTR7;5-HT7	5-hydroxytryptamine (serotonin) receptor 7 (adenylate cyclase-coupled)	0.8	0.8	1.03	0.72
FOXS1	FOXS1;FKHL18;FREAC10;MGC4544	forkhead box S1	0.8	0.76	1.03	0.61
VSIG8	VSIG8	V-set and immunoglobulin domain containing 8	0.8	0.88	1.03	0.62
SH3RF3	SH3RF3;FLJ00204;POSH2;SH3MD4	SH3 domain containing ring finger 3	0.8	0.81	1.03	0.72
FAM46B	FAM46B;MGC16491;MGC20845;RP11-344H11.8	family with sequence similarity 46, member B	0.8	0.88	1.03	0.68
KIAA0528	KIAA0528;DKFZp779N2044	KIAA0528	0.8	0.86	1.03	0.64
HSD17B11	HSD17B11;17BHSD11;DHRS8;PAN1B;RETSDR2;SDR16C2	hydroxysteroid (17-beta) dehydrogenase 11	0.8	0.81	1.03	0.57
PSME1	PSME1;IFI5111;MGC8628;PA28A;PA28alpha;REGalpha	proteasome (prosome, macropain) activator subunit 1 (PA28 alpha)	0.8	0.84	1.03	0.58
DDRGK1	DDRGK1;C20orf116;MGC2592;UFBP1;dJ1187M17.3	DDRGK domain containing 1	0.8	0.84	1.03	0.57
GLT1D1	GLT1D1;FLJ31978	glycosyltransferase 1 domain containing 1	0.79	0.87	1.03	0.66
GLP1R	GLP1R;MGC138331	glucagon-like peptide 1 receptor	0.78	0.76	1.03	0.57
SERPING1	SERPING1;C1IN;C1INH;C1NH;HAE1;HAE2	serpin peptidase inhibitor, clade G (C1 inhibitor), member 1	0.78	0.82	1.03	0.65
USP54	USP54;C10orf29;FLJ37318;bA137L10.3;bA137L10.4	ubiquitin specific peptidase 54	0.78	0.78	1.03	0.59
ZNF833P	ZNF833P;ZNF833	zinc finger protein 833, pseudogene	0.78	0.79	1.03	0.6
KIAA1639	KIAA1639	ENTREZ STATUS: REMAPPED TO GENE ID: 84033	0.78	0.81	1.03	0.67
TTC32	TTC32	tetratricopeptide repeat domain 32	0.77	0.81	1.03	0.61

ANKRD29	ANKRD29;FLJ25053	ankyrin repeat domain 29	0.77	0.72	1.03	0.57
ZAN	ZAN	zonadhesin	0.77	0.7	1.03	0.59
HIST1H2BI	HIST1H2BI;H2B/a;H2B/g;H2B/h;H2B/k;H2B/l;H2BFK	histone cluster 1, H2bi	0.77	0.78	1.03	0.65
MRPL54	MRPL54;L54mt;MRP-L54	mitochondrial ribosomal protein L54	0.77	0.82	1.03	0.67
TMOD2	TMOD2;MGC39481;N-TMOD;NTMOD	tropomodulin 2 (neuronal)	0.76	0.7	1.03	0.57
CSAG1	CSAG1;CSAGE;CT24.1	chondrosarcoma associated gene 1	0.76	0.78	1.03	0.65
RAB9B	RAB9B;RAB9L;Rab-9L	RAB9B, member RAS oncogene family	0.76	0.82	1.03	0.54
CYP2A6	CYP2A6;CPA6;CYP2A;CYP2A3;P450C2A;P450PB	cytochrome P450, family 2, subfamily A, polypeptide 6	0.76	0.74	1.03	0.65
C9orf24	C9orf24;CBE1;MGC32921;MGC33614;NYD-SP22;bA573M23.4	chromosome 9 open reading frame 24	0.76	0.8	1.03	0.6
AKR1C2	AKR1C2;AKR1C-pseudo;BABP;DD;DD2;DDH2;FLJ53800;HAKRD;HBAB;MCDR2;SRXY8	aldo-keto reductase family 1, member C2 (dihydrodiol dehydrogenase 2; bile acid binding protein; 3-alpha hydroxysteroid dehydrogenase, type III)	0.76	0.76	1.03	0.66
ADAMTS13	ADAMTS13;13;ADAM-TS;ADAM-TS13;ADAMTS-13;C9orf8;DKFZp434C2322;FLJ42993;MGC118899;MGC118900;TTP;VWFCP;vWF-CP	ADAM metallopeptidase with thrombospondin type 1 motif, 13	0.76	0.78	1.03	0.58
MESP2	MESP2;SCDO2;bHLHc6	mesoderm posterior 2 homolog (mouse)	0.76	0.76	1.03	0.6
BCL9	BCL9;LGS;MGC131591	B-cell CLL/lymphoma 9	0.76	0.83	1.03	0.67
LOC652591	LOC652591	ENTREZ STATUS: DISCONTINUED	0.76	0.77	1.03	0.69
IFI44L	IFI44L;C1orf29	interferon-induced protein 44-like	0.75	0.82	1.03	0.58
BTD	BTD	biotinidase	0.75	0.76	1.03	0.53
GPN3	GPN3;ATPBD1C;MGC14560;MGC32810	GPN-loop GTPase 3	0.75	0.8	1.03	0.57
LUM	LUM;LDC;SLRR2D	lumican	0.74	0.8	1.03	0.6
BTN3A3	BTN3A3;BTF3	butyrophilin, subfamily 3, member A3	0.74	0.8	1.03	0.6
OBSL1	OBSL1;KIAA0657;MGC71026	obscurin-like 1	0.74	0.82	1.03	0.57
WISP2	WISP2;CCN5;CT58;CTGF-L	WNT1 inducible signaling pathway protein 2	0.74	0.77	1.03	0.6
BSPRY	BSPRY;FLJ20150	B-box and SPRY domain containing	0.74	0.8	1.03	0.64
HOXB2	HOXB2;HOX2;HOX2H;Hox-2.8;K8	homeobox B2	0.74	0.77	1.03	0.6
ARPC2	ARPC2;ARC34;PNAS-139;p34-Arc	actin related protein 2/3 complex, subunit 2, 34kDa	0.74	0.8	1.03	0.64
CCL18	CCL18;AMAC-1;AMAC1;CKb7;DC-CK1;DCCK1;MIP-4;PARC;SCYA18	chemokine (C-C motif) ligand 18 (pulmonary and activation-regulated)	0.73	0.76	1.03	0.53
MAP3K13	MAP3K13;LZK;MEKK13;MGC133196;MLK	mitogen-activated protein kinase kinase kinase 13	0.73	0.8	1.03	0.57
GRIN2D	GRIN2D;EB11;NMDAR2D;NR2D	glutamate receptor, ionotropic, N-methyl D-aspartate 2D	0.73	0.75	1.03	0.58
KCTD9	KCTD9;FLJ20038	potassium channel tetramerisation domain containing 9	0.73	0.72	1.03	0.52
CHMP4B	CHMP4B;C20orf178;CHMP4A;CTPP3;SNF7;SNF7-2;Shax1;VPS32B;Vps32-2;dJ553F4.4	charged multivesicular body protein 4B	0.73	0.78	1.03	0.57
NDUFS5	NDUFS5;CI-15k;CI15K	NADH dehydrogenase (ubiquinone) Fe-S protein 5, 15kDa (NADH-coenzyme Q reductase)	0.73	0.74	1.03	0.54
CACNA1E	CACNA1E;BII;CACH6;CACNL1A6;Cav2.3	calcium channel, voltage-dependent, R type, alpha 1E subunit	0.72	0.79	1.03	0.61
HTR3A	HTR3A;5-HT-3;5-HT3A;5-HT3R;5HT3R;HTR3	5-hydroxytryptamine (serotonin) receptor 3A	0.72	0.73	1.03	0.57
NEK3	NEK3;HSPK36;MGC29949	NIMA (never in mitosis gene a)-related kinase 3	0.72	0.8	1.03	0.57
CMPK2	CMPK2;TMPK2;TYKi;UMP-CMPK2	cytidine monophosphate (UMP-CMP) kinase 2, mitochondrial	0.72	0.76	1.03	0.57
CD58	CD58;FLJ23181;FLJ43722;LFA-3;LFA3;ag3	CD58 molecule	0.72	0.81	1.03	0.6
SNX29	SNX29;A-388D4.1;FLJ00143;FLJ12363;FLJ40609;RUNDC2A	sorting nexin 29	0.72	0.74	1.03	0.5
37865	SEPT3;MGC133218;SEP3;bK250D10.3	septin 3	0.72	0.8	1.03	0.6
C19orf47	C19orf47;DKFZp686P05129;FLJ36888	chromosome 19 open reading frame 47	0.72	0.8	1.03	0.53
GATA2	GATA2;DCML;FLJ45948;MGC2306;MONOMAC;NFE1B	GATA binding protein 2	0.72	0.73	1.03	0.51
ZNF280B	ZNF280B;5'OY11.1;D87009.C22.3;SUHW2;ZNF279;ZNF632	zinc finger protein 280B	0.72	0.8	1.03	0.59
RAPGEF6	RAPGEF6;DKFZp667N084;DKFZp686I15116;KIA001LB;PDZ-GEF2;PDZGEF2;RA-GEF-2;RAGEF2	Rap guanine nucleotide exchange factor (GEF) 6	0.72	0.79	1.03	0.59
FAM114A1	FAM114A1;FLJ33151;Noxp20	family with sequence similarity 114, member A1	0.72	0.81	1.03	0.54
VPS28	VPS28;MGC60323	vacuolar protein sorting 28 homolog (S. cerevisiae)	0.72	0.77	1.03	0.58
SSR2	SSR2;DKFZp686F19123;TLAP;TRAP-BETA;TRAPB	signal sequence receptor, beta (translocon-associated protein beta)	0.72	0.76	1.03	0.58
METTL14	METTL14;KIAA1627	methyltransferase like 14	0.72	0.73	1.03	0.58
PHLDA1	PHLDA1;DT1P1B11;MGC131738;PHRIP;TDAG51	pleckstrin homology-like domain, family A, member 1	0.71	0.76	1.03	0.54
MACROD1	MACROD1;LRP16	MACRO domain containing 1	0.71	0.76	1.03	0.57
FNTA	FNTA;FPTA;MGC99680;PGGT1A;PTAR2	farnesyltransferase, CAAX box, alpha	0.71	0.7	1.03	0.51
ESF1	ESF1;ABTAP;C20orf6;FLJ20368;bA526K24.1	ESF1, nucleolar pre-rRNA processing protein, homolog (S. cerevisiae)	0.71	0.7	1.03	0.56
GINS1	GINS1;KIAA0186;PSF1	GINS complex subunit 1 (Psf1 homolog)	0.71	0.73	1.03	0.53
MPPED2	MPPED2;239FB;C11orf8	metallophosphoesterase domain containing 2	0.7	0.77	1.03	0.58
TAS2R14	TAS2R14;MGC125491;MGC125492;T2R14;TRB1	taste receptor, type 2, member 14	0.7	0.72	1.03	0.49
NR4A2	NR4A2;HZF-3;NOT;NURR1;RNR1;TINUR	nuclear receptor subfamily 4, group A, member 2	0.7	0.68	1.03	0.54
PITPNM2	PITPNM2;KIAA1457;NIR-3;NIR3;RDGB2;RDGBA2	phosphatidylinositol transfer protein, membrane-associated 2	0.7	0.7	1.03	0.52
ZNF114	ZNF114;MGC149700;MGC17986	zinc finger protein 114	0.7	0.7	1.03	0.53
MOCOS	MOCOS;FLJ20733;HMCS;MCS;MOS	molybdenum cofactor sulfurase	0.7	0.68	1.03	0.53
WIPF1	WIPF1;MGC111041;PRPL-2;WASPIP;WIP	WAS/WASL interacting protein family, member 1	0.7	0.69	1.03	0.53
CHPF	CHPF;CHSY2;CSS2;FLJ22678	chondroitin polymerizing factor	0.7	0.78	1.03	0.58
ZNF511	ZNF511;MGC30006	zinc finger protein 511	0.7	0.72	1.03	0.5
ATG7	ATG7;APG7-LIKE;APG7L;DKFZp434N0735;GSA7	ATG7 autophagy related 7 homolog (S. cerevisiae)	0.7	0.7	1.03	0.55
ANXA9	ANXA9;ANX31	annexin A9	0.7	0.74	1.03	0.58
SPRR1A	SPRR1A;SPRK	small proline-rich protein 1A	0.69	0.69	1.03	0.54
ODF2	ODF2;CT134;FLJ44866;MGC111096;MGC9034;ODF2/1;ODF2/2;ODF84	outer dense fiber of sperm tails 2	0.69	0.75	1.03	0.61
RRP36	RRP36;C6orf153;dJ20C7.4	ribosomal RNA processing 36 homolog (S. cerevisiae)	0.69	0.7	1.03	0.52
ZNF454	ZNF454;FLJ37444;MGC138481	zinc finger protein 454	0.68	0.74	1.03	0.49
SCUBE2	SCUBE2;CEGB1;CEGF1;CEGP1;FLJ16792;FLJ35234;MGC133057	signal peptide, CUB domain, EGF-like 2	0.68	0.64	1.03	0.51
CD52	CD52;CDW52	CD52 molecule	0.68	0.76	1.03	0.59
OR11L1	OR11L1	olfactory receptor, family 11, subfamily L, member 1	0.68	0.69	1.03	0.54
CLUAP1	CLUAP1;FAP22;FLJ13297;KIAA0643	clusterin associated protein 1	0.68	0.77	1.03	0.51
SLC39A13	SLC39A13;FLJ25785	solute carrier family 39 (zinc transporter), member 13	0.68	0.68	1.03	0.55
JUNB	JUNB;AP-1	jun B proto-oncogene	0.68	0.68	1.03	0.58
LENG8	LENG8;KIAA1932;MGC40108;pp13842	leukocyte receptor cluster (LRC) member 8	0.68	0.7	1.03	0.48
KAT7	KAT7;HBO1;HBOA;MYST2	K(lysine) acetyltransferase 7	0.68	0.7	1.03	0.55
SLC9A9	SLC9A9;AUTS16;FLJ35613;NHE9	solute carrier family 9 (sodium/hydrogen exchanger), member 9	0.67	0.76	1.03	0.52
RTP2	RTP2;MGC78665	receptor (chemosensory) transporter protein 2	0.67	0.72	1.03	0.59
INHBE	INHBE;MGC4638	inhibin, beta E	0.67	0.72	1.03	0.54
ATP6V0A4	ATP6V0A4;A4;ATP6N1B;ATP6N2;MGC130016;MGC130017;RDRTA2;RTA1C;RTADR;STV1;VPH1;VPP2	ATPase, H+ transporting, lysosomal V0 subunit a4	0.67	0.68	1.03	0.46
ADCK4	ADCK4;FLJ12229	aarF domain containing kinase 4	0.67	0.74	1.03	0.53
TMEM151A	TMEM151A;MGC33486;TMEM151	transmembrane protein 151A	0.66	0.74	1.03	0.49
RPS6KA4	RPS6KA4;MSK2;RSK-B	ribosomal protein S6 kinase, 90kDa, polypeptide 4	0.66	0.68	1.03	0.56
C3orf38	C3orf38;FLJ54270;MGC26717	chromosome 3 open reading frame 38	0.66	0.66	1.03	0.48
TRIP6	TRIP6;MGC10556;MGC10558;MGC29959;MGC3837;MGC4423;OIP-1;OIP1;TRIP-6;TRIP6i2;ZRP-1	thyroid hormone receptor interactor 6	0.66	0.63	1.03	0.56
OR1I1	OR1I1;OR19-20;OR1I1P;OR1I1Q	olfactory receptor, family 1, subfamily I, member 1	0.65	0.74	1.03	0.54
C6orf1	C6orf1;LBH;MGC57858	chromosome 6 open reading frame 1	0.65	0.73	1.03	0.48
C12orf48	C12orf48;AROM;FLJ20641;PARPBP	chromosome 12 open reading frame 48	0.65	0.72	1.03	0.48
GLRX	GLRX;FLJ43648;GRX;GRX1;MGC117407	glutaredoxin (thioltransferase)	0.65	0.62	1.03	0.49
SORT1	SORT1;Gp95;LDLCQ6;NT3	sortilin 1	0.65	0.73	1.03	0.54
FOXF1	FOXF1;ACDMPV;FKHL5;FREAC1;MGC105125	forkhead box F1	0.64	0.64	1.03	0.55
P2RY6	P2RY6;MGC15335;P2Y6	pyrimidinergic receptor P2Y, G-protein coupled, 6	0.64	0.64	1.03	0.58
KIF16B	KIF16B;C20orf23;KISC20ORF;SNX23	kinesin family member 16B	0.64	0.64	1.03	0.46
ZNF16	ZNF16;KOX9	zinc finger protein 16	0.64	0.7	1.03	0.49
PYGO2	PYGO2;1190004M21Rik;FLJ33226	pygopus homolog 2 (Drosophila)	0.64	0.6	1.03	0.5
TMEM128	TMEM128;MGC110944;MGC13159	transmembrane protein 128	0.64	0.64	1.03	0.49
LRRN1	LRRN1;FIGLER3;KIAA1497;NLRR-1	leucine rich repeat neuronal 1	0.64	0.64	1.03	0.48
TOR1B	TOR1B;DQ1;MGC4386	torsin family 1, member B (torsin B)	0.64	0.7	1.03	0.47
ZNF317	ZNF317;KIAA1588	zinc finger protein 317	0.64	0.62	1.03	0.52
YEATS2	YEATS2;FLJ10201;FLJ12841;FLJ13308;FLJ33401;FLJ34335;KIAA1197	YEATS domain containing 2	0.64	0.68	1.03	0.5
MT1F	MT1F;MGC32732;MT1	metallothionein 1F	0.64	0.58	1.03	0.47
EDDM3B	EDDM3B;EP3B;FAM12B;HE3-BETA;HE3B	epididymal protein 3B	0.64	0.68	1.03	0.49
UBE2E3	UBE2E3;UBCH9;UbcM2	ubiquitin-conjugating enzyme E2E 3	0.63	0.6	1.03	0.45
ZBED4	ZBED4	zinc finger, BED-type containing 4	0.63	0.67	1.03	0.51
MAPT	MAPT;DDPAC;FLJ31424;FTDP-17;MAPTL;MGC138549;MSTD;MTBT1;MTBT2;PPND;TAU	microtubule-associated protein tau	0.62	0.64	1.03	0.48
KALRN	KALRN;ARHGEF24;CHDS5;DUET;DUO;FLJ12332;FLJ16443;FLJ18196;FLJ18623;HAPIP;TRAD	kalirin, RhoGEF kinase	0.62	0.68	1.03	0.49
RPL39L	RPL39L;RPL39L1	ribosomal protein L39-like	0.62	0.67	1.03	0.47
SPSB3	SPSB3;C16orf31;SSB3	splA/ryanodine receptor domain and SOCS box containing 3	0.62	0.61	1.03	0.44
KIAA2013	KIAA2013;MGC33867	KIAA2013	0.62	0.62	1.03	0.47
EMD	EMD;EDMD;LEMD5;STA	emerin	0.62	0.65	1.03	0.47
TGOLN2	TGOLN2;MGC14722;TGN38;TGN46;TGN48;TGN51;TTGN2	trans-golgi network protein 2	0.61	0.66	1.03	0.46
SH3KBP1	SH3KBP1;CD2BP3;CIN85;GIG10;HSB-1;HSB1;MIG18	SH3-domain kinase binding protein 1	0.61	0.66	1.03	0.44
OR52J3	OR52J3;OR11-32	olfactory receptor, family 52, subfamily J, member 3	0.84	0.9	1.04	0.62
KRT40	KRT40;CK-40;FLJ36600;K40;KA36	keratin 40	0.84	0.86	1.04	0.64
CASS4	CASS4;C20orf32;CAS4;HEFL;HEPL	Cas scaffolding protein family member 4	0.84	0.79	1.04	0.66
HTR3C	HTR3C	5-hydroxytryptamine (serotonin) receptor 3, family member C	0.84	0.92	1.04	0.71
SLC6A12	SLC6A12;BGT-1;BGT1;FLJ38727;GAT2	solute carrier family 6 (neurotransmitter transporter, betaine/GABA), member 12	0.84	0.86	1.04	0.64
FOXL2	FOXL2;BPES;BPES1;PFRK;PINTO;POF3	forkhead box L2	0.84	0.86	1.04	0.63
RSBN1	RSBN1;DKFZp781E21150;ROSBIN;RP11-324J2.1	round spermatid basic protein 1	0.84	0.94	1.04	0.63
FAM110A	FAM110A;C20orf55;F10;MGC2450;MGC4675;bA371L19.3	family with sequence similarity 110, member A	0.84	0.9	1.04	0.65
GLRX2	GLRX2;CGI-133;GRX2	glutaredoxin 2	0.84	0.88	1.04	0.68
DCUN1D3	DCUN1D3;44M2.4;DKFZp686G23203;DKFZp686O0290;FLJ41725;MGC48972	DCN1, defective in cullin neddylation 1, domain containing 3 (S. cerevisiae)	0.84	0.8	1.04	0.6
MRPS26	MRPS26;C20orf193;GI008;MRP-S13;MRP-S26;MRPS13;NY-BR-87;RPMS13;dJ534B8.3	mitochondrial ribosomal protein S26	0.84	0.79	1.04	0.66
C9orf100	C9orf100;FLJ14642;MGC44886;RP11-331F9.7	chromosome 9 open reading frame 100	0.83	0.9	1.04	0.61
ZC3H12A	ZC3H12A;FLJ23231;MCPIP;MCPIP1;RP3-423B22.1;dJ423B22.1	zinc finger CCCH-type containing 12A	0.83	0.84	1.04	0.63
SNX10	SNX10;MGC33054	sorting nexin 10	0.83	0.91	1.04	0.74
SNTA1	SNTA1;LQT12;SNT1;TACIP1;dJ1187J4.5	syntrophin, alpha 1 (dystrophin-associated protein A1, 59kDa, acidic component)	0.83	0.83	1.04	0.65
TEKT1	TEKT1	tektin 1	0.82	0.9	1.04	0.63
ARMC2	ARMC2;DKFZp434P0714;RP11-787I22.1;bA787I22.1	armadillo repeat containing 2	0.82	0.86	1.04	0.6
MYOM1	MYOM1;MGC134946;MGC134947;SKELEMIN	myomesin 1, 185kDa	0.82	0.82	1.04	0.67
ACTG2	ACTG2;ACT;ACTA3;ACTE;ACTL3;ACTSG	actin, gamma 2, smooth muscle, enteric	0.82	0.94	1.04	0.69
MRPL44	MRPL44;FLJ12701;FLJ13990;FLJ37688;L44MT;MRP-L44	mitochondrial ribosomal protein L44	0.82	0.89	1.04	0.66

DMXL2	DMXL2;FLJ26672;KIAA0856;RC3	Dmx-like 2	0.82	0.82	1.04	0.63
FAM63B	FAM63B;KIAA1164	family with sequence similarity 63, member B	0.82	0.81	1.04	0.65
MTERFD1	MTERFD1;FLJ10939;mTERF3	MTERF domain containing 1	0.82	0.86	1.04	0.61
ZBED1	ZBED1;ALTE;DREF;KIAA0785;TRAMP;hDREF	zinc finger, BED-type containing 1	0.82	0.88	1.04	0.67
DMWD	DMWD;D19S593E;DMR-N9;DMRN9;gene59	dystrophia myotonica, WD repeat containing	0.82	0.87	1.04	0.66
ZMYM3	ZMYM3;DXS6673E;KIAA0385;MYM;XFIM;ZNF198L2;ZNF261	zinc finger, MYM-type 3	0.82	0.89	1.04	0.6
MTA1	MTA1	metastasis associated 1	0.82	0.84	1.04	0.62
IRF3	IRF3	interferon regulatory factor 3	0.82	0.8	1.04	0.64
IDH3A	IDH3A	isocitrate dehydrogenase 3 (NAD+) alpha	0.82	0.83	1.04	0.58
DNLZ	DNLZ;C9orf151;HEP;MGC87491;TIMM15;ZIM17;bA413M3.2	DNL-type zinc finger	0.82	0.82	1.04	0.62
EXD3	EXD3;FLJ20433;FLJ30442;MGC131904;MGC74981;mut-7	exonuclease 3'-5' domain containing 3	0.82	0.87	1.04	0.66
C17orf104	C17orf104;FLJ35848;FLJ40428;MGC43301	chromosome 17 open reading frame 104	0.82	0.93	1.04	0.68
HYAL3	HYAL3;HYAL-3;LUCA-3;LUCA3	hyaluronoglucosaminidase 3	0.81	0.85	1.04	0.66
BTBD9	BTBD9;FLJ32945;KIAA1880;MGC120517;MGC120519;MGC120520;dJ322I12.1	BTB (POZ) domain containing 9	0.81	0.86	1.04	0.62
MINA	MINA;DKFZp762O1912;FLJ14393;MDIG;MINA53;NO52	MYC induced nuclear antigen	0.81	0.86	1.04	0.65
OR51G1	OR51G1;OR11-29;OR51G3P	olfactory receptor, family 51, subfamily G, member 1	0.8	0.81	1.04	0.65
FLJ45139	FLJ45139	FLJ45139 protein	0.8	0.84	1.04	0.57
SPRYD5	SPRYD5;MGC10977;TRIM51	SPRY domain containing 5	0.8	0.82	1.04	0.73
ZNF595	ZNF595;FLJ31740	zinc finger protein 595	0.8	0.82	1.04	0.68
RG9MTD3	RG9MTD3;FLJ31455;RP11-3J10.9;bA3J10.9	RNA (guanine-9-) methyltransferase domain containing 3	0.8	0.82	1.04	0.68
DNM3	DNM3;KIAA0820;MGC70433	dynamin 3	0.8	0.88	1.04	0.58
C20orf160	C20orf160;FLJ43600;dJ310O13.5	chromosome 20 open reading frame 160	0.8	0.74	1.04	0.62
HNF4A	HNF4A;FLJ39654;HNF4;HNF4a7;HNF4a8;HNF4a9;HNF4alpha;MODY;MODY1;NR2A1;NR2A21;TCF;TCF14	hepatocyte nuclear factor 4, alpha	0.8	0.79	1.04	0.57
CNTD2	CNTD2;FLJ13265	cyclin N-terminal domain containing 2	0.8	0.86	1.04	0.64
WFIKKN1	WFIKKN1;C16orf12;MGC126651;MGC126655;RJD2;WFIKKN	WAP, follistatin/kazal, immunoglobulin, kunitz and netrin domain containing 1	0.8	0.8	1.04	0.61
RNPEPL1	RNPEPL1;FLJ10806;FLJ26675;MGC99544	arginyl aminopeptidase (aminopeptidase B)-like 1	0.8	0.76	1.04	0.58
MDN1	MDN1;DKFZp686H16106;FLJ23395;FLJ25587;FLJ42031;FLJ43191;KIAA0301	MDN1, midasin homolog (yeast)	0.8	0.84	1.04	0.65
SLC6A9	SLC6A9;DKFZp547A1118;GLYT1	solute carrier family 6 (neurotransmitter transporter, glycine), member 9	0.8	0.9	1.04	0.67
SNX1	SNX1;HsT17379;MGC8664;VPS5	sorting nexin 1	0.8	0.85	1.04	0.65
VBP1	VBP1;PFD3;PFDN3;VBP-1	von Hippel-Lindau binding protein 1	0.8	0.88	1.04	0.65
H2AFY2	H2AFY2;macroH2A2	H2A histone family, member Y2	0.8	0.86	1.04	0.66
NFIB	NFIB;CTF;HMGIC/NFIB;NF-I/B;NF1-B;NFI-B;NFI-RED;NFIB2;NFIB3	nuclear factor I/B	0.8	0.76	1.04	0.67
FAM96A	FAM96A;FLJ22875	family with sequence similarity 96, member A	0.8	0.9	1.04	0.6
HGS	HGS;FLJ11421;FLJ39878;HRS	hepatocyte growth factor-regulated tyrosine kinase substrate	0.8	0.88	1.04	0.62
AHCYL1	AHCYL1;DCAL;IRBIT;PRO0233;XPVKONA	adenosylhomocysteinase-like 1	0.8	0.9	1.04	0.67
CDC23	CDC23;ANAPC8;APC8;CUT23	cell division cycle 23 homolog (S. cerevisiae)	0.8	0.77	1.04	0.68
NUP107	NUP107;NUP84	nucleoporin 107kDa	0.8	0.86	1.04	0.65
C8B	C8B;MGC163447	complement component 8, beta polypeptide	0.79	0.8	1.04	0.64
TEX28	TEX28;CXorf2;fTEX	testis expressed 28	0.79	0.73	1.04	0.57
SLC9A4	SLC9A4;DKFZp313B031;NHE4	solute carrier family 9 (sodium/hydrogen exchanger), member 4	0.79	0.85	1.04	0.64
ARL4D	ARL4D;ARF4L;ARL6	ADP-ribosylation factor-like 4D	0.79	0.79	1.04	0.57
AMZ1	AMZ1;KIAA1950	archaelysin family metallopeptidase 1	0.79	0.86	1.04	0.62
APBB3	APBB3;FE65L2;MGC150555;MGC87674;SRA	amyloid beta (A4) precursor protein-binding, family B, member 3	0.79	0.87	1.04	0.7
SNHG3-RCC1	SNHG3-RCC1	ENTREZ STATUS: REMAPPED TO GENE ID: 1104	0.79	0.86	1.04	0.57
CEL	CEL;BAL;BSDL;BSSL;CELL;CEase;FAP;FAPP;LIPA;MODY8	carboxyl ester lipase (bile salt-stimulated lipase)	0.78	0.8	1.04	0.56
ZSWIM3	ZSWIM3;C20orf164	zinc finger, SWIM-type containing 3	0.78	0.88	1.04	0.65
MUC4	MUC4;ASGP;HSA276359;MUC-4	mucin 4, cell surface associated	0.78	0.8	1.04	0.66
ZBTB48	ZBTB48;HKR3;ZNF855;pp9964	zinc finger and BTB domain containing 48	0.78	0.78	1.04	0.57
SPG7	SPG7;CAR;CMAR;FLJ37308;MGC126331;MGC126332;PGN;SPG5C	spastic paraplegia 7 (pure and complicated autosomal recessive)	0.78	0.8	1.04	0.6
ZNF273	ZNF273;HZF9;MGC12518	zinc finger protein 273	0.78	0.78	1.04	0.63
RGL2	RGL2;HKE1.5;KE1.5;RAB2L	ral guanine nucleotide dissociation stimulator-like 2	0.78	0.85	1.04	0.57
ECE2	ECE2;KIAA0604;MGC17664;MGC2408;MGC78487	endothelin converting enzyme 2	0.78	0.8	1.04	0.61
SOS1	SOS1;GF1;GGF1;GINGF;HGF;NS4	son of sevenless homolog 1 (Drosophila)	0.78	0.79	1.04	0.68
SGPL1	SGPL1;FLJ13811;KIAA1252;S1PL;SPL	sphingosine-1-phosphate lyase 1	0.78	0.78	1.04	0.61
CES4A	CES4A;CES6;CES8;FLJ37002;FLJ37464	carboxylesterase 4A	0.78	0.86	1.04	0.57
FAM187B	FAM187B;FLJ25660;TMEM162	family with sequence similarity 187, member B	0.78	0.8	1.04	0.58
KCNV1	KCNV1;HNKA;KCNB3;KV2.3;KV8.1	potassium channel, subfamily V, member 1	0.77	0.8	1.04	0.62
VWA2	VWA2;AMACO;CCSP-2;MGC157755;NET42	von Willebrand factor A domain containing 2	0.77	0.78	1.04	0.59
MGAT4A	MGAT4A;GNT-IV;GNT-IVA;GnT-4a	mannosyl (alpha-1,3-)-glycoprotein beta-1,4-N-acetylglucosaminyltransferase, isozyme A	0.77	0.84	1.04	0.65
NIPBL	NIPBL;CDLS;CDLS1;DKFZp434L1319;FLJ11203;FLJ12597;FLJ13354;FLJ13648;FLJ44854;IDN3;IDN3-B;Scc2	Nipped-B homolog (Drosophila)	0.77	0.78	1.04	0.6
CALU	CALU;FLJ90608	calumenin	0.77	0.78	1.04	0.59
BRAT1	BRAT1;BAAT1;C7orf27;MGC22916	BRCA1-associated ATM activator 1	0.77	0.83	1.04	0.63
CT45A5	CT45A5;CT45.5;CT455	cancer/testis antigen family 45, member A5	0.77	0.84	1.04	0.63
TCTE3	TCTE3;MGC142199;TCTEX1D3	t-complex-associated-testis-expressed 3	0.76	0.82	1.04	0.58
ZNF549	ZNF549;FLJ34917	zinc finger protein 549	0.76	0.86	1.04	0.63
PLXNA4	PLXNA4;DKFZp434G0625;DKFZp566O0546;FAYV2820;FLJ35026;FLJ38287;KIAA1550;PLEXA4;PLXNA4A;PLXNA4B;PRO34003	plexin A4	0.76	0.78	1.04	0.58
TMEM17	TMEM17;FLJ34583	transmembrane protein 17	0.76	0.82	1.04	0.56
CAMTA2	CAMTA2;KIAA0909	calmodulin binding transcription activator 2	0.76	0.82	1.04	0.63
C10orf26	C10orf26;FLJ20154;FLJ20367;MGC125648;OPA1L;OPAL1	chromosome 10 open reading frame 26	0.76	0.71	1.04	0.59
ZCCHC2	ZCCHC2;DKFZp451A185;FLJ20281;KIAA1744;MGC13269	zinc finger, CCHC domain containing 2	0.76	0.76	1.04	0.58
CASP9	CASP9;APAF-3;APAF3;CASPASE-9c;ICE-LAP6;MCH6;PPP1R56	caspase 9, apoptosis-related cysteine peptidase	0.76	0.78	1.04	0.59
TMEM200B	TMEM200B;MGC102864;MGC90489;TTMB	transmembrane protein 200B	0.76	0.74	1.04	0.6
NOL8	NOL8;C9orf34;DKFZp686P12242;FLJ20736;Nop132;bA62C3.3;bA62C3.4	nucleolar protein 8	0.76	0.78	1.04	0.65
LEPROTL1	LEPROTL1;HSPC112;Vps55;my047	leptin receptor overlapping transcript-like 1	0.76	0.79	1.04	0.53
CBWD5	CBWD5;DC36;FLJ34856;FLJ40320;FLJ77192;MGC167043;MGC44166	COBW domain containing 5	0.76	0.7	1.04	0.55
FAF1	FAF1;FLJ37524;HFAF1s;UBXD12;UBXN3A;hFAF1	Fas (TNFRSF6) associated factor 1	0.76	0.71	1.04	0.59
BCAS2	BCAS2;DAM1;SPF27;Snt309	breast carcinoma amplified sequence 2	0.76	0.78	1.04	0.61
HAX1	HAX1;FLJ17042;FLJ18492;FLJ93803;HCLSBP1;HS1BP1;SCN3	HCLS1 associated protein X-1	0.76	0.74	1.04	0.62
GCGR	GCGR;FLJ97182;GGR;MGC138246	glucagon receptor	0.75	0.75	1.04	0.68
ARMCX5	ARMCX5;DKFZp686A22185;FLJ12969;FLJ13382	armadillo repeat containing, X-linked 5	0.75	0.81	1.04	0.58
NUPR1	NUPR1;COM1;P8	nuclear protein, transcriptional regulator, 1	0.75	0.7	1.04	0.55
FAM160B1	FAM160B1;DKFZp686D10123;KIAA1600;bA106M7.3	family with sequence similarity 160, member B1	0.75	0.76	1.04	0.59
C3P1	C3P1;CPLP	complement component 3 precursor pseudogene	0.75	0.82	1.04	0.61
JMJD7-PLA2G	JMJD7-PLA2G4B;HsT16992;cPLA2-beta	JMJD7-PLA2G4B readthrough	0.75	0.86	1.04	0.6
TMEM8C	TMEM8C;TMEM226	transmembrane protein 8C	0.75	0.86	1.04	0.63
CYP4Z1	CYP4Z1;CYP4A20	cytochrome P450, family 4, subfamily Z, polypeptide 1	0.74	0.75	1.04	0.58
OSTalpha	OSTalpha;MGC39807;OSTA	organic solute transporter alpha	0.74	0.74	1.04	0.57
MAGEA10	MAGEA10;CT1.10;MAGE10;MGC10599	melanoma antigen family A, 10	0.74	0.7	1.04	0.56
PTPRN	PTPRN;FLJ16131;IA-2;IA-2/PTP;IA2;ICA512;R-PTP-N	protein tyrosine phosphatase, receptor type, N	0.74	0.76	1.04	0.6
C6orf126	C6orf126;UNQ3045;dJ510O8.5	chromosome 6 open reading frame 126	0.74	0.83	1.04	0.58
KRT78	KRT78;K5B;Kb40	keratin 78	0.74	0.83	1.04	0.55
CST6	CST6	cystatin E/M	0.74	0.78	1.04	0.57
PFKFB4	PFKFB4	6-phosphofructo-2-kinase/fructose-2,6-biphosphatase 4	0.74	0.82	1.04	0.59
PROCR	PROCR;CCCA;CCD41;CD201;EPCR;MGC23024;bA42O4.2	protein C receptor, endothelial	0.74	0.79	1.04	0.63
DCAKD	DCAKD;FLJ22955	dephospho-CoA kinase domain containing	0.74	0.79	1.04	0.59
TKT	TKT;FLJ34765;TK;TKT1	transketolase	0.74	0.72	1.04	0.57
ITGAE	ITGAE;CD103;HUMINAE;MGC141996	integrin, alpha E (antigen CD103, human mucosal lymphocyte antigen 1; alpha polypeptide)	0.73	0.74	1.04	0.54
ETV7	ETV7;TEL-2;TEL2;TELB	ets variant 7	0.73	0.75	1.04	0.55
LYZL4	LYZL4;1810009N24Rik;LYC4;MGC26768	lysozyme-like 4	0.73	0.73	1.04	0.53
ABCC6	ABCC6;ABC34;ARA;EST349056;MLP1;MOATE;MRP6;PXE;PXE1;URG7	ATP-binding cassette, sub-family C (CFTR/MRP), member 6	0.73	0.72	1.04	0.56
NSDHL	NSDHL;H105E3;SDR31E1;XAP104	NAD(P) dependent steroid dehydrogenase-like	0.73	0.74	1.04	0.64
TCP1	TCP1;CCT-alpha;CCT1;CCTa;D6S230E;TCP-1-alpha	t-complex 1	0.73	0.76	1.04	0.59
FTH1	FTH1;FHC;FTH;FTHL6;MGC104426;PIG15;PLIF	ferritin, heavy polypeptide 1	0.73	0.7	1.04	0.62
GPR179	GPR179;GPR158L1	G protein-coupled receptor 179	0.72	0.72	1.04	0.65
VTN	VTN;V75;VN;VNT	vitronectin	0.72	0.76	1.04	0.52
SOX2	SOX2;ANOP3;MCOPS3;MGC2413	SRY (sex determining region Y)-box 2	0.72	0.73	1.04	0.63
DOK5	DOK5;C20orf180;MGC16926	docking protein 5	0.72	0.78	1.04	0.55
KLF12	KLF12;AP-2rep;AP2REP	Kruppel-like factor 12	0.72	0.71	1.04	0.56
MIA3	MIA3;ARNT;D320;FLJ21469;FLJ39207;KIAA0268;TANGO;TANGO1;UNQ6077	melanoma inhibitory activity family, member 3	0.72	0.78	1.04	0.59
IQGAP2	IQGAP2	IQ motif containing GTPase activating protein 2	0.72	0.75	1.04	0.58
FECH	FECH;EPP;FCE	ferrochelatase	0.72	0.74	1.04	0.57
PUS7L	PUS7L;DKFZp434G1415;DKFZp781B2386;FLJ34711;FLJ40012	pseudouridylate synthase 7 homolog (S. cerevisiae)-like	0.72	0.82	1.04	0.6
SKAP2	SKAP2;MGC10411;MGC33304;PRAP;RA70;SAPS;SCAP2;SKAP-HOM;SKAP55R	src kinase associated phosphoprotein 2	0.72	0.72	1.04	0.58
SLC33A1	SLC33A1;ACATN;AT-1;AT1;SPG42	solute carrier family 33 (acetyl-CoA transporter), member 1	0.72	0.82	1.04	0.61
IL36RN	IL36RN;FIL1;FIL1(DELTA);FIL1D;IL1F5;IL1HY1;IL1L1;IL1RP3;IL36RA;MGC29840;PSORP	interleukin 36 receptor antagonist	0.72	0.75	1.04	0.58
CDK12	CDK12;CRK7;CRKR;CRKRS;KIAA0904;hCDK12	cyclin-dependent kinase 12	0.72	0.8	1.04	0.57
COL8A1	COL8A1;C3orf7;MGC9568	collagen, type VIII, alpha 1	0.71	0.74	1.04	0.5
MECR	MECR;CGI-63;FASN2B;NRBF1	mitochondrial trans-2-enoyl-CoA reductase	0.71	0.74	1.04	0.5
HEATR3	HEATR3;FLJ20718	HEAT repeat containing 3	0.71	0.74	1.04	0.53
GDF5	GDF5;BMP14;CDMP1;LAP4;OS5;SYNS2	growth differentiation factor 5	0.7	0.74	1.04	0.5
ALDH1A1	ALDH1A1;ALDC;ALDH-E1;ALDH1;ALDH11;MGC2318;PUMB1;RALDH1	aldehyde dehydrogenase 1 family, member A1	0.7	0.73	1.04	0.54
C1orf190	C1orf190;FLJ25163	chromosome 1 open reading frame 190	0.7	0.76	1.04	0.5
ALAS2	ALAS2;ALAS-E;ALASE;ANH1;ASB;FLJ93603;XLDPP;XLEPP;XLSA	aminolevulinate, delta-, synthase 2	0.7	0.74	1.04	0.57
GHRHR	GHRHR;GHRFR;GRFR;IGHD1B	growth hormone releasing hormone receptor	0.7	0.7	1.04	0.64
HMHB1	HMHB1;HB-1;HLA-HB1	histocompatibility (minor) HB-1	0.7	0.79	1.04	0.61
BRSK1	BRSK1;FLJ43009;KIAA1811;hSAD1	BR serine/threonine kinase 1	0.7	0.78	1.04	0.56
BBC3	BBC3;FLJ42994;JFY-1;JFY1;PUMA	BCL2 binding component 3	0.7	0.72	1.04	0.54
AK1	AK1	adenylate kinase 1	0.7	0.72	1.04	0.6
RAD23A	RAD23A;HHR23A;MGC111083	RAD23 homolog A (S. cerevisiae)	0.7	0.7	1.04	0.51

DUSP11	DUSP11;PIR1	dual specificity phosphatase 11 (RNA/RNP complex 1-interacting)	0.7	0.67	1.04	0.51
SYPL1	SYPL1;H-SP1;SYPL	synaptophysin-like 1	0.7	0.74	1.04	0.56
WDR11	WDR11;BRWD2;DKFZp434L1715;DR11;FLJ42531;WDR15	WD repeat domain 11	0.7	0.78	1.04	0.6
HTR3E	HTR3E;5-HT3c1;MGC120035;MGC120036;MGC120037	5-hydroxytryptamine (serotonin) receptor 3, family member E	0.69	0.72	1.04	0.49
CCDC114	CCDC114;FLJ32926	coiled-coil domain containing 114	0.69	0.78	1.04	0.58
CAMKK1	CAMKK1;CAMKKA;DKFZp761M0423;MGC34095	calcium/calmodulin-dependent protein kinase kinase 1, alpha	0.69	0.77	1.04	0.55
FEV	FEV;HSRNAFEV;PET-1	FEV (ETS oncogene family)	0.69	0.74	1.04	0.56
BCAR1	BCAR1;CAS;CAS1;CASS1;CRKAS;FLJ12176;FLJ45059;P130Cas	breast cancer anti-estrogen resistance 1	0.69	0.7	1.04	0.53
ERC1	ERC1;Cast2;ELKS;FLJ31750;KIAA1081;MGC12974;RAB6IP2	ELKS/RAB6-interacting/CAST family member 1	0.69	0.76	1.04	0.53
DDI2	DDI2;MGC14844;RP4-680D5.5	DNA-damage inducible 1 homolog 2 (S. cerevisiae)	0.69	0.7	1.04	0.61
CCDC159	CCDC159;FLJ45351;FLJ79238	coiled-coil domain containing 159	0.69	0.7	1.04	0.59
TM6SF1	TM6SF1	transmembrane 6 superfamily member 1	0.68	0.68	1.04	0.54
DNTT	DNTT;TDT	deoxynucleotidyltransferase, terminal	0.68	0.7	1.04	0.52
IL9	IL9;HP40;IL-9;P40	interleukin 9	0.68	0.76	1.04	0.58
CNDP1	CNDP1;CN1;CPGL2;HsT2308;MGC102737;MGC10825;MGC142072	carnosine dipeptidase 1 (metallopeptidase M20 family)	0.68	0.69	1.04	0.52
SNX18	SNX18;FLJ11997;FLJ32560;FLJ61062;MGC150827;MGC150829;SH3PX2;SH3PXD3B;SNAG1	sorting nexin 18	0.68	0.73	1.04	0.49
R3HDM2	R3HDM2;KIAA1002;PR01365	R3H domain containing 2	0.68	0.76	1.04	0.56
MRPS27	MRPS27;FLJ21764;FLJ23348;FLJ38645;KIAA0264;MRP-S27;S27mt	mitochondrial ribosomal protein S27	0.68	0.72	1.04	0.5
JUN	JUN;AP-1;AP1;c-Jun	jun proto-oncogene	0.68	0.75	1.04	0.49
HSPB1	HSPB1;CMT2F;DKFZp586P1322;HMN2B;HS.76067;HSP27;HSP28;Hsp25;SRP27	heat shock 27kDa protein 1	0.68	0.67	1.04	0.49
RPTOR	RPTOR;KIAA1303;KOG1;Mip1	regulatory associated protein of MTOR, complex 1	0.68	0.68	1.04	0.53
HIST1H4I	HIST1H4I;H4/m;H4FM;H4M;HIST1H4A;HIST1H4B;HIST1H4C;HIST1H4D;HIST1H4E;HIST1H4F;HIST1H4H;HIST1H4J;HIST1H4K;HIST1H4L;HIST2H4A;HIST2H4B;HIST4H4	histone cluster 1, H4i	0.67	0.73	1.04	0.59
GPR114	GPR114;PGR27	G protein-coupled receptor 114	0.67	0.68	1.04	0.61
CXCR4	CXCR4;CD184;D2S201E;FB22;HM89;HSY3RR;LAP3;LCR1;LESTR;NPY3R;NPYR;NPYRL;NPYY3R;WHIM	chemokine (C-X-C motif) receptor 4	0.66	0.66	1.04	0.6
LMX1A	LMX1A;LMX1;LMX1.1;MGC87616	LIM homeobox transcription factor 1, alpha	0.66	0.68	1.04	0.53
DLG4	DLG4;FLJ97752;FLJ98574;PSD95;SAP-90;SAP90	discs, large homolog 4 (Drosophila)	0.66	0.7	1.04	0.58
ATP1A3	ATP1A3;DYT12;MGC13276;RDP	ATPase, Na+/K+ transporting, alpha 3 polypeptide	0.66	0.76	1.04	0.52
C20orf166	C20orf166;FLJ32423;MGC129808;dJ353C17.1	chromosome 20 open reading frame 166	0.66	0.7	1.04	0.5
PRKAR2B	PRKAR2B;PRKAR2;RII-BETA	protein kinase, cAMP-dependent, regulatory, type II, beta	0.66	0.68	1.04	0.61
JDP2	JDP2;JUNDM2	Jun dimerization protein 2	0.66	0.7	1.04	0.48
ATF7	ATF7;ATFA;MGC57182	activating transcription factor 7	0.66	0.66	1.04	0.47
FABP6	FABP6;I-15P;I-BABP;I-BALB;I-BAP;ILBP;ILBP3;ILLBP	fatty acid binding protein 6, ileal	0.66	0.7	1.04	0.56
TAF4	TAF4;FLJ41943;TAF2C;TAF2C1;TAF4A;TAFII130;TAFII135	TAF4 RNA polymerase II, TATA box binding protein (TBP)-associated factor, 135kDa	0.66	0.74	1.04	0.49
THRB	THRB;ERBA-BETA;ERBA2;GRTH;MGC126109;MGC126110;NR1A2;PRTH;THR1;THRB1;THRB2	thyroid hormone receptor, beta (erythroblastic leukemia viral (v-erb-a) oncogene homolog 2, avian)	0.66	0.64	1.04	0.51
TCEB2	TCEB2;ELOB;SIII	transcription elongation factor B (SIII), polypeptide 2 (18kDa, elongin B)	0.66	0.66	1.04	0.51
DARS	DARS;DKFZp781B11202;MGC111579	aspartyl-tRNA synthetase	0.66	0.68	1.04	0.58
C20orf85	C20orf85;LLC1;bA196N14.1	chromosome 20 open reading frame 85	0.65	0.7	1.04	0.48
PIK3R5	PIK3R5;F730038I15Rik;FLJ26116;FOAP-2;P101-PI3K;p101	phosphoinositide-3-kinase, regulatory subunit 5	0.65	0.68	1.04	0.56
OR4F21	OR4F21;OR4F21P	olfactory receptor, family 4, subfamily F, member 21	0.64	0.6	1.04	0.5
OR2G6	OR2G6	olfactory receptor, family 2, subfamily G, member 6	0.64	0.68	1.04	0.51
OR3A3	OR3A3;OR17-137;OR17-16;OR17-201;OR3A6;OR3A7;OR3A8P	olfactory receptor, family 3, subfamily A, member 3	0.64	0.68	1.04	0.49
PDDC1	PDDC1;FLJ34283;FLJ35497;MGC131881;MGC138350	Parkinson disease 7 domain containing 1	0.64	0.7	1.04	0.49
ERP29	ERP29;C12orf8;ERp28;ERp31;PDI-DB;PDIA9	endoplasmic reticulum protein 29	0.64	0.66	1.04	0.48
CEP70	CEP70;BITE;FLJ13036	centrosomal protein 70kDa	0.64	0.66	1.04	0.49
XRN2	XRN2	5'-3' exoribonuclease 2	0.64	0.68	1.04	0.45
S100A4	S100A4;18A2;42A;CAPL;FSP1;MTS1;P9KA;PEL98	S100 calcium binding protein A4	0.63	0.64	1.04	0.49
MPZL3	MPZL3	myelin protein zero-like 3	0.63	0.62	1.04	0.53
DIS3	DIS3;DKFZp667L1817;EXOSC11;FLJ10484;KIAA1008;MGC33035;RP11-342J4.3;RRP44;bA555G22.1;dis3p	DIS3 mitotic control homolog (S. cerevisiae)	0.63	0.73	1.04	0.5
VKORC1L1	VKORC1L1;DKFZp762H0113	vitamin K epoxide reductase complex, subunit 1-like 1	0.63	0.62	1.04	0.52
PRLH	PRLH;PRH;PRRP	prolactin releasing hormone	0.62	0.7	1.04	0.47
AGL	AGL;GDE	amylo-alpha-1, 6-glucosidase, 4-alpha-glucanotransferase	0.62	0.7	1.04	0.52
UVRAG	UVRAG;DHTX;VPS38;p63	UV radiation resistance associated gene	0.62	0.62	1.04	0.54
N6AMT2	N6AMT2;ESP13	N-6 adenine-specific DNA methyltransferase 2 (putative)	0.61	0.63	1.04	0.45
SPOP	SPOP;TEF2	speckle-type POZ protein	0.61	0.57	1.04	0.47
ODF1	ODF1;CT133;HSPB10;MGC129928;MGC129929;ODF;ODF2;ODF27;ODFP;ODFPG;ODFPGA;ODFPGB;RT7;SODF	outer dense fiber of sperm tails 1	0.84	0.82	1.05	0.7
MAP3K15	MAP3K15;ASK3;FLJ16518;bA723P2.3	mitogen-activated protein kinase kinase kinase 15	0.84	0.88	1.05	0.72
SLC22A23	SLC22A23;C6orf85;DKFZp434F011;FLJ22174	solute carrier family 22, member 23	0.84	0.88	1.05	0.59
PKD1	PKD1;PBP;Pc-1;TRPP1	polycystic kidney disease 1 (autosomal dominant)	0.84	0.87	1.05	0.68
HMHA1	HMHA1;ARHGAP45;HA-1;HLA-HA1;KIAA0223	histocompatibility (minor) HA-1	0.84	0.94	1.05	0.7
ALG10B	ALG10B;ALG10;KCR1	asparagine-linked glycosylation 10, alpha-1,2-glucosyltransferase homolog B (yeast)	0.84	0.9	1.05	0.6
SAMD1	SAMD1	sterile alpha motif domain containing 1	0.84	0.95	1.05	0.73
NSD1	NSD1;ARA267;DKFZp666C163;FLJ10684;FLJ22263;FLJ44628;KMT3B;SOTOS;STO	nuclear receptor binding SET domain protein 1	0.84	0.87	1.05	0.78
DHX37	DHX37;DDX37;FLJ41974;KIAA1517;MGC2695;MGC4322;MGC46245	DEAH (Asp-Glu-Ala-His) box polypeptide 37	0.84	0.85	1.05	0.63
ATL2	ATL2;ARL3IP2;ARL6IP2;FLJ23293;atlastin2	atlastin GTPase 2	0.84	0.94	1.05	0.75
MAGED4B	MAGED4B;KIAA1859;MGC3210;MGC88639	melanoma antigen family D, 4B	0.84	0.94	1.05	0.66
BEX2	BEX2;BEX1;DJ79P11.1	brain expressed X-linked 2	0.83	0.88	1.05	0.61
MRPL38	MRPL38;FLJ13996;KIAA1863;L38MT;MGC4810;MRP-L3;MRP-L38;RPML3	mitochondrial ribosomal protein L38	0.83	0.88	1.05	0.61
TMEM69	TMEM69;C1orf154;FLJ21029;MGC104183;RP11-767N6.4	transmembrane protein 69	0.83	0.9	1.05	0.65
PPIP5K2	PPIP5K2;FLJ21506;FLJ23463;HISPPD1;IP7K2;KIAA0433;VIP2	diphosphoinositol pentakisphosphate kinase 2	0.83	0.85	1.05	0.6
BAI3	BAI3;KIAA0550;MGC133100	brain-specific angiogenesis inhibitor 3	0.82	0.83	1.05	0.75
ACSBG1	ACSBG1;BG;BG1;BGM;FLJ30320;GR-LACS;KIAA0631;LPD;MGC14352	acyl-CoA synthetase bubblegum family member 1	0.82	0.86	1.05	0.58
FNDC5	FNDC5;FRCP2	fibronectin type III domain containing 5	0.82	0.95	1.05	0.7
TNFSF10	TNFSF10;APO2L;Apo-2L;CD253;TL2;TRAIL	tumor necrosis factor (ligand) superfamily, member 10	0.82	0.82	1.05	0.6
CEACAM3	CEACAM3;CD66D;CEA;CGM1;MGC119875;W264;W282	carcinoembryonic antigen-related cell adhesion molecule 3	0.82	0.9	1.05	0.6
FOXI1	FOXI1;FKH10;FKHL10;FREAC-6;FREAC6;HFH-3;HFH3;MGC34197	forkhead box I1	0.82	0.84	1.05	0.59
CRTC3	CRTC3;FLJ21868;TORC3	CREB regulated transcription coactivator 3	0.82	0.84	1.05	0.63
SENP7	SENP7;KIAA1707;MGC157730	SUMO1/sentrin specific peptidase 7	0.82	0.79	1.05	0.6
OSTM1	OSTM1;GIPN;GL;OPTB5	osteopetrosis associated transmembrane protein 1	0.82	0.77	1.05	0.64
PHC1	PHC1;EDR1;HPH1;RAE28	polyhomeotic homolog 1 (Drosophila)	0.82	0.78	1.05	0.65
FAM114A2	FAM114A2;133K02;C5orf3	family with sequence similarity 114, member A2	0.82	0.84	1.05	0.63
CHRNA5	CHRNA5;LNCR2	cholinergic receptor, nicotinic, alpha 5	0.82	0.86	1.05	0.67
SIPA1L3	SIPA1L3;SPAL3	signal-induced proliferation-associated 1 like 3	0.82	0.82	1.05	0.69
PDK1	PDK1	pyruvate dehydrogenase kinase, isozyme 1	0.82	0.93	1.05	0.66
NECAP1	NECAP1;DKFZP566B183;MGC131900	NECAP endocytosis associated 1	0.82	0.9	1.05	0.63
STK17B	STK17B;DRAK2	serine/threonine kinase 17b	0.82	0.86	1.05	0.71
SRSF4	SRSF4;SFRS4;SRP75	serine/arginine-rich splicing factor 4	0.82	0.9	1.05	0.73
NCR3	NCR3;1C7;CD337;LY117;MALS;NKp30	natural cytotoxicity triggering receptor 3	0.81	0.8	1.05	0.63
ZNF562	ZNF562;FLJ20079;MGC126160	zinc finger protein 562	0.81	0.88	1.05	0.66
RUSC1	RUSC1;DKFZp761A1822;NESCA	RUN and SH3 domain containing 1	0.81	0.9	1.05	0.72
PADI2	PADI2;KIAA0994;PAD-H19;PAD2;PDI2	peptidyl arginine deiminase, type II	0.81	0.9	1.05	0.6
TRIT1	TRIT1;FLJ20061;IPT;MGC149242;MGC149243;MOD5	tRNA isopentenyltransferase 1	0.81	0.92	1.05	0.68
CHCHD10	CHCHD10;C22orf16;MGC70831;N27C7-4	coiled-coil-helix-coiled-coil-helix domain containing 10	0.81	0.82	1.05	0.59
LOC283116	LOC283116	tripartite motif-containing protein LOC642612-like	0.81	0.84	1.05	0.75
PRSS54	PRSS54;FLJ25339;KLKBL4	protease, serine, 54	0.81	0.86	1.05	0.67
PRRC2C	PRRC2C;BAT2-iso;BAT2D1;BAT2L2;XTP2	proline-rich coiled-coil 2C	0.81	0.76	1.05	0.64
GUCY2F	GUCY2F;CYGF;GC-F;GUC2DL;GUC2F;RETGC-2;ROS-GC2	guanylate cyclase 2F, retinal	0.8	0.82	1.05	0.73
LCTL	LCTL;FLJ33279;KLG;KLPH	lactase-like	0.8	0.78	1.05	0.62
SH3GL3	SH3GL3;CNSA3;EEN-2B-L3;EEN-B2;HsT19371;SH3D2C;SH3P13	SH3-domain GRB2-like 3	0.8	0.8	1.05	0.63
LRRC55	LRRC55;DKFZp667D0425;FLJ13339;FLJ45686	leucine rich repeat containing 55	0.8	0.9	1.05	0.63
MCTP2	MCTP2;FLJ11175;FLJ33303;MGC111402	multiple C2 domains, transmembrane 2	0.8	0.9	1.05	0.63
FRMD4A	FRMD4A;FLJ10210;FRMD4;KIAA1294;bA295P9.4	FERM domain containing 4A	0.8	0.74	1.05	0.59
PTPN5	PTPN5;FLJ14427;PTPSTEP;STEP	protein tyrosine phosphatase, non-receptor type 5 (striatum-enriched)	0.8	0.78	1.05	0.58
PRLHR	PRLHR;GPR10;GR3;MGC126539;MGC126541;PrRPR	prolactin releasing hormone receptor	0.8	0.77	1.05	0.58
SCO1	SCO1;SCOD1	SCO cytochrome oxidase deficient homolog 1 (yeast)	0.8	0.82	1.05	0.62
NFKBIB	NFKBIB;IKBB;TRIP9	nuclear factor of kappa light polypeptide gene enhancer in B-cells inhibitor, beta	0.8	0.88	1.05	0.58
HIST1H1A	HIST1H1A;H1.1;H1A;H1F1;HIST1;MGC126642;MGC138345	histone cluster 1, H1a	0.8	0.8	1.05	0.67
ITCH	ITCH;AIF4;AIP4;NAPP1;dJ468O1.1	itchy E3 ubiquitin protein ligase homolog (mouse)	0.8	0.78	1.05	0.58
DHX9	DHX9;DDX9;FLJ17406;LKP;NDH2;NDHII;RHA	DEAH (Asp-Glu-Ala-His) box polypeptide 9	0.8	0.86	1.05	0.68
NAA20	NAA20;NAT3;NAT5;dJ1002M8.1	N(alpha)-acetyltransferase 20, NatB catalytic subunit	0.8	0.81	1.05	0.58
AWAT2	AWAT2;DC4;DGAT2L4;MFAT;WS	acyl-CoA wax alcohol acyltransferase 2	0.8	0.79	1.05	0.67
MST4	MST4;MASK	serine/threonine protein kinase MST4	0.8	0.82	1.05	0.73
BRIX1	BRIX1;BRIX;BXDC2;FLJ11100	BRX1, biogenesis of ribosomes, homolog (S. cerevisiae)	0.8	0.88	1.05	0.63
SNTG1	SNTG1;G1SYN;SYN4	syntrophin, gamma 1	0.79	0.8	1.05	0.57
SERHL2	SERHL2;MGC149508;MGC157667;MGC157668;dJ222E13.1	serine hydrolase-like 2	0.79	0.84	1.05	0.65
TMEM216	TMEM216;MGC13379	transmembrane protein 216	0.79	0.82	1.05	0.6
TUBA8	TUBA8;TUBAL2	tubulin, alpha 8	0.79	0.92	1.05	0.63
CXorf56	CXorf56;FLJ22965	chromosome X open reading frame 56	0.79	0.86	1.05	0.64
NEIL3	NEIL3;FGP2;FLJ10858;FPG2;NEI3;hFPG2;hNEI3	nei endonuclease VIII-like 3 (E. coli)	0.79	0.75	1.05	0.63
SUMO1	SUMO1;DAP1;GMP1;OFC10;PIC1;SENP2;SMT3;SMT3C;SMT3H3;UBL1	SMT3 suppressor of mif two 3 homolog 1 (S. cerevisiae)	0.79	0.8	1.05	0.7
MFN2	MFN2;CMT2A;CMT2A2;CPRP1;HSG;KIAA0214;MARF	mitofusin 2	0.79	0.76	1.05	0.68
TMED9	TMED9;HSGP25L2G	transmembrane emp24 protein transport domain containing 9	0.79	0.9	1.05	0.66
HSPA9	HSPA9;CSA;GRP-75;GRP75;HSPA9B;MGC4500;MOT;MOT2;MTHSP75;PBP74	heat shock 70kDa protein 9 (mortalin)	0.79	0.82	1.05	0.61
RIMKLB	RIMKLB;FAM80B;KIAA1238;NAAGS;NAAGS-I	ribosomal modification protein rimK-like family member B	0.79	0.91	1.05	0.61
LOC155060	LOC155060;DKFZp667J212;DKFZp762P2111;FLJ16713;FLJ17438;FLJ17495;FLJ17722;FLJ25485;FLJ36716;FLJ38357;FLJ61016;MGC149135	AI894139 pseudogene	0.79	0.88	1.05	0.62
SEPSECS	SEPSECS;DKFZp434B1417;LP;MGC161491;PCH2D;SLA;SLA/LP	Sep (O-phosphoserine) tRNA:Sec (selenocysteine) tRNA synthase	0.78	0.82	1.05	0.59
FRMD6	FRMD6;C14orf31;EX1;MGC17921;Willin;c14_5320	FERM domain containing 6	0.78	0.78	1.05	0.6
RMI1	RMI1;BLAP75;C9orf76;FLJ12888;RP11-346I8.1	RMI1, RecQ mediated genome instability 1, homolog (S. cerevisiae)	0.78	0.77	1.05	0.62
ADM2	ADM2;AM2;FLJ21135;dJ579N16.4	adrenomedullin 2	0.78	0.83	1.05	0.57
MED31	MED31;3110004H13Rik;FLJ27436;FLJ36714;Soh1	mediator complex subunit 31	0.78	0.85	1.05	0.58

CARS	CARS;CARS1;CYSRS;MGC:11246	cysteinyl-tRNA synthetase	0.78	0.88	1.05	0.66
GRPEL1	GRPEL1;FLJ25609;HMGE	GrpE-like 1, mitochondrial (E. coli)	0.78	0.78	1.05	0.6
KLHL13	KLHL13;BKLHD2;FLJ10262;MGC74791	kelch-like 13 (Drosophila)	0.78	0.82	1.05	0.57
NUP93	NUP93;KIAA0095;MGC21106	nucleoporin 93kDa	0.78	0.74	1.05	0.59
MYO10	MYO10;FLJ10639;FLJ21066;FLJ22268;FLJ43256;KIAA0799;MGC131988	myosin X	0.78	0.9	1.05	0.62
MSN	MSN	moesin	0.78	0.84	1.05	0.64
IL3	IL3;IL-3;MCGF;MGC79398;MGC79399;MULTI-CSF	interleukin 3 (colony-stimulating factor, multiple)	0.77	0.82	1.05	0.65
TMEM217	TMEM217;C6orf128;dJ355M6.2	transmembrane protein 217	0.77	0.84	1.05	0.57
CGRRF1	CGRRF1;CGR19;RNF197	cell growth regulator with ring finger domain 1	0.77	0.8	1.05	0.71
HIST1H2AG	HIST1H2AG;H2A.1b;H2A/p;H2AFP;HIST1H2AI;HIST1H2AK;HIST1H2AL;HIST1H2AM;pH2A/f	histone cluster 1, H2ag	0.77	0.82	1.05	0.62
HIF1AN	HIF1AN;DKFZp762F1811;FIH1;FLJ20615;FLJ22027	hypoxia inducible factor 1, alpha subunit inhibitor	0.77	0.82	1.05	0.65
HIST1H2AK	HIST1H2AK;H2A/d;H2AFD;HIST1H2AG;HIST1H2AJ;HIST1H2AL;HIST1H2AM	histone cluster 1, H2ak	0.77	0.82	1.05	0.55
FTSJ1	FTSJ1;CDLIV;MRX44;MRX9;SPB1;TRM7	FtsJ homolog 1 (E. coli)	0.77	0.78	1.05	0.61
CLPTM1	CLPTM1	cleft lip and palate associated transmembrane protein 1	0.77	0.72	1.05	0.6
RPL13A	RPL13A;L13A;TSTA1	ribosomal protein L13a	0.77	0.82	1.05	0.63
CCDC158	CCDC158;FLJ25770;MGC35086	coiled-coil domain containing 158	0.77	0.86	1.05	0.57
GAPDHS	GAPDHS;GAPD2;GAPDH-2;GAPDS;HSD-35	glyceraldehyde-3-phosphate dehydrogenase, spermatogenic	0.76	0.75	1.05	0.66
OLIG3	OLIG3;Bhlhb7;bHLHe20	oligodendrocyte transcription factor 3	0.76	0.8	1.05	0.56
BCL2L10	BCL2L10;BCL-B;Boo;Diva;MGC129810;MGC129811	BCL2-like 10 (apoptosis facilitator)	0.76	0.78	1.05	0.55
CDH22	CDH22;C20orf25;MGC39564;dJ998H6.1	cadherin 22, type 2	0.76	0.78	1.05	0.58
CGB5	CGB5;HCG;MGC119822	chorionic gonadotropin, beta polypeptide 5	0.76	0.84	1.05	0.56
WFDC1	WFDC1;PS20	WAP four-disulfide core domain 1	0.76	0.83	1.05	0.56
ODF3L2	ODF3L2;C19orf19;FLJ40059;MGC129962;MGC129963	outer dense fiber of sperm tails 3-like 2	0.76	0.84	1.05	0.68
NME7	NME7;FLJ37194;MN23H7;nm23-H7	non-metastatic cells 7, protein expressed in (nucleoside-diphosphate kinase)	0.76	0.86	1.05	0.61
SDCBP2	SDCBP2;FLJ12256;SITAC;SITAC18;ST-2	syndecan binding protein (syntenin) 2	0.76	0.72	1.05	0.58
FSTL3	FSTL3;FLRG;FSRP	follistatin-like 3 (secreted glycoprotein)	0.76	0.82	1.05	0.65
INO80	INO80;INO80A;INOC1;hINO80	INO80 homolog (S. cerevisiae)	0.76	0.8	1.05	0.55
ZC3HC1	ZC3HC1;1110054L24Rik;NIPA	zinc finger, C3HC-type containing 1	0.76	0.85	1.05	0.59
MLF1IP	MLF1IP;CENP50;CENPU;CENPU50;FLJ23468;KLIP1;PBIP1	MLF1 interacting protein	0.76	0.86	1.05	0.58
TPRKB	TPRKB	TP53RK binding protein	0.76	0.81	1.05	0.58
ELF4	ELF4;ELFR;MEF	E74-like factor 4 (ets domain transcription factor)	0.76	0.77	1.05	0.56
TPBG	TPBG;5T4;5T4AG;M6P1	trophoblast glycoprotein	0.76	0.78	1.05	0.59
CHP	CHP;SLC9A1BP	calcium binding protein P22	0.76	0.84	1.05	0.56
DCAF10	DCAF10;FLJ23201;MGC10765;MGC131781;WDR32	DDB1 and CUL4 associated factor 10	0.76	0.81	1.05	0.6
IL18R1	IL18R1;CD218a;CDw218a;IL-1Rrp;IL18RA;IL1RRP	interleukin 18 receptor 1	0.75	0.73	1.05	0.58
ANKK1	ANKK1;PKK2	ankyrin repeat and kinase domain containing 1	0.75	0.78	1.05	0.65
CEP63	CEP63;FLJ13386;MGC78416	centrosomal protein 63kDa	0.75	0.77	1.05	0.58
TNFRSF8	TNFRSF8;CD30;D1S166E;Ki-1	tumor necrosis factor receptor superfamily, member 8	0.75	0.78	1.05	0.58
NAGS	NAGS;AGAS;ARGA;MGC133025	N-acetylglutamate synthase	0.75	0.84	1.05	0.59
ST3GAL6	ST3GAL6;SIAT10;ST3GALVI	ST3 beta-galactoside alpha-2,3-sialyltransferase 6	0.75	0.75	1.05	0.59
GNA11	GNA11;GNA-11	guanine nucleotide binding protein (G protein), alpha 11 (Gq class)	0.75	0.76	1.05	0.55
ALDH3A2	ALDH3A2;ALDH10;DKFZp686E23276;FALDH;FLJ20851;SLS	aldehyde dehydrogenase 3 family, member A2	0.75	0.75	1.05	0.59
RBFA	RBFA;C18orf22;FLJ18281;FLJ18505;FLJ21172;FLJ42604;FLJ97160;HsT169	ribosome binding factor A (putative)	0.75	0.76	1.05	0.6
DEFB103A	DEFB103A;BD-3;DEFB-3;DEFB103;DEFB3;HBD3;HBP-3;HBP3;hBD-3	defensin, beta 103A	0.74	0.78	1.05	0.53
CCL24	CCL24;Ckb-6;MPIF-2;MPIF2;SCYA24	chemokine (C-C motif) ligand 24	0.74	0.7	1.05	0.56
SFTPB	SFTPB;PSP-B;SFTB3;SFTP3;SMDP1;SP-B	surfactant protein B	0.74	0.76	1.05	0.63
ZNF136	ZNF136;pHZ-20	zinc finger protein 136	0.74	0.75	1.05	0.58
ACYP2	ACYP2;ACYM;ACYP	acylphosphatase 2, muscle type	0.74	0.75	1.05	0.59
RTN4R	RTN4R;NGR;NOGOR	reticulon 4 receptor	0.74	0.8	1.05	0.62
SEMA4A	SEMA4A;CORD10;FLJ12287;RP35;SEMAB;SEMB	sema domain, immunoglobulin domain (Ig), transmembrane domain (TM) and short cytoplasmic domain, (semaphorin) 4A	0.74	0.76	1.05	0.61
C19orf55	C19orf55;DKFZp434C2231;FLJ30657;MGC131952	chromosome 19 open reading frame 55	0.74	0.84	1.05	0.65
KLRAP1	KLRAP1;KLRA#;KLRA1;LY49L;Ly-49L;Ly49;MGC126520;MGC126522	killer cell lectin-like receptor subfamily A pseudogene 1	0.74	0.82	1.05	0.54
KCNJ1	KCNJ1;KIR1.1;ROMK;ROMK1	potassium inwardly-rectifying channel, subfamily J, member 1	0.73	0.76	1.05	0.6
C17orf58	C17orf58;MGC138278	chromosome 17 open reading frame 58	0.73	0.78	1.05	0.58
NDUFB6	NDUFB6;B17;CI;MGC13675	NADH dehydrogenase (ubiquinone) 1 beta subcomplex, 6, 17kDa	0.73	0.76	1.05	0.63
RAB24	RAB24	RAB24, member RAS oncogene family	0.73	0.8	1.05	0.57
ASH2L	ASH2L;ASH2;ASH2L1;ASH2L2;Bre2	ash2 (absent, small, or homeotic)-like (Drosophila)	0.73	0.68	1.05	0.57
MYH6	MYH6;ASD3;CMD1EE;CMH14;MYHC;MYHCA;SSS3;alpha-MHC	myosin, heavy chain 6, cardiac muscle, alpha	0.72	0.72	1.05	0.52
BEST4	BEST4;MGC126872;VMD2L2	bestrophin 4	0.72	0.78	1.05	0.58
GAB4	GAB4	GRB2-associated binding protein family, member 4	0.72	0.8	1.05	0.62
ZNF629	ZNF629;KIAA0326;ZNF;ZNF65	zinc finger protein 629	0.72	0.72	1.05	0.52
TRIOBP	TRIOBP;DFNB28;FLJ39315;KIAA1662;TARA;dJ37E16.4	TRIO and F-actin binding protein	0.72	0.81	1.05	0.62
PCTP	PCTP;PC-TP;STARD2	phosphatidylcholine transfer protein	0.72	0.76	1.05	0.53
CCDC130	CCDC130;MGC10471	coiled-coil domain containing 130	0.72	0.8	1.05	0.62
CECR5	CECR5	cat eye syndrome chromosome region, candidate 5	0.72	0.74	1.05	0.59
HDAC6	HDAC6;FLJ16239;HD6	histone deacetylase 6	0.72	0.8	1.05	0.6
ARHGAP26	ARHGAP26;FLJ42530;GRAF;GRAF1;KIAA0621;OPHN1L;OPHN1L1	Rho GTPase activating protein 26	0.72	0.84	1.05	0.61
VPS37B	VPS37B;FLJ12750	vacuolar protein sorting 37 homolog B (S. cerevisiae)	0.72	0.79	1.05	0.56
PCM1	PCM1;PTC4	pericentriolar material 1	0.72	0.74	1.05	0.55
CCDC58	CCDC58;FLJ33273	coiled-coil domain containing 58	0.72	0.73	1.05	0.56
FTSJD2	FTSJD2;FLJ22156;KIAA0082;MTr1;hMTr1	FtsJ methyltransferase domain containing 2	0.72	0.78	1.05	0.52
SEH1L	SEH1L;SEC13L;SEH1A;SEH1B;Seh1	SEH1-like (S. cerevisiae)	0.72	0.76	1.05	0.52
TMEM234	TMEM234;AASL548;C1orf91;FLJ90779;PRO1105;RP4-622L5;dJ622L5.7	transmembrane protein 234	0.72	0.73	1.05	0.55
OXT	OXT;MGC126890;MGC126892;OT;OT-NPI	oxytocin, prepropeptide	0.71	0.77	1.05	0.58
ARHGEF1	ARHGEF1;GEF1;LBCL2;LSC;P115-RHOGEF;SUB1.5	Rho guanine nucleotide exchange factor (GEF) 1	0.71	0.76	1.05	0.52
IQGAP1	IQGAP1;HUMORFA01;KIAA0051;SAR1;p195	IQ motif containing GTPase activating protein 1	0.71	0.74	1.05	0.52
PLGLB1	PLGLB1;PLGL;PLGP1;PRGB;PRP-B	plasminogen-like B1	0.71	0.74	1.05	0.52
TRPC3	TRPC3;TRP3	transient receptor potential cation channel, subfamily C, member 3	0.7	0.72	1.05	0.56
PROKR2	PROKR2;GPR73L1;GPR73b;GPRg2;KAL3;PKR2;dJ680N4.3	prokineticin receptor 2	0.7	0.72	1.05	0.65
PHYHIP	PHYHIP;DYRK1AP3;KIAA0273;PAHX-AP;PAHXAP1	phytanoyl-CoA 2-hydroxylase interacting protein	0.7	0.73	1.05	0.55
OTOA	OTOA;CT108;DFNB22;FLJ32773;MGC157747;MGC39813	otoancorin	0.7	0.75	1.05	0.58
KRTAP6-1	KRTAP6-1;C21orf103;KAP6.1	keratin associated protein 6-1	0.7	0.78	1.05	0.63
CSDC2	CSDC2;PIPPIN;dJ347H13.2	cold shock domain containing C2, RNA binding	0.7	0.71	1.05	0.54
ZNF193	ZNF193;PRD51;ZSCAN9	zinc finger protein 193	0.7	0.76	1.05	0.6
SNX33	SNX33;MGC32065;SH3PX3;SH3PXD3C;SNX30	sorting nexin 33	0.7	0.66	1.05	0.53
RCN3	RCN3;RLP49	reticulocalbin 3, EF-hand calcium binding domain	0.7	0.73	1.05	0.6
CEBPB	CEBPB;C/EBP-beta;CRP2;IL6DBP;LAP;MGC32080;NF-IL6;TCF5	CCAAT/enhancer binding protein (C/EBP), beta	0.7	0.78	1.05	0.59
PIP4K2B	PIP4K2B;PI5P4KB;PIP5K2B;PIP5KIIB;PIP5KIIbeta	phosphatidylinositol-5-phosphate 4-kinase, type II, beta	0.7	0.79	1.05	0.56
MT1A	MT1A;MGC32848;MT1;MT1S;MTC	metallothionein 1A	0.7	0.71	1.05	0.51
CC2D2B	CC2D2B;C10orf130;FLJ12141;bA248J23.4	coiled-coil and C2 domain containing 2B	0.69	0.76	1.05	0.51
ZNF32	ZNF32;KOX30	zinc finger protein 32	0.69	0.72	1.05	0.51
SPDYC	SPDYC;RINGOC;Ringo2	speedy homolog C (Xenopus laevis)	0.69	0.78	1.05	0.6
AGER	AGER;MGC22357;RAGE	advanced glycosylation end product-specific receptor	0.69	0.67	1.05	0.53
ANKRD13D	ANKRD13D;MGC50828	ankyrin repeat domain 13 family, member D	0.69	0.78	1.05	0.6
UBXN8	UBXN8;D8S2298E;REP8;UBXD6	UBX domain protein 8	0.69	0.7	1.05	0.54
CLDND2	CLDND2;MGC33839	claudin domain containing 2	0.69	0.72	1.05	0.53
TTLL6	TTLL6;FLJ35808;TTL.6	tubulin tyrosine ligase-like family, member 6	0.68	0.75	1.05	0.53
GNLY	GNLY;519;D2S69E;LAG-2;LAG2;NKG5;TLA519	granulysin	0.68	0.74	1.05	0.5
APITD1	APITD1;CENP-S;CENPS;FAAP16;MGC32686;MHF1	apoptosis-inducing, TAF9-like domain 1	0.68	0.74	1.05	0.52
B3GNT9	B3GNT9;MGC4655	UDP-GlcNAc:betaGal beta-1,3-N-acetylglucosaminyltransferase 9	0.68	0.73	1.05	0.49
C5orf44	C5orf44;FLJ13611;FLJ26957;MGC48585	chromosome 5 open reading frame 44	0.68	0.73	1.05	0.55
PDLIM2	PDLIM2;FLJ34715;MYSTIQUE;SLIM	PDZ and LIM domain 2 (mystique)	0.68	0.73	1.05	0.52
SLC41A3	SLC41A3;FLJ20473;SLC41A1-L2	solute carrier family 41, member 3	0.68	0.72	1.05	0.57
ZNF544	ZNF544	zinc finger protein 544	0.68	0.75	1.05	0.5
BTG2	BTG2;MGC126063;MGC126064;PC3;TIS21	BTG family, member 2	0.68	0.7	1.05	0.52
SLC25A15	SLC25A15;D13S327;HHH;ORC1;ORNT1	solute carrier family 25 (mitochondrial carrier; ornithine transporter) member 15	0.68	0.68	1.05	0.49
CIRBP	CIRBP;CIRP	cold inducible RNA binding protein	0.68	0.7	1.05	0.55
MITF	MITF;MI;WS2;WS2A;bHLHe32	microphthalmia-associated transcription factor	0.68	0.72	1.05	0.49
CCDC117	CCDC117;FLJ33814;dJ366L4.1	coiled-coil domain containing 117	0.68	0.7	1.05	0.52
PLAGL1	PLAGL1;DKFZp781P1017;LOT1;MGC126275;MGC126276;ZAC;ZAC1	pleiomorphic adenoma gene-like 1	0.67	0.77	1.05	0.54
CYP27C1	CYP27C1;FLJ16008	cytochrome P450, family 27, subfamily C, polypeptide 1	0.66	0.64	1.05	0.51
RHEBL1	RHEBL1;FLJ25797;MGC34869;RHEBL1c	Ras homolog enriched in brain like 1	0.66	0.71	1.05	0.51
GABRA5	GABRA5;MGC138184	gamma-aminobutyric acid (GABA) A receptor, alpha 5	0.66	0.68	1.05	0.53
GSTT1	GSTT1	glutathione S-transferase theta 1	0.66	0.68	1.05	0.5
COLEC11	COLEC11;CL-K1-I;CL-K1-II;CL-K1-IIa;CL-K1-IIb;CLK1;DKFZp686N1868;MGC3279	collectin sub-family member 11	0.66	0.72	1.05	0.48
LPAR5	LPAR5;GPR92;GPR93;KPG_010;LPA5	lysophosphatidic acid receptor 5	0.66	0.67	1.05	0.61
CNO	CNO;BCAS4L;FLJ11230	cappuccino homolog (mouse)	0.66	0.66	1.05	0.55
FDXR	FDXR;ADXR	ferredoxin reductase	0.66	0.68	1.05	0.53
TNRC6C	TNRC6C;FLJ20015;KIAA1582	trinucleotide repeat containing 6C	0.66	0.66	1.05	0.54
PTRH2	PTRH2;BIT1;FLJ32471;PTH2	peptidyl-tRNA hydrolase 2	0.66	0.69	1.05	0.52
PUM1	PUM1;HSPUM;KIAA0099;PUMH;PUMH1;PUML1	pumilio homolog 1 (Drosophila)	0.66	0.68	1.05	0.56
RHCE	RHCE;CD240CE;MGC103977;RH;RH30A;RHC;RHE;RHIXB;RHPI;Rh4;RhIVb(J);RhVI;RhVIII	Rh blood group, CcEe antigens	0.65	0.66	1.05	0.57
TMEM40	TMEM40;FLJ11036	transmembrane protein 40	0.65	0.68	1.05	0.49
ITPKA	ITPKA;IP3-3KA;IP3KA	inositol-trisphosphate 3-kinase A	0.65	0.68	1.05	0.56
LGALS9	LGALS9;HUAT;LGALS9A;MGC117375;MGC125973;MGC125974	lectin, galactoside-binding, soluble, 9	0.64	0.66	1.05	0.48
OR1D5	OR1D5;C17orf2;OR17-2;OR17-30;OR17-31	olfactory receptor, family 1, subfamily D, member 5	0.64	0.7	1.05	0.47
MCTP1	MCTP1;FLJ22344	multiple C2 domains, transmembrane 1	0.64	0.72	1.05	0.56
NCF2	NCF2;FLJ93058;NCF-2;NOXA2;P67-PHOX;P67PHOX	neutrophil cytosolic factor 2	0.64	0.64	1.05	0.47
DNAJC9	DNAJC9;HDJC9;JDD1;KIAA0974;SB73	DnaJ (Hsp40) homolog, subfamily C, member 9	0.64	0.66	1.05	0.49

IER3IP1	IER3IP1;MEDS;PRO2309	immediate early response 3 interacting protein 1	0.63	0.7	1.05	0.49
OR4F29	OR4F29;OR4F16;OR4F3;OR7-21	olfactory receptor, family 4, subfamily F, member 29	0.63	0.68	1.05	0.49
LYPD4	LYPD4;MGC42718;SMR	LY6/PLAUR domain containing 4	0.62	0.72	1.05	0.47
ABCB1	ABCB1;ABC20;CD243;CLCS;GP170;MDR1;MGC163296;P-GP;PGY1	ATP-binding cassette, sub-family B (MDR/TAP), member 1	0.62	0.64	1.05	0.5
GPR50	GPR50;H9;MGC125342;Mel1c	G protein-coupled receptor 50	0.62	0.64	1.05	0.57
GREB1	GREB1;KIAA0575	growth regulation by estrogen in breast cancer 1	0.62	0.63	1.05	0.51
FKBP10	FKBP10;FKBP65;FLJ20683;FLJ22041;FLJ23833;OI6;PPIASE;hFKBP65	FK506 binding protein 10, 65 kDa	0.62	0.64	1.05	0.45
CCDC41	CCDC41;MGC149726;NY-REN-58	coiled-coil domain containing 41	0.62	0.66	1.05	0.47
GRIK4	GRIK4;EAA1;GRIK;KA1	glutamate receptor, ionotropic, kainate 4	0.62	0.67	1.05	0.52
C1RL	C1RL;C1RL1;C1RLP;C1r-LP;CLSPa	complement component 1, r subcomponent-like	0.62	0.6	1.05	0.44
TSPYL4	TSPYL4;KIAA0721;dJ486I3.2	TSPY-like 4	0.62	0.64	1.05	0.44
IQSEC3	IQSEC3;KIAA1110;MGC30156	IQ motif and Sec7 domain 3	0.62	0.66	1.05	0.46
CBX1	CBX1;CBX;HP1-BETA;HP1Hs-beta;HP1Hsbeta;M31;MOD1;p25beta	chromobox homolog 1	0.62	0.58	1.05	0.48
THUMPD1	THUMPD1;DKFZp686C1054	THUMP domain containing 1	0.62	0.64	1.05	0.48
RNF19A	RNF19A;DKFZp566B1346;DORFIN;RNF19	ring finger protein 19A	0.62	0.64	1.05	0.57
C10orf58	C10orf58;FLJ12536;FLJ14952;FLJ44312;MGC4248;PAMM	chromosome 10 open reading frame 58	0.62	0.67	1.05	0.51
ATXN10	ATXN10;E46L;FLJ37990;HUMEEP;SCA10	ataxin 10	0.62	0.7	1.05	0.49
SEC22B	SEC22B;ERS-24;SEC22L1	SEC22 vesicle trafficking protein homolog B (S. cerevisiae) (gene/pseudogene)	0.62	0.71	1.05	0.47
FAM65B	FAM65B;C6orf32;DIFF40;DIFF48;KIAA0386;PL48	family with sequence similarity 65, member B	0.61	0.68	1.05	0.47
PDE1B	PDE1B;PDE1B1;PDES1B	phosphodiesterase 1B, calmodulin-dependent	0.61	0.68	1.05	0.45
MSL3	MSL3;DKFZp586J1822;MSL3L1	male-specific lethal 3 homolog (Drosophila)	0.61	0.66	1.05	0.5
OR10C1	OR10C1;OR10C1P;OR10C2;OR6-31;hs6M1-17	olfactory receptor, family 10, subfamily C, member 1	0.84	0.92	1.06	0.63
SFRP2	SFRP2;FRP-2;SARP1;SDF-5	secreted frizzled-related protein 2	0.84	0.9	1.06	0.7
PTPRH	PTPRH;FLJ39938;MGC133058;MGC133059;SAP1	protein tyrosine phosphatase, receptor type, H	0.84	0.82	1.06	0.62
CNTFR	CNTFR;MGC1774	ciliary neurotrophic factor receptor	0.84	0.84	1.06	0.66
TTC12	TTC12;FLJ13859;FLJ20535;TPARM	tetratricopeptide repeat domain 12	0.84	0.94	1.06	0.66
SSTR1	SSTR1;SRIF-2	somatostatin receptor 1	0.84	0.87	1.06	0.79
INPP5E	INPP5E;CORS1;CPD4;JBTS1;MGC117201;MORMS;PPI5PIV	inositol polyphosphate-5-phosphatase, 72 kDa	0.84	0.83	1.06	0.63
UNC119	UNC119;HRG4;POC7;POC7A	unc-119 homolog (C. elegans)	0.84	0.92	1.06	0.69
CDKN1A	CDKN1A;CAP20;CDKN1;CIP1;MDA-6;P21;SDI1;WAF1;p21CIP1	cyclin-dependent kinase inhibitor 1A (p21, Cip1)	0.84	0.95	1.06	0.68
FAM171A1	FAM171A1;C10orf38;FLJ12884;MGC130014;MGC130015	family with sequence similarity 171, member A1	0.84	0.8	1.06	0.67
HPS3	HPS3;DKFZp686F0413;FLJ22704;SUTAL	Hermansky-Pudlak syndrome 3	0.84	0.97	1.06	0.71
IL1R1	IL1R1;CD121A;D2S1473;IL-1R-alpha;IL1R;IL1RA;P80	interleukin 1 receptor, type I	0.84	0.92	1.06	0.72
C14orf182	C14orf182;MGC149108	chromosome 14 open reading frame 182	0.84	0.88	1.06	0.73
PRSS57	PRSS57;PRSSL1;UNQ782	protease, serine, 57	0.84	0.9	1.06	0.71
TNFSF13B	TNFSF13B;BAFF;BLYS;CD257;DTL;TALL-1;TALL1;THANK;TNFSF20;ZTNF4	tumor necrosis factor (ligand) superfamily, member 13b	0.83	0.86	1.06	0.66
SLC24A6	SLC24A6;FLJ22233;NCKX6;NCLX	solute carrier family 24 (sodium/potassium/calcium exchanger), member 6	0.83	0.92	1.06	0.75
ASB1	ASB1;ASB-1;KIAA1146;MGC11165	ankyrin repeat and SOCS box containing 1	0.83	0.84	1.06	0.6
IMPDH1	IMPDH1;DKFZp781N0678;IMPD;IMPD1;LCA11;RP10;sWSS2608	IMP (inosine 5'-monophosphate) dehydrogenase 1	0.83	0.84	1.06	0.61
ZP1	ZP1;MGC87693	zona pellucida glycoprotein 1 (sperm receptor)	0.82	0.9	1.06	0.68
HS3ST5	HS3ST5;3-OST-5;3OST5;HS3OST5;NBLA04021	heparan sulfate (glucosamine) 3-O-sulfotransferase 5	0.82	0.85	1.06	0.62
C1orf64	C1orf64;MGC24047;RP11-5P18.4	chromosome 1 open reading frame 64	0.82	0.93	1.06	0.62
HIPK4	HIPK4;FLJ32818	homeodomain interacting protein kinase 4	0.82	0.94	1.06	0.67
KCNJ4	KCNJ4;HIR;HIRK2;HRK1;IRK-3;IRK3;Kir2.3;MGC142066;MGC142068	potassium inwardly-rectifying channel, subfamily J, member 4	0.82	0.86	1.06	0.68
CISH	CISH;CIS;CIS-1;G18;SOCS	cytokine inducible SH2-containing protein	0.82	0.84	1.06	0.6
GIGYF1	GIGYF1;GYF1;PERQ1	GRB10 interacting GYF protein 1	0.82	0.86	1.06	0.6
ANK3	ANK3;ANKYRIN-G;FLJ45464	ankyrin 3, node of Ranvier (ankyrin G)	0.82	0.86	1.06	0.67
CENPT	CENPT;C16orf56;CENP-T;FLJ13111;FLJ43376	centromere protein T	0.82	0.82	1.06	0.67
NPPC	NPPC;CNP;CNP2	natriuretic peptide C	0.82	0.9	1.06	0.6
FBLN1	FBLN1;FBLN;FIBL1	fibulin 1	0.82	0.9	1.06	0.61
RAPGEF3	RAPGEF3;CAMP-GEFI;EPAC;EPAC1;HSU79275;MGC21410;bcm910	Rap guanine nucleotide exchange factor (GEF) 3	0.82	0.89	1.06	0.61
APBB2	APBB2;DKFZp434E033;FE65L;FE65L1;MGC35575	amyloid beta (A4) precursor protein-binding, family B, member 2	0.82	0.9	1.06	0.67
PARVB	PARVB;CGI-56;FLJ17029	parvin, beta	0.82	0.9	1.06	0.61
JMJD8	JMJD8;C16orf20	jumonji domain containing 8	0.82	0.82	1.06	0.7
RETSAT	RETSAT;FLJ20296	retinol saturase (all-trans-retinol 13,14-reductase)	0.82	0.88	1.06	0.63
PSMD5	PSMD5;KIAA0072;MGC23145;S5B	proteasome (prosome, macropain) 26S subunit, non-ATPase, 5	0.82	0.88	1.06	0.59
PLD3	PLD3;HU-K4;HUK4	phospholipase D family, member 3	0.82	0.85	1.06	0.66
MRPL9	MRPL9;L9mt	mitochondrial ribosomal protein L9	0.82	0.84	1.06	0.62
DUSP1	DUSP1;CL100;HVH1;MKP-1;MKP1;PTPN10	dual specificity phosphatase 1	0.82	0.87	1.06	0.67
ADCY8	ADCY8;AC8;ADCY3;HBAC1	adenylate cyclase 8 (brain)	0.81	0.86	1.06	0.58
COX7A1	COX7A1;COX7A;COX7AH;COX7AM	cytochrome c oxidase subunit VIIa polypeptide 1 (muscle)	0.81	0.88	1.06	0.67
SHANK2	SHANK2;AUTS17;CORTBP1;CTTNBP1;ProSAP1;SHANK;SPANK-3	SH3 and multiple ankyrin repeat domains 2	0.81	0.89	1.06	0.67
C19orf44	C19orf44;FLJ21742;FLJ22492	chromosome 19 open reading frame 44	0.81	0.92	1.06	0.71
ITPR3	ITPR3;FLJ36205;IP3R;IP3R3	inositol 1,4,5-trisphosphate receptor, type 3	0.81	0.86	1.06	0.66
RCN1	RCN1;FLJ37041;FLJ55835;PIG20;RCAL;RCN	reticulocalbin 1, EF-hand calcium binding domain	0.81	0.78	1.06	0.62
PPBP	PPBP;B-TG1;Beta-TG;CTAP-III;CTAP3;CTAPIII;CXCL7;LA-PF4;LDGF;MDGF;NAP-2;PBP;SCAR10;SCYB7;TC1;TC2;TGB;TGB1;THBGB;THBGB1	pro-platelet basic protein (chemokine (C-X-C motif) ligand 7)	0.8	0.79	1.06	0.7
SPRY3	SPRY3;HSPRY3	sprouty homolog 3 (Drosophila)	0.8	0.86	1.06	0.66
AICDA	AICDA;AID;ARP2;CDA2;HIGM2	activation-induced cytidine deaminase	0.8	0.86	1.06	0.67
CAPN10	CAPN10;CANP10;NIDDM1	calpain 10	0.8	0.84	1.06	0.63
LST1	LST1;B144;D6S49E;LST-1;MGC119006;MGC119007	leukocyte specific transcript 1	0.8	0.82	1.06	0.62
FIZ1	FIZ1;FLJ00416;FLJ14768;ZNF798	FLT3-interacting zinc finger 1	0.8	0.92	1.06	0.7
RBM17	RBM17;DKFZp686F13131;MGC14439;SPF45	RNA binding motif protein 17	0.8	0.87	1.06	0.66
TRAPPC2L	TRAPPC2L;FLJ44827;HSPC176;MGC111156	trafficking protein particle complex 2-like	0.8	0.84	1.06	0.61
CCDC14	CCDC14;DKFZp434L1050;FLJ12892;FLJ41065	coiled-coil domain containing 14	0.8	0.81	1.06	0.66
KCNIP1	KCNIP1;KCHIP1;MGC95;VABP	Kv channel interacting protein 1	0.8	0.88	1.06	0.69
NDRG3	NDRG3;FLJ13556	NDRG family member 3	0.8	0.88	1.06	0.71
HSPA1B	HSPA1B;FLJ54328;HSP70-1B;HSP70-2;HSPA1A	heat shock 70kDa protein 1B	0.8	0.84	1.06	0.63
ECHDC1	ECHDC1;DKFZp762M1110;FLJ40827;dJ351K20.2	enoyl CoA hydratase domain containing 1	0.8	0.84	1.06	0.59
LGR4	LGR4;GPR48	leucine-rich repeat containing G protein-coupled receptor 4	0.8	0.82	1.06	0.74
DNAJA2	DNAJA2;CPR3;DJ3;DJA2;DNAJ;DNJ3;HIRIP4;PRO3015;RDJ2	DnaJ (Hsp40) homolog, subfamily A, member 2	0.8	0.84	1.06	0.65
SLC2A4	SLC2A4;GLUT4	solute carrier family 2 (facilitated glucose transporter), member 4	0.79	0.83	1.06	0.62
NAPRT1	NAPRT1;PP3856	nicotinate phosphoribosyltransferase domain containing 1	0.79	0.8	1.06	0.62
C12orf41	C12orf41;FLJ12670;FLJ20436;NSL2	chromosome 12 open reading frame 41	0.79	0.82	1.06	0.62
IRAK2	IRAK2;IRAK-2;MGC150550	interleukin-1 receptor-associated kinase 2	0.78	0.83	1.06	0.69
PARD6A	PARD6A;PAR-6A;PAR6;PAR6C;PAR6alpha;TAX40;TIP-40	par-6 partitioning defective 6 homolog alpha (C. elegans)	0.78	0.82	1.06	0.56
NGFR	NGFR;CD271;Gp80-LNGFR;TNFRSF16;p75(NTR);p75NTR	nerve growth factor receptor	0.78	0.82	1.06	0.55
SOCS3	SOCS3;ATOD4;CIS3;Cish3;MGC71791;SOCS-3;SSI-3;SSI3	suppressor of cytokine signaling 3	0.78	0.8	1.06	0.57
MECP2	MECP2;AUTSX3;DKFZp686A24160;MRX16;MRX79;MRXS13;MRXSL;PPMX;RS;RTS;RTT	methyl CpG binding protein 2 (Rett syndrome)	0.78	0.76	1.06	0.67
NT5C3L	NT5C3L;MGC20781;MGC21375	5'-nucleotidase, cytosolic III-like	0.78	0.83	1.06	0.6
HIST1H3A	HIST1H3A;H3/A;H3FA	histone cluster 1, H3a	0.78	0.88	1.06	0.71
NPB	NPB;L7;PPL7;PPNPB	neuropeptide B	0.78	0.82	1.06	0.61
AJAP1	AJAP1;MOT8;RP3-426F10.1;SHREW-1;SHREW1	adherens junctions associated protein 1	0.78	0.83	1.06	0.6
DHX29	DHX29;DDX29;FLJ21492	DEAH (Asp-Glu-Ala-His) box polypeptide 29	0.78	0.89	1.06	0.59
VDAC3	VDAC3;HD-VDAC3;VDAC-3	voltage-dependent anion channel 3	0.78	0.88	1.06	0.68
PPP2R1A	PPP2R1A;MGC786;PP2A-Aalpha;PP2AAALPHA;PR65A	protein phosphatase 2, regulatory subunit A, alpha	0.78	0.77	1.06	0.58
UTP14C	UTP14C;2700066J21Rik;KIAA0266;MGC104971;UTP14B	UTP14, U3 small nucleolar ribonucleoprotein, homolog C (yeast)	0.78	0.84	1.06	0.57
MTFP1	MTFP1;MTP18	mitochondrial fission process 1	0.78	0.91	1.06	0.67
RTEL1	RTEL1;C20orf41;DKFZp434C013;KIAA1088;NHL;RTEL;bK3184A7.3	regulator of telomere elongation helicase 1	0.77	0.82	1.06	0.55
SMG5	SMG5;EST1B;FLJ12287;FLJ34864;KIAA1089;LPTS-RP1;LPTSRP1;SMG-5	smg-5 homolog, nonsense mediated mRNA decay factor (C. elegans)	0.77	0.88	1.06	0.61
LEPREL4	LEPREL4;NO55;NOL55;SC65	leprecan-like 4	0.77	0.79	1.06	0.57
FAM186B	FAM186B;C12orf25;DKFZp434J0113;DKFZp434N2350	family with sequence similarity 186, member B	0.77	0.78	1.06	0.59
C8orf44	C8orf44;FLJ11267	chromosome 8 open reading frame 44	0.76	0.76	1.06	0.58
TMIE	TMIE;DFNB6	transmembrane inner ear	0.76	0.82	1.06	0.63
HDGFL1	HDGFL1;PWWP1;dJ309H15.1	hepatoma derived growth factor-like 1	0.76	0.82	1.06	0.62
HACE1	HACE1	HECT domain and ankyrin repeat containing, E3 ubiquitin protein ligase 1	0.76	0.74	1.06	0.55
ZNF621	ZNF621;DKFZp686B11107;FLJ23786;FLJ45246;MGC126668	zinc finger protein 621	0.76	0.85	1.06	0.6
RXRB	RXRB;DAUDI6;H-2RIIBP;MGC1831;NR2B2;RCoR-1	retinoid X receptor, beta	0.76	0.86	1.06	0.65
HELZ	HELZ;DHRC;DKFZp586G1924;DRHC;HUMORF5;KIAA0054;MGC163454	helicase with zinc finger	0.76	0.75	1.06	0.6
STXBP1	STXBP1;FLJ37475;MUNC18-1;NSEC1;P67;RBSEC1;UNC18	syntaxin binding protein 1	0.76	0.82	1.06	0.62
UROD	UROD;PCT;UPD	uroporphyrinogen decarboxylase	0.76	0.88	1.06	0.61
BCL7C	BCL7C	B-cell CLL/lymphoma 7C	0.76	0.84	1.06	0.56
BET1	BET1;DKFZp781C0425;HBET1	blocked early in transport 1 homolog (S. cerevisiae)	0.76	0.73	1.06	0.61
CDH6	CDH6;CAD6;DKFZp564N1116;FLJ14176;KCAD	cadherin 6, type 2, K-cadherin (fetal kidney)	0.76	0.76	1.06	0.59
NANOG	NANOG	Nanog homeobox	0.75	0.81	1.06	0.64
SYNJ1	SYNJ1;INPP5G	synaptojanin 1	0.75	0.79	1.06	0.62
C16orf57	C16orf57;FLJ13154;HVSL1;PN	chromosome 16 open reading frame 57	0.75	0.78	1.06	0.59
KPNA6	KPNA6;FLJ11249;IPOA7;KPNA7;MGC17918	karyopherin alpha 6 (importin alpha 7)	0.75	0.76	1.06	0.55
DPPA5	DPPA5;ESG1	developmental pluripotency associated 5	0.74	0.81	1.06	0.61
SERPINA5	SERPINA5;PAI-3;PAI3;PCI;PLANH3;PROCI	serpin peptidase inhibitor, clade A (alpha-1 antiproteinase, antitrypsin), member 5	0.74	0.74	1.06	0.59
TBC1D29	TBC1D29;DKFZp434O047	TBC1 domain family, member 29	0.74	0.78	1.06	0.53
FAM20A	FAM20A;AIGFS;DKFZp434F2322;FP2747	family with sequence similarity 20, member A	0.74	0.76	1.06	0.64
SUSD3	SUSD3;MGC26847	sushi domain containing 3	0.74	0.82	1.06	0.58
TNFRSF11B	TNFRSF11B;MGC29565;OCIF;OPG;TR1	tumor necrosis factor receptor superfamily, member 11b	0.74	0.84	1.06	0.55
MAP1LC3A	MAP1LC3A;ATG8E;LC3;LC3A;MAP1ALC3;MAP1BLC3	microtubule-associated protein 1 light chain 3 alpha	0.74	0.86	1.06	0.63
TMEM105	TMEM105;FLJ38792	transmembrane protein 105	0.74	0.86	1.06	0.63
ELMOD2	ELMOD2;9830169G11Rik;FLJ35768;FLJ38038;MGC10084	ELMO/CED-12 domain containing 2	0.74	0.84	1.06	0.64
MAFK	MAFK;FLJ32205;MGC71717;NFE2U;P18	v-maf musculoaponeurotic fibrosarcoma oncogene homolog K (avian)	0.74	0.78	1.06	0.52
KIF15	KIF15;FLJ25667;HKLP2;KNSL7;NY-BR-62	kinesin family member 15	0.74	0.78	1.06	0.61
NCAPH2	NCAPH2;CAPH2;MGC15858;MGC18000;MGC2455;MGC4133;MGC5305;MGC8640	non-SMC condensin II complex, subunit H2	0.74	0.86	1.06	0.57

TSPO	TSPO;BPBS;BZRP;DBI;IBP;MBR;PBR;PBS;PKBS;PTBR;mDRC;pk18	translocator protein (18kDa)	0.74	0.82	1.06	0.63
HMGB3	HMGB3;HMG-2a;HMG-4;HMG2A;HMG4;MGC90319	high mobility group box 3	0.74	0.77	1.06	0.62
LYAR	LYAR;FLJ20425;ZLYAR	Ly1 antibody reactive homolog (mouse)	0.74	0.7	1.06	0.56
TNPO1	TNPO1;IPO2;KPNB2;MIP;MIP1;TRN	transportin 1	0.74	0.76	1.06	0.53
FAM195B	FAM195B;DKFZp686A17165;MGC133184	family with sequence similarity 195, member B	0.74	0.82	1.06	0.58
CHST15	CHST15;BRAG;DKFZp781H1369;GALNAC4S-6ST;KIAA0598;MGC34346;RP11-47G11.1	carbohydrate (N-acetylgalactosamine 4-sulfate 6-O) sulfotransferase 15	0.74	0.76	1.06	0.59
GUCY1A2	GUCY1A2;GC-SA2;GUC1A2	guanylate cyclase 1, soluble, alpha 2	0.73	0.75	1.06	0.55
GFRA2	GFRA2;GDNFRB;NRTNR-ALPHA;NTNRA;RETL2;TRNR2	GDNF family receptor alpha 2	0.73	0.74	1.06	0.6
KRBA2	KRBA2	KRAB-A domain containing 2	0.73	0.73	1.06	0.59
HSD11B2	HSD11B2;AME;AME1;HSD11K;HSD2;SDR9C3	hydroxysteroid (11-beta) dehydrogenase 2	0.73	0.75	1.06	0.59
SPATA2	SPATA2;FLJ13167;KIAA0757;PD1;tamo	spermatogenesis associated 2	0.73	0.8	1.06	0.6
LY6D	LY6D;E48;Ly-6D	lymphocyte antigen 6 complex, locus D	0.73	0.75	1.06	0.57
GSTT2	GSTT2;GSTT2B;MGC182032	glutathione S-transferase theta 2	0.73	0.76	1.06	0.65
ETFB	ETFB;MADD	electron-transfer-flavoprotein, beta polypeptide	0.73	0.71	1.06	0.62
NPEPPS	NPEPPS;AAP-S;MP100;PSA	aminopeptidase puromycin sensitive	0.73	0.76	1.06	0.57
VHLL	VHLL;VHLP;VLP	von Hippel-Lindau tumor suppressor-like	0.72	0.78	1.06	0.59
CCKAR	CCKAR;CCK-A;CCK1-R;CCKRA	cholecystokinin A receptor	0.72	0.74	1.06	0.67
PTGS2	PTGS2;COX-2;COX2;GRIPGHS;PGG/HS;PGHS-2;PHS-2;hCox-2	prostaglandin-endoperoxide synthase 2 (prostaglandin G/H synthase and cyclooxygenase)	0.72	0.74	1.06	0.54
CRHR2	CRHR2;CRF-RB;CRF2;CRFR2;HM-CRF	corticotropin releasing hormone receptor 2	0.72	0.74	1.06	0.66
AMPD2	AMPD2	adenosine monophosphate deaminase 2	0.72	0.71	1.06	0.63
MUC20	MUC20;FLJ14408;FLJ53153;KIAA1359;MUC-20	mucin 20, cell surface associated	0.72	0.78	1.06	0.6
UTP23	UTP23;C8orf53;MGC14595	UTP23, small subunit (SSU) processome component, homolog (yeast)	0.72	0.82	1.06	0.63
HIST1H3H	HIST1H3H;FLJ92264;H3/k;H3F1K;H3FK;HIST1H3A;HIST1H3B;HIST1H3C;HIST1H3D;HIST1H3E;HIST1H3F;HIST1H3G;HIST1H3I;HIST1H3J	histone cluster 1, H3h	0.72	0.78	1.06	0.59
FBXW2	FBXW2;FBW2;Fwd2;MGC117371;Md6	F-box and WD repeat domain containing 2	0.72	0.73	1.06	0.53
CTR9	CTR9;KIAA0155;SH2BP1;TSBP;p150;p150TSP	Ctr9, Paf1/RNA polymerase II complex component, homolog (S. cerevisiae)	0.72	0.74	1.06	0.56
RABGGTB	RABGGTB;GGTB	Rab geranylgeranyltransferase, beta subunit	0.72	0.76	1.06	0.51
MT1X	MT1X;MT-1l;MT1	metallothionein 1X	0.72	0.78	1.06	0.53
KIAA0930	KIAA0930;C22orf9	KIAA0930	0.72	0.8	1.06	0.57
KRTAP19-1	KRTAP19-1;KAP19.1	keratin associated protein 19-1	0.71	0.78	1.06	0.53
MRGPRX4	MRGPRX4;GPCR;MGC129753;MGC129754;MRGX4;SNSR6	MAS-related GPR, member X4	0.71	0.7	1.06	0.53
XPNPEP2	XPNPEP2;APP2	X-prolyl aminopeptidase (aminopeptidase P) 2, membrane-bound	0.71	0.74	1.06	0.56
AMIGO3	AMIGO3;AMIGO-3;MGC120552	adhesion molecule with Ig-like domain 3	0.71	0.78	1.06	0.59
POLL	POLL;BETAN;FLJ46002;POLKAPPA	polymerase (DNA directed), lambda	0.71	0.82	1.06	0.61
METTL1	METTL1;C12orf1;FLJ95748;TRM8;YDL201w	methyltransferase like 1	0.71	0.82	1.06	0.61
NMI	NMI	N-myc (and STAT) interactor	0.71	0.72	1.06	0.56
LRRTM4	LRRTM4;FLJ12568;MGC120633;MGC120636	leucine rich repeat transmembrane neuronal 4	0.7	0.7	1.06	0.59
CNR2	CNR2;CB2;CX5	cannabinoid receptor 2 (macrophage)	0.7	0.69	1.06	0.52
SPNS3	SPNS3;MGC29671	spinster homolog 3 (Drosophila)	0.7	0.78	1.06	0.6
HRH3	HRH3;GPCR97;HH3R	histamine receptor H3	0.7	0.72	1.06	0.65
SUMF1	SUMF1;AAPA3037;FGE;MGC131853;MGC150436	sulfatase modifying factor 1	0.7	0.66	1.06	0.56
RPAP1	RPAP1;DKFZp727M111;FLJ12732;KIAA1403;MGC858	RNA polymerase II associated protein 1	0.7	0.72	1.06	0.57
CTDSP1	CTDSP1;NIF3;NLI-IF;NLIIF;SCP1	CTD (carboxy-terminal domain, RNA polymerase II, polypeptide A) small phosphatase 1	0.7	0.7	1.06	0.53
PLAGL2	PLAGL2;FLJ23283;ZNF900	pleiomorphic adenoma gene-like 2	0.7	0.75	1.06	0.58
POP7	POP7;0610037N12Rik;RPP2;RPP20	processing of precursor 7, ribonuclease P/MRP subunit (S. cerevisiae)	0.7	0.79	1.06	0.59
SLC25A44	SLC25A44;FLJ90431;KIAA0446;RP11-54H19.3	solute carrier family 25, member 44	0.7	0.78	1.06	0.52
HIST1H4D	HIST1H4D;H4/b;H4FB;dJ221C16.9	histone cluster 1, H4d	0.7	0.77	1.06	0.52
PAK4	PAK4	p21 protein (Cdc42/Rac)-activated kinase 4	0.7	0.74	1.06	0.61
ZDHHC14	ZDHHC14;FLJ20984;NEW1CP	zinc finger, DHHC-type containing 14	0.69	0.76	1.06	0.54
SLMO1	SLMO1;C18orf43;DKFZp547K106;FLJ31484;HFL-EDDG1;MGC120170;PRELID3A	slowmo homolog 1 (Drosophila)	0.69	0.8	1.06	0.53
KRTCAP2	KRTCAP2;KCP2	keratinocyte associated protein 2	0.69	0.74	1.06	0.53
CD9	CD9;BTCC-1;DRAP-27;FLJ99568;MIC3;MRP-1;TSPAN-29;TSPAN29	CD9 molecule	0.69	0.77	1.06	0.54
SLC3A1	SLC3A1;ATR1;CSNU1;D2H;FLJ34681;NBAT;RBAT	solute carrier family 3 (cystine, dibasic and neutral amino acid transporters, activator of cystine, dibasic and neutral amino acid transport), member 1	0.68	0.79	1.06	0.59
SIGLEC11	SIGLEC11	sialic acid binding Ig-like lectin 11	0.68	0.64	1.06	0.51
HS3ST3B1	HS3ST3B1;30ST3B1;3OST3B1	heparan sulfate (glucosamine) 3-O-sulfotransferase 3B1	0.68	0.74	1.06	0.58
SLC24A1	SLC24A1;CSNB1D;HsT17412;KIAA0702;NCKX;NCKX1;RODX	solute carrier family 24 (sodium/potassium/calcium exchanger), member 1	0.68	0.72	1.06	0.56
OBSCN	OBSCN;ARHGEF30;DKFZp666E245;FLJ14124;KIAA1556;KIAA1639;MGC120409;MGC120410;MGC120411;MGC120412;MGC138590;UNC89	obscurin, cytoskeletal calmodulin and titin-interacting RhoGEF	0.68	0.76	1.06	0.62
RGS2	RGS2;G0S8	regulator of G-protein signaling 2, 24kDa	0.68	0.75	1.06	0.61
MAFF	MAFF;U-MAF;hMafF	v-maf musculoaponeurotic fibrosarcoma oncogene homolog F (avian)	0.68	0.68	1.06	0.54
ARL4C	ARL4C;ARL7;LAK	ADP-ribosylation factor-like 4C	0.68	0.76	1.06	0.62
RUNX1	RUNX1;AML1;AML1-EVI-1;AMLCR1;CBFA2;EVI-1;PEBP2aB	runt-related transcription factor 1	0.68	0.69	1.06	0.54
CLP1	CLP1;HEAB;hClp1	CLP1, cleavage and polyadenylation factor I subunit, homolog (S. cerevisiae)	0.68	0.71	1.06	0.53
C11orf61	C11orf61;FLJ23342	chromosome 11 open reading frame 61	0.68	0.72	1.06	0.52
LPHN2	LPHN2;CIRL2;CL2;LEC1;LPHH1	latrophilin 2	0.68	0.7	1.06	0.63
DDX1	DDX1;DBP-RB;UKVH5d	DEAD (Asp-Glu-Ala-Asp) box polypeptide 1	0.68	0.72	1.06	0.56
GAGE6	GAGE6;CT4.6	G antigen 6	0.68	0.7	1.06	0.54
OVCA2	OVCA2	ovarian tumor suppressor candidate 2	0.68	0.7	1.06	0.61
CYP8B1	CYP8B1;CP8B;CYP12;FLJ17826	cytochrome P450, family 8, subfamily B, polypeptide 1	0.67	0.76	1.06	0.5
FRMPD1	FRMPD1;FRMD2;KIAA0967;MGC138833	FERM and PDZ domain containing 1	0.67	0.64	1.06	0.53
CDRT1	CDRT1;C170RF1;C17ORF1;C17ORF1A;DKFZp434O1826;HREP;SM25H2	CMT1A duplicated region transcript 1	0.67	0.74	1.06	0.56
DNAJC16	DNAJC16;DKFZp686G1298;DKFZp686N0387;DKFZp781I1547;KIAA0962	DnaJ (Hsp40) homolog, subfamily C, member 16	0.67	0.73	1.06	0.55
KRT24	KRT24;FLJ20261;K24;KA24;MGC138169;MGC138173	keratin 24	0.66	0.71	1.06	0.51
ZMYND15	ZMYND15;DKFZp434N127	zinc finger, MYND-type containing 15	0.66	0.73	1.06	0.48
NPR1	NPR1;ANPRA;ANPa;GUC2A;GUCY2A;NPRA	natriuretic peptide receptor A/guanylate cyclase A (atrionatriuretic peptide receptor A)	0.66	0.69	1.06	0.62
KIAA1468	KIAA1468;FLJ33841;HsT3308;HsT885	KIAA1468	0.66	0.66	1.06	0.55
ZBTB24	ZBTB24;BIF1;ICF2;PATZ2;ZNF450	zinc finger and BTB domain containing 24	0.66	0.68	1.06	0.55
PSKH1	PSKH1	protein serine kinase H1	0.66	0.7	1.06	0.58
TMEM161A	TMEM161A;AROS-29;FLJ20422;FLJ39645	transmembrane protein 161A	0.66	0.75	1.06	0.58
SLC25A29	SLC25A29;C14orf69;CACL;FLJ38975	solute carrier family 25, member 29	0.66	0.67	1.06	0.48
CCDC109B	CCDC109B;FLJ20647	coiled-coil domain containing 109B	0.66	0.66	1.06	0.53
FOXP4	FOXP4;FLJ40908;FLJ44184;hFKHLA	forkhead box P4	0.66	0.7	1.06	0.52
POM121	POM121;DKFZp586G1822;DKFZp586P2220;FLJ41820;KIAA0618;MGC3792;POM121A	POM121 membrane glycoprotein	0.66	0.66	1.06	0.56
TMEM111	TMEM111;POB	transmembrane protein 111	0.66	0.68	1.06	0.52
LSM14A	LSM14A;C19orf13;DKFZp434D1335;FAM61A;RAP55;RAP55A	LSM14A, SCD6 homolog A (S. cerevisiae)	0.66	0.62	1.06	0.49
MYH3	MYH3;HEMHC;MYHC-EMB;MYHSE1;SMHCE	myosin, heavy chain 3, skeletal muscle, embryonic	0.65	0.76	1.06	0.52
WNT8B	WNT8B	wingless-type MMTV integration site family, member 8B	0.65	0.68	1.06	0.54
MXD3	MXD3;BHLHC13;FLJ35523;MAD3;MGC2383;MYX	MAX dimerization protein 3	0.65	0.7	1.06	0.51
TUBB1	TUBB1	tubulin, beta 1 class VI	0.64	0.68	1.06	0.54
MUCL1	MUCL1;SBEM	mucin-like 1	0.64	0.65	1.06	0.53
SERP2	SERP2;C13orf21;MGC35505;RP11-269C23.1;bA269C23.1	stress-associated endoplasmic reticulum protein family member 2	0.64	0.66	1.06	0.49
ZSCAN10	ZSCAN10;FLJ14549;ZNF206	zinc finger and SCAN domain containing 10	0.64	0.73	1.06	0.51
OLFM2	OLFM2;NOE2;NOELIN2;NOELIN2_V1;OlfC	olfactomedin 2	0.64	0.69	1.06	0.49
GPR172B	GPR172B;FLJ10060;GPCR42;PAR2;RFT1;hRFT1	G protein-coupled receptor 172B	0.64	0.66	1.06	0.57
PRAM1	PRAM1;MGC39864;PML-RAR;PRAM-1	PML-RARA regulated adaptor molecule 1	0.64	0.74	1.06	0.49
LRCH4	LRCH4;FLJ40101;FLJ46315;LRN;LRRN1;LRRN4;PP14183	leucine-rich repeats and calponin homology (CH) domain containing 4	0.64	0.66	1.06	0.5
WDR18	WDR18;MGC2436;R32184_1	WD repeat domain 18	0.64	0.66	1.06	0.52
CCDC94	CCDC94;FLJ10374	coiled-coil domain containing 94	0.64	0.6	1.06	0.47
EYA4	EYA4;CMD1J;DFNA10	eyes absent homolog 4 (Drosophila)	0.64	0.64	1.06	0.49
RSAD2	RSAD2;2510004L01Rik;cig33;cig5;vig1	radical S-adenosyl methionine domain containing 2	0.63	0.62	1.06	0.5
ZNF572	ZNF572;FLJ38002	zinc finger protein 572	0.63	0.7	1.06	0.56
DLL1	DLL1;DELTA1;DL1;Delta	delta-like 1 (Drosophila)	0.63	0.62	1.06	0.5
GPRIN3	GPRIN3;FLJ42625;GRIN3	GPRIN family member 3	0.62	0.62	1.06	0.51
HSD17B6	HSD17B6;HSE;RODH;SDR9C6	hydroxysteroid (17-beta) dehydrogenase 6 homolog (mouse)	0.62	0.67	1.06	0.45
NHSL2	NHSL2	NHS-like 2	0.62	0.72	1.06	0.5
CCL25	CCL25;Ckb15;MGC150327;SCYA25;TECK	chemokine (C-C motif) ligand 25	0.62	0.68	1.06	0.51
VSX2	VSX2;CHX10;HOX10;MCOP2;MCOPCB3;RET1	visual system homeobox 2	0.62	0.68	1.06	0.46
SIGLEC12	SIGLEC12;FLJ38600;S2V;SIGLECL1;SLG;Siglec-XII	sialic acid binding Ig-like lectin 12 (gene/pseudogene)	0.62	0.62	1.06	0.5
LRCH3	LRCH3;FLJ20994;FLJ43245;FLJ44976;MGC4126	leucine-rich repeats and calponin homology (CH) domain containing 3	0.62	0.68	1.06	0.52
YWHAG	YWHAG;14-3-3GAMMA	tyrosine 3-monooxygenase/tryptophan 5-monooxygenase activation protein, gamma polypeptide	0.62	0.7	1.06	0.52
C16orf72	C16orf72;FLJ41272;PRO0149	chromosome 16 open reading frame 72	0.62	0.66	1.06	0.48
DMRT3	DMRT3;DMRTA3;MGC142144	doublesex and mab-3 related transcription factor 3	0.61	0.62	1.06	0.48
GATS	GATS;DKFZp686B07267;STAG3OS	GATS, stromal antigen 3 opposite strand	0.61	0.62	1.06	0.49
CYP4A22	CYP4A22	cytochrome P450, family 4, subfamily A, polypeptide 22	0.84	0.86	1.07	0.62
ARHGAP9	ARHGAP9;10C;FLJ16525;MGC1295;RGL1	Rho GTPase activating protein 9	0.84	0.93	1.07	0.7
EXTL2	EXTL2;EXTR2	exostoses (multiple)-like 2	0.84	0.88	1.07	0.66
KCNJ9	KCNJ9;GIRK3;KIR3.3	potassium inwardly-rectifying channel, subfamily J, member 9	0.84	0.94	1.07	0.72
MAGEA9	MAGEA9;CT1.9;MAGE9;MAGEA9B;MGC8421	melanoma antigen family A, 9	0.84	0.93	1.07	0.7
FBXO4	FBXO4;DKFZp547N213;FBX4;FLJ10141	F-box protein 4	0.84	0.86	1.07	0.62
SEMA6B	SEMA6B;SEM-SEMA-Y;SEMA-VIB;SEMAN;semaZ	sema domain, transmembrane domain (TM), and cytoplasmic domain, (semaphorin) 6B	0.84	0.86	1.07	0.72
LHB	LHB;CGB4;LSH-B;hLHB	luteinizing hormone beta polypeptide	0.84	0.88	1.07	0.7
TICAM1	TICAM1;MGC35334;PRVTIRB;TICAM-1;TRIF	toll-like receptor adaptor molecule 1	0.84	0.91	1.07	0.68
DOK7	DOK7;C4orf25;CMS1B;FLJ33718;FLJ39137;FLJ90556	docking protein 7	0.84	0.95	1.07	0.73
FLYWCH1	FLYWCH1	FLYWCH-type zinc finger 1	0.84	0.96	1.07	0.67
COL6A2	COL6A2;DKFZp586E1322;FLJ46862;PP3610	collagen, type VI, alpha 2	0.84	0.94	1.07	0.76
EYA2	EYA2;EAB1;MGC10614	eyes absent homolog 2 (Drosophila)	0.84	0.83	1.07	0.63
BAG3	BAG3;BAG-3;BIS;CAIR-1;MGC104307	BCL2-associated athanogene 3	0.84	0.85	1.07	0.66
KDM2B	KDM2B;CXXC2;FBXL10;Fbl10;JHDM1B;PCCX2	lysine (K)-specific demethylase 2B	0.84	0.86	1.07	0.62
SGK223	SGK223;DKFZp761P0423;PRAGMIN	homolog of rat pragma of Rnd2	0.84	0.9	1.07	0.74
METTL21A	METTL21A;FAM119A;HCA557b;MGC45373	methyltransferase like 21A	0.84	0.94	1.07	0.67
CCR1	CCR1;CD191;CKR-1;CKR1;CMKBR1;HM145;MIP1aR;SCYAR1	chemokine (C-C motif) receptor 1	0.83	0.86	1.07	0.78

ZNF20	ZNF20;FLJ39241;KOX13	zinc finger protein 20	0.83	0.9	1.07	0.69
ZNF589	ZNF589;SZF1	zinc finger protein 589	0.83	0.85	1.07	0.62
FLG2	FLG2;IFPS	filaggrin family member 2	0.82	0.9	1.07	0.61
C9orf68	C9orf68;FLJ10058;bA6J24.2	chromosome 9 open reading frame 68	0.82	0.92	1.07	0.68
MAML3	MAML3;CAGH3;ERDA3;GDN;MAM-2;MAM2;TNRC3	mastermind-like 3 (Drosophila)	0.82	0.86	1.07	0.71
LSAMP	LSAMP;FLJ34254;FLJ35396;FLJ37216;FLJ54658;IGLON3;LAMP	limbic system-associated membrane protein	0.82	0.78	1.07	0.66
MYL4	MYL4;ALC1;AMLC;GT1	myosin, light chain 4, alkali; atrial, embryonic	0.82	0.92	1.07	0.68
RTN4RL1	RTN4RL1;DKFZp547J144;NGRH2;NgR3	reticulon 4 receptor-like 1	0.82	0.9	1.07	0.69
CRYGN	CRYGN;MGC119042;MGC119043;MGC119044;MGC119045	crystallin, gamma N	0.82	0.9	1.07	0.68
MAGEA3	MAGEA3;CT1.3;HIP8;HYPD;MAGE3;MAGEA6;MGC14613	melanoma antigen family A, 3	0.82	0.91	1.07	0.69
FBXL6	FBXL6;FBL6;FBL6A;PP14630	F-box and leucine-rich repeat protein 6	0.82	0.84	1.07	0.6
TMEM159	TMEM159;PROMETHIN	transmembrane protein 159	0.82	0.94	1.07	0.66
MPDZ	MPDZ;DKFZp781P216;FLJ25909;FLJ34626;FLJ90240;MUPP1	multiple PDZ domain protein	0.82	0.79	1.07	0.66
ZNF496	ZNF496;MGC15548;NIZP1;ZFP496;ZKSCAN17	zinc finger protein 496	0.82	0.85	1.07	0.71
VPS37A	VPS37A;FLJ32642;FLJ42616;HCRP1;PQBP2	vacuolar protein sorting 37 homolog A (S. cerevisiae)	0.82	0.82	1.07	0.67
BOK	BOK;BCL2L9;BOKL;MGC4631	BCL2-related ovarian killer	0.82	0.84	1.07	0.61
UTP3	UTP3;CRL1;CRLZ1;FLJ23256;SAS10	UTP3, small subunit (SSU) processome component, homolog (S. cerevisiae)	0.82	0.79	1.07	0.66
C5orf25	C5orf25;FLJ44216;FLJ56998;MGC33586;MGC40402;MGC90345	chromosome 5 open reading frame 25	0.82	0.87	1.07	0.63
DVL3	DVL3;KIAA0208	dishevelled, dsh homolog 3 (Drosophila)	0.82	0.88	1.07	0.6
SLC39A7	SLC39A7;D6S115E;D6S2244E;H2-KE4;HKE4;KE4;RING5;ZIP7	solute carrier family 39 (zinc transporter), member 7	0.82	0.84	1.07	0.61
PRKAG1	PRKAG1;AMPKG;MGC8666	protein kinase, AMP-activated, gamma 1 non-catalytic subunit	0.82	0.86	1.07	0.71
CAST	CAST;BS-17;MGC9402	calpastatin	0.82	0.78	1.07	0.63
NME2	NME2;MGC111212;MGC2212;NDKB;NDPK-B;NDPKB;NM23-H2;NM23B;PUF	non-metastatic cells 2, protein (NM23B) expressed in	0.82	0.94	1.07	0.67
SULF1	SULF1;FLJ30905;FLJ38022;FLJ41750;HSULF-1;KIAA1077;SULF-1	sulfatase 1	0.81	0.93	1.07	0.7
GALR2	GALR2;GALNR2;MGC125983;MGC125984	galanin receptor 2	0.81	0.84	1.07	0.75
NDUFAF2	NDUFAF2;B17.2L;FLJ22398;MMTN;NDUFA12L;mimitin	NADH dehydrogenase (ubiquinone) 1 alpha subcomplex, assembly factor 2	0.81	0.86	1.07	0.62
SMARCC2	SMARCC2;BAF170;CRACC2;DKFZp313D0632;Rsc8	SWI/SNF related, matrix associated, actin dependent regulator of chromatin, subfamily c, member 2	0.81	0.82	1.07	0.65
FANCI	FANCI;FLJ10719;KIAA1794	Fanconi anemia, complementation group I	0.81	0.96	1.07	0.7
C11orf55	C11orf55;FLJ45212	chromosome 11 open reading frame 55	0.81	0.9	1.07	0.65
C7orf53	C7orf53;FLJ39575;MGC161427;MGC163440;MGC42850	chromosome 7 open reading frame 53	0.8	0.88	1.07	0.67
OR1A2	OR1A2;MGC119930;MGC119931;OR17-6	olfactory receptor, family 1, subfamily A, member 2	0.8	0.83	1.07	0.63
C10orf93	C10orf93;RP13-137A17.3;bB137A17.3	chromosome 10 open reading frame 93	0.8	0.76	1.07	0.64
C1QB	C1QB	complement component 1, q subcomponent, B chain	0.8	0.88	1.07	0.66
PSD2	PSD2;DKFZp761B0514	pleckstrin and Sec7 domain containing 2	0.8	0.88	1.07	0.66
DZIP1L	DZIP1L;DZIP2;FLJ32844	DAZ interacting protein 1-like	0.8	0.91	1.07	0.64
C10orf11	C10orf11	chromosome 10 open reading frame 11	0.8	0.82	1.07	0.63
CREB3L3	CREB3L3;CREB-H;CREBH;MGC126553;MGC126557	cAMP responsive element binding protein 3-like 3	0.8	0.94	1.07	0.69
TXNDC16	TXNDC16;KIAA1344	thioredoxin domain containing 16	0.8	0.83	1.07	0.58
SPRY2	SPRY2;MGC23039;hSPRY2	sprouty homolog 2 (Drosophila)	0.8	0.84	1.07	0.63
PPP1R14C	PPP1R14C;CPI17-like;KEPI;NY-BR-81	protein phosphatase 1, regulatory (inhibitor) subunit 14C	0.8	0.85	1.07	0.71
PRDM12	PRDM12;PFM9	PR domain containing 12	0.8	0.91	1.07	0.6
TGFA	TGFA;TFGA	transforming growth factor, alpha	0.8	0.9	1.07	0.67
ZC3H4	ZC3H4;C19orf7;KIAA1064	zinc finger CCCH-type containing 4	0.8	0.87	1.07	0.59
TRIP4	TRIP4;ASC-1;ASC1;HsT17391	thyroid hormone receptor interactor 4	0.8	0.88	1.07	0.7
RNF34	RNF34;CARP-1;CARP1;FLJ21786;RFI;RIF;RIFF;hRFI	ring finger protein 34	0.8	0.84	1.07	0.75
AMOTL2	AMOTL2;LCCP	angiomotin like 2	0.8	0.84	1.07	0.63
TPT1	TPT1;FLJ27337;HRF;TCTP;p02	tumor protein, translationally-controlled 1	0.8	0.88	1.07	0.67
RAP1B	RAP1B;DKFZp586H0723;FLJ18601;K-REV;RAL1B	RAP1B, member of RAS oncogene family	0.8	0.81	1.07	0.64
CSNK1D	CSNK1D;HCKID	casein kinase 1, delta	0.8	0.92	1.07	0.65
PRDX6	PRDX6;1-Cys;AOP2;KIAA0106;MGC46173;NSGPx;PRX;aiPLA2;p29	peroxiredoxin 6	0.8	0.92	1.07	0.69
PRSS38	PRSS38;MGC163272;MPN2	protease, serine, 38	0.8	0.85	1.07	0.64
OR2B3	OR2B3;6M1-1;OR2B3P;OR6-14;OR6-4	olfactory receptor, family 2, subfamily B, member 3	0.8	0.78	1.07	0.62
DEFB111	DEFB111	ENTREZ STATUS: REMAPPED TO GENE ID: 245913	0.8	0.85	1.07	0.59
ZBTB49	ZBTB49;FLJ38559;MGC126279;MGC126280;ZNF509	zinc finger and BTB domain containing 49	0.8	0.83	1.07	0.63
OR4D1	OR4D1;OR17-23;OR4D3;OR4D4P;TPCR16	olfactory receptor, family 4, subfamily D, member 1	0.79	0.87	1.07	0.66
C3orf32	C3orf32;fls485	chromosome 3 open reading frame 32	0.79	0.88	1.07	0.63
SCUBE3	SCUBE3;CEGF3;DKFZp686B09105;DKFZp686B1223;DKFZp686D20108;FLJ34743	signal peptide, CUB domain, EGF-like 3	0.79	0.88	1.07	0.58
GTF2B	GTF2B;TF2B;TFIIB	general transcription factor IIB	0.79	0.82	1.07	0.64
THBS1	THBS1;THBS;THBS-1;TSP;TSP-1;TSP1	thrombospondin 1	0.79	0.82	1.07	0.63
CPNE4	CPNE4;COPN4;CPN4;MGC15604	copine IV	0.78	0.8	1.07	0.65
OVGP1	OVGP1;CHIT5;EGP;MUC9;OGP	oviductal glycoprotein 1, 120kDa	0.78	0.85	1.07	0.67
DNAJB5	DNAJB5;Hsc40	DnaJ (Hsp40) homolog, subfamily B, member 5	0.78	0.87	1.07	0.65
LMLN	LMLN;GP63;INV;IX14;MSP	leishmanolysin-like (metallopeptidase M8 family)	0.78	0.82	1.07	0.62
E2F1	E2F1;E2F-1;RBAP1;RBBP3;RBP3	E2F transcription factor 1	0.78	0.92	1.07	0.63
RNF138	RNF138;HSD-4;MGC8758;NARF;STRIN	ring finger protein 138	0.78	0.82	1.07	0.73
CLTC	CLTC;CHC;CHC17;CLH-17;CLTCL2;Hc;KIAA0034	clathrin, heavy chain (Hc)	0.78	0.88	1.07	0.6
TPRN	TPRN;C9orf75;DFNB79;FLJ90254;MGC131933;RP11-350O14.7	taperin	0.78	0.81	1.07	0.65
ZNF219	ZNF219;ZFP219	zinc finger protein 219	0.77	0.9	1.07	0.66
ZNF343	ZNF343;FLJ39592;MGC10715;MGC20504;dJ734P14.5	zinc finger protein 343	0.77	0.8	1.07	0.61
SERGEF	SERGEF;DELGEF;Gnefr	secretion regulating guanine nucleotide exchange factor	0.77	0.9	1.07	0.6
RPL21P44	RPL21P44;RPL21_17_477	ribosomal protein L21 pseudogene 44	0.77	0.77	1.07	0.66
SCML4	SCML4;FLJ36252;dJ47M23.1	sex comb on midleg-like 4 (Drosophila)	0.76	0.85	1.07	0.64
SLC6A7	SLC6A7;PROT	solute carrier family 6 (neurotransmitter transporter, L-proline), member 7	0.76	0.76	1.07	0.67
NMB	NMB;MGC17211;MGC2277;MGC3936	neuromedin B	0.76	0.8	1.07	0.56
ZNF643	ZNF643;FLJ34293;RP11-656D10.1	zinc finger protein 643	0.76	0.88	1.07	0.67
SLC2A13	SLC2A13;HMIT;MGC48624	solute carrier family 2 (facilitated glucose transporter), member 13	0.76	0.78	1.07	0.68
C6orf136	C6orf136;MGC15854	chromosome 6 open reading frame 136	0.76	0.83	1.07	0.63
AMACR	AMACR;AMACRD;CBAS4;RACE;RM	alpha-methylacyl-CoA racemase	0.76	0.78	1.07	0.56
SDCCAG3	SDCCAG3;NY-CO-3	serologically defined colon cancer antigen 3	0.76	0.84	1.07	0.6
FZD8	FZD8;FZ-8;hFZ8	frizzled family receptor 8	0.76	0.78	1.07	0.7
NCOA1	NCOA1;F-SRC-1;KAT13A;MGC129719;MGC129720;RIP160;SRC1;bHLHe42;bHLHe74	nuclear receptor coactivator 1	0.76	0.82	1.07	0.63
ARIH1	ARIH1;ARI;DKFZp686O13120;FLJ20329;FLJ93118;HARI;HHARI;UBCH7BP	ariadne homolog, ubiquitin-conjugating enzyme E2 binding protein, 1 (Drosophila)	0.76	0.74	1.07	0.56
UBE2J1	UBE2J1;HSPC153;HSPC205;HSU93243;MGC12555;NCUBE-1;NCUBE1;UBC6;Ubc6p	ubiquitin-conjugating enzyme E2, J1, U	0.76	0.75	1.07	0.56
CDK2	CDK2;p33(CDK2)	cyclin-dependent kinase 2	0.76	0.79	1.07	0.71
RAB7A	RAB7A;FLJ20819;PRO2706;RAB7	RAB7A, member RAS oncogene family	0.76	0.82	1.07	0.63
CCT3	CCT3;CCT-gamma;CCTG;PIG48;TCP-1-gamma;TRIC5	chaperonin containing TCP1, subunit 3 (gamma)	0.76	0.78	1.07	0.68
GIMAP1	GIMAP1;HIMAP1;IMAP1;IMAP38	GTPase, IMAP family member 1	0.75	0.8	1.07	0.56
NPFF	NPFF;FMRFAL	neuropeptide FF-amide peptide precursor	0.75	0.85	1.07	0.68
ABCD4	ABCD4;ABC41;EST352188;P70R;P79R;PMP69;PXMP1L	ATP-binding cassette, sub-family D (ALD), member 4	0.75	0.81	1.07	0.63
GTPBP10	GTPBP10;DKFZp686A10121;FLJ38242;MGC104191;ObgH2	GTP-binding protein 10 (putative)	0.75	0.88	1.07	0.58
C1orf162	C1orf162;MGC24133	chromosome 1 open reading frame 162	0.74	0.76	1.07	0.55
LAT2	LAT2;LAB;NTAL;WBSCR15;WBSCR5;WSCR5	linker for activation of T cells family, member 2	0.74	0.86	1.07	0.6
ZFP1	ZFP1;FLJ34243;ZNF475	zinc finger protein 1 homolog (mouse)	0.74	0.77	1.07	0.65
ANKS6	ANKS6;ANKRD14;DKFZp686D24121;DKFZp781I0117;MGC70366;SAMD6	ankyrin repeat and sterile alpha motif domain containing 6	0.74	0.82	1.07	0.59
KIAA2018	KIAA2018;DKFZp781O0144	KIAA2018	0.74	0.81	1.07	0.55
TBL3	TBL3;SAZD;UTP13	transducin (beta)-like 3	0.74	0.72	1.07	0.6
PPWD1	PPWD1;KIAA0073	peptidylprolyl isomerase domain and WD repeat containing 1	0.74	0.76	1.07	0.54
BCAT2	BCAT2;BCAM;BCATM;BCT2;PP18	branched chain amino-acid transaminase 2, mitochondrial	0.74	0.78	1.07	0.55
ACER1	ACER1;ALKCDase1;ASAH3;MGC138327;MGC138329	alkaline ceramidase 1	0.74	0.78	1.07	0.67
OR5H14	OR5H14	olfactory receptor, family 5, subfamily H, member 14	0.73	0.82	1.07	0.61
MPP4	MPP4;ALS2CR5;DLG6	membrane protein, palmitoylated 4 (MAGUK p55 subfamily member 4)	0.73	0.76	1.07	0.54
FHIT	FHIT;AP3Aase;FRA3B	fragile histidine triad gene	0.73	0.76	1.07	0.56
C1orf187	C1orf187;AGPA3119;FLJ34999;MGC117222;UNQ3119;neucrin	chromosome 1 open reading frame 187	0.73	0.8	1.07	0.59
TMEM52	TMEM52	transmembrane protein 52	0.73	0.76	1.07	0.58
CALY	CALY;DRD1IP;NSG3	calcyon neuron-specific vesicular protein	0.73	0.82	1.07	0.58
DSCAM	DSCAM;CHD2-42;CHD2-52	Down syndrome cell adhesion molecule	0.72	0.8	1.07	0.61
MMP1	MMP1;CLG;CLGN	matrix metallopeptidase 1 (interstitial collagenase)	0.72	0.7	1.07	0.53
GPHA2	GPHA2;A2;GPA2;MGC126572;ZSIG51	glycoprotein hormone alpha 2	0.72	0.8	1.07	0.53
SNAI1	SNAI1;SLUGH2;SNA;SNAH;SNAIL;SNAIL1;dJ710H13.1	snail homolog 1 (Drosophila)	0.72	0.73	1.07	0.61
KCNK4	KCNK4;K2p4.1;TRAAK;TRAAK1	potassium channel, subfamily K, member 4	0.72	0.77	1.07	0.6
LARP7	LARP7;DKFZp564K112;MGC104360;PIP7S	La ribonucleoprotein domain family, member 7	0.72	0.8	1.07	0.57
GIT1	GIT1	G protein-coupled receptor kinase interacting ArfGAP 1	0.72	0.77	1.07	0.52
SLC30A5	SLC30A5;FLJ12496;FLJ12756;MGC5499;ZNT5;ZNTL1;ZTL1;ZnT-5	solute carrier family 30 (zinc transporter), member 5	0.72	0.72	1.07	0.64
DLL3	DLL3;SCDO1	delta-like 3 (Drosophila)	0.71	0.8	1.07	0.65
CENPP	CENPP;CENP-P;FLJ33928	centromere protein P	0.71	0.74	1.07	0.55
PCNXL2	PCNXL2;FLJ11383;KIAA0435	pecanex-like 2 (Drosophila)	0.71	0.78	1.07	0.59
FAM120AOS	FAM120AOS;C9orf10OS;FLJ31534	family with sequence similarity 120A opposite strand	0.71	0.8	1.07	0.56
SKA1	SKA1;C18orf24;MGC10200	spindle and kinetochore associated complex subunit 1	0.71	0.78	1.07	0.52
COL6A3	COL6A3;DKFZp686D23123;DKFZp686K04147;DKFZp686N0262;FLJ34702;FLJ98399	collagen, type VI, alpha 3	0.7	0.76	1.07	0.52
SPATA21	SPATA21;MGC27074;spergen-2;spergen2	spermatogenesis associated 21	0.7	0.82	1.07	0.54
CCDC18	CCDC18;NY-SAR-41;dJ717I23.1	coiled-coil domain containing 18	0.7	0.82	1.07	0.55
SYNPO	SYNPO;KIAA1029	synaptopodin	0.7	0.73	1.07	0.55
PAIP1	PAIP1;MGC12360	poly(A) binding protein interacting protein 1	0.7	0.82	1.07	0.61
ZFAND1	ZFAND1;FLJ14007	zinc finger, AN1-type domain 1	0.7	0.8	1.07	0.56
GOLPH3L	GOLPH3L;FLJ10687;GPP34R	golgi phosphoprotein 3-like	0.7	0.78	1.07	0.52
AP2S1	AP2S1;AP17;AP17-DELTA;CLAPS2	adaptor-related protein complex 2, sigma 1 subunit	0.7	0.79	1.07	0.59
SEC63	SEC63;DNAJC23;ERdj2;PRO2507;SEC63L	SEC63 homolog (S. cerevisiae)	0.7	0.72	1.07	0.53
PGAP2	PGAP2;CWH43-N;FLJ26520;FRAG1;MGC799	post-GPI attachment to proteins 2	0.7	0.77	1.07	0.59
LILRA3	LILRA3;CD85E;HM31;HM43;ILT6;LIR-4;LIR4;e3	leukocyte immunoglobulin-like receptor, subfamily A (without TM domain), member 3	0.69	0.76	1.07	0.58

RFX7	RFX7;FLJ12994;FLJ21104;MGC131836;RFXDC2	regulatory factor X, 7	0.69	0.8	1.07	0.56
ELF5	ELF5;ESE2	E74-like factor 5 (ets domain transcription factor)	0.68	0.67	1.07	0.54
PLA2G4F	PLA2G4F;DKFZp666G192;DKFZp781B229;PLA2G4FZ	phospholipase A2, group IVF	0.68	0.77	1.07	0.6
SRGN	SRGN;FLJ12930;MGC9289;PPG;PRG;PRG1	serglycin	0.68	0.8	1.07	0.53
CCDC102A	CCDC102A;MGC10992;MGC13119	coiled-coil domain containing 102A	0.68	0.69	1.07	0.56
SPRED2	SPRED2;FLJ21897;FLJ31917;MGC163164;Spred-2	sprouty-related, EVH1 domain containing 2	0.68	0.72	1.07	0.5
TTC38	TTC38;FLJ20699	tetratricopeptide repeat domain 38	0.68	0.76	1.07	0.51
PHLDB1	PHLDB1;DKFZp686H039;DKFZp686O24210;FLJ00141;FLJ90266;KIAA0638;LL5A;MGC111531	pleckstrin homology-like domain, family B, member 1	0.68	0.76	1.07	0.54
ZFX	ZFX;ZNF926	zinc finger protein, X-linked	0.68	0.73	1.07	0.49
CD320	CD320;8D6;8D6A;TCBLR	CD320 molecule	0.68	0.72	1.07	0.52
CLIP4	CLIP4;FLJ21069;FLJ32705;RSNL2	CAP-GLY domain containing linker protein family, member 4	0.68	0.77	1.07	0.55
SRRM2	SRRM2;300-KD;CWF21;Cwc21;DKFZp667L0421;DKFZp686O15166;FLJ21926;FLJ22250;KIAA0324;MGC40295;SRL300;SRm300	serine/arginine repetitive matrix 2	0.68	0.68	1.07	0.53
SMG8	SMG8;C17orf71;FLJ10587	smg-8 homolog, nonsense mediated mRNA decay factor (C. elegans)	0.68	0.8	1.07	0.53
SLC22A16	SLC22A16;CT2;FLIPT2;OCT6;OKB1;dJ261K5.1	solute carrier family 22 (organic cation/carnitine transporter), member 16	0.67	0.7	1.07	0.53
LMO1	LMO1;MGC116692;RBTN1;RHOM1;TTG1	LIM domain only 1 (rhombotin 1)	0.67	0.7	1.07	0.54
GTF2IRD1	GTF2IRD1;BEN;CREAM1;GTF3;MUSTRD1;RBAP2;WBS;WBSCR11;WBSCR12;hMusTRD1alpha1	GTF2I repeat domain containing 1	0.67	0.71	1.07	0.53
WDR67	WDR67;Gm85;MGC104222;MGC126773;MGC138159;MGC21654	WD repeat domain 67	0.67	0.74	1.07	0.49
CBX6	CBX6	chromobox homolog 6	0.67	0.74	1.07	0.49
RAD51D	RAD51D;BROVCA4;R51H3;RAD51L3;TRAD	RAD51 homolog D (S. cerevisiae)	0.67	0.72	1.07	0.56
SYNC	SYNC;MGC149625;MGC149626;SYNC1;SYNCOILIN	syncoilin, intermediate filament protein	0.67	0.76	1.07	0.54
HYDIN	HYDIN;DKFZp434D0513;DKFZp434L0850;FLJ12871;FLJ14665;HYDIN1;HYDIN2;KIAA1864;PPP1R31	hydrocephalus inducing homolog (mouse)	0.66	0.71	1.07	0.51
FRMD3	FRMD3;4.1O;EPB41L4O;EPB41LO;MGC20553;P410	FERM domain containing 3	0.66	0.66	1.07	0.56
KRTAP19-5	KRTAP19-5;KAP19.5	keratin associated protein 19-5	0.66	0.7	1.07	0.49
TNFRSF9	TNFRSF9;4-1BB;CD137;CDw137;FLJ43501;ILA;MGC2172	tumor necrosis factor receptor superfamily, member 9	0.66	0.66	1.07	0.53
C19orf52	C19orf52	chromosome 19 open reading frame 52	0.66	0.77	1.07	0.51
PDCD4	PDCD4;H731;MGC33046;MGC33047	programmed cell death 4 (neoplastic transformation inhibitor)	0.66	0.72	1.07	0.55
NCOR1	NCOR1;KIAA1047;MGC104216;N-CoR;N-CoR1;TRAC1;hN-CoR	nuclear receptor corepressor 1	0.66	0.76	1.07	0.5
ZCRB1	ZCRB1;MADP-1;MADP1;MGC26805;RBM36;ZCCHC19	zinc finger CCHC-type and RNA binding motif 1	0.66	0.72	1.07	0.54
SHISA6	SHISA6;FLJ37030;FLJ37733;FLJ45455	shisa homolog 6 (Xenopus laevis)	0.66	0.68	1.07	0.54
SERPINA6	SERPINA6;CBG	serpin peptidase inhibitor, clade A (alpha-1 antiproteinase, antitrypsin), member 6	0.65	0.68	1.07	0.48
HOXD8	HOXD8;HOX4;HOX4E;HOX5.4	homeobox D8	0.64	0.67	1.07	0.47
CCDC151	CCDC151;FLJ31801;MGC20983	coiled-coil domain containing 151	0.64	0.66	1.07	0.52
HSN2	HSN2	ENTREZ STATUS: REMAPPED TO GENE ID: 65125	0.64	0.66	1.07	0.5
CLDN11	CLDN11;OSP;OTM	claudin 11	0.64	0.7	1.07	0.49
ORAI2	ORAI2;C7orf19;CBCIP2;FLJ12474;FLJ14733;FLJ44818;MEM142B;TMEM142B	ORAI calcium release-activated calcium modulator 2	0.64	0.7	1.07	0.53
RPUSD3	RPUSD3;FLJ34707;FLJ37268;MGC29784	RNA pseudouridylate synthase domain containing 3	0.64	0.74	1.07	0.52
RHEB	RHEB;MGC111559;RHEB2	Ras homolog enriched in brain	0.64	0.66	1.07	0.52
ASAP1	ASAP1;AMAP1;CENTB4;DDEF1;KIAA1249;PAG2;PAP;ZG14P	ArfGAP with SH3 domain, ankyrin repeat and PH domain 1	0.64	0.72	1.07	0.48
TMOD4	TMOD4;DKFZp779I0852;SK-TMOD	tropomodulin 4 (muscle)	0.63	0.71	1.07	0.5
CD300E	CD300E;CD300LE;CLM-2;CLM2;CMRF35-A5;IREM-2;IREM2;PlgR-2;PlgR2	CD300e molecule	0.63	0.7	1.07	0.52
C14orf169	C14orf169;FLJ21802;MAPJD;NO66	chromosome 14 open reading frame 169	0.63	0.7	1.07	0.57
NCOA7	NCOA7;ERAP140;ESNA1;FLJ45605;MGC88425;Nbla00052;Nbla10993;dJ187J11.3	nuclear receptor coactivator 7	0.63	0.71	1.07	0.57
CLTB	CLTB;LCB	clathrin, light chain B	0.63	0.69	1.07	0.54
RNF128	RNF128;FLJ23516;GRAIL	ring finger protein 128	0.62	0.66	1.07	0.59
MAD1L1	MAD1L1;MAD1;PIG9;TP53I9;TXBP181	MAD1 mitotic arrest deficient-like 1 (yeast)	0.62	0.68	1.07	0.46
ABCC12	ABCC12;MGC27071;MRP9	ATP-binding cassette, sub-family C (CFTR/MRP), member 12	0.61	0.64	1.07	0.47
RAB40A	RAB40A;MGC142061;RAR2;RAR2A	RAB40A, member RAS oncogene family	0.61	0.63	1.07	0.45
SCAP	SCAP;KIAA0199	SREBF chaperone	0.61	0.63	1.07	0.45
FLOT1	FLOT1	flotillin 1	0.61	0.67	1.07	0.46
GORASP2	GORASP2;DKFZp434D156;FLJ13139;GOLPH6;GRASP55;GRS2;p59	golgi reassembly stacking protein 2, 55kDa	0.61	0.68	1.07	0.45
TUSC3	TUSC3;D8S1992;DKFZp686B22120;M33;MGC13453;MRT7;N33;OST3A	tumor suppressor candidate 3	0.84	0.86	1.08	0.63
SLC5A4	SLC5A4;DJ90G24.4;SAAT1;SGLT3	solute carrier family 5 (low affinity glucose cotransporter), member 4	0.84	0.88	1.08	0.69
ITGBL1	ITGBL1;OSCP;TIED	integrin, beta-like 1 (with EGF-like repeat domains)	0.84	0.94	1.08	0.63
NAT8L	NAT8L;CML3;DKFZp434B0650;DKFZp566H184;FLJ37478;MGC117272;NACED;NAT8-LIKE	N-acetyltransferase 8-like (GCN5-related, putative)	0.84	0.91	1.08	0.71
SLC6A3	SLC6A3;DAT;DAT1	solute carrier family 6 (neurotransmitter transporter, dopamine), member 3	0.84	0.98	1.08	0.73
ENOX2	ENOX2;APK1;COVA1;tNOX	ecto-NOX disulfide-thiol exchanger 2	0.84	0.94	1.08	0.7
SLC7A6	SLC7A6;DKFZp686K15246;KIAA0245;LAT-2;LAT3;y+LAT-2	solute carrier family 7 (amino acid transporter light chain, y+L system), member 6	0.84	0.84	1.08	0.75
TUFM	TUFM;COXPD4;EF-TuMT;EFTU;P43	Tu translation elongation factor, mitochondrial	0.84	0.88	1.08	0.63
HAUS6	HAUS6;Dgt6;FAM29A;MGC102696;MGC138798;MGC138799	HAUS augmin-like complex, subunit 6	0.84	0.95	1.08	0.63
ABCG4	ABCG4;WHITE2	ATP-binding cassette, sub-family G (WHITE), member 4	0.83	0.92	1.08	0.72
DNER	DNER;UNQ26;bet	delta/notch-like EGF repeat containing	0.83	0.92	1.08	0.7
ENO2	ENO2;NSE	enolase 2 (gamma, neuronal)	0.83	0.89	1.08	0.66
IARS2	IARS2;FLJ10326;ILERS	isoleucyl-tRNA synthetase 2, mitochondrial	0.83	0.97	1.08	0.74
TGM3	TGM3;MGC126249;MGC126250;TGE	transglutaminase 3 (E polypeptide, protein-glutamine-gamma-glutamyltransferase)	0.82	0.89	1.08	0.69
KIAA1324	KIAA1324;EIG121;MGC150624	KIAA1324	0.82	0.86	1.08	0.65
COL21A1	COL21A1;COLA1L;DKFZp564B052;FLJ39125;FLJ44623;MGC26619;dJ682J15.1;dJ708F5.1	collagen, type XXI, alpha 1	0.82	0.92	1.08	0.69
KCNH6	KCNH6;ERG2;HERG2;Kv11.2	potassium voltage-gated channel, subfamily H (eag-related), member 6	0.82	0.94	1.08	0.73
ABCA5	ABCA5;ABC13;DKFZp451F117;DKFZp779N2435;EST90625;FLJ16381	ATP-binding cassette, sub-family A (ABC1), member 5	0.82	0.88	1.08	0.6
FILIP1L	FILIP1L;DOC-1;DOC1;GIP130;GIP130a;GIP130b;GIP130c;GIP90	filamin A interacting protein 1-like	0.82	0.84	1.08	0.68
SERF2	SERF2;4F5REL;FAM2C;FLJ20431;FLJ37527;FLJ38557;H4F5REL;HsT17089;MGC48826	small EDRK-rich factor 2	0.82	0.9	1.08	0.63
MICALL2	MICALL2;FLJ23471;FLJ41996;FLJ44858;FLJ45410;JRAB;MGC46023;MICAL-L2	MICAL-like 2	0.82	0.84	1.08	0.68
C17orf65	C17orf65;DKFZp762C2414;MGC119234	chromosome 17 open reading frame 65	0.82	0.94	1.08	0.73
SEC22A	SEC22A;SEC22L2	SEC22 vesicle trafficking protein homolog A (S. cerevisiae)	0.82	0.92	1.08	0.66
C21orf56	C21orf56;DKFZp434N0650;MGC99490	chromosome 21 open reading frame 56	0.82	0.84	1.08	0.71
ZNF302	ZNF302;HSD16;MST154;MSTP154;ZNF135L;ZNF140L;ZNF327	zinc finger protein 302	0.82	0.88	1.08	0.73
ZNF131	ZNF131;pHZ-10	zinc finger protein 131	0.82	0.84	1.08	0.61
EMP2	EMP2;MGC9056;XMP	epithelial membrane protein 2	0.82	0.93	1.08	0.66
SLX1B	SLX1B;FLJ23439;GIYD1;GIYD2;MGC2532;MGC5178;SLX1A	SLX1 structure-specific endonuclease subunit homolog B (S. cerevisiae)	0.82	0.92	1.08	0.66
ACCSL	ACCSL	1-aminocyclopropane-1-carboxylate synthase homolog (Arabidopsis)(non-functional)-like	0.82	0.92	1.08	0.63
ZNF213	ZNF213;CR53;ZKSCAN21	zinc finger protein 213	0.81	0.88	1.08	0.61
ZADH2	ZADH2;MGC45594	zinc binding alcohol dehydrogenase domain containing 2	0.81	0.88	1.08	0.69
MIDN	MIDN;DKFZp547M072	midnolin	0.81	0.84	1.08	0.64
ZNF558	ZNF558;FLJ30932	zinc finger protein 558	0.81	0.9	1.08	0.68
C6orf35	C6orf35;BM033;FLJ10029;MGC34308	chromosome 6 open reading frame 35	0.81	0.86	1.08	0.64
CCDC108	CCDC108;DKFZp434O0527;MGC35338	coiled-coil domain containing 108	0.8	0.89	1.08	0.66
MYO15A	MYO15A;DFNB3;DKFZp686N18198;FLJ17274;FLJ31311;MYO15	myosin XVA	0.8	0.84	1.08	0.59
C4orf19	C4orf19;FLJ11017	chromosome 4 open reading frame 19	0.8	0.94	1.08	0.63
TMEM169	TMEM169;DKFZp781L2456;FLJ34263	transmembrane protein 169	0.8	0.82	1.08	0.63
NUDT17	NUDT17;FLJ34433	nudix (nucleoside diphosphate linked moiety X)-type motif 17	0.8	0.78	1.08	0.62
MMP11	MMP11;SL-3;ST3;STMY3	matrix metallopeptidase 11 (stromelysin 3)	0.8	0.8	1.08	0.6
CYP2J2	CYP2J2;CPJ2	cytochrome P450, family 2, subfamily J, polypeptide 2	0.8	0.85	1.08	0.66
C10orf125	C10orf125;FLJ26016;FUCU;MGC149258	chromosome 10 open reading frame 125	0.8	0.84	1.08	0.63
PARP8	PARP8;FLJ21308;MGC42864;pART16	poly (ADP-ribose) polymerase family, member 8	0.8	0.83	1.08	0.64
COX11	COX11;COX11P	COX11 cytochrome c oxidase assembly homolog (yeast)	0.8	0.86	1.08	0.58
HMGCL	HMGCL;HL	3-hydroxymethyl-3-methylglutaryl-CoA lyase	0.8	0.82	1.08	0.64
ATMIN	ATMIN;ASCIZ;DKFZp779K1455;FLJ50270;FLJ61620;FLJ76795;KIAA0431;ZNF822	ATM interactor	0.8	0.86	1.08	0.62
POLR3A	POLR3A;ADDH;HLD7;RPC1;RPC155;hRPC155	polymerase (RNA) III (DNA directed) polypeptide A, 155kDa	0.8	0.85	1.08	0.66
EIF2B3	EIF2B3;EIF-2B;EIF2Bgamma	eukaryotic translation initiation factor 2B, subunit 3 gamma, 58kDa	0.8	0.92	1.08	0.61
F11R	F11R;CD321;JAM;JAM1;JAMA;JCAM;KAT;PAM-1	F11 receptor	0.8	0.91	1.08	0.6
TMED2	TMED2;FLJ21323;P24A;RNP24	transmembrane emp24 domain trafficking protein 2	0.8	0.82	1.08	0.63
C6orf174	C6orf174;dJ403A15.3	chromosome 6 open reading frame 174	0.8	0.85	1.08	0.7
LIN28A	LIN28A;CSDD1;FLJ12457;LIN-28;LIN28;ZCCHC1;lin-28A	lin-28 homolog A (C. elegans)	0.8	0.78	1.08	0.65
TXLNG2P	TXLNG2P;CYorf15A;CYorf15B;DKFZp451G0616;FLJ33216;MGC131732;MGC21662	taxilin gamma 2, pseudogene	0.8	0.9	1.08	0.62
SDHAF2	SDHAF2;C11orf79;FLJ20487;PGL2;SDH5	succinate dehydrogenase complex assembly factor 2	0.8	0.93	1.08	0.62
FOXD4L6	FOXD4L6;MGC119257	forkhead box D4-like 6	0.79	0.88	1.08	0.65
NCKIPSD	NCKIPSD;AF3P21;DIP;DIP1;MGC23891;ORF1;SPIN90;WASLBP;WISH	NCK interacting protein with SH3 domain	0.79	0.86	1.08	0.66
PLD6	PLD6	phospholipase D family, member 6	0.79	0.88	1.08	0.59
HLA-F	HLA-F;CDA12;HLA-5.4;HLA-CDA12;HLAF	major histocompatibility complex, class I, F	0.79	0.9	1.08	0.6
RSPH10B	RSPH10B;MGC50833;RSPH10B2	radial spoke head 10 homolog B (Chlamydomonas)	0.78	0.76	1.08	0.64
STMN4	STMN4;MGC111012;RB3	stathmin-like 4	0.78	0.86	1.08	0.65
WIPF3	WIPF3;CR16;FLJ36931	WAS/WASL interacting protein family, member 3	0.78	0.82	1.08	0.62
PRM3	PRM3	protamine 3	0.78	0.84	1.08	0.59
NLGN2	NLGN2;KIAA1366	neuroligin 2	0.78	0.81	1.08	0.6
HSCB	HSCB;DNAJC20;HSC20;JAC1;MGC2637;MGC74462;dJ366L4.2	HscB iron-sulfur cluster co-chaperone homolog (E. coli)	0.78	0.9	1.08	0.63
NOTCH3	NOTCH3;CADASIL;CASIL	notch 3	0.78	0.84	1.08	0.66
KCTD14	KCTD14;MGC2376	potassium channel tetramerisation domain containing 14	0.78	0.89	1.08	0.69
BMP6	BMP6;VGR;VGR1	bone morphogenetic protein 6	0.78	0.86	1.08	0.57
ALDH2	ALDH2;ALDH-E2;ALDHI;ALDM;MGC1806	aldehyde dehydrogenase 2 family (mitochondrial)	0.78	0.8	1.08	0.63
LDB1	LDB1;CLIM2;NLI	LIM domain binding 1	0.78	0.92	1.08	0.64
ABHD3	ABHD3;LABH3;MGC11259	abhydrolase domain containing 3	0.78	0.78	1.08	0.69
C19orf2	C19orf2;FLJ10575;NNX3;RMP;URI	chromosome 19 open reading frame 2	0.78	0.82	1.08	0.62
FOLR4	FOLR4;Folbp3	folate receptor 4 (delta) homolog (mouse)	0.78	0.92	1.08	0.68
UBTD1	UBTD1;FLJ11807	ubiquitin domain containing 1	0.77	0.88	1.08	0.58
NDUFB10	NDUFB10;PDSW	NADH dehydrogenase (ubiquinone) 1 beta subcomplex, 10, 22kDa	0.77	0.77	1.08	0.61
NKIRAS2	NKIRAS2;DKFZp434N1526;KBRAS2;MGC74742;kappaB-Ras2	NFKB inhibitor interacting Ras-like 2	0.77	0.92	1.08	0.63
P4HA1	P4HA1;P4HA	prolyl 4-hydroxylase, alpha polypeptide I	0.77	0.82	1.08	0.57
MS4A10	MS4A10;CD20L7;FLJ16054;MS4A9	membrane-spanning 4-domains, subfamily A, member 10	0.76	0.84	1.08	0.57
TRIM4	TRIM4;RNF87	tripartite motif containing 4	0.76	0.81	1.08	0.72
ATM	ATM;AT1;ATA;ATC;ATD;ATDC;ATE;DKFZp781A0353;MGC74674;TEL1;TELO1	ataxia telangiectasia mutated	0.76	0.82	1.08	0.68

TBCCD1	TBCCD1;FLJ10560	TBCC domain containing 1	0.76	0.84	1.08	0.63
HERC3	HERC3;KIAA0032	hect domain and RLD 3	0.76	0.76	1.08	0.57
HOMER2	HOMER2;ACPD;CPD;HOMER-2;VESL-2	homer homolog 2 (Drosophila)	0.76	0.84	1.08	0.64
STX18	STX18;DKFZp686O15149;Ufe1	syntaxin 18	0.76	0.84	1.08	0.66
SPG20	SPG20;KIAA0610;SPARTIN;TAHCCP1	spastic paraplegia 20 (Troyer syndrome)	0.76	0.86	1.08	0.61
ETS2	ETS2;ETS2IT1	v-ets erythroblastosis virus E26 oncogene homolog 2 (avian)	0.76	0.76	1.08	0.6
EIF2B2	EIF2B2;EIF-2Bbeta;EIF2B	eukaryotic translation initiation factor 2B, subunit 2 beta, 39kDa	0.76	0.8	1.08	0.7
WDR45L	WDR45L;WIPI-3;WIPI3	WDR45-like	0.76	0.89	1.08	0.62
GMPS	GMPS	guanine monphosphate synthetase	0.76	0.82	1.08	0.64
PATE2	PATE2;C11orf38;PATE-M;UNQ3112	prostate and testis expressed 2	0.76	0.82	1.08	0.59
FRG2	FRG2;FRG2A	FSHD region gene 2	0.75	0.8	1.08	0.6
NKX3-1	NKX3-1;BAPX2;NKX3;NKX3.1;NKX3A	NK3 homeobox 1	0.75	0.82	1.08	0.64
HIST1H2BB	HIST1H2BB;H2B.1;H2B/f;H2BFF;MGC119804	histone cluster 1, H2bb	0.74	0.85	1.08	0.69
SLC30A8	SLC30A8;ZNT8;ZnT-8	solute carrier family 30 (zinc transporter), member 8	0.74	0.78	1.08	0.68
ZMYND12	ZMYND12;DKFZp434N2435	zinc finger, MYND-type containing 12	0.74	0.72	1.08	0.6
PLEKHB1	PLEKHB1;KPL1;PHR1;PHRET1	pleckstrin homology domain containing, family B (evectins) member 1	0.74	0.78	1.08	0.59
C20orf7	C20orf7;FLJ22324;MGC90272;bA526K24.2;dJ842G6.1	chromosome 20 open reading frame 7	0.74	0.78	1.08	0.65
CGB2	CGB2	chorionic gonadotropin, beta polypeptide 2	0.74	0.82	1.08	0.62
ALG2	ALG2;CDGli;FLJ14511;NET38;hALPG2	asparagine-linked glycosylation 2, alpha-1,3-mannosyltransferase homolog (S. cerevisiae)	0.74	0.86	1.08	0.64
MED1	MED1;CRSP1;CRSP200;DRIP205;DRIP230;MGC71488;PBP;PPARBP;PPARGBP;RB18A;TRAP220;TRIP2	mediator complex subunit 1	0.74	0.74	1.08	0.59
PRPSAP1	PRPSAP1;PAP39	phosphoribosyl pyrophosphate synthetase-associated protein 1	0.74	0.71	1.08	0.59
MPZL1	MPZL1;FLJ21047;MPZL1b;PZR;PZR1b;PZRa;PZRb	myelin protein zero-like 1	0.74	0.84	1.08	0.56
NEDD9	NEDD9;CAS-L;CAS2;CASL;CASS2;HEF1;dJ49G10.2;dJ761I2.1	neural precursor cell expressed, developmentally down-regulated 9	0.74	0.87	1.08	0.64
TRIM16	TRIM16;DKFZp434O1826;EBBP	tripartite motif containing 16	0.74	0.8	1.08	0.66
RYR2	RYR2;ARVC2;ARVD2;RYR-2;RyR;VTSIP	ryanodine receptor 2 (cardiac)	0.73	0.8	1.08	0.62
DAPL1	DAPL1	death associated protein-like 1	0.73	0.76	1.08	0.55
TRPV5	TRPV5;CAT2;ECAC1;OTRPC3	transient receptor potential cation channel, subfamily V, member 5	0.73	0.82	1.08	0.64
PNPLA5	PNPLA5;GS2L;dJ388M5;dJ388M5.4	patatin-like phospholipase domain containing 5	0.73	0.86	1.08	0.57
PAX1	PAX1;HUP48	paired box 1	0.73	0.75	1.08	0.59
ZFP90	ZFP90;NK10;ZNF756	zinc finger protein 90 homolog (mouse)	0.73	0.78	1.08	0.65
NMNAT1	NMNAT1;NMNAT;PNAT1	nicotinamide nucleotide adenylyltransferase 1	0.73	0.77	1.08	0.67
KRTAP10-10	KRTAP10-10;KAP10.10;KAP18.10;KRTAP18-10;KRTAP18.10	keratin associated protein 10-10	0.73	0.7	1.08	0.59
DUSP8	DUSP8;C11orf81;FLJ42476;FLJ42958;HB5;HVH-5;HVH8	dual specificity phosphatase 8	0.73	0.78	1.08	0.61
TNFRSF10A	TNFRSF10A;APO2;CD261;DR4;MGC9365;TRAILR-1;TRAILR1	tumor necrosis factor receptor superfamily, member 10a	0.73	0.84	1.08	0.63
MORC2	MORC2;ZCW3;ZCWCC1	MORC family CW-type zinc finger 2	0.73	0.84	1.08	0.59
STAT6	STAT6;D12S1644;IL-4-STAT;STAT6B;STAT6C	signal transducer and activator of transcription 6, interleukin-4 induced	0.73	0.78	1.08	0.58
FBXO5	FBXO5;EMI1;FBX5;Fbxo31	F-box protein 5	0.73	0.76	1.08	0.54
FAM50A	FAM50A;9F;DXS9928E;HXC-26;XAP5	family with sequence similarity 50, member A	0.73	0.74	1.08	0.6
PGRMC1	PGRMC1;HPR6.6;MPR	progesterone receptor membrane component 1	0.73	0.73	1.08	0.58
C2CD4B	C2CD4B;FAM148B;NLF2	C2 calcium-dependent domain containing 4B	0.73	0.81	1.08	0.6
PKDCC	PKDCC;FLJ18197;MGC125960;SGK493;Vlk	protein kinase domain containing, cytoplasmic homolog (mouse)	0.73	0.78	1.08	0.65
NPBWR2	NPBWR2;GPR8	neuropeptides B/W receptor 2	0.72	0.75	1.08	0.68
SATB2	SATB2;FLJ21474;FLJ32076;KIAA1034;MGC119474;MGC119477	SATB homeobox 2	0.72	0.81	1.08	0.61
MAT1A	MAT1A;MAT;MATA1;SAMS;SAMS1	methionine adenosyltransferase I, alpha	0.72	0.76	1.08	0.59
NAF1	NAF1	nuclear assembly factor 1 homolog (S. cerevisiae)	0.72	0.78	1.08	0.53
MYO9A	MYO9A;FLJ11061;FLJ13244;MGC71859	myosin IXA	0.72	0.8	1.08	0.53
TSC22D3	TSC22D3;DIP;DKFZp313A1123;DSIPI;GILZ;TSC-22R;hDIP	TSC22 domain family, member 3	0.72	0.78	1.08	0.56
C12orf32	C12orf32;MGC13204;RHINO	chromosome 12 open reading frame 32	0.72	0.76	1.08	0.57
REEP3	REEP3;C10orf74;MGC87479	receptor accessory protein 3	0.72	0.82	1.08	0.57
TMEM200C	TMEM200C;TTMA	transmembrane protein 200C	0.72	0.76	1.08	0.57
SEMA4D	SEMA4D;C9orf164;CD100;FLJ33485;FLJ34282;FLJ39737;FLJ46484;M-sema-G;MGC169138;MGC169141;SEMAJ;coll-4	sema domain, immunoglobulin domain (Ig), transmembrane domain (TM) and short cytoplasmic domain, (semaphorin) 4D	0.71	0.72	1.08	0.61
SLC35E3	SLC35E3;BLOV1	solute carrier family 35, member E3	0.71	0.8	1.08	0.55
USP32	USP32;NY-REN-60;USP10	ubiquitin specific peptidase 32	0.71	0.76	1.08	0.58
SLC25A48	SLC25A48;HDMCP	solute carrier family 25, member 48	0.71	0.74	1.08	0.6
IL2RA	IL2RA;CD25;IDDM10;IL2R;TCGFR	interleukin 2 receptor, alpha	0.7	0.76	1.08	0.59
SLC22A7	SLC22A7;MGC24091;MGC45202;NLT;OAT2	solute carrier family 22 (organic anion transporter), member 7	0.7	0.8	1.08	0.53
SCN5A	SCN5A;CDCD2;CMD1E;CMPD2;HB1;HB2;HBBD;HH1;ICCD;IVF;LQT3;Nav1.5;PFHB1;SSS1;VF1	sodium channel, voltage-gated, type V, alpha subunit	0.7	0.78	1.08	0.61
SAA2	SAA2	serum amyloid A2	0.7	0.76	1.08	0.58
MED19	MED19;DT2P1G7;LCMR1	mediator complex subunit 19	0.7	0.74	1.08	0.55
PRDM6	PRDM6	PR domain containing 6	0.7	0.76	1.08	0.59
FAM123B	FAM123B;AMER1;FLJ39827;OSCS;WTX	family with sequence similarity 123B	0.7	0.77	1.08	0.58
DPM2	DPM2;FLJ80013;MGC111193;MGC21559	dolichyl-phosphate mannosyltransferase polypeptide 2, regulatory subunit	0.7	0.74	1.08	0.59
DECR2	DECR2;PDCR;SDR17C1	2,4-dienoyl CoA reductase 2, peroxisomal	0.7	0.73	1.08	0.53
TMEM45B	TMEM45B;FLJ40787	transmembrane protein 45B	0.7	0.72	1.08	0.55
SASH1	SASH1;KIAA0790;RP3-323M4.1;SH3D6A;dJ323M4;dJ323M4.1	SAM and SH3 domain containing 1	0.7	0.74	1.08	0.56
SPAG9	SPAG9;CT89;FLJ13450;FLJ14006;FLJ26141;FLJ34602;HLC-6;HLC4;JIP-4;JIP4;JLP;KIAA0516;MGC117291;MGC14967;MGC74461;PHET;PIG6	sperm associated antigen 9	0.7	0.74	1.08	0.56
KTN1	KTN1;CG1;KIAA0004;KNT;MGC133337;MU-RMS-40.19	kinectin 1 (kinesin receptor)	0.7	0.72	1.08	0.61
DEFB103B	DEFB103B;BD-3;DEFB103;DEFB103A;DEFB3;HBD3;HBP3	defensin, beta 103B	0.7	0.78	1.08	0.59
SYT3	SYT3;DKFZp761O132;SytIII	synaptotagmin III	0.69	0.72	1.08	0.52
LRRC57	LRRC57;DKFZp686H1865;FLJ36812	leucine rich repeat containing 57	0.69	0.81	1.08	0.62
CHKA	CHKA;CHK;CK;CKI;EK	choline kinase alpha	0.69	0.8	1.08	0.57
OR10Q1	OR10Q1;OR11-233	olfactory receptor, family 10, subfamily Q, member 1	0.68	0.76	1.08	0.57
NEUROG1	NEUROG1;AKA;Math4C;NEUROD3;bHLHa6;ngn1	neurogenin 1	0.68	0.71	1.08	0.54
TTC31	TTC31;FLJ12788;FLJ33201;MGC120200	tetratricopeptide repeat domain 31	0.68	0.66	1.08	0.52
MAP2K7	MAP2K7;Jnkk2;MAPKK7;MKK7;PRKMK7	mitogen-activated protein kinase kinase 7	0.68	0.72	1.08	0.6
COMMD6	COMMD6;Acrg	COMM domain containing 6	0.68	0.7	1.08	0.55
FBXO9	FBXO9;DKFZp434C0118;FBX9;KIAA0936;NY-REN-57;VCIA1;dJ341E18.2	F-box protein 9	0.68	0.7	1.08	0.51
TK1	TK1;TK2	thymidine kinase 1, soluble	0.68	0.72	1.08	0.6
HDHD1	HDHD1;DXF68S1E;FAM16AX;GS1;HDHD1A	haloacid dehalogenase-like hydrolase domain containing 1	0.68	0.73	1.08	0.56
AQP3	AQP3;AQP-3;GIL	aquaporin 3 (Gill blood group)	0.67	0.78	1.08	0.59
IL13RA1	IL13RA1;CD213A1;IL-13Ra;NR4	interleukin 13 receptor, alpha 1	0.67	0.74	1.08	0.59
CH25H	CH25H;C25H	cholesterol 25-hydroxylase	0.66	0.68	1.08	0.49
OVOS2	OVOS2;DKFZp686C0338	ovostatin 2	0.66	0.74	1.08	0.54
DARC	DARC;CCBP1;CD234;Dfy;FY;GPD;GpFy;WBCQ1	Duffy blood group, chemokine receptor	0.66	0.66	1.08	0.5
QRICH2	QRICH2;DKFZp434P0316	glutamine rich 2	0.66	0.74	1.08	0.55
CYB5R4	CYB5R4;NCB5OR;RP4-676J13.1;cb5/cb5R;dJ676J13.1	cytochrome b5 reductase 4	0.66	0.69	1.08	0.6
TRMT11	TRMT11;C6orf75;MDS024;TRM11;TRMT11-1	tRNA methyltransferase 11 homolog (S. cerevisiae)	0.66	0.74	1.08	0.53
CC2D1A	CC2D1A;FLJ20241;FLJ41160;FREUD-1;Freud-1/Aki1;MRT3	coiled-coil and C2 domain containing 1A	0.66	0.74	1.08	0.55
ELF3	ELF3;EPR-1;ERT;ESE-1;ESX	E74-like factor 3 (ets domain transcription factor, epithelial-specific)	0.66	0.77	1.08	0.57
RPP21	RPP21;C6orf135;CAT60;FLJ22638	ribonuclease P/MRP 21kDa subunit	0.66	0.74	1.08	0.56
WDR5	WDR5;BIG-3;SWD3	WD repeat domain 5	0.66	0.73	1.08	0.55
ANKRD27	ANKRD27;DKFZp434L0718;FLJ00040;VARP	ankyrin repeat domain 27 (VPS9 domain)	0.66	0.74	1.08	0.5
LRRC47	LRRC47;KIAA1185	leucine rich repeat containing 47	0.65	0.74	1.08	0.52
ZC3HAV1	ZC3HAV1;DKFZp686F2052;DKFZp686H1869;DKFZp686O19171;FLB6421;FLJ13288;MGC48898;PARP13;ZAP;ZC3H2;ZC3HDC2	zinc finger CCCH-type, antiviral 1	0.65	0.71	1.08	0.52
PPA1	PPA1;IOPPP;MGC111556;PP;PP1;SID6-8061	pyrophosphatase (inorganic) 1	0.65	0.69	1.08	0.59
ODF3L1	ODF3L1;MGC48986	outer dense fiber of sperm tails 3-like 1	0.64	0.72	1.08	0.52
RNF151	RNF151;MGC129921	ring finger protein 151	0.64	0.69	1.08	0.62
NDRG4	NDRG4;BDM1;DKFZp686I1615;FLJ30586;FLJ42011;KIAA1180;MGC19632;SMAP-8	NDRG family member 4	0.64	0.66	1.08	0.54
BACH1	BACH1;BACH-1	BTB and CNC homology 1, basic leucine zipper transcription factor 1	0.64	0.74	1.08	0.55
C10orf57	C10orf57;FLJ13263;bA369J21.6	chromosome 10 open reading frame 57	0.64	0.66	1.08	0.55
PPIG	PPIG;CARS-Cyp;CYP;MGC133241;SCAF10;SRCyp	peptidylprolyl isomerase G (cyclophilin G)	0.64	0.66	1.08	0.51
STAU2	STAU2;39K2;39K3;DKFZp781K0371;MGC119606	staufen, RNA binding protein, homolog 2 (Drosophila)	0.64	0.74	1.08	0.6
SLC4A2	SLC4A2;AE2;BND3L;EPB3L1;FLJ59028;HKB3;NBND3	solute carrier family 4, anion exchanger, member 2 (erythrocyte membrane protein band 3-like 1)	0.64	0.74	1.08	0.56
APOL1	APOL1;APO-L;APOL;APOL-I;FSGS4	apolipoprotein L, 1	0.63	0.66	1.08	0.51
TNP2	TNP2;MGC116783;MGC116785;TP2	transition protein 2 (during histone to protamine replacement)	0.62	0.69	1.08	0.46
C3orf25	C3orf25	chromosome 3 open reading frame 25	0.62	0.7	1.08	0.52
UPK1A	UPK1A;MGC129751;MGC129752;MGC14388;TSPAN21;UP1A;UPIA;UPKA	uroplakin 1A	0.62	0.69	1.08	0.5
ZNF197	ZNF197;D3S1363E;P18;VHLaK;ZKSCAN9;ZNF166;ZNF20	zinc finger protein 197	0.62	0.68	1.08	0.46
MYT1L	MYT1L;NZF1	myelin transcription factor 1-like	0.62	0.73	1.08	0.55
SPTBN4	SPTBN4;KIAA1642;QV;SPNB4;SPTBN3	spectrin, beta, non-erythrocytic 4	0.62	0.7	1.08	0.57
TNNI2	TNNI2;AMCD2B;DA2B;FSSV;fsTnI	troponin I type 2 (skeletal, fast)	0.62	0.66	1.08	0.49
PPAP2A	PPAP2A;LLP1a;LPP1;PAP-2a;PAP2;PAP2a2;PAP2alpha2;PAPalpha1	phosphatidic acid phosphatase type 2A	0.62	0.68	1.08	0.52
MAPK8IP1	MAPK8IP1;IB1;JIP-1;JIP1;PRKM8IP	mitogen-activated protein kinase 8 interacting protein 1	0.62	0.7	1.08	0.53
HSD17B7	HSD17B7;MGC12523;MGC75018;PRAP;SDR37C1	hydroxysteroid (17-beta) dehydrogenase 7	0.62	0.66	1.08	0.49
PGPEP1	PGPEP1;FLJ20208;MGC10812;PGP;PGP-I;PGPI;Pcp	pyroglutamyl-peptidase I	0.62	0.62	1.08	0.47
NELL1	NELL1;FLJ45906;IDH3GL;NRP1	NEL-like 1 (chicken)	0.61	0.64	1.08	0.45
IFITM1	IFITM1;9-27;CD225;IFI17;LEU13	interferon induced transmembrane protein 1 (9-27)	0.61	0.69	1.08	0.49
ATG5	ATG5;APG5;APG5-LIKE;APG5L;ASP;hAPG5	ATG5 autophagy related 5 homolog (S. cerevisiae)	0.61	0.65	1.08	0.48
NACC1	NACC1;BEND8;BTBD14B;FLJ37383;NAC-1;NAC1	nucleus accumbens associated 1, BEN and BTB (POZ) domain containing	0.61	0.68	1.08	0.46
PKHD1	PKHD1;ARPKD;DKFZp686C01112;FCYT;FLJ46150;TIGM1	polycystic kidney and hepatic disease 1 (autosomal recessive)	0.84	0.94	1.09	0.71
KLHL38	KLHL38;C8ORFK36	kelch-like 38 (Drosophila)	0.84	0.9	1.09	0.67
SLC25A18	SLC25A18;GC2	solute carrier family 25 (mitochondrial carrier), member 18	0.84	0.9	1.09	0.67
SOX15	SOX15;SOX20;SOX26;SOX27	SRY (sex determining region Y)-box 15	0.84	0.97	1.09	0.64
SPTBN5	SPTBN5;BSPECV;HUBSPECV;HUSPECV	spectrin, beta, non-erythrocytic 5	0.84	0.82	1.09	0.69
MYOZ3	MYOZ3;CS-3;CS3;FRP3	myozenin 3	0.84	0.96	1.09	0.66
USP43	USP43;FLJ30626	ubiquitin specific peptidase 43	0.84	0.84	1.09	0.64
UBE2NL	UBE2NL;Li174	ubiquitin-conjugating enzyme E2N-like	0.84	0.84	1.09	0.63
CCDC106	CCDC106;HSU79303;ZNF581	coiled-coil domain containing 106	0.84	0.81	1.09	0.63
BLMH	BLMH;BH;BMH	bleomycin hydrolase	0.84	0.9	1.09	0.68

FOXE3	FOXE3;FKHL12;FREAC8	forkhead box E3	0.84	0.9	1.09	0.63
KAT2A	KAT2A;GCN5;GCN5L2;MGC102791;PCAF-b;hGCN5	K(lysine) acetyltransferase 2A	0.84	0.86	1.09	0.68
ARL5B	ARL5B;ARL8	ADP-ribosylation factor-like 5B	0.84	0.87	1.09	0.7
TNNC1	TNNC1;CMD1Z;CMH13;TN-C;TNC;TNNC	troponin C type 1 (slow)	0.84	0.88	1.09	0.67
LRRC3B	LRRC3B;LRP15;MGC102927	leucine rich repeat containing 3B	0.83	0.98	1.09	0.75
GUCA2B	GUCA2B;GCAP-II;UGN	guanylate cyclase activator 2B (uroguanylin)	0.83	0.88	1.09	0.62
SOX13	SOX13;ICA12;MGC117216;Sox-13	SRY (sex determining region Y)-box 13	0.83	0.94	1.09	0.64
HDX	HDX;CXorf43;D030011N01Rik;FLJ30678;MGC126769;MGC126771	highly divergent homeobox	0.82	0.94	1.09	0.67
TRAF3IP3	TRAF3IP3;DJ434O14.3;FLJ44151;MGC117354;MGC163289;T3JAM	TRAF3 interacting protein 3	0.82	0.89	1.09	0.74
RIMS2	RIMS2;DKFZp781A0653;KIAA0751;OBOE;RAB3IP3;RIM2	regulating synaptic membrane exocytosis 2	0.82	0.88	1.09	0.7
TAS2R60	TAS2R60;MGC119154;MGC119155;T2R56;T2R60	taste receptor, type 2, member 60	0.82	0.88	1.09	0.73
BAALC	BAALC;FLJ12015	brain and acute leukemia, cytoplasmic	0.82	0.95	1.09	0.77
ZNF280D	ZNF280D;MGC21637;MGC61687;SUHW4;ZNF634	zinc finger protein 280D	0.82	0.94	1.09	0.7
UCHL3	UCHL3;UCH-L3	ubiquitin carboxyl-terminal esterase L3 (ubiquitin thiolesterase)	0.82	0.82	1.09	0.62
C6ORF114	C6ORF114	ENTREZ STATUS: REMAPPED TO GENE ID: 54438	0.82	0.8	1.09	0.67
ITFG1	ITFG1;TIP	integrin alpha FG-GAP repeat containing 1	0.81	0.78	1.09	0.61
NEUROD4	NEUROD4;ATH-3;ATH3;Atoh3;MATH-3;bHLHa4	neurogenic differentiation 4	0.8	0.86	1.09	0.64
GLDN	GLDN;CLOM;COLM;CRG-L2;CRGL2;FLJ23917;UNC-112	gliomedin	0.8	0.93	1.09	0.65
DGKI	DGKI;DGK-IOTA	diacylglycerol kinase, iota	0.8	0.93	1.09	0.66
LMX1B	LMX1B;LMX1.2;MGC138325;MGC142051;NPS1	LIM homeobox transcription factor 1, beta	0.8	0.92	1.09	0.61
FAM19A5	FAM19A5;QLLK5208;TAFA-5;TAFA5;UNQ5208	family with sequence similarity 19 (chemokine (C-C motif)-like), member A5	0.8	0.94	1.09	0.72
PDCL	PDCL;DKFZp564M1863;PhLP	phosducin-like	0.8	0.84	1.09	0.6
ORAOV1	ORAOV1;TAOS1	oral cancer overexpressed 1	0.8	0.92	1.09	0.61
BCAS4	BCAS4;CNOL;FLJ20495	breast carcinoma amplified sequence 4	0.8	0.83	1.09	0.7
JUND	JUND;AP-1	jun D proto-oncogene	0.8	0.83	1.09	0.6
RSRC2	RSRC2;FLJ11021	arginine/serine-rich coiled-coil 2	0.8	0.9	1.09	0.65
PPP3CA	PPP3CA;CALN;CALNA;CALNA1;CCN1;CNA1;PPP2B	protein phosphatase 3, catalytic subunit, alpha isozyme	0.8	0.81	1.09	0.61
PDLIM5	PDLIM5;ENH;ENH1;LIM	PDZ and LIM domain 5	0.8	0.92	1.09	0.61
FAM199X	FAM199X;CXorf39	family with sequence similarity 199, X-linked	0.8	0.88	1.09	0.61
ITK	ITK;EMT;LYK;MGC126257;MGC126258;PSCTK2	IL2-inducible T-cell kinase	0.79	0.86	1.09	0.71
APPL2	APPL2;DIP13B;FLJ10659	adaptor protein, phosphotyrosine interaction, PH domain and leucine zipper containing 2	0.79	0.84	1.09	0.63
ARHGEF7	ARHGEF7;BETA-PIX;COOL-1;COOL1;DKFZp686C12170;DKFZp761K1021;KIAA0142;KIAA0412;Nbla10314;P50;P50BP;P85;P85COOL1;P85SPR;PAK3;PIXB	Rho guanine nucleotide exchange factor (GEF) 7	0.79	0.83	1.09	0.61
ALK	ALK;CD246;NBLST3	anaplastic lymphoma receptor tyrosine kinase	0.78	0.92	1.09	0.65
ACTC1	ACTC1;ACTC;ASD5;CMD1R;CMH11;LVNC4	actin, alpha, cardiac muscle 1	0.78	0.92	1.09	0.64
THAP6	THAP6;MGC30052	THAP domain containing 6	0.78	0.88	1.09	0.67
DND1	DND1;MGC34750;RBMS4	dead end homolog 1 (zebrafish)	0.78	0.84	1.09	0.63
MMAB	MMAB;ATR;MGC20496;cblB;cob	methylmalonic aciduria (cobalamin deficiency) cblB type	0.78	0.85	1.09	0.66
GSTCD	GSTCD;DKFZp686I10174;FLJ13273	glutathione S-transferase, C-terminal domain containing	0.78	0.85	1.09	0.62
MPI	MPI;CDG1B;FLJ39201;PMI;PMI1	mannose phosphate isomerase	0.78	0.94	1.09	0.65
PANK1	PANK1;MGC24596;PANK	pantothenate kinase 1	0.78	0.86	1.09	0.71
ATP6V0A2	ATP6V0A2;A2;ARCL;ATP6A2;ATP6N1D;J6B7;RTF;STV1;TJ6;TJ6M;TJ6S;VPH1;WSS	ATPase, H+ transporting, lysosomal V0 subunit a2	0.78	0.89	1.09	0.63
DHX30	DHX30;DDX30;FLJ11214;KIAA0890;RETCOR	DEAH (Asp-Glu-Ala-His) box polypeptide 30	0.78	0.79	1.09	0.63
ZNF121	ZNF121;D19S204;ZHC32;ZNF20	zinc finger protein 121	0.78	0.87	1.09	0.58
MYO1D	MYO1D;KIAA0727;myr4	myosin ID	0.78	0.88	1.09	0.67
AKAP8	AKAP8;AKAP 95;AKAP-8;AKAP-95;AKAP95;DKFZp586B1222	A kinase (PRKA) anchor protein 8	0.78	0.8	1.09	0.63
ZNF217	ZNF217;ZABC1	zinc finger protein 217	0.78	0.9	1.09	0.72
NUP153	NUP153;HNUP153;N153	nucleoporin 153kDa	0.78	0.82	1.09	0.6
ZNF658B	ZNF658B;MGC168709	zinc finger protein 658B, pseudogene	0.78	0.93	1.09	0.62
XKR8	XKR8;FLJ10307;RP11-460I13.3;XRG8	XK, Kell blood group complex subunit-related family, member 8	0.77	0.9	1.09	0.69
HS2ST1	HS2ST1;FLJ11317;KIAA0448;MGC131986;dJ604K5.2	heparan sulfate 2-O-sulfotransferase 1	0.77	0.82	1.09	0.64
ISCU	ISCU;2310020H20Rik;HML;ISU2;MGC74517;NIFU;NIFUN;hnifU	iron-sulfur cluster scaffold homolog (E. coli)	0.77	0.9	1.09	0.63
TMEFF2	TMEFF2;CT120.2;HPP1;TENB2;TPEF;TR	transmembrane protein with EGF-like and two follistatin-like domains 2	0.76	0.86	1.09	0.65
NR4A3	NR4A3;CHN;CSMF;MINOR;NOR1;TEC	nuclear receptor subfamily 4, group A, member 3	0.76	0.84	1.09	0.67
STAC2	STAC2;24b2;24b2/STAC2;MGC129694	SH3 and cysteine rich domain 2	0.76	0.84	1.09	0.58
EN1	EN1	engrailed homeobox 1	0.76	0.87	1.09	0.57
ANKRD26	ANKRD26;KIAA1074;MGC163325;THC2;bA145E8.1	ankyrin repeat domain 26	0.76	0.84	1.09	0.58
C14orf21	C14orf21;KIAA2021	chromosome 14 open reading frame 21	0.76	0.85	1.09	0.57
DAGLA	DAGLA;C11orf11;DAGL(ALPHA);DAGLALPHA;NSDDR	diacylglycerol lipase, alpha	0.76	0.8	1.09	0.64
BEX4	BEX4;BEXL1;FLJ10097	brain expressed, X-linked 4	0.76	0.88	1.09	0.62
TYK2	TYK2;JTK1	tyrosine kinase 2	0.76	0.88	1.09	0.63
SGOL2	SGOL2;FLJ25211;MGC129665;SGO2;TRIPIN	shugoshin-like 2 (S. pombe)	0.76	0.81	1.09	0.61
ARMC1	ARMC1;Arcp;FLJ10511	armadillo repeat containing 1	0.76	0.86	1.09	0.64
KIAA1715	KIAA1715;DKFZp686G1442;LNP;MGC102862;Ul;ulnaless	KIAA1715	0.76	0.86	1.09	0.65
LAP3	LAP3;LAP;LAPEP;PEPS	leucine aminopeptidase 3	0.76	0.82	1.09	0.62
TUBGCP3	TUBGCP3;GCP3;SPBC98;Spc98p	tubulin, gamma complex associated protein 3	0.76	0.8	1.09	0.61
FOXN3	FOXN3;C14orf116;CHES1;PRO1635	forkhead box N3	0.76	0.79	1.09	0.62
PAICS	PAICS;ADE2;ADE2H1;AIRC;DKFZp781N1372;MGC1343;MGC5024;PAIS	phosphoribosylaminoimidazole carboxylase, phosphoribosylaminoimidazole succinocarboxamide synthetase	0.76	0.8	1.09	0.58
SLC22A12	SLC22A12;OAT4L;RST;URAT1	solute carrier family 22 (organic anion/urate transporter), member 12	0.75	0.79	1.09	0.58
SLC34A1	SLC34A1;FRTS2;NAPI-3;NPHLOP1;NPT2;NPTIIa;SLC11;SLC17A2	solute carrier family 34 (sodium phosphate), member 1	0.75	0.87	1.09	0.65
B9D2	B9D2;ICIS-1;MGC4093;MKS10;MKSR2	B9 protein domain 2	0.75	0.8	1.09	0.6
CABLES1	CABLES1;CABL1;CABLES;FLJ35924;HsT2563;IK3-1	Cdk5 and Abl enzyme substrate 1	0.75	0.89	1.09	0.65
ATF7IP	ATF7IP;AM;FLJ10139;FLJ10688;MCAF;MCAF1;p621	activating transcription factor 7 interacting protein	0.75	0.76	1.09	0.67
PCYT1A	PCYT1A;CCTA;CT;CTA;CTPCT;PCYT1	phosphate cytidylyltransferase 1, choline, alpha	0.75	0.83	1.09	0.64
MAGEB10	MAGEB10;FLJ32965;MGC120394	melanoma antigen family B, 10	0.74	0.73	1.09	0.57
SPACA3	SPACA3;1700025M08Rik;ALLP17;CT54;LYC3;LYZL3;MGC119058;SLLP1	sperm acrosome associated 3	0.74	0.76	1.09	0.67
C9orf156	C9orf156;HSPC219;NAP1	chromosome 9 open reading frame 156	0.74	0.82	1.09	0.55
PDE9A	PDE9A;FLJ90181;HSPDE9A2	phosphodiesterase 9A	0.74	0.82	1.09	0.65
FAM86A	FAM86A;MGC19636	family with sequence similarity 86, member A	0.74	0.85	1.09	0.61
MANBA	MANBA;MANB1	mannosidase, beta A, lysosomal	0.74	0.78	1.09	0.61
UBE3A	UBE3A;ANCR;AS;E6-AP;EPVE6AP;FLJ26981;HPVE6A	ubiquitin protein ligase E3A	0.74	0.74	1.09	0.55
ZC3H7A	ZC3H7A;FLJ10027;FLJ20318;ZC3H7;ZC3HDC7	zinc finger CCCH-type containing 7A	0.74	0.8	1.09	0.58
PEA15	PEA15;HMAT1;HUMMAT1H;MAT1;MAT1H;PEA-15;PED	phosphoprotein enriched in astrocytes 15	0.74	0.78	1.09	0.58
C12orf71	C12orf71	chromosome 12 open reading frame 71	0.74	0.82	1.09	0.63
TUBB4A	TUBB4A;TUBB4;TUBB5;beta-5	tubulin, beta 4A class IVa	0.74	0.76	1.09	0.64
MS4A8B	MS4A8B;4SPAN4;MS4A4	membrane-spanning 4-domains, subfamily A, member 8B	0.73	0.85	1.09	0.64
C2orf51	C2orf51;FLJ25369;TSC21	chromosome 2 open reading frame 51	0.73	0.79	1.09	0.57
NRBF2	NRBF2;COPR1;COPR2;DKFZp564C1664;FLJ30395;NRBF-2	nuclear receptor binding factor 2	0.73	0.76	1.09	0.55
ZFP36L1	ZFP36L1;BRF1;Berg36;ERF-1;ERF1;RNF162B;TIS11B;cMG1	zinc finger protein 36, C3H type-like 1	0.73	0.76	1.09	0.55
MYLIP	MYLIP;IDOL;MIR	myosin regulatory light chain interacting protein	0.73	0.78	1.09	0.71
PORCN	PORCN;DHOF;FODH;MG61;MGC29687;PORC;PPN;por	porcupine homolog (Drosophila)	0.73	0.74	1.09	0.59
FAM71F2	FAM71F2;FAM137B	family with sequence similarity 71, member F2	0.72	0.81	1.09	0.62
SLA2	SLA2;C20orf156;FLJ21992;MGC49845;SLAP-2;SLAP2	Src-like-adaptor 2	0.72	0.71	1.09	0.59
DNAH10	DNAH10;FLJ38262;FLJ43486;FLJ43808;KIAA2017	dynein, axonemal, heavy chain 10	0.72	0.82	1.09	0.62
LRRC48	LRRC48;DKFZp586M1120	leucine rich repeat containing 48	0.72	0.78	1.09	0.58
GPR156	GPR156;GABABL;MGC142261;PGR28	G protein-coupled receptor 156	0.72	0.73	1.09	0.55
HAND1	HAND1;Hxt;Thing1;bHLHa27;eHand	heart and neural crest derivatives expressed 1	0.72	0.78	1.09	0.61
SETBP1	SETBP1;DKFZp666J1210;KIAA0437;SEB	SET binding protein 1	0.72	0.81	1.09	0.61
SAMD11	SAMD11;MGC45873	sterile alpha motif domain containing 11	0.72	0.75	1.09	0.58
CCDC107	CCDC107;FLJ97171;MGC31967;PSEC0222	coiled-coil domain containing 107	0.72	0.81	1.09	0.58
WDR48	WDR48;DKFZp686G1794;KIAA1449;P80;UAF1	WD repeat domain 48	0.72	0.83	1.09	0.64
NFE2L1	NFE2L1;FLJ00380;LCR-F1;NRF1;TCF11	nuclear factor (erythroid-derived 2)-like 1	0.72	0.74	1.09	0.58
CEP55	CEP55;C10orf3;CT111;FLJ10540;URCC6	centrosomal protein 55kDa	0.72	0.84	1.09	0.59
WWC2	WWC2;BOMB;FLJ22029;FLJ34082	WW and C2 domain containing 2	0.72	0.82	1.09	0.61
LAMTOR3	LAMTOR3;MAP2K1IP1;MAPBP;MAPKSP1;MP1;PRO0633;Ragulator3	late endosomal/lysosomal adaptor, MAPK and MTOR activator 3	0.72	0.84	1.09	0.55
GTSE1	GTSE1;B99	G-2 and S-phase expressed 1	0.71	0.77	1.09	0.57
C17orf108	C17orf108	chromosome 17 open reading frame 108	0.71	0.72	1.09	0.61
GJA5	GJA5;ATFB11;CX40;MGC11185	gap junction protein, alpha 5, 40kDa	0.7	0.71	1.09	0.56
TAAR5	TAAR5;MGC138414;MGC138416;PNR;RP11-295F4.5	trace amine associated receptor 5	0.7	0.72	1.09	0.54
MATN1	MATN1;CMP;CRTM	matrilin 1, cartilage matrix protein	0.7	0.8	1.09	0.56
KIAA0913	KIAA0913;FLJ34302;MGC168869;MGC168870	KIAA0913	0.7	0.78	1.09	0.56
TTC39B	TTC39B;C9orf52;FLJ33868	tetratricopeptide repeat domain 39B	0.7	0.78	1.09	0.57
GLTPD1	GLTPD1;MGC10334	glycolipid transfer protein domain containing 1	0.7	0.79	1.09	0.64
PTDSS2	PTDSS2;FLJ46373;PSS2	phosphatidylserine synthase 2	0.7	0.78	1.09	0.52
RBMS3	RBMS3;FLJ36544	RNA binding motif, single stranded interacting protein 3	0.7	0.77	1.09	0.52
CMTM4	CMTM4;CKLFSF4	CKLF-like MARVEL transmembrane domain containing 4	0.7	0.79	1.09	0.59
DCPS	DCPS;HINT-5;HSL1	decapping enzyme, scavenger	0.7	0.86	1.09	0.59
CHMP1B	CHMP1B;C10orf2;C18-ORF2;C18orf2;CHMP1.5;Vps46-2;Vps46B;hVps46-2	charged multivesicular body protein 1B	0.7	0.84	1.09	0.56
NIT1	NIT1;MGC57670	nitrilase 1	0.7	0.84	1.09	0.58
KIAA0319L	KIAA0319L;FLJ44532;KIAA1837	KIAA0319-like	0.7	0.78	1.09	0.54
NOTCH2NL	NOTCH2NL;FLJ35032;FLJ55807;N2N	notch 2 N-terminal like	0.7	0.68	1.09	0.56
RHOA	RHOA;ARH12;ARHA;RHO12;RHOH12	ras homolog gene family, member A	0.7	0.8	1.09	0.62
C1orf226	C1orf226;FLJ13137;FLJ43132;FLJ55152	chromosome 1 open reading frame 226	0.7	0.72	1.09	0.58
SSX4B	SSX4B;CT5.4;MGC169015;MGC169016;SSX4	synovial sarcoma, X breakpoint 4B	0.7	0.79	1.09	0.59
MDGA2	MDGA2;MAMDC1;c14_5286	MAM domain containing glycosylphosphatidylinositol anchor 2	0.69	0.67	1.09	0.53
COL10A1	COL10A1	collagen, type X, alpha 1	0.69	0.73	1.09	0.59
KRT75	KRT75;K6HF;KB18;PFB	keratin 75	0.69	0.8	1.09	0.56
DUOXA2	DUOXA2;SIMNIPHOM;TDH5	dual oxidase maturation factor 2	0.69	0.73	1.09	0.56
LETM2	LETM2;FLJ25409	leucine zipper-EF-hand containing transmembrane protein 2	0.69	0.68	1.09	0.56
KRT6C	KRT6C;K6E;KRT6E;MGC102925;MGC163455;MGC163457	keratin 6C	0.69	0.72	1.09	0.58

GNAQ	GNAQ;G-ALPHA-q;GAQ	guanine nucleotide binding protein (G protein), q polypeptide	0.69	0.68	1.09	0.57
CBLN4	CBLN4;CBLNL1	cerebellin 4 precursor	0.68	0.8	1.09	0.55
ZNF25	ZNF25;DKFZp564C206;FLJ31890;KOX19;Zfp9	zinc finger protein 25	0.68	0.78	1.09	0.52
APOC4	APOC4;APO-CIV;APOC-IV	apolipoprotein C-IV	0.68	0.7	1.09	0.51
D2HGDH	D2HGDH;D2HGD;FLJ42195;MGC25181	D-2-hydroxyglutarate dehydrogenase	0.68	0.77	1.09	0.52
B4GALNT1	B4GALNT1;GALGT;GALNACT	beta-1,4-N-acetyl-galactosaminyl transferase 1	0.68	0.73	1.09	0.57
NUPL2	NUPL2;CG1;NLP-1;NLP_1;hCG1	nucleoporin like 2	0.68	0.81	1.09	0.59
CDT1	CDT1;DUP;RIS2	chromatin licensing and DNA replication factor 1	0.68	0.66	1.09	0.55
MAP2K4	MAP2K4;JNKK;JNKK1;MAPKK4;MEK4;MKK4;PRKMK4;SEK1;SERK1	mitogen-activated protein kinase kinase 4	0.67	0.79	1.09	0.56
DDX47	DDX47;DKFZp564O176;E4-DBP;FLJ30012;HQ0256;MSTP162;RRP3	DEAD (Asp-Glu-Ala-Asp) box polypeptide 47	0.67	0.72	1.09	0.61
CKMT2	CKMT2;SMTCK	creatine kinase, mitochondrial 2 (sarcomeric)	0.66	0.72	1.09	0.59
ACAD10	ACAD10;MGC5601	acyl-CoA dehydrogenase family, member 10	0.66	0.68	1.09	0.49
GPR157	GPR157;FLJ12132	G protein-coupled receptor 157	0.66	0.7	1.09	0.63
ZNF580	ZNF580	zinc finger protein 580	0.66	0.74	1.09	0.51
TSEN2	TSEN2;MGC2776;MGC4440;PCH2B;SEN2;SEN2L	tRNA splicing endonuclease 2 homolog (S. cerevisiae)	0.66	0.66	1.09	0.53
CXorf26	CXorf26;MGC874	chromosome X open reading frame 26	0.66	0.71	1.09	0.59
CASP4	CASP4;ICE(rel)II;ICEREL-II;ICH-2;Mih1/TX;TX	caspase 4, apoptosis-related cysteine peptidase	0.66	0.72	1.09	0.54
TMEM33	TMEM33;1600019D15Rik;FLJ10525;SHINC3	transmembrane protein 33	0.66	0.75	1.09	0.5
CCDC124	CCDC124	coiled-coil domain containing 124	0.66	0.74	1.09	0.53
PSMC2	PSMC2;MGC3004;MSS1;Nbla10058;S7	proteasome (prosome, macropain) 26S subunit, ATPase, 2	0.66	0.7	1.09	0.5
UBOX5	UBOX5;KIAA0860;RNF37;UBCE7IP5;UIP5	U-box domain containing 5	0.66	0.72	1.09	0.64
OR2A42	OR2A42;OR2A1	olfactory receptor, family 2, subfamily A, member 42	0.66	0.68	1.09	0.49
SP6	SP6;EPFN;EPIPROFIN;KLF14;MGC119662;MGC119663;MGC119664;MGC119665	Sp6 transcription factor	0.65	0.68	1.09	0.49
CPLX3	CPLX3;CPX-III;CPXIII;DKFZp434E1719;FLJ00167;FLJ00250;FLJ13993	complexin 3	0.65	0.68	1.09	0.49
PIGB	PIGB;MGC21236	phosphatidylinositol glycan anchor biosynthesis, class B	0.65	0.66	1.09	0.53
IPO4	IPO4;FLJ23338;Imp4;MGC131665	importin 4	0.65	0.73	1.09	0.55
LGALS3BP	LGALS3BP;90K;BTBD17B;MAC-2-BP	lectin, galactoside-binding, soluble, 3 binding protein	0.65	0.65	1.09	0.58
CADPS	CADPS;CADPS1;CAPS;CAPS1;KIAA1121	Ca++-dependent secretion activator	0.64	0.71	1.09	0.51
POM121L2	POM121L2;POM121-L;POM121L	POM121 membrane glycoprotein-like 2	0.64	0.66	1.09	0.51
C11orf40	C11orf40;NOV1	chromosome 11 open reading frame 40	0.64	0.67	1.09	0.51
OR1D4	OR1D4	ENTREZ STATUS: REMAPPED TO GENE ID: 653166	0.64	0.72	1.09	0.54
WNT9A	WNT9A;MGC138165;MGC141991;WNT14	wingless-type MMTV integration site family, member 9A	0.64	0.74	1.09	0.49
PKMYT1	PKMYT1;DKFZp547K1610;FLJ20093;MYT1	protein kinase, membrane associated tyrosine/threonine 1	0.64	0.75	1.09	0.53
C1orf56	C1orf56;FLJ20519;RP11-316M1.9	chromosome 1 open reading frame 56	0.64	0.7	1.09	0.5
TPD52	TPD52;D52;N8L;PC-1;PrLZ;hD52	tumor protein D52	0.64	0.73	1.09	0.52
HAPLN4	HAPLN4;BRAL2;KIAA1926	hyaluronan and proteoglycan link protein 4	0.64	0.72	1.09	0.54
LBH	LBH;DKFZp566J091;MGC104312;MGC163287	limb bud and heart development homolog (mouse)	0.64	0.68	1.09	0.53
CASC5	CASC5;AF15Q14;CT29;D40;KIAA1570;KNL1;PPP1R55;hKNL-1;hSpc105	cancer susceptibility candidate 5	0.64	0.63	1.09	0.49
IGF2BP3	IGF2BP3;CT98;DKFZp686F1078;IMP-3;IMP3;KOC1;VICKZ3	insulin-like growth factor 2 mRNA binding protein 3	0.64	0.66	1.09	0.54
STX4	STX4;STX4A;p35-2	syntaxin 4	0.64	0.75	1.09	0.58
KIAA1191	KIAA1191;FLJ21022;p60MONOX	KIAA1191	0.64	0.73	1.09	0.49
NOP58	NOP58;NOP5;NOP5/NOP58	NOP58 ribonucleoprotein homolog (yeast)	0.64	0.68	1.09	0.52
DGAT2	DGAT2;DKFZp686A15125;GS1999FULL	diacylglycerol O-acyltransferase 2	0.63	0.7	1.09	0.55
DIXDC1	DIXDC1;CCD1;KIAA1735	DIX domain containing 1	0.62	0.72	1.09	0.58
ZNF830	ZNF830;CCDC16;MGC20398;OMCG1;SEL13	zinc finger protein 830	0.62	0.74	1.09	0.55
HIC1	HIC1;ZBTB29;ZNF901;hic-1	hypermethylated in cancer 1	0.62	0.66	1.09	0.49
COBRA1	COBRA1;DKFZp586B0519;KIAA1182;NELF-B;NELFB	cofactor of BRCA1	0.62	0.74	1.09	0.54
SLC6A8	SLC6A8;CRT;CRTR;CT1;MGC87396	solute carrier family 6 (neurotransmitter transporter, creatine), member 8	0.62	0.65	1.09	0.51
ACACA	ACACA;ACAC;ACACAD;ACC;ACC1;ACCA	acetyl-CoA carboxylase alpha	0.62	0.68	1.09	0.53
ATOX1	ATOX1;ATX1;HAH1;MGC138453;MGC138455	ATX1 antioxidant protein 1 homolog (yeast)	0.62	0.74	1.09	0.54
FAM84B	FAM84B;BCMP101;NSE2	family with sequence similarity 84, member B	0.62	0.66	1.09	0.49
AHCTF1	AHCTF1;DKFZp434N093;ELYS;MST108;TMBS62	AT hook containing transcription factor 1	0.62	0.68	1.09	0.5
DCAF12L2	DCAF12L2;WDR40C	DDB1 and CUL4 associated factor 12-like 2	0.62	0.66	1.09	0.48
TOMM20L	TOMM20L;UNQ9438	translocase of outer mitochondrial membrane 20 homolog (yeast)-like	0.84	0.9	1.1	0.75
HTR3D	HTR3D;5HT3D;MGC119636;MGC119637	5-hydroxytryptamine (serotonin) receptor 3 family member D	0.84	0.94	1.1	0.72
CDCP2	CDCP2	CUB domain containing protein 2	0.84	0.98	1.1	0.68
SP100	SP100;DKFZp686E07254;FLJ00340;FLJ34579;lysp100b	SP100 nuclear antigen	0.84	0.98	1.1	0.79
PCMTD2	PCMTD2;C20orf36;FLJ10883;KIAA0835;KIAA1050	protein-L-isoaspartate (D-aspartate) O-methyltransferase domain containing 2	0.84	0.88	1.1	0.63
GALNTL6	GALNTL6;GALNT17;MGC44629	UDP-N-acetyl-alpha-D-galactosamine:polypeptide N-acetylgalactosaminyltransferase-like 6	0.84	0.92	1.1	0.66
NBPF9	NBPF9;AE01;FLJ37711;FLJ60244;KIAA1245;NBPF10	neuroblastoma breakpoint family, member 9	0.84	1	1.1	0.66
TSGA13	TSGA13	testis specific, 13	0.84	0.9	1.1	0.72
C6orf222	C6orf222;DKFZp779B1540	chromosome 6 open reading frame 222	0.83	0.98	1.1	0.68
SEMA3B	SEMA3B;FLJ34863;LUCA-1;SEMA5;SEMAA;SemA;semaV	sema domain, immunoglobulin domain (Ig), short basic domain, secreted, (semaphorin) 3B	0.83	0.92	1.1	0.63
DOPEY1	DOPEY1;FLJ35610;KIAA1117;dJ202D23.2	dopey family member 1	0.83	0.94	1.1	0.69
RNH1	RNH1;MGC18200;MGC4569;MGC54054;RAI;RNH	ribonuclease/angiogenin inhibitor 1	0.83	0.94	1.1	0.67
ARHGEF5	ARHGEF5;DKFZp686N1969;GEF5;P60;TIM;TIM1	Rho guanine nucleotide exchange factor (GEF) 5	0.83	0.89	1.1	0.7
VRK2	VRK2	vaccinia related kinase 2	0.83	0.99	1.1	0.7
SLC20A2	SLC20A2;GLVR2;Glvr-2;MLVAR;PIT-2	solute carrier family 20 (phosphate transporter), member 2	0.83	0.87	1.1	0.63
CACNA1I	CACNA1I;Cav3.3;KIAA1120;ca(v)3.3	calcium channel, voltage-dependent, T type, alpha 1I subunit	0.82	0.92	1.1	0.72
PLD2	PLD2	phospholipase D2	0.82	0.88	1.1	0.62
PKN3	PKN3;RP11-545E17.1	protein kinase N3	0.82	0.9	1.1	0.75
SFXN4	SFXN4;BCRM1	sideroflexin 4	0.82	0.86	1.1	0.62
KIAA0196	KIAA0196;MGC111053;SPG8	KIAA0196	0.82	0.89	1.1	0.74
HEBP1	HEBP1;HBP;HEBP	heme binding protein 1	0.82	0.96	1.1	0.77
TTC37	TTC37;KIAA0372;MGC32587	tetratricopeptide repeat domain 37	0.82	0.86	1.1	0.66
SLC22A5	SLC22A5;CDSP;FLJ46769;OCTN2;OCTN2VT	solute carrier family 22 (organic cation/carnitine transporter), member 5	0.82	0.9	1.1	0.67
TSPAN12	TSPAN12;EVR5;NET-2;NET2;TM4SF12	tetraspanin 12	0.82	0.88	1.1	0.71
CMTM6	CMTM6;CKLFSF6;FLJ20396;PRO2219	CKLF-like MARVEL transmembrane domain containing 6	0.82	0.85	1.1	0.69
WDR12	WDR12;FLJ10881;FLJ12719;FLJ12720;YTM1	WD repeat domain 12	0.82	0.87	1.1	0.68
RSRC1	RSRC1;MGC12197;SFRS21;SRrp53	arginine/serine-rich coiled-coil 1	0.82	0.88	1.1	0.66
PON2	PON2	paraoxonase 2	0.82	0.83	1.1	0.63
STOX1	STOX1;C10orf24;PEE4	storkhead box 1	0.81	0.98	1.1	0.72
LSM6	LSM6;YDR378C	LSM6 homolog, U6 small nuclear RNA associated (S. cerevisiae)	0.81	0.92	1.1	0.7
OPRD1	OPRD1;OPRD	opioid receptor, delta 1	0.81	0.86	1.1	0.77
ADAMTS5	ADAMTS5;ADAM-TS 11;ADAM-TS 5;ADAM-TS5;ADAMTS-11;ADAMTS-5;ADAMTS11;ADMP-2;FLJ36738	ADAM metallopeptidase with thrombospondin type 1 motif, 5	0.81	0.88	1.1	0.66
DLX6	DLX6;MGC125282;MGC125283;MGC125284;MGC125285	distal-less homeobox 6	0.81	0.9	1.1	0.6
KCNMB2	KCNMB2;MGC22431	potassium large conductance calcium-activated channel, subfamily M, beta member 2	0.8	0.88	1.1	0.68
EGFLAM	EGFLAM;AGRINL;AGRNL;FLJ39155	EGF-like, fibronectin type III and laminin G domains	0.8	0.85	1.1	0.65
PGBD5	PGBD5;DKFZp761A0620;FLJ11413	piggyBac transposable element derived 5	0.8	0.96	1.1	0.71
TRIM10	TRIM10;HERF1;MGC141979;RFB30;RNF9	tripartite motif containing 10	0.8	0.88	1.1	0.78
SLC7A9	SLC7A9;BAT1;CSNU3;FLJ94301	solute carrier family 7 (glycoprotein-associated amino acid transporter light chain, bo,+ system), member 9	0.8	0.85	1.1	0.63
PKNOX2	PKNOX2;FLJ13074;PREP2	PBX/knotted 1 homeobox 2	0.8	0.9	1.1	0.68
EDARADD	EDARADD;ED3;EDA3	EDAR-associated death domain	0.8	0.91	1.1	0.69
FGF13	FGF13;FGF-13;FGF2;FHF-2;FHF2	fibroblast growth factor 13	0.8	0.93	1.1	0.61
PLXND1	PLXND1;KIAA0620;MGC75353;PLEXD1	plexin D1	0.8	0.88	1.1	0.61
FBXW5	FBXW5;DKFZp434B205;Fbw5;MGC20962;RP11-229P13.10	F-box and WD repeat domain containing 5	0.8	0.83	1.1	0.6
BAG4	BAG4;BAG-4;DKFZp586O2022;SODD	BCL2-associated athanogene 4	0.8	0.93	1.1	0.61
C19orf63	C19orf63;HSM1;HSS1;INM02;MGC33203	chromosome 19 open reading frame 63	0.8	0.92	1.1	0.69
NCCRP1	NCCRP1;NCCRP-1	non-specific cytotoxic cell receptor protein 1 homolog (zebrafish)	0.8	0.84	1.1	0.6
ZNF404	ZNF404;MGC120455	zinc finger protein 404	0.79	0.85	1.1	0.64
PFN4	PFN4	profilin family, member 4	0.79	0.78	1.1	0.63
FSTL4	FSTL4	follistatin-like 4	0.78	0.88	1.1	0.68
PIGL	PIGL	phosphatidylinositol glycan anchor biosynthesis, class L	0.78	0.83	1.1	0.71
FAM83E	FAM83E;FLJ20200;MGC138175;MGC138177	family with sequence similarity 83, member E	0.78	0.84	1.1	0.63
BRD4	BRD4;CAP;HUNK1;HUNKI;MCAP	bromodomain containing 4	0.78	0.94	1.1	0.67
B4GALT7	B4GALT7;XGALT1;XGPT1	xylosylprotein beta 1,4-galactosyltransferase, polypeptide 7 (galactosyltransferase I)	0.78	0.82	1.1	0.6
CD276	CD276;4Ig-B7-H3;B7-H3;B7H3	CD276 molecule	0.78	0.86	1.1	0.59
ERAP1	ERAP1;A-LAP;ALAP;APPILS;ARTS-1;ARTS1;ERAAP;ERAAP1;KIAA0525;PILS-AP;PILSAP	endoplasmic reticulum aminopeptidase 1	0.78	0.86	1.1	0.64
GPR151	GPR151;GALR4;GALRL;GPCR;PGR7	G protein-coupled receptor 151	0.77	0.82	1.1	0.74
C10orf68	C10orf68;FLJ13031;MGC149767;dJ1104A8.1	chromosome 10 open reading frame 68	0.77	0.78	1.1	0.62
KRT32	KRT32;HA2;HKA2;KRTHA2;hHa2	keratin 32	0.77	0.84	1.1	0.61
CSPG5	CSPG5;MGC44034;NGC	chondroitin sulfate proteoglycan 5 (neuroglycan C)	0.77	0.88	1.1	0.66
ZNF138	ZNF138;pHZ-32	zinc finger protein 138	0.77	0.86	1.1	0.63
PEX6	PEX6;PAF-2;PAF2;PXAAA1	peroxisomal biogenesis factor 6	0.77	0.86	1.1	0.69
FAM55C	FAM55C;FLJ30102;MGC15606;MST115	family with sequence similarity 55, member C	0.77	0.8	1.1	0.65
AGTR1	AGTR1;AG2S;AGTR1A;AGTR1B;AT1;AT1B;AT1R;AT2R1;AT2R1A;AT2R1B;HAT1R	angiotensin II receptor, type 1	0.76	0.81	1.1	0.73
ACSL6	ACSL6;ACS2;FACL6;FLJ16173;KIAA0837;LACS 6;LACS2;LACS5	acyl-CoA synthetase long-chain family member 6	0.76	0.78	1.1	0.68
ANK2	ANK2;ANK-2;DKFZp686M09125;DKFZp686P0948;FLJ38277;LQT4;brank-2	ankyrin 2, neuronal	0.76	0.92	1.1	0.64
MMRN2	MMRN2;EMILIN-3;EMILIN3;EndoGlyx-1;FLJ13465	multimerin 2	0.76	0.86	1.1	0.65
SUFU	SUFU;PRO1280;SUFUH;SUFUXL	suppressor of fused homolog (Drosophila)	0.76	0.83	1.1	0.62
WDR19	WDR19;DYF-2;FLJ23127;IFT144;KIAA1638;ORF26;Oseg6;PWDMP	WD repeat domain 19	0.76	0.82	1.1	0.61
PCCA	PCCA	propionyl CoA carboxylase, alpha polypeptide	0.76	0.81	1.1	0.6
RAB3IL1	RAB3IL1;GRAB	RAB3A interacting protein (rabin3)-like 1	0.76	0.82	1.1	0.61
FAM43A	FAM43A;FLJ90022	family with sequence similarity 43, member A	0.76	0.74	1.1	0.62
MAGEE1	MAGEE1;DAMAGE;HCA1;KIAA1587	melanoma antigen family E, 1	0.76	0.82	1.1	0.66
AADAT	AADAT;KAT2;KATII	aminoadipate aminotransferase	0.76	0.82	1.1	0.64
ZFP30	ZFP30;KIAA0961;ZNF745	zinc finger protein 30 homolog (mouse)	0.76	0.87	1.1	0.63
C1orf122	C1orf122;ALAESM;FLJ23476;FLJ45459	chromosome 1 open reading frame 122	0.76	0.83	1.1	0.6
TGFB2	TGFB2;MGC116892;TGF-beta2	transforming growth factor, beta 2	0.76	0.8	1.1	0.57

MOCS3	MOCS3;MGC9252;UBA4;dJ914P20.3	molybdenum cofactor synthesis 3	0.76	0.8	1.1	0.58
NIPAL3	NIPAL3;DJ462O23.2;DKFZp686E22155;NPAL3	NIPA-like domain containing 3	0.76	0.8	1.1	0.62
C14orf166B	C14orf166B	chromosome 14 open reading frame 166B	0.75	0.85	1.1	0.63
PCDHB5	PCDHB5;DKFZp586B0217;PCDH-BETA5	protocadherin beta 5	0.75	0.84	1.1	0.65
THRA	THRA;AR7;EAR7;ERB-T-1;ERBA;ERBA1;MGC000261;MGC43240;NR1A1;THRA1;THRA2;c-ERBA-1	thyroid hormone receptor, alpha	0.75	0.87	1.1	0.58
EML2	EML2;ELP70;EMAP-2;EMAP2	echinoderm microtubule associated protein like 2	0.75	0.84	1.1	0.64
LIPG	LIPG;EDL;EL;PRO719	lipase, endothelial	0.75	0.83	1.1	0.65
FOXN1	FOXN1;FKHL20;RONU;WHN	forkhead box N1	0.74	0.8	1.1	0.58
RNF212	RNF212;FLJ38841;FLJ42587;MGC120227;MGC120228;ZHP3	ring finger protein 212	0.74	0.72	1.1	0.57
CTSW	CTSW;LYPN	cathepsin W	0.74	0.8	1.1	0.6
TRIM31	TRIM31;C6orf13;HCG1;HCGI;RNF	tripartite motif containing 31	0.74	0.8	1.1	0.72
AMT	AMT;GCE;GCST;GCVT;NKH	aminomethyltransferase	0.74	0.83	1.1	0.56
SGTB	SGTB;FLJ39002;SGT2	small glutamine-rich tetratricopeptide repeat (TPR)-containing, beta	0.74	0.79	1.1	0.6
SLC35C1	SLC35C1;CDG2C;FLJ11320;FLJ14841;FUCT1	solute carrier family 35, member C1	0.74	0.8	1.1	0.56
DUSP7	DUSP7;DKFZp586F2224;MKPX;PYST2	dual specificity phosphatase 7	0.74	0.76	1.1	0.57
CCNE2	CCNE2;CYCE2	cyclin E2	0.74	0.82	1.1	0.55
ADSL	ADSL;AMPS;ASASE;ASL	adenylosuccinate lyase	0.74	0.81	1.1	0.55
PRCC	PRCC;MGC17178;MGC4723;RCCP1;TPRC	papillary renal cell carcinoma (translocation-associated)	0.74	0.84	1.1	0.64
HSP90AB1	HSP90AB1;D6S182;FLJ26984;HSP90-BETA;HSP90B;HSPC2;HSPCB	heat shock protein 90kDa alpha (cytosolic), class B member 1	0.74	0.8	1.1	0.69
PEX11A	PEX11A;MGC119947;MGC138534;PEX11-ALPHA	peroxisomal biogenesis factor 11 alpha	0.73	0.76	1.1	0.59
HCLS1	HCLS1;CTTNL;HS1	hematopoietic cell-specific Lyn substrate 1	0.73	0.82	1.1	0.62
USP5	USP5;ISOT	ubiquitin specific peptidase 5 (isopeptidase T)	0.73	0.79	1.1	0.59
LOC120824	LOC120824	tripartite motif-containing protein ENSP00000309378-like	0.73	0.79	1.1	0.71
LCNL1	LCNL1;FLJ45224	lipocalin-like 1	0.73	0.73	1.1	0.58
OR10T2	OR10T2;OR1-3	olfactory receptor, family 10, subfamily T, member 2	0.72	0.82	1.1	0.56
CCDC11	CCDC11;FLJ32743	coiled-coil domain containing 11	0.72	0.79	1.1	0.57
OR10G3	OR10G3;OR14-40	olfactory receptor, family 10, subfamily G, member 3	0.72	0.84	1.1	0.68
PDE3A	PDE3A;CGI-PDE;CGI-PDE A;CGI-PDE-A;FLJ37884	phosphodiesterase 3A, cGMP-inhibited	0.72	0.77	1.1	0.6
GPR31	GPR31;12-HETER;HETER;HETER1	G protein-coupled receptor 31	0.72	0.74	1.1	0.56
CENPH	CENPH	centromere protein H	0.72	0.78	1.1	0.58
C3orf18	C3orf18;G20	chromosome 3 open reading frame 18	0.72	0.82	1.1	0.61
LBX2	LBX2	ladybird homeobox 2	0.72	0.82	1.1	0.67
ENTPD2	ENTPD2;CD39L1;NTPDase-2	ectonucleoside triphosphate diphosphohydrolase 2	0.72	0.76	1.1	0.55
N4BP2	N4BP2;B3BP;FLJ10680;KIAA1413	NEDD4 binding protein 2	0.72	0.76	1.1	0.68
ZDHHC2	ZDHHC2;DHHC2;ZNF372	zinc finger, DHHC-type containing 2	0.72	0.71	1.1	0.59
WDR85	WDR85;C9orf112;FLJ90634;RP11-48C7.3	WD repeat domain 85	0.72	0.76	1.1	0.59
STK40	STK40;MGC4796;RP11-268J15.4;SHIK;SgK495	serine/threonine kinase 40	0.72	0.84	1.1	0.6
SLC35C2	SLC35C2;BA394O2.1;C20orf5;CGI-15;FLJ37039;FLJ46434;MGC20633;MGC32079;MGC39183;OVCOV1	solute carrier family 35, member C2	0.72	0.78	1.1	0.61
ANAPC7	ANAPC7;APC7	anaphase promoting complex subunit 7	0.72	0.83	1.1	0.61
PSPC1	PSPC1;DKFZp566B1447;FLJ10955;FLJ33554;PSP1	paraspeckle component 1	0.72	0.82	1.1	0.58
C11orf92	C11orf92;DKFZp781P19126;FLJ45803	chromosome 11 open reading frame 92	0.72	0.79	1.1	0.66
IGIP	IGIP;C5orf53	IgA-inducing protein homolog (Bos taurus)	0.72	0.79	1.1	0.54
CEACAM5	CEACAM5;CD66e;CEA;DKFZp781M2392	carcinoembryonic antigen-related cell adhesion molecule 5	0.71	0.81	1.1	0.61
IGSF6	IGSF6;DORA	immunoglobulin superfamily, member 6	0.71	0.82	1.1	0.58
MMP21	MMP21	matrix metallopeptidase 21	0.71	0.77	1.1	0.58
E2F6	E2F6;E2F-6;MGC111545	E2F transcription factor 6	0.71	0.87	1.1	0.6
BLOC1S1	BLOC1S1;BLOS1;FLJ39337;FLJ97089;GCN5L1;MGC87455;MICoA;RT14	biogenesis of lysosomal organelles complex-1, subunit 1	0.71	0.86	1.1	0.63
FAM98A	FAM98A;DKFZp564F0522;DKFZp686O03192	family with sequence similarity 98, member A	0.71	0.84	1.1	0.56
TMEM156	TMEM156;FLJ23235	transmembrane protein 156	0.7	0.84	1.1	0.64
CYP4A11	CYP4A11;CP4Y;CYP4A2;CYP4AII	cytochrome P450, family 4, subfamily A, polypeptide 11	0.7	0.8	1.1	0.62
DEFA4	DEFA4;DEF4;HNP-4;HP-4;HP4;MGC120099;MGC138296	defensin, alpha 4, corticostatin	0.7	0.74	1.1	0.62
PF4V1	PF4V1;CXCL4L1;CXCL4V1;PF4-ALT;PF4A;SCYB4V1	platelet factor 4 variant 1	0.7	0.8	1.1	0.64
TANC1	TANC1;FLJ13360;FLJ41166;KIAA1728;ROLSB;TANC	tetratricopeptide repeat, ankyrin repeat and coiled-coil containing 1	0.7	0.73	1.1	0.59
BTBD3	BTBD3;KIAA0952;MGC130038;MGC130039;dJ742J24.1	BTB (POZ) domain containing 3	0.7	0.8	1.1	0.6
CNPY3	CNPY3;CAG4A;ERDA5;PRAT4A;TNRC5	canopy 3 homolog (zebrafish)	0.7	0.74	1.1	0.53
LIX1	LIX1;C5orf11;FLJ25534	Lix1 homolog (chicken)	0.7	0.8	1.1	0.57
DDX24	DDX24	DEAD (Asp-Glu-Ala-Asp) box polypeptide 24	0.7	0.86	1.1	0.6
FLJ12363	FLJ12363	ENTREZ STATUS: REMAPPED TO GENE ID: 92017	0.7	0.73	1.1	0.59
FAIM2	FAIM2;KIAA0950;LFG;LFG2;NGP35;NMP35;TMBIM2	Fas apoptotic inhibitory molecule 2	0.69	0.78	1.1	0.56
FBXO38	FBXO38;Fbx38;MOKA	F-box protein 38	0.69	0.72	1.1	0.52
ZNRF4	ZNRF4;RNF204;SPERIZIN;Ssrzf1;spzn	zinc and ring finger 4	0.68	0.74	1.1	0.56
RHBDL2	RHBDL2;MGC16997;RRP2	rhomboid, veinlet-like 2 (Drosophila)	0.68	0.73	1.1	0.55
SFTPD	SFTPD;COLEC7;PSP-D;SFTP4;SP-D	surfactant protein D	0.68	0.77	1.1	0.58
PRODH2	PRODH2;HSPOX1	proline dehydrogenase (oxidase) 2	0.68	0.82	1.1	0.61
SETD6	SETD6;FLJ21148	SET domain containing 6	0.68	0.8	1.1	0.62
INO80B	INO80B;HMGA1L4;HMGIYL4;IES2;PAP-1BP;PAPA-1;ZNHIT4;hIes2	INO80 complex subunit B	0.68	0.74	1.1	0.56
SPRYD3	SPRYD3;FLJ14800	SPRY domain containing 3	0.68	0.75	1.1	0.54
DCTD	DCTD;MGC111062	dCMP deaminase	0.68	0.82	1.1	0.57
TAX1BP1	TAX1BP1;CALCOCO3;T6BP;TXBP151	Tax1 (human T-cell leukemia virus type I) binding protein 1	0.68	0.73	1.1	0.56
CENPW	CENPW;C6orf173;CUG2;DKFZp686L1553	centromere protein W	0.68	0.76	1.1	0.58
DUX5	DUX5;DUX1	double homeobox 5	0.68	0.7	1.1	0.59
PCDHA12	PCDHA12;MGC138485;MGC141932;PCDH-ALPHA12	protocadherin alpha 12	0.68	0.68	1.1	0.56
SMAD6	SMAD6;HsT17432;MADH6;MADH7	SMAD family member 6	0.67	0.72	1.1	0.56
LSS	LSS;FLJ25486;FLJ35015;FLJ39450;FLJ46393;OSC	lanosterol synthase (2,3-oxidosqualene-lanosterol cyclase)	0.67	0.74	1.1	0.58
PEX5L	PEX5L;PEX5R;PXR2;PXR2B	peroxisomal biogenesis factor 5-like	0.66	0.68	1.1	0.58
PDGFRA	PDGFRA;CD140A;MGC74795;PDGFR2;RHEPDGFRA	platelet-derived growth factor receptor, alpha polypeptide	0.66	0.78	1.1	0.56
C22orf23	C22orf23;EVG1;FLJ32787;dJ1039K5.6	chromosome 22 open reading frame 23	0.66	0.75	1.1	0.61
NFATC2	NFATC2;NFAT1;NFATP	nuclear factor of activated T-cells, cytoplasmic, calcineurin-dependent 2	0.66	0.7	1.1	0.55
F12	F12;HAE3;HAEX;HAF	coagulation factor XII (Hageman factor)	0.66	0.68	1.1	0.5
CRYBB1	CRYBB1;CATCN3	crystallin, beta B1	0.66	0.71	1.1	0.59
VAMP1	VAMP1;DKFZp686H12131;SYB1;VAMP-1	vesicle-associated membrane protein 1 (synaptobrevin 1)	0.66	0.75	1.1	0.61
ZNF670	ZNF670;FLJ12606;MGC12466	zinc finger protein 670	0.66	0.73	1.1	0.53
H1FNT	H1FNT;H1T2	H1 histone family, member N, testis-specific	0.66	0.74	1.1	0.56
SMARCAD1	SMARCAD1;ADERM;DKFZp762K2015;ETL1;HEL1;KIAA1122	SWI/SNF-related, matrix-associated actin-dependent regulator of chromatin, subfamily a, containing DEAD/H box 1	0.66	0.73	1.1	0.49
PPAP2C	PPAP2C;LPP2;PAP-2c;PAP2-g	phosphatidic acid phosphatase type 2C	0.66	0.68	1.1	0.51
AVPI1	AVPI1;PP5395;RP11-548K23.7;VIP32;VIT32	arginine vasopressin-induced 1	0.66	0.72	1.1	0.51
ACTR1A	ACTR1A;ARP1;CTRN1;FLJ52695;FLJ52800;FLJ55002	ARP1 actin-related protein 1 homolog A, centractin alpha (yeast)	0.66	0.7	1.1	0.53
POLD2	POLD2	polymerase (DNA directed), delta 2, regulatory subunit 50kDa	0.66	0.67	1.1	0.54
LGALSL	LGALSL;GRP;MGC33751;MGC71953	lectin, galactoside-binding-like	0.66	0.74	1.1	0.53
TDG	TDG	thymine-DNA glycosylase	0.65	0.7	1.1	0.54
BBS1	BBS1;BBS2L2;FLJ23590;MGC126183;MGC126184;MGC51114	Bardet-Biedl syndrome 1	0.65	0.74	1.1	0.6
MFSD2B	MFSD2B	major facilitator superfamily domain containing 2B	0.65	0.74	1.1	0.51
UNC5CL	UNC5CL;MGC34763;ZUD	unc-5 homolog C (C. elegans)-like	0.64	0.73	1.1	0.55
DISC1	DISC1;C1orf136;FLJ13381;FLJ21640;FLJ25311;FLJ41105;KIAA0457;SCZD9	disrupted in schizophrenia 1	0.64	0.74	1.1	0.5
TNFRSF10D	TNFRSF10D;CD264;DCR2;TRAIL-R4;TRAILR4;TRUNDD	tumor necrosis factor receptor superfamily, member 10d, decoy with truncated death domain	0.64	0.65	1.1	0.57
GPR155	GPR155;DEP.7;DEPDC3;FLJ31819;FLJ39346;PGR22	G protein-coupled receptor 155	0.64	0.69	1.1	0.62
CDKN1C	CDKN1C;BWCR;BWS;KIP2;WBS;p57	cyclin-dependent kinase inhibitor 1C (p57, Kip2)	0.64	0.76	1.1	0.53
POLE4	POLE4;YHHQ1;p12	polymerase (DNA-directed), epsilon 4 (p12 subunit)	0.64	0.65	1.1	0.57
FAM127C	FAM127C;CXX1c;FLJ25577;MAR8B	family with sequence similarity 127, member C	0.64	0.7	1.1	0.51
CDHR5	CDHR5;FLJ20219;MU-PCDH;MUCDHL;MUPCDH	cadherin-related family member 5	0.64	0.68	1.1	0.52
GPX5	GPX5	glutathione peroxidase 5 (epididymal androgen-related protein)	0.63	0.66	1.1	0.52
PCDHB13	PCDHB13;PCDH-BETA13	protocadherin beta 13	0.63	0.72	1.1	0.51
MADD	MADD;DENN;FLJ35600;FLJ36300;IG20;KIAA0358;RAB3GEP	MAP-kinase activating death domain	0.63	0.67	1.1	0.5
CYFIP2	CYFIP2;PIR121	cytoplasmic FMR1 interacting protein 2	0.63	0.72	1.1	0.58
UGCG	UGCG;GCS;GLCT1	UDP-glucose ceramide glucosyltransferase	0.63	0.67	1.1	0.58
IGFN1	IGFN1;DKFZp434B1231;EEF1A2BP1	immunoglobulin-like and fibronectin type III domain containing 1	0.62	0.66	1.1	0.5
GTF2IRD2B	GTF2IRD2B	GTF2I repeat domain containing 2B	0.62	0.7	1.1	0.47
TMEM180	TMEM180;C10orf77;FLJ22529;bA18I14.8	transmembrane protein 180	0.62	0.7	1.1	0.52
RAB36	RAB36	RAB36, member RAS oncogene family	0.62	0.69	1.1	0.54
SCARA3	SCARA3;APC7;CSR;CSR1;MSLR1;MSRL1	scavenger receptor class A, member 3	0.62	0.74	1.1	0.56
BCL7B	BCL7B	B-cell CLL/lymphoma 7B	0.62	0.62	1.1	0.49
MLF2	MLF2;NTN4	myeloid leukemia factor 2	0.62	0.61	1.1	0.51
GPR42	GPR42;FFAR1L;FFAR3L;GPR41L;GPR42P	G protein-coupled receptor 42 (gene/pseudogene)	0.62	0.64	1.1	0.48
STRAP	STRAP;MAWD;PT-WD;UNRIP	serine/threonine kinase receptor associated protein	0.61	0.64	1.1	0.47
PGM1	PGM1;GSD14	phosphoglucomutase 1	0.61	0.63	1.1	0.5
NME9	NME9;MGC129586;TXL-2;TXL2;TXNDC6	NME gene family member 9	0.61	0.64	1.1	0.47
CCL16	CCL16;CKb12;HCC-4;ILINCK;LCC-1;LEC;LMC;MGC117051;Mtn-1;NCC-4;NCC4;SCYA16;SCYL4	chemokine (C-C motif) ligand 16	0.84	0.91	1.11	0.69
PLSCR4	PLSCR4;TRA1	phospholipid scramblase 4	0.84	0.91	1.11	0.67
GPR1	GPR1	G protein-coupled receptor 1	0.84	0.89	1.11	0.81
STAG3	STAG3	stromal antigen 3	0.84	0.94	1.11	0.65
APOC2	APOC2;APO-CII;APOC-II;MGC75082	apolipoprotein C-II	0.84	0.9	1.11	0.7
OXNAD1	OXNAD1;MGC15763	oxidoreductase NAD-binding domain containing 1	0.84	0.89	1.11	0.7
MYH10	MYH10;MGC134913;MGC134914;NMMHCB	myosin, heavy chain 10, non-muscle	0.84	0.96	1.11	0.73
SAMD15	SAMD15;C14orf174;FAM15A;FLJ35963;MGC132494;MGC132496	sterile alpha motif domain containing 15	0.84	0.9	1.11	0.65
ABHD16A	ABHD16A;BAT5;D6S82E;NG26;PP199	abhydrolase domain containing 16A	0.84	0.94	1.11	0.64
TFEB	TFEB;ALPHATFEB;BHLHE35;TCFEB	transcription factor EB	0.83	0.88	1.11	0.64
HDAC5	HDAC5;FLJ90614;HD5;NY-CO-9	histone deacetylase 5	0.83	0.92	1.11	0.72
AWAT1	AWAT1;DGA2;DGAT2L3	acyl-CoA wax alcohol acyltransferase 1	0.83	0.88	1.11	0.67
PRAMEF10	PRAMEF10;MGC138413;MGC138415;RP5-845O24.7	PRAME family member 10	0.82	0.88	1.11	0.67

AGBL4	AGBL4;CCP6;FLJ14442	ATP/GTP binding protein-like 4	0.82	0.81	1.11	0.63
OR5C1	OR5C1;OR5C2P;OR9-31;OR9-F	olfactory receptor, family 5, subfamily C, member 1	0.82	0.89	1.11	0.72
C17orf74	C17orf74	chromosome 17 open reading frame 74	0.82	1	1.11	0.66
NLRC5	NLRC5;CLR16.1;FLJ21709;FLJ39711;NOD27;NOD4	NLR family, CARD domain containing 5	0.82	0.86	1.11	0.62
POP5	POP5;HSPC004;RPP2;RPP20;hPop5	processing of precursor 5, ribonuclease P/MRP subunit (S. cerevisiae)	0.82	0.96	1.11	0.68
SDSL	SDSL;SDH 2;SDS-RS1;TDH	serine dehydratase-like	0.82	0.84	1.11	0.75
CABLES2	CABLES2;C20orf150;dJ908M14.2;ik3-2	Cdk5 and Abl enzyme substrate 2	0.82	0.95	1.11	0.72
TOR3A	TOR3A;ADIR;ADIR2;FLJ22345;MGC111104	torsin family 3, member A	0.82	0.96	1.11	0.63
RAD1	RAD1;HRAD1;REC1	RAD1 homolog (S. pombe)	0.82	0.88	1.11	0.62
CUX1	CUX1;CASP;CDP;CDP/Cut;CDP1;COY1;CUTL1;CUX;Clox;Cux/CDP;FLJ31745;GOLIM6;Nbla10317;p100;p110;p200;p75	cut-like homeobox 1	0.82	0.84	1.11	0.75
PEX19	PEX19;D1S2223E;FLJ55296;HK33;PMP1;PMPI;PXF;PXMP1	peroxisomal biogenesis factor 19	0.82	0.87	1.11	0.67
HNRNPUL2	HNRNPUL2;DKFZp762N1910;HNRPUL2;SAF-A2	heterogeneous nuclear ribonucleoprotein U-like 2	0.82	0.94	1.11	0.63
FAM45B	FAM45B;HT011	family with sequence similarity 45, member B (pseudogene)	0.82	0.8	1.11	0.63
EXOC3L1	EXOC3L1;EXOC3L;FLJ35539;FLJ35587;MGC88052	exocyst complex component 3-like 1	0.82	1	1.11	0.66
ARSF	ARSF;ASF	arylsulfatase F	0.81	0.94	1.11	0.73
DEFB115	DEFB115;DEFB-15	defensin, beta 115	0.81	0.88	1.11	0.66
MYH11	MYH11;AAT4;DKFZp686D10126;DKFZp686D19237;FAA4;FLJ35232;MGC126726;MGC32963;SMHC;SMMHC	myosin, heavy chain 11, smooth muscle	0.81	0.94	1.11	0.7
OLFML3	OLFML3;HNOEL-iso;OLF44	olfactomedin-like 3	0.81	0.88	1.11	0.74
SNX8	SNX8;DKFZp761E1721;Mvp1	sorting nexin 8	0.81	0.94	1.11	0.62
ARSB	ARSB;ASB;G4S;MPS6	arylsulfatase B	0.81	0.86	1.11	0.62
HIST2H2BF	HIST2H2BF;FLJ35099;FLJ56780;FLJ56787;MGC131639	histone cluster 2, H2bf	0.81	0.86	1.11	0.69
HAUS1	HAUS1;CCDC5;FLJ21094;FLJ40084;HEI-C;HEIC;HsT1461	HAUS augmin-like complex, subunit 1	0.81	0.91	1.11	0.62
LOC389834	LOC389834	ankyrin repeat domain 57 pseudogene	0.8	0.87	1.11	0.73
QSOX2	QSOX2;DKFZp762A2013;QSCN6L1;SOXN	quiescin Q6 sulfhydryl oxidase 2	0.8	0.89	1.11	0.69
RHOBTB1	RHOBTB1;KIAA0740;MGC33059;MGC33841	Rho-related BTB domain containing 1	0.8	0.85	1.11	0.61
HERC1	HERC1;p532;p619	hect (homologous to the E6-AP (UBE3A) carboxyl terminus) domain and RCC1 (CHC1)-like domain (RLD) 1	0.8	0.82	1.11	0.62
C16orf79	C16orf79;MGC21830	chromosome 16 open reading frame 79	0.8	0.86	1.11	0.65
RUFY2	RUFY2;FLJ10063;KIAA1537;RABIP4R;ZFYVE13	RUN and FYVE domain containing 2	0.8	0.86	1.11	0.72
CYB5D1	CYB5D1;FLJ32499	cytochrome b5 domain containing 1	0.8	0.96	1.11	0.73
IMMP2L	IMMP2L;IMP2;IMP2-LIKE	IMP2 inner mitochondrial membrane peptidase-like (S. cerevisiae)	0.8	0.88	1.11	0.66
KIAA0391	KIAA0391;MRPP3	KIAA0391	0.8	0.84	1.11	0.71
PPP1R13B	PPP1R13B;ASPP1;KIAA0771;p53BP2-like;p85	protein phosphatase 1, regulatory subunit 13B	0.8	0.83	1.11	0.63
USP24	USP24;FLJ31309;KIAA1057	ubiquitin specific peptidase 24	0.8	0.87	1.11	0.65
PFN1	PFN1	profilin 1	0.8	0.92	1.11	0.69
XBP1	XBP1;TREB5;XBP2	X-box binding protein 1	0.8	0.89	1.11	0.6
LOC196346	LOC196346	ENTREZ STATUS: REMAPPED TO GENE ID: 646759	0.8	0.88	1.11	0.78
ZNF321P	ZNF321P;DKFZp781C2298;FLJ39509;MGC35402;ZNF321	zinc finger protein 321, pseudogene	0.8	0.86	1.11	0.64
ZNF579	ZNF579;FLJ35453	zinc finger protein 579	0.79	0.89	1.11	0.68
PFKFB2	PFKFB2;DKFZp781D2217;MGC138308;MGC138310;PFK-2/FBPase-2	6-phosphofructo-2-kinase/fructose-2,6-biphosphatase 2	0.79	0.93	1.11	0.67
ARID4B	ARID4B;BCAA;BRCAA1;DKFZp313M2420;MGC163290;RBBP1L1;RBP1L1;SAP180	AT rich interactive domain 4B (RBP1-like)	0.79	0.84	1.11	0.64
DNASE2	DNASE2;DNASE2A;DNL;DNL2	deoxyribonuclease II, lysosomal	0.79	0.93	1.11	0.62
DEFA1	DEFA1;DEF1;DEFA1B;DEFA2;HNP-1;HP-1;HP1;MGC138393;MRS	defensin, alpha 1	0.78	0.86	1.11	0.6
IL21R	IL21R;CD360;MGC10967;NILR	interleukin 21 receptor	0.78	0.84	1.11	0.6
CD302	CD302;BIMLEC;CLEC13A;DCL-1;DCL1;FLJ43091;KIAA0022;MGC22301	CD302 molecule	0.78	0.88	1.11	0.59
ALKBH1	ALKBH1;ABH;ABH1;ALKBH;alkB;hABH	alkB, alkylation repair homolog 1 (E. coli)	0.78	0.86	1.11	0.58
TRAF3IP2	TRAF3IP2;ACT1;C6orf2;C6orf4;C6orf5;C6orf6;CIKS;DKFZp586G0522;MGC3581;PSORS13	TRAF3 interacting protein 2	0.78	0.94	1.11	0.63
NT5DC1	NT5DC1;C6orf200;LP2642;MGC131837;MGC24302;NT5C2L1	5'-nucleotidase domain containing 1	0.78	0.94	1.11	0.63
RBFOX1	RBFOX1;2BP1;A2BP1;FOX-1;FOX1;HRNBP1	RNA binding protein, fox-1 homolog (C. elegans) 1	0.78	0.86	1.11	0.71
ABHD15	ABHD15	abhydrolase domain containing 15	0.78	0.85	1.11	0.64
FILIP1	FILIP1;FILIP;KIAA1275	filamin A interacting protein 1	0.77	0.88	1.11	0.66
FUT7	FUT7;FucT-VII	fucosyltransferase 7 (alpha (1,3) fucosyltransferase)	0.77	0.84	1.11	0.62
PRG3	PRG3;MBP2;MBPH;MGC126662;MGC141971	proteoglycan 3	0.77	0.83	1.11	0.67
PEX5	PEX5;FLJ50634;FLJ50721;FLJ51948;PTS1-BP;PTS1R;PXR1	peroxisomal biogenesis factor 5	0.77	0.84	1.11	0.65
STAT5B	STAT5B;STAT5	signal transducer and activator of transcription 5B	0.77	0.84	1.11	0.63
MAK16	MAK16;MAK16L;RBM13	MAK16 homolog (S. cerevisiae)	0.77	0.77	1.11	0.6
ACSL1	ACSL1;ACS1;FACL1;FACL2;LACS;LACS1;LACS2	acyl-CoA synthetase long-chain family member 1	0.77	0.84	1.11	0.59
DUOX2	DUOX2;LNOX2;NOXEF2;P138-TOX;TDH6;THOX2	dual oxidase 2	0.76	0.9	1.11	0.59
SLITRK4	SLITRK4;DKFZp547M2010	SLIT and NTRK-like family, member 4	0.76	0.76	1.11	0.63
FGF1	FGF1;AFGF;ECGF;ECGF-beta;ECGFA;ECGFB;FGF-alpha;FGFA;GLIO703;HBGF1	fibroblast growth factor 1 (acidic)	0.76	0.86	1.11	0.68
PRH1	PRH1;MGC74956;PA	proline-rich protein HaeIII subfamily 1	0.76	0.81	1.11	0.62
NTSR1	NTSR1;NTR	neurotensin receptor 1 (high affinity)	0.76	0.78	1.11	0.58
RADIL	RADIL;FLJ10324;FLJ60975;KIAA1849;MGC161589;RASIP2	Ras association and DIL domains	0.76	0.88	1.11	0.67
FBN3	FBN3;KIAA1776	fibrillin 3	0.76	0.81	1.11	0.62
KIAA1609	KIAA1609;MGC25024	KIAA1609	0.76	0.82	1.11	0.69
ATAD3C	ATAD3C;FLJ34599;FLJ37183	ATPase family, AAA domain containing 3C	0.76	0.8	1.11	0.64
RHBDF1	RHBDF1;C16orf8;Dist1;EGFR-RS;FLJ2235;FLJ22357;gene-89;gene-90;hDist1	rhomboid 5 homolog 1 (Drosophila)	0.76	0.87	1.11	0.66
SPTBN2	SPTBN2;GTRAP41;SCA5	spectrin, beta, non-erythrocytic 2	0.76	0.87	1.11	0.66
ATPAF1	ATPAF1;ATP11;ATP11p;FLJ22351;FLJ55378;FLJ60653;MGC88060	ATP synthase mitochondrial F1 complex assembly factor 1	0.76	0.85	1.11	0.61
VAPA	VAPA;MGC3745;VAP-33;VAP-A;VAP33;hVAP-33	VAMP (vesicle-associated membrane protein)-associated protein A, 33kDa	0.76	0.81	1.11	0.59
ELMO2	ELMO2;CED-12;CED12;ELMO-2;FLJ11656;KIAA1834	engulfment and cell motility 2	0.76	0.87	1.11	0.65
KDELR3	KDELR3;ERD2L3	KDEL (Lys-Asp-Glu-Leu) endoplasmic reticulum protein retention receptor 3	0.76	0.81	1.11	0.58
AK3	AK3;AK3L1;AK6;AKL3L;AKL3L1;FIX	adenylate kinase 3	0.76	0.82	1.11	0.74
ZZZ3	ZZZ3;ATAC1;DKFZp313N0119;DKFZp564I052;FLJ10362	zinc finger, ZZ-type containing 3	0.76	0.76	1.11	0.63
SRI	SRI;CP22;FLJ26259;SCN	sorcin	0.76	0.88	1.11	0.59
RNLS	RNLS;C10orf59;FLJ11218;RENALASE	renalase, FAD-dependent amine oxidase	0.76	0.88	1.11	0.59
DRD1	DRD1;DADR;DRD1A	dopamine receptor D1	0.75	0.81	1.11	0.73
DACH2	DACH2;FLJ31391;MGC138545	dachshund homolog 2 (Drosophila)	0.75	0.82	1.11	0.62
MPO	MPO	myeloperoxidase	0.75	0.78	1.11	0.62
SLC11A1	SLC11A1;LSH;NRAMP;NRAMP1	solute carrier family 11 (proton-coupled divalent metal ion transporters), member 1	0.75	0.82	1.11	0.63
FAHD1	FAHD1;C16orf36;DKFZp566J2046;MGC74876;YISKL	fumarylacetoacetate hydrolase domain containing 1	0.75	0.81	1.11	0.6
NFXL1	NFXL1;FLJ16294;HOZFP;URCC5	nuclear transcription factor, X-box binding-like 1	0.75	0.84	1.11	0.6
KPNA4	KPNA4;FLJ31113;IPOA3;MGC12217;MGC26703;QIP1;SRP3	karyopherin alpha 4 (importin alpha 3)	0.75	0.8	1.11	0.62
FAM81B	FAM81B;FLJ25333	family with sequence similarity 81, member B	0.74	0.78	1.11	0.64
GCKR	GCKR;FGQTL5;GKRP	glucokinase (hexokinase 4) regulator	0.74	0.81	1.11	0.63
OR7C2	OR7C2;CIT-HSP-87M17;OR19-18;OR7C3	olfactory receptor, family 7, subfamily C, member 2	0.74	0.8	1.11	0.62
PNPLA4	PNPLA4;DXS1283E;GS2	patatin-like phospholipase domain containing 4	0.74	0.78	1.11	0.61
CRKL	CRKL	v-crk sarcoma virus CT10 oncogene homolog (avian)-like	0.74	0.88	1.11	0.63
GPNMB	GPNMB;HGFIN;NMB	glycoprotein (transmembrane) nmb	0.74	0.84	1.11	0.64
CMPK1	CMPK1;CMK;CMPK;UMK;UMP-CMPK;UMPK	cytidine monophosphate (UMP-CMP) kinase 1, cytosolic	0.74	0.88	1.11	0.63
MARCKSL1	MARCKSL1;F52;MACMARCKS;MLP;MLP1;MRP	MARCKS-like 1	0.74	0.73	1.11	0.57
EIF4B	EIF4B;EIF-4B;PRO1843	eukaryotic translation initiation factor 4B	0.74	0.82	1.11	0.64
ALDOB	ALDOB;ALDB;ALDO2	aldolase B, fructose-bisphosphate	0.73	0.8	1.11	0.56
BCL6B	BCL6B;BAZF;ZBTB28;ZNF62	B-cell CLL/lymphoma 6, member B	0.73	0.76	1.11	0.55
ZNF143	ZNF143;SBF;STAF;pHZ-1	zinc finger protein 143	0.73	0.79	1.11	0.61
TTF1	TTF1;TTF-1;TTF-I	transcription termination factor, RNA polymerase I	0.73	0.78	1.11	0.56
MRPS9	MRPS9;MRP-S9;RPMS9;S9mt	mitochondrial ribosomal protein S9	0.73	0.79	1.11	0.57
DHCR7	DHCR7;SLOS	7-dehydrocholesterol reductase	0.73	0.74	1.11	0.66
PTBP1	PTBP1;HNRNP-I;HNRNPI;HNRPI;MGC10830;MGC8461;PTB;PTB-1;PTB-T;PTB2;PTB3;PTB4;pPTB	polypyrimidine tract binding protein 1	0.73	0.76	1.11	0.6
NKAPL	NKAPL;C6orf194;MGC126730;MGC126734;bA424I5.1	NFKB activating protein-like	0.72	0.81	1.11	0.58
CCR8	CCR8;CC-CKR-8;CCR-8;CDw198;CKRL1;CMKBR8;CMKBRL2;CY6;GPRCY6;MGC129966;MGC129973;TER1	chemokine (C-C motif) receptor 8	0.72	0.76	1.11	0.69
ATP1A4	ATP1A4;ATP1A1;ATP1AL2;MGC25056	ATPase, Na+/K+ transporting, alpha 4 polypeptide	0.72	0.81	1.11	0.54
CXXC4	CXXC4;IDAX;MGC149872	CXXC finger protein 4	0.72	0.76	1.11	0.58
MYCN	MYCN;MODED;N-myc;NMYC;ODED;bHLHe37	v-myc myelocytomatosis viral related oncogene, neuroblastoma derived (avian)	0.72	0.8	1.11	0.54
GRHL3	GRHL3;MGC46624;SOM;TFCP2L4	grainyhead-like 3 (Drosophila)	0.72	0.87	1.11	0.58
PGBD2	PGBD2	piggyBac transposable element derived 2	0.72	0.85	1.11	0.57
SASS6	SASS6;FLJ22097;MGC119440;SAS-6;SAS6	spindle assembly 6 homolog (C. elegans)	0.72	0.82	1.11	0.59
CES1	CES1;ACAT;CEH;CES2;HMSE;HMSE1;MGC117365;PCE-1;REH;SES1;TGH	carboxylesterase 1	0.72	0.75	1.11	0.59
PTPN23	PTPN23;DKFZp564F0923;HD-PTP;HDPTP;KIAA1471;PTP-TD14	protein tyrosine phosphatase, non-receptor type 23	0.72	0.74	1.11	0.56
CDH2	CDH2;CD325;CDHN;CDw325;NCAD	cadherin 2, type 1, N-cadherin (neuronal)	0.72	0.82	1.11	0.62
SRP19	SRP19	signal recognition particle 19kDa	0.72	0.82	1.11	0.62
DERA	DERA;DEOC	deoxyribose-phosphate aldolase (putative)	0.72	0.77	1.11	0.67
DHDDS	DHDDS;CIT;CPT;DS;FLJ13102;HDS;RP59	dehydrodolichyl diphosphate synthase	0.72	0.76	1.11	0.56
UROS	UROS;UROIIIS	uroporphyrinogen III synthase	0.72	0.85	1.11	0.56
HLA-DRB1	HLA-DRB1;DRB1;DRw10;FLJ75017;FLJ76359;HLA-DR1B;HLA-DRB;SS1	major histocompatibility complex, class II, DR beta 1	0.72	0.8	1.11	0.59
DNAJA4	DNAJA4;MST104;MSTP104;PRO1472	DnaJ (Hsp40) homolog, subfamily A, member 4	0.71	0.87	1.11	0.6
TXLNA	TXLNA;DKFZp451J0118;IL14;MGC118870;MGC118871;RP4-622L5.4;TXLN	taxilin alpha	0.71	0.81	1.11	0.58
LINC00207	LINC00207;NCRNA00207	long intergenic non-protein coding RNA 207	0.71	0.7	1.11	0.55
PLEKHG4	PLEKHG4;ARHGEF44;DKFZp434I216;PRTPHN1;SCA4	pleckstrin homology domain containing, family G (with RhoGef domain) member 4	0.7	0.76	1.11	0.63
SLC44A4	SLC44A4;C6orf29;CTL4;FLJ14491;NG22	solute carrier family 44, member 4	0.7	0.74	1.11	0.57
NEURL	NEURL;NEURL1;RNF67;bA416N2.1;neu;neu-1	neuralized homolog (Drosophila)	0.7	0.76	1.11	0.68
BTN3A1	BTN3A1;BT3.1;BTF5;CD277;MGC141880	butyrophilin, subfamily 3, member A1	0.7	0.74	1.11	0.57
MTCP1	MTCP1;P13MTCP1;p8MTCP1	mature T-cell proliferation 1	0.7	0.74	1.11	0.57
ELP2	ELP2;FLJ10879;SHINC-2;STATIP1;StIP	elongation protein 2 homolog (S. cerevisiae)	0.7	0.76	1.11	0.56
SNX19	SNX19;CHET8;DKFZp667I205;KIAA0254	sorting nexin 19	0.7	0.79	1.11	0.57
MED18	MED18;FLJ20045;p28b	mediator complex subunit 18	0.69	0.78	1.11	0.59
FLG	FLG;ATOD2	filaggrin	0.68	0.72	1.11	0.52
FOXH1	FOXH1;FAST-1;FAST1	forkhead box H1	0.68	0.75	1.11	0.58
DHDH	DHDH;2DD;HUM2DD	dihydrodiol dehydrogenase (dimeric)	0.68	0.76	1.11	0.59
ZNF57	ZNF57;ZNF424	zinc finger protein 57	0.68	0.67	1.11	0.53

LYRM4	LYRM4;C6orf149;CGI-203;ISD11	LYR motif containing 4	0.68	0.77	1.11	0.58
COMMD1	COMMD1;C2orf5;MGC27155;MURR1	copper metabolism (Murr1) domain containing 1	0.68	0.79	1.11	0.56
MOSPD1	MOSPD1;DJ473B4	motile sperm domain containing 1	0.68	0.78	1.11	0.53
TICAM2	TICAM2;MGC129876;MGC129877;TICAM-2;TIRAP3;TIRP;TRAM	toll-like receptor adaptor molecule 2	0.68	0.76	1.11	0.55
FAM57A	FAM57A;CT120;FLJ22282	family with sequence similarity 57, member A	0.68	0.78	1.11	0.52
C5orf51	C5orf51	chromosome 5 open reading frame 51	0.68	0.73	1.11	0.58
SELRC1	SELRC1;C1orf163;FLJ12439	Sel1 repeat containing 1	0.68	0.73	1.11	0.56
OR14I1	OR14I1;OR5BU1;OR5BU1P	olfactory receptor, family 14, subfamily I, member 1	0.66	0.73	1.11	0.51
CNTN2	CNTN2;AXT;DKFZp781D102;FLJ37193;FLJ42746;MGC157722;TAG-1;TAX;TAX1	contactin 2 (axonal)	0.66	0.77	1.11	0.51
ROM1	ROM1;ROM;ROSP1;RP7;TSPAN23	retinal outer segment membrane protein 1	0.66	0.74	1.11	0.57
HSPB6	HSPB6;FLJ32389;Hsp20	heat shock protein, alpha-crystallin-related, B6	0.66	0.72	1.11	0.54
VSIG2	VSIG2;2210413P10Rik;CTH;CTXL	V-set and immunoglobulin domain containing 2	0.66	0.7	1.11	0.53
CTRB1	CTRB1;CTRB;FLJ42412;MGC88037	chymotrypsinogen B1	0.66	0.66	1.11	0.5
FAM158A	FAM158A;C14orf122	family with sequence similarity 158, member A	0.66	0.75	1.11	0.59
ARIH2	ARIH2;ARI2;FLJ10938;FLJ33921;TRIAD1	ariadne homolog 2 (Drosophila)	0.66	0.72	1.11	0.64
MRPS35	MRPS35;DKFZp762P093;MDS023;MGC104278;MRP-S28;MRPS28	mitochondrial ribosomal protein S35	0.66	0.76	1.11	0.54
SERINC2	SERINC2;MGC90340;PRO0899;TDE2;TDE2L	serine incorporator 2	0.66	0.77	1.11	0.55
UGT1A6	UGT1A6;GNT1;HLUGP;HLUGP1;MGC29860;UDPGT;UDPGT 1-6;UGT1;UGT1A6S;UGT1F	UDP glucuronosyltransferase 1 family, polypeptide A6	0.66	0.7	1.11	0.61
TP53I11	TP53I11;PIG11	tumor protein p53 inducible protein 11	0.65	0.74	1.11	0.56
ZSCAN21	ZSCAN21;DKFZp434L134;DKFZp686H10254;NY-REN-21;ZNF38;Zipro1	zinc finger and SCAN domain containing 21	0.65	0.7	1.11	0.53
ZDHHC24	ZDHHC24	zinc finger, DHHC-type containing 24	0.65	0.7	1.11	0.53
SRCAP	SRCAP;DOMO1;EAF1;FLJ44499;KIAA0309;SWR1	Snf2-related CREBBP activator protein	0.65	0.71	1.11	0.5
ARHGAP44	ARHGAP44;KIAA0672;NPC-A-10;RICH2	Rho GTPase activating protein 44	0.65	0.75	1.11	0.56
SLC35G3	SLC35G3;AMAC1;FLJ40154;TMEM21A	solute carrier family 35, member G3	0.65	0.71	1.11	0.53
AKR1C1	AKR1C1;2-ALPHA-HSD;20-ALPHA-HSD;C9;DD1;DD1/DD2;DDH;DDH1;H-37;HAKRC;HBAB;MBAB;MGC8954	aldo-keto reductase family 1, member C1 (dihydrodiol dehydrogenase 1; 20-alpha (3-alpha)-hydroxysteroid dehydrogenase)	0.64	0.7	1.11	0.54
MYH7B	MYH7B;MGC96928;MGC96940;MHC14;MYH14	myosin, heavy chain 7B, cardiac muscle, beta	0.64	0.78	1.11	0.58
GRB14	GRB14	growth factor receptor-bound protein 14	0.64	0.68	1.11	0.52
RASSF8	RASSF8;C12orf2;DKFZp434O0227;FLJ11542;HOJ1	Ras association (RalGDS/AF-6) domain family (N-terminal) member 8	0.64	0.7	1.11	0.51
PLEKHH3	PLEKHH3;FLJ21019	pleckstrin homology domain containing, family H (with MyTH4 domain) member 3	0.64	0.69	1.11	0.53
PLCG2	PLCG2	phospholipase C, gamma 2 (phosphatidylinositol-specific)	0.64	0.66	1.11	0.52
EPS8L2	EPS8L2;EPS8R2;FLJ16738;FLJ21935;FLJ22171;MGC126530;MGC3088	EPS8-like 2	0.64	0.74	1.11	0.51
ARAF	ARAF;A-RAF;ARAF1;PKS2;RAFA1	v-raf murine sarcoma 3611 viral oncogene homolog	0.64	0.76	1.11	0.54
PEPD	PEPD;MGC10905;PROLIDASE	peptidase D	0.64	0.71	1.11	0.53
CAPN2	CAPN2;CANP2;CANPL2;CANPml;FLJ39928;mCANP	calpain 2, (m/II) large subunit	0.64	0.7	1.11	0.52
CDK14	CDK14;KIAA0834;PFTAIRE1;PFTK1	cyclin-dependent kinase 14	0.64	0.71	1.11	0.59
PADI4	PADI4;PAD;PAD4;PADI5;PDI4;PDI5	peptidyl arginine deiminase, type IV	0.63	0.66	1.11	0.52
AMD1	AMD1;ADOMETDC;AMD;DKFZp313L1234;FLJ26964;SAMDC	adenosylmethionine decarboxylase 1	0.63	0.72	1.11	0.57
RASSF6	RASSF6;DKFZp686K23225	Ras association (RalGDS/AF-6) domain family member 6	0.62	0.7	1.11	0.48
GDPD3	GDPD3;FLJ22603;MGC4171	glycerophosphodiester phosphodiesterase domain containing 3	0.62	0.68	1.11	0.58
FAM5B	FAM5B;BRINP2;DBCCR1L2;FLJ41342;KIAA1747;RP5-1026E2.1	family with sequence similarity 5, member B	0.62	0.61	1.11	0.48
COLEC12	COLEC12;CLP1;NSR2;SCARA4;SRCL	collectin sub-family member 12	0.62	0.71	1.11	0.54
C11orf31	C11orf31;C17orf10;SELH	chromosome 11 open reading frame 31	0.62	0.71	1.11	0.54
GEMIN4	GEMIN4;DKFZp434B131;DKFZp434D174;HC56;HCAP1;HHRF-1;p97	gem (nuclear organelle) associated protein 4	0.62	0.66	1.11	0.55
HM13	HM13;H13;IMP1;IMPAS;IMPAS-1;MSTP086;PSENL3;PSL3;SPP;dJ324O17.1	histocompatibility (minor) 13	0.62	0.64	1.11	0.48
KCNU1	KCNU1;KCNMC1;KCa5;KCa5.1;Kcnma3;Slo3	potassium channel, subfamily U, member 1	0.62	0.72	1.11	0.56
SRRM3	SRRM3;FLJ35271;FLJ37078	serine/arginine repetitive matrix 3	0.62	0.68	1.11	0.57
ORM2	ORM2;AGP-B;AGP-B';AGP2	orosomucoid 2	0.61	0.74	1.11	0.55
INMT	INMT;MGC125940;MGC125941	indolethylamine N-methyltransferase	0.61	0.72	1.11	0.47
HLA-DOA	HLA-DOA;HLA-DNA;HLA-DZA;HLADZ	major histocompatibility complex, class II, DO alpha	0.61	0.65	1.11	0.47
U2AF1L4	U2AF1L4;FLJ35525;MGC33901;U2AF1-RS3;U2AF1L3;U2AF1L3V1;U2AF1RS3;U2af26	U2 small nuclear RNA auxiliary factor 1-like 4	0.61	0.65	1.11	0.5
PIM1	PIM1;PIM	pim-1 oncogene	0.61	0.68	1.11	0.56
FGFR2	FGFR2;BEK;BFR-1;CD332;CEK3;CFD1;ECT1;FLJ98662;JWS;K-SAM;KGFR;TK14;TK25	fibroblast growth factor receptor 2	0.61	0.73	1.11	0.52
YKT6	YKT6	YKT6 v-SNARE homolog (S. cerevisiae)	0.61	0.7	1.11	0.5
SCGBL	SCGBL	secretoglobin-like	0.61	0.7	1.11	0.47
TNFRSF10C	TNFRSF10C;CD263;DCR1;DCR1-TNFR;LIT;MGC149501;MGC149502;TRAIL-R3;TRAILR3;TRID	tumor necrosis factor receptor superfamily, member 10c, decoy without an intracellular domain	0.84	0.91	1.12	0.71
IL10RA	IL10RA;CD210a;CDW210A;HIL-10R;IL-10R1;IL10R	interleukin 10 receptor, alpha	0.84	1.01	1.12	0.76
C2orf76	C2orf76;AIM29;MGC104437	chromosome 2 open reading frame 76	0.84	0.92	1.12	0.7
ERMAP	ERMAP;MGC118810;MGC118811;MGC118812;MGC118813;PRO2801;RD;SC	erythroblast membrane-associated protein (Scianna blood group)	0.84	0.95	1.12	0.64
GPR144	GPR144;PGR24	G protein-coupled receptor 144	0.84	0.92	1.12	0.83
EZH1	EZH1;KIAA0388;KMT6B	enhancer of zeste homolog 1 (Drosophila)	0.84	0.9	1.12	0.66
NELF	NELF;MGC125369	nasal embryonic LHRH factor	0.84	0.97	1.12	0.74
KIAA1737	KIAA1737	KIAA1737	0.84	0.98	1.12	0.74
WBP5	WBP5;DKFZp313K1940;TCEAL9	WW domain binding protein 5	0.84	0.91	1.12	0.74
NDUFC2	NDUFC2;B14.5b;CI-B14.5b;HLC-1;NADHDH2	NADH dehydrogenase (ubiquinone) 1, subcomplex unknown, 2, 14.5kDa	0.84	0.91	1.12	0.79
ATP5O	ATP5O;ATPO;OSCP	ATP synthase, H+ transporting, mitochondrial F1 complex, O subunit	0.84	0.94	1.12	0.69
HIST2H2AB	HIST2H2AB	histone cluster 2, H2ab	0.84	0.84	1.12	0.7
SNRNP48	SNRNP48;C6orf151;FLJ32234;MGC138904;MGC138905;dJ336K20B.1;dJ512B11.2	small nuclear ribonucleoprotein 48kDa (U11/U12)	0.84	0.84	1.12	0.7
CACNB4	CACNB4;CAB4;CACNLB4;EA5;EIG9;EJM;EJM4;EJM6	calcium channel, voltage-dependent, beta 4 subunit	0.83	0.99	1.12	0.77
RAB44	RAB44;FLJ43093;RASD3;RASL13;dJ431A14.3	RAB44, member RAS oncogene family	0.83	0.98	1.12	0.69
CFP	CFP;BFD;PFC;PFD;PROPERDIN	complement factor properdin	0.83	0.93	1.12	0.67
PODXL2	PODXL2;PODLX2	podocalyxin-like 2	0.83	0.91	1.12	0.69
DEXI	DEXI;MYLE	Dexi homolog (mouse)	0.83	1.02	1.12	0.75
FOLR2	FOLR2;BETA-HFR;FBP/PL-1;FR-BETA;FR-P3	folate receptor 2 (fetal)	0.82	0.88	1.12	0.63
HBD	HBD	hemoglobin, delta	0.82	0.9	1.12	0.77
KLK12	KLK12;DKFZp686H1078;KLK-L5;KLKL5;MGC42603	kallikrein-related peptidase 12	0.82	0.9	1.12	0.68
ARSI	ARSI;FLJ16069	arylsulfatase family, member I	0.82	0.86	1.12	0.69
RALGDS	RALGDS;FLJ20922;RGDS;RGF;RalGEF	ral guanine nucleotide dissociation stimulator	0.82	0.9	1.12	0.71
C1orf172	C1orf172;FLJ34633;RP11-344H11.3	chromosome 1 open reading frame 172	0.82	0.9	1.12	0.67
MMP14	MMP14;1;MMP-14;MMP-X1;MT-MMP;MT-MMP 1;MT1-MMP;MT1MMP;MTMMP1	matrix metallopeptidase 14 (membrane-inserted)	0.82	0.92	1.12	0.69
C20orf94	C20orf94;bA204H22.1;bA254M13.1;dJ1099D15.3	chromosome 20 open reading frame 94	0.82	0.98	1.12	0.69
IL27RA	IL27RA;CRL1;IL27R;TCCR;WSX1;zcytor1	interleukin 27 receptor, alpha	0.82	0.92	1.12	0.72
NUFIP2	NUFIP2;182-FIP;82-FIP;FIP-82;FLJ10976;KIAA1321;MGC117262	nuclear fragile X mental retardation protein interacting protein 2	0.82	0.98	1.12	0.69
MCM4	MCM4;CDC21;CDC54;MGC33310;P1-CDC21;hCdc21	minichromosome maintenance complex component 4	0.82	0.9	1.12	0.66
HIST2H3C	HIST2H3C;H3;H3.2;H3/M;H3F2;H3FM;H3FN;HIST2H3A;HIST2H3D;MGC9629	histone cluster 2, H3c	0.82	0.88	1.12	0.64
DUSP6	DUSP6;MKP3;PYST1	dual specificity phosphatase 6	0.81	0.9	1.12	0.7
SOCS6	SOCS6;CIS-4;CIS4;HSPC060;SOCS-4;SOCS-6;SOCS4;SSI4;STAI4;STATI4	suppressor of cytokine signaling 6	0.81	0.87	1.12	0.63
KIAA1143	KIAA1143	KIAA1143	0.81	0.94	1.12	0.71
LOC653566	LOC653566	signal peptidase complex subunit 2 homolog pseudogene	0.81	0.94	1.12	0.64
SERPINF1	SERPINF1;EPC-1;OI12;PEDF	serpin peptidase inhibitor, clade F (alpha-2 antiplasmin, pigment epithelium derived factor), member 1	0.8	0.88	1.12	0.66
HECW2	HECW2;DKFZp686M17164;NEDL2	HECT, C2 and WW domain containing E3 ubiquitin protein ligase 2	0.8	0.82	1.12	0.62
SNED1	SNED1;DKFZp586B2420;FLJ00133;SST3;Snep	sushi, nidogen and EGF-like domains 1	0.8	0.81	1.12	0.63
DNAJC27	DNAJC27;DKFZp434N211;RBJ;RabJS	DnaJ (Hsp40) homolog, subfamily C, member 27	0.8	0.92	1.12	0.73
HDHD3	HDHD3;2810435D12Rik;C9orf158;MGC12904	haloacid dehalogenase-like hydrolase domain containing 3	0.8	0.83	1.12	0.73
LRRC8C	LRRC8C;AD158;DKFZp586J1119;FAD158;MGC138551	leucine rich repeat containing 8 family, member C	0.8	0.96	1.12	0.67
ERBB3	ERBB3;ErbB-3;HER3;LCCS2;MDA-BF-1;MGC88033;c-erbB-3;c-erbB3;erbB3-S;p180-ErbB3;p45-sErbB3;p85-sErbB3	v-erb-b2 erythroblastic leukemia viral oncogene homolog 3 (avian)	0.8	0.97	1.12	0.69
RIF1	RIF1;DKFZp434D1026;DKFZp781N1478;FLJ12870	RAP1 interacting factor homolog (yeast)	0.8	0.88	1.12	0.73
TTC3	TTC3;DCRR1;DKFZp686M0150;RNF105;TPRDIII	tetratricopeptide repeat domain 3	0.8	0.88	1.12	0.79
C16orf91	C16orf91;CCSMST1;MGC104723;gs103	chromosome 16 open reading frame 91	0.8	0.92	1.12	0.62
P4HTM	P4HTM;EGLN4;HIFPH4;P4H-TM;PH-4;PH4;PHD4	prolyl 4-hydroxylase, transmembrane (endoplasmic reticulum)	0.8	0.88	1.12	0.65
GP2	GP2;DKFZp779K0533;ZAP75	glycoprotein 2 (zymogen granule membrane)	0.79	0.93	1.12	0.75
THAP4	THAP4;PP238	THAP domain containing 4	0.79	0.85	1.12	0.62
ARHGAP12	ARHGAP12;DKFZp779N2050;FLJ10971;FLJ20737;FLJ21785;FLJ45709	Rho GTPase activating protein 12	0.79	0.92	1.12	0.69
HNF4G	HNF4G;NR2A2;NR2A3	hepatocyte nuclear factor 4, gamma	0.78	0.92	1.12	0.61
C9orf66	C9orf66;FLJ31158;RP11-59O6.1	chromosome 9 open reading frame 66	0.78	0.86	1.12	0.65
AKAP6	AKAP6;ADAP100;ADAP6;AKAP100;KIAA0311;MGC165020;PRKA6;mAKAP	A kinase (PRKA) anchor protein 6	0.78	0.84	1.12	0.63
CCL22	CCL22;ABCD-1;DC/B-CK;MDC;MGC34554;SCYA22;STCP-1	chemokine (C-C motif) ligand 22	0.78	0.85	1.12	0.68
GLIS1	GLIS1;FLJ36155	GLIS family zinc finger 1	0.78	0.84	1.12	0.65
PPARA	PPARA;MGC2237;MGC2452;NR1C1;PPAR;PPARalpha;hPPAR	peroxisome proliferator-activated receptor alpha	0.78	0.82	1.12	0.65
STK11IP	STK11IP;KIAA1898;LIP1;LKB1IP;STK11IP1	serine/threonine kinase 11 interacting protein	0.78	0.9	1.12	0.65
TRIB3	TRIB3;C20orf97;NIPK;SINK;SKIP3;TRB3	tribbles homolog 3 (Drosophila)	0.78	0.94	1.12	0.67
POMT2	POMT2;DKFZp686G10254;FLJ22309;MDDGA2;MDDGB2;MDDGC2	protein-O-mannosyltransferase 2	0.78	0.82	1.12	0.65
SLC25A22	SLC25A22;EIEE3;FLJ13044;GC1;NET44	solute carrier family 25 (mitochondrial carrier: glutamate), member 22	0.78	0.97	1.12	0.67
NEK6	NEK6;SID6-1512	NIMA (never in mitosis gene a)-related kinase 6	0.78	0.87	1.12	0.72
PTGR2	PTGR2;DKFZp686P10120;FLJ39091;FLJ99229;PGR2;ZADH1	prostaglandin reductase 2	0.78	0.81	1.12	0.72
FAM120A	FAM120A;C9orf10;DNAPTP1;DNAPTP5;MGC111527;MGC133257;OSSA	family with sequence similarity 120A	0.78	0.91	1.12	0.65
OBFC2B	OBFC2B;MGC2731;SOSS-B1;SSB1;hSSB1	oligonucleotide/oligosaccharide-binding fold containing 2B	0.78	0.77	1.12	0.61
NBPF14	NBPF14;DJ328E19.C1.1;FLJ35032;FLJ60520;NBPF	neuroblastoma breakpoint family, member 14	0.78	0.87	1.12	0.63
GNAL	GNAL	guanine nucleotide binding protein (G protein), alpha activating activity polypeptide, olfactory type	0.77	0.84	1.12	0.59
TTC16	TTC16;FLJ32780	tetratricopeptide repeat domain 16	0.77	0.83	1.12	0.6
GLG1	GLG1;CFR-1;DKFZp686L08213;ESL-1;FLJ23319;FLJ23967;FLJ32797;MG-160;MG160	golgi glycoprotein 1	0.77	0.89	1.12	0.64
CENPI	CENPI;CENP-I;FSHPRH1;LRPR1;Mis6	centromere protein I	0.77	0.89	1.12	0.66
DPP4	DPP4;ADABP;ADCP2;CD26;DPPIV;TP103	dipeptidyl-peptidase 4	0.76	0.84	1.12	0.63
INO80C	INO80C;C18orf37;FLJ38183;IES6;hIes6	INO80 complex subunit C	0.76	0.88	1.12	0.67
CALB2	CALB2;CAB29;CAL2;CR	calbindin 2	0.76	0.83	1.12	0.67
FBXO41	FBXO41;FBX41;FLJ37709	F-box protein 41	0.76	0.82	1.12	0.59
PRR15	PRR15	proline rich 15	0.76	0.86	1.12	0.62
ANKRD50	ANKRD50;KIAA1223	ankyrin repeat domain 50	0.76	0.8	1.12	0.68
TRAF7	TRAF7;DKFZp586I021;MGC7807;RFWD1;RNF119	TNF receptor-associated factor 7	0.76	0.84	1.12	0.76
STARD10	STARD10;MGC14401;NY-CO-28;PCTP2;SDCCAG28	StAR-related lipid transfer (START) domain containing 10	0.76	0.77	1.12	0.61

GTPBP2	GTPBP2;MGC74725	GTP binding protein 2	0.76	0.85	1.12	0.66
BAMBI	BAMBI;NMA	BMP and activin membrane-bound inhibitor homolog (Xenopus laevis)	0.76	0.81	1.12	0.68
ACTL6A	ACTL6A;ACTL6;ARPN-BETA;Arp4;BAF53A;INO80K;MGC5382	actin-like 6A	0.76	0.86	1.12	0.66
NFE4	NFE4;NF-E4	transcription factor NF-E4	0.76	0.88	1.12	0.63
ATP4B	ATP4B;ATP6B	ATPase, H+/K+ exchanging, beta polypeptide	0.75	0.85	1.12	0.57
TBX2	TBX2;FLJ10169	T-box 2	0.75	0.82	1.12	0.64
ENOSF1	ENOSF1;HSRTSBETA;RTS;TYMSAS	enolase superfamily member 1	0.75	0.86	1.12	0.64
LMBRD2	LMBRD2;DKFZp434H2226;DKFZp686G1057;MGC125692;MGC125693	LMBR1 domain containing 2	0.75	0.89	1.12	0.63
MCM7	MCM7;CDC47;MCM2;P1.1-MCM3;P1CDC47;P85MCM;PNAS146	minichromosome maintenance complex component 7	0.75	0.84	1.12	0.57
EIF4EBP3	EIF4EBP3;4E-BP3;4EBP3	eukaryotic translation initiation factor 4E binding protein 3	0.75	0.8	1.12	0.67
ZEB1	ZEB1;AREB6;BZP;DELTAEF1;FECD6;MGC133261;NIL2A;PPCD3;TCF8;ZFHEP;ZFHX1A	zinc finger E-box binding homeobox 1	0.74	0.8	1.12	0.56
RPGR	RPGR;COD1;CORDX1;CRD;PCDX;RP15;RP3;XLRP3;orf15	retinitis pigmentosa GTPase regulator	0.74	0.81	1.12	0.67
CEBPA	CEBPA;C/EBP-alpha;CEBP	CCAAT/enhancer binding protein (C/EBP), alpha	0.74	0.81	1.12	0.57
INHBB	INHBB;MGC157939	inhibin, beta B	0.74	0.77	1.12	0.61
OS9	OS9;ERLEC2;OS-9	osteosarcoma amplified 9, endoplasmic reticulum lectin	0.74	0.8	1.12	0.61
TMEM48	TMEM48;FLJ10407;FLJ12556;FLJ34120;NDC1;NET3	transmembrane protein 48	0.74	0.8	1.12	0.58
TM7SF3	TM7SF3	transmembrane 7 superfamily member 3	0.74	0.85	1.12	0.61
MARK2	MARK2;EMK-1;EMK1;MGC99619;PAR-1;Par-1b;Par1b	MAP/microtubule affinity-regulating kinase 2	0.73	0.82	1.12	0.67
PPP1R35	PPP1R35;C7orf47;MGC22793	protein phosphatase 1, regulatory subunit 35	0.73	0.78	1.12	0.63
OPRK1	OPRK1;KOR;OPRK	opioid receptor, kappa 1	0.72	0.75	1.12	0.57
HES5	HES5;bHLHb38	hairy and enhancer of split 5 (Drosophila)	0.72	0.84	1.12	0.6
UHRF1	UHRF1;FLJ21925;ICBP90;MGC138707;Np95;RNF106;hNP95	ubiquitin-like with PHD and ring finger domains 1	0.72	0.8	1.12	0.71
WT1-AS	WT1-AS;MGC120207;MGC120208;MGC120209;WIT-1;WIT1;WT1-AS1;WT1AS;dJ74J1.1	WT1 antisense RNA (non-protein coding)	0.72	0.86	1.12	0.6
UNC5D	UNC5D;FLJ16019;KIAA1777;PRO34692;Unc5h4	unc-5 homolog D (C. elegans)	0.71	0.81	1.12	0.58
HIC2	HIC2;HRG22;KIAA1020;ZBTB30;ZNF907	hypermethylated in cancer 2	0.71	0.84	1.12	0.68
ZNF74	ZNF74;Cos52;ZNF520;Zfp520	zinc finger protein 74	0.71	0.77	1.12	0.55
TCTN2	TCTN2;C12orf38;FLJ12975;MKS8;TECT2	tectonic family member 2	0.71	0.8	1.12	0.58
NBPF8	NBPF8	ENTREZ STATUS: REMAPPED TO GENE ID: 728841	0.71	0.84	1.12	0.67
B3GALT4	B3GALT4;BETA3GALT4;GALT2;GALT4	UDP-Gal:betaGlcNAc beta 1,3-galactosyltransferase, polypeptide 4	0.7	0.78	1.12	0.58
C20orf141	C20orf141;MGC26144;TMEM239;dJ860F19.4	chromosome 20 open reading frame 141	0.7	0.82	1.12	0.55
VASN	VASN;SLITL2	vasorin	0.7	0.8	1.12	0.6
IL6	IL6;BSF2;HGF;HSF;IFNB2;IL-6	interleukin 6 (interferon, beta 2)	0.7	0.86	1.12	0.61
DYRK2	DYRK2;FLJ21217;FLJ21365	dual-specificity tyrosine-(Y)-phosphorylation regulated kinase 2	0.7	0.78	1.12	0.65
CA12	CA12;CAXII;FLJ20151;HsT18816	carbonic anhydrase XII	0.7	0.78	1.12	0.6
EFR3A	EFR3A;DKFZp781J0562;KIAA0143	EFR3 homolog A (S. cerevisiae)	0.7	0.82	1.12	0.61
NBPF10	NBPF10	ENTREZ STATUS: REMAPPED TO GENE ID: 100132406	0.7	0.87	1.12	0.64
POLR1B	POLR1B;FLJ10816;FLJ21921;MGC131780;RPA135;RPA2;Rpo1-2	polymerase (RNA) I polypeptide B, 128kDa	0.69	0.72	1.12	0.64
SLC6A6	SLC6A6;MGC10619;MGC131729;TAUT	solute carrier family 6 (neurotransmitter transporter, taurine), member 6	0.69	0.76	1.12	0.65
TCN1	TCN1;TC-1;TC1;TCI	transcobalamin I (vitamin B12 binding protein, R binder family)	0.68	0.68	1.12	0.55
C11orf63	C11orf63;FLJ23554	chromosome 11 open reading frame 63	0.68	0.74	1.12	0.57
RTN1	RTN1;MGC133250;NSP	reticulon 1	0.68	0.74	1.12	0.62
CGB1	CGB1	chorionic gonadotropin, beta polypeptide 1	0.68	0.77	1.12	0.59
HS3ST1	HS3ST1;3OST;3OST1	heparan sulfate (glucosamine) 3-O-sulfotransferase 1	0.68	0.74	1.12	0.55
TFCP2L1	TFCP2L1;CRTR1;LBP-9;LBP9	transcription factor CP2-like 1	0.68	0.76	1.12	0.53
RERE	RERE;ARG;ARP;ATN1L;DNB1;FLJ38775;KIAA0458	arginine-glutamic acid dipeptide (RE) repeats	0.68	0.74	1.12	0.55
PEX11B	PEX11B;PEX11-BETA	peroxisomal biogenesis factor 11 beta	0.68	0.74	1.12	0.56
NUDCD3	NUDCD3;KIAA1068;NudCL	NudC domain containing 3	0.68	0.8	1.12	0.57
EGLN1	EGLN1;C1orf12;DKFZp761F179;ECYT3;HIF-PH2;HIFPH2;HPH-2;HPH2;PHD2;SM20;ZMYND6	egl nine homolog 1 (C. elegans)	0.68	0.75	1.12	0.65
EIF4A2	EIF4A2;BM-010;DDX2B;EIF4A;EIF4F;eIF-4A-II;eIF4A-II	eukaryotic translation initiation factor 4A2	0.68	0.7	1.12	0.63
DBP	DBP;DABP	D site of albumin promoter (albumin D-box) binding protein	0.67	0.7	1.12	0.56
GTPBP1	GTPBP1;GP-1;GP1;HSPC018;MGC20069	GTP binding protein 1	0.67	0.73	1.12	0.53
KCND3	KCND3;KCND3L;KCND3S;KSHIVB;KV4.3;MGC142035;MGC142037	potassium voltage-gated channel, Shal-related subfamily, member 3	0.66	0.78	1.12	0.61
KLK11	KLK11;MGC33060;PRSS20;TLSP	kallikrein-related peptidase 11	0.66	0.74	1.12	0.55
STX1B	STX1B;STX1B1;STX1B2	syntaxin 1B	0.66	0.77	1.12	0.6
HSD11B1L	HSD11B1L;SCDR10;SDR26C2	hydroxysteroid (11-beta) dehydrogenase 1-like	0.66	0.78	1.12	0.56
C10orf54	C10orf54;B7-H5;GI24;PP2135;SISP1	chromosome 10 open reading frame 54	0.66	0.72	1.12	0.6
ACY1	ACY1;ACY-1;ACY1D	aminoacylase 1	0.66	0.74	1.12	0.55
GOSR2	GOSR2;Bos1;EPM6;GS27	golgi SNAP receptor complex member 2	0.66	0.74	1.12	0.55
CKMT1A	CKMT1A;CKMT1;CKMT1B;FLJ50967;UMTCK	creatine kinase, mitochondrial 1A	0.66	0.72	1.12	0.6
TTLL4	TTLL4;KIAA0173	tubulin tyrosine ligase-like family, member 4	0.66	0.75	1.12	0.5
SCARB2	SCARB2;AMRF;CD36L2;EPM4;HLGP85;LGP85;LIMP-2;LIMPII;SR-BII	scavenger receptor class B, member 2	0.66	0.71	1.12	0.52
COMMD10	COMMD10;FLJ11285;PTD002	COMM domain containing 10	0.66	0.72	1.12	0.55
SHMT2	SHMT2;GLYA;SHMT	serine hydroxymethyltransferase 2 (mitochondrial)	0.66	0.71	1.12	0.51
PI4KA	PI4KA;FLJ16556;PI4K-ALPHA;PIK4CA;pi4K230	phosphatidylinositol 4-kinase, catalytic, alpha	0.66	0.74	1.12	0.61
PDIA4	PDIA4;ERP70;ERP72;ERp-72	protein disulfide isomerase family A, member 4	0.66	0.72	1.12	0.56
PRSS37	PRSS37;TRYX2	protease, serine, 37	0.66	0.74	1.12	0.5
FAM193B	FAM193B;DKFZp313I142;FLJ10404;IRIZIO;KIAA1931	family with sequence similarity 193, member B	0.66	0.72	1.12	0.57
MGST3	MGST3;GST-III	microsomal glutathione S-transferase 3	0.65	0.74	1.12	0.49
TMEM39A	TMEM39A;FLJ10902	transmembrane protein 39A	0.65	0.7	1.12	0.5
ZBTB38	ZBTB38;CIBZ;FLJ22332;FLJ31131;FLJ35036;ZNF921	zinc finger and BTB domain containing 38	0.65	0.69	1.12	0.58
CCT7	CCT7;CCTETA;CCTH;MGC110985;NIP7-1;TCP1ETA	chaperonin containing TCP1, subunit 7 (eta)	0.65	0.67	1.12	0.54
TRAF1	TRAF1;EBI6;MGC:10353	TNF receptor-associated factor 1	0.64	0.75	1.12	0.53
MYOM3	MYOM3;FLJ35961;MGC71483;RP11-293P20.1	myomesin family, member 3	0.64	0.73	1.12	0.53
DNAL1	DNAL1;C14orf168;CILD16;MGC12435	dynein, axonemal, light chain 1	0.64	0.63	1.12	0.49
PAEP	PAEP;GD;GdA;GdF;GdS;MGC138509;MGC142288;PAEG;PEP;PP14	progestagen-associated endometrial protein	0.64	0.72	1.12	0.56
TBX1	TBX1;CAFS;CTHM;DGCR;DGS;DORV;TBX1C;TGA;VCFS	T-box 1	0.64	0.66	1.12	0.58
ATG10	ATG10;APG10;APG10L;DKFZp586I0418;FLJ13954;pp12616	ATG10 autophagy related 10 homolog (S. cerevisiae)	0.64	0.74	1.12	0.56
PEX3	PEX3;DKFZp686N14184;FLJ13531;TRG18	peroxisomal biogenesis factor 3	0.64	0.76	1.12	0.56
ZNF124	ZNF124;HZF-16;HZF16;MGC117046;ZK7	zinc finger protein 124	0.64	0.75	1.12	0.53
ANKHD1-EIF4	ANKHD1-EIF4EBP3;MASK-BP3;MASK-BP3ARF	ANKHD1-EIF4EBP3 readthrough	0.64	0.7	1.12	0.56
PARD3	PARD3;ASIP;Baz;Bazooka;FLJ21015;PAR3;PAR3alpha;PARD3A;SE2-5L16;SE2-5LT1;SE2-5T2	par-3 partitioning defective 3 homolog (C. elegans)	0.64	0.76	1.12	0.54
SPDYE1	SPDYE1;DKFZp434A1014;Ringo1;SPDYE;WBSCR19	speedy homolog E1 (Xenopus laevis)	0.64	0.73	1.12	0.56
HMP19	HMP19	HMP19 protein	0.63	0.72	1.12	0.52
YPEL4	YPEL4;FLJ30213;MGC102723;MGC138324	yippee-like 4 (Drosophila)	0.63	0.76	1.12	0.58
NUDT16L1	NUDT16L1;MGC11275;SDOS	nudix (nucleoside diphosphate linked moiety X)-type motif 16-like 1	0.63	0.76	1.12	0.51
KAL1	KAL1;ADMLX;HHA;KAL;KALIG-1;KMS	Kallmann syndrome 1 sequence	0.62	0.64	1.12	0.57
HGFAC	HGFAC;HGFA;MGC138395;MGC138397	HGF activator	0.62	0.64	1.12	0.48
CHID1	CHID1;FLJ42707;MGC3234;SI-CLP	chitinase domain containing 1	0.62	0.68	1.12	0.48
ZNF414	ZNF414;MGC15716;ZFP414	zinc finger protein 414	0.62	0.74	1.12	0.52
TMEM11	TMEM11;C17orf35;PM1;PMI	transmembrane protein 11	0.62	0.68	1.12	0.61
DPH5	DPH5;CGI-30;HSPC143;MGC61450;NPD015	DPH5 homolog (S. cerevisiae)	0.62	0.7	1.12	0.54
GINS2	GINS2;HSPC037;PSF2;Pfs2	GINS complex subunit 2 (Psf2 homolog)	0.62	0.7	1.12	0.51
H2AFZ	H2AFZ;H2A.z;H2A/z;H2AZ;MGC117173	H2A histone family, member Z	0.62	0.67	1.12	0.54
LAPTM4B	LAPTM4B;LAPTM4beta;LC27	lysosomal protein transmembrane 4 beta	0.62	0.72	1.12	0.55
KRTAP5-8	KRTAP5-8;KRTAP5-2;KRTAP5.8;UHSKerB	keratin associated protein 5-8	0.62	0.63	1.12	0.5
CERS1	CERS1;LAG1;LASS1;MGC90349;UOG1	ceramide synthase 1	0.62	0.71	1.12	0.54
GPR87	GPR87;GPR95;KPG_002;MGC131898	G protein-coupled receptor 87	0.61	0.66	1.12	0.6
PRKDC	PRKDC;DNA-PKcs;DNAPK;DNPK1;HYRC;HYRC1;XRCC7;p350	protein kinase, DNA-activated, catalytic polypeptide	0.61	0.66	1.12	0.6
HTR2C	HTR2C;5-HT2C;5-HTR2C;HTR1C	5-hydroxytryptamine (serotonin) receptor 2C	0.84	0.92	1.13	0.83
RNASE13	RNASE13	ribonuclease, RNase A family, 13 (non-active)	0.84	0.91	1.13	0.7
TMEM81	TMEM81;HC3107;KVLA2788;MGC75217;UNQ2788	transmembrane protein 81	0.84	0.94	1.13	0.68
SLC34A3	SLC34A3;FLJ38680;HHRH;NPTIIc	solute carrier family 34 (sodium phosphate), member 3	0.84	0.91	1.13	0.66
MTERFD3	MTERFD3;FLJ14062;mTERF2;mTERFL	MTERF domain containing 3	0.84	0.84	1.13	0.66
ZNF26	ZNF26;FLJ20755;KOX20	zinc finger protein 26	0.84	0.93	1.13	0.78
BNC2	BNC2;BSN2;DKFZp686A01127;FLJ20043;FLJ34928	basonuclin 2	0.84	1.04	1.13	0.76
ATG16L1	ATG16L1;APG16L;ATG16A;ATG16L;FLJ00045;FLJ10035;FLJ10828;FLJ22677;IBD10;WDR30	ATG16 autophagy related 16-like 1 (S. cerevisiae)	0.84	0.99	1.13	0.68
CHM	CHM;DXS540;FLJ38564;GGTA;HSD-32;MGC102710;REP-1;TCD	choroideremia (Rab escort protein 1)	0.84	0.94	1.13	0.73
RABGEF1	RABGEF1;FLJ32302;RABEX5;rabex-5	RAB guanine nucleotide exchange factor (GEF) 1	0.84	0.98	1.13	0.74
ALG8	ALG8;CDG1H;MGC2840	asparagine-linked glycosylation 8, alpha-1,3-glucosyltransferase homolog (S. cerevisiae)	0.84	0.97	1.13	0.75
PSEN1	PSEN1;AD3;FAD;PS-1;PS1;S182	presenilin 1	0.84	0.92	1.13	0.65
UBE2Z	UBE2Z;FLJ13855;USE1	ubiquitin-conjugating enzyme E2Z	0.84	0.88	1.13	0.66
SRPK1	SRPK1;SFRSK1	SRSF protein kinase 1	0.84	1.02	1.13	0.73
CEP112	CEP112;CCDC46;FLJ39610;MACOCO;MGC33887	centrosomal protein 112kDa	0.84	1.01	1.13	0.78
GFAP	GFAP;FLJ42474;FLJ45472	glial fibrillary acidic protein	0.83	0.9	1.13	0.73
SLC6A4	SLC6A4;5-HTT;5-HTTLPR;5HTT;HTT;OCD1;SERT;SERT1;hSERT	solute carrier family 6 (neurotransmitter transporter, serotonin), member 4	0.82	0.88	1.13	0.7
FFAR3	FFAR3;FFA3R;GPR41	free fatty acid receptor 3	0.82	0.91	1.13	0.82
HARBI1	HARBI1;C11orf77;FLJ32675	harbinger transposase derived 1	0.82	0.88	1.13	0.72
FTSJD1	FTSJD1;AFT;FLJ11171	FtsJ methyltransferase domain containing 1	0.82	0.9	1.13	0.73
RASA3	RASA3;GAP1IP4BP;GAPIII;MGC46517;MGC47588	RAS p21 protein activator 3	0.82	0.88	1.13	0.64
RAD52	RAD52	RAD52 homolog (S. cerevisiae)	0.82	0.9	1.13	0.67
ZMYND19	ZMYND19;MIZIP;RP11-48C7.4	zinc finger, MYND-type containing 19	0.82	0.85	1.13	0.67
CASP6	CASP6;MCH2	caspase 6, apoptosis-related cysteine peptidase	0.82	0.86	1.13	0.64
BTG3	BTG3;ANA;MGC8928;TOB5;TOB55;TOFA	BTG family, member 3	0.82	0.96	1.13	0.73
LAMB1	LAMB1;CLM;MGC142015	laminin, beta 1	0.82	0.96	1.13	0.71
PLEC	PLEC;EBS1;EBSO;HD1;LGMD2Q;PCN;PLEC1;PLEC1b;PLTN	plectin	0.82	0.88	1.13	0.68
AGBL1	AGBL1;FLJ32310;MGC149239	ATP/GTP binding protein-like 1	0.81	0.9	1.13	0.67
LYPD1	LYPD1;FLJ41033;LYPDC1;MGC29643;PHTS	LY6/PLAUR domain containing 1	0.81	0.88	1.13	0.67
KIF19	KIF19;FLJ37300;KIF19A	kinesin family member 19	0.81	0.88	1.13	0.68
GPR135	GPR135;HUMNPIIY20	G protein-coupled receptor 135	0.81	0.88	1.13	0.8

FCN2	FCN2;EBP-37;FCNL;P35;ficolin-2	ficolin (collagen/fibrinogen domain containing lectin) 2 (hucolin)	0.81	0.94	1.13	0.71
REXO4	REXO4;REX4;XPMC2;XPMC2H	REX4, RNA exonuclease 4 homolog (S. cerevisiae)	0.81	0.98	1.13	0.64
STXBP2	STXBP2;FHL5;Hunc18b;MUNC18-2;UNC18-2;UNC18B;pp10122	syntaxin binding protein 2	0.81	0.9	1.13	0.75
SHCBP1L	SHCBP1L;C1orf14;GE36;MGC26911;MGC48296	SHC SH2-domain binding protein 1-like	0.81	0.92	1.13	0.64
MRGPRX2	MRGPRX2;MRGX2	MAS-related GPR, member X2	0.8	0.84	1.13	0.63
EDA	EDA;ED1;ED1-A1;ED1-A2;EDA1;EDA2;HED;ODT1;STHAGX1;XHED;XLHED	ectodysplasin A	0.8	0.93	1.13	0.69
ATHL1	ATHL1;FLJ22635;MGC129858;MGC129859	ATH1, acid trehalase-like 1 (yeast)	0.8	0.88	1.13	0.76
RBP5	RBP5;CRBP-III;CRBP3;CRBPIII	retinol binding protein 5, cellular	0.8	0.88	1.13	0.66
CACNB3	CACNB3;CAB3;CACNLB3;FLJ58949	calcium channel, voltage-dependent, beta 3 subunit	0.8	0.9	1.13	0.7
MSLNL	MSLNL;C16orf37;MPFL	mesothelin-like	0.8	0.97	1.13	0.68
BAIAP2	BAIAP2;BAP2;FLAF3;IRSP53	BAI1-associated protein 2	0.8	0.97	1.13	0.72
PRKAA2	PRKAA2;AMPK;AMPK2;AMPKa2;PRKAA	protein kinase, AMP-activated, alpha 2 catalytic subunit	0.8	0.86	1.13	0.78
NR3C1	NR3C1;GCCR;GCR;GR;GRL	nuclear receptor subfamily 3, group C, member 1 (glucocorticoid receptor)	0.8	0.97	1.13	0.64
TCF25	TCF25;Hulp1;KIAA1049;NULP1;PRO2620;hKIAA1049	transcription factor 25 (basic helix-loop-helix)	0.8	0.92	1.13	0.68
POP4	POP4;RPP29	processing of precursor 4, ribonuclease P/MRP subunit (S. cerevisiae)	0.8	0.91	1.13	0.61
MRPS10	MRPS10;FLJ10567;MRP-S10;PNAS-122	mitochondrial ribosomal protein S10	0.8	0.96	1.13	0.71
FNDC4	FNDC4;FLJ22362;FRCP1	fibronectin type III domain containing 4	0.79	0.92	1.13	0.7
MAN2C1	MAN2C1;DKFZp686E23167;MAN6A8;MANA;MANA1;MGC87979	mannosidase, alpha, class 2C, member 1	0.79	0.88	1.13	0.62
THAP5	THAP5;DKFZp313O1132	THAP domain containing 5	0.79	0.93	1.13	0.7
CALML4	CALML4;MGC4809;NY-BR-20	calmodulin-like 4	0.78	0.94	1.13	0.72
ZNF835	ZNF835;BC37295_3	zinc finger protein 835	0.78	0.87	1.13	0.65
SIX5	SIX5;BOR2;DMAHP	SIX homeobox 5	0.78	0.92	1.13	0.62
MKRN2	MKRN2;RNF62	makorin ring finger protein 2	0.78	0.88	1.13	0.79
BCL2L14	BCL2L14;BCLG	BCL2-like 14 (apoptosis facilitator)	0.78	0.81	1.13	0.72
ZNF331	ZNF331;DKFZp686L0787;RITA;ZNF361;ZNF463	zinc finger protein 331	0.77	0.85	1.13	0.68
TRDMT1	TRDMT1;DMNT2;DNMT2;MHSAIIP;PUMET;RNMT1	tRNA aspartic acid methyltransferase 1	0.76	0.83	1.13	0.72
SCN2B	SCN2B	sodium channel, voltage-gated, type II, beta	0.76	0.83	1.13	0.65
CST7	CST7;CMAP	cystatin F (leukocystatin)	0.76	0.81	1.13	0.64
SGSM2	SGSM2;KIAA0397;RUTBC1	small G protein signaling modulator 2	0.76	0.84	1.13	0.67
ZNF444	ZNF444;EZF-2;EZF2;FLJ11137;ZSCAN17	zinc finger protein 444	0.76	0.86	1.13	0.58
HUS1	HUS1;hHUS1	HUS1 checkpoint homolog (S. pombe)	0.76	0.84	1.13	0.75
MLL5	MLL5;FLJ10078;FLJ14026;HDCMC04P;KMT2E;MGC70452	myeloid/lymphoid or mixed-lineage leukemia 5 (trithorax homolog, Drosophila)	0.76	0.82	1.13	0.59
HIATL1	HIATL1;FLJ14753;MGC117350	hippocampus abundant transcript-like 1	0.76	0.82	1.13	0.66
ATL1	ATL1;AD-FSP;FSP1;GBP3;HSN1D;SPG3;SPG3A;atlastin1	atlastin GTPase 1	0.76	0.8	1.13	0.64
CTNNA2	CTNNA2;CAP-R;CAPR;CT114;CTNR;DKFZp686H02198	catenin (cadherin-associated protein), alpha 2	0.75	0.88	1.13	0.66
RBM24	RBM24;FLJ26355;FLJ30829;FLJ37697;RNPC6;dJ259A10.1	RNA binding motif protein 24	0.75	0.8	1.13	0.67
C5orf42	C5orf42;DKFZp686K02105;FLJ13231;FLJ21126	chromosome 5 open reading frame 42	0.75	0.82	1.13	0.62
DHRS1	DHRS1;DKFZp586I0523;FLJ14250;FLJ25430;MGC20204;SDR19C1	dehydrogenase/reductase (SDR family) member 1	0.75	0.91	1.13	0.69
MLLT11	MLLT11;AF1Q;RP11-316M1.10	myeloid/lymphoid or mixed-lineage leukemia (trithorax homolog, Drosophila); translocated to, 11	0.75	0.8	1.13	0.62
POMT1	POMT1;FLJ37239;LGMD2K;MDDGA1;MDDGB1;MDDGC1;RT	protein-O-mannosyltransferase 1	0.75	0.94	1.13	0.65
GNAT1	GNAT1;CSNBAD3;GBT1;GNATR	guanine nucleotide binding protein (G protein), alpha transducing activity polypeptide 1	0.74	0.79	1.13	0.67
PHYHD1	PHYHD1;MGC16638	phytanoyl-CoA dioxygenase domain containing 1	0.74	0.77	1.13	0.61
TTC9B	TTC9B;FLJ30373;MGC33962	tetratricopeptide repeat domain 9B	0.74	0.91	1.13	0.61
SPAG16	SPAG16;DKFZp666P1710;FLJ22724;FLJ37717;MGC87036;PF20;WDR29	sperm associated antigen 16	0.74	0.81	1.13	0.62
NOS1	NOS1;IHPS1;N-NOS;NC-NOS;NOS;bNOS;nNOS	nitric oxide synthase 1 (neuronal)	0.74	0.84	1.13	0.65
GSTZ1	GSTZ1;GSTZ1-1;MAAI;MAI;MGC2029	glutathione transferase zeta 1	0.74	0.77	1.13	0.68
CPT1B	CPT1B;CPT1-M;CPT1M;CPTI;CPTI-M;FLJ55729;FLJ58750;KIAA1670;M-CPT1;MCCPT1;MCPT1	carnitine palmitoyltransferase 1B (muscle)	0.74	0.85	1.13	0.66
ZNRD1	ZNRD1;HTEX-6;MGC13376;Rpa12;TEX6;hZR14;tctex-6	zinc ribbon domain containing 1	0.74	0.88	1.13	0.58
C4orf29	C4orf29;FLJ21106;MGC149227;MGC149228	chromosome 4 open reading frame 29	0.74	0.87	1.13	0.62
C2orf68	C2orf68;FLJ14112;FLJ35653;MGC131675	chromosome 2 open reading frame 68	0.74	0.78	1.13	0.66
RPL28	RPL28;FLJ43307;L28	ribosomal protein L28	0.74	0.85	1.13	0.67
ACADSB	ACADSB;2-MEBCAD;ACAD7;SBCAD	acyl-CoA dehydrogenase, short/branched chain	0.73	0.9	1.13	0.66
ZRANB2	ZRANB2;DKFZp686J1831;DKFZp686N09117;FLJ41119;ZIS;ZIS1;ZIS2;ZNF265	zinc finger, RAN-binding domain containing 2	0.73	0.8	1.13	0.61
PLRG1	PLRG1;MGC110980;PRL1;PRP46;PRPF46	pleiotropic regulator 1	0.73	0.86	1.13	0.57
PROX1	PROX1	prospero homeobox 1	0.72	0.8	1.13	0.59
GABRG3	GABRG3	gamma-aminobutyric acid (GABA) A receptor, gamma 3	0.72	0.86	1.13	0.67
ACCN1	ACCN1;ACCN;ASIC2;ASIC2a;BNC1;BNaC1;MDEG;hBNaC1	amiloride-sensitive cation channel 1, neuronal	0.72	0.87	1.13	0.67
OR10P1	OR10P1;OR10P1P;OR10P2P;OR10P3P;OR12-7;OST701	olfactory receptor, family 10, subfamily P, member 1	0.72	0.85	1.13	0.64
PAQR6	PAQR6;FLJ22672	progestin and adipoQ receptor family member VI	0.72	0.85	1.13	0.57
GFI1B	GFI1B	growth factor independent 1B transcription repressor	0.72	0.82	1.13	0.54
CYP4V2	CYP4V2;BCD;CYP4AH1;FLJ18432;MGC43534	cytochrome P450, family 4, subfamily V, polypeptide 2	0.72	0.83	1.13	0.64
FAM132A	FAM132A;C1QDC2;C1QTNF12;CTRP12;MGC105127	family with sequence similarity 132, member A	0.72	0.84	1.13	0.63
HCN2	HCN2;BCNG-2;BCNG2;HAC-1	hyperpolarization activated cyclic nucleotide-gated potassium channel 2	0.72	0.85	1.13	0.66
DOLPP1	DOLPP1;LSFR2	dolichyl pyrophosphate phosphatase 1	0.72	0.78	1.13	0.61
C1orf229	C1orf229;FLJ45717	chromosome 1 open reading frame 229	0.72	0.73	1.13	0.61
OR4D6	OR4D6;OR11-250	olfactory receptor, family 4, subfamily D, member 6	0.71	0.84	1.13	0.56
DHRS4L2	DHRS4L2;FLJ11525;MGC905;SDR25C3	dehydrogenase/reductase (SDR family) member 4 like 2	0.71	0.72	1.13	0.57
LATS1	LATS1;WARTS;wts	LATS, large tumor suppressor, homolog 1 (Drosophila)	0.71	0.79	1.13	0.65
C10orf129	C10orf129;ACSM6;bA310E22.3	chromosome 10 open reading frame 129	0.7	0.76	1.13	0.55
KRCC1	KRCC1;CHBP2;FLJ22333	lysine-rich coiled-coil 1	0.7	0.76	1.13	0.61
HIST1H2AC	HIST1H2AC;H2A/l;H2AFL;MGC99519;dJ221C16.4	histone cluster 1, H2ac	0.7	0.76	1.13	0.6
SMARCA4	SMARCA4;BAF190;BRG1;FLJ39786;RTPS2;SNF2;SNF2-BETA;SNF2L4;SNF2LB;SWI2;hSNF2b	SWI/SNF related, matrix associated, actin dependent regulator of chromatin, subfamily a, member 4	0.7	0.77	1.13	0.58
B4GALT5	B4GALT5;B4Gal-T5;BETA4-GALT-IV;MGC138470;beta4Gal-T5;beta4GalT-V;gt-V	UDP-Gal:betaGlcNAc beta 1,4- galactosyltransferase, polypeptide 5	0.7	0.79	1.13	0.61
PA2G4	PA2G4;EBP1;HG4-1;p38-2G4	proliferation-associated 2G4, 38kDa	0.7	0.78	1.13	0.59
ASAP2	ASAP2;AMAP2;CENTB3;DDEF2;FLJ42910;KIAA0400;PAG3;PAP;Pap-alpha;SHAG1	ArfGAP with SH3 domain, ankyrin repeat and PH domain 2	0.7	0.76	1.13	0.57
SERTM1	SERTM1;C13orf36;MGC33996	serine-rich and transmembrane domain containing 1	0.7	0.84	1.13	0.59
EPHA3	EPHA3;EK4;ETK;ETK1;HEK;HEK4;TYRO4	EPH receptor A3	0.69	0.84	1.13	0.6
LGALS7B	LGALS7B;GAL7;Gal-7;HKL-14;LGALS7;MGC75149;MGC88745;PI7	lectin, galactoside-binding, soluble, 7B	0.69	0.78	1.13	0.54
KLK10	KLK10;NES1;PRSSL1	kallikrein-related peptidase 10	0.69	0.71	1.13	0.53
CCL13	CCL13;CKb10;MCP-4;MGC17134;NCC-1;NCC1;SCYA13;SCYL1	chemokine (C-C motif) ligand 13	0.68	0.78	1.13	0.6
IL1F10	IL1F10;FIL1-theta;IL-1HY2;IL1-theta;MGC119831;MGC119832;MGC119833	interleukin 1 family, member 10 (theta)	0.68	0.78	1.13	0.6
LTA	LTA;LT;TNFB;TNFSF1	lymphotoxin alpha (TNF superfamily, member 1)	0.68	0.73	1.13	0.53
ZNF793	ZNF793	zinc finger protein 793	0.68	0.82	1.13	0.58
C10orf99	C10orf99;FLJ21763;UNQ1833	chromosome 10 open reading frame 99	0.68	0.78	1.13	0.53
APPBP2	APPBP2;HS.84084;KIAA0228;PAT1	amyloid beta precursor protein (cytoplasmic tail) binding protein 2	0.68	0.74	1.13	0.53
ZNF28	ZNF28;DKFZp781D0275;KOX24	zinc finger protein 28	0.68	0.76	1.13	0.63
NDUFB7	NDUFB7;B18;CI-B18;MGC2480	NADH dehydrogenase (ubiquinone) 1 beta subcomplex, 7, 18kDa	0.68	0.75	1.13	0.65
ST14	ST14;HAI;MT-SP1;MTSP1;PRSS14;SNC19;TADG15;TMPRSS14	suppression of tumorigenicity 14 (colon carcinoma)	0.68	0.71	1.13	0.53
GCFC1	GCFC1;BM020;C21orf66;FLJ90561;FSAP105;GCFC	GC-rich sequence DNA-binding factor 1	0.68	0.79	1.13	0.6
HIST2H4B	HIST2H4B;H4/o;HIST1H4A;HIST1H4B;HIST1H4C;HIST1H4D;HIST1H4E;HIST1H4F;HIST1H4H;HIST1H4I;HIST1H4J;HIST1H4K;HIST1H4L;HIST2H4A;HIST4H4	histone cluster 2, H4b	0.68	0.8	1.13	0.57
PSORS1C1	PSORS1C1;C6orf16;MGC132530;SEEK1	psoriasis susceptibility 1 candidate 1	0.67	0.8	1.13	0.53
SLC6A1	SLC6A1;GABATHG;GABATR;GAT1	solute carrier family 6 (neurotransmitter transporter, GABA), member 1	0.67	0.75	1.13	0.56
ITGAD	ITGAD;ADB2;CD11D;FLJ39841	integrin, alpha D	0.67	0.68	1.13	0.57
KLHL23	KLHL23;DITHP;FLJ37812;FLJ41460;MGC22679;MGC2610	kelch-like 23 (Drosophila)	0.67	0.82	1.13	0.63
LRRC36	LRRC36;FLJ11004;RORBP70;XLHSRF2	leucine rich repeat containing 36	0.66	0.71	1.13	0.58
PYDC2	PYDC2;POP2;cPOP2	pyrin domain containing 2	0.66	0.72	1.13	0.54
KRTAP1-3	KRTAP1-3;KAP1.2;KAP1.3;KAP1.6;KAP1.8A;KAP1.8B;KAP1.9;KRTAP1.3;MGC119903;MGC119904;MGC119905	keratin associated protein 1-3	0.66	0.77	1.13	0.59
STX8	STX8;CARB	syntaxin 8	0.66	0.74	1.13	0.51
BOLA1	BOLA1;MGC75015;RP11-196G18.18;hBolA	bolA homolog 1 (E. coli)	0.66	0.8	1.13	0.62
ERLIN2	ERLIN2;C8orf2;Erlin-2;MGC87072;NET32;SPFH2	ER lipid raft associated 2	0.66	0.8	1.13	0.62
CSPP1	CSPP1;CSPP;FLJ22490;FLJ38886	centrosome and spindle pole associated protein 1	0.66	0.77	1.13	0.55
MCAM	MCAM;CD146;MUC18	melanoma cell adhesion molecule	0.66	0.74	1.13	0.52
YTHDC1	YTHDC1;KIAA1966;YT521;YT521-B	YTH domain containing 1	0.66	0.72	1.13	0.55
SYT8	SYT8;DKFZp434K0322	synaptotagmin VIII	0.65	0.76	1.13	0.51
PSMB10	PSMB10;FLJ00366;LMP10;MECL1;MGC1665;beta2i	proteasome (prosome, macropain) subunit, beta type, 10	0.65	0.72	1.13	0.54
MSI1	MSI1	musashi homolog 1 (Drosophila)	0.65	0.67	1.13	0.53
RBM19	RBM19;DKFZp586F1023;KIAA0682	RNA binding motif protein 19	0.65	0.66	1.13	0.55
MIPOL1	MIPOL1;DKFZp313M2036;MGC34010	mirror-image polydactyly 1	0.64	0.74	1.13	0.56
OBP2A	OBP2A;OBP;OBP2C;OBPIIa;hOBPIIa	odorant binding protein 2A	0.64	0.78	1.13	0.58
CBR3	CBR3;SDR21C2;hCBR3	carbonyl reductase 3	0.64	0.68	1.13	0.54
HIP1R	HIP1R;FLJ14000;FLJ27022;HIP12;HIP3;ILWEQ;KIAA0655;MGC47513	huntingtin interacting protein 1 related	0.64	0.74	1.13	0.51
IRF6	IRF6;LPS;OFC6;PIT;PPS;VWS;VWS1	interferon regulatory factor 6	0.64	0.68	1.13	0.53
ZWINT	ZWINT;HZwint-1;KNTC2AP;MGC117174;ZWINT1	ZW10 interactor	0.64	0.76	1.13	0.5
FAM49B	FAM49B;DKFZp686B04128;L1	family with sequence similarity 49, member B	0.64	0.75	1.13	0.57
C3orf56	C3orf56;FLJ40141	chromosome 3 open reading frame 56	0.63	0.68	1.13	0.52
ZNF653	ZNF653;E430039K05Rik;ZIP67	zinc finger protein 653	0.63	0.76	1.13	0.53
IRAK1	IRAK1;IRAK;pelle	interleukin-1 receptor-associated kinase 1	0.63	0.7	1.13	0.58
NOG	NOG;SYM1;SYNS1	noggin	0.62	0.68	1.13	0.55
SFRP5	SFRP5;SARP3	secreted frizzled-related protein 5	0.62	0.69	1.13	0.49
NTNG2	NTNG2;KIAA0625;KIAA1857;LHLL9381;Lmnt2;MGC21884;NTNG1;bA479K20.1	netrin G2	0.62	0.64	1.13	0.5
ZNF695	ZNF695;MGC61907;RP11-551G24.1;SBZF3	zinc finger protein 695	0.62	0.7	1.13	0.5
C7orf26	C7orf26;MGC2718	chromosome 7 open reading frame 26	0.62	0.73	1.13	0.54
LETMD1	LETMD1;1110019O13Rik;DKFZp586A011;HCCR-1;HCCR-2;HCCR1;HCRR-2	LETM1 domain containing 1	0.62	0.71	1.13	0.53
TBC1D10A	TBC1D10A;EPI64;TBC1D10;dJ130H16.1;dJ130H16.2	TBC1 domain family, member 10A	0.62	0.66	1.13	0.54
NDUFA6	NDUFA6;B14;CI-B14;LYRM6;NADHB14	NADH dehydrogenase (ubiquinone) 1 alpha subcomplex, 6, 14kDa	0.62	0.68	1.13	0.49
HDAC3	HDAC3;HD3;RPD3;RPD3-2	histone deacetylase 3	0.62	0.69	1.13	0.51
TRIM16L	TRIM16L;TRIM70	tripartite motif containing 16-like	0.62	0.69	1.13	0.53
SLC25A3	SLC25A3;PHC;PTP	solute carrier family 25 (mitochondrial carrier; phosphate carrier), member 3	0.62	0.77	1.13	0.53
KIAA0226L	KIAA0226L;C13orf18;FLJ21562;FLJ43762	KIAA0226-like	0.62	0.72	1.13	0.52

C7orf66	C7orf66	chromosome 7 open reading frame 66	0.62	0.73	1.13	0.51
ZC3H14	ZC3H14;FLJ11806;MGC26892;MSUT-2;NY-REN-37;SUT2;UKp68	zinc finger CCCH-type containing 14	0.61	0.74	1.13	0.5
TEK	TEK;CD202B;TIE-2;TIE2;VMCM;VMCM1	TEK tyrosine kinase, endothelial	0.84	0.94	1.14	0.78
FGF5	FGF5;HBGF-5;Smag-82	fibroblast growth factor 5	0.84	1.02	1.14	0.67
SLC14A2	SLC14A2;FLJ16167;HUT2;MGC119566;MGC119567;UT-A2;UT2;UTA;UTR;hUT-A6	solute carrier family 14 (urea transporter), member 2	0.84	0.96	1.14	0.75
TRPV2	TRPV2;MGC12549;VRL;VRL-1;VRL1	transient receptor potential cation channel, subfamily V, member 2	0.84	0.94	1.14	0.74
AMOT	AMOT;KIAA1071	angiomotin	0.84	0.98	1.14	0.74
NPR2	NPR2;AMDM;ANPRB;ANPb;GUC2B;GUCY2B;NPRB;NPRBi	natriuretic peptide receptor B/guanylate cyclase B (atrionatriuretic peptide receptor B)	0.84	0.92	1.14	0.82
EOMES	EOMES;TBR2	eomesodermin	0.84	0.91	1.14	0.66
C1orf52	C1orf52;FLJ44982;RP11-234D19.1;gm117	chromosome 1 open reading frame 52	0.84	0.92	1.14	0.7
SOX3	SOX3;GHDX;MRGH;PHP;PHPX;SOXB	SRY (sex determining region Y)-box 3	0.84	0.98	1.14	0.78
AZI2	AZI2;AZ2;NAP1;TILP	5-azacytidine induced 2	0.84	0.98	1.14	0.74
HK2	HK2;DKFZp686M1669;HKII;HXK2	hexokinase 2	0.84	0.96	1.14	0.79
ACLY	ACLY;ACL;ATPCL;CLATP	ATP citrate lyase	0.84	0.97	1.14	0.65
ADRA2B	ADRA2B;ADRA2L1;ADRA2RL1;ADRARL1;ALPHA2BAR	adrenergic, alpha-2B-, receptor	0.83	0.88	1.14	0.66
TMEM190	TMEM190;MDAC1;MGC149301;MGC149302	transmembrane protein 190	0.83	0.86	1.14	0.68
DENND1B	DENND1B;C1ORF18;C1orf218;DKFZp547O0715;FAM31B;FLJ20054;FLJ21179;FLJ33888;MGC103810;MGC27044	DENN/MADD domain containing 1B	0.83	1.03	1.14	0.69
LRRC8B	LRRC8B;KIAA0231;MGC42220;TA-LRRP;TALRRP	leucine rich repeat containing 8 family, member B	0.83	0.9	1.14	0.69
MOSC2	MOSC2;FLJ20605;MARC2;RP11-270A6.1	MOCO sulphurase C-terminal domain containing 2	0.83	0.92	1.14	0.69
GADD45G	GADD45G;CR6;DDIT2;GADD45gamma;GRP17	growth arrest and DNA-damage-inducible, gamma	0.83	0.93	1.14	0.69
C16orf61	C16orf61;2310061C15Rik	chromosome 16 open reading frame 61	0.83	0.94	1.14	0.68
ATF6B	ATF6B;CREB-RP;CREBL1;FLJ10066;G13	activating transcription factor 6 beta	0.83	0.9	1.14	0.7
ZNF415	ZNF415;FLJ11191	zinc finger protein 415	0.82	0.9	1.14	0.71
SLC7A14	SLC7A14;FLJ37228;KIAA1613	solute carrier family 7 (orphan transporter), member 14	0.82	0.91	1.14	0.76
NR0B1	NR0B1;AHC;AHCH;AHX;DAX-1;DAX1;DSS;GTD;HHG;NROB1;SRXY2	nuclear receptor subfamily 0, group B, member 1	0.82	0.9	1.14	0.71
SIGLEC9	SIGLEC9;CD329;CDw329;FOAP-9;OBBP-LIKE;siglec-9	sialic acid binding Ig-like lectin 9	0.82	0.9	1.14	0.65
ZNF530	ZNF530;KIAA1508	zinc finger protein 530	0.82	0.97	1.14	0.65
HOXA6	HOXA6;HOX1;HOX1.2;HOX1B	homeobox A6	0.82	0.92	1.14	0.72
PGF	PGF;D12S1900;PGFL;PLGF;PlGF-2;SHGC-10760	placental growth factor	0.82	0.99	1.14	0.66
USP3	USP3;MGC129878;MGC129879;SIH003;UBP	ubiquitin specific peptidase 3	0.82	0.87	1.14	0.65
MSTO1	MSTO1;DKFZp686B1757;DKFZp686I01261;FLJ10504;MST;RP11-29H23.3	misato homolog 1 (Drosophila)	0.82	0.9	1.14	0.72
BID	BID;FP497;MGC15319;MGC42355	BH3 interacting domain death agonist	0.82	0.89	1.14	0.7
ABCC1	ABCC1;ABC29;ABCC;DKFZp686N04233;DKFZp781G125;GS-X;MRP;MRP1	ATP-binding cassette, sub-family C (CFTR/MRP), member 1	0.82	0.87	1.14	0.7
DLG1	DLG1;DKFZp761P0818;DKFZp781B0426;DLGH1;SAP-97;SAP97;dJ1061C18.1.1;hdlg	discs, large homolog 1 (Drosophila)	0.82	0.86	1.14	0.65
STAM	STAM;DKFZp686J2352;STAM-1;STAM1	signal transducing adaptor molecule (SH3 domain and ITAM motif) 1	0.82	0.9	1.14	0.65
FLII	FLII;FLI;FLIL;Fli1;MGC39265	flightless I homolog (Drosophila)	0.82	0.91	1.14	0.69
TAF1D	TAF1D;FLJ23363;FLJ32621;FLJ46181;JOSD3;MGC5306;RAFI41;TAF(I)41;TAFI41	TATA box binding protein (TBP)-associated factor, RNA polymerase I, D, 41kDa	0.82	1	1.14	0.77
ASCL1	ASCL1;ASH1;HASH1;MASH1;bHLHa46	achaete-scute complex homolog 1 (Drosophila)	0.81	0.92	1.14	0.68
ZNF773	ZNF773;FLJ00301;MGC4728;ZNF419B	zinc finger protein 773	0.81	0.92	1.14	0.66
ANTXR1	ANTXR1;ATR;FLJ10601;FLJ11298;FLJ21776;TEM8	anthrax toxin receptor 1	0.81	0.89	1.14	0.72
GTF2H1	GTF2H1;BTF2;TFB1;TFIIH	general transcription factor IIH, polypeptide 1, 62kDa	0.81	1	1.14	0.71
PLS3	PLS3;T-plastin	plastin 3	0.81	0.9	1.14	0.75
HOGA1	HOGA1;C10orf65;DHDPS2;DHDPSL;FLJ37472;FLJ56960;HP3;NPL2	4-hydroxy-2-oxoglutarate aldolase 1	0.81	0.96	1.14	0.72
SVEP1	SVEP1;C9orf13;CCP22;FLJ16013;FLJ90719;POLYDOM;SEL-OB;SELOB	sushi, von Willebrand factor type A, EGF and pentraxin domain containing 1	0.8	0.87	1.14	0.66
NOD2	NOD2;ACUG;BLAU;CARD15;CD;CLR16.3;IBD1;NLRC2;NOD2B;PSORAS1	nucleotide-binding oligomerization domain containing 2	0.8	0.88	1.14	0.62
RDM1	RDM1;MGC33977;RAD52B	RAD52 motif 1	0.8	0.89	1.14	0.68
C13orf27	C13orf27	chromosome 13 open reading frame 27	0.8	0.82	1.14	0.64
PRKCE	PRKCE;MGC125656;MGC125657;PKCE;nPKC-epsilon	protein kinase C, epsilon	0.8	0.92	1.14	0.76
NUAK2	NUAK2;DKFZp434J037;DKFZp686F01113;FLJ90349;SNARK	NUAK family, SNF1-like kinase, 2	0.8	0.98	1.14	0.7
POLR3K	POLR3K;C11;C11-RNP3;RPC10;RPC11;RPC12.5;hRPC11	polymerase (RNA) III (DNA directed) polypeptide K, 12.3 kDa	0.8	0.85	1.14	0.75
LCMT2	LCMT2;FLJ51321;KIAA0547;MGC9534;PPM2;TYW4	leucine carboxyl methyltransferase 2	0.8	0.87	1.14	0.68
NPTN	NPTN;DKFZp686L2477;GP55;GP65;MGC102805;SDFR1;SDR1;np55;np65	neuroplastin	0.8	0.88	1.14	0.65
SGCE	SGCE;DYT11;ESG	sarcoglycan, epsilon	0.8	0.89	1.14	0.67
PMF1	PMF1	polyamine-modulated factor 1	0.8	0.9	1.14	0.67
GALP	GALP	galanin-like peptide	0.79	0.88	1.14	0.74
LOC643923	LOC643923	uncharacterized LOC643923	0.79	0.93	1.14	0.7
ZDHHC5	ZDHHC5;DKFZp586K0524;KIAA1748;ZNF375	zinc finger, DHHC-type containing 5	0.79	0.86	1.14	0.62
METTL21B	METTL21B;DKFZp586D0919;FAM119B	methyltransferase like 21B	0.79	0.97	1.14	0.67
PGR	PGR;NR3C3;PR	progesterone receptor	0.78	0.82	1.14	0.65
RIPPLY2	RIPPLY2;C6orf159;dJ237I15.1	ripply2 homolog (zebrafish)	0.78	0.87	1.14	0.65
LILRA1	LILRA1;CD85I;LIR-6;LIR6;MGC126563	leukocyte immunoglobulin-like receptor, subfamily A (with TM domain), member 1	0.78	0.92	1.14	0.7
CCDC69	CCDC69;DKFZp434C171;FLJ13705	coiled-coil domain containing 69	0.78	0.8	1.14	0.63
CPA1	CPA1;CPA	carboxypeptidase A1 (pancreatic)	0.78	0.88	1.14	0.66
KRT84	KRT84;HB4;KRTHB4	keratin 84	0.78	0.88	1.14	0.64
F2RL3	F2RL3;PAR4	coagulation factor II (thrombin) receptor-like 3	0.78	0.84	1.14	0.63
RPS27L	RPS27L	ribosomal protein S27-like	0.78	0.91	1.14	0.69
LIN9	LIN9;BARA;BARPsv;Lin-9;TGS;TGS1	lin-9 homolog (C. elegans)	0.78	0.94	1.14	0.62
C1GALT1	C1GALT1;C1GALT;T-synthase	core 1 synthase, glycoprotein-N-acetylgalactosamine 3-beta-galactosyltransferase, 1	0.78	0.88	1.14	0.68
USP13	USP13;ISOT3;IsoT-3	ubiquitin specific peptidase 13 (isopeptidase T-3)	0.78	0.88	1.14	0.66
DYNLL2	DYNLL2;DNCL1B;Dlc2;MGC17810;RSPH22	dynein, light chain, LC8-type 2	0.78	0.84	1.14	0.68
LANCL2	LANCL2;GPR69B;MGC87139;TASP	LanC lantibiotic synthetase component C-like 2 (bacterial)	0.78	0.94	1.14	0.7
OGFOD1	OGFOD1;FLJ10826;KIAA1612;TPA1	2-oxoglutarate and iron-dependent oxygenase domain containing 1	0.78	0.8	1.14	0.62
UMPS	UMPS;OPRT	uridine monophosphate synthetase	0.78	0.87	1.14	0.68
REEP5	REEP5;C5orf18;D5S346;DP1;MGC70440;TB2;YOP1	receptor accessory protein 5	0.78	0.96	1.14	0.71
CTNNA1	CTNNA1;CAP102;FLJ36832;FLJ52416	catenin (cadherin-associated protein), alpha 1, 102kDa	0.78	0.86	1.14	0.65
RASSF2	RASSF2;CENP-34;DKFZp781O1747;KIAA0168;RASFADIN	Ras association (RalGDS/AF-6) domain family member 2	0.77	0.9	1.14	0.69
ALPPL2	ALPPL2;ALPG;ALPPL;GCAP	alkaline phosphatase, placental-like 2	0.77	0.82	1.14	0.62
TAZ	TAZ;BTHS;CMD3A;EFE;EFE2;FLJ27390;G4.5;LVNCX;Taz1;XAP-2	tafazzin	0.77	0.86	1.14	0.6
TAOK1	TAOK1;FLJ14314;KFC-B;KIAA1361;MAP3K16;MARKK;PSK-2;PSK2;TAO1;hKFC-B;hTAOK1	TAO kinase 1	0.77	0.89	1.14	0.73
MT1G	MT1G;MGC12386;MT1;MT1K	metallothionein 1G	0.77	0.92	1.14	0.63
SUMO3	SUMO3;SMT3A;SMT3H1;SUMO-3;Smt3B	SMT3 suppressor of mif two 3 homolog 3 (S. cerevisiae)	0.77	0.92	1.14	0.69
LDHC	LDHC;CT32;LDH3;LDHX;MGC111073	lactate dehydrogenase C	0.76	0.84	1.14	0.62
TTLL7	TTLL7;FLJ23033;FLJ36855;RP5-836J3.2	tubulin tyrosine ligase-like family, member 7	0.76	0.92	1.14	0.66
FBXO3	FBXO3;DKFZp564B092;FBA;FBX3	F-box protein 3	0.76	0.84	1.14	0.6
EDEM2	EDEM2;C20orf31;C20orf49;FLJ10783;bA4204.1	ER degradation enhancer, mannosidase alpha-like 2	0.76	0.82	1.14	0.65
ZCCHC17	ZCCHC17;HSPC251;PS1D;RP11-266K22.1;pNO40	zinc finger, CCHC domain containing 17	0.76	0.86	1.14	0.62
ZDHHC13	ZDHHC13;FLJ10852;FLJ10941;HIP14L;HIP3RP;MGC64994	zinc finger, DHHC-type containing 13	0.76	0.85	1.14	0.64
TYW5	TYW5;C2orf60;FLJ37953;MGC70509;hTYW5	tRNA-yW synthesizing protein 5	0.76	0.9	1.14	0.62
KRTAP4-12	KRTAP4-12;KAP4.12;KRTAP4.12	keratin associated protein 4-12	0.75	0.94	1.14	0.69
SUSD2	SUSD2;BK65A6.2;FLJ22778	sushi domain containing 2	0.75	0.87	1.14	0.66
HSD17B8	HSD17B8;D6S2245E;FABG;FABGL;H2-KE6;HKE6;KE6;RING2;SDR30C1;dJ1033B10.9	hydroxysteroid (17-beta) dehydrogenase 8	0.75	0.8	1.14	0.63
YTHDF3	YTHDF3;FLJ31657	YTH domain family, member 3	0.75	0.87	1.14	0.69
LGI4	LGI4;LGIL3	leucine-rich repeat LGI family, member 4	0.74	0.88	1.14	0.66
EDN3	EDN3;ET-3;ET3;HSCR4;MGC15067;MGC61498;PPET3;WS4B	endothelin 3	0.74	0.82	1.14	0.64
ANKRD37	ANKRD37;Lrp2bp;MGC111507	ankyrin repeat domain 37	0.74	0.8	1.14	0.65
C6orf57	C6orf57;MGC104225	chromosome 6 open reading frame 57	0.74	0.82	1.14	0.62
MNS1	MNS1;FLJ11222;FLJ26051	meiosis-specific nuclear structural 1	0.74	0.81	1.14	0.62
CACHD1	CACHD1;KIAA1573;RP4-655E10.1	cache domain containing 1	0.74	0.92	1.14	0.61
WDR59	WDR59;FLJ12270;MGC11230	WD repeat domain 59	0.74	0.84	1.14	0.75
TBC1D3C	TBC1D3C;FLJ78248;MGC44903;TBC1D3D	TBC1 domain family, member 3C	0.74	0.76	1.14	0.59
ATXN2	ATXN2;ATX2;FLJ46772;SCA2;TNRC13	ataxin 2	0.74	0.9	1.14	0.63
SNF8	SNF8;Dot3;EAP30;VPS22	SNF8, ESCRT-II complex subunit, homolog (S. cerevisiae)	0.74	0.92	1.14	0.61
COX6A1	COX6A1;COX6A;COX6AL;MGC104500	cytochrome c oxidase subunit VIa polypeptide 1	0.74	0.86	1.14	0.58
EIF6	EIF6;CAB;EIF3A;ITGB4BP;b(2)gcn;eIF-6;p27(BBP);p27BBP	eukaryotic translation initiation factor 6	0.74	0.82	1.14	0.71
EPHX4	EPHX4;ABHD7;EPHXRP;FLJ90341	epoxide hydrolase 4	0.74	0.81	1.14	0.7
PRDM5	PRDM5;BCS2;PFM2	PR domain containing 5	0.73	0.81	1.14	0.7
GALR1	GALR1;GALNR;GALNR1	galanin receptor 1	0.73	0.8	1.14	0.73
DUOXA1	DUOXA1;FLJ32334;NIP;NUMBIP;mol	dual oxidase maturation factor 1	0.73	0.79	1.14	0.64
DAAM1	DAAM1;FLJ41657;KIAA0666	dishevelled associated activator of morphogenesis 1	0.73	0.82	1.14	0.64
NBPF15	NBPF15;AB14;FLJ59046;FLJ78393;MGC8902	neuroblastoma breakpoint family, member 15	0.73	0.83	1.14	0.6
CGB8	CGB8	chorionic gonadotropin, beta polypeptide 8	0.73	0.84	1.14	0.65
TPPP3	TPPP3;p20;p25gamma	tubulin polymerization-promoting protein family member 3	0.72	0.84	1.14	0.63
C15orf59	C15orf59;MGC131524	chromosome 15 open reading frame 59	0.72	0.74	1.14	0.62
EPHB3	EPHB3;ETK2;HEK2;TYRO6	EPH receptor B3	0.72	0.79	1.14	0.71
DHRS3	DHRS3;DD83.1;RDH17;Rsdr1;SDR1;SDR16C1;retSDR1	dehydrogenase/reductase (SDR family) member 3	0.72	0.87	1.14	0.57
OPA3	OPA3;FLJ22187;FLJ25932;MGA3;MGC75494	optic atrophy 3 (autosomal recessive, with chorea and spastic paraplegia)	0.72	0.76	1.14	0.6
MEGF8	MEGF8;C19orf49;EGFL4;FLJ22365;MGC120684;MGC138147;SBP1	multiple EGF-like-domains 8	0.72	0.78	1.14	0.63
HPS5	HPS5;AIBP63;KIAA1017	Hermansky-Pudlak syndrome 5	0.72	0.86	1.14	0.57
WHSC2	WHSC2;FLJ10442;FLJ25112;NELF-A;NELFA	Wolf-Hirschhorn syndrome candidate 2	0.72	0.83	1.14	0.59
RFC1	RFC1;A1;MGC51786;MHCBFB;PO-GA;RECC1;RFC;RFC140	replication factor C (activator 1) 1, 145kDa	0.72	0.8	1.14	0.62
TBCA	TBCA	tubulin folding cofactor A	0.72	0.84	1.14	0.56
DDX56	DDX56;DDX21;DDX26;NOH61	DEAD (Asp-Glu-Ala-Asp) box polypeptide 56	0.72	0.76	1.14	0.67
IST1	IST1;KIAA0174;MGC117220	increased sodium tolerance 1 homolog (yeast)	0.72	0.73	1.14	0.61
ITLN2	ITLN2;HL-2;HL2	intelectin 2	0.71	0.84	1.14	0.6
NPM2	NPM2;MGC78655	nucleophosmin/nucleoplasmin 2	0.71	0.72	1.14	0.58
ADRA2C	ADRA2C;ADRA2L2;ADRA2RL2;ADRARL2;ALPHA2CAR	adrenergic, alpha-2C-, receptor	0.71	0.74	1.14	0.56
IRF2BPL	IRF2BPL;C14orf4;EAP1;KIAA1865	interferon regulatory factor 2 binding protein-like	0.71	0.84	1.14	0.63
TNFAIP8L3	TNFAIP8L3;FLJ41287	tumor necrosis factor, alpha-induced protein 8-like 3	0.7	0.76	1.14	0.58
OTUB2	OTUB2;C14orf137;FLJ21916;MGC3102;OTB2;OTU2	OTU domain, ubiquitin aldehyde binding 2	0.7	0.76	1.14	0.59

KRTAP10-2	KRTAP10-2;KAP10.2;KAP18-2;KAP18.2;KRTAP10.2;KRTAP18-2;KRTAP18.2	keratin associated protein 10-2	0.7	0.76	1.14	0.58
TRPT1	TRPT1;MGC11134	tRNA phosphotransferase 1	0.7	0.85	1.14	0.66
TMEM19	TMEM19;FLJ10936	transmembrane protein 19	0.7	0.78	1.14	0.65
NIPSNAP1	NIPSNAP1	nipsnap homolog 1 (C. elegans)	0.7	0.83	1.14	0.62
MBLAC1	MBLAC1;MGC39819	metallo-beta-lactamase domain containing 1	0.7	0.83	1.14	0.55
BROX	BROX;C1orf58;FLJ32421;MGC142195;MGC142197;RP11-452F19.1	BRO1 domain and CAAX motif containing	0.7	0.82	1.14	0.61
A3GALT2P	A3GALT2P;A3GALT2;IGBS3S	alpha 1,3-galactosyltransferase 2, pseudogene	0.7	0.78	1.14	0.67
OR51D1	OR51D1;OR11-14;OR51D1Q	olfactory receptor, family 51, subfamily D, member 1	0.69	0.77	1.14	0.64
VPS33B	VPS33B;FLJ14848	vacuolar protein sorting 33 homolog B (yeast)	0.69	0.79	1.14	0.55
DDX10	DDX10;HRH-J8	DEAD (Asp-Glu-Ala-Asp) box polypeptide 10	0.69	0.8	1.14	0.53
TBL1Y	TBL1Y;TBL1	transducin (beta)-like 1, Y-linked	0.68	0.74	1.14	0.57
ZNF91	ZNF91;HPF7;HTF10	zinc finger protein 91	0.68	0.77	1.14	0.54
AXIN2	AXIN2;AXIL;DKFZp781B0869;MGC10366;MGC126582	axin 2	0.68	0.8	1.14	0.54
AP4E1	AP4E1;CPSQ4;DKFZp686L12167	adaptor-related protein complex 4, epsilon 1 subunit	0.68	0.8	1.14	0.57
ELP4	ELP4;C11orf19;FLJ20498;PAX6NEB;PAXNEB;dJ68P15A.1	elongation protein 4 homolog (S. cerevisiae)	0.68	0.78	1.14	0.6
PLEKHO1	PLEKHO1;CKIP-1;OC120	pleckstrin homology domain containing, family O member 1	0.68	0.79	1.14	0.59
CLK4	CLK4;DKFZp686A20267	CDC-like kinase 4	0.68	0.84	1.14	0.6
LMAN2L	LMAN2L;DKFZp564L2423;MGC11139;VIPL	lectin, mannose-binding 2-like	0.68	0.8	1.14	0.6
RNF187	RNF187;RACO-1;RACO1	ring finger protein 187	0.68	0.78	1.14	0.69
PQBP1	PQBP1;MRX55;MRXS3;MRXS8;NPW38;RENS1;SHS	polyglutamine binding protein 1	0.68	0.77	1.14	0.64
PLAC8	PLAC8;C15;onzin	placenta-specific 8	0.67	0.77	1.14	0.59
BUB1B	BUB1B;BUB1beta;BUBR1;Bub1A;MAD3L;SSK1;hBUBR1	budding uninhibited by benzimidazoles 1 homolog beta (yeast)	0.67	0.83	1.14	0.59
CHRAC1	CHRAC1;CHARC1;CHARC15;CHRAC-1;CHRAC-15;CHRAC15;YCL1	chromatin accessibility complex 1	0.67	0.78	1.14	0.6
GIMAP5	GIMAP5;FLJ11296;HIMAP3;IAN-5;IAN4;IAN4L1;IAN5;IMAP3;IROD	GTPase, IMAP family member 5	0.66	0.76	1.14	0.52
LPO	LPO;MGC129990;MGC129991;SPO	lactoperoxidase	0.66	0.72	1.14	0.63
RGS11	RGS11;RS11	regulator of G-protein signaling 11	0.66	0.72	1.14	0.55
C1orf96	C1orf96;FLJ37296;FLJ41471	chromosome 1 open reading frame 96	0.66	0.78	1.14	0.58
GLCCI1	GLCCI1;FAM117C;GIG18;TSSN1	glucocorticoid induced transcript 1	0.66	0.78	1.14	0.52
MGAT3	MGAT3;FLJ43371;GNT-III;GNT3;MGC141943;MGC142278	mannosyl (beta-1,4-)-glycoprotein beta-1,4-N-acetylglucosaminyltransferase	0.66	0.72	1.14	0.62
ARSK	ARSK;DKFZp313G1735;TSULF	arylsulfatase family, member K	0.66	0.72	1.14	0.52
KIF18B	KIF18B	kinesin family member 18B	0.66	0.78	1.14	0.52
MRPS5	MRPS5;MRP-S5;S5mt	mitochondrial ribosomal protein S5	0.66	0.78	1.14	0.59
TPM1	TPM1;C15orf13;CMD1Y;CMH3;HTM-alpha;TMSA	tropomyosin 1 (alpha)	0.66	0.75	1.14	0.58
SFRP1	SFRP1;FRP;FRP-1;FRP1;FrzA;SARP2	secreted frizzled-related protein 1	0.65	0.78	1.14	0.52
HLA-C	HLA-C;D6S204;FLJ27082;HLA-JY3;HLC-C;PSORS1	major histocompatibility complex, class I, C	0.65	0.71	1.14	0.56
SLCO5A1	SLCO5A1;FLJ39560;OATP-J;OATP-RP4;OATP5A1;OATPRP4;SLC21A15	solute carrier organic anion transporter family, member 5A1	0.64	0.71	1.14	0.51
CALML3	CALML3;CLP	calmodulin-like 3	0.64	0.75	1.14	0.54
JMJD5	JMJD5;FLJ13798	jumonji domain containing 5	0.64	0.75	1.14	0.5
BAG1	BAG1;HAP;RAP46	BCL2-associated athanogene	0.64	0.78	1.14	0.51
SLC25A28	SLC25A28;DKFZp547C109;MFRN2;MRS3/4;MRS4L	solute carrier family 25, member 28	0.64	0.7	1.14	0.55
PHF14	PHF14;MGC176640	PHD finger protein 14	0.64	0.72	1.14	0.65
RPAP3	RPAP3;FLJ21908	RNA polymerase II associated protein 3	0.64	0.75	1.14	0.57
PRKAB1	PRKAB1;AMPK;HAMPKb;MGC17785	protein kinase, AMP-activated, beta 1 non-catalytic subunit	0.64	0.7	1.14	0.63
HOXB9	HOXB9;HOX-2.5;HOX2;HOX2E	homeobox B9	0.64	0.76	1.14	0.57
ITGB1	ITGB1;CD29;FNRB;GPIIA;MDF2;MSK12;VLA-BETA;VLAB	integrin, beta 1 (fibronectin receptor, beta polypeptide, antigen CD29 includes MDF2, MSK12)	0.64	0.74	1.14	0.58
NSRP1	NSRP1;CCDC55;DKFZp434K1421;FLJ37233;HSPC095;NSrp70	nuclear speckle splicing regulatory protein 1	0.64	0.79	1.14	0.61
SCAF11	SCAF11;CASP11;SFRS2IP;SIP1;SRRP129;SRSF2IP	SR-related CTD-associated factor 11	0.64	0.72	1.14	0.54
EBI3	EBI3;IL-27B;IL27B	Epstein-Barr virus induced 3	0.63	0.66	1.14	0.59
KATNAL1	KATNAL1;MGC2599	katanin p60 subunit A-like 1	0.63	0.7	1.14	0.6
PPARGC1A	PPARGC1A;LEM6;PGC-1(alpha);PGC-1v;PGC1;PGC1A;PPARGC1	peroxisome proliferator-activated receptor gamma, coactivator 1 alpha	0.63	0.7	1.14	0.6
FAM177A1	FAM177A1;C14orf24;DKFZp686J1254;FLJ38854	family with sequence similarity 177, member A1	0.63	0.69	1.14	0.5
ERCC6L	ERCC6L;FLJ20105;MGC131695;PICH	excision repair cross-complementing rodent repair deficiency, complementation group 6-like	0.63	0.7	1.14	0.53
CWF19L1	CWF19L1;FLJ10998	CWF19-like 1, cell cycle control (S. pombe)	0.63	0.74	1.14	0.56
NAA40	NAA40;FLJ13848;NAT11;PATT1	N(alpha)-acetyltransferase 40, NatD catalytic subunit, homolog (S. cerevisiae)	0.63	0.68	1.14	0.53
ZNF501	ZNF501;MGC21738;ZNF;ZNF52	zinc finger protein 501	0.62	0.67	1.14	0.53
C21orf63	C21orf63;B18;B19;C21orf64;SUE21	chromosome 21 open reading frame 63	0.62	0.75	1.14	0.53
TGFBI	TGFBI;BIGH3;CDB1;CDG2;CDGG1;CSD;CSD1;CSD2;CSD3;EBMD;LCD1	transforming growth factor, beta-induced, 68kDa	0.62	0.7	1.14	0.52
WDR61	WDR61;REC14;SKI8	WD repeat domain 61	0.62	0.76	1.14	0.58
PMM1	PMM1;Sec53	phosphomannomutase 1	0.62	0.66	1.14	0.53
HHEX	HHEX;HEX;HMPH;HOX11L-PEN;PRH;PRHX	hematopoietically expressed homeobox	0.62	0.68	1.14	0.59
STK25	STK25;DKFZp686J1430;SOK1;YSK1	serine/threonine kinase 25	0.62	0.7	1.14	0.57
HIST1H4K	HIST1H4K;H4/d;H4F2iii;H4FD;HIST1H4A;HIST1H4B;HIST1H4C;HIST1H4D;HIST1H4E;HIST1H4F;HIST1H4H;HIST1H4I;HIST1H4J;HIST1H4L;HIST2H4A;HIST2H4B;HIST4H4;dJ160A22.1	histone cluster 1, H4k	0.62	0.72	1.14	0.55
SMARCB1	SMARCB1;BAF47;INI1;RDT;RTPS1;SNF5;SNF5L1;Sfh1p;Snr1;hSNFS	SWI/SNF related, matrix associated, actin dependent regulator of chromatin, subfamily b, member 1	0.62	0.68	1.14	0.59
COL13A1	COL13A1;COLXIIIA1;FLJ42485	collagen, type XIII, alpha 1	0.61	0.72	1.14	0.54
CLEC18C	CLEC18C;MGC34761;MRCL;MRCL3	C-type lectin domain family 18, member C	0.61	0.72	1.14	0.48
TRIM21	TRIM21;RNF81;RO52;SSA;SSA1	tripartite motif containing 21	0.84	0.96	1.15	0.86
BARHL1	BARHL1	BarH-like homeobox 1	0.84	0.87	1.15	0.69
TNFRSF25	TNFRSF25;APO-3;DDR3;DR3;LARD;TNFRSF12;TR3;TRAMP;WSL-1;WSL-LR	tumor necrosis factor receptor superfamily, member 25	0.84	0.95	1.15	0.85
BTBD1	BTBD1;C15orf1;NS5ATP8	BTB (POZ) domain containing 1	0.84	0.88	1.15	0.73
ZBTB44	ZBTB44;BTBD15;HSPC063;MGC26123;MGC57431;MGC60348;MGC88058;ZNF851	zinc finger and BTB domain containing 44	0.84	1.03	1.15	0.72
GNG10	GNG10	guanine nucleotide binding protein (G protein), gamma 10	0.84	0.96	1.15	0.71
FAM161A	FAM161A;FLJ13305;MGC129982;MGC129983;RP28	family with sequence similarity 161, member A	0.83	0.86	1.15	0.69
SYDE1	SYDE1;7h3;FLJ13511	synapse defective 1, Rho GTPase, homolog 1 (C. elegans)	0.83	0.98	1.15	0.71
UPK1B	UPK1B;TSPAN20;UPIB;UPK1	uroplakin 1B	0.83	0.96	1.15	0.67
MAP2K1	MAP2K1;MAPKK1;MEK1;MKK1;PRKMK1	mitogen-activated protein kinase kinase 1	0.83	0.92	1.15	0.83
AGAP4	AGAP4;CTGLF1;MRIP2	ArfGAP with GTPase domain, ankyrin repeat and PH domain 4	0.83	0.94	1.15	0.75
ZNF30	ZNF30;DKFZp686N19164;FLJ20562;KOX28	zinc finger protein 30	0.82	0.86	1.15	0.68
MMP19	MMP19;MMP18;RASI-1	matrix metallopeptidase 19	0.82	0.94	1.15	0.71
TAF1A	TAF1A;MGC:17061;RAFI48;SL1;TAFI48	TATA box binding protein (TBP)-associated factor, RNA polymerase I, A, 48kDa	0.82	1.04	1.15	0.72
ACYP1	ACYP1;ACYPE	acylphosphatase 1, erythrocyte (common) type	0.82	0.88	1.15	0.78
GZF1	GZF1;ZBTB23;ZNF336	GDNF-inducible zinc finger protein 1	0.82	1.01	1.15	0.78
CHMP7	CHMP7;MGC29816	charged multivesicular body protein 7	0.82	0.97	1.15	0.69
PLLP	PLLP;PMLP;TM4SF11	plasmolipin	0.82	0.92	1.15	0.69
ZNF277	ZNF277;NRIF4;ZNF277P	zinc finger protein 277	0.82	0.9	1.15	0.72
LEPR	LEPR;CD295;DKFZp686B1731;LEP-R;OB-R;OBR	leptin receptor	0.82	0.92	1.15	0.83
RAB32	RAB32	RAB32, member RAS oncogene family	0.82	0.9	1.15	0.67
CCDC90A	CCDC90A;C6orf79;FLJ20958	coiled-coil domain containing 90A	0.82	0.88	1.15	0.72
CDC37	CDC37;P50CDC37	cell division cycle 37 homolog (S. cerevisiae)	0.82	0.94	1.15	0.66
SRRM4	SRRM4;KIAA1853;MGC176729;MU-MB-2.76;nSR100	serine/arginine repetitive matrix 4	0.82	0.94	1.15	0.64
WSCD2	WSCD2;KIAA0789;MGC117165	WSC domain containing 2	0.81	0.88	1.15	0.72
TRNT1	TRNT1;CCA1;MtCCA	tRNA nucleotidyl transferase, CCA-adding, 1	0.81	0.86	1.15	0.76
HDDC3	HDDC3;MESH1;MGC45386	HD domain containing 3	0.81	0.9	1.15	0.69
HRSP12	HRSP12;P14.5;PSP;UK114	heat-responsive protein 12	0.81	0.9	1.15	0.73
SLC7A11	SLC7A11;CCBR1;xCT	solute carrier family 7 (anionic amino acid transporter light chain, xc- system), member 11	0.81	0.92	1.15	0.65
KRT17	KRT17;K17;PC;PC2;PCHC1	keratin 17	0.81	0.88	1.15	0.74
SLFN11	SLFN11;FLJ34922;SLFN8/9	schlafen family member 11	0.8	0.87	1.15	0.63
IGSF11	IGSF11;BT-IgSF;CT119;CXADRL1;Igsf13;MGC35227;VSIG3	immunoglobulin superfamily, member 11	0.8	1.01	1.15	0.74
C3orf24	C3orf24;MGC40179	chromosome 3 open reading frame 24	0.8	0.92	1.15	0.66
MSRA	MSRA;PMSR	methionine sulfoxide reductase A	0.8	0.86	1.15	0.68
PLAT	PLAT;DKFZp686I03148;T-PA;TPA	plasminogen activator, tissue	0.8	0.91	1.15	0.68
B4GALNT3	B4GALNT3;FLJ16224;FLJ40362	beta-1,4-N-acetyl-galactosaminyl transferase 3	0.8	0.9	1.15	0.63
ABCA13	ABCA13;DKFZp313D2411;FLJ16398;FLJ33876;FLJ33951	ATP-binding cassette, sub-family A (ABC1), member 13	0.79	0.92	1.15	0.73
OCLN	OCLN	ENTREZ STATUS: REMAPPED TO GENE ID: 100506658	0.79	0.84	1.15	0.67
TMEM184B	TMEM184B;C22orf5;DKFZp586A1024;DKFZp686F07174;DKFZp686N1150;FM08;HS5O6A	transmembrane protein 184B	0.79	0.94	1.15	0.71
BPIFB6	BPIFB6;BPIL3;LPLUNC6	BPI fold containing family B, member 6	0.79	0.92	1.15	0.62
TM4SF4	TM4SF4;FLJ31015;ILTMP;il-TMP	transmembrane 4 L six family member 4	0.78	0.84	1.15	0.71
ASAH2	ASAH2;BCDase;HNAC1;LCDase;MGC129777;N-CDase;NCDase	N-acylsphingosine amidohydrolase (non-lysosomal ceramidase) 2	0.78	0.85	1.15	0.61
SPACA5	SPACA5;LYC5;LYZL5;SLLP2;SPACA5B;UNQ6288;dJ54B20.3	sperm acrosome associated 5	0.78	0.88	1.15	0.66
GFOD2	GFOD2;FLJ23802;FLJ39316;MGC11335	glucose-fructose oxidoreductase domain containing 2	0.78	0.86	1.15	0.75
TIMM9	TIMM9;TIM9;TIM9A	translocase of inner mitochondrial membrane 9 homolog (yeast)	0.78	0.93	1.15	0.64
SLC9A7	SLC9A7;NHE7;SLC9A6	solute carrier family 9 (sodium/hydrogen exchanger), member 7	0.78	0.88	1.15	0.66
PSRC1	PSRC1;DDA3;FP3214;MGC1780	proline/serine-rich coiled-coil 1	0.78	0.8	1.15	0.62
SLC35D1	SLC35D1;KIAA0260;MGC138236;UGTREL7	solute carrier family 35 (UDP-glucuronic acid/UDP-N-acetylgalactosamine dual transporter), member D1	0.78	0.98	1.15	0.68
ATP6AP2	ATP6AP2;APT6M8-9;ATP6IP2;ATP6M8-9;ELDF10;M8-9;MGC99577;MRXE;MSTP009;XMRE	ATPase, H+ transporting, lysosomal accessory protein 2	0.78	0.89	1.15	0.74
UBAP2L	UBAP2L;FLJ42300;KIAA0144;NICE-4	ubiquitin associated protein 2-like	0.78	0.96	1.15	0.74
SNX31	SNX31;MGC39715	sorting nexin 31	0.77	0.86	1.15	0.68
LILRB3	LILRB3;CD85A;HL9;ILT5;LIR-3;LIR3;MGC138403;PIRB	leukocyte immunoglobulin-like receptor, subfamily B (with TM and ITIM domains), member 3	0.77	0.84	1.15	0.71
KRT33A	KRT33A;HA3I;Ha-3I;KRTHA3A;Krt1-3;hHa3-I	keratin 33A	0.77	0.86	1.15	0.65
DIAPH2	DIAPH2;DIA;DIA2;DRF2;FLJ11167;POF;POF2	diaphanous homolog 2 (Drosophila)	0.77	0.86	1.15	0.7
MYCBP	MYCBP;AMY-1;FLJ41056	c-myc binding protein	0.77	0.89	1.15	0.69
CRLS1	CRLS1;C20orf155;CLS;CLS1;GCD10;dJ967N21.6	cardiolipin synthase 1	0.77	0.88	1.15	0.65
C20orf3	C20orf3;APMAP;BSCv	chromosome 20 open reading frame 3	0.77	0.8	1.15	0.64
ACADM	ACADM;ACAD1;FLJ18227;FLJ93013;FLJ99884;MCAD;MCADH	acyl-CoA dehydrogenase, C-4 to C-12 straight chain	0.77	0.86	1.15	0.64
MDS031	MDS031	ENTREZ STATUS: REMAPPED TO GENE ID: 79868	0.77	0.92	1.15	0.66
SULT1C2	SULT1C2;ST1C1;ST1C2;SULT1C1;humSULTC2	sulfotransferase family, cytosolic, 1C, member 2	0.76	0.82	1.15	0.65
NOL4	NOL4;CT125;NOLP	nucleolar protein 4	0.76	0.86	1.15	0.71
MKX	MKX;C10orf48;IFRX;IRXL1;MGC39616	mohawk homeobox	0.76	0.92	1.15	0.71
CYP2U1	CYP2U1;P450TEC	cytochrome P450, family 2, subfamily U, polypeptide 1	0.76	0.85	1.15	0.63
TFAP2E	TFAP2E;AP2E;MGC49007	transcription factor AP-2 epsilon (activating enhancer binding protein 2 epsilon)	0.76	0.87	1.15	0.63

UBE2L6	UBE2L6;MGC40331;RIG-B;UBCH8	ubiquitin-conjugating enzyme E2L 6	0.76	0.81	1.15	0.61
GYS1	GYS1;GSY;GYS	glycogen synthase 1 (muscle)	0.76	0.88	1.15	0.65
NF2	NF2;ACN;BANF;SCH	neurofibromin 2 (merlin)	0.76	0.83	1.15	0.7
COPA	COPA;FLJ26320;HEP-COP	coatomer protein complex, subunit alpha	0.76	0.91	1.15	0.69
C12orf24	C12orf24;HSU79274	chromosome 12 open reading frame 24	0.75	0.92	1.15	0.65
CLEC17A	CLEC17A;FLJ45910	C-type lectin domain family 17, member A	0.75	0.84	1.15	0.64
CD47	CD47;IAP;MER6;OA3	CD47 molecule	0.75	0.77	1.15	0.65
CNTRL	CNTRL;CEP1;CEP110;FAN;bA165P4.1	centriolin	0.75	0.84	1.15	0.73
ANO2	ANO2;C12orf3;DKFZp434P102;TMEM16B	anoctamin 2	0.74	0.8	1.15	0.68
RPUSD2	RPUSD2;C15orf19;C18B11;FLJ31409	RNA pseudouridylate synthase domain containing 2	0.74	0.86	1.15	0.58
NUDT12	NUDT12;DKFZp761I172	nudix (nucleoside diphosphate linked moiety X)-type motif 12	0.74	0.8	1.15	0.6
RRP15	RRP15;KIAA0507;MGC22291	ribosomal RNA processing 15 homolog (S. cerevisiae)	0.74	0.88	1.15	0.64
FDX1	FDX1;ADX;FDX;LOH11CR1D	ferredoxin 1	0.74	0.82	1.15	0.71
SMYD3	SMYD3;FLJ21080;KMT3E;MGC104324;ZMYND1;ZNFN3A1;bA74P14.1	SET and MYND domain containing 3	0.74	0.8	1.15	0.64
ETHE1	ETHE1;HSCO;YF13H12	ethylmalonic encephalopathy 1	0.74	0.87	1.15	0.66
MTRF1L	MTRF1L;HMRF1L;MGC102748;MRF1L;mtRF1a	mitochondrial translational release factor 1-like	0.74	0.86	1.15	0.66
ZCCHC12	ZCCHC12;FLJ16123;SIZN;SIZN1	zinc finger, CCHC domain containing 12	0.73	0.86	1.15	0.64
ANO1	ANO1;DOG1;FLJ10261;ORAOV2;TAOS2;TMEM16A	anoctamin 1, calcium activated chloride channel	0.73	0.82	1.15	0.66
PGLYRP4	PGLYRP4;PGLYRPIbeta;PGRP-Ibeta;PGRPIB	peptidoglycan recognition protein 4	0.73	0.82	1.15	0.6
SOX14	SOX14;MGC119898;MGC119899;SOX28	SRY (sex determining region Y)-box 14	0.73	0.86	1.15	0.59
BTN3A2	BTN3A2;BT3.2;BT3.3;BTF4;FLJ40011	butyrophilin, subfamily 3, member A2	0.73	0.8	1.15	0.61
ABTB2	ABTB2;DKFZp586C1619	ankyrin repeat and BTB (POZ) domain containing 2	0.73	0.82	1.15	0.61
LAT	LAT;LAT1;pp36	linker for activation of T cells	0.73	0.76	1.15	0.6
ATG2B	ATG2B;C14orf103;FLJ10242	ATG2 autophagy related 2 homolog B (S. cerevisiae)	0.73	0.83	1.15	0.69
SSBP3	SSBP3;CSDP;FLJ10355;SSDP;SSDP1	single stranded DNA binding protein 3	0.73	0.86	1.15	0.65
CT47A2	CT47A2;CT47.2;CT47A1;CT47A10;CT47A11;CT47A12;CT47A3;CT47A4;CT47A5;CT47A6;CT47A7;CT47A8;CT47A9	cancer/testis antigen family 47, member A2	0.73	0.88	1.15	0.62
TRMT112	TRMT112;HSPC152;HSPC170;TRM112;TRMT11-2	tRNA methyltransferase 11-2 homolog (S. cerevisiae)	0.73	0.76	1.15	0.63
ANGPTL7	ANGPTL7;AngX;CDT6;RP4-647M16.2;dJ647M16.1	angiopoietin-like 7	0.72	0.88	1.15	0.58
KY	KY;FLJ33207	kyphoscoliosis peptidase	0.72	0.9	1.15	0.62
COQ10A	COQ10A;FLJ32452	coenzyme Q10 homolog A (S. cerevisiae)	0.72	0.82	1.15	0.59
REEP1	REEP1;C2orf23;FLJ13110;SPG31	receptor accessory protein 1	0.72	0.88	1.15	0.67
UHRF1BP1L	UHRF1BP1L;KIAA0701	UHRF1 binding protein 1-like	0.72	0.79	1.15	0.6
HEY2	HEY2;CHF1;GRIDLOCK;GRL;HERP1;HESR2;HRT2;MGC10720;bHLHb32	hairy/enhancer-of-split related with YRPW motif 2	0.72	0.8	1.15	0.56
TMEM188	TMEM188;C16orf69;DKFZp313D2416;FLJ38101;TMP125	transmembrane protein 188	0.72	0.88	1.15	0.61
ASNSD1	ASNSD1;FLJ20752;NBLA00058;NS3TP1	asparagine synthetase domain containing 1	0.72	0.79	1.15	0.57
C14orf2	C14orf2;MP68;PLPM	chromosome 14 open reading frame 2	0.72	0.84	1.15	0.64
TMEM189-UB	TMEM189-UBE2V1;CROC-1B;CROC1B;KUA-UEV	TMEM189-UBE2V1 readthrough	0.72	0.76	1.15	0.57
HAUS7	HAUS7;HSXQ28ORF;UCHL5IP;UIP1	HAUS augmin-like complex, subunit 7	0.72	0.89	1.15	0.66
AVL9	AVL9;DKFZp686G0344;KIAA0241	AVL9 homolog (S. cerevisiase)	0.72	0.84	1.15	0.57
HLA-DRB5	HLA-DRB5;FLJ76359;HLA-DRB	major histocompatibility complex, class II, DR beta 5	0.71	0.79	1.15	0.57
CXCL14	CXCL14;BMAC;BRAK;KEC;KS1;MGC10687;MIP-2g;MIP2G;NJAC;SCYB14	chemokine (C-X-C motif) ligand 14	0.71	0.85	1.15	0.57
CGNL1	CGNL1;FLJ14957;JACOP;KIAA1749;MGC138254	cingulin-like 1	0.71	0.78	1.15	0.6
FKBP11	FKBP11;FKBP19;MGC54182	FK506 binding protein 11, 19 kDa	0.71	0.82	1.15	0.64
BCKDHA	BCKDHA;BCKDE1A;FLJ45695;MSU;MSUD1;OVD1A	branched chain keto acid dehydrogenase E1, alpha polypeptide	0.71	0.78	1.15	0.56
PAIP2	PAIP2;MGC72018;PAIP2A	poly(A) binding protein interacting protein 2	0.71	0.86	1.15	0.57
CADPS2	CADPS2;FLJ40851;KIAA1591	Ca++-dependent secretion activator 2	0.7	0.77	1.15	0.61
LDOC1	LDOC1;BCUR1;Mar7;Mart7	leucine zipper, down-regulated in cancer 1	0.7	0.84	1.15	0.56
ATG4C	ATG4C;APG4-C;APG4C;AUTL1;AUTL3;FLJ14867	ATG4 autophagy related 4 homolog C (S. cerevisiae)	0.7	0.8	1.15	0.59
CARHSP1	CARHSP1;CRHSP-24;CSDC1;MGC111446	calcium regulated heat stable protein 1, 24kDa	0.7	0.84	1.15	0.63
EVI5	EVI5;NB4S	ecotropic viral integration site 5	0.7	0.84	1.15	0.6
C1QL1	C1QL1;C1QRF;C1QTNF14;CRF	complement component 1, q subcomponent-like 1	0.7	0.82	1.15	0.55
ST3GAL1	ST3GAL1;DKFZp666E036;DKFZp779K2051;FLJ36548;Gal-NAc6S;MGC9183;SIAT4A;SIATFL;ST3GalA;ST3GalA.1;ST3GalIA;ST3GalIA,1;ST3O	ST3 beta-galactoside alpha-2,3-sialyltransferase 1	0.7	0.74	1.15	0.66
ERAL1	ERAL1;ERA;ERAL1A;H-ERA;HERA-A;HERA-B	Era G-protein-like 1 (E. coli)	0.7	0.78	1.15	0.55
RBM4	RBM4;DKFZp547K0918;FLJ36727;LARK;MGC75138;RBM4A;ZCCHC21;ZCRB3A	RNA binding motif protein 4	0.7	0.78	1.15	0.62
ADAMTS1	ADAMTS1;C3-C5;KIAA1346;METH1	ADAM metallopeptidase with thrombospondin type 1 motif, 1	0.7	0.74	1.15	0.56
MMGT1	MMGT1;TMEM32	membrane magnesium transporter 1	0.7	0.78	1.15	0.59
DTWD1	DTWD1;MGC111207	DTW domain containing 1	0.69	0.75	1.15	0.61
KIAA0100	KIAA0100;BCOX;BCOX1;CT101;DKFZp686M0843;MGC111488;MGC134981	KIAA0100	0.69	0.7	1.15	0.56
TAS2R4	TAS2R4;MGC163311;T2R4	taste receptor, type 2, member 4	0.68	0.77	1.15	0.58
IL19	IL19;IL-10C;MDA1;NG.1;ZMDA1	interleukin 19	0.68	0.77	1.15	0.62
PTPLAD2	PTPLAD2;DKFZp686F01145;DKFZp686G24132;HACD4	protein tyrosine phosphatase-like A domain containing 2	0.68	0.75	1.15	0.58
IL28RA	IL28RA;CRF2/12;IFNLR;IFNLR1;IL-28R1;LICR2	interleukin 28 receptor, alpha (interferon, lambda receptor)	0.68	0.76	1.15	0.66
RECQL5	RECQL5;FLJ90603;RECQ5	RecQ protein-like 5	0.68	0.73	1.15	0.65
ZNF618	ZNF618;FP13169;NEDD10	zinc finger protein 618	0.68	0.78	1.15	0.65
KIAA1919	KIAA1919;MGC33953;NaGLT1	KIAA1919	0.68	0.76	1.15	0.62
STK40	STK40;MGC4796;RP11-268J15.4;SHIK;SgK495	serine/threonine kinase 40	0.68	0.86	1.15	0.6
PLAU	PLAU;ATF;BDPLT5;QPD;UPA;URK;u-PA	plasminogen activator, urokinase	0.68	0.78	1.15	0.58
HRAS	HRAS;C-BAS/HAS;C-H-RAS;C-HA-RAS1;CTLO;H-RASIDX;HAMSV;HRAS1;K-RAS;N-RAS;RASH1	v-Ha-ras Harvey rat sarcoma viral oncogene homolog	0.68	0.75	1.15	0.59
CA6	CA6;CA-VI;GUSTIN;MGC21256	carbonic anhydrase VI	0.67	0.76	1.15	0.53
MPZL2	MPZL2;EVA;EVA1	myelin protein zero-like 2	0.67	0.8	1.15	0.6
CTNNA3	CTNNA3;MGC26194;MGC75041;VR22	catenin (cadherin-associated protein), alpha 3	0.66	0.74	1.15	0.61
CAB39L	CAB39L;FLJ12577;MO25-BETA;MO2L;RP11-103J18.3;bA103J18.3	calcium binding protein 39-like	0.66	0.8	1.15	0.64
KBTBD8	KBTBD8;FLJ57592;KIAA1842;TA-KRP	kelch repeat and BTB (POZ) domain containing 8	0.66	0.77	1.15	0.55
VMAC	VMAC;MGC131658	vimentin-type intermediate filament associated coiled-coil protein	0.66	0.82	1.15	0.63
TNFRSF4	TNFRSF4;ACT35;CD134;OX40;TXGP1L	tumor necrosis factor receptor superfamily, member 4	0.66	0.74	1.15	0.57
APOA1	APOA1;MGC117399	apolipoprotein A-I	0.66	0.82	1.15	0.57
CDKN2D	CDKN2D;INK4D;p19;p19-INK4D	cyclin-dependent kinase inhibitor 2D (p19, inhibits CDK4)	0.66	0.83	1.15	0.59
EPS15L1	EPS15L1;EPS15R	epidermal growth factor receptor pathway substrate 15-like 1	0.66	0.78	1.15	0.54
RGS10	RGS10	regulator of G-protein signaling 10	0.66	0.83	1.15	0.61
C5orf32	C5orf32	chromosome 5 open reading frame 32	0.66	0.75	1.15	0.56
C3	C3;AHUS5;ARMD9;ASP;CPAMD1	complement component 3	0.66	0.79	1.15	0.59
TSTD2	TSTD2;C9orf97;FLJ36724	thiosulfate sulfurtransferase (rhodanese)-like domain containing 2	0.66	0.77	1.15	0.59
LW-1	LW-1	ENTREZ STATUS: REMAPPED TO GENE ID: 100506164	0.66	0.78	1.15	0.56
ADAMTS2	ADAMTS2;ADAM-TS2;ADAMTS-2;ADAMTS-3;DKFZp686F12218;NPI;PC I-NP;PCI-NP;PCINP;PCPNI;PNPI	ADAM metallopeptidase with thrombospondin type 1 motif, 2	0.65	0.69	1.15	0.52
TOMM40L	TOMM40L;FLJ12770;RP11-297K8.10;TOMM40B	translocase of outer mitochondrial membrane 40 homolog (yeast)-like	0.65	0.8	1.15	0.56
RANBP17	RANBP17;FLJ32916	RAN binding protein 17	0.65	0.78	1.15	0.58
ZNF521	ZNF521;DKFZp564D0764;EHZF;Evi3;MGC142182;MGC142208	zinc finger protein 521	0.64	0.75	1.15	0.51
C7orf63	C7orf63;FLJ21062	chromosome 7 open reading frame 63	0.64	0.76	1.15	0.52
LPAL2	LPAL2;APOA2;APOAL;APOARGC;MGC129532;RP11-72O9.2	lipoprotein, Lp(a)-like 2, pseudogene	0.64	0.7	1.15	0.55
SPOCK1	SPOCK1;FLJ37170;SPOCK;TESTICAN;TIC1	sparc/osteonectin, cwcv and kazal-like domains proteoglycan (testican) 1	0.64	0.76	1.15	0.55
GMFG	GMFG;GMF-GAMMA;MGC126867	glia maturation factor, gamma	0.64	0.67	1.15	0.53
PILRA	PILRA;FDF03	paired immunoglobin-like type 2 receptor alpha	0.64	0.75	1.15	0.54
HIST1H2BG	HIST1H2BG;H2B.1A;H2B/a;H2B/g;H2B/h;H2B/k;H2B/l;H2BFA;dJ221C16.8	histone cluster 1, H2bg	0.64	0.76	1.15	0.51
C4orf27	C4orf27;FLJ20534;FLJ33423;FLJ42042	chromosome 4 open reading frame 27	0.64	0.8	1.15	0.61
PRMT7	PRMT7;FLJ10640;KIAA1933	protein arginine methyltransferase 7	0.64	0.82	1.15	0.61
MATN2	MATN2	matrilin 2	0.64	0.72	1.15	0.56
RARS2	RARS2;ArgRS;DALRD2;MGC14993;MGC23778;PCH6;PRO1992;RARSL;dJ382I10.6	arginyl-tRNA synthetase 2, mitochondrial	0.64	0.8	1.15	0.55
ILF2	ILF2;MGC8391;NF45;PRO3063	interleukin enhancer binding factor 2, 45kDa	0.64	0.78	1.15	0.52
TULP2	TULP2;CT65;TUBL2	tubby like protein 2	0.63	0.78	1.15	0.59
FLCN	FLCN;BHD;DKFZp547A118;FLCL;FLJ45004;FLJ99377;MGC17998;MGC23445	folliculin	0.63	0.72	1.15	0.52
BRK1	BRK1;C3orf10;MDS027;hHBrk1	BRICK1, SCAR/WAVE actin-nucleating complex subunit	0.63	0.79	1.15	0.58
OR9Q2	OR9Q2;OR9Q2P	olfactory receptor, family 9, subfamily Q, member 2	0.62	0.66	1.15	0.56
TTYH2	TTYH2;C17orf29;MGC126172;MGC4837	tweety homolog 2 (Drosophila)	0.62	0.69	1.15	0.52
LRRC56	LRRC56;DKFZp761L1518;FLJ00101	leucine rich repeat containing 56	0.62	0.73	1.15	0.52
PSG7	PSG7;PSBG-7;PSG1;PSGGA	pregnancy specific beta-1-glycoprotein 7 (gene/pseudogene)	0.62	0.64	1.15	0.54
CLEC3B	CLEC3B;DKFZp686H17246;TN;TNA	C-type lectin domain family 3, member B	0.62	0.68	1.15	0.52
PNKD	PNKD;BRP17;DKFZp564N1362;DYT8;FPD1;KIAA1184;KIPP1184;MGC31943;MR-1;MR1;PDC;PKND1;TAHCCP2	paroxysmal nonkinesigenic dyskinesia	0.62	0.72	1.15	0.57
AK2	AK2;ADK2;AK 2	adenylate kinase 2	0.62	0.7	1.15	0.63
CEP89	CEP89;CCDC123;FLJ14640	centrosomal protein 89kDa	0.62	0.74	1.15	0.54
PRDM10	PRDM10;KIAA1231;MGC131802;PFM7	PR domain containing 10	0.61	0.68	1.15	0.49
CHAC1	CHAC1;MGC4504	ChaC, cation transport regulator homolog 1 (E. coli)	0.61	0.74	1.15	0.53
OR8B12	OR8B12;OR11-317	olfactory receptor, family 8, subfamily B, member 12	0.84	0.99	1.16	0.68
WASF1	WASF1;FLJ31482;KIAA0269;SCAR1;WAVE;WAVE1	WAS protein family, member 1	0.84	0.98	1.16	0.68
SENP8	SENP8;DEN1;NEDP1;PRSC2	SUMO/sentrin specific peptidase family member 8	0.84	0.96	1.16	0.72
KIAA0895	KIAA0895	KIAA0895	0.84	0.98	1.16	0.66
FMR1	FMR1;FMRP;FRAXA;MGC87458;POF;POF1	fragile X mental retardation 1	0.84	0.91	1.16	0.77
ANO6	ANO6;BDPLT7;MGC104751;SCTS;TMEM16F	anoctamin 6	0.84	1.01	1.16	0.68
CCDC101	CCDC101;FLJ32446;SGF29;STAF36	coiled-coil domain containing 101	0.84	0.86	1.16	0.67
CNOT10	CNOT10;DKFZp434K115;FLJ12890;FLJ13165	CCR4-NOT transcription complex, subunit 10	0.84	1	1.16	0.76
ME1	ME1;HUMNDME;MES	malic enzyme 1, NADP(+)-dependent, cytosolic	0.84	1.02	1.16	0.68
LIMK2	LIMK2	LIM domain kinase 2	0.84	1.04	1.16	0.74
ATP6V1H	ATP6V1H;MSTP042;NBP1;SFD;SFDalpha;SFDbeta;VMA13	ATPase, H+ transporting, lysosomal 50/57kDa, V1 subunit H	0.84	0.94	1.16	0.73
IFIT1	IFIT1;C56;G10P1;GARG-16;IFI-56;IFI-56K;IFI56;IFIT-1;IFNAI1;ISG56;RNM561	interferon-induced protein with tetratricopeptide repeats 1	0.83	0.92	1.16	0.73
NPRL2	NPRL2;NPR2;NPR2L;TUSC4	nitrogen permease regulator-like 2 (S. cerevisiae)	0.83	0.94	1.16	0.79
OR13A1	OR13A1;FLJ16797	olfactory receptor, family 13, subfamily A, member 1	0.82	0.93	1.16	0.7
OR7G1	OR7G1;OR19-15;OR19-8;OR7G1P	olfactory receptor, family 7, subfamily G, member 1	0.82	1	1.16	0.74
MOGAT2	MOGAT2;DGAT2L5;FLJ22644;MGAT2;MGC119183;MGC119184;MGC119185	monoacylglycerol O-acyltransferase 2	0.82	0.88	1.16	0.77
ZNF555	ZNF555;FLJ41894;MGC26707	zinc finger protein 555	0.82	0.93	1.16	0.77
C7orf29	C7orf29	chromosome 7 open reading frame 29	0.82	0.92	1.16	0.73

ST6GALNAC4	ST6GALNAC4;SIAT3C;SIAT7D;ST6GALNACIV	ST6 (alpha-N-acetyl-neuraminyl-2,3-beta-galactosyl-1,3)-N-acetylgalactosaminide alpha-2,6-sialyltransferase 4	0.82	0.92	1.16	0.66
KEAP1	KEAP1;INrf2;KIAA0132;KLHL19;MGC10630;MGC1114;MGC20887;MGC4407;MGC9454	kelch-like ECH-associated protein 1	0.82	0.91	1.16	0.66
HAS2	HAS2;MGC126241;MGC126242	hyaluronan synthase 2	0.81	0.9	1.16	0.65
APLP1	APLP1;APLP	amyloid beta (A4) precursor-like protein 1	0.81	0.98	1.16	0.73
ADAR	ADAR;ADAR1;DRADA;DSH;DSRAD;G1P1;IFI-4;IFI4;K88DSRBP;P136	adenosine deaminase, RNA-specific	0.81	0.93	1.16	0.69
OR2A5	OR2A5;OR2A11P;OR2A26;OR2A8;OR7-138;OR7-141	olfactory receptor, family 2, subfamily A, member 5	0.8	0.84	1.16	0.67
TNFSF11	TNFSF11;CD254;ODF;OPGL;OPTB2;RANKL;TRANCE;hRANKL2;sOdf	tumor necrosis factor (ligand) superfamily, member 11	0.8	0.96	1.16	0.73
STK32A	STK32A;MGC22688;YANK1	serine/threonine kinase 32A	0.8	0.99	1.16	0.7
KIT	KIT;C-Kit;CD117;PBT;SCFR	v-kit Hardy-Zuckerman 4 feline sarcoma viral oncogene homolog	0.8	0.92	1.16	0.76
LZTFL1	LZTFL1;FLJ36386	leucine zipper transcription factor-like 1	0.8	0.96	1.16	0.65
KIR3DL3	KIR3DL3;CD158Z;KIR3DL7;KIR44;KIRC1	killer cell immunoglobulin-like receptor, three domains, long cytoplasmic tail, 3	0.8	0.88	1.16	0.64
CETN3	CETN3;CEN3;MGC12502;MGC138245	centrin, EF-hand protein, 3	0.8	0.9	1.16	0.71
GPR78	GPR78	G protein-coupled receptor 78	0.8	0.86	1.16	0.65
CAPN5	CAPN5;FLJ46245;HTRA3;nCL-3	calpain 5	0.8	0.86	1.16	0.64
MAP3K5	MAP3K5;ASK1;MAPKKK5;MEKK5	mitogen-activated protein kinase kinase kinase 5	0.8	0.92	1.16	0.76
KLHL8	KLHL8;FLJ46304;KIAA1378	kelch-like 8 (Drosophila)	0.8	0.88	1.16	0.74
CRIP2	CRIP2;CRIP;CRP2;ESP1	cysteine-rich protein 2	0.8	0.93	1.16	0.65
ARF1	ARF1	ADP-ribosylation factor 1	0.8	1.03	1.16	0.71
MGC50722	MGC50722	uncharacterized MGC50722	0.8	1.01	1.16	0.74
FAM151B	FAM151B;UNQ9217	family with sequence similarity 151, member B	0.79	0.9	1.16	0.68
LGR6	LGR6;FLJ14471;GPCR;VTS20631	leucine-rich repeat containing G protein-coupled receptor 6	0.79	0.88	1.16	0.79
CRIP3	CRIP3;TLP;TLP-A;bA480N24.2	cysteine-rich protein 3	0.79	0.94	1.16	0.7
FAM76B	FAM76B;MGC33371	family with sequence similarity 76, member B	0.79	0.87	1.16	0.71
DGCR8	DGCR8;C22orf12;DGCRK6;Gy1;pasha	DiGeorge syndrome critical region gene 8	0.79	0.9	1.16	0.67
SPHAR	SPHAR;MGC88280;RP4-803J11.4	S-phase response (cyclin related)	0.79	0.97	1.16	0.78
SLC18A2	SLC18A2;MGC120477;MGC120478;MGC26538;SVAT;SVMT;VAT2;VMAT2	solute carrier family 18 (vesicular monoamine), member 2	0.78	0.92	1.16	0.71
SLC12A3	SLC12A3;FLJ96318;NCCT;TSC	solute carrier family 12 (sodium/chloride transporters), member 3	0.78	0.84	1.16	0.74
GANAB	GANAB;G2AN;GLUII;KIAA0088	glucosidase, alpha; neutral AB	0.78	0.9	1.16	0.7
CBX5	CBX5;HP1;HP1A	chromobox homolog 5	0.78	0.86	1.16	0.66
PAX4	PAX4;KPD;MGC129960;MODY9	paired box 4	0.77	0.86	1.16	0.67
GIPC3	GIPC3;C19orf64;DFNB15;DFNB72;DFNB95;DKFZp686J1198;FLJ40925	GIPC PDZ domain containing family, member 3	0.77	0.81	1.16	0.64
VILL	VILL	villin-like	0.77	0.93	1.16	0.62
KRBA1	KRBA1;KIAA1862;MGC176633	KRAB-A domain containing 1	0.77	0.92	1.16	0.68
CST11	CST11;CST8L;SC13;dJ322G13.6	cystatin 11	0.76	0.78	1.16	0.63
CACNA1D	CACNA1D;CACH3;CACN4;CACNL1A2;CCHL1A2;Cav1.3	calcium channel, voltage-dependent, L type, alpha 1D subunit	0.76	0.92	1.16	0.71
CACNG6	CACNG6	calcium channel, voltage-dependent, gamma subunit 6	0.76	0.92	1.16	0.71
EXOC7	EXOC7;2-5-3p;DKFZp686J04253;EX070;EXO70;EXOC1;Exo70p;FLJ40965;FLJ46415;YJL085W	exocyst complex component 7	0.76	0.86	1.16	0.67
ZXDC	ZXDC;DKFZp547N024;FLJ13861;FLJ36477;FLJ37830;MGC11349;ZXDL	ZXD family zinc finger C	0.76	0.86	1.16	0.64
ATP2B1	ATP2B1;PMCA1;PMCA1kb	ATPase, Ca++ transporting, plasma membrane 1	0.76	0.9	1.16	0.6
SMAP2	SMAP2;RP1-228H13.3;SMAP1L	small ArfGAP2	0.76	0.85	1.16	0.65
PRR13	PRR13;DKFZp564J157;FLJ23818;FLJ58476;TXR1	proline rich 13	0.76	0.96	1.16	0.63
NCKAP1	NCKAP1;FLJ11291;HEM2;KIAA0587;MGC8981;NAP1;NAP125	NCK-associated protein 1	0.76	0.91	1.16	0.69
PCDHGA4	PCDHGA4;PCDH-GAMMA-A4	protocadherin gamma subfamily A, 4	0.76	0.8	1.16	0.66
KLK8	KLK8;HNP;NP;NRPN;PRSS19;TADG14	kallikrein-related peptidase 8	0.75	0.86	1.16	0.64
ORAI3	ORAI3;MGC13024;TMEM142C	ORAI calcium release-activated calcium modulator 3	0.75	0.79	1.16	0.61
C6orf48	C6orf48;D6S57;G8	chromosome 6 open reading frame 48	0.75	0.84	1.16	0.61
FBXO27	FBXO27;FBG5;Fbx27	F-box protein 27	0.75	0.84	1.16	0.61
IFITM3	IFITM3;1-8U;IP15	interferon induced transmembrane protein 3	0.75	0.85	1.16	0.64
HSD17B3	HSD17B3;EDH17B3;SDR12C2	hydroxysteroid (17-beta) dehydrogenase 3	0.74	0.94	1.16	0.64
KIRREL2	KIRREL2;DKFZp564A1164;FILTRIN;MGC15718;NEPH3;NLG1	kin of IRRE like 2 (Drosophila)	0.74	0.88	1.16	0.67
ETV4	ETV4;E1A-F;E1AF;PEA3;PEAS3	ets variant 4	0.74	0.8	1.16	0.71
GPR6	GPR6	G protein-coupled receptor 6	0.74	0.84	1.16	0.75
SLC39A4	SLC39A4;AEZ;FLJ20327;MGC74741;ZIP4	solute carrier family 39 (zinc transporter), member 4	0.74	0.81	1.16	0.64
KRT13	KRT13;CK13;K13;MGC161462;MGC3781	keratin 13	0.74	0.88	1.16	0.59
AHNAK	AHNAK;AHNAKRS;MGC5395	AHNAK nucleoprotein	0.74	0.83	1.16	0.64
C7orf49	C7orf49;FLJ22450;FLJ27285;MGC5242;MRI	chromosome 7 open reading frame 49	0.74	0.82	1.16	0.66
PHACTR4	PHACTR4;DKFZp686L07205;FLJ13171;MGC20618;MGC34186	phosphatase and actin regulator 4	0.74	0.88	1.16	0.59
NAMPT	NAMPT;1110035O14Rik;DKFZp666B131;MGC117256;PBEF;PBEF1;VF;VISFATIN	nicotinamide phosphoribosyltransferase	0.74	0.86	1.16	0.64
DQX1	DQX1;FLJ23757	DEAQ box RNA-dependent ATPase 1	0.73	0.89	1.16	0.72
WASF3	WASF3;Brush-1;KIAA0900;SCAR3;WAVE3	WAS protein family, member 3	0.73	0.86	1.16	0.62
ZNF777	ZNF777;KIAA1285	zinc finger protein 777	0.73	0.82	1.16	0.62
CST9	CST9;CLM	cystatin 9 (testatin)	0.72	0.75	1.16	0.59
SNAI3	SNAI3;MGC129606;SMUC;SNAIL3;ZNF293;Zfp293	snail homolog 3 (Drosophila)	0.72	0.76	1.16	0.63
ALDH3B1	ALDH3B1;ALDH4;ALDH7;FLJ26433;FLJ34710	aldehyde dehydrogenase 3 family, member B1	0.72	0.78	1.16	0.62
NDUFA12	NDUFA12;B17.2;DAP13	NADH dehydrogenase (ubiquinone) 1 alpha subcomplex, 12	0.72	0.91	1.16	0.67
ATP5J2	ATP5J2;ATP5JL	ATP synthase, H+ transporting, mitochondrial Fo complex, subunit F2	0.72	0.8	1.16	0.63
GPR19	GPR19	G protein-coupled receptor 19	0.71	0.8	1.16	0.72
PDZD7	PDZD7;PDZK7	PDZ domain containing 7	0.71	0.74	1.16	0.57
INSM2	INSM2;IA-6;IA6;mlt1	insulinoma-associated 2	0.71	0.78	1.16	0.57
RORC	RORC;MGC129539;NR1F3;RORG;RZR-GAMMA;RZRG;TOR	RAR-related orphan receptor C	0.71	0.84	1.16	0.66
ESPNL	ESPNL;FLJ42568;MGC99829	espin-like	0.71	0.78	1.16	0.63
TMEM177	TMEM177;MGC10993	transmembrane protein 177	0.71	0.8	1.16	0.6
RASL10B	RASL10B;MGC47540;RRP17;VTS58635	RAS-like, family 10, member B	0.71	0.86	1.16	0.7
IRAK3	IRAK3;ASRT5;FLJ13601;IRAKM	interleukin-1 receptor-associated kinase 3	0.7	0.88	1.16	0.62
C2orf47	C2orf47;DKFZp666A212;FLJ22555	chromosome 2 open reading frame 47	0.7	0.8	1.16	0.6
ZNF271	ZNF271;HZF7;ZNFEB	zinc finger protein 271	0.7	0.89	1.16	0.65
AP4B1	AP4B1;BETA-4;CPSQ5	adaptor-related protein complex 4, beta 1 subunit	0.7	0.86	1.16	0.7
HIST1H3F	HIST1H3F;H3/i;H3FI	histone cluster 1, H3f	0.7	0.8	1.16	0.67
C5orf56	C5orf56;FLJ21568;FLJ26121	chromosome 5 open reading frame 56	0.7	0.79	1.16	0.64
LAMA4	LAMA4;DKFZp686D23145;LAMA3;LAMA4*-1	laminin, alpha 4	0.69	0.77	1.16	0.55
CDHR1	CDHR1;CORD15;DKFZp434A132;KIAA1775;PCDH21;PRCAD	cadherin-related family member 1	0.69	0.82	1.16	0.56
DKK2	DKK2;DKK-2	dickkopf homolog 2 (Xenopus laevis)	0.68	0.84	1.16	0.68
ART1	ART1;ART2;CD296;MGC133217;RT6	ADP-ribosyltransferase 1	0.68	0.79	1.16	0.61
FOXF2	FOXF2;FKHL6;FREAC2	forkhead box F2	0.68	0.81	1.16	0.54
PI3	PI3;ESI;MGC13613;SKALP;WAP3;WFDC14;cementoin	peptidase inhibitor 3, skin-derived	0.68	0.78	1.16	0.54
KRT5	KRT5;CK5;DDD;EBS2;K5;KRT5A	keratin 5	0.68	0.75	1.16	0.58
ST5	ST5;DENND2B;HTS1;MGC33090;p126	suppression of tumorigenicity 5	0.68	0.74	1.16	0.55
EARS2	EARS2;KIAA1970;MSE1	glutamyl-tRNA synthetase 2, mitochondrial (putative)	0.68	0.78	1.16	0.57
LRWD1	LRWD1;CENP-33;DKFZp434K1815;ORCA	leucine-rich repeats and WD repeat domain containing 1	0.68	0.72	1.16	0.57
C11orf82	C11orf82;FLJ13936;FLJ25416;FLJ38838;noxin	chromosome 11 open reading frame 82	0.68	0.72	1.16	0.6
CCNL1	CCNL1;ANIA6A;PRO1073;ania-6a	cyclin L1	0.68	0.76	1.16	0.55
JRKL	JRKL;HHMJG;MGC129608	jerky homolog-like (mouse)	0.68	0.77	1.16	0.61
HNRNPH2	HNRNPH2;FTP3;HNRPH';HNRPH2;hnRNPH'	heterogeneous nuclear ribonucleoprotein H2 (H')	0.68	0.82	1.16	0.62
CLK3	CLK3;FLJ22858;PHCLK3;PHCLK3/152	CDC-like kinase 3	0.67	0.84	1.16	0.6
AP1S1	AP1S1;AP19;CLAPS1;FLJ92436;SIGMA1A;WUGSC:H_DJ0747G18.2	adaptor-related protein complex 1, sigma 1 subunit	0.67	0.8	1.16	0.55
CANX	CANX;CNX;FLJ26570;IP90;P90	calnexin	0.67	0.7	1.16	0.58
GPR111	GPR111;PGR20;hGPCR35	G protein-coupled receptor 111	0.66	0.74	1.16	0.67
SLC4A4	SLC4A4;DKFZp781H1314;HNBC1;KNBC;NBC1;NBC2;SLC4A5;hhNMC;pNBC	solute carrier family 4, sodium bicarbonate cotransporter, member 4	0.66	0.85	1.16	0.59
PRRX2	PRRX2;MGC19843;PMX2;PRX2	paired related homeobox 2	0.66	0.73	1.16	0.57
HTRA2	HTRA2;OMI;PARK13;PRSS25	HtrA serine peptidase 2	0.66	0.77	1.16	0.57
SLC37A1	SLC37A1;FLJ22340;G3PP	solute carrier family 37 (glycerol-3-phosphate transporter), member 1	0.66	0.84	1.16	0.62
FLOT2	FLOT2;ECS-1;ECS1;ESA;ESA1;M17S1	flotillin 2	0.66	0.8	1.16	0.59
CPSF6	CPSF6;CFIM;CFIM68;HPBRII-4;HPBRII-7	cleavage and polyadenylation specific factor 6, 68kDa	0.66	0.79	1.16	0.53
FLJ44635	FLJ44635	TPT1-like protein	0.65	0.74	1.16	0.59
ACOT1	ACOT1;ACH2;CTE-1;LACH2	acyl-CoA thioesterase 1	0.65	0.74	1.16	0.54
DNTTIP2	DNTTIP2;ERBP;FCF2;FLJ98849;HSU15552;LPTS-RP2;MGC163494;TdIF2	deoxynucleotidyltransferase, terminal, interacting protein 2	0.65	0.73	1.16	0.55
C20orf46	C20orf46;FLJ11190	chromosome 20 open reading frame 46	0.64	0.76	1.16	0.51
PASD1	PASD1;CT63;OXTES1	PAS domain containing 1	0.64	0.72	1.16	0.55
TGM6	TGM6;SCA35;TG6;TGM3L;TGY;dJ734P14.3	transglutaminase 6	0.64	0.74	1.16	0.49
RUFY4	RUFY4;FLJ46536	RUN and FYVE domain containing 4	0.64	0.8	1.16	0.54
C1orf109	C1orf109;FLJ20508	chromosome 1 open reading frame 109	0.64	0.78	1.16	0.61
EIF1B	EIF1B;GC20	eukaryotic translation initiation factor 1B	0.64	0.7	1.16	0.62
VAMP2	VAMP2;FLJ11460;SYB2;VAMP-2	vesicle-associated membrane protein 2 (synaptobrevin 2)	0.64	0.74	1.16	0.51
C6orf47	C6orf47;D6S53E;G4;NG34	chromosome 6 open reading frame 47	0.64	0.68	1.16	0.57
LAGE3	LAGE3;CVG5;DXS9879E;DXS9951E;ESO3;ITBA2	L antigen family, member 3	0.64	0.76	1.16	0.56
SAMD10	SAMD10;C20orf136;dJ591C20;dJ591C20.7	sterile alpha motif domain containing 10	0.64	0.75	1.16	0.56
SP4	SP4;HF1B;MGC130008;MGC130009;SPR-1	Sp4 transcription factor	0.64	0.7	1.16	0.55
EPHX1	EPHX1;EPHX;EPOX;HYL1;MEH	epoxide hydrolase 1, microsomal (xenobiotic)	0.64	0.71	1.16	0.61
MEA1	MEA1;HYS;MEA	male-enhanced antigen 1	0.64	0.77	1.16	0.57
C2orf29	C2orf29	chromosome 2 open reading frame 29	0.64	0.76	1.16	0.52
TM4SF1	TM4SF1;H-L6;L6;M3S1;TAAL6	transmembrane 4 L six family member 1	0.64	0.74	1.16	0.55
OXA1L	OXA1L;MGC133129;OXA1	oxidase (cytochrome c) assembly 1-like	0.64	0.68	1.16	0.54
C17orf102	C17orf102;FLJ44815	chromosome 17 open reading frame 102	0.64	0.74	1.16	0.61
SUGP1	SUGP1;DKFZp434E2216;F23858;RBP;SF4	SURP and G patch domain containing 1	0.64	0.75	1.16	0.51
PNMAL2	PNMAL2;FLJ42730;KIAA1183	PNMA-like 2	0.64	0.74	1.16	0.62
SLITRK1	SLITRK1;FLJ54428;KIAA0918;KIAA1910;LRRC12;RP11-395N17.1;TTM	SLIT and NTRK-like family, member 1	0.63	0.76	1.16	0.52
SLAMF7	SLAMF7;19A;CD319;CRACC;CS1	SLAM family member 7	0.63	0.75	1.16	0.54
PEG3	PEG3;DKFZp781A095;KIAA0287;PW1;ZNF904;ZSCAN24	paternally expressed 3	0.63	0.73	1.16	0.49
E2F3	E2F3;DKFZp686C18211;E2F-3;KIAA0075;MGC104598	E2F transcription factor 3	0.63	0.7	1.16	0.52

PES1	PES1;FLJ43790;PES	pescadillo homolog 1, containing BRCT domain (zebrafish)	0.63	0.76	1.16	0.57
IRF8	IRF8;H-ICSBP;ICSBP;ICSBP1;IRF-8	interferon regulatory factor 8	0.62	0.7	1.16	0.6
SRMS	SRMS;C20orf148;SRM;dJ697K14.1	src-related kinase lacking C-terminal regulatory tyrosine and N-terminal myristylation sites	0.62	0.76	1.16	0.54
RREB1	RREB1;FINB;HNT;LZ321;RREB-1;Zep-1	ras responsive element binding protein 1	0.62	0.7	1.16	0.49
BACE1	BACE1;ASP2;BACE;FLJ90568;HSPC104;KIAA1149	beta-site APP-cleaving enzyme 1	0.62	0.72	1.16	0.53
KIFC3	KIFC3;DKFZp686D23201;FLJ34694	kinesin family member C3	0.62	0.7	1.16	0.52
MRPS17	MRPS17;HSPC011;MRP-S17;RPMS17;S17mt	mitochondrial ribosomal protein S17	0.62	0.75	1.16	0.57
PKP4	PKP4;FLJ31261;FLJ42243;p0071	plakophilin 4	0.62	0.69	1.16	0.5
AURKAIP1	AURKAIP1;AIP;AKIP;FLJ20608	aurora kinase A interacting protein 1	0.62	0.7	1.16	0.55
NQO1	NQO1;DHQU;DIA4;DTD;NMOR1;NMORI;QR1	NAD(P)H dehydrogenase, quinone 1	0.62	0.7	1.16	0.52
C11orf42	C11orf42;MGC34805	chromosome 11 open reading frame 42	0.61	0.7	1.16	0.51
CTTNBP2	CTTNBP2;C7orf8;CORTBP2;FLJ34229;KIAA1758;MGC104579;Orf4	cortactin binding protein 2	0.61	0.69	1.16	0.56
ILK-2	ILK-2	ENTREZ STATUS: REMAPPED TO GENE ID: 3611	0.61	0.77	1.16	0.54
ZBTB32	ZBTB32;FAXF;FAZF;Rog;TZFP;ZNF538	zinc finger and BTB domain containing 32	0.84	1.05	1.17	0.69
CD74	CD74;DHLAG;FLJ98970;HLADG;II;Ia-GAMMA	CD74 molecule, major histocompatibility complex, class II invariant chain	0.84	0.95	1.17	0.71
DDX39A	DDX39A;BAT1;BAT1L;DDX39;DDXL;MGC18203;MGC8417;URH49	DEAD (Asp-Glu-Ala-Asp) box polypeptide 39A	0.84	1.04	1.17	0.69
FAM78B	FAM78B;MGC131653	family with sequence similarity 78, member B	0.83	0.92	1.17	0.75
LCORL	LCORL;FLJ30696;MLR1	ligand dependent nuclear receptor corepressor-like	0.83	0.94	1.17	0.67
XYLT2	XYLT2;PXYLT2;XT-II;XT2;xylT-II	xylosyltransferase II	0.83	0.98	1.17	0.76
ACOX3	ACOX3	acyl-CoA oxidase 3, pristanoyl	0.83	0.97	1.17	0.76
KCNN1	KCNN1;KCa2.1;SK1;SKCA1;hSK1	potassium intermediate/small conductance calcium-activated channel, subfamily N, member 1	0.82	0.96	1.17	0.75
OCA2	OCA2;BEY;BEY1;BEY2;BOCA;D15S12;EYCL;EYCL2;EYCL3;HCL3;P;PED;SHEP1	oculocutaneous albinism II	0.82	1.02	1.17	0.67
TBC1D26	TBC1D26;FLJ55788;MGC51025	TBC1 domain family, member 26	0.82	0.99	1.17	0.74
L3MBTL3	L3MBTL3;MBT-1;MBT1;RP11-73O6.1	l(3)mbt-like 3 (Drosophila)	0.82	0.9	1.17	0.76
ZNF696	ZNF696;FLJ14129	zinc finger protein 696	0.82	1	1.17	0.81
KATNA1	KATNA1	katanin p60 (ATPase containing) subunit A 1	0.82	0.92	1.17	0.69
SESN2	SESN2;DKFZp761M0212;DKFZp761M02121;HI95;SES2;SEST2	sestrin 2	0.82	1.01	1.17	0.82
SFXN3	SFXN3;BA108L7.2;SFX3	sideroflexin 3	0.82	0.94	1.17	0.71
UBE2Q2	UBE2Q2;DKFZp762C143	ubiquitin-conjugating enzyme E2Q family member 2	0.82	0.87	1.17	0.66
MBTPS2	MBTPS2;FLJ32174;IFAP;KFSDX;S2P	membrane-bound transcription factor peptidase, site 2	0.82	0.94	1.17	0.71
WDR77	WDR77;MEP-50;MEP50;MGC2722;Nbla10071;RP11-552M11.3;p44;p44/Mep50	WD repeat domain 77	0.82	0.86	1.17	0.67
TMEM165	TMEM165;TMPT27;TPARL	transmembrane protein 165	0.82	1	1.17	0.71
PRMT10	PRMT10;FLJ46629	protein arginine methyltransferase 10 (putative)	0.82	0.86	1.17	0.69
C9orf72	C9orf72;MGC23980;RP11-27J8.2	chromosome 9 open reading frame 72	0.81	0.94	1.17	0.78
MFAP4	MFAP4	microfibrillar-associated protein 4	0.8	0.92	1.17	0.69
CA7	CA7;CAVII	carbonic anhydrase VII	0.8	0.92	1.17	0.68
CAMK2B	CAMK2B;CAM2;CAMK2;CAMKB;MGC29528	calcium/calmodulin-dependent protein kinase II beta	0.8	0.94	1.17	0.77
TMEM39B	TMEM39B;FLJ10315	transmembrane protein 39B	0.8	0.97	1.17	0.72
UNG	UNG;DGU;DKFZp781L1143;HIGM4;UDG;UNG1;UNG15;UNG2	uracil-DNA glycosylase	0.8	0.89	1.17	0.64
PMPCA	PMPCA;Alpha-MPP;FLJ26258;INPP5E;KIAA0123;MGC104197	peptidase (mitochondrial processing) alpha	0.8	0.94	1.17	0.75
PAWR	PAWR;PAR4;Par-4	PRKC, apoptosis, WT1, regulator	0.8	0.92	1.17	0.65
ITPK1	ITPK1;ITRPK1	inositol-tetrakisphosphate 1-kinase	0.8	1.01	1.17	0.72
ZSWIM7	ZSWIM7;SWS1	zinc finger, SWIM-type containing 7	0.8	0.96	1.17	0.72
HIST1H2AH	HIST1H2AH;H2A/S;H2AFALii;MGC171151;dJ86C11.1	histone cluster 1, H2ah	0.79	0.9	1.17	0.71
GGT5	GGT5;DKFZp566O011;FLJ92733;GGT-REL;GGTLA1	gamma-glutamyltransferase 5	0.79	0.92	1.17	0.68
CCDC138	CCDC138;FLJ32745	coiled-coil domain containing 138	0.79	0.88	1.17	0.71
ALKBH6	ALKBH6;ABH6;MGC15677	alkB, alkylation repair homolog 6 (E. coli)	0.79	1	1.17	0.67
PLS1	PLS1	plastin 1	0.79	0.98	1.17	0.68
GAGE7	GAGE7;AL4;CT4.7;GAGE-7	G antigen 7	0.79	0.96	1.17	0.64
NAP1L3	NAP1L3;MB20;MGC26312;NPL3	nucleosome assembly protein 1-like 3	0.78	0.82	1.17	0.68
ZNF233	ZNF233;FLJ38032	zinc finger protein 233	0.78	0.94	1.17	0.72
ZNF189	ZNF189	zinc finger protein 189	0.78	0.94	1.17	0.63
P2RY2	P2RY2;HP2U;MGC20088;MGC40010;P2RU1;P2U;P2U1;P2UR;P2Y2;P2Y2R	purinergic receptor P2Y, G-protein coupled, 2	0.78	0.88	1.17	0.79
PPRC1	PPRC1;KIAA0595;MGC74642;PRC;RP11-302K17.6	peroxisome proliferator-activated receptor gamma, coactivator-related 1	0.78	0.88	1.17	0.63
DYNLT3	DYNLT3;RP3;TCTE1L;TCTEX1L	dynein, light chain, Tctex-type 3	0.78	0.97	1.17	0.68
TRIM74	TRIM74;MGC45440;TRIM50C	tripartite motif containing 74	0.78	0.9	1.17	0.81
ACER3	ACER3;APHC;FLJ11238;PHCA	alkaline ceramidase 3	0.78	0.92	1.17	0.71
HBG2	HBG2;FLJ76540;TNCY	hemoglobin, gamma G	0.78	1	1.17	0.73
UGT1A5	UGT1A5;UDPGT;UDPGT 1-5;UGT1E	UDP glucuronosyltransferase 1 family, polypeptide A5	0.78	0.98	1.17	0.64
HIP1	HIP1;ILWEQ;MGC126506	huntingtin interacting protein 1	0.77	0.98	1.17	0.72
GRHL2	GRHL2;BOM;DFNA28;FLJ11172;FLJ13782;MGC149294;MGC149295;TFCP2L3	grainyhead-like 2 (Drosophila)	0.77	0.98	1.17	0.75
LIMS2	LIMS2;FLJ10044;PINCH2	LIM and senescent cell antigen-like domains 2	0.76	0.92	1.17	0.69
NUDT16	NUDT16;FLJ31265;FLJ34034;FLJ36248	nudix (nucleoside diphosphate linked moiety X)-type motif 16	0.76	0.95	1.17	0.67
CDCA4	CDCA4;FLJ20764;FLJ52878;HEPP;MGC19517;SEI-3/HEPP	cell division cycle associated 4	0.76	0.8	1.17	0.67
ATAD2	ATAD2;ANCCA;DKFZp667N1320;MGC131938;MGC142216;MGC29843;MGC5254;PRO2000	ATPase family, AAA domain containing 2	0.76	0.84	1.17	0.66
PAPD5	PAPD5;FLJ40270;TRF4-2	PAP associated domain containing 5	0.76	0.86	1.17	0.61
GPRC5B	GPRC5B;RAIG-2;RAIG2	G protein-coupled receptor, family C, group 5, member B	0.76	0.84	1.17	0.63
TRNP1	TRNP1;C1orf225;TNRP	TMF1-regulated nuclear protein 1	0.76	0.88	1.17	0.73
FAM184A	FAM184A;C6orf60;FLJ13942	family with sequence similarity 184, member A	0.75	0.86	1.17	0.65
ZNF620	ZNF620;MGC50836	zinc finger protein 620	0.75	0.93	1.17	0.65
KIAA0907	KIAA0907;BLOM7;RP11-336K24.1	KIAA0907	0.75	0.9	1.17	0.62
RDH13	RDH13;DKFZp313H0740;FLJ39479;SDR7C3	retinol dehydrogenase 13 (all-trans/9-cis)	0.75	0.81	1.17	0.65
NIPAL2	NIPAL2;FLJ13955;NPAL2	NIPA-like domain containing 2	0.75	0.88	1.17	0.63
AKAP17A	AKAP17A;721P;AKAP-17A;CCDC133;CXYorf3;DXYS155E;FLJ98315;MGC125365;MGC125366;MGC39904;PRKA17A;SFRS17A;XE7;XE7Y	A kinase (PRKA) anchor protein 17A	0.75	0.94	1.17	0.66
FOXG1	FOXG1;BF1;BF2;FHKL3;FKH2;FKHL1;FKHL2;FKHL3;FKHL4;FOXG1A;FOXG1B;FOXG1C;HBF-1;HBF-2;HBF-3;HBF-G2;HBF2;HFK1;HFK2;HFK3;KHL2;QIN	forkhead box G1	0.74	0.8	1.17	0.64
PSKH2	PSKH2	protein serine kinase H2	0.74	0.84	1.17	0.75
ZNF2	ZNF2;A1-5;ZNF661;Zfp661	zinc finger protein 2	0.74	0.9	1.17	0.67
KCNIP3	KCNIP3;CSEN;DREAM;KCHIP3;MGC18289	Kv channel interacting protein 3, calsenilin	0.74	0.85	1.17	0.62
KSR1	KSR1;KSR;RSU2	kinase suppressor of ras 1	0.74	0.85	1.17	0.71
ABHD4	ABHD4;ABH4;FLJ12816	abhydrolase domain containing 4	0.74	0.86	1.17	0.64
DENND2C	DENND2C;DKFZp686G0351;DKFZp686N1631;DKFZp779P1149;FLJ37099;dJ1156J9.1	DENN/MADD domain containing 2C	0.74	0.84	1.17	0.64
KLC4	KLC4;KNSL8;MGC111777;bA387M24.3	kinesin light chain 4	0.74	0.89	1.17	0.64
REPIN1	REPIN1;AP4;RIP60;ZNF464;Zfp464	replication initiator 1	0.74	0.87	1.17	0.59
HEATR1	HEATR1;BAP28;FLJ10359;MGC72083;UTP10	HEAT repeat containing 1	0.74	0.88	1.17	0.67
WWC1	WWC1;FLJ10865;FLJ23369;HBEBP3;HBEBP36;KIAA0869;KIBRA	WW and C2 domain containing 1	0.74	0.94	1.17	0.63
ADRM1	ADRM1;ARM1;GP110;MGC29536;Rpn13	adhesion regulating molecule 1	0.74	0.89	1.17	0.68
CPNE3	CPNE3;CPN3;KIAA0636;PRO1071	copine III	0.74	0.86	1.17	0.71
COG5	COG5;CDG2I;FLJ41732;FLJ44289;GOLTC1;GTC90	component of oligomeric golgi complex 5	0.74	0.86	1.17	0.59
TRAM1	TRAM1;PNAS8;PRO1292;TRAM;TRAMP	translocation associated membrane protein 1	0.74	0.91	1.17	0.61
BTBD17	BTBD17;BTBD17A;LGALS3BPL	BTB (POZ) domain containing 17	0.74	0.88	1.17	0.59
WLS	WLS;C1orf139;DKFZp686I0788;EVI;FLJ23091;GPR177;MGC131760;MGC14878;MRP	wntless homolog (Drosophila)	0.74	0.92	1.17	0.65
RBMY1F	RBMY1F;MGC33094;YRRM2	RNA binding motif protein, Y-linked, family 1, member F	0.74	0.92	1.17	0.65
ZNF680	ZNF680;FLJ90430	zinc finger protein 680	0.73	0.87	1.17	0.65
ZNF532	ZNF532;FLJ10697	zinc finger protein 532	0.73	0.76	1.17	0.61
GCH1	GCH1;DYT14;DYT5;DYT5a;GCH;GTP-CH-1;GTPCH1;HPABH4B	GTP cyclohydrolase 1	0.73	0.8	1.17	0.63
RAP2C	RAP2C;DKFZp313B211	RAP2C, member of RAS oncogene family	0.73	0.82	1.17	0.59
RCOR1	RCOR1;COREST;KIAA0071;RCOR	REST corepressor 1	0.73	0.82	1.17	0.63
ZMAT4	ZMAT4;FLJ13842	zinc finger, matrin-type 4	0.72	0.77	1.17	0.62
KRT9	KRT9;CK-9;EPPK;K9	keratin 9	0.72	0.88	1.17	0.71
UBASH3B	UBASH3B;KIAA1959;MGC15437;STS-1;STS1;TULA-2;p70	ubiquitin associated and SH3 domain containing B	0.72	0.78	1.17	0.62
DYSF	DYSF;FER1L1;FLJ00175;FLJ90168;LGMD2B;MMD1	dysferlin, limb girdle muscular dystrophy 2B (autosomal recessive)	0.72	0.87	1.17	0.66
MPP6	MPP6;PALS2;VAM-1;VAM1;p55T	membrane protein, palmitoylated 6 (MAGUK p55 subfamily member 6)	0.72	0.84	1.17	0.62
RRP1	RRP1;D21S2056E;NNP-1;NOP52;RRP1A	ribosomal RNA processing 1 homolog (S. cerevisiae)	0.72	0.8	1.17	0.64
GADD45A	GADD45A;DDIT1;GADD45	growth arrest and DNA-damage-inducible, alpha	0.72	0.82	1.17	0.7
PIEZO2	PIEZO2;C18orf30;C18orf58;FAM38B;FAM38B2;FLJ23144;FLJ23403;FLJ25916;FLJ34907;FLJ37734;FLJ45725;HsT748;HsT771	piezo-type mechanosensitive ion channel component 2	0.72	0.81	1.17	0.65
TAC3	TAC3;NKB;NKNB;PRO1155;ZNEUROK1	tachykinin 3	0.71	0.82	1.17	0.61
C12orf34	C12orf34;FLJ14721	chromosome 12 open reading frame 34	0.71	0.86	1.17	0.61
TBL2	TBL2;DKFZp434N024;MGC134739;WBSCR13;WS-betaTRP	transducin (beta)-like 2	0.71	0.75	1.17	0.63
TCEB3	TCEB3;EloA;FLJ38760;FLJ42849;SIII;SIII p110;TCEB3A	transcription elongation factor B (SIII), polypeptide 3 (110kDa, elongin A)	0.71	0.81	1.17	0.6
JUP	JUP;ARVD12;CTNNG;DP3;DPIII;PDGB;PKGB	junction plakoglobin	0.71	0.82	1.17	0.69
ZNF235	ZNF235;ANF270;HZF6;ZFP93;ZNF270	zinc finger protein 235	0.7	0.79	1.17	0.62
PHEX	PHEX;HPDR;HPDR1;HYP;HYP1;LXHR;PEX;XLH	phosphate regulating endopeptidase homolog, X-linked	0.7	0.82	1.17	0.61
PGA3	PGA3;DKFZp666J2410;PGA4;PGA5	pepsinogen 3, group I (pepsinogen A)	0.7	0.82	1.17	0.63
C5orf13	C5orf13;D4S114;P311;PRO1873;PTZ17	chromosome 5 open reading frame 13	0.7	0.78	1.17	0.66
FLNB	FLNB;ABP-278;ABP-280;AOI;DKFZp686A1668;DKFZp686O033;FH1;FLN-B;FLN1L;LRS1;SCT;TABP;TAP	filamin B, beta	0.7	0.8	1.17	0.6
TFRC	TFRC;CD71;T9;TFR;TFR1;TR;TRFR;p90	transferrin receptor (p90, CD71)	0.7	0.81	1.17	0.61
SLX1A	SLX1A;GIYD1;GIYD2;SLX1B	SLX1 structure-specific endonuclease subunit homolog A (S. cerevisiae)	0.7	0.86	1.17	0.57
CD97	CD97;TM7LN1	CD97 molecule	0.69	0.79	1.17	0.71
LAMTOR2	LAMTOR2;ENDAP;HSPC003;MAPBPIP;MAPKSP1AP;ROBLD3;Ragulator2;p14	late endosomal/lysosomal adaptor, MAPK and MTOR activator 2	0.69	0.78	1.17	0.59
TMEM62	TMEM62;FLJ23375	transmembrane protein 62	0.68	0.8	1.17	0.62
POU3F1	POU3F1;OCT6;OTF6;SCIP	POU class 3 homeobox 1	0.68	0.81	1.17	0.54
ATP5SL	ATP5SL;FLJ10241;FLJ31003;FLJ60315	ATP5S-like	0.68	0.82	1.17	0.62
CCNA1	CCNA1	cyclin A1	0.68	0.77	1.17	0.67
TRAPPC1	TRAPPC1;BET5;MUM2	trafficking protein particle complex 1	0.68	0.77	1.17	0.58
ZFYVE28	ZFYVE28;LST2;LYST2;MGC43699;MGC61592	zinc finger, FYVE domain containing 28	0.67	0.76	1.17	0.55
WNT1	WNT1;INT1	wingless-type MMTV integration site family, member 1	0.67	0.82	1.17	0.61
EREG	EREG;ER	epiregulin	0.66	0.71	1.17	0.63
ADCY1	ADCY1;AC1	adenylate cyclase 1 (brain)	0.66	0.73	1.17	0.57
ZNF23	ZNF23;KOX16;MGC57283;ZNF359;ZNF612;Zfp612	zinc finger protein 23 (KOX 16)	0.66	0.84	1.17	0.61

LIPE	LIPE;HSL;LHS	lipase, hormone-sensitive	0.66	0.77	1.17	0.6
C9orf25	C9orf25;FLJ39031;bA573M23.5	chromosome 9 open reading frame 25	0.66	0.79	1.17	0.57
FAM156A	FAM156A;DKFZp686B22211;FAM156B;PRO0659;TMEM29	family with sequence similarity 156, member A	0.66	0.74	1.17	0.57
PEX14	PEX14;MGC12767;NAPP2;Pex14p;dJ734G22.2	peroxisomal biogenesis factor 14	0.66	0.76	1.17	0.64
C20orf24	C20orf24;RIP5	chromosome 20 open reading frame 24	0.66	0.78	1.17	0.6
RABAC1	RABAC1;PRA1;PRAF1;YIP3	Rab acceptor 1 (prenylated)	0.66	0.78	1.17	0.54
ANKDD1A	ANKDD1A;FLJ18078;FLJ25870;MGC120305;MGC120306;MGC120307	ankyrin repeat and death domain containing 1A	0.65	0.82	1.17	0.57
ISYNA1	ISYNA1;INO1;INOS;IPS	inositol-3-phosphate synthase 1	0.65	0.7	1.17	0.56
SLAMF9	SLAMF9;CD2F-10;CD2F10;CD84-H1;CD84H1;MGC88194;SF2001	SLAM family member 9	0.64	0.77	1.17	0.58
SULT4A1	SULT4A1;BR-STL-1;BRSTL1;DJ388M5.3;MGC40032;NST;SULTX3;hBR-STL-1	sulfotransferase family 4A, member 1	0.64	0.72	1.17	0.56
SEMA4B	SEMA4B;KIAA1745;MGC131831;SEMAC;SemC	sema domain, immunoglobulin domain (Ig), transmembrane domain (TM) and short cytoplasmic domain, (semaphorin) 4B	0.64	0.7	1.17	0.59
CTSH	CTSH;ACC-4;ACC-5;CPSB;DKFZp686B24257;MGC1519;minichain	cathepsin H	0.64	0.74	1.17	0.56
GPX3	GPX3;GPx-P;GSHPx-3;GSHPx-P	glutathione peroxidase 3 (plasma)	0.64	0.73	1.17	0.51
C10orf114	C10orf114;bA418C1.3	chromosome 10 open reading frame 114	0.63	0.77	1.17	0.55
CXXC1	CXXC1;2410002I16Rik;5830420C16Rik;CFP1;CGBP;HsT2645;PCCX1;PHF18;SPP1;ZCGPC1;hCGBP	CXXC finger protein 1	0.63	0.73	1.17	0.65
PTGFRN	PTGFRN;CD315;CD9P-1;EWI-F;FLJ11001;FPRP;KIAA1436;SMAP-6	prostaglandin F2 receptor negative regulator	0.63	0.74	1.17	0.52
ASB18	ASB18;ASB-18;DKFZp313I1130	ankyrin repeat and SOCS box containing 18	0.62	0.68	1.17	0.49
POTEE	POTEE;A26C1;A26C1A;CT104.2;POTE-2;POTE2;POTE2gamma	POTE ankyrin domain family, member E	0.62	0.75	1.17	0.54
POU5F2	POU5F2;DKFZp686P02123;FLJ25680;SPRM-1	POU domain class 5, transcription factor 2	0.61	0.74	1.17	0.56
FAM173A	FAM173A;C16orf24;MGC2494	family with sequence similarity 173, member A	0.61	0.75	1.17	0.53
S100B	S100B;NEF;S100;S100-B;S100beta	S100 calcium binding protein B	0.84	0.99	1.18	0.69
OR56B1	OR56B1;OR11-65;OR56B1P	olfactory receptor, family 56, subfamily B, member 1	0.84	0.97	1.18	0.81
PLCL2	PLCL2;FLJ13484;KIAA1092;PLCE2	phospholipase C-like 2	0.84	1	1.18	0.67
SYNE1	SYNE1;8B;ARCA1;C6orf98;CPG2;DKFZp781J13156;EDMD4;FLJ30878;FLJ41140;KIAA0796;KIAA1262;KIAA1756;MYNE1;SCAR8;dJ45H2.2	spectrin repeat containing, nuclear envelope 1	0.84	0.98	1.18	0.74
HTR1D	HTR1D;5-HT1D;HT1DA;HTR1DA;HTRL;RDC4	5-hydroxytryptamine (serotonin) receptor 1D	0.84	0.96	1.18	0.86
FMNL1	FMNL1;C17orf1;C17orf1B;FHOD4;FMNL;KW-13;MGC133052;MGC1894;MGC21878	formin-like 1	0.84	1.02	1.18	0.77
ZAK	ZAK;AZK;MLK7;MLT;MLTK;MRK;mlklak;pk	sterile alpha motif and leucine zipper containing kinase AZK	0.84	1.06	1.18	0.75
MOSPD2	MOSPD2;DKFZp686C1553;MGC26706	motile sperm domain containing 2	0.84	1.01	1.18	0.77
QRICH1	QRICH1;FLJ20259;MGC131838	glutamine-rich 1	0.84	0.98	1.18	0.73
DYRK3	DYRK3;DYRK5;RED;REDK;hYAK3-2	dual-specificity tyrosine-(Y)-phosphorylation regulated kinase 3	0.83	1.06	1.18	0.75
ORMDL3	ORMDL3	ORM1-like 3 (S. cerevisiae)	0.83	1.03	1.18	0.84
EFHA2	EFHA2;DKFZp313A0139	EF-hand domain family, member A2	0.82	1	1.18	0.72
WISP1	WISP1;CCN4;FLJ14388;WISP1c;WISP1i;WISP1tc	WNT1 inducible signaling pathway protein 1	0.82	1	1.18	0.76
SRFBP1	SRFBP1;BUD22;FLJ25286;P49;Rlb1;STRAP;p49/STRAP	serum response factor binding protein 1	0.82	1.03	1.18	0.79
MRPS24	MRPS24;MRP-S24;S24mt;bMRP-47;bMRP47	mitochondrial ribosomal protein S24	0.82	0.88	1.18	0.73
EIF5B	EIF5B;DKFZp434I036;FLJ10524;IF2;KIAA0741	eukaryotic translation initiation factor 5B	0.82	0.98	1.18	0.76
KCTD13	KCTD13;PDIP1;POLDIP1;hBACURD1	potassium channel tetramerisation domain containing 13	0.81	0.94	1.18	0.74
TMCO6	TMCO6;FLJ39769	transmembrane and coiled-coil domains 6	0.81	1.04	1.18	0.69
LOC653978	LOC653978	ENTREZ STATUS: DISCONTINUED	0.81	0.94	1.18	0.84
EPT1	EPT1;KIAA1724;SELI;SEPI	ethanolaminephosphotransferase 1 (CDP-ethanolamine-specific)	0.81	0.96	1.18	0.74
ZNF167	ZNF167;FLJ12738;ZFP;ZKSCAN7;ZNF448;ZNF64	zinc finger protein 167	0.8	0.97	1.18	0.66
RIBC1	RIBC1;2610028I09Rik;FLJ32783;MGC46233	RIB43A domain with coiled-coils 1	0.8	0.93	1.18	0.7
EPHA4	EPHA4;HEK8;SEK;TYRO1	EPH receptor A4	0.8	0.92	1.18	0.83
LRRC4B	LRRC4B;DKFZp761A179;HSM;LRIG4	leucine rich repeat containing 4B	0.8	0.85	1.18	0.71
FAM165B	FAM165B;C21orf51	family with sequence similarity 165, member B	0.8	0.9	1.18	0.7
DNAJB14	DNAJB14;EGNR9427;FLJ14281;MGC22187;PRO34683	DnaJ (Hsp40) homolog, subfamily B, member 14	0.8	0.92	1.18	0.8
PPP1R9A	PPP1R9A;FLJ20068;KIAA1222;NRB1;NRBI;Neurabin-I	protein phosphatase 1, regulatory subunit 9A	0.8	0.99	1.18	0.74
ARRDC5	ARRDC5	arrestin domain containing 5	0.8	1	1.18	0.77
NISCH	NISCH;FLJ14425;FLJ40413;FLJ90519;I-1;IRAS;KIAA0975	nischarin	0.79	0.9	1.18	0.67
CENPM	CENPM;C22orf18;CENP-M;MGC861;PANE1;bK250D10.2	centromere protein M	0.79	0.84	1.18	0.65
PURA	PURA;PUR-ALPHA;PUR1;PURALPHA	purine-rich element binding protein A	0.79	0.94	1.18	0.75
SNCAIP	SNCAIP;MGC39814;SYPH1;Sph1	synuclein, alpha interacting protein	0.78	0.88	1.18	0.74
RNF208	RNF208;DKFZp761H1710;MGC88636	ring finger protein 208	0.78	0.92	1.18	0.82
HOXA10	HOXA10;HOX1;HOX1.8;HOX1H;MGC12859;PL	homeobox A10	0.78	0.92	1.18	0.71
GNAZ	GNAZ	guanine nucleotide binding protein (G protein), alpha z polypeptide	0.78	1.02	1.18	0.7
AZI1	AZI1;AZ1;Cep131;ZA1	5-azacytidine induced 1	0.78	0.92	1.18	0.64
WDR41	WDR41;FLJ10904	WD repeat domain 41	0.78	1	1.18	0.67
TNS4	TNS4;CTEN;FLJ14950	tensin 4	0.78	0.88	1.18	0.71
C11orf74	C11orf74;FLJ38678	chromosome 11 open reading frame 74	0.77	0.94	1.18	0.7
TAX1BP3	TAX1BP3;TIP-1	Tax1 (human T-cell leukemia virus type I) binding protein 3	0.77	0.9	1.18	0.75
PABPC1	PABPC1;PAB1;PABP;PABP1;PABPC2;PABPL1	poly(A) binding protein, cytoplasmic 1	0.77	0.88	1.18	0.76
41153	SEPT12;FLJ25410	septin 12	0.76	0.92	1.18	0.66
AUTS2	AUTS2;FBRSL2;KIAA0442;MGC13140	autism susceptibility candidate 2	0.76	0.82	1.18	0.65
IFIT5	IFIT5;FLJ53857;FLJ92678;RI58	interferon-induced protein with tetratricopeptide repeats 5	0.76	0.84	1.18	0.71
VPS13D	VPS13D;FLJ23066	vacuolar protein sorting 13 homolog D (S. cerevisiae)	0.76	0.88	1.18	0.69
NDUFB1	NDUFB1;CI-MNLL;CI-SGDH;MNLL	NADH dehydrogenase (ubiquinone) 1 beta subcomplex, 1, 7kDa	0.76	0.83	1.18	0.66
AGPAT5	AGPAT5;1AGPAT5;LPAATE	1-acylglycerol-3-phosphate O-acyltransferase 5 (lysophosphatidic acid acyltransferase, epsilon)	0.76	0.99	1.18	0.69
CCL3L3	CCL3L3;464.2;D17S1718;LD78;LD78BETA;MGC12815;SCYA3L;SCYA3L1	chemokine (C-C motif) ligand 3-like 3	0.76	0.88	1.18	0.62
NFAM1	NFAM1;CNAIP;FLJ40652;bK126B4.4	NFAT activating protein with ITAM motif 1	0.75	0.92	1.18	0.62
CCNT2	CCNT2;CYCT2;FLJ13583;FLJ90560;MGC134840	cyclin T2	0.75	0.82	1.18	0.73
OR6K2	OR6K2;OR1-17	olfactory receptor, family 6, subfamily K, member 2	0.74	0.88	1.18	0.61
NLRP2	NLRP2;CLR19.9;FLJ20510;NALP2;NBS1;PAN1;PYPAF2	NLR family, pyrin domain containing 2	0.74	0.84	1.18	0.66
FAM163B	FAM163B;C9orf166	family with sequence similarity 163, member B	0.74	0.82	1.18	0.67
SLC25A37	SLC25A37;MFRN;MFRN1;MSC;MSCP;PRO1278;PRO1584;PRO2217	solute carrier family 25, member 37	0.74	0.85	1.18	0.63
ZNF267	ZNF267;HZF2	zinc finger protein 267	0.74	0.85	1.18	0.69
BRMS1	BRMS1;DKFZp564A063	breast cancer metastasis suppressor 1	0.74	0.9	1.18	0.68
ATP6V0A1	ATP6V0A1;ATP6N1;ATP6N1A;DKFZp781J1951;Stv1;VPP1;Vph1;a1	ATPase, H+ transporting, lysosomal V0 subunit a1	0.74	0.86	1.18	0.74
APOO	APOO;FAM121B;MGC4825	apolipoprotein O	0.74	0.86	1.18	0.64
C18orf54	C18orf54;LAS2;MGC33382	chromosome 18 open reading frame 54	0.73	0.93	1.18	0.62
FAM83C	FAM83C;C20orf128;MGC142043;dJ614O4.7	family with sequence similarity 83, member C	0.73	0.82	1.18	0.66
PPM1B	PPM1B;MGC21657;PP2C-beta-X;PP2CB;PP2CBETA;PPC2BETAX	protein phosphatase, Mg2+/Mn2+ dependent, 1B	0.73	0.8	1.18	0.6
RSU1	RSU1;FLJ31034;RSP-1	Ras suppressor protein 1	0.73	0.8	1.18	0.68
C17orf49	C17orf49;BAP18;MGC49942	chromosome 17 open reading frame 49	0.73	0.94	1.18	0.63
PLBD2	PLBD2;P76	phospholipase B domain containing 2	0.73	0.78	1.18	0.62
FOXR1	FOXR1;FOXN5;MGC149486	forkhead box R1	0.72	0.86	1.18	0.63
TAL1	TAL1;SCL;TCL5;bHLHa17;tal-1	T-cell acute lymphocytic leukemia 1	0.72	0.84	1.18	0.63
SNAP25	SNAP25;FLJ23079;RIC-4;RIC4;SEC9;SNAP;SNAP-25;bA416N4.2;dJ1068F16.2	synaptosomal-associated protein, 25kDa	0.72	0.84	1.18	0.66
ZNF598	ZNF598;DKFZp762F135;FLJ00086	zinc finger protein 598	0.72	0.82	1.18	0.69
GCDH	GCDH;ACAD5;GCD	glutaryl-CoA dehydrogenase	0.72	0.78	1.18	0.69
ZNF692	ZNF692;AREBP;FLJ20531;Zfp692	zinc finger protein 692	0.71	0.86	1.18	0.64
MXRA7	MXRA7;FLJ41492;FLJ46603;PS1TP1;TMAP1	matrix-remodelling associated 7	0.71	0.82	1.18	0.62
DNAJA1	DNAJA1;DJ-2;DjA1;HDJ2;HSDJ;HSJ2;HSPF4;NEDD7;hDJ-2	DnaJ (Hsp40) homolog, subfamily A, member 1	0.71	0.8	1.18	0.7
ATP13A4	ATP13A4;DKFZp761I1011;MGC126545	ATPase type 13A4	0.7	0.76	1.18	0.67
GBP4	GBP4;Mpa2	guanylate binding protein 4	0.7	0.83	1.18	0.58
CTSK	CTSK;CTS02;CTSO;CTSO1;CTSO2;MGC23107;PKND;PYCD	cathepsin K	0.7	0.81	1.18	0.61
CORO2B	CORO2B;CLIPINC;KIAA0925	coronin, actin binding protein, 2B	0.7	0.78	1.18	0.66
ERO1LB	ERO1LB;DKFZp779C1042;DKFZp779I0141;FLJ11003	ERO1-like beta (S. cerevisiae)	0.7	0.87	1.18	0.58
ASCL2	ASCL2;ASH2;HASH2;MASH2;bHLHa45	achaete-scute complex homolog 2 (Drosophila)	0.7	0.82	1.18	0.56
KLK5	KLK5;KLK-L2;KLKL2;SCTE	kallikrein-related peptidase 5	0.7	0.82	1.18	0.61
TMEM184A	TMEM184A;FLJ24011;MGC9712	transmembrane protein 184A	0.7	0.8	1.18	0.65
RASSF7	RASSF7;C11orf13;HRAS1;HRC1;MGC126069;MGC126070	Ras association (RalGDS/AF-6) domain family (N-terminal) member 7	0.7	0.74	1.18	0.57
ACTG1	ACTG1;ACT;ACTG;DFNA20;DFNA26	actin, gamma 1	0.7	0.86	1.18	0.65
C1QL2	C1QL2;C1QTNF10;CTRP10	complement component 1, q subcomponent-like 2	0.69	0.84	1.18	0.64
LMO3	LMO3;DAT1;MGC26081;RBTN3;RBTNL2;RHOM3;Rhom-3	LIM domain only 3 (rhombotin-like 2)	0.69	0.8	1.18	0.64
EXOC5	EXOC5;DKFZp666H126;HSEC10;PRO1912;SEC10;SEC10L1;SEC10P	exocyst complex component 5	0.69	0.85	1.18	0.61
ADAM10	ADAM10;AD10;CD156c;HsT18717;MADM;kuz	ADAM metallopeptidase domain 10	0.69	0.81	1.18	0.61
MYO1F	MYO1F	myosin IF	0.68	0.82	1.18	0.63
ZNF256	ZNF256;BMZF-3;BMZF3	zinc finger protein 256	0.68	0.84	1.18	0.63
C21orf58	C21orf58	chromosome 21 open reading frame 58	0.68	0.77	1.18	0.59
FGFR3	FGFR3;ACH;CD333;CEK2;HSFGFR3EX;JTK4	fibroblast growth factor receptor 3	0.68	0.8	1.18	0.66
TCEA2	TCEA2;TFIIS	transcription elongation factor A (SII), 2	0.68	0.89	1.18	0.62
CRHR1	CRHR1;CRF-R;CRF-R-1;CRF-R1;CRF1;CRFR-1;CRFR1;CRH-R-1;CRH-R1;CRH-R1h;CRHR;CRHR1L;CRHR1f	corticotropin releasing hormone receptor 1	0.68	0.78	1.18	0.7
PPAN-P2RY1	PPAN-P2RY11	PPAN-P2RY11 readthrough	0.68	0.78	1.18	0.59
CHI3L1	CHI3L1;ASRT7;CGP-39;DKFZp686N19119;FLJ38139;GP-39;GP39;HC-gp39;HCGP-3P;YKL-40;YKL40;YYL-40;hCGP-39	chitinase 3-like 1 (cartilage glycoprotein-39)	0.68	0.81	1.18	0.54
NOM1	NOM1;C7orf3;FLJ16401;SGD1	nucleolar protein with MIF4G domain 1	0.68	0.77	1.18	0.59
TMEM56	TMEM56;FLJ31842;MGC102912	transmembrane protein 56	0.68	0.76	1.18	0.62
RAP2B	RAP2B;MGC20484	RAP2B, member of RAS oncogene family	0.68	0.84	1.18	0.56
MAML2	MAML2;DKFZp686N0150;KIAA1819;MAM-3;MAM2;MAM3;MGC176701;MLL-MAML2	mastermind-like 2 (Drosophila)	0.68	0.78	1.18	0.55
ARHGAP31	ARHGAP31;CDGAP;KIAA1204;MGC138368;MGC138370	Rho GTPase activating protein 31	0.68	0.8	1.18	0.54
FAM203A	FAM203A;BRP16;C8orf30A;FLJ40907	family with sequence similarity 203, member A	0.68	0.86	1.18	0.58
LGR6	LGR6;FLJ14471;GPCR;VTS20631	leucine-rich repeat containing G protein-coupled receptor 6	0.67	0.76	1.18	0.55
HRCT1	HRCT1;LGLL338;PRO537;UNQ338	histidine rich carboxyl terminus 1	0.67	0.8	1.18	0.58
HCG9	HCG9;HCGIX;HCGIX4	HLA complex group 9 (non-protein coding)	0.66	0.71	1.18	0.56
LILRA4	LILRA4;CD85g;ILT7;MGC129597;MGC129598	leukocyte immunoglobulin-like receptor, subfamily A (with TM domain), member 4	0.66	0.78	1.18	0.57
HIST1H4G	HIST1H4G;H4/I;H4FL	histone cluster 1, H4g	0.66	0.8	1.18	0.61
MAGEA4	MAGEA4;CT1.4;MAGE-41;MAGE-X2;MAGE4;MAGE4A;MAGE4B;MGC21336	melanoma antigen family A, 4	0.66	0.76	1.18	0.61
RTTN	RTTN;DKFZp434G145;FLJ26356;FLJ39085	rotatin	0.66	0.82	1.18	0.66
CD72	CD72;CD72b;LYB2	CD72 molecule	0.66	0.76	1.18	0.54
SLC5A10	SLC5A10;FLJ25217;SGLT5	solute carrier family 5 (sodium/glucose cotransporter), member 10	0.66	0.78	1.18	0.52

CNNM2	CNNM2;ACDP2	cyclin M2	0.66	0.81	1.18	0.54
TTC39A	TTC39A;C1orf34;DEME-6;KIAA0452	tetratricopeptide repeat domain 39A	0.66	0.76	1.18	0.57
PTGDS	PTGDS;L-PGDS;LPGDS;PDS;PGD2;PGDS;PGDS2	prostaglandin D2 synthase 21kDa (brain)	0.66	0.78	1.18	0.6
CYP2D6	CYP2D6;CPD6;CYP2D;CYP2D7AP;CYP2D7BP;CYP2D7P2;CYP2D8P2;CYP2DL1;CYPIID6;MGC120389;MGC120390;P450-DB1;P450C2D;P450DB1	cytochrome P450, family 2, subfamily D, polypeptide 6	0.66	0.86	1.18	0.64
NUDT22	NUDT22;MGC13045	nudix (nucleoside diphosphate linked moiety X)-type motif 22	0.66	0.8	1.18	0.58
VAC14	VAC14;ArPIKfyve;FLJ10305;FLJ36622;FLJ46582;MGC149815;MGC149816;TAX1BP2;TRX	Vac14 homolog (S. cerevisiae)	0.66	0.8	1.18	0.57
TAGLN	TAGLN;DKFZp686B01212;DKFZp686P11128;SM22;SMCC;TAGLN1;WS3-10	transgelin	0.66	0.8	1.18	0.55
TMX2	TMX2;DKFZp781O2021;MGC111151;PDIA12;PIG26;TXNDC14	thioredoxin-related transmembrane protein 2	0.66	0.77	1.18	0.57
CTXN1	CTXN1;CTXN;FLJ25968	cortexin 1	0.65	0.7	1.18	0.56
TMCC1	TMCC1;DKFZp686M0169;FLJ42680	transmembrane and coiled-coil domain family 1	0.65	0.8	1.18	0.53
CTTNBP2NL	CTTNBP2NL;DKFZp547A023;FLJ13278	CTTNBP2 N-terminal like	0.65	0.75	1.18	0.56
PLAUR	PLAUR;CD87;U-PAR;UPAR;URKR	plasminogen activator, urokinase receptor	0.65	0.72	1.18	0.57
NRK	NRK;DKFZp686A17109;FLJ16788;MGC131849;NESK	Nik related kinase	0.64	0.82	1.18	0.58
APBA1	APBA1;D9S411E;LIN10;MINT1;X11;X11A;X11ALPHA	amyloid beta (A4) precursor protein-binding, family A, member 1	0.64	0.76	1.18	0.55
C20orf195	C20orf195;MGC5356	chromosome 20 open reading frame 195	0.64	0.72	1.18	0.55
PTPDC1	PTPDC1;FLJ42922;PTP9Q22	protein tyrosine phosphatase domain containing 1	0.64	0.71	1.18	0.53
UFSP1	UFSP1;UFSP	UFM1-specific peptidase 1 (non-functional)	0.64	0.72	1.18	0.58
TSPAN15	TSPAN15;2700063A19Rik;NET-7;NET7;TM4SF15	tetraspanin 15	0.64	0.68	1.18	0.53
AASDHPPT	AASDHPPT;AASD-PPT;DKFZp566E2346;LYS2;LYS5	aminoadipate-semialdehyde dehydrogenase-phosphopantetheinyl transferase	0.64	0.8	1.18	0.52
KRT18P55	KRT18P55;FLJ40504;MGC138231;MGC138233	keratin 18 pseudogene 55	0.64	0.76	1.18	0.51
SPATA2L	SPATA2L;C16orf76;MGC26885;tamo	spermatogenesis associated 2-like	0.63	0.73	1.18	0.55
PLCB4	PLCB4;FLJ16169;PI-PLC	phospholipase C, beta 4	0.63	0.7	1.18	0.55
RAB11FIP3	RAB11FIP3;CART1;KIAA0665;Rab11-FIP3	RAB11 family interacting protein 3 (class II)	0.63	0.74	1.18	0.53
NENF	NENF;CIR2;SCIRP10;SPUF	neudesin neurotrophic factor	0.63	0.66	1.18	0.51
NUP88	NUP88;MGC8530	nucleoporin 88kDa	0.63	0.75	1.18	0.52
SCIN	SCIN;KIAA1905	scinderin	0.62	0.76	1.18	0.51
CDKL1	CDKL1;KKIALRE;P42	cyclin-dependent kinase-like 1 (CDC2-related kinase)	0.62	0.79	1.18	0.56
ALOX12B	ALOX12B;12R-LOX	arachidonate 12-lipoxygenase, 12R type	0.62	0.76	1.18	0.59
C1orf216	C1orf216;FLJ38984	chromosome 1 open reading frame 216	0.62	0.75	1.18	0.54
TBC1D8	TBC1D8;AD3;HBLP1;TBC1D8A;VRP	TBC1 domain family, member 8 (with GRAM domain)	0.62	0.7	1.18	0.51
C15orf41	C15orf41;FLJ22851;HH114;MGC11326	chromosome 15 open reading frame 41	0.62	0.78	1.18	0.6
DHX57	DHX57;DDX57;FLJ32861	DEAH (Asp-Glu-Ala-Asp/His) box polypeptide 57	0.62	0.74	1.18	0.57
KIAA1033	KIAA1033	KIAA1033	0.62	0.73	1.18	0.5
PRPF31	PRPF31;DKFZp566J153;NY-BR-99;PRP31;RP11	PRP31 pre-mRNA processing factor 31 homolog (S. cerevisiae)	0.62	0.76	1.18	0.6
BSG	BSG;5F7;CD147;EMMPRIN;M6;OK;TCSF	basigin (Ok blood group)	0.62	0.76	1.18	0.54
KDM2A	KDM2A;CXXC8;DKFZp434M1735;FBL11;FBL7;FBXL11;FLJ00115;FLJ46431;JHDM1A;KIAA1004;LILINA;SF-KDM2A	lysine (K)-specific demethylase 2A	0.62	0.7	1.18	0.51
CD163L1	CD163L1;CD163B;M160	CD163 molecule-like 1	0.61	0.7	1.18	0.6
ABI2	ABI2;ABI-2;ABI2B;AIP-1;AblBP3;SSH3BP2;argBPIA;argBPIB	abl-interactor 2	0.61	0.72	1.18	0.49
IFITM5	IFITM5;BRIL;Hrmp1;fragilis4	interferon induced transmembrane protein 5	0.84	0.96	1.19	0.74
DISP1	DISP1;DISPA;DKFZp434I0428;FLJ43740;MGC104180;MGC13130;MGC16796	dispatched homolog 1 (Drosophila)	0.84	1.03	1.19	0.78
TAF4B	TAF4B;TAF2C2;TAFII105	TAF4b RNA polymerase II, TATA box binding protein (TBP)-associated factor, 105kDa	0.84	1.06	1.19	0.7
ZMYM1	ZMYM1;FLJ23151;FLJ43753;MYM;RP11-181E22.4	zinc finger, MYM-type 1	0.84	0.9	1.19	0.75
FLJ20054	FLJ20054	ENTREZ STATUS: REMAPPED TO GENE ID: 163486	0.84	0.95	1.19	0.8
IL20RB	IL20RB;DIRS1;FNDC6;IL-20R2;MGC34923	interleukin 20 receptor beta	0.83	0.96	1.19	0.73
C22orf15	C22orf15;FLJ36561;N27C7-3	chromosome 22 open reading frame 15	0.83	0.97	1.19	0.73
C8orf46	C8orf46;MGC33510	chromosome 8 open reading frame 46	0.83	0.94	1.19	0.72
PIGK	PIGK;GPI8;MGC22559	phosphatidylinositol glycan anchor biosynthesis, class K	0.83	0.9	1.19	0.68
37226	DEC1;CTS9	deleted in esophageal cancer 1	0.82	0.88	1.19	0.7
PRSS36	PRSS36;FLJ90661	protease, serine, 36	0.82	0.97	1.19	0.72
IREB2	IREB2;ACO3;FLJ23381;IRP2;IRP2AD	iron-responsive element binding protein 2	0.82	0.97	1.19	0.72
ELANE	ELANE;ELA2;GE;HLE;HNE;NE;PMN-E;SCN1	elastase, neutrophil expressed	0.82	0.96	1.19	0.72
HTR5A	HTR5A;5-HT5A;MGC138226	5-hydroxytryptamine (serotonin) receptor 5A	0.81	0.94	1.19	0.85
ZNF524	ZNF524;MGC23143	zinc finger protein 524	0.81	0.94	1.19	0.76
CERS3	CERS3;LASS3;MGC27091	ceramide synthase 3	0.81	0.94	1.19	0.74
COX8C	COX8C;COX8-3;MGC119774;MGC119775	cytochrome c oxidase subunit VIIIC	0.8	0.92	1.19	0.66
CEMP1	CEMP1;CP-23;CP23	cementum protein 1	0.8	0.98	1.19	0.67
STBD1	STBD1;FLJ41801;GENEX3414	starch binding domain 1	0.8	1	1.19	0.75
CCDC115	CCDC115;FLJ30131;MGC12981;ccp1	coiled-coil domain containing 115	0.8	0.84	1.19	0.66
TAF1B	TAF1B;MGC:9349;RAF1B;RAFI63;SL1;TAFI63	TATA box binding protein (TBP)-associated factor, RNA polymerase I, B, 63kDa	0.8	0.91	1.19	0.66
C6orf211	C6orf211;DKFZp566I174;FLJ12910	chromosome 6 open reading frame 211	0.8	0.99	1.19	0.66
HDAC9	HDAC9;DKFZp779K1053;HD7;HD7b;HD9;HDAC;HDAC7;HDAC7B;HDAC9B;HDAC9FL;HDRP;KIAA0744;MITR	histone deacetylase 9	0.8	0.88	1.19	0.78
AES	AES;AES-1;AES-2;ESP1;GRG;GRG5;TLE5	amino-terminal enhancer of split	0.8	0.9	1.19	0.69
C11orf54	C11orf54;PTD012	chromosome 11 open reading frame 54	0.8	1	1.19	0.74
METAP1D	METAP1D;MAP1D;Metap1l	methionyl aminopeptidase type 1D (mitochondrial)	0.8	0.94	1.19	0.71
HHATL	HHATL;C3orf3;GUP1;KIAA1173;MBOAT3;MSTP002;OACT3	hedgehog acyltransferase-like	0.79	0.9	1.19	0.72
PLK3	PLK3;CNK;FNK;PRK	polo-like kinase 3	0.79	0.9	1.19	0.82
C10orf76	C10orf76;FLJ13114;MGC125927	chromosome 10 open reading frame 76	0.79	0.9	1.19	0.65
KRR1	KRR1;HRB2;RIP-1	KRR1, small subunit (SSU) processome component, homolog (yeast)	0.79	0.97	1.19	0.72
MKRN1	MKRN1;FLJ21334;RNF61	makorin ring finger protein 1	0.79	0.93	1.19	0.83
FMN2	FMN2	formin 2	0.78	0.96	1.19	0.64
MME	MME;CALLA;CD10;DKFZp686O16152;MGC126681;MGC126707;NEP;SFE	membrane metallo-endopeptidase	0.78	0.92	1.19	0.69
H2AFB1	H2AFB1;H2A.Bbd	H2A histone family, member B1	0.78	0.9	1.19	0.71
ADAMTS17	ADAMTS17;FLJ16363;FLJ32769	ADAM metallopeptidase with thrombospondin type 1 motif, 17	0.78	0.92	1.19	0.69
KRT79	KRT79;FLJ26646;K6L;KRT6L	keratin 79	0.78	0.9	1.19	0.75
FAM46A	FAM46A;C6orf37;FLJ20037;FLJ31495;XTP11	family with sequence similarity 46, member A	0.78	1	1.19	0.77
ALG6	ALG6;CDG1C	asparagine-linked glycosylation 6, alpha-1,3-glucosyltransferase homolog (S. cerevisiae)	0.78	0.93	1.19	0.72
NDUFAB1	NDUFAB1;ACP;FASN2A;MGC65095;SDAP	NADH dehydrogenase (ubiquinone) 1, alpha/beta subcomplex, 1, 8kDa	0.78	0.97	1.19	0.75
MTMR14	MTMR14;C3orf29;FLJ11546;FLJ22405;FLJ46453;FLJ90311	myotubularin related protein 14	0.78	0.86	1.19	0.65
MFSD6L	MFSD6L;FLJ35773	major facilitator superfamily domain containing 6-like	0.78	0.93	1.19	0.63
MYL12A	MYL12A;MLCB;MRCL3;MRLC3;MYL2B	myosin, light chain 12A, regulatory, non-sarcomeric	0.78	0.92	1.19	0.77
TRAPPC8	TRAPPC8;GSG1;HsT2706;KIAA1012;MGC71660;TRS85	trafficking protein particle complex 8	0.78	0.92	1.19	0.73
HIST2H2BE	HIST2H2BE;GL105;H2B;H2B.1;H2BFQ;H2BGL105;H2BQ;MGC119802;MGC119804;MGC129733;MGC129734	histone cluster 2, H2be	0.77	0.96	1.19	0.72
NUDT8	NUDT8;FLJ41567	nudix (nucleoside diphosphate linked moiety X)-type motif 8	0.77	0.97	1.19	0.65
MGST1	MGST1;GST12;MGC14525;MGST;MGST-I	microsomal glutathione S-transferase 1	0.77	0.85	1.19	0.68
ZNF746	ZNF746;FLJ31413;PARIS	zinc finger protein 746	0.77	0.86	1.19	0.7
SYTL5	SYTL5;slp5	synaptotagmin-like 5	0.76	0.88	1.19	0.67
HSD17B2	HSD17B2;EDH17B2;HSD17;SDR9C2	hydroxysteroid (17-beta) dehydrogenase 2	0.76	0.84	1.19	0.67
ZNF418	ZNF418;FLJ31551;KIAA1956;MGC138449	zinc finger protein 418	0.76	0.96	1.19	0.74
ATPBD4	ATPBD4;MGC14798	ATP binding domain 4	0.76	0.97	1.19	0.75
HS3ST2	HS3ST2;30ST2;3OST2	heparan sulfate (glucosamine) 3-O-sulfotransferase 2	0.76	0.86	1.19	0.68
TCF3	TCF3;E2A;E47;ITF1;MGC129647;MGC129648;TCF-3;VDIR;bHLHb21	transcription factor 3 (E2A immunoglobulin enhancer binding factors E12/E47)	0.76	0.88	1.19	0.69
CENPF	CENPF;CENF;PRO1779;hcp-1	centromere protein F, 350/400kDa (mitosin)	0.76	0.93	1.19	0.71
PMPCB	PMPCB;Beta-MPP;MPP11;MPPB;MPPP52;P-52	peptidase (mitochondrial processing) beta	0.76	0.9	1.19	0.67
METTL16	METTL16;METT10D;MGC3329	methyltransferase like 16	0.76	0.92	1.19	0.69
OR8U8	OR8U8	olfactory receptor, family 8, subfamily U, member 8	0.76	0.92	1.19	0.64
KCNJ8	KCNJ8;KIR6.1;uKATP-1	potassium inwardly-rectifying channel, subfamily J, member 8	0.75	0.93	1.19	0.72
FAM91A1	FAM91A1;DKFZp666B104;FLJ23790	family with sequence similarity 91, member A1	0.75	0.84	1.19	0.68
CIDEC	CIDEC;CIDE-3;FLJ20871;FSP27	cell death-inducing DFFA-like effector c	0.74	0.89	1.19	0.67
KRT37	KRT37;HA7;K37;KRTHA7	keratin 37	0.74	0.86	1.19	0.65
ZBTB43	ZBTB43;ZBTB22B;ZNF-X;ZNF297B	zinc finger and BTB domain containing 43	0.74	0.84	1.19	0.68
C2orf44	C2orf44;FLJ21945	chromosome 2 open reading frame 44	0.74	0.91	1.19	0.65
TEAD4	TEAD4;EFTR-2;MGC9014;RTEF1;TCF13L1;TEF-3;TEF3;TEFR-1;hRTEF-1B	TEA domain family member 4	0.74	0.89	1.19	0.69
HTR6	HTR6;5-HT6;5-HT6R	5-hydroxytryptamine (serotonin) receptor 6	0.73	0.86	1.19	0.76
VPS37D	VPS37D;MGC35352;WBSCR24	vacuolar protein sorting 37 homolog D (S. cerevisiae)	0.73	0.94	1.19	0.72
TAF5L	TAF5L;PAF65B	TAF5-like RNA polymerase II, p300/CBP-associated factor (PCAF)-associated factor, 65kDa	0.73	0.95	1.19	0.66
NUBP1	NUBP1;MGC117406;MGC130052;MGC130053;NBP;NBP1	nucleotide binding protein 1	0.73	0.84	1.19	0.6
C19orf73	C19orf73;FLJ10490	chromosome 19 open reading frame 73	0.73	0.84	1.19	0.66
STEAP1	STEAP1;MGC19484;PRSS24;STEAP	six transmembrane epithelial antigen of the prostate 1	0.72	0.84	1.19	0.59
PDCD11	PDCD11;ALG-4;ALG4;KIAA0185;NFBP;RRP5	programmed cell death 11	0.72	0.88	1.19	0.59
WASL	WASL;DKFZp779G0847;MGC48327;N-WASP;NWASP	Wiskott-Aldrich syndrome-like	0.72	0.83	1.19	0.67
PIWIL3	PIWIL3;HIWI3	piwi-like 3 (Drosophila)	0.71	0.88	1.19	0.66
FER1L6	FER1L6;C8ORFK23	fer-1-like 6 (C. elegans)	0.71	0.81	1.19	0.62
NIPA2	NIPA2;MGC5466	non imprinted in Prader-Willi/Angelman syndrome 2	0.71	0.85	1.19	0.59
FAM110B	FAM110B;C8orf72;MGC39325	family with sequence similarity 110, member B	0.7	0.86	1.19	0.64
KIF3C	KIF3C	kinesin family member 3C	0.7	0.82	1.19	0.63
C10orf35	C10orf35	chromosome 10 open reading frame 35	0.7	0.86	1.19	0.62
KCNK5	KCNK5;FLJ11035;K2p5.1;TASK-2;TASK2	potassium channel, subfamily K, member 5	0.7	0.83	1.19	0.65
XPNPEP3	XPNPEP3;APP3;NPHPL1	X-prolyl aminopeptidase (aminopeptidase P) 3, putative	0.7	0.82	1.19	0.61
TNFRSF1A	TNFRSF1A;CD120a;FPF;MGC19588;TBP1;TNF-R;TNF-R-I;TNF-R55;TNFAR;TNFR1;TNFR55;TNFR60;p55;p55-R;p60	tumor necrosis factor receptor superfamily, member 1A	0.7	0.83	1.19	0.65
EIF1AX	EIF1AX;EIF1A;EIF1AP1;EIF4C;eIF-1A;eIF-4C	eukaryotic translation initiation factor 1A, X-linked	0.7	0.82	1.19	0.58
NEK8	NEK8;JCK;MGC138445;NEK12A;NPHP9	NIMA (never in mitosis gene a)- related kinase 8	0.69	0.82	1.19	0.67
LGALS3	LGALS3;CBP35;GAL3;GALBP;GALIG;L31;LGALS2;MAC2	lectin, galactoside-binding, soluble, 3	0.69	0.79	1.19	0.68
SPATA19	SPATA19;CT132;FLJ25851;SPAS1;spergen1	spermatogenesis associated 19	0.68	0.84	1.19	0.62
S100Z	S100Z;Gm625;S100-zeta	S100 calcium binding protein Z	0.68	0.78	1.19	0.62
SEMA6C	SEMA6C;SEMAY;m-SemaY;m-SemaY2	sema domain, transmembrane domain (TM), and cytoplasmic domain, (semaphorin) 6C	0.68	0.73	1.19	0.58
C14orf149	C14orf149;FLJ25436	chromosome 14 open reading frame 149	0.68	0.79	1.19	0.68
ST3GAL2	ST3GAL2;Gal-NAc6S;SIAT4B;ST3GALII;ST3GalA.2	ST3 beta-galactoside alpha-2,3-sialyltransferase 2	0.68	0.82	1.19	0.6
TRPM4	TRPM4;FLJ20041;PFHB1B;TRPM4B	transient receptor potential cation channel, subfamily M, member 4	0.68	0.85	1.19	0.66
TMEM55B	TMEM55B;C14orf9;DKFZp434M0519;MGC26684	transmembrane protein 55B	0.68	0.76	1.19	0.62

VPS11	VPS11;END1;PEP5;RNF108;hVPS11	vacuolar protein sorting 11 homolog (S. cerevisiae)	0.68	0.8	1.19	0.72
SPIRE1	SPIRE1;MGC150621;MGC150622;Spir-1	spire homolog 1 (Drosophila)	0.68	0.78	1.19	0.59
SOS2	SOS2;FLJ25596	son of sevenless homolog 2 (Drosophila)	0.68	0.84	1.19	0.63
DCAF4L2	DCAF4L2;FLJ35775;MGC133227;WDR21C	DDB1 and CUL4 associated factor 4-like 2	0.68	0.76	1.19	0.62
NEGR1	NEGR1;DMML2433;IGLON4;KILON;MGC46680;Ntra	neuronal growth regulator 1	0.67	0.82	1.19	0.63
NEUROD6	NEUROD6;Atoh2;MATH2;Math-2;NEX1M;bHLHa2	neurogenic differentiation 6	0.66	0.78	1.19	0.65
COL28A1	COL28A1;COL28	collagen, type XXVIII, alpha 1	0.66	0.71	1.19	0.55
TSSK6	TSSK6;CT72;FLJ24002;SSTK;TSSK4	testis-specific serine kinase 6	0.66	0.78	1.19	0.64
IHH	IHH;BDA1;HHG2	Indian hedgehog	0.66	0.77	1.19	0.58
THAP1	THAP1;DYT6;FLJ10477;MGC33014	THAP domain containing, apoptosis associated protein 1	0.66	0.77	1.19	0.64
EPS8L1	EPS8L1;DRC3;EPS8R1;FLJ20258;MGC23164;MGC4642	EPS8-like 1	0.66	0.82	1.19	0.61
NUMBL	NUMBL;CAG3A;CTG3a;NBL;NUMB-R;NUMBLIKE;NUMBR;TNRC23	numb homolog (Drosophila)-like	0.66	0.81	1.19	0.61
SENP2	SENP2;AXAM2;DKFZp762A2316;KIAA1331;SMT3IP2	SUMO1/sentrin/SMT3 specific peptidase 2	0.66	0.78	1.19	0.59
NUP160	NUP160;DKFZp686M14102;MGC150678;MGC150679	nucleoporin 160kDa	0.66	0.74	1.19	0.57
MRPL24	MRPL24;FLJ20917;L24mt;MGC22737;MGC9831;MRP-L18;MRP-L24	mitochondrial ribosomal protein L24	0.66	0.8	1.19	0.54
GAR1	GAR1;NOLA1	GAR1 ribonucleoprotein homolog (yeast)	0.66	0.84	1.19	0.65
CXCL12	CXCL12;IRH;PBSF;SCYB12;SDF1;SDF1A;SDF1B;TLSF;TPAR1	chemokine (C-X-C motif) ligand 12	0.65	0.7	1.19	0.56
ANKS3	ANKS3;FLJ32345;FLJ32767;KIAA1977	ankyrin repeat and sterile alpha motif domain containing 3	0.65	0.79	1.19	0.59
IBA57	IBA57;C1orf69;FLJ12734;FLJ13849	IBA57, iron-sulfur cluster assembly homolog (S. cerevisiae)	0.65	0.8	1.19	0.58
SLC27A1	SLC27A1;ACSVL5;FATP;FATP1;FLJ00336;MGC71751	solute carrier family 27 (fatty acid transporter), member 1	0.64	0.73	1.19	0.53
C3orf19	C3orf19;FLJ33839	chromosome 3 open reading frame 19	0.64	0.82	1.19	0.57
LOC389458	LOC389458;MGC35170	uncharacterized LOC389458	0.64	0.68	1.19	0.54
C11orf80	C11orf80;FLJ22531	chromosome 11 open reading frame 80	0.64	0.76	1.19	0.55
CCDC88A	CCDC88A;APE;DKFZp686D0630;GIRDIN;GIV;GRDN;HkRP1;KIAA1212	coiled-coil domain containing 88A	0.64	0.8	1.19	0.55
EML3	EML3;ELP95;FLJ35827;MGC111422	echinoderm microtubule associated protein like 3	0.64	0.74	1.19	0.57
MARK4	MARK4;FLJ12177;FLJ90097;KIAA1860;MARK4L;MARK4S;MARKL1;MARKL1L	MAP/microtubule affinity-regulating kinase 4	0.64	0.83	1.19	0.59
C5orf15	C5orf15;KCT2	chromosome 5 open reading frame 15	0.64	0.79	1.19	0.56
TMTC4	TMTC4;FLJ14624;FLJ22153	transmembrane and tetratricopeptide repeat containing 4	0.64	0.76	1.19	0.55
MIS18A	MIS18A;B28;C21orf45;C21orf46;FASP1;MIS18alpha;hMis18alpha	MIS18 kinetochore protein homolog A (S. pombe)	0.64	0.78	1.19	0.59
USP6	USP6;HRP1;TRE17;TRE2;Tre-2;USP6-short	ubiquitin specific peptidase 6 (Tre-2 oncogene)	0.63	0.76	1.19	0.56
C2	C2;CO2;DKFZp779M0311	complement component 2	0.63	0.69	1.19	0.52
AP3B2	AP3B2;DKFZp686D17136;NAPTB	adaptor-related protein complex 3, beta 2 subunit	0.63	0.68	1.19	0.55
SPATA17	SPATA17;IQCH;MSRG-11;MSRG11;RP11-144C20.1	spermatogenesis associated 17	0.62	0.73	1.19	0.54
PARD6G	PARD6G;FLJ45701;PAR-6G;PAR6gamma	par-6 partitioning defective 6 homolog gamma (C. elegans)	0.62	0.76	1.19	0.54
C9orf167	C9orf167;FLJ20245	chromosome 9 open reading frame 167	0.62	0.7	1.19	0.56
CLIP3	CLIP3;CLIPR-59;CLIPR59;DKFZp586N1922;FLJ33413;RSNL1	CAP-GLY domain containing linker protein 3	0.62	0.7	1.19	0.57
TCF7L1	TCF7L1;TCF-3;TCF3	transcription factor 7-like 1 (T-cell specific, HMG-box)	0.62	0.7	1.19	0.5
GORASP1	GORASP1;FLJ23443;GOLPH5;GRASP65;MGC118894;MGC118897;P65	golgi reassembly stacking protein 1, 65kDa	0.62	0.77	1.19	0.52
PELP1	PELP1;HMX3;MNAR;P160	proline, glutamate and leucine rich protein 1	0.62	0.8	1.19	0.59
NR2F6	NR2F6;EAR-2;EAR2;ERBAL2	nuclear receptor subfamily 2, group F, member 6	0.62	0.72	1.19	0.5
ZCCHC14	ZCCHC14;BDG-29;BDG29;MGC126527;MGC14139	zinc finger, CCHC domain containing 14	0.61	0.7	1.19	0.53
SCAF4	SCAF4;DKFZp434E098;FLJ23364;KIAA1172;SFRS15;SRA4	SR-related CTD-associated factor 4	0.61	0.76	1.19	0.56
PARP11	PARP11;C12orf6;DKFZp779H0122	poly (ADP-ribose) polymerase family, member 11	0.84	1	1.2	0.75
FUT5	FUT5;FUC-TV	fucosyltransferase 5 (alpha (1,3) fucosyltransferase)	0.84	0.96	1.2	0.75
PUSL1	PUSL1;FLJ90811	pseudouridylate synthase-like 1	0.84	0.98	1.2	0.72
ZNF184	ZNF184	zinc finger protein 184	0.84	0.98	1.2	0.77
SEPN1	SEPN1;FLJ24021;MDRS1;RSMD1;RSS;SELN	selenoprotein N, 1	0.84	0.9	1.2	0.76
HEATR2	HEATR2;FLJ20397;FLJ25564;FLJ31671;FLJ39381	HEAT repeat containing 2	0.84	1.08	1.2	0.83
FLYWCH2	FLYWCH2	FLYWCH family member 2	0.84	0.9	1.2	0.75
FOXC1	FOXC1;ARA;FKHL7;FREAC-3;FREAC3;IGDA;IHG1;IRID1;RIEG3	forkhead box C1	0.83	0.95	1.2	0.71
PGM3	PGM3;AGM1;DKFZp434B187;FLJ11614;FLJ13623;PAGM;PGM 3	phosphoglucomutase 3	0.83	0.96	1.2	0.69
MAPRE1	MAPRE1;EB1;MGC117374;MGC129946	microtubule-associated protein, RP/EB family, member 1	0.83	0.96	1.2	0.7
TMEM55A	TMEM55A;DKFZp762O076	transmembrane protein 55A	0.82	1.02	1.2	0.73
PDIA6	PDIA6;ERP5;P5;TXNDC7	protein disulfide isomerase family A, member 6	0.82	0.94	1.2	0.74
GGTLC1	GGTLC1;GGTL6;GGTLA3;GGTLA4;MGC50550;dJ831C21.1;dJ831C21.2	gamma-glutamyltransferase light chain 1	0.81	1.02	1.2	0.83
YPEL1	YPEL1;MGC64992	yippee-like 1 (Drosophila)	0.81	0.88	1.2	0.7
TNXB	TNXB;HXBL;TENX;TN-X;TNX;TNXB1;TNXB2;TNXBS;XB;XBS	tenascin XB	0.81	0.99	1.2	0.79
BBS5	BBS5	Bardet-Biedl syndrome 5	0.81	0.98	1.2	0.68
SULT1B1	SULT1B1;MGC13356;ST1B1;ST1B2;SULT1B2	sulfotransferase family, cytosolic, 1B, member 1	0.8	0.98	1.2	0.77
MAMLD1	MAMLD1;CG1;CXorf6;F18;HYSP2	mastermind-like domain containing 1	0.8	0.92	1.2	0.66
SPRED3	SPRED3;Eve-3	sprouty-related, EVH1 domain containing 3	0.8	0.99	1.2	0.71
ZNF436	ZNF436;KIAA1710;ZNF;Zfp46	zinc finger protein 436	0.8	1	1.2	0.75
DPP9	DPP9;DKFZp762F117;DP9;DPLP9;DPRP-2;DPRP2;FLJ16073	dipeptidyl-peptidase 9	0.8	0.88	1.2	0.66
GHITM	GHITM;DERP2;DKFZp566C0746;FLJ26584;HSPC282;MICS1;PTD010;TMBIM5	growth hormone inducible transmembrane protein	0.8	1	1.2	0.68
IDO1	IDO1;IDO;IDO-1;INDO	indoleamine 2,3-dioxygenase 1	0.8	1.04	1.2	0.73
C17orf47	C17orf47;FLJ40121	chromosome 17 open reading frame 47	0.79	1.01	1.2	0.71
HSPB11	HSPB11;C1orf41;HSPCO34;IFT25;PP25	heat shock protein family B (small), member 11	0.79	0.93	1.2	0.69
AP1G2	AP1G2;G2AD	adaptor-related protein complex 1, gamma 2 subunit	0.79	0.97	1.2	0.65
PLEKHA1	PLEKHA1;TAPP1	pleckstrin homology domain containing, family A (phosphoinositide binding specific) member 1	0.79	0.92	1.2	0.74
PRKAR1A	PRKAR1A;ADOHR;CAR;CNC;CNC1;DKFZp779L0468;MGC17251;PKR1;PPNAD1;PRKAR1;TSE1	protein kinase, cAMP-dependent, regulatory, type I, alpha (tissue specific extinguisher 1)	0.79	0.95	1.2	0.78
MYPOP	MYPOP;P42pop	Myb-related transcription factor, partner of profilin	0.79	0.85	1.2	0.68
SV2C	SV2C;MGC118874;MGC118875;MGC118876;MGC118877	synaptic vesicle glycoprotein 2C	0.78	0.91	1.2	0.66
EGR2	EGR2;AT591;CMT1D;CMT4E;DKFZp686J1957;FLJ14547;KROX20	early growth response 2	0.78	0.91	1.2	0.71
MAPK4	MAPK4;ERK3;Erk4;PRKM4;p63MAPK	mitogen-activated protein kinase 4	0.78	0.94	1.2	0.78
NR6A1	NR6A1;GCNF;GCNF1;NR61;RTR	nuclear receptor subfamily 6, group A, member 1	0.78	1.01	1.2	0.76
NCR1	NCR1;CD335;FLJ99094;LY94;NK-p46;NKP46	natural cytotoxicity triggering receptor 1	0.78	0.89	1.2	0.7
G0S2	G0S2;RP1-28O10.2	G0/G1switch 2	0.78	0.94	1.2	0.66
C8orf58	C8orf58;FLJ34715	chromosome 8 open reading frame 58	0.78	0.96	1.2	0.73
TRAK2	TRAK2;ALS2CR3;CALS-C;GRIF-1;GRIF1;KIAA0549;MILT2;OIP98	trafficking protein, kinesin binding 2	0.78	0.87	1.2	0.69
TRIM27	TRIM27;RFP;RNF76	tripartite motif containing 27	0.78	0.93	1.2	0.77
USP33	USP33;KIAA1097;MGC16868;VDU1	ubiquitin specific peptidase 33	0.78	0.93	1.2	0.7
PCSK6	PCSK6;PACE4;SPC4	proprotein convertase subtilisin/kexin type 6	0.77	0.92	1.2	0.69
FAM19A3	FAM19A3;MGC138473;TAFA-3;TAFA3	family with sequence similarity 19 (chemokine (C-C motif)-like), member A3	0.77	0.88	1.2	0.71
BCAR3	BCAR3;KIAA0554;NSP2;SH2D3B	breast cancer anti-estrogen resistance 3	0.77	0.88	1.2	0.68
SMARCD1	SMARCD1;BAF60A;CRACD1;Rsc6p	SWI/SNF related, matrix associated, actin dependent regulator of chromatin, subfamily d, member 1	0.77	0.99	1.2	0.66
METAP2	METAP2;MAP2;MNPEP;p67;p67eIF2	methionyl aminopeptidase 2	0.77	0.92	1.2	0.69
FLJ46838	FLJ46838	FLJ46838 protein	0.76	0.82	1.2	0.69
LAMP3	LAMP3;CD208;DC LAMP;DC-LAMP;DCLAMP;LAMP;LAMP-3;TSC403	lysosomal-associated membrane protein 3	0.76	0.88	1.2	0.69
MTX3	MTX3;DKFZp686O1788	metaxin 3	0.76	0.95	1.2	0.68
JOSD2	JOSD2;FLJ29018	Josephin domain containing 2	0.76	1	1.2	0.66
LDLRAP1	LDLRAP1;ARH;ARH1;ARH2;DKFZp586D0624;FHCB1;FHCB2;MGC34705	low density lipoprotein receptor adaptor protein 1	0.76	0.9	1.2	0.77
PKP2	PKP2;ARVD9;MGC177501	plakophilin 2	0.76	0.88	1.2	0.67
C12orf62	C12orf62;MGC14288	chromosome 12 open reading frame 62	0.75	0.9	1.2	0.65
DCTN4	DCTN4	dynactin 4 (p62)	0.75	0.92	1.2	0.7
C4orf3	C4orf3	chromosome 4 open reading frame 3	0.75	0.85	1.2	0.72
DEPDC4	DEPDC4;DEP.4;FLJ33505	DEP domain containing 4	0.74	0.92	1.2	0.63
RASGRP2	RASGRP2;CALDAG-GEFI;CDC25L	RAS guanyl releasing protein 2 (calcium and DAG-regulated)	0.74	0.84	1.2	0.71
NR1D1	NR1D1;EAR1;THRA1;THRAL;ear-1;hRev	nuclear receptor subfamily 1, group D, member 1	0.74	0.9	1.2	0.7
HS1BP3	HS1BP3;ETM2;FLJ14249;HS1-BP3	HCLS1 binding protein 3	0.74	0.94	1.2	0.67
HPS4	HPS4;KIAA1667;LE;bK1048E9.4;bK1048E9.5	Hermansky-Pudlak syndrome 4	0.74	0.91	1.2	0.66
GOLGA8B	GOLGA8B;GOLGA5;KIAA0855	golgin A8 family, member B	0.74	0.8	1.2	0.64
PPIL2	PPIL2;CYC4;CYP60;Cyp-60;FLJ39930;MGC33174;MGC787;hCyP-60	peptidylprolyl isomerase (cyclophilin)-like 2	0.74	0.88	1.2	0.79
PFDN5	PFDN5;MGC5329;MGC71907;MM-1;MM1;PFD5	prefoldin subunit 5	0.74	0.84	1.2	0.73
PERP	PERP;KCP1;KRTCAP1;PIGPC1;RP3-496H19.1;THW;dJ496H19.1	PERP, TP53 apoptosis effector	0.74	0.92	1.2	0.62
NAPB	NAPB;MGC26066;MGC48335;SNAP-BETA;SNAPB	N-ethylmaleimide-sensitive factor attachment protein, beta	0.73	0.92	1.2	0.72
PTGER4	PTGER4;EP4;EP4R;MGC126583	prostaglandin E receptor 4 (subtype EP4)	0.73	0.84	1.2	0.76
APOE	APOE;AD2;LDLCQ5;LPG;MGC1571	apolipoprotein E	0.73	0.84	1.2	0.63
GALE	GALE;FLJ95174;FLJ97302;SDR1E1	UDP-galactose-4-epimerase	0.73	0.82	1.2	0.65
LAS1L	LAS1L;FLJ12525;FLJ30133;FLJ45274;Las1-like;dJ475B7.2	LAS1-like (S. cerevisiae)	0.73	0.84	1.2	0.64
NCSTN	NCSTN;APH2;KIAA0253	nicastrin	0.73	0.9	1.2	0.69
CMTM2	CMTM2;CKLFSF2;MGC39436	CKLF-like MARVEL transmembrane domain containing 2	0.72	0.84	1.2	0.64
NTNG1	NTNG1;KIAA0976;Lmnt1	netrin G1	0.72	0.84	1.2	0.63
KAZALD1	KAZALD1;BONO1;FKSG28;FKSG40;FLJ24094;IGFBP-rP10	Kazal-type serine peptidase inhibitor domain 1	0.72	0.84	1.2	0.64
ZSCAN5A	ZSCAN5A;MGC4161;ZNF495;ZSCAN5	zinc finger and SCAN domain containing 5A	0.72	0.94	1.2	0.63
DPY19L3	DPY19L3;DKFZp686J17135;MGC35440	dpy-19-like 3 (C. elegans)	0.72	0.86	1.2	0.62
C10orf47	C10orf47;MGC35403	chromosome 10 open reading frame 47	0.72	0.9	1.2	0.63
FAHD2A	FAHD2A;FLJ35661;MGC131995	fumarylacetoacetate hydrolase domain containing 2A	0.72	0.82	1.2	0.64
MRPL21	MRPL21;L21mt;MGC62013;MRP-L21	mitochondrial ribosomal protein L21	0.72	0.89	1.2	0.59
PNPO	PNPO;FLJ10535;PDXPO	pyridoxamine 5'-phosphate oxidase	0.72	0.86	1.2	0.67
UIMC1	UIMC1;RAP80;X2HRIP110	ubiquitin interaction motif containing 1	0.72	0.84	1.2	0.62
LARS	LARS;FLJ10595;FLJ21788;HSPC192;KIAA1352;LARS1;LEURS;LEUS;LRS;PIG44;RNTLS;hr025Cl	leucyl-tRNA synthetase	0.72	0.86	1.2	0.58
ITGA1	ITGA1;CD49a;VLA1	integrin, alpha 1	0.72	0.89	1.2	0.68
CCDC74B	CCDC74B;DKFZp434E2321	coiled-coil domain containing 74B	0.72	0.84	1.2	0.63
ARL16	ARL16	ADP-ribosylation factor-like 16	0.72	0.87	1.2	0.63
BMS1	BMS1;BMS1L;KIAA0187	BMS1 homolog, ribosome assembly protein (yeast)	0.71	0.84	1.2	0.62
GABPB2	GABPB2;MGC29891	GA binding protein transcription factor, beta subunit 2	0.71	0.82	1.2	0.65
ZNF709	ZNF709;FLJ38281	zinc finger protein 709	0.7	0.81	1.2	0.62
C6orf170	C6orf170;BROMI;C6orf171;FLJ30899;FLJ34235;bA301B7.2;bA57L9.1;dJ310J6.1	chromosome 6 open reading frame 170	0.7	0.76	1.2	0.61
C9orf139	C9orf139;FLJ36268;FLJ42909	chromosome 9 open reading frame 139	0.7	0.91	1.2	0.7

ARHGEF16	ARHGEF16;GEF16;NBR	Rho guanine nucleotide exchange factor (GEF) 16	0.7	0.86	1.2	0.62
BAI2	BAI2	brain-specific angiogenesis inhibitor 2	0.7	0.82	1.2	0.73
ANAPC10	ANAPC10;APC10;DKFZp564L0562;DOC1	anaphase promoting complex subunit 10	0.7	0.86	1.2	0.66
EIF2B5	EIF2B5;CACH;CLE;EIF-2B;EIF2Bepsilon;LVWM	eukaryotic translation initiation factor 2B, subunit 5 epsilon, 82kDa	0.7	0.81	1.2	0.71
FAM43B	FAM43B;FLJ44952	family with sequence similarity 43, member B	0.7	0.9	1.2	0.63
MAD2L1	MAD2L1;HSMAD2;MAD2	MAD2 mitotic arrest deficient-like 1 (yeast)	0.7	0.82	1.2	0.57
GTF2I	GTF2I;BAP135;BTKAP1;DIWS;FLJ38776;FLJ56355;GTFII-I;IB291;SPIN;TFII-I;WBS;WBSCR6	general transcription factor IIi	0.7	0.84	1.2	0.57
C4orf44	C4orf44	chromosome 4 open reading frame 44	0.69	0.86	1.2	0.64
SPOCK2	SPOCK2;FLJ97039;testican-2	sparc/osteonectin, cwcv and kazal-like domains proteoglycan (testican) 2	0.69	0.86	1.2	0.58
ZBTB6	ZBTB6;ZID;ZNF482	zinc finger and BTB domain containing 6	0.69	0.82	1.2	0.59
SHMT1	SHMT1;CSHMT;MGC15229;MGC24556;SHMT	serine hydroxymethyltransferase 1 (soluble)	0.69	0.8	1.2	0.63
GKAP1	GKAP1;FLJ25469;GKAP42	G kinase anchoring protein 1	0.68	0.82	1.2	0.55
KIAA1024	KIAA1024	KIAA1024	0.68	0.79	1.2	0.62
AIM1	AIM1;CRYBG1;ST4	absent in melanoma 1	0.68	0.8	1.2	0.61
RSF1	RSF1;HBXAP;RSF-1;XAP8;p325	remodeling and spacing factor 1	0.68	0.79	1.2	0.71
CINP	CINP;MGC849	cyclin-dependent kinase 2 interacting protein	0.68	0.78	1.2	0.6
TMOD1	TMOD1;D9S57E;ETMOD;TMOD	tropomodulin 1	0.68	0.76	1.2	0.65
MTDH	MTDH;3D3;AEG-1;AEG1;LYRIC;LYRIC/3D3	metadherin	0.68	0.79	1.2	0.6
HSFY1	HSFY1;HSF2L;HSFY;HSFY2	heat shock transcription factor, Y-linked 1	0.68	0.79	1.2	0.68
SPAG11A	SPAG11A;EDDM2A;HE2	sperm associated antigen 11A	0.68	0.84	1.2	0.64
C15orf63	C15orf63;HYPK	chromosome 15 open reading frame 63	0.68	0.84	1.2	0.6
LAX1	LAX1;FLJ20340;LAX	lymphocyte transmembrane adaptor 1	0.66	0.75	1.2	0.63
NKD1	NKD1;FLJ11639;FLJ13722;FLJ20269;Naked1	naked cuticle homolog 1 (Drosophila)	0.66	0.78	1.2	0.65
PTN	PTN;HARP;HBGF8;HBNF;NEGF1	pleiotrophin	0.66	0.78	1.2	0.61
RG9MTD2	RG9MTD2;FLJ20325;MGC27034;TRM10	RNA (guanine-9-) methyltransferase domain containing 2	0.66	0.8	1.2	0.62
EXOSC7	EXOSC7;EAP1;FLJ26543;KIAA0116;RRP42;Rrp42p;hRrp42p;p8	exosome component 7	0.66	0.77	1.2	0.54
TMEM199	TMEM199;C17orf32;MGC45714	transmembrane protein 199	0.66	0.75	1.2	0.64
TTC1	TTC1;FLJ46404;TPR1	tetratricopeptide repeat domain 1	0.66	0.81	1.2	0.54
FKBP15	FKBP15;FKBP133;KIAA0674;PPP1R76	FK506 binding protein 15, 133kDa	0.65	0.79	1.2	0.53
FAM125B	FAM125B;C9orf28;FLJ00001;MVB12B	family with sequence similarity 125, member B	0.64	0.77	1.2	0.58
NXT2	NXT2;P15-2	nuclear transport factor 2-like export factor 2	0.64	0.75	1.2	0.59
RAD50	RAD50;NBSLD;RAD502;hRad50	RAD50 homolog (S. cerevisiae)	0.64	0.74	1.2	0.53
DEFB4A	DEFB4A;BD-2;DEFB-2;DEFB102;DEFB2;DEFB4;HBD-2;SAP1	defensin, beta 4A	0.64	0.79	1.2	0.59
ARVCF	ARVCF;FLJ35345	armadillo repeat gene deleted in velocardiofacial syndrome	0.63	0.76	1.2	0.53
RNF215	RNF215	ring finger protein 215	0.63	0.74	1.2	0.66
KCNH8	KCNH8;ELK;ELK1;Kv12.1;elk3	potassium voltage-gated channel, subfamily H (eag-related), member 8	0.62	0.75	1.2	0.58
LGALS9C	LGALS9C;MGC125972	lectin, galactoside-binding, soluble, 9C	0.62	0.76	1.2	0.52
TIGD1	TIGD1;EEYORE	tigger transposable element derived 1	0.62	0.71	1.2	0.51
EPN1	EPN1	epsin 1	0.62	0.78	1.2	0.58
DPAGT1	DPAGT1;ALG7;CDG-Ij;CDG1J;D11S366;DGPT;DPAGT;DPAGT2;G1PT;GPT;UAGT;UGAT	dolichyl-phosphate (UDP-N-acetylglucosamine) N-acetylglucosaminephosphotransferase 1 (GlcNAc-1-P transferase)	0.62	0.82	1.2	0.61
TSPEAR	TSPEAR;C21orf29;MGC11251	thrombospondin-type laminin G domain and EAR repeats	0.62	0.72	1.2	0.55
SDHC	SDHC;CYB560;CYBL;PGL3;QPS1;SDH3	succinate dehydrogenase complex, subunit C, integral membrane protein, 15kDa	0.61	0.7	1.2	0.51
ERGIC1	ERGIC1;ERGIC-32;ERGIC32;FLJ39864;KIAA1181;MGC14345;NET24	endoplasmic reticulum-golgi intermediate compartment (ERGIC) 1	0.61	0.78	1.2	0.55
HNRNPAB	HNRNPAB;ABBP1;FLJ40338;HNRPAB	heterogeneous nuclear ribonucleoprotein A/B	0.61	0.71	1.2	0.56
ZNF135	ZNF135;ZNF61;ZNF78L1;pHZ-17;pT3	zinc finger protein 135	0.84	1.01	1.21	0.69
TRMT1L	TRMT1L;C1orf25;MGC25112;MGC57134;MST070;TRM1L;bG120K12.3	TRM1 tRNA methyltransferase 1-like	0.84	0.94	1.21	0.83
FBP2	FBP2;MGC142192	fructose-1,6-bisphosphatase 2	0.83	0.94	1.21	0.71
APH1B	APH1B;APH-1B;DKFZp564D0372;FLJ33115;PRO1328;PSFL;TAAV688	anterior pharynx defective 1 homolog B (C. elegans)	0.83	0.93	1.21	0.69
TADA3	TADA3;ADA3;FLJ20221;FLJ21329;NGG1;TADA3L;hADA3	transcriptional adaptor 3	0.83	0.94	1.21	0.82
C3orf70	C3orf70	chromosome 3 open reading frame 70	0.82	0.96	1.21	0.77
HEPN1	HEPN1	hepatocellular carcinoma, down-regulated 1	0.82	1.05	1.21	0.74
STAB1	STAB1;CLEVER-1;FEEL-1;FELE-1;FEX1;KIAA0246;STAB-1	stabilin 1	0.82	1	1.21	0.67
TDRD3	TDRD3;FLJ21007	tudor domain containing 3	0.82	0.98	1.21	0.67
OVOL1	OVOL1;HOVO1	ovo-like 1(Drosophila)	0.82	0.95	1.21	0.69
SNX3	SNX3;Grd19;MCOPS8;MGC17570;SDP3	sorting nexin 3	0.82	1.01	1.21	0.77
PPFIA1	PPFIA1;FLJ41337;FLJ42630;FLJ43474;LIP.1;LIP1;LIPRIN;MGC26800	protein tyrosine phosphatase, receptor type, f polypeptide (PTPRF), interacting protein (liprin), alpha 1	0.82	0.92	1.21	0.69
PCDH19	PCDH19;DKFZp686P1843;EFMR;EIEE9;KIAA1313	protocadherin 19	0.8	1.02	1.21	0.71
CIDEA	CIDEA;CIDE-A	cell death-inducing DFFA-like effector a	0.8	0.92	1.21	0.77
REP15	REP15;RAB15EP	RAB15 effector protein	0.8	1	1.21	0.67
PPM1K	PPM1K;DKFZp667B084;DKFZp761G058;PP2Ckappa;PP2Cm;PTMP;UG0882E07;hPTMP	protein phosphatase, Mg2+/Mn2+ dependent, 1K	0.8	0.9	1.21	0.68
RNASEL	RNASEL;DKFZp781D08126;MGC104972;MGC133329;PRCA1;RNS4	ribonuclease L (2',5'-oligoisoadenylate synthetase-dependent)	0.8	0.98	1.21	0.81
MCM9	MCM9;C6orf61;FLJ13942;FLJ20170;FLJ56845;MCMDC1;MGC35304;dJ329L24.1;dJ329L24.3	minichromosome maintenance complex component 9	0.8	1.02	1.21	0.73
TMEM186	TMEM186;C16orf51;DKFZp564K2062	transmembrane protein 186	0.8	0.94	1.21	0.72
NDUFS6	NDUFS6;CI-13kA;CI-13kD-A;CI13KDA	NADH dehydrogenase (ubiquinone) Fe-S protein 6, 13kDa (NADH-coenzyme Q reductase)	0.8	0.97	1.21	0.75
B4GALT1	B4GALT1;B4GAL-T1;CDG2D;DKFZp686N19253;GGTB2;GT1;GTB;MGC50983;beta4Gal-T1	UDP-Gal:betaGlcNAc beta 1,4- galactosyltransferase, polypeptide 1	0.8	0.97	1.21	0.72
PPP2R5A	PPP2R5A;B56A;MGC131915;PR61A	protein phosphatase 2, regulatory subunit B', alpha	0.8	0.96	1.21	0.75
SNX20	SNX20;MGC35578;SLIC1	sorting nexin 20	0.79	0.98	1.21	0.74
FATE1	FATE1;CT43;FATE	fetal and adult testis expressed 1	0.78	0.97	1.21	0.73
IGFL2	IGFL2;UNQ645;VPRI645	IGF-like family member 2	0.78	0.9	1.21	0.7
TMEM99	TMEM99;MGC21518	transmembrane protein 99	0.78	0.93	1.21	0.69
CCDC71	CCDC71;FLJ12800;FLJ13629	coiled-coil domain containing 71	0.78	0.97	1.21	0.72
RAD17	RAD17;CCYC;FLJ41520;HRAD17;R24L;RAD17SP;RAD24	RAD17 homolog (S. pombe)	0.78	0.95	1.21	0.74
ZDHHC4	ZDHHC4;FLJ10479;ZNF374	zinc finger, DHHC-type containing 4	0.78	0.91	1.21	0.72
DPY19L4	DPY19L4;MGC131885	dpy-19-like 4 (C. elegans)	0.78	1	1.21	0.7
RBBP7	RBBP7;MGC138867;MGC138868;RbAp46	retinoblastoma binding protein 7	0.78	0.9	1.21	0.65
ZNF385B	ZNF385B;FLJ25270;ZFN533;ZNF533	zinc finger protein 385B	0.77	0.88	1.21	0.75
RICTOR	RICTOR;DKFZp686B11164;KIAA1999;MGC39830;PIA;mAVO3	RPTOR independent companion of MTOR, complex 2	0.77	0.91	1.21	0.69
NHLRC3	NHLRC3;DKFZp313M1221;DKFZp686E1140	NHL repeat containing 3	0.77	0.94	1.21	0.68
TTN	TTN;CMD1G;CMH9;CMPD4;DKFZp451N061;EOMFC;FLJ26020;FLJ26409;FLJ32040;FLJ34413;FLJ39564;FLJ43066;HMERF;LGMD2J;MYLK5;TMD	titin	0.76	0.88	1.21	0.65
SLAIN1	SLAIN1;C13orf32;FLJ30046;MGC131899	SLAIN motif family, member 1	0.76	0.94	1.21	0.7
DNAJB3	DNAJB3;HCG3;MGC26879	DnaJ (Hsp40) homolog, subfamily B, member 3	0.76	0.84	1.21	0.67
WDR35	WDR35;CED2;KIAA1336;MGC33196	WD repeat domain 35	0.76	0.88	1.21	0.67
DENND1A	DENND1A;FAM31A;FLJ38464;KIAA1608;RP11-230L22.3	DENN/MADD domain containing 1A	0.76	0.86	1.21	0.7
GLA	GLA;GALA	galactosidase, alpha	0.76	0.87	1.21	0.68
CCL3	CCL3;G0S19-1;LD78ALPHA;MIP-1-alpha;MIP1A;SCYA3	chemokine (C-C motif) ligand 3	0.75	0.95	1.21	0.63
RMND5B	RMND5B;DKFZp434K0926;FLJ22318	required for meiotic nuclear division 5 homolog B (S. cerevisiae)	0.75	0.96	1.21	0.68
PAFAH1B2	PAFAH1B2	platelet-activating factor acetylhydrolase 1b, catalytic subunit 2 (30kDa)	0.75	0.89	1.21	0.63
GCK	GCK;FGQTL3;GK;GLK;HHF3;HK4;HKIV;HXKP;LGLK;MODY2	glucokinase (hexokinase 4)	0.74	0.98	1.21	0.69
GPER	GPER;CEPR;CMKRL2;DRY12;FEG-1;GPCR-Br;GPR30;LERGU;LERGU2;LyGPR;MGC99678	G protein-coupled estrogen receptor 1	0.74	0.86	1.21	0.78
ZNF200	ZNF200;MGC45293	zinc finger protein 200	0.74	0.98	1.21	0.72
WWC3	WWC3;BM042;KIAA1280	WWC family member 3	0.74	0.88	1.21	0.65
TOP3A	TOP3A;TOP3	topoisomerase (DNA) III alpha	0.74	0.86	1.21	0.62
NFIX	NFIX;NF1A	nuclear factor I/X (CCAAT-binding transcription factor)	0.74	0.86	1.21	0.64
HMOX2	HMOX2;HO-2	heme oxygenase (decycling) 2	0.74	0.83	1.21	0.66
CSDE1	CSDE1;D1S155E;DKFZp779B0247;DKFZp779J1455;FLJ26882;RP5-1000E10.3;UNR	cold shock domain containing E1, RNA-binding	0.74	0.88	1.21	0.62
ARHGEF26	ARHGEF26;CSGEF;DKFZp434D146;HMFN1864;SGEF	Rho guanine nucleotide exchange factor (GEF) 26	0.74	0.92	1.21	0.7
CCR6	CCR6;BN-1;C-C CKR-6;CC-CKR-6;CCR-6;CD196;CKR-L3;CKRL3;CMKBR6;DCR2;DRY6;GPR29;GPRCY4;STRL22	chemokine (C-C motif) receptor 6	0.73	0.84	1.21	0.77
ARID5A	ARID5A;MRF-1;MRF1;RP11-363D14	AT rich interactive domain 5A (MRF1-like)	0.73	0.92	1.21	0.68
RELT	RELT;FLJ14993;TNFRSF19L	RELT tumor necrosis factor receptor	0.73	0.88	1.21	0.65
N4BP2L2	N4BP2L2;92M18.3;CG005;FLJ36195;FLJ41089;FLJ43077;PFAAP5	NEDD4 binding protein 2-like 2	0.73	0.94	1.21	0.62
SFMBT2	SFMBT2	Scm-like with four mbt domains 2	0.72	0.9	1.21	0.64
NAP1L2	NAP1L2;BPX;MGC26243	nucleosome assembly protein 1-like 2	0.72	0.91	1.21	0.68
GABBR2	GABBR2;FLJ36928;GABABR2;GPR51;GPRC3B;HG20;HRIHFB2099	gamma-aminobutyric acid (GABA) B receptor, 2	0.72	0.85	1.21	0.76
TUT1	TUT1;FLJ21850;FLJ22267;FLJ22347;MGC131987;MGC149809;PAPD2;RBM21;STARPAP;TUTase	terminal uridylyl transferase 1, U6 snRNA-specific	0.72	0.91	1.21	0.65
MED16	MED16;DRIP92;THRAP5;TRAP95	mediator complex subunit 16	0.72	0.84	1.21	0.72
RG9MTD1	RG9MTD1;FLJ20432;MRPP1	RNA (guanine-9-) methyltransferase domain containing 1	0.72	0.89	1.21	0.68
CHD1L	CHD1L;ALC1;CHDL;FLJ22530	chromodomain helicase DNA binding protein 1-like	0.72	0.93	1.21	0.71
CGN	CGN;DKFZp779N1112;FLJ39281;KIAA1319	cingulin	0.72	0.9	1.21	0.61
LPPR1	LPPR1;MGC26189;PRG-3	lipid phosphate phosphatase-related protein type 1	0.72	0.92	1.21	0.65
MOG	MOG;MGC26137;MOGIG2;NRCLP7	myelin oligodendrocyte glycoprotein	0.71	0.9	1.21	0.6
PTF1A	PTF1A;PTF1-p48;bHLHa29	pancreas specific transcription factor, 1a	0.71	0.88	1.21	0.61
C22orf42	C22orf42;dJ90G24.6	chromosome 22 open reading frame 42	0.71	0.84	1.21	0.63
LGI3	LGI3;LGIL4	leucine-rich repeat LGI family, member 3	0.7	0.8	1.21	0.68
LPCAT2	LPCAT2;AYTL1;DKFZp686H22112;FLJ20481;LysoPAFAT	lysophosphatidylcholine acyltransferase 2	0.7	0.88	1.21	0.63
STK38	STK38;NDR;NDR1	serine/threonine kinase 38	0.7	0.9	1.21	0.64
FUT8	FUT8;MGC26465	fucosyltransferase 8 (alpha (1,6) fucosyltransferase)	0.7	0.79	1.21	0.7
ESYT3	ESYT3;CHR3SYT;E-Syt3;FAM62C	extended synaptotagmin-like protein 3	0.7	0.88	1.21	0.62
CRB1	CRB1;LCA8;RP12	crumbs homolog 1 (Drosophila)	0.69	0.86	1.21	0.65
LGALS12	LGALS12;GAL12;GRIP1	lectin, galactoside-binding, soluble, 12	0.69	0.82	1.21	0.64
SP7	SP7;MGC126598;OI11;OSX;osterix	Sp7 transcription factor	0.69	0.9	1.21	0.67
PPP1R18	PPP1R18;HKMT1098;KIAA1949	protein phosphatase 1, regulatory subunit 18	0.69	0.84	1.21	0.61
PAH	PAH;PH;PKU;PKU1	phenylalanine hydroxylase	0.68	0.8	1.21	0.62
ZNF597	ZNF597;FLJ33071	zinc finger protein 597	0.68	0.84	1.21	0.64
SNAI2	SNAI2;MGC10182;SLUG;SLUGH1;SNAIL2;WS2D	snail homolog 2 (Drosophila)	0.68	0.82	1.21	0.57
RBM43	RBM43;C2orf38;FLJ45645	RNA binding motif protein 43	0.68	0.8	1.21	0.61
GPR182	GPR182;7TMR;ADMR;AM-R;AMR;G10D;MGC34399;gamrh;hrhAMR	G protein-coupled receptor 182	0.68	0.76	1.21	0.58
PLA2G12B	PLA2G12B;GXIIB;GXIIIsPLA2;MGC138151;PLA2G13	phospholipase A2, group XIIB	0.68	0.86	1.21	0.57
MIF4GD	MIF4GD;AD023;MGC45027;MIFD;SLIP1	MIF4G domain containing	0.68	0.88	1.21	0.61
ARL2	ARL2;ARFL2	ADP-ribosylation factor-like 2	0.68	0.8	1.21	0.57

PBRM1	PBRM1;BAF180;MGC156155;MGC156156;PB1	polybromo 1	0.68	0.84	1.21	0.6
EIF4H	EIF4H;KIAA0038;WBSCR1;WSCR1;eIF-4H	eukaryotic translation initiation factor 4H	0.68	0.82	1.21	0.63
GNB1	GNB1	guanine nucleotide binding protein (G protein), beta polypeptide 1	0.68	0.86	1.21	0.57
NDN	NDN;HsT16328;PWCR	necdin homolog (mouse)	0.67	0.72	1.21	0.61
LELP1	LELP1	late cornified envelope-like proline-rich 1	0.67	0.87	1.21	0.61
TBCD	TBCD;KIAA0988	tubulin folding cofactor D	0.67	0.72	1.21	0.58
B3GNT8	B3GNT8;B3GALT7;BGALT15;beta3Gn-T8	UDP-GlcNAc:betaGal beta-1,3-N-acetylglucosaminyltransferase 8	0.66	0.77	1.21	0.6
SMARCAL1	SMARCAL1;HARP;HHARP	SWI/SNF related, matrix associated, actin dependent regulator of chromatin, subfamily a-like 1	0.66	0.74	1.21	0.59
GXYLT2	GXYLT2;GLT8D4	glucoside xylosyltransferase 2	0.66	0.79	1.21	0.57
DOK4	DOK4;FLJ10488	docking protein 4	0.65	0.82	1.21	0.55
BOC	BOC	Boc homolog (mouse)	0.64	0.8	1.21	0.57
SEMA5A	SEMA5A;FLJ12815;SEMAF;semF	sema domain, seven thrombospondin repeats (type 1 and type 1-like), transmembrane domain (TM) and short cytoplasmic domain, (semaphorin) 5A	0.64	0.8	1.21	0.53
MS4A15	MS4A15;FLJ34527;MGC35295	membrane-spanning 4-domains, subfamily A, member 15	0.64	0.75	1.21	0.6
PHKA2	PHKA2;GSD9A;MGC133071;PHK;PYK;PYKL;XLG;XLG2	phosphorylase kinase, alpha 2 (liver)	0.64	0.77	1.21	0.64
KIAA0664	KIAA0664;CLU1;DKFZp686L05242;DKFZp686M09233;FLJ23672	KIAA0664	0.64	0.74	1.21	0.55
ING4	ING4;MGC12557;my036;p29ING4	inhibitor of growth family, member 4	0.64	0.76	1.21	0.59
ARL6IP5	ARL6IP5;DERP11;GTRAP3-18;JWA;PRAF3;addicsin;hp22;jmx	ADP-ribosylation-like factor 6 interacting protein 5	0.64	0.75	1.21	0.6
IVNS1ABP	IVNS1ABP;DKFZp686K06216;FLARA3;FLJ10069;FLJ10411;FLJ10962;FLJ35593;FLJ36593;HSPC068;KIAA0850;KLHL39;ND1;NS-1;NS1-BP;NS1BP	influenza virus NS1A binding protein	0.64	0.76	1.21	0.57
DCAF8	DCAF8;DKFZp781G1096;FLJ35857;H326;MGC117276;MGC118891;MGC99640;WDR42A	DDB1 and CUL4 associated factor 8	0.64	0.84	1.21	0.62
C4orf51	C4orf51	chromosome 4 open reading frame 51	0.64	0.74	1.21	0.54
RSPH1	RSPH1;MGC126568;MGC141927;RSP44;RSPH10A;TSA2;TSGA2	radial spoke head 1 homolog (Chlamydomonas)	0.63	0.78	1.21	0.59
LRRC8A	LRRC8A;AGM5;FLJ10337;FLJ41617;KIAA1437;LRRC8	leucine rich repeat containing 8 family, member A	0.63	0.74	1.21	0.56
WBSCR22	WBSCR22;FLJ44236;HASJ4442;HUSSY-3;MGC19709;MGC2022;MGC5140;PP3381;WBMT	Williams Beuren syndrome chromosome region 22	0.63	0.7	1.21	0.62
CLEC4G	CLEC4G;DTTR431;LP2698;LSECtin;UNQ431	C-type lectin domain family 4, member G	0.62	0.72	1.21	0.55
PNPLA3	PNPLA3;ADPN;C22orf20;iPLA(2)epsilon	patatin-like phospholipase domain containing 3	0.62	0.74	1.21	0.55
MAD2L1BP	MAD2L1BP;CMT2;KIAA0110;MGC11282;RP1-261G23.6	MAD2L1 binding protein	0.62	0.68	1.21	0.56
C6orf162	C6orf162;DKFZp586E1923;dJ102H19.2	chromosome 6 open reading frame 162	0.62	0.77	1.21	0.52
SLC11A2	SLC11A2;DCT1;DMT1;FLJ37416;NRAMP2	solute carrier family 11 (proton-coupled divalent metal ion transporters), member 2	0.62	0.72	1.21	0.56
CD27	CD27;MGC20393;S152;T14;TNFRSF7;Tp55	CD27 molecule	0.61	0.72	1.21	0.53
GABRA6	GABRA6;MGC116903;MGC116904	gamma-aminobutyric acid (GABA) A receptor, alpha 6	0.84	1.01	1.22	0.79
DNAJB8	DNAJB8;DJ6;MGC33884	DnaJ (Hsp40) homolog, subfamily B, member 8	0.84	1.02	1.22	0.71
CTAGE5	CTAGE5;MEA6;MGEA;MGEA11;MGEA6	CTAGE family, member 5	0.84	0.98	1.22	0.79
TUBGCP4	TUBGCP4;76P;FLJ14797;GCP4	tubulin, gamma complex associated protein 4	0.84	0.92	1.22	0.73
SLC35E1	SLC35E1;DKFZp564G0462;FLJ14251;FLJ36689;MGC44954	solute carrier family 35, member E1	0.84	0.92	1.22	0.77
RNF145	RNF145;DKFZp686M11215;FLJ31951;FLJ44310	ring finger protein 145	0.83	1	1.22	0.9
GPR45	GPR45;PSP24;PSP24(ALPHA);PSP24A	G protein-coupled receptor 45	0.82	0.97	1.22	0.87
ACVR1C	ACVR1C;ACVRLK7;ALK7	activin A receptor, type IC	0.82	1	1.22	0.83
SCGB3A1	SCGB3A1;HIN-1;HIN1;LU105;MGC87867;PnSP-2;UGRP2	secretoglobin, family 3A, member 1	0.82	0.98	1.22	0.74
SLC12A8	SLC12A8;CCC9;DKFZp686L18248;FLJ23188	solute carrier family 12 (potassium/chloride transporters), member 8	0.82	0.98	1.22	0.74
ATPIF1	ATPIF1;ATPI;ATPIP;IP;MGC1167;MGC8898	ATPase inhibitory factor 1	0.82	1.02	1.22	0.78
PODXL	PODXL;Gp200;MGC138240;PC;PCLP;PCLP-1	podocalyxin-like	0.82	0.96	1.22	0.7
EZR	EZR;CVIL;CVL;DKFZp762H157;FLJ26216;MGC1584;VIL2	ezrin	0.82	1	1.22	0.68
ABHD16B	ABHD16B;C20orf135;MGC42974;dJ591C20.1	abhydrolase domain containing 16B	0.82	0.98	1.22	0.79
RAB6B	RAB6B	RAB6B, member RAS oncogene family	0.8	0.96	1.22	0.75
VDR	VDR;NR1I1	vitamin D (1,25- dihydroxyvitamin D3) receptor	0.8	0.94	1.22	0.74
TRRAP	TRRAP;FLJ10671;PAF350/400;PAF400;STAF40;TR-AP;Tra1	transformation/transcription domain-associated protein	0.8	0.92	1.22	0.72
PDE5A	PDE5A;CGB-PDE;CN5A;PDE5	phosphodiesterase 5A, cGMP-specific	0.79	0.96	1.22	0.65
VASH1	VASH1;KIAA1036	vasohibin 1	0.79	0.98	1.22	0.74
TM9SF4	TM9SF4;KIAA0255;dJ836N17.2	transmembrane 9 superfamily protein member 4	0.79	0.99	1.22	0.75
ZFAND5	ZFAND5;ZA20D2;ZFAND5A;ZNF216	zinc finger, AN1-type domain 5	0.79	0.96	1.22	0.85
APOBEC1	APOBEC1;APOBEC-1;BEDP;CDAR1;HEPR	apolipoprotein B mRNA editing enzyme, catalytic polypeptide 1	0.78	0.92	1.22	0.65
ZNF619	ZNF619;FLJ90764	zinc finger protein 619	0.78	0.85	1.22	0.68
JMJD4	JMJD4;FLJ12517;MGC129896	jumonji domain containing 4	0.78	0.96	1.22	0.65
ASCL4	ASCL4;HASH4;bHLHa44	achaete-scute complex homolog 4 (Drosophila)	0.77	0.98	1.22	0.74
OR52R1	OR52R1;OR11-22	olfactory receptor, family 52, subfamily R, member 1	0.77	0.9	1.22	0.71
LLGL1	LLGL1;DLG4;HUGL;HUGL-1;HUGL1;LLGL	lethal giant larvae homolog 1 (Drosophila)	0.77	0.94	1.22	0.65
APOL5	APOL5;APOL-V;APOLV	apolipoprotein L, 5	0.76	0.9	1.22	0.73
PRDM7	PRDM7;MGC129525;PFM4;ZNF910	PR domain containing 7	0.76	0.99	1.22	0.69
CXCL6	CXCL6;CKA-3;GCP-2;GCP2;SCYB6	chemokine (C-X-C motif) ligand 6 (granulocyte chemotactic protein 2)	0.76	0.88	1.22	0.74
NMNAT2	NMNAT2;C1orf15;KIAA0479;MGC2756;PNAT2	nicotinamide nucleotide adenylyltransferase 2	0.76	0.85	1.22	0.75
VANGL2	VANGL2;KIAA1215;LPP1;LTAP;MGC119403;MGC119404;STB1;STBM;STBM1	vang-like 2 (van gogh, Drosophila)	0.76	0.89	1.22	0.64
BMP4	BMP4;BMP2B;BMP2B1;MCOPS6;OFC11;ZYME	bone morphogenetic protein 4	0.76	0.96	1.22	0.75
HSPA12B	HSPA12B;C20orf60;FLJ32150;MGC131912;dJ1009E24.2	heat shock 70kD protein 12B	0.76	0.94	1.22	0.72
CSF1	CSF1;CSF-1;MCSF;MGC31930	colony stimulating factor 1 (macrophage)	0.76	0.92	1.22	0.65
CST4	CST4;MGC71923	cystatin S	0.76	0.94	1.22	0.65
GNL1	GNL1;DKFZp547E038;DKFZp547E128;FLJ37752;HSR1	guanine nucleotide binding protein-like 1	0.76	0.98	1.22	0.79
LSM3	LSM3;SMX4;USS2;YLR438C	LSM3 homolog, U6 small nuclear RNA associated (S. cerevisiae)	0.76	0.97	1.22	0.73
TECRL	TECRL;GPSN2L;MGC62104;SRD5A2L2;TERL	trans-2,3-enoyl-CoA reductase-like	0.76	0.97	1.22	0.65
ITM2B	ITM2B;ABRI;BRI;BRI2;BRICD2B;E25B;E3-16;FBD	integral membrane protein 2B	0.75	0.92	1.22	0.67
GNG3	GNG3	guanine nucleotide binding protein (G protein), gamma 3	0.74	0.92	1.22	0.7
LHFPL3	LHFPL3;LHFPL4	lipoma HMGIC fusion partner-like 3	0.74	0.82	1.22	0.68
SLFNL1	SLFNL1;FLJ23878;MGC43873	schlafen-like 1	0.74	0.8	1.22	0.63
SMOC1	SMOC1;OAS	SPARC related modular calcium binding 1	0.74	0.92	1.22	0.66
TMIGD2	TMIGD2;MGC23244	transmembrane and immunoglobulin domain containing 2	0.74	0.95	1.22	0.68
PELI2	PELI2	pellino homolog 2 (Drosophila)	0.74	0.86	1.22	0.63
RMND1	RMND1;C6orf96;FLJ20627;MGC117362;MGC149570;MGC88260;RMD1;bA351K16;bA351K16.3	required for meiotic nuclear division 1 homolog (S. cerevisiae)	0.74	0.93	1.22	0.69
MMACHC	MMACHC;DKFZp564I122;FLJ25671;RP11-291L19.3;cblC	methylmalonic aciduria (cobalamin deficiency) cblC type, with homocystinuria	0.74	0.86	1.22	0.66
KRTAP5-1	KRTAP5-1;KRN1L;KRTAP5.1	keratin associated protein 5-1	0.74	0.86	1.22	0.73
NCAPG2	NCAPG2;CAP-G2;FLJ20311;LUZP5;MTB;hCAP-G2	non-SMC condensin II complex, subunit G2	0.74	0.96	1.22	0.74
CCDC85B	CCDC85B;DIPA	coiled-coil domain containing 85B	0.73	0.79	1.22	0.62
PDE10A	PDE10A;FLJ11894;FLJ25677;HSPDE10A	phosphodiesterase 10A	0.72	0.82	1.22	0.65
C11orf45	C11orf45;FLJ43646;MGC35558	chromosome 11 open reading frame 45	0.72	0.89	1.22	0.67
ZNF642	ZNF642;FLJ16030;RP11-656D10.2;Zfp69	zinc finger protein 642	0.72	0.82	1.22	0.67
NOB1	NOB1;ART-4;MST158;NOB1P;PSMD8BP1	NIN1/RPN12 binding protein 1 homolog (S. cerevisiae)	0.72	0.78	1.22	0.61
C1orf43	C1orf43;DKFZp586G1722;MGC111001;NICE-3;NS5ATP4;S863-3	chromosome 1 open reading frame 43	0.72	0.79	1.22	0.62
EYA1	EYA1;BOP;BOR;MGC141875	eyes absent homolog 1 (Drosophila)	0.71	0.87	1.22	0.68
PCBP4	PCBP4;CBP;LIP4;MCG10	poly(rC) binding protein 4	0.71	0.82	1.22	0.69
LMNA	LMNA;CDCD1;CDDC;CMD1A;CMT2B1;EMD2;FPL;FPLD;FPLD2;HGPS;IDC;LDP1;LFP;LGMD1B;LMN1;LMNC;LMNL1;PRO1	lamin A/C	0.71	0.84	1.22	0.6
KRTAP19-3	KRTAP19-3;GTRHP;KAP19.3	keratin associated protein 19-3	0.7	0.86	1.22	0.58
40603	MARCH11;MARCH-XI	membrane-associated ring finger (C3HC4) 11	0.7	0.84	1.22	0.75
LRRK1	LRRK1;FLJ23119;FLJ27465;KIAA1790;RIPK6;Roco1	leucine-rich repeat kinase 1	0.7	0.92	1.22	0.65
LMTK3	LMTK3;KIAA1883;LMR3;TYKLM3	lemur tyrosine kinase 3	0.7	0.92	1.22	0.66
U2SURP	U2SURP;KIAA0332;MGC133197;SR140;fSAPa	U2 snRNP-associated SURP domain containing	0.7	0.83	1.22	0.62
OR5A2	OR5A2	olfactory receptor, family 5, subfamily A, member 2	0.69	0.88	1.22	0.66
HOXC9	HOXC9;HOX3;HOX3B	homeobox C9	0.69	0.86	1.22	0.68
ANKRD9	ANKRD9;MGC21990	ankyrin repeat domain 9	0.69	0.88	1.22	0.6
C6orf165	C6orf165;FLJ25974;FLJ46091;dJ382I10.1	chromosome 6 open reading frame 165	0.68	0.86	1.22	0.59
TBX4	TBX4;SPS	T-box 4	0.68	0.76	1.22	0.68
PEAR1	PEAR1;FLJ00193;JEDI;MEGF12	platelet endothelial aggregation receptor 1	0.68	0.9	1.22	0.68
DENND2A	DENND2A;FAM31D;KIAA1277	DENN/MADD domain containing 2A	0.68	0.75	1.22	0.58
NRF1	NRF1;ALPHA-PAL	nuclear respiratory factor 1	0.68	0.79	1.22	0.57
ZNF195	ZNF195;DKFZp666D035;HRF1	zinc finger protein 195	0.68	0.74	1.22	0.62
TMEM14C	TMEM14C;C6orf53;MSTP073;NET26;bA421M1.6	transmembrane protein 14C	0.68	0.87	1.22	0.65
MLXIPL	MLXIPL;CHREBP;MIO;MONDOB;WBSCR14;WS-bHLH;bHLHd14	MLX interacting protein-like	0.67	0.79	1.22	0.58
CYB561	CYB561;FRRS2	cytochrome b-561	0.67	0.74	1.22	0.59
RPS6KC1	RPS6KC1;RPK118;humS6PKh1	ribosomal protein S6 kinase, 52kDa, polypeptide 1	0.67	0.88	1.22	0.62
C19orf10	C19orf10;EUROIMAGE1875335;IL25;IL27;IL27w;R33729_1;SF20	chromosome 19 open reading frame 10	0.67	0.82	1.22	0.64
MC5R	MC5R;MC2	melanocortin 5 receptor	0.66	0.76	1.22	0.57
SLC6A13	SLC6A13;GAT-2;GAT2	solute carrier family 6 (neurotransmitter transporter, GABA), member 13	0.66	0.8	1.22	0.62
USP51	USP51	ubiquitin specific peptidase 51	0.66	0.78	1.22	0.67
ZCCHC24	ZCCHC24;C10orf56;FLJ90798	zinc finger, CCHC domain containing 24	0.66	0.84	1.22	0.56
UBN1	UBN1;VT;VT4	ubinuclein 1	0.66	0.89	1.22	0.62
PEX1	PEX1;ZWS;ZWS1	peroxisomal biogenesis factor 1	0.66	0.78	1.22	0.56
ATP5J	ATP5J;ATP5;ATP5A;ATPM;CF6;F6	ATP synthase, H+ transporting, mitochondrial Fo complex, subunit F6	0.66	0.88	1.22	0.57
TERT	TERT;DKCA2;DKCB4;EST2;TCS1;TP2;TRT;hEST2;hTRT	telomerase reverse transcriptase	0.65	0.76	1.22	0.59
C9orf16	C9orf16;FLJ12823;MGC4639	chromosome 9 open reading frame 16	0.65	0.72	1.22	0.6
PPIE	PPIE;CYP-33;CYP33;MGC111222;MGC3736	peptidylprolyl isomerase E (cyclophilin E)	0.65	0.8	1.22	0.54
ATAD3A	ATAD3A;FLJ10709;FLJ35514	ATPase family, AAA domain containing 3A	0.65	0.83	1.22	0.55
SIGLEC8	SIGLEC8;MGC59785;SAF2;SIGLEC-8;SIGLEC8L	sialic acid binding Ig-like lectin 8	0.64	0.71	1.22	0.56
HOXA5	HOXA5;HOX1;HOX1.3;HOX1C;MGC9376	homeobox A5	0.64	0.85	1.22	0.59
TTC23	TTC23;FLJ12572;FLJ12939;HCC-8	tetratricopeptide repeat domain 23	0.64	0.76	1.22	0.58
ZKSCAN2	ZKSCAN2;FLJ23199;ZNF694;ZSCAN31	zinc finger with KRAB and SCAN domains 2	0.64	0.72	1.22	0.64
SULT1A3	SULT1A3;HAST;HAST3;M-PST;MGC117469;ST1A3/ST1A4;ST1A5;STM;TL-PST	sulfotransferase family, cytosolic, 1A, phenol-preferring, member 3	0.64	0.74	1.22	0.64
USP17L2	USP17L2;DUB-3;DUB3	ubiquitin specific peptidase 17-like 2	0.64	0.74	1.22	0.54
CLU	CLU;APO-J;APOJ;CLI;KUB1;MGC24903;NA1/NA2;SGP-2;SGP2;SP-40;TRPM-2;TRPM2	clusterin	0.63	0.8	1.22	0.53
NAA11	NAA11;ARD1B;ARD2;MGC10646;hARD2	N(alpha)-acetyltransferase 11, NatA catalytic subunit	0.63	0.69	1.22	0.54
PCDHA9	PCDHA9;KIAA0345;PCDH-ALPHA9	protocadherin alpha 9	0.63	0.74	1.22	0.57
ZNF671	ZNF671;FLJ23506	zinc finger protein 671	0.62	0.68	1.22	0.53
SCN4A	SCN4A;HOKPP2;HYKPP;HYPP;NAC1A;Na(V)1.4;Nav1.4;SkM1	sodium channel, voltage-gated, type IV, alpha subunit	0.62	0.74	1.22	0.58

EHMT1	EHMT1;DKFZp667M072;EUHMTASE1;Eu-HMTase1;FLJ12879;FP13812;GLP;GLP1;KIAA1876;KMT1D;bA188C12.1	euchromatic histone-lysine N-methyltransferase 1	0.62	0.74	1.22	0.53
RWDD1	RWDD1;PTD013	RWD domain containing 1	0.62	0.76	1.22	0.55
C15orf24	C15orf24;C11orf3;ORF1-FL1	chromosome 15 open reading frame 24	0.62	0.73	1.22	0.55
ALCAM	ALCAM;CD166;FLJ38514;MEMD;MGC71733	activated leukocyte cell adhesion molecule	0.62	0.76	1.22	0.53
UNC79	UNC79;FLJ43337;KIAA1409	unc-79 homolog (C. elegans)	0.62	0.77	1.22	0.59
KRTAP12-4	KRTAP12-4;KRTAP12.4;MGC150594	keratin associated protein 12-4	0.61	0.72	1.22	0.58
RC3H1	RC3H1;KIAA2025;RNF198;ROQUIN	ring finger and CCCH-type domains 1	0.61	0.74	1.22	0.66
FBF1	FBF1;Alb;FBF-1;FLJ00103;FLJ13996;KIAA1863	Fas (TNFRSF6) binding factor 1	0.84	0.98	1.23	0.75
ARHGAP11B	ARHGAP11B;B'-T;FAM7B1;MGC104367	Rho GTPase activating protein 11B	0.84	1.06	1.23	0.8
SOCS4	SOCS4;DKFZp686J1568;SOCS7	suppressor of cytokine signaling 4	0.84	0.99	1.23	0.71
LIMK1	LIMK1;LIMK;LIMK-1	LIM domain kinase 1	0.84	1.04	1.23	0.86
CTSO	CTSO;CTSO1	cathepsin O	0.83	1.02	1.23	0.76
SLC15A1	SLC15A1;HPECT1;HPEPT1;PEPT1	solute carrier family 15 (oligopeptide transporter), member 1	0.82	0.98	1.23	0.69
AMPD3	AMPD3	adenosine monophosphate deaminase 3	0.82	0.97	1.23	0.72
ARMCX1	ARMCX1;ALEX1;DKFZp686P06199	armadillo repeat containing, X-linked 1	0.82	1.03	1.23	0.79
UBE2E2	UBE2E2;FLJ25157;UBCH8	ubiquitin-conjugating enzyme E2E 2	0.82	0.93	1.23	0.7
GPHB5	GPHB5;B5;GPB5;ZLUT1	glycoprotein hormone beta 5	0.81	0.9	1.23	0.72
VPS4B	VPS4B;SKD1;SKD1B;VPS4-2	vacuolar protein sorting 4 homolog B (S. cerevisiae)	0.81	1	1.23	0.78
HTR3E	HTR3E;5-HT3c1;MGC120035;MGC120036;MGC120037	5-hydroxytryptamine (serotonin) receptor 3, family member E	0.8	1.03	1.23	0.8
SYP	SYP;MRXSYP	synaptophysin	0.8	0.96	1.23	0.72
TNIP2	TNIP2;ABIN2;DKFZp434J1313;FLIP1;KLIP;MGC4289	TNFAIP3 interacting protein 2	0.8	0.94	1.23	0.76
MBTPS1	MBTPS1;KIAA0091;MGC138711;MGC138712;PCSK8;S1P;SKI-1	membrane-bound transcription factor peptidase, site 1	0.8	0.96	1.23	0.73
CNIH3	CNIH3;CNIH-3;FLJ38993	cornichon homolog 3 (Drosophila)	0.79	0.98	1.23	0.68
FAM3B	FAM3B;2-21;C21orf11;C21orf76;ORF9;PANDER	family with sequence similarity 3, member B	0.78	0.94	1.23	0.79
PLA2G2A	PLA2G2A;MOM1;PLA2;PLA2B;PLA2L;PLA2S;PLAS1;sPLA2	phospholipase A2, group IIA (platelets, synovial fluid)	0.78	0.92	1.23	0.72
NCOA6	NCOA6;AIB3;ASC2;KIAA0181;NRC;PRIP;RAP250;TRBP	nuclear receptor coactivator 6	0.78	0.9	1.23	0.71
C5orf22	C5orf22;DKFZp667N066;FLJ11193;FLJ23805;MGC33010	chromosome 5 open reading frame 22	0.78	0.94	1.23	0.7
UTP6	UTP6;C17orf40;HCA66	UTP6, small subunit (SSU) processome component, homolog (yeast)	0.78	0.93	1.23	0.7
IDH1	IDH1;IDCD;IDH;IDP;IDPC;PICD	isocitrate dehydrogenase 1 (NADP+), soluble	0.78	0.94	1.23	0.66
UNC13D	UNC13D;FHL3;HLH3;HPLH3;Munc13-4	unc-13 homolog D (C. elegans)	0.77	0.97	1.23	0.7
SESN1	SESN1;MGC138241;MGC142129;PA26;SEST1	sestrin 1	0.77	0.85	1.23	0.71
PEX13	PEX13;NALD;ZWS	peroxisomal biogenesis factor 13	0.77	0.93	1.23	0.65
GLTSCR1	GLTSCR1	glioma tumor suppressor candidate region gene 1	0.76	0.97	1.23	0.64
SNCG	SNCG;BCSG1;SR	synuclein, gamma (breast cancer-specific protein 1)	0.76	0.96	1.23	0.73
SMOX	SMOX;C20orf16;FLJ20746;MGC1010;PAO;PAOH1;SMO	spermine oxidase	0.76	0.88	1.23	0.69
CLDN1	CLDN1;CLD1;ILVASC;SEMP1	claudin 1	0.76	0.88	1.23	0.75
C12orf74	C12orf74;MGC125584	chromosome 12 open reading frame 74	0.76	0.85	1.23	0.67
ARMC7	ARMC7;FLJ22160	armadillo repeat containing 7	0.75	0.83	1.23	0.65
GREM1	GREM1;CKTSF1B1;DAND2;DRM;GREMLIN;IHG-2;MGC126660	gremlin 1	0.75	0.96	1.23	0.71
ASCC1	ASCC1;ASC1p50;RP11-150D20.4;p50	activating signal cointegrator 1 complex subunit 1	0.75	0.9	1.23	0.72
FRMD1	FRMD1;DKFZp434O0117;FLJ00181;FLJ22615;FLJ40260;bA164L23.1	FERM domain containing 1	0.74	0.95	1.23	0.72
ARHGAP24	ARHGAP24;DKFZp564B1162;FILGAP;FLJ33877;RC-GAP72;RCGAP72;p73;p73RhoGAP	Rho GTPase activating protein 24	0.74	0.92	1.23	0.64
ANKMY1	ANKMY1;DKFZp686D20155;DKFZp686L21237;FLJ20499;ZMYND13	ankyrin repeat and MYND domain containing 1	0.74	0.88	1.23	0.7
QRFP	QRFP;26RFa;MGC119794;P518	pyroglutamylated RFamide peptide	0.74	0.83	1.23	0.66
MRPL20	MRPL20;L20mt;MGC4779;MGC74465;MRP-L20	mitochondrial ribosomal protein L20	0.74	0.94	1.23	0.71
DCAF16	DCAF16;C4orf30;FLJ20280;MGC126765;MGC126767	DDB1 and CUL4 associated factor 16	0.74	0.81	1.23	0.63
ZNF878	ZNF878	zinc finger protein 878	0.74	0.88	1.23	0.67
LINC00471	LINC00471;C2orf52;MGC43122	long intergenic non-protein coding RNA 471	0.74	0.88	1.23	0.67
FXYD3	FXYD3;MAT8;MGC111076;PLML	FXYD domain containing ion transport regulator 3	0.73	0.88	1.23	0.69
MAGEC1	MAGEC1;CT7;CT7.1;MGC39366	melanoma antigen family C, 1	0.72	0.8	1.23	0.67
CBLN1	CBLN1	cerebellin 1 precursor	0.72	0.86	1.23	0.67
GDF6	GDF6;BMP13;CDMP2;KFM;KFS;KFS1;KFSL;MCOP4;MCOPCB6;MGC158100;MGC158101;SCDO4;SGM1	growth differentiation factor 6	0.72	0.87	1.23	0.72
ATP6V1C2	ATP6V1C2;ATP6C2;VMA5	ATPase, H+ transporting, lysosomal 42kDa, V1 subunit C2	0.72	0.84	1.23	0.66
SRF	SRF;MCM1	serum response factor (c-fos serum response element-binding transcription factor)	0.72	0.9	1.23	0.69
GRK6	GRK6;FLJ32135;GPRK6	G protein-coupled receptor kinase 6	0.72	0.87	1.23	0.72
HCAR2	HCAR2;GPR109A;HCA2;HM74a;HM74b;NIACR1;PUMAG;Puma-g	hydroxycarboxylic acid receptor 2	0.72	0.86	1.23	0.77
C22orf25	C22orf25;DKFZp761P1121	chromosome 22 open reading frame 25	0.71	0.85	1.23	0.64
TIGD4	TIGD4;MGC43837	tigger transposable element derived 4	0.7	0.88	1.23	0.67
ASPRV1	ASPRV1;MUNO;SASP;SASPase;Taps	aspartic peptidase, retroviral-like 1	0.7	0.84	1.23	0.63
NEO1	NEO1;DKFZp547A066;DKFZp547B146;IGDCC2;NGN	neogenin 1	0.7	0.88	1.23	0.7
NFIA	NFIA;CTF;DKFZp434L0422;DKFZp686J23256;FLJ39164;KIAA1439;NF-I/A;NF1-A;NFI-A;NFI-L	nuclear factor I/A	0.7	0.82	1.23	0.59
GULP1	GULP1;CED-6;CED6;FLJ31156;GULP	GULP, engulfment adaptor PTB domain containing 1	0.7	0.92	1.23	0.61
TMC8	TMC8;EV2;EVER2;EVIN2;FLJ40668;FLJ43684;MGC102701;MGC40121	transmembrane channel-like 8	0.7	0.93	1.23	0.71
MRPL10	MRPL10;L10MT;MGC17973;MRP-L10;MRP-L8;MRPL8;RPML8	mitochondrial ribosomal protein L10	0.7	0.88	1.23	0.6
ABHD2	ABHD2;HS1-2;LABH2;MGC111112;MGC26249;PHPS1-2	abhydrolase domain containing 2	0.7	0.9	1.23	0.6
FBXO10	FBXO10;FBX10;FLJ41992;MGC149840;PRMT11	F-box protein 10	0.7	0.82	1.23	0.6
MAPK14	MAPK14;CSBP;CSBP1;CSBP2;CSPB1;EXIP;Mxi2;PRKM14;PRKM15;RK;SAPK2A;p38;p38ALPHA	mitogen-activated protein kinase 14	0.7	0.92	1.23	0.66
GPR108	GPR108;LUSTR2;MGC14393	G protein-coupled receptor 108	0.7	0.8	1.23	0.61
ESRP2	ESRP2;FLJ21918;FLJ22248;RBM35B	epithelial splicing regulatory protein 2	0.7	0.86	1.23	0.6
ZDHHC11	ZDHHC11;FLJ13153;ZNF399	zinc finger, DHHC-type containing 11	0.69	0.88	1.23	0.59
RFX1	RFX1;EFC;RFX	regulatory factor X, 1 (influences HLA class II expression)	0.69	0.79	1.23	0.63
H2BFWT	H2BFWT;MGC148130;MGC148131	H2B histone family, member W, testis-specific	0.68	0.86	1.23	0.65
C9orf4	C9orf4;CG-6;CG6	chromosome 9 open reading frame 4	0.68	0.86	1.23	0.62
PSAPL1	PSAPL1;FLJ40379	prosaposin-like 1 (gene/pseudogene)	0.68	0.8	1.23	0.58
MAP2K6	MAP2K6;MAPKK6;MEK6;MKK6;PRKMK6;SAPKK3	mitogen-activated protein kinase kinase 6	0.68	0.9	1.23	0.64
YPEL3	YPEL3;MGC10500	yippee-like 3 (Drosophila)	0.68	0.85	1.23	0.63
ATPAF2	ATPAF2;ATP12;ATP12p;MC5DN1;MGC29736	ATP synthase mitochondrial F1 complex assembly factor 2	0.68	0.82	1.23	0.66
SLC2A6	SLC2A6;GLUT6;GLUT9;HSA011372	solute carrier family 2 (facilitated glucose transporter), member 6	0.68	0.89	1.23	0.58
NKAP	NKAP;FLJ22626	NFKB activating protein	0.68	0.82	1.23	0.69
NSUN3	NSUN3;FLJ22109;FLJ22609;MST077	NOP2/Sun domain family, member 3	0.68	0.86	1.23	0.64
EPB41L5	EPB41L5;BE37;FLJ12957;KIAA1548;YMO1	erythrocyte membrane protein band 4.1 like 5	0.68	0.92	1.23	0.67
DHTKD1	DHTKD1;DKFZp762M115;KIAA1630;MGC3090	dehydrogenase E1 and transketolase domain containing 1	0.68	0.83	1.23	0.58
SLC4A1AP	SLC4A1AP;FLJ10624;FLJ41004;MGC120646;MGC120648	solute carrier family 4 (anion exchanger), member 1, adaptor protein	0.68	0.84	1.23	0.56
LEFTY1	LEFTY1;LEFTB;LEFTYB	left-right determination factor 1	0.67	0.88	1.23	0.62
ATP11C	ATP11C;ATPIG;ATPIQ	ATPase, class VI, type 11C	0.67	0.83	1.23	0.56
OR14K1	OR14K1;OR1-39;OR1.5.9;OR5AY1	olfactory receptor, family 14, subfamily K, member 1	0.67	0.78	1.23	0.66
TREM2	TREM2;TREM-2;Trem2a;Trem2b;Trem2c	triggering receptor expressed on myeloid cells 2	0.66	0.86	1.23	0.57
TTC21A	TTC21A;DKFZp686P18239;MGC156293;MGC70523;STI2	tetratricopeptide repeat domain 21A	0.66	0.84	1.23	0.63
ZC3HAV1L	ZC3HAV1L;C7orf39;MGC14289	zinc finger CCCH-type, antiviral 1-like	0.66	0.84	1.23	0.63
PRPF6	PRPF6;ANT-1;ANT1;C20orf14;Prp6;TOM;U5-102K;hPrp6	PRP6 pre-mRNA processing factor 6 homolog (S. cerevisiae)	0.65	0.8	1.23	0.57
SEC23A	SEC23A;CLSD;MGC26267	Sec23 homolog A (S. cerevisiae)	0.65	0.78	1.23	0.62
SPINT2	SPINT2;DIAR3;FLJ45571;HAI-2;HAI2;Kop;PB	serine peptidase inhibitor, Kunitz type, 2	0.65	0.77	1.23	0.67
ZFY	ZFY;MGC138710;ZNF911	zinc finger protein, Y-linked	0.64	0.72	1.23	0.57
G6PC	G6PC;G6PC1;G6PT;GSD1;GSD1a;MGC163350	glucose-6-phosphatase, catalytic subunit	0.64	0.73	1.23	0.55
POLR3F	POLR3F;MGC13517;RPC39;RPC6	polymerase (RNA) III (DNA directed) polypeptide F, 39 kDa	0.64	0.76	1.23	0.58
FOXQ1	FOXQ1;HFH1	forkhead box Q1	0.64	0.77	1.23	0.64
TDRKH	TDRKH;TDRD2	tudor and KH domain containing	0.64	0.72	1.23	0.57
SHISA5	SHISA5;SCOTIN;hShisa5	shisa homolog 5 (Xenopus laevis)	0.64	0.8	1.23	0.56
NOP56	NOP56;NOL5A;SCA36	NOP56 ribonucleoprotein homolog (yeast)	0.64	0.82	1.23	0.58
LRR1	LRR1;4-1BBLRR;LRR-1;MGC20689;PPIL5	leucine rich repeat protein 1	0.64	0.75	1.23	0.59
WDR96	WDR96;C10orf79;FLJ22944;FLJ36006;bA373N18.2	WD repeat domain 96	0.64	0.8	1.23	0.56
FSCN3	FSCN3	fascin homolog 3, actin-bundling protein, testicular (Strongylocentrotus purpuratus)	0.62	0.74	1.23	0.58
EGF	EGF;HOMG4;URG	epidermal growth factor	0.62	0.73	1.23	0.57
IL2RB	IL2RB;CD122;IL15RB;P70-75	interleukin 2 receptor, beta	0.62	0.78	1.23	0.62
ADAMTSL5	ADAMTSL5;THSD6	ADAMTS-like 5	0.62	0.84	1.23	0.56
SCFD1	SCFD1;C14orf163;KIAA0917;RA410;SLY1;STXBP1L2	sec1 family domain containing 1	0.62	0.75	1.23	0.59
GFM2	GFM2;EF-G2mt;EFG2;MRRF2;MST027;RRF2;RRF2mt;hEFG2;mEF-G 2	G elongation factor, mitochondrial 2	0.62	0.73	1.23	0.64
CHIA	CHIA;AMCASE;CHIT2;DKFZp313J1722;TSA1902	chitinase, acidic	0.61	0.72	1.23	0.53
OAZ1	OAZ1;AZI;MGC138338;OAZ	ornithine decarboxylase antizyme 1	0.61	0.71	1.23	0.56
PFAS	PFAS;FGAMS;FGARAT;KIAA0361;PURL	phosphoribosylformylglycinamidine synthase	0.84	0.97	1.24	0.86
MEMO1	MEMO1;C2orf4;DKFZp434I0135;FLJ25031;MEMO;NS5ATP7	mediator of cell motility 1	0.84	1.07	1.24	0.8
RPP40	RPP40;RNASEP1;bA428J1.3	ribonuclease P/MRP 40kDa subunit	0.84	0.94	1.24	0.75
NCAPD3	NCAPD3;CAP-D3;FLJ42888;KIAA0056;MGC104671;hCAP-D3;hHCP-6;hcp-6	non-SMC condensin II complex, subunit D3	0.83	1.06	1.24	0.77
DEFA6	DEFA6;DEF6;HD-6	defensin, alpha 6, Paneth cell-specific	0.82	0.94	1.24	0.74
KBTBD11	KBTBD11;KIAA0711;KLHDC7C	kelch repeat and BTB (POZ) domain containing 11	0.82	0.99	1.24	0.83
EVX2	EVX2;EVX-2	even-skipped homeobox 2	0.82	0.98	1.24	0.74
TLE3	TLE3;ESG;ESG3;FLJ39460;GRG3;HsT18976;KIAA1547	transducin-like enhancer of split 3 (E(sp1) homolog, Drosophila)	0.82	1.05	1.24	0.79
UPF2	UPF2;DKFZp434D222;HUPF2;KIAA1408;MGC138834;MGC138835;RENT2;smg-3	UPF2 regulator of nonsense transcripts homolog (yeast)	0.82	0.98	1.24	0.7
CRLF3	CRLF3;CREME-9;CREME9;CYTOR4;FRWS;MGC20661;p48.2	cytokine receptor-like factor 3	0.82	0.91	1.24	0.76
PARD6B	PARD6B;PAR6B	par-6 partitioning defective 6 homolog beta (C. elegans)	0.82	1.05	1.24	0.79
RAB43	RAB43;MGC90481;RAB11B;RAB41	RAB43, member RAS oncogene family	0.81	1.04	1.24	0.78
SERPINB3	SERPINB3;HsT1196;SCC;SCCA-1;SCCA-PD;SCCA1;T4-A	serpin peptidase inhibitor, clade B (ovalbumin), member 3	0.8	0.99	1.24	0.77
STX19	STX19;MGC21382	syntaxin 19	0.8	0.97	1.24	0.76
SYT10	SYT10;MGC119436;MGC119437	synaptotagmin X	0.8	0.97	1.24	0.7
DIS3L	DIS3L;DIS3L1;FLJ38088;KIAA1955;MGC4562	DIS3 mitotic control homolog (S. cerevisiae)-like	0.8	1.02	1.24	0.76
PNPLA8	PNPLA8;IPLA2(GAMMA);IPLA2-2;IPLA2G	patatin-like phospholipase domain containing 8	0.8	0.92	1.24	0.81
TSPAN6	TSPAN6;T245;TM4SF6;TSPAN-6	tetraspanin 6	0.8	0.96	1.24	0.69
BANF1	BANF1;BAF;BCRP1;D14S1460;MGC111161;NGPS	barrier to autointegration factor 1	0.8	0.91	1.24	0.72
FAM198B	FAM198B;AD036;C4orf18;DKFZp434L142;FLJ23966;FLJ38155	family with sequence similarity 198, member B	0.8	0.88	1.24	0.69

UMOD	UMOD;ADMCKD2;FJHN;HNFJ;HNFJ1;MCKD2;THGP;THP	uromodulin	0.79	1.01	1.24	0.68
POLG	POLG;FLJ27114;MDP1;MIRAS;MTDPS4A;MTDPS4B;PEO;POLG1;POLGA;SANDO;SCAE	polymerase (DNA directed), gamma	0.79	0.94	1.24	0.81
MLANA	MLANA;MART-1;MART1	melan-A	0.78	1	1.24	0.75
TMPRSS13	TMPRSS13;MSP;MSPL;MSPS;TMPRSS11	transmembrane protease, serine 13	0.78	0.96	1.24	0.72
DENND3	DENND3;KIAA0870	DENN/MADD domain containing 3	0.78	0.94	1.24	0.72
KRI1	KRI1;FLJ12949	KRI1 homolog (S. cerevisiae)	0.78	1	1.24	0.67
DSCR3	DSCR3;DCRA;DSCRA;MGC117385	Down syndrome critical region gene 3	0.78	1	1.24	0.76
TNFSF13	TNFSF13;APRIL;CD256;FLJ57090;TALL-2;TALL2;TRDL-1;ZTNF2	tumor necrosis factor (ligand) superfamily, member 13	0.78	0.94	1.24	0.71
IGSF21	IGSF21;FLJ41177;MGC15730	immunoglobin superfamily, member 21	0.76	0.85	1.24	0.66
SLC1A1	SLC1A1;EAAC1;EAAT3	solute carrier family 1 (neuronal/epithelial high affinity glutamate transporter, system Xag), member 1	0.76	0.92	1.24	0.79
MTR	MTR;FLJ33168;FLJ43216;FLJ45386;MS;cblG	5-methyltetrahydrofolate-homocysteine methyltransferase	0.76	0.96	1.24	0.74
SNAP29	SNAP29;CEDNIK;FLJ21051;SNAP-29	synaptosomal-associated protein, 29kDa	0.76	0.94	1.24	0.64
SKIL	SKIL;SNO;SnoA;SnoI;SnoN	SKI-like oncogene	0.75	0.94	1.24	0.72
MRC1L1	MRC1L1	ENTREZ STATUS: REMAPPED TO GENE ID: 4360	0.75	0.9	1.24	0.69
OR2T33	OR2T33;OR1-56	olfactory receptor, family 2, subfamily T, member 33	0.74	0.86	1.24	0.73
SHOX2	SHOX2;OG12;OG12X;SHOT	short stature homeobox 2	0.74	0.89	1.24	0.77
CGB	CGB;CGB3;CGB5;CGB7;CGB8;hCGB	chorionic gonadotropin, beta polypeptide	0.74	0.92	1.24	0.63
LZTR1	LZTR1;LZTR-1;MGC21205	leucine-zipper-like transcription regulator 1	0.74	0.89	1.24	0.63
FOXRED2	FOXRED2;ERFAD;FLJ23322;FLJ33734	FAD-dependent oxidoreductase domain containing 2	0.74	0.88	1.24	0.68
QTRTD1	QTRTD1;FLJ12960	queuine tRNA-ribosyltransferase domain containing 1	0.74	0.95	1.24	0.63
AKAP8L	AKAP8L;DKFZp434L0650;HA95;HAP95;NAKAP;NAKAP95	A kinase (PRKA) anchor protein 8-like	0.74	0.86	1.24	0.67
PPP1R27	PPP1R27;DYSFIP1;MGC138299	protein phosphatase 1, regulatory subunit 27	0.74	0.9	1.24	0.64
AKAP7	AKAP7;AKAP15;AKAP18	A kinase (PRKA) anchor protein 7	0.73	0.84	1.24	0.67
OR2G3	OR2G3;OR1-33	olfactory receptor, family 2, subfamily G, member 3	0.72	0.85	1.24	0.61
HCN1	HCN1;BCNG-1;BCNG1;HAC-2	hyperpolarization activated cyclic nucleotide-gated potassium channel 1	0.72	0.88	1.24	0.69
NKAIN1	NKAIN1;FAM77C;FLJ12650	Na+/K+ transporting ATPase interacting 1	0.72	0.84	1.24	0.68
SMTNL2	SMTNL2;FLJ42461;MGC131847;MGC138382	smoothelin-like 2	0.72	0.84	1.24	0.65
AQP10	AQP10;AQPA_HUMAN	aquaporin 10	0.71	0.82	1.24	0.72
UBE2CBP	UBE2CBP;C6orf157;DKFZp434A1520;H10BH;MGC126561;MGC126587;YJR141W	ubiquitin-conjugating enzyme E2C binding protein	0.71	0.92	1.24	0.65
ZFHX4	ZFHX4;FLJ16514;FLJ20980;ZFH4;ZHF4	zinc finger homeobox 4	0.71	0.9	1.24	0.65
SMPD3	SMPD3;FLJ22593;MGC138443;NSMASE2	sphingomyelin phosphodiesterase 3, neutral membrane (neutral sphingomyelinase II)	0.7	0.8	1.24	0.64
MAPRE3	MAPRE3;EB3;EBF3;EBF3-S;RP3	microtubule-associated protein, RP/EB family, member 3	0.7	0.88	1.24	0.69
CRTC1	CRTC1;FLJ14027;KIAA0616;MECT1;TORC1;WAMTP1	CREB regulated transcription coactivator 1	0.7	0.86	1.24	0.6
LIMD2	LIMD2;MGC10986	LIM domain containing 2	0.7	0.94	1.24	0.63
GTF3C5	GTF3C5;FLJ20857;TFIIIC63;TFIIICepsilon;TFiiiC2-63	general transcription factor IIIC, polypeptide 5, 63kDa	0.7	0.83	1.24	0.6
PRR18	PRR18;MGC35308	proline rich 18	0.69	0.82	1.24	0.63
SLC10A1	SLC10A1;NTCP	solute carrier family 10 (sodium/bile acid cotransporter family), member 1	0.68	0.85	1.24	0.57
NT5C1A	NT5C1A;CN-I;CN-IA;CN1;CN1A;MGC119199;MGC119201	5'-nucleotidase, cytosolic IA	0.68	0.8	1.24	0.6
CD44	CD44;CDW44;CSPG8;ECMR-III;HCELL;HUTCH-I;IN;LHR;MC56;MDU2;MDU3;MGC10468;MIC4;Pgp1	CD44 molecule (Indian blood group)	0.68	0.88	1.24	0.66
SIX4	SIX4;AREC3;MGC119450;MGC119452;MGC119453	SIX homeobox 4	0.68	0.78	1.24	0.63
KLHDC2	KLHDC2;HCLP-1;LCP	kelch domain containing 2	0.68	0.81	1.24	0.62
ADPRH	ADPRH;ARH1	ADP-ribosylarginine hydrolase	0.67	0.84	1.24	0.56
HNRNPCL1	HNRNPCL1;HNRPCL1	heterogeneous nuclear ribonucleoprotein C-like 1	0.66	0.8	1.24	0.6
PAGE4	PAGE4;CT16.7;FLJ35184;GAGE-9;GAGEC1;JM-27;PAGE-1;PAGE-4	P antigen family, member 4 (prostate associated)	0.66	0.79	1.24	0.59
ARSH	ARSH;sulfatase	arylsulfatase family, member H	0.66	0.78	1.24	0.58
SCO2	SCO2;MGC125823;MGC125825;SCO1L	SCO cytochrome oxidase deficient homolog 2 (yeast)	0.66	0.8	1.24	0.61
C9orf140	C9orf140;p42.3	chromosome 9 open reading frame 140	0.66	0.74	1.24	0.59
TERF2IP	TERF2IP;DRIP5;RAP1	telomeric repeat binding factor 2, interacting protein	0.66	0.83	1.24	0.66
NAIF1	NAIF1;C9orf90;DKFZp762G199;RP11-379C10.2;bA379C10.2	nuclear apoptosis inducing factor 1	0.65	0.74	1.24	0.58
ZNF586	ZNF586;FLJ20070	zinc finger protein 586	0.64	0.82	1.24	0.55
TNFSF15	TNFSF15;MGC129934;MGC129935;TL1;TL1A;VEGI;VEGI192A	tumor necrosis factor (ligand) superfamily, member 15	0.64	0.82	1.24	0.61
ZNF311	ZNF311;zf31	zinc finger protein 311	0.64	0.84	1.24	0.62
ALAD	ALAD;ALADH;MGC5057;PBGS	aminolevulinate dehydratase	0.64	0.78	1.24	0.55
ZIC2	ZIC2;HPE5	Zic family member 2	0.64	0.84	1.24	0.55
FAM59A	FAM59A;C18orf11;GAREM;Gm944	family with sequence similarity 59, member A	0.64	0.79	1.24	0.57
MFSD9	MFSD9;MGC11332	major facilitator superfamily domain containing 9	0.64	0.81	1.24	0.58
NDUFC1	NDUFC1;KFYI;MGC117464;MGC126847;MGC138266	NADH dehydrogenase (ubiquinone) 1, subcomplex unknown, 1, 6kDa	0.64	0.84	1.24	0.67
ZNF573	ZNF573;FLJ30921	zinc finger protein 573	0.62	0.7	1.24	0.55
UPF1	UPF1;FLJ43809;FLJ46894;HUPF1;KIAA0221;NORF1;RENT1;pNORF1;smg-2	UPF1 regulator of nonsense transcripts homolog (yeast)	0.62	0.71	1.24	0.63
THNSL2	THNSL2;FLJ10916;FLJ35504;SOFAT;THS2;TSH2	threonine synthase-like 2 (S. cerevisiae)	0.61	0.72	1.24	0.52
SDF2	SDF2	stromal cell-derived factor 2	0.61	0.77	1.24	0.53
BAX	BAX;BCL2L4	BCL2-associated X protein	0.61	0.7	1.24	0.62
CHRM5	CHRM5;HM5;MGC41838	cholinergic receptor, muscarinic 5	0.59	0.26	0.45	0.23
KCNG2	KCNG2;KCNF2;KV6.2	potassium voltage-gated channel, subfamily G, member 2	0.58	0.31	0.49	0.23
HIST1H1E	HIST1H1E;H1.4;H1E;H1F4;MGC116819;dJ221C16.5	histone cluster 1, H1e	0.43	0.21	0.46	0.16
SMARCA2	SMARCA2;BAF190;BRM;FLJ36757;MGC74511;SNF2;SNF2L2;SNF2LA;SWI2;Sth1p;hBRM;hSNF2a	SWI/SNF related, matrix associated, actin dependent regulator of chromatin, subfamily a, member 2	0.57	0.36	0.64	0.25
PLEKHG6	PLEKHG6;FLJ10665;MGC126315;MGC126353;MGC126354;MyoGEF	pleckstrin homology domain containing, family G (with RhoGef domain) member 6	0.6	0.4	0.65	0.3
ARRDC2	ARRDC2;CLONE24945	arrestin domain containing 2	0.57	0.36	0.7	0.3
LRFN3	LRFN3;FIGLER1;MGC2656;SALM4	leucine rich repeat and fibronectin type III domain containing 3	0.58	0.42	0.67	0.33
PEMT	PEMT;MGC2483;PEAMT;PEMPT;PEMT2;PNMT	phosphatidylethanolamine N-methyltransferase	0.57	0.38	0.67	0.29
BCS1L	BCS1L;BCS;BCS1;BJS;FLNMS;GRACILE;Hs.6719;PTD;h-BCS	BCS1-like (S. cerevisiae)	0.53	0.34	0.67	0.3
MAST1	MAST1;KIAA0973;SAST;SAST170	microtubule associated serine/threonine kinase 1	0.55	0.4	0.68	0.29
UBB	UBB;FLJ25987;MGC8385;RPS27A;UBA52;UBC	ubiquitin B	0.02	0.01	0.69	0.01
CCDC6	CCDC6;D10S170;FLJ32286;H4;PTC;TPC;TST1	coiled-coil domain containing 6	0.58	0.42	0.7	0.32
EME2	EME2;FLJ00151;SLX2B;gs125	essential meiotic endonuclease 1 homolog 2 (S. pombe)	0.55	0.4	0.7	0.27
IL17C	IL17C;CX2;IL-17C;IL-21;MGC126884;MGC138401	interleukin 17C	0.54	0.4	0.7	0.28
LRRC14	LRRC14;KIAA0014;LRRC14A	leucine rich repeat containing 14	0.55	0.39	0.71	0.31
C9orf30	C9orf30;FLJ34973;MGC17337	chromosome 9 open reading frame 30	0.55	0.38	0.72	0.31
NOX4	NOX4;KOX;KOX-1;RENOX	NADPH oxidase 4	0.44	0.36	0.74	0.25
PMFBP1	PMFBP1;DKFZp434G131;FLJ40146	polyamine modulated factor 1 binding protein 1	0.58	0.45	0.75	0.34
C5AR1	C5AR1;C5A;C5AR;C5R1;CD88	complement component 5a receptor 1	0.56	0.4	0.75	0.36
ENOPH1	ENOPH1;DKFZp586M0524;E1;FLJ12594;MASA;MST145	enolase-phosphatase 1	0.52	0.38	0.75	0.29
RBM15B	RBM15B;HUMAGCGB;OTT3	RNA binding motif protein 15B	0.6	0.5	0.76	0.33
KLHL36	KLHL36;C16orf44;FLJ12543	kelch-like 36 (Drosophila)	0.57	0.45	0.76	0.32
BPIFC	BPIFC;BPIL2	BPI fold containing family C	0.55	0.43	0.76	0.29
ZC3H7B	ZC3H7B;DKFZp434K0920;FLJ13787;KIAA1031;RoXaN	zinc finger CCCH-type containing 7B	0.46	0.38	0.76	0.25
ITGA6	ITGA6;CD49f;DKFZp686J01244;FLJ18737;ITGA6B;VLA-6	integrin, alpha 6	0.6	0.51	0.77	0.35
SOBP	SOBP;FLJ10159;JXC1;MRAMS	sine oculis binding protein homolog (Drosophila)	0.58	0.46	0.77	0.33
C10orf116	C10orf116;APM2;apM-2	chromosome 10 open reading frame 116	0.56	0.41	0.77	0.3
DCAF7	DCAF7;AN11;HAN11;SWAN-1;WDR68	DDB1 and CUL4 associated factor 7	0.54	0.45	0.77	0.3
JAK2	JAK2;JTK10	Janus kinase 2	0.34	0.26	0.77	0.22
SHFM1	SHFM1;DSS1;ECD;SEM1;SHFD1;SHSF1;Shfdg1	split hand/foot malformation (ectrodactyly) type 1	0.59	0.46	0.78	0.31
LRRC24	LRRC24;LRRC14OS;MGC111484	leucine rich repeat containing 24	0.58	0.44	0.78	0.33
ENHO	ENHO;C9orf165;UNQ470	energy homeostasis associated	0.56	0.48	0.78	0.32
EGR4	EGR4;NGFI-C;NGFIC;PAT133	early growth response 4	0.55	0.48	0.78	0.33
SCNM1	SCNM1;MGC3180	sodium channel modifier 1	0.5	0.38	0.78	0.31
SLC1A3	SLC1A3;EA6;EAAT1;FLJ25094;GLAST;GLAST1	solute carrier family 1 (glial high affinity glutamate transporter), member 3	0.18	0.16	0.78	0.12
MPND	MPND;FLJ14981	MPN domain containing	0.6	0.52	0.79	0.34
DAG1	DAG1;156DAG;A3a;AGRNR;DAG;MDDGC7	dystroglycan 1 (dystrophin-associated glycoprotein 1)	0.56	0.44	0.79	0.32
MR1	MR1;FLJ31593;HLALS	major histocompatibility complex, class I-related	0.55	0.44	0.79	0.34
KIF18A	KIF18A;DKFZp434G2226;MS-KIF18A	kinesin family member 18A	0.55	0.46	0.79	0.35
CCNYL1	CCNYL1;FLJ40432	cyclin Y-like 1	0.55	0.47	0.79	0.31
SHROOM1	SHROOM1;APXL2;KIAA1960	shroom family member 1	0.6	0.51	0.8	0.36
RANBP9	RANBP9;BPM-L;BPM90;RANBPM;RanBP7	RAN binding protein 9	0.6	0.49	0.8	0.36
RAP1A	RAP1A;KREV-1;KREV1;RAP1;SMGP21	RAP1A, member of RAS oncogene family	0.58	0.45	0.8	0.34
IGDCC3	IGDCC3;HsT18880;PUNC	immunoglobulin superfamily, DCC subclass, member 3	0.58	0.49	0.8	0.34
MAGEA1	MAGEA1;CT1.1;MAGE1;MGC9326	melanoma antigen family A, 1 (directs expression of antigen MZ2-E)	0.56	0.44	0.8	0.33
IL12A	IL12A;CLMF;IL-12A;NFSK;NKSF1;P35	interleukin 12A (natural killer cell stimulatory factor 1, cytotoxic lymphocyte maturation factor 1, p35)	0.56	0.46	0.8	0.35
PTGES2	PTGES2;C9orf15;FLJ14038;GBF1;MGC11289;PGES2;mPGES-2	prostaglandin E synthase 2	0.53	0.42	0.8	0.36
UGT1A4	UGT1A4;HUG-BR2;UDPGT;UDPGT 1-4;UGT-1D;UGT1-04;UGT1.4;UGT1D	UDP glucuronosyltransferase 1 family, polypeptide A4	0.52	0.46	0.8	0.32
CRNN	CRNN;C1orf10;DRC1;PDRC1;SEP53	cornulin	0.49	0.4	0.8	0.3
ALDH9A1	ALDH9A1;ALDH4;ALDH7;ALDH9;E3;TMABADH	aldehyde dehydrogenase 9 family, member A1	0.48	0.42	0.8	0.31
HOXD9	HOXD9;HOX4;HOX4C;Hox-4.3;Hox-5.2	homeobox D9	0.47	0.36	0.8	0.31
LRRC33	LRRC33;GARPL1;MGC50789;UNQ3030	leucine rich repeat containing 33	0.4	0.34	0.8	0.24
PAX5	PAX5;BSAP	paired box 5	0.5	0.76	1.62	0.6
PPP1R42	PPP1R42;LRRC67;TLLR;dtr	protein phosphatase 1, regulatory subunit 42	0.46	0.74	1.62	0.62
LAMA2	LAMA2;LAMM	laminin, alpha 2	0.4	0.67	1.63	0.47
GPR133	GPR133;DKFZp434B1272;FLJ16770;MGC138512;MGC138514;PGR25	G protein-coupled receptor 133	0.56	0.85	1.64	0.64
SEZ6	SEZ6	seizure related 6 homolog (mouse)	0.52	0.88	1.64	0.67
ERGIC3	ERGIC3;C20orf47;CGI-54;Erv46;NY-BR-84;PRO0989;SDBCAG84;dJ477O4.2	ERGIC and golgi 3	0.5	0.84	1.64	0.6
CRYAA	CRYAA;CRYA1;HSPB4	crystallin, alpha A	0.5	0.85	1.66	0.57
LEMD3	LEMD3;MAN1	LEM domain containing 3	0.6	1.04	1.67	0.7
ALPK2	ALPK2;FLJ34875;FLJ43253;HAK	alpha-kinase 2	0.54	0.88	1.67	0.79
PHYH	PHYH;LN1;LNAP1;PAHX;PHYH1;RD	phytanoyl-CoA 2-hydroxylase	0.5	0.78	1.67	0.62
GRK4	GRK4;GPRK2L;GPRK4;GRK4a;IT11	G protein-coupled receptor kinase 4	0.46	0.82	1.67	0.59
SCAND3	SCAND3;DKFZp434N092;FLJ31087;KIAA1925;MGC131782;MGC133310;MGC133311;ZFP38-L;ZNF305P2;ZNF452;dJ1186N24.3	SCAN domain containing 3	0.43	0.7	1.67	0.5
IL17B	IL17B;IL-17B;IL-20;MGC138900;MGC138901;NIRF;ZCYTO7	interleukin 17B	0.42	0.7	1.67	0.48
CEPT1	CEPT1;DKFZp313G0615;MGC45223	choline/ethanolamine phosphotransferase 1	0.55	0.88	1.68	0.77
HNRNPM	HNRNPM;CEAR;DKFZp547H118;HNRNPM4;HNRPM;HNRPM4;HTGR1;NAGR1;hnRNP M	heterogeneous nuclear ribonucleoprotein M	0.39	0.7	1.7	0.49

EPB41L3	EPB41L3;4.1B;DAL-1;DAL1;FLJ37633;KIAA0987	erythrocyte membrane protein band 4.1-like 3	0.55	0.96	1.71	0.64
KCNB2	KCNB2;KV2.2	potassium voltage-gated channel, Shab-related subfamily, member 2	0.55	0.92	1.71	0.72
NOL11	NOL11;DKFZp586L0724	nucleolar protein 11	0.52	0.96	1.71	0.74
LSM2	LSM2;C6orf28;G7B;YBL026W;snRNP	LSM2 homolog, U6 small nuclear RNA associated (S. cerevisiae)	0.52	0.86	1.71	0.65
GOLGA1	GOLGA1;MGC33154;golgin-97	golgin A1	0.41	0.7	1.72	0.49
FOSL2	FOSL2;FLJ23306;FRA2	FOS-like antigen 2	0.49	0.86	1.73	0.67
GTF2H2	GTF2H2;BTF2;BTF2P44;MGC102806;T-BTF2P44;TFIIH	general transcription factor IIH, polypeptide 2, 44kDa	0.38	0.64	1.73	0.55
XCR1	XCR1;CCXCR1;GPR5	chemokine (C motif) receptor 1	0.48	0.81	1.74	0.73
SPSB4	SPSB4;SSB-4;SSB4	splA/ryanodine receptor domain and SOCS box containing 4	0.6	1	1.75	0.72
COPS8	COPS8;COP9;CSN8;MGC1297;MGC43256;SGN8	COP9 constitutive photomorphogenic homolog subunit 8 (Arabidopsis)	0.52	0.88	1.75	0.63
CHCHD4	CHCHD4;FLJ31709;MIA40;TIMM40	coiled-coil-helix-coiled-coil-helix domain containing 4	0.42	0.74	1.75	0.57
ZBTB5	ZBTB5	zinc finger and BTB domain containing 5	0.56	1.02	1.76	0.7
PLXNA3	PLXNA3;6.3;HSSEXGENE;PLEXIN-A3;PLXN3;PLXN4;SEX;XAP-6	plexin A3	0.55	0.91	1.76	0.72
IQSEC1	IQSEC1;ARF-GEP100;ARFGEP100;BRAG2;GEP100;KIAA0763	IQ motif and Sec7 domain 1	0.36	0.65	1.77	0.45
COG7	COG7;CDG2E	component of oligomeric golgi complex 7	0.36	0.68	1.77	0.45
WNT5A	WNT5A;hWNT5A	wingless-type MMTV integration site family, member 5A	0.34	0.62	1.77	0.44
ANXA5	ANXA5;ANX5;ENX2;PP4	annexin A5	0.51	0.9	1.79	0.74
SMPD2	SMPD2;ISC1;NSMASE;NSMASE1	sphingomyelin phosphodiesterase 2, neutral membrane (neutral sphingomyelinase)	0.42	0.74	1.79	0.63
NOL12	NOL12;FLJ34609;MGC3731;Nop25;dJ37E16.7	nucleolar protein 12	0.5	0.93	1.8	0.66
RPE	RPE;MGC2636;RPE2-1	ribulose-5-phosphate-3-epimerase	0.38	0.74	1.81	0.51
C11orf10	C11orf10	chromosome 11 open reading frame 10	0.32	0.58	1.86	0.4
UNC50	UNC50;DKFZp564G0222;GMH1;UNCL;URP	unc-50 homolog (C. elegans)	0.6	1.06	1.87	0.9
SMG1	SMG1;61E3.4;ATX;KIAA0421;LIP	smg-1 homolog, phosphatidylinositol 3-kinase-related kinase (C. elegans)	0.46	0.82	1.87	0.75
C16orf3	C16orf3;MGC132509	chromosome 16 open reading frame 3	0.38	0.7	1.88	0.5
EP300	EP300;KAT3B;RSTS2;p300	E1A binding protein p300	0.38	0.68	1.89	0.53
POLR2J	POLR2J;MGC71910;POLR2J1;RPB11;RPB11A;RPB11m;hRPB14	polymerase (RNA) II (DNA directed) polypeptide J, 13.3kDa	0.33	0.62	1.92	0.46
CDK18	CDK18;PCTAIRE;PCTAIRE3;PCTK3	cyclin-dependent kinase 18	0.31	0.6	1.94	0.49
CREB3L2	CREB3L2;BBF2H7;MGC131709;MGC71006	cAMP responsive element binding protein 3-like 2	0.53	1.02	1.98	0.77
DCTN1	DCTN1;DAP-150;DP-150;P135	dynactin 1	0.32	0.69	2.08	0.47
DOCK9	DOCK9;DKFZp686C11110;DKFZp686D2047;DKFZp686N04132;FLJ11949;FLJ16744;FLJ44528;FLJ45282;FLJ45601;KIAA1058;KIAA1085;ZIZ1;ZIZIMIN1	dedicator of cytokinesis 9	0.4	0.86	2.09	0.62
SERPINA3	SERPINA3;AACT;ACT;GIG25;MGC88254	serpin peptidase inhibitor, clade A (alpha-1 antiproteinase, antitrypsin), member 3	0.34	0.66	2.1	0.5
ARSG	ARSG;KIAA1001	arylsulfatase G	0.57	1.23	2.28	0.96
IP6K2	IP6K2;IHPK2;PIUS	inositol hexakisphosphate kinase 2	0.37	0.85	2.28	0.77
ST6GAL1	ST6GAL1;MGC48859;SIAT1;ST6Gall;ST6N	ST6 beta-galactosamide alpha-2,6-sialyltranferase 1	0.22	0.5	2.33	0.36
PEF1	PEF1;ABP32;PEF1A	penta-EF-hand domain containing 1	0.34	0.96	2.65	0.73
RHOV	RHOV;ARHV;CHP;WRCH2	ras homolog gene family, member V	0.55	0.84	1.44	0.55
BPIFB2	BPIFB2;BPIL1;C20orf184;LPLUNC2;RYSR;dJ726C3.2	BPI fold containing family B, member 2	0.54	0.8	1.44	0.61
VKORC1	VKORC1;EDTP308;FLJ00289;IMAGE3455200;MGC2694;MST134;MST576;VKCFD2;VKOR	vitamin K epoxide reductase complex, subunit 1	0.45	0.71	1.44	0.49
CCDC60	CCDC60;MGC39827	coiled-coil domain containing 60	0.58	0.9	1.45	0.63
C6orf127	C6orf127;ESP32;dJ510O8.6	chromosome 6 open reading frame 127	0.54	0.82	1.45	0.61
SAMD9	SAMD9;C7orf5;DRIF1;FLJ20073;KIAA2004;NFTC;OEF1;OEF2	sterile alpha motif domain containing 9	0.53	0.8	1.45	0.53
RSBN1L	RSBN1L;FLJ42526;FLJ45813;MGC71764	round spermatid basic protein 1-like	0.52	0.81	1.45	0.56
RABEPK	RABEPK;DKFZp686P1077;RAB9P40;bA65N13.1;p40	Rab9 effector protein with kelch motifs	0.51	0.8	1.45	0.59
DYRK1B	DYRK1B;MIRK	dual-specificity tyrosine-(Y)-phosphorylation regulated kinase 1B	0.5	0.8	1.45	0.56
IFNAR1	IFNAR1;AVP;IFN-alpha-REC;IFNAR;IFNBR;IFRC	interferon (alpha, beta and omega) receptor 1	0.6	0.89	1.46	0.6
FAM116A	FAM116A;FLJ34969	family with sequence similarity 116, member A	0.6	0.94	1.47	0.72
EVL	EVL;RNB6	Enah/Vasp-like	0.58	0.84	1.47	0.64
KLHL15	KLHL15;KIAA1677;MGC126148;MGC126149	kelch-like 15 (Drosophila)	0.56	0.86	1.47	0.59
AKAP12	AKAP12;AKAP250;DKFZp686M0430;DKFZp686O0331;FLJ20945;FLJ97621;SSeCKS	A kinase (PRKA) anchor protein 12	0.53	0.81	1.47	0.54
RNASE7	RNASE7;MGC133220	ribonuclease, RNase A family, 7	0.52	0.82	1.47	0.62
UNC45B	UNC45B;CMYA4;FLJ38610;MGC119540;MGC119541;SMUNC45;UNC45	unc-45 homolog B (C. elegans)	0.6	0.88	1.49	0.67
ZNF550	ZNF550;MGC41917	zinc finger protein 550	0.57	0.89	1.49	0.64
CASC4	CASC4;DKFZp459F1927;H63;MGC74708	cancer susceptibility candidate 4	0.57	0.83	1.49	0.62
OR2W3	OR2W3;OR2W3P;OR2W8P;OST718	olfactory receptor, family 2, subfamily W, member 3	0.56	0.87	1.49	0.58
KRT2	KRT2;CK-2e;K2e;KRT2A;KRT2E;KRTE;MGC116967;MGC116968	keratin 2	0.48	0.74	1.49	0.53
TPRG1L	TPRG1L;FAM79A;FLJ21811;RP11-46F15.3;h-mover;mover	tumor protein p63 regulated 1-like	0.48	0.76	1.49	0.53
VSIG4	VSIG4;CRIg;Z39IG	V-set and immunoglobulin domain containing 4	0.47	0.73	1.49	0.55
RPS6KA2	RPS6KA2;HU-2;MAPKAPK1C;RSK;RSK3;S6K-alpha;S6K-alpha2;p90-RSK3;pp90RSK3	ribosomal protein S6 kinase, 90kDa, polypeptide 2	0.46	0.75	1.49	0.53
SLC39A9	SLC39A9;FLJ11274;MGC74989;ZIP-9;ZIP9	solute carrier family 39 (zinc transporter), member 9	0.44	0.72	1.49	0.52
FGD3	FGD3;FLJ00004;MGC117260;ZFYVE5	FYVE, RhoGEF and PH domain containing 3	0.55	0.86	1.5	0.57
CYTH1	CYTH1;B2-1;CYTOHESIN-1;D17S811E;FLJ34050;FLJ41900;PSCD1;SEC7	cytohesin 1	0.53	0.82	1.5	0.62
TLK2	TLK2;MGC44450;PKU-ALPHA	tousled-like kinase 2	0.52	0.84	1.5	0.59
FGR	FGR;FLJ43153;MGC75096;SRC2;c-fgr;c-src2;p55-Fgr;p55c-fgr;p58-Fgr;p58c-fgr	Gardner-Rasheed feline sarcoma viral (v-fgr) oncogene homolog	0.5	0.8	1.5	0.56
TMEM45A	TMEM45A;DERP7;FLJ10134	transmembrane protein 45A	0.49	0.78	1.5	0.55
KCTD11	KCTD11;C17orf36;KCASH1;MGC129844;REN;REN/KCTD11	potassium channel tetramerisation domain containing 11	0.48	0.76	1.5	0.51
BMP15	BMP15;GDF9B;ODG2;POF4	bone morphogenetic protein 15	0.58	0.88	1.51	0.65
RUNX2	RUNX2;AML3;CBFA1;CCD;CCD1;MGC120022;MGC120023;OSF-2;OSF2;PEA2aA;PEBP2A1;PEBP2A2;PEBP2aA;PEBP2aA1	runt-related transcription factor 2	0.56	0.84	1.51	0.59
ARL10	ARL10;ARL10A;FLJ39249	ADP-ribosylation factor-like 10	0.53	0.82	1.51	0.59
GABARAPL2	GABARAPL2;ATG8;ATG8C;GATE-16;GATE16;GEF2	GABA(A) receptor-associated protein-like 2	0.51	0.78	1.51	0.52
AMBP	AMBP;A1M;EDC1;HCP;HI30;IATIL;ITI;ITIL;ITILC;UTI	alpha-1-microglobulin/bikunin precursor	0.48	0.74	1.51	0.57
SWT1	SWT1;C1orf26;FLJ20121;FLJ35944;HsSwt1	SWT1 RNA endoribonuclease homolog (S. cerevisiae)	0.48	0.76	1.51	0.51
KIAA0408	KIAA0408;FLJ43995;RP3-403A15.2	KIAA0408	0.6	0.88	1.52	0.66
GLI3	GLI3;ACLS;GCPS;GLI3-190;GLI3FL;PAP-A;PAPA;PAPA1;PAPB;PHS;PPDIV	GLI family zinc finger 3	0.6	0.89	1.52	0.63
TMEM164	TMEM164;FLJ20173;FLJ22679;bB360B22.3	transmembrane protein 164	0.5	0.8	1.52	0.57
MYPN	MYPN;MYOP	myopalladin	0.47	0.75	1.52	0.53
CALM2	CALM2;CALM1;CALM3;CAMII;FLJ99410;PHKD;PHKD2	calmodulin 2 (phosphorylase kinase, delta)	0.56	0.84	1.53	0.76
MFN1	MFN1;DKFZp762F247;FLJ20693;MGC41806;hfzo1;hfzo2	mitofusin 1	0.54	0.9	1.53	0.62
FAM109B	FAM109B;DKFZp686J07229;IPIP27B;MGC125831;MGC125832;Ses2	family with sequence similarity 109, member B	0.52	0.85	1.53	0.66
PDLIM7	PDLIM7;LMP1	PDZ and LIM domain 7 (enigma)	0.51	0.8	1.54	0.61
PTCH2	PTCH2;PTC2	patched 2	0.6	0.9	1.55	0.64
C4orf41	C4orf41;FLJ12716;TRAPPC11;foigr;gry	chromosome 4 open reading frame 41	0.6	0.9	1.55	0.71
PNPLA2	PNPLA2;1110001C14Rik;ATGL;DKFZp667M109;PEDF-R;TTS-2.2;TTS2	patatin-like phospholipase domain containing 2	0.59	0.94	1.55	0.63
PTCD1	PTCD1;KIAA0632	pentatricopeptide repeat domain 1	0.58	0.92	1.55	0.62
DRD4	DRD4;D4DR	dopamine receptor D4	0.55	0.84	1.55	0.76
CDK5RAP1	CDK5RAP1;C20orf34;C42;CDK5RAP1.3;CDK5RAP1.4;CGI-05;HSPC167	CDK5 regulatory subunit associated protein 1	0.51	0.8	1.55	0.62
DNAJB1	DNAJB1;HSPF1;Hdj1;Hsp40;RSPH16B;Sis1	DnaJ (Hsp40) homolog, subfamily B, member 1	0.42	0.68	1.55	0.45
OSGIN1	OSGIN1;BDGI;OKL38	oxidative stress induced growth inhibitor 1	0.55	0.81	1.56	0.66
ABCB7	ABCB7;ABC7;ASAT;Atm1p;EST140535	ATP-binding cassette, sub-family B (MDR/TAP), member 7	0.52	0.78	1.56	0.61
DRD5	DRD5;DBDR;DRD1B;DRD1L2;MGC10601	dopamine receptor D5	0.5	0.76	1.56	0.69
DUSP12	DUSP12;DUSP1;YVH1	dual specificity phosphatase 12	0.46	0.72	1.56	0.57
NUDT21	NUDT21;CFIM25;CPSF5;DKFZp686H1588	nudix (nucleoside diphosphate linked moiety X)-type motif 21	0.42	0.68	1.56	0.49
IFNA8	IFNA8;IFN-alphaB	interferon, alpha 8	0.58	0.88	1.57	0.7
SEC14L3	SEC14L3;MGC119366;MGC119367;MGC119368;TAP2	SEC14-like 3 (S. cerevisiae)	0.58	0.86	1.57	0.68
HAS1	HAS1;HAS	hyaluronan synthase 1	0.58	0.92	1.57	0.71
PHLPP1	PHLPP1;MGC161555;PHLPP;PLEKHE1;SCOP	PH domain and leucine rich repeat protein phosphatase 1	0.55	0.83	1.57	0.65
ZIC5	ZIC5	Zic family member 5	0.44	0.72	1.57	0.48
LIN7C	LIN7C;FLJ11215;LIN-7-C;LIN-7C;MALS-3;MALS3;VELI3	lin-7 homolog C (C. elegans)	0.57	0.88	1.58	0.66
PRM1	PRM1;CT94.1;P1	protamine 1	0.44	0.7	1.58	0.49
RAD23B	RAD23B;HHR23B;HR23B;P58	RAD23 homolog B (S. cerevisiae)	0.6	0.9	1.61	0.79
THY1	THY1;CD90;FLJ33325	Thy-1 cell surface antigen	0.44	0.7	1.61	0.56
DNAH9	DNAH9;DNAH17L;DNEL1;DYH9;Dnahc9;HL-20;HL20;KIAA0357	dynein, axonemal, heavy chain 9	0.4	0.66	1.61	0.5
RRAGB	RRAGB;RAGB;bA465E19.1	Ras-related GTP binding B	0.58	0.85	1.31	0.58
NFE2L2	NFE2L2;NRF2	nuclear factor (erythroid-derived 2)-like 2	0.56	0.82	1.33	0.57
RBFOX3	RBFOX3;FLJ56884;FLJ58356;FOX-3;FOX3;HRNBP3;NEUN	RNA binding protein, fox-1 homolog (C. elegans) 3	0.57	0.84	1.35	0.56
GNPNAT1	GNPNAT1;FLJ10607;GNPNAT;Gpnat1	glucosamine-phosphate N-acetyltransferase 1	0.56	0.82	1.36	0.54
TUBB4B	TUBB4B;Beta2;TUBB2;TUBB2C	tubulin, beta 4B class IVb	0.52	0.78	1.37	0.57
SYNJ2	SYNJ2;INPP5H;KIAA0348;MGC44422	synaptojanin 2	0.6	0.86	1.41	0.67
C1QTNF4	C1QTNF4;CTRP4;ZACRP4	C1q and tumor necrosis factor related protein 4	0.6	0.89	1.41	0.6
KRTAP27-1	KRTAP27-1	keratin associated protein 27-1	0.56	0.82	1.41	0.61
CD93	CD93;C1QR1;C1qR(P);C1qRP;CDw93;ECSM3;MXRA4;dJ737E23.1	CD93 molecule	0.55	0.82	1.41	0.54
PROM1	PROM1;AC133;CD133;CORD12;MCDR2;PROML1;RP41;STGD4	prominin 1	0.6	0.88	1.42	0.59
D4S234E	D4S234E;D4S234;NEEP21;NSG1;P21	DNA segment on chromosome 4 (unique) 234 expressed sequence	0.56	0.84	1.42	0.68
CD3EAP	CD3EAP;ASE-1;CAST;MGC118851;PAF49	CD3e molecule, epsilon associated protein	0.54	0.82	1.42	0.57
SLC16A8	SLC16A8;MCT3;REMP	solute carrier family 16, member 8 (monocarboxylic acid transporter 3)	0.5	0.76	1.42	0.5
NECAB2	NECAB2;EFCBP2	N-terminal EF-hand calcium binding protein 2	0.54	0.8	1.43	0.61
PPIL4	PPIL4;HDCME13P	peptidylprolyl isomerase (cyclophilin)-like 4	0.49	0.74	1.62	0.59
TTLL3	TTLL3;DKFZp434B103;DKFZp686D076;FLJ13898;HOTTL;MGC120529;MGC120530;MGC120532	tubulin tyrosine ligase-like family, member 3	0.31	0.56	1.62	0.38
RAC1	RAC1;MGC111543;Rac-1;TC-25;p21-Rac1	ras-related C3 botulinum toxin substrate 1 (rho family, small GTP binding protein Rac1)	0.48	0.73	1.63	0.64
SRSF1	SRSF1;ASF;FLJ53078;MGC5228;SF2;SF2p33;SFRS1;SRp30a	serine/arginine-rich splicing factor 1	0.32	0.57	1.73	0.42
IL17A	IL17A;CTLA8;IL-17;IL-17A;IL17	interleukin 17A	0.35	0.6	1.95	0.47
TKTL1	TKTL1;TKR;TKT2	transketolase-like 1	0.3	0.55	2.03	0.49
PNLIPRP2	PNLIPRP2;PLRP2	pancreatic lipase-related protein 2	0.6	0.85	1.45	0.6
RASGRF2	RASGRF2;DKFZp781H1715;GRF2;RAS-GRF2	Ras protein-specific guanine nucleotide-releasing factor 2	0.5	0.75	1.46	0.5
TNFRSF1B	TNFRSF1B;CD120b;TBPII;TNF-R-II;TNF-R75;TNFBR;TNFR1B;TNFR2;TNFR80;p75;p75TNFR	tumor necrosis factor receptor superfamily, member 1B	0.45	0.7	1.46	0.46
ST7L	ST7L;FAM4B;FLJ11657;FLJ20284;ST7R;STLR	suppression of tumorigenicity 7 like	0.57	0.82	1.47	0.65
RPS25	RPS25;S25	ribosomal protein S25	0.57	0.82	1.47	0.62
HLA-DRB3	HLA-DRB3;HLA-DR3B;HLA-DRB1;MGC117330	major histocompatibility complex, class II, DR beta 3	0.47	0.72	1.47	0.54
MRPL50	MRPL50;FLJ20493;FLJ21990;MRP-L50	mitochondrial ribosomal protein L50	0.57	0.82	1.48	0.65
MARCKS	MARCKS;80K-L;FLJ14368;FLJ90045;MACS;PKCSL;PRKCSL	myristoylated alanine-rich protein kinase C substrate	0.44	0.69	1.48	0.46

AKAP1	AKAP1;AKAP;AKAP121;AKAP149;AKAP84;D-AKAP1;MGC1807;PPP1R43;PRKA1;SAKAP84	A kinase (PRKA) anchor protein 1	0.56	0.81	1.49	0.64
UBAP1	UBAP1;MGC119669;MGC8710;NAG20;UAP;UBAP;UBAP-1	ubiquitin associated protein 1	0.52	0.77	1.5	0.64
EXOSC1	EXOSC1;CGI-108;CSL4;Csl4p;SKI4;Ski4p;hCsl4p;p13	exosome component 1	0.46	0.71	1.5	0.54
TSPY3	TSPY3	testis specific protein, Y-linked 3	0.43	0.68	1.53	0.51
KRTAP1-1	KRTAP1-1;HB2A;KAP1.1;KAP1.1A;KAP1.1B;KAP1.6;KAP1.7;KRTAP1.1;KRTAP1A;hKAP1.7	keratin associated protein 1-1	0.48	0.73	1.54	0.57
ARHGAP18	ARHGAP18;FLJ25728;MGC126757;MGC138145;MacGAP;bA307O14.2	Rho GTPase activating protein 18	0.49	0.74	1.56	0.56
AZIN1	AZIN1;MGC3832;MGC691;OAZI;OAZIN;ODC1L	antizyme inhibitor 1	0.59	0.84	1.29	0.58
PDE7A	PDE7A;HCP1;PDE7	phosphodiesterase 7A	0.55	0.8	1.33	0.6
PIK3CD	PIK3CD;P110DELTA;PI3K;p110D	phosphoinositide-3-kinase, catalytic, delta polypeptide	0.55	0.8	1.34	0.57
GPBP1L1	GPBP1L1;RP11-767N6.1	GC-rich promoter binding protein 1-like 1	0.57	0.82	1.35	0.58
SLC24A3	SLC24A3;NCKX3	solute carrier family 24 (sodium/potassium/calcium exchanger), member 3	0.52	0.77	1.35	0.53
TK2	TK2;MTDPS2;MTTK	thymidine kinase 2, mitochondrial	0.53	0.78	1.36	0.56
MRPS28	MRPS28;FLJ22853;MRP-S28;MRP-S35;MRPS35	mitochondrial ribosomal protein S28	0.59	0.84	1.37	0.56
PRPSAP2	PRPSAP2;MGC117304;MGC126719;MGC126721;PAP41	phosphoribosyl pyrophosphate synthetase-associated protein 2	0.57	0.82	1.39	0.59
ITGAM	ITGAM;CD11B;CR3A;MAC-1;MAC1A;MGC117044;MO1A;SLEB6	integrin, alpha M (complement component 3 receptor 3 subunit)	0.55	0.8	1.4	0.54
SIM1	SIM1;bHLHe14	single-minded homolog 1 (Drosophila)	0.52	0.77	1.4	0.52
METTL2B	METTL2B;FLJ11350;FLJ12760;METL;METTL2;METTL2A;PSENIP1	methyltransferase like 2B	0.6	0.85	1.42	0.57
SCAMP5	SCAMP5;DKFZp686L1799;MGC24969	secretory carrier membrane protein 5	0.52	0.77	1.43	0.52
MYBPH	MYBPH	myosin binding protein H	0.33	0.51	1.62	0.37
RACGAP1	RACGAP1;HsCYK-4;ID-GAP;MgcRacGAP	Rac GTPase activating protein 1	0.32	0.55	1.62	0.37
APBA3	APBA3;MGC:15815;X11L2;mint3	amyloid beta (A4) precursor protein-binding, family A, member 3	0.43	0.65	1.63	0.57
PSMD3	PSMD3;P58;RPN3;S3;TSTA2	proteasome (prosome, macropain) 26S subunit, non-ATPase, 3	0.17	0.26	1.63	0.19
POLR2L	POLR2L;RBP10;RPABC5;RPB10;RPB10beta;RPB7.6;hRPB7.6;hsRPB10b	polymerase (RNA) II (DNA directed) polypeptide L, 7.6kDa	0.41	0.62	1.64	0.55
ASAH1	ASAH1;AC;ACDase;ASAH;FLJ21558;FLJ22079;PHP;PHP32	N-acylsphingosine amidohydrolase (acid ceramidase) 1	0.24	0.4	1.64	0.31
RGS16	RGS16;A28-RGS14;A28-RGS14P;RGS-R	regulator of G-protein signaling 16	0.19	0.34	1.64	0.27
SNRPD1	SNRPD1;HsT2456;SMD1;SNRPD;Sm-D1	small nuclear ribonucleoprotein D1 polypeptide 16kDa	0.13	0.24	1.64	0.17
C17orf62	C17orf62;FLJ00406;FLJ90469;MGC4368	chromosome 17 open reading frame 62	0.38	0.56	1.65	0.44
KRTAP3-1	KRTAP3-1;KAP3.1;KRTAP3.1	keratin associated protein 3-1	0.44	0.68	1.66	0.54
KLK3	KLK3;APS;KLK2A1;PSA;hK3	kallikrein-related peptidase 3	0.24	0.4	1.66	0.3
CALR	CALR;CRT;FLJ26680;RO;SSA;cC1qR	calreticulin	0.37	0.6	1.67	0.45
PSMD8	PSMD8;HIP6;HYPF;MGC1660;Nin1p;Rpn12;S14;p31	proteasome (prosome, macropain) 26S subunit, non-ATPase, 8	0.16	0.29	1.67	0.2
SNW1	SNW1;Bx42;MGC119379;NCOA-62;PRPF45;Prp45;SKIIP;SKIP	SNW domain containing 1	0.24	0.38	1.68	0.31
RBM8A	RBM8A;BOV-1A;BOV-1B;BOV-1C;MDS014;RBM8;RBM8B;Y14;ZNRP;ZRNP1	RNA binding motif protein 8A	0.31	0.54	1.71	0.37
SIAH1	SIAH1;FLJ08065;SIAH1A	seven in absentia homolog 1 (Drosophila)	0.24	0.45	1.71	0.31
CCNB2	CCNB2;HsT17299	cyclin B2	0.14	0.24	1.71	0.2
MKS1	MKS1;BBS13;FLJ20345;MES;MKS;POC12	Meckel syndrome, type 1	0.28	0.46	1.72	0.36
ProSAPiP1	ProSAPiP1;KIAA0552	ProSAPiP1 protein	0.18	0.3	1.72	0.23
PSMD1	PSMD1;MGC133040;MGC133041;P112;Rpn2;S1	proteasome (prosome, macropain) 26S subunit, non-ATPase, 1	0.16	0.26	1.73	0.19
VCP	VCP;ALS14;IBMPFD;MGC131997;MGC148092;MGC8560;TERA;p97	valosin containing protein	0.08	0.15	1.74	0.1
VPS36	VPS36;C13orf9;DKFZp781E0871;EAP45	vacuolar protein sorting 36 homolog (S. cerevisiae)	0.44	0.68	1.75	0.53
ROBO1	ROBO1;DUTT1;FLJ21882;MGC131599;MGC133277;SAX3	roundabout, axon guidance receptor, homolog 1 (Drosophila)	0.3	0.52	1.75	0.38
COMT	COMT	catechol-O-methyltransferase	0.06	0.11	1.76	0.08
OR51T1	OR51T1;OR11-26	olfactory receptor, family 51, subfamily T, member 1	0.24	0.44	1.77	0.3
NEU1	NEU1;FLJ93471;NANH;NEU;SIAL1	sialidase 1 (lysosomal sialidase)	0.2	0.34	1.78	0.29
POLM	POLM;FLJ35482;Pol Mu;Tdt-N	polymerase (DNA directed), mu	0.06	0.11	1.78	0.09
INTS7	INTS7;C1orf73;DKFZp434B168;INT7	integrator complex subunit 7	0.4	0.64	1.8	0.51
HMGN1	HMGN1;FLJ27265;FLJ31471;HMG14;MGC104230;MGC117425	high mobility group nucleosome binding domain 1	0.28	0.48	1.81	0.42
C19orf43	C19orf43;MGC2803;fSAP18	chromosome 19 open reading frame 43	0.22	0.38	1.82	0.31
PSMD6	PSMD6;KIAA0107;Rpn7;S10;SGA-113M;p44S10	proteasome (prosome, macropain) 26S subunit, non-ATPase, 6	0.06	0.11	1.83	0.08
PSMD11	PSMD11;MGC3844;Rpn6;S9;p44.5	proteasome (prosome, macropain) 26S subunit, non-ATPase, 11	0.2	0.37	1.84	0.25
POLR2E	POLR2E;RPABC1;RPB5;XAP4;hRPB25;hsRPB5	polymerase (RNA) II (DNA directed) polypeptide E, 25kDa	0.28	0.5	1.85	0.35
ADAM33	ADAM33;C20orf153;DJ964F7.1;DKFZp434K0521;FLJ35308;FLJ36751;MGC149823;MGC71889	ADAM metallopeptidase domain 33	0.12	0.22	1.85	0.17
NGFRAP1	NGFRAP1;BEX3;Bex;DXS6984E;HGR74;NADE	nerve growth factor receptor (TNFRSF16) associated protein 1	0.12	0.23	1.87	0.18
UBC	UBC;HMG20	ubiquitin C	0	0.02	1.87	0.01
MED21	MED21;SRB7;SURB7	mediator complex subunit 21	0.09	0.17	1.88	0.14
ELAVL3	ELAVL3;DKFZp547J036;HUC;HUCL;MGC20653;PLE21	ELAV (embryonic lethal, abnormal vision, Drosophila)-like 3 (Hu antigen C)	0.06	0.12	1.89	0.08
NR1H2	NR1H2;LXR-b;LXRB;NER;NER-I;RIP15;UNR	nuclear receptor subfamily 1, group H, member 2	0.12	0.22	1.91	0.17
SUPT6H	SUPT6H;KIAA0162;MGC87943;SPT6;SPT6H;emb-5	suppressor of Ty 6 homolog (S. cerevisiae)	0.18	0.34	1.93	0.26
ISY1	ISY1;FSAP33;KIAA1160	ISY1 splicing factor homolog (S. cerevisiae)	0.16	0.28	1.93	0.22
PTPN6	PTPN6;HCP;HCPH;HPTP1C;PTP-1C;SH-PTP1;SHP-1;SHP-1L;SHP1	protein tyrosine phosphatase, non-receptor type 6	0.3	0.52	1.94	0.39
MAT2A	MAT2A;MATA2;MATII;SAMS2	methionine adenosyltransferase II, alpha	0.18	0.36	2.02	0.31
CCDC113	CCDC113;DKFZp434N1418;FLJ23555	coiled-coil domain containing 113	0.07	0.15	2.03	0.1
GABRB2	GABRB2;MGC119386;MGC119388;MGC119389	gamma-aminobutyric acid (GABA) A receptor, beta 2	0.13	0.26	2.04	0.2
POLR2B	POLR2B;POL2RB;RPB2;hRPB140;hsRPB2	polymerase (RNA) II (DNA directed) polypeptide B, 140kDa	0.12	0.24	2.05	0.19
GRIP2	GRIP2	glutamate receptor interacting protein 2	0.04	0.08	2.05	0.06
HLA-DOB	HLA-DOB;DOB;FLJ57033	major histocompatibility complex, class II, DO beta	0.06	0.12	2.07	0.09
UQCRQ	UQCRQ;QCR8;QP-C;QPC;UQCR7	ubiquinol-cytochrome c reductase, complex III subunit VII, 9.5kDa	0.08	0.18	2.1	0.13
SLC35B4	SLC35B4;FLJ14697;YEA;YEA4	solute carrier family 35, member B4	0.08	0.17	2.11	0.13
TCHHL1	TCHHL1;S100A17;THHL1;basalin	trichohyalin-like 1	0.11	0.22	2.15	0.18
ASMTL	ASMTL;ASMTLX;ASMTLY;ASTML	acetylserotonin O-methyltransferase-like	0.04	0.08	2.16	0.06
POLR2D	POLR2D;HSRBP4;HSRPB4;RBP4;RPB16	polymerase (RNA) II (DNA directed) polypeptide D	0.04	0.08	2.18	0.06
PARP6	PARP6;MGC131971;pART17	poly (ADP-ribose) polymerase family, member 6	0.18	0.38	2.23	0.27
POLR2C	POLR2C;RPB3;RPB31;hRPB33;hsRPB3	polymerase (RNA) II (DNA directed) polypeptide C, 33kDa	0.1	0.22	2.27	0.17
POLR2G	POLR2G;MGC138367;MGC138369;RPB19;RPB7;hRPB19;hsRPB7	polymerase (RNA) II (DNA directed) polypeptide G	0.07	0.14	2.28	0.12
C14orf177	C14orf177;FLJ25773	chromosome 14 open reading frame 177	0.12	0.26	2.34	0.2
PGAM5	PGAM5;BXLBV68;MGC5352	phosphoglycerate mutase family member 5	0.05	0.12	2.46	0.08
RPH3A	RPH3A;KIAA0985	rabphilin 3A homolog (mouse)	0.07	0.18	2.49	0.12
LTBR	LTBR;CD18;D12S370;LT-BETA-R;TNF-R-III;TNFCR;TNFR-RP;TNFR2-RP;TNFRSF3	lymphotoxin beta receptor (TNFR superfamily, member 3)	0.1	0.3	2.63	0.19
LCE3C	LCE3C;LEP15;SPRL3A	late cornified envelope 3C	0.08	0.22	2.75	0.17
STAR	STAR;STARD1	steroidogenic acute regulatory protein	0.05	0.14	2.75	0.1
JMJD6	JMJD6;KIAA0585;PSR;PTDSR;PTDSR1	jumonji domain containing 6	0.06	0.16	2.79	0.13
SEC31B	SEC31B;DKFZp434M183;SEC31B-1;SEC31L2	SEC31 homolog B (S. cerevisiae)	0.58	0.82	1.44	0.61
PDGFB	PDGFB;FLJ12858;PDGF2;SIS;SSV;c-sis	platelet-derived growth factor beta polypeptide	0.52	0.72	1.44	0.52
MOB1B	MOB1B;MATS2;MGC33910;MOB4A;MOBKL1A	MOB kinase activator 1B	0.48	0.7	1.44	0.5
RPL23AP82	RPL23AP82;MGC70863;RPL23A_43_1761	ribosomal protein L23a pseudogene 82	0.46	0.68	1.44	0.49
GRB2	GRB2;ASH;EGFRBP-GRB2;Grb3-3;MST084;MSTP084;NCKAP2	growth factor receptor-bound protein 2	0.44	0.62	1.44	0.48
DAO	DAO;DAAO;DAMOX;MGC35381;OXDA	D-amino-acid oxidase	0.43	0.6	1.44	0.47
HHLA3	HHLA3	HERV-H LTR-associating 3	0.42	0.66	1.44	0.49
PIK3IP1	PIK3IP1;HGFL;MGC17330;hHGFL(S)	phosphoinositide-3-kinase interacting protein 1	0.4	0.56	1.44	0.43
PARP3	PARP3;ADPRT3;ADPRTL2;ADPRTL3;IRT1;PADPRT-3	poly (ADP-ribose) polymerase family, member 3	0.4	0.58	1.44	0.39
PVRL3	PVRL3;CD113;CDW113;DKFZp566B0846;FLJ90624;NECTIN-3;PPR3;PRR3;PVRR3	poliovirus receptor-related 3	0.39	0.55	1.44	0.47
PSMB1	PSMB1;FLJ25321;HC5;KIAA1838;PMSB1;PSC5	proteasome (prosome, macropain) subunit, beta type, 1	0.38	0.56	1.44	0.41
RPL10	RPL10;AUTSX5;DKFZp686J1851;DXS648;DXS648E;FLJ23544;FLJ27072;L10;NOV;QM	ribosomal protein L10	0.38	0.56	1.44	0.38
KLF16	KLF16;BTEB4;DRRF;NSLP2	Kruppel-like factor 16	0.36	0.49	1.44	0.43
FBXO7	FBXO7;DKFZp686B08113;FBX;FBX07;FBX7;PARK15;PKPS	F-box protein 7	0.35	0.48	1.44	0.35
UBTD2	UBTD2;DCUBP;MGC30022	ubiquitin domain containing 2	0.32	0.47	1.44	0.34
SOCS7	SOCS7;NAP4;NCKAP4	suppressor of cytokine signaling 7	0.29	0.4	1.44	0.29
GUCA1A	GUCA1A;C6orf131;COD3;CORD14;GCAP;GCAP1;GUCA;GUCA1;dJ139D8.6	guanylate cyclase activator 1A (retina)	0.27	0.38	1.44	0.27
SMU1	SMU1;BWD;DKFZp761L0916;FLJ10805;FLJ10870;FLJ11970;MGC117363;RP11-54K16.3;SMU-1;fSAP57	smu-1 suppressor of mec-8 and unc-52 homolog (C. elegans)	0.12	0.2	1.44	0.13
STAMBPL1	STAMBPL1;ALMalpha;AMSH-FP;AMSH-LP;FLJ31524;KIAA1373;bA399O19.2	STAM binding protein-like 1	0.6	0.84	1.45	0.65
BRCC3	BRCC3;BRCC36;C6.1A;CXorf53	BRCA1/BRCA2-containing complex, subunit 3	0.57	0.77	1.45	0.63
FBXW7	FBXW7;AGO;CDC4;DKFZp686F23254;FBW6;FBW7;FBX30;FBXO30;FBXW6;FLJ16457;SEL-10;SEL10	F-box and WD repeat domain containing 7	0.56	0.78	1.45	0.56
CHST14	CHST14;ATCS;D4ST1;HNK1ST	carbohydrate (N-acetylgalactosamine 4-0) sulfotransferase 14	0.55	0.74	1.45	0.59
TYMS	TYMS;HST422;MGC88736;TMS;TS	thymidylate synthetase	0.55	0.78	1.45	0.55
KIF13A	KIF13A;FLJ27232;RBKIN;bA500C11.2	kinesin family member 13A	0.54	0.78	1.45	0.6
EWSR1	EWSR1;EWS;bK984G1.4	Ewing sarcoma breakpoint region 1	0.53	0.72	1.45	0.58
MRPL53	MRPL53;FLJ90728;L53MT;MGC20402	mitochondrial ribosomal protein L53	0.51	0.68	1.45	0.54
RIMS3	RIMS3;KIAA0237;NIM3;RIM3	regulating synaptic membrane exocytosis 3	0.46	0.64	1.45	0.49
KLHDC5	KLHDC5;Ctb9;KIAA1340;MGC131714	kelch domain containing 5	0.44	0.66	1.45	0.5
AGXT2	AGXT2;AGT2;DAIBAT	alanine--glyoxylate aminotransferase 2	0.42	0.56	1.45	0.45
RGS20	RGS20;RGSZ1;ZGAP1	regulator of G-protein signaling 20	0.41	0.6	1.45	0.41
NAB1	NAB1	NGFI-A binding protein 1 (EGR1 binding protein 1)	0.36	0.55	1.45	0.37
MOB3A	MOB3A;MOB1C;MOBKL2A;moblak	MOB kinase activator 3A	0.36	0.52	1.45	0.36
DEFB105B	DEFB105B;DEFB105A	defensin, beta 105B	0.3	0.44	1.45	0.33
PSMC6	PSMC6;CADP44;MGC12520;P44;SUG2;p42	proteasome (prosome, macropain) 26S subunit, ATPase, 6	0.26	0.36	1.45	0.25
WSB2	WSB2;MGC10210;SBA2	WD repeat and SOCS box containing 2	0.6	0.84	1.46	0.61
S100A11	S100A11;MLN70;S100C	S100 calcium binding protein A11	0.6	0.84	1.46	0.61
CYP51A1	CYP51A1;CP51;CYP51;CYPL1;LDM;P450-14DM;P450L1	cytochrome P450, family 51, subfamily A, polypeptide 1	0.56	0.8	1.46	0.56
WDR46	WDR46;BING4;C6orf11;FP221;UTP7	WD repeat domain 46	0.54	0.7	1.46	0.55
EEF2	EEF2;EEF-2;EF2	eukaryotic translation elongation factor 2	0.51	0.71	1.46	0.56
PLEKHM1P	PLEKHM1P	pleckstrin homology domain containing, family M (with RUN domain) member 1 pseudogene	0.5	0.69	1.46	0.5
IL12RB1	IL12RB1;CD212;IL-12R-BETA1;IL12RB;MGC34454	interleukin 12 receptor, beta 1	0.48	0.68	1.46	0.48
OR10W1	OR10W1;OR10W1P;OR10W1Q;OR11-236;UNQ6469	olfactory receptor, family 10, subfamily W, member 1	0.45	0.64	1.46	0.49
TACC2	TACC2;AZU-1;ECTACC	transforming, acidic coiled-coil containing protein 2	0.44	0.66	1.46	0.47
HMGA2	HMGA2;BABL;HMGI-C;HMGIC;LIPO;STQTL9	high mobility group AT-hook 2	0.44	0.62	1.46	0.47
MVD	MVD;FP17780;MPD	mevalonate (diphospho) decarboxylase	0.43	0.65	1.46	0.44
HBB	HBB;CD113t-C;beta-globin	hemoglobin, beta	0.42	0.58	1.46	0.45
NCBP1	NCBP1;CBP80;MGC2087;NCBP;Sto1	nuclear cap binding protein subunit 1, 80kDa	0.42	0.62	1.46	0.47
SRSF3	SRSF3;SFRS3;SRp20	serine/arginine-rich splicing factor 3	0.42	0.62	1.46	0.47

KRTAP5-7	KRTAP5-7;KRTAP5-3;KRTAP5.7	keratin associated protein 5-7	0.4	0.62	1.46	0.44
BDAG1	BDAG1;KIAA1529;MGC131724	LOC100499484-C9orf174 readthrough	0.39	0.57	1.46	0.4
SLC13A2	SLC13A2;NADC1;NaCT;NaDC-1;SDCT1	solute carrier family 13 (sodium-dependent dicarboxylate transporter), member 2	0.29	0.46	1.46	0.32
MX2	MX2;MXB	myxovirus (influenza virus) resistance 2 (mouse)	0.29	0.47	1.46	0.33
CPSF1	CPSF1;CPSF160;HSU37012;P/cl.18	cleavage and polyadenylation specific factor 1, 160kDa	0.12	0.18	1.46	0.13
RAD54B	RAD54B;FSBP;RDH54	RAD54 homolog B (S. cerevisiae)	0.56	0.79	1.47	0.57
TMSB4X	TMSB4X;FX;PTMB4;TB4X;TMSB4	thymosin beta 4, X-linked	0.56	0.78	1.47	0.6
CALHM2	CALHM2;FAM26B	calcium homeostasis modulator 2	0.55	0.79	1.47	0.57
ITM2C	ITM2C;BRI3;BRICD2C;E25;E25C;ITM3	integral membrane protein 2C	0.55	0.77	1.47	0.59
TNN	TNN;TN-W	tenascin N	0.54	0.76	1.47	0.55
FAM58A	FAM58A;MGC29729;STAR	family with sequence similarity 58, member A	0.51	0.74	1.47	0.54
ZNF69	ZNF69;Cos5;MGC59928	zinc finger protein 69	0.48	0.66	1.47	0.51
CAD	CAD	carbamoyl-phosphate synthetase 2, aspartate transcarbamylase, and dihydroorotase	0.48	0.7	1.47	0.51
EMID1	EMID1;EMI5;EMU1;MGC50657;hEmu1	EMI domain containing 1	0.47	0.71	1.47	0.51
PDPR	PDPR;DKFZp686A088;DKFZp686D16130;FLJ10079;PDP3	pyruvate dehydrogenase phosphatase regulatory subunit	0.46	0.64	1.47	0.51
MTF1	MTF1;MGC23036;MTF-1;ZRF	metal-regulatory transcription factor 1	0.45	0.64	1.47	0.45
ADD2	ADD2;ADDB	adducin 2 (beta)	0.44	0.66	1.47	0.44
PDS5A	PDS5A;DKFZp686B19246;FLJ41012;KIAA0648;MGC131948;MGC161503;SCC-112;SCC112	PDS5, regulator of cohesion maintenance, homolog A (S. cerevisiae)	0.43	0.64	1.47	0.48
CYBASC3	CYBASC3;FLJ39724;LCYTB;MGC20446	cytochrome b, ascorbate dependent 3	0.42	0.62	1.47	0.42
MED6	MED6;NY-REN-28	mediator complex subunit 6	0.42	0.63	1.47	0.49
BYSL	BYSL;BYSTIN	bystin-like	0.4	0.55	1.47	0.4
INA	INA;FLJ18662;FLJ57501;MGC12702;NEF5;NF-66;TXBP-1	internexin neuronal intermediate filament protein, alpha	0.4	0.6	1.47	0.45
GGTLC2	GGTLC2;GGTL4;MGC96916	gamma-glutamyltransferase light chain 2	0.38	0.56	1.47	0.42
PCDHB15	PCDHB15;PCDH-BETA15	protocadherin beta 15	0.36	0.52	1.47	0.37
RBP4	RBP4	retinol binding protein 4, plasma	0.32	0.44	1.47	0.35
CCDC8	CCDC8;3M3;DKFZp564K0322	coiled-coil domain containing 8	0.21	0.32	1.47	0.24
CNNM1	CNNM1;ACDP1;CLP-1;FLJ31632	cyclin M1	0.58	0.82	1.48	0.63
FRMD8	FRMD8;FLJ32216;FLJ90369;MGC31785	FERM domain containing 8	0.52	0.68	1.48	0.57
CREB3L1	CREB3L1;OASIS	cAMP responsive element binding protein 3-like 1	0.5	0.74	1.48	0.5
MYO9B	MYO9B;CELIAC4;MYR5	myosin IXB	0.48	0.68	1.48	0.49
FAM176A	FAM176A;FLJ13391;TMEM166	family with sequence similarity 176, member A	0.44	0.57	1.48	0.44
GK5	GK5;FLJ33582;FLJ45739;MGC40579	glycerol kinase 5 (putative)	0.44	0.6	1.48	0.49
TUBGCP2	TUBGCP2;GCP2;MGC138162;SPBC97;Spc97p	tubulin, gamma complex associated protein 2	0.44	0.62	1.48	0.45
EFNB1	EFNB1;CFND;CFNS;EFL3;EPLG2;Elk-L;LERK2;MGC8782	ephrin-B1	0.38	0.6	1.48	0.42
RPS23	RPS23;FLJ35016;S23	ribosomal protein S23	0.36	0.55	1.48	0.44
OR4K14	OR4K14;OR14-18;OR14-22	olfactory receptor, family 4, subfamily K, member 14	0.35	0.52	1.48	0.36
SLC6A2	SLC6A2;NAT1;NET;NET1;SLC6A5	solute carrier family 6 (neurotransmitter transporter, noradrenalin), member 2	0.32	0.46	1.48	0.36
ABCC4	ABCC4;EST170205;MOAT-B;MOATB;MRP4	ATP-binding cassette, sub-family C (CFTR/MRP), member 4	0.3	0.48	1.48	0.34
OTUD3	OTUD3;DUBA4;KIAA0459;RP11-460G22.1	OTU domain containing 3	0.28	0.4	1.48	0.35
CDC20	CDC20;CDC20A;MGC102824;bA276H19.3;p55CDC	cell division cycle 20 homolog (S. cerevisiae)	0.28	0.43	1.48	0.28
PSMC4	PSMC4;MGC13687;MGC23214;MGC8570;MIP224;S6;TBP7	proteasome (prosome, macropain) 26S subunit, ATPase, 4	0.18	0.25	1.48	0.22
MFAP1	MFAP1	microfibrillar-associated protein 1	0.15	0.21	1.48	0.15
POLR2A	POLR2A;MGC75453;POLR2;POLRA;RPB1;RPBh1;RPO2;RPOL2;RpIILS;hRPB220;hsRPB1	polymerase (RNA) II (DNA directed) polypeptide A, 220kDa	0.02	0.03	1.48	0.02
ZNF518A	ZNF518A;DKFZp781O2147;MGC125707;MGC125710;ZNF518	zinc finger protein 518A	0.56	0.76	1.49	0.6
FAR1	FAR1;DKFZp686A0370;DKFZp686P18247;FLJ22728;FLJ33561;MLSTD2;SDR10E1	fatty acyl CoA reductase 1	0.52	0.76	1.49	0.58
SOX9	SOX9;CMD1;CMPD1;SRA1	SRY (sex determining region Y)-box 9	0.48	0.65	1.49	0.58
OTOG	OTOG;FLJ46346;OTGN;mlemp	otogelin	0.45	0.66	1.49	0.46
POLH	POLH;FLJ16395;FLJ21978;RAD30;RAD30A;XP-V;XPV	polymerase (DNA directed), eta	0.41	0.58	1.49	0.46
LCN8	LCN8;EP17;LCN5	lipocalin 8	0.37	0.56	1.49	0.38
NDC80	NDC80;HEC;HEC1;HsHec1;KNTC2;TID3;hsNDC80	NDC80 homolog, kinetochore complex component (S. cerevisiae)	0.36	0.48	1.49	0.37
NWD1	NWD1;MGC134940	NACHT and WD repeat domain containing 1	0.34	0.53	1.49	0.37
MERTK	MERTK;MER;MGC133349;RP38;c-mer	c-mer proto-oncogene tyrosine kinase	0.34	0.54	1.49	0.38
FOXA1	FOXA1;HNF3A;MGC33105;TCF3A	forkhead box A1	0.15	0.21	1.49	0.17
LRRC46	LRRC46;FLJ23553;MGC16309	leucine rich repeat containing 46	0.57	0.81	1.5	0.66
CITED2	CITED2;MRG1;P35SRJ	Cbp/p300-interacting transactivator, with Glu/Asp-rich carboxy-terminal domain, 2	0.51	0.74	1.5	0.58
DICER1	DICER1;DCR1;Dicer;HERNA;KIAA0928;MNG1	dicer 1, ribonuclease type III	0.5	0.72	1.5	0.55
P2RX5	P2RX5;LRH-1;MGC47755;P2X5;P2X5R	purinergic receptor P2X, ligand-gated ion channel, 5	0.46	0.68	1.5	0.54
MRM1	MRM1;FLJ22578	mitochondrial rRNA methyltransferase 1 homolog (S. cerevisiae)	0.43	0.64	1.5	0.43
CENPJ	CENPJ;BM032;CENP-J;CPAP;LAP;LIP1;MCPH6;MGC131581;MGC131582;MGC142222;MGC142224;SASS4;SCKL4;Sas-4	centromere protein J	0.42	0.61	1.5	0.43
ARHGEF12	ARHGEF12;DKFZp686O2372;KIAA0382;LARG;PRO2792	Rho guanine nucleotide exchange factor (GEF) 12	0.4	0.59	1.5	0.51
BOP1	BOP1;KIAA0124	block of proliferation 1	0.38	0.55	1.5	0.41
EGLN2	EGLN2;DKFZp434E026;EIT6;FLJ95603;HIF-PH1;HIFPH1;HPH-1;HPH-3;PHD1	egl nine homolog 2 (C. elegans)	0.32	0.48	1.5	0.37
MGA	MGA;FLJ12634;KIAA0518;MAD5;MXD5	MAX gene associated	0.31	0.45	1.5	0.34
KIF26B	KIF26B;FLJ10157;MGC35030	kinesin family member 26B	0.22	0.32	1.5	0.25
PALB2	PALB2;DKFZp667I166;DKFZp686E1054;FANCN;FLJ21816;PNCA3	partner and localizer of BRCA2	0.16	0.23	1.5	0.18
CSNK2A2	CSNK2A2;CK2A2;CSNK2A1;FLJ43934	casein kinase 2, alpha prime polypeptide	0.1	0.15	1.5	0.11
RAB35	RAB35;H-ray;RAB1C;RAY	RAB35, member RAS oncogene family	0.53	0.74	1.51	0.6
XRCC1	XRCC1;RCC	X-ray repair complementing defective repair in Chinese hamster cells 1	0.48	0.66	1.51	0.59
PHOX2B	PHOX2B;NBLST2;NBPhox;PMX2B	paired-like homeobox 2b	0.46	0.63	1.51	0.56
ALDH5A1	ALDH5A1;SSADH;SSDH	aldehyde dehydrogenase 5 family, member A1	0.45	0.69	1.51	0.54
SEC14L4	SEC14L4;TAP3	SEC14-like 4 (S. cerevisiae)	0.38	0.6	1.51	0.45
SCRT1	SCRT1;DKFZp547F072;SCRT;ZNF898	scratch homolog 1, zinc finger protein (Drosophila)	0.31	0.43	1.51	0.34
CADM4	CADM4;IGSF4C;NECL4;Necl-4;TSLL2;synCAM4	cell adhesion molecule 4	0.29	0.46	1.51	0.35
LOC400509	LOC400509	ENTREZ STATUS: REMAPPED TO GENE ID: 100652781	0.52	0.76	1.52	0.61
CCL17	CCL17;A-152E5.3;ABCD-2;MGC138271;MGC138273;SCYA17;TARC	chemokine (C-C motif) ligand 17	0.46	0.68	1.52	0.49
C11orf49	C11orf49;FLJ22210;MGC4707	chromosome 11 open reading frame 49	0.42	0.62	1.52	0.47
VPS54	VPS54;HCC8;SLP-8p;VPS54L;hVps54L	vacuolar protein sorting 54 homolog (S. cerevisiae)	0.41	0.62	1.52	0.43
TMED5	TMED5;CGI-100;DKFZp781B2248	transmembrane emp24 protein transport domain containing 5	0.38	0.52	1.52	0.42
ZNF382	ZNF382;FLJ14686;KS1	zinc finger protein 382	0.33	0.52	1.52	0.35
SLC25A14	SLC25A14;BMCP1;MGC149543;UCP5	solute carrier family 25 (mitochondrial carrier, brain), member 14	0.32	0.47	1.52	0.33
FAM164A	FAM164A;C8orf70	family with sequence similarity 164, member A	0.32	0.49	1.52	0.35
RPL23	RPL23;L23;MGC111167;MGC117346;MGC72008;rpL17	ribosomal protein L23	0.23	0.36	1.52	0.26
GPR132	GPR132;G2A;MGC99642	G protein-coupled receptor 132	0.22	0.32	1.52	0.29
PCBD1	PCBD1;DCOH;PCBD;PCD;PHS	pterin-4 alpha-carbinolamine dehydratase/dimerization cofactor of hepatocyte nuclear factor 1 alpha	0.2	0.3	1.52	0.22
ATP6V1B2	ATP6V1B2;ATP6B1B2;ATP6B2;HO57;VATB;VPP3;Vma2	ATPase, H+ transporting, lysosomal 56/58kDa, V1 subunit B2	0.13	0.19	1.52	0.17
GATM	GATM;AGAT;AT	glycine amidinotransferase (L-arginine:glycine amidinotransferase)	0.57	0.8	1.53	0.71
RFC2	RFC2;A1;MGC3665;RFC40	replication factor C (activator 1) 2, 40kDa	0.46	0.68	1.53	0.53
LHX8	LHX8;Lhx7	LIM homeobox 8	0.38	0.6	1.53	0.4
CFL1	CFL1;CFL	cofilin 1 (non-muscle)	0.38	0.56	1.53	0.43
PNPLA7	PNPLA7;C9orf111;FLJ31318;FLJ43070;FLJ44279;NTE-R1;NTEL1	patatin-like phospholipase domain containing 7	0.36	0.56	1.53	0.38
CBFA2T3	CBFA2T3;ETO2;MTG16;MTGR2;ZMYND4	core-binding factor, runt domain, alpha subunit 2; translocated to, 3	0.34	0.5	1.53	0.43
OBFC2A	OBFC2A;DKFZp667M1322;FLJ13624;FLJ22833;MGC111163;SOSS-B2;SSB2	oligonucleotide/oligosaccharide-binding fold containing 2A	0.12	0.2	1.53	0.15
RASIP1	RASIP1;FLJ20401;RAIN	Ras interacting protein 1	0.42	0.62	1.54	0.45
C16orf71	C16orf71;DKFZp686H2240;FLJ43261	chromosome 16 open reading frame 71	0.39	0.58	1.54	0.45
SVOPL	SVOPL;MGC46715	SVOP-like	0.35	0.53	1.54	0.39
WDR33	WDR33;FLJ11294;NET14;WDC146	WD repeat domain 33	0.35	0.54	1.54	0.4
GPR32	GPR32	G protein-coupled receptor 32	0.54	0.78	1.55	0.59
PYCR2	PYCR2;FLJ54750;P5CR2	pyrroline-5-carboxylate reductase family, member 2	0.34	0.5	1.55	0.39
TMEM51	TMEM51;C1orf72;FLJ10199	transmembrane protein 51	0.2	0.32	1.55	0.24
INCENP	INCENP;FLJ31633;MGC111393	inner centromere protein antigens 135/155kDa	0.18	0.29	1.55	0.24
DYNC1H1	DYNC1H1;CMT20;DHC1;DHC1a;DKFZp686P2245;DNCH1;DNCL;DNECL;DYHC;Dnchc1;HL-3;KIAA0325;p22	dynein, cytoplasmic 1, heavy chain 1	0.42	0.62	1.56	0.54
NMRAL1	NMRAL1;FLJ25918;HSCARG;SDR48A1	NmrA-like family domain containing 1	0.38	0.59	1.56	0.42
DHX40	DHX40;DDX40;FLJ22060;FLJ34015;PAD	DEAH (Asp-Glu-Ala-His) box polypeptide 40	0.32	0.49	1.56	0.36
TINF2	TINF2;DKCA3;TIN2	TERF1 (TRF1)-interacting nuclear factor 2	0.31	0.5	1.56	0.33
C15orf38	C15orf38;MGC61550	chromosome 15 open reading frame 38	0.27	0.42	1.56	0.31
CECR6	CECR6	cat eye syndrome chromosome region, candidate 6	0.26	0.39	1.56	0.3
C1QA	C1QA	complement component 1, q subcomponent, A chain	0.14	0.2	1.56	0.15
GEMIN7	GEMIN7;SIP3	gem (nuclear organelle) associated protein 7	0.44	0.67	1.57	0.51
POU2F2	POU2F2;OCT2;OTF2;Oct-2	POU class 2 homeobox 2	0.42	0.66	1.57	0.51
ZNF442	ZNF442;FLJ14356	zinc finger protein 442	0.38	0.61	1.57	0.46
ACOT9	ACOT9;ACATE2;MT-ACT48;MTACT48	acyl-CoA thioesterase 9	0.38	0.58	1.57	0.41
GLI4	GLI4;HKR4;ZNF928	GLI family zinc finger 4	0.33	0.47	1.57	0.38
C16orf54	C16orf54;FLJ35681;MGC129957;MGC129958	chromosome 16 open reading frame 54	0.29	0.47	1.57	0.34
WEE1	WEE1;DKFZp686I18166;FLJ16446;WEE1A;WEE1hu	WEE1 homolog (S. pombe)	0.27	0.46	1.57	0.32
FAM107B	FAM107B;C10orf45;FLJ45505;MGC11034;MGC90261	family with sequence similarity 107, member B	0.46	0.64	1.58	0.51
C20orf201	C20orf201	chromosome 20 open reading frame 201	0.4	0.64	1.58	0.51
CNNM3	CNNM3;ACDP3;DKFZp434I1016;FLJ20018	cyclin M3	0.39	0.56	1.58	0.45
POPDC2	POPDC2;POP2	popeye domain containing 2	0.36	0.57	1.58	0.44
TCF23	TCF23;OUT;TCF-23;bHLHa24	transcription factor 23	0.2	0.31	1.58	0.25
STRA6	STRA6;FLJ12541;MCOPS9	stimulated by retinoic acid gene 6 homolog (mouse)	0.52	0.74	1.59	0.58
ANKRD13C	ANKRD13C;DKFZp566D1346;FLJ14998;RP4-677H15.5;dJ677H15.3	ankyrin repeat domain 13C	0.48	0.72	1.59	0.58
MED14	MED14;CRSP150;CRSP2;CSRP;CXorf4;DRIP150;EXLM1;MGC104513;RGR1;TRAP170	mediator complex subunit 14	0.36	0.6	1.59	0.39
TP53INP1	TP53INP1;DKFZp434M1317;FLJ22139;SIP;TP53DINP1;TP53INP1A;TP53INP1B;Teap;p53DINP1	tumor protein p53 inducible nuclear protein 1	0.32	0.48	1.59	0.35
ARCN1	ARCN1;COPD	archain 1	0.15	0.24	1.59	0.18
POLR2F	POLR2F;HRBP14.4;POLRF;RPABC2;RPB14.4;RPB6	polymerase (RNA) II (DNA directed) polypeptide F	0.12	0.2	1.59	0.16
MVP	MVP;LRP;VAULT1	major vault protein	0.38	0.61	1.6	0.42
LAD1	LAD1;LadA;MGC10355	ladinin 1	0.2	0.34	1.6	0.27
ARID3B	ARID3B;BDP;DRIL2	AT rich interactive domain 3B (BRIGHT-like)	0.16	0.27	1.6	0.18
SON	SON;BASS1;C21orf50;DBP-5;FLJ21099;FLJ33914;KIAA1019;NREBP;SON3	SON DNA binding protein	0.06	0.1	1.6	0.07

USP2	USP2;UBP41;USP9	ubiquitin specific peptidase 2	0.46	0.69	1.61	0.52
BSCL2	BSCL2;FLJ16651;GNG3LG;HMN5;MGC4694;SPG17	Berardinelli-Seip congenital lipodystrophy 2 (seipin)	0.38	0.59	1.61	0.45
TBC1D14	TBC1D14;FLJ32400;KIAA1322	TBC1 domain family, member 14	0.38	0.62	1.61	0.42
KIF23	KIF23;CHO1;KNSL5;MKLP-1;MKLP1	kinesin family member 23	0.3	0.46	1.61	0.36
DDX19B	DDX19B;DBP5;DDX19;RNAh	DEAD (Asp-Glu-Ala-As) box polypeptide 19B	0.12	0.18	1.61	0.13
CMTM5	CMTM5;CKLFSF5;FLJ37521	CKLF-like MARVEL transmembrane domain containing 5	0.6	0.81	1.26	0.62
EVX1	EVX1	even-skipped homeobox 1	0.6	0.74	1.26	0.64
TMEM116	TMEM116;FLJ90167	transmembrane protein 116	0.6	0.68	1.26	0.53
PER3	PER3;GIG13	period homolog 3 (Drosophila)	0.6	0.77	1.26	0.53
AKAP13	AKAP13;AKAP-Lbc;ARHGEF13;BRX;FLJ11952;FLJ43341;HA-3;Ht31;LBC;PROTO-LB;PROTO-LBC;c-lbc	A kinase (PRKA) anchor protein 13	0.6	0.8	1.26	0.53
RND2	RND2;ARHN;RHO7;RhoN	Rho family GTPase 2	0.59	0.76	1.26	0.6
C1orf65	C1orf65;FLJ35728;RP11-76K24.1	chromosome 1 open reading frame 65	0.59	0.77	1.26	0.57
NDUFA7	NDUFA7;B14.5a	NADH dehydrogenase (ubiquinone) 1 alpha subcomplex, 7, 14.5kDa	0.59	0.74	1.26	0.58
AKIP1	AKIP1;BCA3;C11orf17	A kinase (PRKA) interacting protein 1	0.59	0.78	1.26	0.52
HSPB2	HSPB2;HSP27;Hs.78846;LOH11CR1K;MGC133245;MKBP	heat shock 27kDa protein 2	0.58	0.75	1.26	0.52
ERCC3	ERCC3;BTF2;GTF2H;RAD25;TFIIH;XPB	excision repair cross-complementing rodent repair deficiency, complementation group 3 (xeroderma pigmentosum group B complementing)	0.58	0.68	1.26	0.55
PLAA	PLAA;DOA1;FLJ11281;FLJ12699;PLA2P;PLAP	phospholipase A2-activating protein	0.58	0.72	1.26	0.51
SRSF7	SRSF7;9G8;AAG3;SFRS7	serine/arginine-rich splicing factor 7	0.58	0.76	1.26	0.5
ZNF229	ZNF229;FLJ34222	zinc finger protein 229	0.57	0.7	1.26	0.56
RAET1L	RAET1L;ULBP6	retinoic acid early transcript 1L	0.57	0.7	1.26	0.53
RDH14	RDH14;PAN2;SDR7C4	retinol dehydrogenase 14 (all-trans/9-cis/11-cis)	0.57	0.7	1.26	0.5
PRUNE	PRUNE;DRES-17;DRES17;HTCD37	prune homolog (Drosophila)	0.57	0.72	1.26	0.49
MTM1	MTM1;CNM;MTMX;XLMTM	myotubularin 1	0.57	0.68	1.26	0.51
DCAF4	DCAF4;DKFZp434K114;MGC20547;MGC46524;WDR21;WDR21A	DDB1 and CUL4 associated factor 4	0.57	0.74	1.26	0.55
ELOVL3	ELOVL3;CIG-30;CIG30;MGC21435	ELOVL fatty acid elongase 3	0.56	0.73	1.26	0.53
C17orf42	C17orf42;FLJ22729;MGC24674;TEFM	chromosome 17 open reading frame 42	0.56	0.76	1.26	0.53
RGS14	RGS14	regulator of G-protein signaling 14	0.56	0.74	1.26	0.5
INF2	INF2;C14orf151;C14orf173;DKFZp762A0214;FLJ22056;FSGS5;MGC13251;pp9484	inverted formin, FH2 and WH2 domain containing	0.56	0.7	1.26	0.52
IFI30	IFI30;GILT;IFI-30;IP30;MGC32056	interferon, gamma-inducible protein 30	0.56	0.67	1.26	0.53
DCLK1	DCLK1;CL1;CLICK1;DCAMKL1;DCDC3A;DCLK;KIAA0369	doublecortin-like kinase 1	0.55	0.68	1.26	0.61
ST6GAL2	ST6GAL2;FLJ30711;FLJ37730;FLJ38334;KIAA1877;SIAT2;ST6GalII	ST6 beta-galactosamide alpha-2,6-sialyltranferase 2	0.55	0.7	1.26	0.54
JPH2	JPH2;CMH17;FLJ40969;JP-2;JP2	junctophilin 2	0.55	0.68	1.26	0.51
PIR	PIR	pirin (iron-binding nuclear protein)	0.55	0.68	1.26	0.53
C20orf30	C20orf30;HSPC274;dJ1116H23.2.1	chromosome 20 open reading frame 30	0.55	0.66	1.26	0.55
VRK1	VRK1;MGC117401;MGC138280;MGC142070;PCH1	vaccinia related kinase 1	0.55	0.75	1.26	0.54
CAMSAP3	CAMSAP3;KIAA1543;NEZHA	calmodulin regulated spectrin-associated protein family, member 3	0.55	0.68	1.26	0.57
SGCD	SGCD;35DAG;CMD1L;DAGD;MGC22567;SG-delta;SGCDP;SGD	sarcoglycan, delta (35kDa dystrophin-associated glycoprotein)	0.54	0.66	1.26	0.55
PPP2R3B	PPP2R3B;FLJ60425;NYREN8;PPP2R3L;PPP2R3LY;PR48	protein phosphatase 2, regulatory subunit B", beta	0.54	0.64	1.26	0.48
WDTC1	WDTC1;ADP;DCAF9;KIAA1037	WD and tetratricopeptide repeats 1	0.54	0.62	1.26	0.51
GMEB1	GMEB1;P96PIF;PIF96	glucocorticoid modulatory element binding protein 1	0.52	0.62	1.26	0.48
HMGCR	HMGCR;LDLCQ3	3-hydroxy-3-methylglutaryl-CoA reductase	0.52	0.62	1.26	0.49
KIF20A	KIF20A;FLJ21151;MKLP2;RAB6KIFL	kinesin family member 20A	0.52	0.64	1.26	0.55
NUP205	NUP205;C7orf14	nucleoporin 205kDa	0.52	0.64	1.26	0.51
AOAH	AOAH	acyloxyacyl hydrolase (neutrophil)	0.51	0.62	1.26	0.48
LSM4	LSM4;GRP;YER112W	LSM4 homolog, U6 small nuclear RNA associated (S. cerevisiae)	0.51	0.58	1.26	0.46
SPEF1	SPEF1;C20orf28;CLAMP;DKFZp434I114;SPEF1A	sperm flagellar 1	0.5	0.64	1.26	0.46
KIAA0182	KIAA0182;GSE1	KIAA0182	0.5	0.66	1.26	0.49
MMAA	MMAA;MGC120010;MGC120011;MGC120012;MGC120013;cblA	methylmalonic aciduria (cobalamin deficiency) cblA type	0.49	0.62	1.26	0.42
SLC17A7	SLC17A7;BNPI;VGLUT1	solute carrier family 17 (sodium-dependent inorganic phosphate cotransporter), member 7	0.48	0.61	1.26	0.47
FRMD5	FRMD5;FLJ41022;MGC14161	FERM domain containing 5	0.48	0.52	1.26	0.41
SEMA3C	SEMA3C;SEMAE;SemE	sema domain, immunoglobulin domain (Ig), short basic domain, secreted, (semaphorin) 3C	0.47	0.64	1.26	0.47
CLINT1	CLINT1;CLINT;ENTH;EPN4;EPNR;FLJ46753;KIAA0171	clathrin interactor 1	0.47	0.63	1.26	0.44
STOML1	STOML1;FLJ36370;SLP-1;STORP;hUNC-24	stomatin (EPB72)-like 1	0.46	0.57	1.26	0.43
NIPAL1	NIPAL1;DKFZp686A06115;NPAL1	NIPA-like domain containing 1	0.45	0.58	1.26	0.43
ZNF567	ZNF567;MGC45586	zinc finger protein 567	0.44	0.57	1.26	0.41
NAPA	NAPA;SNAPA	N-ethylmaleimide-sensitive factor attachment protein, alpha	0.44	0.54	1.26	0.44
TFIP11	TFIP11;FLJ22086;NTR1;Spp382;TIP39;bK445C9.6;hNtr1	tuftelin interacting protein 11	0.44	0.55	1.26	0.41
TTLL8	TTLL8	tubulin tyrosine ligase-like family, member 8	0.43	0.58	1.26	0.45
JPH3	JPH3;CAGL237;FLJ44707;HDL2;JP-3;JP3;TNRC22	junctophilin 3	0.43	0.56	1.26	0.42
MZB1	MZB1;FLJ32987;MGC29506;PACAP;pERp1	marginal zone B and B1 cell-specific protein	0.43	0.58	1.26	0.45
C1orf50	C1orf50;MGC955	chromosome 1 open reading frame 50	0.42	0.53	1.26	0.38
SARS2	SARS2;FLJ20450;SARS;SARSM;SERS;SYS;SerRS;SerRSmt;mtSerRS	seryl-tRNA synthetase 2, mitochondrial	0.42	0.5	1.26	0.4
SETD8	SETD8;KMT5A;PR-Set7;SET07;SET8	SET domain containing (lysine methyltransferase) 8	0.42	0.5	1.26	0.36
SUPT5H	SUPT5H;FLJ34157;SPT5;SPT5H;Tat-CT1	suppressor of Ty 5 homolog (S. cerevisiae)	0.42	0.52	1.26	0.45
PSMA2	PSMA2;HC3;MU;PMSA2;PSC2	proteasome (prosome, macropain) subunit, alpha type, 2	0.4	0.5	1.26	0.37
SDHD	SDHD;CBT1;PGL;PGL1;SDH4	succinate dehydrogenase complex, subunit D, integral membrane protein	0.4	0.51	1.26	0.38
C4orf39	C4orf39;FLJ31659	chromosome 4 open reading frame 39	0.39	0.5	1.26	0.38
MYLK2	MYLK2;KMLC;MLCK;MLCK2;skMLCK	myosin light chain kinase 2	0.39	0.49	1.26	0.4
CADM1	CADM1;BL2;DKFZp686F1789;IGSF4;IGSF4A;MGC149785;MGC51880;NECL2;Necl-2;RA175;ST17;SYNCAM;TSLC1;sTSLC-1;sgIGSF;synCAM1	cell adhesion molecule 1	0.38	0.5	1.26	0.37
RPS17	RPS17;DBA4;MGC72007;RPS17L;RPS17L1;RPS17L2;S17	ribosomal protein S17	0.38	0.44	1.26	0.37
USPL1	USPL1;C13orf22;D13S106E;DKFZp781K2286;FLJ32952;RP11-121O19.1;bA121O19.1	ubiquitin specific peptidase like 1	0.37	0.45	1.26	0.33
BST2	BST2;CD317;TETHERIN	bone marrow stromal cell antigen 2	0.36	0.46	1.26	0.33
TUFT1	TUFT1	tuftelin 1	0.36	0.48	1.26	0.39
FADS3	FADS3;CYB5RP;LLCDL3	fatty acid desaturase 3	0.33	0.38	1.26	0.34
DOCK11	DOCK11;ACG;FLJ32122;FLJ43653;ZIZ2;bB128O4.1	dedicator of cytokinesis 11	0.32	0.42	1.26	0.34
ARHGAP36	ARHGAP36;FLJ30058	Rho GTPase activating protein 36	0.32	0.38	1.26	0.3
DTL	DTL;CDT2;DCAF2;L2DTL;RAMP	denticleless homolog (Drosophila)	0.3	0.36	1.26	0.29
TUBA1C	TUBA1C;MGC10851;MGC14580;TUBA6;bcm948	tubulin, alpha 1c	0.29	0.36	1.26	0.27
MPST	MPST;MGC24539;MST;TST2	mercaptopyruvate sulfurtransferase	0.28	0.38	1.26	0.26
WIPI1	WIPI1;ATG18;ATG18A;FLJ10055;WIPI49	WD repeat domain, phosphoinositide interacting 1	0.26	0.33	1.26	0.24
IK	IK;CSA2;MGC59741;RED	IK cytokine, down-regulator of HLA II	0.13	0.16	1.26	0.12
TTLL1	TTLL1;C22orf7;HS323M22B	tubulin tyrosine ligase-like family, member 1	0.6	0.76	1.27	0.65
DZANK1	DZANK1;ANKRD64;C20orf12;C20orf84;DKFZp781J2425;FLJ10600;FLJ30892;bA189K21.1;bA189K21.8;dJ568F9.2	double zinc ribbon and ankyrin repeat domains 1	0.6	0.74	1.27	0.53
CUTC	CUTC;RP11-483F11.3	cutC copper transporter homolog (E. coli)	0.59	0.78	1.27	0.58
OR1B1	OR1B1;OR9-26;OR9-B	olfactory receptor, family 1, subfamily B, member 1	0.58	0.72	1.27	0.56
EGFL6	EGFL6;DKFZp564P2063;MAEG;W80	EGF-like-domain, multiple 6	0.58	0.76	1.27	0.51
TNFRSF21	TNFRSF21;BM-018;CD358;DR6;MGC31965	tumor necrosis factor receptor superfamily, member 21	0.58	0.66	1.27	0.55
CRYL1	CRYL1;GDH;MGC149525;MGC149526;lambda-CRY	crystallin, lambda 1	0.57	0.72	1.27	0.59
ZNF700	ZNF700;DKFZp434I1610	zinc finger protein 700	0.57	0.78	1.27	0.54
CCDC84	CCDC84	coiled-coil domain containing 84	0.57	0.74	1.27	0.56
PIAS2	PIAS2;ARIP3;DIP;MGC102682;MIZ;MIZ1;PIASX;PIASX-ALPHA;PIASX-BETA;SIZ2;ZMIZ4	protein inhibitor of activated STAT, 2	0.57	0.72	1.27	0.53
EIF4G1	EIF4G1;DKFZp686A1451;EIF-4G1;EIF4F;EIF4G;EIF4GI;P220;PARK18	eukaryotic translation initiation factor 4 gamma, 1	0.57	0.7	1.27	0.52
UCN3	UCN3;MGC119002;SCP;SPC;UCNIII	urocortin 3 (stresscopin)	0.56	0.68	1.27	0.53
IRF5	IRF5;SLEB10	interferon regulatory factor 5	0.56	0.8	1.27	0.55
ACSF3	ACSF3;FLJ39242	acyl-CoA synthetase family member 3	0.56	0.73	1.27	0.49
ALG1	ALG1;CDG1K;HMAT1;HMT-1;HMT1;MT-1;Mat-1;hMat-1	asparagine-linked glycosylation 1, beta-1,4-mannosyltransferase homolog (S. cerevisiae)	0.56	0.69	1.27	0.49
STK35	STK35;CLIK1;STK35L1	serine/threonine kinase 35	0.56	0.71	1.27	0.59
NDFIP1	NDFIP1;MGC10924;N4WBP5	Nedd4 family interacting protein 1	0.56	0.75	1.27	0.5
DAND5	DAND5;CER2;CERL2;CKTSF1B3;COCO;CRL2;DANTE;GREM3;MGC126849;SP1	DAN domain family, member 5	0.55	0.68	1.27	0.52
LCE1C	LCE1C;LEP3	late cornified envelope 1C	0.55	0.71	1.27	0.48
KRT81	KRT81;HB1;Hb-1;KRTHB1;MLN137;ghHkb1;hHAKB2-1	keratin 81	0.55	0.68	1.27	0.52
GPATCH2	GPATCH2;CT110;FLJ10252;FLJ21048;GPATC2;MGC74998;PPP1R30;RP11-361K17.1	G patch domain containing 2	0.55	0.68	1.27	0.51
RNF2	RNF2;BAP-1;BAP1;DING;HIPI3;RING1B;RING2	ring finger protein 2	0.55	0.69	1.27	0.62
MYRIP	MYRIP;DKFZp586F1018;FLJ44025;MGC130034;MGC130035;SLAC2-C;SLAC2C	myosin VIIA and Rab interacting protein	0.54	0.62	1.27	0.5
AANAT	AANAT;DSPS;SNAT	aralkylamine N-acetyltransferase	0.54	0.62	1.27	0.55
ATP2A3	ATP2A3;SERCA3	ATPase, Ca++ transporting, ubiquitous	0.54	0.69	1.27	0.46
SERP1	SERP1;MGC117327;MGC133321;MGC133322;RAMP4	stress-associated endoplasmic reticulum protein 1	0.54	0.66	1.27	0.51
C16orf11	C16orf11	chromosome 16 open reading frame 11	0.54	0.7	1.27	0.48
TCL1A	TCL1A;TCL1	T-cell leukemia/lymphoma 1A	0.53	0.65	1.27	0.47
NSMCE2	NSMCE2;C8orf36;FLJ32440;MMS21;NSE2	non-SMC element 2, MMS21 homolog (S. cerevisiae)	0.53	0.72	1.27	0.5
ANGPT1	ANGPT1;AGP1;AGPT;ANG1	angiopoietin 1	0.52	0.64	1.27	0.56
CDC6	CDC6;CDC18L;HsCDC18;HsCDC6	cell division cycle 6 homolog (S. cerevisiae)	0.52	0.67	1.27	0.45
AK4	AK4;AK3;AK3L1;AK3L2;MGC166959	adenylate kinase 4	0.52	0.72	1.27	0.51
NEMF	NEMF;FLJ10051;NY-CO-1;SDCCAG1	nuclear export mediator factor	0.52	0.68	1.27	0.52
FAM108C1	FAM108C1;FLJ34461;MGC131546	family with sequence similarity 108, member C1	0.5	0.6	1.27	0.47
DNAJB12	DNAJB12;DJ10;DKFZp586B2023	DnaJ (Hsp40) homolog, subfamily B, member 12	0.5	0.62	1.27	0.5
DHX8	DHX8;DDX8;HRH1;PRP22;PRPF22	DEAH (Asp-Glu-Ala-His) box polypeptide 8	0.5	0.62	1.27	0.45
DDB1	DDB1;DDBA;UV-DDB1;XAP1;XPCE;XPE;XPE-BF	damage-specific DNA binding protein 1, 127kDa	0.5	0.64	1.27	0.5
FOLR1	FOLR1;FBP;FOLR	folate receptor 1 (adult)	0.49	0.62	1.27	0.48
SLC6A16	SLC6A16;NTT5	solute carrier family 6, member 16	0.48	0.6	1.27	0.47
PIGV	PIGV;FLJ20477;GPI-MT-II;PIG-V	phosphatidylinositol glycan anchor biosynthesis, class V	0.48	0.65	1.27	0.5
HYAL2	HYAL2;LUCA2	hyaluronoglucosaminidase 2	0.47	0.57	1.27	0.43
SLMAP	SLMAP;FLJ42206;KIAA1601;MGC138760;MGC138761;SLAP	sarcolemma associated protein	0.47	0.64	1.27	0.42
LSR	LSR;ILDR3;LISCH7;MGC10659;MGC48312;MGC48503	lipolysis stimulated lipoprotein receptor	0.47	0.59	1.27	0.44
ERP44	ERP44;KIAA0573;PDIA10;TXNDC4	endoplasmic reticulum protein 44	0.47	0.66	1.27	0.46
TNFSF4	TNFSF4;CD134L;CD252;GP34;OX-40L;OX4OL;TXGP1	tumor necrosis factor (ligand) superfamily, member 4	0.45	0.6	1.27	0.43
MMP25	MMP25;MMP-25;MMP20;MMP20A;MMPL1;MT-MMP 6;MT-MMP6;MT6-MMP;MT6MMP;MTMMP6	matrix metallopeptidase 25	0.45	0.56	1.27	0.42
AHSA1	AHSA1;AHA1;C14orf3;p38	AHA1, activator of heat shock 90kDa protein ATPase homolog 1 (yeast)	0.42	0.54	1.27	0.42
PRRT1	PRRT1;C6orf31;IFITMD7;NG5	proline-rich transmembrane protein 1	0.4	0.52	1.27	0.37

C5orf24	C5orf24;FLJ37562	chromosome 5 open reading frame 24	0.4	0.53	1.27	0.38
TTC22	TTC22;FLJ20619	tetratricopeptide repeat domain 22	0.38	0.5	1.27	0.35
IRS1	IRS1;HIRS-1	insulin receptor substrate 1	0.38	0.5	1.27	0.35
RALB	RALB	v-ral simian leukemia viral oncogene homolog B (ras related; GTP binding protein)	0.38	0.48	1.27	0.41
PGM2L1	PGM2L1;BM32A;FLJ32029	phosphoglucomutase 2-like 1	0.38	0.47	1.27	0.41
C8orf74	C8orf74	chromosome 8 open reading frame 74	0.37	0.5	1.27	0.35
PLOD3	PLOD3;LH3	procollagen-lysine, 2-oxoglutarate 5-dioxygenase 3	0.34	0.43	1.27	0.33
SF3B5	SF3B5;MGC3133;SF3b10;Ysf3	splicing factor 3b, subunit 5, 10kDa	0.32	0.44	1.27	0.32
RPS13	RPS13;S13	ribosomal protein S13	0.32	0.43	1.27	0.28
ZBTB2	ZBTB2;ZNF437	zinc finger and BTB domain containing 2	0.3	0.36	1.27	0.29
UBQLN4	UBQLN4;A1U;C1orf6;CIP75;UBIN	ubiquilin 4	0.3	0.42	1.27	0.28
BIRC5	BIRC5;API4;EPR-1	baculoviral IAP repeat containing 5	0.24	0.32	1.27	0.24
SRD5A2	SRD5A2;MGC138457	steroid-5-alpha-reductase, alpha polypeptide 2 (3-oxo-5 alpha-steroid delta 4-dehydrogenase alpha 2)	0.21	0.25	1.27	0.2
LYST	LYST;CHS;CHS1	lysosomal trafficking regulator	0.6	0.74	1.28	0.52
NKAIN4	NKAIN4;C20orf58;FAM77A;bA261N11.2	Na+/K+ transporting ATPase interacting 4	0.6	0.68	1.28	0.55
MED4	MED4;ARC36;DRIP36;FLJ10956;HSPC126;TRAP36;VDRIP	mediator complex subunit 4	0.6	0.76	1.28	0.54
SYNCRIP	SYNCRIP;FLJ31626;GRY-RBP;GRYRBP;HNRPQ1;NSAP1;PP68;dJ3J17.2;hnRNP-Q	synaptotagmin binding, cytoplasmic RNA interacting protein	0.6	0.74	1.28	0.56
TAF10	TAF10;TAF2A;TAF2H;TAFII30	TAF10 RNA polymerase II, TATA box binding protein (TBP)-associated factor, 30kDa	0.6	0.82	1.28	0.54
SIGLEC15	SIGLEC15;CD33L3;HsT1361;SIGLEC-15	sialic acid binding Ig-like lectin 15	0.59	0.78	1.28	0.52
RNF207	RNF207;C1orf188;FLJ32096;FLJ46380;FLJ46593	ring finger protein 207	0.59	0.74	1.28	0.67
POLE2	POLE2;DPE2	polymerase (DNA directed), epsilon 2 (p59 subunit)	0.58	0.73	1.28	0.53
FGF7	FGF7;HBGF-7;KGF	fibroblast growth factor 7	0.57	0.71	1.28	0.51
PPARGC1B	PPARGC1B;DKFZp686C1790;ERRL1;FLJ14284;PERC;PGC-1(beta);PGC1B	peroxisome proliferator-activated receptor gamma, coactivator 1 beta	0.57	0.68	1.28	0.6
ELN	ELN;FLJ38671;FLJ43523;SVAS;WBS;WS	elastin	0.57	0.7	1.28	0.51
RABEP2	RABEP2;FLJ23282;FRA	rabaptin, RAB GTPase binding effector protein 2	0.57	0.72	1.28	0.54
KLF3	KLF3;BKLF;MGC48279	Kruppel-like factor 3 (basic)	0.57	0.69	1.28	0.54
HDC	HDC;MGC163399	histidine decarboxylase	0.56	0.68	1.28	0.54
KIF21A	KIF21A;CFEOM1;DKFZp779C159;FEOM1;FEOM3A;FLJ20052;KIAA1708	kinesin family member 21A	0.56	0.76	1.28	0.51
SEC13	SEC13;D3S1231E;SEC13L1;SEC13R;npp-20	SEC13 homolog (S. cerevisiae)	0.56	0.7	1.28	0.52
C2orf62	C2orf62;MGC50811	chromosome 2 open reading frame 62	0.55	0.73	1.28	0.55
MORN4	MORN4;C10orf83;FLJ25925;bA548K23.4	MORN repeat containing 4	0.55	0.69	1.28	0.52
COL6A1	COL6A1;OPLL	collagen, type VI, alpha 1	0.55	0.72	1.28	0.49
NAT6	NAT6;FUS-2;FUS2	N-acetyltransferase 6 (GCN5-related)	0.55	0.7	1.28	0.47
KRTAP20-2	KRTAP20-2;KAP20.2;MGC133104	keratin associated protein 20-2	0.54	0.68	1.28	0.52
SELPLG	SELPLG;CD162;CLA;PSGL-1;PSGL1	selectin P ligand	0.54	0.73	1.28	0.54
S100PBP	S100PBP;DKFZp313K2325;FLJ12903;S100PBPR	S100P binding protein	0.53	0.67	1.28	0.49
MOB4	MOB4;2C4D;MGC12264;MOB1;MOB3;MOBKL3;PHOCN;PREI3	MOB family member 4, phocein	0.53	0.71	1.28	0.54
SYT4	SYT4;HsT1192;KIAA1342	synaptotagmin IV	0.52	0.65	1.28	0.52
SPEM1	SPEM1;C17orf83;FLJ40081	spermatid maturation 1	0.52	0.69	1.28	0.51
DNAJC12	DNAJC12;JDP1	DnaJ (Hsp40) homolog, subfamily C, member 12	0.52	0.68	1.28	0.52
SURF1	SURF1	surfeit 1	0.52	0.66	1.28	0.44
RPS24	RPS24;DBA3;DKFZp686N1586;S24	ribosomal protein S24	0.52	0.6	1.28	0.5
ATP6V0C	ATP6V0C;ATP6C;ATP6L;ATPL;VATL;VPPC;Vma3	ATPase, H+ transporting, lysosomal 16kDa, V0 subunit c	0.52	0.64	1.28	0.51
PSMB2	PSMB2;HC7-I;MGC104215;MGC126885	proteasome (prosome, macropain) subunit, beta type, 2	0.52	0.66	1.28	0.49
SKA3	SKA3;C13orf3;MGC4832;RAMA1	spindle and kinetochore associated complex subunit 3	0.52	0.7	1.28	0.53
ABRA	ABRA;STARS	actin-binding Rho activating protein	0.5	0.72	1.28	0.49
CCNK	CCNK;CPR4;MGC9113	cyclin K	0.5	0.64	1.28	0.44
OR11H12	OR11H12	olfactory receptor, family 11, subfamily H, member 12	0.5	0.6	1.28	0.46
ZXDB	ZXDB;ZNF905;dJ83L6.1	zinc finger, X-linked, duplicated B	0.49	0.6	1.28	0.46
PCDHAC1	PCDHAC1;PCDH-ALPHA-C1	protocadherin alpha subfamily C, 1	0.48	0.63	1.28	0.51
LRRC32	LRRC32;D11S833E;GARP	leucine rich repeat containing 32	0.48	0.59	1.28	0.48
CRADD	CRADD;MGC9163;RAIDD	CASP2 and RIPK1 domain containing adaptor with death domain	0.48	0.62	1.28	0.45
PTGIS	PTGIS;CYP8;CYP8A1;MGC126858;MGC126860;PGIS;PTGI	prostaglandin I2 (prostacyclin) synthase	0.48	0.66	1.28	0.43
POU3F2	POU3F2;BRN2;OCT7;OTF-7;OTF7;POUF3;brn-2;oct-7	POU class 3 homeobox 2	0.47	0.58	1.28	0.5
ZNF70	ZNF70;Cos17;MGC48959	zinc finger protein 70	0.47	0.6	1.28	0.41
CLLU1OS	CLLU1OS	chronic lymphocytic leukemia up-regulated 1 opposite strand	0.46	0.6	1.28	0.42
EFS	EFS;CAS3;CASS3;EFS1;EFS2;HEFS;SIN	embryonal Fyn-associated substrate	0.46	0.57	1.28	0.44
PALM2-AKAP	PALM2-AKAP2;AKAP2;DKFZp686H1948	PALM2-AKAP2 readthrough	0.46	0.58	1.28	0.47
GAS6	GAS6;AXLLG;AXSF;DKFZp666G247;FLJ34709	growth arrest-specific 6	0.46	0.63	1.28	0.42
ZCCHC9	ZCCHC9;DKFZp761J139;PPP1R41	zinc finger, CCHC domain containing 9	0.46	0.59	1.28	0.41
GPX1	GPX1;GPXD;GSHPX1;MGC14399;MGC88245	glutathione peroxidase 1	0.46	0.6	1.28	0.4
SNX9	SNX9;MST155;MSTP155;SDP1;SH3PX1;SH3PXD3A;WISP	sorting nexin 9	0.45	0.56	1.28	0.41
LCN9	LCN9;9230102I19Rik	lipocalin 9	0.44	0.53	1.28	0.47
MUSTN1	MUSTN1;DKFZp686G21125;MUSTANG	musculoskeletal, embryonic nuclear protein 1	0.44	0.58	1.28	0.39
EIF2S1	EIF2S1;EIF-2;EIF-2A;EIF-2alpha;EIF2;EIF2A	eukaryotic translation initiation factor 2, subunit 1 alpha, 35kDa	0.42	0.53	1.28	0.41
SLC2A5	SLC2A5;GLUT-5;GLUT5	solute carrier family 2 (facilitated glucose/fructose transporter), member 5	0.41	0.53	1.28	0.41
SLC4A7	SLC4A7;DKFZp686H168;NBC2;NBC3;SBC2;SLC4A6	solute carrier family 4, sodium bicarbonate cotransporter, member 7	0.36	0.46	1.28	0.31
ITGB8	ITGB8	integrin, beta 8	0.34	0.41	1.28	0.36
ACADL	ACADL;ACAD4;FLJ94052;LCAD	acyl-CoA dehydrogenase, long chain	0.31	0.38	1.28	0.33
PRC1	PRC1;ASE1;MGC1671;MGC3669	protein regulator of cytokinesis 1	0.26	0.33	1.28	0.25
C12orf10	C12orf10;MST024;MSTP024;MYG;MYG1	chromosome 12 open reading frame 10	0.22	0.28	1.28	0.2
SF3B14	SF3B14;HSPC175;Ht006;P14;SAP14;SF3B14a	splicing factor 3B, 14 kDa subunit	0.18	0.24	1.28	0.16
CARD11	CARD11;BIMP3;CARMA1;MGC133069	caspase recruitment domain family, member 11	0.6	0.76	1.29	0.66
ANAPC2	ANAPC2;APC2;RP11-350O14.5	anaphase promoting complex subunit 2	0.6	0.74	1.29	0.58
RTDR1	RTDR1;MGC16968	rhabdoid tumor deletion region gene 1	0.59	0.68	1.29	0.57
ANKRD33	ANKRD33;C12orf7;DKFZp686O1689	ankyrin repeat domain 33	0.58	0.72	1.29	0.58
TREX1	TREX1;AGS1;CRV;DKFZp434J0310;DRN3;HERNS	three prime repair exonuclease 1	0.58	0.74	1.29	0.57
SHF	SHF;MGC126650;MGC126652	Src homology 2 domain containing F	0.57	0.79	1.29	0.55
SALL3	SALL3;ZNF796	sal-like 3 (Drosophila)	0.57	0.71	1.29	0.57
WDR62	WDR62;C19orf14;DKFZp434J046;DKFZp686G1024;FLJ33298;MCPH2;MGC166976	WD repeat domain 62	0.57	0.74	1.29	0.53
SARS	SARS;FLJ36399;SERRS;SERS	seryl-tRNA synthetase	0.57	0.7	1.29	0.55
RGS5	RGS5;MST092;MST106;MST129;MSTP032;MSTP092;MSTP106;MSTP129	regulator of G-protein signaling 5	0.56	0.7	1.29	0.51
C17orf56	C17orf56;DKFZp451M1616;FLJ31528	chromosome 17 open reading frame 56	0.56	0.72	1.29	0.52
GART	GART;AIRS;GARS;GARTF;MGC47764;PAIS;PGFT;PRGS	phosphoribosylglycinamide formyltransferase, phosphoribosylglycinamide synthetase, phosphoribosylaminoimidazole synthetase	0.56	0.75	1.29	0.59
IL13	IL13;ALRH;BHR1;IL-13;MGC116786;MGC116788;MGC116789;P600	interleukin 13	0.55	0.74	1.29	0.55
CALM1	CALM1;CALM2;CALM3;CALML2;CAMI;DD132;PHKD;caM	calmodulin 1 (phosphorylase kinase, delta)	0.54	0.69	1.29	0.57
TRA2B	TRA2B;DKFZp686F18120;Htra2-beta;SFRS10;SRFS10;TRA2-BETA;TRAN2B	transformer 2 beta homolog (Drosophila)	0.54	0.62	1.29	0.52
TET1	TET1;CXXC6;FLJ10839;FLJ41442;KIAA1676;LCX;bA119F7.1	tet methylcytosine dioxygenase 1	0.53	0.64	1.29	0.54
HSPA14	HSPA14;HSP70-4;HSP70L1;MGC131990	heat shock 70kDa protein 14	0.52	0.62	1.29	0.55
SRBD1	SRBD1;FLJ10379	S1 RNA binding domain 1	0.52	0.73	1.29	0.51
GRHPR	GRHPR;GLXR;GLYD;PH2	glyoxylate reductase/hydroxypyruvate reductase	0.52	0.66	1.29	0.45
HOMER3	HOMER3;HOMER-3;VESL3	homer homolog 3 (Drosophila)	0.52	0.69	1.29	0.46
PFKL	PFKL;DKFZp686G1648;DKFZp686L2097;FLJ30173;FLJ40909;PFK-B	phosphofructokinase, liver	0.52	0.74	1.29	0.52
MSRB3	MSRB3;DFNB74;DKFZp686C1178;FLJ36866	methionine sulfoxide reductase B3	0.51	0.64	1.29	0.5
OMA1	OMA1;2010001O09Rik;DAB1;FLJ33782;MPRP-1;YKR087C;ZMPOMA1	OMA1 homolog, zinc metallopeptidase (S. cerevisiae)	0.51	0.64	1.29	0.48
SPHK1	SPHK1;SPHK	sphingosine kinase 1	0.5	0.7	1.29	0.49
C20orf111	C20orf111;HSPC207;Perit1;dJ1183I21.1	chromosome 20 open reading frame 111	0.5	0.66	1.29	0.44
ONECUT3	ONECUT3;OC3	one cut homeobox 3	0.5	0.65	1.29	0.47
C16orf59	C16orf59;FLJ13909	chromosome 16 open reading frame 59	0.49	0.62	1.29	0.46
CLN6	CLN6;CLN4A;FLJ20561;HsT18960;nclf	ceroid-lipofuscinosis, neuronal 6, late infantile, variant	0.49	0.65	1.29	0.49
RANBP2	RANBP2;ANE1;IIAE3;NUP358;TRP1;TRP2	RAN binding protein 2	0.49	0.64	1.29	0.5
C6orf64	C6orf64;DKFZp434H012;FLJ11101	chromosome 6 open reading frame 64	0.48	0.6	1.29	0.43
TAF9B	TAF9B;DN-7;DN7;TAF9L;TAFII31L;TFIID-31	TAF9B RNA polymerase II, TATA box binding protein (TBP)-associated factor, 31kDa	0.48	0.58	1.29	0.46
PITHD1	PITHD1;C1orf128;HT014;RP5-886K2.4;TXNL1CL	PITH (C-terminal proteasome-interacting domain of thioredoxin-like) domain containing 1	0.48	0.6	1.29	0.46
RPS8	RPS8;S8	ribosomal protein S8	0.47	0.58	1.29	0.45
PPP1R15B	PPP1R15B;CREP;FLJ14744	protein phosphatase 1, regulatory subunit 15B	0.44	0.55	1.29	0.43
POFUT1	POFUT1;FUT12;KIAA0180;MGC2482;O-FUT;O-Fuc-T;O-FucT-1	protein O-fucosyltransferase 1	0.43	0.54	1.29	0.42
RPL36	RPL36;DKFZp566B023;L36	ribosomal protein L36	0.42	0.56	1.29	0.46
ISM2	ISM2;DKFZp686E0215;FLJ32147;MGC119416;TAIL1;THSD3	isthmin 2 homolog (zebrafish)	0.41	0.53	1.29	0.37
CIAPIN1	CIAPIN1;2810413N20Rik;Anamorsin;DRE2;PRO0915	cytokine induced apoptosis inhibitor 1	0.4	0.49	1.29	0.4
SELT	SELT	selenoprotein T	0.4	0.5	1.29	0.38
SIX6	SIX6;MCOPCT2;OPTX2;Six9	SIX homeobox 6	0.38	0.47	1.29	0.37
POLR3B	POLR3B;C128;DKFZp686B10117;FLJ10388;RPC2	polymerase (RNA) III (DNA directed) polypeptide B	0.38	0.47	1.29	0.35
ELF1	ELF1	E74-like factor 1 (ets domain transcription factor)	0.36	0.51	1.29	0.35
CSNK2B	CSNK2B;CK2B;CK2N;CSK2B;G5A;MGC138222;MGC138224	casein kinase 2, beta polypeptide	0.36	0.52	1.29	0.37
OSBP2	OSBP2;ORP-4;ORP4;OSBPL1;OSBPL4	oxysterol binding protein 2	0.35	0.44	1.29	0.35
KIAA1967	KIAA1967;DBC-1;DBC1;NET35;p30 DBC;p30DBC	KIAA1967	0.34	0.45	1.29	0.31
HIST1H3D	HIST1H3D;H3/b;H3FB	histone cluster 1, H3d	0.34	0.46	1.29	0.31
TNPO2	TNPO2;FLJ12155;IPO3;KPNB2B;TRN2	transportin 2	0.26	0.38	1.29	0.26
TMEM220	TMEM220	transmembrane protein 220	0.24	0.31	1.29	0.24
PFN3	PFN3	profilin 3	0.18	0.22	1.29	0.17
NDE1	NDE1;FLJ20101;HOM-TES-87;LIS4;NUDE;NUDE1	nudE nuclear distribution gene E homolog 1 (A. nidulans)	0.17	0.21	1.29	0.16
SLC12A5	SLC12A5;KCC2;KIAA1176	solute carrier family 12 (potassium/chloride transporter), member 5	0.6	0.78	1.3	0.61
PDHA2	PDHA2;MGC149517;MGC149518;PDHAL	pyruvate dehydrogenase (lipoamide) alpha 2	0.6	0.74	1.3	0.53
RCSD1	RCSD1;CAPZIP;MGC126585;MGC126611;MGC21854;MK2S4	RCSD domain containing 1	0.6	0.74	1.3	0.6
ANXA1	ANXA1;ANX1;LPC1	annexin A1	0.6	0.82	1.3	0.61
C10orf55	C10orf55;bA417O11.3	chromosome 10 open reading frame 55	0.58	0.78	1.3	0.58
FAT2	FAT2;CDHF8;CDHR9;HFAT2;MEGF1	FAT tumor suppressor homolog 2 (Drosophila)	0.58	0.73	1.3	0.57
GTF3C6	GTF3C6;C6orf51;TFIIIC35;bA397G5.3	general transcription factor IIIC, polypeptide 6, alpha 35kDa	0.58	0.78	1.3	0.64

HIST1H2AI	HIST1H2AI;FLJ92027;H2A/c;H2AFC;HIST1H2AG;HIST1H2AK;HIST1H2AL;HIST1H2AM	histone cluster 1, H2ai	0.58	0.72	1.3	0.6
KLHL14	KLHL14	kelch-like 14 (Drosophila)	0.57	0.76	1.3	0.58
DACH1	DACH1;DACH;FLJ10138	dachshund homolog 1 (Drosophila)	0.56	0.72	1.3	0.53
F10	F10;FX;FXA	coagulation factor X	0.55	0.7	1.3	0.53
LOXL3	LOXL3;LOXL	lysyl oxidase-like 3	0.55	0.7	1.3	0.5
SFSWAP	SFSWAP;MGC167082;SFRS8;SWAP	splicing factor, suppressor of white-apricot homolog (Drosophila)	0.55	0.68	1.3	0.57
SLC7A7	SLC7A7;LAT3;LPI;MOP-2;Y+LAT1;y+LAT-1	solute carrier family 7 (amino acid transporter light chain, y+L system), member 7	0.54	0.69	1.3	0.51
GOLGA8A	GOLGA8A;GM88	golgin A8 family, member A	0.54	0.76	1.3	0.59
SLC10A4	SLC10A4;MGC29802;P4	solute carrier family 10 (sodium/bile acid cotransporter family), member 4	0.52	0.66	1.3	0.57
TLX2	TLX2;HOX11L1;NCX	T-cell leukemia homeobox 2	0.52	0.66	1.3	0.56
COASY	COASY;DPCK;FLJ35179;NBP;PPAT;UKR1;pOV-2	CoA synthase	0.52	0.68	1.3	0.56
ZSCAN20	ZSCAN20;KOX29;ZFP-31;ZNF31;ZNF360	zinc finger and SCAN domain containing 20	0.51	0.68	1.3	0.51
CYB5R1	CYB5R1;B5R.1;B5R1;NQO3A2;humb5R2	cytochrome b5 reductase 1	0.51	0.64	1.3	0.55
PXDNL	PXDNL;FLJ25471;VPO2	peroxidasin homolog (Drosophila)-like	0.5	0.61	1.3	0.48
TSHZ3	TSHZ3;FLJ54422;KIAA1474;TSH3;ZNF537	teashirt zinc finger homeobox 3	0.5	0.64	1.3	0.47
PDZD11	PDZD11;AIPP1;PDZK11;PISP	PDZ domain containing 11	0.5	0.7	1.3	0.47
HSD3B7	HSD3B7;PFIC4;SDR11E3	hydroxy-delta-5-steroid dehydrogenase, 3 beta- and steroid delta-isomerase 7	0.5	0.63	1.3	0.49
GAB2	GAB2;KIAA0571	GRB2-associated binding protein 2	0.5	0.68	1.3	0.45
CDK19	CDK19;CDC2L6;CDK11;KIAA1028;bA346C16.3	cyclin-dependent kinase 19	0.5	0.7	1.3	0.5
ALDH8A1	ALDH8A1;ALDH12;DJ352A20.2;DKFZp779D2315;MGC138650	aldehyde dehydrogenase 8 family, member A1	0.49	0.64	1.3	0.43
NR2C2	NR2C2;TAK1;TR2R1;TR4;hTAK1	nuclear receptor subfamily 2, group C, member 2	0.49	0.64	1.3	0.43
BRE	BRE;BRCC4;BRCC45	brain and reproductive organ-expressed (TNFRSF1A modulator)	0.49	0.7	1.3	0.51
FGF22	FGF22	fibroblast growth factor 22	0.46	0.56	1.3	0.48
MFSD3	MFSD3	major facilitator superfamily domain containing 3	0.46	0.6	1.3	0.44
ITGB3BP	ITGB3BP;CENP-R;CENPR;HSU37139;NRIF3;TAP20	integrin beta 3 binding protein (beta3-endonexin)	0.45	0.6	1.3	0.41
TNFAIP8L1	TNFAIP8L1;FLJ39932;MGC17791	tumor necrosis factor, alpha-induced protein 8-like 1	0.44	0.6	1.3	0.43
DENND1C	DENND1C;FAM31C;FLJ22757	DENN/MADD domain containing 1C	0.44	0.58	1.3	0.42
FOXL1	FOXL1;FKH6;FKHL11;FREAC7	forkhead box L1	0.44	0.6	1.3	0.47
TNKS1BP1	TNKS1BP1;FLJ45975;KIAA1741;TAB182	tankyrase 1 binding protein 1, 182kDa	0.44	0.57	1.3	0.4
COX7A2L	COX7A2L;COX7AR;COX7RP;EB1;SIG81	cytochrome c oxidase subunit VIIa polypeptide 2 like	0.44	0.56	1.3	0.44
RTN4IP1	RTN4IP1;MGC12934;NIMP	reticulon 4 interacting protein 1	0.42	0.59	1.3	0.44
IGLL1	IGLL1;14.1;AGM2;CD179b;IGL1;IGL5;IGLJ14.1;IGLL;IGO;IGVPB;VPREB2	immunoglobulin lambda-like polypeptide 1	0.4	0.54	1.3	0.4
RPS12	RPS12;S12	ribosomal protein S12	0.4	0.54	1.3	0.41
MRPS18C	MRPS18C;FLJ11146;FLJ22967;MRP-S18-1;MRPS18-1	mitochondrial ribosomal protein S18C	0.39	0.52	1.3	0.38
RPS4Y1	RPS4Y1;MGC119100;MGC5070;RPS4Y;S4	ribosomal protein S4, Y-linked 1	0.38	0.5	1.3	0.33
C13orf33	C13orf33;AWMS3;FLJ14834;MGC126673;MGC126675;hAWMS3	chromosome 13 open reading frame 33	0.38	0.52	1.3	0.37
NPTXR	NPTXR;NPR	neuronal pentraxin receptor	0.38	0.5	1.3	0.34
OR1L8	OR1L8;OR9-24	olfactory receptor, family 1, subfamily L, member 8	0.36	0.48	1.3	0.37
PLIN3	PLIN3;M6PRBP1;MGC11117;MGC2012;PP17;TIP47	perilipin 3	0.36	0.52	1.3	0.38
CDCA5	CDCA5;MGC16386;SORORIN	cell division cycle associated 5	0.32	0.4	1.3	0.29
GLIS3	GLIS3;FLJ38999;FLJ90578;MGC33662;ZNF515	GLIS family zinc finger 3	0.3	0.38	1.3	0.29
GJC2	GJC2;CX46.6;Cx47;GJA12;HLD2;LMPH1C;MGC105119;PMLDAR;SPG44	gap junction protein, gamma 2, 47kDa	0.23	0.32	1.3	0.21
SGOL1	SGOL1;FLJ14230;NY-BR-85;SGO;Sgo1	shugoshin-like 1 (S. pombe)	0.2	0.27	1.3	0.19
PVALB	PVALB;D22S749;MGC116759	parvalbumin	0.6	0.76	1.31	0.6
GNG13	GNG13;G(gamma)13;h2-35	guanine nucleotide binding protein (G protein), gamma 13	0.6	0.76	1.31	0.58
EDN1	EDN1;ET1;HDLCQ7;PPET1	endothelin 1	0.6	0.72	1.31	0.57
ARPP19	ARPP19;ARPP-16;ARPP-19;ARPP16;ENSAL;FLJ41622	cAMP-regulated phosphoprotein, 19kDa	0.6	0.8	1.31	0.61
SPATS1	SPATS1;FLJ25442;MGC119210;MGC119211;MGC119212;MGC119213;MGC133020;SPATA8;SRSP1	spermatogenesis associated, serine-rich 1	0.58	0.73	1.31	0.56
ZNF34	ZNF34;KOX32;MGC10520	zinc finger protein 34	0.58	0.8	1.31	0.53
TREH	TREH;MGC129621;TRE;TREA	trehalase (brush-border membrane glycoprotein)	0.57	0.73	1.31	0.54
DLST	DLST;DLTS	dihydrolipoamide S-succinyltransferase (E2 component of 2-oxo-glutarate complex)	0.57	0.74	1.31	0.63
FAM19A4	FAM19A4;FLJ25161;TAFA-4;TAFA4	family with sequence similarity 19 (chemokine (C-C motif)-like), member A4	0.56	0.8	1.31	0.62
FER1L5	FER1L5	fer-1-like 5 (C. elegans)	0.56	0.73	1.31	0.52
C17orf80	C17orf80;FLJ20721;HLC-8;MIG3	chromosome 17 open reading frame 80	0.56	0.66	1.31	0.52
SH3BP5L	SH3BP5L;FLJ33845;KIAA1720	SH3-binding domain protein 5-like	0.56	0.78	1.31	0.6
CKAP2	CKAP2;DKFZp686L1238;FLJ10749;LB1;TMAP;se20-10	cytoskeleton associated protein 2	0.56	0.74	1.31	0.54
FASTKD5	FASTKD5;FLJ13149;FLJ58294;dJ1187M17.5	FAST kinase domains 5	0.55	0.77	1.31	0.54
PIK3CA	PIK3CA;MGC142161;MGC142163;PI3K;p110-alpha	phosphoinositide-3-kinase, catalytic, alpha polypeptide	0.55	0.72	1.31	0.6
RPL26L1	RPL26L1;FLJ46904;RPL26P1	ribosomal protein L26-like 1	0.55	0.66	1.31	0.52
AKT2	AKT2;PKBB;PKBBETA;PRKBB;RAC-BETA	v-akt murine thymoma viral oncogene homolog 2	0.54	0.7	1.31	0.58
SLC26A9	SLC26A9	solute carrier family 26, member 9	0.53	0.65	1.31	0.58
MAGEH1	MAGEH1;APR-1;APR1;MAGEH	melanoma antigen family H, 1	0.53	0.72	1.31	0.5
ZNF827	ZNF827	zinc finger protein 827	0.52	0.7	1.31	0.53
KRTAP4-7	KRTAP4-7	ENTREZ STATUS: REMAPPED TO GENE ID: 100132476	0.52	0.7	1.31	0.48
TBC1D17	TBC1D17;FLJ12168	TBC1 domain family, member 17	0.52	0.64	1.31	0.54
SLC31A1	SLC31A1;COPT1;CTR1;MGC75487	solute carrier family 31 (copper transporters), member 1	0.52	0.66	1.31	0.47
TRAPPC6B	TRAPPC6B;FLJ17772;FLJ79549;TPC6	trafficking protein particle complex 6B	0.52	0.7	1.31	0.53
MYADM	MYADM;SB135	myeloid-associated differentiation marker	0.52	0.7	1.31	0.51
SH3TC2	SH3TC2;CMT4C;FLJ13605;KIAA1985;MNMN	SH3 domain and tetratricopeptide repeats 2	0.5	0.62	1.31	0.5
PSD3	PSD3;DKFZp761K1423;EFA6R;HCA67	pleckstrin and Sec7 domain containing 3	0.5	0.64	1.31	0.48
CALB1	CALB1;CALB	calbindin 1, 28kDa	0.48	0.66	1.31	0.47
DDX26B	DDX26B;DKFZp686G0470;FLJ41215;MGC88298	DEAD/H (Asp-Glu-Ala-Asp/His) box polypeptide 26B	0.48	0.68	1.31	0.52
RTN3	RTN3;ASYIP;HAP;NSPL2;NSPLII;RTN3-A1	reticulon 3	0.48	0.67	1.31	0.47
PYCRL	PYCRL;FLJ13852	pyrroline-5-carboxylate reductase-like	0.48	0.6	1.31	0.52
GPATCH1	GPATCH1;ECGP;FLJ10206;FLJ34516;FLJ38686;GPATC1	G patch domain containing 1	0.48	0.6	1.31	0.46
ARMC6	ARMC6;MGC19595;R30923_1	armadillo repeat containing 6	0.48	0.6	1.31	0.5
LOC650293	LOC650293	seven transmembrane helix receptor	0.46	0.59	1.31	0.45
CNIH2	CNIH2;CNIH-2;Cnil;MGC50896	cornichon homolog 2 (Drosophila)	0.46	0.64	1.31	0.48
DAAM2	DAAM2;KIAA0381;MGC90515;dJ90A20A.1	dishevelled associated activator of morphogenesis 2	0.45	0.6	1.31	0.4
SNRPA1	SNRPA1;Lea1	small nuclear ribonucleoprotein polypeptide A'	0.45	0.56	1.31	0.44
CHPT1	CHPT1;CPT;CPT1	choline phosphotransferase 1	0.45	0.64	1.31	0.48
MAP3K1	MAP3K1;MAPKKK1;MEKK;MEKK1;SRXY6	mitogen-activated protein kinase kinase kinase 1	0.45	0.58	1.31	0.52
PARK7	PARK7;DJ-1;DJ1;FLJ27376;FLJ34360;FLJ92274	parkinson protein 7	0.42	0.52	1.31	0.45
COPB2	COPB2;beta'-COP	coatomer protein complex, subunit beta 2 (beta prime)	0.42	0.57	1.31	0.44
BATF3	BATF3;FLJ36352;FLJ37535;JDP1;JUNDM1;SNFT	basic leucine zipper transcription factor, ATF-like 3	0.38	0.52	1.31	0.4
EXOSC9	EXOSC9;PM/Scl-75;PMSCL1;RRP45;Rrp45p;p5;p6	exosome component 9	0.38	0.47	1.31	0.41
RPS11	RPS11;S11	ribosomal protein S11	0.38	0.48	1.31	0.36
ZNF775	ZNF775;MGC33584	zinc finger protein 775	0.37	0.46	1.31	0.37
POLR2K	POLR2K;ABC10-alpha;RPABC4;RPB10alpha;RPB12;RPB7.0;hRPB7.0;hsRPB10a	polymerase (RNA) II (DNA directed) polypeptide K, 7.0kDa	0.34	0.43	1.31	0.33
EPM2AIP1	EPM2AIP1;FLJ11207;KIAA0766	EPM2A (laforin) interacting protein 1	0.32	0.4	1.31	0.29
BCAP29	BCAP29;BAP29;DKFZp686M2086;FLJ53907	B-cell receptor-associated protein 29	0.3	0.38	1.31	0.3
SUV39H1	SUV39H1;KMT1A;MG44;SUV39H	suppressor of variegation 3-9 homolog 1 (Drosophila)	0.3	0.42	1.31	0.28
RPL14	RPL14;CAG-ISL-7;CTG-B33;L14;MGC88594;RL14;hRL14	ribosomal protein L14	0.25	0.34	1.31	0.24
AQR	AQR;DKFZp686B23123;IBP160;KIAA0560;fSAP164	aquarius homolog (mouse)	0.15	0.21	1.31	0.15
PHLDA2	PHLDA2;BRW1C;BWR1C;HLDA2;IPL;TSSC3	pleckstrin homology-like domain, family A, member 2	0.1	0.12	1.31	0.09
AOC2	AOC2;DAO2;RAO	amine oxidase, copper containing 2 (retina-specific)	0.6	0.79	1.32	0.53
COL4A3	COL4A3	collagen, type IV, alpha 3 (Goodpasture antigen)	0.6	0.83	1.32	0.62
SEL1L	SEL1L;PRO1063;SEL1-LIKE;SEL1L1	sel-1 suppressor of lin-12-like (C. elegans)	0.6	0.78	1.32	0.55
SLC2A7	SLC2A7;GLUT7	solute carrier family 2 (facilitated glucose transporter), member 7	0.59	0.8	1.32	0.61
PDE4DIP	PDE4DIP;CMYA2;DKFZp781J054;MGC75440;MMGL	phosphodiesterase 4D interacting protein	0.59	0.77	1.32	0.58
C1orf61	C1orf61;CROC4;FLJ38303	chromosome 1 open reading frame 61	0.58	0.75	1.32	0.56
DYNC2LI1	DYNC2LI1;D2LIC;DKFZp564A033;LIC3	dynein, cytoplasmic 2, light intermediate chain 1	0.58	0.81	1.32	0.57
PROSC	PROSC;FLJ11861	proline synthetase co-transcribed homolog (bacterial)	0.58	0.77	1.32	0.6
OR2Z1	OR2Z1;OR19-4;OR2Z2	olfactory receptor, family 2, subfamily Z, member 1	0.57	0.78	1.32	0.59
ETF1	ETF1;D5S1995;ERF;ERF1;MGC111066;RF1;SUP45L1;TB3-1	eukaryotic translation termination factor 1	0.57	0.75	1.32	0.59
DALRD3	DALRD3;FLJ10496	DALR anticodon binding domain containing 3	0.56	0.72	1.32	0.56
BTN2A1	BTN2A1;BK14H9.1;BT2.1;BTF1;DJ3E1.1;FLJ36567	butyrophilin, subfamily 2, member A1	0.55	0.75	1.32	0.51
AIP	AIP;ARA9;FKBP16;FKBP37;SMTPHN;XAP-2;XAP2	aryl hydrocarbon receptor interacting protein	0.55	0.77	1.32	0.51
NPTX1	NPTX1;DKFZp686J2446;MGC105123;NP1	neuronal pentraxin I	0.54	0.7	1.32	0.58
FAM54B	FAM54B;MST116;MSTP116	family with sequence similarity 54, member B	0.53	0.76	1.32	0.51
HIST1H2AB	HIST1H2AB;H2A/m;H2AFM	histone cluster 1, H2ab	0.53	0.68	1.32	0.52
ACBD3	ACBD3;GCP60;GOCAP1;GOLPH1;PAP7	acyl-CoA binding domain containing 3	0.53	0.73	1.32	0.55
GNGT2	GNGT2;G-GAMMA-8;G-GAMMA-C;GNG8;GNG9;GNGT8	guanine nucleotide binding protein (G protein), gamma transducing activity polypeptide 2	0.52	0.68	1.32	0.51
ZNF512B	ZNF512B;GM632;KIAA1196;MGC149845;MGC149846	zinc finger protein 512B	0.52	0.7	1.32	0.52
AGPAT2	AGPAT2;1-AGPAT2;BSCL;BSCL1;LPAAB;LPAAT-beta	1-acylglycerol-3-phosphate O-acyltransferase 2 (lysophosphatidic acid acyltransferase, beta)	0.52	0.7	1.32	0.46
VPS45	VPS45;H1;H1VPS45;VPS45A;VPS45B;VPS54A;VSP45;VSP45A	vacuolar protein sorting 45 homolog (S. cerevisiae)	0.52	0.67	1.32	0.5
HTRA3	HTRA3;Prsp;Tasp	HtrA serine peptidase 3	0.51	0.62	1.32	0.47
H2AFX	H2AFX;H2A.X;H2A/X;H2AX	H2A histone family, member X	0.51	0.62	1.32	0.5
LTB4R	LTB4R;BLT1;BLTR;CMKRL1;GPR16;LTB4R1;LTBR1;P2RY7;P2Y7	leukotriene B4 receptor	0.48	0.58	1.32	0.44
HTT	HTT;HD;IT15	huntingtin	0.48	0.62	1.32	0.46
TTK	TTK;CT96;ESK;FLJ38280;MPS1;MPS1L1;PYT	TTK protein kinase	0.48	0.63	1.32	0.52
BUB3	BUB3;BUB3L;hBUB3	budding uninhibited by benzimidazoles 3 homolog (yeast)	0.47	0.57	1.32	0.51
ZNF250	ZNF250;FLJ57354;MGC111123;MGC9718;ZFP647;ZNF647	zinc finger protein 250	0.46	0.66	1.32	0.46
UBXN2A	UBXN2A;MGC138202;UBXD4	UBX domain protein 2A	0.46	0.63	1.32	0.47
NDST1	NDST1;HSST;NST1	N-deacetylase/N-sulfotransferase (heparan glucosaminyl) 1	0.46	0.62	1.32	0.41
SSBP2	SSBP2;DKFZp686F03273;HSPC116;SOSS-B2	single-stranded DNA binding protein 2	0.45	0.66	1.32	0.49
THAP8	THAP8;FLJ32891	THAP domain containing 8	0.44	0.59	1.32	0.41
RPL31	RPL31;L31;MGC88191	ribosomal protein L31	0.44	0.58	1.32	0.45
SF3A3	SF3A3;PRP9;PRPF9;SAP61;SF3a60	splicing factor 3a, subunit 3, 60kDa	0.43	0.55	1.32	0.4

OR10J3	OR10J3;OR1-25;OR10J3P	olfactory receptor, family 10, subfamily J, member 3	0.42	0.56	1.32	0.41
ONECUT2	ONECUT2;MGC120377;MGC120378;OC-2;OC2	one cut homeobox 2	0.41	0.53	1.32	0.37
ZFPL1	ZFPL1;D11S750;MCG4	zinc finger protein-like 1	0.4	0.54	1.32	0.41
WNT7B	WNT7B	wingless-type MMTV integration site family, member 7B	0.38	0.48	1.32	0.35
SLC22A18	SLC22A18;BWR1A;BWSCR1A;DKFZp667A184;HET;IMPT1;ITM;ORCTL2;SLC22A1L;TSSC5;p45-BWR1A	solute carrier family 22, member 18	0.37	0.47	1.32	0.41
PXMP2	PXMP2;FLJ54922;PMP22	peroxisomal membrane protein 2, 22kDa	0.35	0.45	1.32	0.36
COMMD8	COMMD8;FLJ20502	COMM domain containing 8	0.34	0.46	1.32	0.33
AHSP	AHSP;EDRF;ERAF	alpha hemoglobin stabilizing protein	0.3	0.39	1.32	0.28
PRAP1	PRAP1;MGC126792;PRO1195;RP11-122K13.6;UPA	proline-rich acidic protein 1	0.25	0.32	1.32	0.24
FCRL3	FCRL3;CD307c;FCRH3;IFGP3;IRTA3;SPAP2	Fc receptor-like 3	0.6	0.71	1.33	0.56
GOLT1B	GOLT1B;GOT1;YMR292W	golgi transport 1B	0.6	0.78	1.33	0.56
LTV1	LTV1;C6orf93;FLJ14909;FLJ18519;dJ468K18.4	LTV1 homolog (S. cerevisiae)	0.59	0.74	1.33	0.6
C1orf112	C1orf112;FLJ10706;FLJ13470;MGC130018;MGC130019;RP1-97P20.1	chromosome 1 open reading frame 112	0.58	0.82	1.33	0.58
FBXL15	FBXL15;FBXO37;FLJ16137;Fbl15;JET;MGC11279;PSD	F-box and leucine-rich repeat protein 15	0.57	0.73	1.33	0.52
GP9	GP9;CD42a;GPIX	glycoprotein IX (platelet)	0.57	0.72	1.33	0.61
PLEK2	PLEK2	pleckstrin 2	0.57	0.7	1.33	0.55
MORF4L1	MORF4L1;Eaf3;HsT17725;MEAF3;MGC10631;MORFRG15;MRG15;S863-6	mortality factor 4 like 1	0.57	0.78	1.33	0.53
LMAN2	LMAN2;C5orf8;GP36B;VIP36	lectin, mannose-binding 2	0.57	0.76	1.33	0.52
39873	MARCH9;FLJ36578;MARCH-IX;RNF179	membrane-associated ring finger (C3HC4) 9	0.56	0.74	1.33	0.66
RDX	RDX;DFNB24	radixin	0.56	0.68	1.33	0.54
CTNNB1	CTNNB1;CTNNB;DKFZp686D02253;FLJ25606;FLJ37923	catenin (cadherin-associated protein), beta 1, 88kDa	0.56	0.75	1.33	0.58
CTIF	CTIF;Gm672;KIAA0427	CBP80/20-dependent translation initiation factor	0.56	0.72	1.33	0.55
LINC00477	LINC00477;C12orf67;FAM191B;FLJ32894;MGC129798;MGC129799	long intergenic non-protein coding RNA 477	0.56	0.68	1.33	0.54
MYH2	MYH2;MYH2A;MYHSA2;MYHas8;MyHC-2A;MyHC-IIa	myosin, heavy chain 2, skeletal muscle, adult	0.55	0.73	1.33	0.54
ATP12A	ATP12A;ATP1AL1	ATPase, H+/K+ transporting, nongastric, alpha polypeptide	0.55	0.7	1.33	0.52
GRRP1	GRRP1;FAM110D;FLJ14050;RP11-96L14.5	glycine/arginine rich protein 1	0.55	0.71	1.33	0.54
MRPS30	MRPS30;DKFZp566B2024;MRP-S30;PAP;PDCD9;S30mt	mitochondrial ribosomal protein S30	0.55	0.72	1.33	0.56
KANK4	KANK4;ANKRD38;FLJ10884;KIAA0172;RP5-1155K23.5;dJ1078M7.1	KN motif and ankyrin repeat domains 4	0.53	0.7	1.33	0.51
PDZD9	PDZD9;C16orf65;MGC50721	PDZ domain containing 9	0.53	0.76	1.33	0.51
CCDC142	CCDC142;FLJ14397;FLJ90728	coiled-coil domain containing 142	0.52	0.69	1.33	0.48
POU3F4	POU3F4;BRAIN-4;BRN4;DFN3;DFNX2;OTF9	POU class 3 homeobox 4	0.5	0.64	1.33	0.55
SNX27	SNX27;KIAA0488;MGC126871;MGC126873;MGC20471;MRT1;MY014	sorting nexin family member 27	0.5	0.69	1.33	0.47
CDC42BPA	CDC42BPA;DKFZp686L1738;DKFZp686P1738;FLJ23347;KIAA0451;MRCK;MRCKA;PK428	CDC42 binding protein kinase alpha (DMPK-like)	0.49	0.65	1.33	0.54
SLC6A5	SLC6A5;GLYT-2;GLYT2;NET1	solute carrier family 6 (neurotransmitter transporter, glycine), member 5	0.48	0.62	1.33	0.54
DNAJC6	DNAJC6;DJC6;KIAA0473;MGC129914;MGC129915;MGC48436	DnaJ (Hsp40) homolog, subfamily C, member 6	0.48	0.68	1.33	0.45
SH3BGRL3	SH3BGRL3;SH3BP-1;TIP-B1	SH3 domain binding glutamic acid-rich protein like 3	0.47	0.61	1.33	0.46
RCVRN	RCVRN;RCV1	recoverin	0.45	0.61	1.33	0.45
TCERG1L	TCERG1L;MGC126584	transcription elongation regulator 1-like	0.45	0.57	1.33	0.44
MCM3AP	MCM3AP;FLJ44336;FLJ45306;GANP;KIAA0572;MAP80	minichromosome maintenance complex component 3 associated protein	0.45	0.62	1.33	0.47
SLC6A18	SLC6A18;FLJ31236;Xtrp2	solute carrier family 6, member 18	0.43	0.57	1.33	0.39
FAM161B	FAM161B;C14orf44;FLJ31697;c14_5547	family with sequence similarity 161, member B	0.43	0.55	1.33	0.42
C7orf23	C7orf23;MGC4175;MM-TRAG	chromosome 7 open reading frame 23	0.43	0.57	1.33	0.41
CORO1C	CORO1C;HCRNN4	coronin, actin binding protein, 1C	0.42	0.55	1.33	0.38
SLC48A1	SLC48A1;FLJ20489;HRG-1;HRG1;hHRG-1	solute carrier family 48 (heme transporter), member 1	0.42	0.54	1.33	0.45
PGBD1	PGBD1;HUCEP-4;SCAND4;dJ874C20.4	piggyBac transposable element derived 1	0.4	0.54	1.33	0.41
HRH2	HRH2;H2R	histamine receptor H2	0.38	0.5	1.33	0.45
CCNE1	CCNE1;CCNE	cyclin E1	0.38	0.49	1.33	0.34
CELF5	CELF5;BRUNOL-5;BRUNOL5	CUGBP, Elav-like family member 5	0.38	0.53	1.33	0.43
CDC45	CDC45;CDC45L;CDC45L2;PORC-PI-1	cell division cycle 45 homolog (S. cerevisiae)	0.38	0.48	1.33	0.35
POP1	POP1;KIAA0061;MGC17365	processing of precursor 1, ribonuclease P/MRP subunit (S. cerevisiae)	0.34	0.45	1.33	0.33
OMP	OMP	olfactory marker protein	0.32	0.38	1.33	0.3
KIF11	KIF11;EG5;HKSP;KNSL1;TRIP5	kinesin family member 11	0.32	0.41	1.33	0.29
PIK3R6	PIK3R6;C17orf38;DKFZp666P158;FLJ34500;HsT41028;p84;p87(PIKAP);p87PIKAP	phosphoinositide-3-kinase, regulatory subunit 6	0.31	0.42	1.33	0.32
RPS16	RPS16;S16	ribosomal protein S16	0.31	0.42	1.33	0.28
PIGC	PIGC;GPI2;MGC2049	phosphatidylinositol glycan anchor biosynthesis, class C	0.6	0.8	1.34	0.62
SNAPIN	SNAPIN;SNAPAP	SNAP-associated protein	0.6	0.84	1.34	0.63
ARHGAP5	ARHGAP5;GFI2;RhoGAP5;p190-B;p190BRhoGAP	Rho GTPase activating protein 5	0.59	0.76	1.34	0.55
BLID	BLID;BRCC2;MGC163233;MGC163235	BH3-like motif containing, cell death inducer	0.58	0.74	1.34	0.54
GNPTG	GNPTG;C16orf27;GNPTAG;LP2537;RJD9	N-acetylglucosamine-1-phosphate transferase, gamma subunit	0.58	0.8	1.34	0.6
SNRNP25	SNRNP25;C16orf33;FLJ22940	small nuclear ribonucleoprotein 25kDa (U11/U12)	0.58	0.78	1.34	0.53
GORAB	GORAB;FLJ11752;GO;MGC51263;MGC70512;NTKLBP1;SCYL1BP1	golgin, RAB6-interacting	0.58	0.76	1.34	0.56
BBS10	BBS10;C12orf58;FLJ23560	Bardet-Biedl syndrome 10	0.57	0.74	1.34	0.56
LRRC59	LRRC59;FLJ21675	leucine rich repeat containing 59	0.56	0.66	1.34	0.52
SLC5A5	SLC5A5;NIS;TDH1	solute carrier family 5 (sodium iodide symporter), member 5	0.55	0.68	1.34	0.54
RPP38	RPP38	ribonuclease P/MRP 38kDa subunit	0.55	0.74	1.34	0.52
SPINT1	SPINT1;HAI;HAI1;MANSC2	serine peptidase inhibitor, Kunitz type 1	0.54	0.73	1.34	0.49
MMP15	MMP15;MT2-MMP;MTMMP2;SMCP-2	matrix metallopeptidase 15 (membrane-inserted)	0.52	0.64	1.34	0.48
TCTN3	TCTN3;C10orf61;DKFZp564D116;TECT3	tectonic family member 3	0.52	0.68	1.34	0.49
CCT5	CCT5;CCT-epsilon;CCTE;KIAA0098;TCP-1-epsilon	chaperonin containing TCP1, subunit 5 (epsilon)	0.52	0.76	1.34	0.52
NDP	NDP;EVR2;FEVR;ND	Norrie disease (pseudoglioma)	0.51	0.64	1.34	0.56
INSM1	INSM1;IA-1;IA1	insulinoma-associated 1	0.51	0.67	1.34	0.47
MTUS2	MTUS2;CAZIP;KIAA0774;TIP150	microtubule associated tumor suppressor candidate 2	0.5	0.69	1.34	0.46
UBE2T	UBE2T;PIG50	ubiquitin-conjugating enzyme E2T (putative)	0.49	0.61	1.34	0.46
CYB5R3	CYB5R3;B5R;DIA1	cytochrome b5 reductase 3	0.49	0.64	1.34	0.47
DKKL1	DKKL1;CT34;SGY;SGY-1;SGY1	dickkopf-like 1	0.48	0.57	1.34	0.45
C14orf178	C14orf178;FLJ25976;MGC129942	chromosome 14 open reading frame 178	0.48	0.63	1.34	0.47
ARHGAP27	ARHGAP27;CAMGAP1;FLJ35572;FLJ43547;FLJ76025;MGC120624;MGC78393;PP905;SH3D20;SH3P20	Rho GTPase activating protein 27	0.48	0.68	1.34	0.48
UBE2D3	UBE2D3;E2(17)KB3;MGC43926;MGC5416;UBC4/5;UBCH5C	ubiquitin-conjugating enzyme E2D 3	0.48	0.59	1.34	0.44
CPN2	CPN2;ACBP	carboxypeptidase N, polypeptide 2	0.46	0.63	1.34	0.49
GFER	GFER;ALR;ERV1;HERV1;HPO;HPO1;HPO2;HSS	growth factor, augmenter of liver regeneration	0.45	0.56	1.34	0.45
HRNR	HRNR;S100A16;S100a18	hornerin	0.44	0.6	1.34	0.45
NR5A2	NR5A2;B1F;B1F2;CPF;FTF;FTZ-F1;FTZ-F1beta;LRH-1;LRH1;hB1F-2	nuclear receptor subfamily 5, group A, member 2	0.44	0.55	1.34	0.48
GPR142	GPR142;PGR2	G protein-coupled receptor 142	0.44	0.54	1.34	0.41
CHST3	CHST3;C6ST;C6ST1;HSD	carbohydrate (chondroitin 6) sulfotransferase 3	0.44	0.58	1.34	0.5
RPSA	RPSA;37LRP;67LR;LAMBR;LAMR1;LBP;LBP/p40;LRP;LRP/LR;NEM/1CHD4;SA;lamR;p40	ribosomal protein SA	0.44	0.62	1.34	0.46
RBM42	RBM42;MGC10433	RNA binding motif protein 42	0.43	0.52	1.34	0.4
ZNF48	ZNF48;DKFZp762K013;FLJ31751;MGC43952;ZNF553	zinc finger protein 48	0.4	0.54	1.34	0.37
SFPQ	SFPQ;POMP100;PSF	splicing factor proline/glutamine-rich	0.39	0.54	1.34	0.41
EIF3A	EIF3A;EIF3;EIF3S10;KIAA0139;P167;TIF32;eIF3-p170;eIF3-theta;p180;p185	eukaryotic translation initiation factor 3, subunit A	0.39	0.48	1.34	0.43
ANKZF1	ANKZF1;FLJ10415;FLJ13144;ZNF744	ankyrin repeat and zinc finger domain containing 1	0.38	0.49	1.34	0.37
VPS4A	VPS4A;FLJ22197;SKD1;SKD1A;SKD2;VPS4;VPS4-1	vacuolar protein sorting 4 homolog A (S. cerevisiae)	0.38	0.5	1.34	0.38
BCL11A	BCL11A;BCL11A-L;BCL11A-S;BCL11A-XL;CTIP1;EVI9;FLJ10173;FLJ34997;HBFQTL5;KIAA1809;ZNF856	B-cell CLL/lymphoma 11A (zinc finger protein)	0.37	0.52	1.34	0.35
ANKS1A	ANKS1A;ANKS1;KIAA0229;MGC42354	ankyrin repeat and sterile alpha motif domain containing 1A	0.37	0.47	1.34	0.38
FERMT2	FERMT2;DKFZp686G11125;FLJ34213;FLJ44462;KIND2;MIG2;PLEKHC1;UNC112;UNC112B;mig-2	fermitin family member 2	0.37	0.48	1.34	0.35
LINC00174	LINC00174;NCRNA00174	long intergenic non-protein coding RNA 174	0.34	0.44	1.34	0.34
TBC1D2	TBC1D2;DKFZp761D1823;FLJ10702;FLJ16244;FLJ42782;PARIS-1;PARIS1;TBC1D2A	TBC1 domain family, member 2	0.33	0.46	1.34	0.35
CNOT2	CNOT2;CDC36;FLJ26456;NOT2;NOT2H	CCR4-NOT transcription complex, subunit 2	0.33	0.44	1.34	0.3
DVL2	DVL2	dishevelled, dsh homolog 2 (Drosophila)	0.32	0.38	1.34	0.32
SNRPE	SNRPE;B-raf;SME;Sm-E	small nuclear ribonucleoprotein polypeptide E	0.32	0.44	1.34	0.33
CASP8AP2	CASP8AP2;CED-4;FLASH;FLJ11208;KIAA1315;RIP25	caspase 8 associated protein 2	0.23	0.29	1.34	0.23
PSMD12	PSMD12;MGC75406;Rpn5;p55	proteasome (prosome, macropain) 26S subunit, non-ATPase, 12	0.22	0.29	1.34	0.2
PCIF1	PCIF1;C20orf67	PDX1 C-terminal inhibiting factor 1	0.6	0.78	1.35	0.59
YARS2	YARS2;FLJ13995;MLASA2;MT-TYRRS;TYRRS	tyrosyl-tRNA synthetase 2, mitochondrial	0.59	0.71	1.35	0.56
C9orf80	C9orf80;HSPC043;MISE;SOSSC;SSBIP1;hSSBIP1	chromosome 9 open reading frame 80	0.59	0.82	1.35	0.62
NPPA	NPPA;ANF;ANP;ATFB6;CDD-ANF;PND	natriuretic peptide A	0.58	0.81	1.35	0.61
COL11A2	COL11A2;DFNA13;DFNB53;HKE5;PARP;STL3	collagen, type XI, alpha 2	0.58	0.82	1.35	0.61
EPHA7	EPHA7;EHK3;HEK11	EPH receptor A7	0.56	0.74	1.35	0.62
FUS	FUS;ALS6;FUS1;HNRNPP2;POMP75;TLS	fused in sarcoma	0.56	0.71	1.35	0.56
UGDH	UGDH;GDH;UDP-GlcDH;UDPGDH;UGD	UDP-glucose 6-dehydrogenase	0.55	0.69	1.35	0.55
DHFR	DHFR;DHFRP1;DYR	dihydrofolate reductase	0.55	0.73	1.35	0.51
IFNA7	IFNA7;IFN-alphaJ;IFNA-J	interferon, alpha 7	0.55	0.77	1.35	0.56
LILRA2	LILRA2;CD85H;ILT1;LIR-7;LIR7	leukocyte immunoglobulin-like receptor, subfamily A (with TM domain), member 2	0.54	0.75	1.35	0.5
BHLHE40	BHLHE40;BHLHB2;DEC1;FLJ99214;HLHB2;SHARP-2;STRA13;Stra14	basic helix-loop-helix family, member e40	0.54	0.74	1.35	0.49
CCDC75	CCDC75;CENP-Y;CENPY;FLJ38348;GPATCH11	coiled-coil domain containing 75	0.53	0.76	1.35	0.54
AIFM3	AIFM3;AIFL;FLJ30473;FLJ45137	apoptosis-inducing factor, mitochondrion-associated, 3	0.52	0.66	1.35	0.48
B4GALNT2	B4GALNT2;B4GALT;GALGT2;MGC142235;MGC142237	beta-1,4-N-acetyl-galactosaminyl transferase 2	0.52	0.74	1.35	0.49
GAL3ST4	GAL3ST4;FLJ12116;GAL3ST-4	galactose-3-O-sulfotransferase 4	0.52	0.71	1.35	0.56
ZNF426	ZNF426;MGC2663	zinc finger protein 426	0.52	0.67	1.35	0.51
RAB17	RAB17;FLJ12538	RAB17, member RAS oncogene family	0.52	0.68	1.35	0.48
TRUB2	TRUB2;CLONE24922;RP11-339B21.1	TruB pseudouridine (psi) synthase homolog 2 (E. coli)	0.52	0.75	1.35	0.5
CNPY4	CNPY4;MGC40499;PRAT4B	canopy 4 homolog (zebrafish)	0.48	0.68	1.35	0.46
H1FX	H1FX;H1X;MGC15959;MGC8350	H1 histone family, member X	0.48	0.63	1.35	0.47
PLK4	PLK4;SAK;STK18	polo-like kinase 4	0.44	0.59	1.35	0.49
LRRC61	LRRC61;FLJ22216;FLJ31392;HSPC295;MGC3036	leucine rich repeat containing 61	0.41	0.54	1.35	0.41
JAG2	JAG2;HJ2;SER2	jagged 2	0.38	0.48	1.35	0.38
RNPS1	RNPS1;E5.1;MGC117332	RNA binding protein S1, serine-rich domain	0.38	0.48	1.35	0.38
TSSK3	TSSK3;SPOGA3;STK22C;STK22D;TSK3	testis-specific serine kinase 3	0.37	0.5	1.35	0.41
CDY1B	CDY1B;CDY;CDY1	chromodomain protein, Y-linked, 1B	0.37	0.51	1.35	0.34
SPTBN1	SPTBN1;ELF;SPTB2;betaSpII	spectrin, beta, non-erythrocytic 1	0.36	0.46	1.35	0.33

TF	TF;DKFZp781D0156;PRO1557;PRO2086;TFQTL1	transferrin	0.34	0.44	1.35	0.34
FANCM	FANCM;FAAP250;KIAA1596;MGC176453	Fanconi anemia, complementation group M	0.34	0.43	1.35	0.34
CDR2	CDR2;CDR62;Yo	cerebellar degeneration-related protein 2, 62kDa	0.34	0.48	1.35	0.36
KIR2DL5A	KIR2DL5A;CD158F;KIR2DL5;KIR2DL5.1;KIR2DL5.3;KIR2DL5B	killer cell immunoglobulin-like receptor, two domains, long cytoplasmic tail, 5A	0.28	0.37	1.35	0.28
DONSON	DONSON;B17;C21orf60;C2TA;DKFZp434M035	downstream neighbor of SON	0.25	0.36	1.35	0.24
KIF12	KIF12;RP11-56P10.3	kinesin family member 12	0.22	0.3	1.35	0.23
OR2F1	OR2F1;OLF3;OR14-60;OR2F3;OR2F3P;OR2F4;OR2F5;OR7-139;OR7-140	olfactory receptor, family 2, subfamily F, member 1	0.16	0.2	1.35	0.15
UBL5	UBL5;FLJ46917;HUB1;MGC131795	ubiquitin-like 5	0.14	0.2	1.35	0.15
STIM2	STIM2;FLJ39527;KIAA1482	stromal interaction molecule 2	0.1	0.14	1.35	0.09
ANO8	ANO8;KIAA1623;TMEM16H	anoctamin 8	0.6	0.81	1.36	0.58
ZNF281	ZNF281;FLJ12859;FLJ14378;ZBP-99;ZNP-99	zinc finger protein 281	0.6	0.8	1.36	0.69
PCDHGA8	PCDHGA8;KIAA0327;PCDH-GAMMA-A8	protocadherin gamma subfamily A, 8	0.6	0.78	1.36	0.6
SLC36A1	SLC36A1;Dct1;LYAAT1;PAT1;TRAMD3	solute carrier family 36 (proton/amino acid symporter), member 1	0.59	0.81	1.36	0.62
TRO	TRO;KIAA1114;MAGE-d3;MAGED3	trophinin	0.57	0.75	1.36	0.54
IGHMBP2	IGHMBP2;CATF1;FLJ34220;FLJ41171;HCSA;HMN6;SMARD1;SMUBP2	immunoglobulin mu binding protein 2	0.57	0.73	1.36	0.58
C1orf186	C1orf186;FLJ16052	chromosome 1 open reading frame 186	0.56	0.74	1.36	0.56
RBBP6	RBBP6;DKFZp686P0638;DKFZp761B2423;MY038;P2P-R;PACT;RBQ-1;SNAMA	retinoblastoma binding protein 6	0.56	0.75	1.36	0.67
KLF8	KLF8;BKLF3;DKFZp686O08126;MGC138314;ZNF741	Kruppel-like factor 8	0.54	0.76	1.36	0.58
RPL37	RPL37;DKFZp686G1699;L37;MGC99572	ribosomal protein L37	0.54	0.75	1.36	0.57
SNRPA	SNRPA;Mud1;U1-A;U1A	small nuclear ribonucleoprotein polypeptide A	0.54	0.76	1.36	0.52
HIST1H2AM	HIST1H2AM;H2A.1;H2A/n;H2AFN;HIST1H2AG;HIST1H2AI;HIST1H2AK;HIST1H2AL;dJ193B12.1	histone cluster 1, H2am	0.53	0.76	1.36	0.62
PPAPDC1B	PPAPDC1B;DPPL1;HTPAP	phosphatidic acid phosphatase type 2 domain containing 1B	0.53	0.71	1.36	0.5
AKIRIN2	AKIRIN2;C6orf166;FBI1;FLJ10342;dJ486L4.2	akirin 2	0.53	0.7	1.36	0.53
SET	SET;2PP2A;I2PP2A;IGAAD;IPP2A2;PHAPII;TAF-I;TAF-IBETA	SET nuclear oncogene	0.53	0.67	1.36	0.51
MFSD4	MFSD4;DKFZp564C1416;DKFZp761N1114;FLJ25004;FLJ34577;FLJ45392;UNQ3064	major facilitator superfamily domain containing 4	0.52	0.62	1.36	0.49
AIMP1	AIMP1;EMAP2;EMAPII;HLD3;SCYE1;p43	aminoacyl tRNA synthetase complex-interacting multifunctional protein 1	0.5	0.66	1.36	0.51
SIAE	SIAE;AIS6;CSE-C;CSEC;LSE;MGC87009;YSG2	sialic acid acetylesterase	0.49	0.63	1.36	0.51
ABCG5	ABCG5;STSL	ATP-binding cassette, sub-family G (WHITE), member 5	0.48	0.62	1.36	0.48
ABHD10	ABHD10;FLJ11342	abhydrolase domain containing 10	0.48	0.68	1.36	0.53
ZCCHC3	ZCCHC3;C20orf99;MGC104290	zinc finger, CCHC domain containing 3	0.47	0.62	1.36	0.47
GFRA3	GFRA3	GDNF family receptor alpha 3	0.46	0.57	1.36	0.51
NSMCE4A	NSMCE4A;C10orf86;FLJ20003;NSE4A	non-SMC element 4 homolog A (S. cerevisiae)	0.46	0.56	1.36	0.44
TOM1	TOM1;FLJ33404	target of myb1 (chicken)	0.45	0.6	1.36	0.47
RLF	RLF;MGC142226;ZN-15L;ZNF292L	rearranged L-myc fusion	0.45	0.67	1.36	0.46
TPO	TPO;MSA;TDH2A;TPX	thyroid peroxidase	0.44	0.59	1.36	0.44
FZD9	FZD9;CD349;FZD3	frizzled family receptor 9	0.41	0.54	1.36	0.49
GATAD2B	GATAD2B;FLJ37346;KIAA1150;MGC138257;MGC138285;P66beta;RP11-216N14.6	GATA zinc finger domain containing 2B	0.4	0.55	1.36	0.43
NUTF2	NUTF2;NTF2;PP15	nuclear transport factor 2	0.4	0.52	1.36	0.38
MAGIX	MAGIX;FLJ21687;JM10;MGC138889;PDZX	MAGI family member, X-linked	0.39	0.5	1.36	0.41
ALMS1	ALMS1;ALSS;DKFZp686A118;DKFZp686D1828;KIAA0328	Alstrom syndrome 1	0.38	0.53	1.36	0.36
MYO1B	MYO1B;myr1	myosin IB	0.38	0.52	1.36	0.4
HSPA13	HSPA13;STCH	heat shock protein 70kDa family, member 13	0.38	0.56	1.36	0.39
C11orf68	C11orf68;BLES03;P5326	chromosome 11 open reading frame 68	0.37	0.54	1.36	0.42
CYR61	CYR61;CCN1;GIG1;IGFBP10	cysteine-rich, angiogenic inducer, 61	0.37	0.53	1.36	0.39
CLN5	CLN5;FLJ90628;NCL	ceroid-lipofuscinosis, neuronal 5	0.3	0.42	1.36	0.29
DDX19A	DDX19A;DDX19-DDX19L;DDX19L;DKFZp686C21137;FLJ11126	DEAD (Asp-Glu-Ala-As) box polypeptide 19A	0.3	0.4	1.36	0.27
CKAP5	CKAP5;CHTOG;FLJ35359;KIAA0097;MSPS;TOG;TOGp;ch-TOG	cytoskeleton associated protein 5	0.26	0.34	1.36	0.27
ALKBH7	ALKBH7;ABH7;MGC10974;SPATA11;UNQ6002	alkB, alkylation repair homolog 7 (E. coli)	0.22	0.31	1.36	0.24
PCP2	PCP2;DKFZp686M0187;FLJ27345;GPSM4;L7;MGC41903	Purkinje cell protein 2	0.6	0.74	1.37	0.58
NAA25	NAA25;C12orf30;DKFZp667K2112;FLJ13089;MDM20;NAP1	N(alpha)-acetyltransferase 25, NatB auxiliary subunit	0.6	0.78	1.37	0.63
EXOC3L4	EXOC3L4;C14orf73	exocyst complex component 3-like 4	0.6	0.82	1.37	0.57
FHL2	FHL2;AAG11;DRAL;FHL-2;SLIM-3;SLIM3	four and a half LIM domains 2	0.59	0.78	1.37	0.6
SUMO4	SUMO4;IDDM5;SMT3H4;SUMO-4;dJ281H8.4	SMT3 suppressor of mif two 3 homolog 4 (S. cerevisiae)	0.58	0.78	1.37	0.59
MPHOSPH8	MPHOSPH8;FLJ35237;HSMPP8;TWA3;mpp8	M-phase phosphoprotein 8	0.58	0.82	1.37	0.59
MFSD11	MFSD11;ET;FLJ20226;FLJ22196	major facilitator superfamily domain containing 11	0.57	0.76	1.37	0.58
CCDC89	CCDC89;FLJ38159	coiled-coil domain containing 89	0.56	0.73	1.37	0.59
FAM168A	FAM168A;KIAA0280;TCRP1	family with sequence similarity 168, member A	0.55	0.68	1.37	0.54
HSH2D	HSH2D;ALX;FLJ14886;HSH2	hematopoietic SH2 domain containing	0.54	0.72	1.37	0.54
SULT2B1	SULT2B1;HSST2	sulfotransferase family, cytosolic, 2B, member 1	0.54	0.72	1.37	0.52
HJURP	HJURP;DKFZp762E1312;FAKTS;URLC9;hFLEG1	Holliday junction recognition protein	0.54	0.77	1.37	0.52
ACPL2	ACPL2;FLJ23751	acid phosphatase-like 2	0.53	0.68	1.37	0.51
BDP1	BDP1;DKFZp686C01233;DKFZp686K0831;HSA238520;KIAA1241;KIAA1689;TAF3B1;TFC5;TFIIIB";TFIIIB150;TFIIIB90;TFNR	B double prime 1, subunit of RNA polymerase III transcription initiation factor IIIB	0.53	0.74	1.37	0.57
ST7	ST7;DKFZp762O2113;ETS7q;FAM4A;FAM4A1;HELG;RAY1;SEN4;TSG7	suppression of tumorigenicity 7	0.52	0.7	1.37	0.53
CDH24	CDH24;CDH11L;FLJ25193;MGC131880	cadherin 24, type 2	0.51	0.71	1.37	0.48
CA5B	CA5B;CA-VB;MGC39962	carbonic anhydrase VB, mitochondrial	0.5	0.68	1.37	0.51
NUF2	NUF2;CDCA1;CT106;NUF2R	NUF2, NDC80 kinetochore complex component, homolog (S. cerevisiae)	0.5	0.72	1.37	0.55
CLEC4A	CLEC4A;CLECSF6;DCIR;DDB27;LLIR	C-type lectin domain family 4, member A	0.48	0.68	1.37	0.51
NLE1	NLE1;FLJ10458;Nle	notchless homolog 1 (Drosophila)	0.48	0.63	1.37	0.49
DENND2D	DENND2D;FLJ22457;RP5-1180E21.2	DENN/MADD domain containing 2D	0.48	0.7	1.37	0.5
PDK2	PDK2;PDHK2	pyruvate dehydrogenase kinase, isozyme 2	0.48	0.66	1.37	0.54
VNN1	VNN1;HDLCQ8;MGC116930;MGC116931;MGC116932;MGC116933;Tiff66	vanin 1	0.46	0.58	1.37	0.47
NCOA4	NCOA4;ARA70;DKFZp762E1112;ELE1;PTC3;RFG	nuclear receptor coactivator 4	0.44	0.58	1.37	0.45
F8A3	F8A3	coagulation factor VIII-associated 3	0.43	0.61	1.37	0.41
FAM113A	FAM113A;C20orf81;DKFZp547L054;FLJ22376;bA12M19.1	family with sequence similarity 113, member A	0.4	0.52	1.37	0.42
RGP1	RGP1;KIAA0258	RGP1 retrograde golgi transport homolog (S. cerevisiae)	0.4	0.57	1.37	0.43
MGST2	MGST2;FLJ27438;GST2;MGC14097;MGST-II	microsomal glutathione S-transferase 2	0.4	0.55	1.37	0.37
TEP1	TEP1;TLP1;TP1;TROVE1;VAULT2;p240	telomerase-associated protein 1	0.38	0.52	1.37	0.36
SLC44A1	SLC44A1;CD92;CDW92;CHTL1;CTL1;RP11-287A8.1	solute carrier family 44, member 1	0.34	0.45	1.37	0.35
CDY2B	CDY2B;CDY;CDY2;CDY2A;MGC163298;MGC163300	chromodomain protein, Y-linked, 2B	0.32	0.42	1.37	0.33
HIST1H3C	HIST1H3C;H3.1;H3/c;H3FC	histone cluster 1, H3c	0.31	0.45	1.37	0.3
KRT14	KRT14;CK14;EBS3;EBS4;K14;NFJ	keratin 14	0.2	0.26	1.37	0.22
EED	EED;HEED;WAIT1	embryonic ectoderm development	0.6	0.82	1.38	0.6
MPDU1	MPDU1;CDGIF;FLJ14836;HBEBP2BPA;Lec35;My008;PP3958;PQLC5;SL15	mannose-P-dolichol utilization defect 1	0.59	0.8	1.38	0.64
FBP1	FBP1;FBP	fructose-1,6-bisphosphatase 1	0.58	0.76	1.38	0.57
C3orf14	C3orf14	chromosome 3 open reading frame 14	0.57	0.77	1.38	0.58
ASCC3	ASCC3;ASC1p200;DJ467N11.1;HELIC1;MGC26074;RNAH;dJ121G13.4;p200	activating signal cointegrator 1 complex subunit 3	0.57	0.76	1.38	0.59
HDGFRP3	HDGFRP3;HDGF2	hepatoma-derived growth factor, related protein 3	0.57	0.74	1.38	0.63
MAGEA5	MAGEA5;CT1.5;MAGE5;MAGEA4;MGC129526	melanoma antigen family A, 5	0.56	0.74	1.38	0.56
XIAP	XIAP;API3;BIRC4;FLJ26913;IAP-3;ILP1;MIHA;XLP2;hIAP-3;hIAP3	X-linked inhibitor of apoptosis	0.56	0.77	1.38	0.69
APOBEC3C	APOBEC3C;A3C;APOBEC1L;ARDC2;ARDC4;ARP5;FLJ37251;MGC19485;PBI;bK150C2.3	apolipoprotein B mRNA editing enzyme, catalytic polypeptide-like 3C	0.55	0.71	1.38	0.63
PIH1D1	PIH1D1;FLJ20643;NOP17	PIH1 domain containing 1	0.55	0.68	1.38	0.53
SASH3	SASH3;753P9;CXorf9;HACS2;SH3D6C;SLY	SAM and SH3 domain containing 3	0.54	0.66	1.38	0.53
MPV17	MPV17;MTDPS6;SYM1	MpV17 mitochondrial inner membrane protein	0.54	0.71	1.38	0.54
DNAH5	DNAH5;CILD3;DNAHC5;FLJ46759;HL1;KIAA1603;KTGNR;PCD	dynein, axonemal, heavy chain 5	0.53	0.7	1.38	0.61
RPL34	RPL34;L34;MGC111005	ribosomal protein L34	0.52	0.74	1.38	0.51
CCDC164	CCDC164;C2orf39;FLJ32660;MGC16372	coiled-coil domain containing 164	0.52	0.71	1.38	0.51
ADAMTS7	ADAMTS7;ADAM-TS 7;ADAM-TS7;ADAMTS-7;DKFZp434H204	ADAM metallopeptidase with thrombospondin type 1 motif, 7	0.51	0.69	1.38	0.52
TUBB6	TUBB6;HsT1601;MGC132410;MGC4083;TUBB-5	tubulin, beta 6 class V	0.51	0.65	1.38	0.58
FITM2	FITM2;C20orf142;Fit2;MGC30135;dJ881L22.2	fat storage-inducing transmembrane protein 2	0.51	0.7	1.38	0.58
ZNF383	ZNF383;FLJ35863	zinc finger protein 383	0.5	0.68	1.38	0.51
YDJC	YDJC;MGC133160	YdjC homolog (bacterial)	0.5	0.72	1.38	0.54
C9orf171	C9orf171;FLJ46082;MGC148067	chromosome 9 open reading frame 171	0.5	0.7	1.38	0.53
LRIF1	LRIF1;C1orf103;FLJ11269;RIF1;RP11-96K19.1	ligand dependent nuclear receptor interacting factor 1	0.5	0.66	1.38	0.53
GPR35	GPR35	G protein-coupled receptor 35	0.49	0.62	1.38	0.47
RIBC2	RIBC2;C22orf11	RIB43A domain with coiled-coils 2	0.49	0.64	1.38	0.52
CLDN7	CLDN7;CEPTRL2;CLDN-7;CPETRL2;Hs.84359;claudin-1	claudin 7	0.49	0.66	1.38	0.5
RFXAP	RFXAP	regulatory factor X-associated protein	0.48	0.64	1.38	0.5
ZNF552	ZNF552;FLJ21603	zinc finger protein 552	0.48	0.62	1.38	0.49
ADARB1	ADARB1;ADAR2;DRABA2;DRADA2;RED1	adenosine deaminase, RNA-specific, B1	0.45	0.58	1.38	0.52
HMGN2P46	HMGN2P46;C15orf21;D-PCa-2;FLJ39426	high mobility group nucleosomal binding domain 2 pseudogene 46	0.45	0.62	1.38	0.43
POMC	POMC;ACTH;CLIP;LPH;MSH;NPP;POC	proopiomelanocortin	0.44	0.62	1.38	0.47
ATR	ATR;FRP1;MEC1;SCKL;SCKL1	ataxia telangiectasia and Rad3 related	0.43	0.64	1.38	0.45
SVIL	SVIL;DKFZp686A17191	supervillin	0.43	0.62	1.38	0.5
OR4C46	OR4C46	olfactory receptor, family 4, subfamily C, member 46	0.42	0.55	1.38	0.44
NMNAT3	NMNAT3;PNAT-3;PNAT3	nicotinamide nucleotide adenylyltransferase 3	0.42	0.55	1.38	0.48
SCML2	SCML2	sex comb on midleg-like 2 (Drosophila)	0.41	0.53	1.38	0.42
RPL37A	RPL37A;FLJ13357;L37A;MGC74786	ribosomal protein L37a	0.38	0.52	1.38	0.37
KAT5	KAT5;ESA1;HTATIP;HTATIP1;PLIP;TIP;TIP60;cPLA2	K(lysine) acetyltransferase 5	0.36	0.46	1.38	0.41
RPS21	RPS21;S21	ribosomal protein S21	0.36	0.49	1.38	0.39
SNRPD3	SNRPD3;SMD3;Sm-D3	small nuclear ribonucleoprotein D3 polypeptide 18kDa	0.3	0.42	1.38	0.29
RPS15A	RPS15A;FLJ27457;MGC111208;S15a	ribosomal protein S15a	0.3	0.37	1.38	0.29
TMPRSS7	TMPRSS7	transmembrane protease, serine 7	0.26	0.36	1.38	0.28
PLK1	PLK1;PLK;STPK13	polo-like kinase 1	0.2	0.29	1.38	0.24
GRM5	GRM5;GPRC1E;MGLUR5;mGlu5	glutamate receptor, metabotropic 5	0.14	0.18	1.38	0.16
KPNB1	KPNB1;IMB1;IPO1;IPOB;Impnb;MGC2155;MGC2156;MGC2157;NTF97	karyopherin (importin) beta 1	0.12	0.17	1.38	0.12
FAM124A	FAM124A;FLJ30707;FLJ32081	family with sequence similarity 124A	0.6	0.81	1.39	0.61
PNMA6A	PNMA6A;MA6;MGC15827;PNMA6	paraneoplastic antigen like 6A	0.58	0.73	1.39	0.58
ARMC9	ARMC9;ARM;FLJ12584;KU-MEL-1	armadillo repeat containing 9	0.57	0.72	1.39	0.56
FAM172A	FAM172A;C5orf21;DKFZp564D172	family with sequence similarity 172, member A	0.57	0.77	1.39	0.58

PDLIM3	PDLIM3;ALP;DKFZp686L0362;FLJ26096	PDZ and LIM domain 3	0.56	0.8	1.39	0.55
PRR19	PRR19;MGC70924	proline rich 19	0.52	0.7	1.39	0.56
ATF3	ATF3;FLJ41705	activating transcription factor 3	0.52	0.7	1.39	0.5
CDHR4	CDHR4;CDH29;MGC164982;PRO34300	cadherin-related family member 4	0.5	0.67	1.39	0.53
SP110	SP110;FLJ22835;IFI41;IFI75;IPR1;VODI	SP110 nuclear body protein	0.46	0.58	1.39	0.46
MFAP3L	MFAP3L;NYD-sp9	microfibrillar-associated protein 3-like	0.46	0.62	1.39	0.47
GNG8	GNG8	guanine nucleotide binding protein (G protein), gamma 8	0.46	0.61	1.39	0.52
APCDD1L	APCDD1L;FLJ90166;MGC126807;MGC126809;RP4-685L9.2	adenomatosis polyposis coli down-regulated 1-like	0.46	0.62	1.39	0.45
ZC3H18	ZC3H18;FLJ22664;FLJ34530;FLJ36075;NHN1	zinc finger CCCH-type containing 18	0.45	0.64	1.39	0.48
HOXB13	HOXB13;PSGD	homeobox B13	0.44	0.61	1.39	0.45
KNCN	KNCN;FLJ32011;Kino;L5;MGC120370;MGC120371;MGC120372	kinocilin	0.44	0.67	1.39	0.45
AIM1L	AIM1L;CRYBG2;DKFZp434L1713;FLJ10040;FLJ38020	absent in melanoma 1-like	0.44	0.59	1.39	0.43
IL18BP	IL18BP;IL18BPa	interleukin 18 binding protein	0.42	0.57	1.39	0.39
RND3	RND3;ARHE;Rho8;RhoE;memB	Rho family GTPase 3	0.42	0.55	1.39	0.41
C15orf42	C15orf42;FLJ41618;MGC45866;TICRR;Treslin	chromosome 15 open reading frame 42	0.4	0.61	1.39	0.41
HIST1H2BO	HIST1H2BO;H2B.2;H2B/n;H2BFN;MGC119806;dJ193B12.2	histone cluster 1, H2bo	0.36	0.52	1.39	0.42
SLC16A10	SLC16A10;PRO0813;TAT1	solute carrier family 16, member 10 (aromatic amino acid transporter)	0.35	0.46	1.39	0.34
NANS	NANS;SAS	N-acetylneuraminic acid synthase	0.34	0.46	1.39	0.4
CHAF1A	CHAF1A;CAF-1;CAF1;CAF1B;CAF1P150;MGC71229;P150	chromatin assembly factor 1, subunit A (p150)	0.32	0.44	1.39	0.31
GATA1	GATA1;ERYF1;GATA-1;GF-1;GF1;NFE1;XLTT	GATA binding protein 1 (globin transcription factor 1)	0.31	0.4	1.39	0.35
C22orf29	C22orf29;FLJ21125	chromosome 22 open reading frame 29	0.26	0.35	1.39	0.26
ZNF483	ZNF483;ZKSCAN16	zinc finger protein 483	0.11	0.15	1.39	0.12
DDX46	DDX46;FLJ25329;KIAA0801;MGC9936;PRPF5;Prp5	DEAD (Asp-Glu-Ala-Asp) box polypeptide 46	0.6	0.77	1.4	0.68
HADHA	HADHA;ECHA;GBP;HADH;LCEH;LCHAD;MGC1728;MTPA;TP-ALPHA	hydroxyacyl-CoA dehydrogenase/3-ketoacyl-CoA thiolase/enoyl-CoA hydratase (trifunctional protein), alpha subunit	0.6	0.84	1.4	0.57
ARHGEF35	ARHGEF35;ARHGEF5L;CTAGE4;FLJ43692	Rho guanine nucleotide exchange factor (GEF) 35	0.6	0.76	1.4	0.61
UTP15	UTP15;FLJ12787;NET21	UTP15, U3 small nucleolar ribonucleoprotein, homolog (S. cerevisiae)	0.58	0.77	1.4	0.62
MYCT1	MYCT1;FLJ21269;MGC156309;MGC156310;MTLC	myc target 1	0.56	0.75	1.4	0.54
TMEM218	TMEM218;DKFZp667A247	transmembrane protein 218	0.54	0.72	1.4	0.56
CLDN6	CLDN6	claudin 6	0.52	0.72	1.4	0.49
YIPF2	YIPF2;FinGER2;MGC3262	Yip1 domain family, member 2	0.52	0.75	1.4	0.54
ITGB1BP1	ITGB1BP1;DKFZp686K08158;ICAP-1A;ICAP-1B;ICAP-1alpha;ICAP1;ICAP1A;ICAP1B	integrin beta 1 binding protein 1	0.52	0.7	1.4	0.55
RBM4B	RBM4B;MGC10871;RBM30;RBM4L;ZCCHC15;ZCRB3B	RNA binding motif protein 4B	0.52	0.7	1.4	0.54
TM4SF19	TM4SF19	transmembrane 4 L six family member 19	0.5	0.69	1.4	0.52
FHDC1	FHDC1;KIAA1727	FH2 domain containing 1	0.48	0.61	1.4	0.48
CABYR	CABYR;CABYRa;CABYRc;CABYRc/d;CABYRe;CBP86;CT88;FSP-2;FSP2;MGC9117	calcium binding tyrosine-(Y)-phosphorylation regulated	0.48	0.64	1.4	0.46
TIGD5	TIGD5;FLJ14926;MGC44883	tigger transposable element derived 5	0.48	0.64	1.4	0.47
ANXA4	ANXA4;ANX4;DKFZp686H02120;MGC75105;PIG28;ZAP36	annexin A4	0.44	0.64	1.4	0.43
ZNF202	ZNF202;ZKSCAN10	zinc finger protein 202	0.43	0.57	1.4	0.46
RPL6	RPL6;L6;SHUJUN-2;TAXREB107;TXREB1	ribosomal protein L6	0.43	0.61	1.4	0.41
PLA2G4A	PLA2G4A;MGC126350;PLA2G4;cPLA2-alpha	phospholipase A2, group IVA (cytosolic, calcium-dependent)	0.42	0.62	1.4	0.47
HYAL1	HYAL1;HYAL-1;LUCA1;MGC45987;NAT6	hyaluronoglucosaminidase 1	0.41	0.54	1.4	0.42
HEXA	HEXA;MGC99608;TSD	hexosaminidase A (alpha polypeptide)	0.41	0.55	1.4	0.48
KRTDAP	KRTDAP;KDAP;MGC163373;UNQ467	keratinocyte differentiation-associated protein	0.39	0.57	1.4	0.43
PCNT	PCNT;KEN;MOPD2;PCN;PCNT2;PCNTB;PCTN2;SCKL4	pericentrin	0.38	0.52	1.4	0.39
RPL18A	RPL18A;L18A	ribosomal protein L18a	0.38	0.5	1.4	0.38
CNKSR1	CNKSR1;CNK;CNK1;KSR	connector enhancer of kinase suppressor of Ras 1	0.37	0.46	1.4	0.39
FAM89A	FAM89A;C1orf153;MGC15887;RP11-423F24.2	family with sequence similarity 89, member A	0.36	0.49	1.4	0.39
EI24	EI24;EPG4;PIG8;TP53I8	etoposide induced 2.4 mRNA	0.34	0.5	1.4	0.33
NPIPL2	NPIPL2;A-761H5.4	nuclear pore complex interacting protein-like 2	0.32	0.44	1.4	0.35
TUBB7P	TUBB7P;TUBB4Q	tubulin, beta 7, pseudogene	0.22	0.32	1.4	0.25
COPB1	COPB1;COPB;DKFZp761K102;FLJ10341;FLJ46444;FLJ57957	coatomer protein complex, subunit beta 1	0.18	0.24	1.4	0.18
PTH2	PTH2;TIP39	parathyroid hormone 2	0.6	0.84	1.41	0.57
KRTAP5-3	KRTAP5-3;KRTAP5-9;KRTAP5.3	keratin associated protein 5-3	0.6	0.83	1.41	0.65
AKR1CL1	AKR1CL1;FLJ16347	aldo-keto reductase family 1, member C-like 1	0.59	0.81	1.41	0.61
GPC3	GPC3;DGSX;GTR2-2;MXR7;OCI-5;SDYS;SGB;SGBS;SGBS1	glypican 3	0.59	0.81	1.41	0.64
NOSIP	NOSIP	nitric oxide synthase interacting protein	0.57	0.72	1.41	0.57
C10orf27	C10orf27;FLJ32820;spatial	chromosome 10 open reading frame 27	0.53	0.77	1.41	0.53
LARP1	LARP1;KIAA0731;LARP;MGC19556	La ribonucleoprotein domain family, member 1	0.51	0.75	1.41	0.54
NRXN2	NRXN2;FLJ40892;KIAA0921	neurexin 2	0.5	0.72	1.41	0.48
SAP18	SAP18;2HOR0202;MGC27131;SAP18P	Sin3A-associated protein, 18kDa	0.5	0.65	1.41	0.52
MAST3	MAST3;KIAA0561	microtubule associated serine/threonine kinase 3	0.49	0.7	1.41	0.57
AGPS	AGPS;ADAP-S;ADAS;ADHAPS;ADPS;ALDHPSY;DKFZp762O2215;FLJ99755	alkylglycerone phosphate synthase	0.49	0.66	1.41	0.49
PCP4	PCP4;PEP-19	Purkinje cell protein 4	0.48	0.7	1.41	0.46
COMMD2	COMMD2;MGC57611	COMM domain containing 2	0.48	0.68	1.41	0.52
ARHGAP21	ARHGAP21;ARHGAP10;DKFZp761L0424;FLJ33323;FLJ90108	Rho GTPase activating protein 21	0.46	0.66	1.41	0.51
RHOC	RHOC;ARH9;ARHC;H9;MGC1448;MGC61427;RHOH9	ras homolog gene family, member C	0.44	0.57	1.41	0.51
LOC283331	LOC283331	ENTREZ STATUS: REMAPPED TO GENE ID: 57701	0.43	0.62	1.41	0.42
RPS18	RPS18;D6S218E;HKE3;KE-3;KE3;MGC117351;MGC126835;MGC126837;S18	ribosomal protein S18	0.39	0.57	1.41	0.39
NUP43	NUP43;FLJ13287;bA350J20.1;p42	nucleoporin 43kDa	0.38	0.56	1.41	0.37
SLC16A3	SLC16A3;MCT 3;MCT 4;MCT-3;MCT-4;MCT3;MCT4;MGC138472;MGC138474	solute carrier family 16, member 3 (monocarboxylic acid transporter 4)	0.34	0.47	1.41	0.34
RPS6	RPS6;S6	ribosomal protein S6	0.34	0.46	1.41	0.36
POLR2I	POLR2I;RPB9;hRPB14.5	polymerase (RNA) II (DNA directed) polypeptide I, 14.5kDa	0.3	0.4	1.41	0.29
RRM2	RRM2;R2;RR2;RR2M	ribonucleotide reductase M2	0.25	0.35	1.41	0.27
C21orf62	C21orf62;B37;C21orf120;PRED81	chromosome 21 open reading frame 62	0.22	0.29	1.41	0.24
LCP2	LCP2;SLP-76;SLP76	lymphocyte cytosolic protein 2 (SH2 domain containing leukocyte protein of 76kDa)	0.6	0.78	1.42	0.65
GPR37L1	GPR37L1;ET(B)R-LP-2;ETBR-LP-2	G protein-coupled receptor 37 like 1	0.6	0.8	1.42	0.6
RASA1	RASA1;CM-AVM;CMAVM;DKFZp434N071;GAP;PKWS;RASA;RASGAP;p120GAP;p120RASGAP	RAS p21 protein activator (GTPase activating protein) 1	0.6	0.79	1.42	0.7
PLXDC2	PLXDC2;FLJ14623;TEM7R	plexin domain containing 2	0.57	0.73	1.42	0.58
PPCDC	PPCDC;FLJ14585	phosphopantothenoylcysteine decarboxylase	0.56	0.74	1.42	0.59
ZFP64	ZFP64;MGC940;ZNF338	zinc finger protein 64 homolog (mouse)	0.55	0.77	1.42	0.6
ADH5	ADH5;ADH-3;ADHX;FALDH;FDH;GSH-FDH;GSNOR	alcohol dehydrogenase 5 (class III), chi polypeptide	0.52	0.69	1.42	0.61
OR2AE1	OR2AE1;OR2AE2	olfactory receptor, family 2, subfamily AE, member 1	0.51	0.69	1.42	0.5
SLC22A8	SLC22A8;MGC24086;OAT3	solute carrier family 22 (organic anion transporter), member 8	0.5	0.66	1.42	0.53
AZU1	AZU1;AZAMP;AZU;CAP37;HBP;HUMAZUR;NAZC	azurocidin 1	0.49	0.68	1.42	0.51
ZNHIT3	ZNHIT3;TRIP3	zinc finger, HIT-type containing 3	0.49	0.7	1.42	0.47
PSCA	PSCA;PRO232	prostate stem cell antigen	0.46	0.64	1.42	0.49
GSC	GSC	goosecoid homeobox	0.45	0.65	1.42	0.44
C2CD3	C2CD3;DKFZp586P0123;FLJ34770	C2 calcium-dependent domain containing 3	0.45	0.61	1.42	0.44
LPAR4	LPAR4;GPR23;LPA4;P2RY9;P2Y5-LIKE;P2Y9	lysophosphatidic acid receptor 4	0.44	0.62	1.42	0.56
NANOS1	NANOS1;NOS1	nanos homolog 1 (Drosophila)	0.43	0.6	1.42	0.48
GH1	GH1;GH;GH-N;GHN;IGHD1B;hGH-N	growth hormone 1	0.42	0.63	1.42	0.44
C9orf89	C9orf89;BinCARD;MGC110898;MGC11115;bA370F5.1	chromosome 9 open reading frame 89	0.4	0.58	1.42	0.42
LPAR3	LPAR3;EDG7;Edg-7;FLJ98231;GPCR;HOFNH30;LP-A3;LPA3;RP4-678I3	lysophosphatidic acid receptor 3	0.4	0.52	1.42	0.39
GPN1	GPN1;ATPBD1A;FLJ51176;MBDIN;NTPBP;RPAP4;XAB1	GPN-loop GTPase 1	0.39	0.5	1.42	0.39
FUT6	FUT6;FCT3A;FLJ40754;FT1A;Fuc-TVI;FucT-VI	fucosyltransferase 6 (alpha (1,3) fucosyltransferase)	0.38	0.52	1.42	0.41
DSC3	DSC3;CDHF3;DSC;DSC1;DSC2;DSC4;HT-CP	desmocollin 3	0.28	0.4	1.42	0.3
PSMC5	PSMC5;S8;SUG-1;SUG1;TBP10;TRIP1;p45;p45/SUG	proteasome (prosome, macropain) 26S subunit, ATPase, 5	0.27	0.38	1.42	0.3
TUBA1A	TUBA1A;B-ALPHA-1;FLJ25113;LIS3;TUBA3	tubulin, alpha 1a	0.25	0.36	1.42	0.26
TMEM95	TMEM95;MGC129793;UNQ9390	transmembrane protein 95	0.56	0.72	1.43	0.56
CACNA1F	CACNA1F;AIED;COD3;COD4;CORDX;CORDX3;CSNB2;CSNB2A;CSNBX2;Cav1.4;JM8;JMC8;OA2	calcium channel, voltage-dependent, L type, alpha 1F subunit	0.54	0.76	1.43	0.6
ASB2	ASB2;ASB-2;MGC40044	ankyrin repeat and SOCS box containing 2	0.52	0.72	1.43	0.52
ZBTB10	ZBTB10;FLJ12752;RINZF	zinc finger and BTB domain containing 10	0.52	0.74	1.43	0.52
RHOT1	RHOT1;ARHT1;FLJ11040;FLJ12633;MIRO-1;MIRO1	ras homolog gene family, member T1	0.52	0.76	1.43	0.51
RGMB	RGMB;DKFZp434P228;DRAGON;FLJ90406;MGC86970	RGM domain family, member B	0.47	0.6	1.43	0.5
IL37	IL37;FIL1;FIL1(ZETA);FIL1Z;IL-1F7;IL-1H;IL-1H4;IL-1RP1;IL-37;IL1F7;IL1H4;IL1RP1	interleukin 37	0.47	0.69	1.43	0.52
FLI1	FLI1;EWSR2;SIC-1	Friend leukemia virus integration 1	0.45	0.64	1.43	0.54
FCRL1	FCRL1;CD307a;DKFZp667O1421;FCRH1;IFGP1;IRTA5	Fc receptor-like 1	0.44	0.6	1.43	0.46
OTUD7A	OTUD7A;C15orf16;C16ORF15;CEZANNE2;OTUD7	OTU domain containing 7A	0.42	0.58	1.43	0.44
SPRR1B	SPRR1B;CORNIFIN;GADD33;MGC61901;SPRR1	small proline-rich protein 1B	0.37	0.5	1.43	0.42
GZMM	GZMM;LMET1;MET1	granzyme M (lymphocyte met-ase 1)	0.35	0.48	1.43	0.4
SYNGR1	SYNGR1;MGC1939	synaptogyrin 1	0.34	0.48	1.43	0.36
AGXT	AGXT;AGT;AGT1;AGXT1;PH1;SPAT;SPT;TLH6	alanine-glyoxylate aminotransferase	0.3	0.42	1.43	0.33
ARL14	ARL14;ARF7;FLJ22595	ADP-ribosylation factor-like 14	0.27	0.38	1.43	0.29
TPX2	TPX2;C20orf1;C20orf2;DIL-2;DIL2;FLS353;GD:C20orf1;HCA519;HCTP4;REPP86;p100	TPX2, microtubule-associated, homolog (Xenopus laevis)	0.17	0.22	1.43	0.19
NEUROD2	NEUROD2;MGC26304;NDRF;bHLHa1	neurogenic differentiation 2	0.6	0.74	1.25	0.6
MTCH1	MTCH1;CGI-64;MGC131998;PIG60;PSAP	mitochondrial carrier 1	0.6	0.76	1.25	0.51
TACC3	TACC3;ERIC-1;ERIC1;MGC117382;MGC133242	transforming, acidic coiled-coil containing protein 3	0.6	0.76	1.25	0.53
RWDD4	RWDD4;FAM28A;MGC10198;RWDD4A	RWD domain containing 4	0.6	0.73	1.25	0.54
GPCPD1	GPCPD1;FLJ11085;GDE5;GDPD6;KIAA1434;MGC26147;PREI4	glycerophosphocholine phosphodiesterase GDE1 homolog (S. cerevisiae)	0.6	0.82	1.25	0.54
SUN2	SUN2;KIAA0668;MGC133055;MGC133056;UNC84B	Sad1 and UNC84 domain containing 2	0.6	0.74	1.25	0.52
SRR	SRR;ILV1;ISO1	serine racemase	0.58	0.68	1.25	0.54
UTP11L	UTP11L;CGI94	UTP11-like, U3 small nucleolar ribonucleoprotein, (yeast)	0.58	0.79	1.25	0.52
NPNT	NPNT;EGFL6L;POEM	nephronectin	0.57	0.7	1.25	0.53
WBSCR28	WBSCR28;MGC26719	Williams-Beuren syndrome chromosome region 28	0.57	0.76	1.25	0.61
SEC24D	SEC24D;FLJ43974;KIAA0755	SEC24 family, member D (S. cerevisiae)	0.57	0.68	1.25	0.57
PHC3	PHC3;DKFZp313K1221;EDR3;FLJ12729;FLJ12967;HPH3;MGC88144	polyhomeotic homolog 3 (Drosophila)	0.57	0.7	1.25	0.58
CDHR3	CDHR3;CDH28;FLJ23834;FLJ43271;MGC133292;MGC133293	cadherin-related family member 3	0.57	0.74	1.25	0.5
SPICE1	SPICE1;CCDC52;FLJ26064;FLJ44949;SPICE	spindle and centriole associated protein 1	0.57	0.76	1.25	0.59
LAIR2	LAIR2;CD306;MGC71634	leukocyte-associated immunoglobulin-like receptor 2	0.56	0.68	1.25	0.55

CDC42EP5	CDC42EP5;Borg3;CEP5;MGC21945;MGC71153	CDC42 effector protein (Rho GTPase binding) 5	0.56	0.68	1.25	0.52
FOXD4L4	FOXD4L4;FOXD4b;bA460E7.2	forkhead box D4-like 4	0.56	0.68	1.25	0.51
C15orf48	C15orf48;FLJ22645;FOAP-11;MGC32925;NMES1	chromosome 15 open reading frame 48	0.55	0.71	1.25	0.53
GATA3	GATA3;HDR;HDRS;MGC2346;MGC5199;MGC5445	GATA binding protein 3	0.55	0.7	1.25	0.46
ZC3H10	ZC3H10;FLJ14451;ZC3HDC10	zinc finger CCCH-type containing 10	0.55	0.74	1.25	0.57
NOL3	NOL3;ARC;FLJ35304;MYP;NOP;NOP30	nucleolar protein 3 (apoptosis repressor with CARD domain)	0.55	0.72	1.25	0.48
MAPK3	MAPK3;ERK-1;ERK1;ERT2;HS44KDAP;HUMKER1A;MGC20180;P44ERK1;P44MAPK;PRKM3;p44-ERK1;p44-MAPK	mitogen-activated protein kinase 3	0.55	0.68	1.25	0.57
NAA30	NAA30;C14orf35;FLJ35355;MAK3;MGC141884;MGC141885;Mak3p;NAT12	N(alpha)-acetyltransferase 30, NatC catalytic subunit	0.55	0.72	1.25	0.53
HAL	HAL;HIS;HSTD	histidine ammonia-lyase	0.54	0.72	1.25	0.47
NMS	NMS	neuromedin S	0.53	0.7	1.25	0.49
ZNF107	ZNF107;Y8;ZFD25;ZNF588;smap-7	zinc finger protein 107	0.53	0.68	1.25	0.51
HSPBAP1	HSPBAP1;FLJ22623;FLJ39386;PASS1	HSPB (heat shock 27kDa) associated protein 1	0.53	0.69	1.25	0.52
TNFRSF13C	TNFRSF13C;BAFF-R;BAFFR;BROMIX;CD268;CVID4;MGC138235;prolixin	tumor necrosis factor receptor superfamily, member 13C	0.53	0.68	1.25	0.47
SETD3	SETD3;C14orf154;DKFZp761E1415;FLJ23027;MGC87236	SET domain containing 3	0.53	0.64	1.25	0.49
ZNF493	ZNF493;FLJ36504	zinc finger protein 493	0.52	0.63	1.25	0.48
KRTAP11-1	KRTAP11-1;HACL-1;HACL1;KAP11.1	keratin associated protein 11-1	0.52	0.63	1.25	0.47
HTR3A	HTR3A;5-HT-3;5-HT3A;5-HT3R;5HT3R;HTR3	5-hydroxytryptamine (serotonin) receptor 3A	0.52	0.6	1.25	0.46
ZNF470	ZNF470;CZF-1;FLJ26175;FLJ26846	zinc finger protein 470	0.52	0.7	1.25	0.47
ZNF490	ZNF490;KIAA1198	zinc finger protein 490	0.52	0.62	1.25	0.47
C4orf21	C4orf21;DKFZp313L226;DKFZp434C0927;FLJ11331;FLJ33237;FLJ44066	chromosome 4 open reading frame 21	0.52	0.66	1.25	0.49
HSPA6	HSPA6	heat shock 70kDa protein 6 (HSP70B')	0.52	0.62	1.25	0.49
MRPS7	MRPS7;MRP-S;MRP-S7;RP-S7;RPMS7;S7mt;bMRP27a	mitochondrial ribosomal protein S7	0.52	0.66	1.25	0.46
ZNF76	ZNF76;D6S229E;ZNF523;Zfp523	zinc finger protein 76	0.51	0.66	1.25	0.45
MATR3	MATR3;DKFZp686K0542;DKFZp686K23100;KIAA0723;MGC9105;MPD2;VCPDM	matrin 3	0.51	0.68	1.25	0.55
OR2G2	OR2G2;OR1-32	olfactory receptor, family 2, subfamily G, member 2	0.5	0.65	1.25	0.49
ZP4	ZP4;ZBP;ZP1;ZPB;Zp-4	zona pellucida glycoprotein 4	0.5	0.64	1.25	0.49
HOXD12	HOXD12;HOX4H	homeobox D12	0.5	0.62	1.25	0.42
ZBTB20	ZBTB20;DKFZp566F123;DPZF;FLJ26458;HOF;ODA-8S;ZNF288	zinc finger and BTB domain containing 20	0.5	0.65	1.25	0.48
SEPW1	SEPW1;selW	selenoprotein W, 1	0.5	0.63	1.25	0.43
CSTF3	CSTF3;CSTF-77;MGC117398;MGC43001;MGC75122	cleavage stimulation factor, 3' pre-RNA, subunit 3, 77kDa	0.5	0.57	1.25	0.48
ENTPD1	ENTPD1;ATPDase;CD39;DKFZp686D194;DKFZp686I093;FLJ40921;FLJ40959;NTPDase-1	ectonucleoside triphosphate diphosphohydrolase 1	0.49	0.57	1.25	0.5
MTBP	MTBP;MDM2BP	Mdm2, transformed 3T3 cell double minute 2, p53 binding protein (mouse) binding protein, 104kDa	0.48	0.64	1.25	0.45
MAGI1	MAGI1;AIP-3;AIP3;BAIAP1;BAP-1;BAP1;MAGI-1;TNRC19;WWP3	membrane associated guanylate kinase, WW and PDZ domain containing 1	0.48	0.64	1.25	0.45
SIX3	SIX3;HPE2	SIX homeobox 3	0.47	0.57	1.25	0.43
REM2	REM2;FLJ38964	RAS (RAD and GEM)-like GTP binding 2	0.47	0.62	1.25	0.44
VPS37C	VPS37C;FLJ20847	vacuolar protein sorting 37 homolog C (S. cerevisiae)	0.47	0.57	1.25	0.43
SLC38A3	SLC38A3;G17;NAT1;SN1	solute carrier family 38, member 3	0.46	0.62	1.25	0.47
PODNL1	PODNL1;FLJ23447;SLRR5B	podocan-like 1	0.46	0.56	1.25	0.43
INTS1	INTS1;DKFZp586J0619;FLJ46624;INT1;KIAA1440;NET28	integrator complex subunit 1	0.46	0.62	1.25	0.47
SDC1	SDC1;CD138;SDC;SYND1;syndecan	syndecan 1	0.46	0.6	1.25	0.45
HGD	HGD;AKU;FLJ94126;HGO	homogentisate 1,2-dioxygenase	0.45	0.59	1.25	0.39
CORO2A	CORO2A;CLIPINB;DKFZp686G19226;IR10;WDR2	coronin, actin binding protein, 2A	0.44	0.56	1.25	0.4
PSMA4	PSMA4;HC9;HsT17706;MGC111191;MGC12467;MGC24813;PSC9	proteasome (prosome, macropain) subunit, alpha type, 4	0.44	0.54	1.25	0.41
ORC1	ORC1;HSORC1;ORC1L;PARC1	origin recognition complex, subunit 1	0.44	0.53	1.25	0.43
SNRNP200	SNRNP200;ASCC3L1;BRR2;FLJ11521;HELIC2;RP33;U5-200KD	small nuclear ribonucleoprotein 200kDa (U5)	0.44	0.56	1.25	0.44
ZFP37	ZFP37;FLJ38524;ZNF906	zinc finger protein 37 homolog (mouse)	0.42	0.51	1.25	0.44
RBAK	RBAK;ZNF769	RB-associated KRAB zinc finger	0.42	0.53	1.25	0.35
CIT	CIT;CRIK;KIAA0949;STK21	citron (rho-interacting, serine/threonine kinase 21)	0.42	0.56	1.25	0.4
PCCB	PCCB;DKFZp451E113	propionyl CoA carboxylase, beta polypeptide	0.42	0.54	1.25	0.36
MLIP	MLIP;C6orf142;FLJ54673;FLJ79056;MGC18257	muscular LMNA-interacting protein	0.42	0.5	1.25	0.41
RPL19P9	RPL19P9;RPL19_7_643	ribosomal protein L19 pseudogene 9	0.42	0.46	1.25	0.38
MAGEF1	MAGEF1;MGC19617	melanoma antigen family F, 1	0.4	0.5	1.25	0.34
RPS19	RPS19;DBA;DBA1;S19	ribosomal protein S19	0.4	0.47	1.25	0.34
PAX9	PAX9;STHAG3	paired box 9	0.38	0.51	1.25	0.35
CNOT1	CNOT1;CDC39;DKFZp686E0722;DKFZp686O168;FLJ36492;FLJ90644;KIAA1007;NOT1;NOT1H	CCR4-NOT transcription complex, subunit 1	0.38	0.51	1.25	0.35
WFDC6	WFDC6;C20orf171;MGC126649;MGC126653;WAP6;dJ461P17.11	WAP four-disulfide core domain 6	0.36	0.48	1.25	0.38
THAP3	THAP3;MGC33488	THAP domain containing, apoptosis associated protein 3	0.36	0.4	1.25	0.31
CEP164	CEP164;FLJ54767;KIAA1052	centrosomal protein 164kDa	0.34	0.45	1.25	0.32
SNRPF	SNRPF;SMF;Sm-F	small nuclear ribonucleoprotein polypeptide F	0.32	0.4	1.25	0.28
SOAT2	SOAT2;ACACT2;ACAT2;ARGP2;MGC116732	sterol O-acyltransferase 2	0.31	0.39	1.25	0.26
MAGEA2	MAGEA2;CT1.2;MAGE2;MAGEA2A;MAGEA2B;MGC131923	melanoma antigen family A, 2	0.31	0.37	1.25	0.3
DIO1	DIO1;5DI;MGC130050;MGC130051;TXDI1	deiodinase, iodothyronine, type I	0.3	0.37	1.25	0.27
CD79A	CD79A;IGA;MB-1	CD79a molecule, immunoglobulin-associated alpha	0.3	0.35	1.25	0.31
G6PC3	G6PC3;SCN4;UGRP	glucose 6 phosphatase, catalytic, 3	0.3	0.36	1.25	0.27
TUBA1B	TUBA1B;K-ALPHA-1	tubulin, alpha 1b	0.24	0.29	1.25	0.22
HMGA1	HMGA1;HMG-R;HMGA1A;HMGIY;MGC12816;MGC4242;MGC4854	high mobility group AT-hook 1	0.6	0.48	0.81	0.35
CYBB	CYBB;AMCBX2;CGD;GP91-1;GP91-PHOX;GP91PHOX;NOX2;p91-PHOX	cytochrome b-245, beta polypeptide	0.58	0.5	0.81	0.38
PYY	PYY;PYY1	peptide YY	0.55	0.44	0.81	0.3
SAP130	SAP130;FLJ12761	Sin3A-associated protein, 130kDa	0.54	0.45	0.81	0.35
CDC7	CDC7;CDC7L1;HsCDC7;Hsk1;MGC117361;MGC126237;MGC126238;huCDC7	cell division cycle 7 homolog (S. cerevisiae)	0.48	0.39	0.81	0.32
PDCD2	PDCD2;MGC12347;RP8;ZMYND7	programmed cell death 2	0.44	0.34	0.81	0.27
MYF6	MYF6;MRF4;bHLHc4;myf-6	myogenic factor 6 (herculin)	0.42	0.38	0.81	0.26
DUS3L	DUS3L;DUS3;FLJ13896	dihydrouridine synthase 3-like (S. cerevisiae)	0.35	0.28	0.81	0.21
OR8U9	OR8U9	olfactory receptor, family 8, subfamily U, member 9	0.3	0.24	0.81	0.17
DISP2	DISP2;DISPB;DKFZp547N223;HsT16908;KIAA1742	dispatched homolog 2 (Drosophila)	0.26	0.22	0.81	0.16
C9orf117	C9orf117	chromosome 9 open reading frame 117	0.6	0.48	0.82	0.36
IL17RD	IL17RD;DKFZp434N1928;FLJ35755;IL-17RD;IL17RLM;MGC133309;SEF	interleukin 17 receptor D	0.6	0.48	0.82	0.38
RPA2	RPA2;REPA2;RPA32	replication protein A2, 32kDa	0.58	0.5	0.82	0.37
DACT2	DACT2;C6orf116;DAPPER2;DPR2;FLJ31232;MGC133141;MGC133142;RP11-503C24.7;bA503C24.7	dapper, antagonist of beta-catenin, homolog 2 (Xenopus laevis)	0.57	0.48	0.82	0.35
CENPK	CENPK;AF5alpha;CENP-K;P33;Solt	centromere protein K	0.56	0.49	0.82	0.34
ISG15	ISG15;G1P2;IFI15;IP17;UCRP;hUCRP	ISG15 ubiquitin-like modifier	0.55	0.49	0.82	0.33
IGFBP4	IGFBP4;BP-4;HT29-IGFBP;IBP4;IGFBP-4	insulin-like growth factor binding protein 4	0.55	0.43	0.82	0.33
LIG4	LIG4	ligase IV, DNA, ATP-dependent	0.52	0.42	0.82	0.3
BCL2L12	BCL2L12;MGC120313;MGC120314;MGC120315	BCL2-like 12 (proline rich)	0.51	0.44	0.82	0.33
EEF2K	EEF2K;HSU93850;MGC45041;eEF-2K	eukaryotic elongation factor-2 kinase	0.5	0.42	0.82	0.34
EIF3F	EIF3F;EIF3S5;eIF3-p47	eukaryotic translation initiation factor 3, subunit F	0.59	0.49	0.83	0.33
GJB1	GJB1;CMTX;CMTX1;CX32	gap junction protein, beta 1, 32kDa	0.58	0.52	0.83	0.34
ZBTB26	ZBTB26;KIAA1572;ZNF481;bioref	zinc finger and BTB domain containing 26	0.58	0.49	0.83	0.34
LNP1	LNP1	leukemia NUP98 fusion partner 1	0.58	0.49	0.83	0.33
NINJ2	NINJ2;FLJ56349	ninjurin 2	0.57	0.5	0.83	0.33
KIF1A	KIF1A;ATSV;C2orf20;DKFZp686I2094;FLJ30229;HSN2C;HUNC-104;MGC133285;MGC133286;MRD9;SPG30;UNC104	kinesin family member 1A	0.56	0.47	0.83	0.32
KIF22	KIF22;A-328A3.2;KID;KNSL4;OBP;OBP-1;OBP-2	kinesin family member 22	0.55	0.45	0.83	0.33
IL15RA	IL15RA;CD215;MGC104179	interleukin 15 receptor, alpha	0.54	0.5	0.83	0.34
TRIM47	TRIM47;GOA;RNF100	tripartite motif containing 47	0.5	0.42	0.83	0.37
STAMBP	STAMBP;AMSH;MGC126516;MGC126518	STAM binding protein	0.5	0.4	0.83	0.32
PHRF1	PHRF1;KIAA1542;RNF221	PHD and ring finger domains 1	0.39	0.32	0.83	0.29
CSF1R	CSF1R;C-FMS;CD115;CSFR;FIM2;FMS	colony stimulating factor 1 receptor	0.29	0.24	0.83	0.2
SCGB1C1	SCGB1C1;RYD5	secretoglobin, family 1C, member 1	0.6	0.5	0.84	0.36
MPV17L2	MPV17L2;FKSG24;MGC110861;MGC12972	MPV17 mitochondrial membrane protein-like 2	0.59	0.48	0.84	0.38
ZNF571	ZNF571;FLJ16374;MGC138690	zinc finger protein 571	0.56	0.49	0.84	0.37
SH3GL1	SH3GL1;CNSA1;EEN;MGC111371;SH3D2B;SH3P8	SH3-domain GRB2-like 1	0.54	0.46	0.84	0.31
TMED4	TMED4;ERS25;HNLF	transmembrane emp24 protein transport domain containing 4	0.53	0.44	0.84	0.33
CACNA2D1	CACNA2D1;CACNA2;CACNL2A;CCHL2A	calcium channel, voltage-dependent, alpha 2/delta subunit 1	0.52	0.47	0.84	0.36
IGFL3	IGFL3;UNQ483	IGF-like family member 3	0.51	0.46	0.84	0.31
MCPH1	MCPH1;BRIT1;FLJ12847;MCT	microcephalin 1	0.51	0.38	0.84	0.3
AKD1	AKD1;AKD2;C6orf199;C6orf224;FLJ16163;FLJ25791;FLJ34784;FLJ42177;MGC126763;MGC138153;MGC177059;MGC180194;MGC184281;MGC26954;RP1-70A9.1;dJ70A9.1	adenylate kinase domain containing 1	0.49	0.4	0.84	0.3
RRAD	RRAD;RAD;RAD1;REM3	Ras-related associated with diabetes	0.44	0.4	0.84	0.3
ATP6V1B1	ATP6V1B1;ATP6B1;MGC32642;RTA1B;VATB;VMA2;VPP3	ATPase, H+ transporting, lysosomal 56/58kDa, V1 subunit B1	0.38	0.31	0.84	0.27
FAM208A	FAM208A;C3orf63;DKFZp686C2456;KIAA1105;RAP140;se89-1	family with sequence similarity 208, member A	0.37	0.34	0.84	0.22
HFE	HFE;HFE1;HH;HLA-H;IMAGE:40125754;MGC103790;MGC:150812;MVCD7;TFQTL2;dJ221C16.10.1	hemochromatosis	0.36	0.32	0.84	0.24
CYBA	CYBA;p22-PHOX	cytochrome b-245, alpha polypeptide	0.6	0.51	0.85	0.4
FN3KRP	FN3KRP;FLJ12171;FN3KL	fructosamine 3 kinase related protein	0.6	0.52	0.85	0.42
ALDH1L1	ALDH1L1;10-fTHF;DKFZp781N0997;FDH;FTHFD	aldehyde dehydrogenase 1 family, member L1	0.59	0.52	0.85	0.41
CASQ1	CASQ1;CASQ;PDIB1	calsequestrin 1 (fast-twitch, skeletal muscle)	0.58	0.48	0.85	0.38
FAM178A	FAM178A;C10orf6;MGC90157	family with sequence similarity 178, member A	0.58	0.5	0.85	0.34
GTF3C1	GTF3C1;DKFZp686A111;TFIIIC;TFIIIC220;TFIIICalpha	general transcription factor IIIC, polypeptide 1, alpha 220kDa	0.56	0.48	0.85	0.37
DDI1	DDI1;FLJ36017	DNA-damage inducible 1 homolog 1 (S. cerevisiae)	0.55	0.44	0.85	0.37
G2E3	G2E3;FLJ20333;KIAA1333;PHF7B	G2/M-phase specific E3 ubiquitin protein ligase	0.54	0.46	0.85	0.41
ROGDI	ROGDI;FLJ22386	rogdi homolog (Drosophila)	0.5	0.4	0.85	0.31
C9orf86	C9orf86;FLJ10101;FLJ13045;KIAA1984;Parf;RBEL1;bA216L13.9;pp8875	chromosome 9 open reading frame 86	0.5	0.42	0.85	0.31
TMEM43	TMEM43;ARVC5;ARVD5;DKFZp586G1919;LUMA;MGC3222	transmembrane protein 43	0.5	0.44	0.85	0.33
SNIP1	SNIP1;FLJ12553;RP3-423B22.3;dJ423B22.2	Smad nuclear interacting protein 1	0.46	0.38	0.85	0.27
ALKBH5	ALKBH5;ABH5;FLJ20308;OFOXD;OFOXD1	alkB, alkylation repair homolog 5 (E. coli)	0.44	0.4	0.85	0.27
SRA1	SRA1;MGC87674;SRA;SRAP;STRAA1;pp7684	steroid receptor RNA activator 1	0.38	0.3	0.85	0.22
METTL3	METTL3;IME4;M6A;MGC4336;MT-A70;Spo8	methyltransferase like 3	0.6	0.58	0.86	0.4
SLC26A10	SLC26A10;FLJ32854	solute carrier family 26, member 10	0.58	0.47	0.86	0.37
EPHX3	EPHX3;ABHD9;FLJ22408;MGC131519	epoxide hydrolase 3	0.58	0.46	0.86	0.37
APOA4	APOA4;MGC142154;MGC142156	apolipoprotein A-IV	0.57	0.48	0.86	0.34
C6orf218	C6orf218;MGC40222	chromosome 6 open reading frame 218	0.56	0.48	0.86	0.34

HIPK3	HIPK3;DYRK6;FIST3;PKY;YAK1	homeodomain interacting protein kinase 3	0.54	0.46	0.86	0.38
CAMK4	CAMK4;CaMK IV;CaMK-GR;IV;MGC36771;caMK	calcium/calmodulin-dependent protein kinase IV	0.52	0.48	0.86	0.34
CDK17	CDK17;PCTAIRE2;PCTK2	cyclin-dependent kinase 17	0.48	0.45	0.86	0.32
UBA52	UBA52;CEP52;HUBCEP52;L40;MGC126879;MGC126881;MGC57125;RPL40	ubiquitin A-52 residue ribosomal protein fusion product 1	0.4	0.36	0.86	0.28
TLE6	TLE6;FLJ14009;GRG6;MGC14966	transducin-like enhancer of split 6 (E(sp1) homolog, Drosophila)	0.6	0.56	0.87	0.38
AGPHD1	AGPHD1	aminoglycoside phosphotransferase domain containing 1	0.59	0.53	0.87	0.36
C14orf166	C14orf166;CGI99;CLE;CLE7;LCRP369;RLLM1	chromosome 14 open reading frame 166	0.57	0.51	0.87	0.36
C2orf27A	C2orf27A;C2orf27;C2orf27B;MGC138394	chromosome 2 open reading frame 27A	0.57	0.48	0.87	0.37
CYP46A1	CYP46A1;CP46;CYP46	cytochrome P450, family 46, subfamily A, polypeptide 1	0.56	0.52	0.87	0.34
SERPINI1	SERPINI1;DKFZp781N13156;PI12;neuroserpin	serpin peptidase inhibitor, clade I (neuroserpin), member 1	0.55	0.49	0.87	0.39
SEMA4G	SEMA4G;FLJ20590;KIAA1619;MGC102867	sema domain, immunoglobulin domain (Ig), transmembrane domain (TM) and short cytoplasmic domain, (semaphorin) 4G	0.55	0.5	0.87	0.38
TNK1	TNK1;MGC46193	tyrosine kinase, non-receptor, 1	0.55	0.52	0.87	0.37
HSP90B1	HSP90B1;ECGP;GP96;GRP94;TRA1	heat shock protein 90kDa beta (Grp94), member 1	0.55	0.47	0.87	0.35
PPM1E	PPM1E;CaMKP-N;DKFZp781F1422;KIAA1072;POPX1;PP2CH	protein phosphatase, Mg2+/Mn2+ dependent, 1E	0.54	0.43	0.87	0.33
DMAP1	DMAP1;DKFZp686L09142;DNMAP1;DNMTAP1;EAF2;FLJ11543;KIAA1425;MEAF2;SWC4	DNA methyltransferase 1 associated protein 1	0.54	0.52	0.87	0.38
KRT83	KRT83;HB3;Hb-3;KRTHB3;MGC141663	keratin 83	0.5	0.41	0.87	0.32
CCDC121	CCDC121;FLJ13646;FLJ43364	coiled-coil domain containing 121	0.5	0.46	0.87	0.33
FNDC9	FNDC9;C5orf40;MGC27121	fibronectin type III domain containing 9	0.49	0.45	0.87	0.36
CHIT1	CHIT1;CHI3;CHIT;CHITD;FLJ00314;MGC125322	chitinase 1 (chitotriosidase)	0.48	0.41	0.87	0.35
TUB	TUB;rd5	tubby homolog (mouse)	0.46	0.44	0.87	0.33
KHSRP	KHSRP;FBP2;FUBP2;KSRP;MGC99676	KH-type splicing regulatory protein	0.45	0.44	0.87	0.3
SSTR3	SSTR3	somatostatin receptor 3	0.42	0.34	0.87	0.26
FITM1	FITM1;FIT1;MGC46490	fat storage-inducing transmembrane protein 1	0.42	0.37	0.87	0.25
PNPLA1	PNPLA1;FLJ38755;MGC119319;MGC119320;MGC119321;dJ50J22.1	patatin-like phospholipase domain containing 1	0.6	0.58	0.88	0.4
SYT9	SYT9;FLJ45896	synaptotagmin IX	0.59	0.51	0.88	0.38
ZNF185	ZNF185	zinc finger protein 185 (LIM domain)	0.59	0.52	0.88	0.46
LXN	LXN;ECI;TCI	latexin	0.58	0.5	0.88	0.4
38231	SEPT4;ARTS;BRADEION;CE5B3;H5;MART;PNUTL2;SEP4;hCDCREL-2	septin 4	0.58	0.47	0.88	0.42
PRSS53	PRSS53;FLJ00289;POL3S;UNQ308	protease, serine, 53	0.57	0.55	0.88	0.36
MUM1	MUM1;EXPAND1;FLJ14868;FLJ22283;HSPC211;MGC131891;MGC163315;MUM-1	melanoma associated antigen (mutated) 1	0.56	0.48	0.88	0.36
RBMX	RBMX;HNRPG;RBMXP1;RBMXRT;RNMX;hnRNP-G	RNA binding motif protein, X-linked	0.55	0.46	0.88	0.38
GALNT10	GALNT10;DKFZp586H0623;FLJ00205;FLJ11715;GALNACT10;PPGALNACT10;PPGANTASE10	UDP-N-acetyl-alpha-D-galactosamine:polypeptide N-acetylgalactosaminyltransferase 10 (GalNAc-T10)	0.53	0.45	0.88	0.33
38961	SEPT6;KIAA0128;MGC16619;MGC20339;SEP2;SEPT2	septin 6	0.52	0.5	0.88	0.37
PAPOLA	PAPOLA;MGC5378;PAP	poly(A) polymerase alpha	0.52	0.44	0.88	0.34
EPG5	EPG5;DKFZp667P2311;HEEW1;KIAA1632;hEPG5	ectopic P-granules autophagy protein 5 homolog (C. elegans)	0.52	0.42	0.88	0.34
KCNN4	KCNN4;IK1;IKCA1;KCA4;KCa3.1;SK4;hIKCa1;hKCa4;hSK4	potassium intermediate/small conductance calcium-activated channel, subfamily N, member 4	0.5	0.43	0.88	0.34
ATP1B3	ATP1B3;ATPB-3;CD298;FLJ29027	ATPase, Na+/K+ transporting, beta 3 polypeptide	0.5	0.46	0.88	0.35
PTCD2	PTCD2;FLJ12598	pentatricopeptide repeat domain 2	0.48	0.44	0.88	0.34
C19orf56	C19orf56;PTD008	chromosome 19 open reading frame 56	0.48	0.43	0.88	0.32
UCN	UCN;MGC129974;MGC129975;UI;UROC	urocortin	0.47	0.4	0.88	0.3
TMEM61	TMEM61	transmembrane protein 61	0.24	0.24	0.88	0.16
DCK	DCK;MGC117410;MGC138632	deoxycytidine kinase	0.14	0.14	0.88	0.09
SMCR8	SMCR8;FLJ34716;FLJ60657	Smith-Magenis syndrome chromosome region, candidate 8	0.6	0.51	0.89	0.43
LCN1	LCN1;MGC71975;PMFA;TLC;TP;VEGP	lipocalin 1 (tear prealbumin)	0.6	0.54	0.89	0.36
IMP4	IMP4;BXDC4;MGC19606	IMP4, U3 small nucleolar ribonucleoprotein, homolog (yeast)	0.6	0.55	0.89	0.38
SMAD2	SMAD2;JV18;JV18-1;MADH2;MADR2;MGC22139;MGC34440;hMAD-2;hSMAD2	SMAD family member 2	0.6	0.58	0.89	0.41
ADAMTSL2	ADAMTSL2;FLJ45164;GPHYSD1;KIAA0605	ADAMTS-like 2	0.57	0.46	0.89	0.35
FAM75D1	FAM75D1;FLJ46321	family with sequence similarity 75, member D1	0.57	0.5	0.89	0.37
DCHS1	DCHS1;CDH25;CDHR6;FIB1;FLJ11790;KIAA1773;PCDH16	dachsous 1 (Drosophila)	0.56	0.55	0.89	0.37
MRPS15	MRPS15;FLJ11564;MPR-S15;RPMS15;S15mt	mitochondrial ribosomal protein S15	0.55	0.5	0.89	0.36
LCA10	LCA10;LCAP	lung carcinoma-associated protein 10	0.55	0.49	0.89	0.35
MEGF6	MEGF6;EGFL3;KIAA0815	multiple EGF-like-domains 6	0.54	0.52	0.89	0.36
FERMT1	FERMT1;C20orf42;DTGCU2;FLJ20116;FLJ23423;KIND1;UNC112A;URP1	fermitin family member 1	0.52	0.46	0.89	0.32
STON2	STON2;STN2;STNB;STNB2	stonin 2	0.5	0.44	0.89	0.33
FIP1L1	FIP1L1;DKFZp586K0717;FLJ33619;Rhe;hFip1	FIP1 like 1 (S. cerevisiae)	0.5	0.46	0.89	0.35
NAA10	NAA10;ARD1;ARD1A;DXS707;MGC71248;NATD;TE2	N(alpha)-acetyltransferase 10, NatA catalytic subunit	0.48	0.46	0.89	0.3
SUN5	SUN5;MGC33594;SPAG4L;TSARG4;dJ726C3.1	Sad1 and UNC84 domain containing 5	0.48	0.42	0.89	0.35
FLJ25791	FLJ25791	ENTREZ STATUS: REMAPPED TO GENE ID: 221264	0.48	0.42	0.89	0.3
KLF11	KLF11;FKLF;FKLF1;MODY7;TIEG2;Tieg3	Kruppel-like factor 11	0.43	0.42	0.89	0.31
MAU2	MAU2;KIAA0892;MAU2L;MGC75361;SCC4;mau-2	MAU2 chromatid cohesion factor homolog (C. elegans)	0.41	0.36	0.89	0.27
XAB2	XAB2;DKFZp762C1015;HCNP;HCRN;NTC90;SYF1	XPA binding protein 2	0.23	0.22	0.89	0.16
ZNF611	ZNF611;MGC5384	zinc finger protein 611	0.6	0.56	0.9	0.46
ATP6AP1	ATP6AP1;16A;ATP6IP1;ATP6S1;Ac45;CF2;MGC129781;VATPS1;XAP-3;XAP3	ATPase, H+ transporting, lysosomal accessory protein 1	0.6	0.52	0.9	0.37
RPE65	RPE65;LCA2;RP20;mRPE65;rd12;sRPE65	retinal pigment epithelium-specific protein 65kDa	0.58	0.56	0.9	0.37
CYP1A2	CYP1A2;CP12;P3-450;P450(PA)	cytochrome P450, family 1, subfamily A, polypeptide 2	0.58	0.58	0.9	0.4
NTN1	NTN1;NTN1L	netrin 1	0.58	0.54	0.9	0.39
CCDC64	CCDC64;BICDR-1;FLJ26450;H_267D11.1	coiled-coil domain containing 64	0.57	0.45	0.9	0.35
MPP2	MPP2;DKFZp686A06252;DKFZp686J2189;DKFZp761D0712;DLG2	membrane protein, palmitoylated 2 (MAGUK p55 subfamily member 2)	0.56	0.5	0.9	0.42
MFSD10	MFSD10;TETRAN;TETTRAN	major facilitator superfamily domain containing 10	0.56	0.55	0.9	0.4
PXK	PXK;FLJ20335;MONaKA	PX domain containing serine/threonine kinase	0.56	0.54	0.9	0.38
SKIV2L2	SKIV2L2;Dob1;KIAA0052;MGC142069;Mtr4;fSAP118	superkiller viralicidic activity 2-like 2 (S. cerevisiae)	0.55	0.52	0.9	0.39
CTAG1B	CTAG1B;CT6.1;CTAG;CTAG1;CTAG1A;ESO1;LAGE-2;LAGE2B;NY-ESO-1	cancer/testis antigen 1B	0.55	0.48	0.9	0.37
CHST9	CHST9;FLJ42328;GALNAC4ST-2	carbohydrate (N-acetylgalactosamine 4-0) sulfotransferase 9	0.54	0.52	0.9	0.39
LOC201175	LOC201175	ENTREZ STATUS: REMAPPED TO GENE ID: 201176	0.54	0.48	0.9	0.36
SIK3	SIK3;FLJ12240;KIAA0999;QSK;SIK-3	SIK family kinase 3	0.54	0.49	0.9	0.4
TTC4	TTC4;DKFZp781B0622;FLJ41930;MGC5097	tetratricopeptide repeat domain 4	0.53	0.5	0.9	0.37
KIF1C	KIF1C;KIAA0706;LTXS1	kinesin family member 1C	0.53	0.51	0.9	0.34
MYL7	MYL7;MYL2A;MYLC2A	myosin, light chain 7, regulatory	0.52	0.48	0.9	0.37
BRAF	BRAF;B-RAF1;BRAF1;FLJ95109;MGC126806;MGC138284;NS7;RAFB1	v-raf murine sarcoma viral oncogene homolog B1	0.52	0.45	0.9	0.41
VAMP3	VAMP3;CEB	vesicle-associated membrane protein 3 (cellubrevin)	0.52	0.46	0.9	0.38
C14orf28	C14orf28;DRIP1;c14_5270	chromosome 14 open reading frame 28	0.5	0.46	0.9	0.31
PLA2G4D	PLA2G4D;cPLA2delta	phospholipase A2, group IVD (cytosolic)	0.5	0.44	0.9	0.38
TMEM63B	TMEM63B;C6orf110;DKFZp686N2275;RP3-421H19.2	transmembrane protein 63B	0.48	0.44	0.9	0.32
UGT1A7	UGT1A7;UDPGT;UDPGT 1-7;UGT-1G;UGT1-07;UGT1.7;UGT1G	UDP glucuronosyltransferase 1 family, polypeptide A7	0.44	0.36	0.9	0.32
RABGGTA	RABGGTA;PTAR3	Rab geranylgeranyltransferase, alpha subunit	0.42	0.4	0.9	0.26
LCE5A	LCE5A;LEP18;SPRL5A	late cornified envelope 5A	0.4	0.37	0.9	0.27
C1orf74	C1orf74;FLJ25078	chromosome 1 open reading frame 74	0.4	0.4	0.9	0.27
RPL21	RPL21;DKFZp686C06101;FLJ27458;L21;MGC104274;MGC104275;MGC71252	ribosomal protein L21	0.38	0.35	0.9	0.25
CFLAR	CFLAR;CASH;CASP8AP1;CLARP;Casper;FLAME;FLAME-1;FLAME1;FLIP;I-FLICE;MRIT;c-FLIP;c-FLIPL;c-FLIPR;c-FLIPS	CASP8 and FADD-like apoptosis regulator	0.24	0.2	0.9	0.15
TOX	TOX;KIAA0808;TOX1	thymocyte selection-associated high mobility group box	0.6	0.56	0.91	0.43
ERCC1	ERCC1;COFS4;RAD10;UV20	excision repair cross-complementing rodent repair deficiency, complementation group 1 (includes overlapping antisense sequence)	0.6	0.54	0.91	0.42
PFDN6	PFDN6;H2-KE2;HKE2;KE-2;MGC70744;PFD6	prefoldin subunit 6	0.6	0.55	0.91	0.42
TXNDC9	TXNDC9;APACD;PHLP3	thioredoxin domain containing 9	0.58	0.56	0.91	0.37
EPRS	EPRS;DKFZp313B047;EARS;GLUPRORS;PARS;QARS;QPRS	glutamyl-prolyl-tRNA synthetase	0.58	0.52	0.91	0.4
DDX12P	DDX12P;CHLR2;DDX12	DEAD/H (Asp-Glu-Ala-Asp/His) box polypeptide 12, pseudogene	0.58	0.52	0.91	0.4
BZRAP1	BZRAP1;DKFZp686F02123;KIAA0612;PRAX-1;PRAX1;RIM-BP1;RIMBP1	benzodiazapine receptor (peripheral) associated protein 1	0.57	0.54	0.91	0.38
TMEM80	TMEM80;FLJ38216;FLJ55823	transmembrane protein 80	0.57	0.47	0.91	0.37
ACTB	ACTB;PS1TP5BP1	actin, beta	0.57	0.53	0.91	0.41
NUMA1	NUMA1;NUMA	nuclear mitotic apparatus protein 1	0.54	0.54	0.91	0.4
SLX4	SLX4;BTBD12;FANCP;KIAA1784;KIAA1987;MUS312	SLX4 structure-specific endonuclease subunit homolog (S. cerevisiae)	0.53	0.44	0.91	0.36
UBE4A	UBE4A;E4;KIAA0126;MGC133315;UBOX2;UFD2	ubiquitination factor E4A	0.52	0.47	0.91	0.42
FAM111A	FAM111A;DKFZp686A06175;FLJ22794;KIAA1895	family with sequence similarity 111, member A	0.43	0.43	0.91	0.29
DNAH11	DNAH11;CILD7;DNAHBL;DNAHC11;DNHBL;DPL11;FLJ30095;FLJ37699	dynein, axonemal, heavy chain 11	0.6	0.54	0.92	0.42
ZNF141	ZNF141;D4S90;pHZ-44	zinc finger protein 141	0.6	0.53	0.92	0.43
PHF2	PHF2;CENP-35;GRC5;JHDM1E;KIAA0662;MGC176680	PHD finger protein 2	0.6	0.54	0.92	0.48
SIKE1	SIKE1;DKFZp686A0768;FLJ21168;SIKE	suppressor of IKBKE 1	0.59	0.57	0.92	0.41
ZNF583	ZNF583;FLJ31030;MGC133237	zinc finger protein 583	0.58	0.56	0.92	0.42
RHOBTB2	RHOBTB2;DBC2;KIAA0717	Rho-related BTB domain containing 2	0.57	0.53	0.92	0.35
IFIT2	IFIT2;G10P2;GARG-39;IFI-54;IFI-54K;IFI54;IFIT-2;ISG-54 K;ISG-54K;ISG54;cig42	interferon-induced protein with tetratricopeptide repeats 2	0.56	0.55	0.92	0.39
SFXN5	SFXN5;BBG-TCC;MGC120413;MGC120415	sideroflexin 5	0.56	0.49	0.92	0.38
EME1	EME1;FLJ31364;MMS4L;SLX2A	essential meiotic endonuclease 1 homolog 1 (S. pombe)	0.55	0.52	0.92	0.41
RGPD2	RGPD2;FLJ56975;NUP358;RANBP2L2;RGP2	RANBP2-like and GRIP domain containing 2	0.52	0.5	0.92	0.38
OAZ3	OAZ3;AZ3;OAZ-t;TISP15	ornithine decarboxylase antizyme 3	0.51	0.46	0.92	0.36
FAM123A	FAM123A;FLJ25477	family with sequence similarity 123A	0.5	0.48	0.92	0.35
CCT8	CCT8;C21orf112;Cctq;D21S246;KIAA0002;PRED71	chaperonin containing TCP1, subunit 8 (theta)	0.5	0.49	0.92	0.36
PPP1R15A	PPP1R15A;GADD34	protein phosphatase 1, regulatory subunit 15A	0.45	0.42	0.92	0.32
CDC5L	CDC5L;CDC5;CDC5-LIKE;CEF1;KIAA0432;PCDC5RP;dJ319D22.1	CDC5 cell division cycle 5-like (S. pombe)	0.45	0.44	0.92	0.34
C9orf163	C9orf163;FLJ36779;RP11-413M3.11	chromosome 9 open reading frame 163	0.44	0.4	0.92	0.29
RPL11	RPL11;DBA7;GIG34;L11	ribosomal protein L11	0.37	0.36	0.92	0.29
RPS15	RPS15;MGC111130;RIG;S15	ribosomal protein S15	0.34	0.32	0.92	0.25
AATK	AATK;AATYK;AATYK1;KIAA0641;LMR1;LMTK1;p35BP	apoptosis-associated tyrosine kinase	0.6	0.6	0.93	0.43
STS	STS;ARSC;ARSC1;ASC;ES;SSDD;XLI	steroid sulfatase (microsomal), isozyme S	0.6	0.53	0.93	0.4
MICA	MICA	ENTREZ STATUS: REMAPPED TO GENE ID: 100507436	0.6	0.54	0.93	0.44
DAP	DAP;MGC99796	death-associated protein	0.6	0.54	0.93	0.4
TOR2A	TOR2A;FLJ14771;MGC99558;TORP1	torsin family 2, member A	0.58	0.52	0.93	0.4
GDPD5	GDPD5;GDE2;PP1665	glycerophosphodiester phosphodiesterase domain containing 5	0.58	0.52	0.93	0.4
SAMD3	SAMD3;FLJ34563;MGC35163	sterile alpha motif domain containing 3	0.57	0.56	0.93	0.39
CRYBB3	CRYBB3;CATCN2;CRYB3;MGC125772;MGC125773;MGC125774	crystallin, beta B3	0.57	0.55	0.93	0.38
WIPI2	WIPI2;ATG18B;Atg21;DKFZp434J154;DKFZp686P02188;FLJ12979;FLJ14217;FLJ42984;WIPI-2	WD repeat domain, phosphoinositide interacting 2	0.57	0.51	0.93	0.39

ZNF226	ZNF226	zinc finger protein 226	0.56	0.54	0.93	0.41
GPR52	GPR52;MGC111751	G protein-coupled receptor 52	0.55	0.5	0.93	0.44
SYT11	SYT11;DKFZp781D015;KIAA0080;MGC10881;MGC17226;SYT12;sytXI	synaptotagmin XI	0.55	0.52	0.93	0.4
CTSB	CTSB;APPS;CPSB	cathepsin B	0.55	0.52	0.93	0.39
TGM4	TGM4;FLJ26776;TGP;hTGP	transglutaminase 4 (prostate)	0.54	0.53	0.93	0.35
C1QTNF6	C1QTNF6;CTRP6;ZACRP6	C1q and tumor necrosis factor related protein 6	0.54	0.54	0.93	0.4
ANAPC16	ANAPC16;APC16;C10orf104;CENP-27;FLJ33728;MSAG;bA570G20.3	anaphase promoting complex subunit 16	0.54	0.52	0.93	0.35
CCNB3	CCNB3	cyclin B3	0.53	0.5	0.93	0.38
TP53	TP53;FLJ92943;LFS1;P53;TRP53	tumor protein p53	0.53	0.5	0.93	0.33
C1QTNF8	C1QTNF8;CTRP8;UNQ5829	C1q and tumor necrosis factor related protein 8	0.52	0.49	0.93	0.34
ZNF276	ZNF276;CENP-Z;CENPZ;FLJ38685;FLJ77809;MGC111410;MGC126480;MGC45417;ZFP276;ZNF477	zinc finger protein 276	0.52	0.49	0.93	0.35
KRT80	KRT80;KB20	keratin 80	0.52	0.48	0.93	0.35
C1QC	C1QC;C1Q-C;C1QG;FLJ27103	complement component 1, q subcomponent, C chain	0.51	0.48	0.93	0.36
GAL3ST3	GAL3ST3;GAL3ST-3;GAL3ST2;MGC142112;MGC142114	galactose-3-O-sulfotransferase 3	0.51	0.46	0.93	0.36
ABCA2	ABCA2;ABC2;MGC129761	ATP-binding cassette, sub-family A (ABC1), member 2	0.51	0.46	0.93	0.34
CCNL2	CCNL2;ANIA-6B;CCNM;DKFZp761A1210;DKFZp762O195;HCLA-ISO;HLA-ISO;PCEE;SB138	cyclin L2	0.5	0.49	0.93	0.33
JSRP1	JSRP1;FLJ32416;JP-45	junctional sarcoplasmic reticulum protein 1	0.48	0.47	0.93	0.34
ZCCHC7	ZCCHC7;AIR1;FLJ22611;RP11-397D12.1	zinc finger, CCHC domain containing 7	0.45	0.4	0.93	0.31
TMEM117	TMEM117;DKFZp434K2435	transmembrane protein 117	0.45	0.44	0.93	0.31
GPS1	GPS1;COPS1;CSN1;MGC71287	G protein pathway suppressor 1	0.44	0.4	0.93	0.32
SF3B3	SF3B3;KIAA0017;RSE1;SAP130;SF3b130;STAF130	splicing factor 3b, subunit 3, 130kDa	0.44	0.42	0.93	0.33
MOK	MOK;RAGE;RAGE-1;RAGE1	MOK protein kinase	0.43	0.44	0.93	0.3
SPAG1	SPAG1;FLJ32920;HSD-3.8;SP75;TPIS	sperm associated antigen 1	0.42	0.38	0.93	0.29
GPI	GPI;AMF;DKFZp686C13233;GNPI;NLK;PGI;PHI;SA-36;SA36	glucose-6-phosphate isomerase	0.42	0.42	0.93	0.32
NEK1	NEK1;DKFZp686D06121;DKFZp686K12169;KIAA1901;MGC138800;NY-REN-55;SRPS2	NIMA (never in mitosis gene a)-related kinase 1	0.4	0.36	0.93	0.32
DAB2IP	DAB2IP;AF9Q34;AIP1;DIP1/2;FLJ39072;KIAA1743	DAB2 interacting protein	0.6	0.51	0.94	0.42
PIGW	PIGW;FLJ37433;Gwt1	phosphatidylinositol glycan anchor biosynthesis, class W	0.6	0.55	0.94	0.42
PIGM	PIGM;GPI-MT-I;MGC29896	phosphatidylinositol glycan anchor biosynthesis, class M	0.6	0.55	0.94	0.39
ADAP2	ADAP2;CENTA2;HSA272195;cent-b	ArfGAP with dual PH domains 2	0.6	0.52	0.94	0.43
ASTL	ASTL;MGC129843	astacin-like metallo-endopeptidase (M12 family)	0.59	0.55	0.94	0.41
GSG1	GSG1;MGC111023;MGC3146	germ cell associated 1	0.58	0.56	0.94	0.42
C1orf182	C1orf182;MGC26877;RP11-443G18.5;SIP;SSTK-IP	chromosome 1 open reading frame 182	0.58	0.54	0.94	0.4
NAALADL1	NAALADL1;I100;NAALADASEL	N-acetylated alpha-linked acidic dipeptidase-like 1	0.58	0.54	0.94	0.41
METTL22	METTL22;C16orf68;FLJ12433;MGC2654	methyltransferase like 22	0.58	0.6	0.94	0.4
YARS	YARS;CMTDIC;TYRRS;YRS;YTS	tyrosyl-tRNA synthetase	0.57	0.55	0.94	0.37
F8A2	F8A2	coagulation factor VIII-associated 2	0.57	0.52	0.94	0.42
CYTH4	CYTH4;CYT4;DJ63G5.1;PSCD4	cytohesin 4	0.56	0.55	0.94	0.41
NOXA1	NOXA1;FLJ25475;MGC131800;NY-CO-31;SDCCAG31;p51NOX	NADPH oxidase activator 1	0.56	0.57	0.94	0.42
ZNF212	ZNF212;C2H2-150;MGC9707;ZNF182;ZNFC150	zinc finger protein 212	0.56	0.52	0.94	0.39
DHX36	DHX36;DDX36;G4R1;KIAA1488;MLEL1;RHAU	DEAH (Asp-Glu-Ala-His) box polypeptide 36	0.56	0.54	0.94	0.42
S100A14	S100A14;BCMP84;S100A15	S100 calcium binding protein A14	0.55	0.52	0.94	0.38
TLL2	TLL2;KIAA0932;MGC133312;MGC133313	tolloid-like 2	0.54	0.5	0.94	0.38
SNX22	SNX22;FLJ13952	sorting nexin 22	0.54	0.5	0.94	0.42
BMP10	BMP10;MGC126783	bone morphogenetic protein 10	0.53	0.45	0.94	0.36
CLEC10A	CLEC10A;CD301;CLECSF13;CLECSF14;HML;HML2;MGL	C-type lectin domain family 10, member A	0.52	0.52	0.94	0.42
GIGYF2	GIGYF2;DKFZp686I15154;DKFZp686J17223;FLJ23368;GYF2;KIAA0642;PARK11;PERQ2;PERQ3;TNRC15	GRB10 interacting GYF protein 2	0.52	0.48	0.94	0.36
HLA-G	HLA-G;MHC-G	major histocompatibility complex, class I, G	0.52	0.54	0.94	0.4
SYK	SYK;DKFZp313N1010;FLJ25043;FLJ37489	spleen tyrosine kinase	0.5	0.46	0.94	0.38
ARHGEF4	ARHGEF4;ASEF;ASEF1;GEF4;STM6	Rho guanine nucleotide exchange factor (GEF) 4	0.48	0.47	0.94	0.32
CCDC72	CCDC72	coiled-coil domain containing 72	0.46	0.42	0.94	0.32
NDUFV2	NDUFV2;CI-24k	NADH dehydrogenase (ubiquinone) flavoprotein 2, 24kDa	0.46	0.44	0.94	0.32
TCP11L2	TCP11L2;MGC40368	t-complex 11 (mouse)-like 2	0.43	0.44	0.94	0.29
PIPOX	PIPOX;LPIPOX	pipecolic acid oxidase	0.41	0.38	0.94	0.28
CCDC51	CCDC51;FLJ12436	coiled-coil domain containing 51	0.41	0.34	0.94	0.27
CLSPN	CLSPN;MGC131612;MGC131613;MGC131615	claspin	0.41	0.38	0.94	0.28
DHRS4	DHRS4;CR;FLJ11008;NRDR;PHCR;PSCD;SCAD-SRL;SDR-SRL;SDR25C1;SDR25C2	dehydrogenase/reductase (SDR family) member 4	0.3	0.26	0.94	0.21
ZFP82	ZFP82;KIAA1948;MGC45380;ZNF545	zinc finger protein 82 homolog (mouse)	0.6	0.59	0.95	0.45
CXCR3	CXCR3;CD182;CD183;CKR-L2;CMKAR3;GPR9;IP10-R;Mig-R;MigR	chemokine (C-X-C motif) receptor 3	0.6	0.56	0.95	0.5
CHN1	CHN1;ARHGAP2;CHN;DURS2;RHOGAP2	chimerin (chimaerin) 1	0.6	0.55	0.95	0.39
SYF2	SYF2;CBPIN;DKFZp564O2082;NTC31;P29;fSAP29	SYF2 homolog, RNA splicing factor (S. cerevisiae)	0.6	0.62	0.95	0.42
KIF3B	KIF3B;HH0048;KIAA0359	kinesin family member 3B	0.6	0.6	0.95	0.46
SLC5A1	SLC5A1;D22S675;NAGT;SGLT1	solute carrier family 5 (sodium/glucose cotransporter), member 1	0.58	0.56	0.95	0.45
KCNK1	KCNK1;DPK;HOHO;K2P1;K2p1.1;KCNO1;TWIK-1;TWIK1	potassium channel, subfamily K, member 1	0.58	0.58	0.95	0.45
ID1	ID1;ID;bHLHb24	inhibitor of DNA binding 1, dominant negative helix-loop-helix protein	0.58	0.56	0.95	0.44
C17orf53	C17orf53;FLJ11594;MGC3130	chromosome 17 open reading frame 53	0.57	0.53	0.95	0.4
SNAPC5	SNAPC5;SNAP19	small nuclear RNA activating complex, polypeptide 5, 19kDa	0.57	0.56	0.95	0.43
HES1	HES1;FLJ20408;HES-1;HHL;HRY;bHLHb39	hairy and enhancer of split 1, (Drosophila)	0.57	0.59	0.95	0.44
FAM103A1	FAM103A1;C15orf18;HsT19360;MGC102778;MGC2560	family with sequence similarity 103, member A1	0.56	0.5	0.95	0.41
LEPREL1	LEPREL1;FLJ10718;MCVD;MLAT4;P3H2	leprecan-like 1	0.56	0.6	0.95	0.41
SERPINA10	SERPINA10;PZI;ZPI	serpin peptidase inhibitor, clade A (alpha-1 antiproteinase, antitrypsin), member 10	0.55	0.56	0.95	0.39
HLX	HLX;HB24;HLX1	H2.0-like homeobox	0.55	0.56	0.95	0.42
CREG1	CREG1;CREG	cellular repressor of E1A-stimulated genes 1	0.55	0.52	0.95	0.44
CHCHD5	CHCHD5;C2orf9;FLJ39671;MGC11104	coiled-coil-helix-coiled-coil-helix domain containing 5	0.54	0.52	0.95	0.4
CHD9	CHD9;AD013;CReMM;KISH2;PRIC320	chromodomain helicase DNA binding protein 9	0.54	0.57	0.95	0.4
PBX1	PBX1;DKFZp686B09108;MGC126627	pre-B-cell leukemia homeobox 1	0.54	0.56	0.95	0.42
C12orf11	C12orf11;FLJ10630;FLJ10637;NET48	chromosome 12 open reading frame 11	0.54	0.56	0.95	0.37
SNPH	SNPH;KIAA0374;MGC46096;bA314N13.5	syntaphilin	0.53	0.46	0.95	0.36
C19orf29	C19orf29;FLJ17482;FLJ59622;NY-REN-24;cactin;fSAPc	chromosome 19 open reading frame 29	0.53	0.5	0.95	0.37
CLCF1	CLCF1;BSF-3;BSF3;CISS2;CLC;NNT-1;NNT1;NR6	cardiotrophin-like cytokine factor 1	0.53	0.56	0.95	0.39
CDK2AP1	CDK2AP1;DOC1;DORC1;ST19;doc-1;p12DOC-1	cyclin-dependent kinase 2 associated protein 1	0.53	0.52	0.95	0.41
ATP6V1G2	ATP6V1G2;ATP6G;ATP6G2;NG38;VMA10	ATPase, H+ transporting, lysosomal 13kDa, V1 subunit G2	0.52	0.48	0.95	0.42
CEP41	CEP41;DKFZp762H1311;TSGA14	centrosomal protein 41kDa	0.52	0.52	0.95	0.4
MRPL30	MRPL30;FLJ44438;L28MT;L30MT;MGC24095;MGC3314;MRP-L28;MRP-L30;MRPL28;MRPL28M;RPML28	mitochondrial ribosomal protein L30	0.51	0.49	0.95	0.34
RPL15	RPL15;EC45;FLJ26304;L15;MGC88603;RPL10;RPLY10;RPYL10	ribosomal protein L15	0.5	0.5	0.95	0.37
SCRT2	SCRT2;FLJ43353;ZNF898B	scratch homolog 2, zinc finger protein (Drosophila)	0.48	0.47	0.95	0.36
PKLR	PKLR;PK1;PKL;PKR;PKRL;RPK	pyruvate kinase, liver and RBC	0.48	0.5	0.95	0.35
HAGHL	HAGHL;MGC2605	hydroxyacylglutathione hydrolase-like	0.48	0.5	0.95	0.35
C9orf142	C9orf142	chromosome 9 open reading frame 142	0.46	0.44	0.95	0.32
STK11	STK11;LKB1;PJS;hLKB1	serine/threonine kinase 11	0.45	0.43	0.95	0.35
LIF	LIF;CDF;DIA;HILDA	leukemia inhibitory factor (cholinergic differentiation factor)	0.19	0.2	0.95	0.15
ALDOA	ALDOA;ALDA;GSD12;MGC10942;MGC17716;MGC17767	aldolase A, fructose-bisphosphate	0.16	0.15	0.95	0.12
IKZF2	IKZF2;HELIOS;MGC34330;ZNF1A2;ZNFN1A2	IKAROS family zinc finger 2 (Helios)	0.6	0.62	0.96	0.41
C15orf56	C15orf56;FLJ38596	chromosome 15 open reading frame 56	0.6	0.56	0.96	0.44
ITGB5	ITGB5;FLJ26658	integrin, beta 5	0.6	0.64	0.96	0.44
CST1	CST1	cystatin SN	0.58	0.57	0.96	0.43
TCEB3B	TCEB3B;ELOA2;HsT832;MGC119351;TCEB3L	transcription elongation factor B polypeptide 3B (elongin A2)	0.58	0.56	0.96	0.38
PROKR1	PROKR1;GPR73;GPR73a;PKR1;ZAQ	prokineticin receptor 1	0.58	0.54	0.96	0.49
GPX7	GPX7;CL683;FLJ14777;GPX6;GPx-7;GSHPx-7;NPGPx	glutathione peroxidase 7	0.58	0.53	0.96	0.42
PITPNM3	PITPNM3;CORD5;MGC157740;MGC157741;NIR1;RDGBA3	PITPNM family member 3	0.58	0.6	0.96	0.45
DNA2	DNA2;DNA2L;FLJ10063;KIAA0083;MGC133297	DNA replication helicase 2 homolog (yeast)	0.58	0.52	0.96	0.46
DNM1L	DNM1L;DLP1;DRP1;DVLP;DYMPLE;DYNIV-11;FLJ41912;HDYNIV;VPS1	dynamin 1-like	0.58	0.52	0.96	0.41
PCDHB9	PCDHB9;MGC119555;MGC119556;PCDH-BETA9;PCDH3H	protocadherin beta 9	0.57	0.55	0.96	0.38
NOLC1	NOLC1;KIAA0035;NOPP130;NOPP140;NS5ATP13;P130	nucleolar and coiled-body phosphoprotein 1	0.57	0.54	0.96	0.45
BRI3	BRI3;I3	brain protein I3	0.56	0.55	0.96	0.42
PIEZO1	PIEZO1;FAM38A;KIAA0233;Mib	piezo-type mechanosensitive ion channel component 1	0.56	0.56	0.96	0.42
NOP14	NOP14;C4orf9;NOL14;RES425;UTP2	NOP14 nucleolar protein homolog (yeast)	0.56	0.56	0.96	0.4
MOV10L1	MOV10L1;DJ402G11.8;DKFZp434B0717;FLJ33421	Mov10l1, Moloney leukemia virus 10-like 1, homolog (mouse)	0.55	0.58	0.96	0.4
GPR39	GPR39;MGC149541	G protein-coupled receptor 39	0.55	0.51	0.96	0.46
GIPC2	GIPC2;FLJ20075;SEMCAP-2;SEMCAP2	GIPC PDZ domain containing family, member 2	0.54	0.54	0.96	0.36
ORAI1	ORAI1;CRACM1;FLJ14466;ORAT1;TMEM142A	ORAI calcium release-activated calcium modulator 1	0.54	0.55	0.96	0.43
CHAF1B	CHAF1B;CAF-1;CAF-IP60;CAF1;CAF1A;CAF1P60;MPHOSPH7;MPP7	chromatin assembly factor 1, subunit B (p60)	0.54	0.54	0.96	0.4
VWC2L	VWC2L	von Willebrand factor C domain containing protein 2-like	0.54	0.53	0.96	0.41
C20orf134	C20orf134;dJ63M2.2	chromosome 20 open reading frame 134	0.52	0.48	0.96	0.38
ANKRD57	ANKRD57;C2orf26	ankyrin repeat domain 57	0.52	0.5	0.96	0.36
SRP68	SRP68	signal recognition particle 68kDa	0.52	0.48	0.96	0.37
RBM12	RBM12;HRIHFB2091;KIAA0765;SWAN	RNA binding motif protein 12	0.5	0.5	0.96	0.34
SNAPC4	SNAPC4;FLJ13451;PTFalpha;SNAP190	small nuclear RNA activating complex, polypeptide 4, 190kDa	0.48	0.48	0.96	0.39
RRP9	RRP9;RNU3IP2;U3-55K	ribosomal RNA processing 9, small subunit (SSU) processome component, homolog (yeast)	0.48	0.5	0.96	0.33
PSMB5	PSMB5;LMPX;MB1;MGC104214;X	proteasome (prosome, macropain) subunit, beta type, 5	0.48	0.42	0.96	0.32
NUBPL	NUBPL;C14orf127;FLJ12660;IND1;huInd1	nucleotide binding protein-like	0.47	0.5	0.96	0.33
KCNRG	KCNRG;DLTET	potassium channel regulator	0.46	0.47	0.96	0.37
WRAP73	WRAP73;FLJ20430;MGC99569;WDR8	WD repeat containing, antisense to TP73	0.46	0.45	0.96	0.32
CAMK2N1	CAMK2N1;MGC22256;PRO1489;RP11-401M16.1	calcium/calmodulin-dependent protein kinase II inhibitor 1	0.44	0.42	0.96	0.34
SLC35A5	SLC35A5;DKFZp434E102;FLJ11130;FLJ20730;FLJ25973	solute carrier family 35, member A5	0.44	0.41	0.96	0.31
SERPINF2	SERPINF2;A2AP;AAP;ALPHA-2-PI;API;PLI	serpin peptidase inhibitor, clade F (alpha-2 antiplasmin, pigment epithelium derived factor), member 2	0.43	0.42	0.96	0.28
NUDT11	NUDT11;APS1;ASP1;DIPP3b;DIPP3beta;FLJ10628;hDIPP3beta	nudix (nucleoside diphosphate linked moiety X)-type motif 11	0.42	0.4	0.96	0.31
LYPLA2	LYPLA2;APT-2;DJ886K2.4	lysophospholipase II	0.4	0.38	0.96	0.26
TRPM1	TRPM1;CSNB1C;LTRPC1;MLSN1	transient receptor potential cation channel, subfamily M, member 1	0.38	0.38	0.96	0.29
MANEAL	MANEAL;FLJ31434;MGC78681;RP11-109P14.3	mannosidase, endo-alpha-like	0.38	0.36	0.96	0.26

COL20A1	COL20A1;KIAA1510;bA261N11.4	collagen, type XX, alpha 1	0.34	0.34	0.96	0.25
CDH15	CDH15;CDH14;CDH3;CDHM;MCAD;MRD3	cadherin 15, type 1, M-cadherin (myotubule)	0.6	0.62	0.97	0.5
BATF2	BATF2;MGC20410;SARI	basic leucine zipper transcription factor, ATF-like 2	0.6	0.56	0.97	0.43
SPTLC3	SPTLC3;C20orf38;FLJ11112;FLJ90790;LCB2B;LCB3;SPT3;SPTLC2L;dJ718P11;dJ718P11.1;hLCB2b	serine palmitoyltransferase, long chain base subunit 3	0.6	0.58	0.97	0.39
LTBP2	LTBP2;C14orf141;GLC3D;LTBP3;MSTP031	latent transforming growth factor beta binding protein 2	0.6	0.6	0.97	0.46
SDAD1	SDAD1;DKFZp686E22207;FLJ10498	SDA1 domain containing 1	0.6	0.6	0.97	0.41
POU5F1	POU5F1;MGC22487;OCT3;OCT4;OTF-3;OTF3;OTF4;Oct-3;Oct-4	POU class 5 homeobox 1	0.59	0.64	0.97	0.44
TMEM8B	TMEM8B;C9orf127;MGC120460;NAG-5;NGX6;RP11-112J3.10	transmembrane protein 8B	0.59	0.6	0.97	0.41
APOBEC3A	APOBEC3A;ARP3;PHRBN;bK150C2.1	apolipoprotein B mRNA editing enzyme, catalytic polypeptide-like 3A	0.58	0.62	0.97	0.44
KCNMB3	KCNMB3;BKBETA3;HBETA3;KCNMB2;KCNMBL;SLOBETA3	potassium large conductance calcium-activated channel, subfamily M beta member 3	0.57	0.58	0.97	0.45
ANKRD32	ANKRD32;BRCTD1;BRCTx;DKFZp564C0469;DKFZp761C121	ankyrin repeat domain 32	0.57	0.58	0.97	0.39
IRX4	IRX4;IRXA3;MGC131996	iroquois homeobox 4	0.57	0.6	0.97	0.42
INTS6	INTS6;DBI-1;DDX26;DDX26A;DICE1;DKFZp434B105;HDB;INT6;Notchl2	integrator complex subunit 6	0.57	0.54	0.97	0.46
SH2D3C	SH2D3C;CHAT;FLJ39664;NSP3;PRO34088	SH2 domain containing 3C	0.57	0.55	0.97	0.38
NNMT	NNMT	nicotinamide N-methyltransferase	0.57	0.54	0.97	0.39
AIFM1	AIFM1;AIF;COXPD6;MGC111425;PDCD8	apoptosis-inducing factor, mitochondrion-associated, 1	0.57	0.54	0.97	0.47
ILF3	ILF3;CBTF;DRBF;DRBP76;MMP4;MPHOSPH4;MPP4;NF-AT-90;NF110;NF110b;NF90;NF90a;NF90b;NFAR;NFAR-1;NFAR2;TCP110;TCP80	interleukin enhancer binding factor 3, 90kDa	0.57	0.56	0.97	0.41
EIF3D	EIF3D;EIF3S7;MGC126526;MGC17258;eIF3-p66;eIF3-zeta	eukaryotic translation initiation factor 3, subunit D	0.55	0.59	0.97	0.41
METTL21D	METTL21D;C14orf138;FLJ13920	methyltransferase like 21D	0.55	0.55	0.97	0.37
CCDC105	CCDC105;FLJ40365	coiled-coil domain containing 105	0.54	0.52	0.97	0.37
ACD	ACD;PIP1;PTOP;TINT1;TPP1	adrenocortical dysplasia homolog (mouse)	0.54	0.55	0.97	0.4
OTOS	OTOS;OTOSP	otospiralin	0.54	0.51	0.97	0.41
SLC35A2	SLC35A2;UGALT;UGAT;UGT;UGT1;UGT2;UGTL	solute carrier family 35 (UDP-galactose transporter), member A2	0.53	0.56	0.97	0.39
TXNL4A	TXNL4A;DIB1;DIM1;HsT161;TXNL4;U5-15kD	thioredoxin-like 4A	0.53	0.48	0.97	0.42
PSME2	PSME2;PA28B;PA28beta;REGbeta	proteasome (prosome, macropain) activator subunit 2 (PA28 beta)	0.53	0.52	0.97	0.38
L2HGDH	L2HGDH;C14orf160;FLJ12618	L-2-hydroxyglutarate dehydrogenase	0.52	0.5	0.97	0.39
SLC25A26	SLC25A26;DKFZp434E079;FLJ77340;SAMC	solute carrier family 25, member 26	0.5	0.5	0.97	0.33
RFWD3	RFWD3;FLJ10520;RNF201	ring finger and WD repeat domain 3	0.5	0.48	0.97	0.43
OR1N1	OR1N1;OR1-26;OR1N3;OR9-22	olfactory receptor, family 1, subfamily N, member 1	0.48	0.49	0.97	0.4
DEFB124	DEFB124;DEFB-24	defensin, beta 124	0.48	0.48	0.97	0.36
EN2	EN2	engrailed homeobox 2	0.46	0.5	0.97	0.34
CDKL2	CDKL2;KKIAMRE;P56	cyclin-dependent kinase-like 2 (CDC2-related kinase)	0.45	0.42	0.97	0.38
SLC35B3	SLC35B3;C6orf196;PAPST2	solute carrier family 35, member B3	0.45	0.48	0.97	0.35
TCAP	TCAP;CMD1N;LGMD2G;T-cap;TELE;telethonin	titin-cap (telethonin)	0.44	0.45	0.97	0.34
TGDS	TGDS;SDR2E1;TDPGD	TDP-glucose 4,6-dehydratase	0.44	0.42	0.97	0.36
MAFG	MAFG;MGC13090;MGC20149;hMAF	v-maf musculoaponeurotic fibrosarcoma oncogene homolog G (avian)	0.42	0.38	0.97	0.3
PTCRA	PTCRA;PT-ALPHA;PTA	pre T-cell antigen receptor alpha	0.39	0.4	0.97	0.27
ADAP1	ADAP1;CENTA1;GCS1L;p42IP4	ArfGAP with dual PH domains 1	0.38	0.38	0.97	0.29
ZNF205	ZNF205;ZNF210;Zfp13	zinc finger protein 205	0.35	0.34	0.97	0.27
OR6F1	OR6F1;OR1-34;OR1-38;OST731	olfactory receptor, family 6, subfamily F, member 1	0.6	0.64	0.98	0.47
RARA	RARA;NR1B1;RAR	retinoic acid receptor, alpha	0.6	0.57	0.98	0.43
TM2D2	TM2D2;BLP1;MGC125813;MGC125814	TM2 domain containing 2	0.6	0.6	0.98	0.45
NTN5	NTN5	netrin 5	0.6	0.6	0.98	0.46
SYT16	SYT16;CHR14SYT;SYT14L;Strep14;syt14r;yt14r	synaptotagmin XVI	0.59	0.57	0.98	0.41
TMBIM4	TMBIM4;FLJ45352;GAAP;LFG4;S1R;ZPRO	transmembrane BAX inhibitor motif containing 4	0.59	0.6	0.98	0.45
SCN10A	SCN10A;Nav1.8;PN3;SNS;hPN3	sodium channel, voltage-gated, type X, alpha subunit	0.58	0.61	0.98	0.47
CATSPER3	CATSPER3;CACRC;MGC126741	cation channel, sperm associated 3	0.58	0.57	0.98	0.45
TLN2	TLN2;DKFZp451B1011;DKFZp686I0976;DKFZp686K0979;ILWEQ;KIAA0320	talin 2	0.58	0.56	0.98	0.42
C20orf27	C20orf27;FLJ20550	chromosome 20 open reading frame 27	0.58	0.55	0.98	0.4
SLC2A11	SLC2A11;GLUT10;GLUT11;MGC118830;MGC118833;SLC2A11-a;SLC2A11-c	solute carrier family 2 (facilitated glucose transporter), member 11	0.58	0.6	0.98	0.45
EAF1	EAF1	ELL associated factor 1	0.58	0.55	0.98	0.46
NOP2	NOP2;MGC117384;MGC149287;MGC149288;NOL1;NOP120;NSUN1;p120	NOP2 nucleolar protein homolog (yeast)	0.58	0.56	0.98	0.41
ABCA3	ABCA3;ABC-C;ABC3;EST111653;LBM180;MGC166979;MGC72201;SMDP3	ATP-binding cassette, sub-family A (ABC1), member 3	0.57	0.57	0.98	0.45
MLEC	MLEC;KIAA0152	malectin	0.56	0.56	0.98	0.38
FAM104B	FAM104B;CXorf44;FLJ18375;FLJ20434	family with sequence similarity 104, member B	0.55	0.52	0.98	0.4
POU4F1	POU4F1;BRN3A;FLJ13449;Oct-T1;RDC-1	POU class 4 homeobox 1	0.55	0.6	0.98	0.41
PPP1R9B	PPP1R9B;FLJ30345;PPP1R6;PPP1R9;SPINO;Spn	protein phosphatase 1, regulatory subunit 9B	0.55	0.53	0.98	0.45
ORAI1	ORAI1;CRACM1;FLJ14466;ORAT1;TMEM142A	ORAI calcium release-activated calcium modulator 1	0.55	0.55	0.98	0.43
SIX2	SIX2	SIX homeobox 2	0.55	0.6	0.98	0.41
ZNF761	ZNF761;FLJ16231;FLJ35333;KIAA2033;MGC87789;ZNF468	zinc finger protein 761	0.55	0.59	0.98	0.39
HIF3A	HIF3A;HIF-3A;IPAS;MOP7;PASD7;bHLHe17	hypoxia inducible factor 3, alpha subunit	0.54	0.53	0.98	0.37
POU6F2	POU6F2;RPF-1;WT5;WTSL	POU class 6 homeobox 2	0.53	0.53	0.98	0.38
ATP7A	ATP7A;DSMAX;FLJ17790;MK;MNK;SMAX3	ATPase, Cu++ transporting, alpha polypeptide	0.53	0.52	0.98	0.38
RASSF1	RASSF1;123F2;NORE2A;RASSF1A;RDA32;REH3P21	Ras association (RalGDS/AF-6) domain family member 1	0.52	0.55	0.98	0.41
GSDMB	GSDMB;GSDML;PRO2521	gasdermin B	0.5	0.48	0.98	0.36
MTX1	MTX1;MTX;MTXN	metaxin 1	0.49	0.44	0.98	0.39
HSF4	HSF4;CTM	heat shock transcription factor 4	0.48	0.46	0.98	0.4
ZNF207	ZNF207;DKFZp761N202	zinc finger protein 207	0.47	0.48	0.98	0.36
PRRT3	PRRT3;FLJ33674;MGC105134;MGC33990	proline-rich transmembrane protein 3	0.46	0.42	0.98	0.34
NNAT	NNAT;MGC1439;Peg5	neuronatin	0.46	0.44	0.98	0.37
CDK5RAP2	CDK5RAP2;C48;Cep215;DKFZp686B1070;DKFZp686D1070;KIAA1633;MCPH3	CDK5 regulatory subunit associated protein 2	0.46	0.44	0.98	0.33
ACOT4	ACOT4;PTE-Ib;PTE1B;PTE2B	acyl-CoA thioesterase 4	0.43	0.47	0.98	0.32
RPLP2	RPLP2;D11S2243E;LP2;MGC71408;P2;RPP2	ribosomal protein, large, P2	0.42	0.4	0.98	0.32
SLBP	SLBP;HBP	stem-loop binding protein	0.41	0.39	0.98	0.28
PSMD10	PSMD10;dJ889N15.2;p28;p28(GANK)	proteasome (prosome, macropain) 26S subunit, non-ATPase, 10	0.4	0.43	0.98	0.31
SS18L2	SS18L2;KIAA-iso	synovial sarcoma translocation gene on chromosome 18-like 2	0.39	0.38	0.98	0.26
ZIC1	ZIC1;ZIC;ZNF201	Zic family member 1	0.38	0.38	0.98	0.25
TBC1D20	TBC1D20;C20orf140;FLJ45119	TBC1 domain family, member 20	0.36	0.37	0.98	0.3
LOC652433	LOC652433	ENTREZ STATUS: REMAPPED TO GENE ID: 646754	0.29	0.28	0.98	0.25
RAD51	RAD51;BRCC5;HRAD51;HsRad51;HsT16930;RAD51A;RECA	RAD51 homolog (S. cerevisiae)	0.28	0.3	0.98	0.22
OXGR1	OXGR1;GPR80;GPR99;MGC119206;MGC119207;MGC119208;P2RY15;P2Y15	oxoglutarate (alpha-ketoglutarate) receptor 1	0.6	0.57	0.99	0.52
HPSE2	HPSE2;FLJ11684;FLJ44022;HPA2;HPR2;MGC133234;UFS	heparanase 2	0.6	0.57	0.99	0.43
CEBPE	CEBPE;C/EBP-epsilon;CRP1	CCAAT/enhancer binding protein (C/EBP), epsilon	0.6	0.6	0.99	0.4
FAM101A	FAM101A;FLJ44614	family with sequence similarity 101, member A	0.6	0.53	0.99	0.42
IQGAP3	IQGAP3;MGC10170;MGC10831;MGC1947	IQ motif containing GTPase activating protein 3	0.6	0.62	0.99	0.49
NARS2	NARS2;FLJ23441;SLM5;asnRS	asparaginyl-tRNA synthetase 2, mitochondrial (putative)	0.6	0.56	0.99	0.46
C2orf71	C2orf71;FLJ34931	chromosome 2 open reading frame 71	0.58	0.62	0.99	0.43
CCDC53	CCDC53;CGI-116	coiled-coil domain containing 53	0.58	0.63	0.99	0.43
PLSCR3	PLSCR3	phospholipid scramblase 3	0.58	0.61	0.99	0.46
HSDL2	HSDL2;C9orf99;FLJ25855;MGC10940;SDR13C1	hydroxysteroid dehydrogenase like 2	0.58	0.57	0.99	0.39
CALML5	CALML5;CLSP	calmodulin-like 5	0.57	0.62	0.99	0.45
BARX1	BARX1	BARX homeobox 1	0.57	0.6	0.99	0.45
KBTBD4	KBTBD4;BKLHD4;FLJ10450;HSPC252	kelch repeat and BTB (POZ) domain containing 4	0.56	0.56	0.99	0.45
DNAJC1	DNAJC1;DNAJL1;ERdj1;HTJ1;MGC131954;MTJ1	DnaJ (Hsp40) homolog, subfamily C, member 1	0.56	0.57	0.99	0.43
POLR2H	POLR2H;RPABC3;RPB17;RPB8;hsRPB8	polymerase (RNA) II (DNA directed) polypeptide H	0.56	0.62	0.99	0.43
MRPL37	MRPL37;L37mt;MGC878;MRP-L2;MRP-L37;MRPL2;RPML2	mitochondrial ribosomal protein L37	0.56	0.58	0.99	0.44
PPEF2	PPEF2;PPP7CB	protein phosphatase, EF-hand calcium binding domain 2	0.54	0.53	0.99	0.41
KRTAP10-7	KRTAP10-7;KAP10.7;KAP18.7;KRTAP18-7	keratin associated protein 10-7	0.54	0.58	0.99	0.4
SSTR5	SSTR5;SS-5-R	somatostatin receptor 5	0.54	0.49	0.99	0.37
PDGFA	PDGFA;PDGF-A;PDGF1	platelet-derived growth factor alpha polypeptide	0.53	0.51	0.99	0.44
ZNF574	ZNF574;FLJ22059;FP972	zinc finger protein 574	0.53	0.52	0.99	0.41
EPHB6	EPHB6;HEP;MGC129910;MGC129911	EPH receptor B6	0.52	0.5	0.99	0.42
TGFB3	TGFB3;ARVD;FLJ16571;TGF-beta3	transforming growth factor, beta 3	0.52	0.56	0.99	0.39
FAM120B	FAM120B;CCPG;FLJ55614;KIAA0183;KIAA1838;PGCC1;dJ894D12.1	family with sequence similarity 120B	0.51	0.48	0.99	0.39
TCTEX1D1	TCTEX1D1;FLJ40873;MGC125768;RP11-266I14.2	Tctex1 domain containing 1	0.5	0.52	0.99	0.37
CSRP2BP	CSRP2BP;ATAC2;CRP2BP;KAT14;MGC15388;PRO1194;dJ717M23.1	CSRP2 binding protein	0.5	0.52	0.99	0.36
CXorf40B	CXorf40B	chromosome X open reading frame 40B	0.5	0.48	0.99	0.39
STX6	STX6	syntaxin 6	0.49	0.5	0.99	0.38
HOXD11	HOXD11;HOX4;HOX4F	homeobox D11	0.48	0.48	0.99	0.38
UBXN11	UBXN11;COA-1;DKFZp686F04228;SOC;SOCI;UBXD5	UBX domain protein 11	0.48	0.51	0.99	0.34
ZBTB40	ZBTB40;KIAA0478;MGC133098;ZNF923	zinc finger and BTB domain containing 40	0.48	0.5	0.99	0.35
RDBP	RDBP;D6S45;NELF-E;RD;RDP	RD RNA binding protein	0.48	0.46	0.99	0.35
RPS14	RPS14;EMTB;S14	ribosomal protein S14	0.48	0.46	0.99	0.35
SCAF1	SCAF1;FLJ00034;SRA1	SR-related CTD-associated factor 1	0.47	0.48	0.99	0.36
UPK3A	UPK3A;MGC119178;UP3A;UPIII;UPIIIA;UPK3	uroplakin 3A	0.46	0.45	0.99	0.34
TBXA2R	TBXA2R;BDPLT13;TXA2-R	thromboxane A2 receptor	0.46	0.45	0.99	0.4
C1orf35	C1orf35;MGC4174;MMTAG2;hMMTAG2	chromosome 1 open reading frame 35	0.45	0.45	0.99	0.35
CCDC93	CCDC93;FLJ10996;FLJ25197;MGC13033	coiled-coil domain containing 93	0.45	0.49	0.99	0.33
CEP72	CEP72;FLJ10565;KIAA1519;MGC5307	centrosomal protein 72kDa	0.44	0.46	0.99	0.37
SELV	SELV	selenoprotein V	0.42	0.4	0.99	0.31
FKBP3	FKBP3;FKBP-25;FKBP-3;FKBP25;PPIase	FK506 binding protein 3, 25kDa	0.38	0.4	0.99	0.28
CCNB1	CCNB1;CCNB	cyclin B1	0.38	0.34	0.99	0.27
LGALS1	LGALS1;DKFZp686E23103;GAL1;GBP	lectin, galactoside-binding, soluble, 1	0.38	0.41	0.99	0.28
CSTB	CSTB;CST6;EPM1;PME;STFB	cystatin B (stefin B)	0.34	0.34	0.99	0.26
HES4	HES4;bHLHb42	hairy and enhancer of split 4 (Drosophila)	0.31	0.32	0.99	0.26
MTFMT	MTFMT;FMT1	mitochondrial methionyl-tRNA formyltransferase	0.29	0.3	0.99	0.23
PRND	PRND;DOPPEL;DPL;MGC41841;PrPLP;dJ1068H6.4	prion protein 2 (dublet)	0.6	0.64	1	0.45
ELK4	ELK4;DKFZp434N185;SAP1	ELK4, ETS-domain protein (SRF accessory protein 1)	0.6	0.6	1	0.44
MICAL2	MICAL2;DKFZp686H03148;DKFZp686H2469;KIAA0750;MICAL2PV1;MICAL2PV2	microtubule associated monoxygenase, calponin and LIM domain containing 2	0.6	0.56	1	0.5

C17orf101	C17orf101;FLJ22222	chromosome 17 open reading frame 101	0.6	0.58	1	0.43
CENPE	CENPE;CENP-E;KIF10;PPP1R61	centromere protein E, 312kDa	0.59	0.57	1	0.4
BCL3	BCL3;BCL4;D19S37	B-cell CLL/lymphoma 3	0.58	0.57	1	0.49
ABCE1	ABCE1;ABC38;OABP;RLI;RNASEL1;RNASELI;RNS4I	ATP-binding cassette, sub-family E (OABP), member 1	0.58	0.55	1	0.41
NONO	NONO;NMT55;NRB54;P54;P54NRB	non-POU domain containing, octamer-binding	0.58	0.61	1	0.41
NRIP2	NRIP2;DKFZp761G1913	nuclear receptor interacting protein 2	0.57	0.55	1	0.41
C19orf24	C19orf24;FLJ20640	chromosome 19 open reading frame 24	0.57	0.64	1	0.46
TAPT1	TAPT1;CMVFR;FLJ90013	transmembrane anterior posterior transformation 1	0.56	0.54	1	0.41
GNA12	GNA12;MGC104623;MGC99644;NNX3;RMP;gep	guanine nucleotide binding protein (G protein) alpha 12	0.56	0.57	1	0.45
GPD1L	GPD1L;GPD1-L;KIAA0089	glycerol-3-phosphate dehydrogenase 1-like	0.56	0.63	1	0.43
TSPYL1	TSPYL1;TSPYL	TSPY-like 1	0.56	0.57	1	0.41
TIMM17A	TIMM17A;TIM17;TIM17A	translocase of inner mitochondrial membrane 17 homolog A (yeast)	0.56	0.54	1	0.44
PRPF3	PRPF3;HPRP3;HPRP3P;PRP3;Prp3p;RP18	PRP3 pre-mRNA processing factor 3 homolog (S. cerevisiae)	0.55	0.6	1	0.44
ATP6V1C1	ATP6V1C1;ATP6C;ATP6D;FLJ20057;VATC;Vma5	ATPase, H+ transporting, lysosomal 42kDa, V1 subunit C1	0.55	0.55	1	0.41
CYCS	CYCS;CYC;HCS;THC4	cytochrome c, somatic	0.54	0.58	1	0.4
HIST1H1B	HIST1H1B;H1;H1.5;H1B;H1F5;MGC126630;MGC126632	histone cluster 1, H1b	0.54	0.55	1	0.42
MRC1	MRC1;CD206;CLEC13D;CLEC13DL;MGC181940;MMR;MRC1L1;bA541I19.1	mannose receptor, C type 1	0.53	0.51	1	0.38
GCA	GCA;GCL	grancalcin, EF-hand calcium binding protein	0.53	0.54	1	0.41
CRLF1	CRLF1;CISS;CISS1;CLF;CLF-1;NR6;zcytor5	cytokine receptor-like factor 1	0.53	0.52	1	0.42
HOXC5	HOXC5;CP11;HOX3;HOX3D	homeobox C5	0.52	0.52	1	0.4
NOP16	NOP16;HSPC111;HSPC185	NOP16 nucleolar protein homolog (yeast)	0.52	0.55	1	0.37
OLIG2	OLIG2;BHLHB1;OLIGO2;PRKCBP2;RACK17;bHLHe19	oligodendrocyte lineage transcription factor 2	0.51	0.51	1	0.38
PSIP1	PSIP1;DFS70;LEDGF;MGC74712;PAIP;PSIP2;p52;p75	PC4 and SFRS1 interacting protein 1	0.51	0.46	1	0.37
HPSE	HPSE;HPA;HPA1;HPR1;HPSE1;HSE1	heparanase	0.5	0.48	1	0.42
TTBK2	TTBK2;KIAA0847;SCA11;TTBK	tau tubulin kinase 2	0.5	0.48	1	0.4
RBPMS2	RBPMS2	RNA binding protein with multiple splicing 2	0.5	0.52	1	0.34
FBXO21	FBXO21;DKFZp434G058;FBX21;FLJ90233;KIAA0875;MGC26682	F-box protein 21	0.5	0.48	1	0.34
PRR22	PRR22;MGC24975	proline rich 22	0.5	0.52	1	0.35
MT3	MT3;GIF;GIFB;GRIF	metallothionein 3	0.49	0.5	1	0.4
COPZ2	COPZ2;MGC23008	coatomer protein complex, subunit zeta 2	0.48	0.52	1	0.38
TUBB2A	TUBB2A;TUBB;TUBB2;dJ40E16.7	tubulin, beta 2A class IIa	0.47	0.44	1	0.39
DERL2	DERL2;F-LAN-1;F-LANa;FLANa	Der1-like domain family, member 2	0.46	0.44	1	0.37
SMTN	SMTN;FLJ35168;FLJ35365;FLJ35620;FLJ38597	smoothelin	0.44	0.44	1	0.32
SORD	SORD;SORD1	sorbitol dehydrogenase	0.44	0.48	1	0.36
LOC389832	LOC389832	ENTREZ STATUS: REMAPPED TO GENE ID: 100288884	0.43	0.38	1	0.32
MLYCD	MLYCD;MCD;MGC59795	malonyl-CoA decarboxylase	0.43	0.48	1	0.33
ACTRT2	ACTRT2;ARPM2;ARPT2;Arp-T2;FLJ25424;HARPM2	actin-related protein T2	0.42	0.39	1	0.32
NDUFAF1	NDUFAF1;CGI65;CIA30	NADH dehydrogenase (ubiquinone) 1 alpha subcomplex, assembly factor 1	0.4	0.43	1	0.34
CD24	CD24	ENTREZ STATUS: REMAPPED TO GENE ID: 100133941	0.38	0.4	1	0.27
F7	F7	coagulation factor VII (serum prothrombin conversion accelerator)	0.36	0.34	1	0.25
SNRPD2	SNRPD2;SMD2;SNRPD1;Sm-D2	small nuclear ribonucleoprotein D2 polypeptide 16.5kDa	0.36	0.36	1	0.25
PLEKHJ1	PLEKHJ1;FLJ10297;GNRPX	pleckstrin homology domain containing, family J member 1	0.33	0.32	1	0.23
SF3B1	SF3B1;Hsh155;MDS;PRP10;PRPF10;SAP155;SF3b155	splicing factor 3b, subunit 1, 155kDa	0.32	0.34	1	0.24
LIN54	LIN54;CXCDC1;DKFZp686L1814;JC8.6;MGC129905;MIP120	lin-54 homolog (C. elegans)	0.28	0.31	1	0.2
EIF4A3	EIF4A3;DDX48;DKFZp686O16189;KIAA0111;MGC10862;NMP265;NUK34;eIF4AIII	eukaryotic translation initiation factor 4A3	0.26	0.26	1	0.2
ZBP1	ZBP1;C20orf183;DAI;DLM-1;DLM1	Z-DNA binding protein 1	0.6	0.66	1.01	0.49
KRTAP5-5	KRTAP5-5;KRTAP5-11;KRTAP5.5	keratin associated protein 5-5	0.6	0.62	1.01	0.42
C3orf64	C3orf64;AER61;FLJ13078;FLJ33770;FLJ41219;MGC34132	chromosome 3 open reading frame 64	0.6	0.66	1.01	0.44
ZBTB22	ZBTB22;BING1;ZBTB22A;ZNF297;ZNF297A;fru;fruitless	zinc finger and BTB domain containing 22	0.6	0.65	1.01	0.5
SKP1	SKP1;EMC19;MGC34403;OCP-II;OCP2;SKP1A;TCEB1L;p19A	S-phase kinase-associated protein 1	0.6	0.58	1.01	0.46
GRAMD3	GRAMD3;FLJ21313;NS3TP2	GRAM domain containing 3	0.6	0.64	1.01	0.52
BTK	BTK;AGMX1;AT;ATK;BPK;IMD1;MGC126261;MGC126262;PSCTK1;XLA	Bruton agammaglobulinemia tyrosine kinase	0.59	0.59	1.01	0.49
CAND2	CAND2;KIAA0667;TIP120B;Tp120b	cullin-associated and neddylation-dissociated 2 (putative)	0.58	0.54	1.01	0.41
NDNL2	NDNL2;HCA4;MAGEG1;MAGEL3;NSE3;NSMCE3	necdin-like 2	0.58	0.56	1.01	0.47
MAGT1	MAGT1;DKFZp564K142;FLJ14726;IAP;MGC64926;MRX95;OST3B;PRO0756;XMEN;bA217H1.1	magnesium transporter 1	0.58	0.6	1.01	0.45
DIO2	DIO2;5DII;D2;DIOII;SelY;TXDI2	deiodinase, iodothyronine, type II	0.57	0.58	1.01	0.39
COL17A1	COL17A1;BA16H23.2;BP180;BPA-2;BPAG2;FLJ60881;KIAA0204;LAD-1	collagen, type XVII, alpha 1	0.57	0.59	1.01	0.43
SLC4A5	SLC4A5;MGC129662;NBC4	solute carrier family 4, sodium bicarbonate cotransporter, member 5	0.56	0.64	1.01	0.44
SLC1A6	SLC1A6;EAAT4;MGC33092;MGC43671	solute carrier family 1 (high affinity aspartate/glutamate transporter), member 6	0.56	0.62	1.01	0.46
SCUBE1	SCUBE1	signal peptide, CUB domain, EGF-like 1	0.56	0.6	1.01	0.4
MRPL55	MRPL55;AAVG5835;DKFZp686D1387;L55nt;MGC61802;MRP-L55;PRO19675	mitochondrial ribosomal protein L55	0.56	0.56	1.01	0.47
LONRF3	LONRF3;FLJ22612;MGC119463;MGC119465;RNF127	LON peptidase N-terminal domain and ring finger 3	0.56	0.56	1.01	0.5
RPL5	RPL5;DBA6;L5;MGC117339	ribosomal protein L5	0.56	0.58	1.01	0.44
SLC25A27	SLC25A27;FLJ33552;UCP4	solute carrier family 25, member 27	0.55	0.55	1.01	0.41
BNIPL	BNIPL;BNIP-S;BNIPL-1;BNIPL-2;PP753	BCL2/adenovirus E1B 19kD interacting protein like	0.55	0.57	1.01	0.44
FAM179A	FAM179A	family with sequence similarity 179, member A	0.55	0.54	1.01	0.41
RAB3B	RAB3B	RAB3B, member RAS oncogene family	0.54	0.53	1.01	0.4
IL1RAPL1	IL1RAPL1;IL1R8;IL1RAPL;MRX10;MRX21;MRX34;OPHN4;TIGIRR-2	interleukin 1 receptor accessory protein-like 1	0.52	0.56	1.01	0.42
SGCA	SGCA;50-DAG;A2;ADL;DAG2;DMDA2;LGMD2D;SCARMD1;adhalin	sarcoglycan, alpha (50kDa dystrophin-associated glycoprotein)	0.52	0.53	1.01	0.36
CSNK1E	CSNK1E;HCKIE;MGC10398	casein kinase 1, epsilon	0.51	0.5	1.01	0.42
RPL3	RPL3;L3;MGC104284;TARBP-B	ribosomal protein L3	0.51	0.5	1.01	0.4
NCF4	NCF4;MGC3810;NCF;P40PHOX;SH3PXD4	neutrophil cytosolic factor 4, 40kDa	0.5	0.49	1.01	0.38
VGF	VGF	VGF nerve growth factor inducible	0.5	0.5	1.01	0.38
SOX18	SOX18;HLTS	SRY (sex determining region Y)-box 18	0.5	0.5	1.01	0.43
MXD1	MXD1;BHLHC58;MAD;MAD1;MGC104659	MAX dimerization protein 1	0.5	0.55	1.01	0.38
WIBG	WIBG;MGC13064;PYM	within bgcn homolog (Drosophila)	0.5	0.52	1.01	0.39
ASH1L	ASH1L;ASH1;ASH1L1;FLJ10504;KIAA1420;KMT2H	ash1 (absent, small, or homeotic)-like (Drosophila)	0.5	0.55	1.01	0.38
LSM12	LSM12;FLJ30656;MGC104211;PNAS-135	LSM12 homolog (S. cerevisiae)	0.5	0.5	1.01	0.36
TUBB	TUBB;M40;MGC117247;MGC16435;OK/SW-cl.56;TUBB1;TUBB5	tubulin, beta class I	0.5	0.55	1.01	0.4
TECPR2	TECPR2;KIAA0297;KIAA0329	tectonin beta-propeller repeat containing 2	0.5	0.52	1.01	0.39
SLC25A47	SLC25A47;C14orf68;HDMCP	solute carrier family 25, member 47	0.49	0.5	1.01	0.34
PPOX	PPOX;MGC8485;PPO;V290M;VP	protoporphyrinogen oxidase	0.48	0.48	1.01	0.36
YRDC	YRDC;DRIP3;FLJ23476;FLJ26165;IRIP;RP11-109P14.4;SUA5	yrdC domain containing (E. coli)	0.48	0.51	1.01	0.36
CCDC56	CCDC56;FLJ50764	coiled-coil domain containing 56	0.48	0.52	1.01	0.35
MRPL47	MRPL47;L47mt;MGC45403;MRP-L47;NCM1	mitochondrial ribosomal protein L47	0.48	0.52	1.01	0.34
RASAL2	RASAL2;MGC129919;NGAP	RAS protein activator like 2	0.47	0.52	1.01	0.36
VWA5A	VWA5A;BCSC-1;BCSC1;LOH11CR2A	von Willebrand factor A domain containing 5A	0.46	0.48	1.01	0.39
ALOX15	ALOX15;15-LOX-1;15LOX-1	arachidonate 15-lipoxygenase	0.45	0.45	1.01	0.35
SLC38A8	SLC38A8	solute carrier family 38, member 8	0.45	0.48	1.01	0.32
OPLAH	OPLAH;5-Opase;DKFZp434H244;OPLA	5-oxoprolinase (ATP-hydrolysing)	0.45	0.44	1.01	0.39
VGLL1	VGLL1;TDU;VGL1	vestigial like 1 (Drosophila)	0.44	0.45	1.01	0.31
PSMA3	PSMA3;HC8;MGC12306;MGC32631;PSC3	proteasome (prosome, macropain) subunit, alpha type, 3	0.44	0.44	1.01	0.33
PRG2	PRG2;BMPG;MBP;MBP1;MGC14537	proteoglycan 2, bone marrow (natural killer cell activator, eosinophil granule major basic protein)	0.38	0.37	1.01	0.26
TFF1	TFF1;BCEI;D21S21;HP1.A;HPS2;pNR-2;pS2	trefoil factor 1	0.29	0.31	1.01	0.25
PSMC3	PSMC3;MGC8487;TBP1	proteasome (prosome, macropain) 26S subunit, ATPase, 3	0.29	0.27	1.01	0.22
DKK3	DKK3;REIC;RIG	dickkopf homolog 3 (Xenopus laevis)	0.08	0.08	1.01	0.06
HEPACAM	HEPACAM;FLJ25530;GlialCAM;MLC2A;MLC2B	hepatic and glial cell adhesion molecule	0.6	0.66	1.02	0.44
CPNE6	CPNE6;N-COPINE	copine VI (neuronal)	0.6	0.6	1.02	0.48
SERPINE2	SERPINE2;DKFZp686A13110;GDN;PI-7;PI7;PN-1;PN1;PNI	serpin peptidase inhibitor, clade E (nexin, plasminogen activator inhibitor type 1), member 2	0.6	0.64	1.02	0.44
TAS1R3	TAS1R3;T1R3	taste receptor, type 1, member 3	0.6	0.61	1.02	0.41
KLF1	KLF1;EKLF;HBFQTL6;INLU	Kruppel-like factor 1 (erythroid)	0.6	0.65	1.02	0.43
SPATA5	SPATA5;AFG2;SPAF	spermatogenesis associated 5	0.6	0.6	1.02	0.42
NPDC1	NPDC1;CAB;CAB-;CAB-1;CAB1;DKFZp586J0523;NPDC-1	neural proliferation, differentiation and control, 1	0.6	0.62	1.02	0.49
ANKFY1	ANKFY1;ANKHZN;DKFZp686M19106;KIAA1255;ZFYVE14	ankyrin repeat and FYVE domain containing 1	0.6	0.64	1.02	0.47
ZYX	ZYX;ESP-2;HED-2	zyxin	0.6	0.66	1.02	0.49
CSDA	CSDA;CSDA1;DBPA;ZONAB	cold shock domain protein A	0.6	0.66	1.02	0.43
C17orf87	C17orf87;FLJ32580;MGC163426;MGC163428;SCIMP;UNQ5783	chromosome 17 open reading frame 87	0.59	0.62	1.02	0.42
GJC3	GJC3;CX29;CX30.2;CX31.3;GJE1	gap junction protein, gamma 3, 30.2kDa	0.59	0.6	1.02	0.41
UGT1A9	UGT1A9;HLUGP4;LUGP4;UDPGT;UDPGT 1-9;UGT-1I;UGT1-09;UGT1-9;UGT1.9;UGT1AI;UGT1I	UDP glucuronosyltransferase 1 family, polypeptide A9	0.59	0.64	1.02	0.49
ABCG8	ABCG8;GBD4;MGC142217;STSL	ATP-binding cassette, sub-family G (WHITE), member 8	0.58	0.58	1.02	0.46
MMP23B	MMP23B;MIFR;MIFR-1;MMP22;MMP23A	matrix metallopeptidase 23B	0.58	0.63	1.02	0.44
C14orf126	C14orf126;FLJ51408;MGC9912	chromosome 14 open reading frame 126	0.58	0.62	1.02	0.47
CHML	CHML;FLJ10071;FLJ13361;REP2	choroideremia-like (Rab escort protein 2)	0.58	0.58	1.02	0.44
KDM5C	KDM5C;DXS1272E;JARID1C;MRXJ;MRXSCJ;MRXSJ;SMCX;XE169	lysine (K)-specific demethylase 5C	0.58	0.59	1.02	0.53
SPRR3	SPRR3	small proline-rich protein 3	0.57	0.57	1.02	0.43
SIVA1	SIVA1;CD27BP;SIVA;Siva-1;Siva-2	SIVA1, apoptosis-inducing factor	0.57	0.59	1.02	0.46
C14orf129	C14orf129;GSKIP;MGC4945	chromosome 14 open reading frame 129	0.57	0.57	1.02	0.46
IGFLR1	IGFLR1;FLJ22573;TMEM149;U2AF1L4	IGF-like family receptor 1	0.57	0.55	1.02	0.43
LACE1	LACE1;AFG1	lactation elevated 1	0.56	0.55	1.02	0.45
EFCAB4B	EFCAB4B;CRACR2A;DKFZp686G13246;FLJ33046;FLJ33805;MGC4266	EF-hand calcium binding domain 4B	0.55	0.54	1.02	0.41
ZNF585B	ZNF585B;FLJ14928;FLJ33255;SZFP41	zinc finger protein 585B	0.55	0.58	1.02	0.47
PIGO	PIGO;DKFZp434M222;FLJ00135;MGC20536;MGC3079;RP11-182N22.4	phosphatidylinositol glycan anchor biosynthesis, class O	0.55	0.55	1.02	0.41
TMEM203	TMEM203;HBEBP1;MGC14327;MGC15961	transmembrane protein 203	0.55	0.57	1.02	0.41
AK8	AK8;C9orf98;DDX31;FLJ32704;FLJ36014;RP11-143F18.1	adenylate kinase 8	0.55	0.58	1.02	0.42
HLF	HLF;MGC33822	hepatic leukemia factor	0.54	0.54	1.02	0.46
C1orf200	C1orf200	chromosome 1 open reading frame 200	0.54	0.56	1.02	0.38
PIK3C2B	PIK3C2B;C2-PI3K;DKFZp686G16234	phosphoinositide-3-kinase, class 2, beta polypeptide	0.54	0.6	1.02	0.42
LCN2	LCN2;24p3;MSFI;NGAL	lipocalin 2	0.54	0.54	1.02	0.46
SLC29A4	SLC29A4;ENT4;FLJ34923;PMAT	solute carrier family 29 (nucleoside transporters), member 4	0.53	0.56	1.02	0.38

WWOX	WWOX;D16S432E;FOR;FRA16D;HHCMA56;PRO0128;SDR41C1;WOX1	WW domain containing oxidoreductase	0.53	0.53	1.02	0.41
GLCE	GLCE;HSEPI;KIAA0836	glucuronic acid epimerase	0.53	0.6	1.02	0.42
HTR1E	HTR1E;5-HT1E	5-hydroxytryptamine (serotonin) receptor 1E	0.52	0.5	1.02	0.46
RASGRF1	RASGRF1;CDC25;CDC25L;GNRP;GRF1;GRF55;H-GRF55;PP13187;ras-GRF1	Ras protein-specific guanine nucleotide-releasing factor 1	0.52	0.54	1.02	0.41
EFNA5	EFNA5;AF1;EFL5;EPLG7;GLC1M;LERK7;RAGS	ephrin-A5	0.51	0.56	1.02	0.4
POLD3	POLD3;KIAA0039;MGC119642;MGC119643;P66;P68	polymerase (DNA-directed), delta 3, accessory subunit	0.51	0.56	1.02	0.42
SOHLH2	SOHLH2;FLJ20449;FLJ57222;TEB1;bHLHe81	spermatogenesis and oogenesis specific basic helix-loop-helix 2	0.5	0.56	1.02	0.37
ZNF41	ZNF41;MGC8941;MRX89	zinc finger protein 41	0.5	0.47	1.02	0.42
NCK2	NCK2;GRB4;NCKbeta	NCK adaptor protein 2	0.5	0.55	1.02	0.41
CLASRP	CLASRP;CLASP;FLJ90109;SFRS16;SWAP2	CLK4-associating serine/arginine rich protein	0.49	0.49	1.02	0.37
SAP30L	SAP30L;DKFZp667L2214;FLJ11526;FLJ23595;FLJ36497;NS4ATP2	SAP30-like	0.47	0.47	1.02	0.35
UEVLD	UEVLD;ATTP;FLJ11068;UEV3	UEV and lactate/malate dehyrogenase domains	0.47	0.44	1.02	0.34
FAM18B2	FAM18B2;MGC8763	family with sequence similarity 18, member B2	0.47	0.52	1.02	0.35
FBXO2	FBXO2;FBG1;FBX2;Fbs1;NFB42;OCP1	F-box protein 2	0.46	0.45	1.02	0.33
ZNF770	ZNF770;FLJ20582;PRO1914	zinc finger protein 770	0.46	0.47	1.02	0.34
PRPF4	PRPF4;HPRP4;HPRP4P;PRP4;Prp4p	PRP4 pre-mRNA processing factor 4 homolog (yeast)	0.44	0.44	1.02	0.36
TDRD7	TDRD7;CATC4;KIAA1529;PCTAIRE2BP;RP11-508D10.1;TRAP	tudor domain containing 7	0.43	0.44	1.02	0.32
GHDC	GHDC;D11LGP1;LGP1	GH3 domain containing	0.32	0.34	1.02	0.25
CHMP2A	CHMP2A;BC-2;BC2;CHMP2;VPS2;VPS2A	charged multivesicular body protein 2A	0.3	0.32	1.02	0.22
RPN2	RPN2;RIBIIR;RPN-II;RPNII;SWP1	ribophorin II	0.24	0.24	1.02	0.16
CHODL	CHODL;C21orf68;FLJ12627;MT75	chondrolectin	0.6	0.64	1.03	0.43
ZNF646	ZNF646;KIAA0296	zinc finger protein 646	0.6	0.62	1.03	0.42
C17orf63	C17orf63;FLJ10700	chromosome 17 open reading frame 63	0.6	0.6	1.03	0.48
CHSY1	CHSY1;CHSY;CSS1;ChSy-1;DKFZp434M032;FLJ58581;KIAA0990;TPBS	chondroitin sulfate synthase 1	0.6	0.63	1.03	0.49
SF3B4	SF3B4;Hsh49;MGC10828;SAP49;SF3b49	splicing factor 3b, subunit 4, 49kDa	0.6	0.65	1.03	0.53
LAMC2	LAMC2;B2T;BM600;CSF;EBR2;EBR2A;LAMB2T;LAMNB2;MGC138491;MGC141938	laminin, gamma 2	0.6	0.67	1.03	0.49
RGN	RGN;GNL;RC;SMP30	regucalcin (senescence marker protein-30)	0.59	0.54	1.03	0.46
ITGB2	ITGB2;CD18;LAD;LCAMB;LFA-1;MAC-1;MF17;MFI7	integrin, beta 2 (complement component 3 receptor 3 and 4 subunit)	0.59	0.62	1.03	0.47
PTK2	PTK2;FADK;FAK;FAK1;FRNK;PPP1R71;pp125FAK	PTK2 protein tyrosine kinase 2	0.59	0.6	1.03	0.5
NTPCR	NTPCR;C1orf57;FLJ11383;HCR-NTPase;MGC13186;RP4-678E16.2	nucleoside-triphosphatase, cancer-related	0.59	0.66	1.03	0.44
CD274	CD274;B7-H;B7H1;MGC142294;MGC142296;PD-L1;PDCD1L1;PDCD1LG1;PDL1	CD274 molecule	0.58	0.62	1.03	0.46
ZNF445	ZNF445;MGC126535;ZKSCAN15;ZNF168	zinc finger protein 445	0.58	0.62	1.03	0.44
TNRC6A	TNRC6A;CAGH26;DKFZp666E117;FLJ22043;GW1;GW182;KIAA1460;MGC75384;TNRC6	trinucleotide repeat containing 6A	0.58	0.62	1.03	0.45
UBR4	UBR4;FLJ41863;KIAA0462;KIAA1307;RBAF600;ZUBR1;p600	ubiquitin protein ligase E3 component n-recognin 4	0.58	0.65	1.03	0.43
NSUN5P2	NSUN5P2;DKFZp434K058;DKFZp666P104;FLJ11626;MGC129801;MGC15057;NOL1R2;NSUN5C;WBSCR20B;WBSCR20C	NOP2/Sun domain family, member 5 pseudogene 2	0.58	0.63	1.03	0.45
FASLG	FASLG;APT1LG1;CD178;CD95-L;CD95L;FASL;TNFSF6	Fas ligand (TNF superfamily, member 6)	0.57	0.64	1.03	0.44
RTP4	RTP4;IFRG28	receptor (chemosensory) transporter protein 4	0.57	0.64	1.03	0.43
FUT10	FUT10;MGC11141	fucosyltransferase 10 (alpha (1,3) fucosyltransferase)	0.57	0.55	1.03	0.47
PITX3	PITX3;CTPP4;MGC12766;PTX3	paired-like homeodomain 3	0.57	0.57	1.03	0.5
CCHCR1	CCHCR1;C6orf18;HCR;MGC126371;MGC126372;SBP	coiled-coil alpha-helical rod protein 1	0.57	0.62	1.03	0.43
MSH3	MSH3;DUP;MGC163306;MGC163308;MRP1	mutS homolog 3 (E. coli)	0.57	0.64	1.03	0.48
CXXC5	CXXC5;CF5;RINF;WID	CXXC finger protein 5	0.57	0.61	1.03	0.46
SFTPA1	SFTPA1;COLEC4;FLJ50593;FLJ51913;FLJ61144;FLJ77898;FLJ79095;FLJ99559;MGC133365;MGC198590;PSAP;PSP-A;PSPA;SFTP1;SFTPA1B;SP-A;SP-A1;SPA;SPA1	surfactant protein A1	0.56	0.62	1.03	0.47
PLCE1	PLCE1;FLJ23659;KIAA1516;MGC167842;NPHS3;PLCE;PPLC	phospholipase C, epsilon 1	0.55	0.64	1.03	0.44
NDRG1	NDRG1;CAP43;CMT4D;DRG1;GC4;HMSNL;NDR1;NMSL;PROXY1;RIT42;RTP;TARG1;TDD5	N-myc downstream regulated 1	0.55	0.56	1.03	0.43
41883	SEPT14;FLJ44060	septin 14	0.54	0.5	1.03	0.39
SERTAD3	SERTAD3;RBT1	SERTA domain containing 3	0.54	0.51	1.03	0.4
LSM5	LSM5;FLJ12710;YER146W	LSM5 homolog, U6 small nuclear RNA associated (S. cerevisiae)	0.54	0.56	1.03	0.41
DNAJC15	DNAJC15;DNAJD1;HSD18;MCJ	DnaJ (Hsp40) homolog, subfamily C, member 15	0.53	0.6	1.03	0.4
GNB1L	GNB1L;DGCRK3;GY2;KIAA1645;WDR14;WDVCF	guanine nucleotide binding protein (G protein), beta polypeptide 1-like	0.53	0.53	1.03	0.4
LOC644006	LOC644006	ring finger protein 4 pseudogene	0.53	0.54	1.03	0.48
MAP4K1	MAP4K1;HPK1	mitogen-activated protein kinase kinase kinase kinase 1	0.52	0.58	1.03	0.41
EPSTI1	EPSTI1;BRESI1;MGC29634	epithelial stromal interaction 1 (breast)	0.52	0.56	1.03	0.45
DNAJC4	DNAJC4;DANJC4;HSPF2;MCG18;MGC19482;MGC57189;MGC71863	DnaJ (Hsp40) homolog, subfamily C, member 4	0.52	0.55	1.03	0.42
KCTD20	KCTD20;C6orf69;MGC14254;dJ108K11.3	potassium channel tetramerisation domain containing 20	0.52	0.57	1.03	0.44
PSMD2	PSMD2;MGC14274;P97;Rpn1;S2;TRAP2	proteasome (prosome, macropain) 26S subunit, non-ATPase, 2	0.52	0.51	1.03	0.39
ZNF408	ZNF408;FLJ12827	zinc finger protein 408	0.51	0.54	1.03	0.36
LOC284009	LOC284009;MGC138239	uncharacterized LOC284009	0.5	0.5	1.03	0.38
WDHD1	WDHD1;AND-1;CHTF4;CTF4	WD repeat and HMG-box DNA binding protein 1	0.5	0.5	1.03	0.38
POMP	POMP;C13orf12;PNAS-110;UMP1	proteasome maturation protein	0.5	0.48	1.03	0.38
RPS10	RPS10;DBA9;MGC88819;S10	ribosomal protein S10	0.5	0.5	1.03	0.38
FNDC8	FNDC8;DKFZp434H2215	fibronectin type III domain containing 8	0.48	0.51	1.03	0.38
XIRP1	XIRP1;CMYA1;DKFZp451D042;DKFZp779C1255;DKFZp779C1947;Xin	xin actin-binding repeat containing 1	0.48	0.5	1.03	0.39
TPK1	TPK1;HTPK1;PP20	thiamin pyrophosphokinase 1	0.48	0.54	1.03	0.38
RPL27A	RPL27A;FLJ43464;L27A;MGC10850;MGC17878;MGC87238	ribosomal protein L27a	0.48	0.52	1.03	0.39
VPS18	VPS18;KIAA1475;PEP3	vacuolar protein sorting 18 homolog (S. cerevisiae)	0.47	0.48	1.03	0.43
HSPA5	HSPA5;BIP;FLJ26106;GRP78;MIF2	heat shock 70kDa protein 5 (glucose-regulated protein, 78kDa)	0.47	0.46	1.03	0.33
NODAL	NODAL;HTX5;MGC138230	nodal homolog (mouse)	0.46	0.46	1.03	0.37
TTR	TTR;CTS;CTS1;HsT2651;PALB;TBPA	transthyretin	0.46	0.46	1.03	0.35
FAM13B	FAM13B;ARHGAP49;C5orf5;DKFZp667F249;FAM13B1;FLJ26735;KHCHP;MGC57159;N61	family with sequence similarity 13, member B	0.46	0.48	1.03	0.32
CARTPT	CARTPT;CART	CART prepropeptide	0.45	0.51	1.03	0.34
UBE2F	UBE2F;MGC18120;NCE2	ubiquitin-conjugating enzyme E2F (putative)	0.45	0.43	1.03	0.32
CEBPD	CEBPD;C/EBP-delta;CELF;CRP3;NF-IL6-beta	CCAAT/enhancer binding protein (C/EBP), delta	0.42	0.46	1.03	0.31
FAM86B1	FAM86B1;MGC104828;MGC16279	family with sequence similarity 86, member B1	0.42	0.41	1.03	0.33
ITPRIPL1	ITPRIPL1;KIAA1754L	inositol 1,4,5-trisphosphate receptor interacting protein-like 1	0.39	0.41	1.03	0.3
OR8D2	OR8D2;JCG2	olfactory receptor, family 8, subfamily D, member 2	0.38	0.41	1.03	0.33
OR2T12	OR2T12;OR1-57	olfactory receptor, family 2, subfamily T, member 12	0.38	0.42	1.03	0.31
COL9A1	COL9A1;DJ149L1.1.2;EDM6;FLJ40263;MED;STL4	collagen, type IX, alpha 1	0.38	0.39	1.03	0.27
RPL26	RPL26;L26	ribosomal protein L26	0.38	0.43	1.03	0.31
ZNF300	ZNF300;DKFZp686B24204	zinc finger protein 300	0.36	0.37	1.03	0.29
RPAP2	RPAP2;C1orf82;FLJ13150	RNA polymerase II associated protein 2	0.35	0.36	1.03	0.26
SLC26A11	SLC26A11;MGC46523	solute carrier family 26, member 11	0.34	0.34	1.03	0.26
PSMF1	PSMF1;PI31	proteasome (prosome, macropain) inhibitor subunit 1 (PI31)	0.33	0.36	1.03	0.29
CCDC59	CCDC59;BR22;DKFZp686K1021;FLJ10294;TAP26	coiled-coil domain containing 59	0.3	0.34	1.03	0.22
ERCC6	ERCC6;ARMD5;CKN2;COFS;COFS1;CSB;RAD26	excision repair cross-complementing rodent repair deficiency, complementation group 6	0.29	0.3	1.03	0.22
FBXO32	FBXO32;FLJ32424;Fbx32;MAFbx;MGC33610	F-box protein 32	0.23	0.23	1.03	0.17
ZIC4	ZIC4;FLJ42609;FLJ45833	Zic family member 4	0.6	0.64	1.04	0.46
ZNF678	ZNF678;FLJ18355;FLJ22822;MGC15634;MGC42493	zinc finger protein 678	0.6	0.65	1.04	0.46
CHPF2	CHPF2;CSGLCA-T;CSGlcAT;ChSy-3;KIAA1402	chondroitin polymerizing factor 2	0.6	0.6	1.04	0.52
FLJ42957	FLJ42957	ENTREZ STATUS: REMAPPED TO GENE ID: 100287569	0.59	0.64	1.04	0.52
ZFYVE19	ZFYVE19;FLJ14840;MPFYVE	zinc finger, FYVE domain containing 19	0.59	0.6	1.04	0.45
ITGB1BP3	ITGB1BP3;MGC126624;MIBP;NRK2	integrin beta 1 binding protein 3	0.59	0.64	1.04	0.45
RUNX1T1	RUNX1T1;AML1T1;CBFA2T1;CDR;DKFZp564B213;ETO;FLJ33145;MGC2796;MTG8;ZMYND2	runt-related transcription factor 1; translocated to, 1 (cyclin D-related)	0.58	0.64	1.04	0.42
TCP11	TCP11;D6S230E;KIAA0229;MGC111103	t-complex 11 homolog (mouse)	0.58	0.62	1.04	0.42
ZFHX2	ZFHX2;FLJ17566;FLJ42478;KIAA1056;KIAA1762;MGC141820;ZFH-5;ZNF409	zinc finger homeobox 2	0.58	0.64	1.04	0.52
LEPREL2	LEPREL2;GRCB;HSU47926;P3H3	leprecan-like 2	0.58	0.6	1.04	0.44
DUSP15	DUSP15;FLJ40111;VHY	dual specificity phosphatase 15	0.58	0.56	1.04	0.42
ADCK2	ADCK2;AARF;MGC20727	aarF domain containing kinase 2	0.58	0.65	1.04	0.46
VGLL3	VGLL3;DKFZp686O1845;FLJ38507;VGL-3;VGL3	vestigial like 3 (Drosophila)	0.57	0.57	1.04	0.43
ZCCHC10	ZCCHC10;FLJ20094	zinc finger, CCHC domain containing 10	0.57	0.62	1.04	0.47
APC	APC;BTPS2;DP2;DP2.5;DP3;GS;PPP1R46	adenomatous polyposis coli	0.57	0.56	1.04	0.44
HMX1	HMX1;H6;NKX5-3	H6 family homeobox 1	0.57	0.64	1.04	0.48
CHMP1A	CHMP1A;CHMP1;KIAA0047;PCOLN3;PRSM1;VPS46-1;VPS46A	chromatin modifying protein 1A	0.57	0.59	1.04	0.44
IFRG15	IFRG15	ENTREZ STATUS: REMAPPED TO GENE ID: 163590	0.57	0.61	1.04	0.47
TMED10	TMED10;P24(DELTA);S31I125;S31III125;TMP21;Tmp-21-I;p23	transmembrane emp24-like trafficking protein 10 (yeast)	0.57	0.56	1.04	0.44
IQCF1	IQCF1;FLJ27508;MGC39725	IQ motif containing F1	0.56	0.63	1.04	0.44
GPR4	GPR4	G protein-coupled receptor 4	0.56	0.57	1.04	0.51
IVL	IVL	involucrin	0.56	0.55	1.04	0.4
NARG2	NARG2;BRCC1	NMDA receptor regulated 2	0.56	0.6	1.04	0.4
HMGN3	HMGN3;DKFZp686E20226;FLJ42187;PNAS-25;TRIP7	high mobility group nucleosomal binding domain 3	0.56	0.6	1.04	0.46
FOXM1	FOXM1;FKHL16;FOXM1B;HFH-11;HFH11;HNF-3;INS-1;MPHOSPH2;MPP-2;MPP2;PIG29;TGT3;TRIDENT	forkhead box M1	0.56	0.59	1.04	0.45
KDM3B	KDM3B;5qNCA;C5orf7;JMJD1B;KIAA1082;NET22	lysine (K)-specific demethylase 3B	0.56	0.57	1.04	0.46
RASGRP4	RASGRP4	RAS guanyl releasing protein 4	0.55	0.6	1.04	0.49
DMRTA1	DMRTA1;DMO;MGC163307;MGC163309	DMRT-like family A1	0.55	0.58	1.04	0.45
PAGE3	PAGE3;CT16.6;GAGED1;PAGE-3	P antigen family, member 3 (prostate associated)	0.54	0.61	1.04	0.41
PDILT	PDILT;PDIA7	protein disulfide isomerase-like, testis expressed	0.54	0.6	1.04	0.42
CAMP	CAMP;CAP-18;CAP18;CRAMP;FALL-39;FALL39;LL37	cathelicidin antimicrobial peptide	0.54	0.58	1.04	0.42
COL8A2	COL8A2;FECD;FECD1;FLJ00201;MGC116970;MGC116972;PPCD;PPCD2	collagen, type VIII, alpha 2	0.54	0.59	1.04	0.48
REEP2	REEP2;C5orf19;SGC32445	receptor accessory protein 2	0.54	0.53	1.04	0.43
DIP2B	DIP2B;KIAA1463;MGC104005	DIP2 disco-interacting protein 2 homolog B (Drosophila)	0.54	0.55	1.04	0.4
CHUK	CHUK;IKBKA;IKK-alpha;IKK1;IKKA;NFKBIKA;TCF16	conserved helix-loop-helix ubiquitous kinase	0.54	0.6	1.04	0.43
TWIST1	TWIST1;ACS3;BPES2;BPES3;CRS1;SCS;TWIST;bHLHa38	twist homolog 1 (Drosophila)	0.53	0.54	1.04	0.38
WFDC12	WFDC12;C20orf122;SWAM2;WAP2;dJ211D12.4	WAP four-disulfide core domain 12	0.52	0.55	1.04	0.42
HOXC6	HOXC6;CP25;HHO.C8;HOX3;HOX3C	homeobox C6	0.52	0.52	1.04	0.41
TIGD6	TIGD6;DKFZp761E2110	tigger transposable element derived 6	0.52	0.52	1.04	0.37
MED28	MED28;1500003D12Rik;DKFZp434N185;EG1;magicin	mediator complex subunit 28	0.52	0.52	1.04	0.43
LRRC8D	LRRC8D;FLJ10470;FLJ20403;LRRC5	leucine rich repeat containing 8 family, member D	0.52	0.51	1.04	0.39
MYBL1	MYBL1;A-MYB;AMYB;MGC120059;MGC120061	v-myb myeloblastosis viral oncogene homolog (avian)-like 1	0.51	0.54	1.04	0.36
LYPD5	LYPD5;FLJ30469;PRO4356	LY6/PLAUR domain containing 5	0.5	0.49	1.04	0.4

CORO1B	CORO1B;CORONIN-2;DKFZp762I166	coronin, actin binding protein, 1B	0.5	0.52	1.04	0.37
FAM35A	FAM35A;FAM35A1;MGC5560;bA163M19.1	family with sequence similarity 35, member A	0.5	0.54	1.04	0.41
TMEM50A	TMEM50A;IFNRC;SMP1	transmembrane protein 50A	0.5	0.55	1.04	0.36
OGFR	OGFR	opioid growth factor receptor	0.49	0.52	1.04	0.42
HNRNPA2B1	HNRNPA2B1;DKFZp779B0244;FLJ22720;HNRNPA2;HNRNPB1;HNRPA2;HNRPA2B1;HNRPB1;RNPA2;SNRPB1	heterogeneous nuclear ribonucleoprotein A2/B1	0.49	0.52	1.04	0.37
SLC30A2	SLC30A2;FLJ36708;MGC11303;PP12488;ZNT2;ZnT-2	solute carrier family 30 (zinc transporter), member 2	0.48	0.52	1.04	0.4
GOSR1	GOSR1;GOLIM2;GOS-28;GOS28;GOS28/P28;GS28;P28	golgi SNAP receptor complex member 1	0.48	0.5	1.04	0.33
REST	REST;NRSF;XBR	RE1-silencing transcription factor	0.48	0.48	1.04	0.34
TACSTD2	TACSTD2;EGP-1;EGP1;GA733-1;GA7331;GP50;M1S1;TROP2	tumor-associated calcium signal transducer 2	0.48	0.51	1.04	0.37
PRAMEF16	PRAMEF16	PRAME family member 16	0.47	0.48	1.04	0.35
PDGFRB	PDGFRB;CD140B;JTK12;PDGFR;PDGFR1	platelet-derived growth factor receptor, beta polypeptide	0.46	0.52	1.04	0.37
C17orf39	C17orf39;MGC3048	chromosome 17 open reading frame 39	0.46	0.5	1.04	0.36
KIAA0232	KIAA0232	KIAA0232	0.46	0.5	1.04	0.33
PTMA	PTMA;MGC104802;TMSA	prothymosin, alpha	0.46	0.46	1.04	0.32
NCAN	NCAN;CSPG3;FLJ44681	neurocan	0.45	0.44	1.04	0.36
OR5M9	OR5M9;OR11-190	olfactory receptor, family 5, subfamily M, member 9	0.44	0.48	1.04	0.32
CLDN23	CLDN23;CLDNL;hCG1646163	claudin 23	0.44	0.42	1.04	0.33
SERTAD2	SERTAD2;MGC126688;MGC126690;Sei-2;TRIP-Br2	SERTA domain containing 2	0.44	0.45	1.04	0.34
ZNF117	ZNF117;H-plk;HPF9;MGC22613	zinc finger protein 117	0.42	0.42	1.04	0.31
CSAD	CSAD;CSD;FLJ44987;FLJ45500;MGC119354;MGC119355;MGC119357;PCAP	cysteine sulfinic acid decarboxylase	0.42	0.44	1.04	0.29
HUNK	HUNK	hormonally up-regulated Neu-associated kinase	0.42	0.47	1.04	0.34
NDUFA1	NDUFA1;CI-MWFE;MWFE;ZNF183	NADH dehydrogenase (ubiquinone) 1 alpha subcomplex, 1, 7.5kDa	0.41	0.45	1.04	0.3
PCDHGA1	PCDHGA1;MGC138287;MGC138289;PCDH-GAMMA-A1	protocadherin gamma subfamily A, 1	0.4	0.4	1.04	0.28
PLA2G4E	PLA2G4E;FLJ45651;MGC126633;MGC126661	phospholipase A2, group IVE	0.3	0.3	1.04	0.23
PEX26	PEX26;FLJ20695;PEX26M1T;Pex26pM1T	peroxisomal biogenesis factor 26	0.2	0.22	1.04	0.17
SFXN2	SFXN2	sideroflexin 2	0.18	0.19	1.04	0.15
GPR158	GPR158;FLJ37801;KIAA1136;RP11-59G22.1	G protein-coupled receptor 158	0.6	0.61	1.05	0.55
ZNF366	ZNF366;DCSCRIPT;FLJ39796	zinc finger protein 366	0.6	0.57	1.05	0.45
CTNND2	CTNND2;GT24;NPRAP	catenin (cadherin-associated protein), delta 2 (neural plakophilin-related arm-repeat protein)	0.6	0.67	1.05	0.54
PRPH	PRPH;NEF4;PRPH1	peripherin	0.6	0.61	1.05	0.49
C19orf34	C19orf34;MGC39696	chromosome 19 open reading frame 34	0.6	0.67	1.05	0.47
POLG2	POLG2;HP55;MTPOLB;PEOA4;POLB;POLG-BETA;POLGB	polymerase (DNA directed), gamma 2, accessory subunit	0.6	0.61	1.05	0.53
ACVR2B	ACVR2B;ACTRIIB;ActR-IIB;HTX4;MGC116908	activin A receptor, type IIB	0.6	0.68	1.05	0.48
KCTD17	KCTD17;FLJ12242;FLJ98761	potassium channel tetramerisation domain containing 17	0.6	0.63	1.05	0.43
TTC13	TTC13;FLJ22584	tetratricopeptide repeat domain 13	0.6	0.62	1.05	0.52
ACTR2	ACTR2;ARP2	ARP2 actin-related protein 2 homolog (yeast)	0.6	0.65	1.05	0.5
SUPT4H1	SUPT4H1;SPT4;SPT4H;SUPT4H	suppressor of Ty 4 homolog 1 (S. cerevisiae)	0.59	0.66	1.05	0.5
DCP1B	DCP1B;DCP1;hDcp1b	DCP1 decapping enzyme homolog B (S. cerevisiae)	0.58	0.64	1.05	0.46
STOM	STOM;BND7;EPB7;EPB72	stomatin	0.58	0.62	1.05	0.47
FAM53C	FAM53C;C5orf6	family with sequence similarity 53, member C	0.58	0.66	1.05	0.46
CCDC136	CCDC136;DKFZp434G156;MGC129657;MGC176632	coiled-coil domain containing 136	0.57	0.54	1.05	0.42
ANG	ANG;ALS9;HEL168;MGC22466;MGC71966;RNASE4;RNASE5	angiogenin, ribonuclease, RNase A family, 5	0.57	0.59	1.05	0.51
AGPAT6	AGPAT6;1-AGPAT 6;DKFZp586M1819;LPAAT-zeta;LPAATZ;TSARG7	1-acylglycerol-3-phosphate O-acyltransferase 6 (lysophosphatidic acid acyltransferase, zeta)	0.57	0.61	1.05	0.48
NUP98	NUP98;ADIR2;NUP196;NUP96	nucleoporin 98kDa	0.57	0.57	1.05	0.45
RFC3	RFC3;MGC5276;RFC38	replication factor C (activator 1) 3, 38kDa	0.57	0.58	1.05	0.46
ZDHHC19	ZDHHC19;DHHC19;MGC33345	zinc finger, DHHC-type containing 19	0.56	0.61	1.05	0.44
TLR9	TLR9;CD289	toll-like receptor 9	0.56	0.66	1.05	0.45
TRA2A	TRA2A;HSU53209	transformer 2 alpha homolog (Drosophila)	0.56	0.62	1.05	0.5
RBP3	RBP3;D10S64;D10S65;D10S66;IRBP;RBPI	retinol binding protein 3, interstitial	0.55	0.56	1.05	0.43
SYPL2	SYPL2;MG29	synaptophysin-like 2	0.55	0.6	1.05	0.45
SLC2A4RG	SLC2A4RG;GEF;HDBP-1;HDBP1;Si-1-2;Si-1-2-19	SLC2A4 regulator	0.55	0.59	1.05	0.43
ADCY7	ADCY7;AC7;FLJ36387;KIAA0037	adenylate cyclase 7	0.55	0.56	1.05	0.42
C12orf61	C12orf61;FLJ25590	chromosome 12 open reading frame 61	0.54	0.56	1.05	0.42
SYNJ2BP	SYNJ2BP;ARIP2;FLJ11271;FLJ41973;OMP25	synaptojanin 2 binding protein	0.54	0.6	1.05	0.4
EEF1E1	EEF1E1;AIMP3;P18	eukaryotic translation elongation factor 1 epsilon 1	0.54	0.57	1.05	0.45
RGL3	RGL3;FLJ00153;FLJ32585;FLJ44275;MGC126805;MGC138163	ral guanine nucleotide dissociation stimulator-like 3	0.54	0.51	1.05	0.42
RPS17P16	RPS17P16;RPS17_8_1748	ribosomal protein S17 pseudogene 16	0.54	0.58	1.05	0.44
RXRG	RXRG;NR2B3;RXRC	retinoid X receptor, gamma	0.53	0.57	1.05	0.45
IKZF4	IKZF4;EOS;KIAA1782;ZNFN1A4	IKAROS family zinc finger 4 (Eos)	0.53	0.56	1.05	0.42
CYP26C1	CYP26C1;FLJ45301	cytochrome P450, family 26, subfamily C, polypeptide 1	0.53	0.54	1.05	0.39
DGCR6L	DGCR6L;FLJ10666	DiGeorge syndrome critical region gene 6-like	0.53	0.56	1.05	0.45
AP2M1	AP2M1;AP50;CLAPM1;mu2	adaptor-related protein complex 2, mu 1 subunit	0.53	0.6	1.05	0.48
PNMT	PNMT;MGC34570;PENT;PNMTase	phenylethanolamine N-methyltransferase	0.52	0.58	1.05	0.44
TLL1	TLL1;ASD6;TLL	tolloid-like 1	0.52	0.54	1.05	0.41
MRPL2	MRPL2;MRP-L14;RPML14	mitochondrial ribosomal protein L2	0.52	0.52	1.05	0.44
C16orf13	C16orf13;MGC13114	chromosome 16 open reading frame 13	0.51	0.53	1.05	0.37
BPIFA1	BPIFA1;LPLUNC3;LUNX;NASG;PLUNC;SPLUNC1;SPURT;bA49G10.5	BPI fold containing family A, member 1	0.51	0.55	1.05	0.39
FAM118A	FAM118A;C22orf8;FLJ20635	family with sequence similarity 118, member A	0.5	0.56	1.05	0.43
EGFL7	EGFL7;MGC111117;NEU1;RP11-251M1.2;VE-STATIN;ZNEU1	EGF-like-domain, multiple 7	0.47	0.52	1.05	0.39
SART1	SART1;Ara1;HOMS1;MGC2038;SART1259;SNRNP110;Snu66	squamous cell carcinoma antigen recognized by T cells	0.47	0.5	1.05	0.35
RAD21	RAD21;FLJ25655;FLJ40596;HR21;HRAD21;KIAA0078;MCD1;NXP1;SCC1;hHR21	RAD21 homolog (S. pombe)	0.46	0.48	1.05	0.35
MLN	MLN;MGC138519	motilin	0.45	0.52	1.05	0.38
HNRNPF	HNRNPF;HNRPF;MGC110997;OK/SW-cl.23;mcs94-1	heterogeneous nuclear ribonucleoprotein F	0.42	0.46	1.05	0.36
INPP4A	INPP4A;INPP4;TVAS1	inositol polyphosphate-4-phosphatase, type I, 107kDa	0.4	0.39	1.05	0.29
PMVK	PMVK;HUMPMKI;PMK;PMKA;PMKASE	phosphomevalonate kinase	0.39	0.44	1.05	0.31
CDRT4	CDRT4;FLJ36674;MGC33988	CMT1A duplicated region transcript 4	0.38	0.38	1.05	0.31
POTEH	POTEH;A26C3;ACTBL1;CT104.7;POTE22	POTE ankyrin domain family, member H	0.36	0.38	1.05	0.26
TRIM68	TRIM68;FLJ10369;GC109;MGC126176;RNF137;SS-56;SS56	tripartite motif containing 68	0.32	0.32	1.05	0.29
CDK5R1	CDK5R1;CDK5P35;CDK5R;MGC33831;NCK5A;p23;p25;p35;p35nck5a	cyclin-dependent kinase 5, regulatory subunit 1 (p35)	0.31	0.34	1.05	0.25
ACCN2	ACCN2;ASIC;ASIC1;ASIC1A;BNaC2;hBNaC2	amiloride-sensitive cation channel 2, neuronal	0.24	0.27	1.05	0.21
TTC25	TTC25;DKFZp434H0115	tetratricopeptide repeat domain 25	0.6	0.66	1.06	0.47
KIF24	KIF24;C9orf48;FLJ10933;FLJ43884;MGC125677;MGC125678;bA571F15.4	kinesin family member 24	0.6	0.66	1.06	0.45
CROT	CROT;COT	carnitine O-octanoyltransferase	0.6	0.58	1.06	0.51
TMEM141	TMEM141;MGC14141;RP11-216L13.7	transmembrane protein 141	0.6	0.68	1.06	0.48
RCOR3	RCOR3;FLJ10876;FLJ16298;RP11-318L16.1	REST corepressor 3	0.6	0.63	1.06	0.44
PZP	PZP;CPAMD6;MGC133093	pregnancy-zone protein	0.6	0.61	1.06	0.47
TMEM155	TMEM155;FLJ30834	transmembrane protein 155	0.59	0.64	1.06	0.48
HLA-DQA2	HLA-DQA2;DX-ALPHA;HLA-DXA	major histocompatibility complex, class II, DQ alpha 2	0.58	0.64	1.06	0.46
ABCB8	ABCB8;EST328128;M-ABC1;MABC1	ATP-binding cassette, sub-family B (MDR/TAP), member 8	0.58	0.62	1.06	0.42
PDRG1	PDRG1;C20orf126;PDRG	p53 and DNA-damage regulated 1	0.58	0.66	1.06	0.46
HLA-E	HLA-E;DKFZp686P19218;EA1.2;EA2.1;HLA-6.2;MHC;QA1	major histocompatibility complex, class I, E	0.58	0.65	1.06	0.43
CREBZF	CREBZF;FLJ94018;SMILE;ZF	CREB/ATF bZIP transcription factor	0.57	0.65	1.06	0.5
AARS	AARS;CMT2N	alanyl-tRNA synthetase	0.57	0.58	1.06	0.5
SQSTM1	SQSTM1;A170;OSIL;PDB3;ZIP3;p60;p62;p62B	sequestosome 1	0.57	0.63	1.06	0.51
IRAK1BP1	IRAK1BP1;AIP70;MGC138458;MGC138460;SIMPL	interleukin-1 receptor-associated kinase 1 binding protein 1	0.56	0.57	1.06	0.44
CABP5	CABP5;CABP3	calcium binding protein 5	0.56	0.58	1.06	0.47
ACRBP	ACRBP;CT23;FLJ51160;OY-TES-1;SP32	acrosin binding protein	0.56	0.66	1.06	0.48
CAMK2N2	CAMK2N2;CAM-KIIN;CAMKIIN	calcium/calmodulin-dependent protein kinase II inhibitor 2	0.56	0.64	1.06	0.43
NOL10	NOL10;FLJ13938;FLJ14075;PQBP5	nucleolar protein 10	0.56	0.6	1.06	0.43
TMEM54	TMEM54;BCLP;CAC-1;CAC1;MGC10137	transmembrane protein 54	0.56	0.64	1.06	0.49
FAM73A	FAM73A;DKFZp686M07166;FLJ35093	family with sequence similarity 73, member A	0.56	0.64	1.06	0.43
MDK	MDK;FLJ27379;MK;NEGF2	midkine (neurite growth-promoting factor 2)	0.56	0.57	1.06	0.5
STARD7	STARD7;GTT1	StAR-related lipid transfer (START) domain containing 7	0.56	0.62	1.06	0.46
ZDHHC22	ZDHHC22;C14orf59	zinc finger, DHHC-type containing 22	0.55	0.56	1.06	0.45
PLAC9	PLAC9;MGC104710	placenta-specific 9	0.55	0.6	1.06	0.43
ZNF174	ZNF174;ZSCAN8	zinc finger protein 174	0.55	0.56	1.06	0.49
CDC25C	CDC25C;CDC25;PPP1R60	cell division cycle 25 homolog C (S. pombe)	0.55	0.54	1.06	0.41
R3HDM1	R3HDM1;FLJ23334;KIAA0029;R3HDM	R3H domain containing 1	0.55	0.6	1.06	0.44
TMEM109	TMEM109;MGC5508	transmembrane protein 109	0.55	0.6	1.06	0.43
CYP1B1	CYP1B1;CP1B;GLC3A;P4501B1	cytochrome P450, family 1, subfamily B, polypeptide 1	0.54	0.58	1.06	0.39
PGAP1	PGAP1;Bst1;FLJ42774;ISPD3024	post-GPI attachment to proteins 1	0.54	0.55	1.06	0.48
PRKY	PRKY;PRKXP3;PRKYP	protein kinase, Y-linked, pseudogene	0.54	0.61	1.06	0.44
KIAA1524	KIAA1524;CIP2A;FLJ12850;MGC163436;p90	KIAA1524	0.54	0.57	1.06	0.4
C5orf64	C5orf64;FLJ37543;FLJ45901;MGC138187;MGC138213	chromosome 5 open reading frame 64	0.54	0.55	1.06	0.42
COL3A1	COL3A1;EDS4A;FLJ34534	collagen, type III, alpha 1	0.5	0.56	1.06	0.45
GDF10	GDF10;BMP-3b;BMP3B	growth differentiation factor 10	0.5	0.52	1.06	0.45
TBC1D2B	TBC1D2B;FLJ20166;KIAA1055	TBC1 domain family, member 2B	0.5	0.51	1.06	0.41
TATDN1	TATDN1;FLJ43280	TatD DNase domain containing 1	0.5	0.52	1.06	0.41
RPL10A	RPL10A;Csa-19;L10A;NEDD6	ribosomal protein L10a	0.5	0.54	1.06	0.41
ANKRD23	ANKRD23;DARP;FLJ32449;MARP3;MGC129593	ankyrin repeat domain 23	0.49	0.54	1.06	0.44
DDX51	DDX51;DKFZp686N2081;MGC42193	DEAD (Asp-Glu-Ala-Asp) box polypeptide 51	0.49	0.57	1.06	0.4
PRY	PRY;PRY1;PTPN13LY	PTPN13-like, Y-linked	0.48	0.56	1.06	0.41
SHD	SHD	Src homology 2 domain containing transforming protein D	0.48	0.54	1.06	0.38
OXCT2	OXCT2;FLJ00030;SCOTT	3-oxoacid CoA transferase 2	0.48	0.57	1.06	0.39
OCEL1	OCEL1;FLJ22709;FWP009;S863-9	occludin/ELL domain containing 1	0.48	0.55	1.06	0.38
SMARCE1	SMARCE1;BAF57;FLJ35648	SWI/SNF related, matrix associated, actin dependent regulator of chromatin, subfamily e, member 1	0.48	0.56	1.06	0.39
DEM1	DEM1;C1orf176;Exo V;FLJ11445;FLJ13183;FLJ21144	defects in morphology 1 homolog (S. cerevisiae)	0.48	0.56	1.06	0.37
CDK1	CDK1;CDC2;CDC28A;DKFZp686L20222;MGC111195;P34CDC2	cyclin-dependent kinase 1	0.48	0.5	1.06	0.42
DNMT3B	DNMT3B;ICF;ICF1;M.HsaIIIB	DNA (cytosine-5-)-methyltransferase 3 beta	0.47	0.48	1.06	0.36

ACTL9	ACTL9;MGC33407	actin-like 9	0.47	0.5	1.06	0.35
C8orf4	C8orf4;MGC22806;TC-1;TC1	chromosome 8 open reading frame 4	0.46	0.5	1.06	0.34
FAM82A2	FAM82A2;FAM82C;FLJ10579;RMD3;hRMD-3;ptpip51	family with sequence similarity 82, member A2	0.46	0.46	1.06	0.38
STK17A	STK17A;DRAK1	serine/threonine kinase 17a	0.46	0.52	1.06	0.37
HLA-B	HLA-B;AS;HLAB;MGC111087;SPDA1	major histocompatibility complex, class I, B	0.46	0.48	1.06	0.42
TMEM89	TMEM89	transmembrane protein 89	0.45	0.5	1.06	0.34
NKD2	NKD2;Naked2	naked cuticle homolog 2 (Drosophila)	0.44	0.48	1.06	0.37
SYS1	SYS1;C20orf169;dJ453C12.4;dJ453C12.4.1	SYS1 Golgi-localized integral membrane protein homolog (S. cerevisiae)	0.44	0.52	1.06	0.38
RPL35A	RPL35A;DBA5;L35A	ribosomal protein L35a	0.43	0.44	1.06	0.37
LCE2C	LCE2C;LEP11	late cornified envelope 2C	0.39	0.42	1.06	0.29
CCNA2	CCNA2;CCN1;CCNA	cyclin A2	0.35	0.4	1.06	0.3
METTL21C	METTL21C;C13orf39	methyltransferase like 21C	0.32	0.34	1.06	0.25
LMO2	LMO2;RBTN2;RBTNL1;RHOM2;TTG2	LIM domain only 2 (rhombotin-like 1)	0.6	0.66	1.07	0.5
SLC7A4	SLC7A4;CAT-4;CAT4;HCAT3;MGC129976;MGC129977;VH	solute carrier family 7 (orphan transporter), member 4	0.6	0.62	1.07	0.44
NXPH3	NXPH3;NPH3	neurexophilin 3	0.6	0.63	1.07	0.53
RAB38	RAB38;NY-MEL-1;rrGTPbp	RAB38, member RAS oncogene family	0.6	0.68	1.07	0.45
COX10	COX10	COX10 homolog, cytochrome c oxidase assembly protein, heme A: farnesyltransferase (yeast)	0.6	0.6	1.07	0.54
SAFB2	SAFB2;KIAA0138	scaffold attachment factor B2	0.6	0.68	1.07	0.55
UXT	UXT;ART-27;STAP1	ubiquitously-expressed transcript	0.6	0.66	1.07	0.47
SLC17A9	SLC17A9;C20orf59;FLJ23412	solute carrier family 17, member 9	0.6	0.68	1.07	0.45
NEIL1	NEIL1;FLJ22402;FPG1;NEI1;hFPG1	nei endonuclease VIII-like 1 (E. coli)	0.59	0.6	1.07	0.47
TSPAN4	TSPAN4;NAG-2;NAG2;TETRASPAN;TM4SF7;TSPAN-4	tetraspanin 4	0.59	0.62	1.07	0.45
DNAAF1	DNAAF1;CILD13;DKFZp434A119;FLJ25330;LRRC50;ODA7	dynein, axonemal, assembly factor 1	0.59	0.56	1.07	0.44
WISP3	WISP3;CCN6;LIBC;MGC125987;MGC125988;MGC125989;PPAC;PPD	WNT1 inducible signaling pathway protein 3	0.58	0.63	1.07	0.49
TBL1X	TBL1X;EBI;SMAP55;TBL1	transducin (beta)-like 1X-linked	0.58	0.64	1.07	0.52
CORO6	CORO6;FLJ14871	coronin 6	0.57	0.63	1.07	0.47
HRASLS2	HRASLS2;FLJ20556;PLA1/2-2	HRAS-like suppressor 2	0.56	0.62	1.07	0.47
SDPR	SDPR;CAVIN2;PS-p68;SDR;cavin-2	serum deprivation response	0.56	0.61	1.07	0.44
NDUFA8	NDUFA8;CI-19KD;CI-PGIV;MGC793;PGIV	NADH dehydrogenase (ubiquinone) 1 alpha subcomplex, 8, 19kDa	0.56	0.6	1.07	0.47
ELAC2	ELAC2;ELC2;FLJ10530;FLJ36693;FLJ42848;HPC2	elaC homolog 2 (E. coli)	0.56	0.58	1.07	0.41
AIF1L	AIF1L;C9orf58;FLJ12783;IBA2;MGC29466	allograft inflammatory factor 1-like	0.56	0.63	1.07	0.51
C14orf49	C14orf49;FLJ25605;MGC75267;NET53	chromosome 14 open reading frame 49	0.55	0.57	1.07	0.46
TIGD3	TIGD3	tigger transposable element derived 3	0.55	0.6	1.07	0.4
TES	TES;DKFZp586B2022;MGC1146;TESS;TESS-2	testis derived transcript (3 LIM domains)	0.54	0.62	1.07	0.46
OR2T34	OR2T34	olfactory receptor, family 2, subfamily T, member 34	0.53	0.58	1.07	0.44
IL8	IL8;CXCL8;GCP-1;GCP1;LECT;LUCT;LYNAP;MDNCF;MONAP;NAF;NAP-1;NAP1	interleukin 8	0.53	0.58	1.07	0.42
CIB2	CIB2;KIP2	calcium and integrin binding family member 2	0.52	0.54	1.07	0.45
PLXNA1	PLXNA1;DKFZp761P19121;NOV;NOVP;PLEXIN-A1;PLXN1	plexin A1	0.52	0.61	1.07	0.45
BCR	BCR;ALL;BCR1;CML;D22S11;D22S662;FLJ16453;PHL	breakpoint cluster region	0.52	0.6	1.07	0.43
OSBPL10	OSBPL10;FLJ20363;ORP10;OSBP9	oxysterol binding protein-like 10	0.52	0.56	1.07	0.38
AKR1C3	AKR1C3;DD3;DDX;HA1753;HAKRB;HAKRe;HSD17B5;KIAA0119;PGFS;hluPGFS	aldo-keto reductase family 1, member C3 (3-alpha hydroxysteroid dehydrogenase, type II)	0.51	0.61	1.07	0.42
SNRPC	SNRPC;FLJ20302;U1C;Yhc1	small nuclear ribonucleoprotein polypeptide C	0.51	0.56	1.07	0.42
SMC4	SMC4;CAPC;SMC4L1;hCAP-C	structural maintenance of chromosomes 4	0.5	0.6	1.07	0.41
EEF1G	EEF1G;EF1G;GIG35	eukaryotic translation elongation factor 1 gamma	0.5	0.52	1.07	0.36
SCARF1	SCARF1;KIAA0149;MGC47738;SREC;SREC-I;SREC1	scavenger receptor class F, member 1	0.48	0.5	1.07	0.39
GPRIN1	GPRIN1;GRIN1;KIAA1893	G protein regulated inducer of neurite outgrowth 1	0.48	0.54	1.07	0.39
METTL19	METTL19;C4orf23;FLJ12891;FLJ35725	methyltransferase like 19	0.48	0.55	1.07	0.39
TIE1	TIE1;JTK14;TIE	tyrosine kinase with immunoglobulin-like and EGF-like domains 1	0.47	0.56	1.07	0.39
TIMP2	TIMP2;CSC-21K	TIMP metallopeptidase inhibitor 2	0.47	0.5	1.07	0.38
C10orf81	C10orf81;FLJ23537;HEL185;bA211N11.2	chromosome 10 open reading frame 81	0.46	0.52	1.07	0.42
SALL4	SALL4;DRRS;HSAL4;MGC133050;ZNF797;dJ1112F19.1	sal-like 4 (Drosophila)	0.45	0.5	1.07	0.38
VGLL2	VGLL2;VGL2;VITO1	vestigial like 2 (Drosophila)	0.44	0.49	1.07	0.37
LGALS2	LGALS2;HL14;MGC75071	lectin, galactoside-binding, soluble, 2	0.44	0.48	1.07	0.38
C11orf30	C11orf30;EMSY;FLJ90741	chromosome 11 open reading frame 30	0.44	0.52	1.07	0.38
TXNDC2	TXNDC2;DKFZp434H0311;MGC35026;SPTRX;SPTRX1	thioredoxin domain containing 2 (spermatozoa)	0.42	0.45	1.07	0.33
ADRB3	ADRB3;BETA3AR;FLJ99960	adrenergic, beta-3-, receptor	0.42	0.42	1.07	0.32
CEP250	CEP250;C-NAP1;CEP2;CNAP1;MGC88542	centrosomal protein 250kDa	0.42	0.47	1.07	0.32
CDY2A	CDY2A;CDY;CDY2;CDY2B	chromodomain protein, Y-linked, 2A	0.42	0.42	1.07	0.37
THSD7B	THSD7B;KIAA1679	thrombospondin, type I, domain containing 7B	0.4	0.42	1.07	0.31
DCLRE1A	DCLRE1A;KIAA0086;PSO2;SNM1;SNM1A	DNA cross-link repair 1A	0.39	0.4	1.07	0.32
ANLN	ANLN;DKFZp779A055;Scraps;scra	anillin, actin binding protein	0.26	0.28	1.07	0.22
GLB1L2	GLB1L2;MST114;MSTP114	galactosidase, beta 1-like 2	0.16	0.18	1.07	0.14
C11orf70	C11orf70;MGC13040	chromosome 11 open reading frame 70	0.6	0.64	1.08	0.46
CD4	CD4;CD4mut	CD4 molecule	0.6	0.65	1.08	0.44
C9orf9	C9orf9;FLJ26879	chromosome 9 open reading frame 9	0.6	0.64	1.08	0.51
BCL9L	BCL9L;BCL9-2;DLNB11	B-cell CLL/lymphoma 9-like	0.6	0.64	1.08	0.44
CCNT1	CCNT1;CCNT;CYCT1;HIVE1	cyclin T1	0.6	0.72	1.08	0.5
SPAG5	SPAG5;DEEPEST;MAP126;hMAP126	sperm associated antigen 5	0.6	0.66	1.08	0.45
VCY1B	VCY1B;BPY1;BPY1B;VCY;VCY1	variable charge, Y-linked 1B	0.6	0.66	1.08	0.5
FMOD	FMOD;SLRR2E	fibromodulin	0.59	0.66	1.08	0.45
DSEL	DSEL;C18orf4	dermatan sulfate epimerase-like	0.59	0.62	1.08	0.48
TGM1	TGM1;ICR2;KTG;LI;LI1;TGASE;TGK	transglutaminase 1 (K polypeptide epidermal type I, protein-glutamine-gamma-glutamyltransferase)	0.59	0.6	1.08	0.47
NR2F2	NR2F2;ARP1;COUPTFB;COUPTFII;MGC117452;NF-E3;NR2F1;SVP40;TFCOUP2	nuclear receptor subfamily 2, group F, member 2	0.59	0.61	1.08	0.45
FAM48A	FAM48A;C13;C13orf19;P38IP;SPT20;bA421P11.4	family with sequence similarity 48, member A	0.59	0.68	1.08	0.53
GPR148	GPR148;BTR;PGR6	G protein-coupled receptor 148	0.58	0.62	1.08	0.56
XAGE3	XAGE3;CT12.3a;CT12.3b;GAGED4;MGC71925;PLAC6;XAGE-3;pp9012	X antigen family, member 3	0.58	0.62	1.08	0.46
ZBTB8OS	ZBTB8OS;ARCH;ARCH2;MGC62007	zinc finger and BTB domain containing 8 opposite strand	0.58	0.6	1.08	0.46
FANCC	FANCC;FA3;FAC;FACC;FLJ14675	Fanconi anemia, complementation group C	0.58	0.61	1.08	0.43
CNOT3	CNOT3;KIAA0691;LENG2;NOT3;NOT3H	CCR4-NOT transcription complex, subunit 3	0.58	0.66	1.08	0.5
RNASE3	RNASE3;ECP;RNS3	ribonuclease, RNase A family, 3	0.57	0.66	1.08	0.43
CCDC77	CCDC77;MGC13183	coiled-coil domain containing 77	0.57	0.6	1.08	0.45
ARHGAP29	ARHGAP29;PARG1;RP11-255E17.1	Rho GTPase activating protein 29	0.57	0.66	1.08	0.46
ITSN2	ITSN2;KIAA1256;PRO2015;SH3D1B;SH3P18;SWA;SWAP	intersectin 2	0.56	0.58	1.08	0.46
KPNA3	KPNA3;IPOA4;SRP1;SRP1gamma;SRP4;hSRP1	karyopherin alpha 3 (importin alpha 4)	0.56	0.56	1.08	0.5
RPL35	RPL35;L35	ribosomal protein L35	0.56	0.59	1.08	0.45
ANTXR2	ANTXR2;CMG-2;CMG2;FLJ31074;ISH;JHF;MGC111533;MGC45856	anthrax toxin receptor 2	0.55	0.66	1.08	0.48
CBX4	CBX4;NBP16;PC2	chromobox homolog 4	0.55	0.62	1.08	0.41
BRMS1L	BRMS1L;BRMS1;MGC11296	breast cancer metastasis-suppressor 1-like	0.55	0.6	1.08	0.43
SPACA7	SPACA7;C13orf28;FLJ27356	sperm acrosome associated 7	0.55	0.62	1.08	0.44
CST2	CST2;MGC71924	cystatin SA	0.54	0.57	1.08	0.43
CYB561D2	CYB561D2;101F6;TSP10	cytochrome b-561 domain containing 2	0.54	0.57	1.08	0.49
SERBP1	SERBP1;CHD3IP;DKFZp564M2423;FLJ90489;HABP4L;PAI-RBP1;PAIRBP1	SERPINE1 mRNA binding protein 1	0.54	0.55	1.08	0.45
FRG1	FRG1;FRG1A;FSG1	FSHD region gene 1	0.54	0.57	1.08	0.43
DAP3	DAP3;DAP-3;DKFZp686G12159;FLJ12817;MGC126058;MGC126059;MRP-S29;MRPS29;bMRP-10	death associated protein 3	0.53	0.53	1.08	0.47
RLBP1	RLBP1;CRALBP;MGC3663	retinaldehyde binding protein 1	0.52	0.57	1.08	0.39
LOR	LOR;MGC111513	loricrin	0.52	0.59	1.08	0.44
WNT4	WNT4;SERKAL;WNT-4	wingless-type MMTV integration site family, member 4	0.52	0.6	1.08	0.42
SHPK	SHPK;CARKL;FLJ32478;SHK	sedoheptulokinase	0.5	0.53	1.08	0.44
APOB	APOB;FLDB;LDLCQ4	apolipoprotein B (including Ag(x) antigen)	0.49	0.51	1.08	0.44
RGS17	RGS17;RGS-17;RGSZ2;hRGS17	regulator of G-protein signaling 17	0.48	0.52	1.08	0.38
EPOR	EPOR;EPO-R;MGC138358	erythropoietin receptor	0.48	0.52	1.08	0.41
PLTP	PLTP;BPIFE;HDLCQ9	phospholipid transfer protein	0.48	0.52	1.08	0.4
DSP	DSP;DP;DPI;DPII	desmoplakin	0.48	0.54	1.08	0.43
RPRML	RPRML;MGC131790;MGC43894	reprimo-like	0.47	0.53	1.08	0.38
C1orf63	C1orf63;DJ465N24.2.1;NPD014;RP3-465N24.4	chromosome 1 open reading frame 63	0.46	0.5	1.08	0.36
DPYSL2	DPYSL2;CRMP-2;CRMP2;DHPRP2;DRP-2;DRP2;N2A3;ULIP-2;ULIP2	dihydropyrimidinase-like 2	0.45	0.48	1.08	0.36
FAM50B	FAM50B;D6S2654E;X5L	family with sequence similarity 50, member B	0.45	0.53	1.08	0.41
ALKBH2	ALKBH2;ABH2;FLJ99103;MGC90512	alkB, alkylation repair homolog 2 (E. coli)	0.45	0.48	1.08	0.37
IQCF3	IQCF3	IQ motif containing F3	0.45	0.52	1.08	0.4
PDE6H	PDE6H;RCD3	phosphodiesterase 6H, cGMP-specific, cone, gamma	0.44	0.45	1.08	0.4
OBFC1	OBFC1;AAF-44;AAF44;FLJ22559;RP11-541N10.2;RPA-32;STN1;bA541N10.2	oligonucleotide/oligosaccharide-binding fold containing 1	0.44	0.48	1.08	0.32
DAB2	DAB2;DOC-2;DOC2;FLJ26626	disabled homolog 2, mitogen-responsive phosphoprotein (Drosophila)	0.44	0.45	1.08	0.33
HMGN2	HMGN2;HMG17;MGC5629;MGC88718	high mobility group nucleosomal binding domain 2	0.44	0.45	1.08	0.38
VSTM2L	VSTM2L;C20orf102;dJ1118M15.2	V-set and transmembrane domain containing 2 like	0.42	0.48	1.08	0.32
COL9A3	COL9A3;DJ885L7.4.1;EDM3;FLJ90759;IDD;MED	collagen, type IX, alpha 3	0.42	0.41	1.08	0.34
PHC2	PHC2;EDR2;HPH2;MGC163502;PH2	polyhomeotic homolog 2 (Drosophila)	0.4	0.48	1.08	0.33
SMC5	SMC5;KIAA0594;SMC5L1	structural maintenance of chromosomes 5	0.39	0.42	1.08	0.3
RPS28	RPS28;S28	ribosomal protein S28	0.39	0.44	1.08	0.29
CGA	CGA;CG-ALPHA;FSHA;GPHA1;GPHa;HCG;LHA;TSHA	glycoprotein hormones, alpha polypeptide	0.34	0.36	1.08	0.26
RAB7L1	RAB7L1;DKFZp686P1051;RAB7L	RAB7, member RAS oncogene family-like 1	0.34	0.38	1.08	0.25
PFDN2	PFDN2;PFD2	prefoldin subunit 2	0.6	0.64	1.09	0.46
COX5A	COX5A;COX;COX-VA;VA	cytochrome c oxidase subunit Va	0.6	0.66	1.09	0.44
VAMP8	VAMP8;EDB;VAMP-8	vesicle-associated membrane protein 8 (endobrevin)	0.6	0.7	1.09	0.49
C17orf103	C17orf103;DKFZp761I1115;DKFZp762H185;Gtlf3b;MGC33894	chromosome 17 open reading frame 103	0.6	0.62	1.09	0.47
FLJ44054	FLJ44054	uncharacterized LOC643365	0.6	0.64	1.09	0.5
ZNF420	ZNF420;APAK;FLJ32191	zinc finger protein 420	0.59	0.7	1.09	0.47
PSMB9	PSMB9;LMP2;MGC70470;PSMB6i;RING12;beta1i	proteasome (prosome, macropain) subunit, beta type, 9 (large multifunctional peptidase 2)	0.59	0.64	1.09	0.48
TNNT2	TNNT2;CMH2;CMPD2;LVNC6;MGC3889;RCM3;TnTC;cTnT	troponin T type 2 (cardiac)	0.58	0.64	1.09	0.47
C1orf53	C1orf53	chromosome 1 open reading frame 53	0.58	0.6	1.09	0.48

RARS	RARS;ArgRS;DALRD1;MGC8641	arginyl-tRNA synthetase	0.58	0.6	1.09	0.43
SMCR7L	SMCR7L;FLJ20232;HSU79252;MID51;MIEF1;dJ1104E15.3	Smith-Magenis syndrome chromosome region, candidate 7-like	0.57	0.67	1.09	0.47
GALNT11	GALNT11;FLJ21634;MGC71630	UDP-N-acetyl-alpha-D-galactosamine:polypeptide N-acetylgalactosaminyltransferase 11 (GalNAc-T11)	0.57	0.6	1.09	0.45
EDF1	EDF1;EDF-1;MBF1;MGC9058	endothelial differentiation-related factor 1	0.57	0.6	1.09	0.44
CBX7	CBX7	chromobox homolog 7	0.56	0.6	1.09	0.45
PLEKHG1	PLEKHG1;ARHGEF41;FLJ31738;KIAA1209	pleckstrin homology domain containing, family G (with RhoGef domain) member 1	0.56	0.58	1.09	0.49
AP3B1	AP3B1;ADTB3;ADTB3A;HPS;HPS2;PE	adaptor-related protein complex 3, beta 1 subunit	0.56	0.58	1.09	0.51
MAP3K9	MAP3K9;MEKK9;MLK1;PRKE1	mitogen-activated protein kinase kinase kinase 9	0.55	0.6	1.09	0.5
FAM126B	FAM126B;FLJ10981;FLJ37917;FLJ42947;HYCC2;MGC39518	family with sequence similarity 126, member B	0.54	0.57	1.09	0.44
MBD2	MBD2;DKFZp586O0821;DMTase;NY-CO-41	methyl-CpG binding domain protein 2	0.54	0.57	1.09	0.45
MRPS21	MRPS21;MRP-S21;RPMS21	mitochondrial ribosomal protein S21	0.54	0.6	1.09	0.45
NHLRC4	NHLRC4;FLJ36208	NHL repeat containing 4	0.54	0.61	1.09	0.41
OR5AP2	OR5AP2;OR9J1	olfactory receptor, family 5, subfamily AP, member 2	0.53	0.59	1.09	0.43
C1orf21	C1orf21;PIG13	chromosome 1 open reading frame 21	0.53	0.6	1.09	0.43
SIPA1L1	SIPA1L1;DKFZp686G1344;E6TP1;FLJ37213;KIAA0440	signal-induced proliferation-associated 1 like 1	0.53	0.6	1.09	0.43
TAGLN3	TAGLN3;NP22;NP25	transgelin 3	0.52	0.61	1.09	0.43
NARFL	NARFL;FLJ21988;HPRN;IOP1;LET1L;PRN	nuclear prelamin A recognition factor-like	0.52	0.62	1.09	0.46
ZNF551	ZNF551;DKFZp686H1038;MGC52307	zinc finger protein 551	0.52	0.5	1.09	0.4
PACSIN2	PACSIN2;SDPII	protein kinase C and casein kinase substrate in neurons 2	0.52	0.56	1.09	0.39
ARPP21	ARPP21;ARPP-21;FLJ32997;R3HDM3;RCS;TARPP	cAMP-regulated phosphoprotein, 21kDa	0.52	0.58	1.09	0.42
TONSL	TONSL;FLJ40087;IKBR;NFKBIL2	tonsoku-like, DNA repair protein	0.52	0.57	1.09	0.38
CREG2	CREG2	cellular repressor of E1A-stimulated genes 2	0.51	0.54	1.09	0.43
PLEKHA4	PLEKHA4;PEPP1	pleckstrin homology domain containing, family A (phosphoinositide binding specific) member 4	0.5	0.57	1.09	0.4
GJB2	GJB2;CX26;DFNA3;DFNA3A;DFNB1;DFNB1A;HID;KID;NSRD1;PPK	gap junction protein, beta 2, 26kDa	0.5	0.53	1.09	0.42
SLC4A11	SLC4A11;BTR1;CHED2;MGC126418;MGC126419;NABC1;dJ794I6.2	solute carrier family 4, sodium borate transporter, member 11	0.5	0.56	1.09	0.43
HAUS4	HAUS4;C14orf94;FLJ20424;FLJ40661	HAUS augmin-like complex, subunit 4	0.5	0.55	1.09	0.4
LDB3	LDB3;CMD1C;CYPHER;FLJ35865;KIAA01613;KIAA0613;LDB3Z1;LDB3Z4;LVNC3;ORACLE;PDLIM6;ZASP	LIM domain binding 3	0.49	0.56	1.09	0.37
38596	SEPT5;CDCREL;CDCREL-1;CDCREL1;H5;HCDCREL-1;PNUTL1	septin 5	0.49	0.53	1.09	0.39
CLCN1	CLCN1;CLC1;MGC138361;MGC142055	chloride channel 1, skeletal muscle	0.48	0.52	1.09	0.41
MKKS	MKKS;BBS6;HMCS;KMS;MKS	McKusick-Kaufman syndrome	0.48	0.56	1.09	0.42
FAM100A	FAM100A;FLJ31223;FLJ32185	family with sequence similarity 100, member A	0.48	0.52	1.09	0.38
ATP2B2	ATP2B2;PMCA2;PMCA2a;PMCA2i	ATPase, Ca++ transporting, plasma membrane 2	0.48	0.54	1.09	0.41
NINJ1	NINJ1;NIN1;NINJURIN	ninjurin 1	0.48	0.52	1.09	0.4
RBFOX2	RBFOX2;FOX2;Fox-2;HNRBP2;HRNBP2;RBM9;RTA;dJ106I20.3;fxh	RNA binding protein, fox-1 homolog (C. elegans) 2	0.48	0.51	1.09	0.39
DIEXF	DIEXF;C1orf107;DEF;DJ434O14.5;DKFZp686E19106;FLJ12704;FLJ41680;MGC29875;UTP25	digestive organ expansion factor homolog (zebrafish)	0.48	0.52	1.09	0.38
LRRCC1	LRRCC1;CLERC;KIAA1764;SAP2	leucine rich repeat and coiled-coil domain containing 1	0.47	0.56	1.09	0.37
RBM39	RBM39;CAPER;CAPERalpha;DKFZp781C0423;FLJ44170;FSAP59;HCC1;RNPC2	RNA binding motif protein 39	0.47	0.49	1.09	0.39
LRRC34	LRRC34;FLJ27346;MGC27085	leucine rich repeat containing 34	0.46	0.53	1.09	0.43
AGT	AGT;ANHU;FLJ92595;FLJ97926;SERPINA8	angiotensinogen (serpin peptidase inhibitor, clade A, member 8)	0.46	0.49	1.09	0.36
TOPBP1	TOPBP1;TOP2BP1	topoisomerase (DNA) II binding protein 1	0.46	0.5	1.09	0.38
RPL8	RPL8;L8	ribosomal protein L8	0.46	0.44	1.09	0.37
SPANXA2	SPANXA2;CT11.1;SPAN-X;SPANX;SPANX-A;SPANXA;SPANXA1	SPANX family, member A2	0.45	0.52	1.09	0.42
GSR	GSR;MGC78522	glutathione reductase	0.45	0.48	1.09	0.38
C17orf59	C17orf59;FLJ20014;PRO2472	chromosome 17 open reading frame 59	0.44	0.45	1.09	0.37
RAB1B	RAB1B	RAB1B, member RAS oncogene family	0.44	0.46	1.09	0.4
ITIH4	ITIH4;DKFZp686G21125;GP120;H4P;IHRP;ITI-HC4;ITIHL1;PK-120;PK120	inter-alpha-trypsin inhibitor heavy chain family, member 4	0.42	0.46	1.09	0.31
TMEM219	TMEM219	transmembrane protein 219	0.42	0.5	1.09	0.33
SF3A2	SF3A2;PRP11;PRPF11;SAP62;SF3a66	splicing factor 3a, subunit 2, 66kDa	0.42	0.48	1.09	0.39
CBLN2	CBLN2	cerebellin 2 precursor	0.4	0.46	1.09	0.31
SNRPB	SNRPB;COD;SNRPB1;Sm-B/B';SmB/B';SmB/SmB';snRNP-B	small nuclear ribonucleoprotein polypeptides B and B1	0.4	0.44	1.09	0.34
DGKE	DGKE;DAGK5;DAGK6;DGK	diacylglycerol kinase, epsilon 64kDa	0.38	0.39	1.09	0.3
OTOF	OTOF;AUNB1;DFNB6;DFNB9;FER1L2;NSRD9	otoferlin	0.37	0.42	1.09	0.29
MYT1	MYT1;C20orf36;MTF1;MYTI;PLPB1	myelin transcription factor 1	0.36	0.42	1.09	0.31
IGSF9	IGSF9;FP18798;IGSF9A;KIAA1355;Nrt1	immunoglobulin superfamily, member 9	0.33	0.36	1.09	0.28
PRPF38A	PRPF38A;FLJ14936;MGC3320;Prp38;RP5-965L7.1	PRP38 pre-mRNA processing factor 38 (yeast) domain containing A	0.33	0.34	1.09	0.28
C10orf120	C10orf120;bA318C4.1	chromosome 10 open reading frame 120	0.6	0.62	1.1	0.48
FIBIN	FIBIN;MGC24932	fin bud initiation factor homolog (zebrafish)	0.6	0.64	1.1	0.49
CCDC42	CCDC42;CCDC42A;FLJ32734	coiled-coil domain containing 42	0.6	0.66	1.1	0.48
PCDHB11	PCDHB11;ME2;MGC138337;MGC142171;PCDH-BETA11	protocadherin beta 11	0.6	0.69	1.1	0.56
LBX1	LBX1;HPX-6;HPX6;LBX1H;homeobox	ladybird homeobox 1	0.6	0.66	1.1	0.49
RAB15	RAB15	RAB15, member RAS onocogene family	0.6	0.65	1.1	0.44
SYTL2	SYTL2;CHR11SYT;EXO4;FLJ21219;FLJ34802;FLJ42764;FLJ45597;KIAA1597;MGC102768;SGA72M;SLP2;SLP2A	synaptotagmin-like 2	0.6	0.68	1.1	0.51
MAN2B1	MAN2B1;LAMAN;MANB	mannosidase, alpha, class 2B, member 1	0.6	0.65	1.1	0.56
SLC25A36	SLC25A36;DKFZp564C053;FLJ10618	solute carrier family 25, member 36	0.6	0.65	1.1	0.48
MKI67	MKI67;KIA	antigen identified by monoclonal antibody Ki-67	0.6	0.68	1.1	0.46
ERH	ERH;DROER;FLJ27340	enhancer of rudimentary homolog (Drosophila)	0.6	0.62	1.1	0.48
SCTR	SCTR;SR	secretin receptor	0.59	0.6	1.1	0.45
NARF	NARF;DKFZp434G0420;FLJ10067;IOP2	nuclear prelamin A recognition factor	0.59	0.6	1.1	0.53
OSCAR	OSCAR;MGC33613;PIGR3	osteoclast associated, immunoglobulin-like receptor	0.58	0.62	1.1	0.47
CYP11B1	CYP11B1;CPN1;CYP11B;DKFZp686B05283;FHI;FLJ36771;P450C11	cytochrome P450, family 11, subfamily B, polypeptide 1	0.58	0.6	1.1	0.48
CALCOCO1	CALCOCO1;Cocoa;KIAA1536;calphoglin	calcium binding and coiled-coil domain 1	0.58	0.62	1.1	0.48
EXOSC4	EXOSC4;FLJ20591;RRP41;RRP41A;Rrp41p;SKI6;Ski6p;hRrp41p;p12A	exosome component 4	0.58	0.61	1.1	0.48
CCNJL	CCNJL;FLJ14166	cyclin J-like	0.58	0.6	1.1	0.49
GSS	GSS;GSHS;MGC14098	glutathione synthetase	0.58	0.66	1.1	0.51
IPO11	IPO11;RanBP11	importin 11	0.58	0.64	1.1	0.44
BLCAP	BLCAP;BC10	bladder cancer associated protein	0.58	0.63	1.1	0.52
BORA	BORA;C13orf34;FLJ22624	bora, aurora kinase A activator	0.58	0.68	1.1	0.48
KIAA1370	KIAA1370;FLJ10980;MGC126494;MGC126495	KIAA1370	0.57	0.68	1.1	0.48
ABCB6	ABCB6;ABC;ABC14;FLJ22414;MTABC3;PRP;umat	ATP-binding cassette, sub-family B (MDR/TAP), member 6	0.57	0.62	1.1	0.44
IPP	IPP;KLHL27	intracisternal A particle-promoted polypeptide	0.57	0.67	1.1	0.47
AHCY	AHCY;SAHH	adenosylhomocysteinase	0.56	0.67	1.1	0.5
PRDX1	PRDX1;MSP23;NKEFA;PAG;PAGA;PAGB;PRX1;PRXI;TDPX2	peroxiredoxin 1	0.56	0.57	1.1	0.5
PQLC3	PQLC3;C2orf22;MGC33602	PQ loop repeat containing 3	0.55	0.58	1.1	0.44
CTBP1	CTBP1;BARS;MGC104684	C-terminal binding protein 1	0.55	0.6	1.1	0.42
C19orf42	C19orf42;MGC2747	chromosome 19 open reading frame 42	0.55	0.62	1.1	0.46
ORM1	ORM1;AGP-A;AGP1;ORM	orosomucoid 1	0.54	0.57	1.1	0.41
PEX12	PEX12;PAF-3	peroxisomal biogenesis factor 12	0.54	0.61	1.1	0.44
DLX1	DLX1	distal-less homeobox 1	0.54	0.55	1.1	0.47
PINK1	PINK1;BRPK;FLJ27236;PARK6	PTEN induced putative kinase 1	0.54	0.6	1.1	0.49
ZNF462	ZNF462;DKFZp686B2325;DKFZp762N2316;FLJ14960;FLJ45904;KIAA1803;Zfp462	zinc finger protein 462	0.54	0.62	1.1	0.42
CIRH1A	CIRH1A;CIRHIN;FLJ14728;FLJ17146;KIAA1988;NAIC;TEX292;UTP4	cirrhosis, autosomal recessive 1A (cirhin)	0.54	0.62	1.1	0.41
SLC47A2	SLC47A2;FLJ31196;MATE2;MATE2-B;MATE2-K;MATE2K	solute carrier family 47, member 2	0.53	0.62	1.1	0.44
DSCAML1	DSCAML1;KIAA1132	Down syndrome cell adhesion molecule like 1	0.53	0.58	1.1	0.45
MACF1	MACF1;ABP620;ACF7;FLJ45612;FLJ46776;KIAA0465;KIAA1251;MACF;OFC4	microtubule-actin crosslinking factor 1	0.53	0.61	1.1	0.45
CCND3	CCND3	cyclin D3	0.53	0.54	1.1	0.48
DHCR24	DHCR24;DCE;KIAA0018;Nbla03646;SELADIN1;seladin-1	24-dehydrocholesterol reductase	0.53	0.63	1.1	0.47
APOF	APOF;Apo-F;DKFZp781G18150;LTIP;MGC22520	apolipoprotein F	0.52	0.55	1.1	0.43
CCDC129	CCDC129;FLJ38344	coiled-coil domain containing 129	0.52	0.55	1.1	0.42
BNIP2	BNIP2;BNIP-2;NIP2	BCL2/adenovirus E1B 19kDa interacting protein 2	0.52	0.54	1.1	0.4
BZW1	BZW1;BZAP45;KIAA0005;Nbla10236	basic leucine zipper and W2 domains 1	0.52	0.54	1.1	0.43
HLA-A	HLA-A;FLJ26655;HLAA	major histocompatibility complex, class I, A	0.52	0.62	1.1	0.43
PIGT	PIGT;FLJ41596;MGC8909;NDAP	phosphatidylinositol glycan anchor biosynthesis, class T	0.51	0.58	1.1	0.47
ENDOG	ENDOG;FLJ27463	endonuclease G	0.5	0.53	1.1	0.39
RAB11B	RAB11B;H-YPT3;MGC133246	RAB11B, member RAS oncogene family	0.5	0.6	1.1	0.42
LRRC18	LRRC18;MGC34773;UNQ933;UNQ9338;VKGE9338	leucine rich repeat containing 18	0.48	0.51	1.1	0.39
SLC46A2	SLC46A2;Ly110;TSCOT	solute carrier family 46, member 2	0.48	0.5	1.1	0.39
SLC45A3	SLC45A3;IPCA-2;IPCA-6;IPCA-8;IPCA6;PCANAP2;PCANAP6;PCANAP8;PRST	solute carrier family 45, member 3	0.48	0.48	1.1	0.38
SECISBP2	SECISBP2;DKFZp686C09169;SBP2	SECIS binding protein 2	0.48	0.52	1.1	0.4
SUGT1	SUGT1;SGT1	SGT1, suppressor of G2 allele of SKP1 (S. cerevisiae)	0.48	0.52	1.1	0.43
FAM24A	FAM24A	family with sequence similarity 24, member A	0.46	0.45	1.1	0.36
ARC	ARC;Arg3.1;KIAA0278	activity-regulated cytoskeleton-associated protein	0.46	0.55	1.1	0.41
RPL30	RPL30;L30	ribosomal protein L30	0.46	0.53	1.1	0.36
CTBS	CTBS;CTB	chitobiase, di-N-acetyl-	0.45	0.48	1.1	0.42
PAX7	PAX7;FLJ37460;HUP1;PAX7B;RMS2	paired box 7	0.44	0.48	1.1	0.34
NAV2	NAV2;FLJ10633;FLJ11030;FLJ23707;FLJ77876;HELAD1;KIAA1419;POMFIL2;RAINB1;STEERIN2;UNC53H2	neuron navigator 2	0.44	0.5	1.1	0.33
YIF1B	YIF1B;FLJ23477;FLJ50074;FinGER8;MGC39135	Yip1 interacting factor homolog B (S. cerevisiae)	0.44	0.48	1.1	0.35
SLC30A9	SLC30A9;C4orf1;GAC63;HUEL;ZNT9	solute carrier family 30 (zinc transporter), member 9	0.43	0.48	1.1	0.39
CHD8	CHD8;DKFZp686N17164;HELSNF1;KIAA1564	chromodomain helicase DNA binding protein 8	0.42	0.5	1.1	0.37
PRY2	PRY2;PRY;PTPN13LY2	PTPN13-like, Y-linked 2	0.42	0.48	1.1	0.36
WAC	WAC;BM-016;MGC10753;PRO1741;Wwp4	WW domain containing adaptor with coiled-coil	0.4	0.43	1.1	0.32
PLA2G16	PLA2G16;AdPLA;H-REV107-1;HRASLS3;HREV107;HREV107-1;HREV107-3;HRSL3;MGC118754	phospholipase A2, group XVI	0.4	0.45	1.1	0.33
PAX8	PAX8	paired box 8	0.39	0.44	1.1	0.29
MAST2	MAST2;FLJ39200;KIAA0807;MAST205;MTSSK;RP4-533D7.1	microtubule associated serine/threonine kinase 2	0.37	0.44	1.1	0.31
TNFRSF13B	TNFRSF13B;CD267;CVID;CVID2;FLJ39942;MGC133214;MGC39952;TACI;TNFRSF14B	tumor necrosis factor receptor superfamily, member 13B	0.34	0.35	1.1	0.28
SELL	SELL;CD62L;LAM1;LECAM1;LEU8;LNHR;LSEL;LYAM1;PLNHR;TQ1	selectin L	0.6	0.7	1.11	0.49
BTBD16	BTBD16;C10orf87;FLJ25359;MGC129864;MGC129865	BTB (POZ) domain containing 16	0.6	0.71	1.11	0.5
DPEP3	DPEP3;MBD3	dipeptidase 3	0.6	0.68	1.11	0.5
MYEF2	MYEF2;FLJ11213;HsT18564;KIAA1341;MEF-2;MGC87325;MST156;MSTP156	myelin expression factor 2	0.6	0.6	1.11	0.5
CFC1B	CFC1B	cripto, FRL-1, cryptic family 1B	0.6	0.67	1.11	0.47

FZD2	FZD2;Fz2;fz-2;fzE2;hFz2	frizzled family receptor 2	0.6	0.62	1.11	0.46
MTF2	MTF2;M96;PCL2;RP5-976O13.1;dJ976O13.2	metal response element binding transcription factor 2	0.6	0.66	1.11	0.59
WFDC5	WFDC5;PRG5;WAP1;dJ211D12.5	WAP four-disulfide core domain 5	0.59	0.68	1.11	0.46
PRPF38B	PRPF38B;FLJ10330;MGC163218;MGC41809;NET1;RP11-293A10.1	PRP38 pre-mRNA processing factor 38 (yeast) domain containing B	0.59	0.62	1.11	0.48
UCK1	UCK1;FLJ12255;URK1	uridine-cytidine kinase 1	0.59	0.7	1.11	0.5
GPR3	GPR3;ACCA	G protein-coupled receptor 3	0.58	0.63	1.11	0.57
KIRREL	KIRREL;FLJ10845;MGC129542;MGC129543;NEPH1	kin of IRRE like (Drosophila)	0.58	0.65	1.11	0.44
BFAR	BFAR;BAR;RNF47	bifunctional apoptosis regulator	0.58	0.64	1.11	0.57
PHB	PHB;PHB1	prohibitin	0.58	0.63	1.11	0.54
C9orf128	C9orf128	chromosome 9 open reading frame 128	0.57	0.65	1.11	0.44
USP6NL	USP6NL;KIAA0019;RNTRE;TRE2NL	USP6 N-terminal like	0.57	0.64	1.11	0.43
KIAA0247	KIAA0247	KIAA0247	0.57	0.65	1.11	0.47
NPRL3	NPRL3;C16orf35;CGTHBA;HS-40;MARE;NPR3;RMD11	nitrogen permease regulator-like 3 (S. cerevisiae)	0.57	0.66	1.11	0.5
ACTN2	ACTN2;CMD1AA	actinin, alpha 2	0.56	0.63	1.11	0.49
ST3GAL3	ST3GAL3;MRT12;SIAT6;ST3GALII;ST3GalIII;ST3N	ST3 beta-galactoside alpha-2,3-sialyltransferase 3	0.56	0.62	1.11	0.48
SLC8A1	SLC8A1;DKFZp779F0871;FLJ37694;FLJ43417;MGC119581;NCX1	solute carrier family 8 (sodium/calcium exchanger), member 1	0.56	0.58	1.11	0.52
RALGPS2	RALGPS2;FLJ10244;FLJ25604;KIAA0351;dJ595C2.1	Ral GEF with PH domain and SH3 binding motif 2	0.56	0.63	1.11	0.42
GPAA1	GPAA1;GAA1;hGAA1	glycosylphosphatidylinositol anchor attachment protein 1 homolog (yeast)	0.56	0.6	1.11	0.45
YWHAH	YWHAH;YWHA1	tyrosine 3-monooxygenase/tryptophan 5-monooxygenase activation protein, eta polypeptide	0.56	0.62	1.11	0.48
NTF3	NTF3;HDNF;MGC129711;NGF-2;NGF2;NT3	neurotrophin 3	0.55	0.57	1.11	0.45
C1D	C1D;LRP1;MGC12261;MGC14659;SUN-CoR;SUNCOR;hC1D	C1D nuclear receptor corepressor	0.55	0.64	1.11	0.48
CIDEB	CIDEB	cell death-inducing DFFA-like effector b	0.54	0.62	1.11	0.47
SYT1	SYT1;DKFZp781D2042;P65;SVP65;SYT	synaptotagmin I	0.54	0.55	1.11	0.49
KAAG1	KAAG1;MGC78738;RU2AS	kidney associated antigen 1	0.54	0.56	1.11	0.48
C8orf76	C8orf76;FLJ14825;MGC9784	chromosome 8 open reading frame 76	0.54	0.64	1.11	0.45
KIF4A	KIF4A;FLJ12530;FLJ12655;FLJ14204;FLJ20631;KIF4;KIF4G1	kinesin family member 4A	0.54	0.62	1.11	0.49
CAPN12	CAPN12;MGC20576	calpain 12	0.53	0.55	1.11	0.48
CRYBA2	CRYBA2	crystallin, beta A2	0.52	0.58	1.11	0.39
PARP16	PARP16;C15orf30;FLJ20509;FLJ25281;pART15	poly (ADP-ribose) polymerase family, member 16	0.51	0.55	1.11	0.41
LZTS2	LZTS2;KIAA1813;LAPSER1	leucine zipper, putative tumor suppressor 2	0.51	0.62	1.11	0.45
RTCD1	RTCD1;RPC	RNA terminal phosphate cyclase domain 1	0.5	0.52	1.11	0.46
NSUN2	NSUN2;FLJ20303;MISU;SAKI;TRM4	NOP2/Sun domain family, member 2	0.5	0.58	1.11	0.41
RASD1	RASD1;AGS1;DEXRAS1;MGC:26290	RAS, dexamethasone-induced 1	0.49	0.53	1.11	0.41
TLR4	TLR4;ARMD10;CD284;TOLL;hToll	toll-like receptor 4	0.48	0.5	1.11	0.4
PHPT1	PHPT1;CGI-202;DKFZp564M173;HSPC141;PHP14;RP11-216L13.10;bA216L13.10	phosphohistidine phosphatase 1	0.48	0.5	1.11	0.38
C12orf29	C12orf29;DKFZp313K0436;DKFZp434N2030;DKFZp686L04169;FLJ38158;MGC102978	chromosome 12 open reading frame 29	0.48	0.56	1.11	0.39
ZHX2	ZHX2;AFR1;KIAA0854;RAF	zinc fingers and homeoboxes 2	0.48	0.52	1.11	0.39
CES2	CES2;CE-2;CES2A1;PCE-2;iCE	carboxylesterase 2	0.48	0.52	1.11	0.37
MTO1	MTO1	mitochondrial translation optimization 1 homolog (S. cerevisiae)	0.48	0.52	1.11	0.41
PCF11	PCF11;KIAA0824	PCF11, cleavage and polyadenylation factor subunit, homolog (S. cerevisiae)	0.48	0.55	1.11	0.38
EDN2	EDN2;ET2;PPET2	endothelin 2	0.48	0.52	1.11	0.42
GNAS	GNAS;AHO;C20orf45;GNAS1;GPSA;GSA;GSP;MGC33735;NESP;PHP1A;PHP1B;PHP1C;POH	GNAS complex locus	0.48	0.52	1.11	0.4
PPIA	PPIA;CYPA;CYPH;MGC117158;MGC12404;MGC23397	peptidylprolyl isomerase A (cyclophilin A)	0.48	0.52	1.11	0.41
SDHAF1	SDHAF1	succinate dehydrogenase complex assembly factor 1	0.48	0.58	1.11	0.43
C2orf27B	C2orf27B;MGC50273	chromosome 2 open reading frame 27B	0.46	0.5	1.11	0.39
SHISA4	SHISA4;C1orf40;FLJ18233;MGC102893;MGC129812;MGC129813;TMEM58	shisa homolog 4 (Xenopus laevis)	0.45	0.51	1.11	0.38
PIK3C2A	PIK3C2A;CPK;DKFZp686L193;MGC142218;PI3-K-C2(ALPHA);PI3-K-C2A	phosphoinositide-3-kinase, class 2, alpha polypeptide	0.45	0.54	1.11	0.38
RPL7A	RPL7A;L7A;SURF3;TRUP	ribosomal protein L7a	0.45	0.55	1.11	0.4
PLEKHG3	PLEKHG3;ARHGEF43;KIAA0599;MGC156299;MGC156300	pleckstrin homology domain containing, family G (with RhoGef domain) member 3	0.44	0.46	1.11	0.39
SHISA3	SHISA3;hShisa3	shisa homolog 3 (Xenopus laevis)	0.42	0.48	1.11	0.32
IMPA2	IMPA2	inositol(myo)-1(or 4)-monophosphatase 2	0.42	0.43	1.11	0.32
GRSF1	GRSF1;FLJ13125	G-rich RNA sequence binding factor 1	0.42	0.5	1.11	0.4
FSCN1	FSCN1;FAN1;FLJ38511;FLJ43305;HSN;SNL;p55	fascin homolog 1, actin-bundling protein (Strongylocentrotus purpuratus)	0.42	0.48	1.11	0.36
PSMB6	PSMB6;DELTA;LMPY;MGC5169;Y	proteasome (prosome, macropain) subunit, beta type, 6	0.42	0.44	1.11	0.33
COPZ1	COPZ1;COPZ;zeta1-COP	coatomer protein complex, subunit zeta 1	0.42	0.42	1.11	0.35
NMUR2	NMUR2;FM-4;FM4;NMU-R2;NMU2R;TGR-1;TGR1	neuromedin U receptor 2	0.41	0.45	1.11	0.35
MPV17L	MPV17L;FLJ39599;M-LPH;MGC70356;MLPH1;MLPH2;MPV17L1	MPV17 mitochondrial membrane protein-like	0.4	0.48	1.11	0.32
PLXNB1	PLXNB1;KIAA0407;MGC149167;PLEXIN-B1;PLXN5;SEP	plexin B1	0.38	0.46	1.11	0.3
DAD1	DAD1;OST2	defender against cell death 1	0.38	0.4	1.11	0.32
RSPO4	RSPO4;C20orf182;CRISTIN4;FLJ16018	R-spondin 4	0.37	0.44	1.11	0.31
MATN4	MATN4;FLJ14417;HE6WCR54	matrilin 4	0.36	0.4	1.11	0.28
PXN	PXN;FLJ16691	paxillin	0.36	0.44	1.11	0.31
RUVBL2	RUVBL2;ECP51;INO80J;REPTIN;RVB2;TIH2;TIP48;TIP49B	RuvB-like 2 (E. coli)	0.3	0.32	1.11	0.25
RPS3A	RPS3A;FTE1;MFTL;MGC23240;S3A	ribosomal protein S3A	0.29	0.32	1.11	0.27
RGS7BP	RGS7BP;R7BP	regulator of G-protein signaling 7 binding protein	0.6	0.66	1.12	0.48
CNTD1	CNTD1;CNTD;FLJ40137	cyclin N-terminal domain containing 1	0.6	0.68	1.12	0.51
MTERFD2	MTERFD2;FLJ16261;MGC61716	MTERF domain containing 2	0.6	0.6	1.12	0.47
TAF2	TAF2;CIF150;TAF2B;TAFII150	TAF2 RNA polymerase II, TATA box binding protein (TBP)-associated factor, 150kDa	0.59	0.64	1.12	0.55
FEN1	FEN1;FEN-1;MF1;RAD2	flap structure-specific endonuclease 1	0.59	0.64	1.12	0.56
TLX1NB	TLX1NB;APT-B7;TD1;TDI	TLX1 neighbor	0.59	0.63	1.12	0.46
RPAIN	RPAIN;FLJ25625;FLJ30490;FLJ42429;HRIP;MGC4189;RIP	RPA interacting protein	0.58	0.62	1.12	0.5
PAPSS2	PAPSS2;ATPSK2;SK2	3'-phosphoadenosine 5'-phosphosulfate synthase 2	0.58	0.66	1.12	0.54
SOX17	SOX17;FLJ22252;VUR3	SRY (sex determining region Y)-box 17	0.58	0.66	1.12	0.5
SLC7A5	SLC7A5;4F2LC;CD98;D16S469E;E16;LAT1;MPE16;hLAT1	solute carrier family 7 (amino acid transporter light chain, L system), member 5	0.58	0.71	1.12	0.53
EFCAB11	EFCAB11;C14orf143;DKFZp547I1415;FLJ51529	EF-hand calcium binding domain 11	0.58	0.68	1.12	0.48
OR56A1	OR56A1;OR11-75	olfactory receptor, family 56, subfamily A, member 1	0.57	0.6	1.12	0.51
OR6K3	OR6K3;OR1-18	olfactory receptor, family 6, subfamily K, member 3	0.57	0.66	1.12	0.51
RGS9BP	RGS9BP;FLJ45744;PERRS;R9AP;RGS9	regulator of G protein signaling 9 binding protein	0.57	0.7	1.12	0.46
FAM169B	FAM169B;FLJ39743	family with sequence similarity 169, member B	0.57	0.66	1.12	0.45
ZNF467	ZNF467;EZI;Zfp467	zinc finger protein 467	0.57	0.68	1.12	0.52
C18orf55	C18orf55	chromosome 18 open reading frame 55	0.57	0.66	1.12	0.5
COX4NB	COX4NB;C16orf2;C16orf4;FAM158B;NOC4	COX4 neighbor	0.57	0.68	1.12	0.48
KIR3DP1	KIR3DP1	ENTREZ STATUS: REMAPPED TO GENE ID: 548594	0.57	0.64	1.12	0.45
APOBEC2	APOBEC2;ARCD1;ARP1	apolipoprotein B mRNA editing enzyme, catalytic polypeptide-like 2	0.56	0.62	1.12	0.42
ZNF772	ZNF772;DKFZp686I1569	zinc finger protein 772	0.56	0.62	1.12	0.46
FOXB2	FOXB2;bA159H20.4	forkhead box B2	0.56	0.62	1.12	0.45
PFKP	PFKP;FLJ40226;FLJ44165;PFK-C;PFKF	phosphofructokinase, platelet	0.56	0.64	1.12	0.52
MYC	MYC;MRTL;bHLHe39;c-Myc	v-myc myelocytomatosis viral oncogene homolog (avian)	0.56	0.64	1.12	0.49
RIOK1	RIOK1;AD034;FLJ30006;MGC12903;RRP10;bA288G3.1	RIO kinase 1 (yeast)	0.56	0.62	1.12	0.52
PAK3	PAK3;CDKN1A;MRX30;MRX47;OPHN3;PAK3beta;bPAK;hPAK3	p21 protein (Cdc42/Rac)-activated kinase 3	0.55	0.66	1.12	0.47
KRT6A	KRT6A;CK6A;CK6C;CK6D;K6A;K6C;K6D;KRT6C;KRT6D	keratin 6A	0.55	0.65	1.12	0.53
ALS2	ALS2;ALS2CR6;ALSJ;FLJ31851;IAHSP;KIAA1563;MGC87187;PLSJ	amyotrophic lateral sclerosis 2 (juvenile)	0.55	0.68	1.12	0.5
LRRC42	LRRC42;MGC8974;dJ167A19.4	leucine rich repeat containing 42	0.55	0.58	1.12	0.48
MAD2L2	MAD2L2;MAD2B;REV7	MAD2 mitotic arrest deficient-like 2 (yeast)	0.55	0.59	1.12	0.46
CPPED1	CPPED1;CSTP1;FLJ11151	calcineurin-like phosphoesterase domain containing 1	0.55	0.6	1.12	0.52
MAG	MAG;GMA;S-MAG;SIGLEC-4A;SIGLEC4A	myelin associated glycoprotein	0.54	0.58	1.12	0.46
SIT1	SIT1;MGC125908;MGC125909;MGC125910;RP11-331F9.5;SIT	signaling threshold regulating transmembrane adaptor 1	0.54	0.64	1.12	0.45
HDAC2	HDAC2;HD2;RPD3;YAF1	histone deacetylase 2	0.54	0.66	1.12	0.46
IZUMO4	IZUMO4;C19orf36;IMAGE:4215339	IZUMO family member 4	0.54	0.66	1.12	0.46
DDX28	DDX28;FLJ11282;MDDX28	DEAD (Asp-Glu-Ala-Asp) box polypeptide 28	0.53	0.62	1.12	0.48
MTHFSD	MTHFSD;FLJ12998;FLJ13893;MGC138262;MGC138264;MGC177233	methenyltetrahydrofolate synthetase domain containing	0.53	0.63	1.12	0.44
APTX	APTX;AOA;AOA1;AXA1;EAOH;EOAHA;FHA-HIT;FLJ20157;MGC1072	aprataxin	0.53	0.58	1.12	0.45
CHMP4C	CHMP4C;MGC22825;SNF7-3;Shax3;VPS32C	charged multivesicular body protein 4C	0.53	0.64	1.12	0.49
SERPINA4	SERPINA4;KAL;KLST;KST;PI-4;PI4;kallistatin	serpin peptidase inhibitor, clade A (alpha-1 antiproteinase, antitrypsin), member 4	0.52	0.64	1.12	0.44
ZNF354B	ZNF354B;FLJ25008;KID2;MGC138316	zinc finger protein 354B	0.52	0.6	1.12	0.46
RAI1	RAI1;DKFZp434A139;KIAA1820;MGC12824;SMCR;SMS	retinoic acid induced 1	0.52	0.61	1.12	0.43
LNX2	LNX2;FLJ12933;FLJ23932;FLJ38000;MGC46315;PDZRN1	ligand of numb-protein X 2	0.51	0.56	1.12	0.51
USP25	USP25;USP21	ubiquitin specific peptidase 25	0.51	0.52	1.12	0.4
F11	F11;FXI;MGC141891	coagulation factor XI	0.48	0.54	1.12	0.4
SLC16A5	SLC16A5;MCT5;MCT6	solute carrier family 16, member 5 (monocarboxylic acid transporter 6)	0.48	0.56	1.12	0.44
LIAS	LIAS;LAS;LIP1;LS;MGC23245	lipoic acid synthetase	0.48	0.52	1.12	0.41
PUF60	PUF60;FIR;FLJ31379;RoBPI;SIAHBP1	poly-U binding splicing factor 60KDa	0.44	0.5	1.12	0.34
PRB1	PRB1;PM;PMF;PMS;PRB1L;PRB1M	proline-rich protein BstNI subfamily 1	0.43	0.5	1.12	0.33
NR2C1	NR2C1;TR2	nuclear receptor subfamily 2, group C, member 1	0.43	0.46	1.12	0.4
PPDPF	PPDPF;C20orf149;FLJ21046;MGC2479;dJ697K14.9	pancreatic progenitor cell differentiation and proliferation factor homolog (zebrafish)	0.43	0.46	1.12	0.33
CYP39A1	CYP39A1	cytochrome P450, family 39, subfamily A, polypeptide 1	0.42	0.45	1.12	0.33
FTHL17	FTHL17;CT38	ferritin, heavy polypeptide-like 17	0.42	0.42	1.12	0.34
SNAP23	SNAP23;HsT17016;SNAP-23;SNAP23A;SNAP23B	synaptosomal-associated protein, 23kDa	0.42	0.46	1.12	0.35
QSOX1	QSOX1;FLJ34858;Q6;QSCN6	quiescin Q6 sulfhydryl oxidase 1	0.42	0.45	1.12	0.33
RCL1	RCL1;RNAC;RPCL1	RNA terminal phosphate cyclase-like 1	0.41	0.5	1.12	0.35
MEPCE	MEPCE;BCDIN3;FLJ20257	methylphosphate capping enzyme	0.4	0.45	1.12	0.3
C6orf105	C6orf105;ADTRP;MGC12335;dJ413H6.1	chromosome 6 open reading frame 105	0.39	0.43	1.12	0.36
CDH16	CDH16	cadherin 16, KSP-cadherin	0.39	0.42	1.12	0.3
UBL7	UBL7;BMSC-UbP;MGC14421;TCBA1	ubiquitin-like 7 (bone marrow stromal cell-derived)	0.38	0.42	1.12	0.31
RPS5	RPS5;S5	ribosomal protein S5	0.38	0.45	1.12	0.36
RPL24	RPL24;L24	ribosomal protein L24	0.34	0.4	1.12	0.3
MIOX	MIOX;ALDRL6;MGC90217	myo-inositol oxygenase	0.32	0.34	1.12	0.24
SIN3B	SIN3B;KIAA0700	SIN3 homolog B, transcription regulator (yeast)	0.32	0.32	1.12	0.26
MYBL2	MYBL2;B-MYB;BMYB;MGC15600	v-myb myeloblastosis viral oncogene homolog (avian)-like 2	0.32	0.34	1.12	0.3

MRPS6	MRPS6;C21orf101;MRP-S6;RPMS6;S6mt	mitochondrial ribosomal protein S6	0.32	0.35	1.12	0.27
ZNF576	ZNF576;FLJ22700;FLJ56397;MGC2508	zinc finger protein 576	0.1	0.12	1.12	0.08
TSPY2	TSPY2;MGC103998;TSPYQ1	testis specific protein, Y-linked 2	0.6	0.69	1.13	0.5
STEAP2	STEAP2;IPCA1;PCANAP1;PUMPCn;STAMP1;STMP	STEAP family member 2, metalloreductase	0.6	0.68	1.13	0.53
PHOX2A	PHOX2A;ARIX;CFEOM2;FEOM2;MGC52227;NCAM2;PMX2A	paired-like homeobox 2a	0.6	0.65	1.13	0.57
HIST1H3G	HIST1H3G;H3/h;H3FH	histone cluster 1, H3g	0.6	0.69	1.13	0.53
CSRP2	CSRP2;CRP2;LMO5;SmLIM	cysteine and glycine-rich protein 2	0.6	0.66	1.13	0.47
ABHD13	ABHD13;BEM46L1;C13orf6;FLJ14906;MGC27058;RP11-153I24.2;bA153I24.2	abhydrolase domain containing 13	0.6	0.66	1.13	0.57
OSBPL8	OSBPL8;DKFZp686A11164;MGC126578;MGC133203;MST120;MSTP120;ORP8;OSBP10	oxysterol binding protein-like 8	0.6	0.66	1.13	0.52
GTF2E1	GTF2E1;FE;TF2E1;TFIIE-A	general transcription factor IIE, polypeptide 1, alpha 56kDa	0.6	0.63	1.13	0.56
C10orf91	C10orf91;RP11-432J24.4;bA432J24.4	chromosome 10 open reading frame 91	0.59	0.68	1.13	0.49
SDS	SDS;SDH	serine dehydratase	0.59	0.62	1.13	0.54
IL4I1	IL4I1;FIG1	interleukin 4 induced 1	0.59	0.62	1.13	0.55
ADAMTS10	ADAMTS10;ADAM-TS10;WMS;WMS1	ADAM metallopeptidase with thrombospondin type 1 motif, 10	0.58	0.65	1.13	0.49
MDS1	MDS1	ENTREZ STATUS: REMAPPED TO GENE ID: 2122	0.58	0.64	1.13	0.47
C3orf52	C3orf52;FLJ23186;TTMP	chromosome 3 open reading frame 52	0.58	0.59	1.13	0.46
KREMEN1	KREMEN1;FLJ31863;KREMEN;KRM1	kringle containing transmembrane protein 1	0.58	0.64	1.13	0.53
GCAT	GCAT;KBL;MGC23053	glycine C-acetyltransferase	0.58	0.6	1.13	0.53
RORA	RORA;DKFZp686M2414;MGC119326;MGC119329;NR1F1;ROR1;ROR2;ROR3;RZR-ALPHA;RZRA	RAR-related orphan receptor A	0.57	0.69	1.13	0.46
GPR162	GPR162;A-2;FLJ78837;GRCA	G protein-coupled receptor 162	0.57	0.64	1.13	0.57
DUSP28	DUSP28;DUSP26;VHP	dual specificity phosphatase 28	0.57	0.67	1.13	0.5
LENG9	LENG9	leukocyte receptor cluster (LRC) member 9	0.57	0.66	1.13	0.51
STOX2	STOX2;DKFZp762K222	storkhead box 2	0.57	0.69	1.13	0.48
SIL1	SIL1;BAP;MSS;ULG5	SIL1 homolog, endoplasmic reticulum chaperone (S. cerevisiae)	0.57	0.67	1.13	0.45
GLIPR2	GLIPR2;C9orf19;GAPR-1	GLI pathogenesis-related 2	0.57	0.66	1.13	0.5
TAF7	TAF7;TAF2F;TAFII55	TAF7 RNA polymerase II, TATA box binding protein (TBP)-associated factor, 55kDa	0.57	0.6	1.13	0.48
TRIM38	TRIM38;MGC8946;RNF15;RORET	tripartite motif containing 38	0.57	0.64	1.13	0.57
SERPINB1	SERPINB1;EI;ELANH2;LEI;M/NEI;MNEI;PI2	serpin peptidase inhibitor, clade B (ovalbumin), member 1	0.57	0.62	1.13	0.49
HPRT1	HPRT1;HGPRT;HPRT	hypoxanthine phosphoribosyltransferase 1	0.57	0.64	1.13	0.55
TFPI2	TFPI2;FLJ21164;PP5;REF1;TFPI-2	tissue factor pathway inhibitor 2	0.57	0.61	1.13	0.48
MLNR	MLNR;GPR38;MTLR1	motilin receptor	0.56	0.62	1.13	0.55
GALNT2	GALNT2;GalNAc-T2	UDP-N-acetyl-alpha-D-galactosamine:polypeptide N-acetylgalactosaminyltransferase 2 (GalNAc-T2)	0.56	0.58	1.13	0.52
PSAP	PSAP;FLJ00245;GLBA;MGC110993;SAP1	prosaposin	0.56	0.6	1.13	0.51
PYGM	PYGM	phosphorylase, glycogen, muscle	0.55	0.58	1.13	0.46
IFI27	IFI27;FAM14D;ISG12;ISG12A;P27	interferon, alpha-inducible protein 27	0.55	0.59	1.13	0.43
DVL1	DVL1;DVL;DVL1L1;MGC54245	dishevelled, dsh homolog 1 (Drosophila)	0.55	0.64	1.13	0.43
NCAM1	NCAM1;CD56;MSK39;NCAM	neural cell adhesion molecule 1	0.55	0.6	1.13	0.46
CUZD1	CUZD1;ERG-1;UO-44	CUB and zona pellucida-like domains 1	0.54	0.64	1.13	0.45
DYNC2H1	DYNC2H1;ATD3;DHC1b;DHC2;DNCH2;DYH1B;FLJ11756;hdhc11	dynein, cytoplasmic 2, heavy chain 1	0.54	0.63	1.13	0.47
RIN1	RIN1	Ras and Rab interactor 1	0.54	0.62	1.13	0.45
VAT1L	VAT1L;KIAA1576	vesicle amine transport protein 1 homolog (T. californica)-like	0.54	0.57	1.13	0.46
ZNF787	ZNF787;TIP20	zinc finger protein 787	0.53	0.54	1.13	0.42
COPS3	COPS3;CSN3;SGN3	COP9 constitutive photomorphogenic homolog subunit 3 (Arabidopsis)	0.53	0.62	1.13	0.47
FAM115A	FAM115A;FLJ56782;KIAA0738	family with sequence similarity 115, member A	0.52	0.6	1.13	0.42
CCDC78	CCDC78;C16orf25;FLJ34512;JFP10	coiled-coil domain containing 78	0.52	0.62	1.13	0.48
TBC1D24	TBC1D24;FIME;KIAA1171;MGC102885	TBC1 domain family, member 24	0.52	0.56	1.13	0.43
ATP5F1	ATP5F1;MGC24431;PIG47	ATP synthase, H+ transporting, mitochondrial Fo complex, subunit B1	0.52	0.62	1.13	0.44
KCTD12	KCTD12;C13orf2;FLJ33073;KIAA1778;PFET1;PFETIN	potassium channel tetramerisation domain containing 12	0.52	0.58	1.13	0.45
UBA5	UBA5;FLJ17281;FLJ23251;FLJ23251UBA5;THIFP1;UBE1DC1	ubiquitin-like modifier activating enzyme 5	0.52	0.54	1.13	0.4
TMEM41A	TMEM41A;2900010K02Rik;DKFZp686N06149;DKFZp686N107;MGC15397	transmembrane protein 41A	0.52	0.58	1.13	0.42
SEC23B	SEC23B;CDA-II;CDAII;CDAN2;HEMPAS	Sec23 homolog B (S. cerevisiae)	0.52	0.59	1.13	0.41
CCDC122	CCDC122;FLJ31846	coiled-coil domain containing 122	0.51	0.58	1.13	0.42
RHBG	RHBG;SLC42A2	Rh family, B glycoprotein (gene/pseudogene)	0.51	0.54	1.13	0.46
OR51S1	OR51S1;OR11-24	olfactory receptor, family 51, subfamily S, member 1	0.51	0.56	1.13	0.42
DBR1	DBR1	debranching enzyme homolog 1 (S. cerevisiae)	0.51	0.58	1.13	0.39
EIF3B	EIF3B;EIF3-ETA;EIF3-P110;EIF3-P116;EIF3S9;MGC104664;MGC131875;PRT1	eukaryotic translation initiation factor 3, subunit B	0.51	0.64	1.13	0.44
NPC1L1	NPC1L1;NPC11L1	NPC1 (Niemann-Pick disease, type C1, gene)-like 1	0.5	0.55	1.13	0.39
RNF113B	RNF113B;MGC26599;RNF161;ZNF183L1;bA10G5.1	ring finger protein 113B	0.5	0.55	1.13	0.5
FOXD1	FOXD1;FKHL8;FREAC4	forkhead box D1	0.5	0.55	1.13	0.43
ADORA1	ADORA1;RDC7	adenosine A1 receptor	0.5	0.54	1.13	0.49
TP53I3	TP53I3;PIG3	tumor protein p53 inducible protein 3	0.5	0.55	1.13	0.4
FARSB	FARSB;FARSLB;FRSB;PheHB;PheRS	phenylalanyl-tRNA synthetase, beta subunit	0.5	0.55	1.13	0.4
CTDSPL2	CTDSPL2;FLJ10523;HSPC129	CTD (carboxy-terminal domain, RNA polymerase II, polypeptide A) small phosphatase like 2	0.49	0.6	1.13	0.4
PLXDC1	PLXDC1;DKFZp686F0937;FLJ36270;FLJ45632;TEM3;TEM7	plexin domain containing 1	0.48	0.6	1.13	0.44
C11orf52	C11orf52;FLJ25219;MGC131888;MGC14839	chromosome 11 open reading frame 52	0.48	0.53	1.13	0.4
LYSMD3	LYSMD3;DKFZp686F0735;FLJ13542	LysM, putative peptidoglycan-binding, domain containing 3	0.48	0.54	1.13	0.4
OGDHL	OGDHL	oxoglutarate dehydrogenase-like	0.47	0.5	1.13	0.39
CLDN20	CLDN20	claudin 20	0.46	0.53	1.13	0.41
SPANXE	SPANXE;MGC71908	SPANX family, member E	0.46	0.54	1.13	0.41
PABPC1L2B	PABPC1L2B;MGC168104;PABPC1L2A;RBM32B	poly(A) binding protein, cytoplasmic 1-like 2B	0.46	0.55	1.13	0.45
MAP3K4	MAP3K4;FLJ42439;KIAA0213;MAPKKK4;MEKK4;MTK1;PRO0412	mitogen-activated protein kinase kinase kinase 4	0.45	0.55	1.13	0.39
CCDC157	CCDC157	coiled-coil domain containing 157	0.45	0.46	1.13	0.38
GCHFR	GCHFR;GFRP;HsT16933;MGC138467;MGC138469;P35	GTP cyclohydrolase I feedback regulator	0.44	0.51	1.13	0.39
RHBDD3	RHBDD3;C22orf3;HS984G1A;PTAG	rhomboid domain containing 3	0.44	0.5	1.13	0.36
SBF2	SBF2;CMT4B2;DKFZp779B2327;FLJ22918;FLJ41627;KIAA1766;MTMR13	SET binding factor 2	0.44	0.52	1.13	0.38
ACO2	ACO2;ACONM;MGC20605;MGC33908	aconitase 2, mitochondrial	0.42	0.48	1.13	0.38
EIF3C	EIF3C;EIF3CL;EIF3S8;FLJ53378;FLJ54400;FLJ54404;FLJ55450;FLJ55750;FLJ78287;MGC189737;MGC189744;eIF3-p110	eukaryotic translation initiation factor 3, subunit C	0.42	0.51	1.13	0.34
LCE3D	LCE3D;LEP16;MGC126833;SPRL6A;SPRL6B	late cornified envelope 3D	0.4	0.48	1.13	0.36
RPS3	RPS3;FLJ26283;FLJ27450;MGC87870;S3	ribosomal protein S3	0.38	0.44	1.13	0.31
RPL27	RPL27;L27	ribosomal protein L27	0.36	0.42	1.13	0.28
LDLRAD2	LDLRAD2	low density lipoprotein receptor class A domain containing 2	0.32	0.39	1.13	0.26
TRMT2A	TRMT2A;HTF9C;MGC102728	TRM2 tRNA methyltransferase 2 homolog A (S. cerevisiae)	0.32	0.38	1.13	0.25
HYLS1	HYLS1;FLJ32915;HLS	hydrolethalus syndrome 1	0.3	0.36	1.13	0.26
NXF1	NXF1;DKFZp667O0311;MEX67;TAP	nuclear RNA export factor 1	0.06	0.08	1.13	0.06
SPANXN4	SPANXN4;CT11.9	SPANX family, member N4	0.6	0.74	1.14	0.56
RPL10L	RPL10L;FLJ27353;RPL10_5_1358	ribosomal protein L10-like	0.6	0.74	1.14	0.54
FAM164C	FAM164C;C14orf140;FLJ23093	family with sequence similarity 164, member C	0.6	0.72	1.14	0.58
OVOL2	OVOL2;EUROIMAGE566589;ZNF339	ovo-like 2 (Drosophila)	0.6	0.6	1.14	0.47
PPT2	PPT2;C6orf8;DKFZp564P1516;G14;PPT-2	palmitoyl-protein thioesterase 2	0.6	0.68	1.14	0.53
NFAT5	NFAT5;KIAA0827;NF-AT5;NFATL1;NFATZ;OREBP;TONEBP	nuclear factor of activated T-cells 5, tonicity-responsive	0.6	0.66	1.14	0.47
NGDN	NGDN;C14orf120;DKFZp564O092;LCP5;NGD;lpd-2	neuroguidin, EIF4E binding protein	0.6	0.66	1.14	0.53
PTPLAD1	PTPLAD1;B-IND1;FLJ90376;HACD3;HSPC121	protein tyrosine phosphatase-like A domain containing 1	0.6	0.7	1.14	0.52
NAA15	NAA15;Ga19;NARG1;NATH;TBDN100	N(alpha)-acetyltransferase 15, NatA auxiliary subunit	0.6	0.68	1.14	0.58
DLGAP3	DLGAP3;DAP3;SAPAP3	discs, large (Drosophila) homolog-associated protein 3	0.59	0.7	1.14	0.5
HOXD1	HOXD1;HOX4;HOX4G;Hox-4.7	homeobox D1	0.59	0.7	1.14	0.49
UBE2K	UBE2K;DKFZp564C1216;DKFZp686J24237;E2-25K;HIP2;HYPG;LIG;UBC1	ubiquitin-conjugating enzyme E2K	0.59	0.62	1.14	0.47
SIRPD	SIRPD;PTPNS1L2;dJ576H24.4	signal-regulatory protein delta	0.58	0.66	1.14	0.48
SERPINA13	SERPINA13;UNQ6121	serpin peptidase inhibitor, clade A (alpha-1 antiproteinase, antitrypsin), member 13 (pseudogene)	0.58	0.64	1.14	0.51
GAL3ST1	GAL3ST1;CST	galactose-3-O-sulfotransferase 1	0.58	0.67	1.14	0.45
FURIN	FURIN;FUR;PACE;PCSK3;SPC1	furin (paired basic amino acid cleaving enzyme)	0.58	0.66	1.14	0.49
NBL1	NBL1;D1S1733E;DAN;DAND1;MGC8972;NB;NO3	neuroblastoma, suppression of tumorigenicity 1	0.58	0.65	1.14	0.49
PELI1	PELI1;DKFZp686C18116;MGC50990	pellino homolog 1 (Drosophila)	0.58	0.66	1.14	0.45
SLIT1	SLIT1;MEGF4;MGC164811;SLIL1;SLIT-1;SLIT3	slit homolog 1 (Drosophila)	0.57	0.66	1.14	0.5
FIBCD1	FIBCD1;FLJ14810	fibrinogen C domain containing 1	0.57	0.64	1.14	0.5
CNFN	CNFN;PLAC8L2	cornifelin	0.57	0.71	1.14	0.54
DGCR6	DGCR6	DiGeorge syndrome critical region gene 6	0.57	0.68	1.14	0.45
NUDT1	NUDT1;MTH1	nudix (nucleoside diphosphate linked moiety X)-type motif 1	0.57	0.6	1.14	0.45
SCD	SCD;FADS5;MSTP008;SCD1;SCDOS	stearoyl-CoA desaturase (delta-9-desaturase)	0.57	0.72	1.14	0.49
LAMP1	LAMP1;CD107a;LAMPA;LGP120	lysosomal-associated membrane protein 1	0.57	0.68	1.14	0.5
PREX2	PREX2;6230420N16Rik;DEP.2;DEPDC2;P-REX2	phosphatidylinositol-3,4,5-trisphosphate-dependent Rac exchange factor 2	0.57	0.67	1.14	0.51
SLC35G5	SLC35G5;AMAC;AMAC1L2	solute carrier family 35, member G5	0.57	0.66	1.14	0.45
PRNT	PRNT;M8	prion protein (testis specific)	0.56	0.68	1.14	0.48
TREML1	TREML1;GLTL1825;MGC119173;PRO3438;TLT-1;TLT1;dJ238O23.3	triggering receptor expressed on myeloid cells-like 1	0.56	0.67	1.14	0.46
PEBP4	PEBP4;CORK-1;CORK1;GWTM1933;MGC22776;PEBP-4;PRO4408;hPEBP4	phosphatidylethanolamine-binding protein 4	0.56	0.7	1.14	0.46
MFSD2A	MFSD2A;FLJ14490;FLJ35904;MFSD2	major facilitator superfamily domain containing 2A	0.56	0.7	1.14	0.47
WDR20	WDR20;DKFZp686B2131;DMR;FLJ27314;FLJ33659;MGC33177;MGC33183	WD repeat domain 20	0.55	0.62	1.14	0.47
RBM26	RBM26;ARRS2;C13orf10;FLJ20957;MGC133295;MGC133296;RP11-255E21.1;SE70-2;ZC3H17	RNA binding motif protein 26	0.55	0.6	1.14	0.46
MBNL1	MBNL1;DKFZp686P06174;EXP;EXP35;EXP40;EXP42;KIAA0428;MBNL	muscleblind-like (Drosophila)	0.55	0.57	1.14	0.45
PIP5KL1	PIP5KL1;MGC46424;PIPKH;bA203J24.5	phosphatidylinositol-4-phosphate 5-kinase-like 1	0.54	0.62	1.14	0.52
PRMT2	PRMT2;HRMT1L1;MGC111373	protein arginine methyltransferase 2	0.53	0.6	1.14	0.42
TLE1	TLE1;ESG;ESG1;GRG1	transducin-like enhancer of split 1 (E(sp1) homolog, Drosophila)	0.53	0.6	1.14	0.47
COMMD7	COMMD7;C20orf92;FLJ14987;MGC33315;dJ1085F17.3	COMM domain containing 7	0.53	0.64	1.14	0.45
OR2W5	OR2W5;OR2W5P;OST722	olfactory receptor, family 2, subfamily W, member 5	0.52	0.6	1.14	0.44
CHRNB2	CHRNB2;EFNL3;nAChRB2	cholinergic receptor, nicotinic, beta 2 (neuronal)	0.52	0.64	1.14	0.49
FARP2	FARP2;FIR;FRG;KIAA0793;PLEKHC3	FERM, RhoGEF and pleckstrin domain protein 2	0.52	0.6	1.14	0.43
C9orf23	C9orf23;MGC29635;bA296L22.5	chromosome 9 open reading frame 23	0.52	0.61	1.14	0.46
TESK1	TESK1	testis-specific kinase 1	0.52	0.63	1.14	0.45
C8A	C8A	complement component 8, alpha polypeptide	0.51	0.55	1.14	0.46
ZNF358	ZNF358;FLJ10390;ZFEND	zinc finger protein 358	0.51	0.6	1.14	0.43

ECHDC2	ECHDC2;DKFZp686F0985;DKFZp686K13244;FLJ10948;FLJ45240;FLJ51075;FLJ52213;FLJ52450;FLJ78805;FLJ99576;MGC57155	enoyl CoA hydratase domain containing 2	0.5	0.61	1.14	0.42
C22orf40	C22orf40	chromosome 22 open reading frame 40	0.5	0.6	1.14	0.49
DIAPH1	DIAPH1;DFNA1;DIA1;DRF1;FLJ25265;LFHL1;hDIA1	diaphanous homolog 1 (Drosophila)	0.5	0.55	1.14	0.45
SPRR2F	SPRR2F	small proline-rich protein 2F	0.5	0.54	1.14	0.44
PGCP	PGCP	plasma glutamate carboxypeptidase	0.49	0.52	1.14	0.39
C2orf82	C2orf82;MGC163179;MGC163181;UNQ830	chromosome 2 open reading frame 82	0.49	0.5	1.14	0.4
ANKRD54	ANKRD54;LIAR	ankyrin repeat domain 54	0.48	0.54	1.14	0.39
INTS5	INTS5;INT5;KIAA1698	integrator complex subunit 5	0.48	0.58	1.14	0.41
EIF4E2	EIF4E2;4E-LP;4EHP;EIF4EL3;IF4e	eukaryotic translation initiation factor 4E family member 2	0.48	0.52	1.14	0.45
UBE2M	UBE2M;UBC-RS2;UBC12;hUbc12	ubiquitin-conjugating enzyme E2M	0.46	0.48	1.14	0.36
RPL7	RPL7;L7;MGC117326;humL7-1	ribosomal protein L7	0.46	0.52	1.14	0.39
ACER2	ACER2;ALKCDase2;ASAH3L;FLJ41587	alkaline ceramidase 2	0.46	0.54	1.14	0.39
PF4	PF4;CXCL4;MGC138298;PF-4;SCYB4	platelet factor 4	0.45	0.5	1.14	0.4
PDCD1	PDCD1;CD279;PD-1;PD1;SLEB2;hPD-1;hPD-l	programmed cell death 1	0.45	0.52	1.14	0.36
HNRNPL	HNRNPL;FLJ35509;HNRPL;hnRNP-L	heterogeneous nuclear ribonucleoprotein L	0.45	0.49	1.14	0.35
MAPK1	MAPK1;ERK;ERK2;ERT1;MAPK2;P42MAPK;PRKM1;PRKM2;p38;p40;p41;p41mapk	mitogen-activated protein kinase 1	0.45	0.52	1.14	0.43
SPANXD	SPANXD;CT11.4;MGC119769;SPANX-C;SPANX-D;dJ171K16.1	SPANX family, member D	0.45	0.54	1.14	0.41
KDM6A	KDM6A;DKFZp686A03225;MGC141941;UTX;bA386N14.2	lysine (K)-specific demethylase 6A	0.45	0.53	1.14	0.36
FAM168B	FAM168B;MGC87527	family with sequence similarity 168, member B	0.44	0.51	1.14	0.35
POLR2J3	POLR2J3;MGC54043;POLR2J2;RPB11b1;RPB11b2	polymerase (RNA) II (DNA directed) polypeptide J3	0.44	0.5	1.14	0.4
ZNF792	ZNF792;FLJ38451;MGC117233;MGC119731	zinc finger protein 792	0.42	0.52	1.14	0.34
NMUR2	NMUR2;FM-4;FM4;NMU-R2;NMU2R;TGR-1;TGR1	neuromedin U receptor 2	0.42	0.45	1.14	0.41
THBS4	THBS4;TSP4	thrombospondin 4	0.42	0.47	1.14	0.38
SLC7A6OS	SLC7A6OS;FLJ13291	solute carrier family 7, member 6 opposite strand	0.42	0.5	1.14	0.39
STARD8	STARD8;ARHGAP38;DKFZp686H1668;DLC3;KIAA0189;STARTGAP3	StAR-related lipid transfer (START) domain containing 8	0.4	0.47	1.14	0.32
ESPL1	ESPL1;ESP1;FLJ46492;KIAA0165;SEPA	extra spindle pole bodies homolog 1 (S. cerevisiae)	0.4	0.45	1.14	0.34
SIPA1L2	SIPA1L2;FLJ23126;FLJ23632;KIAA1389;SPAL2	signal-induced proliferation-associated 1 like 2	0.4	0.45	1.14	0.34
EBNA1BP2	EBNA1BP2;EBP2;NOBP;P40	EBNA1 binding protein 2	0.4	0.46	1.14	0.35
ZNF684	ZNF684;MGC27466	zinc finger protein 684	0.39	0.43	1.14	0.33
USP40	USP40;FLJ10785;FLJ42100	ubiquitin specific peptidase 40	0.38	0.42	1.14	0.33
RPL19	RPL19;DKFZp779D216;FLJ27452;L19;MGC71997	ribosomal protein L19	0.37	0.44	1.14	0.36
ZIC3	ZIC3;HTX;HTX1;ZNF203	Zic family member 3	0.36	0.4	1.14	0.28
PSMA7	PSMA7;C6;HSPC;MGC3755;RC6-1;XAPC7	proteasome (prosome, macropain) subunit, alpha type, 7	0.36	0.38	1.14	0.28
HNRNPK	HNRNPK;CSBP;FLJ41122;HNRPK;TUNP	heterogeneous nuclear ribonucleoprotein K	0.34	0.4	1.14	0.27
PEX11G	PEX11G;MGC4281	peroxisomal biogenesis factor 11 gamma	0.31	0.34	1.14	0.26
ATG9A	ATG9A;APG9L1;MGD3208;mATG9	ATG9 autophagy related 9 homolog A (S. cerevisiae)	0.31	0.36	1.14	0.26
SLC13A4	SLC13A4;SUT-1;SUT1	solute carrier family 13 (sodium/sulfate symporters), member 4	0.3	0.33	1.14	0.28
LOXL2	LOXL2;LOR2;WS9-14	lysyl oxidase-like 2	0.29	0.32	1.14	0.22
CDK11A	CDK11A;CDC2L2;CDC2L3;CDK11-p110;CDK11-p46;CDK11-p58;MGC131975;PITSLRE;p58GTA	cyclin-dependent kinase 11A	0.29	0.33	1.14	0.27
PRTN3	PRTN3;ACPA;AGP7;C-ANCA;CANCA;MBN;MBT;NP4;P29;PR-3;PR3	proteinase 3	0.25	0.28	1.14	0.21
CACNA1H	CACNA1H;CACNA1HB;Cav3.2;ECA6;EIG6;FLJ90484	calcium channel, voltage-dependent, T type, alpha 1H subunit	0.6	0.68	1.15	0.53
LHX6	LHX6;LHX6.1;MGC119542;MGC119544;MGC119545	LIM homeobox 6	0.6	0.76	1.15	0.52
ISCA2	ISCA2;HBLD1;ISA2;c14_5557	iron-sulfur cluster assembly 2 homolog (S. cerevisiae)	0.6	0.7	1.15	0.52
XRCC4	XRCC4	X-ray repair complementing defective repair in Chinese hamster cells 4	0.6	0.76	1.15	0.53
ADM	ADM;AM	adrenomedullin	0.6	0.71	1.15	0.54
C6orf106	C6orf106;FLJ22195;FLJ32402;FP852;MGC4614;dJ391O22.4	chromosome 6 open reading frame 106	0.6	0.66	1.15	0.56
ERGIC2	ERGIC2;Erv41;MGC111152;PTX1;cd002	ERGIC and golgi 2	0.6	0.75	1.15	0.5
RIOK2	RIOK2;FLJ11159;RIO2	RIO kinase 2 (yeast)	0.59	0.68	1.15	0.56
LDB2	LDB2;CLIM1;LDB1	LIM domain binding 2	0.58	0.66	1.15	0.56
FST	FST;FS	follistatin	0.58	0.7	1.15	0.57
CA10	CA10;CA-RPX;CARPX;HUCEP-15	carbonic anhydrase X	0.57	0.63	1.15	0.5
SH3YL1	SH3YL1;DKFZp586F1318;FLJ39121;RAY	SH3 domain containing, Ysc84-like 1 (S. cerevisiae)	0.57	0.63	1.15	0.45
C20orf29	C20orf29;FLJ11168	chromosome 20 open reading frame 29	0.57	0.64	1.15	0.5
FGFRL1	FGFRL1;FGFR5;FHFR	fibroblast growth factor receptor-like 1	0.57	0.71	1.15	0.5
NQO2	NQO2;DHQV;DIA6;FLJ39868;NMOR2;QR2	NAD(P)H dehydrogenase, quinone 2	0.57	0.7	1.15	0.47
USP21	USP21;MGC3394;USP16;USP23	ubiquitin specific peptidase 21	0.57	0.64	1.15	0.48
SALL1	SALL1;HSAL1;Sal-1;TBS;ZNF794	sal-like 1 (Drosophila)	0.56	0.63	1.15	0.44
MGAT2	MGAT2;CDG2A;CDGS2;GLCNACTII;GNT-II;GNT2	mannosyl (alpha-1,6-)-glycoprotein beta-1,2-N-acetylglucosaminyltransferase	0.56	0.6	1.15	0.53
SAP30BP	SAP30BP;DKFZp586L2022;HCNGP;HTRG;HTRP	SAP30 binding protein	0.56	0.6	1.15	0.48
NCBP2	NCBP2;CBC2;CBP20;NIP1	nuclear cap binding protein subunit 2, 20kDa	0.56	0.68	1.15	0.47
ILDR2	ILDR2;C1orf32;RP4-782G3.2;dJ782G3.1	immunoglobulin-like domain containing receptor 2	0.56	0.58	1.15	0.46
TRAG3	TRAG3	ENTREZ STATUS: REMAPPED TO GENE ID: 728461	0.56	0.57	1.15	0.45
NEK10	NEK10;FLJ32685	NIMA (never in mitosis gene a)- related kinase 10	0.55	0.64	1.15	0.52
CAPNS2	CAPNS2;MGC12536;MGC14804	calpain, small subunit 2	0.55	0.64	1.15	0.49
UGT1A1	UGT1A1;BILIQTL1;GNT1;HUG-BR1;UDPGT;UDPGT 1-1;UGT1;UGT1A	UDP glucuronosyltransferase 1 family, polypeptide A1	0.55	0.6	1.15	0.53
MIXL1	MIXL1;MGC138179;MILD1;MIX;MIXL	Mix paired-like homeobox	0.55	0.62	1.15	0.48
C18orf21	C18orf21;HsT3108;MGC131953;PNAS-131;XTP13	chromosome 18 open reading frame 21	0.55	0.62	1.15	0.44
ANKRD46	ANKRD46	ankyrin repeat domain 46	0.55	0.6	1.15	0.49
RPUSD4	RPUSD4;FLJ14494	RNA pseudouridylate synthase domain containing 4	0.55	0.66	1.15	0.45
RAI14	RAI14;DKFZp564G013;KIAA1334;NORPEG;RAI13	retinoic acid induced 14	0.55	0.65	1.15	0.49
EIF3I	EIF3I;EIF3S2;PRO2242;TRIP-1;TRIP1;eIF3-beta;eIF3-p36	eukaryotic translation initiation factor 3, subunit I	0.55	0.68	1.15	0.45
RHBDF2	RHBDF2;FLJ22341;RHBDL5;RHBDL6	rhomboid 5 homolog 2 (Drosophila)	0.54	0.55	1.15	0.43
PRPF18	PRPF18;FLJ10210;PRP18;hPrp18	PRP18 pre-mRNA processing factor 18 homolog (S. cerevisiae)	0.54	0.6	1.15	0.47
TEX19	TEX19;FLJ35767	testis expressed 19	0.54	0.65	1.15	0.45
APOBEC3H	APOBEC3H;ARP10	apolipoprotein B mRNA editing enzyme, catalytic polypeptide-like 3H	0.53	0.62	1.15	0.44
SLC25A45	SLC25A45	solute carrier family 25, member 45	0.53	0.6	1.15	0.42
C1orf183	C1orf183;FLJ31105	chromosome 1 open reading frame 183	0.53	0.6	1.15	0.45
HAUS2	HAUS2;C15orf25;CEP27;FLJ10460;HsT17025	HAUS augmin-like complex, subunit 2	0.53	0.65	1.15	0.46
CCDC134	CCDC134;FLJ22349;MGC21013;dJ821D11.3	coiled-coil domain containing 134	0.52	0.61	1.15	0.46
PSMA5	PSMA5;FLJ42315;MGC117302;MGC125802;MGC125803;MGC125804;PSC5;ZETA	proteasome (prosome, macropain) subunit, alpha type, 5	0.52	0.59	1.15	0.44
BFSP1	BFSP1;CP115;CP94;LIFL-H	beaded filament structural protein 1, filensin	0.5	0.58	1.15	0.39
ASIP	ASIP;AGSW;AGTI;AGTIL;ASP;MGC126092;MGC126093;SHEP9	agouti signaling protein	0.5	0.61	1.15	0.41
FXYD2	FXYD2;ATP1G1;HOMG2;MGC12372	FXYD domain containing ion transport regulator 2	0.5	0.57	1.15	0.44
COG8	COG8;CDG2H;DOR1;FLJ22315	component of oligomeric golgi complex 8	0.5	0.56	1.15	0.4
C1orf106	C1orf106;FLJ10901;MGC125608	chromosome 1 open reading frame 106	0.5	0.61	1.15	0.43
PSME4	PSME4;FLJ21864;KIAA0077;MGC138374;MGC142228;PA200	proteasome (prosome, macropain) activator subunit 4	0.5	0.6	1.15	0.45
BCL2L1	BCL2L1;BCL-XL/S;BCL2L;BCLX;BCLXL;BCLXS;Bcl-X;DKFZp781P2092;PPP1R52;bcl-xL;bcl-xS	BCL2-like 1	0.5	0.58	1.15	0.39
C6orf15	C6orf15;STG	chromosome 6 open reading frame 15	0.49	0.56	1.15	0.39
SLC46A3	SLC46A3;FKSG16;FLJ42613	solute carrier family 46, member 3	0.48	0.52	1.15	0.42
REC8	REC8;HR21spB;MGC950;REC8L1;Rec8p	REC8 homolog (yeast)	0.48	0.55	1.15	0.4
UTP18	UTP18;CGI-48;WDR50	UTP18, small subunit (SSU) processome component, homolog (yeast)	0.48	0.5	1.15	0.39
CLCN7	CLCN7;CLC-7;CLC7;FLJ26686;FLJ39644;FLJ46423;OPTA2;OPTB4;PPP1R63	chloride channel 7	0.48	0.57	1.15	0.44
NRD1	NRD1;hNRD1;hNRD2	nardilysin (N-arginine dibasic convertase)	0.48	0.52	1.15	0.39
DNAJB13	DNAJB13;FLJ46748;RSPH16A;TSARG5;TSARG6	DnaJ (Hsp40) homolog, subfamily B, member 13	0.47	0.57	1.15	0.41
RBBP9	RBBP9;BOG;MGC9236;RBBP10	retinoblastoma binding protein 9	0.46	0.53	1.15	0.39
RAB11FIP5	RAB11FIP5;DKFZp434H018;GAF1;KIAA0857;RIP11;pp75	RAB11 family interacting protein 5 (class I)	0.45	0.5	1.15	0.4
C17orf75	C17orf75;NJMU-R1	chromosome 17 open reading frame 75	0.45	0.48	1.15	0.39
SGSH	SGSH;HSS;MPS3A;SFMD	N-sulfoglucosamine sulfohydrolase	0.45	0.55	1.15	0.36
YTHDC2	YTHDC2	YTH domain containing 2	0.45	0.54	1.15	0.43
PIKFYVE	PIKFYVE;CFD;FAB1;FLJ37746;KIAA0981;MGC40423;PIP5K;PIP5K3	phosphoinositide kinase, FYVE finger containing	0.45	0.56	1.15	0.4
C5orf38	C5orf38;CEI;IRX2NB;MGC126657;MGC126683	chromosome 5 open reading frame 38	0.44	0.54	1.15	0.36
SAMD4B	SAMD4B;FLJ10211;MGC99832;SMGB;hSmaug2	sterile alpha motif domain containing 4B	0.44	0.45	1.15	0.36
RPS29	RPS29;S29	ribosomal protein S29	0.42	0.46	1.15	0.39
TUBB3	TUBB3;CDCBM;CFEOM3A;TUBB4;beta-4	tubulin, beta 3 class III	0.42	0.42	1.15	0.33
CSTF2	CSTF2;CstF-64	cleavage stimulation factor, 3' pre-RNA, subunit 2, 64kDa	0.42	0.5	1.15	0.38
RPS20	RPS20;FLJ27451;MGC102930;S20	ribosomal protein S20	0.41	0.45	1.15	0.35
C3orf17	C3orf17;DKFZp434F2021;NET17	chromosome 3 open reading frame 17	0.4	0.5	1.15	0.33
SF3A1	SF3A1;PRP21;PRPF21;SAP114;SF3A120	splicing factor 3a, subunit 1, 120kDa	0.4	0.44	1.15	0.32
SLC6A11	SLC6A11;GAT-3;GAT3;GAT4	solute carrier family 6 (neurotransmitter transporter, GABA), member 11	0.39	0.5	1.15	0.35
IL1B	IL1B;IL-1;IL1-BETA;IL1F2	interleukin 1, beta	0.38	0.43	1.15	0.32
CD5	CD5;LEU1;T1	CD5 molecule	0.38	0.42	1.15	0.31
SLCO2B1	SLCO2B1;DKFZp686E0517;KIAA0880;OATP-B;OATP2B1;OATPB;SLC21A9	solute carrier organic anion transporter family, member 2B1	0.37	0.46	1.15	0.32
AGBL5	AGBL5;FLJ21839	ATP/GTP binding protein-like 5	0.36	0.4	1.15	0.3
OR10G9	OR10G9;OR10G10P	olfactory receptor, family 10, subfamily G, member 9	0.34	0.4	1.15	0.27
SHCBP1	SHCBP1;FLJ22009;MGC26900;PAL	SHC SH2-domain binding protein 1	0.34	0.4	1.15	0.3
TIAF1	TIAF1;MAJN;SPR210	TGFB1-induced anti-apoptotic factor 1	0.34	0.4	1.15	0.27
OR10H1	OR10H1	olfactory receptor, family 10, subfamily H, member 1	0.32	0.37	1.15	0.26
IFNW1	IFNW1	interferon, omega 1	0.31	0.36	1.15	0.24
SP1	SP1	Sp1 transcription factor	0.28	0.32	1.15	0.23
MTA2	MTA2;DKFZp686F2281;MTA1L1;PID	metastasis associated 1 family, member 2	0.18	0.2	1.15	0.14
GUCY1A3	GUCY1A3;GC-SA3;GUC1A3;GUCA3;GUCSA3;GUCY1A1	guanylate cyclase 1, soluble, alpha 3	0.6	0.77	1.16	0.54
CALN1	CALN1;CABP8	calneuron 1	0.6	0.72	1.16	0.49
CHST5	CHST5;I-GlcNAc-6-ST;I-GlcNAc6ST;MGC74625;glcNAc6ST-3;gn6st-3;hIGn6ST	carbohydrate (N-acetylglucosamine 6-O) sulfotransferase 5	0.6	0.67	1.16	0.48
TMEM86A	TMEM86A;FLJ90119	transmembrane protein 86A	0.6	0.72	1.16	0.51
SOLH	SOLH;CAPN15;MGC131491	small optic lobes homolog (Drosophila)	0.6	0.7	1.16	0.52
PIK3C3	PIK3C3;MGC61518;VPS34;hVps34	phosphoinositide-3-kinase, class 3	0.6	0.75	1.16	0.53
RPP14	RPP14;FLJ31508;P14	ribonuclease P/MRP 14kDa subunit	0.6	0.7	1.16	0.55
TATDN3	TATDN3;MGC142198	TatD DNase domain containing 3	0.6	0.68	1.16	0.55
MAP3K11	MAP3K11;MEKK11;MGC17114;MLK-3;MLK3;PTK1;SPRK	mitogen-activated protein kinase kinase kinase 11	0.59	0.74	1.16	0.52

EIF4E	EIF4E;CBP;EIF4E1;EIF4EL1;EIF4F;MGC111573	eukaryotic translation initiation factor 4E	0.58	0.72	1.16	0.47
DEFB132	DEFB132;DEFB32;UNQ827	defensin, beta 132	0.58	0.74	1.16	0.49
USP22	USP22;KIAA1063;USP3L	ubiquitin specific peptidase 22	0.58	0.71	1.16	0.47
STAT3	STAT3;APRF;FLJ20882;HIES;MGC16063	signal transducer and activator of transcription 3 (acute-phase response factor)	0.58	0.64	1.16	0.5
GPX4	GPX4;MCSP;PHGPx;snGPx;snPHGPx	glutathione peroxidase 4 (phospholipid hydroperoxidase)	0.58	0.62	1.16	0.55
MYOF	MYOF;FER1L3;FLJ36571;FLJ90777	myoferlin	0.58	0.74	1.16	0.54
PIH1D2	PIH1D2	PIH1 domain containing 2	0.57	0.64	1.16	0.49
NAT8	NAT8;CML1;GLA;Hcml1;TSC501;TSC510	N-acetyltransferase 8 (GCN5-related, putative)	0.57	0.65	1.16	0.56
FBN2	FBN2;CCA;DA9	fibrillin 2	0.57	0.66	1.16	0.45
GBA	GBA;GBA1;GCB;GLUC	glucosidase, beta, acid	0.57	0.62	1.16	0.55
CTSC	CTSC;CPPI;DPP-I;DPP1;DPPI;HMS;JP;JPD;PALS;PLS	cathepsin C	0.57	0.66	1.16	0.49
ACTN1	ACTN1;FLJ40884;FLJ54432	actinin, alpha 1	0.57	0.7	1.16	0.54
CSGALNACT1	CSGALNACT1;CSGalNAcT-1;ChGn;FLJ11264;FLJ13760;beta4GalNAcT	chondroitin sulfate N-acetylgalactosaminyltransferase 1	0.56	0.65	1.16	0.51
ZUFSP	ZUFSP;C6orf113;dJ412I7.3	zinc finger with UFM1-specific peptidase domain	0.56	0.66	1.16	0.5
TMEM187	TMEM187;CXorf12;DXS9878E;FLJ95849;ITBA1	transmembrane protein 187	0.56	0.61	1.16	0.49
FOXI2	FOXI2;FLJ46831	forkhead box I2	0.56	0.7	1.16	0.47
NDUFS3	NDUFS3;CI-30	NADH dehydrogenase (ubiquinone) Fe-S protein 3, 30kDa (NADH-coenzyme Q reductase)	0.56	0.62	1.16	0.54
TCEAL7	TCEAL7;MGC23947;MPMGp800C04260Q003	transcription elongation factor A (SII)-like 7	0.55	0.6	1.16	0.49
PLCD1	PLCD1;NDNC3	phospholipase C, delta 1	0.55	0.62	1.16	0.44
ARID1B	ARID1B;6A3-5;BAF250B;BRIGHT;DAN15;ELD/OSA1;KIAA1235;OSA2;P250R	AT rich interactive domain 1B (SWI1-like)	0.54	0.62	1.16	0.5
PTPRS	PTPRS;PTPSIGMA	protein tyrosine phosphatase, receptor type, S	0.54	0.62	1.16	0.49
LRP1	LRP1;A2MR;APOER;APR;CD91;FLJ16451;IGFBP3R;LRP;MGC88725;TGFBR5	low density lipoprotein receptor-related protein 1	0.53	0.6	1.16	0.47
PWP2	PWP2;EHOC-17;PWP2H;UTP1	PWP2 periodic tryptophan protein homolog (yeast)	0.53	0.55	1.16	0.43
MYO19	MYO19;FLJ22865;MYOHD1	myosin XIX	0.53	0.64	1.16	0.46
MAPKAP1	MAPKAP1;JC310;MGC2745;MIP1;SIN1;SIN1b;SIN1g	mitogen-activated protein kinase associated protein 1	0.53	0.6	1.16	0.46
C22orf26	C22orf26;FLJ10945	chromosome 22 open reading frame 26	0.52	0.64	1.16	0.46
KIAA1530	KIAA1530;MGC117169	KIAA1530	0.52	0.62	1.16	0.47
C19orf57	C19orf57;MGC11271;MGC149720	chromosome 19 open reading frame 57	0.52	0.58	1.16	0.44
PIF1	PIF1;C15orf20;PIF	PIF1 5'-to-3' DNA helicase homolog (S. cerevisiae)	0.52	0.57	1.16	0.45
IMP3	IMP3;BRMS2;C15orf12;DKFZp586L0118;FLJ10968;MRPS4	IMP3, U3 small nucleolar ribonucleoprotein, homolog (yeast)	0.52	0.57	1.16	0.46
AP2A2	AP2A2;ADTAB;CLAPA2;FLJ37177;HIP-9;HIP9;HYPJ	adaptor-related protein complex 2, alpha 2 subunit	0.52	0.6	1.16	0.42
CDC26	CDC26;ANAPC12;APC12;C9orf17	cell division cycle 26 homolog (S. cerevisiae)	0.52	0.62	1.16	0.42
RER1	RER1	RER1 retention in endoplasmic reticulum 1 homolog (S. cerevisiae)	0.52	0.62	1.16	0.46
CYP2R1	CYP2R1;MGC4663	cytochrome P450, family 2, subfamily R, polypeptide 1	0.51	0.58	1.16	0.43
SLC10A2	SLC10A2;ASBT;IBAT;ISBT;NTCP2;PBAM	solute carrier family 10 (sodium/bile acid cotransporter family), member 2	0.5	0.57	1.16	0.45
CD200	CD200;MOX1;MOX2;MRC;OX-2	CD200 molecule	0.5	0.62	1.16	0.44
ZNF398	ZNF398;KIAA1339;P51;P71;ZER6	zinc finger protein 398	0.5	0.61	1.16	0.46
TTC7A	TTC7A;MGC131720;MGC134830;TTC7	tetratricopeptide repeat domain 7A	0.5	0.63	1.16	0.46
PSMB3	PSMB3;HC10-II;MGC4147	proteasome (prosome, macropain) subunit, beta type, 3	0.49	0.56	1.16	0.42
C20orf132	C20orf132;C20orf131;DKFZp434N0426;FLJ36113;dJ621N11.3;dJ621N11.4	chromosome 20 open reading frame 132	0.48	0.58	1.16	0.47
FBXL22	FBXL22;FLJ39626;Fbl22;MGC75496	F-box and leucine-rich repeat protein 22	0.48	0.52	1.16	0.38
ABLIM3	ABLIM3	actin binding LIM protein family, member 3	0.48	0.52	1.16	0.4
PANX2	PANX2;MGC119432;PX2;hPANX2	pannexin 2	0.48	0.5	1.16	0.4
C8orf31	C8orf31;FLJ37131;MGC90155	chromosome 8 open reading frame 31	0.47	0.58	1.16	0.41
IRF2BP2	IRF2BP2;MGC72189	interferon regulatory factor 2 binding protein 2	0.47	0.56	1.16	0.43
SSR3	SSR3;TRAPG	signal sequence receptor, gamma (translocon-associated protein gamma)	0.47	0.56	1.16	0.42
DLEC1	DLEC1;DLC1;F56	deleted in lung and esophageal cancer 1	0.46	0.57	1.16	0.43
PPIL1	PPIL1;CYPL1;MGC678;PPIase;hCyPX	peptidylprolyl isomerase (cyclophilin)-like 1	0.46	0.59	1.16	0.41
CLDND1	CLDND1;C3orf4;GENX-3745;MGC111162;MGC3316;MGC9861	claudin domain containing 1	0.46	0.55	1.16	0.4
TLR6	TLR6;CD286	toll-like receptor 6	0.44	0.51	1.16	0.41
SPANXC	SPANXC;C;CT11.3;CTp11;SPANX-C	SPANX family, member C	0.44	0.49	1.16	0.35
ENTPD6	ENTPD6;CD39L2;DKFZp781G2277;DKFZp781K21102;FLJ36711;IL-6SAG;IL6ST2;NTPDase-6;dJ738P15.3	ectonucleoside triphosphate diphosphohydrolase 6 (putative)	0.44	0.55	1.16	0.39
POLR3H	POLR3H;KIAA1665;MGC111097;MGC29654;RPC22.9;RPC8	polymerase (RNA) III (DNA directed) polypeptide H (22.9kD)	0.44	0.55	1.16	0.36
GAS1	GAS1	growth arrest-specific 1	0.43	0.48	1.16	0.39
HMGCS2	HMGCS2	3-hydroxy-3-methylglutaryl-CoA synthase 2 (mitochondrial)	0.42	0.44	1.16	0.39
SLU7	SLU7;9G8;MGC9280;hSlu7	SLU7 splicing factor homolog (S. cerevisiae)	0.42	0.48	1.16	0.38
NYX	NYX;CLRP;CSNB1;CSNB1A;CSNB4;MGC138447;NBM1	nyctalopin	0.41	0.5	1.16	0.37
ZNF655	ZNF655;DKFZp686M1631;FLJ23461;MGC10859;MGC16203;MGC5521;VIK;VIK-1	zinc finger protein 655	0.41	0.52	1.16	0.35
KRTAP4-14	KRTAP4-14	ENTREZ STATUS: REMAPPED TO GENE ID: 653240	0.37	0.45	1.16	0.36
RPL32	RPL32;L32	ribosomal protein L32	0.36	0.4	1.16	0.33
PSMB4	PSMB4;HN3;HsN3;PROS-26;PROS26	proteasome (prosome, macropain) subunit, beta type, 4	0.33	0.38	1.16	0.29
CRY2	CRY2;FLJ10332;HCRY2;KIAA0658;PHLL2	cryptochrome 2 (photolyase-like)	0.32	0.42	1.16	0.31
CBR1	CBR1;CBR;SDR21C1;hCBR1	carbonyl reductase 1	0.32	0.42	1.16	0.28
PSMD14	PSMD14;PAD1;POH1;RPN11	proteasome (prosome, macropain) 26S subunit, non-ATPase, 14	0.25	0.32	1.16	0.22
PACRG	PACRG;FLJ32724;GLUP;HAK005771;PARK2CRG;RP3-495O10.2	PARK2 co-regulated	0.6	0.67	1.17	0.51
TBC1D28	TBC1D28;FLJ40244;MGC138217;MGC138221	TBC1 domain family, member 28	0.6	0.78	1.17	0.57
NLRP7	NLRP7;CLR19.4;FLJ94610;HYDM;MGC126470;MGC126471;NALP7;NOD12;PAN7;PYPAF3	NLR family, pyrin domain containing 7	0.6	0.7	1.17	0.51
CCDC28A	CCDC28A;C6orf80;CCRL1AP;DKFZp586D0623;MGC131913	coiled-coil domain containing 28A	0.6	0.72	1.17	0.51
TIAM2	TIAM2;FLJ41865;STEF;TIAM-2	T-cell lymphoma invasion and metastasis 2	0.6	0.68	1.17	0.54
ACOT11	ACOT11;BFIT;DKFZp667O1916;KIAA0707;STARD14;THEA;THEM1	acyl-CoA thioesterase 11	0.6	0.68	1.17	0.53
HS3ST3A1	HS3ST3A1;30ST3A1;3OST3A1	heparan sulfate (glucosamine) 3-O-sulfotransferase 3A1	0.6	0.68	1.17	0.58
MXD4	MXD4;MAD4;MST149;MSTP149;bHLHc12	MAX dimerization protein 4	0.6	0.76	1.17	0.57
CCDC104	CCDC104;MGC15407	coiled-coil domain containing 104	0.6	0.76	1.17	0.58
GMPPB	GMPPB;KIAA1851	GDP-mannose pyrophosphorylase B	0.6	0.72	1.17	0.57
STARD4	STARD4	StAR-related lipid transfer (START) domain containing 4	0.6	0.68	1.17	0.54
SAMM50	SAMM50;FLJ35825;FLJ42905;FLJ99036;OMP85;SAM50;TOB55;TRG-3;YNL026W	sorting and assembly machinery component 50 homolog (S. cerevisiae)	0.6	0.77	1.17	0.51
KDM4C	KDM4C;FLJ25949;GASC1;JHDM3C;JMJD2C;KIAA0780;bA146B14.1	lysine (K)-specific demethylase 4C	0.6	0.74	1.17	0.56
VPS16	VPS16;hVPS16	vacuolar protein sorting 16 homolog (S. cerevisiae)	0.6	0.72	1.17	0.48
HAPLN3	HAPLN3;EXLD1;HsT19883	hyaluronan and proteoglycan link protein 3	0.59	0.71	1.17	0.54
PACS1	PACS1;FLJ10209;KIAA1175	phosphofurin acidic cluster sorting protein 1	0.59	0.72	1.17	0.54
VTA1	VTA1;C6orf55;DRG-1;DRG1;FLJ27228;LIP5;My012;SBP1	Vps20-associated 1 homolog (S. cerevisiae)	0.59	0.62	1.17	0.49
DUB1A	DUB1A	ENTREZ STATUS: REMAPPED TO GENE ID: 391627	0.59	0.68	1.17	0.48
TPD52L1	TPD52L1;D53;MGC8556;TPD52L2;hD53	tumor protein D52-like 1	0.58	0.72	1.17	0.53
HNRNPA1L2	HNRNPA1L2;MGC102957	heterogeneous nuclear ribonucleoprotein A1-like 2	0.58	0.68	1.17	0.49
ROMO1	ROMO1;C20orf52;MGC111180;MTGMP;bA353C18.2	reactive oxygen species modulator 1	0.58	0.72	1.17	0.48
PLSCR2	PLSCR2	phospholipid scramblase 2	0.57	0.68	1.17	0.45
HHIP	HHIP;FLJ20992;FLJ90230;HIP	hedgehog interacting protein	0.57	0.65	1.17	0.47
NHLH1	NHLH1;HEN1;NSCL;NSCL1;bHLHa35	nescient helix loop helix 1	0.57	0.7	1.17	0.53
PGLYRP2	PGLYRP2;HMFT0141;PGLYRPL;PGRP-L;PGRPL;TAGL-like;tagL;tagL-alpha;tagl-beta	peptidoglycan recognition protein 2	0.57	0.62	1.17	0.5
ZNF831	ZNF831;C20orf174;dJ492J12.1	zinc finger protein 831	0.57	0.66	1.17	0.55
BST1	BST1;CD157	bone marrow stromal cell antigen 1	0.57	0.65	1.17	0.46
ZNF639	ZNF639;6230400O18Rik;ANC-2H01;ANC_2H01;ZASC1	zinc finger protein 639	0.57	0.66	1.17	0.49
BPTF	BPTF;FAC1;FALZ;NURF301	bromodomain PHD finger transcription factor	0.57	0.63	1.17	0.49
OSCP1	OSCP1;C1orf102;MGC26685;NOR1	organic solute carrier partner 1	0.57	0.63	1.17	0.51
C7orf33	C7orf33	chromosome 7 open reading frame 33	0.56	0.69	1.17	0.47
USP49	USP49;MGC20741	ubiquitin specific peptidase 49	0.56	0.6	1.17	0.45
ZSCAN22	ZSCAN22;HKR2;MGC126679;MGC138482;ZNF50	zinc finger and SCAN domain containing 22	0.56	0.68	1.17	0.51
GBP3	GBP3;DKFZp686E0974;DKFZp686L15228;FLJ10961	guanylate binding protein 3	0.56	0.62	1.17	0.53
KRAS	KRAS;C-K-RAS;K-RAS2A;K-RAS2B;K-RAS4A;K-RAS4B;KI-RAS;KRAS1;KRAS2;NS;NS3;RASK2	v-Ki-ras2 Kirsten rat sarcoma viral oncogene homolog	0.56	0.64	1.17	0.47
RAB1A	RAB1A;DKFZp564B163;RAB1;YPT1	RAB1A, member RAS oncogene family	0.56	0.62	1.17	0.49
ANXA11	ANXA11;ANX11;CAP50	annexin A11	0.56	0.59	1.17	0.49
NBPF1	NBPF1;AB13;AB14;AB23;AD2;FLJ20719;KIAA1693;NBG;NBPF	neuroblastoma breakpoint family, member 1	0.56	0.6	1.17	0.46
HBG1	HBG1;HBGA;HBGR;HSGGL1	hemoglobin, gamma A	0.55	0.62	1.17	0.53
ZNF268	ZNF268;HZF3;MGC126498	zinc finger protein 268	0.55	0.67	1.17	0.45
DHRS13	DHRS13;MGC23280;SDR7C5	dehydrogenase/reductase (SDR family) member 13	0.55	0.62	1.17	0.54
KLF5	KLF5;BTEB2;CKLF;IKLF	Kruppel-like factor 5 (intestinal)	0.55	0.64	1.17	0.43
ODC1	ODC1;ODC	ornithine decarboxylase 1	0.55	0.65	1.17	0.48
ATP8B3	ATP8B3;ATPIK	ATPase, aminophospholipid transporter, class I, type 8B, member 3	0.54	0.64	1.17	0.47
CCDC149	CCDC149;DKFZp761B107;FLJ13410	coiled-coil domain containing 149	0.54	0.65	1.17	0.49
TIMM44	TIMM44;DKFZp686H05241;TIM44	translocase of inner mitochondrial membrane 44 homolog (yeast)	0.54	0.62	1.17	0.47
KLK15	KLK15;ACO;HSRNASPH	kallikrein-related peptidase 15	0.53	0.62	1.17	0.46
ILVBL	ILVBL;209L8;AHAS;ILV2H;MGC1269;MGC19535	ilvB (bacterial acetolactate synthase)-like	0.53	0.68	1.17	0.5
MGAT4B	MGAT4B;GNT-IV;GNT-IVB	mannosyl (alpha-1,3-)-glycoprotein beta-1,4-N-acetylglucosaminyltransferase, isozyme B	0.53	0.6	1.17	0.47
VMP1	VMP1;DKFZp566I133;EPG3;TMEM49	vacuole membrane protein 1	0.53	0.56	1.17	0.45
SLC26A5	SLC26A5;DFNB61;MGC118886;MGC118887;MGC118888;MGC118889;PRES	solute carrier family 26, member 5 (prestin)	0.52	0.56	1.17	0.45
SOCS2	SOCS2;CIS2;Cish2;SOCS-2;SSI-2;SSI2;STATI2	suppressor of cytokine signaling 2	0.52	0.58	1.17	0.42
TIFA	TIFA;MGC20791;T2BP;T6BP;TIFAA	TRAF-interacting protein with forkhead-associated domain	0.52	0.64	1.17	0.46
GTPBP6	GTPBP6;FLJ20977;FLJ22809;FLJ56662;FLJ56805;FLJ90047;PGPL	GTP binding protein 6 (putative)	0.52	0.63	1.17	0.47
AGA	AGA;AGU;ASRG;GA	aspartylglucosaminidase	0.52	0.68	1.17	0.47
NFIC	NFIC;CTF;CTF5;MGC20153;NF-I;NFI	nuclear factor I/C (CCAAT-binding transcription factor)	0.52	0.68	1.17	0.47
VARS	VARS;G7A;VARS1;VARS2	valyl-tRNA synthetase	0.52	0.66	1.17	0.46
TBP	TBP;GTF2D;GTF2D1;HDL4;MGC117320;MGC126054;MGC126055;SCA17;TFIID	TATA box binding protein	0.52	0.56	1.17	0.44
SPATA20	SPATA20;DKFZp686H1839;FLJ21347;FLJ21969;MGC111032;SSP411;Tisp78	spermatogenesis associated 20	0.51	0.64	1.17	0.43
HLA-A29.1	HLA-A29.1	ENTREZ STATUS: REMAPPED TO GENE ID: 3138	0.51	0.63	1.17	0.44
TRPC1	TRPC1;HTRP-1;MGC133334;MGC133335;TRP1	transient receptor potential cation channel, subfamily C, member 1	0.5	0.63	1.17	0.49
B9D1	B9D1;B9;EPPB9;MKS9;MKSR1	B9 protein domain 1	0.5	0.58	1.17	0.41
SLC2A8	SLC2A8;GLUT8;GLUTX1	solute carrier family 2 (facilitated glucose transporter), member 8	0.5	0.66	1.17	0.45
ZNF324B	ZNF324B;FLJ45850	zinc finger protein 324B	0.5	0.55	1.17	0.43
MCM6	MCM6;MCG40308;Mis5;P105MCM	minichromosome maintenance complex component 6	0.5	0.56	1.17	0.44

LARGE	LARGE;KIAA0609;MDC1D;MDDGA6;MDDGB6	like-glycosyltransferase	0.49	0.6	1.17	0.4
NAA60	NAA60;FLJ11693;FLJ14154;NAT15	N(alpha)-acetyltransferase 60, NatF catalytic subunit	0.49	0.58	1.17	0.45
C1orf130	C1orf130;FLJ42528	chromosome 1 open reading frame 130	0.48	0.57	1.17	0.41
LENEP	LENEP;LEP503	lens epithelial protein	0.48	0.55	1.17	0.39
EXOC3L2	EXOC3L2;FLJ36147;MGC16332;XTP7	exocyst complex component 3-like 2	0.48	0.59	1.17	0.42
NSA2	NSA2;CDK105;HCL-G1;HCLG1;HUSSY29;TINP1;YR-29	NSA2 ribosome biogenesis homolog (S. cerevisiae)	0.48	0.58	1.17	0.39
FANK1	FANK1	fibronectin type III and ankyrin repeat domains 1	0.47	0.52	1.17	0.43
EGLN3	EGLN3;FLJ21620;HIFPH3;MGC125998;MGC125999;PHD3	egl nine homolog 3 (C. elegans)	0.47	0.53	1.17	0.46
ADH1C	ADH1C;ADH3	alcohol dehydrogenase 1C (class I), gamma polypeptide	0.46	0.5	1.17	0.4
C20orf103	C20orf103	chromosome 20 open reading frame 103	0.46	0.57	1.17	0.46
ALX4	ALX4;FND2;KIAA1788	ALX homeobox 4	0.46	0.52	1.17	0.41
CROCC	CROCC;KIAA0445;ROLT	ciliary rootlet coiled-coil, rootletin	0.46	0.55	1.17	0.4
TRIM35	TRIM35;HLS5;KIAA1098;MAIR;MGC17233	tripartite motif containing 35	0.46	0.52	1.17	0.47
MED8	MED8;ARC32;MGC17544;MGC19641	mediator complex subunit 8	0.46	0.54	1.17	0.38
SNRPB2	SNRPB2;MGC24807;MGC45309;Msl1	small nuclear ribonucleoprotein polypeptide B	0.46	0.55	1.17	0.4
GP1BA	GP1BA;BDPLT1;BDPLT3;BSS;CD42B;CD42b-alpha;DBPLT3;GP1B;GPIbA;MGC34595;VWDP	glycoprotein Ib (platelet), alpha polypeptide	0.45	0.53	1.17	0.36
OPA1	OPA1;FLJ12460;KIAA0567;MGM1;NPG;NTG;largeG	optic atrophy 1 (autosomal dominant)	0.45	0.5	1.17	0.36
S100A7	S100A7;PSOR1;S100A7c	S100 calcium binding protein A7	0.44	0.54	1.17	0.44
RCAN1	RCAN1;ADAPT78;CSP1;DSC1;DSCR1;MCIP1;RCN1	regulator of calcineurin 1	0.44	0.56	1.17	0.42
WNT9B	WNT9B;WNT14B;WNT15	wingless-type MMTV integration site family, member 9B	0.43	0.49	1.17	0.37
CDS2	CDS2;FLJ12936;FLJ38111;FLJ41837	CDP-diacylglycerol synthase (phosphatidate cytidylyltransferase) 2	0.43	0.47	1.17	0.38
FMO4	FMO4;FMO2	flavin containing monooxygenase 4	0.42	0.56	1.17	0.39
TMEM82	TMEM82	transmembrane protein 82	0.42	0.51	1.17	0.38
UPK3B	UPK3B;FLJ32198;MGC10902;P35;UPIIIb	uroplakin 3B	0.42	0.48	1.17	0.34
EIF3E	EIF3E;EIF3-P48;EIF3S6;INT6;eIF3-p46	eukaryotic translation initiation factor 3, subunit E	0.42	0.49	1.17	0.38
PSMA1	PSMA1;HC2;MGC14542;MGC14575;MGC14751;MGC1667;MGC21459;MGC22853;MGC23915;NU;PROS30	proteasome (prosome, macropain) subunit, alpha type, 1	0.42	0.49	1.17	0.37
TSEN54	TSEN54;FLJ37147;PCH2A;PCH4;SEN54L;sen54	tRNA splicing endonuclease 54 homolog (S. cerevisiae)	0.41	0.48	1.17	0.35
ZNF701	ZNF701;FLJ10891	zinc finger protein 701	0.4	0.45	1.17	0.37
RPL18	RPL18;L18	ribosomal protein L18	0.39	0.44	1.17	0.35
LBP	LBP;BPIFD2;MGC22233	lipopolysaccharide binding protein	0.38	0.47	1.17	0.31
SPEN	SPEN;HIAA0929;KIAA0929;MINT;RBM15C;SHARP	spen homolog, transcriptional regulator (Drosophila)	0.38	0.5	1.17	0.34
C21orf2	C21orf2;A2;YF5;YF5/A2	chromosome 21 open reading frame 2	0.37	0.45	1.17	0.34
CDCA8	CDCA8;BOR;BOREALIN;DasraB;FLJ10468;FLJ12042;MESRGP	cell division cycle associated 8	0.36	0.38	1.17	0.32
MCM5	MCM5;CDC46;MGC5315;P1-CDC46	minichromosome maintenance complex component 5	0.34	0.44	1.17	0.3
FAU	FAU;FAU1;FLJ22986;Fub1;Fubi;MNSFbeta;RPS30;S30;asr1	Finkel-Biskis-Reilly murine sarcoma virus (FBR-MuSV) ubiquitously expressed	0.34	0.41	1.17	0.3
PSMA6	PSMA6;IOTA;MGC22756;MGC2333;MGC23846;PROS27;p27K	proteasome (prosome, macropain) subunit, alpha type, 6	0.34	0.36	1.17	0.27
KLHDC8A	KLHDC8A;FLJ10748	kelch domain containing 8A	0.3	0.38	1.17	0.25
HIRIP3	HIRIP3	HIRA interacting protein 3	0.3	0.36	1.17	0.27
AURKB	AURKB;AIK2;AIM-1;AIM1;ARK2;AurB;IPL1;PPP1R48;STK12;STK5;aurkb-sv1;aurkb-sv2	aurora kinase B	0.28	0.35	1.17	0.25
GDF9	GDF9	growth differentiation factor 9	0.6	0.69	1.18	0.59
PAOX	PAOX;DKFZp434J245;MGC45464;PAO	polyamine oxidase (exo-N4-amino)	0.6	0.68	1.18	0.5
CTAG1A	CTAG1A;CTAG1B;ESO1;LAGE2A	cancer/testis antigen 1A	0.6	0.78	1.18	0.57
MADCAM1	MADCAM1;MACAM1	mucosal vascular addressin cell adhesion molecule 1	0.6	0.74	1.18	0.53
FNBP4	FNBP4;DKFZp686M14102;DKFZp779I1064;FBP30;FLJ41904;KIAA1014	formin binding protein 4	0.6	0.71	1.18	0.5
PKD2	PKD2;APKD2;MGC138466;MGC138468;PC2;PKD4;Pc-2;TRPP2	polycystic kidney disease 2 (autosomal dominant)	0.6	0.7	1.18	0.55
TUBG1	TUBG1;GCP-1;TUBG;TUBGCP1	tubulin, gamma 1	0.6	0.76	1.18	0.57
FAM189A2	FAM189A2;C9orf61;MGC142243;MGC142245;RP11-548B3.1;X123	family with sequence similarity 189, member A2	0.6	0.7	1.18	0.58
PRSS50	PRSS50;CT20;TSP50	protease, serine, 50	0.6	0.7	1.18	0.53
IFIH1	IFIH1;Hlcd;IDDM19;MDA-5;MDA5;MGC133047	interferon induced with helicase C domain 1	0.59	0.66	1.18	0.52
SPAG7	SPAG7;ACRP;FSA-1;MGC20134	sperm associated antigen 7	0.59	0.66	1.18	0.48
GFM1	GFM1;COXPD1;EFG;EFG1;EFGM;EGF1;FLJ12662;FLJ13632;FLJ20773;GFM;hEFG1	G elongation factor, mitochondrial 1	0.59	0.7	1.18	0.47
SLC1A5	SLC1A5;AAAT;ASCT2;ATBO;FLJ31068;M7V1;M7VS1;R16;RDRC	solute carrier family 1 (neutral amino acid transporter), member 5	0.59	0.7	1.18	0.47
TAS2R38	TAS2R38;PTC;T2R38;T2R61	taste receptor, type 2, member 38	0.58	0.76	1.18	0.56
ZEB2	ZEB2;FLJ42816;HSPC082;KIAA0569;SIP-1;SIP1;SMADIP1;ZFHX1B	zinc finger E-box binding homeobox 2	0.58	0.66	1.18	0.48
HEATR8	HEATR8;C1orf175;DKFZp434K085;DKFZp686L2332;DKFZp781B0622;FLJ45063;FLJ46354	HEAT repeat containing 8	0.58	0.73	1.18	0.51
FSHB	FSHB	follicle stimulating hormone, beta polypeptide	0.57	0.66	1.18	0.5
ZNF484	ZNF484;BA526D8.4;MGC133033;MGC133034	zinc finger protein 484	0.57	0.66	1.18	0.49
DUPD1	DUPD1;DUSP27;FMDSP	dual specificity phosphatase and pro isomerase domain containing 1	0.57	0.65	1.18	0.52
CYP1A1	CYP1A1;AHH;AHRR;CP11;CYP1;P1-450;P450-C;P450DX	cytochrome P450, family 1, subfamily A, polypeptide 1	0.57	0.65	1.18	0.51
NUP133	NUP133;FLJ10814;MGC21133;hNUP133	nucleoporin 133kDa	0.57	0.68	1.18	0.46
AACS	AACS;ACSF1;FLJ12389;FLJ41251;SUR-5	acetoacetyl-CoA synthetase	0.57	0.66	1.18	0.57
CCDC12	CCDC12;FLJ39430;FLJ40801;MGC23918	coiled-coil domain containing 12	0.57	0.73	1.18	0.56
TFAP2C	TFAP2C;AP2-GAMMA;ERF1;TFAP2G;hAP-2g	transcription factor AP-2 gamma (activating enhancer binding protein 2 gamma)	0.57	0.72	1.18	0.47
ZNF33B	ZNF33B;FLJ23327;KOX2;KOX31;MGC129696;ZNF11B	zinc finger protein 33B	0.56	0.72	1.18	0.53
FAM175B	FAM175B;ABRO1;FLJ22338;KIAA0157	family with sequence similarity 175, member B	0.56	0.68	1.18	0.46
CYB561D1	CYB561D1;FLJ39035;FLJ44753;MGC138204	cytochrome b-561 domain containing 1	0.55	0.66	1.18	0.48
LETM1	LETM1	leucine zipper-EF-hand containing transmembrane protein 1	0.55	0.69	1.18	0.55
BAG6	BAG6;BAG-6;BAT3;D6S52E;G3	BCL2-associated athanogene 6	0.55	0.58	1.18	0.49
PSG6	PSG6;PSBG-10;PSBG-12;PSBG-6;PSG10	pregnancy specific beta-1-glycoprotein 6	0.54	0.62	1.18	0.5
ACSM5	ACSM5;FLJ20581	acyl-CoA synthetase medium-chain family member 5	0.54	0.64	1.18	0.44
TSEN15	TSEN15;C1orf19;sen15	tRNA splicing endonuclease 15 homolog (S. cerevisiae)	0.54	0.62	1.18	0.47
C19orf33	C19orf33;H2RSP;IMUP;IMUP-1;IMUP-2;MGC39135;MGC75180	chromosome 19 open reading frame 33	0.54	0.66	1.18	0.5
MGAT5	MGAT5;DKFZp686B24166;FLJ31899;FLJ43217;GNT-V;GNT-VA	mannosyl (alpha-1,6-)-glycoprotein beta-1,6-N-acetyl-glucosaminyltransferase	0.54	0.62	1.18	0.46
PPP2R2A	PPP2R2A;B55A;B55ALPHA;DKFZp686N05117;FLJ26613;FLJ41727;MGC52248;PR52A;PR55A	protein phosphatase 2, regulatory subunit B, alpha	0.54	0.59	1.18	0.44
SYMPK	SYMPK;FLJ27092;SPK;SYM	symplekin	0.53	0.66	1.18	0.46
DOK6	DOK6;DOK5L;HsT3226;MGC20785	docking protein 6	0.52	0.64	1.18	0.45
C19orf45	C19orf45;FLJ35784;FLJ56642	chromosome 19 open reading frame 45	0.52	0.58	1.18	0.45
CKAP4	CKAP4;CLIMP-63;ERGIC-63;MGC99554;p63	cytoskeleton-associated protein 4	0.52	0.58	1.18	0.49
CHMP2B	CHMP2B;CHMP2.5;DKFZp564O123;DMT1;VPS2-2;VPS2B	charged multivesicular body protein 2B	0.52	0.58	1.18	0.47
C11orf75	C11orf75;FN5	chromosome 11 open reading frame 75	0.51	0.57	1.18	0.46
PRPH2	PRPH2;AOFMD;AVMD;CACD2;DS;PRPH;RDS;RP7;TSPAN22;rd2	peripherin 2 (retinal degeneration, slow)	0.5	0.55	1.18	0.44
LYRM7	LYRM7;C5orf31;FLJ20796;MGC24679	Lyrm7 homolog (mouse)	0.5	0.61	1.18	0.44
DLGAP4	DLGAP4;DAP4;DLP4;FLJ20588;KIAA0964;MGC131862;RP5-977B1.6;SAPAP4	discs, large (Drosophila) homolog-associated protein 4	0.5	0.58	1.18	0.41
EIF3G	EIF3G;EIF3-P42;EIF3S4;eIF3-delta;eIF3-p44	eukaryotic translation initiation factor 3, subunit G	0.5	0.62	1.18	0.48
SUN1	SUN1;FLJ12407;KIAA0810;MGC176649;UNC84A	Sad1 and UNC84 domain containing 1	0.5	0.56	1.18	0.41
NSFL1C	NSFL1C;FLJ46889;MGC3347;P47;UBX1;UBXD10;UBXN2C;dJ776F14.1	NSFL1 (p97) cofactor (p47)	0.49	0.6	1.18	0.43
DLK1	DLK1;DLK;FA1;PREF1;Pref-1;ZOG;pG2	delta-like 1 homolog (Drosophila)	0.48	0.58	1.18	0.44
MRPL34	MRPL34;L34mt;MGC24974;MGC2633	mitochondrial ribosomal protein L34	0.48	0.57	1.18	0.39
TAPBP	TAPBP;NGS17;TAPA;TPN;TPSN	TAP binding protein (tapasin)	0.48	0.6	1.18	0.39
ZNF528	ZNF528;KIAA1827;MGC126761;MGC138155	zinc finger protein 528	0.46	0.52	1.18	0.41
PRIC285	PRIC285;FLJ00244;KIAA1769;MGC132634;MGC138228;PDIP-1	peroxisomal proliferator-activated receptor A interacting complex 285	0.46	0.58	1.18	0.38
PPP1R3B	PPP1R3B;FLJ14005;FLJ34675;GL;PPP1R4	protein phosphatase 1, regulatory subunit 3B	0.46	0.54	1.18	0.39
HPGDS	HPGDS;GSTS;PGDS	hematopoietic prostaglandin D synthase	0.46	0.6	1.18	0.42
PSMD13	PSMD13;HSPC027;Rpn9;S11;p40.5	proteasome (prosome, macropain) 26S subunit, non-ATPase, 13	0.44	0.53	1.18	0.36
RFC4	RFC4;A1;MGC27291;RFC37	replication factor C (activator 1) 4, 37kDa	0.44	0.5	1.18	0.36
CCDC9	CCDC9;DKFZp586M1019	coiled-coil domain containing 9	0.42	0.52	1.18	0.36
RPS4X	RPS4X;CCG2;DXS306;FLJ40595;RPS4;S4;SCAR;SCR10	ribosomal protein S4, X-linked	0.42	0.47	1.18	0.36
PIM2	PIM2	pim-2 oncogene	0.41	0.48	1.18	0.4
IFITM2	IFITM2;1-8D	interferon induced transmembrane protein 2 (1-8D)	0.38	0.42	1.18	0.36
PRIM2	PRIM2;MGC75142;PRIM2A;p58	primase, DNA, polypeptide 2 (58kDa)	0.38	0.48	1.18	0.36
RPL9	RPL9;DKFZp313J1510;FLJ27456;L9;MGC15545;NPC-A-16	ribosomal protein L9	0.38	0.48	1.18	0.38
MSH5	MSH5;DKFZp434C1615;G7;MGC2939;MUTSH5;NG23	mutS homolog 5 (E. coli)	0.37	0.42	1.18	0.37
FAM54A	FAM54A;DUFD1	family with sequence similarity 54, member A	0.36	0.48	1.18	0.32
EIF4A1	EIF4A1;DDX2A;EIF-4A;EIF4A;eIF-4A-I;eIF4A-I	eukaryotic translation initiation factor 4A1	0.36	0.42	1.18	0.33
RPA1	RPA1;HSSB;MST075;REPA1;RF-A;RP-A;RPA70	replication protein A1, 70kDa	0.35	0.44	1.18	0.29
NBR1	NBR1;1A1-3B;FLJ55359;FLJ98272;KIAA0049;M17S2	neighbor of BRCA1 gene 1	0.32	0.41	1.18	0.27
PI4K2B	PI4K2B;FLJ11105;PI4KIIB;PIK42B	phosphatidylinositol 4-kinase type 2 beta	0.3	0.38	1.18	0.27
CYBRD1	CYBRD1;DCYTB;FLJ23462;FRRS3	cytochrome b reductase 1	0.3	0.36	1.18	0.28
CRNKL1	CRNKL1;CLF;CRN;Clf1;HCRN;MSTP021;SYF3	crooked neck pre-mRNA splicing factor-like 1 (Drosophila)	0.1	0.12	1.18	0.08
C4BPB	C4BPB;C4BP	complement component 4 binding protein, beta	0.6	0.7	1.19	0.58
P2RY8	P2RY8;MGC50878;P2Y8	purinergic receptor P2Y, G-protein coupled, 8	0.6	0.68	1.19	0.61
C6orf26	C6orf26;DKFZp434C1615;NG23	chromosome 6 open reading frame 26	0.6	0.74	1.19	0.56
ARG2	ARG2	arginase, type II	0.6	0.72	1.19	0.48
FAM104A	FAM104A;FLJ14775	family with sequence similarity 104, member A	0.6	0.73	1.19	0.54
KRTAP10-4	KRTAP10-4;KAP10.4;KAP18-4;KRTAP10.4;KRTAP18-4;KRTAP18.4;MGC149545;MGC149546	keratin associated protein 10-4	0.59	0.7	1.19	0.5
NLRC4	NLRC4;CARD12;CLAN;CLAN1;CLANA;CLANB;CLANC;CLAND;CLR2.1;IPAF	NLR family, CARD domain containing 4	0.58	0.7	1.19	0.54
SNAP91	SNAP91;AP180;CALM;DKFZp781O0519;KIAA0656	synaptosomal-associated protein, 91kDa homolog (mouse)	0.58	0.68	1.19	0.55
KLHDC8B	KLHDC8B;FLJ11302;MGC35097	kelch domain containing 8B	0.58	0.66	1.19	0.53
HEXDC	HEXDC;FLJ23825	hexosaminidase (glycosyl hydrolase family 20, catalytic domain) containing	0.58	0.71	1.19	0.53
MNT	MNT;MAD6;MXD6;ROX;bHLHd3	MAX binding protein	0.58	0.7	1.19	0.54
ATP10D	ATP10D;ATPVD;KIAA1487	ATPase, class V, type 10D	0.58	0.68	1.19	0.48
KPRP	KPRP;C1orf45	keratinocyte proline-rich protein	0.57	0.65	1.19	0.53
INTS2	INTS2;INT2;KIAA1287	integrator complex subunit 2	0.57	0.72	1.19	0.56
CCS	CCS;MGC138260	copper chaperone for superoxide dismutase	0.57	0.63	1.19	0.56
BCL2	BCL2;Bcl-2;PPP1R50	B-cell CLL/lymphoma 2	0.56	0.72	1.19	0.54
GGT6	GGT6;FLJ25990;FLJ90165	gamma-glutamyltransferase 6	0.56	0.62	1.19	0.5
KIF7	KIF7;ACLS;HLS2;JBTS12;MGC120653;MGC138476;MGC138478;UNQ340	kinesin family member 7	0.56	0.64	1.19	0.49
PMAIP1	PMAIP1;APR;NOXA	phorbol-12-myristate-13-acetate-induced protein 1	0.56	0.64	1.19	0.47
IL12RB2	IL12RB2	interleukin 12 receptor, beta 2	0.55	0.64	1.19	0.47

GBP5	GBP5;GBP-5	guanylate binding protein 5	0.55	0.66	1.19	0.45
HMGB4	HMGB4;FLJ40388;MGC88128;dJ1007G16.5	high mobility group box 4	0.54	0.7	1.19	0.54
MAGEC3	MAGEC3;CT7.2;HCA2;MAGE-C3;MAGEC4;MGC119270;MGC119271	melanoma antigen family C, 3	0.54	0.66	1.19	0.47
CLPS	CLPS	colipase, pancreatic	0.54	0.63	1.19	0.53
SLC22A15	SLC22A15;DKFZp761G0313;FLIPT1;PRO34686	solute carrier family 22, member 15	0.54	0.69	1.19	0.46
GALK2	GALK2;GK2;MGC1745	galactokinase 2	0.54	0.62	1.19	0.56
C4orf50	C4orf50;FLJ46481	chromosome 4 open reading frame 50	0.54	0.66	1.19	0.45
TRAM1L1	TRAM1L1;MGC26568	translocation associated membrane protein 1-like 1	0.53	0.65	1.19	0.43
CYYR1	CYYR1;C21orf95	cysteine/tyrosine-rich 1	0.52	0.6	1.19	0.43
KDELC2	KDELC2;MGC33424	KDEL (Lys-Asp-Glu-Leu) containing 2	0.52	0.58	1.19	0.47
AARSD1	AARSD1;MGC2744	alanyl-tRNA synthetase domain containing 1	0.51	0.67	1.19	0.47
VHL	VHL;HRCA1;RCA1;VHL1	von Hippel-Lindau tumor suppressor	0.5	0.64	1.19	0.45
RAP2A	RAP2A;K-REV;KREV;RAP2;RbBP-30	RAP2A, member of RAS oncogene family	0.5	0.56	1.19	0.44
PCDHA11	PCDHA11;CNR7;CNRN7;CNRS7;CRNR7;PCDH-ALPHA11	protocadherin alpha 11	0.5	0.6	1.19	0.41
C8orf42	C8orf42;DKFZp686J15216;INM01;TDRP	chromosome 8 open reading frame 42	0.49	0.6	1.19	0.45
RPN1	RPN1;DKFZp686B16177;OST1;RBPH1	ribophorin I	0.49	0.62	1.19	0.47
LCE1E	LCE1E;LEP5	late cornified envelope 1E	0.48	0.6	1.19	0.49
EEF1A1	EEF1A1;CCS-3;CCS3;EE1A1;EEF-1;EEF1A;EF-Tu;EF1A;FLJ25721;GRAF-1EF;HNGC:16303;LENG7;MGC102687;MGC131894;MGC16224;PTI1;eEF1A-1	eukaryotic translation elongation factor 1 alpha 1	0.48	0.58	1.19	0.45
RAB8B	RAB8B;FLJ38125	RAB8B, member RAS oncogene family	0.48	0.57	1.19	0.39
MOV10	MOV10;DKFZp667O1423;FLJ32791;KIAA1631;fSAP113;gb110	Mov10, Moloney leukemia virus 10, homolog (mouse)	0.48	0.6	1.19	0.46
JPH1	JPH1;DKFZp762L0313;JP-1;JP1	junctophilin 1	0.48	0.51	1.19	0.43
PPP6R1	PPP6R1;KIAA1115;MGC138185;MGC142003;PP6R1;SAP190;SAPS1	protein phosphatase 6, regulatory subunit 1	0.47	0.57	1.19	0.43
PELO	PELO;PRO1770	pelota homolog (Drosophila)	0.46	0.56	1.19	0.38
PTS	PTS;FLJ97081;PTPS	6-pyruvoyltetrahydropterin synthase	0.46	0.55	1.19	0.41
FLT4	FLT4;FLT41;LMPH1A;PCL;VEGFR3	fms-related tyrosine kinase 4	0.44	0.52	1.19	0.43
RPL13	RPL13;BBC1;D16S444E;D16S44E;FLJ27453;FLJ27454;FLJ38635;L13;MGC117342;MGC71373	ribosomal protein L13	0.44	0.56	1.19	0.45
RPL3L	RPL3L	ribosomal protein L3-like	0.44	0.5	1.19	0.4
GLTSCR2	GLTSCR2;PICT-1;PICT1	glioma tumor suppressor candidate region gene 2	0.44	0.53	1.19	0.39
DDX42	DDX42;FLJ43179;RHELP;RNAHP;SF3b125	DEAD (Asp-Glu-Ala-Asp) box polypeptide 42	0.44	0.52	1.19	0.35
EFNB2	EFNB2;EPLG5;HTKL;Htk-L;LERK5;MGC126226;MGC126227;MGC126228	ephrin-B2	0.44	0.53	1.19	0.36
GRK7	GRK7;GPRK7	G protein-coupled receptor kinase 7	0.42	0.54	1.19	0.38
OR2A2	OR2A2;OR2A17P;OR2A2P;OR7-11;OST008	olfactory receptor, family 2, subfamily A, member 2	0.42	0.5	1.19	0.38
EIF4E3	EIF4E3;MGC39820;MGC86971	eukaryotic translation initiation factor 4E family member 3	0.42	0.46	1.19	0.37
CENPA	CENPA;CENP-A	centromere protein A	0.4	0.48	1.19	0.33
EFTUD2	EFTUD2;DKFZp686E24196;FLJ44695;KIAA0031;Snrp116;Snu114;U5-116KD	elongation factor Tu GTP binding domain containing 2	0.39	0.49	1.19	0.33
POLE	POLE;DKFZp434F222;FLJ21434;POLE1	polymerase (DNA directed), epsilon	0.37	0.47	1.19	0.35
GNA15	GNA15;GNA16	guanine nucleotide binding protein (G protein), alpha 15 (Gq class)	0.36	0.48	1.19	0.33
PPP4R4	PPP4R4;KIAA1622;PP4R4	protein phosphatase 4, regulatory subunit 4	0.36	0.44	1.19	0.33
RPS2	RPS2;LLREP3;MGC102851;MGC117344;MGC117345;S2	ribosomal protein S2	0.34	0.38	1.19	0.3
GPR84	GPR84;EX33;GPCR4	G protein-coupled receptor 84	0.3	0.32	1.19	0.25
SNRPG	SNRPG;MGC117317;SMG;Sm-G	small nuclear ribonucleoprotein polypeptide G	0.22	0.26	1.19	0.19
AURKA	AURKA;AIK;ARK1;AURA;AURORA2;BTAK;MGC34538;PPP1R47;STK15;STK6;STK7	aurora kinase A	0.2	0.24	1.19	0.2
DOCK2	DOCK2;FLJ46592;KIAA0209	dedicator of cytokinesis 2	0.6	0.7	1.2	0.54
ENOX1	ENOX1;CNOX;FLJ10094;FLJ30915;PIG38;bA64J21.1;cCNOX	ecto-NOX disulfide-thiol exchanger 1	0.6	0.64	1.2	0.51
MC3R	MC3R;BMIQ9;MC3;MC3-R;OB20;OQTL	melanocortin 3 receptor	0.6	0.66	1.2	0.5
ATOH7	ATOH7;Math5;bHLHa13	atonal homolog 7 (Drosophila)	0.6	0.73	1.2	0.53
SIGLEC7	SIGLEC7;AIRM1;CD328;CDw328;D-siglec;QA79;SIGLEC-7;p75;p75/AIRM1	sialic acid binding Ig-like lectin 7	0.6	0.7	1.2	0.56
RASGEF1C	RASGEF1C;FLJ35841	RasGEF domain family, member 1C	0.6	0.75	1.2	0.61
AURKC	AURKC;AIE2;AIK3;ARK3;AurC;SPGF5;STK13;aurora-C	aurora kinase C	0.6	0.72	1.2	0.6
GGN	GGN;FLJ35713;MGC33369	gametogenetin	0.6	0.71	1.2	0.54
DAK	DAK;DKFZp586B1621;MGC5621;NET45	dihydroxyacetone kinase 2 homolog (S. cerevisiae)	0.6	0.68	1.2	0.55
FBRS	FBRS;FBS;FBS1;FLJ11618	fibrosin	0.6	0.72	1.2	0.52
ACAA1	ACAA1;ACAA;PTHIO;THIO	acetyl-CoA acyltransferase 1	0.6	0.78	1.2	0.55
DEGS1	DEGS1;DEGS;DES1;Des-1;FADS7;MGC5079;MLD	degenerative spermatocyte homolog 1, lipid desaturase (Drosophila)	0.6	0.77	1.2	0.51
MFI2	MFI2;CD228;FLJ38863;MAP97;MGC4856;MTF1	antigen p97 (melanoma associated) identified by monoclonal antibodies 133.2 and 96.5	0.59	0.73	1.2	0.5
NAGA	NAGA;D22S674;GALB	N-acetylgalactosaminidase, alpha-	0.59	0.68	1.2	0.53
37681	MARCH3;FLJ20096;MARCH-III;MGC48332;RNF173	membrane-associated ring finger (C3HC4) 3	0.58	0.7	1.2	0.63
NDEL1	NDEL1;DKFZp451M0318;EOPA;MITAP1;NUDEL	nudE nuclear distribution gene E homolog (A. nidulans)-like 1	0.58	0.7	1.2	0.49
MARK3	MARK3;CTAK1;KP78;PAR1A;Par-1a	MAP/microtubule affinity-regulating kinase 3	0.58	0.7	1.2	0.58
CAPZB	CAPZB;CAPB;CAPPB;CAPZ;DKFZp686K0524;FLJ12274;FLJ44693;MGC104401;MGC129749;MGC129750	capping protein (actin filament) muscle Z-line, beta	0.58	0.68	1.2	0.55
AKR1B1	AKR1B1;ADR;ALDR1;ALR2;AR;MGC1804	aldo-keto reductase family 1, member B1 (aldose reductase)	0.57	0.66	1.2	0.52
SEC11B	SEC11B;SEC11L2;SPCS4B	SEC11 homolog B (S. cerevisiae)	0.56	0.72	1.2	0.48
ZCCHC11	ZCCHC11;DKFZp779C1943;FLJ42878;PAPD3;TUT4	zinc finger, CCHC domain containing 11	0.56	0.74	1.2	0.55
EPB41L2	EPB41L2;4.1-G;DKFZp781D1972;DKFZp781H1755	erythrocyte membrane protein band 4.1-like 2	0.56	0.68	1.2	0.46
MRPL42	MRPL42;L31MT;L42MT;MRP-L31;MRP-L42;MRP-S32;MRPL31;MRPS32;PTD007;RPML31;S32MT	mitochondrial ribosomal protein L42	0.56	0.7	1.2	0.52
CLIC4	CLIC4;CLIC4L;DKFZp566G223;FLJ38640;H1;MTCLIC;huH1;p64H1	chloride intracellular channel 4	0.56	0.66	1.2	0.52
WDR78	WDR78;FLJ23129	WD repeat domain 78	0.55	0.7	1.2	0.49
TMEM133	TMEM133;MGC138255	transmembrane protein 133	0.55	0.63	1.2	0.48
FBXO17	FBXO17;FBG4;FBX26;FBXO26;FLJ11798;FLJ25205;FLJ58300;Fbx17;MGC9379	F-box protein 17	0.55	0.63	1.2	0.46
SLC35D2	SLC35D2;HFRC1;MGC117215;MGC142139;SQV7L;UGTrel8;hfrc	solute carrier family 35, member D2	0.54	0.66	1.2	0.5
ISX	ISX;DKFZp781N2395;MGC138417;Pix-1;RAXLX	intestine-specific homeobox	0.53	0.66	1.2	0.5
HDGF	HDGF;DKFZp686J1764;FLJ96580;HMG1L2	hepatoma-derived growth factor	0.53	0.68	1.2	0.45
SLC39A5	SLC39A5;LZT-Hs7;MGC34778;ZIP5	solute carrier family 39 (metal ion transporter), member 5	0.52	0.56	1.2	0.46
TOMM7	TOMM7;TOM7	translocase of outer mitochondrial membrane 7 homolog (yeast)	0.52	0.66	1.2	0.44
ZNF451	ZNF451;COASTER;FLJ23421;FLJ53246;FLJ90693;KIAA0576;KIAA1702;MGC26701;dJ417I1.1	zinc finger protein 451	0.52	0.64	1.2	0.44
OSTF1	OSTF1;FLJ20559;OSF;SH3P2;bA235O14.1	osteoclast stimulating factor 1	0.51	0.6	1.2	0.52
EID2B	EID2B;EID-3;FLJ38944	EP300 interacting inhibitor of differentiation 2B	0.5	0.62	1.2	0.46
KANK3	KANK3;ANKRD47;FLJ46061	KN motif and ankyrin repeat domains 3	0.5	0.57	1.2	0.46
YJEFN3	YJEFN3;FLJ44968;MGC138490	YjeF N-terminal domain containing 3	0.5	0.57	1.2	0.45
C2orf69	C2orf69;FLJ38973	chromosome 2 open reading frame 69	0.5	0.66	1.2	0.49
TIRAP	TIRAP;FLJ42305;Mal;wyatt	toll-interleukin 1 receptor (TIR) domain containing adaptor protein	0.48	0.58	1.2	0.45
SH3GLB1	SH3GLB1;Bif-1;KIAA0491;PPP1R70;dJ612B15.2	SH3-domain GRB2-like endophilin B1	0.48	0.62	1.2	0.41
NNT	NNT;MGC126502;MGC126503	nicotinamide nucleotide transhydrogenase	0.48	0.63	1.2	0.44
LMNB1	LMNB1;ADLD;LMN;LMN2;LMNB;MGC111419	lamin B1	0.48	0.6	1.2	0.4
B3GNT1	B3GNT1;B3GN-T1;B3GNT6;BETA3GNTI;iGAT;iGNT	UDP-GlcNAc:betaGal beta-1,3-N-acetylglucosaminyltransferase 1	0.47	0.54	1.2	0.39
WDR44	WDR44;DKFZp686L20145;FLJ13230;MGC26781;RAB11BP;RPH11	WD repeat domain 44	0.47	0.57	1.2	0.39
LRRC40	LRRC40;FLJ20331;dJ677H15.1	leucine rich repeat containing 40	0.46	0.6	1.2	0.4
PCSK2	PCSK2;NEC 2;NEC-2;NEC2;PC2;SPC2	proprotein convertase subtilisin/kexin type 2	0.45	0.54	1.2	0.4
GSTA1	GSTA1;GST2;GSTA1-1;GTH1;MGC131939	glutathione S-transferase alpha 1	0.44	0.5	1.2	0.44
LCE3A	LCE3A;LEP13;MGC184217	late cornified envelope 3A	0.44	0.55	1.2	0.41
EFHD2	EFHD2;MGC4342;SWS1	EF-hand domain family, member D2	0.44	0.52	1.2	0.36
GPATCH4	GPATCH4;GPATC4	G patch domain containing 4	0.42	0.5	1.2	0.36
AQP2	AQP2;AQP-CD;MGC34501;WCH-CD	aquaporin 2 (collecting duct)	0.4	0.5	1.2	0.39
VIM	VIM;FLJ36605	vimentin	0.4	0.48	1.2	0.35
ST8SIA2	ST8SIA2;HsT19690;MGC116854;MGC116857;SIAT8B;ST8SIA-II;STX	ST8 alpha-N-acetyl-neuraminide alpha-2,8-sialyltransferase 2	0.38	0.45	1.2	0.35
BLNK	BLNK;AGM4;BASH;BLNK-S;LY57;MGC111051;SLP-65;SLP65;bca	B-cell linker	0.37	0.47	1.2	0.33
ARSA	ARSA;MLD	arylsulfatase A	0.36	0.48	1.2	0.33
HTRA1	HTRA1;ARMD7;HtrA;L56;ORF480;PRSS11	HtrA serine peptidase 1	0.36	0.42	1.2	0.31
PSMC1	PSMC1;MGC24583;MGC8541;P26S4;S4;p56	proteasome (prosome, macropain) 26S subunit, ATPase, 1	0.36	0.44	1.2	0.35
PPP1R10	PPP1R10;CAT53;FB19;PNUTS;PP1R10	protein phosphatase 1, regulatory subunit 10	0.34	0.4	1.2	0.29
LGI2	LGI2;FLJ10675;KIAA1916;LGIL2;MGC126808;MGC126810	leucine-rich repeat LGI family, member 2	0.33	0.41	1.2	0.28
NCLN	NCLN;NET59	nicalin	0.33	0.38	1.2	0.29
PIGZ	PIGZ;FLJ12768;GPI-MT-IV;MGC52163;PIG-Z;SMP3	phosphatidylinositol glycan anchor biosynthesis, class Z	0.32	0.38	1.2	0.26
RAN	RAN;ARA24;Gsp1;TC4	RAN, member RAS oncogene family	0.32	0.38	1.2	0.33
C19orf75	C19orf75;FLJ40235;MGC161635;MGC161637	chromosome 19 open reading frame 75	0.3	0.36	1.2	0.26
OPTN	OPTN;ALS12;FIP2;GLC1E;HIP7;HYPL;NRP;TFIIIA-INTP	optineurin	0.21	0.24	1.2	0.2
C16orf45	C16orf45;FLJ32618	chromosome 16 open reading frame 45	0.6	0.72	1.21	0.54
FGFBP1	FGFBP1;FGF-BP;FGF-BP1;FGFBP;FGFBP-1;HBP17	fibroblast growth factor binding protein 1	0.6	0.64	1.21	0.5
LIN7B	LIN7B;LIN-7B;MALS-2;MALS2;VELI2	lin-7 homolog B (C. elegans)	0.6	0.7	1.21	0.57
RAPH1	RAPH1;ALS2CR18;ALS2CR9;KIAA1681;LPD;PREL-2;PREL2;RMO1;RalGDS/AF-6	Ras association (RalGDS/AF-6) and pleckstrin homology domains 1	0.6	0.76	1.21	0.51
SSPN	SSPN;DAGA5;KRAG;NSPN;SPN1;SPN2;nanospan	sarcospan (Kras oncogene-associated gene)	0.6	0.71	1.21	0.51
CTDP1	CTDP1;CCFDN;FCP1	CTD (carboxy-terminal domain, RNA polymerase II, polypeptide A) phosphatase, subunit 1	0.6	0.68	1.21	0.51
KLHL24	KLHL24;DRE1;FLJ25796;KRIP6	kelch-like 24 (Drosophila)	0.6	0.73	1.21	0.53
CREB3	CREB3;LUMAN;LZIP;MGC15333;MGC19782	cAMP responsive element binding protein 3	0.6	0.72	1.21	0.56
MPHOSPH10	MPHOSPH10;CT90;MPP10;MPP10P	M-phase phosphoprotein 10 (U3 small nucleolar ribonucleoprotein)	0.6	0.72	1.21	0.55
GPAT2	GPAT2;CT123	glycerol-3-phosphate acyltransferase 2, mitochondrial	0.6	0.69	1.21	0.5
C19orf35	C19orf35;FLJ45778	chromosome 19 open reading frame 35	0.59	0.71	1.21	0.51
EFCAB2	EFCAB2;FLJ33608;MGC12458	EF-hand calcium binding domain 2	0.59	0.68	1.21	0.55
TMEM185A	TMEM185A;CXorf13;FAM11A;FRAXF;MGC118844;MGC118845	transmembrane protein 185A	0.59	0.78	1.21	0.52
CNP	CNP;CNP1	2',3'-cyclic nucleotide 3' phosphodiesterase	0.59	0.7	1.21	0.51
USP14	USP14;TGT	ubiquitin specific peptidase 14 (tRNA-guanine transglycosylase)	0.59	0.7	1.21	0.53
MYH7	MYH7;CMD1S;CMH1;DKFZp451F047;MGC138376;MGC138378;MPD1;MYHCB;SPMD;SPMM	myosin, heavy chain 7, cardiac muscle, beta	0.58	0.68	1.21	0.5
SLC13A5	SLC13A5;DKFZp686E17257;MGC138356;NACT	solute carrier family 13 (sodium-dependent citrate transporter), member 5	0.58	0.7	1.21	0.55
INO80E	INO80E;CCDC95;FLJ00079;FLJ90652	INO80 complex subunit E	0.58	0.71	1.21	0.51
RGPD1	RGPD1;FLJ58076;RGP1;RGPD2;RanBP2L2	RANBP2-like and GRIP domain containing 1	0.58	0.72	1.21	0.5
PCDHGA9	PCDHGA9;PCDH-GAMMA-A9	protocadherin gamma subfamily A, 9	0.58	0.73	1.21	0.49
IFI27L2	IFI27L2;FAM14A;ISG12B;MGC44913;TLH29	interferon, alpha-inducible protein 27-like 2	0.58	0.72	1.21	0.48
TRPM8	TRPM8;LTRPC6;MGC2849;TRPP8	transient receptor potential cation channel, subfamily M, member 8	0.57	0.7	1.21	0.54
LHFPL5	LHFPL5;DFNB67;MGC33835;TMHS;dJ510O8.8	lipoma HMGIC fusion partner-like 5	0.57	0.73	1.21	0.56

PCDH17	PCDH17;PCDH68;PCH68	protocadherin 17	0.57	0.72	1.21	0.48
KIF26A	KIF26A;DKFZp434N178;FLJ22753;KIAA1236	kinesin family member 26A	0.57	0.69	1.21	0.54
NR4A1	NR4A1;GFRP1;HMR;MGC9485;N10;NAK-1;NGFIB;NP10;NUR77;TR3	nuclear receptor subfamily 4, group A, member 1	0.57	0.64	1.21	0.57
SLC25A17	SLC25A17;PMP34	solute carrier family 25 (mitochondrial carrier; peroxisomal membrane protein, 34kDa), member 17	0.57	0.68	1.21	0.54
MIB1	MIB1;DIP-1;DKFZp686I0769;DKFZp761M1710;FLJ90676;MGC129659;MGC129660;MIB;ZZANK2;ZZZ6	mindbomb homolog 1 (Drosophila)	0.57	0.68	1.21	0.62
DDX59	DDX59;DKFZp564B1023;ZNHIT5	DEAD (Asp-Glu-Ala-Asp) box polypeptide 59	0.56	0.74	1.21	0.51
MBLAC2	MBLAC2;DKFZp686P15118;MGC46734	metallo-beta-lactamase domain containing 2	0.56	0.7	1.21	0.47
RUSC2	RUSC2;Iporin;KIAA0375	RUN and SH3 domain containing 2	0.55	0.64	1.21	0.49
DEF8	DEF8;FLJ20186;MGC104349	differentially expressed in FDCP 8 homolog (mouse)	0.55	0.7	1.21	0.49
AP3M2	AP3M2;AP47B;CLA20;P47B	adaptor-related protein complex 3, mu 2 subunit	0.55	0.64	1.21	0.51
ALG11	ALG11;CDG1P;GT8;KIAA0266	asparagine-linked glycosylation 11, alpha-1,2-mannosyltransferase homolog (yeast)	0.55	0.72	1.21	0.5
FLJ32679	FLJ32679	ENTREZ STATUS: REMAPPED TO GENE ID: 100132565	0.55	0.7	1.21	0.56
POC1B	POC1B;FLJ14923;FLJ41111;PIX1;TUWD12;WDR51B	POC1 centriolar protein homolog B (Chlamydomonas)	0.55	0.7	1.21	0.5
ZNF319	ZNF319;MGC126816;ZFP319	zinc finger protein 319	0.54	0.61	1.21	0.44
C10orf88	C10orf88;FLJ13490	chromosome 10 open reading frame 88	0.54	0.65	1.21	0.44
ATP6V1F	ATP6V1F;ATP6S14;MGC117321;MGC126037;MGC126038;VATF;Vma7	ATPase, H+ transporting, lysosomal 14kDa, V1 subunit F	0.54	0.64	1.21	0.46
DCTN5	DCTN5;MGC3248	dynactin 5 (p25)	0.54	0.64	1.21	0.48
ZNF704	ZNF704;FLJ16218;Gig1;MGC10858	zinc finger protein 704	0.53	0.62	1.21	0.5
NASP	NASP;DKFZp547F162;FLB7527;FLJ31599;FLJ35510;MGC19722;MGC20372;MGC2297;PRO1999	nuclear autoantigenic sperm protein (histone-binding)	0.53	0.66	1.21	0.5
LOC120824	LOC120824	tripartite motif-containing protein ENSP00000309378-like	0.53	0.63	1.21	0.57
PGA4	PGA4;FLJ58952;FLJ77962;PGA3;PGA5	pepsinogen 4, group I (pepsinogen A)	0.52	0.62	1.21	0.53
RIT1	RIT1;MGC125864;MGC125865;RIBB;RIT;ROC1	Ras-like without CAAX 1	0.52	0.58	1.21	0.52
PNPT1	PNPT1;DKFZp762K1914;OLD35;PNPASE;old-35	polyribonucleotide nucleotidyltransferase 1	0.52	0.7	1.21	0.48
ETV3	ETV3;FLJ79173;METS;PE-1;PE1;bA110J1.4	ets variant 3	0.5	0.64	1.21	0.42
ACAD9	ACAD9;FLJ23533;MGC14452;NPD002	acyl-CoA dehydrogenase family, member 9	0.5	0.64	1.21	0.42
ASAP3	ASAP3;ACAP4;CENTB6;DDEFL1;FLJ20199;UPLC1	ArfGAP with SH3 domain, ankyrin repeat and PH domain 3	0.5	0.58	1.21	0.46
MILR1	MILR1;Allergin-1;C17orf60;MCA-32;MCA32;MGC163278	mast cell immunoglobulin-like receptor 1	0.49	0.61	1.21	0.45
BRSK1	BRSK1;FLJ43009;KIAA1811;hSAD1	BR serine/threonine kinase 1	0.48	0.58	1.21	0.48
FAM78A	FAM78A;C9orf59;FLJ00024	family with sequence similarity 78, member A	0.46	0.57	1.21	0.41
PNMA3	PNMA3;MA3;MA5;MGC132756;MGC132758	paraneoplastic antigen MA3	0.46	0.57	1.21	0.39
PRKAG3	PRKAG3;AMPKG3	protein kinase, AMP-activated, gamma 3 non-catalytic subunit	0.45	0.55	1.21	0.46
C19orf6	C19orf6;ASBABP1;MBRL;MEMBRALIN;MGC4022;R32184_3	chromosome 19 open reading frame 6	0.45	0.55	1.21	0.42
RPLP0	RPLP0;L10E;LP0;MGC111226;MGC88175;P0;PRLP0;RPP0	ribosomal protein, large, P0	0.45	0.52	1.21	0.42
LCE3E	LCE3E;LEP17	late cornified envelope 3E	0.44	0.5	1.21	0.4
SYT12	SYT12;SYT11;sytXII	synaptotagmin XII	0.44	0.55	1.21	0.4
FAM100B	FAM100B;MGC29814;MGC71792	family with sequence similarity 100, member B	0.44	0.55	1.21	0.41
SLC29A1	SLC29A1;ENT1;MGC1465;MGC3778	solute carrier family 29 (nucleoside transporters), member 1	0.44	0.59	1.21	0.41
TOP2A	TOP2A;TOP2;TP2A	topoisomerase (DNA) II alpha 170kDa	0.44	0.59	1.21	0.41
MCU	MCU;C10orf42;CCDC109A;FLJ46135	mitochondrial calcium uniporter	0.43	0.52	1.21	0.38
ZKSCAN5	ZKSCAN5;FLJ39233;KIAA1015;MGC33710;ZFP95;ZNF914	zinc finger with KRAB and SCAN domains 5	0.4	0.45	1.21	0.36
POLR1D	POLR1D;AC19;FLJ20616;MGC9850;POLR1C;RPA16;RPA9;RPAC2;RPC16;RPO1-3;TCS2	polymerase (RNA) I polypeptide D, 16kDa	0.4	0.5	1.21	0.38
FKBP1A	FKBP1A;FKBP-12;FKBP-1A;FKBP1;FKBP12;PKC12;PKCI2;PPIASE	FK506 binding protein 1A, 12kDa	0.4	0.5	1.21	0.33
MUC7	MUC7;DKFZp686J03256;FLJ27047;MG2;MGC34772	mucin 7, secreted	0.38	0.45	1.21	0.38
TEAD1	TEAD1;AA;FLJ17970;NTEF-1;REF1;TCF-13;TCF13;TEAD-1;TEF-1	TEA domain family member 1 (SV40 transcriptional enhancer factor)	0.37	0.5	1.21	0.34
IQCK	IQCK;FLJ20115;FLJ36575;MGC35048	IQ motif containing K	0.36	0.48	1.21	0.32
CWC22	CWC22;EIF4GL;KIAA1604;NCM;fSAPb	CWC22 spliceosome-associated protein homolog (S. cerevisiae)	0.36	0.45	1.21	0.33
CRK	CRK;CRKII	v-crk sarcoma virus CT10 oncogene homolog (avian)	0.35	0.42	1.21	0.3
OTUB1	OTUB1;FLJ20113;FLJ40710;MGC111158;MGC4584;OTB1;OTU1	OTU domain, ubiquitin aldehyde binding 1	0.34	0.4	1.21	0.28
HNRNPC	HNRNPC;C1;C2;HNRNP;HNRPC;MGC104306;MGC105117;MGC117353;MGC131677;SNRPC	heterogeneous nuclear ribonucleoprotein C (C1/C2)	0.34	0.44	1.21	0.33
FAM83A	FAM83A;BJ-TSA-9;MGC14128	family with sequence similarity 83, member A	0.33	0.35	1.21	0.27
KISS1	KISS1;KiSS-1;MGC39258	KiSS-1 metastasis-suppressor	0.31	0.39	1.21	0.27
BEND4	BEND4;CCDC4;FLJ35632;FLJ43965;MGC157807;MGC157808	BEN domain containing 4	0.3	0.36	1.21	0.27
LYN	LYN;FLJ26625;JTK8	v-yes-1 Yamaguchi sarcoma viral related oncogene homolog	0.28	0.34	1.21	0.28
MYLPF	MYLPF;DKFZp779C0757;HUMMLC2B;MGC13450;MRLC2;MYL11	myosin light chain, phosphorylatable, fast skeletal muscle	0.6	0.7	1.22	0.53
GALK1	GALK1;GALK;GK1	galactokinase 1	0.6	0.72	1.22	0.64
GFRA4	GFRA4	GDNF family receptor alpha 4	0.6	0.71	1.22	0.5
C9orf173	C9orf173;FLJ40246	chromosome 9 open reading frame 173	0.6	0.75	1.22	0.52
CELF1	CELF1;BRUNOL2;CUG-BP;CUGBP;CUGBP1;EDEN-BP;NAB50;NAPOR;hNab50	CUGBP, Elav-like family member 1	0.6	0.81	1.22	0.56
C12orf76	C12orf76;FLJ11385;FLJ40142	chromosome 12 open reading frame 76	0.6	0.76	1.22	0.58
KAT6B	KAT6B;DKFZp313G1618;FLJ90335;KIAA0383;MORF;MOZ2;MYST4;qkf;querkopf	K(lysine) acetyltransferase 6B	0.6	0.8	1.22	0.6
MOB1A	MOB1A;C2orf6;FLJ10788;FLJ11595;MATS1;MOB1;MOBK1B;MOBKL1B;Mob4B	MOB kinase activator 1A	0.6	0.72	1.22	0.54
RFTN1	RFTN1;FLJ23866;KIAA0084;MGC141678;MGC44480;PIB10;PIG9;RAFTLIN	raftlin, lipid raft linker 1	0.58	0.74	1.22	0.53
CFL2	CFL2;NEM7	cofilin 2 (muscle)	0.58	0.68	1.22	0.49
ATF2	ATF2;CRE-BP1;CREB2;HB16;MGC111558;TREB7	activating transcription factor 2	0.58	0.7	1.22	0.6
SH3PXD2A	SH3PXD2A;FISH;SH3MD1;TSK5	SH3 and PX domains 2A	0.57	0.66	1.22	0.53
AIPL1	AIPL1;AIPL2;LCA4	aryl hydrocarbon receptor interacting protein-like 1	0.57	0.74	1.22	0.56
CREB3L4	CREB3L4;AIBZIP;ATCE1;CREB3;CREB4;JAL;hJAL	cAMP responsive element binding protein 3-like 4	0.57	0.68	1.22	0.57
TOMM34	TOMM34;HTOM34P;TOM34;URCC3	translocase of outer mitochondrial membrane 34	0.57	0.68	1.22	0.51
PLAC4	PLAC4;C21orf115;MGC126664;MGC126666;PRED78	placenta-specific 4	0.56	0.75	1.22	0.55
WSCD1	WSCD1;KIAA0523	WSC domain containing 1	0.56	0.75	1.22	0.5
SLC25A12	SLC25A12;AGC1;ARALAR	solute carrier family 25 (mitochondrial carrier, Aralar), member 12	0.56	0.68	1.22	0.46
DERL1	DERL1;DER-1;DER1;FLJ13784;FLJ42092;MGC3067;PRO2577	Der1-like domain family, member 1	0.56	0.66	1.22	0.5
ARAP1	ARAP1;CENTD2;KIAA0782	ArfGAP with RhoGAP domain, ankyrin repeat and PH domain 1	0.56	0.7	1.22	0.54
EFTUD1	EFTUD1;FAM42A;FLJ13119;HsT19294;RIA1	elongation factor Tu GTP binding domain containing 1	0.55	0.68	1.22	0.45
PTGIR	PTGIR;IP;MGC102830;PRIPR	prostaglandin I2 (prostacyclin) receptor (IP)	0.55	0.62	1.22	0.47
LCE2A	LCE2A;LEP9	late cornified envelope 2A	0.55	0.74	1.22	0.54
TRPS1	TRPS1;GC79;LGCR;MGC134928	trichorhinophalangeal syndrome I	0.55	0.66	1.22	0.49
YTHDF2	YTHDF2;HGRG8;NY-REN-2	YTH domain family, member 2	0.55	0.64	1.22	0.54
CD300LG	CD300LG;CLM-9;CLM9;NEPMUCIN;TREM-4;TREM4	CD300 molecule-like family member g	0.54	0.68	1.22	0.46
ISL2	ISL2;FLJ10160	ISL LIM homeobox 2	0.54	0.66	1.22	0.51
C20orf166-AS	C20orf166-AS1;C20orf200;DKFZp313D1938;FLJ30313;MGC138143;NCRNA00335	C20orf166 antisense RNA 1 (non-protein coding)	0.54	0.69	1.22	0.52
RALGAPA1	RALGAPA1;DKFZp566D133;DKFZp667F074;GARNL1;GRIPE;KIAA0884;TULIP1	Ral GTPase activating protein, alpha subunit 1 (catalytic)	0.54	0.68	1.22	0.46
SPATC1	SPATC1;MGC61633;SPATA15	spermatogenesis and centriole associated 1	0.53	0.68	1.22	0.48
DYNC1I2	DYNC1I2;DNCI2;FLJ21089;FLJ90842;IC2;MGC104199;MGC111094;MGC9324	dynein, cytoplasmic 1, intermediate chain 2	0.53	0.63	1.22	0.47
HIST1H2AL	HIST1H2AL;H2A.i;H2A/i;H2AFI;HIST1H2AG;HIST1H2AI;HIST1H2AK;HIST1H2AM;dJ193B12.9	histone cluster 1, H2al	0.52	0.66	1.22	0.5
AQP12A	AQP12A;AQP12;AQPX2	aquaporin 12A	0.52	0.6	1.22	0.46
KIAA0195	KIAA0195;DKFZp781M1056;FLJ37545;TMEM94	KIAA0195	0.52	0.62	1.22	0.5
MBNL2	MBNL2;DKFZp781H1296;MBLL;MBLL39;MGC120625;MGC120626;MGC120628;PRO2032	muscleblind-like 2 (Drosophila)	0.52	0.64	1.22	0.45
CCDC99	CCDC99;FLJ20364;FLJ40690	coiled-coil domain containing 99	0.52	0.62	1.22	0.49
ABCB10	ABCB10;EST20237;M-ABC2;MTABC2	ATP-binding cassette, sub-family B (MDR/TAP), member 10	0.51	0.62	1.22	0.53
DDX18	DDX18;FLJ33908;MrDb	DEAD (Asp-Glu-Ala-Asp) box polypeptide 18	0.51	0.61	1.22	0.52
CSAG2	CSAG2;CSAG3;CSAG3B;CT24.2;MGC149851;MGC149852;TRAG-3;TRAG3	CSAG family, member 2	0.5	0.62	1.22	0.47
KCNA7	KCNA7;HAK6;KV1.7	potassium voltage-gated channel, shaker-related subfamily, member 7	0.5	0.64	1.22	0.5
C2orf28	C2orf28;APR--3;APR-3;APR3;PRO240;p18	chromosome 2 open reading frame 28	0.5	0.62	1.22	0.47
CRB3	CRB3	crumbs homolog 3 (Drosophila)	0.49	0.58	1.22	0.46
AFTPH	AFTPH;FLJ20080;FLJ23793;MGC33965;Nbla10388	aftiphilin	0.49	0.56	1.22	0.46
OR52B4	OR52B4;OR11-3	olfactory receptor, family 52, subfamily B, member 4	0.48	0.58	1.22	0.43
KRTAP10-5	KRTAP10-5;KAP10.5;KAP18-5;KAP18.5;KRTAP10.5;KRTAP18-5;KRTAP18.1;KRTAP18.5	keratin associated protein 10-5	0.48	0.64	1.22	0.43
PURB	PURB;MGC126784;MGC126786;PURBETA	purine-rich element binding protein B	0.48	0.56	1.22	0.4
ID4	ID4;IDB4;bHLHb27	inhibitor of DNA binding 4, dominant negative helix-loop-helix protein	0.48	0.54	1.22	0.43
WNT2	WNT2;INT1L1;IRP	wingless-type MMTV integration site family member 2	0.47	0.6	1.22	0.49
COQ2	COQ2;CL640;FLJ13014;FLJ26072	coenzyme Q2 homolog, prenyltransferase (yeast)	0.47	0.54	1.22	0.43
ELMOD1	ELMOD1;DKFZp547C176	ELMO/CED-12 domain containing 1	0.46	0.54	1.22	0.42
NAT8B	NAT8B;CML2;Hcml2;MGC97061;NAT8BP	N-acetyltransferase 8B (GCN5-related, putative, gene/pseudogene)	0.46	0.62	1.22	0.43
CSRNP1	CSRNP1;AXUD1;CSRNP-1;DKFZp566F164;FAM130B;TAIP-3;URAX1	cysteine-serine-rich nuclear protein 1	0.45	0.54	1.22	0.4
TM4SF18	TM4SF18;L6D	transmembrane 4 L six family member 18	0.44	0.54	1.22	0.4
ZNF672	ZNF672;FLJ22301	zinc finger protein 672	0.44	0.55	1.22	0.39
PPP1R8	PPP1R8;ARD-1;ARD1;NIPP-1;NIPP1;PRO2047	protein phosphatase 1, regulatory subunit 8	0.44	0.5	1.22	0.38
P4HA2	P4HA2	prolyl 4-hydroxylase, alpha polypeptide II	0.44	0.5	1.22	0.44
LRRTM1	LRRTM1;FLJ32082	leucine rich repeat transmembrane neuronal 1	0.43	0.55	1.22	0.36
TAS1R2	TAS1R2;GPR71;T1R2;TR2	taste receptor, type 1, member 2	0.43	0.54	1.22	0.41
METTL7A	METTL7A;AAM-B;DKFZp586A0522	methyltransferase like 7A	0.42	0.48	1.22	0.39
RASSF4	RASSF4;AD037;MGC44914	Ras association (RalGDS/AF-6) domain family member 4	0.42	0.53	1.22	0.35
PSORS1C2	PSORS1C2;C6orf17;SPR1	psoriasis susceptibility 1 candidate 2	0.42	0.51	1.22	0.36
GTF2H4	GTF2H4;TFB2;TFIIH	general transcription factor IIH, polypeptide 4, 52kDa	0.42	0.5	1.22	0.35
PISD	PISD;DJ858B16;DKFZp566G2246;PSD;PSDC;PSSC;dJ858B16.2	phosphatidylserine decarboxylase	0.42	0.52	1.22	0.35
POLA1	POLA1;DKFZp686K1672;POLA;p180	polymerase (DNA directed), alpha 1, catalytic subunit	0.42	0.47	1.22	0.41
RPL38	RPL38;L38	ribosomal protein L38	0.4	0.46	1.22	0.34
RPL36AL	RPL36AL;MGC111574;RPL36A	ribosomal protein L36a-like	0.4	0.52	1.22	0.42
GMPR2	GMPR2;MGC15084;MGC830	guanosine monophosphate reductase 2	0.39	0.53	1.22	0.36
CCBL2	CCBL2;DKFZp547N1117;DKFZp667D0223;KAT3;KATIII;MGC9398;RP4-531M19.2	cysteine conjugate-beta lyase 2	0.39	0.46	1.22	0.34
SREK1IP1	SREK1IP1;FLJ36754;MGC131910;MGC150548;MGC150549;P18SRP;SFRS12IP1	SREK1-interacting protein 1	0.39	0.52	1.22	0.34
GRP	GRP;BN;GRP-10;preproGRP;proGRP	gastrin-releasing peptide	0.38	0.48	1.22	0.32
FIG4	FIG4;ALS11;CMT4J;KIAA0274;SAC3;dJ249I4.1	FIG4 homolog, SAC1 lipid phosphatase domain containing (S. cerevisiae)	0.38	0.48	1.22	0.36
TBC1D25	TBC1D25;MG81;MGC126866;MGC126868;MGC149731;MGC149732;OATL1	TBC1 domain family, member 25	0.36	0.44	1.22	0.32
PSMD7	PSMD7;MOV34;P40;Rpn8;S12	proteasome (prosome, macropain) 26S subunit, non-ATPase, 7	0.32	0.36	1.22	0.26
TMED3	TMED3;C15orf22;MGC133022;P24B	transmembrane emp24 protein transport domain containing 3	0.3	0.38	1.22	0.26
PRPF8	PRPF8;HPRP8;PRP8;PRPC8;RP13	PRP8 pre-mRNA processing factor 8 homolog (S. cerevisiae)	0.17	0.2	1.22	0.17
C17orf97	C17orf97	chromosome 17 open reading frame 97	0.6	0.76	1.23	0.51

C2orf48	C2orf48;FLJ25102	chromosome 2 open reading frame 48	0.6	0.81	1.23	0.59
CSHL1	CSHL1;CS-5;CSHP1;CSL;MGC149868;hCS-L	chorionic somatomammotropin hormone-like 1	0.6	0.78	1.23	0.58
ZNF24	ZNF24;KOX17;RSG-A;ZNF191;ZSCAN3;Zfp191	zinc finger protein 24	0.6	0.7	1.23	0.51
TYW3	TYW3;C1orf171;FLJ40918	tRNA-yW synthesizing protein 3 homolog (S. cerevisiae)	0.6	0.7	1.23	0.57
HNRNPR	HNRNPR;FLJ25714;HNRPR;hnRNP R;hnRNP-R	heterogeneous nuclear ribonucleoprotein R	0.6	0.72	1.23	0.52
MRPL51	MRPL51;HSPC241;MRP64;bMRP64	mitochondrial ribosomal protein L51	0.6	0.71	1.23	0.51
MEF2BNB-ME	MEF2BNB-MEF2B;LOC729991-MEF2B;MEF2B	MEF2BNB-MEF2B readthrough	0.6	0.73	1.23	0.54
CCL26	CCL26;IMAC;MGC126714;MIP-4a;MIP-4alpha;SCYA26;TSC-1	chemokine (C-C motif) ligand 26	0.59	0.69	1.23	0.53
METTL15	METTL15;FLJ33979;METT5D1	methyltransferase like 15	0.59	0.68	1.23	0.55
C10orf111	C10orf111;MGC35468;bA455B2.4	chromosome 10 open reading frame 111	0.58	0.69	1.23	0.56
WDR27	WDR27;MGC43690	WD repeat domain 27	0.58	0.75	1.23	0.53
CREBBP	CREBBP;CBP;KAT3A;RSTS	CREB binding protein	0.58	0.72	1.23	0.48
NYNRIN	NYNRIN;CGIN1;FLJ11811;KIAA1305	NYN domain and retroviral integrase containing	0.58	0.74	1.23	0.56
C7orf69	C7orf69;FLJ21075	chromosome 7 open reading frame 69	0.58	0.64	1.23	0.52
NOTCH4	NOTCH4;FLJ16302;INT3;MGC74442;NOTCH3	notch 4	0.57	0.68	1.23	0.59
EDC3	EDC3;FLJ21128;FLJ31777;LSM16;YJDC;YJEFN2	enhancer of mRNA decapping 3 homolog (S. cerevisiae)	0.57	0.68	1.23	0.52
MRPL41	MRPL41;BMRP;MRP-L27;MRPL27;RPML27	mitochondrial ribosomal protein L41	0.57	0.68	1.23	0.52
STIL	STIL;DKFZp686O09161;MCPH7;SIL	SCL/TAL1 interrupting locus	0.57	0.72	1.23	0.54
AREG	AREG;AR;AREGB;CRDGF;MGC13647;SDGF	amphiregulin	0.56	0.74	1.23	0.57
MUC2	MUC2;MLP;MUC-2;SMUC	mucin 2, oligomeric mucus/gel-forming	0.56	0.7	1.23	0.53
ATN1	ATN1;B37;D12S755E;DRPLA;HRS;NOD	atrophin 1	0.56	0.64	1.23	0.51
FUCA2	FUCA2;MGC1314;dJ20N2.5	fucosidase, alpha-L- 2, plasma	0.56	0.64	1.23	0.57
MFAP5	MFAP5;MAGP2;MP25	microfibrillar associated protein 5	0.56	0.66	1.23	0.48
DMRTC1B	DMRTC1B;DMRTC1	DMRT-like family C1B	0.56	0.67	1.23	0.51
CUX2	CUX2;CDP2;CUTL2	cut-like homeobox 2	0.55	0.64	1.23	0.47
PDX1	PDX1;GSF;IDX-1;IPF1;IUF1;MODY4;PDX-1;STF-1	pancreatic and duodenal homeobox 1	0.55	0.66	1.23	0.57
C8orf40	C8orf40	chromosome 8 open reading frame 40	0.55	0.62	1.23	0.48
PSMD9	PSMD9;MGC8644;Rpn4;p27	proteasome (prosome, macropain) 26S subunit, non-ATPase, 9	0.55	0.67	1.23	0.5
MED10	MED10;MGC5309;NUT2;TRG20	mediator complex subunit 10	0.55	0.7	1.23	0.5
PIK3AP1	PIK3AP1;BCAP;RP11-34E5.3	phosphoinositide-3-kinase adaptor protein 1	0.54	0.7	1.23	0.46
COTL1	COTL1;CLP;FLJ43657;MGC19733	coactosin-like 1 (Dictyostelium)	0.54	0.64	1.23	0.49
SRPR	SRPR;DP;MGC17355;MGC3650;MGC9571;Sralpha	signal recognition particle receptor (docking protein)	0.54	0.72	1.23	0.47
OR1E1	OR1E1;HGM071;OR13-66;OR17-2;OR17-32;OR1E5;OR1E6;OR1E8P;OR1E9P;OST547	olfactory receptor, family 1, subfamily E, member 1	0.53	0.63	1.23	0.45
HIST1H2AE	HIST1H2AE;H2A.1;H2A.2;H2A/a;H2AFA	histone cluster 1, H2ae	0.53	0.68	1.23	0.46
GLYCTK	GLYCTK;HBEBP2;HBEBP4;HBeAgBP4A	glycerate kinase	0.52	0.62	1.23	0.54
HSPE1	HSPE1;CPN10;EPF;GROES;HSP10	heat shock 10kDa protein 1 (chaperonin 10)	0.52	0.6	1.23	0.47
CD300C	CD300C;CLM-6;CMRF-35;CMRF-35A;CMRF35;CMRF35-A1;CMRF35A;CMRF35A1;IGSF16;LIR	CD300c molecule	0.51	0.64	1.23	0.49
PPP3R2	PPP3R2;PPP3RL	protein phosphatase 3, regulatory subunit B, beta	0.5	0.57	1.23	0.43
HUS1B	HUS1B;MGC126746;MGC126748;RP11-532F6.1	HUS1 checkpoint homolog b (S. pombe)	0.5	0.56	1.23	0.46
NFATC4	NFATC4;NF-ATc4;NFAT3	nuclear factor of activated T-cells, cytoplasmic, calcineurin-dependent 4	0.5	0.68	1.23	0.47
IQCD	IQCD;4933433C09Rik	IQ motif containing D	0.5	0.62	1.23	0.45
PSME3	PSME3;Ki;PA28-gamma;PA28G;REG-GAMMA	proteasome (prosome, macropain) activator subunit 3 (PA28 gamma; Ki)	0.5	0.56	1.23	0.44
KDM6B	KDM6B;JMJD3;KIAA0346	lysine (K)-specific demethylase 6B	0.5	0.6	1.23	0.48
NFE2L3	NFE2L3;NRF3	nuclear factor (erythroid-derived 2)-like 3	0.49	0.58	1.23	0.51
KRTAP5-6	KRTAP5-6;KRTAP5.6	keratin associated protein 5-6	0.48	0.57	1.23	0.45
VEGFB	VEGFB;VEGFL;VRF	vascular endothelial growth factor B	0.48	0.56	1.23	0.44
ALG1L	ALG1L;ALG1L1	asparagine-linked glycosylation 1-like	0.48	0.62	1.23	0.47
CXCL1	CXCL1;FSP;GRO1;GROa;MGSA;MGSA-a;NAP-3;SCYB1	chemokine (C-X-C motif) ligand 1 (melanoma growth stimulating activity, alpha)	0.47	0.56	1.23	0.4
PCBP3	PCBP3;ALPHA-CP3	poly(rC) binding protein 3	0.46	0.56	1.23	0.45
AVP	AVP;ADH;ARVP;AVP-NPII;AVRP;VP	arginine vasopressin	0.46	0.54	1.23	0.45
SETD4	SETD4;C21orf18;C21orf27	SET domain containing 4	0.46	0.54	1.23	0.44
CDK5RAP3	CDK5RAP3;C53;HSF-27;IC53;LZAP;MST016;OK/SW-cl.114	CDK5 regulatory subunit associated protein 3	0.46	0.58	1.23	0.39
TRIAP1	TRIAP1;P53CSV;WF-1	TP53 regulated inhibitor of apoptosis 1	0.46	0.61	1.23	0.41
CEP57L1	CEP57L1;C6orf182;MGC21731;MGC70837;bA487F23.2;cep57R	centrosomal protein 57kDa-like 1	0.46	0.55	1.23	0.46
C11orf48	C11orf48;MGC2477	chromosome 11 open reading frame 48	0.45	0.5	1.23	0.39
PSMB7	PSMB7;Z	proteasome (prosome, macropain) subunit, beta type, 7	0.45	0.55	1.23	0.41
ADNP2	ADNP2;KIAA0863;ZNF508	ADNP homeobox 2	0.44	0.58	1.23	0.45
KIAA1267	KIAA1267;CENP-36;DKFZp686P06109;DKFZp727C091;KANSL1;MGC102843;MSL1v1;NSL1	KIAA1267	0.44	0.53	1.23	0.41
NVL	NVL	nuclear VCP-like	0.43	0.54	1.23	0.39
HMGN5	HMGN5;NBP-45;NSBP1	high mobility group nucleosome binding domain 5	0.42	0.52	1.23	0.37
DAPK3	DAPK3;FLJ36473;ZIP;ZIPK	death-associated protein kinase 3	0.41	0.54	1.23	0.39
PDCD7	PDCD7;ES18;FLJ54213;HES18;MGC22015	programmed cell death 7	0.4	0.55	1.23	0.4
FABP9	FABP9;PERF;PERF15;T-FABP	fatty acid binding protein 9, testis	0.4	0.51	1.23	0.36
FAM70B	FAM70B;FLJ57938;MGC20579;RP11-199F6.1	family with sequence similarity 70, member B	0.38	0.52	1.23	0.34
PRDM8	PRDM8;PFM5	PR domain containing 8	0.34	0.42	1.23	0.29
CCND2	CCND2;KIAK0002;MGC102758	cyclin D2	0.32	0.36	1.23	0.29
GUCY2D	GUCY2D;CORD5;CORD6;CYGD;GUC1A4;GUC2D;LCA;LCA1;RCD2;RETGC-1;ROS-GC1;ROSGC;retGC	guanylate cyclase 2D, membrane (retina-specific)	0.31	0.38	1.23	0.32
B3GAT1	B3GAT1;CD57;GLCATP;GLCUATP;HNK1;LEU7;NK-1;NK1	beta-1,3-glucuronyltransferase 1 (glucuronosyltransferase P)	0.3	0.37	1.23	0.29
DDIT3	DDIT3;CEBPZ;CHOP;CHOP-10;CHOP10;GADD153;MGC4154	DNA-damage-inducible transcript 3	0.29	0.34	1.23	0.27
ZBTB16	ZBTB16;PLZF;ZNF145	zinc finger and BTB domain containing 16	0.24	0.34	1.23	0.23
FSD1L	FSD1L;CCDC10;CSDUFD1;FSD1CL;FSD1NL;MGC45564;MIR1	fibronectin type III and SPRY domain containing 1-like	0.22	0.27	1.23	0.24
C1orf210	C1orf210;MGC52423	chromosome 1 open reading frame 210	0.19	0.24	1.23	0.19
HAO2	HAO2;GIG16;HAOX2	hydroxyacid oxidase 2 (long chain)	0.6	0.76	1.24	0.51
PPP1R12B	PPP1R12B;FLJ40942;MGC131980;MGC87886;MYPT2;PP1bp55	protein phosphatase 1, regulatory subunit 12B	0.6	0.7	1.24	0.53
CLPP	CLPP	ClpP caseinolytic peptidase, ATP-dependent, proteolytic subunit homolog (E. coli)	0.6	0.74	1.24	0.55
RABGAP1	RABGAP1;DKFZp586D2123;GAPCENA;RP11-123N4.2;TBC1D11	RAB GTPase activating protein 1	0.6	0.8	1.24	0.53
SRPK2	SRPK2;FLJ36101;SFRSK2	SRSF protein kinase 2	0.6	0.8	1.24	0.57
SRSF2	SRSF2;PR264;SC-35;SC35;SFRS2;SFRS2A;SRp30b	serine/arginine-rich splicing factor 2	0.6	0.74	1.24	0.63
LCN15	LCN15;PRO6093;UNQ2541	lipocalin 15	0.6	0.72	1.24	0.55
PRPS1L1	PRPS1L1;PRPS1;PRPS3;PRPSL;PRS-III	phosphoribosyl pyrophosphate synthetase 1-like 1	0.58	0.78	1.24	0.56
ANK1	ANK1;ANK;SPH1;SPH2	ankyrin 1, erythrocytic	0.58	0.75	1.24	0.57
TRIB2	TRIB2;C5FW;FLJ57420;GS3955;TRB2	tribbles homolog 2 (Drosophila)	0.58	0.78	1.24	0.56
C6orf223	C6orf223;MGC45491	chromosome 6 open reading frame 223	0.58	0.75	1.24	0.57
C1QTNF5	C1QTNF5;CTRP5;DKFZp586B0621;FLJ30570;LORD	C1q and tumor necrosis factor related protein 5	0.58	0.74	1.24	0.53
SYNGR4	SYNGR4;MGC125805	synaptogyrin 4	0.57	0.68	1.24	0.57
LYSMD1	LYSMD1;MGC35223;RP11-68I18.5	LysM, putative peptidoglycan-binding, domain containing 1	0.57	0.75	1.24	0.58
JAGN1	JAGN1;FLJ14602;GL009	jagunal homolog 1 (Drosophila)	0.57	0.71	1.24	0.51
CTSA	CTSA;GLB2;GSL;NGBE;PPCA;PPGB	cathepsin A	0.57	0.65	1.24	0.49
WDR83	WDR83;MGC4238;MORG1	WD repeat domain 83	0.57	0.64	1.24	0.51
CCR9	CCR9;CDw199;GPR-9-6;GPR28	chemokine (C-C motif) receptor 9	0.56	0.68	1.24	0.62
TMEM25	TMEM25;FLJ14399	transmembrane protein 25	0.56	0.68	1.24	0.51
MRPL4	MRPL4;CGI-28;L4mt;MGC16367;MGC2681	mitochondrial ribosomal protein L4	0.56	0.72	1.24	0.54
GPKOW	GPKOW;GPATC5;GPATCH5;Spp2;T54	G patch domain and KOW motifs	0.56	0.72	1.24	0.48
KARS	KARS;CMTRIB;KARS2;KIAA0070;KRS	lysyl-tRNA synthetase	0.56	0.7	1.24	0.54
INTS4	INTS4;INT4;MGC16733;MST093	integrator complex subunit 4	0.55	0.72	1.24	0.56
CCM2	CCM2;C7orf22;MGC4067;MGC4607;MGC74868;OSM	cerebral cavernous malformation 2	0.54	0.64	1.24	0.47
DHX15	DHX15;DBP1;DDX15;HRH2;PRP43;PRPF43;PrPp43p	DEAH (Asp-Glu-Ala-His) box polypeptide 15	0.54	0.65	1.24	0.46
SPRYD7	SPRYD7;C13orf1;CLLD6	SPRY domain containing 7	0.54	0.68	1.24	0.52
MAPK8IP2	MAPK8IP2;IB2;JIP2;PRKM8IPL	mitogen-activated protein kinase 8 interacting protein 2	0.53	0.7	1.24	0.49
ZNF649	ZNF649;FLJ12644	zinc finger protein 649	0.52	0.61	1.24	0.49
ING3	ING3;Eaf4;FLJ20089;ING2;MEAF4;p47ING3	inhibitor of growth family, member 3	0.52	0.71	1.24	0.52
TRAK1	TRAK1;MILT1;OIP106	trafficking protein, kinesin binding 1	0.52	0.62	1.24	0.5
CD6	CD6;FLJ44171;TP120	CD6 molecule	0.5	0.69	1.24	0.48
TXNRD2	TXNRD2;SELZ;TR;TR-BETA;TR3;TRXR2	thioredoxin reductase 2	0.5	0.6	1.24	0.52
EIF2C1	EIF2C1;AGO1;DKFZp686M13167;EIF2C;GERP95;Q99	eukaryotic translation initiation factor 2C, 1	0.5	0.6	1.24	0.47
PATL1	PATL1;FLJ36874;MGC125671;MGC125672;Pat1b;hPat1b	protein associated with topoisomerase II homolog 1 (yeast)	0.5	0.64	1.24	0.46
CTAGE15P	CTAGE15P;MGC167090	CTAGE family, member 15, pseudogene	0.5	0.63	1.24	0.43
C17orf98	C17orf98	chromosome 17 open reading frame 98	0.5	0.62	1.24	0.51
LUC7L	LUC7L;FLJ10231;LUC7-LIKE;LUC7B1;Luc7;SR+89;hLuc7B1	LUC7-like (S. cerevisiae)	0.49	0.64	1.24	0.48
PNKP	PNKP;EIEE10;MCSZ;PNK	polynucleotide kinase 3'-phosphatase	0.49	0.6	1.24	0.5
CADM2	CADM2;IGSF4D;MGC104534;MGC138341;MGC138343;NECL3;Necl-3;synCAM2	cell adhesion molecule 2	0.48	0.64	1.24	0.45
GNAT2	GNAT2;ACHM4;GNATC	guanine nucleotide binding protein (G protein), alpha transducing activity polypeptide 2	0.48	0.55	1.24	0.41
PLCH2	PLCH2;PLC-eta2;PLCL4;PLCeta2	phospholipase C, eta 2	0.48	0.6	1.24	0.44
CRABP1	CRABP1;CRABP;CRABP-I;CRABPI;RBP5	cellular retinoic acid binding protein 1	0.48	0.6	1.24	0.47
ZFYVE16	ZFYVE16;DKFZp686E13162;ENDOFIN;KIAA0305;PPP1R69	zinc finger, FYVE domain containing 16	0.48	0.6	1.24	0.4
MKI67IP	MKI67IP;NIFK;Nopp34	MKI67 (FHA domain) interacting nucleolar phosphoprotein	0.48	0.6	1.24	0.42
RHPN2	RHPN2;P76RBE;RHOBP	rhophilin, Rho GTPase binding protein 2	0.48	0.61	1.24	0.44
PAMR1	PAMR1;DKFZP586H2123;RAMP	peptidase domain containing associated with muscle regeneration 1	0.48	0.55	1.24	0.41
C2orf15	C2orf15;FLJ44438;MGC29762	chromosome 2 open reading frame 15	0.47	0.55	1.24	0.47
C1orf93	C1orf93;DKFZp547M123;MGC26818	chromosome 1 open reading frame 93	0.47	0.55	1.24	0.45
CSTF2T	CSTF2T;CstF-64T;DKFZp434C1013;KIAA0689	cleavage stimulation factor, 3' pre-RNA, subunit 2, 64kDa, tau variant	0.46	0.52	1.24	0.42
TXNIP	TXNIP;EST01027;HHCPA78;THIF;VDUP1	thioredoxin interacting protein	0.46	0.62	1.24	0.46
VPREB3	VPREB3;8HS20;N27C7-2	pre-B lymphocyte 3	0.45	0.55	1.24	0.44
TMCC3	TMCC3;KIAA1145	transmembrane and coiled-coil domain family 3	0.44	0.56	1.24	0.38
KIF14	KIF14;KIAA0042;MGC142302	kinesin family member 14	0.44	0.53	1.24	0.39
COPG	COPG;COPG1;FLJ21068	coatomer protein complex, subunit gamma	0.44	0.55	1.24	0.38
CLVS2	CLVS2;C6orf212;C6orf213;MGC120572;RLBP1L2;bA160A10.4	clavesin 2	0.44	0.56	1.24	0.38
TTC5	TTC5;Strap	tetratricopeptide repeat domain 5	0.42	0.58	1.24	0.43
CLSTN3	CLSTN3;CDHR14;CSTN3;KIAA0726;MGC131797;MGC138488;alcbeta	calsyntenin 3	0.42	0.53	1.24	0.4

RPS7	RPS7;DBA8;S7	ribosomal protein S7	0.42	0.54	1.24	0.4
MCM10	MCM10;CNA43;DNA43;MGC126776	minichromosome maintenance complex component 10	0.41	0.5	1.24	0.37
FUK	FUK;1110046B12Rik;FLJ39408	fucokinase	0.4	0.54	1.24	0.38
PBXIP1	PBXIP1;HPIP	pre-B-cell leukemia homeobox interacting protein 1	0.4	0.5	1.24	0.33
RPS27A	RPS27A;CEP80;S27A;UBA80;UBC;UBCEP1;UBCEP80	ribosomal protein S27a	0.4	0.52	1.24	0.34
GML	GML;LY6DL	glycosylphosphatidylinositol anchored molecule like protein	0.38	0.46	1.24	0.35
RPL23A	RPL23A;FLJ27455;L23A;MDA20	ribosomal protein L23a	0.37	0.44	1.24	0.34
STK39	STK39;DCHT;DKFZp686K05124;PASK;SPAK	serine threonine kinase 39	0.36	0.44	1.24	0.37
CA14	CA14;CAXiV	carbonic anhydrase XIV	0.34	0.4	1.24	0.31
RCAN2	RCAN2;CSP2;DSCR1L1;MCIP2;RCN2;ZAKI-4;ZAKI4	regulator of calcineurin 2	0.34	0.44	1.24	0.3
PRDM13	PRDM13;MU-MB-20.220;PFM10	PR domain containing 13	0.33	0.42	1.24	0.29
DYX1C1	DYX1C1;DYX1;DYXC1;EKN1;FLJ37882;MGC70618;RD	dyslexia susceptibility 1 candidate 1	0.26	0.34	1.24	0.25
CHEK1	CHEK1;CHK1	CHK1 checkpoint homolog (S. pombe)	0.24	0.3	1.24	0.24
RRM1	RRM1;R1;RIR1;RR1	ribonucleotide reductase M1	0.24	0.28	1.24	0.22
CYP2C18	CYP2C18;CPCI;CYP2C;CYP2C17;DKFZp686I24235;P450-6B/29C;P450IIC17	cytochrome P450, family 2, subfamily C, polypeptide 18	0.86	0.5	0.59	0.37
CLEC4E	CLEC4E;CLECSF9;MINCLE	C-type lectin domain family 4, member E	0.86	0.55	0.61	0.42
AMY2A	AMY2A;AMY2;AMY2B;PA	amylase, alpha 2A (pancreatic)	1.09	0.66	0.62	0.51
MGP	MGP;MGLAP;NTI	matrix Gla protein	0.88	0.57	0.62	0.38
ITLN1	ITLN1;FLJ20022;HL-1;HL1;INTL;ITLN;LFR;hIntL;omentin	intelectin 1 (galactofuranose binding)	1.16	0.66	0.63	0.55
FLT1	FLT1;FLT;VEGFR1	fms-related tyrosine kinase 1 (vascular endothelial growth factor/vascular permeability factor receptor)	0.98	0.62	0.64	0.55
PTPRO	PTPRO;GLEPP1;NPHS6;PTP-OC;PTP-U2;PTPROT;PTPU2;R-PTP-O	protein tyrosine phosphatase, receptor type, O	1	0.61	0.65	0.46
RIT2	RIT2;RIBA;RIN;ROC2	Ras-like without CAAX 2	1.06	0.7	0.67	0.52
FAM133A	FAM133A;CT115;FLJ37659	family with sequence similarity 133, member A	1.29	0.92	0.68	0.65
OR5B12	OR5B12;OR11-241;OR5B12P;OR5B16;OST743	olfactory receptor, family 5, subfamily B, member 12	1.28	0.84	0.68	0.67
C1orf173	C1orf173;DKFZp547I048;DKFZp761G1720;DKFZp781L0319;MGC90412;RP11-653A5.1	chromosome 1 open reading frame 173	0.9	0.64	0.69	0.48
FAM26D	FAM26D;C6orf78;FLJ32239	family with sequence similarity 26, member D	1.25	0.86	0.7	0.64
FAM71D	FAM71D;C14orf54;FLJ36130	family with sequence similarity 71, member D	1.21	0.83	0.71	0.63
IL5RA	IL5RA;CD125;CDw125;HSIL5R3;IL5R;MGC26560	interleukin 5 receptor, alpha	1.1	0.74	0.72	0.59
OR6C70	OR6C70	olfactory receptor, family 6, subfamily C, member 70	1.02	0.71	0.72	0.54
OR13C2	OR13C2;OR37K	olfactory receptor, family 13, subfamily C, member 2	0.97	0.72	0.72	0.55
GLIPR1L2	GLIPR1L2;MGC39497	GLI pathogenesis-related 1 like 2	0.96	0.66	0.72	0.5
TAS2R40	TAS2R40;GPR60;MGC126844;MGC126846;T2R40;T2R58	taste receptor, type 2, member 40	0.88	0.61	0.72	0.47
KBTBD10	KBTBD10;FLJ60989;SARCOSIN	kelch repeat and BTB (POZ) domain containing 10	1.15	0.82	0.73	0.62
KRTAP15-1	KRTAP15-1;KAP15.1	keratin associated protein 15-1	1.1	0.78	0.73	0.59
LYZL2	LYZL2	lysozyme-like 2	0.89	0.64	0.73	0.5
F13B	F13B;FXIIIB	coagulation factor XIII, B polypeptide	0.88	0.63	0.74	0.46
ASB11	ASB11;DKFZp779E2460;MGC119168;MGC119169	ankyrin repeat and SOCS box containing 11	1	0.72	0.75	0.52
OR4D9	OR4D9;OR11-253	olfactory receptor, family 4, subfamily D, member 9	1.25	0.94	0.76	0.71
CLIC6	CLIC6;CLIC1L	chloride intracellular channel 6	1.12	0.85	0.76	0.66
RXFP2	RXFP2;GPR106;GREAT;INSL3R;LGR8;LGR8.1;RXFPR2	relaxin/insulin-like family peptide receptor 2	1.01	0.74	0.76	0.67
CCR2	CCR2	ENTREZ STATUS: REMAPPED TO GENE ID: 729230	1.03	0.77	0.78	0.7
ENPEP	ENPEP;APA;CD249;gp160	glutamyl aminopeptidase (aminopeptidase A)	0.98	0.7	0.78	0.52
MUC13	MUC13;DRCC1;FLJ20063;MUC-13	mucin 13, cell surface associated	1.36	0.98	0.8	0.81
RTP3	RTP3;LTM1;TMEM7	receptor (chemosensory) transporter protein 3	0.9	0.7	0.73	0.49
SPACA1	SPACA1;MGC32952;SAMP32	sperm acrosome associated 1	0.9	0.68	0.75	0.47
ANKRD22	ANKRD22;MGC22805	ankyrin repeat domain 22	0.91	0.7	0.76	0.49
PMP2	PMP2;FABP8;M-FABP;MP2;P2	peripheral myelin protein 2	1.02	0.78	0.77	0.53
TNNI3K	TNNI3K;CARK;MGC142099;MGC33828	TNNI3 interacting kinase	1.1	0.86	0.78	0.71
CEACAM7	CEACAM7;CEA;CGM2	carcinoembryonic antigen-related cell adhesion molecule 7	0.9	0.68	0.78	0.48
MAS1L	MAS1L;MAS-L;MGC119987;MRG;dJ994E9.2	MAS1 oncogene-like	1	0.76	0.79	0.69
SRY	SRY;SRXX1;SRXY1;TDF;TDY	sex determining region Y	0.94	0.7	0.79	0.61
ART3	ART3;FLJ26404	ADP-ribosyltransferase 3	0.97	0.76	0.8	0.65
NDST4	NDST4;NDST-4;NHSST4	N-deacetylase/N-sulfotransferase (heparan glucosaminyl) 4	0.88	0.68	0.8	0.51
PRKG2	PRKG2;PRKGR2;cGKII	protein kinase, cGMP-dependent, type II	0.8	0.46	0.58	0.39
AMY2B	AMY2B;AMY2	amylase, alpha 2B (pancreatic)	0.82	0.54	0.63	0.42
CD163	CD163;M130;MM130	CD163 molecule	0.7	0.45	0.67	0.33
KRTAP19-7	KRTAP19-7;KAP19.7;MGC118928	keratin associated protein 19-7	0.81	0.58	0.68	0.39
TDGF1	TDGF1;CR;CRGF;CRIPTO	teratocarcinoma-derived growth factor 1	0.76	0.52	0.69	0.4
SLC1A2	SLC1A2;EAAT2;GLT-1	solute carrier family 1 (glial high affinity glutamate transporter), member 2	0.9	0.72	0.74	0.54
RPS6KA6	RPS6KA6;PP90RSK4;RSK4	ribosomal protein S6 kinase, 90kDa, polypeptide 6	0.95	0.77	0.76	0.55
OR1J2	OR1J2;FLJ16828;HG152;HSA5;OR1J3;OR1J5;OR9-19;OST044	olfactory receptor, family 1, subfamily J, member 2	0.92	0.75	0.77	0.53
KIAA0825	KIAA0825;C5orf36;DKFZp686F0372;FLJ27431;MGC34713	KIAA0825	1.17	0.98	0.78	0.69
FAM55D	FAM55D;C11orf33;FLJ20127	family with sequence similarity 55, member D	1.08	0.92	0.78	0.61
MUSK	MUSK;MGC126323;MGC126324	muscle, skeletal, receptor tyrosine kinase	0.92	0.73	0.79	0.6
FGFBP2	FGFBP2;HBP17RP;KSP37	fibroblast growth factor binding protein 2	0.85	0.72	0.79	0.55
BEST3	BEST3;MGC13168;MGC40411;VMD2L3	bestrophin 3	1	0.83	0.8	0.65
BAGE2	BAGE2;CT2.2	B melanoma antigen family, member 2	0.7	0.53	0.72	0.43
TMC1	TMC1;DFNA36;DFNB11;DFNB7	transmembrane channel-like 1	0.74	0.56	0.74	0.43
C1orf168	C1orf168;FLJ43208	chromosome 1 open reading frame 168	0.64	0.5	0.74	0.37
CETN1	CETN1;CEN1;CETN	centrin, EF-hand protein, 1	0.8	0.66	0.75	0.46
MYH4	MYH4;MYH2B;MyHC-2B;MyHC-IIb	myosin, heavy chain 4, skeletal muscle	0.73	0.57	0.76	0.44
OR5M11	OR5M11;OR11-199	olfactory receptor, family 5, subfamily M, member 11	0.66	0.53	0.77	0.4
TTC23L	TTC23L;FLJ25439	tetratricopeptide repeat domain 23-like	0.84	0.68	0.78	0.51
REG3A	REG3A;FLJ18565;HIP;PAP;PAP-H;PAP1;PBCGF;REG-III;REG3	regenerating islet-derived 3 alpha	0.78	0.6	0.78	0.45
HSFY2	HSFY2;FLJ25453;HSF2L;HSFY;HSFY1	heat shock transcription factor, Y linked 2	0.74	0.56	0.78	0.43
C13orf26	C13orf26;MGC40178	chromosome 13 open reading frame 26	0.84	0.72	0.79	0.48
CD69	CD69;AIM;BL-AC/P26;CLEC2C;EA1;GP32/28;MLR-3	CD69 molecule	0.83	0.64	0.79	0.55
LRP2BP	LRP2BP;DKFZp761O0113;FLJ44965	LRP2 binding protein	0.78	0.66	0.79	0.51
UGT2B4	UGT2B4;HLUG25;UDPGT2B4;UDPGTH1;UGT2B11	UDP glucuronosyltransferase 2 family, polypeptide B4	0.71	0.56	0.8	0.42
CXorf1	CXorf1;MGC142160;MGC142164	chromosome X open reading frame 1	0.71	0.57	0.8	0.39
OR51B4	OR51B4;HOR5'Beta1	olfactory receptor, family 51, subfamily B, member 4	1.03	0.76	0.82	0.63
SATL1	SATL1	spermidine/spermine N1-acetyl transferase-like 1	1.04	0.84	0.81	0.57
BMP5	BMP5;MGC34244	bone morphogenetic protein 5	0.99	0.78	0.81	0.58
TPTE2	TPTE2;MGC149231;TPIP	transmembrane phosphoinositide 3-phosphatase and tensin homolog 2	1.04	0.8	0.83	0.61
PCDHB6	PCDHB6;PCDH-BETA6	protocadherin beta 6	1.23	0.99	0.84	0.76
CDH17	CDH17;CDH16;FLJ26931;HPT-1;HPT1;MGC138218;MGC142024	cadherin 17, LI cadherin (liver-intestine)	1.17	0.96	0.84	0.75
LRRC66	LRRC66	leucine rich repeat containing 66	0.92	0.7	0.84	0.56
ARMC3	ARMC3;CT81;FLJ25845;FLJ32827;KU-CT-1	armadillo repeat containing 3	1.23	0.99	0.85	0.83
MAS1	MAS1;MAS;MGC119966	MAS1 oncogene	1.16	0.95	0.85	0.85
CNGB3	CNGB3	cyclic nucleotide gated channel beta 3	1.28	1.08	0.86	0.84
C18orf26	C18orf26;DYNAP;FLJ39106	chromosome 18 open reading frame 26	1.25	1.04	0.86	0.79
OR6A2	OR6A2;I7;MGC126538;MGC126540;OR11-55;OR6A1;OR6A2P	olfactory receptor, family 6, subfamily A, member 2	1.21	1	0.86	0.79
C10orf96	C10orf96;MGC35062	chromosome 10 open reading frame 96	1.07	0.84	0.87	0.7
SPRR2A	SPRR2A	small proline-rich protein 2A	1.06	0.82	0.87	0.69
USP9Y	USP9Y;DFFRY;FLJ33043;SPGFY2	ubiquitin specific peptidase 9, Y-linked	1.2	1	0.91	0.75
GNRHR	GNRHR;GNRHR1;GRHR;LHRHR;LRHR	gonadotropin-releasing hormone receptor	1.29	1.09	0.92	0.83
DCT	DCT;TRP-2;TYRP2	dopachrome tautomerase (dopachrome delta-isomerase, tyrosine-related protein 2)	0.91	1.32	1.44	1.03
OR51L1	OR51L1;OR11-31	olfactory receptor, family 51, subfamily L, member 1	0.88	1.33	1.5	0.9
LRRK2	LRRK2;AURA17;DARDARIN;PARK8;RIPK7;ROCO2	leucine-rich repeat kinase 2	1.1	1.38	1.26	1.15
B3GALT1	B3GALT1;MGC126594;beta3Gal-T1	UDP-Gal:betaGlcNAc beta 1,3-galactosyltransferase, polypeptide 1	0.97	1.23	1.27	0.96
CAPSL	CAPSL;MGC26610	calcyphosine-like	0.96	1.26	1.27	0.95
C2orf73	C2orf73;FLJ40298	chromosome 2 open reading frame 73	0.92	1.22	1.27	0.91
AIM2	AIM2;PYHIN4	absent in melanoma 2	0.88	1.14	1.27	0.87
HAVCR1	HAVCR1;HAVCR;HAVCR-1;KIM-1;KIM1;TIM;TIM-1;TIM1;TIMD-1;TIMD1	hepatitis A virus cellular receptor 1	1.02	1.34	1.28	1.05
AIF1	AIF1;AIF-1;IBA1;IRT-1;IRT1	allograft inflammatory factor 1	0.9	1.23	1.3	0.86
OR10K1	OR10K1;OR1-6	olfactory receptor, family 10, subfamily K, member 1	0.86	1.17	1.3	0.87
LRIT3	LRIT3;FIGLER4;FLJ44691;MGC120618	leucine-rich repeat, immunoglobulin-like and transmembrane domains 3	0.88	1.14	1.31	0.95
GYPB	GYPB;CD235b;GPB;GPB.NY;GYPHe.NY;HGpMiVI;MNS;PAS-3;SS	glycophorin B (MNS blood group)	0.9	1.25	1.39	0.85
EPYC	EPYC;DSPG3;PGLB;Pg-Lb;SLRR3B	epiphycan	1.07	1.34	1.41	1.05
HTR2A	HTR2A;5-HT2A;HTR2	5-hydroxytryptamine (serotonin) receptor 2A	0.88	1.14	1.41	0.86
OR5H6	OR5H6;OR3-11	olfactory receptor, family 5, subfamily H, member 6	1.04	1.29	1.26	0.97
OR8B3	OR8B3;OR11-311	olfactory receptor, family 8, subfamily B, member 3	1.08	1.33	1.27	1
TLR10	TLR10;CD290;MGC104967;MGC126398;MGC126399	toll-like receptor 10	0.86	1.11	1.27	0.86
LRRIQ1	LRRIQ1;FLJ12303	leucine-rich repeats and IQ motif containing 1	1.04	1.34	1.25	0.92
OR10A3	OR10A3;HSHTPCRX12;HTPCRX12	olfactory receptor, family 10, subfamily A, member 3	0.76	1.12	1.44	0.77
GC	GC;DBP;DBP/GC;GRD3;VDBG;VDBP	group-specific component (vitamin D binding protein)	0.61	0.9	1.46	0.72
TNIP3	TNIP3;ABIN-3;FLJ21162;LIND	TNFAIP3 interacting protein 3	0.68	1.03	1.47	0.78
PARP15	PARP15;BAL3;FLJ40196;FLJ40597;MGC126750;MGC126752;pART7	poly (ADP-ribose) polymerase family, member 15	0.76	1.03	1.52	0.81
SCGB1D2	SCGB1D2;LIPB;LPHB	secretoglobin, family 1D, member 2	0.82	1.08	1.28	0.72
OR4N2	OR4N2;OR14-13;OR14-8	olfactory receptor, family 4, subfamily N, member 2	0.76	1.04	1.31	0.79
HTN3	HTN3;HIS2;HTN2;HTN5	histatin 3	0.84	1.17	1.32	0.94
CYP2B6	CYP2B6;CPB6;CYP2B;CYP2B7;CYP2B7P;CYPIIB6;IIB1;P450	cytochrome P450, family 2, subfamily B, polypeptide 6	0.74	1	1.32	0.75
AADACL2	AADACL2;MGC72001	arylacetamide deacetylase-like 2	0.66	0.98	1.36	0.72
GABRA4	GABRA4	gamma-aminobutyric acid (GABA) A receptor, alpha 4	0.63	0.9	1.36	0.7
SPINK6	SPINK6;BUSI2;MGC21394;UNQ844	serine peptidase inhibitor, Kazal type 6	0.62	0.88	1.36	0.65
CDH12	CDH12;CDHB;FLJ34857	cadherin 12, type 2 (N-cadherin 2)	0.78	1.04	1.37	0.82
FHL5	FHL5;ACT;FLJ33049;KIAA0776;RP3-393D12.2;dJ393D12.2	four and a half LIM domains 5	0.62	0.94	1.37	0.65
ECM2	ECM2;MGC126355;MGC126356	extracellular matrix protein 2, female organ and adipocyte specific	0.76	1.03	1.38	0.83
CSN3	CSN3;CSN10;CSNK;KCA	casein kappa	0.83	1.21	1.39	0.81

HTR2B	HTR2B;5-HT(2B);5-HT2B	5-hydroxytryptamine (serotonin) receptor 2B	0.78	1.04	1.39	0.94
CTLA4	CTLA4;CD;CD152;CELIAC3;CTLA-4;GRD4;GSE;ICOS;IDDM12	cytotoxic T-lymphocyte-associated protein 4	0.68	1	1.4	0.66
LRRC31	LRRC31;FLJ23259;FLJ40019	leucine rich repeat containing 31	0.83	1.17	1.41	0.8
PTCHD3	PTCHD3;FLJ44037;MGC129888;PTR	patched domain containing 3	0.74	1.06	1.41	0.73
HHLA2	HHLA2	HERV-H LTR-associating 2	0.77	1.08	1.43	0.82
C6orf191	C6orf191;bA174C7.4	chromosome 6 open reading frame 191	0.79	1.04	1.27	0.79
OR10AG1	OR10AG1;OR11-160	olfactory receptor, family 10, subfamily AG, member 1	0.73	1	1.25	0.73
SAMD7	SAMD7;DKFZp686E1583	sterile alpha motif domain containing 7	1.18	1.46	1.12	1.06
OR8D1	OR8D1;JCG9;OR8D3;OST004;PDJ9J14	olfactory receptor, family 8, subfamily D, member 1	1.14	1.42	1.14	1.04
SLC39A12	SLC39A12;FLJ30499;LZT-Hs8;MGC43205;MGC51099;ZIP-12;bA570F3.1	solute carrier family 39 (zinc transporter), member 12	1.21	1.56	1.19	1.15
DEFB116	DEFB116;DEFB-16	defensin, beta 116	0.84	1.1	1.21	0.81
LYPLAL1	LYPLAL1;FLJ99730;KIAA1238	lysophospholipase-like 1	0.84	1.14	1.22	0.79
SMC1B	SMC1B;FLJ43748;SMC1BETA;SMC1L2;bK268H5;bK268H5.5	structural maintenance of chromosomes 1B	1.02	1.2	1.27	1.06
OR6C6	OR6C6	olfactory receptor, family 6, subfamily C, member 6	0.98	1.21	1.27	0.91
ZFP14	ZFP14;KIAA1559;ZNF531	zinc finger protein 14 homolog (mouse)	0.87	1.04	1.27	0.84
C10orf32	C10orf32;DKFZp686B2219;FLJ40752;MGC27171;RP11-753C18.6	chromosome 10 open reading frame 32	0.92	1.07	1.28	0.85
TAS2R50	TAS2R50;MGC138305;T2R50;T2R51;TAS2R51	taste receptor, type 2, member 50	0.9	1.1	1.28	0.8
OR9A2	OR9A2	olfactory receptor, family 9, subfamily A, member 2	0.98	1.15	1.3	0.91
TAAR8	TAAR8;GPR102;TA5;TAR5;TRAR5;TaR-5;TaR-8	trace amine associated receptor 8	1.05	1.27	1.31	0.96
CXCL13	CXCL13;ANGIE;ANGIE2;BCA-1;BCA1;BLC;BLR1L;SCYB13	chemokine (C-X-C motif) ligand 13	0.88	1.11	1.31	0.8
ANKFN1	ANKFN1;FLJ38335	ankyrin-repeat and fibronectin type III domain containing 1	0.9	1.08	1.33	0.84
CCDC67	CCDC67;FLJ25393	coiled-coil domain containing 67	0.86	1.02	1.34	0.8
PDE1A	PDE1A;HCAM1;HSPDE1A;MGC26303	phosphodiesterase 1A, calmodulin-dependent	0.9	1.03	1.25	0.91
OR13C8	OR13C8;OR37H;OR9-10	olfactory receptor, family 13, subfamily C, member 8	0.9	1.1	1.25	0.83
OR5D14	OR5D14;OR11-141;OR11-150	olfactory receptor, family 5, subfamily D, member 14	0.87	0.98	1.25	0.78
OR10H4	OR10H4;OR19-28	olfactory receptor, family 10, subfamily H, member 4	0.64	0.82	1.45	0.69
CYP2C19	CYP2C19;CPCJ;CYP2C;P450C2C;P450IIC19	cytochrome P450, family 2, subfamily C, polypeptide 19	0.72	0.96	1.46	0.85
CCDC83	CCDC83;FLJ42119;MGC34732	coiled-coil domain containing 83	0.84	0.94	1.26	0.74
STAP1	STAP1;BRDG1;STAP-1	signal transducing adaptor family member 1	0.74	0.96	1.26	0.73
CASP5	CASP5;ICE(rel)III;ICEREL-III;ICH-3;MGC141966	caspase 5, apoptosis-related cysteine peptidase	0.71	0.82	1.26	0.61
CD200R1	CD200R1;CD200R;HCRTR2;MOX2R;OX2R	CD200 receptor 1	0.7	0.93	1.26	0.67
MS4A13	MS4A13	membrane-spanning 4-domains, subfamily A, member 13	0.65	0.84	1.26	0.64
SSX3	SSX3;CT5.3;MGC119054;MGC14495	synovial sarcoma, X breakpoint 3	0.65	0.74	1.26	0.59
CXorf58	CXorf58;FLJ25444	chromosome X open reading frame 58	0.62	0.79	1.26	0.57
GPR18	GPR18	G protein-coupled receptor 18	0.62	0.75	1.26	0.68
IQCF2	IQCF2;MGC50809	IQ motif containing F2	0.61	0.74	1.26	0.57
SSX9	SSX9	synovial sarcoma, X breakpoint 9	0.8	1.01	1.27	0.83
OR6B2	OR6B2;OR2-1;OR6B2P	olfactory receptor, family 6, subfamily B, member 2	0.72	0.84	1.27	0.66
ANP32D	ANP32D;PP32R2	acidic (leucine-rich) nuclear phosphoprotein 32 family, member D	0.72	0.88	1.27	0.68
CNTN6	CNTN6;MGC133256;NB3	contactin 6	0.68	0.88	1.27	0.68
SLCO1B1	SLCO1B1;LST-1;LST1;MGC133282;OATP-C;OATP1B1;OATP2;OATPC;SLC21A6	solute carrier organic anion transporter family, member 1B1	0.67	0.86	1.27	0.67
PLAC1L	PLAC1L;FLJ36198;TMEM122	placenta-specific 1-like	0.66	0.82	1.27	0.62
FGA	FGA;Fib2;MGC119422;MGC119423;MGC119425	fibrinogen alpha chain	0.66	0.9	1.27	0.59
IL1RAPL2	IL1RAPL2;IL-1R9;IL1R9;IL1RAPL-2;TIGIRR-1	interleukin 1 receptor accessory protein-like 2	0.66	0.82	1.27	0.64
CSTA	CSTA;AREI;STF1;STFA	cystatin A (stefin A)	0.81	0.99	1.28	0.77
ZNF99	ZNF99;C19orf9;F8281;MGC24986	zinc finger protein 99	0.74	0.92	1.28	0.7
C4orf45	C4orf45;FLJ25371	chromosome 4 open reading frame 45	0.73	0.9	1.28	0.69
GPRC6A	GPRC6A;GPCR;bA86F4.3	G protein-coupled receptor, family C, group 6, member A	0.64	0.75	1.28	0.57
BRDT	BRDT;BRD6;CT9	bromodomain, testis-specific	0.61	0.84	1.28	0.6
DYNLRB2	DYNLRB2;DNCL2B;DNLC2B;MGC62033;ROBLD2	dynein, light chain, roadblock-type 2	0.7	0.84	1.29	0.75
GDPD4	GDPD4;GDE6	glycerophosphodiester phosphodiesterase domain containing 4	0.7	0.88	1.29	0.66
AGR2	AGR2;AG2;GOB-4;HAG-2;PDIA17;XAG-2	anterior gradient homolog 2 (Xenopus laevis)	0.69	0.88	1.29	0.73
RPS4Y2	RPS4Y2;RPS4Y2P	ribosomal protein S4, Y-linked 2	0.67	0.89	1.29	0.6
OR5B21	OR5B21	olfactory receptor, family 5, subfamily B, member 21	0.64	0.86	1.29	0.62
C1orf192	C1orf192	chromosome 1 open reading frame 192	0.62	0.82	1.29	0.59
C10orf107	C10orf107;MGC44593;bA63A2.1	chromosome 10 open reading frame 107	0.76	0.96	1.3	0.73
KLHDC1	KLHDC1;MGC126644;MGC126646;MST025	kelch domain containing 1	0.75	0.97	1.3	0.8
FAP	FAP;DKFZp686G13158;DPPIV;FAPA	fibroblast activation protein, alpha	0.8	0.95	1.31	0.71
CPB1	CPB1;CPB;DKFZp779K1333;PASP;PCPB	carboxypeptidase B1 (tissue)	0.75	0.98	1.31	0.73
MS4A3	MS4A3;CD20L;HTM4	membrane-spanning 4-domains, subfamily A, member 3 (hematopoietic cell-specific)	0.74	0.94	1.31	0.79
C3orf15	C3orf15;AAT1;AAT1alpha;DKFZp781A2221	chromosome 3 open reading frame 15	0.66	0.86	1.31	0.62
IFNA6	IFNA6;IFN-alphaK	interferon, alpha 6	0.66	0.77	1.31	0.65
FPR3	FPR3;FML2_HUMAN;FMLPY;FPRH1;FPRH2;FPRL2;RMLP-R-I	formyl peptide receptor 3	0.76	0.97	1.32	0.88
AFP	AFP;FETA;HPAFP	alpha-fetoprotein	0.7	0.91	1.32	0.66
PDZRN4	PDZRN4;LNX4;SAMCAP3L	PDZ domain containing ring finger 4	0.68	0.89	1.32	0.8
BRS3	BRS3	bombesin-like receptor 3	0.67	0.82	1.32	0.62
OR13G1	OR13G1;OR1-37	olfactory receptor, family 13, subfamily G, member 1	0.63	0.84	1.32	0.57
SULT1E1	SULT1E1;EST;EST-1;MGC34459;ST1E1;STE	sulfotransferase family 1E, estrogen-preferring, member 1	0.7	0.94	1.33	0.73
MYH1	MYH1;MGC133384;MYHSA1;MYHa;MyHC-2X/D;MyHC-2x	myosin, heavy chain 1, skeletal muscle, adult	0.64	0.8	1.33	0.71
ELAVL4	ELAVL4;HUD;PNEM	ELAV (embryonic lethal, abnormal vision, Drosophila)-like 4 (Hu antigen D)	0.63	0.86	1.33	0.66
NPVF	NPVF;C7orf9;RFRP	neuropeptide VF precursor	0.8	1.03	1.34	0.79
KRTAP13-1	KRTAP13-1;KAP13.1;KRTAP13.1	keratin associated protein 13-1	0.74	0.91	1.34	0.72
CLIC2	CLIC2;CLIC2b;XAP121	chloride intracellular channel 2	0.62	0.82	1.34	0.58
P2RY10	P2RY10;P2Y10	purinergic receptor P2Y, G-protein coupled, 10	0.67	0.88	1.35	0.79
UBE2U	UBE2U;MGC35130;RP4-636O23.1	ubiquitin-conjugating enzyme E2U (putative)	0.82	1.03	1.36	0.77
OR6X1	OR6X1;OR11-270	olfactory receptor, family 6, subfamily X, member 1	0.66	0.88	1.36	0.66
GSTA5	GSTA5	glutathione S-transferase alpha 5	0.62	0.79	1.37	0.63
OR9K2	OR9K2;OR12-2	olfactory receptor, family 9, subfamily K, member 2	0.8	1	1.38	0.79
OR10A5	OR10A5;JCG6;OR10A1;OR11-403	olfactory receptor, family 10, subfamily A, member 5	0.7	0.93	1.38	0.73
OMG	OMG;OMGP	oligodendrocyte myelin glycoprotein	0.74	0.95	1.39	0.72
TDRD5	TDRD5;FLJ34823;MGC163404;RP11-427G13.1;TUDOR3	tudor domain containing 5	0.76	0.9	1.25	0.65
AMTN	AMTN;MGC148132;MGC148133;UNQ689	amelotin	0.74	0.88	1.25	0.68
TUBAL3	TUBAL3;FLJ21665;MGC119347;MGC119349	tubulin, alpha-like 3	0.71	0.84	1.25	0.66
OR52N1	OR52N1;OR11-61	olfactory receptor, family 52, subfamily N, member 1	0.71	0.9	1.25	0.62
OR8K3	OR8K3;OR11-181	olfactory receptor, family 8, subfamily K, member 3	0.69	0.9	1.25	0.6
RAB39	RAB39	RAB39, member RAS oncogene family	0.69	0.9	1.25	0.6
TAS2R9	TAS2R9;T2R9;TRB6	taste receptor, type 2, member 9	0.68	0.8	1.25	0.62
CD36	CD36;BDPLT10;CHDS7;FAT;GP3B;GP4;GPIV;PASIV;SCARB3	CD36 molecule (thrombospondin receptor)	0.66	0.9	1.25	0.67
DSG3	DSG3;CDHF6;DKFZp686P23184;PVA	desmoglein 3	0.64	0.77	1.25	0.62
IFNA1	IFNA1;IFL;IFN;IFN-ALPHA;IFN-alphaD;IFNA13;IFNA@;MGC138207;MGC138505;MGC138507	interferon, alpha 1	1.15	0.98	0.81	0.74
OR5M3	OR5M3;OR11-191	olfactory receptor, family 5, subfamily M, member 3	1.02	0.84	0.81	0.57
DKK1	DKK1;DKK-1;SK	dickkopf homolog 1 (Xenopus laevis)	1	0.84	0.81	0.6
CDH9	CDH9;MGC125386	cadherin 9, type 2 (T1-cadherin)	1.17	0.98	0.82	0.77
OR5M10	OR5M10;OR11-207	olfactory receptor, family 5, subfamily M, member 10	0.97	0.82	0.82	0.62
CCDC36	CCDC36;CT74;FLJ25320	coiled-coil domain containing 36	0.94	0.76	0.82	0.55
CDR1	CDR1;CDR;CDR34;CDR62A	cerebellar degeneration-related protein 1, 34kDa	0.92	0.78	0.82	0.56
SCGB2A2	SCGB2A2;MGB1;MGC71974;UGB2	secretoglobin, family 2A, member 2	0.88	0.7	0.82	0.57
FABP4	FABP4;A-FABP;AFABP;ALBP;aP2	fatty acid binding protein 4, adipocyte	1.08	0.96	0.83	0.63
GLRA3	GLRA3	glycine receptor, alpha 3	1.08	0.94	0.83	0.73
IL2	IL2;IL-2;TCGF;lymphokine	interleukin 2	1.04	0.9	0.83	0.6
OR14A16	OR14A16;OR5AT1	olfactory receptor, family 14, subfamily A, member 16	1.02	0.92	0.83	0.68
SCN3A	SCN3A;KIAA1356;NAC3;Nav1.3	sodium channel, voltage-gated, type III, alpha subunit	0.95	0.84	0.83	0.65
OR5J2	OR5J2;OR11-266	olfactory receptor, family 5, subfamily J, member 2	0.95	0.86	0.83	0.63
ST18	ST18;KIAA0535;ZNF387	suppression of tumorigenicity 18 (breast carcinoma) (zinc finger protein)	0.88	0.76	0.83	0.62
LRRC19	LRRC19;FLJ21302	leucine rich repeat containing 19	1.02	0.88	0.84	0.67
ABCC9	ABCC9;ABC37;ATFB12;CMD1O;FLJ36852;SUR2	ATP-binding cassette, sub-family C (CFTR/MRP), member 9	1.02	0.89	0.84	0.69
DSG4	DSG4;CDGF13;CDHF13;LAH	desmoglein 4	1.02	0.88	0.84	0.67
BPY2	BPY2;BPY2A;VCY2;VCY2A	basic charge, Y-linked, 2	1	0.89	0.84	0.63
ACCN5	ACCN5;HINAC;INAC	amiloride-sensitive cation channel 5, intestinal	0.97	0.84	0.84	0.65
ZNF846	ZNF846	zinc finger protein 846	0.92	0.8	0.84	0.53
ABHD12B	ABHD12B;BEM46L3;C14orf29;MGC129926;MGC129927;c14_5314	abhydrolase domain containing 12B	0.9	0.84	0.84	0.58
MYOC	MYOC;GLC1A;GPOA;JOAG;JOAG1;TIGR;myocilin	myocilin, trabecular meshwork inducible glucocorticoid response	0.88	0.77	0.84	0.52
LIPF	LIPF;GL;HGL;HLAL;MGC138477;MGC142271	lipase, gastric	1.34	1.16	0.85	0.92
GRIA3	GRIA3;GLUR-C;GLUR-K3;GLUR3;GLURC;GluA3;MRX94	glutamate receptor, ionotrophic, AMPA 3	1.1	0.92	0.85	0.72
PTPRZ1	PTPRZ1;HPTPZ;HPTPzeta;PTP-ZETA;PTP18;PTPRZ;PTPZ;RPTPB;RPTPbeta;phosphacan	protein tyrosine phosphatase, receptor-type, Z polypeptide 1	1.05	0.9	0.85	0.7
YIPF7	YIPF7;FLJ39576;FinGER9;MGC120164	Yip1 domain family, member 7	0.98	0.89	0.85	0.68
DCLK3	DCLK3;CLR;DCAMKL3;DCDC3C;DCK3;KIAA1765	doublecortin-like kinase 3	0.96	0.79	0.85	0.71
CHRNA6	CHRNA6;CHNRA6	cholinergic receptor, nicotinic, alpha 6	0.92	0.82	0.85	0.64
OR2J3	OR2J3;6M1-3;HS6M1-3;OR6-16;OR6-6;OR6.3.6;ORL671	olfactory receptor, family 2, subfamily J, member 3	0.92	0.76	0.85	0.58
RNASE10	RNASE10;RNASE9	ribonuclease, RNase A family, 10 (non-active)	0.87	0.8	0.85	0.61
PRAMEF2	PRAMEF2;DJ845O24.3;FLJ43580;RP5-845O24.1	PRAME family member 2	1.14	0.98	0.86	0.71
AMBN	AMBN	ameloblastin (enamel matrix protein)	1.07	0.88	0.86	0.67
KRTAP19-4	KRTAP19-4;KAP19.4	keratin associated protein 19-4	1.06	0.93	0.86	0.71
RFPL3	RFPL3	ret finger protein-like 3	1.03	0.88	0.86	0.79
TTC29	TTC29;NYD-SP14;TBPP2A	tetratricopeptide repeat domain 29	0.99	0.89	0.86	0.64
OR4B1	OR4B1;OR11-106;OST208	olfactory receptor, family 4, subfamily B, member 1	0.99	0.82	0.86	0.63
OVCH2	OVCH2;OVTN	ovochymase 2 (gene/pseudogene)	0.97	0.8	0.86	0.58
OR4S2	OR4S2;OR11-137;OR4S2P;OST725	olfactory receptor, family 4, subfamily S, member 2	0.94	0.84	0.86	0.69
STK31	STK31;FLJ16102;SGK396;TDRD8	serine/threonine kinase 31	0.93	0.8	0.86	0.66

NOX1	NOX1;GP91-2;MOX1;NOH-1;NOH1	NADPH oxidase 1	0.9	0.82	0.86	0.63
HORMAD1	HORMAD1;CT46;DKFZp434A1315;NOHMA	HORMA domain containing 1	0.86	0.78	0.86	0.55
MYOT	MYOT;LGMD1;LGMD1A;TTID	myotilin	1.38	1.19	0.87	0.89
RGS21	RGS21	regulator of G-protein signaling 21	1.17	0.98	0.87	0.79
RNF133	RNF133;MGC27072	ring finger protein 133	1.15	0.96	0.87	0.69
EFHB	EFHB;FLJ25200	EF-hand domain family, member B	1.09	0.94	0.87	0.68
ZNF214	ZNF214;BAZ-1;BAZ1	zinc finger protein 214	1.08	0.91	0.87	0.69
C12orf69	C12orf69	chromosome 12 open reading frame 69	1.07	0.99	0.87	0.69
ASB15	ASB15;DKFZp779M1258;FLJ43370	ankyrin repeat and SOCS box containing 15	1.07	0.89	0.87	0.64
ZSWIM2	ZSWIM2;MEX;MGC33890;ZZZ2	zinc finger, SWIM-type containing 2	1.06	0.91	0.87	0.82
AKR1C4	AKR1C4;3-alpha-HSD;C11;CDR;CHDR;DD-4;DD4;HAKRA;MGC22581	aldo-keto reductase family 1, member C4 (chlordecone reductase; 3-alpha hydroxysteroid dehydrogenase, type I; dihydrodiol dehydrogenase 4)	1.05	0.93	0.87	0.62
PDC	PDC;MEKA;PHD;PhLOP;PhLP	phosducin	0.97	0.82	0.87	0.74
C9orf153	C9orf153;MGC131702;bA507D14.1	chromosome 9 open reading frame 153	0.9	0.8	0.87	0.59
PSG11	PSG11;MGC22484;PSBG-11;PSBG-13;PSG13;PSG14	pregnancy specific beta-1-glycoprotein 11	0.88	0.76	0.87	0.57
OR5M1	OR5M1;OR11-208;OST050	olfactory receptor, family 5, subfamily M, member 1	1.11	0.93	0.88	0.72
C3orf43	C3orf43;DKFZp313B0440;FLJ41923;MGC141734;MGC141735	chromosome 3 open reading frame 43	1.09	0.99	0.88	0.74
EDNRB	EDNRB;ABCDS;ET-B;ET-BR;ETB;ETBR;ETRB;HSCR;HSCR2;WS4A	endothelin receptor type B	1.09	0.9	0.88	0.67
TMEM100	TMEM100;FLJ10970;FLJ37856	transmembrane protein 100	0.94	0.9	0.88	0.6
OR4A47	OR4A47;OR11-113	olfactory receptor, family 4, subfamily A, member 47	0.92	0.79	0.88	0.62
OR4A5	OR4A5;OR11-111	olfactory receptor, family 4, subfamily A, member 5	0.87	0.8	0.88	0.56
MS4A1	MS4A1;B1;Bp35;CD20;CVID5;LEU-16;MGC3969;MS4A2;S7	membrane-spanning 4-domains, subfamily A, member 1	0.87	0.76	0.88	0.53
GIMAP4	GIMAP4;FLJ11110;HIMAP4;IAN1;IMAP4;hIAN1	GTPase, IMAP family member 4	0.87	0.82	0.88	0.57
TRPA1	TRPA1;ANKTM1	transient receptor potential cation channel, subfamily A, member 1	1.2	1.12	0.89	0.86
C6orf163	C6orf163	chromosome 6 open reading frame 163	1.05	0.96	0.89	0.66
CYP3A43	CYP3A43;MGC119315;MGC119316	cytochrome P450, family 3, subfamily A, polypeptide 43	1	0.86	0.89	0.68
ITGA8	ITGA8	integrin, alpha 8	0.96	0.86	0.89	0.6
BPI	BPI;BPIFD1;rBPI	bactericidal/permeability-increasing protein	0.96	0.95	0.89	0.65
PDE6C	PDE6C;COD4;PDEA2	phosphodiesterase 6C, cGMP-specific, cone, alpha prime	0.93	0.8	0.89	0.6
PROX2	PROX2;FLJ36749;PROX-2	prospero homeobox 2	0.93	0.8	0.89	0.61
C1QTNF9	C1QTNF9;AQL1;C1QTNF9A;CTRP9;MGC48915	C1q and tumor necrosis factor related protein 9	0.92	0.86	0.89	0.62
OR10J1	OR10J1;HGMP07J;HSHGMP07J;MGC138495;MGC138497	olfactory receptor, family 10, subfamily J, member 1	0.91	0.85	0.89	0.69
C16orf73	C16orf73;MGC35212;gs129	chromosome 16 open reading frame 73	0.86	0.82	0.89	0.58
C9	C9	complement component 9	0.86	0.68	0.89	0.57
MCF2	MCF2;ARHGEF21;DBL	MCF.2 cell line derived transforming sequence	1.19	1.07	0.9	0.79
CCL8	CCL8;HC14;MCP-2;MCP2;SCYA10;SCYA8	chemokine (C-C motif) ligand 8	1.18	1.09	0.9	0.83
DMP1	DMP1;ARHP;ARHR;DMP-1	dentin matrix acidic phosphoprotein 1	1.08	0.97	0.9	0.66
OR4E2	OR4E2;OR14-42	olfactory receptor, family 4, subfamily E, member 2	1.07	0.95	0.9	0.72
AMELX	AMELX;AI1E;AIH1;ALGN;AMG;AMGL;AMGX	amelogenin, X-linked	1.06	0.92	0.9	0.73
OR52E2	OR52E2	olfactory receptor, family 52, subfamily E, member 2	1	0.92	0.9	0.62
CRYGB	CRYGB;CRYG2	crystallin, gamma B	0.96	0.77	0.9	0.62
SCN7A	SCN7A;SCN6A	sodium channel, voltage-gated, type VII, alpha	0.94	0.89	0.9	0.69
PTTG2	PTTG2	pituitary tumor-transforming 2	0.9	0.84	0.9	0.56
HSD3B2	HSD3B2;HSD3B;HSDB;SDR11E2	hydroxy-delta-5-steroid dehydrogenase, 3 beta- and steroid delta-isomerase 2	0.9	0.86	0.9	0.56
NLRP13	NLRP13;CLR19.7;NALP13;NOD14;PAN13	NLR family, pyrin domain containing 13	0.88	0.83	0.9	0.55
CCDC73	CCDC73;NY-SAR-79	coiled-coil domain containing 73	0.86	0.77	0.9	0.56
CHRNB3	CHRNB3	cholinergic receptor, nicotinic, beta 3	0.86	0.8	0.9	0.62
OR4C15	OR4C15;OR11-127;OR11-134	olfactory receptor, family 4, subfamily C, member 15	1.25	1.17	0.91	0.89
ZNF645	ZNF645;FLJ25735;HAKAIL	zinc finger protein 645	1.25	1.08	0.91	0.78
FOXP2	FOXP2;CAGH44;DKFZp686H1726;SPCH1;TNRC10	forkhead box P2	1.2	1.14	0.91	0.88
SYCP1	SYCP1;CT8;HOM-TES-14;MGC104417;SCP1	synaptonemal complex protein 1	1.14	0.99	0.91	0.76
NLRP11	NLRP11;CLR19.6;FLJ26273;NALP11;NOD17;PAN10;PYPAF6;PYPAF7	NLR family, pyrin domain containing 11	1.07	0.96	0.91	0.74
CPA3	CPA3;MC-CPA	carboxypeptidase A3 (mast cell)	1.06	0.95	0.91	0.71
C8orf45	C8orf45;FLJ25692	chromosome 8 open reading frame 45	1.02	0.88	0.91	0.71
CSN2	CSN2;CASB	casein beta	0.99	0.9	0.91	0.63
OR6C2	OR6C2;OR6C67	olfactory receptor, family 6, subfamily C, member 2	0.96	0.82	0.91	0.69
PRKG1	PRKG1;1;DKFZp686K042;FLJ36117;MGC71944;PKG;PRKG1B;PRKGR1B;cGK;cGK 1;cGK1;cGKI;cGKI-BETA;cGKI-alpha	protein kinase, cGMP-dependent, type I	0.86	0.78	0.91	0.64
FIGF	FIGF;VEGF-D;VEGFD	c-fos induced growth factor (vascular endothelial growth factor D)	1.33	1.25	0.92	0.99
GABRG2	GABRG2;CAE2;ECA2;GEFSP3	gamma-aminobutyric acid (GABA) A receptor, gamma 2	1.18	1.08	0.92	0.84
HMGCLL1	HMGCLL1;DKFZp434G1411;bA418P12.1	3-hydroxymethyl-3-methylglutaryl-CoA lyase-like 1	1.15	0.99	0.92	0.87
FKSG2	FKSG2	tumor protein, translationally-controlled 1 pseudogene	1.13	1	0.92	0.76
RFPL1	RFPL1;MGC132428;RNF78	ret finger protein-like 1	1.11	1.01	0.92	0.91
NKAIN3	NKAIN3;FAM77D;FLJ39630	Na+/K+ transporting ATPase interacting 3	1.08	1.1	0.92	0.8
NHLH2	NHLH2;HEN2;KIAA0490;NSCL2;bHLHa34	nescient helix loop helix 2	1.06	0.92	0.92	0.78
CD40LG	CD40LG;CD154;CD40L;HIGM1;IGM;IMD3;T-BAM;TNFSF5;TRAP;gp39;hCD40L	CD40 ligand	1.02	0.88	0.92	0.71
KRTAP3-3	KRTAP3-3;KAP3.3;KRTAP3.3;MGC95374	keratin associated protein 3-3	1.02	0.88	0.92	0.75
MEP1B	MEP1B	meprin A, beta	1	0.9	0.92	0.68
DHRS9	DHRS9;3ALPHA-HSD;RDH15;RDHL;RDHTBE;RETSDR8;SDR9C4	dehydrogenase/reductase (SDR family) member 9	0.99	0.92	0.92	0.73
CCDC148	CCDC148;MGC125588;MGC125590	coiled-coil domain containing 148	0.88	0.83	0.92	0.62
BMX	BMX;ETK;PSCTK2;PSCTK3	BMX non-receptor tyrosine kinase	0.87	0.86	0.92	0.61
LY96	LY96;ESOP-1;MD-2;MD2;ly-96	lymphocyte antigen 96	0.86	0.82	0.92	0.56
SLCO1C1	SLCO1C1;OATP-F;OATP1;OATP14;OATP1C1;OATPF;OATPRP5;SLC21A14	solute carrier organic anion transporter family, member 1C1	1.38	1.25	0.93	0.88
TSLP	TSLP	thymic stromal lymphopoietin	1.25	1.18	0.93	0.8
CR1	CR1;C3BR;C4BR;CD35;KN	complement component (3b/4b) receptor 1 (Knops blood group)	1.2	1.1	0.93	0.83
TAAR1	TAAR1;MGC126874;MGC138399;RP11-295F4.9;TA1;TAR1;TRAR1	trace amine associated receptor 1	1.17	1.06	0.93	0.95
LRRN3	LRRN3;FIGLER5;FLJ11129;NLRR-3;NLRR3	leucine rich repeat neuronal 3	1.09	1.06	0.93	0.74
ZFP42	ZFP42;REX1;ZNF754	zinc finger protein 42 homolog (mouse)	0.99	0.88	0.93	0.64
UGT2B17	UGT2B17;UDPGT2B17	UDP glucuronosyltransferase 2 family, polypeptide B17	0.97	0.94	0.93	0.73
ZNF677	ZNF677;MGC48625	zinc finger protein 677	0.97	0.88	0.93	0.67
S100A7L2	S100A7L2;S100a7b	S100 calcium binding protein A7-like 2	0.96	0.8	0.93	0.62
PROL1	PROL1;BPLP;PRL1;opiorphin	proline rich, lacrimal 1	0.95	0.86	0.93	0.69
TTC6	TTC6	ENTREZ STATUS: REMAPPED TO GENE ID: 319089	0.94	0.88	0.93	0.64
PLN	PLN;CMD1P;CMH18;PLB	phospholamban	0.9	0.8	0.93	0.67
LMOD3	LMOD3;DKFZp313F0135	leiomodin 3 (fetal)	0.9	0.83	0.93	0.57
OR8B4	OR8B4;OR11-315;OR8B4P	olfactory receptor, family 8, subfamily B, member 4	0.9	0.82	0.93	0.68
CXCL11	CXCL11;H174;I-TAC;IP-9;IP9;MGC102770;SCYB11;SCYB9B;b-R1	chemokine (C-X-C motif) ligand 11	0.89	0.86	0.93	0.58
C1orf105	C1orf105	chromosome 1 open reading frame 105	0.88	0.88	0.93	0.67
OR2D3	OR2D3;OR11-89	olfactory receptor, family 2, subfamily D, member 3	0.86	0.77	0.93	0.56
CA1	CA1;CA-I;CAB;Car1	carbonic anhydrase I	1.38	1.27	0.94	0.99
PTGER3	PTGER3;EP3;EP3-I;EP3-II;EP3-III;EP3-IV;EP3e;MGC141828;MGC141829;MGC27302;PGE2-R	prostaglandin E receptor 3 (subtype EP3)	1.21	1.11	0.94	1
OR8H1	OR8H1;OR11-180	olfactory receptor, family 8, subfamily H, member 1	1.15	0.98	0.94	0.78
GYPA	GYPA;CD235a;GPA;GPErik;GPSAT;GpMiIII;HGpMiIII;HGpMiV;HGpMiX;HGpMiXI;HGpSta(C);MN;MNS;PAS-2	glycophorin A (MNS blood group)	1.08	0.98	0.94	0.7
FLJ44838	FLJ44838	uncharacterized LOC644767	1.07	1.06	0.94	0.74
OR10A7	OR10A7;OR12-6	olfactory receptor, family 10, subfamily A, member 7	1.06	1.04	0.94	0.77
CTAGE1	CTAGE1;CT21.1;CT21.2;CTAGE;CTAGE-1;CTAGE-2	cutaneous T-cell lymphoma-associated antigen 1	0.99	0.96	0.94	0.66
FREM2	FREM2;DKFZp686J0811;DKFZp781I048	FRAS1 related extracellular matrix protein 2	0.99	0.84	0.94	0.7
SCN1A	SCN1A;EIEE6;FEB3;FEB3A;FHM3;GEFSP2;HBSCI;NAC1;Nav1.1;SCN1;SMEI	sodium channel, voltage-gated, type I, alpha subunit	0.97	0.96	0.94	0.74
OR4A16	OR4A16;OR11-117;OR4A16Q	olfactory receptor, family 4, subfamily A, member 16	0.96	0.88	0.94	0.66
GUCA1C	GUCA1C;GCAP3;MGC120158;MGC120159	guanylate cyclase activator 1C	0.95	0.85	0.94	0.67
DLG2	DLG2;DKFZp781D1854;DKFZp781E0954;FLJ37266;MGC131811;PPP1R58;PSD-93;PSD93;chapsyn-110	discs, large homolog 2 (Drosophila)	0.94	0.96	0.94	0.68
RAB3C	RAB3C	RAB3C, member RAS oncogene family	0.91	0.8	0.94	0.63
GTSF1	GTSF1;FAM112B;FLJ32942	gametocyte specific factor 1	0.9	0.86	0.94	0.64
RBM11	RBM11	RNA binding motif protein 11	0.9	0.84	0.94	0.62
C4orf17	C4orf17;DKFZp434G072	chromosome 4 open reading frame 17	0.89	0.9	0.94	0.63
PRDM9	PRDM9;MEISETZ;MSBP3;PFM6;PRMD9;ZNF899	PR domain containing 9	0.88	0.78	0.94	0.57
RNASE11	RNASE11;C14orf6	ribonuclease, RNase A family, 11 (non-active)	1.1	1.12	0.95	0.86
ASB5	ASB5;ASB-5	ankyrin repeat and SOCS box containing 5	1.1	1	0.95	0.72
LRMP	LRMP;JAW1	lymphoid-restricted membrane protein	1.06	0.98	0.95	0.79
EMCN	EMCN;EMCN2;MUC14	endomucin	1.05	1.02	0.95	0.73
WDR17	WDR17;FLJ26618	WD repeat domain 17	1.04	0.96	0.95	0.73
DEFB110	DEFB110;DEFB-10;DEFB-11;DEFB111;MGC183056;MGC190230	defensin, beta 110 locus	1.02	1	0.95	0.68
NAALAD2	NAALAD2;GCPIII;GPCIII;MGC116996;MGC26353;NAADALASE2;NAALADASE2	N-acetylated alpha-linked acidic dipeptidase 2	1	0.88	0.95	0.66
OR5W2	OR5W2;OR5W2P;OR5W3P	olfactory receptor, family 5, subfamily W, member 2	1	0.99	0.95	0.74
CASP14	CASP14;MGC119078;MGC119079	caspase 14, apoptosis-related cysteine peptidase	0.99	0.92	0.95	0.69
OR5T2	OR5T2;OR11-177	olfactory receptor, family 5, subfamily T, member 2	0.94	0.86	0.95	0.66
C1orf129	C1orf129;ARMC11;FLJ23550	chromosome 1 open reading frame 129	0.92	0.91	0.95	0.65
PRG4	PRG4;CACP;FLJ32635;HAPO;JCAP;MSF;SZP;bG174L6.2	proteoglycan 4	0.92	0.92	0.95	0.75
DSG1	DSG1;CDHF4;DG1;DSG;PPKS1;SPPK1	desmoglein 1	0.92	0.91	0.95	0.74
IFNA2	IFNA2;IFN-alphaA;IFNA;INFA2;MGC125764;MGC125765	interferon, alpha 2	0.9	0.86	0.95	0.6
GLIPR1L1	GLIPR1L1;ALKN2972;MGC26856;PRO7434	GLI pathogenesis-related 1 like 1	0.86	0.86	0.95	0.62
FGG	FGG	fibrinogen gamma chain	1.14	1.11	0.96	0.88
FBXO15	FBXO15;FBX15;MGC39671	F-box protein 15	1.12	1.04	0.96	0.75
CLLU1	CLLU1	chronic lymphocytic leukemia up-regulated 1	1.03	1.08	0.96	0.79
ABCB4	ABCB4;ABC21;GBD1;MDR2;MDR2/3;MDR3;PFIC-3;PGY3	ATP-binding cassette, sub-family B (MDR/TAP), member 4	0.95	0.9	0.96	0.67
FAM9C	FAM9C	family with sequence similarity 9, member C	0.94	0.99	0.96	0.73
MBD3L1	MBD3L1;MBD3L;MGC138263;MGC138269	methyl-CpG binding domain protein 3-like 1	0.9	0.9	0.96	0.68
GSTA2	GSTA2;GST2;GSTA2-2;GTA2;GTH2;MGC10525	glutathione S-transferase alpha 2	0.89	0.84	0.96	0.72
BTNL3	BTNL3;BTNLR	butyrophilin-like 3	0.86	0.86	0.96	0.59
DPPA4	DPPA4;2410091M23Rik;FLJ10713	developmental pluripotency associated 4	1.21	1.06	0.97	0.88
RNF133	RNF133;MGC27072	ring finger protein 133	1.17	1.12	0.97	0.84

MGAM	MGAM;MG;MGA	maltase-glucoamylase (alpha-glucosidase)	1.12	1.04	0.97	0.75
ZNF705A	ZNF705A;FLJ16353	zinc finger protein 705A	1.06	1.02	0.97	0.74
MMP13	MMP13;CLG3;MANDP1	matrix metallopeptidase 13 (collagenase 3)	1.05	0.94	0.97	0.71
ZNF540	ZNF540;DKFZp313K2238;DKFZp547B0714;FLJ16004;Nbla10512	zinc finger protein 540	1.04	0.97	0.97	0.73
TFPI	TFPI;EPI;LACI;TFI;TFPI1	tissue factor pathway inhibitor (lipoprotein-associated coagulation inhibitor)	1.02	0.92	0.97	0.73
C12orf39	C12orf39;MGC10946	chromosome 12 open reading frame 39	1	0.97	0.97	0.69
CRTAM	CRTAM;CD355	cytotoxic and regulatory T cell molecule	0.97	1.02	0.97	0.75
OR10R2	OR10R2;OR1-8;OR10R2Q	olfactory receptor, family 10, subfamily R, member 2	0.96	0.96	0.97	0.73
IKZF1	IKZF1;Hs.54452;IK1;IKAROS;LYF1;PRO0758;ZNFN1A1;hIk-1	IKAROS family zinc finger 1 (Ikaros)	0.96	0.86	0.97	0.76
SULT2A1	SULT2A1;DHEA-ST;DHEAS;HST;ST2;ST2A1;ST2A3;STD;hSTa	sulfotransferase family, cytosolic, 2A, dehydroepiandrosterone (DHEA)-preferring, member 1	0.96	0.89	0.97	0.7
OR8K5	OR8K5;OR11-174	olfactory receptor, family 8, subfamily K, member 5	0.94	0.86	0.97	0.73
DNAH7	DNAH7;DKFZp686C09101;FLJ37196;KIAA0944;MGC39580	dynein, axonemal, heavy chain 7	0.9	0.85	0.97	0.66
GPR171	GPR171;H963	G protein-coupled receptor 171	0.88	0.84	0.97	0.76
UGT2A3	UGT2A3;FLJ21934	UDP glucuronosyltransferase 2 family, polypeptide A3	0.86	0.93	0.97	0.64
SH2D1B	SH2D1B;EAT2	SH2 domain containing 1B	0.86	0.79	0.97	0.64
OR11H4	OR11H4;OR14-36	olfactory receptor, family 11, subfamily H, member 4	1.33	1.33	0.98	1.05
SPZ1	SPZ1;FLJ25709;NYD-TSP1	spermatogenic leucine zipper 1	1.17	1.11	0.98	0.83
RBMY1B	RBMY1B	RNA binding motif protein, Y-linked, family 1, member B	1.09	1.08	0.98	0.73
OR4C12	OR4C12;OR11-259	olfactory receptor, family 4, subfamily C, member 12	1.08	1	0.98	0.84
KCNT2	KCNT2;KCa4.2;MGC119610;MGC119611;MGC119612;MGC119613;SLICK;SLO2.1	potassium channel, subfamily T, member 2	1.08	1.06	0.98	0.83
ARL13A	ARL13A;ARL13;dJ341D10.2	ADP-ribosylation factor-like 13A	1.07	1.08	0.98	0.78
SLC13A1	SLC13A1;NAS1;NaSi-1	solute carrier family 13 (sodium/sulfate symporters), member 1	1.07	1.14	0.98	0.85
OR7A10	OR7A10;BC85395_3;OR19-18	olfactory receptor, family 7, subfamily A, member 10	1.02	0.98	0.98	0.78
FIGLA	FIGLA;BHLHC8;FIGALPHA;POF6	folliculogenesis specific basic helix-loop-helix	1	0.93	0.98	0.78
AKR1D1	AKR1D1;3o5bred;CBAS2;SRD5B1	aldo-keto reductase family 1, member D1 (delta 4-3-ketosteroid-5-beta-reductase)	0.99	0.92	0.98	0.73
TAS2R46	TAS2R46;MGC134865;T2R46;T2R54	taste receptor, type 2, member 46	0.98	1.01	0.98	0.75
ACOT12	ACOT12;CACH-1;Cach;MGC105114;STARD15;THEAL	acyl-CoA thioesterase 12	0.96	0.88	0.98	0.69
OR4A15	OR4A15;OR11-118	olfactory receptor, family 4, subfamily A, member 15	0.94	0.89	0.98	0.68
C1orf150	C1orf150;FLJ41804;FLJ44728	chromosome 1 open reading frame 150	0.94	0.89	0.98	0.67
KLHL10	KLHL10;FLJ32662	kelch-like 10 (Drosophila)	0.94	0.9	0.98	0.68
C1orf141	C1orf141;MGC48454	chromosome 1 open reading frame 141	0.92	0.86	0.98	0.66
CALCRL	CALCRL;CGRPR;CRLR	calcitonin receptor-like	0.86	0.82	0.98	0.74
TEX15	TEX15;CT42;DKFZp434M2415	testis expressed 15	0.85	0.88	0.98	0.72
KRT36	KRT36;HA6;KRTHA6;hHa6	keratin 36	0.85	0.91	0.98	0.61
CYLC2	CYLC2;MGC129591	cylicin, basic protein of sperm head cytoskeleton 2	1.27	1.31	0.99	0.99
HGF	HGF;DFNB39;F-TCF;HGFB;HPTA;SF	hepatocyte growth factor (hepapoietin A; scatter factor)	1.15	1.05	0.99	0.79
TPTE	TPTE;CT44;PTEN2	transmembrane phosphatase with tensin homology	1.12	1.1	0.99	0.86
TDO2	TDO2;TDO;TPH2;TRPO	tryptophan 2,3-dioxygenase	1.12	1.07	0.99	0.93
TMPRSS12	TMPRSS12;MGC57341	transmembrane (C-terminal) protease, serine 12	1.07	1	0.99	0.79
USP26	USP26;MGC120066;MGC120067;MGC120068	ubiquitin specific peptidase 26	1.06	1.05	0.99	0.78
OR1E2	OR1E2;OR17-135;OR17-93;OR1E4;OR1E7;OST529	olfactory receptor, family 1, subfamily E, member 2	1	1.05	0.99	0.85
OR4C45	OR4C45	olfactory receptor, family 4, subfamily C, member 45	0.99	1	0.99	0.67
GRIA2	GRIA2;GLUR2;GLURB;GluA2;GluR-K2;HBGR2	glutamate receptor, ionotropic, AMPA 2	0.99	1.02	0.99	0.79
PMCH	PMCH;MCH	pro-melanin-concentrating hormone	0.98	0.96	0.99	0.72
EFCAB3	EFCAB3;FLJ25818;MGC126801;MGC126827	EF-hand calcium binding domain 3	0.98	1.04	0.99	0.73
LRRC63	LRRC63	leucine rich repeat containing 63	0.98	1.02	0.99	0.73
RNASE9	RNASE9;HEL128;h461	ribonuclease, RNase A family, 9 (non-active)	0.98	0.99	0.99	0.76
OR4F17	OR4F17;OR4F11P;OR4F18;OR4F19	olfactory receptor, family 4, subfamily F, member 17	0.95	0.97	0.99	0.69
CSTL1	CSTL1;CTES1;RCET11;dJ322G13.4	cystatin-like 1	0.94	0.98	0.99	0.69
TSHR	TSHR;CHNG1;LGR3;MGC75129;hTSHR-I	thyroid stimulating hormone receptor	0.94	0.91	0.99	0.82
OR4K13	OR4K13;OR14-27	olfactory receptor, family 4, subfamily K, member 13	0.94	0.97	0.99	0.68
TSHB	TSHB;TSH-BETA	thyroid stimulating hormone, beta	0.92	0.95	0.99	0.77
KCNH5	KCNH5;EAG2;H-EAG2;Kv10.2	potassium voltage-gated channel, subfamily H (eag-related), member 5	1.16	1.21	1	0.94
SPINK1	SPINK1;PCTT;PSTI;Spink3;TATI	serine peptidase inhibitor, Kazal type 1	1.06	1.15	1	0.86
MMP12	MMP12;HME;ME;MGC138506;MME;MMP-12	matrix metallopeptidase 12 (macrophage elastase)	1.05	1.04	1	0.78
HFM1	HFM1;FLJ36760;FLJ39011;MER3;MGC163397;RP11-539G11.1;SEC63D1;Si-11;Si-11-6;helicase	HFM1, ATP-dependent DNA helicase homolog (S. cerevisiae)	1.04	1.08	1	0.77
GYS2	GYS2	glycogen synthase 2 (liver)	1.04	0.97	1	0.77
RALYL	RALYL;HNRPCL3	RALY RNA binding protein-like	1.03	1.01	1	0.76
SPINK9	SPINK9;LEKTI2	serine peptidase inhibitor, Kazal type 9	1.02	1.06	1	0.79
RAET1E	RAET1E;LETAL;MGC125308;MGC125309;N2DL-4;NKG2DL4;RAET1E2;RL-4;ULBP4;bA350J20.7	retinoic acid early transcript 1E	1.01	0.98	1	0.79
SPAG17	SPAG17;DKFZp434B0610;FLJ34497;FLJ44343;FLJ44353;PF6;RP4-776P7.2	sperm associated antigen 17	1	1.03	1	0.77
PDCL2	PDCL2;GCPHLP	phosducin-like 2	0.97	1.06	1	0.78
GABRG1	GABRG1;DKFZp686H2042;MGC33838	gamma-aminobutyric acid (GABA) A receptor, gamma 1	0.96	1.01	1	0.79
LEF1	LEF1;DKFZp586H0919;FLJ46390;TCF10;TCF1ALPHA;TCF7L3	lymphoid enhancer-binding factor 1	0.93	0.94	1	0.75
DEFB131	DEFB131;DEFB-31	defensin, beta 131	0.92	0.98	1	0.69
DSC1	DSC1;CDHF1;DG2/DG3	desmocollin 1	0.92	0.96	1	0.77
DUSP21	DUSP21;LMWDSP21;MGC149878	dual specificity phosphatase 21	0.9	0.84	1	0.63
PCDH11Y	PCDH11Y;PCDH-PC;PCDH22;PCDHY	protocadherin 11 Y-linked	0.88	0.9	1	0.69
OR8K1	OR8K1;OR11-182;OR8N1P	olfactory receptor, family 8, subfamily K, member 1	0.86	0.78	1	0.62
TM7SF4	TM7SF4;DCSTAMP;FIND;MGC138223;MGC138225	transmembrane 7 superfamily member 4	0.86	0.9	1	0.62
PRH2	PRH2;DKFZp686B01256;DKFZp686F14256;DKFZp686I11251;DKFZp686J06255;DKFZp686L01253;DKFZp686L16244;DKFZp686M04243;DKFZp686N24248;PIF-S;PRH1;PRP-1/PRP-2;Pr;db-s;pa;pr1/Pr2	proline-rich protein HaeIII subfamily 2	0.85	0.9	1	0.63
CDH19	CDH19;CDH7;CDH7L2	cadherin 19, type 2	0.85	0.83	1	0.62
TRIML1	TRIML1;FLJ36180;MGC138638;MGC138639;RNF209	tripartite motif family-like 1	1.27	1.23	1.01	0.89
OR8H3	OR8H3;OR11-172	olfactory receptor, family 8, subfamily H, member 3	1.26	1.24	1.01	0.94
C4orf37	C4orf37;MGC46496	chromosome 4 open reading frame 37	1.19	1.18	1.01	0.95
OR13D1	OR13D1;OR9-15	olfactory receptor, family 13, subfamily D, member 1	1.17	1.23	1.01	0.92
RBMY1E	RBMY1E	RNA binding motif protein, Y-linked, family 1, member E	1.12	1.03	1.01	0.82
MOGAT1	MOGAT1;DGAT2L;DGAT2L1;MGAT1	monoacylglycerol O-acyltransferase 1	1.04	0.97	1.01	0.86
AGR3	AGR3;AG3;BCMP11;HAG3;PDIA18;hAG-3	anterior gradient homolog 3 (Xenopus laevis)	1	1.09	1.01	0.76
C12orf63	C12orf63;FLJ44112	chromosome 12 open reading frame 63	1	1.08	1.01	0.83
MMRN1	MMRN1;ECM;EMILIN4;GPIa*;MMRN	multimerin 1	1	1.05	1.01	0.7
DPP10	DPP10;DPL2;DPPY;DPRP3	dipeptidyl-peptidase 10 (non-functional)	1	0.99	1.01	0.74
DEFB107A	DEFB107A;BD-7;DEFB-7;DEFB107;DEFB107B	defensin, beta 107A	0.99	0.94	1.01	0.8
ANGPTL1	ANGPTL1;ANG3;ANGPT3;ARP1;AngY;KIAA0351;UNQ162;dJ595C2.2	angiopoietin-like 1	0.98	0.9	1.01	0.8
ADAM28	ADAM28;ADAM 28;ADAM23;MDC-L;MDC-Lm;MDC-Ls;MDCL;eMDC II;eMDCII	ADAM metallopeptidase domain 28	0.97	0.96	1.01	0.72
IL26	IL26;AK155;IL-26	interleukin 26	0.96	1.01	1.01	0.68
AKAP14	AKAP14;AKAP28	A kinase (PRKA) anchor protein 14	0.96	0.92	1.01	0.67
DEFB128	DEFB128;DEFB-28;DEFB28;hBD-28	defensin, beta 128	0.92	0.95	1.01	0.73
AADAC	AADAC;CES5A1;DAC	arylacetamide deacetylase (esterase)	0.9	0.88	1.01	0.76
C4orf7	C4orf7;FDC-SP;FDCSP;MGC71894	chromosome 4 open reading frame 7	0.9	0.88	1.01	0.74
HEMGN	HEMGN;EDAG;EDAG-1	hemogen	0.89	0.92	1.01	0.7
SCN2A	SCN2A;BFIC3;BFIS3;BFNIS;EIEE11;HBA;HBSCI;HBSCII;NAC2;Na(v)1.2;Nav1.2;SCN2A1;SCN2A2	sodium channel, voltage-gated, type II, alpha subunit	0.86	0.88	1.01	0.68
IFI44	IFI44;MTAP44;p44	interferon-induced protein 44	0.86	0.86	1.01	0.64
CLCA1	CLCA1;CACC;CACC1;CLCRG1;CaCC-1;FLJ95147;GOB5;hCLCA1;hCaCC-1	chloride channel accessory 1	1.25	1.32	1.02	1.03
CNTN4	CNTN4;AXCAM;BIG-2;MGC33615	contactin 4	1.19	1.18	1.02	0.93
OTC	OTC;MGC129967;MGC129968;MGC138856;OCTD	ornithine carbamoyltransferase	1.15	1.15	1.02	0.89
GPR98	GPR98;DKFZp761P0710;FEB4;KIAA0686;MASS1;USH2B;USH2C;VLGR1;VLGR1b	G protein-coupled receptor 98	1.12	1.1	1.02	0.99
SLC10A5	SLC10A5;P5	solute carrier family 10 (sodium/bile acid cotransporter family), member 5	1.04	1.03	1.02	0.86
FAM5C	FAM5C;BRINP3;DBCCR1L;DBCCR1L1;RP11-445K1.1	family with sequence similarity 5, member C	1.02	0.99	1.02	0.8
CMYA5	CMYA5;C5orf10;DKFZp451G182;DKFZp451G223;SPRYD2;TRIM76	cardiomyopathy associated 5	1.02	1.14	1.02	0.84
NPFFR2	NPFFR2;GPR74;HLWAR77;NPFF2;NPGPR	neuropeptide FF receptor 2	1.01	0.95	1.02	0.72
BCHE	BCHE;CHE1;E1	butyrylcholinesterase	0.98	0.94	1.02	0.82
TPH1	TPH1;MGC119994;TPRH;TRPH	tryptophan hydroxylase 1	0.96	0.97	1.02	0.72
LIN28B	LIN28B;CSDD2;FLJ16517	lin-28 homolog B (C. elegans)	0.95	0.92	1.02	0.74
S100A7A	S100A7A;NICE-2;S100A15;S100A7L1;S100A7f	S100 calcium binding protein A7A	0.94	0.94	1.02	0.71
OR6C4	OR6C4;OR12-10	olfactory receptor, family 6, subfamily C, member 4	0.9	0.89	1.02	0.7
SPATA16	SPATA16;NYD-SP12;SPGF6	spermatogenesis associated 16	0.9	0.91	1.02	0.66
RNF175	RNF175;FLJ34190	ring finger protein 175	0.86	0.87	1.02	0.78
STATH	STATH;STR	statherin	0.85	0.83	1.02	0.63
FGL1	FGL1;HFREP1;HP-041;LFIRE1;MGC12455	fibrinogen-like 1	1.09	1.14	1.03	0.79
GTSF1L	GTSF1L;C20orf65;FAM112A;MGC50820;dJ1028D15.4	gametocyte specific factor 1-like	1.06	1.04	1.03	0.84
ADAM30	ADAM30;svph4	ADAM metallopeptidase domain 30	1.05	1.08	1.03	0.81
OR8J1	OR8J1;OR11-183	olfactory receptor, family 8, subfamily J, member 1	1.04	1.12	1.03	0.83
SLC26A7	SLC26A7;MGC126268;SUT2	solute carrier family 26, member 7	1.04	1.07	1.03	0.83
CRH	CRH;CRF	corticotropin releasing hormone	1.03	1.15	1.03	0.86
OR4D11	OR4D11;OR4D11P	olfactory receptor, family 4, subfamily D, member 11	1.01	1.02	1.03	0.85
ADAM7	ADAM7;ADAM 7;ADAM-7;EAPI;GP-83;GP83	ADAM metallopeptidase domain 7	1	1.02	1.03	0.77
C1orf110	C1orf110;FLJ41579;MGC48998	chromosome 1 open reading frame 110	0.98	1.07	1.03	0.75
ST8SIA4	ST8SIA4;MGC34450;MGC61459;PST;PST1;SIAT8D;ST8SIA-IV	ST8 alpha-N-acetyl-neuraminide alpha-2,8-sialyltransferase 4	0.94	1.03	1.03	0.8
OR13C9	OR13C9;OR37L;OR9-13	olfactory receptor, family 13, subfamily C, member 9	0.94	0.99	1.03	0.68
GUCY2C	GUCY2C;GUC2C;STAR	guanylate cyclase 2C (heat stable enterotoxin receptor)	0.94	1.02	1.03	0.73
IL21	IL21;IL-21;Za11	interleukin 21	0.9	0.93	1.03	0.63
IFLTD1	IFLTD1;FLJ36004;Pas1c1	intermediate filament tail domain containing 1	0.88	0.8	1.03	0.63
OR52E4	OR52E4;OR11-55	olfactory receptor, family 52, subfamily E, member 4	0.88	0.93	1.03	0.7
ALLC	ALLC;ALC	allantoicase	0.88	0.84	1.03	0.67
TAAR2	TAAR2;GPR58	trace amine associated receptor 2	0.86	0.82	1.03	0.62
IFNA4	IFNA4;IFN-alpha4a;INFA4;MGC142200	interferon, alpha 4	1.14	1.25	1.04	0.88
CYSLTR1	CYSLTR1;CYSLT1;CYSLT1R;CYSLTR;HG55;HMTMF81;MGC46139	cysteinyl leukotriene receptor 1	1.09	1.04	1.04	0.79
NT5C1B	NT5C1B;AIRP;CN-IB;CN1B;MGC26640	5'-nucleotidase, cytosolic IB	1.04	1	1.04	0.76

TAC1	TAC1;Hs.2563;NK2;NKNA;NPK;TAC2	tachykinin, precursor 1	1.02	1.02	1.04	0.78
FAM154A	FAM154A;C9orf138;FLJ35283;MGC35182	family with sequence similarity 154, member A	0.99	1.03	1.04	0.72
GRIA4	GRIA4;GLUR4;GLUR4C;GLURD;GluA4	glutamate receptor, ionotrophic, AMPA 4	0.98	1.08	1.04	0.84
BCL2A1	BCL2A1;ACC-1;ACC-2;BCL2L5;BFL1;GRS;HBPA1	BCL2-related protein A1	0.97	0.99	1.04	0.73
P2RY13	P2RY13;GPCR1;GPR86;GPR94;P2Y13;SP174	purinergic receptor P2Y, G-protein coupled, 13	0.88	0.88	1.04	0.8
CNR1	CNR1;CANN6;CB-R;CB1;CB1A;CB1K5;CB1R;CNR	cannabinoid receptor 1 (brain)	0.88	0.89	1.04	0.8
OGN	OGN;DKFZp586P2421;OG;OIF;SLRR3A	osteoglycin	0.88	1	1.04	0.73
C6	C6	complement component 6	0.88	0.82	1.04	0.69
CPLX4	CPLX4;CPX-IV;CPXIV;DKFZp686A0185;DKFZp686O0683;FLJ41190;MGC125769;MGC125783;MGC125784	complexin 4	0.86	0.8	1.04	0.67
OR5AS1	OR5AS1;OR11-168	olfactory receptor, family 5, subfamily AS, member 1	1.2	1.21	1.05	0.92
TPTE	TPTE;CT44;PTEN2	transmembrane phosphatase with tensin homology	1.12	1.16	1.05	0.9
TXNDC3	TXNDC3;CILD6;NM23-H8;NME8;SPTRX2;sptrx-2	thioredoxin domain containing 3 (spermatozoa)	1.05	1.11	1.05	0.86
PSMA8	PSMA8;MGC26605;PSMA7L	proteasome (prosome, macropain) subunit, alpha type, 8	0.94	0.97	1.05	0.73
WBP2NL	WBP2NL;FLJ26145;MGC26816;PAWP	WBP2 N-terminal like	0.92	0.99	1.05	0.74
SPARCL1	SPARCL1;PIG33;SC1	SPARC-like 1 (hevin)	0.92	0.96	1.05	0.79
ASPA	ASPA;ACY2;ASP	aspartoacylase	0.91	0.92	1.05	0.65
TCEAL5	TCEAL5	transcription elongation factor A (SII)-like 5	0.9	0.98	1.05	0.73
FAM177B	FAM177B;FLJ43505	family with sequence similarity 177, member B	0.88	0.94	1.05	0.68
OR6C1	OR6C1;OST267	olfactory receptor, family 6, subfamily C, member 1	0.87	0.97	1.05	0.68
SYNPR	SYNPR;DKFZp686G0883;MGC26651;SPO	synaptoporin	0.85	0.87	1.05	0.66
CLCA2	CLCA2;CACC;CACC3;CLCRG2;CaCC-3;FLJ97885	chloride channel accessory 2	1.21	1.27	1.06	0.99
LOC650137	LOC650137	ENTREZ STATUS: REMAPPED TO GENE ID: 390539	1.2	1.2	1.06	1.01
OR8G5	OR8G5;OR11-298;OR8G5P;OR8G6	olfactory receptor, family 8, subfamily G, member 5	1.18	1.2	1.06	0.91
SATB1	SATB1	SATB homeobox 1	1.13	1.18	1.06	0.88
PLGLB2	PLGLB2;PLGLB1;PLGP1	plasminogen-like B2	0.98	1.02	1.06	0.77
C4orf26	C4orf26;FLJ23657;FLJ23937;FLJ53097;MGC150951	chromosome 4 open reading frame 26	0.96	0.98	1.06	0.75
TMPRSS11A	TMPRSS11A;ECRG1;MGC133183	transmembrane protease, serine 11A	0.94	0.9	1.06	0.68
TRPC7	TRPC7;TRP7	transient receptor potential cation channel, subfamily C, member 7	0.9	0.94	1.06	0.74
KLRC2	KLRC2;CD159c;MGC138244;NKG2-C;NKG2C	killer cell lectin-like receptor subfamily C, member 2	0.88	0.9	1.06	0.69
CSMD3	CSMD3;KIAA1894	CUB and Sushi multiple domains 3	0.88	0.98	1.06	0.73
OR10A6	OR10A6;OR11-96	olfactory receptor, family 10, subfamily A, member 6	0.86	0.94	1.06	0.71
FCGR2A	FCGR2A;CD32;CD32A;CDw32;FCG2;FCGR2;FCGR2A1;FcGR;IGFR2;MGC23887;MGC30032	Fc fragment of IgG, low affinity IIa, receptor (CD32)	1.09	1.28	1.07	0.89
ZNF208	ZNF208;PMIDP;ZNF95	zinc finger protein 208	1.02	1.12	1.07	0.75
PNLIPRP3	PNLIPRP3	pancreatic lipase-related protein 3	1.02	1.04	1.07	0.75
CR2	CR2;C3DR;CD21;CR;SLEB9	complement component (3d/Epstein Barr virus) receptor 2	1	1.05	1.07	0.79
CCDC54	CCDC54;FLJ25362;NYD-SP17	coiled-coil domain containing 54	0.97	0.92	1.07	0.72
TNFSF18	TNFSF18;AITRL;GITRL;MGC138237;TL6;hGITRL	tumor necrosis factor (ligand) superfamily, member 18	0.96	1.06	1.07	0.8
ARHGAP15	ARHGAP15;BM046	Rho GTPase activating protein 15	0.92	0.95	1.07	0.75
DNASE1L3	DNASE1L3;DHP2;DNAS1L3;LSD	deoxyribonuclease I-like 3	0.92	0.95	1.07	0.74
UGT3A1	UGT3A1;FLJ26528;FLJ34658	UDP glycosyltransferase 3 family, polypeptide A1	0.89	0.89	1.07	0.71
OR5H15	OR5H15	olfactory receptor, family 5, subfamily H, member 15	0.88	0.92	1.07	0.74
CLEC2B	CLEC2B;AICL;CLECSF2;HP10085;IFNRG1	C-type lectin domain family 2, member B	0.88	0.94	1.07	0.68
OR13C3	OR13C3;OR37G;OR9-8	olfactory receptor, family 13, subfamily C, member 3	0.86	0.9	1.07	0.68
OR1L4	OR1L4;OR1L5;OR9-29;OR9-E;OST046	olfactory receptor, family 1, subfamily L, member 4	0.86	0.9	1.07	0.67
LRRC39	LRRC39;DKFZp313O1122;MGC14816	leucine rich repeat containing 39	0.85	0.92	1.07	0.64
SPO11	SPO11;CT35;MGC39953	SPO11 meiotic protein covalently bound to DSB homolog (S. cerevisiae)	1.27	1.4	1.08	1.11
AMY1A	AMY1A;AMY1;AMY1B;AMY1C	amylase, alpha 1A (salivary)	1.27	1.34	1.08	1.04
GPR22	GPR22;MGC129847	G protein-coupled receptor 22	1.13	1.14	1.08	0.86
CYP4F8	CYP4F8;CPF8;CYPIVF8	cytochrome P450, family 4, subfamily F, polypeptide 8	1.09	1.12	1.08	0.81
UGT2B15	UGT2B15;HLUG4;UDPGT 2B8;UDPGT2B15;UDPGTH3;UGT2B8	UDP glucuronosyltransferase 2 family, polypeptide B15	1.08	1.25	1.08	0.94
KYNU	KYNU	kynureninase	1.02	1.01	1.08	0.76
MTTP	MTTP;ABL;MGC149819;MGC149820;MTP	microsomal triglyceride transfer protein	1	1.09	1.08	0.87
SPIC	SPIC;MGC40611;SPI-C	Spi-C transcription factor (Spi-1/PU.1 related)	0.99	1.04	1.08	0.77
OR6M1	OR6M1;OR11-271	olfactory receptor, family 6, subfamily M, member 1	0.93	1.04	1.08	0.79
FAM9B	FAM9B;FLJ40182	family with sequence similarity 9, member B	0.92	1.08	1.08	0.79
OR13C4	OR13C4;HSHTPCRX17;HTPCRX17;OR2K1;OR37F;OR9-7	olfactory receptor, family 13, subfamily C, member 4	0.91	0.96	1.08	0.72
PLEKHG7	PLEKHG7;FLJ46688	pleckstrin homology domain containing, family G (with RhoGef domain) member 7	0.9	0.99	1.08	0.67
C1orf101	C1orf101;MGC33370;RP11-523K4.1	chromosome 1 open reading frame 101	0.9	0.86	1.08	0.67
FSIP2	FSIP2	ENTREZ STATUS: REMAPPED TO GENE ID: 401024	0.89	1	1.08	0.67
MS4A5	MS4A5;CD20-L2;CD20L2;MGC34184;TETM4	membrane-spanning 4-domains, subfamily A, member 5	0.87	0.97	1.08	0.73
VIP	VIP;MGC13587;PHM27	vasoactive intestinal peptide	0.86	0.96	1.08	0.72
TRPC6	TRPC6;FLJ11098;FLJ14863;FSGS2;TRP6	transient receptor potential cation channel, subfamily C, member 6	0.86	0.99	1.08	0.77
C6orf58	C6orf58;MGC72003	chromosome 6 open reading frame 58	1.02	1.12	1.09	0.78
C15orf33	C15orf33;FLJ32800;MGC57432	chromosome 15 open reading frame 33	1.02	1.05	1.09	0.85
SLCO6A1	SLCO6A1;CT48;GST;MGC26949;OATP6A1;OATPY	solute carrier organic anion transporter family, member 6A1	1.01	1.02	1.09	0.81
CTXN3	CTXN3;KABE	cortexin 3	0.98	1.17	1.09	0.85
CXCR6	CXCR6;BONZO;CD186;STRL33;TYMSTR	chemokine (C-X-C motif) receptor 6	0.98	1	1.09	0.75
RXFP1	RXFP1;LGR7;LGR7.1;LGR7.10;LGR7.2;MGC138347;MGC142177;RXFPR1	relaxin/insulin-like family peptide receptor 1	0.97	1.02	1.09	0.93
SERPINB4	SERPINB4;LEUPIN;PI11;SCCA-2;SCCA1;SCCA2	serpin peptidase inhibitor, clade B (ovalbumin), member 4	0.94	1	1.09	0.78
MMP8	MMP8;CLG1;HNC;MMP-8;PMNL-CL	matrix metallopeptidase 8 (neutrophil collagenase)	0.94	1.01	1.09	0.76
GZMA	GZMA;CTLA3;HFSP	granzyme A (granzyme 1, cytotoxic T-lymphocyte-associated serine esterase 3)	0.9	0.98	1.09	0.73
SPATA1	SPATA1	ENTREZ STATUS: REMAPPED TO GENE ID: 100505741	0.88	0.99	1.09	0.66
WDR72	WDR72;AI2A3;FLJ38736;MGC126663;MGC126665	WD repeat domain 72	0.86	0.94	1.09	0.68
ZNF681	ZNF681;FLJ31526	zinc finger protein 681	0.86	0.9	1.09	0.73
TBPL2	TBPL2;TBP2;TRF3	TATA box binding protein like 2	0.85	0.94	1.09	0.65
RFPL4B	RFPL4B;RNF211	ret finger protein-like 4B	1.22	1.29	1.1	0.93
PTPN22	PTPN22;LYP;LYP1;LYP2;PEP;PTPN8	protein tyrosine phosphatase, non-receptor type 22 (lymphoid)	1.1	1.12	1.1	0.84
OR5K3	OR5K3	olfactory receptor, family 5, subfamily K, member 3	1.05	1.2	1.1	0.82
FAM9A	FAM9A;MGC138227;MGC142009	family with sequence similarity 9, member A	1.02	1.14	1.1	0.79
ROPN1B	ROPN1B	rhophilin associated tail protein 1B	1.02	1.17	1.1	0.88
AGXT2L1	AGXT2L1	alanine-glyoxylate aminotransferase 2-like 1	1	1.12	1.1	0.89
SLC5A8	SLC5A8;AIT;MGC125354;SMCT;SMCT1	solute carrier family 5 (iodide transporter), member 8	0.99	0.98	1.1	0.81
INSL4	INSL4;EPIL;PLACENTIN	insulin-like 4 (placenta)	0.99	1.11	1.1	0.8
ST8SIA6	ST8SIA6;SIA8F;SIAT8F;ST8SIA-VI	ST8 alpha-N-acetyl-neuraminide alpha-2,8-sialyltransferase 6	0.96	1.03	1.1	0.73
MSTN	MSTN;GDF8;MSLHP	myostatin	0.91	0.98	1.1	0.76
TRIM48	TRIM48;MGC4827;RNF101	tripartite motif containing 48	0.91	0.99	1.1	0.88
NME5	NME5;NM23-H5;NM23H5;RSPH23	non-metastatic cells 5, protein expressed in (nucleoside-diphosphate kinase)	0.88	0.96	1.1	0.8
SH2D1A	SH2D1A;DSHP;EBVS;FLJ18687;FLJ92177;IMD5;LYP;MTCP1;SAP;SAP/SH2D1A;XLP;XLPD	SH2 domain containing 1A	0.88	1	1.1	0.76
OR6C74	OR6C74	olfactory receptor, family 6, subfamily C, member 74	0.86	0.98	1.1	0.65
OR2T4	OR2T4;OR1-60;OR2T4Q	olfactory receptor, family 2, subfamily T, member 4	0.86	0.98	1.1	0.67
GPR115	GPR115;FLJ38076;PGR18	G protein-coupled receptor 115	0.85	0.87	1.1	0.66
HESX1	HESX1;ANF;CPHD5;MGC138294;RPX	HESX homeobox 1	0.85	0.98	1.1	0.76
OR8D4	OR8D4;OR11-275	olfactory receptor, family 8, subfamily D, member 4	1.24	1.34	1.11	1.01
GRM3	GRM3;GLUR3;GPRC1C;MGLUR3;mGlu3	glutamate receptor, metabotropic 3	1.12	1.15	1.11	0.87
PPEF1	PPEF1;PP7;PPEF;PPP7C;PPP7CA	protein phosphatase, EF-hand calcium binding domain 1	1.1	1.22	1.11	0.95
OR10X1	OR10X1;OR1-13;OR1-14;OR10X1P	olfactory receptor, family 10, subfamily X, member 1	0.99	1.06	1.11	0.83
ZNF502	ZNF502;FLJ12515;FLJ14855	zinc finger protein 502	0.96	0.96	1.11	0.8
CMKLR1	CMKLR1;CHEMERINR;ChemR23;DEZ;MGC126105;MGC126106	chemokine-like receptor 1	0.93	0.96	1.11	0.72
IFNA14	IFNA14;IFN-alphaH;LEIF2H;MGC125756;MGC125757	interferon, alpha 14	0.92	0.99	1.11	0.75
CLDN8	CLDN8	claudin 8	0.9	0.98	1.11	0.82
C9orf11	C9orf11;AFAF;equatorin	chromosome 9 open reading frame 11	0.88	0.92	1.11	0.78
OR11H1	OR11H1;OR22-1	olfactory receptor, family 11, subfamily H, member 1	0.88	0.95	1.11	0.79
KLHL32	KLHL32;BKLHD5;KIAA1900;MGC51280;MGC87753;RP1-39B17.1;UG0030H05;dJ21F7.1	kelch-like 32 (Drosophila)	0.86	0.92	1.11	0.71
OR8J3	OR8J3;OR11-173	olfactory receptor, family 8, subfamily J, member 3	1.06	1.15	1.12	0.9
GJB7	GJB7;CX25;MGC71746;bA136M9.1;connexin25	gap junction protein, beta 7, 25kDa	0.91	0.99	1.12	0.78
FAM19A1	FAM19A1;TAFA-1;TAFA1	family with sequence similarity 19 (chemokine (C-C motif)-like), member A1	0.9	1.05	1.12	0.78
CFHR3	CFHR3;CFHL3;DOWN16;FHR-3;FHR3;HLF4	complement factor H-related 3	0.89	1.03	1.12	0.77
TNMD	TNMD;BRICD4;CHM1L;TEM	tenomodulin	0.89	0.95	1.12	0.69
CCDC141	CCDC141;CAMDI;DKFZp686G197;FLJ26337;FLJ39502;MGC134803	coiled-coil domain containing 141	0.89	1.04	1.12	0.69
ASZ1	ASZ1;ALP1;ANKL1;C7orf7;CT1.19;GASZ;MGC26634;Orf3	ankyrin repeat, SAM and basic leucine zipper domain containing 1	0.88	0.98	1.12	0.71
RGS13	RGS13;MGC17173	regulator of G-protein signaling 13	0.88	0.94	1.12	0.74
LRRIQ4	LRRIQ4;LRRC64	leucine-rich repeats and IQ motif containing 4	0.86	0.92	1.12	0.7
SPP2	SPP2;SPP-24;SPP24	secreted phosphoprotein 2, 24kDa	0.85	0.92	1.12	0.66
GK2	GK2;GKP2;GKTA	glycerol kinase 2	1.19	1.3	1.13	1.18
C12orf36	C12orf36;FLJ33810;FLJ97127;MGC120140	chromosome 12 open reading frame 36	1.04	1.17	1.13	0.85
SPANXN5	SPANXN5;CT11.10;SPANX-N5	SPANX family, member N5	1.04	1.04	1.13	0.81
C13orf30	C13orf30;FLJ40919;MGC138442	chromosome 13 open reading frame 30	0.95	1.02	1.13	0.82
MAGEB18	MAGEB18;MGC33889	melanoma antigen family B, 18	0.95	1.14	1.13	0.8
XKR3	XKR3;MGC57211;XRG3;XTES	XK, Kell blood group complex subunit-related family, member 3	0.92	1.12	1.13	0.86
ROPN1	ROPN1;CT91;DKFZp434B1222;ODF6;RHPNAP1;ROPN1A;ropporin	rhophilin associated tail protein 1	0.9	1.02	1.13	0.71
LRRC2	LRRC2	leucine rich repeat containing 2	0.88	0.97	1.13	0.78
C3orf30	C3orf30;FLJ32859	chromosome 3 open reading frame 30	0.87	1.04	1.13	0.73
OR8B2	OR8B2;OR11-309;OR11-310	olfactory receptor, family 8, subfamily B, member 2	0.87	0.88	1.13	0.73
EIF1AY	EIF1AY	eukaryotic translation initiation factor 1A, Y-linked	0.86	0.92	1.13	0.73
LY86	LY86;MD-1;MMD-1;dJ80N2.1	lymphocyte antigen 86	0.85	0.87	1.13	0.69
MEIG1	MEIG1;bA2K17.3	meiosis expressed gene 1 homolog (mouse)	1.03	1.25	1.14	0.88
CPB2	CPB2;CPU;PCPB;TAFI	carboxypeptidase B2 (plasma)	1.02	1.15	1.14	0.86
OR4K17	OR4K17;OR14-29	olfactory receptor, family 4, subfamily K, member 17	0.96	1.1	1.14	0.75
OR5B3	OR5B3;OR11-239;OR5B13;OST129	olfactory receptor, family 5, subfamily B, member 3	0.92	1.09	1.14	0.83

ZCCHC16	ZCCHC16;FLJ46608;Mar4;Mart4	zinc finger, CCHC domain containing 16	0.92	1	1.14	0.76
LIPI	LIPI;CT17;LPDL	lipase, member I	0.9	0.96	1.14	0.76
OR8H2	OR8H2;OR11-171	olfactory receptor, family 8, subfamily H, member 2	0.89	1.04	1.14	0.7
C4BPA	C4BPA;C4BP;PRP	complement component 4 binding protein, alpha	0.88	1.05	1.14	0.78
CLEC12B	CLEC12B;UNQ5782	C-type lectin domain family 12, member B	0.88	0.95	1.14	0.77
OR2A25	OR2A25;OR2A24P;OR2A25P;OR2A27	olfactory receptor, family 2, subfamily A, member 25	0.87	0.96	1.14	0.76
HRH4	HRH4;AXOR35;BG26;GPCR105;GPRv53;H4;H4R;HH4R;MGC133027	histamine receptor H4	0.86	0.96	1.14	0.86
MMP26	MMP26;MGC126590;MGC126592	matrix metallopeptidase 26	1.03	1.08	1.15	0.82
FAM55B	FAM55B;FLJ25224	family with sequence similarity 55, member B	0.98	1.21	1.15	0.84
PIWIL1	PIWIL1;HIWI;MIWI;PIWI	piwi-like 1 (Drosophila)	0.94	1.06	1.15	0.75
CALCR	CALCR;CRT;CT-R;CTR;CTR1	calcitonin receptor	0.92	0.99	1.15	0.74
RRH	RRH	retinal pigment epithelium-derived rhodopsin homolog	0.92	1.02	1.15	0.92
ZCWPW2	ZCWPW2;ZCW2	zinc finger, CW type with PWWP domain 2	0.91	1.12	1.15	0.86
OLFML1	OLFML1;UNQ564	olfactomedin-like 1	0.91	1.07	1.15	0.8
OR5H1	OR5H1;HSHTPCRX14;HTPCRX14	olfactory receptor, family 5, subfamily H, member 1	0.9	1.07	1.15	0.8
SMPX	SMPX;DFNX4	small muscle protein, X-linked	0.9	1.08	1.15	0.72
OR2K2	OR2K2;HSHTPCRH06;HTPCRH06;MGC133152;OR2AN1P;OR2AR1P	olfactory receptor, family 2, subfamily K, member 2	0.88	1.03	1.15	0.78
RNASE12	RNASE12	ribonuclease, RNase A family, 12 (non-active)	0.86	0.96	1.15	0.75
ZNF560	ZNF560;FLJ31986;MGC119490;MGC119493	zinc finger protein 560	1.05	1.09	1.16	0.87
HAO1	HAO1;GOX;GOX1;HAOX1;MGC142225;MGC142227	hydroxyacid oxidase (glycolate oxidase) 1	1	1.07	1.16	0.95
SLC26A3	SLC26A3;CLD;DRA	solute carrier family 26, member 3	0.96	1.08	1.16	0.76
IL5	IL5;EDF;IL-5;TRF	interleukin 5 (colony-stimulating factor, eosinophil)	0.95	1.12	1.16	0.89
ADAMTS20	ADAMTS20;ADAM-TS20;ADAMTS-20;GON-1	ADAM metallopeptidase with thrombospondin type 1 motif, 20	0.9	1.02	1.16	0.77
GPR34	GPR34	G protein-coupled receptor 34	0.86	0.96	1.16	0.87
GZMK	GZMK;TRYP2	granzyme K (granzyme 3; tryptase II)	0.85	0.98	1.16	0.73
SPATA4	SPATA4;MGC33432;SPEF1B;TSARG2	spermatogenesis associated 4	1.1	1.25	1.17	0.98
OR4F15	OR4F15	olfactory receptor, family 4, subfamily F, member 15	0.99	1.21	1.17	0.86
GADL1	GADL1;MGC138191	glutamate decarboxylase-like 1	0.98	1.21	1.17	0.8
VN1R5	VN1R5;V1RL5	vomeronasal 1 receptor 5 (gene/pseudogene)	0.96	1.1	1.17	0.99
PLCZ1	PLCZ1;MGC149685;NYD-SP27;PLCzeta	phospholipase C, zeta 1	0.88	1	1.17	0.78
LPHN3	LPHN3;CIRL3;LEC3	latrophilin 3	1.28	1.47	1.18	1.32
DEFB106A	DEFB106A;BD-6;DEFB-6;DEFB106;MGC118938;MGC118939;MGC118940;MGC118941;MGC133011;MGC133012	defensin, beta 106A	1.07	1.13	1.18	0.88
GABRA2	GABRA2;FLJ97076	gamma-aminobutyric acid (GABA) A receptor, alpha 2	1.03	1.21	1.18	0.95
C8orf22	C8orf22	chromosome 8 open reading frame 22	1.02	1.1	1.18	0.88
FRZB	FRZB;FRE;FRITZ;FRP-3;FRZB-1;FRZB-PEN;FRZB1;FZRB;OS1;SFRP3;SRFP3;hFIZ	frizzled-related protein	0.9	1.06	1.18	0.72
OR2B2	OR2B2;OR2B2Q;OR2B9;OR6-1;dJ193B12.4;hs6M1-10	olfactory receptor, family 2, subfamily B, member 2	0.9	1.02	1.18	0.83
SPANXN1	SPANXN1;CT11.6	SPANX family, member N1	0.89	1.08	1.19	0.81
KMO	KMO;dJ317G22.1	kynurenine 3-monooxygenase (kynurenine 3-hydroxylase)	0.89	1.04	1.19	0.78
EFCAB1	EFCAB1;FLJ11767	EF-hand calcium binding domain 1	0.85	1.08	1.19	0.84
RNF148	RNF148;MGC35222	ring finger protein 148	0.97	1.12	1.2	0.81
SAMSN1	SAMSN1;HACS1;NASH1;SASH2;SH3D6B;SLy2	SAM domain, SH3 domain and nuclear localization signals 1	0.88	1.02	1.2	0.82
NKAIN2	NKAIN2;FAM77B;MGC41924;NKAIP2;TCBA;TCBA1	Na+/K+ transporting ATPase interacting 2	0.88	0.94	1.2	0.78
OR12D2	OR12D2;DJ994E9.8;HS6M1-20;MGC126791;MGC126795	olfactory receptor, family 12, subfamily D, member 2	1.07	1.17	1.21	0.93
OPN1LW	OPN1LW;CBBM;CBP;COD5;RCP;ROP	opsin 1 (cone pigments), long-wave-sensitive	1.06	1.24	1.21	1.12
ABCA10	ABCA10;EST698739	ATP-binding cassette, sub-family A (ABC1), member 10	1.03	1.25	1.21	0.84
ALB	ALB;DKFZp779N1935;PRO0883;PRO0903;PRO1341	albumin	0.98	1.16	1.21	0.82
CLRN3	CLRN3;DKFZp686F11218;MGC32871;TMEM12;USH3AL1	clarin 3	0.94	1.17	1.21	0.84
KLKB1	KLKB1;KLK3;PPK	kallikrein B, plasma (Fletcher factor) 1	0.94	1.04	1.21	0.78
SLC25A31	SLC25A31;AAC4;ANT4;DKFZp434N1235;SFEC35kDa	solute carrier family 25 (mitochondrial carrier; adenine nucleotide translocator), member 31	0.91	1.06	1.21	0.76
OR51B6	OR51B6;HOR5'Beta6	olfactory receptor, family 51, subfamily B, member 6	0.89	1.02	1.21	0.74
GABRA3	GABRA3;MGC33793	gamma-aminobutyric acid (GABA) A receptor, alpha 3	0.86	1.03	1.21	0.8
OR51A7	OR51A7;OR11-27	olfactory receptor, family 51, subfamily A, member 7	0.97	1.15	1.22	0.87
PPP1R3A	PPP1R3A;GM;PP1G;PPP1R3	protein phosphatase 1, regulatory subunit 3A	0.9	1.03	1.22	0.77
CYP7A1	CYP7A1;CP7A;CYP7;CYPVII;MGC126826;MGC138389	cytochrome P450, family 7, subfamily A, polypeptide 1	0.87	1.06	1.22	0.78
SLC17A6	SLC17A6;DNPI;VGLUT2	solute carrier family 17 (sodium-dependent inorganic phosphate cotransporter), member 6	0.87	1.03	1.22	0.76
ZNF596	ZNF596;FLJ36123	zinc finger protein 596	0.87	1.07	1.23	0.74
OR6S1	OR6S1;OR14-37;OR6S1Q	olfactory receptor, family 6, subfamily S, member 1	0.89	1.07	1.24	0.87
OR51A2	OR51A2	olfactory receptor, family 51, subfamily A, member 2	0.84	0.66	0.81	0.55
CYSLTR2	CYSLTR2;CYSLT2;CYSLT2R;HG57;HPN321;KPG_011;hGPCR21	cysteinyl leukotriene receptor 2	0.8	0.64	0.81	0.57
LOC651503	LOC651503	ENTREZ STATUS: REMAPPED TO GENE ID: 26695	0.81	0.68	0.82	0.46
GDF3	GDF3;KFS3;MCOP7;MCOPCB6	growth differentiation factor 3	0.61	0.52	0.82	0.39
ARG1	ARG1	arginase, liver	0.84	0.74	0.83	0.56
SPINT4	SPINT4;C20orf137;SPINT3;dJ601O1.1	serine peptidase inhibitor, Kunitz type 4	0.8	0.62	0.83	0.51
UGT2B28	UGT2B28	UDP glucuronosyltransferase 2 family, polypeptide B28	0.78	0.7	0.83	0.52
SLCO1A2	SLCO1A2;OATP;OATP-A;OATP1A2;SLC21A3	solute carrier organic anion transporter family, member 1A2	0.64	0.59	0.83	0.41
PKD2L2	PKD2L2;TRPP5	polycystic kidney disease 2-like 2	0.78	0.68	0.84	0.53
OR4K15	OR4K15;OR14-20;OR4K15Q	olfactory receptor, family 4, subfamily K, member 15	0.78	0.69	0.84	0.48
CAPS2	CAPS2;FLJ34520	calcyphosine 2	0.76	0.64	0.84	0.44
SPINK7	SPINK7;ECG2;ECRG2;MGC133105;MGC133106	serine peptidase inhibitor, Kazal type 7 (putative)	0.68	0.56	0.84	0.41
NLGN1	NLGN1;KIAA1070;MGC45115;NL1	neuroligin 1	0.68	0.56	0.84	0.42
CCL4	CCL4;ACT2;AT744.1;G-26;HC21;LAG-1;LAG1;MGC104418;MGC126025;MGC126026;MIP-1-beta;MIP1B;MIP1B1;SCYA2;SCYA4	chemokine (C-C motif) ligand 4	0.63	0.55	0.84	0.38
NLRP14	NLRP14;CLR11.2;GC-LRR;NALP14;NOD5;PAN8	NLR family, pyrin domain containing 14	0.82	0.76	0.85	0.57
FCER1A	FCER1A;FCE1A;FcERI	Fc fragment of IgE, high affinity I, receptor for; alpha polypeptide	0.8	0.67	0.85	0.52
CD86	CD86;B7-2;B7.2;B70;CD28LG2;LAB72;MGC34413	CD86 molecule	0.8	0.66	0.85	0.52
OR2A12	OR2A12;OR2A12P;OR2A16P	olfactory receptor, family 2, subfamily A, member 12	0.84	0.66	0.86	0.54
CLRN1	CLRN1;RP61;USH3;USH3A	clarin 1	0.83	0.77	0.86	0.52
MYO3A	MYO3A;DFNB30	myosin IIIA	0.79	0.74	0.86	0.52
ELAVL2	ELAVL2;HEL-N1;HELN1;HUB	ELAV (embryonic lethal, abnormal vision, Drosophila)-like 2 (Hu antigen B)	0.79	0.7	0.86	0.47
IL33	IL33;C9orf26;DKFZp586H0523;DVS27;IL1F11;NF-HEV;NFEHEV;RP11-575C20.2	interleukin 33	0.76	0.7	0.86	0.47
FAM46D	FAM46D;CT1.26;CT112;MGC26999	family with sequence similarity 46, member D	0.74	0.64	0.86	0.46
OR5P2	OR5P2;JCG3;JCG4;MGC126759;MGC142117	olfactory receptor, family 5, subfamily P, member 2	0.68	0.6	0.86	0.45
ZCCHC5	ZCCHC5;FLJ38865;Mar3;Mart3;ZHC5	zinc finger, CCHC domain containing 5	0.66	0.59	0.86	0.45
RBMY1A1	RBMY1A1;MGC181956;RBM;RBM1;RBM2;RBMY;RBMY1C;YRRM1;YRRM2	RNA binding motif protein, Y-linked, family 1, member A1	0.62	0.55	0.86	0.42
PRAMEF1	PRAMEF1	PRAME family member 1	0.75	0.67	0.87	0.5
P2RY14	P2RY14;GPR105;KIAA0001;P2Y14	purinergic receptor P2Y, G-protein coupled, 14	0.75	0.61	0.87	0.46
ZNF471	ZNF471;ERP1;KIAA1396;MGC150657;MGC150658;Z1971	zinc finger protein 471	0.63	0.6	0.87	0.44
OR2T11	OR2T11;OR2T11Q	olfactory receptor, family 2, subfamily T, member 11	0.83	0.76	0.88	0.54
AKT3	AKT3;DKFZp434N0250;PKB-GAMMA;PKBG;PRKBG;RAC-PK-gamma;RAC-gamma;STK-2	v-akt murine thymoma viral oncogene homolog 3 (protein kinase B, gamma)	0.8	0.7	0.88	0.58
PIK3C2G	PIK3C2G;MGC163149;PI3K-C2GAMMA	phosphoinositide-3-kinase, class 2, gamma polypeptide	0.76	0.67	0.88	0.55
EVI2B	EVI2B;CD361;D17S376;EVDB	ecotropic viral integration site 2B	0.71	0.64	0.88	0.46
ZP2	ZP2;ZPA;Zp-2	zona pellucida glycoprotein 2 (sperm receptor)	0.66	0.61	0.88	0.43
SULT1C4	SULT1C4;MGC149521;MGC34422;SULT1C;SULT1C2	sulfotransferase family, cytosolic, 1C, member 4	0.84	0.74	0.89	0.58
JAKMIP2	JAKMIP2;JAMIP2;KIAA0555;NECC1	janus kinase and microtubule interacting protein 2	0.82	0.7	0.89	0.53
POU1F1	POU1F1;CPHD1;GHF-1;PIT1;POU1F1a;Pit-1	POU class 1 homeobox 1	0.82	0.74	0.89	0.59
CNBD1	CNBD1;FLJ35802	cyclic nucleotide binding domain containing 1	0.76	0.74	0.89	0.49
OR56B4	OR56B4;OR11-67	olfactory receptor, family 56, subfamily B, member 4	0.73	0.58	0.89	0.49
LGALS13	LGALS13;GAL13;PLAC8;PP13	lectin, galactoside-binding, soluble, 13	0.66	0.57	0.89	0.44
AFM	AFM;ALB2;ALBA;ALF;MGC125338;MGC125339	afamin	0.8	0.73	0.9	0.51
PCDHB4	PCDHB4;PCDH-BETA4	protocadherin beta 4	0.79	0.7	0.9	0.52
FBXL21	FBXL21;FBL3B;FBXL3B;FBXL3P;Fbl21;MGC120237	F-box and leucine-rich repeat protein 21 (gene/pseudogene)	0.78	0.66	0.9	0.48
WDR49	WDR49;FLJ33620	WD repeat domain 49	0.76	0.65	0.9	0.53
HTR3B	HTR3B;5-HT3B	5-hydroxytryptamine (serotonin) receptor 3B	0.65	0.62	0.9	0.48
CPA2	CPA2	carboxypeptidase A2 (pancreatic)	0.83	0.7	0.91	0.52
TAS2R10	TAS2R10;MGC126811;MGC126813;T2R10;TRB2	taste receptor, type 2, member 10	0.82	0.78	0.91	0.52
SLCO4C1	SLCO4C1;OATP-H;OATP-M1;OATP4C1;OATPX;PRO2176;SLC21A20	solute carrier organic anion transporter family, member 4C1	0.81	0.68	0.91	0.54
UTY	UTY;DKFZp686L12190;UTY1	ubiquitously transcribed tetratricopeptide repeat gene, Y-linked	0.76	0.71	0.91	0.54
NRXN1	NRXN1;DKFZp313P2036;FLJ35941;Hs.22998;KIAA0578;PTHSL2	neurexin 1	0.75	0.75	0.91	0.55
SAGE1	SAGE1;CT14;SAGE	sarcoma antigen 1	0.68	0.64	0.91	0.49
OR5L2	OR5L2;HSHTPCRX16;HTPCRX16;OR11-153	olfactory receptor, family 5, subfamily L, member 2	0.66	0.54	0.91	0.45
OR10A2	OR10A2;OR10A2P;OR11-82;OR11-86;OST363	olfactory receptor, family 10, subfamily A, member 2	0.64	0.56	0.91	0.43
SLC26A8	SLC26A8;FLJ32714;TAT1	solute carrier family 26, member 8	0.62	0.62	0.91	0.46
SLC22A10	SLC22A10;OAT5;hOAT5	solute carrier family 22, member 10	0.83	0.73	0.92	0.59
SERPINI2	SERPINI2;MEPI;PANCPIN;PI14;TSA2004	serpin peptidase inhibitor, clade I (pancpin), member 2	0.82	0.7	0.92	0.56
PAGE2B	PAGE2B;CT16.5;GAGEE3;PAGE-2B	P antigen family, member 2B	0.81	0.81	0.92	0.54
TAS2R39	TAS2R39;T2R39;T2R57	taste receptor, type 2, member 39	0.76	0.72	0.92	0.55
OR4C11	OR4C11;OR11-136;OR4C11P	olfactory receptor, family 4, subfamily C, member 11	0.74	0.69	0.92	0.48
RNF17	RNF17;FLJ11045;Mmip-2;SPATA23;TDRD4	ring finger protein 17	0.72	0.66	0.92	0.59
L1TD1	L1TD1;ECAT11;FLJ10884;MGC133253;RP5-1155K23.3	LINE-1 type transposase domain containing 1	0.7	0.71	0.92	0.47
CLEC1A	CLEC1A;CLEC1;MGC34328	C-type lectin domain family 1, member A	0.68	0.62	0.92	0.46
SPEF2	SPEF2;CT122;FLJ23164;FLJ23577;FLJ25395;KIAA1770;KPL2;MGC102842	sperm flagellar 2	0.66	0.64	0.92	0.44
SUCNR1	SUCNR1;GPR91	succinate receptor 1	0.84	0.75	0.93	0.68
PXT1	PXT1;MGC129569;STEPP	peroxisomal, testis specific 1	0.74	0.69	0.93	0.48
NPY1R	NPY1R;NPY1-R;NPYR	neuropeptide Y receptor Y1	0.74	0.68	0.93	0.61
OR7A5	OR7A5;HTPCR2	olfactory receptor, family 7, subfamily A, member 5	0.68	0.69	0.93	0.51
TMPRSS11B	TMPRSS11B;DKFZp686L1818	transmembrane protease, serine 11B	0.82	0.86	0.94	0.59
OR5D13	OR5D13	olfactory receptor, family 5, subfamily D, member 13	0.82	0.8	0.94	0.55
HTN1	HTN1;HIS1	histatin 1	0.81	0.8	0.94	0.64
ABCB5	ABCB5;ABCB5alpha;ABCB5beta;EST422562	ATP-binding cassette, sub-family B (MDR/TAP), member 5	0.8	0.76	0.94	0.59
CLEC7A	CLEC7A;BGR;CANDF4;CLECSF12;DECTIN1	C-type lectin domain family 7, member A	0.78	0.73	0.94	0.51

DEFB136	DEFB136;DEFB137	defensin, beta 136	0.77	0.71	0.94	0.57
UNC13C	UNC13C;DKFZp547H074	unc-13 homolog C (C. elegans)	0.77	0.71	0.94	0.54
LIPK	LIPK;LIPL2;bA186O14.2	lipase, family member K	0.74	0.76	0.94	0.51
LCE1B	LCE1B;LEP2;SPRL2A	late cornified envelope 1B	0.7	0.64	0.94	0.54
CLEC6A	CLEC6A;CLEC4N;CLECSF10	C-type lectin domain family 6, member A	0.69	0.62	0.94	0.49
SV2B	SV2B;FLJ30413;FLJ42732;HsT19680;KIAA0735	synaptic vesicle glycoprotein 2B	0.68	0.62	0.94	0.54
GABRB1	GABRB1	gamma-aminobutyric acid (GABA) A receptor, beta 1	0.66	0.66	0.94	0.51
OR52N5	OR52N5;OR11-62;OR52N5Q	olfactory receptor, family 52, subfamily N, member 5	0.66	0.62	0.94	0.42
OR2J2	OR2J2;MGC119134;MGC119135;MGC119136;MGC119137;OR6-19;OR6-8;OR6.3.8;ORL684;dJ80I19.4;hs6M1-6	olfactory receptor, family 2, subfamily J, member 2	0.64	0.58	0.94	0.44
OLFM3	OLFM3;NOE3;NOELIN3;NOELIN3_V1;NOELIN3_V2;NOELIN3_V3;NOELIN3_V4;NOELIN3_V5;NOELIN3_V6;OPTIMEDIN	olfactomedin 3	0.64	0.67	0.94	0.49
LYG2	LYG2;LYGH;MGC119046;MGC119047;MGC119049	lysozyme G-like 2	0.78	0.71	0.95	0.56
RNASE6	RNASE6;RNS6;RNasek6	ribonuclease, RNase A family, k6	0.76	0.68	0.95	0.54
DIRC1	DIRC1	disrupted in renal carcinoma 1	0.75	0.74	0.95	0.5
BBOX1	BBOX1;BBH;BBOX;G-BBH;gamma-BBH	butyrobetaine (gamma), 2-oxoglutarate dioxygenase (gamma-butyrobetaine hydroxylase) 1	0.75	0.7	0.95	0.52
INSL5	INSL5;MGC126695;MGC126697;PRO182;UNQ156	insulin-like 5	0.72	0.72	0.95	0.58
OR4X2	OR4X2;OR11-105	olfactory receptor, family 4, subfamily X, member 2	0.72	0.73	0.95	0.59
CNTN5	CNTN5;HNB-2s;MGC163491;NB-2	contactin 5	0.7	0.65	0.95	0.51
TAS2R13	TAS2R13;T2R13;TRB3	taste receptor, type 2, member 13	0.84	0.73	0.96	0.58
OR6C75	OR6C75	olfactory receptor, family 6, subfamily C, member 75	0.84	0.83	0.96	0.63
C7	C7	complement component 7	0.82	0.77	0.96	0.58
DEFB125	DEFB125;DEFB-25;MGC57449	defensin, beta 125	0.82	0.8	0.96	0.61
IL1RL1	IL1RL1;DER4;FIT-1;IL33R;MGC32623;ST2;ST2L;ST2V;T1	interleukin 1 receptor-like 1	0.82	0.76	0.96	0.54
PAGE2	PAGE2;CT16.4;GAGEC2;GAGEE2;MGC62094;PAGE-2	P antigen family, member 2 (prostate associated)	0.78	0.73	0.96	0.54
OLAH	OLAH;AURA1;FLJ11106;MGC51852;SAST;THEDC1	oleoyl-ACP hydrolase	0.76	0.73	0.96	0.54
SPAG6	SPAG6;DKFZp434I153;MGC26276;Repro-SA-1;pf16	sperm associated antigen 6	0.74	0.7	0.96	0.56
PSG3	PSG3	pregnancy specific beta-1-glycoprotein 3	0.72	0.66	0.96	0.48
TDRD9	TDRD9;C14orf75;DKFZp434N0820;FLJ36164;HIG-1;MGC135025;NET54	tudor domain containing 9	0.7	0.62	0.96	0.53
RGS1	RGS1;1R20;BL34;IER1;IR20	regulator of G-protein signaling 1	0.65	0.64	0.96	0.43
OR7A17	OR7A17;BC85395_4;HTPCRX19;MGC126636	olfactory receptor, family 7, subfamily A, member 17	0.63	0.62	0.96	0.43
LIN7A	LIN7A;LIN-7A;LIN7;MALS-1;MGC148143;TIP-33;VELI1	lin-7 homolog A (C. elegans)	0.63	0.57	0.96	0.48
IGF1	IGF1;IGF-I;IGF1A;IGFI	insulin-like growth factor 1 (somatomedin C)	0.62	0.57	0.96	0.5
GCG	GCG;GLP1;GLP2;GRPP	glucagon	0.79	0.79	0.97	0.6
GLRA2	GLRA2;GLR	glycine receptor, alpha 2	0.77	0.75	0.97	0.58
C1orf146	C1orf146;RP11-163M2.4	chromosome 1 open reading frame 146	0.76	0.7	0.97	0.57
TRDN	TRDN;DKFZp779I2253;MGC88285;TDN;TRISK	triadin	0.76	0.67	0.97	0.56
CLDN17	CLDN17;MGC126552;MGC126554	claudin 17	0.74	0.75	0.97	0.57
ERMN	ERMN;JN;KIAA1189;ermin	ermin, ERM-like protein	0.7	0.74	0.97	0.54
OR8A1	OR8A1;OR11-318;OST025	olfactory receptor, family 8, subfamily A, member 1	0.65	0.66	0.97	0.49
CEACAM6	CEACAM6;CD66c;CEAL;NCA	carcinoembryonic antigen-related cell adhesion molecule 6 (non-specific cross reacting antigen)	0.63	0.62	0.97	0.48
CRISP1	CRISP1;AEGL1;ARP;CRISP-1;HSCRISP1D;HSCRISP1G;HUMARP	cysteine-rich secretory protein 1	0.84	0.85	0.98	0.64
TEX11	TEX11;TGC1;TSGA3	testis expressed 11	0.82	0.78	0.98	0.59
NTS	NTS;NMN-125;NN;NT;NT/N;NTS1	neurotensin	0.82	0.81	0.98	0.54
C15orf60	C15orf60;FLJ27520;FLJ36860;FLJ44083	chromosome 15 open reading frame 60	0.82	0.83	0.98	0.63
MSMB	MSMB;HPC13;IGBF;MSP;MSPB;PN44;PRPS;PSP;PSP-94;PSP57;PSP94	microseminoprotein, beta-	0.8	0.79	0.98	0.53
TFEC	TFEC;TCFEC;TFE-C;TFEC-L;TFECL;bHLHe34;hTFEC-L	transcription factor EC	0.79	0.74	0.98	0.54
GIMAP2	GIMAP2;DKFZp586D0824;HIMAP2;IMAP2;MGC24275	GTPase, IMAP family member 2	0.78	0.8	0.98	0.54
GPR82	GPR82	G protein-coupled receptor 82	0.74	0.68	0.98	0.62
UGT2B11	UGT2B11;MGC129611;MGC129612	UDP glucuronosyltransferase 2 family, polypeptide B11	0.74	0.7	0.98	0.51
ADAD1	ADAD1;FLJ32741;Tenr	adenosine deaminase domain containing 1 (testis-specific)	0.72	0.67	0.98	0.53
ZBBX	ZBBX;FLJ23049	zinc finger, B-box domain containing	0.72	0.7	0.98	0.53
TNFAIP6	TNFAIP6;TSG-6;TSG6	tumor necrosis factor, alpha-induced protein 6	0.72	0.66	0.98	0.51
ARHGEF6	ARHGEF6;COOL2;Cool-2;KIAA0006;MRX46;PIXA;alpha-PIX;alphaPIX	Rac/Cdc42 guanine nucleotide exchange factor (GEF) 6	0.7	0.75	0.98	0.52
TPH2	TPH2;ADHD7;FLJ37295;MGC138871;MGC138872;NTPH	tryptophan hydroxylase 2	0.68	0.7	0.98	0.47
APOBEC4	APOBEC4;C1orf169;MGC26594	apolipoprotein B mRNA editing enzyme, catalytic polypeptide-like 4 (putative)	0.62	0.55	0.98	0.43
SLAMF1	SLAMF1;CD150;CDw150;SLAM	signaling lymphocytic activation molecule family member 1	0.84	0.85	0.99	0.68
OR9Q1	OR9Q1;MGC131737	olfactory receptor, family 9, subfamily Q, member 1	0.84	0.88	0.99	0.62
KLRF1	KLRF1;CLEC5C;MGC119907;MGC119908;MGC119909;NKp80	killer cell lectin-like receptor subfamily F, member 1	0.83	0.84	0.99	0.67
F9	F9;FIX;HEMB;MGC129641;MGC129642;P19;PTC	coagulation factor IX	0.81	0.8	0.99	0.59
SPATA9	SPATA9;FLJ35906;NYD-SP16	spermatogenesis associated 9	0.77	0.77	0.99	0.52
CYP2C8	CYP2C8;CPC8;CYPIIC8;MP-12/MP-20	cytochrome P450, family 2, subfamily C, polypeptide 8	0.76	0.75	0.99	0.56
ZNF804B	ZNF804B;FLJ32110	zinc finger protein 804B	0.76	0.78	0.99	0.58
LRRTM2	LRRTM2;FLJ76283;FLJ99831	leucine rich repeat transmembrane neuronal 2	0.76	0.76	0.99	0.53
MRO	MRO;B29;C18orf3;FLJ30140	maestro	0.7	0.72	0.99	0.55
CA5A	CA5A;CA5;CAV;CAVA	carbonic anhydrase VA, mitochondrial	0.69	0.73	0.99	0.48
OR2AK2	OR2AK2;OR1-47;OR2AK1P	olfactory receptor, family 2, subfamily AK, member 2	0.69	0.66	0.99	0.53
CXorf21	CXorf21;FLJ11577	chromosome X open reading frame 21	0.62	0.62	0.99	0.43
KCNIP4	KCNIP4;CALP;KCHIP4;MGC44947	Kv channel interacting protein 4	0.84	0.88	1	0.68
ASPN	ASPN;FLJ20129;OS3;PLAP-1;PLAP1;SLRR1C	asporin	0.81	0.81	1	0.56
ZNF345	ZNF345;FLJ36827;FLJ51247;FLJ96023;HZF10;MGC33794	zinc finger protein 345	0.8	0.88	1	0.64
OR11G2	OR11G2;OR14-34	olfactory receptor, family 11, subfamily G, member 2	0.78	0.78	1	0.53
LYVE1	LYVE1;CRSBP-1;HAR;LYVE-1;XLKD1	lymphatic vessel endothelial hyaluronan receptor 1	0.78	0.74	1	0.62
GPR112	GPR112;DKFZp781E1948;PGR17;RP1-299I16	G protein-coupled receptor 112	0.78	0.76	1	0.69
TAS2R7	TAS2R7;MGC142121;T2R7;TRB4	taste receptor, type 2, member 7	0.76	0.79	1	0.6
GPR65	GPR65;TDAG8;hTDAG8	G protein-coupled receptor 65	0.76	0.73	1	0.66
CRLF2	CRLF2;CRL2;CRLF2Y;TSLPR	cytokine receptor-like factor 2	0.76	0.72	1	0.57
OR52B6	OR52B6;OR11-47	olfactory receptor, family 52, subfamily B, member 6	0.74	0.72	1	0.6
HEPH	HEPH;CPL;KIAA0698	hephaestin	0.74	0.82	1	0.57
OR4D2	OR4D2;BC2009;OR17-24	olfactory receptor, family 4, subfamily D, member 2	0.73	0.7	1	0.54
SLITRK6	SLITRK6;MGC119595;MGC119596;MGC119597	SLIT and NTRK-like family, member 6	0.72	0.74	1	0.53
ZNF665	ZNF665;FLJ14345;ZFP160L	zinc finger protein 665	0.7	0.62	1	0.49
CXCL9	CXCL9;CMK;Humig;MIG;SCYB9;crg-10	chemokine (C-X-C motif) ligand 9	0.67	0.71	1	0.5
XIRP2	XIRP2;CMYA3;DKFZp451G055;DKFZp779G1747;FLJ39111;FLJ40200;MYOMAXIN	xin actin-binding repeat containing 2	0.66	0.67	1	0.49
OR4M1	OR4M1;OR14-7	olfactory receptor, family 4, subfamily M, member 1	0.64	0.64	1	0.43
SEMG2	SEMG2;SGII	semenogelin II	0.64	0.66	1	0.44
KLRG1	KLRG1;2F1;CLEC15A;MAFA;MAFA-2F1;MAFA-L;MAFA-LIKE;MGC13600	killer cell lectin-like receptor subfamily G, member 1	0.61	0.58	1	0.42
MS4A2	MS4A2;APY;ATOPY;FCER1B;FCERI;IGEL;IGER;IGHER;MS4A1	membrane-spanning 4-domains, subfamily A, member 2 (Fc fragment of IgE, high affinity I, receptor for; beta polypeptide)	0.84	0.84	1.01	0.66
TXNDC8	TXNDC8;SPTRX-3;TRX6;bA427L11.2	thioredoxin domain containing 8 (spermatozoa)	0.83	0.82	1.01	0.58
KLRK1	KLRK1;CD314;D12S2489E;FLJ17759;FLJ75772;KLR;NKG2-D;NKG2D	killer cell lectin-like receptor subfamily K, member 1	0.83	0.84	1.01	0.58
C6orf146	C6orf146;MGC43581	chromosome 6 open reading frame 146	0.83	0.86	1.01	0.65
OR6B3	OR6B3;OR6B3P;OR6B3Q	olfactory receptor, family 6, subfamily B, member 3	0.83	0.85	1.01	0.59
OR13H1	OR13H1;ORX1	olfactory receptor, family 13, subfamily H, member 1	0.82	0.86	1.01	0.57
TBX22	TBX22;CLPA;CPX;TBXX;dJ795G23.1	T-box 22	0.8	0.82	1.01	0.63
KHDRBS2	KHDRBS2;FLJ38664;MGC26664;SLM-1;SLM1;bA535F17.1	KH domain containing, RNA binding, signal transduction associated 2	0.8	0.84	1.01	0.56
C2orf77	C2orf77	chromosome 2 open reading frame 77	0.78	0.82	1.01	0.61
KCNE2	KCNE2;ATFB4;LQT5;LQT6;MGC138292;MIRP1	potassium voltage-gated channel, Isk-related family, member 2	0.77	0.76	1.01	0.59
C17orf78	C17orf78;FLJ39647;MGC34759	chromosome 17 open reading frame 78	0.76	0.74	1.01	0.56
ZPBP2	ZPBP2;MGC41930;ZPBPL	zona pellucida binding protein 2	0.76	0.77	1.01	0.53
VWA3B	VWA3B;FLJ32686;MGC26733	von Willebrand factor A domain containing 3B	0.76	0.81	1.01	0.66
OR5AN1	OR5AN1;OR11-244	olfactory receptor, family 5, subfamily AN, member 1	0.74	0.79	1.01	0.56
KLRD1	KLRD1;CD94	killer cell lectin-like receptor subfamily D, member 1	0.72	0.77	1.01	0.51
LYZL6	LYZL6;LYC1;PRO1485;TKAL754;UNQ754	lysozyme-like 6	0.72	0.72	1.01	0.56
MAGEB2	MAGEB2;CT3.2;DAM6;MAGE-XP-2;MGC26438	melanoma antigen family B, 2	0.72	0.74	1.01	0.51
SEMG1	SEMG1;CT103;FLJ78262;MGC14719;SEMG;SGI;dJ172H20.2	semenogelin I	0.66	0.66	1.01	0.46
DSPP	DSPP;DFNA39;DGI1;DMP3;DPP;DSP;DTDP2	dentin sialophosphoprotein	0.64	0.71	1.01	0.52
WFDC8	WFDC8;C20orf170;WAP8;dJ461P17.1	WAP four-disulfide core domain 8	0.64	0.62	1.01	0.52
RBM46	RBM46;CT68;MGC27016	RNA binding motif protein 46	0.84	0.9	1.02	0.6
DGKB	DGKB;DAGK2;DGK;DGK-BETA;KIAA0718	diacylglycerol kinase, beta 90kDa	0.84	0.86	1.02	0.71
PKHD1L1	PKHD1L1;DKFZp586C1021;PKHDL1	polycystic kidney and hepatic disease 1 (autosomal recessive)-like 1	0.82	0.84	1.02	0.57
KRTAP19-2	KRTAP19-2;KAP19.2	keratin associated protein 19-2	0.82	0.8	1.02	0.61
EFHC2	EFHC2;DKFZp686G08235;FLJ22601;FLJ22843;dJ1158H2.1	EF-hand domain (C-terminal) containing 2	0.82	0.78	1.02	0.64
RFX6	RFX6;MGC33442;RFXDC1;dJ955L16.1	regulatory factor X, 6	0.81	0.85	1.02	0.57
GJA9	GJA9;CX58;CX59;GJA10;MGC50985	gap junction protein, alpha 9, 59kDa	0.78	0.76	1.02	0.64
PSG2	PSG2;CEA;PSBG2;PSG1;PSGGB	pregnancy specific beta-1-glycoprotein 2	0.78	0.84	1.02	0.59
UGT2A1	UGT2A1	UDP glucuronosyltransferase 2 family, polypeptide A1, complex locus	0.77	0.74	1.02	0.58
DDX4	DDX4;MGC111074;VASA	DEAD (Asp-Glu-Ala-Asp) box polypeptide 4	0.76	0.78	1.02	0.61
PCDH15	PCDH15;CDHR15;DFNB23;DKFZp667A1711;USH1F	protocadherin-related 15	0.74	0.75	1.02	0.58
PNLIP	PNLIP;PL;PTL	pancreatic lipase	0.74	0.74	1.02	0.57
P2RY12	P2RY12;ADPG-R;BDPLT8;HORK3;P2T(AC);P2Y(AC);P2Y(ADP);P2Y(cyc);P2Y12;SP1999	purinergic receptor P2Y, G-protein coupled, 12	0.74	0.74	1.02	0.67
ROS1	ROS1;MCF3;ROS;c-ros-1	c-ros oncogene 1 , receptor tyrosine kinase	0.74	0.8	1.02	0.57
CD80	CD80;B7;B7-1;B7.1;BB1;CD28LG;CD28LG1;LAB7	CD80 molecule	0.72	0.78	1.02	0.51
GAS2	GAS2;MGC32610	growth arrest-specific 2	0.7	0.68	1.02	0.49
RPGRIP1	RPGRIP1;CORD13;DKFZp686P0897;LCA6;RGI1;RGRIP;RPGRIP;RPGRIP1d	retinitis pigmentosa GTPase regulator interacting protein 1	0.69	0.66	1.02	0.57
C1orf49	C1orf49;DKFZp564J047;FLJ40199;MGC26863	chromosome 1 open reading frame 49	0.68	0.74	1.02	0.52
ASB17	ASB17;Asb-17	ankyrin repeat and SOCS box containing 17	0.63	0.62	1.02	0.44
SERPINB10	SERPINB10;PI-10;PI10	serpin peptidase inhibitor, clade B (ovalbumin), member 10	0.83	0.87	1.03	0.63
LYZ	LYZ;LZM	lysozyme	0.82	0.91	1.03	0.69
HSD17B13	HSD17B13;MGC138508;MGC138510;NIIL497;SCDR9;SDR16C3	hydroxysteroid (17-beta) dehydrogenase 13	0.81	0.84	1.03	0.57
RANBP3L	RANBP3L;FLJ25422	RAN binding protein 3-like	0.8	0.9	1.03	0.6
ZNF396	ZNF396;FLJ31213;ZSCAN14	zinc finger protein 396	0.8	0.86	1.03	0.7

MDH1B	MDH1B;FLJ25341;RP11-95H11	malate dehydrogenase 1B, NAD (soluble)	0.79	0.76	1.03	0.6
MYOZ2	MYOZ2;C4orf5;CMH16;CS-1	myozenin 2	0.78	0.82	1.03	0.59
TMEM174	TMEM174;FLJ31268;MGC13034	transmembrane protein 174	0.78	0.72	1.03	0.56
CLEC1B	CLEC1B;1810061I13Rik;CLEC2;CLEC2B;PRO1384;QDED721	C-type lectin domain family 1, member B	0.78	0.86	1.03	0.57
TAAR6	TAAR6;RP11-295F4.3;TA4;TAR4;TAR6;TRAR4	trace amine associated receptor 6	0.76	0.76	1.03	0.68
CCDC38	CCDC38;FLJ40089	coiled-coil domain containing 38	0.74	0.74	1.03	0.55
C6orf118	C6orf118;MGC23884;bA85G2.1;dJ416F21.2	chromosome 6 open reading frame 118	0.74	0.74	1.03	0.62
SERPINA7	SERPINA7;FLJ17820;TBG	serpin peptidase inhibitor, clade A (alpha-1 antiproteinase, antitrypsin), member 7	0.74	0.75	1.03	0.56
GPR174	GPR174	G protein-coupled receptor 174	0.73	0.69	1.03	0.52
DNASE2B	DNASE2B;DLAD	deoxyribonuclease II beta	0.7	0.77	1.03	0.51
OR8G2	OR8G2;HSTPCR120;OR8G2P;OR8G4;ORL206;ORL486;TPCR120	olfactory receptor, family 8, subfamily G, member 2	0.7	0.69	1.03	0.5
DCDC1	DCDC1;FLJ44078	doublecortin domain containing 1	0.7	0.74	1.03	0.57
IL22RA2	IL22RA2;CRF2-10;CRF2-S1;CRF2X;IL-22BP;IL-22RA2;MGC150509;MGC150510	interleukin 22 receptor, alpha 2	0.69	0.68	1.03	0.59
ABCA9	ABCA9;DKFZp686F2450;EST640918;MGC75415	ATP-binding cassette, sub-family A (ABC1), member 9	0.68	0.69	1.03	0.54
MGAT4C	MGAT4C;GNTIVH;HGNT-IV-H	mannosyl (alpha-1,3-)-glycoprotein beta-1,4-N-acetylglucosaminyltransferase, isozyme C (putative)	0.66	0.72	1.03	0.48
OR51Q1	OR51Q1	olfactory receptor, family 51, subfamily Q, member 1	0.62	0.66	1.03	0.44
DEFB108B	DEFB108B	defensin, beta 108B	0.84	0.94	1.04	0.63
SAMD13	SAMD13;RP11-376N17.1	sterile alpha motif domain containing 13	0.83	0.92	1.04	0.65
TYR	TYR;CMM8;OCA1A;OCAIA;SHEP3	tyrosinase (oculocutaneous albinism IA)	0.81	0.86	1.04	0.68
ATP6V0D2	ATP6V0D2;ATP6D2;FLJ38708;VMA6	ATPase, H+ transporting, lysosomal 38kDa, V0 subunit d2	0.79	0.77	1.04	0.61
PIP5K1B	PIP5K1B;MSS4;STM7	phosphatidylinositol-4-phosphate 5-kinase, type I, beta	0.79	0.88	1.04	0.63
OR1G1	OR1G1;OR17-130;OR17-209;OR1G2	olfactory receptor, family 1, subfamily G, member 1	0.79	0.84	1.04	0.57
CSRP3	CSRP3;CLP;CMD1M;CMH12;CRP3;LMO4;MGC14488;MGC61993;MLP	cysteine and glycine-rich protein 3 (cardiac LIM protein)	0.78	0.83	1.04	0.63
OR5K4	OR5K4	olfactory receptor, family 5, subfamily K, member 4	0.76	0.8	1.04	0.59
RFX4	RFX4;NYD-SP10	regulatory factor X, 4 (influences HLA class II expression)	0.76	0.81	1.04	0.58
TMPRSS11E	TMPRSS11E;DESC1;FLJ75331;FLJ94490;MGC141972;MGC141974;TMPRSS11E2	transmembrane protease, serine 11E	0.74	0.75	1.04	0.57
SGCZ	SGCZ;MGC149397;ZSG1	sarcoglycan, zeta	0.74	0.8	1.04	0.6
BTBD8	BTBD8	BTB (POZ) domain containing 8	0.74	0.74	1.04	0.54
RNF148	RNF148;MGC35222	ring finger protein 148	0.73	0.75	1.04	0.56
OR2M2	OR2M2;OR2M2Q;OST423	olfactory receptor, family 2, subfamily M, member 2	0.72	0.68	1.04	0.54
FNDC7	FNDC7;FLJ35838;MGC163293;RP11-293A10.2	fibronectin type III domain containing 7	0.72	0.76	1.04	0.57
PFKFB1	PFKFB1;F6PK;HL2K;MGC116715;MGC116717;PFRX	6-phosphofructo-2-kinase/fructose-2,6-biphosphatase 1	0.7	0.68	1.04	0.51
NR1H4	NR1H4;BAR;FXR;HRR-1;HRR1;MGC163445;RIP14	nuclear receptor subfamily 1, group H, member 4	0.69	0.76	1.04	0.57
CXorf59	CXorf59;FLJ36601;MGC126747;MGC126749	chromosome X open reading frame 59	0.69	0.78	1.04	0.52
PIP	PIP;GCDFP-15;GCDFP15;GPIP4	prolactin-induced protein	0.69	0.72	1.04	0.49
SSX5	SSX5;MGC9494	synovial sarcoma, X breakpoint 5	0.68	0.72	1.04	0.58
CHL1	CHL1;CALL;FLJ44930;L1CAM2;MGC132578	cell adhesion molecule with homology to L1CAM (close homolog of L1)	0.68	0.72	1.04	0.55
OR1L6	OR1L6;HG16;OR1L7;OR9-30	olfactory receptor, family 1, subfamily L, member 6	0.64	0.68	1.04	0.52
GNGT1	GNGT1;GNG1	guanine nucleotide binding protein (G protein), gamma transducing activity polypeptide 1	0.64	0.66	1.04	0.51
FLJ42177	FLJ42177	ENTREZ STATUS: REMAPPED TO GENE ID: 221264	0.62	0.58	1.04	0.49
CYLC1	CYLC1;CYCL1	cylicin, basic protein of sperm head cytoskeleton 1	0.62	0.66	1.04	0.48
VNN3	VNN3;HSA238982;MGC171203	vanin 3	0.62	0.65	1.04	0.52
SPATA8	SPATA8;MGC102678;MGC44294;SRG8	spermatogenesis associated 8	0.83	0.91	1.05	0.65
ZNF763	ZNF763;ZNF;ZNF440L	zinc finger protein 763	0.83	0.84	1.05	0.65
C12orf42	C12orf42;FLJ25323;MGC43592;MGC57409	chromosome 12 open reading frame 42	0.82	0.92	1.05	0.64
OR52E6	OR52E6;OR11-58	olfactory receptor, family 52, subfamily E, member 6	0.82	0.9	1.05	0.67
OR2T6	OR2T6;OR2T6P;OR2T9;OST703	olfactory receptor, family 2, subfamily T, member 6	0.82	0.9	1.05	0.6
CDKL4	CDKL4	cyclin-dependent kinase-like 4	0.81	0.91	1.05	0.65
HIST1H2BA	HIST1H2BA;H2BFU;STBP;TSH2B;bA317E16.3	histone cluster 1, H2ba	0.8	0.82	1.05	0.64
LYZL1	LYZL1;KAAG648;LYC2;MGC33408;PRO1278;bA534G20.1	lysozyme-like 1	0.79	0.82	1.05	0.61
CYP3A7	CYP3A7;CP37;P450-HFLA	cytochrome P450, family 3, subfamily A, polypeptide 7	0.78	0.86	1.05	0.61
EPGN	EPGN;ALGV3072;EPG;FLJ75542;PRO9904;epigen	epithelial mitogen homolog (mouse)	0.77	0.85	1.05	0.69
OR2M3	OR2M3;OR1-54;OR2M3P;OR2M6;OST003	olfactory receptor, family 2, subfamily M, member 3	0.76	0.84	1.05	0.56
OR51E2	OR51E2;OR51E3P;OR52A2;PSGR	olfactory receptor, family 51, subfamily E, member 2	0.76	0.82	1.05	0.62
XKRY	XKRY;XKRY1;XKRY2	XK, Kell blood group complex subunit-related, Y-linked	0.74	0.82	1.05	0.55
DNAJC5B	DNAJC5B;CSP-beta;MGC26226	DnaJ (Hsp40) homolog, subfamily C, member 5 beta	0.68	0.71	1.05	0.61
OR10K2	OR10K2;OR1-4	olfactory receptor, family 10, subfamily K, member 2	0.66	0.74	1.05	0.55
ARGFX	ARGFX	arginine-fifty homeobox	0.66	0.66	1.05	0.52
CCL23	CCL23;CK-BETA-8;CKb8;Ckb-8;Ckb-8-1;MIP-3;MIP3;MPIF-1;SCYA23	chemokine (C-C motif) ligand 23	0.63	0.69	1.05	0.46
PLEK	PLEK;FLJ27168;P47	pleckstrin	0.62	0.63	1.05	0.48
TGIF2LX	TGIF2LX;MGC34726;TGIFLX	TGFB-induced factor homeobox 2-like, X-linked	0.84	0.86	1.06	0.67
LRIT2	LRIT2;LRRC22	leucine-rich repeat, immunoglobulin-like and transmembrane domains 2	0.84	0.98	1.06	0.65
MS4A4A	MS4A4A;4SPAN1;CD20-L1;CD20L1;MGC22311;MS4A4;MS4A7	membrane-spanning 4-domains, subfamily A, member 4	0.83	0.82	1.06	0.7
KLRC4	KLRC4;FLJ17759;FLJ78582;NKG2-F;NKG2F	killer cell lectin-like receptor subfamily C, member 4	0.82	0.9	1.06	0.68
OR5H2	OR5H2;OR3-10	olfactory receptor, family 5, subfamily H, member 2	0.82	0.9	1.06	0.61
TEX12	TEX12	testis expressed 12	0.82	0.92	1.06	0.61
IFNA5	IFNA5;IFN-alphaG;INFA5	interferon, alpha 5	0.8	0.85	1.06	0.59
RNASE2	RNASE2;EDN;RNS2	ribonuclease, RNase A family, 2 (liver, eosinophil-derived neurotoxin)	0.8	0.89	1.06	0.59
ABCB11	ABCB11;ABC16;BRIC2;BSEP;PFIC-2;PFIC2;PGY4;SPGP	ATP-binding cassette, sub-family B (MDR/TAP), member 11	0.79	0.9	1.06	0.67
RGS18	RGS18;RGS13	regulator of G-protein signaling 18	0.78	0.78	1.06	0.66
CXCL10	CXCL10;C7;IFI10;INP10;IP-10;SCYB10;crg-2;gIP-10;mob-1	chemokine (C-X-C motif) ligand 10	0.78	0.88	1.06	0.71
DAZ3	DAZ3;MGC126441;pDP1679	deleted in azoospermia 3	0.76	0.8	1.06	0.66
FMO1	FMO1	flavin containing monooxygenase 1	0.74	0.79	1.06	0.53
POSTN	POSTN;MGC119510;MGC119511;OSF-2;OSF2;PDLPOSTN;PN;RP11-412K4.1;periostin	periostin, osteoblast specific factor	0.73	0.82	1.06	0.57
IFNG	IFNG;IFG;IFI	interferon, gamma	0.72	0.74	1.06	0.55
FAM19A2	FAM19A2;DKFZp761E1217;DKFZp781P0552;MGC42403;TAFA-2;TAFA2	family with sequence similarity 19 (chemokine (C-C motif)-like), member A2	0.71	0.72	1.06	0.55
TM4SF20	TM4SF20;FLJ22800;PRO994;TCCE518	transmembrane 4 L six family member 20	0.7	0.77	1.06	0.54
PPYR1	PPYR1;MGC116897;NPY4-R;NPY4R;PP1;Y4	pancreatic polypeptide receptor 1	0.68	0.68	1.06	0.62
FUT9	FUT9;Fuc-TIX	fucosyltransferase 9 (alpha (1,3) fucosyltransferase)	0.65	0.7	1.06	0.46
SPATA12	SPATA12;SRG5	spermatogenesis associated 12	0.64	0.69	1.06	0.5
C12orf28	C12orf28;C12orf15;FLJ25056;MGC126869;bcm1377	chromosome 12 open reading frame 28	0.63	0.66	1.06	0.48
TMIGD1	TMIGD1;TMIGD;UNQ9372	transmembrane and immunoglobulin domain containing 1	0.62	0.71	1.06	0.47
GKN2	GKN2;GDDR;PRO813;TFIZ1;VLTI465	gastrokine 2	0.84	0.89	1.07	0.64
OR1N2	OR1N2;OR9-23	olfactory receptor, family 1, subfamily N, member 2	0.84	0.93	1.07	0.71
OPN5	OPN5;GPR136;PGR12;TMEM13	opsin 5	0.84	0.86	1.07	0.78
C12orf54	C12orf54;HSD-29;HSD-30;MGC35033	chromosome 12 open reading frame 54	0.82	0.9	1.07	0.68
DEFB104A	DEFB104A;BD-4;DEFB-4;DEFB104;DEFB104B;DEFB4;MGC118942;MGC118944;MGC118945;hBD-4	defensin, beta 104A	0.79	0.92	1.07	0.68
OR7E24	OR7E24;HSHT2;OR19-8;OR7E24P;OR7E24Q	olfactory receptor, family 7, subfamily E, member 24	0.78	0.87	1.07	0.65
OR1J1	OR1J1;OR9-18;hg32	olfactory receptor, family 1, subfamily J, member 1	0.77	0.86	1.07	0.61
S100G	S100G;CABP;CABP1;CABP9K;CALB3;MGC138379	S100 calcium binding protein G	0.76	0.8	1.07	0.58
CFHR2	CFHR2;CFHL2;FHR2;HFL3	complement factor H-related 2	0.75	0.78	1.07	0.59
SLC17A3	SLC17A3;NPT4	solute carrier family 17 (sodium phosphate), member 3	0.75	0.77	1.07	0.56
REG3G	REG3G;MGC118998;MGC118999;MGC119001;PAP IB;PAP1B;PAPIB;REG-III;UNQ429	regenerating islet-derived 3 gamma	0.72	0.76	1.07	0.55
OR2T10	OR2T10;OR1-64	olfactory receptor, family 2, subfamily T, member 10	0.71	0.74	1.07	0.53
SERPINB5	SERPINB5;PI5;maspin	serpin peptidase inhibitor, clade B (ovalbumin), member 5	0.71	0.7	1.07	0.56
HP	HP;BP;HP2ALPHA2;HPA1S;MGC111141	haptoglobin	0.67	0.71	1.07	0.53
DGAT2L6	DGAT2L6;DC3;FLJ25989	diacylglycerol O-acyltransferase 2-like 6	0.66	0.75	1.07	0.52
DEFB1	DEFB1;BD1;DEFB-1;DEFB101;HBD1;MGC51822	defensin, beta 1	0.66	0.7	1.07	0.54
SLC7A13	SLC7A13;AGT-1;AGT1;MGC150476;MGC150477;XAT2	solute carrier family 7 (anionic amino acid transporter), member 13	0.84	0.9	1.08	0.67
GALNTL5	GALNTL5;GALNT15	UDP-N-acetyl-alpha-D-galactosamine:polypeptide N-acetylgalactosaminyltransferase-like 5	0.84	0.85	1.08	0.67
TPD52L3	TPD52L3;MGC26757;MGC45374;NYDSP25;hD55	tumor protein D52-like 3	0.83	0.9	1.08	0.74
CRYGD	CRYGD;CACA;CCA3;CCP;CRYG4;cry-g-D	crystallin, gamma D	0.81	0.88	1.08	0.61
TLR1	TLR1;CD281;DKFZp547I0610;DKFZp564I0682;KIAA0012;MGC104956;MGC126311;MGC126312;TIL;TIL. LPRS5;rsc786	toll-like receptor 1	0.8	0.92	1.08	0.6
SLC17A1	SLC17A1;MGC126794;MGC126796;NAPI-1;NPT-1;NPT1	solute carrier family 17 (sodium phosphate), member 1	0.77	0.78	1.08	0.62
ANGPTL3	ANGPTL3;ANGPT5;FHBL2	angiopoietin-like 3	0.76	0.8	1.08	0.69
DAOA	DAOA;G72;LG72;SG72	D-amino acid oxidase activator	0.76	0.82	1.08	0.56
CAGE1	CAGE1;CT3;CT95;CTAG3;FLJ40441;bA69L16.7	cancer antigen 1	0.74	0.83	1.08	0.6
ANGPTL5	ANGPTL5	angiopoietin-like 5	0.7	0.74	1.08	0.55
OR10J5	OR10J5;OR1-28	olfactory receptor, family 10, subfamily J, member 5	0.69	0.76	1.08	0.58
CCDC102B	CCDC102B;ACY1L;AN;C18orf14;DKFZp434K1426;DKFZp686I08254;FLJ23594;HsT1731;MGC161726;MGC161728	coiled-coil domain containing 102B	0.68	0.78	1.08	0.52
OR1Q1	OR1Q1;HSTPCR106;OR1Q2;OR1Q3;OR9-25;OR9-A;OST226;OST226OR9-A;TPCR106	olfactory receptor, family 1, subfamily Q, member 1	0.68	0.76	1.08	0.62
ZMAT1	ZMAT1;KIAA1789;MGC176597;MGC176728	zinc finger, matrin-type 1	0.66	0.76	1.08	0.53
OR1C1	OR1C1;HSTPCR27;OR1-42;OR1.5.10;ORL211;TPCR27	olfactory receptor, family 1, subfamily C, member 1	0.65	0.73	1.08	0.49
SLC24A5	SLC24A5;JSX;NCKX5;SHEP4	solute carrier family 24, member 5	0.84	0.95	1.09	0.65
ADH6	ADH6;ADH-5	alcohol dehydrogenase 6 (class V)	0.83	0.94	1.09	0.73
GRIK1	GRIK1;EAA3;EEA3;GLR5;GLUR5	glutamate receptor, ionotropic, kainate 1	0.82	0.95	1.09	0.74
MS4A6A	MS4A6A;4SPAN3;4SPAN3.2;CD20L3;MGC131944;MGC22650;MS4A6;MST090;MSTP090	membrane-spanning 4-domains, subfamily A, member 6A	0.8	0.88	1.09	0.63
VSIG1	VSIG1;1700062D20Rik;GPA34;MGC44287;dJ889N15.1	V-set and immunoglobulin domain containing 1	0.78	0.82	1.09	0.69
OR5B2	OR5B2;OR11-240;OST073	olfactory receptor, family 5, subfamily B, member 2	0.77	0.88	1.09	0.72
SLC5A7	SLC5A7;CHT;CHT1;MGC126299;MGC126300;hCHT	solute carrier family 5 (choline transporter), member 7	0.76	0.78	1.09	0.62
MUC15	MUC15;MUC-15;PAS3;PASIII	mucin 15, cell surface associated	0.74	0.78	1.09	0.61
ZNF257	ZNF257;BMZF-4;BMZF4	zinc finger protein 257	0.72	0.83	1.09	0.56
C2orf61	C2orf61;FLJ40172	chromosome 2 open reading frame 61	0.69	0.78	1.09	0.52
ZNF781	ZNF781;FLJ37549;MGC131783	zinc finger protein 781	0.84	0.82	1.1	0.65
FBXO47	FBXO47	F-box protein 47	0.82	0.86	1.1	0.62
PDE3B	PDE3B;HcGIP1;cGIPDE1	phosphodiesterase 3B, cGMP-inhibited	0.81	0.83	1.1	0.66
C14orf39	C14orf39;MGC149706;Six6os1	chromosome 14 open reading frame 39	0.76	0.88	1.1	0.58
CXCR7	CXCR7;CMKOR1;GPR159;RDC1	chemokine (C-X-C motif) receptor 7	0.76	0.8	1.1	0.73
IDI2	IDI2;IPPI2	isopentenyl-diphosphate delta isomerase 2	0.76	0.88	1.1	0.59

OR4N4	OR4N4;OR15-1;OR15-5	olfactory receptor, family 4, subfamily N, member 4	0.75	0.8	1.1	0.58
EVI2A	EVI2A;EVDA;EVI-2A;EVI2	ecotropic viral integration site 2A	0.75	0.74	1.1	0.59
HPGD	HPGD;15-PGDH;PGDH;PGDH1;SDR36C1	hydroxyprostaglandin dehydrogenase 15-(NAD)	0.73	0.85	1.1	0.56
XCL2	XCL2;SCM-1b;SCM1B;SCYC2	chemokine (C motif) ligand 2	0.72	0.84	1.1	0.62
OR52L1	OR52L1;OR11-50	olfactory receptor, family 52, subfamily L, member 1	0.71	0.82	1.1	0.67
SERPINB12	SERPINB12;MGC119247;MGC119248;YUKOPIN	serpin peptidase inhibitor, clade B (ovalbumin), member 12	0.69	0.74	1.1	0.56
TINAG	TINAG;TIN-AG	tubulointerstitial nephritis antigen	0.69	0.75	1.1	0.57
DAZ4	DAZ4;pDP1680;pDP1681	deleted in azoospermia 4	0.68	0.78	1.1	0.59
ABCA6	ABCA6;EST155051;FLJ43498	ATP-binding cassette, sub-family A (ABC1), member 6	0.64	0.68	1.1	0.53
RASGRP3	RASGRP3;GRP3;KIAA0846	RAS guanyl releasing protein 3 (calcium and DAG-regulated)	0.61	0.6	1.1	0.5
TBX15	TBX15;TBX14	T-box 15	0.79	0.89	1.11	0.65
ARHGAP28	ARHGAP28;DKFZp686A2038;FLJ10312;FLJ27160	Rho GTPase activating protein 28	0.76	0.76	1.11	0.6
OR6C76	OR6C76	olfactory receptor, family 6, subfamily C, member 76	0.74	0.77	1.11	0.65
MNDA	MNDA;PYHIN3	myeloid cell nuclear differentiation antigen	0.72	0.82	1.11	0.62
VNN2	VNN2;FOAP-4;GPI-80	vanin 2	0.69	0.73	1.11	0.53
RIMS1	RIMS1;CORD7;KIAA0340;MGC167823;MGC176677;RAB3IP2;RIM;RIM1	regulating synaptic membrane exocytosis 1	0.69	0.79	1.11	0.54
IFNA16	IFNA16;IFN-alphaO	interferon, alpha 16	0.84	0.99	1.12	0.8
OR13C5	OR13C5;OR9-11	olfactory receptor, family 13, subfamily C, member 5	0.82	0.97	1.12	0.68
OR52I2	OR52I2;OR11-12	olfactory receptor, family 52, subfamily I, member 2	0.8	0.88	1.12	0.71
LOC152586	LOC152586;MGC43429	glycosyltransferase 54 domain-containing protein	0.77	0.9	1.12	0.6
PTH	PTH;PTH1	parathyroid hormone	0.76	0.88	1.12	0.72
C9orf84	C9orf84;FLJ32779;FLJ44067;MGC43661	chromosome 9 open reading frame 84	0.74	0.75	1.12	0.62
CNTNAP4	CNTNAP4;CASPR4;KIAA1763	contactin associated protein-like 4	0.74	0.86	1.12	0.58
GLYAT	GLYAT;ACGNAT;CAT;GAT	glycine-N-acyltransferase	0.72	0.77	1.12	0.6
CNTN3	CNTN3;BIG-1;KIAA1496;PANG;PCS	contactin 3 (plasmacytoma associated)	0.72	0.78	1.12	0.61
UTS2	UTS2;PRO1068;U-II;UCN2;UII	urotensin 2	0.7	0.82	1.12	0.58
CCRL2	CCRL2;CKRX;CRAM;CRAM-A;CRAM-B;FLJ55815;HCR;MGC116710;MGC34104	chemokine (C-C motif) receptor-like 2	0.68	0.74	1.12	0.66
OR14J1	OR14J1;OR5U1;OR6-25;bA150A6.4;hs6M1-28	olfactory receptor, family 14, subfamily J, member 1	0.67	0.74	1.12	0.55
ASCL3	ASCL3;HASH3;SGN1;bHLHa42	achaete-scute complex homolog 3 (Drosophila)	0.66	0.68	1.12	0.54
OR2B6	OR2B6;MGC120190;MGC129828;OR2B1;OR2B1P;OR2B5;OR2B6P;OR5-40;OR5-41;OR6-31;dJ408B20.2	olfactory receptor, family 2, subfamily B, member 6	0.64	0.76	1.12	0.51
ST8SIA3	ST8SIA3;SIAT8C;ST8SiaIII	ST8 alpha-N-acetyl-neuraminide alpha-2,8-sialyltransferase 3	0.62	0.64	1.12	0.51
BOLL	BOLL;BOULE	bol, boule-like (Drosophila)	0.62	0.67	1.12	0.49
ZSCAN4	ZSCAN4;FLJ35105;MGC126787;MGC126789;ZNF494	zinc finger and SCAN domain containing 4	0.84	0.89	1.13	0.72
PLA2G7	PLA2G7;LDL-PLA2;LP-PLA2;PAFAD;PAFAH	phospholipase A2, group VII (platelet-activating factor acetylhydrolase, plasma)	0.82	0.88	1.13	0.63
UTS2D	UTS2D;MGC138371;U2B;URP	urotensin 2 domain containing	0.8	0.85	1.13	0.72
GPR85	GPR85;FLJ50993;FLJ51159;FLJ99433;SREB;SREB2	G protein-coupled receptor 85	0.8	0.87	1.13	0.79
SPINK2	SPINK2;HUSI-II	serine peptidase inhibitor, Kazal type 2 (acrosin-trypsin inhibitor)	0.78	0.89	1.13	0.62
SYCP2	SYCP2;SCP2	synaptonemal complex protein 2	0.74	0.86	1.13	0.62
HORMAD2	HORMAD2;MGC26710	HORMA domain containing 2	0.74	0.88	1.13	0.62
OMD	OMD;OSAD;SLRR2C	osteomodulin	0.73	0.86	1.13	0.7
SMR3B	SMR3B;MGC104379;P-B;PBII;PRL3;PROL3;SMR1B	submaxillary gland androgen regulated protein 3B	0.72	0.88	1.13	0.64
GDNF	GDNF;ATF1;ATF2;HFB1-GDNF;HSCR3	glial cell derived neurotrophic factor	0.72	0.82	1.13	0.55
C12orf40	C12orf40;FLJ40126	chromosome 12 open reading frame 40	0.68	0.82	1.13	0.62
LALBA	LALBA;MGC138521;MGC138523	lactalbumin, alpha-	0.66	0.78	1.13	0.61
RIC3	RIC3;AYST720;FLJ11608;PRO1385	resistance to inhibitors of cholinesterase 3 homolog (C. elegans)	0.61	0.7	1.13	0.54
CFHR4	CFHR4;CFHL4;FHR-4;FHR4	complement factor H-related 4	0.82	0.98	1.14	0.71
SLCO1B3	SLCO1B3;LST-3TM13;LST3;OATP1B3;OATP8;SLC21A8	solute carrier organic anion transporter family, member 1B3	0.77	0.84	1.14	0.61
USP50	USP50	ubiquitin specific peptidase 50	0.76	0.86	1.14	0.72
FBXL13	FBXL13;FLJ38068;Fbl13;MGC21636	F-box and leucine-rich repeat protein 13	0.76	0.83	1.14	0.6
MS4A6E	MS4A6E;MGC126832	membrane-spanning 4-domains, subfamily A, member 6E	0.74	0.83	1.14	0.63
AGTR2	AGTR2;AT2;ATGR2;MRX88	angiotensin II receptor, type 2	0.72	0.76	1.14	0.57
NAT2	NAT2;AAC2;PNAT	N-acetyltransferase 2 (arylamine N-acetyltransferase)	0.71	0.76	1.14	0.6
KCNJ13	KCNJ13;KIR1.4;KIR7.1;LCA16;MGC33328;SVD	potassium inwardly-rectifying channel, subfamily J, member 13	0.69	0.83	1.14	0.64
OR5P3	OR5P3;JCG1	olfactory receptor, family 5, subfamily P, member 3	0.68	0.8	1.14	0.58
DDX25	DDX25;GRTH	DEAD (Asp-Glu-Ala-Asp) box polypeptide 25	0.66	0.73	1.14	0.51
MYH8	MYH8;MyHC-peri;MyHC-pn;gtMHC-F	myosin, heavy chain 8, skeletal muscle, perinatal	0.64	0.71	1.14	0.55
SERPINB11	SERPINB11;EPIPIN;MGC163242;MGC163244;SERPIN11	serpin peptidase inhibitor, clade B (ovalbumin), member 11 (gene/pseudogene)	0.64	0.75	1.14	0.57
DCN	DCN;CSCD;DSPG2;PG40;PGII;PGS2;SLRR1B	decorin	0.8	0.95	1.15	0.71
HAPLN1	HAPLN1;CRTL1	hyaluronan and proteoglycan link protein 1	0.78	0.88	1.15	0.71
C22orf31	C22orf31;HS747E2A;bK747E2.1	chromosome 22 open reading frame 31	0.76	0.94	1.15	0.66
KLRB1	KLRB1;CD161;CLEC5B;MGC138614;NKR;NKR-P1;NKR-P1A;NKRP1A;hNKR-P1A	killer cell lectin-like receptor subfamily B, member 1	0.76	0.88	1.15	0.64
ANO5	ANO5;GDD1;LGMD2L;TMEM16E	anoctamin 5	0.67	0.79	1.15	0.6
FBXW12	FBXW12;FBW12;FBXO12;FBXO35;MGC120385;MGC120386;MGC120387	F-box and WD repeat domain containing 12	0.64	0.7	1.15	0.5
IFNA21	IFNA21;IFN-alphaI;LeIF F;MGC126687;MGC126689;leIF-F	interferon, alpha 21	0.62	0.7	1.15	0.58
LRGUK	LRGUK;FLJ32786	leucine-rich repeats and guanylate kinase domain containing	0.82	1.04	1.16	0.69
OR5AC2	OR5AC2;HSA1	olfactory receptor, family 5, subfamily AC, member 2	0.77	0.86	1.16	0.66
PSG8	PSG8;DKFZp781L10202;PSG1	pregnancy specific beta-1-glycoprotein 8	0.72	0.84	1.16	0.59
RAG2	RAG2;RAG-2	recombination activating gene 2	0.71	0.83	1.16	0.64
RERGL	RERGL;FLJ22655	RERG/RAS-like	0.7	0.82	1.16	0.56
MAEL	MAEL;CT128;FLJ14904;RP11-102C16.1	maelstrom homolog (Drosophila)	0.7	0.78	1.16	0.59
ANKRD45	ANKRD45;CT117;FLJ45235;MGC161631;MGC161633;RP3-436N22.4	ankyrin repeat domain 45	0.7	0.78	1.16	0.63
OR1L1	OR1L1;HG23;OR1L2;OR9-27;OR9-C	olfactory receptor, family 1, subfamily L, member 1	0.69	0.8	1.16	0.56
SULT6B1	SULT6B1	sulfotransferase family, cytosolic, 6B, member 1	0.68	0.77	1.16	0.62
ALS2CR11	ALS2CR11;DKFZp686H0636;FLJ25351;FLJ40332	amyotrophic lateral sclerosis 2 (juvenile) chromosome region, candidate 11	0.67	0.74	1.16	0.59
OR4Q3	OR4Q3;C14orf13;HSA6;OR14-3;OR4Q4;c14_5008	olfactory receptor, family 4, subfamily Q, member 3	0.66	0.79	1.16	0.53
GHR	GHR;GHBP	growth hormone receptor	0.66	0.74	1.16	0.54
IL18RAP	IL18RAP;ACPL;CD218b;CDw218b;IL18RB;MGC120589;MGC120590	interleukin 18 receptor accessory protein	0.66	0.75	1.16	0.65
KRTAP21-1	KRTAP21-1;KAP21.1	keratin associated protein 21-1	0.65	0.78	1.16	0.52
MIA2	MIA2;FLJ22404	melanoma inhibitory activity 2	0.64	0.76	1.16	0.55
OFCC1	OFCC1;MGC142101;MGC142103;MRDS1	orofacial cleft 1 candidate 1	0.62	0.7	1.16	0.53
MSR1	MSR1;CD204;SCARA1;SR-A;SRA;phSR1;phSR2	macrophage scavenger receptor 1	0.61	0.7	1.16	0.52
ADAM29	ADAM29;CT73;svph1	ADAM metallopeptidase domain 29	0.83	0.9	1.17	0.67
ANKRD7	ANKRD7;TSA806	ankyrin repeat domain 7	0.8	0.96	1.17	0.75
ADCY10	ADCY10;HCA2;RP1-313L4.2;SAC;SACI;Sacy	adenylate cyclase 10 (soluble)	0.76	0.87	1.17	0.64
LECT1	LECT1;BRICD3;CHM-I;CHM1;MYETS1	leukocyte cell derived chemotaxin 1	0.75	0.9	1.17	0.68
ZNF429	ZNF429	zinc finger protein 429	0.7	0.81	1.17	0.56
OR4P4	OR4P4;OR4P3P	olfactory receptor, family 4, subfamily P, member 4	0.68	0.79	1.17	0.65
DUSP21	DUSP21;LMWDSP21;MGC149878	dual specificity phosphatase 21	0.65	0.74	1.17	0.66
COX7B2	COX7B2;MGC102754	cytochrome c oxidase subunit VIIb2	0.64	0.8	1.17	0.65
PLCL1	PLCL1;MGC126580;MGC138190;PLCE;PLCL;PLDL1;PRIP	phospholipase C-like 1	0.62	0.71	1.17	0.56
FLJ39061	FLJ39061;FLJ52575;KIAA2012;MGC141748	uncharacterized protein FLJ39061	0.84	1.05	1.18	0.74
GRIN3A	GRIN3A;FLJ45414;NMDAR-L;NR3A	glutamate receptor, ionotropic, N-methyl-D-aspartate 3A	0.84	0.99	1.18	0.77
LIPJ	LIPJ;FLJ11218;LIPL1;bA425M17.2	lipase, family member J	0.82	0.94	1.18	0.73
CFHR1	CFHR1;CFHL;CFHL1;CFHL1P;CFHR1P;FHR1;H36-1;H36-2;HFL1;HFL2;MGC104329	complement factor H-related 1	0.8	0.92	1.18	0.7
CLECL1	CLECL1;DCAL1	C-type lectin-like 1	0.8	0.98	1.18	0.69
RAD9B	RAD9B;FLJ40346;MGC75426	RAD9 homolog B (S. pombe)	0.8	0.86	1.18	0.68
FABP1	FABP1;FABPL;L-FABP	fatty acid binding protein 1, liver	0.76	0.88	1.18	0.76
ADAM2	ADAM2;CRYN1;CRYN2;CT15;FTNB;PH-30b;PH30	ADAM metallopeptidase domain 2	0.74	0.86	1.18	0.65
CD84	CD84;DKFZp781E2378;LY9B;SLAMF5;hCD84;mCD84	CD84 molecule	0.72	0.88	1.18	0.59
ATP8B4	ATP8B4;ATPIM;FLJ25418	ATPase, class I, type 8B, member 4	0.66	0.76	1.18	0.66
CLC	CLC;GAL10;Gal-10;LGALS10;LGALS10A;LPPL_HUMAN;MGC149659	Charcot-Leyden crystal protein	0.65	0.73	1.18	0.57
RGS22	RGS22;DKFZp434I092;FLJ40080;FLJ75004;MGC102908;PRTD-NY2	regulator of G-protein signaling 22	0.64	0.82	1.18	0.6
PDE11A	PDE11A;FLJ23693;MGC133355;MGC133356;PPNAD2	phosphodiesterase 11A	0.64	0.74	1.18	0.63
TMEM202	TMEM202;FLJ27523	transmembrane protein 202	0.84	0.98	1.19	0.74
FLJ45079	FLJ45079;FLJ46474	FLJ45079 protein	0.78	0.84	1.19	0.67
SLC2A2	SLC2A2;GLUT2	solute carrier family 2 (facilitated glucose transporter), member 2	0.74	0.93	1.19	0.61
CCDC110	CCDC110;CT52;KM-HN-1;KMHN1;MGC33607	coiled-coil domain containing 110	0.7	0.88	1.19	0.62
OR5K2	OR5K2;OR3-9	olfactory receptor, family 5, subfamily K, member 2	0.68	0.79	1.19	0.59
PCSK1	PCSK1;BMIQ12;NEC1;PC1;PC3;SPC3	proprotein convertase subtilisin/kexin type 1	0.65	0.76	1.19	0.57
STARD6	STARD6	StAR-related lipid transfer (START) domain containing 6	0.62	0.71	1.19	0.56
SLC38A11	SLC38A11;AVT2;FLJ39822;MGC150450	solute carrier family 38, member 11	0.62	0.71	1.19	0.5
GPR183	GPR183;EBI2	G protein-coupled receptor 183	0.82	0.96	1.2	0.86
IL23R	IL23R	interleukin 23 receptor	0.8	0.92	1.2	0.72
ATP6V1G3	ATP6V1G3;ATP6G3;MGC119810;MGC119813;Vma10	ATPase, H+ transporting, lysosomal 13kDa, V1 subunit G3	0.8	0.92	1.2	0.67
OR9I1	OR9I1;OR11-228	olfactory receptor, family 9, subfamily I, member 1	0.73	0.92	1.2	0.65
WDR64	WDR64;FLJ32978	WD repeat domain 64	0.72	0.81	1.2	0.66
OR6C68	OR6C68	olfactory receptor, family 6, subfamily C, member 68	0.71	0.89	1.2	0.63
PRLR	PRLR;hPRLrI	prolactin receptor	0.71	0.84	1.2	0.72
ZDHHC15	ZDHHC15;FLJ31812;MGC119974;MGC119975;MGC119976;MRX91	zinc finger, DHHC-type containing 15	0.7	0.77	1.2	0.61
IL22	IL22;IL-21;IL-22;IL-D110;IL-TIF;ILTIF;MGC79382;MGC79384;TIFIL-23;TIFa;zcyto18	interleukin 22	0.68	0.79	1.2	0.6
IAPP	IAPP;DAP;IAP	islet amyloid polypeptide	0.64	0.76	1.2	0.53
MS4A7	MS4A7;4SPAN2;CD20L4;CFFM4;MGC22368;MS4A8	membrane-spanning 4-domains, subfamily A, member 7	0.84	0.95	1.21	0.8
OR4K1	OR4K1;OR14-19	olfactory receptor, family 4, subfamily K, member 1	0.84	0.99	1.21	0.83
KRTAP21-2	KRTAP21-2;KAP21.2	keratin associated protein 21-2	0.84	0.99	1.21	0.78
IFNA10	IFNA10;IFN-alphaC;MGC119878;MGC119879	interferon, alpha 10	0.8	0.94	1.21	0.71
C15orf54	C15orf54;FLJ39531	chromosome 15 open reading frame 54	0.74	0.91	1.21	0.72
AMELY	AMELY;AMGL;AMGY	amelogenin, Y-linked	0.72	0.8	1.21	0.66
PYHIN1	PYHIN1;IFIX;MGC23885;RP11-520H16.1	pyrin and HIN domain family, member 1	0.72	0.94	1.21	0.66
ANKAR	ANKAR;FLJ25415	ankyrin and armadillo repeat containing	0.71	0.92	1.21	0.71

HEPACAM2	HEPACAM2;MIKI	HEPACAM family member 2	0.7	0.82	1.21	0.67
TACR3	TACR3;MGC148060;MGC148061;NK-3R;NK3R;NKR;TAC3RL	tachykinin receptor 3	0.7	0.84	1.21	0.75
PCDH11X	PCDH11X;PCDH-X;PCDH11;PCDHX	protocadherin 11 X-linked	0.7	0.82	1.21	0.65
MCART2	MCART2;MGC120230;MGC120231;MGC120232	mitochondrial carrier triple repeat 2	0.68	0.82	1.21	0.56
OR52K2	OR52K2;OR11-7	olfactory receptor, family 52, subfamily K, member 2	0.68	0.82	1.21	0.57
OR51M1	OR51M1;HOR5'Beta7;OR11-40	olfactory receptor, family 51, subfamily M, member 1	0.67	0.74	1.21	0.58
OR6C65	OR6C65	olfactory receptor, family 6, subfamily C, member 65	0.62	0.77	1.21	0.53
ADAMDEC1	ADAMDEC1;FLJ79219;M12.219	ADAM-like, decysin 1	0.82	0.99	1.22	0.74
CYP3A4	CYP3A4;CP33;CP34;CYP3A;CYP3A3;CYPIIIA3;CYPIIIA4;HLP;MGC126680;NF-25;P450C3;P450PCN1	cytochrome P450, family 3, subfamily A, polypeptide 4	0.81	0.94	1.22	0.74
RP1	RP1;DCDC4A;FLJ50293;FLJ55454;FLJ79410;ORP1	retinitis pigmentosa 1 (autosomal dominant)	0.78	0.9	1.22	0.76
IL4	IL4;BCGF-1;BCGF1;BSF-1;BSF1;IL-4;MGC79402	interleukin 4	0.77	0.9	1.22	0.67
NUP210L	NUP210L;RP11-144B19.1	nucleoporin 210kDa-like	0.74	0.8	1.22	0.63
IMPG1	IMPG1;GP147;IPM150;SPACR	interphotoreceptor matrix proteoglycan 1	0.7	0.83	1.22	0.65
C2orf63	C2orf63;FLJ31438	chromosome 2 open reading frame 63	0.69	0.84	1.22	0.57
SEPP1	SEPP1;SELP;SeP	selenoprotein P, plasma, 1	0.67	0.84	1.22	0.64
NETO1	NETO1;BCTL1;BTCL1;FLJ41325	neuropilin (NRP) and tolloid (TLL)-like 1	0.64	0.76	1.22	0.61
TDRD12	TDRD12;ECAT8;FLJ13072	tudor domain containing 12	0.64	0.76	1.22	0.58
MMP27	MMP27;MMP-27	matrix metallopeptidase 27	0.84	0.96	1.23	0.72
KLHL4	KLHL4;DKELCHL;KHL4;KIAA1687	kelch-like 4 (Drosophila)	0.79	1.02	1.23	0.76
DAZ2	DAZ2;MGC126442;pDP1678	deleted in azoospermia 2	0.78	0.92	1.23	0.68
OR4C16	OR4C16;OR11-135	olfactory receptor, family 4, subfamily C, member 16	0.76	0.91	1.23	0.68
WFDC11	WFDC11;MGC71905;WAP11	WAP four-disulfide core domain 11	0.74	0.91	1.23	0.63
BAAT	BAAT;BACAT;BAT;FLJ20300;MGC104432	bile acid CoA: amino acid N-acyltransferase (glycine N-choloyltransferase)	0.7	0.84	1.23	0.6
TMEM132B	TMEM132B;KIAA1786;KIAA1906	transmembrane protein 132B	0.69	0.8	1.23	0.65
OR52A1	OR52A1;HPFH1OR	olfactory receptor, family 52, subfamily A, member 1	0.64	0.82	1.23	0.55
KRT28	KRT28;KRT25D	keratin 28	0.61	0.7	1.23	0.57
ELTD1	ELTD1;ETL;KPG_003	EGF, latrophilin and seven transmembrane domain containing 1	0.84	0.97	1.24	0.73
APOH	APOH;B2G1;B2GP1;BG	apolipoprotein H (beta-2-glycoprotein I)	0.78	0.89	1.24	0.79
ART4	ART4;CD297;DO;DOK1	ADP-ribosyltransferase 4 (Dombrock blood group)	0.78	0.95	1.24	0.7
CLEC12A	CLEC12A;CLL-1;CLL1;DCAL-2;MGC70602;MICL	C-type lectin domain family 12, member A	0.69	0.84	1.24	0.63
STOML3	STOML3;Epb7.2l;SRO	stomatin (EPB72)-like 3	0.66	0.74	1.24	0.59
OR4K2	OR4K2;OR14-15	olfactory receptor, family 4, subfamily K, member 2	0.63	0.74	1.24	0.62
SPATA22	SPATA22;NYD-SP20;NYDSP20	spermatogenesis associated 22	0.62	0.72	1.24	0.59
MEPE	MEPE;OF45	matrix extracellular phosphoglycoprotein	0.61	0.78	1.24	0.53
SLC6A14	SLC6A14;BMIQ11	solute carrier family 6 (amino acid transporter), member 14	0.44	0.32	0.74	0.25
C5orf48	C5orf48;FLJ27505;MGC163367;MGC163369	chromosome 5 open reading frame 48	0.6	0.47	0.78	0.32
PFKFB1	PFKFB1;F6PK;HL2K;MGC116715;MGC116717;PFRX	6-phosphofructo-2-kinase/fructose-2,6-biphosphatase 1	0.52	0.44	0.8	0.32
ATP13A5	ATP13A5;FLJ16025	ATPase type 13A5	0.55	0.85	1.62	0.62
ICOS	ICOS;AILIM;CD278;CVID1;MGC39850	inducible T-cell co-stimulator	0.53	0.8	1.47	0.61
GIMAP7	GIMAP7;IAN7;MGC27027;hIAN7	GTPase, IMAP family member 7	0.5	0.82	1.49	0.54
POF1B	POF1B;FLJ22792;POF;POF2B	premature ovarian failure, 1B	0.57	0.89	1.51	0.67
PON3	PON3	paraoxonase 3	0.6	0.92	1.52	0.61
XAGE5	XAGE5;CT12.5;GAGED5;MGC119799;MGC119800;XAGE-5	X antigen family, member 5	0.6	0.87	1.41	0.65
DEFB114	DEFB114;DEFB-14	defensin, beta 114	0.57	0.9	1.43	0.66
CRISP3	CRISP3;Aeg2;CRISP-3;CRS3;MGC126588;SGP28;dJ442L6.3	cysteine-rich secretory protein 3	0.58	0.83	1.45	0.69
C12orf50	C12orf50;FLJ35821	chromosome 12 open reading frame 50	0.57	0.82	1.39	0.61
ACTRT1	ACTRT1;AIP1;ARIP1;ARPT1;HSD27;KIAA0705;MGC26590	actin-related protein T1	0.29	0.48	1.73	0.36
KERA	KERA;CNA2;SLRR2B	keratocan	0.09	0.18	2.19	0.15
YSK4	YSK4;FLJ23074;RCK	YSK4 Sps1/Ste20-related kinase homolog (S. cerevisiae)	0.4	0.63	1.44	0.45
C1QTNF7	C1QTNF7;CTRP7;ZACRP7	C1q and tumor necrosis factor related protein 7	0.33	0.48	1.44	0.35
GABRR3	GABRR3;DKFZp781O1955	gamma-aminobutyric acid (GABA) receptor, rho 3	0.12	0.18	1.44	0.14
ZNF676	ZNF676	zinc finger protein 676	0.36	0.51	1.45	0.39
ANKS1B	ANKS1B;AIDA;AIDA-1;ANKS2;EB-1;EB1;MGC26087;cajalin-2	ankyrin repeat and sterile alpha motif domain containing 1B	0.28	0.38	1.45	0.31
REG1B	REG1B;PSPS2;REGH;REGI-BETA;REGL	regenerating islet-derived 1 beta	0.49	0.72	1.46	0.5
CPXCR1	CPXCR1;CT77	CPX chromosome region, candidate 1	0.48	0.64	1.46	0.5
DEFB126	DEFB126;C20orf8;DEFB-26;DEFB26;ESP13.2;bA530N10.1;hBD-26	defensin, beta 126	0.4	0.6	1.46	0.4
HTR3B	HTR3B;5-HT3B	5-hydroxytryptamine (serotonin) receptor 3B	0.38	0.54	1.46	0.48
C1orf100	C1orf100	chromosome 1 open reading frame 100	0.14	0.18	1.47	0.14
TECTB	TECTB;KIAA1560;MGC142057;MGC142059	tectorin beta	0.6	0.8	1.48	0.63
KNG1	KNG1;BDK;KNG	kininogen 1	0.38	0.55	1.5	0.48
OR5T3	OR5T3;OR11-178;OR5T3Q	olfactory receptor, family 5, subfamily T, member 3	0.31	0.41	1.51	0.34
MSH4	MSH4	mutS homolog 4 (E. coli)	0.22	0.32	1.53	0.25
ASB4	ASB4;ASB-4;MGC142039;MGC142041	ankyrin repeat and SOCS box containing 4	0.48	0.72	1.56	0.51
DAZ1	DAZ1;DAZ;FLJ17175;SPGY	deleted in azoospermia 1	0.44	0.64	1.57	0.51
TAS2R16	TAS2R16;T2R16	taste receptor, type 2, member 16	0.6	0.76	1.26	0.52
LRP1B	LRP1B;LRP-DIT;LRPDIT	low density lipoprotein receptor-related protein 1B	0.6	0.76	1.26	0.59
ADH4	ADH4;ADH-2	alcohol dehydrogenase 4 (class II), pi polypeptide	0.52	0.64	1.26	0.49
SPRR2E	SPRR2E	small proline-rich protein 2E	0.46	0.58	1.26	0.41
CASC1	CASC1;FLJ10921;LAS1;PPP1R54	cancer susceptibility candidate 1	0.37	0.46	1.26	0.34
TSPAN8	TSPAN8;CO-029;TM4SF3	tetraspanin 8	0.6	0.79	1.27	0.6
PKD1L3	PKD1L3	polycystic kidney disease 1-like 3	0.59	0.78	1.27	0.6
ABCA8	ABCA8;KIAA0822;MGC163152	ATP-binding cassette, sub-family A (ABC1), member 8	0.56	0.72	1.27	0.49
CLEC4D	CLEC4D;CLEC-6;CLEC6;CLECSF8;MCL;MGC40078;MPCL	C-type lectin domain family 4, member D	0.56	0.68	1.27	0.52
SLC36A3	SLC36A3;FLJ16658;MGC119638;MGC119639;MGC119641;PAT3;TRAMD2;tramdorin2	solute carrier family 36 (proton/amino acid symporter), member 3	0.42	0.54	1.27	0.36
CYP19A1	CYP19A1;ARO;ARO1;CPV1;CYAR;CYP19;CYPXIX;MGC104309;P-450AROM	cytochrome P450, family 19, subfamily A, polypeptide 1	0.42	0.53	1.27	0.41
SPHKAP	SPHKAP;DKFZp781H143;DKFZp781J171;KIAA1678;MGC132614;MGC132616;SKIP	SPHK1 interactor, AKAP domain containing	0.34	0.43	1.27	0.3
TAS2R8	TAS2R8;T2R8;TRB5	taste receptor, type 2, member 8	0.31	0.38	1.27	0.29
OR1J4	OR1J4;HSHTPCRX01;HTPCRX01;OR9-21	olfactory receptor, family 1, subfamily J, member 4	0.6	0.74	1.28	0.6
KRT25	KRT25;KRT25A	keratin 25	0.57	0.75	1.28	0.54
SULT1C3	SULT1C3;ST1C3	sulfotransferase family, cytosolic, 1C, member 3	0.55	0.66	1.28	0.52
CDY1	CDY1;CDY;CDY1A;FLJ77185	chromodomain protein, Y-linked, 1	0.42	0.5	1.28	0.37
CD1B	CD1B;CD1;CD1A;MGC125990;MGC125991;R1	CD1b molecule	0.38	0.46	1.28	0.39
KLRC3	KLRC3;NKG2-E;NKG2E	killer cell lectin-like receptor subfamily C, member 3	0.48	0.63	1.29	0.43
OR8U1	OR8U1	olfactory receptor, family 8, subfamily U, member 1	0.47	0.62	1.29	0.47
TDRD1	TDRD1;CT41.1;FLJ21082	tudor domain containing 1	0.36	0.47	1.29	0.32
TFAP2D	TFAP2D;TFAP2BL1	transcription factor AP-2 delta (activating enhancer binding protein 2 delta)	0.55	0.7	1.3	0.51
SLC9A10	SLC9A10	solute carrier family 9, member 10	0.48	0.66	1.3	0.5
MYL1	MYL1;MLC1F;MLC3F	myosin, light chain 1, alkali; skeletal, fast	0.48	0.6	1.3	0.46
FAM55A	FAM55A;MGC34290	family with sequence similarity 55, member A	0.44	0.59	1.3	0.44
ANKRD30A	ANKRD30A;NY-BR-1	ankyrin repeat domain 30A	0.24	0.32	1.3	0.23
OR2M7	OR2M7;OR1-58	olfactory receptor, family 2, subfamily M, member 7	0.57	0.72	1.31	0.55
ZPBP	ZPBP;FLJ93221;ZPBP1	zona pellucida binding protein	0.52	0.7	1.31	0.51
CLCA4	CLCA4;CaCC;CaCC2;MGC142247;MGC142249	chloride channel accessory 4	0.51	0.7	1.31	0.54
C9orf135	C9orf135	chromosome 9 open reading frame 135	0.46	0.6	1.31	0.44
C19orf18	C19orf18;MGC41906	chromosome 19 open reading frame 18	0.45	0.6	1.31	0.42
IGSF10	IGSF10;CMF608;FLJ25972	immunoglobulin superfamily, member 10	0.42	0.5	1.31	0.41
KRTAP13-2	KRTAP13-2;KAP13-2	keratin associated protein 13-2	0.41	0.52	1.31	0.42
C4orf22	C4orf22;MGC35043	chromosome 4 open reading frame 22	0.48	0.66	1.32	0.49
IFNB1	IFNB1;IFB;IFF;IFNB;MGC96956	interferon, beta 1, fibroblast	0.47	0.62	1.32	0.42
PLCXD3	PLCXD3	phosphatidylinositol-specific phospholipase C, X domain containing 3	0.46	0.58	1.33	0.47
OR5T1	OR5T1;OR11-179;OR5T1P	olfactory receptor, family 5, subfamily T, member 1	0.38	0.54	1.33	0.38
STRA8	STRA8	stimulated by retinoic acid gene 8 homolog (mouse)	0.55	0.78	1.34	0.55
SPDYA	SPDYA;MGC110856;MGC57218;RINGO3;RINGOA;SPDY1;SPY1	speedy homolog A (Xenopus laevis)	0.58	0.78	1.35	0.56
TRAT1	TRAT1;TCRIM;TRIM	T cell receptor associated transmembrane adaptor 1	0.42	0.6	1.35	0.43
CHRM3	CHRM3;HM3	cholinergic receptor, muscarinic 3	0.43	0.55	1.36	0.42
HYAL4	HYAL4;CSHY	hyaluronoglucosaminidase 4	0.31	0.41	1.36	0.29
RSPO3	RSPO3;CRISTIN1;FLJ14440;PWTSR;THSD2	R-spondin 3	0.57	0.75	1.37	0.57
DPPA2	DPPA2;CT100;ECAT15-2;PESCRG1	developmental pluripotency associated 2	0.52	0.76	1.37	0.59
VN1R4	VN1R4;V1RL4	vomeronasal 1 receptor 4	0.56	0.72	1.38	0.54
SPANXN3	SPANXN3;CT11.8;SPANX-N3	SPANX family, member N3	0.53	0.77	1.39	0.6
OR10AD1	OR10AD1;OR10AD1P;OR12-1	olfactory receptor, family 10, subfamily AD, member 1	0.46	0.66	1.39	0.44
C18orf34	C18orf34;FLJ44050;MGC111498;MGC163407	chromosome 18 open reading frame 34	0.52	0.76	1.41	0.51
OR5L1	OR5L1;OR11-151;OST262	olfactory receptor, family 5, subfamily L, member 1	0.3	0.44	1.42	0.35
HTR1F	HTR1F;5-HT-1F;5-HT1F;5HT6;HTR1EL;MR77	5-hydroxytryptamine (serotonin) receptor 1F	0.54	0.74	1.43	0.66
ADAM18	ADAM18;ADAM27;MGC41836;MGC88272;tMDCIII	ADAM metallopeptidase domain 18	0.48	0.66	1.43	0.5
TMPRSS11D	TMPRSS11D;HAT;MGC150587;MGC150588	transmembrane protease, serine 11D	0.42	0.6	1.43	0.45
SPAM1	SPAM1;HYA1;HYAL1;HYAL3;HYAL5;MGC26532;PH-20;PH20;SPAG15	sperm adhesion molecule 1 (PH-20 hyaluronidase, zona pellucida binding)	0.6	0.69	1.25	0.55
CD226	CD226;DNAM-1;DNAM1;PTA1;TLiSA1	CD226 molecule	0.6	0.76	1.25	0.52
DEFB134	DEFB134;MGC163333;MGC163335	defensin, beta 134	0.6	0.73	1.25	0.59
NPY5R	NPY5R;NPYR5	neuropeptide Y receptor Y5	0.58	0.72	1.25	0.64
IFNA13	IFNA13;IFNA1	interferon, alpha 13	0.58	0.74	1.25	0.56
C2orf66	C2orf66;UNQ6411	chromosome 2 open reading frame 66	0.55	0.72	1.25	0.54
LUZP4	LUZP4;CT-28;CT-8;CT28;HOM-TES-85	leucine zipper protein 4	0.48	0.6	1.25	0.44
GFRAL	GFRAL;C6orf144;GRAL;UNQ9356;bA360D14.1	GDNF family receptor alpha like	0.47	0.55	1.25	0.45
DEFB121	DEFB121;ESC42RELC	defensin, beta 121	0.54	0.46	0.81	0.35
CLEC9A	CLEC9A;DNGR1;UNQ9341	C-type lectin domain family 9, member A	0.58	0.49	0.85	0.36
RPL4	RPL4;L4	ribosomal protein L4	0.46	0.4	0.85	0.32
DYDC1	DYDC1;DPY30D1;FLJ43920;bA36D19.5	DPY30 domain containing 1	0.55	0.44	0.88	0.34
SYCP3	SYCP3;COR1;MGC71888;SCP3;SPGF4	synaptonemal complex protein 3	0.55	0.51	0.89	0.34

SLC12A1	SLC12A1;BSC1;MGC48843;NKCC2	solute carrier family 12 (sodium/potassium/chloride transporters), member 1	0.46	0.46	0.89	0.32
VSNL1	VSNL1;HLP3;HPCAL3;HUVISL1;VILIP;VILIP-1	visinin-like 1	0.5	0.46	0.9	0.31
SLC22A24	SLC22A24;MGC34821;NET46	solute carrier family 22, member 24	0.56	0.48	0.91	0.4
LINGO2	LINGO2;FLJ31810;LERN3;LRRN6C	leucine rich repeat and Ig domain containing 2	0.56	0.5	0.91	0.37
OR11H6	OR11H6	olfactory receptor, family 11, subfamily H, member 6	0.44	0.41	0.91	0.31
TAAR9	TAAR9;TA3;TAR3;TAR9;TRAR3	trace amine associated receptor 9 (gene/pseudogene)	0.58	0.5	0.92	0.38
TNFRSF17	TNFRSF17;BCM;BCMA;CD269	tumor necrosis factor receptor superfamily, member 17	0.59	0.56	0.93	0.4
TMCO5A	TMCO5A;FLJ35807;MGC35118;TMCO5	transmembrane and coiled-coil domains 5A	0.58	0.54	0.93	0.39
TLR8	TLR8;CD288;MGC119599;MGC119600	toll-like receptor 8	0.46	0.44	0.93	0.35
A4GNT	A4GNT;MGC149493;alpha4GnT	alpha-1,4-N-acetylglucosaminyltransferase	0.55	0.53	0.94	0.35
GALNT13	GALNT13;FLJ16031;FLJ41157;GalNAc-T13;H_NH0187G20.1;KIAA1918;MGC119459;MGC119461;WUGSC:H_NH0187G20.1	UDP-N-acetyl-alpha-D-galactosamine:polypeptide N-acetylgalactosaminyltransferase 13 (GalNAc-T13)	0.55	0.52	0.95	0.44
TEX14	TEX14;CT113	testis expressed 14	0.55	0.56	0.95	0.4
GLYATL2	GLYATL2;BXMAS2-10;GATF-B;MGC24009	glycine-N-acyltransferase-like 2	0.58	0.55	0.96	0.47
FYB	FYB;ADAP;PRO0823;SLAP-130	FYN binding protein	0.53	0.5	0.96	0.42
CSN1S1	CSN1S1;CASA;CSN1;MGC149368	casein alpha s1	0.44	0.43	0.96	0.33
OR5I1	OR5I1;HSOlf1;OLF1	olfactory receptor, family 5, subfamily I, member 1	0.57	0.54	0.97	0.43
TMEM71	TMEM71;FLJ33069;MGC111188	transmembrane protein 71	0.44	0.41	0.97	0.31
MORC1	MORC1;CT33;MORC;ZCW6	MORC family CW-type zinc finger 1	0.54	0.58	1	0.44
TRHR	TRHR;MGC141920;TRH-R	thyrotropin-releasing hormone receptor	0.53	0.51	1	0.46
C3orf65	C3orf65;FLJ32900	chromosome 3 open reading frame 65	0.46	0.48	1	0.36
PON1	PON1;ESA;MVCD5;PON	paraoxonase 1	0.6	0.61	1.01	0.47
IL13RA2	IL13RA2;CD213A2;CT19;IL-13R;IL13BP	interleukin 13 receptor, alpha 2	0.59	0.62	1.01	0.41
[truncated: 192,009 more chars]
